# Filling the Gap: Functional Clustering of ABC Proteins for the Investigation of Hormonal Transport *in planta*

**DOI:** 10.3389/fpls.2019.00422

**Published:** 2019-04-17

**Authors:** Lorenzo Borghi, Joohyun Kang, Rita de Brito Francisco

**Affiliations:** Department of Plant and Microbial Biology, University of Zurich, Zurich, Switzerland

**Keywords:** ATP-Binding Cassette, plant hormones, strigolactones, auxins, ABA, vacuolar import, plant-defense, hormonal precursors

## Abstract

Plant hormones regulate a myriad of plant processes, from seed germination to reproduction, from complex organ development to microelement uptake. Much has been discovered on the factors regulating the activity of phytohormones, yet there are gaps in knowledge about their metabolism, signaling as well as transport. In this review we analyze the potential of the characterized phytohormonal transporters belonging to the ATP-Binding Cassette family (ABC proteins), thus to identify new candidate orthologs in model plants and species important for human health and food production. Previous attempts with phylogenetic analyses on transporters belonging to the ABC family suggested that sequence homology *per se* is not a powerful tool for functional characterization. However, we show here that sequence homology might indeed support functional conservation of characterized members of different classes of ABC proteins in several plant species, e.g., in the case of ABC class G transporters of strigolactones and ABC class B transporters of auxinic compounds. Also for the low-affinity, vacuolar abscisic acid (ABA) transporters belonging to the ABCC class we show that localization-, rather than functional-clustering occurs, possibly because of sequence conservation for targeting the tonoplast. The ABC proteins involved in pathogen defense are phylogenetically neighboring despite the different substrate identities, suggesting that sequence conservation might play a role in their activation/induction after pathogen attack. Last but not least, in case of the multiple lipid transporters belong to different ABC classes, we focused on ABC class D proteins, reported to transport/affect the synthesis of hormonal precursors. Based on these results, we propose that phylogenetic approaches followed by transport bioassays and *in vivo* investigations might accelerate the discovery of new hormonal transport routes and allow the designing of transgenic and genome editing approaches, aimed to improve our knowledge on plant development, plant–microbe symbioses, plant nutrient uptake and plant stress resistance.

## Introduction

**FIGURE 1:**
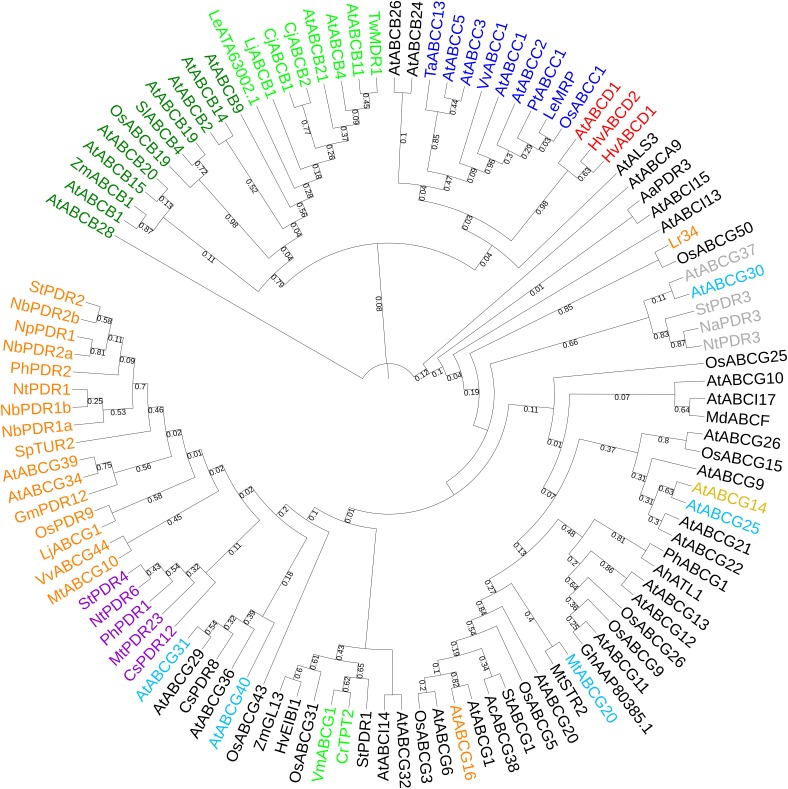
Phylogenetic tree comprising up-to-date characterized ABC proteins (Lefèvre and Boutry, 2018) in plants. Violet clade: strigolactone (SL) transporters and sequence homologs. Orange clade: transporters involved in plant defense. Light green clade: alkaloid transporters. Red clade: lipid, hormonal-precursor transporters. Gray clade: coumarin transporters. Blue clade: ABC class C proteins (tonoplast importers). Dark green clade: auxin transporters. Cyan leaves: ABA transporters. Brown leaf: CKs transporter. Bootstrap n: 100. No branches deleted due to the high variety of sequences included in this analysis. The tree with the highest log likelihood (–4735.73) is shown. The analysis involved 109 amino acid sequences. All positions containing gaps and missing data were eliminated. There were a total of 42 positions in the final dataset.

In plants, hormonal signaling pathways integrate internal and external cues to adapt the growth of these sessile organs to the surrounding, changing environment. Phytohormones tune the regulation of plant morphogenesis, plant development, biotic and abiotic stress resistance and plant–microbe interactions, either individually or in crosstalk network (Santner et al., 2009; Kamiya, 2010). Biosynthesis, perception and catabolism of phytohormones play an important role in the modification of such signaling pathways. As well, cell-to-cell transport, inner allocation and excretion of phytohormones toward the outer environment are important regulators of hormonal action (Park et al., 2017), which might span both long and short distances between the site of biosynthesis and the target of the action. Some years ago scientific works reported the severe growth defects caused by the loss-of-function of phytohormonal transporters (Gälweiler et al., 1998; Noh et al., 2001). This recognized need for hormone transporters during plant development overtook previous hypotheses that considered transporters not as necessary for hormonal distribution. Passive diffusion across the lipid bilayer was considered likely the main drive for hormonal transport (cellular import), because of the protonated form that most of the weak acid phytohormones have in physiological conditions. Instead, we know nowadays that diffusion plays only a partial role, e.g., for auxins (Vanneste and Friml, 2009). Different protein families regulate or facilitate phytohormonal distribution (Abualia et al., 2018) like PIN-FORMED (PIN, Adamowski and Friml, 2015), PIN-like (PILS, Feraru et al., 2012; Grones and Friml, 2015), NITRATE TRANSPORTER (NRT, Fan et al., 2017), MULTIDRUG AND TOXIC COMPOUND EXTRUSION (MATE), SWEET transporters (Takanashi et al., 2014; Julius et al., 2017) and ATP-BINDING CASSETTE (ABC) transporters, the latter being the focus of this review. ABC proteins are one of the largest superfamily of multifunctional, mostly transmembrane transporters that utilize the ATP hydrolysis energy to carry out the translocation of various substrates across membranes (Kang et al., 2011; Hwang et al., 2016; Do et al., 2018). Depending on sequence and domain organization they have been classified in several subfamilies, from A to I that are mostly present in plants, animals and fungi and share function especially related to detoxification mechanisms (Hwang et al., 2016). Originally isolated for their ability to confer multidrug resistance, nowadays we know that ABC transporters are involved in several aspects of plant growth and adaptation also through the mobilization of plant hormones. Shared-function ABC transporters involved into phytohormonal transport have been characterized both in mono- and di-cotyledon species, thus suggesting a high level of conservation in the plant kingdom. In contrast to their animal, low-selective counterparts, several plant ABC proteins transport specific hormones toward storage organelles, target organs and plant outer surfaces. Until recently, phylogeny was not considered a valuable tool to infer protein function in the ABC protein family. Reported cases of sequence homology between strigolactone and abscisic acid (ABA) transporters (Kretzschmar et al., 2012) or the phylogenetic distance between the several characterized ABA transporters supported that assumption. We show here that the increased characterization of ABC proteins in different plant species (Figure 1) that was carried out in the last decade allows now for wider phylogenetic analyses, suggesting the possibility of functional clustering in the ABC family and, based on the previously characterized ABC hormone transporters (Table 1), might permit the isolation of putative orthologs in several model and non-model plant species.

**Table 1:** Characterized ABC hormone transporters after (Lefèvre and Boutry, 2018).

Hormone	Transporter	Subfamily
Abscisic acid	AtABCG25	ABCG
Abscisic acid	AtABCG30	ABCG
Abscisic acid	AtABCG40	ABCG
Abscisic acid	MtABCG20 (Pawela et al., 2019)	ABCG
Auxins	AtABCB1	ABCB
Auxins	AtABCB14	ABCB
Auxins	AtABCB15	ABCB
Auxins	AtABCB19	ABCB
Auxins	AtABCB21	ABCB
Auxins	AtABCB4	ABCB
Auxins	AtABCG36	ABCG
Auxins	AtABCG37	ABCG
Auxins	LjABCB1	ABCB
Auxins	OsABCB14	ABCB
Auxins	ZmABCB1	ABCB
Cytokinins	AtABCG14	ABCG
Jasmonic acid	AtABCG16	ABCG
Strigolactones	PaPDR1	ABCG

*In this list, only the transporters for in vivo identified substrates are present.*

Phylogenetic analyses carried on characterized ABC transporters and their sequence homologs from different plant species (see Supplementary Material and Methods) shows that proteins belonging to the same class, such as ABC class B proteins for auxinic molecule transport could cluster together. Also, proximity is obtained with ABC proteins sharing the same substrate, like for the SL, ABC class G transporters or for the class D transporters of lipids that are precursors of phytohormones. In some cases, only single ABC proteins have been up-to-date characterized for specific hormonal transport, like AtABCG14 for cytokinins (CKs) (Ko et al., 2014; Zhang K. et al., 2014) and therefore phylogenetic analysis is not very informative. In other cases, ABC proteins share same physiological functions despite transporting different substrates, like for the transporters involved in plant–pathogen defense. Or they have just the subcellular localization in common, e.g., the tonoplast in case of the ABC class C, but not the transported substrate. We are therefore aware that phylogenetic analyses are not sufficient to discover new functions and substrates of ABC transporters, unless coupled with parallel strategies aimed to identify the (possibly specific) substrate(s) and functions via molecular and vesicular transport assays. We finally discuss the basic and applied potentials of such phylogenetic analysis and the plant species and agricultural practices that could benefit from it.

## Abcb Subfamily and Transport of Auxinic Molecules

In plants, the second most numerous subfamily of ABC transporters after ABCGs is class B (Geisler, 2014). Several ABCBs were characterized as transporters of the phytohormone auxin in Arabidopsis (ABCB1/PGP1, ABCB19/PGP19/MDR1, ABCB19, ABCB21) but also in rice (*Oryza sativa*, OsABCB14), maize (*Zea mays*, ZmABCB1) and *Lotus japonicus* (LjABCB1) (Xu et al., 2014; Geisler et al., 2017). Still, not all ABCBs are transporters of auxins or auxinic compounds like Indole-3-butyric acid (IBA). For example, in *Coptis japonica* CjABCB1/2 (Shitan et al., 2003, 2013) transport the alkaloid berberine. ABCBs regulating auxin transport are mostly exporters of auxins from biosynthetic active tissues toward the apoplast, therefore they are mainly localized in the plasma membrane of meristematic cells, where auxins are mostly synthetized, and along the stem vasculature where auxins are long-distance transported (Blakeslee et al., 2007). Other proteins like AtABCB14 and AtABCB15 were not directly demonstrated as auxin transporters in cellular or vesicular systems, still they are expressed mostly along the stem vasculature and reported to affect polar auxin transport in those tissues (Kaneda et al., 2011), supporting that ABCBs can regulate both short and long distance transport of auxins (Cho and Cho, 2013). ABCBs were initially characterized after sequence homology to their mammalian *P*-glycoproteins counterparts (Dudler and Hertig, 1992) and their inducibility by auxin (Noh et al., 2001). Through their sensitivity toward 1-N-Naphthylphthalamic acid (NPA), one of the most popular auxin transport inhibitors, their loss-of-function dwarf phenotypes as well as the strong reduction in polar auxin transport, not only ABCBs but also their partner proteins like TWISTED DWARF1 (TWD1, Geisler et al., 2004) were later characterized. Plant ABCBs are selective for their auxinic substrates, in contrast to their mammalian orthologs, isolated for their multidrug resistance ability (MDR). Similar to other proteins in charge of auxin trafficking but belonging to the PIN family, AtABCB1 and AtABCB19 contain a large hydrophilic, cytosolic loop whose phosphorylation regulates protein activity and localization (Henrichs et al., 2012). Interestingly, some ABCB auxin transporters, like AtABCB4 and AtABCB21 (Santelia et al., 2008; Kamimoto et al., 2012; Kubeš et al., 2012) have the ability to regulate auxin trafficking inwards and outwards. This double directional transport of auxins is likely present to finely tune the homeostasis of intra- and extracellular auxins.

Several investigations reported the presence of stable or transient protein–protein interactions, such as TWD1 with AtABCB1/4/19 (Geisler et al., 2004; Bailly et al., 2008) thus to regulate the activity and directionality of ABCBs involved in auxin transport. TWD1 functions as chaperone protein and regulates ABCB transport from the endoplasmic reticulum (ER) to the plasma membrane, this role shared with its mammalian ortholog FKBP38 (Geisler, 2014). In addition, protein–protein interaction or just shared localization has been shown between ABCBs and PINs, like for PIN1/PIN2 with AtABCB1/AtABCB19 and PIN3 with AtABCB4 (Titapiwatanakun et al., 2009; Geisler et al., 2017). The role and duration of these interactions are not fully clear, yet. Still, both specificity and transport efficiency of auxins are increased by co-expressing these proteins in Arabidopsis or in heterologous systems, suggesting that ABCB-PIN co-expression might synergistically or antagonistically co-ordinate auxin transport. Activity and localization of ABCBs, like reported in PINs, can be regulated by phosphorylation through common AGC kinases, such as PINOID and D6PK (Wang et al., 2012; Armengot et al., 2016). These common factors of PINs and ABCBs suggest the presence of crosstalk regulation between these two families of auxin transporters.

**FIGURE 2:**
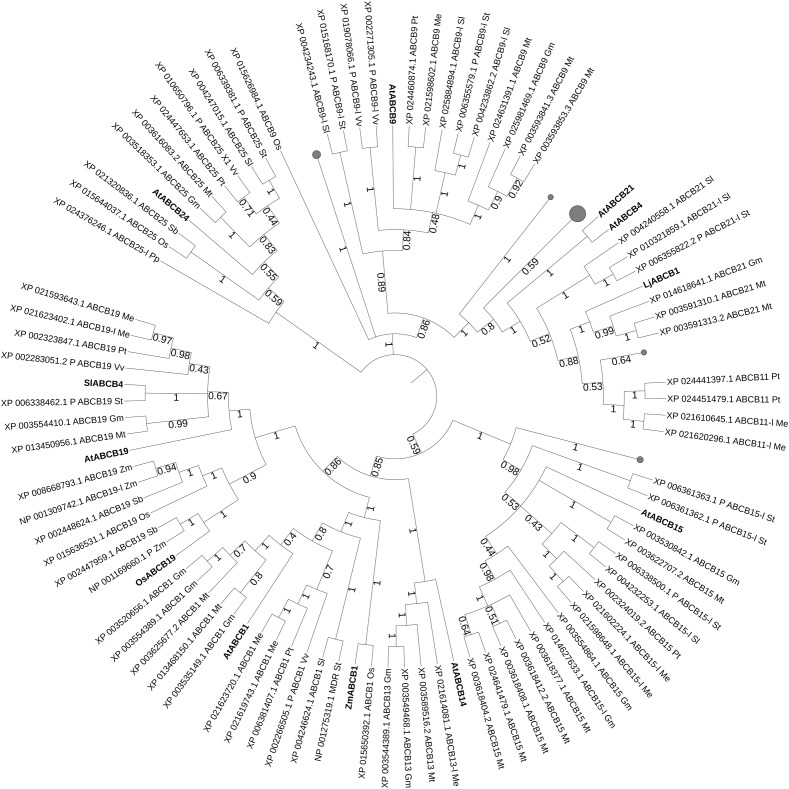
Best hits (sequence identity > 70%) to characterized ABCB proteins. Sequences from *Glycine max* (*Gm*, soya bean), *Populus trichocarpa* (*Pt*, poplar), *Manihot esculenta* (*Me*, manioc), *Solanum lycopersicum* and *tuberosum* (*Sl*, tomato and *St*, potato), *Medicago truncatula* (*Mt*, barrel medic), *Vitis vinifera* (*Vv*, grapevine), *Triticum aestivum* (*Ta*, wheat), *Oryza sativa* (*Os*, rice), *Lotus japonicus* (*Lj*), *Physcomitrella patens* (*Pp*), *Marchantia polymorpha* (*Mp*), *Hordeum vulgare* (*Hv*, barley), *Sorghum bicolor* (*Sb*), and *Zea mays* (*Zm*, maize). P, predicted; -l, -like. Bootstrap n: 100. Branches with bootstrap values < 0.4 are deleted. Maximum 3 isoforms displayed for size restriction. Gray circles (proportionally sized) represent collapsed nodes for size restrictions. The tree with the highest log likelihood (–122794.47) is shown. The analysis involved 136 amino acid sequences. There were a total of 1524 positions in the final dataset.

Through the protein sequences of characterized ABCB transporters we carried phylogenetic analyses for isolating ABCB transporter candidates in staple food/economically relevant model plants: soya bean, poplar, manioc, tomato, potato, barrel medic, grapevine, wheat, rice, *L. japonicus*, *Physcomitrella patens*, *Marchantia polymorpha*, barley, *Sorghum bicolor* and maize. The first 100 unique homolog hits (or the ones with sequence identity > 70%) were selected (Figure 2). AtABCB19 and SlABCB4 group together, while the other characterized ABCBs from Arabidopsis, rice, maize and lotus are dispersed in the tree, except for AtABCB4 and AtABCB21 forming a separated leaf. Despite their proximity and their common substrate, AtABCB19 and SlABCB4 likely do not share the same function. SlABCB4 is expressed in developing tomato (Ofori et al., 2018a), thus it is supposed to be important during fruit development. AtABCB19 regulates hypocotyl gravitropism and phototropism in Arabidopsis (Nagashima et al., 2008). These two characterized ABCBs cluster together with two ABCB19-like or predicted ABCB19 proteins from manioc (*Manihot esculenta*, Me) and one candidate from potato (*Solanum tuberosum*, St), barrel medic (*Medicago truncatula*, Mt), soya bean (*Glycine max*, Gm), poplar (*Populus trichocarpa*, Pt), and grapevine (*Vitis vinifera*, Vv). The closest leaf stemming from the same node bears the characterized OsABCB19 together with candidates from grasses (sorghum, maize, and rice). OsABCB19 is an auxin transporter involved in the regulation of gravity sensing and cytoplasmic streaming (Okamoto et al., 2015). This targeted search for ABCB homologs in model/crop plants shows several putative orthologs from soya bean and barrel medic proximal to AtABCB1. A close by leaf consists of the characterized ZmABCB1 and its putative ortholog OsABCB1. In maize, ABCB1 is expressed in nodal meristems and regulates auxin transport in mesocotyls and coleoptiles, suggesting that ABCB1 function is conserved in dicots and monocots (Knöller et al., 2010). AtABCB4 together with AtABC21, LjABCB1, AtABCB9, AtABCB24, AtABCB14, and AtABCB15 are present in isolated nodes. AtABCB4 is an auxin efflux transporter that regulates the elongation of root hair cells as well as lateral root development (Cho et al., 2007; Santelia et al., 2008). AtABCB21, together with AtABCB4 are facultative, bi-directional auxin transporter: they are cellular importers at low auxin concentrations and cellular exporters when auxin concentrations are high (Kamimoto et al., 2012). LjABCB1 is homolog and, as expected, proximal to AtABCB4. Its expression pattern is close to rhizobium-nodule infected cells and detected during nodulation, only (Takanashi et al., 2012). Therefore LjABCB1 is suggested to regulate auxin transport toward nodulation tissues, where auxins play an important role for the formation of nodule vasculature (Pacios-Bras et al., 2003). AtABCB9 is possibly an auxin transporter involved in pollen germination (Wang et al., 2008). AtABCB24 is mitochondrial expressed and it is involved in iron homeostasis but not in auxin transport. AtABCB24 is included in this analysis as outsider compared to ABCBs that regulate auxin transport. AtABCB14 was initially characterized as a malate importer involved in the modulation of stomatal response to CO_2_ (Lee et al., 2008). Together with AtABCB15, AtABCB14 is expressed in vasculature tissues of stems and they both participate in auxin transport along the inflorescence (Kaneda et al., 2011).

Interestingly, most of the characterized ABCBs cluster together in a group clearly separated from most of the “land plants” candidates (Supplementary Figure S1), showing that the search for orthology through sequence homology was possible and successful in the ABCB family. Out of this cluster, LjABCB1 is part of a leaf comprising several *Fabaceae* candidates. AtABCB21 is proximal to *Arabidopsis lyrata* isoforms and other *Brassicaceae* candidates.

## Abcc Subfamily and Hints for Vacuolar Hormonal Import

Plant ABC class C transporters, previously known as multidrug resistant-associated proteins (MRPs), were first described as being involved in barley (*Hordeum vulgare*) xenobiotics detoxification mechanisms (Martinoia et al., 1993). Recently, Lane et al. (2016) investigated the diversity of ABC transporters across plants via the One Thousand Plants Transcriptome Project^1^. Not surprisingly, algae presented a smaller number of unique ABCC genes when compared to other analyzed plant taxa, which may reflect algae life style and cellular organization, as algae in their living environments have direct access to nutrients.

Genome analysis predicts that Arabidopsis contains a total of 15 ABCC genes, *Brassica rapa* 21, *Hevea brasiliensis* 3 (such low number possibly due to the absence of typical central vacuoles), grapevine 26, lotus 17, maize 13, rice 17, tomato 26, and wheat 18 (Sánchez-Fernández et al., 2001; Garcia et al., 2004; Sugiyama et al., 2006; Çakır and Kılıçkaya, 2013; Pang et al., 2013; Bhati et al., 2015; Zhiyi et al., 2015; Chen et al., 2017; Ofori et al., 2018b).

Arabidopsis ABCC transporters are likely tonoplast localized (Jaquinod et al., 2007; Kang et al., 2011) and the same was suggested for ABCCs of grapevine and rice (Francisco et al., 2013; Song et al., 2014). Vacuoles are multifunctional organelles that accumulate chemically diverse compounds and exert many fundamental functions *in planta*, such as detoxification and storage of nutrients. Plants import into the vacuole endogenous compounds and xenobiotics mostly conjugated to polar β-D-glucosides, glutathione and amino acids. Alternatively or additionally, these compounds are excreted to the apoplast but not via ABCC. As recently discussed, vacuoles are also proposed to play a role in plant hormone homeostasis (Martinoia, 2018). Several reports identified hormones and/or hormone conjugates in the vacuolar sap. Gibberellin conjugates have been isolated in cowpea (*Vigna unguiculata*) and barley vacuoles (Garcia-Martinez et al., 1981). In Arabidopsis, indole-3-acetic acid (IAA), IAA conjugates, CKs and CK glucosides were quantified in isolated vacuoles (Ranocha et al., 2013; Jiskrová et al., 2016). Abscisic acid glucosyl ester (ABA-GE) has been described to accumulate in vacuoles of broad bean (*Vicia faba*) (Bray and Zeevaart, 1985) and in cell suspension cultures of tomato (Lehmann and Glund, 1986). The conjugated forms of the phenolic compound salicylic acid (SA), which represent most of the synthesized SA (Rivas-San Vicente and Plasencia, 2011) are stored in tobacco and Arabidopsis vacuoles (Dean and Mills, 2004; Dean et al., 2005; Vaca et al., 2017). In higher plants, hormone conjugates exist in two major forms: amine-linked bound to amino acids or proteins and ester-linked to sugars, (Staswick, 2009; Ludwig-Müller, 2011; Piotrowska and Bajguz, 2011; Korasick et al., 2013). It is still a matter of debate if these hormonal forms should be considered inactive and if the accumulation of these modified forms efficiently contributes to hormonal homeostasis. In some cases, hormone conjugates have been shown to have specific roles in plant development. It was reported (Staswick, 2009) that in Arabidopsis Jasmonic acid-Tryptophan (JA-Trp) and IAA-Trp interfere with root gravitropism. Recently, *VAS2/GH3.17* (IAA-amido synthetase Gretchen Hagen 3), which is predominantly expressed in hypocotyls, was shown to regulate hypocotyl growth. It was observed that *vas2* mutants accumulate higher levels of IAA, low levels of IAA-Glutamate (IAA-Glu) and presented longer hypocotyls under normal growth conditions compared to wild-type (WT) plants (Zheng et al., 2016). Latest studies also revealed a link between auxin-conjugates and ER-localized auxin carriers (Mravec et al., 2009; Barbez et al., 2012; Ding et al., 2012). *PIN5* and *PIN8* encode functional auxin transporters that regulate intracellular auxin homeostasis (Mravec et al., 2009; Ding et al., 2012). The analysis of *pin5* mutant phenotypes showed defects in lateral root initiation as well as in root and hypocotyl growth. Defective growth in root, hypocotyl and not fully expanded, epinastic cotyledons were present in *PIN5* gain-of-function mutants (Mravec et al., 2009). Auxin metabolic profile confirmed that free IAA levels decreased whereas the capacity to produce amino acid conjugates such as IAA–Aspartate (IAA-Asp) or IAA-Glu upon PIN5 induction increased (Mravec et al., 2009). PIN8 is expressed in the Arabidopsis male gametophyte and has a role in pollen development and functionality. Decreased transmission ability through the male gametophyte was observed in *pin8* mutants compared to wild-type pollen (Ding et al., 2012). In *pin8* mutants no significant alteration of free IAA was observed. However, in *PIN8* overexpressor lines free IAA levels increased whereas IAA-Asp and IAA-Glu decreased. Through results of complementation assays the authors suggested antagonistic roles of PIN5 and PIN8 (Ding et al., 2012). Similarly to PIN5, PILS2 and PILS5 activity was shown to increase free IAA levels and reduce IAA-Asp and IAA-Glu, presumably by regulating auxin compartmentalization into the ER lumen (Barbez et al., 2012). How the activity of IAA-amino synthetases, which biosynthesize the amino-IAA conjugates, and the above PINs and PILS activity is integrated is, however, still poorly understood.

The ABCCs have been long proposed to be involved in the vacuolar import of hormone conjugates, e.g., in case of ABA. ABA-GE was reported to be transported into mesophyll vacuoles via ABC protein-type dependent mechanism (Burla et al., 2013). The vacuolar conjugate ABA-GE was shown to be an important source of free ABA when plants experience abiotic stresses, via the activation of vacuolar β-glucosidase (BG2) under osmotic stress that induces the consequent hydrolyses of ABA-GE and release of free ABA (Xu et al., 2012). Arabidopsis AtABCC1 and especially AtABCC2, when heterologously expressed in yeast exhibit ABA-GE transport activity (Burla et al., 2013). However, since no apparent ABA-related phenotype could be observed either in the single mutant *atabcc1* and *atabcc2* or in *atabcc1 atabcc2* double mutants the authors proposed that multi-specific transporters might be involved in the vacuolar sequestration of conjugated hormonal metabolites (Burla et al., 2013). Interestingly, the vacuolar ABA-GE import was not exclusively performed by an ABC-type mechanism since it was shown that also antiporters are involved in such process. This was also the case for SA conjugates, namely SA 2-O-beta-d-glucose (SAG). SAG uptake by vacuolar membrane-enriched vesicles was stimulated by Mg-ATP and inhibited by vanadate and bafilomycin A1, an ABC transporter inhibitor and a vacuolar H^+^-ATPase inhibitor, respectively (Vaca et al., 2017).

For what concerns auxin conjugates, the function of such compounds in the vacuole is still unclear. Although certain auxin conjugates can be enzymatically hydrolyzed to produce free auxin (Ludwig-Müller, 2011; Piotrowska and Bajguz, 2011), this unlikely occurs in the vacuole, at least for IAA-amino-conjugates, as most of Arabidopsis auxin amino-hydrolases are predicted to be targeted to the ER (LeClere et al., 2002; Campanella et al., 2003). Up to now the identification of transporters regulating the import of auxin conjugates into the vacuole has not been addressed. So far the only functionally characterized tonoplastic transporter is involved in auxin metabolism. WALLS ARE THIN1 (WAT1) is an H^+^-IAA-symporter and it was shown to mediate the export of auxin from vacuoles (Ranocha et al., 2013). *wat1* mutants showed a decrease in stem fiber cell wall thickness that could be restored by exogenous auxin application to stems (Ranocha et al., 2010, 2013).

The maintenance of endogenous CKs levels is mediated by CK dehydrogenases/oxidases, which are the main enzymes mediating CKs degradation. Some members were shown to be localized in the vacuole (Werner et al., 2003; Skalický et al., 2018). Also, very little is known about the role of vacuolar-localized glycosylated-CK; for example, if they can be a source for free CKs as it was demonstrated for ABA-GE (Schroeder and Nambara, 2006). No candidate has yet been proposed for their import into the vacuole.

Although the evidence supports that the vacuole is involved in hormone homeostasis, in general not much is known about the transport mechanisms involved in such processes. According to the recent review by Lefèvre and Boutry (2018) on the substrates of ABC transporters, so far no transporter was univocally functionally characterized to import hormone conjugates into the vacuole.

**FIGURE 3:**
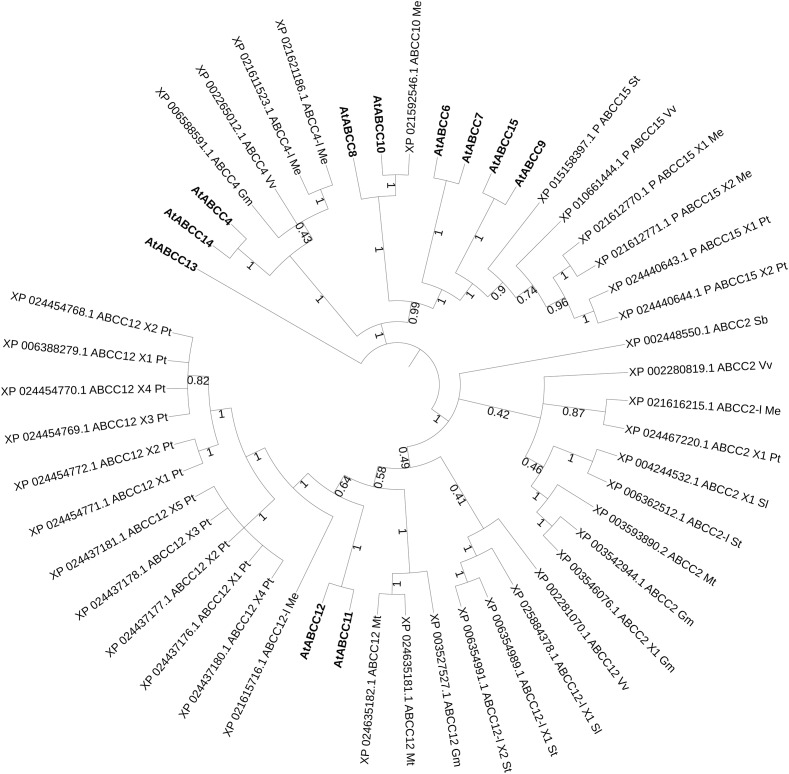
Best hits (sequence identity > 70%) homolog to poorly-characterized ABCC transporters in Arabidopsis. Sequences from *Glycine max* (*Gm*, soya bean), *Populus trichocarpa* (*Pt*, poplar), *Manihot esculenta* (*Me*, manioc), *Solanum lycopersicum* and t*uberosum* (*Sl*, tomato and *St*, potato), *Medicago truncatula* (*Mt*, barrel medic), *Vitis vinifera* (*Vv*, grapevine), *Triticum aestivum* (*Ta*, wheat), *Oryza sativa* (*Os*, rice), *Lotus japonicus* (*Lj*), *Physcomitrella patens* (*Pp*), *Marchantia polymorpha* (*Mp*), *Hordeum vulgare* (*Hv*, barley), *Sorghum bicolor* (*Sb*) and *Zea mays* (*Zm*, maize). P, predicted; -l, -like. Bootstrap n: 100. Branches with bootstrap values < 0.4 are deleted. Maximum 5 isoforms displayed for size restriction. Gray circles (proportionally sized) represent: collapsed nodes for size restriction. The tree with the highest log likelihood (–31375.71) is shown. The analysis involved 50 amino acid sequences. All positions containing gaps and missing data were eliminated. There were a total of 1014 positions in the final dataset.

In the phylogenetic tree with all characterized ABC proteins in plants (Figure 1), ABCCs form a cluster with two separated clades. The first comprises the transporters described as being involved in xenobiotics detoxification (AtABCC1, AtABCC2, PtABCC1, and OsABCC1), in naphthoquinone shikonin transport (LeMRP) and anthocyanin import (VvABCC1). The second clade is composed of transporters that are involved in xenobiotics detoxification (AtABCC3), and organic acids/phytate (AtABCC5; TaABCC13). In the attempt to expand our analysis to yet non-functional characterized ABCC that might putatively be involved in hormonal transport in plant models and crops, we performed a phylogenetic analysis excluding the characterized proteins of Figure 1 and including instead Arabidopsis, less characterized ABCCs (Figure 3), where function or substrate specificity are not yet fully understood.

Several clusters could be identified that could help a future characterization of ABCC protein in several plant species. AtABCC4 and AtABCC14 cluster with predicted ABCC4 proteins from manioc, one candidate from soya bean and one from grapevine. Although it was initially hypothesized that AtABCC14 could be involved in ABA-GE transport, membrane vesicles isolated from yeast heterologously expressing AtABCC14 did not exhibit detectable ABA-GE transport activity (Burla et al., 2013). Up-to-date no ABCC transporter was reported to be exclusively/directly involved in the vacuolar uptake of hormone conjugates possibly due to functional redundancy of several elements of this family, as also highlighted in the phylogeny analysis (Figure 3). In addition, other classes of vacuolar transporters, e.g., MATES could also participate in this process (Burla et al., 2013; Vaca et al., 2017). Strategies that could allow us a wider identification of novel functions of ABCC proteins could include simultaneous knockouts against redundancy, and overexpressing transgenic lines of multiple ABCC transporters. Genome editing technologies, such as CRISPR/Cas9 could be a powerful tool to accelerate our progresses in this field.

## Abcg Subfamily and Strigolactone Transport

Research on hormones belonging to the strigolactone (SL) family began in 1960s, when plant root exudates triggering the germination of parasitic weeds of the Striga family were discovered and named *Strigol* (Cook et al., 1966). Only in 2005 SLs were reported as inducer of hyphal branching in symbiotic arbuscular mycorrhizal fungi belonging to the ancient phylum *Glomeromycota* (Akiyama et al., 2005). In 2008 SLs were further characterized as regulators of plant shoot lateral branching (Gomez-Roldan et al., 2008) and later as phytohormones shaping above and below ground plant architecture (Al-Babili and Bouwmeester, 2015).

In recent years, SL research groups discovered new functions of the SL signaling pathway in different plant species (Goldwasser et al., 2008; Lopez-Raez et al., 2008; Liang et al., 2010; Proust et al., 2011; Delaux et al., 2012; Hamiaux et al., 2012; Kretzschmar et al., 2012; Rasmussen et al., 2012; An et al., 2016; Charnikhova et al., 2017; Rozpądek et al., 2018), thus showing the conservation of the SL signaling pathway in the plant kingdom from green algae to land plants. SL integrates plant development with environmental stimuli such as nutrient abundance, plant-rhizosphere symbioses and light quality (Nagata et al., 2016). Nowadays the scientific community holds a strong knowledge on SL biosynthesis, SL signaling (Lopez-Obando et al., 2015) and moved several steps toward the discovery of SL functions in mono- and di-cotyledon species, e.g., Arabidopsis (Sorefan et al., 2003; Booker et al., 2004, 2005; Bennett and Leyser, 2014; Brewer et al., 2016), rice (Song et al., 2017), *Fabaceae* (Foo et al., 2001; Yoneyama et al., 2008; Dun et al., 2013) and *Solanaceae* (Lopez-Raez et al., 2008; Drummond et al., 2011, 2015; Kretzschmar et al., 2012; Sasse et al., 2015; Xie et al., 2015). Several SL-driven developmental modules have been shown to be highly conserved in the plant kingdom, like shoot lateral branching, root hair elongation, leaf aging and crosstalk with most phytohormones. The recent development of SL-based strategies seems endless, going from approaches targeted to the improvement of good agricultural practices (Pavan et al., 2016; Cochetel et al., 2018; Liu et al., 2018a; Mohemed et al., 2018) to the amelioration of human health through the recently revealed apoptotic function of SL mimics in different kinds of cancers and diseases (Modi et al., 2017, 2018; Hasan et al., 2018).

Several factors affect SL activity in plant tissues and in the rhizosphere. First, in contrast to other hormone-receptor complexes, SLs are reported to permanently bind their heterodimeric receptor MORE AXILLARY GROWTH2/DWARF14, which after activation is targeted to degradation (Shabek et al., 2018; Yao et al., 2018). This means that the receptor-ligand complex might negatively regulate the amount of free SLs in the target tissue. Developmental and environmental signals adjust the amount of SL amounts in plants and rhizosphere, too: SL is highly synthetized in roots and its synthesis is induced, e.g., by high red/far-red ratios or low nutrient (mainly phosphate or nitrogen) conditions (Breuillin et al., 2010; Foo et al., 2013; Nagata et al., 2015; Kameoka and Kyozuka, 2018). Another factor that was shown to regulate the availability of SL in and out plants is the SL transporter PLEIOTROPIC DRUG RESISTANCE 1 from *Petunia hybrida* (PhPDR1). PhPDR1 is an ABC class G protein, originally isolated in petunia (Kretzschmar et al., 2012). PhPDR1 is expressed in root tips and in specialized root cortex cells, named hypodermal passage cells (HPCs) that are unsuberized gates for mycorrhizal hyphae and exudation points for SLs. HPC abundance is regulated by environmental signaling and plant age (Sharda and Koide, 2008). PhPDR1 is also expressed in the shoot lateral axils close to dormant lateral buds, and it is suggested to transport SLs into the buds thus contributing to the inhibition of lateral bud outgrowth. At support of this hypothesis, mutants for *pdr1* exude little SL to the rhizosphere, their roots are scarcely mycorrhized, shoots are heavily branched and *pdr1* seedlings accumulate SLs in biosynthetically active tissues such as the root tip. PhPDR1 is asymmetrically localized in the plasma membrane of cells that transport SLs (Sasse et al., 2015), supporting the hypothesis that PhPDR1 can directionally export SLs from SL biosynthetic-tissues and additionally release a SL-driven negative feedback on the expression of its biosynthetic enzyme *CAROTENOID CLEAVAGE DIOXYGENASE1/DECREASED APICAL DOMINANCE1* (*CCD8/DAD1*). The presence of this feedback regulation between SL transport and synthesis was also assayed by altering PhPDR1 levels with a *PhPDR1* overexpressor based strategy (PDR1 OE) (Liu et al., 2018b). PDR1 OE transgenic plants contained low SL amounts in leaves and a stay-green phenotype was induced, suggesting that PDR1 OE could counteract the reported SL-driven leaf senescence (Yamada and Umehara, 2015). Also, PDR1 OE plants exuded higher than WT amounts of SLs to the rhizosphere and could quickly induce mycorrhization and parasitic weed germination at higher levels than the WT. Last but not least, PDR1 OE plants increased their root and shoot biomass by inducing lateral root development, stem secondary growth and increased plant nutrient uptake (phosphate) on poor soil conditions. These positive effects of PDR1 OE on plant biomass production were also present in simulated-microgravity conditions (Liu et al., 2018a) that could negatively influence the development/branching of mycorrhizal fungi. These several changes in plant development and plant-fungal interactions caused by different expression levels of PhPDR1 show that transport and allocation of SL operated by PhPDR1 play a prominent role in the efficiency of the SL signaling pathway and support the hypothesis that phytohormonal transport is key to regulation of plant development and plant-environment interactions.

What regulates the expression level, cellular and sub-cellular localization of PhPDR1, so that SL transport can be finely tuned in space and time? Does SL transport require an ABCG protein only in *P. hybrida*? Until now, these points were difficult to address. The fact that in Arabidopsis the sequence homolog of PhPDR1 is the ABA transporter ABCG40 (Kang et al., 2010) and that PhPDR1 does not transport ABA (Kretzschmar et al., 2012) did not speed up the investigation of SL transport out of petunia, as the rich molecular too-box, small size and fast developmental speed of Arabidopsis could not be exploited, not considering that Arabidopsis is seen as non-host to arbuscular mycorrhizal fungi, despite that it was shown to have the potential to be colonized (Veiga et al., 2013). A likely PhPDR1 ortholog in *Nicotiana tabacum*, NtPDR6, was recently reported (Xie et al., 2015), but no bioassays or transport experiments were shown yet at proof that NtPDR6 is a SL transporter. An ongoing research line in *M. truncatula* strongly suggests that MtPDR23 (Banasiak and Jasiñski, 2014) is the ortholog of PhPDR1. Confirmation of this hypothesis will be important to show that active SL transport is also required out of *Solanaceae* and even in plants without HPCs. We report here that the PDR1/-like transporters above mentioned share a high sequence similarity, higher than with AtABCG40 or its best scoring sequence homologs. More in detail, BLAST hits similar to NtPDR6 and PhPDR1 from a subset of land plant accessions (Supplementary Figure S2) comprise sequence homologs from *Capsicum annuum* (*Can*), *S. tuberosum* (*St*)*/S. lycopersicum* (*Sl*)*/Solanum pennellii* (*Sp*), *N. sylvestris* (*Ns*)*/N. tabacum* (*Nt*)*/Nicotiana attenuata* (*Na*) and *Nicotiana tomentosiformis* (*Nto*). NtPDR6 and PhPDR1 are proximal to PDR1-like candidates from *Ipomoea nil* (*In*, Japanese morning glory). MtPDR23 is separated and clusters with a candidate from *Cicer arietinum* (*Ca*, chickpea) and it is a subgroup of a clade containing MtPDR23-like candidates from *Cajanus cajan* (*Ccaj*), *Glycine max* (*Gm*), *Phaseolus vulgaris* (*Pv*), and *Vigna radiata* (*Vr*). AtABCG40 forms a clade unrelated to SL characterized transporters together with ABCG40/PDR12 candidates from *A. thaliana/A. lyrata* (*Al*), the related *Capsella rubella* (*Cr*), and proximal to ABCG40 candidate from *Camelina sativa* (*Cs*), *Brassica oleracea* (*Bo*)*/Brassica napus* (*Bn*)*/B. rapa* (*Br*), *Raphanus sativus* (*Rs*) and *Eutrema salsugineum* (*Es*).

**FIGURE 4:**
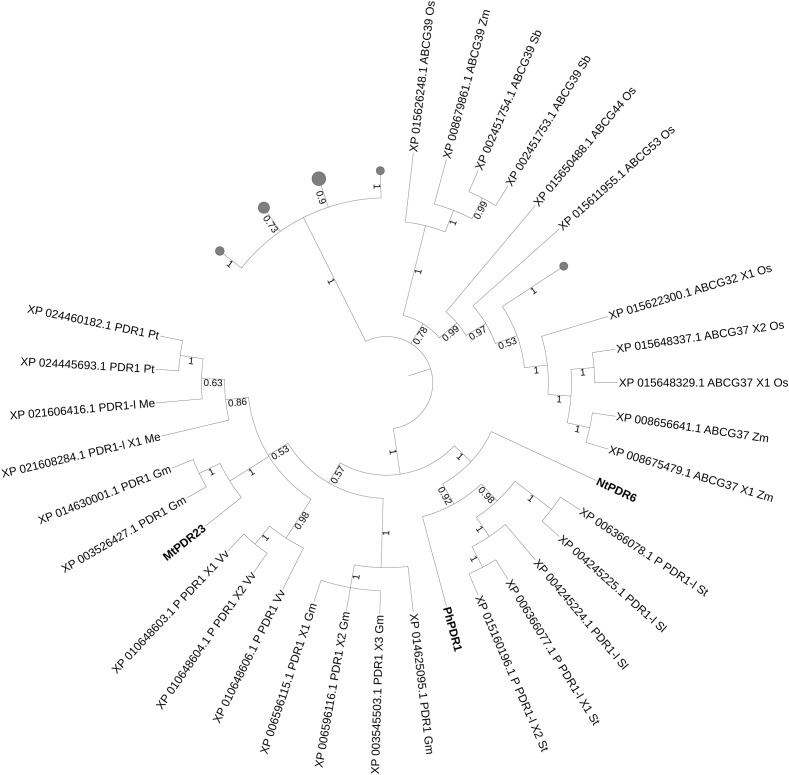
Best hits (sequence identity > 70%) to published SL transporters MtPDR23, PhPDR1, and NtPDR6. Sequences from *Glycine max* (*Gm*, soya bean), *Populus trichocarpa* (*Pt*, poplar), *Manihot esculenta* (*Me*, manioc), *Solanum lycopersicum* and t*uberosum* (*Sl*, tomato and *St*, potato), *Medicago truncatula* (*Mt*, barrel medic), *Vitis vinifera* (*Vv*, grapevine), *Triticum aestivum* (*Ta*, wheat), *Oryza sativa* (*Os*, rice), *Lotus japonicus* (*Lj*), *Physcomitrella patens* (*Pp*), *Marchantia polymorpha* (*Mp*), *Hordeum vulgare* (*Hv*, barley), *Sorghum bicolor* (*Sb*) and *Zea mays* (*Zm*, maize). P, predicted; -l, -like. Bootstrap n: 100. Branches with bootstrap values < 0.4 are deleted. Maximum 3 isoforms displayed for size restriction. Gray circles (proportionally sized) represent collapsed nodes for size restriction. The tree with the highest log likelihood (–77409.74) is shown. The analysis involved 112 amino acid sequences. There were a total of 1755 positions in the final dataset.

We then took advantage of the three published SL transporters to run phylogenetic analyses for isolating SL transporter candidates in staple food/economically relevant model plants: soya bean, poplar, manioc, tomato, potato, barrel medic, grapevine, wheat, rice, *L. japonicus*, *P. patens*, *M. polymorpha*, barley, *S. bicolor* and maize. The first 100 unique homolog hits (or the ones with sequence identity > 70%) to MtPDR23, PhPDR1 and NtPDR6 were selected. No grass sequence homologs clustered together with the known SL transporters (Figure 4). Predicted PDR1-like hits from tomato and from potato clustered with PhPDR1 and NtPDR6. Also, several not characterized PDR1 and isoforms from *Glycine max* grouped with MtPDR23, as well as from *P. trichocarpa*, *M. esculenta*, and *V. vinifera*. Some, but not all ABC transporters are reported to be induced by their substrate (Lefèvre and Boutry, 2018). We previously showed that PhPDR1 but not its closest sequence homolog in Petunia (PhPDR4) is induced by GR24, a SL mimic (Borghi et al., 2016). We suggest therefore that the sensitivity to GR24 or to a SL mimic might help screening for PDR1 putative candidates out of the results from this phylogenetic analysis. From the additional phylogenetic analysis based on all characterized ABC transporters in plants up-to-date, NtPDR6, MtPDR23, and PhPDR1 (violet clade) form a well-separated group with StPDR4 and CsPDR12 (Figure 1). *StPDR4* (Ruocco et al., 2011) expression levels are induced by heavy metals; *CsPDR12* by phytohormones (Migocka et al., 2012). The authors who characterized CsPDR12 speculate these ABC proteins might be involved in the transport of ABA or auxinic precursors. Based on this analysis, we suggest that CsPDR12 and StPDR4 could be further tested for a possible role in SL transport.

## Sl Importance for Crop Design

Strategies focused on the alteration of SL synthesis and/or allocation are promising for the promotion of plant biomass production on nutrient scarce soils. Previous projects based on low SL exuding varieties such as NERICA *New Rice for Africa* (Cissoko et al., 2011) or *S. bicolor* (Mohemed et al., 2018) proved to be successful by reducing the germination of parasitic weeds in arable lands of subtropical areas. In farm lands with no parasitic weeds, enhanced SL exudation could on the contrary improve mycorrhization and likely plant nutrient uptake. Or, alternatively, high SL exuding plants could be included in push-pull strategies, where parasitic-weed infected plants are physically removed from the fields before planting the crop of interest (Parker, 2012). As well, high SL exuding non-host plants to parasitic-weeds could drive suicidal germination (Kgosi et al., 2012) and help clearing agriculture fields before usage, or high mycorrhization levels could induce protection against parasitic weed as shown with *Z. mays* (Othira et al., 2012). Low SL biosynthesis obtained either by knock out or breeding equally affects root and shoot architectures with effects not always desirable for agricultural practices. For example, the loss of control on lateral bud outgrowth obtained in SL biosynthesis mutants decreases the germination of parasitic weeds but increases lateral branching/foliage also when not needed and reduces internode elongation. In contrast, plants with high SL exudation and/or production are advantaged on nutrient scarce soils but also show apical dominance, detrimental in case fruits/seeds are produced on lateral branches. As well, such plants would promote higher germination of parasitic weed seeds if present in the soil. The development of new techniques affecting SL transport/synthesis only above or belowground might be a further step to select/design breeds with enhanced plant–microbe symbioses but with no bushy/dwarf shoot architectures or plants with regulated shoot radial/lateral growth without the collateral germination of detrimental weeds.

## Abc Class G Transporters and Resistance to Plant–Pathogens

Latterly, most studies on the role of hormones in plant–pathogen defense focused on classical defense hormones: jasmonic acid (JA), ethylene (ET) and SA (Glazebrook, 2005). New, ongoing progress has revealed more complex hormonal crosstalk with additional hormones such as auxins (Kazan and Manners, 2009), ABA (Cao et al., 2011), CKs (Choi et al., 2014), and brassinosteroids (Belkhadir et al., 2012). These interactions allow fast defense reactions against plant–pathogens (Berens et al., 2017). We highlight here the up-to-date knowledge on ABC transporters which regulate the distribution of defense hormones. AtJAT1/AtABCG16 modulates the intracellular distribution of JA by mediating both cellular efflux of JA and nuclear influx of jasmonoyl-isoleucine (JA-Ile) (Li et al., 2017), the latter critical to activate JA signaling in the nucleus. Interestingly, AtABCG16 clusters with several ABC class G transporters (Figure 1), which are reported as lipid/lipid-related molecules transporters. This phylogenetic proximity of AtABCG1 and AtABCG16 is supported by their shared function as transporters of hydrophobic molecules for pollen coat formation (Yadav et al., 2014; Yim et al., 2016).

Apart from AtABCG16, several ABC class G transporters regulate plant–pathogen defense by orchestrating SA and JA signaling pathways and/or actively exuding compounds with herbivore/fungal/bacterial toxicity toward the plant surfaces (Hwang et al., 2016; Khare et al., 2017). Phylogenetic analyses with up-to-date characterized ABC transporters in plants (Figure 1) show the presence of a large cluster for plant–pathogen defense. This cluster contains mainly players characterized for biotic stress resistance in grapevine, lotus and several *Solanaceae*. The expression level of some of these transporters is induced by the presence of Pathogen-Associated Molecular Pattern (PAMP)-triggers and/or with the activation of a pathogen-defense, hormonal-signaling pathway. Like in the case of elicitin INF1, an elicitor protein secreted by the potato late blight pathogen *Phytophthora infestans* for NbPDR1a/b and NbPDR2a/b (Pierman et al., 2017), or *Phytophthora medicaginis* cell-wall oligosaccharides for MtABCG10 (Jasinski et al., 2009) or MeJA for NpPDR1, NtPDR1, and LjABCG1 (Grec et al., 2003; Crouzet et al., 2013; Sugiyama et al., 2015). GmPDR12 from soya bean is also induced by MeJa and SA (Eichhorn et al., 2006).

Major substrates of ABC transporters belonging to this cluster are secondary metabolites: terpenoids, alkaloids and flavonoids. The *Solanaceae* NtPDR1, NpPDR1, NbPDR1, and NbPDR2 support the transport of terpenes (Stukkens et al., 2005; Shibata et al., 2016; Pierman et al., 2017; Rin et al., 2017). AtABCG34 secretes to the leaf surface camalexin that is a major phytoalexin in Arabidopsis and thereby fights *Alternaria brassicicola* infection (Khare et al., 2017). The mutant of *atabcg34* was screened out of altered sensitivity to sclareol, a natural diterpene known to act as an antimicrobial and defense-related molecule, whereas the mutant for *atabcg39*, the closest homolog of *AtABCG34* has no altered sensitivity to sclareol (Khare et al., 2017). AtABCG39 plays a role in the cellular import of non-selective paraquat (Xi et al., 2012). MtABCG10 might modulate isoflavonoids amounts related to phytoalexin production (Banasiak et al., 2013). VvABCG44 is suggested as promising transporter of phenolic compounds, such as resveratrol (Suzuki et al., 2014). Last but not least, PhPDR2 is involved in the accumulation of steroidal compounds in leaves and trichomes (petuniasterone precursors) (Sasse et al., 2016).

Interestingly, *Lr34/Yr18/Pm38* multi-pathogen resistance gene exists as solo player and it is not included in the above cluster (Figure 1). The wheat Lr34-resistance (Lr34res) allele increases durable disease resistance against multiple fungal pathogens in different crop species (Krattinger et al., 2009, 2013). Its mechanism of action and substrate are yet unknown. OsABCG50 shares 85% of identity with Lr34 but cannot confer rice blast resistance (Krattinger et al., 2013). Despite being described as ortholog, OsABCG50 does not share a common function with Lr34res as it has the same SNP with Lr34-susceptible allele and not with Lr34res (Krattinger et al., 2013).

**FIGURE 5:**
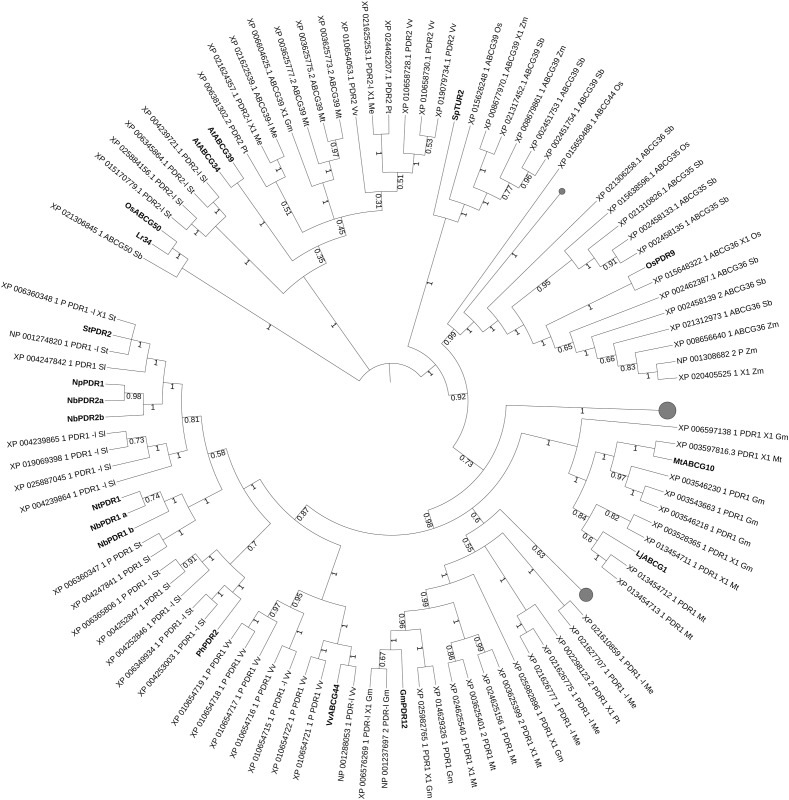
Best hits (sequence identity > 70%) to ABC transporters involved into response to pathogen attack. Sequences from *Glycine max* (*Gm*, soya bean), *Populus trichocarpa* (*Pt*, poplar), *Manihot esculenta* (*Me*, manioc), *Solanum lycopersicum* and t*uberosum* (*Sl*, tomato and *St*, potato), *Medicago truncatula* (*Mt*, barrel medic), *Vitis vinifera* (*Vv*, grapevine), *Triticum aestivum* (*Ta*, wheat), *Oryza sativa* (*Os*, rice), *Lotus japonicus* (*Lj*), *Physcomitrella patens* (*Pp*), *Marchantia polymorpha* (*Mp*), *Hordeum vulgare* (*Hv*, barley), *Sorghum bicolor* (*Sb*) and *Zea mays* (*Zm*, maize). P, predicted; -l, -like. Bootstrap n: 100. No branches deleted as bootstrap values in branches of interest > 0.4. Maximum 1 isoform displayed for size restriction. Gray circles (proportionally sized) represent collapsed nodes for size restriction. The tree with the highest log likelihood (–66697.92) is shown. The analysis involved 130 amino acid sequences. All positions containing gaps and missing data were eliminated. There were a total of 1097 positions in the final dataset.

Clades with candidates for plant–pathogen defense in crop/model plants are present after phylogenetic analyses with characterized plant-defense ABCs (Figure 5). Sequence homologs from selected crop/model plants to the characterized defense-involved transporters were isolated in manioc, barrel medic, grapevine, tomato, potato, soya and sorghum. Several candidates from tomato and potato group together with PhPDR2, StPDR2 and *Nicotiana* spp. PDR1/2. VvABCG44 clusters with and is proximal to multiple grapevine candidates, suggesting that the presence of this compound is mainly, if not only, in grapevine among the analyzed species. A small leaf comprising Lr34 and OsABCG50 shows the presence of a homolog candidate in sorghum. MtABCG10, LjABCG1 and GmPDR12 are proximal to soya and barrel medic transporters, which might be candidates for a role in plant defense. Loss of function of lipid transporters regulating the presence of hydrophobic barriers aimed to protect against harmful environments (Burghardt and Riederer, 2007) can also lead to weaknesses against pathogen attacks. These barriers consist of different types of hydrophobic polymers depending on the organ tissue and several ABC class G transporters regulate the export of each hydrophobic monomers to the right layer (Do et al., 2018). OsABCG31, from rice and HvABCG31/HvEIBI1, from barley also contribute to cuticle formation (Chen et al., 2011a,b). The cuticular alterations in *osabcg31* mutant plants result in reduction of infection structures of the rice blast fungus *Magnaporthe oryzae* in the plant, as well as in the constitutive up-regulation of genes involved in pathogen resistance (Garroum et al., 2016). The abnormal cuticle formation and the consequent lack of surface lipid molecules/transporters may affect the signaling for plant–pathogen defense.

## Abcd SubFamily and Transport of Hormonal Precursors

Particular classes of multiple ABC transporters participate to the transport of different lipids, including fatty acids, waxes, and sterols. It is a very remarkable feature of ABC proteins in all phyla. In case of human, almost half of ABC proteins participate in the translocation of lipids or lipid-related molecules (Tarling et al., 2013). Numerous ABC transporters *in planta* also contribute to lipid transport and are classified on the three main transported substrates: hydrophobic monomers for cell surface lipid barriers, fatty acid precursors for hormonal biosynthesis and components of membrane lipids (Kang et al., 2011). Deeper reporting on ABC proteins involved in the transport of lipid/lipid related molecules is out of the scope of this review. In spite of that the lipid transporters have reason to be mentioned, because they comprise fatty acid transporters for hormone precursors (Theodoulou et al., 2005). In addition, several ABC class G proteins which transport hydrophobic monomers as components for surface lipid barrier assist the role of SA/JA transporters in plant–pathogen defense function (Aragón et al., 2017; Ziv et al., 2018).

Peroxisomal ABC proteins (ABC class D proteins) contribute to the process of phytohormonal biosynthesis by transporting fatty acids that are hormonal precursors. HvABCD1/2 and AtABCD1 are ABC peroxisomal transporters that can complement yeast mutants for fatty acid beta-oxidation (Mendiondo et al., 2014). AtABCD1/CTS has been implicated in the transport of β-oxidation substrates related to hormone precursors: 12-oxo-phpytodienoic acid (OPDA); JA precursor (Theodoulou et al., 2005; Dave et al., 2011), 2, 4-dichlorophenoxybutric acid (2, 4-DB) and IBA; auxin precursors (Zolman et al., 2001; Hayashi et al., 2002). Blasting these characterized ABCD transporters on a plant-specific, narrow multiple-species database resulted in a number of sequence homology-based, annotated candidates for transport of fatty acids (Supplementary Figure S3).

## The Dispersed Aba Transporters

Abscisic acid rapidly accumulates in its target sites in response to a variety of biotic and abiotic stresses, such as pathogen, wounding, drought, radical temperature change or salinity. ABA plays a critical role in plants for overcoming these environmental stresses (Ton et al., 2009; Cutler et al., 2010). ABA research has made great progress during the last century and many different aspects of signal transduction, biosynthesis/catabolism and molecular mechanisms of action have been revealed (Nambara and Marion-Poll, 2005; Cutler et al., 2010; Raghavendra et al., 2010). However, ABA transport was not studied much, partly because ABA was regarded as able to cross the lipid bilayer and enter cells as protonated form and therefore a transporter was considered not necessary (Heilmann et al., 1980). However, results on the kinetics of ABA uptake disprove the mere action of passive diffusion (Windsor et al., 1992; Wilkinson and Davies, 1997). Finally, two different ABC class G ABC proteins were identified as ABA transporters until 2010: AtABCG40, an ABA importer in guard cell plasma-membrane, and AtABCG25, an ABA exporter in vascular tissue where ABA biosynthesis occurs (Kang et al., 2010; Kuromori et al., 2010). Recently, multiple ABA transporters are reported to regulate each cell-, tissue type specific ABA transport also by establishing a complex feedback network with ABA biosynthesis (Kuromori et al., 2018).

When plants suffer water deficit, ABA has to be immediately synthesized and transported from specific biosynthetic sites to the action site thus to cope with drought stress. AtABCG25 pumps *de novo* biosynthetic ABA out of vascular cells into the apoplast (Kuromori et al., 2010). Apoplastic ABA is then rapidly loaded into guard cells via the ABA importer AtABCG40, which is expressed in guard cells to positively regulate stomata closing (Kang et al., 2010). ABA regulates additional developmental processes in plants. *De novo* ABA biosynthesis continuously occurs in the endosperm, but not in embryo, thus to maintain embryo dormancy (Ali-Rachedi et al., 2004; Penfield et al., 2006; Barrero et al., 2010). The endosperm, a single layer tissue surrounding the embryo releases ABA to repress embryonic development and consequent germination (Lee et al., 2010). The cooperation of four different ABA transporters from Arabidopsis ABC class G proteins is responsible for the relocation of ABA to control seed dormancy. AtABCG31 and AtABCG25 are expressed in the endosperm and secrete ABA from the endosperm to the embryo. AtABCG30 and AtABCG40, located in the embryo participate to ABA accumulation in the embryo (Kang et al., 2015). Recently, MtABCG20 was characterized as ABA exporter from *M. truncatula*. MtABCG20 is localized in the plasma membrane of roots and germinating seeds. It secretes ABA from ABA biosynthetic sites in a homo-dimer form and thereby positively affects lateral root primordium formation, inhibits the development of nodule primordia and promotes the embryonic germination in Medicago (Pawela et al., 2019).

The up-to-date characterized ABC proteins involved into ABA transport do not form remarkable clusters. Instead, they are dispersed in different small branches, suggesting that any eventual sequence/domain similarity is not large or not conserved enough in transporters for ABA, or that ABA transporters do not share significant similarities at all. Already reported ABA transporters in Arabidopsis belong also to different protein families: ABC protein superfamily, nitrate transporter 1/Peptide transporter family (NPF; NPF4.6, used to be called AIT1) (Kanno et al., 2012), and detoxification efflux carrier like DTX50, which is a member of the MATE family (Zhang H. et al., 2014).

AtABCG31, an ABA exporter from the endosperm to the embryo thus regulating seed dormancy (Kang et al., 2015) makes a small group with AtABCG40, ABA importer into guard cells. This cluster is composed by full size ABC class G transporters, called PDR: the multi-functional ABC transporter AtABCG36 (Stein et al., 2006; Kim et al., 2007; Strader and Bartel, 2009) and its ortholog CsPDR8 from cucumber (Migocka et al., 2012), and the monolignol transporter AtABCG29 (Alejandro et al., 2012). AtABCG25 exports ABA from its biosynthetic site to the apoplast (Kuromori et al., 2010) and groups with AtABCG21 and AtABCG22, which are putative ABA importers/related to ABA (Kuromori et al., 2011, 2017). This cluster also includes the CKs transporter AtABCG14 (Ko et al., 2014).

AtABCG30, MtABCG20, and AhATL1 exist as solo players (Figure 1). AtABCG30, another embryonic ABA importer regulating seed dormancy together with AtABCG31 (Kang et al., 2015) clusters with a coumarin/IBA transporter from Arabidopsis, AtABCG37 (Ruzicka et al., 2010; Ziegler et al., 2017). MtABCG20, an ABA exporter regulating root morphology and seed germination is close to MtSTR2 that is required for mycorrhizal arbuscule development in Medicago (Zhang et al., 2010). The potential ABA transporter, ABA transporter-like 1(AhATL1; Ah_AQW44869.1) from *Arachis hypogaea* (peanut) belongs to the ABC class G of ABC proteins. AhATL1-overexpressor was shown to reduce drought resistance by inhibiting the drought induced *AtABCG40* expression in guard cells of Arabidopsis (Ge et al., 2017). AhATL1 groups with the floral volatile organic compound transporter PhABCG1 (Adebesin et al., 2017).

## Conclusion and Outlook

These phylogenetic analyses on characterized ABC proteins involved in phytohormonal transport and its regulation generated different amounts of potential functional homologs for each ABC subfamily. Still, as long as no bioassays are carried on for testing the hormonal substrate specificity (if any) of such transporters, these results are only hints toward candidate orthologs. Characterized proteins belonging to the ABCB class and their sequence homologs from selected crop and model plants generally did not group into common clades (Figure 2). Exceptions were SlABCB4 and AtABCB19, auxin transporters with reported different function during fruit and vegetative development, respectively. The proximal leaves from potato, barrel medic, soya, grapevine, and poplar are candidates for auxin transport in these plant species. The clustering of SlABCB4 and AtABCB19 is conserved after the phylogenetic tree was built on the larger “land plants” group sequence homologs (Supplementary Figure S1), with the additional proximity of OsABCB19. Another clade containing characterized ABCB transporters from different plant species consists of ZmABCB1 and AtABCB1. The other characterized candidates are again clearly separated and the sequence homologs present in this setup are mostly from *Brassicaceae*, except for the cluster containing lotus ABCB1 and six *Fabaceae* candidates.

Following the phylogenetic analyses based on SL transport candidates, AtABCG40 and its clade resulted to be no close homologs to known SL transporters (Figure 1 and Supplementary Figure S2). NtPDR6 and PhPDR1 defined a cluster where single candidates from *N. tomentiformis*, *N. sylvestris*, and *N. attenuata* are present. The characterization of these candidates might open the possibility for improving plant nutrient uptake and biomass production in *Nicotiana* species. Pepper and tomato spp. likely underwent duplication of PhPDR1 homologs as two candidates are present in each. An efficient strategy for investigating SL transport in these species, as well as in the tetraploid potato and tobacco might be therefore testing the involvement of these candidates in SL transport with (i) molecular and vesicular approaches and (ii) by targeting the candidates with a CRISPR/Cas9-based strategy thus to (iii) study the effect of eventual SL re-distribution directly *in planta*. MtPDR23 is a promising runner for SL transport in *Fabaceae*. The discovery of additional PhPDR1 orthologs in beans might allow new investigations in this clade, where only single candidates from pea, chickpea and mung bean are present. However, the unicity of these candidates might be as well due to the incomplete coverage of their genomes, e.g., 74% in chickpea (Varshney et al., 2013) and 82% in mung bean (Kang et al., 2014). Grasses were introduced into the phylogenetic analysis (Figure 4) together with known SL transporters and a clear separation was obtained from non-grasses candidates. This division suggests that no ABCG protein is involved into the regulation of SL transport in grasses, and that possibly other transporter families regulate SL transport and exudation in crops. Two branches close to MtPDR23 suggest that SL transporters belonging to the PhPDR1 family might be present also in grapevine, manioc and poplar. Despite the low amounts of sequence homologs to ABCD proteins regulating the transport of hormonal precursors (Supplementary Figure S3), the two barley homologs clearly indicate two rice proteins as putative candidates for functional conservation.

None of the characterized ABC class C proteins is a high affinity transporter for hormones, although hormones and their conjugate forms were identified in the vacuole. This is the reason why in this review a phylogenetic analysis was run on ABCC candidates with yet weakly characterized/unknown function (Figure 3). A CRISPR/Cas9 approach could be set to downregulate the expression of redundant homologs to these Arabidopsis ABCCs, thus to test with *in vitro*, vacuolar transport assays their affinity for hormonal substrates.

The phylogenetic isolation of GmPDR12 and Lr34 does not allow speculating about ortholog candidates for plant defense in other plant species, apart for suggesting a sorghum, yet uncharacterized protein. As well, the *Fabaceae* cluster comprising LjABCG1 and MtABCG10 is not informative on the function of barrel medic and soya proteins, as no substrates were isolated, yet.

Last but not least, although plant ABC transporters are considered being more specific than their human homologs, at least for their high abundance and wide diversification that occurred during evolution (Hwang et al., 2016), also in plants there are cases of strong substrate ambiguity. In addition to the already mentioned ABCC subfamily, AtABCG37 and ABA transporters, AtABCG36/PDR8/PEN3 is a robust example of absent substrate specificity. AtABCG36 has been reported involved in non-host resistance (Stein et al., 2006), sensitivity to IBA (Strader and Bartel, 2009), heavy metal toxicity resistance (Kim et al., 2007) and its overexpression has been shown to improve drought stress resistance by reducing sodium content in plants (Kim et al., 2010). Likely, rather than substrate specificity, AtABCG36 expression patterns, the factors regulating its activation and transcription levels and its possible recruitment for fast-reactive signaling pathways such as pathogen-resistance might have modeled its functions during evolution. As pointed out in Lefèvre and Boutry (2018), substrates of ABC transporters have been often suggested based on physiological data rather than on direct transport assays. Complementary approaches are needed to confirm supposed substrate specificities and to assay new ones, such as combinations of *in vitro* transport assays with monitoring of ATP hydrolysis in the presence of the ABC transporter of interest and the candidate substrate. In a near future, we foresee that automatized assays aimed to identify ABC transporter substrates, coupled to new knowledge on transcriptional and post-translational regulation of ABC proteins will shed new lights on ABC substrate specificity and ABC functions in plants.

## Author Contributions

JK and RdBF contributed significant parts of the manuscript. LB was the main coordinator and wrote large part of the manuscript.

## Conflict of Interest Statement

The authors declare that the research was conducted in the absence of any commercial or financial relationships that could be construed as a potential conflict of interest.

## Footnotes

1 1KP, https://sites.google.com/a/ualberta.ca/onekp/
