# Supplementary material for: Filling the Gap: Functional Clustering of ABC Proteins for the Investigation of Hormonal Transport in planta
Source: Front Plant Sci. 2019 Apr 17;10:422. doi: 10.3389/fpls.2019.00422 (PMC6479136; doi:10.3389/fpls.2019.00422)
Supplement: Supplementary file 2 [file Data_Sheet_1.docx]

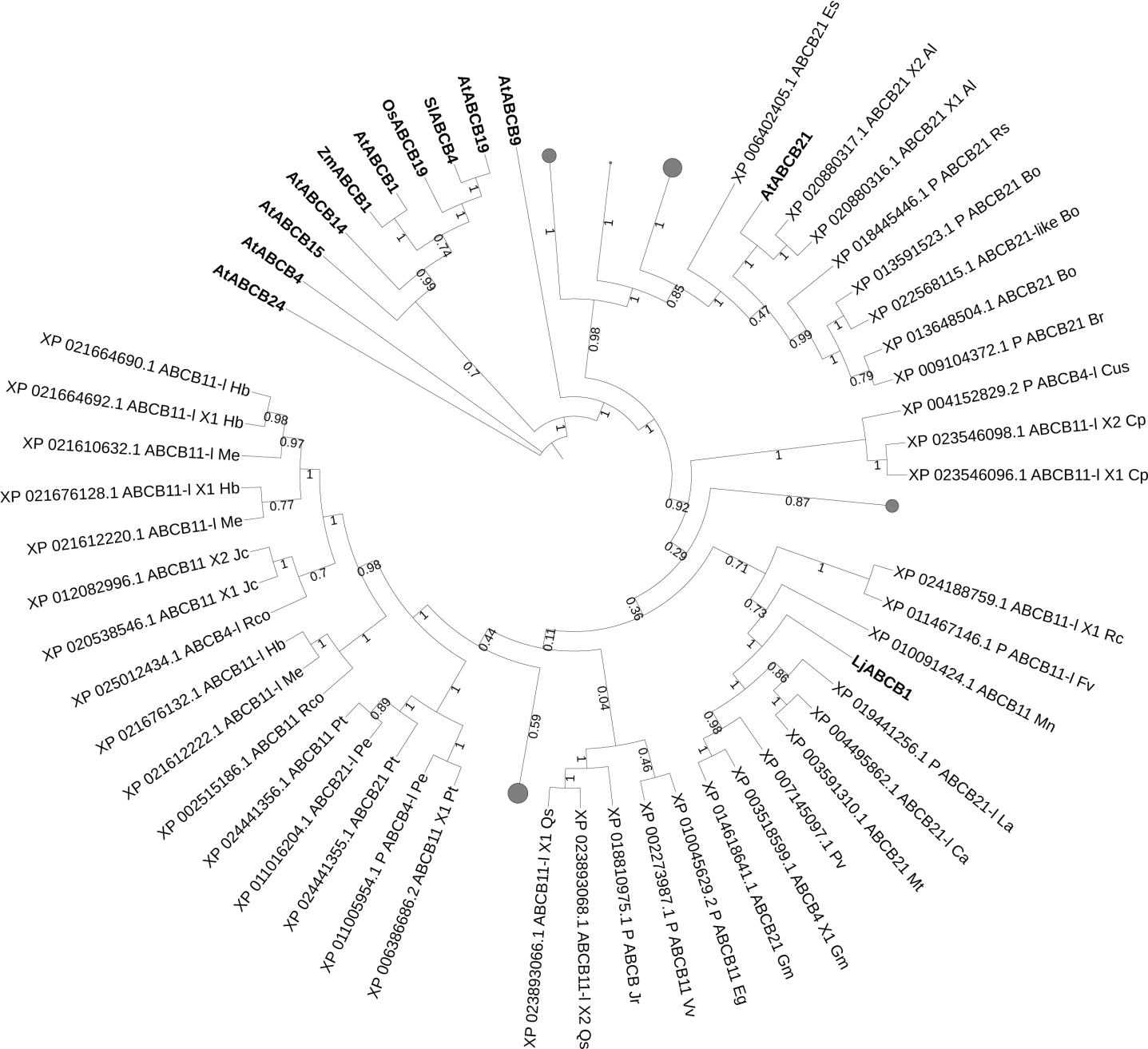


**Supplementary Figure S1. Best hits (sequence identity > 70%) from “land plants” BLAST category to characterized ABCB proteins.** *Arabidopsis lyrata (Al), Arabidopsis thaliana (At), Arachis duranensis (Ad), Arachis hypogaea (Ah), Arachis ipaensis (Ai), Brassica oleracea (Bo), Brassica rapa (Br), Brassican napus (Bn), Cajanus cajan (Ccaj), Camelina sativa (Cs), Capsella rubella (Cr), Capsicum annuum (Can), Cicer arietinum (Ca), Citrus clementina (Cc), Citrus sinensis (Csi), Coffea arabica (Car), Coffea eugenioides (Ce), Cucumis melo (Cm), Cucumis sativus (Csa), Cucurbita maxima (Cma), Cucurbita moschata (Cmo), Cucurbita pepo subsp. pepo (Cp), Cynara cardunculus var. scolymus (Cca), Daucus carota subsp. sativus (Dc), Durio zibethinus (Dz), Erythranthe guttata (Egu), Eucalyptus grandis (Eg), Eutrema salsugineum (Es), Fragaria vesca subsp. vesca (Fv), Glycine max (Gm), Gossypium arboreum (Ga), Gossypium hirsutum (Gh), Gossypium raimondii (Gr), Helianthus annuus (Ha), Herrania umbratica (Hu), Hevea brasiliensis (Hb), Ipomoea nil (In), Jatropha curcas (Jc), Juglans regia (Jr), Lactuca sativa (Ls), Lupinus angustifolius (La), Malus domestica (Md), Manihot esculenta (Me), Medicago truncatula (Mt), Momordica charantia (Mc), Morus notabilis (Mn), Musa acuminata subsp. malaccensis (Ma), Nelumbo nucifera (Nn), Nicotiana attenuata (Na), Nicotiana sylvestris (Ns), Nicotiana tabacum (Nt), Nicotiana tomentosiformis (Nto), Olea europaea (Oe), Phaseolus vulgaris (Pv), Populus euphratica (Pe), Populus trichocarpa (Pt), Prunus avium (Pa), Pyrus bretschneideri (Pb), Quercus suber (Qs), Raphanus sativus (Rs), Ricinus communis (Rco), Rosa chinensis (Rc), Sesamum indicum (Si), Solanum lycopersicum (Sl), Solanum pennellii (Sp), Solanum tuberosum (St), Tarenaya hassleriana (Th), Theobroma cacao (Tc), Vigna angularis (Va), Vigna radiata var. radiata (Vr), Vitis vinifera (Vv)*  . P: predicted: -l: -like. Bootstrap n: 100. No branches deleted as bootstrap values in branches of interest > 0.4. Maximum 3 isoforms displayed for size restriction. Grey circles (proportionally sized) represent collapsed nodes for size restrictionThe tree with the highest log likelihood (-65224.36) is shown. The analysis involved 111 amino acid sequences. There were a total of 1636 positions in the final dataset.


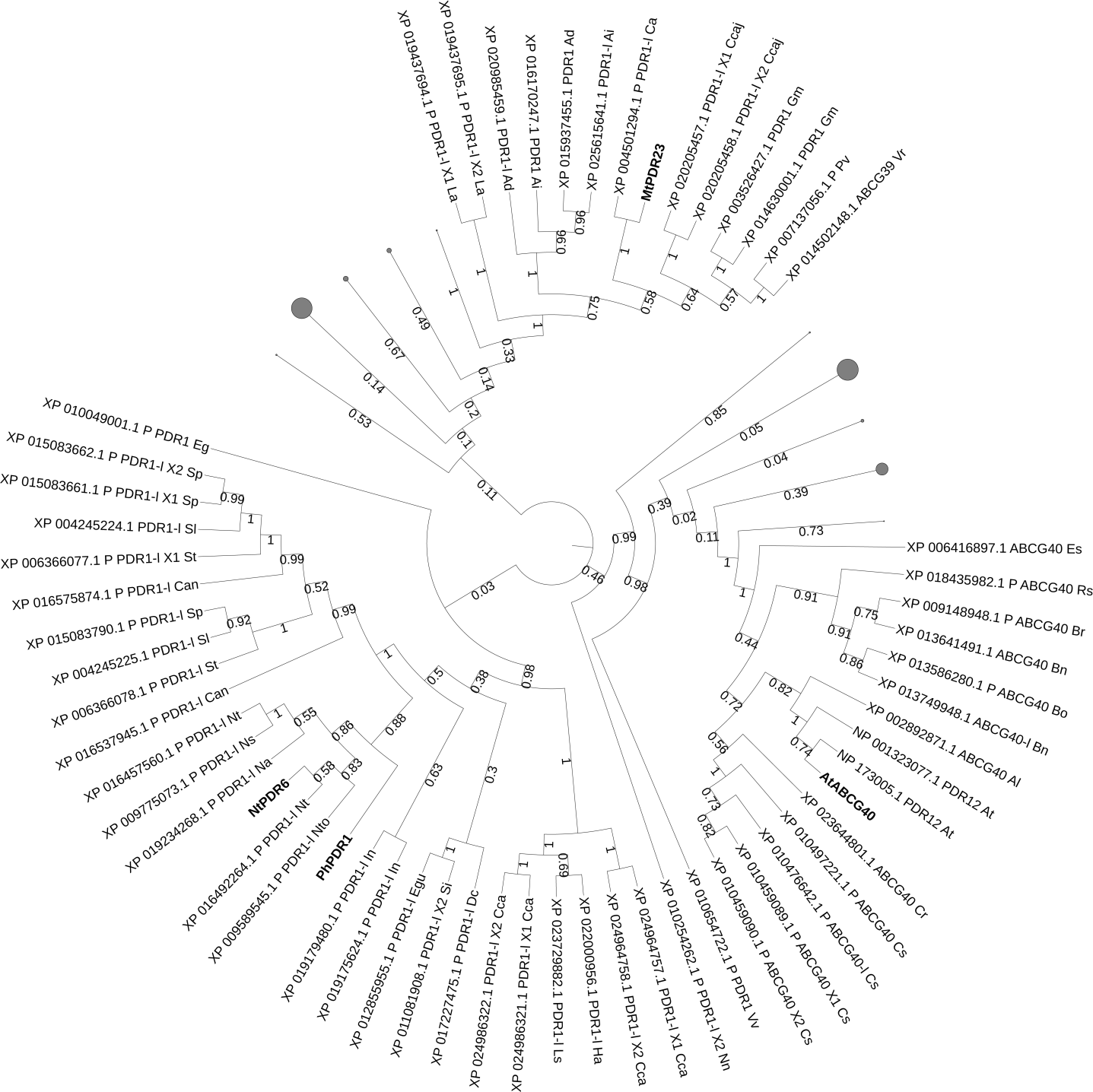


**Supplementary Figure S2. Best hits (sequence identity > 70%) from “land plants” to characterized SL transporters plus AtABCG40.** *Arabidopsis lyrata (Al), Arabidopsis thaliana (At), Arachis duranensis (Ad), Arachis hypogaea (Ah), Arachis ipaensis (Ai), Brassica oleracea (Bo), Brassica rapa (Br), Brassican napus (Bn), Cajanus cajan (Ccaj), Camelina sativa (Cs), Capsella rubella (Cr), Capsicum annuum (Can), Cicer arietinum (Ca), Citrus clementina (Cc), Citrus sinensis (Csi), Coffea arabica (Car), Coffea eugenioides (Ce), Cucumis melo (Cm), Cucumis sativus (Csa), Cucurbita maxima (Cma), Cucurbita moschata (Cmo), Cucurbita pepo subsp. pepo (Cp), Cynara cardunculus var. scolymus (Cca), Daucus carota subsp. sativus (Dc), Durio zibethinus (Dz), Erythranthe guttata (Egu), Eucalyptus grandis (Eg), Eutrema salsugineum (Es), Fragaria vesca subsp. vesca (Fv), Glycine max (Gm), Gossypium arboreum (Ga), Gossypium hirsutum (Gh), Gossypium raimondii (Gr), Helianthus annuus (Ha), Herrania umbratica (Hu), Hevea brasiliensis (Hb), Ipomoea nil (In), Jatropha curcas (Jc), Juglans regia (Jr), Lactuca sativa (Ls), Lupinus angustifolius (La), Malus domestica (Md), Manihot esculenta (Me), Medicago truncatula (Mt), Momordica charantia (Mc), Morus notabilis (Mn), Musa acuminata subsp. malaccensis (Ma), Nelumbo nucifera (Nn), Nicotiana attenuata (Na), Nicotiana sylvestris (Ns), Nicotiana tabacum (Nt), Nicotiana tomentosiformis (Nto), Olea europaea (Oe), Phaseolus vulgaris (Pv), Populus euphratica (Pe), Populus trichocarpa (Pt), Prunus avium (Pa), Pyrus bretschneideri (Pb), Quercus suber (Qs), Raphanus sativus (Rs), Ricinus communis (Rco), Rosa chinensis (Rc), Sesamum indicum (Si), Solanum lycopersicum (Sl), Solanum pennellii (Sp), Solanum tuberosum (St), Tarenaya hassleriana (Th), Theobroma cacao (Tc), Vigna angularis (Va), Vigna radiata var. radiata (Vr), Vitis vinifera (Vv)* . P: predicted: -l: -like. Bootstrap n: 100. No branches deleted as bootstrap values in branches of interest > 0.4. Maximum 3 isoforms displayed for size restriction. Grey circles (proportionally sized) represent collapsed nodes for size restriction. The tree with the highest log likelihood (-49247.73) is shown. The analysis involved 202 amino acid sequences. All positions containing gaps and missing data were eliminated. There were a total of 828 positions in the final dataset.

**
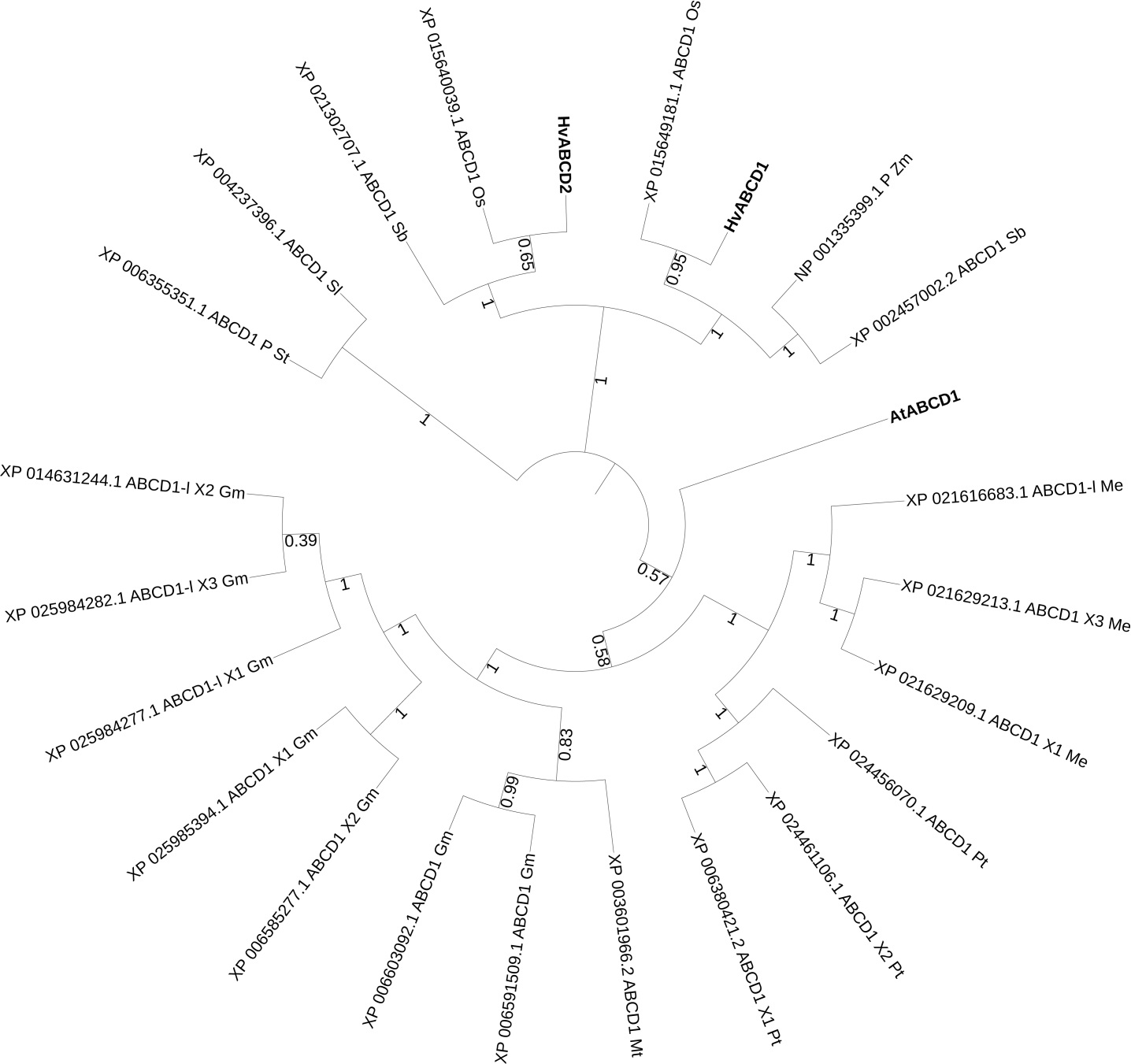
**

**Supplemental Figure S3. Best hits (sequence identity > 70%) to ABC proteins regulating hormonal precursors comprised into the lipid transporter clusters of ABC protein from Figure 1.** Sequences from *Glycine max* (*Gm*, soya bean), *Populus trichocarpa* (*Pt*, poplar), *Manihot esculenta* (*Me*, manioc), *Solanum lycopersicum* and t*uberosum* (*Sl,* tomato and *St*, potato), *Medicago truncatula* (*Mt*, barrel medic), *Vitis vinifera* (*Vv*, grape vine), *Triticum aestivum* (*Ta*, wheat), *Oryza sativa* (*Os*, rice), *Lotus japonicus* (*Lj*), *Physcomitrella patens* (*Pp*), *Marchantia polymorpha* (*Mp*), *Hordeum vulgare* (*Hv*, barley), *Sorghum bicolor* (*Sb*) and *Zea mays* (*Zm*, maize). P: predicted: -l: -like. Bootstrap n: 100. Branches with bootstrap values < 0.4 are deleted. Maximum 3 isoforms displayed for size restriction. Grey circles (proportionally sized) represent collapsed nodes for size restriction. The tree with the highest log likelihood (-18738.18) is shown. The analysis involved 24 amino acid sequences. There were a total of 2084 positions in the final dataset.

**Supplementary Material and Methods**

Protein sequences (NCBI Protein Reference Sequences updated to 2019/01/11) were retrieved from NCBI and are presented as supplementary text files in FASTA format in *Supplementary sequence source.PDF*. Blast searches were run either against land plants (taxid:3193) or a selected collection of model / crop plants as follows: *Glycine max* (taxid:3847) + *Populus trichocarpa* (taxid:3694) + *Manihot esculenta* (taxid:3983) + *Solanum lycopersicum* (taxid:4081) + *Solanum tuberosum* (taxid:4113) + *Medicago truncatula* (taxid:3880) + *Vitis vinifera* (taxid:29760) + *Triticum aestivum* (taxid:4565) + *Oryza sativa* (taxid:4530) + *Lotus japonicus* (taxid:34305) + *Physcomitrella patens* (taxid:3218) + *Marchantia polymorpha* (taxid:3197) + *Hordeum vulgare* (taxid:4513) + *Sorghum bicolor* (taxid:4558) + *Zea mays* (taxid:4577).

Unique sequences were aligned via Clustal X 2.1 (Larkin et al., 2007) with no iteration and default parameters. Maximum likelihood (bootstrap n set to 100) was applied to realize the tree output in MEGA7 (Kumar et al., 2015). The tree with the highest log likelihood is shown Tree visualization and design was obtained through iTOL (Letunic and Bork, 2016). Sequences from branches that were collapsed because of visualization restrictions, when present are in *Supplementary Table 1*.

**Supplementary Literature**

Larkin, M.A., Blackshields, G., Brown, N.P., Chenna, R., McGettigan, P.A., McWilliam, H., et al. (2007). Clustal W and Clustal X version 2.0. *Bioinformatics* 23(21)**,** 2947-2948. doi: 10.1093/bioinformatics/btm404.

Letunic, I., and Bork, P. (2016). Interactive tree of life (iTOL) v3: an online tool for the display and annotation of phylogenetic and other trees. *Nucleic Acids Research* 44(W1)**,** W242-W245. doi: 10.1093/nar/gkw290.

Kumar S., Stecher G., and Tamura K. (2016) MEGA7: Molecular Evolutionary Genetics Analysis version 7.0 for bigger datasets, *Molecular Biology and Evolution*, 33 (7) 1870–1874. doi: 10.1093/molbev/msw054.
