# Supplementary material for: Filling the Gap: Functional Clustering of ABC Proteins for the Investigation of Hormonal Transport in planta
Source: Front Plant Sci. 2019 Apr 17;10:422. doi: 10.3389/fpls.2019.00422 (PMC6479136; doi:10.3389/fpls.2019.00422)
Supplement: Supplementary file 3 [file Data_Sheet_2.pdf]

Supplementary Material and Methods

>AtABCG9
MDNQEVSMDVETPIAKTNDDRSLPFSIFKKANNPVTLKFENLVYTVKLKDSQGCFGKNDK
TEERTILKGLTGIVKPGEILAMLGPSGSGKTSLLTALGGRVGEGKGKLTGNISYNNKPLS
KAVKRTTGFVTQDDALYPNLTVTETLVFTALLRLPNSFKKQEKIKQAKAVMTELGLDRCK
DTIIGGPFLRGVSGGERKRVSIGQEILINPSLLFLDEPTSGLDSTTAQRIVSILWELARG
GRTVVTTIHQPSSRLFYMFDKLLLLSEGNPVYFGLGSNAMDYFASVGYSPLVERINPSDF
LLDIANGVGSDESQRPEAMKAALVAFYKTNLLDSVINEVKGQDDLCNKPRESSRVATNTY
GDWPTTWWQQFCVLLKRGLKQRRHDSFSGMKVAQIFIVSFLCGLLWWQTKISRLQDQIGL
LFFISSFWAFFPLFQQIFTFPQERAMLQKERSSGMYRLSPYFLSRVVGDLPMELILPTCF
LVITYWMAGLNHNLANFFVTLLVLLVHVLVSGGLGLALGALVMDQKSATTLGSVIMLTFL
LAGGYYVQHVPVFISWIKYVSIGYYTYKLLILGQYTANELYPCGDNGKLRCHVGDFEGIK
HIGFNSGLVSALALTAMLVVYRVIAYIALTRIGKTKSG
>AtABCG36
MDYNPNLPPLGGGGVSMRRSISRSVSRASRNIEDIFSSGSRRTQSVNDDEEALKWAAIEK
LPTYSRLRTTLMNAVVEDDVYGNQLMSKEVDVTKLDGEDRQKFIDMVFKVAEQDNERILT
KLRNRIDRVGIKLPTVEVRYEHLTIKADCYTGNRSLPTLLNVVRNMGESALGMIGIQFAK
KAQLTILKDISGVIKPGRMTLLLGPPSSGKTTLLLALAGKLDKSLQVSGDITYNGYQLDE
FVPRKTSAYISQNDLHVGIMTVKETLDFSARCQGVGTRYDLLNELARREKDAGIFPEADV
DLFMKASAAQGVKNSLVTDYTLKILGLDICKDTIVGDDMMRGISGGQKKRVTTGEMIVGP
TKTLFMDEISTGLDSSTTFQIVKCLQQIVHLNEATVLMSLLQPAPETFDLFDDIILVSEG
QIVYQGPRDNILEFFESFGFKCPERKGTADFLQEVTSKKDQEQYWVNPNRPYHYIPVSEF
ASRYKSFHVGTKMSNELAVPFDKSRGHKAALVFDKYSVSKRELLKSCWDKEWLLMQRNAF
FYVFKTVQIVIIAAITSTLFLRTEMNTRNEGDANLYIGALLFGMIINMFNGFAEMAMMVS
RLPVFYKQRDLLFYPSWTFSLPTFLLGIPSSILESTAWMVVTYYSIGFAPDASRFFKQFL
LVFLIQQMAASLFRLIASVCRTMMIANTGGALTLLLVFLLGGFLLPKGKIPDWWGWAYWV
SPLTYAFNGLVVNEMFAPRWMNKMASSNSTIKLGTMVLNTWDVYHQKNWYWISVGALLCF
TALFNILFTLALTYLNPLGKKAGLLPEEENEDADQGKDPMRRSLSTADGNRRGEVAMGRM
SRDSAAEASGGAGNKKGMVLPFTPLAMSFDDVKYFVDMPGEMRDQGVTETRLQLLKGVTG
AFRPGVLTALMGVSGAGKTTLMDVLAGRKTGGYIEGDVRISGFPKVQETFARISGYCEQT
DIHSPQVTVRESLIFSAFLRLPKEVGKDEKMMFVDQVMELVELDSLRDSIVGLPGVTGLS
TEQRKRLTIAVELVANPSIIFMDEPTSGLDARAAAIVMRAVRNTVDTGRTVVCTIHQPSI
DIFEAFDELMLMKRGGQVIYAGPLGQNSHKVVEYFESFPGVSKIPEKYNPATWMLEASSL
AAELKLSVDFAELYNQSALHQRNKALVKELSVPPAGASDLYFATQFSQNTWGQFKSCLWK
QWWTYWRSPDYNLVRFIFTLATSLLIGTVFWQIGGNRSNAGDLTMVIGALYAAIIFVGIN
NCSTVQPMVAVERTVFYRERAAGMYSAMPYAISQVTCELPYVLIQTVYYSLIVYAMVGFE
WKAEKFFWFVFVSYFSFLYWTYYGMMTVSLTPNQQVASIFASAFYGIFNLFSGFFIPRPK
IPKWWIWYYWICPVAWTVYGLIVSQYGDVETRIQVLGGAPDLTVKQYIEDHYGFQSDFMG
PVAAVLIAFTVFFAFIFAFCIRTLNFQTR
>AtABCG1
MARIVAANDDDSMELNTISSIHDSTLGQLLKNVSDVRKMAIGDETPVHESLNQDYNDGYM
RTVPFVLSFDNLTYNVSVRPKLDFRNLFPRRRTEDPEIAQTARPKTKTLLNNISGETRDG
EIMAVLGASGSGKSTLIDALANRIAKGSLKGTVKLNGETLQSRMLKVISAYVMQDDLLFP
MLTVEETLMFAAEFRLPRSLPKSKKKLRVQALIDQLGIRNAAKTIIGDEGHRGISGGERR
RVSIGIDIIHDPILLFLDEPTSGLDSTSAFMVVKVLKRIAQSGSIVIMSIHQPSHRVLGL
LDRLIFLSRGHTVYSGSPASLPRFFTEFGSPIPENENRTEFALDLIRELEGSAGGTRGLI
EFNKKWQEMKKQSNRQPPLTPPSSPYPNLTLKEAIAASISRGKLVSGGESVAHGGATTNT
TTLAVPAFANPMWIEIKTLSKRSMLNSRRQPELFGIRIASVVITGFILATVFWRLDNSPK
GVQERLGFFAFAMSTMFYTCADALPVFLQERYIFMRETAYNAYRRSSYVLSHAIVSFPSL
IFLSVAFAATTYWAVGLDGGLTGLLFYCLIILASFWSGSSFVTFLSGVVPSVMLGYTIVV
AILAYFLLFSGFFINRNRIPDYWIWFHYMSLVKYPYEAVLQNEFSDATKCFVRGVQIFDN
TPLGELPEVMKLKLLGTVSKSLGVTISSTTCLTTGSDILRQQGVVQLSKWNCLFITVAFG
FFFRILFYFTLLLGSKNKRR
>AtABCG16

MSRILVEDDNATPFHSMEIISSSLTLGQLLKNVSDVRKVEVGDETPVHEFFDRDGSSLDG
DNDHLMRPVPFVLSFNNLTYNVSVRRKLDFHDLVPWRRTSFSKTKTLLDNISGETRDGEI
LAVLGASGSGKSTLIDALANRIAKGSLKGTVTLNGEALQSRMLKVISAYVMQDDLLFPML
TVEETLMFAAEFRLPRSLPKSKKKLRVQALIDQLGIRNAAKTIIGDEGHRGISGGERRRV
SIGIDIIHDPIVLFLDEPTSGLDSTSAFMVVKVLKRIAESGSIIIMSIHQPSHRVLSLLD
RLIFLSRGHTVFSGSPASLPSFFAGFGNPIPENENQTEFALDLIRELEGSAGGTRGLVEF
NKKWQEMKKQSNPQTLTPPASPNPNLTLKEAISASISRGKLVSGGGGGGSSVINHGGGTLA
VPAFANPFWIEIKTLTRRSILNSRRQPELLGMRLATVIVTGFILATVFWRLDNSPKGVQE
RLGFFAFAMSTMFYTCADALPVFLQERYIFMRETAYNAYRRSSYVLSHAIVTFPSLIFLS
LAFAVTTFWAVGLEGGLMGFLFYCLIILASFWSGSSFVTFLSGVVPHVMLGYTIVVAILA
YFLLFSGFFINRDRIPQYWIWFHYLSLVKYPYEAVLQNEFSDPTECFVRGVQLFDNSPLG
ELTYGMKLRLLDSVSRSIGMRISSSTCLTTGADVLKQQGVTQLSKWNCLLITVGFGFLFR
ILFYLCLLLGSKNKRR
>AtABCG11
MEIEASRQQTTVPVSVGGGNFPVGGLSPLSEAIWREKAPTEFVGDVSARLTWQDLTVMVT
MGDGETQNVLEGLTGYAEPGSLTALMGPSGSGKSTMLDALASRLAANAFLSGTVLLNGRK
TKLSFGTAAYVTQDDNLIGTLTVRETIWYSARVRLPDKMLRSEKRALVERTIIEMGLQDC
ADTVIGNWHLRGISGGEKRRVSIALEILMRPRLLFLDEPTSGLDSASAFFVTQTLRALSR
DGRTVIASIHQPSSEVFELFDRLYLLSGGKTVYFGQASDAYEFFAQAGFPCPALRNPSDH
FLRCINSDFDKVRATLKGSMKLRFEASDDPLEKITTAEAIRLLVDYYHTSDYYYTAKAKV
EEISQFKGTILDSGGSQASFLLQTYTLTKRSFINMSRDFGYYWLRLLIYILVTVCIGTIY
LNVGTSYSAILARGSCASFVFGFVTFMSIGGFPSFVEDMKVFQRERLNGHYGVAAFVIAN
TLSATPFLIMITFISGTICYFMVGLHPGFTHYLFFVLCLYASVTVVESLMMAIASIVPNF
LMGIIIGAGIQGIFMLVSGFFRLPNDIPKPFWRYPMSYISFHFWALQGQYQNDLRGLTFD
SQGSAFKIPGEYVLENVFQIDLHRSKWINLSVILSMIIIYRIIFFIMIKTNEDVTPWVRG
YIARRRMKQKNGTQNTTVAPDGLTQSPSLRNYIATRTDGARRW
>AtABCG22
MSMEKPPLASGLARTRSEQLYETVAADIRSPHGSMDANGVPATAPAAVGGGGTLSRKSSR
RLMGMSPGRSSGAGTHIRKSRSAQLKLELEEVSSGAALSRASSASLGLSFSFTGFAMPPE
EISDSKPFSDDEMIPEDIEAGKKKPKFQAEPTLPIFLKFRDVTYKVVIKKLTSSVEKEIL
TGISGSVNPGEVLALMGPSGSGKTTLLSLLAGRISQSSTGGSVTYNDKPYSKYLKSKIGF
VTQDDVLFPHLTVKETLTYAARLRLPKTLTREQKKQRALDVIQELGLERCQDTMIGGAFV
RGVSGGERKRVSIGNEIIINPSLLLLDEPTSGLDSTTALRTILMLHDIAEAGKTVITTIH
QPSSRLFHRFDKLILLGRGSLLYFGKSSEALDYFSSIGCSPLIAMNPAEFLLDLANGNIN
DISVPSELDDRVQVGNSGRETQTGKPSPAAVHEYLVEAYETRVAEQEKKKLLDPVPLDEE
AKAKSTRLKRQWGTCWWEQYCILFCRGLKERRHEYFSWLRVTQVLSTAVILGLLWWQSDI
RTPMGLQDQAGLLFFIAVFWGFFPVFTAIFAFPQERAMLNKERAADMYRLSAYFLARTTS
DLPLDFILPSLFLLVVYFMTGLRISPYPFFLSMLTVFLCIIAAQGLGLAIGAILMDLKKA
TTLASVTVMTFMLAGGFFVKKVPVFISWIRYLSFNYHTYKLLLKVQYQDFAVSINGMRID
NGLTEVAALVVMIFGYRLLAYLSLRQMKIVT
>AtABCG29
METLSRSLSKSLGELLASNSNNHFSRRSGSTIDDHDEEALKWAALEKLPTFARLRTTIIH
PHEDLVDVTKLGVDDRQKFIDSIFKVTEEDNEKFLKKFRNRIDRVRIKLPTVEVRFEKVT
IEANCHIGKRALPTLPNAALNIAERGLRLLGFNFTKTTKVTILRDVSGIIKPSRMTLLLG
PPSSGKTTLLLALAGKLDQSLKVTGRVTYNGHGLEEFVPQKTSAYISQNDVHVGVMTVQE
TLDFSARCQGVGTRYDLLSELVRREKDAGILPEPEVDLFMKSIAAGNVKSSLITDYTLRI
LGLDICKDTVVGDEMIRGISGGQKKRVTTGEMIVGPTKTLFMDEISTGLDSSTTYQIVKC
LQEIVRFTDATVLMSLLQPAPETFELFDDIILLSEGQIVYQGPRDHVLTFFETCGFKCPD
RKGTADFLQEVTSRKDQEQYWADSKKPYSYISVSEFSKRFRTFHVGANLEKDLSVPYDRF
KSHPASLVFKKHSVPKSQLFKVCWDRELLLMKRNAFFYITKTVQIIIMALIASTVYLRTE
MGTKNESDGAVYIGALMFSMIVNMFNGFAELALMIQRLPVFYKQRDLLFHPPWTFSLPTF
LLGIPISIFESVVWVTITYYMIGFAPELSRFLKHLLVIFLTQQMAGGIFRFIAATCRSMI
LANTGGALVILLLFLLGGFIVPRGEIPKWWKWAYWVSPMAYTYDALTVNEMLAPRWINQP
SSDNSTSLGLAVLEIFDIFTDPNWYWIGVGGILGFTVLFNILVTLALTFLNPLEKQQAVV

SKENTEENRAENGSKSKSIDVKRGMVLPFTPLTMSFDNVNYYVDMPKEMKEQGVSKDKLQ
LLKEVTGVFRPGVLTALMGVSGAGKTTLMDVLAGRKTGGYIEGDIRISGFPKRQETFARI
SGYCEQNDIHSPQVTVKESLIYSAFLRLPKEVTKYEKMRFVDEVMELVELESLKDAVVGL
PGITGLSTEQRKRLTIAVELVANPSIIFMDEPTSGLDARAAAIVMRTVRNTVDTGRTVVC
TIHQPSIDIFEAFDELLLLKRGGQVIYAGPLGQNSHKIIEYFQAIHGVPKIKEKYNPATW
MLEVSSMAAEAKLEIDFAEHYKTSSLYQQNKNLVKELSTPPQGASDLYFSTRFSQSLLGQ
FKSCLWKQWITYWRTPDYNLARFFFTLAAAVMLGSIFWKVGTKRENANDLTKVIGAMYAA
VLFVGVNNSSSVQPLIAVERSVFYRERAAEMYSALPYALAQVVCEIPYVLIQTTYYTLII
YAMMCFEWTLAKFFWFYFVSFMSFLYFTYYGMMTVALTPNQQVAAVFAGAFYGLFNLFSG
FVIPRPRIPKWWIWYYWICPVAWTVYGLIVSQYGDVEDTIKVPGMANDPTIKWYIENHYG
YDADFMIPIATVLVGFTLFFAFMFAFGIRTLNFQQR
>SlABCB4
maettegksmpeaekkkeqslpfyqlfsfadkydyllmtcgsigailhgssmpvffllfg
emvngfgknqmdlhkmthevskyalyfvylglivcassyaeigcwmytgerqvsalrkky
leavlkqdvgffdtdartgdivfsvstdtllvqdaisekvgnfihylstflaglvvgfvs
awrlallsvavipgiafagglyaytltgltsksresyanagiiaeqaiaqvrtvysyvge
tkalnsysdaiqntlklgykagmakglglgctygiacmswalvfwyagvfirngqsdggk
aftaifsaivggmslgqsfsnlgafskgkaagyklmeiikqkptivqdtldgkclsevsg
niefknvtfsypsrpdviifrdfciffpagktvavvggsgsgkstvvslierfydpndgq
vlldnvdiktlqlrwlrdqiglvnqepalfattilenilygkpdatmaeveaatcasnah
nfitllpngyntqvgergvqlsggqkqriaiaramlknpkillldeatsaldagsesivq
ealdrlmvgrttvvvahrlstirnvdsiaviqqgqvvetgtheeliskagayaslirfqe
mvgnrdfsnpstrrtrstrlshslstkslslrsgslrnlsysystgadgriemisnaetd
rknpapqnyfcrllklnapewpysimgavgsvlsgfigptfaivmsnmievfyytnpatm
erktkeyvfiyigaglyavvayliqhyffsimgenlttrvrrmmlaailrnevgwfdeee
nnssllaarlatdaadvksaiaerisvilqnmtslltsfivafivewrvsllilatfpll
vlanfaqqlslkgfagdtakahaktsmiagegvsnirtvaafnaqekiislfsqelrvpq
mqslrrsqmsgllfgisqlalygsealilwygahlvnngvstfskvikvfvvlvitansv
aetvslapeiirggeavgsvfsildrstrvdpddpegdpvesirgdielrhvdfaypsrp
dvsvfkdlnlriragqsqalvgasgsgkssvialierfydptggkvmidgkdirrlnlks
lrlkiglvqqepalfaasifeniaygkegateaevieaaraanvhtfvsglpegyktpvg
ergvqlsggqkqriaiaravlkdpsillldeatsaldaesecvlqealerlmrgrttvlv
ahrlstirnvdtigvvqdgriveqgshselisrpegaysrllqlqhhri
>AtABCB28
MASATTLLFHHGSTRVLVARRRCQASVLRPYGGLKPFLSFCSLPNSTAPFRDSLRAKSDG
LARAYVTGAPPIVEEPDPKIEESKSEAESKDLISWGLLWSLMSKHKLRLSVCLLTLLGCS
TCTLSMPVFSGRFFEVLIGVRPEPLWRLLSKIAVLYSLEPIFTIAFVTNMTAIWENVMAI
LRAQIFRRVLIQKAEFFDKYKVGELTGLLTSDLGALNSIVNDNISRDRGFRAFTEVFGTI
CILFTLSPQLAPVLGLLMLAVSVLVAVYKRSTVPVYKSHGLAQATMSDCVSETFSAIRTV
RSFSGEKRQMSIFGSQILAYKLSGLKLGTFKSINESITRVAVYISLLALYCLGGSKVKTG
ELAVGTVVSFIGYTFTLTFAVQGLVNTFGDLRGTFAAIDRINSILNAVDIDEALAYGLER
DIHTKKVQDENLKLFLSAGPNVNIRHLDKYYMSNLKSTNNLRTLTWAGDVCLDDVHFAYP
LRPDVKVLDGLSLTLNSGTVTALVGSSGAGKSTIVQLLARFYEPTQGRITVGGEDVRMFD
KSEWAKVVSIVNQEPVLFSLSVAENIAYGLPNEHVSKDDIIKAAKAANAHDFIISLPQGY
DTLVGERGGLLSGGQRQRVAIARSLLKNAPILILDEATSALDAVSERLVQSALNRLMKDR
TTLVIAHRLSTVQSANQIAVCSDGKIIELGTHSELVAQKGSYASLVGTQRLAFE
>AtABCB26
MAQQVLGCTSRPIRVSLHRCSVITTSDTIRRKNLRFVRNPRLSFSLQSSTRNYRLPSINC
STVNGAVAETAEYYEGEGDNVSLAEKIRQCIDFLRTILPGGSWWSFSDEVDGRFIAKPVT
VWRALSRMWELVAEDRWVIFAAFSTLIVAALSEITIPHFLTASIFSAQSGDIAVFHRNVK
LLVTLCVTSGICSGIRGCFFGIANMILVKRMRETLYSTLLFQDISFFDSQTVGDLTSRLG
SDCQQVSRVIGNDLNMIFRNVLQGTGALIYLLILSWPLGLCTLVICCILAAVMFVYGMYQ
KKTAKLIQEITASANEVAQETYSLMRTVRVYGTEKQEFKRYNHWLQRLADISLRQSAAYG
IWNWSFNTLYHATQIIAVLVGGLSILAGQITAEQLTKFLLYSEWLIYATWWVGDNLSSLM

QSVGASEKVFQMMDLKPSDQFISKGTRLQRLTGHIEFVDVSFSYPSRDEVAVVQNVNISV
HPGEVVAIVGLSGSGKSTLVNLLLQLYEPTSGQILLDGVPLKELDVKWLRQRIGYVGQEP
KLFRTDISSNIKYGCDRNISQEDIISAAKQAYAHDFITALPNGYNTIVDDDLLSGGQKQR
IAIARAILRDPRILILDEATSALDAESEHNVKGVLRSIGNDSATKRSVIVIAHRLSTIQA
ADRIVAMDSGRVVEMGSHKELLSKDGLYARLTKRQNDAVL
>AtABCB4
MASESGLNGDPNILEEVSETKRDKEEEEEVKKTEKKDEEHEKTKTVPFYKLFAFADSFDF
LLMILGTLGSIGNGLGFPLMTLLFGDLIDAFGENQTNTTDKVSKVALKFVWLGIGTFAAA
FLQLSGWMISGERQAARIRSLYLKTILRQDIAFFDIDTNTGEVVGRMSGDTVLIQDAMGE
KVGKAIQLLATFVGGFVIAFVRGWLLTLVMLSSIPLLVMAGALLAIVIAKTASRGQTAYA
KAATVVEQTIGSIRTVASFTGEKQAISNYNKHLVTAYKAGVIEGGSTGLGLGTLFLVVFC
SYALAVWYGGKLILDKGYTGGQVLNIIIAVLTGSMSLGQTSPCLSAFAAGQAAAYKMFET
IERRPNIDSYSTNGKVLDDIKGDIELKDVYFTYPARPDEQIFRGFSLFISSGTTVALVGQ
SGSGKSTVVSLIERFYDPQAGDVLIDGINLKEFQLKWIRSKIGLVSQEPVLFTASIKDNI
AYGKEDATTEEIKAAAELANASKFVDKLPQGLDTMVGEHGTQLSGGQKQRIAVARAILKD
PRILLLDEATSALDAESERVVQEALDRIMVNRTTVVVAHRLSTVRNADMIAVIHQGKIVE
KGSHTELLKDPEGAYSQLIRLQEEKKSDENAAEEQKMSSIESFKQSSLRKSSLGRSLSKG
GSSRGNSSRHSFNMFGFPAGIDGNVVQDQEEDDTTQPKTEPKKVSIFRIAALNKPEIPVL
ILGSISAAANGVILPIFGILISSVIKAFFQPPKKLKEDTSFWAIIFMVLGFASIIAYPAQ
TFFFAIAGCKLVQRIRSMCFEKVVHMEVGWFDEPENSSGTIGARLSADAATIRGLVGDSL
AQTVQNLSSILAGLIIAFLACWQLAFVVLAMLPLIALNGFLYMKFMKGFSADAKKMYGEA
SQVANDAVGSIRTVASFCAEDKVMNMYSKKCEGPMKNGIRQGIVSGIGFGFSFFVLFSSY
AASFYVGARLVDDGKTTFDSVFRVFFALTMAAMAISQSSSLSPDSSKADVAAASIFAIMD
RESKIDPSVESGRVLDNVKGDIELRHVSFKYPARPDVQIFQDLCLSIRAGKTVALVGESG
SGKSTVIALLQRFYDPDSGEITLDGVEIKSLRLKWLRQQTGLVSQEPILFNETIRANIAY
GKGGDASESEIVSSAELSNAHGFISGLQQGYDTMVGERGIQLSGGQKQRVAIARAIVKDP
KVLLLDEATSALDAESERVVQDALDRVMVNRTTIVVAHRLSTIKNADVIAVVKNGVIVEK
GKHDTLINIKDGVYASLVQLHLTAAS
>AtABCB1
MDNDGGAPPPPPTLVVEEPKKAEIRGVAFKELFRFADGLDYVLMGIGSVGAFVHGCSLPL
FLRFFADLVNSFGSNSNNVEKMMEEVLKYALYFLVVGAAIWASSWAEISCWMWSGERQTT
KMRIKYLEAALNQDIQFFDTEVRTSDVVFAINTDAVMVQDAISEKLGNFIHYMATFVSGF
IVGFTAVWQLALVTLAVVPLIAVIGGIHTTTLSKLSNKSQESLSQAGNIVEQTVVQIRVV
MAFVGESRASQAYSSALKIAQKLGYKTGLAKGMGLGATYFVVFCCYALLLWYGGYLVRHH
LTNGGLAIATMFAVMIGGLALGQSAPSMAAFAKAKVAAAKIFRIIDHKPTIERNSESGVE
LDSVTGLVELKNVDFSYPSRPDVKILNNFCLSVPAGKTIALVGSSGSGKSTVVSLIERFY
DPNSGQVLLDGQDLKTLKLRWLRQQIGLVSQEPALFATSIKENILLGRPDADQVEIEEAA
RVANAHSFIIKLPDGFDTQVGERGLQLSGGQKQRIAIARAMLKNPAILLLDEATSALDSE
SEKLVQEALDRFMIGRTTLIIAHRLSTIRKADLVAVLQQGSVSEIGTHDELFSKGENGVY
AKLIKMQEAAHETAMSNARKSSARPSSARNSVSSPIMTRNSSYGRSPYSRRLSDFSTSDF
SLSIDASSYPNYRNEKLAFKDQANSFWRLAKMNSPEWKYALLGSVGSVICGSLSAFFAYV
LSAVLSVYYNPDHEYMIKQIDKYCYLLIGLSSAALVFNTLQHSFWDIVGENLTKRVREKM
LSAVLKNEMAWFDQEENESARIAARLALDANNVRSAIGDRISVIVQNTALMLVACTAGFV
LQWRLALVLVAVFPVVVAATVLQKMFMTGFSGDLEAAHAKGTQLAGEAIANVRTVAAFNS
EAKIVRLYTANLEPPLKRCFWKGQIAGSGYGVAQFCLYASYALGLWYASWLVKHGISDFS
KTIRVFMVLMVSANGAAETLTLAPDFIKGGQAMRSVFELLDRKTEIEPDDPDTTPVPDRL
RGEVELKHIDFSYPSRPDIQIFRDLSLRARAGKTLALVGPSGCGKSSVISLIQRFYEPSS
GRVMIDGKDIRKYNLKAIRKHIAIVPQEPCLFGTTIYENIAYGHECATEAEIIQAATLAS
AHKFISALPEGYKTYVGERGVQLSGGQKQRIAIARALVRKAEIMLLDEATSALDAESERS
VQEALDQACSGRTSIVVAHRLSTIRNAHVIAVIDDGKVAEQGSHSHLLKNHPDGIYARMI
QLQRFTHTQVIGMTSGSSSRVKEDDA
>AtABCB2
MYISLIFFLSNHFPPLISIPIFIFLSFSSPTNYTHLKLKKMQPSGDPAPEKEKEMTQPKV
SLLKLFSFADFYDCVLMTLGSVGACIHGASVPIFFIFFGKLINIIGLAYLFPKQASHRVA

KYSLDFVYLSVAILFSSWLEVACWMHTGERQAAKMRRAYLRSMLSQDISLFDTEASTGEV
ISAITSDILVVQDALSEKVGNFLHYISRFIAGFAIGFTSVWQISLVTLSIVPLIALAGGI
YAFVAIGLIARVRKSYIKAGEIAEEVIGNVRTVQAFTGEERAVRLYREALENTYKYGRKA
GLTKGLGLGSMHCVLFLSWALLVWFTSVVVHKDIADGGKSFTTMLNVVIAGLSLGQAAPD
ISAFVRAKAAAYPIFKMIERNTVTKTSAKSGRKLGKVDGHIQFKDATFSYPSRPDVVIFD
RLNLAIPAGKIVALVGGSGSGKSTVISLIERFYEPISGAVLLDGNNISELDIKWLRGQIG
LVNQEPALFATTIRENILYGKDDATAEEITRAAKLSEAISFINNLPEGFETQVGERGIQL
SGGQKQRIAISRAIVKNPSILLLDEATSALDAESEKSVQEALDRVMVGRTTVVVAHRLST
VRNADIIAVVHEGKIVEFGNHENLISNPDGAYSSLLRLQETASLQRNPSLNRTLSRPHSI
KYSRELSRTRSSFCSERESVTRPDGADPSKKVKVTVGRLYSMIRPDWMYGVCGTICAFIA
GSQMPLFALGVSQALVSYYSGWDETQKEIKKIAILFCCASVITLIVYTIEHICFGTMGER
LTLRVRENMFRAILKNEIGWFDEVDNTSSMLASRLESDATLLKTIVVDRSTILLQNLGLV
VTSFIIAFILNWRLTLVVLATYPLVISGHISEKLFMQGYGGDLNKAYLKANMLAGESVSN
IRTVAAFCAEEKILELYSRELLEPSKSSFRRGQIAGLFYGVSQFFIFSSYGLALWYGSTL
MDKGLAGFKSVMKTFMVLIVTALAMGETLALAPDLLKGNQMVASVFEILDRKTQIVGETS
EELNNVEGTIELKGVHFSYPSRPDVVIFRDFDLIVRAGKSMALVGQSGSGKSSVISLILR
FYDPTAGKVMIEGKDIKKLDLKALRKHIGLVQQEPALFATTIYENILYGNEGASQSEVVE
SAMLANAHSFITSLPEGYSTKVGERGVQMSGGQRQRIAIARAILKNPAILLLDEATSALD
VESERVVQQALDRLMANRTTVVVAHRLSTIKNADTISVLHGGKIVEQGSHRKLVLNKSGP
YFKLISLQQQQQP
>AtABCB11
MNGDGAREGDSVSHEPSTSKSPKEGEETKKEEKSEEKANTVPFYKLFAFADSSDVLLMIC
GSIGAIGNGMSLPFMTLLFGDLIDSFGKNQNNKDIVDVVSKVCLKFVYLGLGTLGAAFLQ
VACWMITGERQAARIRSTYLKTILRQDIGFFDVETNTGEVVGRMSGDTVLIQDAMGEKVG
KFIQLVSTFVGGFVLAFIKGWLLTLVMLTSIPLLAMAGAAMALIVTRASSRGQAAYAKAA
TVVEQTIGSIRTVASFTGEKQAINSYKKFITSAYKSSIQQGFSTGLGLGVMFFVFFSSYA
LAIWFGGKMILEKGYTGGAVINVIIIVVAGSMSLGQTSPCVTAFAAGQAAAYKMFETIKR
KPLIDAYDVNGKVLEDIRGDIELKDVHFSYPARPDEEIFDGFSLFIPSGATAALVGESGS
GKSTVISLIERFYDPKSGAVLIDGVNLKEFQLKWIRSKIGLVSQEPVLFSSSIMENIAYG
KENATVEEIKAATELANAAKFIDKLPQGLDTMVGEHGTQLSGGQKQRIAIARAILKDPRI
LLLDEATSALDAESERVVQEALDRVMVNRTTVIVAHRLSTVRNADMIAVIHRGKMVEKGS
HSELLKDSEGAYSQLIRLQEINKDVKTSELSSGSSFRNSNLKKSMEGTSSVGNSSRHHSL
NVLGLTTGLDLGSHSQRAGQDETGTASQEPLPKVSLTRIAALNKPEIPVLLLGTVAAAIN
GAIFPLFGILISRVIEAFFKPAHELKRDSRFWAIIFVALGVTSLIVSPTQMYLFAVAGGK
LIRRIRSMCFEKAVHMEVAWFDEPQNSSGTMGARLSADATLIRALVGDALSLAVQNVASA
ASGLIIAFTASWELALIILVMLPLIGINGFVQVKFMKGFSADAKSKYEEASQVANDAVGS
IRTVASFCAEEKVMQMYKKQCEGPIKDGIKQGFISGLGFGFSFFILFCVYATSFYAGARL
VEDGKTTFNNVFQVFFALTMAAIGISQSSTFAPDSSKAKVAAASIFAIIDRKSKIDSSDE
TGTVLENVKGDIELRHLSFTYPARPDIQIFRDLCLTIRAGKTVALVGESGSGKSTVISLL
QRFYDPDSGHITLDGVELKKLQLKWLRQQMGLVGQEPVLFNDTIRANIAYGKGSEEAATE
SEIIAAAELANAHKFISSIQQGYDTVVGERGIQLSGGQKQRVAIARAIVKEPKILLLDEA
TSALDAESERVVQDALDRVMVNRTTIVVAHRLSTIKNADVIAVVKNGVIAEKGTHETLIK
IEGGVYASLVQLHMTASN
>AtABCB20
MMISRGLFGWSPPHMQPLTPVSEVSEPPESPSPYLDPGAESGGGTGTAAALAEADEEMDD
QDELEPPPAAVPFSQLFACADRFDWVLMIVGSVAAAAHGTALIVYLHYFAKIVDVLAFSN
DSSQQRSEHQFDRLVQLSLTIVYIAGGVFISGWIEVSCWILTGERQTAVIRSKYVQVLLN
QDMSFFDTYGNNGDIVSQVLSDVLLIQSALSEKVGNYIHNMATFISGLVIGFVNCWEIAL
ITLATGPFIVAAGGISNIFLHRLAENIQDAYAEAAGIAEQAISYIRTLYAFTNETLAKYS
YATSLQATLRYGILISLVQGLGLGFTYGLAICSCALQLWIGRFFVHNGRANGGEIIAALF
AVILSGLGLNQAATNFYSFDQGRIAAYRLFEMITRSSSVANQEGAVLASVQGNIEFRNVY
FSYLSRPEIPILSGFYLTVPAKKAVALVGRNGSGKSSIIPLMERFYDPTLGEVLLDGENI
KNLKLEWLRSQIGLVTQEPALLSLSIRENIAYGRDATLDQIEEAAKNAHAHTFISSLEKG
YETQVGRAGLAMTEEQKIKLSIARAVLLNPTILLLDEVTGGLDFEAERIVQEALDLLMLG

RSTIIIARRLSLIKNADYIAVMEEGQLVEMGTHDELINLGGLYAELLKCEEATKLPRRMP
VRNYKESAVFEVERDSSAGCGVQEPSSPKMIKSPSLQRGSGVFRPQELCFDTEESPKAHS
PASEKTGEDGMSLDCADKEPTIKRQDSFEMRLPHLPKVDVQCPQQKSNGSEPESPVSPLL
TSDPKNERSHSQTFSRPLSSPDDTKANGKASKDAQHKESPSFWRLAQLSFPEWLYAVLGS
LGAAIFGSFNPLLAYVIALVVTEYYKSKGGHLREEVDKWCLIIACMGIVTVVANFLQHFY
FGIMGEKMTERVRRMMFSAMLRNEVGWFDDEENSPDTLSMRLANDATFVRAAFSNRLSIF
IQDSFAVIVALLIGLLLGWRLALVALATLPILTLSAIAQKLWLAGFSKGIQEMHRKASLV
LEDAVRNIYTVVAFCAGNKVMELYRMQLQRILRQSYLHGMAIGFAFGFSQFLLFACNALL
LWCTALSVNRGYMKLSTAITEYMVFSFATFALVEPFGLAPYILKRRKSLISVFEIVDRVP
TIEPDDNSALKPPNVYGSIELKNVDFCYPTRPEILVLSNFSLKISGGQTVAVVGVSGSGK
STIISLVERYYDPVAGQVLLDGRDLKLYNLRWLRSHMGLVQQEPIIFSTTIRENIIYARH
NASEAEMKEAARIANAHHFISSLPHGYDTHIGMRGVELTPGQKQRIAIARVVLKNAPIIL
IDEASSSIESESSRVVQEALDTLIMGNKTTILIAHRAAMMRHVDNIVVLNGGRIVEEGTH
DSLAAKNGLYVRLMQPHFGKGLRQHRLI
>TaABCC13
mgaddhdadyahmvanfaslpalgflclvgvmgssgvdlefsdddtgvheplllggqrrg
aeeepgclrvtpygdagilslatlswlspllsvgakrpleladipllahkdrakfcykam
sshyerqrlecpdkepslawailksfwreaaingafaavntvvsyvgpylisyfvdylsg
kiafphegyilasvffvsklietltarqwylgvdvmgihvksgltamvyrkglrlsnask
qshtsgeivnymavdvqrvgdyawyfhdiwmlplqiilalailyknvgiatvstliatal
siaasvpvaklqehyqdklmaakdermrktaeclksmrilklqawedryrimleemrnve
crwlkwalysqaavtfvfwsspifvsvitfgtcillggeltaggvlsalatfrilqeplr
nfpdlismiaqtrvsldrlshflrqeelpddatisvpqgstdkaidikdgsfswnpscst
ptlshiqlsvvrgmrvavcgvigsgksslls silgeiprlsgqvrvsgtaayvsqtawiq
sgnieenvlfgtpmdrprykrvleacslkkdlqllqygdqtiigdrginlsggqkqrvql
aralyqdadiyllddpfsavdahtgsdlfkdyilgalasktviyvthqveflpaadlilv
lkdghitqagkyddllqagtdfnalvsahneaietmdfgedsdgdiapsvpnkrltpsvs
nidnlknkvsengkssntrgikdkkkseerkkkrtvqeeerergrvslnvyltymgeayk
gsliplivlaqtlfqvlqiasnwwmawanpqtegdapktssvvllvvymclafgsslfvf
vrsllvatfglaaaqklfikmlrcvfrapmsffdttpsgrilnrvsvdqsvvdldiafrl
ggfasttiqllgivavmskvtwqvlflivpmamacmwmqryyiassreltrilsvqkspv
ihlfsesiagaatirgfdqekrfmkrnlylldcfarplfsslaaiewlclrmellstfvf
afcmailvsfppgtiepsmaglavtyglnlnarmsrwilsfcklenriisveriyqycki
pseapliiencrppaswpengniqlidlkvrykddlpfvlhgvscifpggkkigivgrtg
sgkstliqalfrlieptggkiiiddidvsaiglhdlrsrlsiipqdptlfegtirmnldp
leersdqeiwealekcqlgevirskeekldspvlengdnwsvgqrqlialgrallkqari
lvldeatasvdtatdnliqkiirsefrdctvctiahriptvidsdlvmvlsdgkiaefdt
pqrlvedkssmfmqlvseystrasci
>AtABCG26
MEIRRSTEEVEENHVMQITGSNGIVHNMEFMPQAYLRNQYSSEIDIDEEFVSTYPLEDAP
LPIFLKFEDVEYKVRNSHASSANLVKTMVSKVVTHTNPDPDGYKHILKGITGSTGPGEIL
ALMGPSGSGKTTLLKIMGGRLTDNVKGKLTYNDIPYSPSVKRRIGFVTQDDVLLPQLTVE
ETLAFAAFLRLPSSMSKEQKYAKIEMIIKELGLERCRRTRVGGGFVKGISGGERKRASIA
YEILVDPSLLLLDEPTSGLDSTSATKLLHILQGVAKAGRTVITTIHQPSSRMFHMFDKLL
LISEGHPAFYGKARESMEYFSSLRILPEIAMNPAEFLLDLATGQVSDISLPDELLAAKTA
QPDSEEVLLKYLKQRYKTDLEPKEKEENHRNRKAPEHLQIAIQVKKDWTLSWWDQFLILS
RRTFRERRRDYFDKLRLVQSLGVAVVLGLLWWKSKTDTEAHLRDQVGLMFYICIFWTSSS
LFGAVYVFPFEKIYLVKERKAEMYRLSVYYVCSTLCDMVAHVLYPTFFMIIVYFMAEFNR
NIPCFLFTVLTILLIAITSQGAGEFLGASVLSIKRAGMIASLVLMLFLLTGGYYVQHIPK
FMQWLKYLSFMHYGFRLLLKVQYSADQLFECGSKGGCRTLQSSSSFDTINLNGGLQELWV
LLAMAFGYRLCAYFCLRKKISICHL
>AtABCG34
MLGRDEDLVRTMSGRGSLGSTSHRSLAGAASKSFRDVFAPPTDDVFGRSDRREEDDVELR
WAALERLPTYDRLRKGMLPQTMVNGKIGLEDVDVTNLAPKEKKHLMEMILKFVEEDNEKF

LRRLRERTDRVGIEVPKIEVRYENLSVEGDVRSASRALPTLFNVTLNTIESILGLFHLLP
SKKRKIEILKDISGIIKPSRMTLLLGPPSSGKTTLLQALAGKLDDTLQMSGRITYCGHEF
REFVPQKTCAYISQHDLHFGEMTVRESLDFSGRCLGVGTRYQLLTELSRREREAGIKPDP
EIDAFMKSIAISGQETSLVTDYVLKLLGLDICADTLVGDVMRRGISGGQRKRLTTGEMLV
GPATALFMDEISTGLDSSTTFQICKFMRQLVHIADVTMVISLLQPAPETFELFDDIILLS
EGQIVYQGSRDNVLEFFEYMGFKCPERKGIADFLQEVTSKKDQEQYWNRREHPYSYVSVH
DFSSGFNSFHAGQQLASEFRVPYDKAKTHPAALVTQKYGISNKDLFKACFDREWLLMKRN
SFVYVFKTVQITIMSLIAMTVYFRTEMHVGTVQDGQKFYGALFFSLINLMFNGMAELAFT
VMRLPVFFKQRDFLFYPPWAFALPGFLLKIPLSLIESVIWIALTYYTIGFAPSAARFFRQ
LLAYFCVNQMALSLFRFLGALGRTEVIANSGGTLALLVVFVLGGFIISKDDIPSWLTWCY
YTSPMMYGQTALVINEFLDERWGSPNNDTRINAKTVGEVLLKSRGFFTEPYWFWICIGAL
LGFTVLFNFCYIIALMYLNPLGNSKATTVVEEGKDKHKGSHSGTGGSVVELTSTSSHGPK
KGMVLPFQPLSLAFNNVNYYVDMPAEMKAQGVEGDRLQLLRDVGGAFRPGVLTALVGVSG
AGKTTLMDVLAGRKTGGYVEGSINISGYPKNQATFARVSGYCEQNDIHSPHVTVYESLIY
SAWLRLSADIDTKTREMFVEEVMELVELKPLRNSIVGLPGVDGLSTEQRKRLTIAVELVA
NPSIIFMDEPTSGLDARAAAIVMRTVRNTVDTGRTVVCTIHQPSIDIFESFDELLLMKRG
GQVIYAGTLGHHSQKLVEYFEAIEGVPKIKDGYNPATWMLDVTTPSMESQMSVDFAQIFV
NSSVNRRNQELIKELSTPPPGSNDLYFRTKYAQPFSTQTKACFWKMYWSNWRYPQYNAIR
FLMTVVIGVLFGLLFWQTGTKIEKEQDLNNFFGAMYAAVLFLGATNAATVQPAVAIERTV
FYREKAAGMYSAIPYAISQVAVEIMYNTIQTGVYTLILYSMIGYDWTVVKFFWFYYYMLT
CFVYFTLYGMMLVALTPNYQIAGICLSFFLSFWNLFSGFLIPRPQIPIWWRWYYWASPVA
WTLYGIITSQVGDRDSIVHITGVGDMSLKTLLKNGFGFDYDFLPVVAVVHIAWILIFLFA
FAYGIKFLNFQRR
>AtABCG14
MPQNCIAPRPEEDGGVMVQGLPDMSDTQSKSVLAFPTITSQPGLQMSMYPITLKFEEVVY
KVKIEQTSQCMGSWKSKEKTILNGITGMVCPGEFLAMLGPSGSGKTTLLSALGGRLSKTF
SGKVMYNGQPFSGCIKRRTGFVAQDDVLYPHLTVWETLFFTALLRLPSSLTRDEKAEHVD
RVIAELGLNRCTNSMIGGPLFRGISGGEKKRVSIGQEMLINPSLLLLDEPTSGLDSTTAH
RIVTTIKRLASGGRTVVTTIHQPSSRIYHMFDKVVLLSEGSPIYYGAASSAVEYFSSLGF
STSLTVNPADLLLDLANGIPPDTQKETSEQEQKTVKETLVSAYEKNISTKLKAELCNAES
HSYEYTKAAAKNLKSEQWCTTWWYQFTVLLQRGVRERRFESFNKLRIFQVISVAFLGGLL
WWHTPKSHIQDRTALLFFFSVFWGFYPLYNAVFTFPQEKRMLIKERSSGMYRLSSYFMAR
NVGDLPLELALPTAFVFIIYWMGGLKPDPTTFILSLLVVLYSVLVAQGLGLAFGALLMNI
KQATTLASVTTLVFLIAGGYYVQQIPPFIVWLKYLSYSYYCYKLLLGIQYTDDDYYECSK
GVWCRVGDFPAIKSMGLNNLWIDVFVMGVMLVGYRLMAYMALHRVKLR
>NtPDR1
mepanlsnlrgsslrgstrgslransnsiwrnngveifsrssrdeddeealkwaaleklp
tfdrlrkgllfgsqgaaaevdindlgfqerknllerlvkvadednekfllklknridrvg
idlptievryehlnidadayvgsrslptfmnfmtnfvetllnslhilssrkrqltilkdi
sgiikpcrmtlllgppssgkttlllalagkldpalkvtgkvsynghelhefvpqrtaayi
sqhdlhigemtvretlefsarcqgvgsrfemlaelsrrekaanikpdadidiymkaaate
gqeanvvtdyvlkilgldicadtmvgddmirgisggqkkrvttgemlvgpskalfmdeis
tgldssttysivnslrqsvqilkgtavisllqpapetynlfddiillsdgyivyqgprdd
vleffesmgfkcpqrkgvadflqevtskkdqqqywskrnepyrfitskefaeayqsfhvg
rklgdelatpfdktkchpaaltnekygigkkellkvcterelllmkrnsfvymfkfsqlt
imalitmtlffrtemprdttddggiyagalffvvimimfngmselamtifklpvfykqrd
llffpswayaipswilkipvtlvevglwviltyyvigfdpnitrflkqflllivvnqmas
gmfrfigavgrtmgvastfgsfalllqfalggfvlsrddvkswwiwgywispmmysvnsi
lvnefdgkkwnhivpggnetlgstvvksrgffpeaywywigvgalvgftvvfnfcyslal
aylnpfdkpqavlpedgenaengevssqipstdggdsisesqnnkkgmvlpfephsitfd
dvvysvdmpqemkeqgagedrlvllkgvsgafrpgvltalmgvsgagkttlmdvlagrkt
ggyidgeikisgypkkqetfarisgyceqndihspyvtvyeslvysawlrlpqdvdektr
kmfvdevmelvelgplrsalvglpgvnglsteqrkrltiavelvanpsiifmdeptsgld
araaaivmrtvrntvdtgrtvvctihqpsidifeafdelflmkrggqeiyvgplgrhsch

likyfesnpgvakikegynpatwmlevtasaqemmlgidftevyknsdlyrrnkalisel
gvprpgskdlhfetqysqsfwtqcvaclwkqhwsywrnpaytavrfifttfialifgtmf
wdlgtkvsksqdllnamgsmyaavlflgvqnassvqpvvaiertvfyreraagmysaipy
afgqvsieipyifvqsvfygiivyamigfewdvgkffwylfimfftllyftfygmmgvav
tpnqnvasivaaffygvwnlfsgfiiprprmpvwwrwyywanpvawtlyglvasqfgdiq
tklsdnetveqflrryfgfkhdflgvvaavltayvfmfaftfafaikafnfqrr
>PhPDR2
mepvnlgnlraaslrgsargslsgslransnsiwrndnvftrssrdendeealkwaalek
lptfdrlrkgllfgsegtapsqidihdigfqerqglldrlvkdpdednekfllklrdrid
rvgldlptievryehlhvvadayiggralptftnfvtnflesllltslhilpskkrkltil
ndvsgiikpcrltlllgppgsgkttfllalagkldpelkvtgkvtynghemtefvpqrta
ayisqhdlhigemtvretlefsarcqgigtryemlaelsrrekaanikpdpdidiymkas
ategqeanvvtdyvlkilgldicadtlvgddmvrgisggqkkrvttgemlvgpskalfmd
eistgldssttysivnslrqtvqilketavisllqpapetynlfddiilltdglvvyqgp
redvlaffesmgfkcpdrkgvadflqevtskkdqqqywarrdepyrfitskefaeayqsf
hvgrkqldelgahlteqshpaalsnqkygigkkqllkvctereyllmkrnsflfifkffq
llimailtmtmflrtemhhnteedggtyvgalffvivmimfngmtelgmvlfklpvfykq
rdlffypswayaipswilkipitfvevalwvfltyyvigfdpnperlfkqfflliivnqm
asglfrfigaagrtlgiaatfgafalllqfalggfvlsrdmmkkwwiwgywtspmmysvn
ailvnefhgkrwrriapngteplgdavvrgrgffpdaswywigvgaligftvlfnilysl
alaylnpigkpqammpedsedakttstekegynsegqnkkrgmvlpfephsitfddviys
vdmpqemkdqgasedrlvllngvsgafrpgvltalmgvsgagkttlmdvlagrktggyie
grihisgypkkqetfarisgyceqndihspyvtvyeslvysawmrlphdvdertrkmfve
evmdlvelrpirsalvglpgvdglsteqrkrltiavelvanpsiifmdeptsgldaraaa
ivmravrntvdtgrtvvctihqpsidifeafdelflmkrggqeiyvgplgrnschlikyf
esmpgvskikrwlhpatwmlevttpgqetmfgvdftdlykksdlygrnkalitelsvprp
gtkdlhfdtqysqpfwtqcmaclwkqhwsywrnpaytavrflftvmislvfgtmfwdlgs
kvsraqdlsnamgclyaavlfigtqnassvqpvvavertvfyreraagmysalpyafaqa
fieipyifvqatfcgtiiyamigfewtvekyfwylffmfftlmyytyygmmtvaitpnvn
vaqvvsaffyglwnlfsgfivprprmaiwwrwyywicptawtlygliasqfgdyqnkltd
detveqylrrffgfkheflpvvgvvtagftvlfaftfafgikafnfqtr
>LeMRP
HGVYICELRWYVRFGLIYAVMGEAVMLNLVLSMRNFFDRFTLYLYISEVVAQVLVGLMLL
VYVPALDPYPGYTPLQTESPDNSTYEVLAAGEQICPERHINIFSKITFAWINPIMQLGYK
RPLTEKDVWKLDTWDTSDTLYNRFQKCWIDETQRPRPWLLRVLNRSIGGRFWWGGFWKVG
NDTSQFIGPLILNQLLQSMQSGDPAWIGYIYAFSIFLGVVFGVLFEAQYFQNVMRVGFRL
RSTLVAAVFRKSLRLSHESRKTFASGKITNLMTTDAETLQLVCQSLHTLWSAPFRIIVAL
VLLYKQLGVASLLGALLLLLMFPVQTLVVSKMQKLSKEGLHRTDKRIGLMNEILAAMDTV
KCYAWENSFRSKVQTVRDDELTWFRKAQLLGALNNFILNSTPVVVIVVSFGMFSFLGGEL
TPARAFTSISLFAVLRFPLILLPNVITQVVNSNVSLKRIEELLLAEERVLLPNPPLEPEL
PAISIKNGYFSWESNSEKLTLSNINLDVPVGSLVAVVGGTGEGKTSLVSAMLGEIPSAVD
ATIVLRGTVAYVPQISWIFNATMRENILFGSPYVPARYDRAIEVTSLQHDLDLLPGGDLT
EIGERGVNISGGQKQRVSMARAVYSDSDVYIFDDPLSALDAHVGQQVFEKCIKGELRGKT
RVLVTNQLHLLSQVDKIILVHDGMVKEEGTFDYLLNNGELFQKLMENAGKMEEYVEEKEN
AETMDSKTTPVANGAIDGFGKDTGGTNKRKEGKSVLIKQEERETGVVSWNVLMRYKNALG
GTLVVMTLFLCYVLTEVLRVGSSTWLSCWTDKSSSSRYSPGYYNLIYALLSSGQVLVTLT
NSFWLIISSLYAAKILHDAMLKSILRVPMIFFHTNPFGRIINRFAKDLGDIDRNVAPFVD
LCLGQVSQLVSTFVLIGIVSTMSLWAILPLLVLFYGAYLYYQSTAREVKRLDSITRSPVY
AKFGEALNGLSSIRAYKAYDRMANINGKSMDNNVRFTLVTMSGNRWLAIRLETVGGIMIW
VTATFAVMQNGRAENQEAFASTTGLLLSYALNITSLLTAALRLASLAENSLNAVERVGAY
IDLPSERPDIIEGNRPHHGWPSEGSIQFEDVVLRYRPELPPVLHGITFTIPPTDKVGVVG
RTGAGKSSMLNALFRIVELERGRILIDNCDISKFGLTDLRKVLGIIPQSPVLFSGTVRFN
LDPFSEHNDAGIWEALERAHLKEVIRRSSLGLDCEVSEAGENFSVGQRQLLSLARALLRR
SKVLVLDEATAAVDVRTDALIQKTIRDEFKSCTMLIIAHRLNTIIDCDEILVLGSGKVLE

YDAPETLLQNEESAFSKMVLSTGPANAQYLRSLVLGVGYDGDTRTVDEKQLLDLPRRWLA
SSRWAAAAQLALAVSLTSSHNDLIQLELRDNDDDVILKKTKDAVLTLQGVLEGKHDKMIA
EKLEQYHVSRDGWWSSLYKMIEGLAIMSRLSQSRFLH
>VvABCG44
MATAEIYRAAGSLRRNGSMWRSSGADVFSRSSRDEDDEEALKWAALEKLPTYNRLRKGLL
MGSQGAASEVDVDNLGFQEKQSLMERLVKIAEEDNEKFLLRLRNRIERVGITIPEIEVRF
EHLTIDAEAFIGSRALPSFHNFMFNKIEDALTGLRILRSRRRKFTILHDVSGIIKPQRMT
LLLGPPSSGKTTLLLALSGKLDPTLKVTGRVTYNGHGMDEFVPQRTAAYISQHDTHIGEM
TVRETLAFSARCQGVGDRYDMLAELSRREKAANIKPDPDLDVFMKAAATEGQKENVVTDY
TLKILGLDICADTMVGDEMIRGISGGQRKRVTTGEMLVGPSKALFMDEISTGLDSSTTFQ
IVNCLKQTIHILNGTAVISLLQPAPETYNLFDDIILLSDGRIIYQGPREDVLEFFESTGF
RCPERKGVADFLQEVTSKKDQQQYWARKEEPYRFVTVKEFAEAFQSFHTGRKVGDELASP
YDKTKSHPAALTTKKYGVNKKELLDANMSREYLLMKRNSFVYVFKLTQLAIMAVITMTLF
LRTEMHKNSVDDGNIYTGALFFTVVMIMFNGMAELAMAIAKLPVFYKQRDLLFYPAWAYA
LPTWILKIPITFIEVGVWVFMTYYVIGFDPNVERLFRQYLLLLLVNQMASGLFRLIASAG
RNMIVSNTFGAFVLLMLLALGGFILSHDDVKKWWIWGYWCSPLMYAQNAIVVNEFLGHSW
KKNVTGSTESLGVTVLNNRGFFTEAYWYWIGAGALFGFILLFNFGYTLCLNFLNPFDKPQ
AVIVEESDNAETGGQIELSQRNSSIDQAASTERGEEIGRSISSTSSAVREEAVAGANHNK
KKGMVLPFQPYSITFDDIRYSVDMPEEMKSQGVVEDKLELLKGVSGAFRPGVLTALMGVS
GAGKTTLMDVLAGRKTGGYIEGNITISGYPKKQETFARISGYCEQNDIHSPHVTVYESLL
YSAWLRLPSDVKSETRQMFIEEVMELVELTPLRDALVGLPGVSGLSTEQRKRLTIAVELV
ANPSIIFMDEPTSGLDARAAAIVMRTVRNTVDTGRTVVCTIHQPSIDIFEAFDELLLLKR
GGQEIYVGPLGRYSCHLINYFEGIEGVSKIKDGYNPATWMLEATTAAQEATLGVDFTEIY
KNSDLYRRNKDLIKELSQPPPGTKDLYFRTQFSQPFFTQFLACLWKQRWSYWRNPPYTAV
RFLFTTFIALMFGTMFWDLGTKWSTQQDLFNAMGSMYAAVLFLGIQNSQSVQPVVVVERT
VFYRERAAGMYSPLSYAFAQALVEIPYIFSQAVVYGLIVYAMIGFQWTAAKFFWYLFFMF
FTLMYFTFYGMMAVAATPNQNIASIVAAAFYGLWNLFSGFIVPRNRIPVWWRWYYWICPV
SWTLYGLVTSQFGDITEELNTGVTVKDYLNDYFGFKHDFLGVVAAVVVGFVVLFLFIFAY
AIKALNFQRR
>AaPDR3
MGSEAKTTLDQSPETPSPAVTDLITELKTCFSSIDFQNVANLLMNREQDLLHKTHVLENE
KDEILKENKKLKGLLKKKDAKIESLEKETKKLNEFSKKKDEKIESLEKENDGLKEELNKK
QTEIGNYEKVHLDYGVRVLSLEKINKELLALEVKKPKNVVVAEGSKPPPPPGFEEILDPN
KPKKEKDSKVPEIIEIDDDDDDEDDHGDRDLMKVSSKRKRDETKRGDSDDDKYISILRRK
KVSREGTQDHKKDNYDEKIPTRTSHLKSPFCDSSSSDSDNEKDKISAKMDLQKDRWETRE
DMVKEFGKDDQLCLNAICALHKQDSSQKRVVLVRLFDASRISYLSRKKTVSELSLFDLKQ
CRQFAMDYSDKLFDIYKEKSDSF
>PhPDR1
meggeelfrvssarlsssnvwrnsamdvfsrssreaddeealkwaaleklptylrirrgi
lteeegqsrevditkldlverrnllerlikitdednekfllklkeridrvgldlptievr
fehlsvdaearvgsralptvfnftvniledflnylhilpnrkqplpilhdvsgiikpgrm
tlllgppssgkttlllalagkldkdlkvsgrvtynghdmnefvaqrssayisqydlhige
mtvretlafsarcqgvgakyeilaelsrrekeanikpdpdvdifmkaawnegqeanvvtd
ytlkilgleicadtivgdemvrgisggqrkrlttgemmvgparalfmdeistgldsstty
qivnsirqsihilqgtavisllqpapetydlfddiillsdgqivyqgprenvleffeymg
ficperkgvadflqevtsrkdqeqywarreesykfitvrefseafqafhigrklgdelav
pfdkskshpaalttkrygvskkellkactareyllmkrnsfvyifkmiqltlmasitmtl
flptemhrnttidgavflgalfyalimimfngfselalsimklpsfykhrdllffppway
alptwilkipitlvevaiwvcmtyyvigfeadvgrffkqllllicvnqmasglfrlmgal
grniivantfgsfvlltvlvmggfvlsrddvkkwwiwgywispmmyaqnaiavneflgks
wahvppnststetlgvsflksrgifpdarwywigagaligyvflfnflfavalaylnpfg
kpqavlseetvaernaskrgevielsslgksssekgndvrrsassrsmssrvgsitaadl
skrrgmilpfeplsitfddiryavdmpqemkaqgftedrlellrgvsgafrpgvltalmg
vsgagkttlmdvlagrktggyidgtisisgypkqqetfariagyceqtdihsphvtvyes

lqfsawlrlprevdtatrkmfieevmelieliplrdalvglpgvnglsteqrkrltvave
lvanpsiifmdeptsgldaraaaivmrtvrntvdtgrtvvctihqpsidifdafdellll
krggeeiyvgplgrqsshlikyfegidgvpkikdgynpatwmleitsvaqegalgndfte
lyknselyrrnkalikelsvpascskdlyfptkysqsfftqcmacfwkqhwsywrnppyt
avrimftffialmfgtifwdlgsrrerqqdllnaigsmyiavlflgvqnattvqpviaie
rtvfyreraagmysampyafgqvmielpylflqtiiygvivyamigfewtvakffwylff
myftllyftlygmmtvavtpnqsiaaiissafyavwnlfcgfivpktrmpvwwrwyyyic
piswtlygliasqfgdiqdrldtnetveqfienffdfkhdfvgyvalilvgisvlflfif
afsiktfnfqkr
>NtPDR6
MEGGEDSFRVSSARLSSNVWRNSAMDVFSRREADDEEALKWAALEKLPTYLRRRGLTEEE
GSRVDTKLDLVERRNLLERLVKADEDNEKFLLKLKKRARVGLDLPTEVRFEHVSVDAEAR
GSRALPTFNFTVNLEDFLNYLHLPSRKKPLPLHEVSGKPGRMTLLLGPPSSGKTTLLLAL
AGKLDKDLKVSGRVTYNGHGMDEFVPRSSAYSNDLHGEMTVRETLAFSARCGVGAKYELA
ELSRREKGANKPDPDVDFMKAWNEGEANVVTDYTLKLGLECADTLVGDMRGSGRKRLTTG
EMMVGPARALFMDESTGLDSSTTYVNSRSHLGTAVSLLPAPETYDLFDDLLSDGVYGPRE
NVLEFFEYMGFMCPERKGVADFLEVTSRKDEYWARRDEPYKYTVREFSESFSFHGRKLGD
ELAVPFDKSKSHPAALTTKKYGSKKELLKACTAREYLLMKRNSFVYFKMLTLMASTMTLP
hcLRTEMHRNTTTDGAVFLGALFYAVMMFNGFSLALSMKLPSFYKRDLLFFPAWAYALPT
WLKPVTLVEVAWVCMTYYVGFEADVGRFFKLFLLCVNMASGLFRFGALGRNVVANTFGSC
ALLTVLVMGGFLSRDDVKKWWWGYWSPMMYANAAVNEFLGKSWAHVPPNSTGTETLGVSF
LKSRGFPEARWYWGAGALGYVLLFNFMFTVALAYLNPFGKSAVLSEETVAERNAKRGEVE
LSPEKRSSERGNDVRRSASSRSMSSRGSTEADLNKRRGMLPFEPLSTFDDRYAVDMPEMK
AGVAEDRLELLKGVSGAFRPGVLTALMGVSGAGKTTLMDVLAGRKTGGYDGTSSGYPKET
FARAGYCETDHSPHVTVYESLFSALLRLPREVDTETRKMFVEEVMELVELTPLREALVGL
PGVNGLSTERKRLTVAVELVANPSFMDEPTSGLDARAAAVMRTVRNTVDTGRTVVCTHPS
DFDAFDELLLLLKRGGEEYGPLRHSSHLKYFEGDGVPKKDGYNPATWMLETSVAEAARVDF
TELYKNSELYRRNKALKELSVPPPCSKDLYFPTKYSSFFTCKACFWKHWSYWRNPPYTAV
RLMFTFFALMFGTFWDLGSRRKRDLLNAGSMYAAVLFLGVNATSVPVAERTERAAGMYSA
LPYAFGVMELPYLFTYGVVYVMGFEWTVAKFFWYLFFMYFTLLYFTLYGMMTVAVTPNHS
ASSFYAWNLFCGFVVPKTDVFYGSKSDGSNFVLSTVCARAVGVRDTWCWLGAGAWHWVCA
RGTVCAGSAVGNMMVCARSVGTRGTDGRPLGAETDYRWDDRGVCVLVTWRCDHGADRGSR
RWEACRDATGRVGVMGR
>NaPDR3
maqlvgpdeiesfrmdlaeigrslrssfrgqslsfrsnsalsasqkddavdeenilawaa
ierlptfdrlrssvfeeingndanvkrkrvtdvtklgalerhvfiekmikhiehdnlqll
hkirkridkvgvelptvevryknltieaecelvhgkplptlwnslksittnlarlpglqs
elakikilndvsgvikpgrmtlllgppgcgktsllkalsgnldkslkvsgeisyngykle
efvpqktsayvsqndlhipemtvretldyssrfqgvgsraeimtdlsrrekeagvvpdpd
idtymkaisiegqkknlqtdyilkilgldicadtlvgdamrrgisggqkkrlttgelivg
ptkalfmdeisngldssttyqivaclqqlahitdatilvsllqpapetfdlfddiilmae
gkilyhgprnsaleffescgfkcperkgvadflqevtskkdqaqywhgtketykfvsvdm
lsrkfkgspyrkklneelsvpydnsrshrnsitfrdyslpkwelfracmsrefllmkrns
fiyifktvqlaiiasitmtvflrtrmdtdlvhanyylgalfyaliillvdgfpelsmtit
rlavfykqselcfypawayaipatilkiplsllesviwvsmtyyvigftpeagrffrqll
llfavhmtsismfrflasvcrtivastvagslsilfvlcfsgfiiprpsmpiwlkwgfwi
splsygeiglavneflaprwqktlstntsigngvlesrgfnfdgyfywisvcalfgftil
fnigftlaltflkapgsraiistdkysqiegssdscdrtdaadnskatipsttmhsnera
grmvlpfeplslvfqdvqyyvdtpatmkelgftqnrlqllsditgalrpgiltalmgvsg
agkttlldvlagrktsgyvegeikvggypkvqetfarvsgyceqtdihspqitveesvif
sawlrlhpqidsktkyefvkevletieldgikdmlvgmpgvsglsteqrkrltiavelva
npsiifmdepttgldarsaaivmravknvadtgrtivctihqpsidifeafdelillktg
grmiywghlgrnsckmieyfegisgvpkiknnynpatwmleitstsseaeitidfaevyk
nsalhknneelvkklsfppagskdlhfptrfsqngwgqfktcfwkqywsywrspsynlmr

slhmlfaslvfgllfwdkgkkldnqqsvfsvfgamftavifcginnsssvlpyvttersv
lyrerfagmyaswafalaqvaieipyllaqalaftvitypmigyywsaykvfwyfysmfc
tllyftylgmmlvsmtpnfpvaailqssfytmfnlfagflmpkaqipkwwiwfyyltpts
wtlngmltsqygdvdteinvfgekksvaaflrdyfgfhhnqlpivavvliayplvfaslf
affigklnfqrr
>AtABCG37
MAHMVGADDIESLRVELAEIGRSIRSSFRRHTSSFRSSSSIYEVENDGDVNDHDAEYALQ
WAEIERLPTVKRMRSTLLDDGDESMTEKGRRVVDVTKLGAVERHLMIEKLIKHIENDNLK
LLKKIRRRIDRVGMELPTIEVRYESLKVVAECEVVEGKALPTLWNTAKRVLSELVKLTGA
KTHEAKINIINDVNGIIKPGRLTLLLGPPSCGKTTLLKALSGNLENNLKCSGEISYNGHR
LDEFVPQKTSAYISQYDLHIAEMTVRETVDFSARCQGVGSRTDIMMEVSKREKEKGIIPD
TEVDAYMKAISVEGLQRSLQTDYILKILGLDICAEILIGDVMRRGISGGQKKRLTTAEMI
VGPTKALFMDEITNGLDSSTAFQIVKSLQQFAHISSATVLVSLLQPAPESYDLFDDIMLM
AKGRIVYHGPRGEVLNFFEDCGFRCPERKGVADFLQEVISKKDQAQYWWHEDLPYSFVSV
EMLSKKFKDLSIGKKIEDTLSKPYDRSKSHKDALSFSVYSLPNWELFIACISREYLLMKR
NYFVYIFKTAQLVMAAFITMTVFIRTRMGIDIIHGNSYMSALFFALIILLVDGFPELSMT
AQRLAVFYKQKQLCFYPAWAYAIPATVLKVPLSFFESLVWTCLSYYVIGYTPEASRFFKQ
FILLFAVHFTSISMFRCLAAIFQTVVASITAGSFGILFTFVFAGFVIPPPSMPAWLKWGF
WANPLSYGEIGLSVNEFLAPRWNQMQPNNFTLGRTILQTRGMDYNGYMYWVSLCALLGFT
VLFNIIFTLALTFLKSPTSSRAMISQDKLSELQGTEKSTEDSSVRKKTTDSPVKTEEEDK
MVLPFKPLTVTFQDLNYFVDMPVEMRDQGYDQKKLQLLSDITGAFRPGILTALMGVSGAG
KTTLLDVLAGRKTSGYIEGDIRISGFPKVQETFARVSGYCEQTDIHSPNITVEESVIYSA
WLRLAPEIDATTKTKFVKQVLETIELDEIKDSLVGVTGVSGLSTEQRKRLTIAVELVANP
SIIFMDEPTTGLDARAAAIVMRAVKNVADTGRTIVCTIHQPSIDIFEAFDELVLLKRGGR
MIYTGPLGQHSRHIIEYFESVPEIPKIKDNHNPATWMLDVSSQSVEIELGVDFAKIYHDS
ALYKRNSELVKQLSQPDSGSSDIQFKRTFAQSWWGQFKSILWKMNLSYWRSPSYNLMRMM
HTLVSSLIFGALFWKQGQNLDTQQSMFTVFGAIYGLVLFLGINNCASALQYFETERNVMY
RERFAGMYSATAYALGQVVTEIPYIFIQAAEFVIVTYPMIGFYPSAYKVFWSLYSMFCSL
LTFNYLAMFLVSITPNFMVAAILQSLFYVGFNLFSGFLIPQTQVPGWWIWLYYLTPTSWT
LNGFISSQYGDIHEEINVFGQSTTVARFLKDYFGFHHDLLAVTAVVQIAFPIALASMFAF
FVGKLNFQRR
>MtPDR23
MEGGELRVASGRVGSSSIWRSGAVDVFSGSSRRDDDEQELQWAAIEKLPTYLRMTRGILN
ESQSEQPIEIDINKLGPLQRKNLVERLVKIAEEDNEKFLLKLRQRIDRVGLDFPTIEVRF
EHLNVEAEAHVGSRALPTILNFSINLLEGFLNNLHLIPSRKKPLTVLHDVSGIIKPKRMT
LLLGPPSSGKTTLLLALAGRLSRDLKFSGRVAYNDHGMEEFVPQRTSAYISQTDLHIGEL
TVRETLAFSARCQGIGTRYDMLAELSRREKAENIKPDPDLDIYMKAEALEGQETNIVTDY
IIKILGLDVCADTMVGDDMIRGISGGQKKRVTTGEMLVGPARALFMDEISTGLDSSTTFQ
MINSLRQSIHILNGTALISLLQPTPETYDLFDDIILLSDGQIVYQGPRENVLEFFEHVGF
KCPERKGVADFLQEVTSRKDQEQYWSNKDKPYTFITVREFAEEFQLFHVGQKLGDELGTP
FDASKGHPAVLTKNKYGVSRKELLKACVSRELLLMKRNSFVYIFKMWQLIFTGIVTMTMF
LRTEMHRNTETDGGIYMGALFFILIVIMFNGYSELSMFIMKLPVFYKQRDLLLFPAWAYS
LPTWILKIPITFVEVGIWVVLTYYVIGFDPCFERFIKQYFLLVCINQMASALFRFIGAVG
RNVIVANTVGSFALLAVLVMGGFILSRVDVKKWWLWGYWVSPMMYGQNAIAVNEFLGKSW
SHIPPDSTEPLGVQILKSRGIFPEAYWYWIGVGASIGYMLLFNFLFPLALHYLDSKYPIY
YMWLSAFGKPQALISEEALAERNAATAGSKQIIELSPKLECSSGNASRRSFSSTTLSTKV
GSINAADHTRKRGMVLPFTPLSITFDEIGYAVDMPQEMKAKGIPEDRLELLTGVNGAFRP
GVLTALMGISGAGKTTLMDVLSGRKTTGYVQGQITISGYPKKQETFSRISGYCEQTDIHS
PHVTVYESLVYSAWLRLPPEVDTSTRKMFIEEVMELIELTSIREALVGLPGVNGLSTEQR
KRLTIAVELVANPSIIFMDEPTSGLDARAAAIVMRTVRNTVDTGRTVVCTIHQPSIDIFD
AFDELLLLKRGGEEIYVGPLGRHCSHLINYFEGINGVPKIKNGYNPATWMLEVTSEAQEE
ALGINFAELYKNSDLYRTNKALIRELSTPPEGSKDLYFTTQHSQSFLTQCMACLWKQNLS
YWRNPPYSAVRLLFTTVIAFLFGTIFWNIGSKRERRQDLFNAMGSMYAAVLFIGVQNATS
VQPVVAIERTVFYREKAAGMYSALPYAFGQVAVEIPYILIQSLVYGVIVYTMVGFERTPT

KFFWYLFFMFFTFLYFTFFGMMLVGATPDHNVAAIVSFGFYLLWNLFSGFVIPRTRMPVW
WRWFFWICPISWTLYGLITTQFGDVNERMDTGETVEEFVRSYFGYRDDFKDVAAAVVVSF
SLIFGSAFAFSIKAFNFQKR*
>AtALS3
MDLKWDDFFNDYEWLIVFLKGMVKPAAALVVVLLAVILSYSQNLSLEGEMIYSVSRSFLQ
LSVIGFVLQFIFNQENSGWIILAYLFMVSVAGYTAGQRARHVPRGKYVAGLSILAGTSIT
MFLLVLLNVFPFTPRYMIPIAGMLVGNAMTVTGVTMKQLRDDIKMQLNLVETALALGATP
RQATLQQVKRALVISLSPVLDSCKTVGLISLPGAMTGMIMGGASPLEAIQLQIVVMNMMV
GAATVSSITSTYLCWPSFFTKAYQLQTHVFSSD
>AtABCI17
MPSLWSNESDGSLREHLVDVVVSGSEPKIRVHDLTRVADDGSRILKGVTIDIPKGMIVGV
IGPSGSGKSTFLRSLNRLWEPPESTVFLDGEDITNVDVIALRRRVGMLFQLPVLFQGTVA
DNVRYGPNLRGEKLSDEEVYKLLSLADLDASFAKKTGAELSVGQAQRVALARTLANEPEV
LLLDEPTSALDPISTENIEDVIVKLKKQRGITTVIVSHSIKQIQKVADIVCLVVDGEIVE
VLKPSELSHATHPMAQRFLQLSS
>AtABCG10
MELPVKAPIPGGREISYRLETKNLSYRIGGNTPKFSNLCGLLSEKEEKVILKDVSCDARS
AEITAIAGPSGAGKTTLLEILAGKVSHGKVSGQVLVNGRPMDGPEYRRVSGFVPQEDALF
PFLTVQETLTYSALLRLKTKRKDAAAKVKRLIQELGLEHVADSRIGQGSRSGISGGERRR
VSIGVELVHDPNVILIDEPTSGLDSASALQVVTLLKDMTIKQGKTIVLTIHQPGFRILEQ
IDRIVLLSNGMVVQNGSVYSLHQKIKFSGHQIPRRVNVLEYAIDIAGSLEPIRTQSCREI
SCYGHSKTWKSCYISAGGELHQSDSHSNSVLEEVQILGQRSCKNIFRTKQLFTTRALQAS
IAGLILGSIYLNVGNQKKEAKVLRTGFFAFILTFLLSSTTEGLPIFLQDRRILMRETSRR
AYRVLSYVLADTLIFIPFLLIISMLFATPVYWLVGLRRELDGFLYFSLVIWIVLLMSNSF
VACFSALVPNFIMGTSVISGLMGSFFLFSGYFIAKDRIPVYWEFMHYLSLFKYPFECLMI
NEYRGDVFLKQQDLKESQKWSNLGIMASFIVGYRVLGFFILWYRCYRTRS
>OsABCG25
MAASQLLAAAVAAAVFLAALLVPPARCQQQQVANPGPRVRQAARIDAVRDELAAEVQAKY
GFCMANVQEDFTQAFSFSNASFVSDCMEETQGQMTGMLCGKAEIEIYVKSLGKKPSTRVS
RNCDQNSWALGCQPGWACARQDSSSSGREVPSRAVNCRPCYPGFFCPRGLTCMIPCPLGA
YCPLATLNDTTGLCDPYSYQITPGSNTACGTADSWADVITTDDVFCPPGHHCPTTTQKFN
CTEGYYCRKGSTEEHKCIWKNTCKENSTKEATALFGGILIVILSVVLLLVYNCSDQFIKI
RAKILSKSRRKAATIAQESATARGRWKLAKELVLSHELEMSESDQLAASSNEARHATEGN
GKRSKNRKKLAHARTERFRRAYSQIGRERVLQPDNDKITLSGVVALAAENRSRRPMFEVV
FKGLTLSIGKKKLLQCVTGKLSPGRVTAIMGPSGAGKTTFLNAVLGKTTGYKKDGLVLIN
GKSGSMQSYKKIIGFVPQDDIVHGNLTVEENLWFSACCRSSKGMSKSDKIIVLERVIGSL
GLQEIRNSLVGTVEKRGISGGQRKRVNVGIEMVMEPSLLILDEPTTGLDSASSQLLLRAL
RHEALQGVNVCAVIHQPSYTLFNMFDDFVLLARGGLIAYLGPISEVETYFSSLGIKVPER
ENPPDYYIDILEGITKTKMRGHAAPKHLPLLWMLRNGYEVPEYMQKDLEDINNVHELYTV
GSMSREESFGDQSENADSVHQNVREPYSLLDRKTPGVLAQYKYYLGRVTKQRLREATLQA
VDYLILCIAGICIGTIAKVKDDTFGVASYGYTIIAVSLLCQLAALRSFSPERLQYWRERE
SGMSTLAYFLARDTIDHFNTLVKPVAFLSTFYFFNNPRSEFKDNYLVFLALVYCVTGIGY
TFAIWFELGLAQLCSALIPVVLVLVGTQPNIPNFIKGLCYPKWALEALIIAGAKKYSGVW
LITRCGALLKGGYDINNFVLCIVIVMLMGVLFRFIALLSLLKLK
>Lr34
meglaretnpsshhqdftacasderpdeselelasrqrqngaantehvsenmlldssklg
alkrreffdnllknleddhlrflrgqkeridrvdvklpaievrynnlfveaecrvtkgnh
lpslwnstkgafsglvkllgfeteraktnvledvsgiikpcrltlllgppgcgkstllra
lagkldkslkvtgdisyngyelhefvpektavyinqhdlhiaemtvretldfsaqcqgvg
rrpkilkevntresvagiipdadidlymkvvaveaserslqtdyilkimgleicadtmvg
damrrgisggqkkrlttaemivgpasayfmdeisngldssttfqiincfqqltniseytm
visllqptpevfdlfddlilmaegkiiyhgprnealnffeecgficperkaaadflqeil
swkdqqqywlgphesyryisphelssmfrenhrgrklheqsvppksqlgkealafnkysl
qklemfkacgareallmkrnmfvyvfktgqlaiialvtmsvflrtrmtisfthanyymga

lffsimimlngipemsmqigrlpsfykqksyyfysswayaipasvlkvpisildslvwis
ityygigytptvsrffcqflilcllhhsvtsqhrfiasyfqtpivsffylflaltvfltf
ggfilpktsmpgwlnwgfwispmtyaeisivineflaprwqkesiqnitignqilvnhgl
yyswhyywisfgallgsillfyiafglaldyrtpteeyhgsrptkslcqqqekdytiqne
sddqsniskakvtipvmhlpitfhnlnyyidtppemlkqgyptrrlrllnnitgalrpgv
lsalmgvsgagkttlldvlagrktggyiegdiriggypkvqetfvrilgyceqvdihspq
ltveesvtysawlrlpshvdeqtrskfvaevletveldqikdvlvgspqknglsmeqrkr
ltiavelvsnpsiilmdepttgldtrsaaiviravknicetgrtvvctihqpsteifeaf
delilmksggktiysgpigersckvieyfekisgvpkiksncnpatwmmdvtstsmevqh
nmdfailyeesslhreaedlveqlsiplpnsenlcfshsfaqngwiqlkaclwkqnityw
rspqynlrrimmtvisaliygilfwkhakvlnneqdmlsvfgamylgfttigayndqtii
pfstterivmyrerfagmysswsysfaqafieipyvfiqvvlytlivypstgyywtahkf
lwffyttfcsilsyvyvglllvsitpnvqvatilasffntmqtlfsgfilpapqipkwwt
wlyyltptswalnalltsqygniekevkafgetksvsiflndyfgfhqdklsvvaavlva
fpfvliilfslsieklnfqkr
>AtABCG25
MSAFDGVENQMNGPDSSPRLSQDPREPRSLLSSSCFPITLKFVDVCYRVKIHGMSNDSCN
IKKLLGLKQKPSDETRSTEERTILSGVTGMISPGEFMAVLGPSGSGKSTLLNAVAGRLHG
SNLTGKILINDGKITKQTLKRTGFVAQDDLLYPHLTVRETLVFVALLRLPRSLTRDVKLR
AAESVISELGLTKCENTVVGNTFIRGISGGERKRVSIAHELLINPSLLVLDEPTSGLDAT
AALRLVQTLAGLAHGKGKTVVTSIHQPSSRVFQMFDTVLLLSEGKCLFVGKGRDAMAYFE
SVGFSPAFPMNPADFLLDLANGVCQTDGVTEREKPNVRQTLVTAYDTLLAPQVKTCIEVS
HFPQDNARFVKTRVNGGGITTCIATWFSQLCILLHRLLKERRHESFDLLRIFQVVAASIL
CGLMWWHSDYRDVHDRLGLLFFISIFWGVLPSFNAVFTFPQERAIFTRERASGMYTLSSY
FMAHVLGSLSMELVLPASFLTFTYWMVYLRPGIVPFLLTLSVLLLYVLASQGLGLALGAA
IMDAKKASTIVTVTMLAFVLTGGYYVNKVPSGMVWMKYVSTTFYCYRLLVAIQYGSGEEI
LRMLGCDSKGKQGASAATSAGCRFVEEEVIGDVGMWTSVGVLFLMFFGYRVLAYLALRRI
KH
>AtABCG31
MAAASNGSEYFEFDVETGRESFARPSNAETVEQDEEDLRWAAIGRLPSQRQGTHNAILRR
SQTQTQTSGYADGNVVQTIDVKKLDRADREMLVRQALATSDQDNFKLLSAIKERLDRVGM
EVPKIEVRFENLNIEADVQAGTRALPTLVNVSRDFFERCLSSLRIIKPRKHKLNILKDIS
GIIKPGRMTLLLGPPGSGKSTLLLALAGKLDKSLKKTGNITYNGENLNKFHVKRTSAYIS
QTDNHIAELTVRETLDFAARCQGASEGFAGYMKDLTRLEKERGIRPSSEIDAFMKAASVK
GEKHSVSTDYVLKVLGLDVCSDTMVGNDMMRGVSGGQRKRVTTGEMTVGPRKTLFMDEIS
TGLDSSTTFQIVKCIRNFVHLMDATVLMALLQPAPETFDLFDDLILLSEGYMVYQGPRED
VIAFFESLGFRLPPRKGVADFLQEVTSKKDQAQYWADPSKPYQFIPVSDIAAAFRNSKYG
HAADSKLAAPFDKKSADPSALCRTKFAISGWENLKVCFVRELLLIKRHKFLYTFRTCQVG
FVGLVTATVFLKTRLHPTSEQFGNEYLSCLFFGLVHMMFNGFSELPLMISRLPVFYKQRD
NSFHPAWSWSIASWLLRVPYSVLEAVVWSGVVYFTVGLAPSAGRFFRYMLLLFSVHQMAL
GLFRMMASLARDMVIANTFGSAAILIVFLLGGFVIPKADIKPWWVWGFWVSPLSYGQRAI
AVNEFTATRWMTPSAISDTTIGLNLLKLRSFPTNDYWYWIGIAVLIGYAILFNNVVTLAL
AYLNPLRKARAVVLDDPNEETALVADANQVISEKKGMILPFKPLTMTFHNVNYYVDMPKE
MRSQGVPETRLQLLSNVSGVFSPGVLTALVGSSGAGKTTLMDVLAGRKTGGYTEGDIRIS
GHPKEQQTFARISGYVEQNDIHSPQVTVEESLWFSASLRLPKEITKEQKKEFVEQVMRLV
ELDTLRYALVGLPGTTGLSTEQRKRLTIAVELVANPSIIFMDEPTSGLDARAAAIVMRTV
RNTVDTGRTVVCTIHQPSIDIFEAFDELLLMKRGGQVIYGGKLGTHSQVLVDYFQGINGV
PPISSGYNPATWMLEVTTPALEEKYNMEFADLYKKSDQFREVEANIKQLSVPPEGSEPIS
FTSRYSQNQLSQFLLCLWKQNLVYWRSPEYNLVRLVFTTIAAFILGTVFWDIGSKRTSSQ
DLITVMGALYSACLFLGVSNASSVQPIVSIERTVFYREKAAGMYAPIPYAAAQGLVEIPY
ILTQTILYGVITYFTIGFERTFSKFVLYLVFMFLTFTYFTFYGMMAVGLTPNQHLAAVIS
SAFYSLWNLLSGFLVQKPLIPVWWIWFYYICPVAWTLQGVILSQLGDVESMINEPLFHGT
VKEFIEYYFGYKPNMIGVSAAVLVGFCALFFSAFALSVKYLNFQRR
>AtABCG30

MIQTGEEDEEKATSLEVEFASGNGVDDEEELRLQWATVERLPTFKRVTTALLARDEVSGK
GRVIDVTRLEGAERRLLIEMLVKQIEDDNLRLLRKIRKRIDKVGIELPTVEVRFNNLSVE
AECQVIHGKPIPTLWNTIKGLLSEFICSKKETKIGILKGVSGIVRPGRMTLLLGPPGCGK
TTLLQALSGKFSDSVKVGGEVCYNGCSLSEFIPEKTSSYISQNDLHIPELSVRETLDFSA
CCQGIGSRMEIMKEISRMEKLQEIIPDPAVDAYMKATSVEGLKNNLQTDYILKILGLDIC
ADTRVGDATRPGISGGEKRRLTTGELVVGPATTLFMDEISNGLDSSTTFQIVSCLQQLAH
IAEATILISLLQPAPETFELFDDVILMGEGKIIYHAPRADICRFFEEFGFKCPERKGVAD
FLQEIMSKKDQEQYWCHRDKPYSYISVDSFINKFKESNLGLLLKEELSKPFNKSQTRKDG
LCYKKYSLGKWEMLKACSRREFLLMKRNSFIYLFKSALLVFNALVTMTVFLQVGATTDSL
HGNYLMGSLFTALFRLLADGLPELTLTISRLGVFCKQKDLYFYPAWAYAIPSIILKIPLS
VLDSFIWTLLTYYVIGYSPEVKRFFLQFLILSTFNLSCVSMFRAIAAIFRTIIASTITGA
ISILVLSLFGGFVIPKSSMPAWLGWGFWLSPLSYAEIGLTANEFFSPRWSKVISSKTTAG
EQMLDIRGLNFGRHSYWTAFGALVGFVLFFNALYVLALTYQNNPQRSRAIISHEKYSRPI
EEDFKPCPKITSRAKTGKIILPFKPLTVTFQNVQYYIETPQGKTRQLLSDITGALKPGVL
TSLMGVSGAGKTTLLDVLSGRKTRGIIKGEIKVGGYPKVQETFARVSGYCEQFDIHSPNI
TVEESLKYSAWLRLPYNIDSKTKNELVKEVLETVELDDIKDSVVGLPGISGLSIEQRKRL
TIAVELVANPSIIFMDEPTTGLDARAAAIVMRAVKNVAETGRTVVCTIHQPSIDIFETFD
ELILMKNGGQLVYYGPPGQNSSKVIEYFESFSGLPKIQKNCNPATWILDITSKSAEEKLG
IDFSQSYKDSTLYKQNKMVVEQLSSASLGSEALRFPSQFSQTAWVQLKACLWKQHYSYWR
NPSHNITRIVFILLDSTLCGLLFWQKAEDINNQQDLISIFGSMYTLVVFPGMNNCAAVIN
FIAAERNVFYRERFARMYSSWAYSFSQVLIEVPYSLLQSLLCTIIVYPTIGYHMSVYKMF
WSLYSIFCSLLIFNYSGMLMVALTPNIHMAVTLRSSFFSMLNLFAGFVIPKQKIPKWWIW
MYYLSPTSWVLEGLLSSQYGDVDKEILVFGEKKRVSAFLEDYFGYKHESLAVVAFVLIAY
PIIVATLFAFFMSKLSFQKK
>AtABCC3
MDFLGSTTGSGTLAMLFSFSESILPLDSRSFLLKPLFLRWLSGFLHSVLLLVLFFSWVRK
KIRGDSGVTESLKDRRDFGFKSALFCSLALSLLNLVLMSLSGFYWYESGWLDNEQLVSSL
GFLLGMVSWGVLSICLHRCRDCEHKKAPFLLRLWLVFYLVVSCYSLVVDFVMYERRETVP
VHLLVFDIVAFIAAVFLGYVAVLKKDRSNSNGVLEEPLLNGGDSRVGGDDSVELNKTNGS
GEATPYSRAGILSLLTFSWMSPLIDIGNKKTLDLEDVPQLHDTDSVVGLAPKFRSMLESP
DGGERSGVTTFKLIKALYFTAQWEILVTAFFAFIYTVASYVGPALIDTFVQYLNGRRQYN
HEGYVLVITFFAAKIVECLSQRHWFFRLQKVGIRMRSALVAMIYEKGLTLSCQSKQGRTS
GEIINFMTVDAERIGNFSWYMHDPWMVLLQVGLALWILYRNLGLASIAALVATIIVMLIN
FPFGRMQERFQEKLMEAKDSRMKSTSEILRNMRILKLQGWEMKFLSKIFDLRKSEEGWLK
KYVYNSAVISFVFWGAPTLVSVSTFGACILLGIPLESGKILSALATFRILQEPIYNLPDT
ISMIVQTKVSLDRLASYLCLDNLQPDIVERLPKGSSDVAVEVINSTLSWDVSSSNPTLKD
INFKVFPGMKVAVCGTVGSGKSSLLSSLLGEVPKVSGSLKVCGTKAYVAQSPWIQSGKIE
DNILFGKPMERERYDKVLEACSLSKDLEILSFGDQTVIGERGINLSGGQKQRIQIARALY
QDADIYLFDDPFSAVDAHTGSHLFKEVLLGLLCSKSVIYVTHQVEFLPAADLILVMKDGR
ISQAGKYNDILNSGTDFMELIGAHQEALAVVDSVDANSVSEKSALGQENVIVKDAIAVDE
KLESQDLKNDKLESVEPQRQIIQEEEREKGSVALDVYWKYITLAYGGALVPFILLGQVLF
QLLQIGSNYWMAWATPVSEDVQAPVKLSTLMIVYVALAFGSSLCILLRATLLVTAGYKTA
TELFHKMHHCIFRSPMSFFDSTPSGRIMSRASTDQSAVDLELPYQFGSVAITVIQLIGII
GVMSQVSWLVFLVFIPVVAASIWYQRYYIAAARELSRLVGVCKAPLIQHFSETISGATTI
RSFSQEFRFRSDNMRLSDGYSRPKFYTAGAMEWLCFRLDMLSSLTFVFSLVFLVSIPTGV
IDPSLAGLAVTYGLSLNTLQAWLIWTLCNLENKIISVERILQYASVPSEPPLVIESNRPE
QSWPSRGEVEIRDLQVRYAPHMPLVLRGITCTFKGGLRTGIVGRTGSGKSTLIQTLFRIV
EPSAGEIRIDGVNILTIGLHDLRLRLSIIPQDPTMFEGTMRSNLDPLEEYTDDQIWEALD
KCQLGDEVRKKEQKLDSSVSENGDNWSMGQRQLVCLGRVLLKRSKILVLDEATASVDTAT
DNLIQKTLREHFSDCTVITIAHRISSVIDSDMVLLLSNGIIEEYDTPVRLLEDKSSSFSK
LVAEYTSRSSSSFD
>AtABCG40
MEGTSFHQASNSMRRNSSVWKKDSGREIFSRSSREEDDEEALRWAALEKLPTFDRLRKGI
LTASHAGGPINEIDIQKLGFQDTKKLLERLIKVGDDEHEKLLWKLKKRIDRVGIDLPTIE

VRFDHLKVEAEVHVGGRALPTFVNFISNFADKFLNTLHLVPNRKKKFTILNDVSGIVKPG
RMALLLGPPSSGKTTLLLALAGKLDQELKQTGRVTYNGHGMNEFVPQRTAAYIGQNDVHI
GEMTVRETFAYAARFQGVGSRYDMLTELARREKEANIKPDPDIDIFMKAMSTAGEKTNVM
TDYILKILGLEVCADTMVGDDMLRGISGGQKKRVTTGEMLVGPSRALFMDEISTGLDSST
TYQIVNSLRNYVHIFNGTALISLLQPAPETFNLFDDIILIAEGEIIYEGPRDHVVEFFET
MGFKCPPRKGVADFLQEVTSKKDQMQYWARRDEPYRFIRVREFAEAFQSFHVGRRIGDEL
ALPFDKTKSHPAALTTKKYGVGIKELVKTSFSREYLLMKRNSFVYYFKFGQLLVMAFLTM
TLFFRTEMQKKTEVDGSLYTGALFFILMMLMFNGMSELSMTIAKLPVFYKQRDLLFYPAW
VYSLPPWLLKIPISFMEAALTTFITYYVIGFDPNVGRLFKQYILLVLMNQMASALFKMVA
ALGRNMIVANTFGAFAMLVFFALGGVVLSRDDIKKWWIWGYWISPIMYGQNAILANEFFG
HSWSRAVENSSETLGVTFLKSRGFLPHAYWYWIGTGALLGFVVLFNFGFTLALTFLNSLG
KPQAVIAEEPASDETELQSARSEGVVEAGANKKRGMVLPFEPHSITFDNVVYSVDMPQEM
IEQGTQEDRLVLLKGVNGAFRPGVLTALMGVSGAGKTTLMDVLAGRKTGGYIDGNITISG
YPKNQQTFARISGYCEQTDIHSPHVTVYESLVYSAWLRLPKEVDKNKRKIFIEEVMELVE
LTPLRQALVGLPGESGLSTEQRKRLTIAVELVANPSIIFMDEPTSGLDARAAAIVMRTVR
NTVDTGRTVVCTIHQPSIDIFEAFDELFLLKRGGEEIYVGPLGHESTHLINYFESIQGIN
KITEGYNPATWMLEVSTTSQEAALGVDFAQVYKNSELYKRNKELIKELSQPAPGSKDLYF
PTQYSQSFLTQCMASLWKQHWSYWRNPPYTAVRFLFTIGIALMFGTMFWDLGGKTKTRQD
LSNAMGSMYTAVLFLGLQNAASVQPVVNVERTVFYREQAAGMYSAMPYAFAQVFIEIPYV
LVQAIVYGLIVYAMIGFEWTAVKFFWYLFFMYGSFLTFTFYGMMAVAMTPNHHIASVVSS
AFYGIWNLFSGFLIPRPSMPVWWEWYYWLCPVAWTLYGLIASQFGDITEPMADSNMSVKQ
FIREFYGYREGFLGVVAAMNVIFPLLFAVIFAIGIKSFNFQKR
>AtABCC1
MGFEPLDWYCKPVPNGVWTKTVDYAFGAYTPCAIDSFVLGISHLVLLILCLYRLWLITKD
HKVDKFCLRSKWFSYFLALLAAYATAEPLFRLVMRISVLDLDGAGFPPYEAFMLVLEAFA
WGSALVMTVVETKTYIHELRWYVRFAVIYALVGDMVLLNLVLSVKEYYGSFKLYLYISEV
AVQVAFGTLLFVYFPNLDPYPGYTPVGTENSEDYEYEELPGGENICPERHANLFDSIFFS
WLNPLMTLGSKRPLTEKDVWHLDTWDKTETLMRSFQKSWDKELEKPKPWLLRALNNSLGG
RFWWGGFWKIGNDCSQFVGPLLLNELLKSMQLNEPAWIGYIYAISIFVGVVLGVLCEAQY
FQNVMRVGYRLRSALIAAVFRKSLRLTNEGRKKFQTGKITNLMTTDAESLQQICQSLHTM
WSAPFRIIVALVLLYQQLGVASIIGALFLVLMFPIQTVIISKTQKLTKEGLQRTDKRIGL
MNEVLAAMDTVKCYAWENSFQSKVQTVRDDELSWFRKAQLLSAFNMFILNSIPVLVTVVS
FGVFSLLGGDLTPARAFTSLSLFSVLRFPLFMLPNIITQMVNANVSLNRLEEVLSTEERV
LLPNPPIEPGQPAISIRNGYFSWDSKADRPTLSNINLDIPLGSLVAVVGSTGEGKTSLIS
AMLGELPARSDATVTLRGSVAYVPQVSWIFNATVRDNILFGAPFDQEKYERVIDVTALQH
DLELLPGGDLTEIGERGVNISGGQKQRVSMARAVYSNSDVCILDDPLSALDAHVGQQVFE
KCIKRELGQTTRVLVTNQLHFLSQVDKILLVHEGTVKEEGTYEELCHSGPLFQRLMENAG
KVEDYSEENGEAEVDQTSVKPVENGNANNLQKDGIETKNSKEGNSVLVKREERETGVVSW
KVLERYQNALGGAWVVMMLVICYVLTQVFRVSSSTWLSEWTDSGTPKTHGPLFYNIVYAL
LSFGQVSVTLINSYWLIMSSLYAAKKMHDAMLGSILRAPMVFFQTNPLGRIINRFAKDMG
DIDRTVAVFVNMFMGSIAQLLSTVILIGIVSTLSLWAIMPLLVVFYGAYLYYQNTSREIK
RMDSTTRSPVYAQFGEALNGLSSIRAYKAYDRMAEINGRSMDNNIRFTLVNMAANRWLGI
RLEVLGGLMVWLTASLAVMQNGKAANQQAYASTMGLLLSYALSITSSLTAVLRLASLAEN
SLNSVERVGNYIEIPSEAPLVIENNRPPPGWPSSGSIKFEDVVLRYRPELPPVLHGVSFL
ISPMDKVGIVGRTGAGKSSLLNALFRIVELEKGRILIDECDIGRFGLMDLRKVLGIIPQA
PVLFSGTVRFNLDPFSEHNDADLWESLERAHLKDTIRRNPLGLDAEVTEAGENFSVGQRQ
LLSLARALLRRSKILVLDEATAAVDVRTDVLIQKTIREEFKSCTMLIIAHRLNTIIDCDK
VLVLDSGKVQEFSSPENLLSNGESSFSKMVQSTGTANAEYLRSITLENKRTREANGDDSQ
PLEGQRKWQASSRWAAAAQFALAVSLTSSHNDLQSLEIEDDNSILKKTKDAVVTLRSVLE
GKHDKEIEDSLNQSDISRERWWPSLYKMVEGLAVMSRLARNRMQHPDYNLEGKSFDWDNV
EM
>AtABCC2
MGFEFIEWYCKPVPNGVWTKQVANAFGAYTPCATDSFVLGISQLVLLVLCLYRIWLALKD
HKVERFCLRSRLYNYFLALLAAYATAEPLFRLIMGISVLDFDGPGLPPFEAFGLGVKAFA

WGAVMVMILMETKIYIRELRWYVRFAVIYALVGDMVLLNLVLSVKEYYSSYVLYLYTSEV
GAQVLFGILLFMHLPNLDTYPGYMPVRSETVDDYEYEEISDGQQICPEKHANIFDKIFFS
WMNPLMTLGSKRPLTEKDVWYLDTWDQTETLFTSFQHSWDKELQKPQPWLLRALNNSLGG
RFWWGGFWKIGNDCSQFVGPLLLNQLLKSMQEDAPAWMGYIYAFSIFVGVVFGVLCEAQY
FQNVMRVGYRLRSALIAAVFRKSLRLTNEGRRKFQTGKITNLMTTDAESLQQICQSLHTM
WSAPFRIIIALILLYQQLGVASLIGALLLVLMFPLQTVIISKMQKLTKEGLQRTDKRIGL
MNEVLAAMDTVKCYAWENSFQSKVQTVRDDELSWFRKSQLLGALNMFILNSIPVLVTIVS
FGVFTLLGGDLTPARAFTSLSLFAVLRFPLFMLPNIITQVVNANVSLKRLEEVLATEERI
LLPNPPIEPGEPAISIRNGYFSWDSKGDRPTLSNINLDVPLGSLVAVVGSTGEGKTSLIS
AILGELPATSDAIVTLRGSVAYVPQVSWIFNATVRDNILFGSPFDREKYERAIDVTSLKH
DLELLPGGDLTEIGERGVNISGGQKQRVSMARAVYSNSDVYIFDDPLSALDAHVGQQVFE
KCIKRELGQKTRVLVTNQLHFLSQVDRIVLVHEGTVKEEGTYEELSSNGPLFQRLMENAG
KVEEYSEENGEAEADQTAEQPVANGNTNGLQMDGSDDKKSKEGNKKGGKSVLIKQEERET
GVVSWRVLKRYQDALGGAWVVMMLLLCYVLTEVFRVTSSTWLSEWTDAGTPKSHGPLFYN
LIYALLSFGQVLVTLTNSYWLIMSSLYAAKKLHDNMLHSILRAPMSFFHTNPLGRIINRF
AKDLGDIDRTVAVFVNMFMGQVSQLLSTVVLIGIVSTLSLWAIMPLLVLFYGAYLYYQNT
AREVKRMDSISRSPVYAQFGEALNGLSTIRAYKAYDRMADINGRSMDNNIRFTLVNMGAN
RWLGIRLETLGGLMIWLTASFAVMQNGRAENQQAFASTMGLLLSYALNITSLLTGVLRLA
SLAENSLNAVERVGNYIEIPPEAPPVIENNRPPPGWPSSGSIKFEDVVLRYRPQLPPVLH
GVSFFIHPTDKVGIVGRTGAGKSSLLNALFRIVEVEKGRILIDDCDVGKFGLMDLRKVLG
IIPQSPVLFSGTVRFNLDPFGEHNDADLWESLERAHLKDTIRRNPLGLDAEVSEAGENFS
VGQRQLLSLSRALLRRSKILVLDEATAAVDVRTDALIQKTIREEFKSCTMLIIAHRLNTI
IDCDKILVLDSGRVQEFSSPENLLSNEGSSFSKMVQSTGAANAEYLRSLVLDNKRAKDDS
HHLQGQRKWLASSRWAAAAQFALAASLTSSHNDLQSLEIEDDSSILKRTNDAVVTLRSVL
EGKHDKEIAESLEEHNISREGWLSSLYRMVEGLAVMSRLARNRMQQPDYNFEGNTFDWDN
VEM
>OsABCG5
msrfvdklplfdrrpspmeeaeglprsgylgqlhhhqyyqphsnmlpleqspptstkhts
vtlaqllkrvndarsgsstpissprytielggskpesvssesddhhsddggsegqpralv
lkftdltysvkqrrkgsclpfrraaadepelpamrtlldgisgeardgeimavlgasgsg
kstlidalanriakeslhgsvtingesidsnllkvisayvrqedllypmltveetlmfaa
efrlprslptrekkkrvkelidqlglkraantiigdeghrgvsggerrrvsigvdiihnp
imlfldeptsgldstsafmvvtvlkaiaqsgsvvvmsihqpsyrilglldrllflsrgkt
vyygppselppffldfgkpipdnenptefaldlikemetetegtkrlaehnaawqlkhhg
egrgyggkpgmslkeaisasisrgklvsgatdgtvsvaasdhsapppssssvskfvnpfw
iemgvltrrafintkrtpevfiirlaavlvtgfilatifwrldespkgvqerlgffaiam
stmyytcsdalpvflseryiflretaynayrrssyvlshtivgfpslvvlsfafalttff
svglaggvngffyfvaivlasfwagsgfatflsgvvthvmlgfpvvlstlayfllfsgff
inrdriprywlwfhyislvkypyeavmqnefgdptrcfvrgvqmfdntplaalpaavkvr
vlqsmsaslgvnigtgtcittgpdflkqqaitdfgkweclwitvawgflfrilfyislll
gsrnkrr
>OsABCG15
MMEISSNEEMMEMAIVEQLPPSSHHLNGGSVEVDMEEDHVWPTKDGPLPIFLKFENVEYK
VKLTPKNPLTAARVAFASHKSTEDQGSCKHILKGIGGSVDPGEILALMGPSGSGKTTLLK
ILGGRLSGGVKGQITYNDTPYSPCLKRRIGFVTQDDVLFPQLTVEETLVFAAFLRLPARM
SKQQKRDRVDAIITELNLERCRHTKIGGAFVRGVSGGERKRTSIGYEILVDPSLLLLDEP
TSGLDSTSAAKLLVVLRRLARSAARRTVITTIHQPSSRMFHMFDKLLLVAEGHAIYHGGA
RGCMRHFAALGFSPGIAMNPAEFLLDLATGNLDGISSPASLLLPSAAAASPDSPEFRSHV
IKYLQARHRAAGEEEAAAAAAREGGGGGGAGRDEAAKQLRMAVRMRKDRRGGIGWLEQFT
VLSRRTFRERAADYLDKMRLAQSVGVALLLGLLWWKSQTSNEAQLRDQVGLIFYICIFWT
SSSLFGSVYVFPFEKLYLVKERKADMYRLSAYYASSTVCDAVPHVVYPVLFTAILYFMAD
LRRTVPCFCLTLLATLLIVLTSQGTGELLGAAILSVKRAGVMASLVLMLFLLTGGYYVQH
IPKFIRWLKYVSFMHYGFNLLLKAQYHGHLTYNCGSRGGCQRLQSSPSFGTVDLDGGMRE
VWILLAMAVAYRLLAYLCLRKRISLMPL

>OsABCG26
MKKQGGGGGVAAAAVAAAGMVKAELEDVGIKAAGGAVAALSPLSETLWREKAAAEFLGDV
SARLAWRDLTVTVVLGGGGGGGGGGTTQDVLQGLTGHAEPGTITALMGPSGSGKSTLLDA
LAGRLAANAFLDGTVLLNGRKANLSFGAAAYVTQDDNLIGTLTVRETISYSARLRLPDKM
PMEEKRALVEGTIVEMGLQDCADTVVGNWHLRGISGGEKRRVSIALEILMRPRLLFLDEP
TSGLDSASAFFVTQTLRGLARDGRTVIASIHQPSSEVFELFDRLYLLSGGKTVYFGLASE
ACQFFAQAGFPCPPLRNPSDHFLRCINADFDKVKATLKGSMKRRFERSDDPLDRIMTSEA
IRRLITYYKNSQYYFAAQQKVNEMARVKGTVLDAGGSQASFWMQAFTLTKRSFINMSRDF
GYYWLRLIIYIVVTVCIGTIYLNVGTRYSSILARGACASFVFGFVTFMSIGGFPSFVEDM
KVFQRERLNGHYGVLAFVISNTISAMPFLILITFISGTMCYFMVRLHPGFTHYLFFVLCL
YASVTVVESLMMAIASVIPNFLMGIIIGAGIQGIFMLVSGYFRLPHDIPKPFWRYPMSYI
SFHYWALQGQYQNDLKGLVFDNQDDELPKIPGEYILENVFQIDVSRSKWLDLAVLFSMIF
IYRLLFFAMIKVSEDVTPWVRGYVARRRVQGKGARGRGADLSAARSPSLRAYVVDAADDL
PPA
>OsABCG9
MRAGGGGGGDTRRTAAGQAMVELQANASSAGGGMVVGLSPLSETLWRDSKAMPGAAAALI
GDVSARLTWKDLSVTVALGPGKTQTVLDELTGYAEPGSLTALMGPSGSGKSTLLDALAGR
LAANAFLSGNVLLNGRKAKLSFGAAAYVTQDDNLIGTLTVRETIGYSAMLRLPDKMPRED
KRALVEGTIVEMGLQDCADTVIGNWHLRGVSGGEKRRVSIALELLMRPRLLFLDEPTSGL
DSSSAFFVTQTLRGLARDGRTVIASIHQPSSEVFELFDMLFLLSSGKTVYFGQASQACEF
FAQTGFPCPPLRNPSDHFLRCVNSDFDKVKATLKGSMKARIERSDDPLDRMTTSEAIRKL
VASYSRSQYYYAARERVNDISRLKGTVLDSGGSQASFLMQAGTLTKRSFINMSRDFGYYW
LRLLIYLLVTVCIGTIYYDVGTKYTSILARAACTAFVFGFVTFMSIGGFPSFVEEMKVFQ
RERLNGHYGVAAFVISNTISALPFLVLICFLSGTICYFMVRLHPGFSHYIFFVLNLYASV
TVVESLMMAIASVIPNFLMGIIIGAGIQGIFMLVSGYFRLPYDIPKPVWRYPMQYISFHY
WALQGQCQNDMDGLVFDNQYPDQPKIPGDFILKYIFQINVHRSKWIDLSVIFSMIFIYRI
LFFLMIKVNEDALPWIRGYIARKRLQKKEPLGKTPSLRGYVVDPELGPNES
>PtABCC1
MGFEALDWYCKPVRDGVWTKAVQNAFGAYTPCATDTLVVSLSYLVLMALCFYKIWLTKKDFKLQRFCLRSK
WYAYLLALLALYSTAEPLYRLVMGISVLNLDGQTGLAPFEIVSLIIEALAWCSLLVMIVVEIKVYIREFRWFVRF
GVIYTLVGDAVMLNLILTVKEFYNNAVLHLYISEVIVQGLFGILLLVYVPDLDPYPGYTPMQIESVDDAEYEEL
PGGEYICPERHANIISKIVFGWMSPLMKLGYRRPITEKDVWKLDTWDRTETLNDRFQKCWAEELRKPKPWLLR
ALHSSLGGRFWWGGFWKIGNDASQFVGPLVLNQLLKSMQEGDPAWIGYVYAFSIFAGVVFGVLCEAQYFQN
VMRVGYRLRATLVAAVFRKSLRLTHEGRRKFASGKITNLMTTDAEALQQICQSLHTLWSAPFRIIVAMVLLYQ
QLNVASLLGALMLVLLFPIQTFVISRMQKLSKEGLQRTDKRIGLMNEILAAMDTVKCYAWESSFQAKVQGVR
DDELSWFRKASLLGACNSFILNSIPVMVTVISFGMYTLLGGNLTPARAFTSLSLFAVLRFPLFMLPNMITQVVNA
NVSLKRLEELFLAEERILLPNPLLDPCLPAVSIKNGYFSWDSKAERPTLSNINLDVPIGSLVAVVGSTGEGKTSLV
SAMLGELPATSDASVVIRGTVAYVPQVSWIFNATVRDNILFGSPFDSARYEKAIDVTALQHDLDLLPGGDLTEI
GERGVNISGGQKQRVSMARAVYSNSDVYIFDDPLSALDAQVGRQVFDKCIKGELSKKTRILVTNQLHFLSQVD
RIILVHEGMVKEEGTFEDLSNNGMLFQKLMENAGKMEEYEEQENNEIVDHKTSSKQVANGVMNNLPKNVSG
TKKPKEGKSVLIKQEERETGVVNLKVLIRYKNALGGAWVVMVLFMCYLMTEVLRVSSSTWLSNWTNQGTSK
RHGPLYYNLIYSFLSIGQVSVTLLNSYWLITSSLYAAKRLHDAMLNSILRAPMVFFHTNPLGRIINRFAKDLGDI
DRNVAIFVNMFMGQISQLLSTFVLIGIVSTMSLWAIMPLLVLFYGAYLYYQSTAREVKRLDSITRSPVYAQFGE
ALNGLSTIRAYKAYDRMASINGKSMDNNVRYTLVNMGANRWLAIRLETLGGIMIWFTATFAVMQNGRADNQ
QAFASTMGLLLSYALNITSLLTAVLRLASLAENSLNSVERVGTYIELPSEAPLVIESNRPPPGWPSSGAIKFEDVV
LRYRPELLPVLHGLSFTIFPSDKVGIVGRTGAGKSSMLNALFRIVELERGRILIDDCNISKFGLMDLRKVLGIIPQ
APVLFSGTVRFNLDPFSEHNDADLWEALERAHLKDVIRRNSLGLDSEVTEAGDNFSVGQRQLLSLARALLRRS
KILVLDEATAAVDVRTDALIQKTIREEFRSCTMLIIAHRLNTIIDCDRVILLDSGRVLEYDTPEELLSNENSAFSK
MVQSTGAANAQYLRSLVMGGERESRSGREENKQLDGPRRWLASSRWAAAAQFALAVSLTSSQNDLQQLEIE
DENSVLKKTKDAVVTLQRVLEGKHDKVIDESLNQYQISRDGWWSALYKMVEGLAMMSRLGRNRLHQSDYG
LEDKTIDWNHVEM

>AcABCG38
MSRFVDKLSFFERRSSPMEEAEDVARNGLLGHHHPLSPPPHPPPPPPPPPQPPAPDSSPSATLAQLLKRVGDARS

DASSPASSPSHYVLELGEVAAPRPP
QHQPFVLSFTDLSYSVKQRQEGGGGACLPYRGSNRLATDPIAAGGGDGARTKTLLDSISGEAREGEILAVLGAS
GSGKSTLIDALANRIAKESLQGSVTL
NGETLDGQLLKVISAYVMQDDLLYPMLTVEETLMFSAEFRLPRSLSASKKKSRVQALIDQLGLRAATKTIIGDE
IHRGVSGGERRRVSIGIDIIHDPIVL
FLDEPTSGLDSTSAFMVVKVLQRIAQSGSIVVMSVHQPSYRILCLLDRLLFLSRGQTVYYGPPDGLPLFFSEFGK
PIPENENPTEFALDLIRELESTNPA
GAASLVEFNRSWQQQKKPPHPKQHNNNHSSASPNNDNNNNTNNNNNMMSLKEAISASISRGKLVSGATDMG
GARADRSVSKFANPFWIEMWVLTKRSFTN
TKRMPELFIIRLAAVLVTGFILGTIFWRLDSSPKGVQERLGFFAIAMSTMFFTCADALPVFLNERYIFMRETAYN
AYRRSSYVLSHAVVGFPPLIFLSVA
FALTTFFAVGLAGGFSGFVYFVLIILAAFWAGSGFVTFLSGVVTHVMLGYTVVVSILAYFLLFSGFFINRDRIPA
YWIWFHYLSLVKYPYEGVMQNEFSD
PSKCFVRGTQMFDNTPLGGLPNALKLNLLKAMSASLGVNINNNTCIMTGADILKGQAITDLNKWNCFWVTVA
WGFLFRLLFYFALLLGSKNKRR
>AtABCB9
MEEKSSKKNDGGNQKVSFFKLFSFADKTDVVLMTVGTIAAAGNGLTQPFMTLIFGQLINA
FGTTDPDHMVREVWKVAVKFIYLAVYSCVVAFLQVSCWMVTGERQSATIRGLYLKTILRQ
DIGYFDTETNTGEVIGRMSGDTILIQDAMGEKVGKFTQLLCTFLGGFAIAFYKGPLLAGV
LCSCIPLIVIAGAAMSLIMSKMAGRGQVAYAEAGNVVEQTVGAIRTVVAFTGEKQATEKY
ESKLEIAYKTVVQQGLISGFGLGTMLAVIFCSYGLAVWYGAKLIMEKGYNGGQVINVIFA
VLTGGMSLGQTSPSLNAFAAGRAAAFKMFETIKRSPKIDAYDMSGSVLEDIRGDIELKDV
YFRYPARPDVQIFAGFSLFVPNGKTVALVGQSGSGKSTVISLIERFYDPESGQVLIDNID
LKKLQLKWIRSKIGLVSQEPVLFATTIKENIAYGKEDATDQEIRTAIELANAAKFIDKLP
QGLDTMVGEHGTQMSGGQKQRLAIARAILKNPKILLLDEATSALDAESERIVQDALVNLM
SNRTTVVVAHRLTTIRTADVIAVVHQGKIVEKGTHDEMIQDPEGAYSQLVRLQEGSKEEA
TESERPETSLDVERSGSLRLSSAMRRSVSRNSSSSRHSFSLASNMFFPGVNVNQTDEMED
EENNVRHKKVSLKRLAHLNKPEIPVLVLGSIAAMVHGTVFPIFGLLLSSSINMFYEPAKI
LKKDSHFWALIYIALGLTNFVMIPVQNYFFGIAGGKLIKRIRSMCFDKVVHQEISWFDDT
ANSRSLVGDALALIVQNIATVTTGLIIAFTANWILALIVLALSPFIVIQGYAQTKFLTGF
SADAKAMYEEASQVANDAVSSIRTVASFCAEEKVMDLYQQKCDGPKKNGVRLGLLSGAGF
GFSFFFLYCINCVCFVSGAGLIQIGKATFGEVFKVFFALTIMAIGVSQTSAMAPDSNKAK
DSAASIFDILDSTPKIDSSSDEGTTLQNVNGDIEFRHVSFRYPMRPDVQIFRDLCLTIPS
GKTVALVGESGSGKSTVISMIERFYNPDSGKILIDQVEIQTFKLSWLRQQMGLVSQEPIL
FNETIRSNIAYGKTGGATEEEIIAAAKAANAHNFISSLPQGYDTSVGERGVQLSGGQKQR
IAIARAILKDPKILLLDEATSALDAESERVVQDALDRVMVNRTTVVVAHRLTTIKNADVI
AVVKNGVIAEKGRHETLMKISGGAYASLVTLHMSAN
>AtABCB14
MDNIEPPFSGNIHAETEVKKEEKKKMKKESVSLMGLFSAADNVDYFLMFLGGLGTCIHGG
TLPLFFVFFGGMLDSLGKLSTDPNAISSRVSQNALYLVYLGLVNLVSAWIGVACWMQTGE
RQTARLRINYLKSILAKDITFFDTEARDSNFIFHISSDAILVQDAIGDKTGHVLRYLCQF
IAGFVIGFLSVWQLTLLTLGVVPLIAIAGGGYAIVMSTISEKSEAAYADAGKVAEEVMSQ
VRTVYAFVGEEKAVKSYSNSLKKALKLSKRSGLAKGLGVGLTYSLLFCAWALLFWYASLL
VRHGKTNGAKAFTTILNVIYSGFALGQAVPSLSAISKGRVAAANIFKMIGNNNLESSERL
ENGTTLQNVVGKIEFCGVSFAYPSRPNMVFENLSFTIHSGKTFAFVGPSGSGKSTIISMV
QRFYEPRSGEILLDGNDIKNLKLKWLREQMGLVSQEPALFATTIASNILLGKEKANMDQI
IEAAKAANADSFIKSLPNGYNTQVGEGGTQLSGGQKQRIAIARAVLRNPKILLLDEATSA
LDAESEKIVQQALDNVMEKRTTIVIAHRLSTIRNVDKIVVLRDGQVRETGSHSELISRGG
DYATLVNCQDTEPQENLRSVMYESCRSQAGSYSSRRVFSSRRTSSFREDQEKTEKDSKGE
DLISSSSMIWELIKLNAPEWLYALLGSIGAVLAGSQPALFSMGLAYVLTTFYSPFPSLIK
REVDKVAIIFVGAGIVTAPIYILQHYFYTLMGERLTSRVRLSLFSAILSNEIGWFDLDEN
NTGSLTSILAADATLVRSAIADRLSTIVQNLSLTITALALAFFYSWRVAAVVTACFPLLI
AASLTEQLFLKGFGGDYTRAYSRATSLAREAISNIRTVAAFSAEKQISEQFTCELSKPTK
SALLRGHISGFGYGLSQCLAFCSYALGLWYISVLIKRNETNFEDSIKSFMVLLVTAYSVA

ETLALTPDIVKGTQALGSVFRVLHRETEIPPDQPNSRLVTHIKGDIEFRNVSFAYPTRPE
IAIFKNLNLRVSAGKSLAVVGPSGSGKSTVIGLIMRFYDPSNGNLCIDGHDIKSVNLRSL
RKKLALVQQEPALFSTSIHENIKYGNENASEAEIIEAAKAANAHEFISRMEEGYMTHVGD
KGVQLSGGQKQRVAIARAVLKDPSVLLLDEATSALDTSAEKQVQEALDKLMKGRTTILVA
HRLSTIRKADTIVVLHKGKVVEKGSHRELVSKSDGFYKKLTSLQEAV
>AtABCB15
MGKEEEKESGRNKMNCFGSVRSIFMHADGVDWLLMGLGLIGAVGDGFTTPLVLLITSKLM
NNIGGSSFNTDTFMQSISKNSVALLYVACGSWVVCFLEGYCWTRTGERQTARMREKYLRA
VLRQDVGYFDLHVTSTSDVITSVSSDSFVIQDVLSEKLPNFLMSASTFVGSYIVGFILLW
RLAIVGLPFIVLLVIPGLMYGRALISISRKIREEYNEAGFVAEQAISSVRTVYAFSGERK
TISKFSTALQGSVKLGIKQGLAKGITIGSNGITFAMWGFMSWYGSRMVMYHGAQGGTVFA
VAAAIAIGGVSLGGGLSNLKYFFEAASVGERIMEVINRVPKIDSDNPDGHKLEKIRGEVE
FKNVKFVYPSRLETSIFDDFCLRVPSGKTVALVGGSGSGKSTVISLLQRFYDPLAGEILI
DGVSIDKLQVKWLRSQMGLVSQEPALFATTIKENILFGKEDASMDDVVEAAKASNAHNFI
SQLPNGYETQVGERGVQMSGGQKQRIAIARAIIKSPTILLLDEATSALDSESERVVQEAL
ENASIGRTTILIAHRLSTIRNADVISVVKNGHIVETGSHDELMENIDGQYSTLVHLQQIE
KQDINVSVKIGPISDPSKDIRNSSRVSTLSRSSSANSVTGPSTIKNLSEDNKPQLPSFKR
LLAMNLPEWKQALYGCISATLFGAIQPAYAYSLGSMVSVYFLTSHDEIKEKTRIYALSFV
GLAVLSFLINISQHYNFAYMGEYLTKRIRERMLSKVLTFEVGWFDRDENSSGAICSRLAK
DANVVRSLVGDRMALVVQTVSAVTIAFTMGLVIAWRLALVMIAVQPVIIVCFYTRRVLLK
SMSKKAIKAQDESSKLAAEAVSNVRTITAFSSQERIMKMLEKAQESPRRESIRQSWFAGF
GLAMSQSLTSCTWALDFWYGGRLIQDGYITAKALFETFMILVSTGRVIADAGSMTTDLAK
GSDAVGSVFAVLDRYTSIDPEDPDGYETERITGQVEFLDVDFSYPTRPDVIIFKNFSIKI
EEGKSTAIVGPSGSGKSTIIGLIERFYDPLKGIVKIDGRDIRSYHLRSLRRHIALVSQEP
TLFAGTIRENIIYGGVSDKIDEAEIIEAAKAANAHDFITSLTEGYDTYCGDRGVQLSGGQ
KQRIAIARAVLKNPSVLLLDEATSALDSQSERVVQDALERVMVGRTSVVIAHRLSTIQNC
DAIAVLDKGKLVERGTHSSLLSKGPTGIYFSLVSLQTTSG
>AtABCB19
MSETNTTDAKTVPAEAEKKKEQSLPFFKLFSFADKFDYLLMFVGSLGAIVHGSSMPVFFL
LFGQMVNGFGKNQMDLHQMVHEVSRYSLYFVYLGLVVCFSSYAEIACWMYSGERQVAALR
KKYLEAVLKQDVGFFDTDARTGDIVFSVSTDTLLVQDAISEKVGNFIHYLSTFLAGLVVG
FVSAWKLALLSVAVIPGIAFAGGLYAYTLTGITSKSRESYANAGVIAEQAIAQVRTVYSY
VGESKALNAYSDAIQYTLKLGYKAGMAKGLGLGCTYGIACMSWALVFWYAGVFIRNGQTD
GGKAFTAIFSAIVGGMSLGQSFSNLGAFSKGKAAGYKLMEIINQRPTIIQDPLDGKCLDQ
VHGNIEFKDVTFSYPSRPDVMIFRNFNIFFPSGKTVAVVGGSGSGKSTVVSLIERFYDPN
SGQILLDGVEIKTLQLKFLREQIGLVNQEPALFATTILENILYGKPDATMVEVEAAASAA
NAHSFITLLPKGYDTQVGERGVQLSGGQKQRIAIARAMLKDPKILLLDEATSALDASSES
IVQEALDRVMVGRTTVVVAHRLCTIRNVDSIAVIQQGQVVETGTHEELIAKSGAYASLIR
FQEMVGTRDFSNPSTRRTRSTRLSHSLSTKSLSLRSGSLRNLSYSYSTGADGRIEMISNA
ETDRKTRAPENYFYRLLKLNSPEWPYSIMGAVGSILSGFIGPTFAIVMSNMIEVFYYTDY
DSMERKTKEYVFIYIGAGLYAVGAYLIQHYFFSIMGENLTTRVRRMMLSAILRNEVGWFD
EDEHNSSLIAARLATDAADVKSAIAERISVILQNMTSLLTSFIVAFIVEWRVSLLILGTF
PLLVLANFAQQLSLKGFAGDTAKAHAKTSMIAGEGVSNIRTVAAFNAQSKILSLFCHELR
VPQKRSLYRSQTSGFLFGLSQLALYGSEALILWYGAHLVSKGVSTFSKVIKVFVVLVITA
NSVAETVSLAPEIIRGGEAVGSVFSVLDRQTRIDPDDADADPVETIRGDIEFRHVDFAYP
SRPDVMVFRDFNLRIRAGHSQALVGASGSGKSSVIAMIERFYDPLAGKVMIDGKDIRRLN
LKSLRLKIGLVQQEPALFAATIFDNIAYGKDGATESEVIDAARAANAHGFISGLPEGYKT
PVGERGVQLSGGQKQRIAIARAVLKNPTVLLLDEATSALDAESECVLQEALERLMRGRTT
VVVAHRLSTIRGVDCIGVIQDGRIVEQGSHSELVSRPEGAYSRLLQLQTHRI
>AtABCB21
MDSVIESEEGLKVDSPNRADAETSNSKIHEEDEKELKTESDLKEEKKKTEKNKQEEDEKT
KTVPFHKLFAFADSFDIILMILGTIGAVGNGLGFPIMTILFGDVIDVFGQNQNSSDVSDK
IAKVALKFVYLGLGTLVAALLQVSGWMISGERQAGRIRSLYLQTILRQDIAFFDVETNTG
EVVGRMSGDTVLIQDAMGEKVGKAIQLVSTFIGGFVIAFTEGWLLTLVMVSSIPLLVMSG

AALAIVISKMASRGQTSYAKAAVVVEQTVGSIRTVASFTGEKQAISNYNKHLVSAYRAGV
FEGASTGLGLGTLNIVIFCTYALAVWYGGKMILEKGYTGGQVLIIIFAVLTGSMSLGQAS
PCLSAFAAGQAAAYKMFEAIKRKPEIDASDTTGKVLDDIRGDIELNNVNFSYPARPEEQI
FRGFSLSISSGSTVALVGQSGSGKSTVVSLIERFYDPQSGEVRIDGINLKEFQLKWIRSK
IGLVSQEPVLFTSSIKENIAYGKENATVEEIRKATELANASKFIDKLPQGLDTMVGEHGT
QLSGGQKQRIAVARAILKDPRILLLDEATSALDAESERIVQEALDRIMVNRTTVVVAHRL
STVRNADMIAVIHQGKIVEKGSHSELLRDPEGAYSQLIRLQEDTKQTEDSTDEQKLSMES
MKRSSLRKSSLSRSLSKRSSSFSMFGFPAGIDTNNEAIPEKDIKVSTPIKEKKVSFFRVA
ALNKPEIPMLILGSIAAVLNGVILPIFGILISSVIKAFFKPPEQLKSDTRFWAIIFMLLG
VASMVVFPAQTIFFSIAGCKLVQRIRSMCFEKVVRMEVGWFDETENSSGAIGARLSADAA
TVRGLVGDALAQTVQNLASVTAGLVIAFVASWQLAFIVLAMLPLIGLNGYIYMKFMVGFS
ADAKRMYEEASQVANDAVGSIRTVASFCAEEKVMKMYKKKCEGPMRTGIRQGIVSGIGFG
VSFFVLFSSYAASFYAGARLVDDGKTTFDSVFRVFFALTMAAVAISQSSSLSPDSSKASN
AAASIFAVIDRESKIDPSDESGRVLDNVKGDIELRHISFKYPSRPDVQIFQDLCLSIRAG
KTIALVGESGSGKSTVIALLQRFYDPDSGQITLDGVEIKTLQLKWLRQQTGLVSQEPVLF
NETIRANIAYGKGGDATETEIVSAAELSNAHGFISGLQQGYDTMVGERGVQLSGGQKQRV
AIARAIVKDPKVLLLDEATSALDAESERVVQDALDRVMVNRTTVVVAHRLSTIKNADVIA
VVKNGVIVEKGKHETLINIKDGVYASLVQLHLSAST
>AtABCB24
MSRGSRFVRAPGLLLCRVNLQPQPKIPSFSYSLRSDYRLHNGFSNYIRRNSIRTSPVINA
FLSDNSPSPSPSPSPSPIRFVQRSSMLNGRLFSTSTPNPDQTTTKTKEIKTTSSDSDSAMAD
MKILRTLAGYLWMRDNPEFRFRVIAALGFLVGAKVLNVQVPFLFKLAVDWLASATGTGAS
LTTFAATNPTLLTVFATPAAVLIGYGIARTGSSAFNELRTAVFSKVALRTIRSVSRKVFS
HLHDLDLRYHLSRETGGLNRIIDRGSRAINFILSAMVFNVVPTILEISMVSGILAYKFGA
AFAWITSLSVGSYIVFTLAVTQWRTKFRKAMNKADNDASTRAIDSLINYETVKYFNNEGY
EAEKYDQFLKKYEDAALQTQRSLAFLNFGQSIIFSTALSTAMVLCSQGIMNGQMTVGDLV
MVNGLLFQLSLPLNFLGSVYRETIQSLVDMKSMFQLLEEKSDITNTSDAKPLVLKGGNIE
FENVHFSYLPERKILDGISFVVPAGKSVAIVGTSGSGKSTILRMLFRFFDTDSGNIRIDG
QDIKEVRLDSLRSSIGVVPQDTVLFNDTIFHNIHYGRLSATEEEVYEAARRAAIHETISN
FPDKYSTIVGERGLKLSGGEKQRVALARTFLKSPAILLCDEATSALDSTTEAEILNALKA
LASNRTSIFIAHRLTTAMQCDEIVVLENGKVVEQGPHDELLGKSGRYAQLWTQQNSSVDM
LDAAIKLE
>CjABCB1
MAEENGLDGDLNNHQATASTSNSPVQGANGTSETKGAQEKSEKSKEEEKIGQSVPYYKLL
SFADSKDVLLMVIGTIAAVANGASMPVMTLLLGDLINAFGQNANNTDTLRVVSKVALKFV
YLSIGAGVASFFQVACWMVTGERQAARIRSLYLKTILRQDVAFFDKETNTGEVVGRMSGD
TVLIQDAIGEKVGKFIQLFSTFIGGFLIAFVKGWLLTLVMLTSIPPLVFCGALMTITISK
MASRGQVAYSQAGIVVEQTIGSIRTVASFTGEKHAVTQYEKYLNKAYLAGIHEGLASGVG
LGSVLLVIFCSYSLAVWFGGKMIIEKGYNGGNVINIIVAVLTGSMSLGQASPCLGAFAAG
QAAAYKMLETIKRKPEIDSYDTSGHKSDDIRGDIELRDVSFTYPARPDEQIFNGFSLFIP
SGTTSALVGQSGSGKSTVISLIERFYDPQAGEVLIDGVNLKDFQLRWIRGKIGLVSQEPV
LFASSIRDNIAYGKDGATVEEIKAATERANASKFIDKLPQGLDTLVGEHGTQLSGGQKQR
IAIARAILKDPRILLLDEATSALDAESEHIVQEALDRIMVNRTTVIVAHRLSTVRNADTI
AVIHRGKIVEKGSHLDLLLNPDGAYCQLIRLQEIGRSEVDKAENVESGLNSSQQHSIGRS
ISRGSSGVGNSSRHSFSVSFGLPTGHIYETTAGLESTSPAPIGQTQEVPLRRLATLNKPE
IPVLLLGVISAMVNGVIFPIFGVLLSSVIKTFYEPEDKLRKDTRFWAFMFIILGVASFVA
APATAYFFAVAGCRLIQRIRSMCFRTVAHMEIDWFDEPEHASGAIGAKLSADASTVRGLV
GDALALLVQNAATAVCGLVIAFVANWTLALIILVLIPLIGVNGYVQMKFMKGFSADAKMM
YEEASQVANDAVGSIRTVASFCAEEKVMQLYKKKCEGPMKTGIRQGLISGIGFGISFFLL
FNVYATSFYAGARLVDAGKTTFSDVFRVFFALTMAALGISQSSSLAPDSSKAKSSTASIF
GILDRKSKIDSSDESGMTVENVKGEIELRHISFKYPTRPDIQIFRDLSLAIHSGKTVALV
GESGSGKSTVISLLQRFYDPDSGHITLDGIEIQKFQLRWLRLQMGLVSQEPVLFNETIRA
NIAYGKEGDATETEILAAAELANAHKFISGLQQGYDTVVGERGIQLSGGQKQRVAIARAM
VKAPKILLLDEATSALDAESERVVQDALDKVMVNRTTVWVAHRLSTIKNADVIAVVKNGV

IAEKGKHNDLINVKDGVYASLVALHTSAS
>CjABCB2
MAEENGFNGDQNNHLQATASTSHSPAKLTNKTSGSTKSDQQDSDKGEGVEKMSAETVPYY
KLFSFADSKDLVLMVIGTIASVANGASMPIMTFLVGDLINAFGQNANNKNTLPVVSRVAL
RFVYLAVGAGVASVFQVACWMVTGERQASRIRSLYLKTILRQDVAFFDKETNTGEVVGRM
SGDIVRIQDAMGEKVGKFIQLFSTFIGGFIVAFVRGWLLTLIMLSSIPVLVISGAFVTIV
VSKMASRGQAAYSQAAITVEQTIGSIRTVASFSGEKHAITQYEKSLQKAYKSGVHEGLAS
GLGLGASMLIFFCSYALAIWFGGRMIIEKDYTGGDIINIIDAILVGSFSLGQASPCLSAF
AAGQAAAFKMFETIKRKPEIDSYDTKGRVLDDIHGDIELKDICFSYPARPDEQIFSGFSL
SLPSGTTSALVGESGSGKSTVISLIERFYDPQAGEVLIDGINLKEFQLRWIRQKIGLVSQ
EPVLFASSIKDNIAYGKDGATLEDIKAAAELANAAKFIDKLPQGLDTLVGEHGTHLSGGQ
KQRVAIARAILKDPRILLLDEATSALDAESEHIVQEALDRVMVNRTTVVVAHRLSTIRSA
DMIAVVHRGKIVEKGSHSELLKDPDGAYSQLIRLQEVNRSSENKAESTEFGRSSSHQQSF
RRSMSRGSSGVGNSSRKSFSMSFGLPTPHIPEVVSAKPESTPEPKKQTEEVPLLRLASLN
KPEIPILLLGAISAAINGLIFPIFGVLLASVIKTFYKPEDELRKDSRFWALMFIVLGIAS
FVASPAGTYFFSVAGCRLIQRIRSMCFEKVVHMEINWFDEPEHSSGAIGAKLSSDAASVR
SLVGDALSLLVQNAASAIAGLAIAFEANWILALIILVLLPLIGLNGYLQTKFMTGFSADA
KMMYEEASQVASDAVGSIRTVASFCAEEKVMQLYKKKCEGPMKTGIRQGLISGIGFGVSF
FLLYNVYATSFYVGARLVEDGKTTFAEVFRVFFALTMAALGISQSSSFAPDSSKARASTA
SIYGILDRKSKIDSSDDSGITLENLNGDIELRHVSFKYSTRPDIQILRDLSLAIRSGKTV
ALVGESGSGKSTVISLLQRFYDPDSGYITLDGVEIQKLQLRWLRQQMGLVSQEPVLFNET
IRANIAYGKEGDATETEILAAAELANAHKFISALQQGYDTMVGERGVQLSGGQKQRVAIA
RAMVKAPKILLLDEATSALDAESERVVQDALDKVMVNRTTIVVAHRLSTIKNADLIAVVK
NGVIVEKGKHDHLINISDGVYASLVALHMTAS
>OsABCC1
MGFDPLEWYCQPVNGGVWSDVENAFGAYTPCGTETLVVCISYFALFGVCFYRIWRTTRDY
TVQRYKLRSPYYNYMLGLLVVLCIAESLYRIATGTSIMNLDGETSLAPFEVTSSIIEIAA
WCCMLVMIALETRIYIYEFRWYIRFVVIYILVGEAAMFNLVLSVRQYYSSSSIFYLYCSE
IIFKLLFGILMVVYLPSLDSYPGYTPVRHEALVDNTDYEPLPGGEQICPERHANIFSRIF
FSWMTPLMQQGFKRPITDKDIWKLDSWDETETLYNRFQKCWNNELQKPKPWLLRALHSSL
GGRFWLGGFFKIGNDASQFVGPLILNLLLESMQKGDPSWSGYIYAFSIFAGVSLGVLSEA
QYFQNVMRTGFRLRSTLIAAVFRKSLRLTNDSRKKFASGRITNLISTDAESLQQVCQQLH
SLWSAPFRIVIAMVLLYAQLGPAALVGAAMLVLLFPIQTVIISKMQKLTKEGLQRTDRRI
SLMNEILAAMDTVKCYAWEQSFQSKVQDIRDDEISWFRSAQLLAALNSFILNSIPVIVTV
VSFGVYSLLGGDLTPAKAFTSLSLFAVLRFPLFMLPNLITQVVNCKVSLKRLEDLLLAEE
RLLLPNPPLDPELPAISIKNGYFSWESQAERPTLSNVNLDVPMGSLVAIVGSTGEGKTSL
ISAMLGEIPPVSGSNTSVVLRGTVAYVPQVSWIFNATVRDNILFGSPFQPPRYEKAIDVT
SLRHDLDLLPGGDLTEIGERGVNISGGQKQRVSMARAVYSDSDVYIFDDPLSALDAHVGR
QVFDKCIKEELQHKTRVLVTNQLHFLPYVDKILVVHDGVIKEEGTFDELSNSGELFKKLM
ENAGKMEEQMEEKQDESQRQDDIKHPENGGSVIADGDMQKSQDTSNKTKQGKSVLIKQEE
RETGVISAKVLSRYKNALGGIWVVSVLFFCYALTEVLRISSSTWLSVWTDQGSTKIHGPG
YYNLIYGLLSFGQVLVTLTNSYWLITSSLRAAKRLHDAMLRSILRAPMVFFHTNPLGRII
NRFSKDLGDIDRNVAIFVNMFMAQISQLLSTFVLIGIVSTMSLWAIMPLLILFYAAYLYY
QTTSREVKRLDSITRSPVYAQFSEALNGLSTIRAYKAYDRMANINGKSMDNNIRFTLVNM
SSNRWLAIRLETLGGIMIWFTATFAVMQNQRAENQKAFASTMGLLLTYTLNITNLLTAVL
RLASLAENSLNAVERVGTYIELPSEAPPVIEDSRPPPGWPSSGVVKFEDVVLRYRPELPP
VLHGISFIINGSEKVGIVGRTGAGKSSMLNALFRIVELERGRILVDDCDTSKFGIWDLRK
VLGIIPQAPVLFSGSVRFNLDPFNEHNDADLWEALERAHLKDVIRRNALGLDAEVSEAGE
NFSVGQRQLLSLARALLRRAKILVLDEATAAVDVRTDALIQKTIREEFKSCTMLIIAHRL
NTVIDCDRLLILSAGKVLEFDSPENLLSNEHSAFSKMVQSTGPSNAEYLKTLVFGDGEER
LRKEESKMQDIQRKWVASNRWAVAAQFALAASLASSHSDLLALEAAEGNNILRKTKDAVI
TLQNVLEGKHNTEIDDTLAQYEVPSDRWWSSLYKVMEGLAMMSRLGRNRLQQPSYNFENN
SSIDWDQM
>LeATA63002.1

MTKENGDHGGKTRVDEASTSKNLSLEMENKDSSGKHESVDKKTNKDKEKTNVVPFYKLFIFADTKDKILM
FIGSVAAIGNGLSLPLMTVLFGELIDSFGETQSGDVVEKVSKVSLKFVYLAIGSGVASFFQVSCWMITGE
RQSARIRGLYLKTILRQDVSFFDMETNTGEVIGRMSGDTVLIQDATGEKVGKFLQLIATFIGGFVIAFTK
GWLLALVMLSAIPPLVLSGGLMAMTLSKMASAGQEAYAKAAIVVEQTIGSIRTVASFTGEKQAVADYDKS
LVKAYKSGVQEGFASGMGLGTVMCILFSSYALAIWFGSKMIIEKGYTGGQVLNVIISVLAGSMSLGQASP
CLTAFAAGRAAAFKMFETIDRKPEIDSYDTRGKILEDIRGDIELKEVYFSYPARPDEQIFRGFSLLIPSG
TTAALVGQSGSGKSTVISLIERFYDPAAGEVLIDGINLKEFQLKWIRQKISLVSQEPVLFTSSIKDNIAY
GKEGATTEEIRAASELANAAKFIDKLPQGLDTMVGEHGTQLSGGQKQRVAIARAILKDPRILLLDEATSA
LDAESERVVQEALDRIMVNRTTVIVAHRLSTVRNANMIAVIHQGKIVEKGTHNVLLKDPEGAYSQLIKLQ
ETNRDTDQAGGEGKDNINASMGSNRQSSQRMSFKRSISRDSSGLGSSSRRSISASYGVPAGVTVTEHHDP
EDTTKEASEKSPNVPIRRLAALNKPEIPVLILGSVAAIINGAIMPVFGILISSIIKSFYKPPHELQKDSR
FWALMFVLLGITSLIAYPARTYLFGIAGNRLIKRIRLMSFRKVVHMEINWFDEPQHSSGMIGARLSADAA
TVRALVGDALAQMVQDTSSLVTGLVIAFTASWQLALIIVAMIPLIGINGWVQIKFMKGFSADAKRMYEDA
SQVANDAVGSIRTVASFCAEDKVMELYKKKCEGPLQKGVKQGLISGIGFGVSFALLFLVYATSFYAGARL
VDDGKITFADVFRVFFALTMAAMAISQSSALAPDSSKAKSATASIFAILDRKSKIDSSDESGTTLETVKG
EIELRHVSFKYPHRPDVQIFKDLCLTIHSGKALALVGESGSGKSTVISLLQRFYDPDAGYITLDGIEIQK
FQLKWFRQQMGLVGQEPVLFNDTIRANIAYGKEGNATEAEIIAAAELANAHKFISGLAQGYDTVVGERGV
QLSGGQKQRVAIARAIVKSPKILLLDEATSALDAESERLVQDALDRVMVNRTTVVVAHRLSTIKGADVIA
VVKNGVIVEKGKHETLIKIKDGFYSSLVSLHMSAST
>LjABCB1
MRPENGGTHKHDGTSSNGEKSRQKEKVEIVPYHRLFTFADSTDILLMIVGTIGAIGNGLS
IPMMSLLFGQMVNSFGNNQFSPDIVNQVSKVSLKFVCLGIGNGVAAFLQVACWMITGERQ
ATRIRCLYLKTILRQNVAFFDKETNTGEVIGRMSGDTVLIQDAMGEKVGKLLQLIATFVG
GYVVAFIKGWLLTVVLLSALPLLVASGAAMALLIGKMTSRGQKAYAKAAHVAEQTIGSIK
TVASFTGEKQAVSSYRRYLAGAYKSGVYEGFVFGMGHGMIMLVVFCTFALAVWFGAKMII
EKGYNGGQVINIIIAVLTASMSLGQASPSMSAFAAGQAAAYKMFQTIERKPEIDAYDPNG
KILEDIHGDIDIKDVYFSYPTRPEELVFNGFSIHIPSGTTTALVGESGSGKSTIISLIER
FYDPLAGEVLIDSINMKDFQLRWIRGKIGLVSQEPALFASSIKDNIAYGKEGATIQEIRV
ALELANAAKFIDRLPQGLDTMVGDHGTQLSGGQKQRIAIARAILKDPRILLLDEATSALD
AQSQRTVQEALDRVMVNRTTVVVAHRLSTVRNADMIALIHRGKMIEKGTHVELLKDPGGA
YSQLIRLQEVNNESKESADNQNKRKLSTESRSSLGNSSRHTFSVSSGLPTGVDVPKAGNE
KLHPKEKSQEVPLLRLASLNKPEIPALLMGCVAAIANGAILPIYGVLLSSVIKTLYEPFP
DMKKDSKFWSLMFVVLGIASLMAIPARCYFFSVAGSRLIQRIRLVCFEKLINMEVGWFEE
PEHSIGAIGARLSTDAAFVRALVGDALGLLIQSISTALTGLIVAFIASWQLALIVVIIAP
LMGMNGYVQIKFMKGFSADAKMMYEEASQVASDAVGSIRTIASFCAEEKVMELYSKKCEG
PVKTGIQQGLISGIGFGVSFFLLFSVYATTFHAGARFVDAGMASFSDVFRVFFALTMTAI
GISRSSSLAPDSSKGKTATASIFEIIDQKSKIDPSDESGGKLDSIKGEIELSHVSFKYPS
RPDIQIFRDLSMTIHSGKTVALVGESGSGKSTVIALLQRFYDPDAGQITIDGIEIQKLQL
KWLRQQMGLVSQEPILFNDTIRANIAYGKEGNATEAEIITAAELANAHRFISGLEQGYDT
VVGERGILLSGGQKQRVAIARAIIKSPNILLLDEATSALDVESERVVQDALDKVMVNRTT
VIVAHRLSTIKSADVIIVLKNGVIVEKGRHETLISIKDGYYASLVQLHTTATTV
>OsABCB19
MADESGSCGGGGGGGGGCEAVKKRVDQSVAFHELFGFADPLDWLLMAAGSAGAVVHGAAM
PVFFLLFGELINGFGKNQHSLRRMTDEVSKYSLYFVYLGLVVCASSYLEIACWMYTGERQ
VGALRRRYLEAVLRQDVGFFDTDARTGDVVFSVSTDTLLVQDAIGEKVGNFIHYLSTFLA
GLVVGFVSAWRLALLSIAVIPGIAFAGGLYAYTLTGLTSKSRDSYANAGIIAEQAIAQVR
TVYSYVGESKALNSYSEAIQNTLKLGYKAGMAKGLGIGCTYGIACMSWALVFWYAGVFIR
NGQTDGGKAFTAIFSAIVGGLSLGQSFSNLGAFSKGKIAGYKLLEVIRQRPTIVQDPADG
RCLDEVHGNIEFKEVAFSYPSRPDVMIFRDFSLFFPAGKTAAVVGGSGSGKSTVVALIER
FYDPNQGQVLLDNVDIKTLQLKWLRDQIGLVNQEPALFATTILENILYGKPDATMAEVEA
AATSANAHSFIALLPNGYNTQVGERGLQLSGGQKQRIAIARAMLKNPKILLLDEATSALD
AGSENIVQEALDRLMVGRTTVVVAHRLSTIRCVDMIAVIQQGQVVETGTHDELLAKGSSG
AYAALIRFQEMARNRDFRGPSTRKSRSSRLSNSLSTRSLSLRSGSLRNLSYSYSTGADGR
IEMVSNADNDRKYPAPKGYFFKLLKLNAPEWPYTILGAIGSILSGFIGPTFAIVMSNMIE

VFYFRDPNAMERKTREYVFIYIGTGLYAVVAYLVQHYFFSIMGENLTTRVRRMMLAAILR
NDVGWFDQEENNSSLVAARLSTDAADVKSAIAERISVILQNMTSLLVSFVVGFIIEWRVA
VLILVTFPLLVLANFAQQLSMKGFAGDTAKAHAKTSMIAGEGVSNIRTVAAFNAQDKVLS
LFCTELRVPQMHSLRRSQISGALFGLSQLSLYASEALILWYGAHLVRHHVSTFSKVIKVF
VVLVITANTVAETVSLAPEIVRGGESIRSVFAILNYRTRIDPDEPETEPVESVRGDIDFR
HVDFAYPSRPDVMVFKDFSLRIRAGQSQALVGASGSGKSTVIALIERFYDPLAGKVMIDG
KDIRRLNVRSLRLKIGLVQQEPVLFATSIFENIAYGKDGATEEEVIEAAKVANMHGFVSA
LPEGYKTPVGERGVQLSGGQKQRIAIARAVLKDPAVLLLDEATSALDAESECVLQEALER
IMKGRTAVLVAHRLSTIRGVDSIAVVQDGRVVEQGSHGELVSRPDGAYSRLLQLQLHHG
>TwMDR1
MAGEDGLGNGDGNREEATTSRGPLEVEKRSSNGEGNTEDEQIGTVPFRKLFAFADSTDILFMI
IGTIGAIGNGLCMPLMTILFGDMINSFGANQSGDVVKVVSKVALKFVYLAIGAGFASFLQ
VTCWIVTGERQAARIRGLYLKTILRQDIAFFDLETNTGEVIGRMSGDTVLIQDAMGEKVG
KCIQLLATFIGGFAIAFIKGWLLALVMMSSIPLLVISGGVTSILISKMATRGQSSYAKAA
TVVEQTIGAIRTVASFTGEKHAISEYNKHLVSAYKSSVSEGLANGFGIGVVMLVVFGTYA
LAVWYGGKLILDKGYDGGQVLNVIVAVLTGSMSLGQASPCLSSFAAGQAAAYKMFETINR
KPEIDAYDTKGKVLDDIRGDIELKDVYFTYPARPDEQIFQGFSLFIPSGTTAALVGQSGS
GKSTVISLVERFYDPQAGQVLIDGINLKEFQLKWIREKIGLVSQEPALFTASIRENIAYG
KPGATNEEIRAATEMANAAKFIDKLPQGLDTMVGEHGTQLSGGQKQRIAIARAILKDPRI
LLLDEATSALDAESERIVQEALDRIMVNRTTVIVAHRLTTVRNADMISVIYRGKMVEKGT
HSELLKDPEGAYSQLIRLQEGNKESEADEPRRRDLSSESFRQSSQRMSFTRSISRASSRG
SSRRSFSVSFGLPTGITVTESAVEALEDAPPYDKGAPEVPLSRLAALNKPEIPVLFFGAI
AAAINGSIMPIFGILISKVVNTFYETPDQLKKDSRFWAFIFLALGFASFLAYPSMNYFFS
IAGGKLVRRIRALCFEKVVHMEVGWFDLTENSSGAIGARLSADAASVRALVGDALAQMVQ
NAATATAGLVIAFTASWQLSLIILALLPLLGFNGYVQVKFMKGFSADAKLMYEEASQVAN
DAVGSIRTVASFCSEEKVMQLYERKCEGPRKTGIRQGLISGIGFGLSFFLLFNVYATSFY
AGARFVKAGVITFADVFQVFFALTMAAVGVTQSSSMGSDSTKAKNAAASIFSMIDRKVLI
DASDESGVKLDSVKGEIELRHISFRYPARPDIHILRDLCLSIRPGKTVALVGESGSGKST
VIALLQRFYDPESGRITIDGTELQKFNLRWMRQQMGLVSQEPSLFNDTIRANIAYGKQGN
ATEAEILAAAEASNAHKFISSLNQGYDTMVGERGVQMSGGQKQRVAIARAIVKDPKILLL
DEATSALDAESERVVQDALDRVMVNRTTVVVAHRLSTIKNADVIAVVKNGVIVEKGRHET
LITIKDGVYASLVALHTSAS
>ZmABCB1
MSSSDPEEIRARVVVLGSPHADGGDEWARPELEAFHLPSPAHQPPGFLAGQPEAAEQPTLPAPAGRSSSS
SNTPTTSAGGGAAPPPPSSPPPPPASLETEQPPNARPASAGANDSKKPTPPAALRDLFRFADGLDCALML
IGTLGALVHGCSLPVFLRFFADLVDSFGSHADDPDTMVRLVVKYAFYFLVVGAAIWASSWAEISCWMWTG
ERQSTRMRIRYLDAALRQDVSFFDTDVRASDVIYAINADAVVVQDAISEKLGNLIHYMATFVAGFVVGFT
AAWQLALVTLAVVPLIAVIGGLSAAALAKLSSRSQDALSGASGIAEQALAQIRIVQAFVGEEREMRAYSA
ALAVAQRIGYRSGFAKGLGLGGTYFTVFCCYGLLLWYGGHLVRAQHTNGGLAIATMFSVMIGGLALGQSA
PSMAAFAKARVAAAKIFRIIDHRPGISSRDGAEPESVTGRVEMRGVDFAYPSRPDVPILRGFSLSVPAGK
TIALVGSSGSGKSTVVSLIERFYDPSAGQILLDGHDLRSLELRWLRRQIGLVSQEPALFATSIRENLLLG
RDSQSATLAEMEEAARVANAHSFIIKLPDGYDTQVGERGLQLSGGQKQRIAIARAMLKNPAILLLDEATS
ALDSESEKLVQEALDRFMIGRTTLVIAHRLSTIRKADVVAVLQGGAVSEMGAHDELMAKGENGTYAKLIR
MQEQAHEAALVNARRSSARPSSARNSVSSPIMTRNSSYGRSPYSRRLSDFSTSDFTLSIHDPHHHHRTMA
DKQLAFRAGASSFLRLARMNSPEWAYALAGSIGSMVCGSFSAIFAYILSAVLSVYYAPDPRYMKREIAKY
CYLLIGMSSAALLFNTVQHVFWDTVGENLTKRVREKMFAAVLRNEIAWFDADENASARVAARLALDAQNV
RSAIGDRISVIVQNSALMLVACTAGFVLQWRLALVLLAVFPLVVGATVLQKMFMKGFSGDLEAAHARATQ
IAGEAVANLRTVAAFNAERKITGLFEANLRGPLRRCFWKGQIAGSGYGVAQFLLYASYALGLWYAAWLVK
HGVSDFSRTIRVFMVLMVSANGAAETLTLAPDFIKGGRAMRSVFETIDRKTEVEPDDVDAAPVPERPRGE
VELKHVDFSYPSRPDIQVFRDLSLRARAGKTLALVGPSGCGKSSVLALVQRFYEPTSGRVLLDGKDVRKY
NLRALRRVVAVVPQEPFLFAASIHENIAYGREGATEAEVVEAAAQANAHRFIAALPEGYRTQVGERGVQL
SGGQRQRIAIARALVKQAAIVLLDEATSALDAESERCVQEALERAGSGRTTIVVAHRLATVRGAHTIAVI
DDGKVAEQGSHSHLLKHHPDGCYARMLQLQRLTGAAAGPGPSTSCNGAA
>AtABCC5

MDFIEISLIFREHLPLLELCSVIINLLLFLVFLFAVSARQILVCVRRGRDRLSKDDTVSA
SNLSLEREVNHVSVGFGFNLSLLCCLYVLGVQVLVLVYDGVKVRREVSDWFVLCFPASQS
LAWFVLSFLVLHLKYKSSEKLPFLVRIWWFLAFSICLCTMYVDGRRLAIEGWSRCSSHVV
ANLAVTPALGFLCFLAWRGVSGIQVTRSSSDLQEPLLVEEEAACLKVTPYSTAGLVSLIT
LSWLDPLLSAGSKRPLELKDIPLLAPRDRAKSSYKVLKSNWKRCKSENPSKPPSLARAIM
KSFWKEAACNAVFAGLNTLVSYVGPYLISYFVDYLGGKEIFPHEGYVLAGIFFTSKLIET
VTTRQWYMGVDILGMHVRSALTAMVYRKGLKLSSIAKQNHTSGEIVNYMAVDVQRIGDYS
WYLHDIWMLPMQIVLALAILYKSVGIAAVATLVATIISILVTIPLAKVQEDYQDKLMTAK
DERMRKTSECLRNMRVLKLQAWEDRYRVRLEEMREEEYGWLRKALYSQAFVTFIFWSSPI
FVAAVTFATSIFLGTQLTAGGVLSALATFRILQEPLRNFPDLVSMMAQTKVSLDRISGFL
QEEELQEDATVVIPRGLSNIAIEIKDGVFCWDPFSSRPTLSGIQMKVEKGMRVAVCGTVG
SGKSSFISCILGEIPKISGEVRICGTTGYVSQSAWIQSGNIEENILFGSPMEKTKYKNVI
QACSLKKDIELFSHGDQTIIGERGINLSGGQKQRVQLARALYQDADIYLLDDPFSALDAH
TGSDLFRDYILSALAEKTVVFVTHQVEFLPAADLILVLKEGRIIQSGKYDDLLQAGTDFK
ALVSAHHEAIEAMDIPSPSSEDSDENPIRDSLVLHNPKSDVFENDIETLAKEVQEGGSAS
DLKAIKEKKKKAKRSRKKQLVQEEERVKGKVSMKVYLSYMGAAYKGALIPLIILAQAAFQ
FLQIASNWWMAWANPQTEGDESKVDPTLLLIVYTALAFGSSVFIFVRAALVATFGLAAAQ
KLFLNMLRSVFRAPMSFFDSTPAGRILNRVSIDQSVVDLDIPFRLGGFASTTIQLCGIVA
VMTNVTWQVFLLVVPVAVACFWMQKYYMASSRELVRIVSIQKSPIIHLFGESIAGAATIR
GFGQEKRFIKRNLYLLDCFVRPFFCSIAAIEWLCLRMELLSTLVFAFCMVLLVSFPHGTI
DPSMAGLAVTYGLNLNGRLSRWILSFCKLENKIISIERIYQYSQIVGEAPAIIEDFRPPS
SWPATGTIELVDVKVRYAENLPTVLHGVSCVFPGGKKIGIVGRTGSGKSTLIQALFRLIE
PTAGKITIDNIDISQIGLHDLRSRLGIIPQDPTLFEGTIRANLDPLEEHSDDKIWEALDK
SQLGDVVRGKDLKLDSPVLENGDNWSVGQRQLVSLGRALLKQAKILVLDEATASVDTATD
NLIQKIIRTEFEDCTVCTIAHRIPTVIDSDLVLVLSDGRVAEFDTPARLLEDKSSMFLKL
VTEYSSRSTGIPEL
>VvABCC1
MGDLWTMFCGEPSCLDSGGCSSEFIFFNHPSSCANHALTVCFDILLFVMFLFTMIQRTSS
KPVHVPGQFQRFSPLQISSAIFNGCLGLVYLCLGVWILEENLRKTQIVLPLHWWLLPLLQ
GFTWLLVGLMVSLRGQYLPRSPLRILSILAFLFSGITGVLSIFSAIVYKEASVEIVLNVL
SLPGAILLLLCAYKGYKYEETDKIVNGSGLYTPLNGEADGSAKTDSVGDVTPFAKAGFFS
SMSFWWLNPLMKRGTKKTLENEDIPKLREEDRAESCYLQFLEELIKQKQIEPSSQPSILR
VIILCYWKDIFISGFFALVKILTLSTGPLLLNAFIKVAEGKELFKNEGYVLAMALLVSKN
VESLSQRQWYFRSRLIGLRVRSLLTAAIYKKQLRLSNAAKMIHSSGEITNYVTVDSYRIG
EFPFWFHQTWTTSLQLCIVLVILFNILGLATFAALVVIILTVLCNAPLAKLQHKFQSKLM
VAQDERLRACSEALVNMKVLKLYAWENHFKNVIEKLRNVEYKWLSGVQLRKGYNGFLFWS
SPVLVSAATFGACFFLGIPLNASNVFTFVAALRLVQDPIRSIPDVIGVVIQAKVAFARIV
KFLEAPELQTSNVRQKSNIENISNAISIKSANFSWEEKLSKSTLRDISLEVRTGEKVAIC
GEVGSGKSTLLAAILGEIPDVQGTIRVYGRIAYVSQTAWIQTGSIQENILFGSSMDPERY
QATLEKCSLVKDLDLLPYGDLTEIGERGVNLSGGQKQRIQLARALYQDADIYLLDDPFSA
VDAHTATSLFNEYVMDALSGKTVLLVTHQVDFLPAFDSVLLMSDGEIIQAAPYQQLLVSS
QEFVDLVNAHKETAGSERLAEVTPEKFENSVREINKTYTEKQFKAPSGDQLIKQEEREIG
DMGFKPYMQYLSQNKGYLFFSLAALSHILFVAGQISQNSWMAANVDNPNISTLQLIVVYL
LIGATSTLFLLSRALFVVALGLQSSKSLFTQLLNSLFRAPMSFYDSTPLGRILSRISNDL
SIVDLDVPFSFVFAFGATTNAYSNLGVLAVVTWQVPFVSIPMIYVAIRLQRYYFASAKEL
MRINGTTKSLVANHLAESIAGAMTIRAFEEEERFFVKNMDFIDTNASPFFHSFAANEWLI
QRLEALSAMVLSSSALCMILLPPGTFTAGFIGMAMSYGLSLNMSLVFSIQNQCILANYII
SVERLNQYMHIPSEAPEVIEGSRPPPNWPAVGRVDIHDLQIRYRPDTPLVLRGINCTFEG
GHKIGIVGRTGSGKTTLIGALFRLVEPAGGKIIVDGIDISTIGLHDLRSHFGIIPQDPTL
FNGTVRYNLDPLSQHTDHEIWEVLGKCQLQEAVQEKEEGLGSIVAEGGSNWSMGQRQLFC
LGRALLRRSRILVLDEATASIDNATDLILQKTIRTEFADCTVITVAHRIPTVMDCTMVLA
ISDGKLVEYDEPAKLMKREGSLFGQLVREYWSHFHSAESH
>MdABCF
MTVASSVVHEVLGRRAEDVDEPIIEYIVNVLADEDFDFGEDGEGAFEALGELLVGAGCVS

DFAECRSVCSIITEKFGKHGLVKAKPTVRSLSAPVRMDDGMDEKVAPKKKVEVIDGPLLT
ERDRAKIERKKRKDDRQREQQYQIHLAEMEAVRAGMPVVSVNHESAGGPNVRDIRLENFN
VSVGGRDLIVDGSVTLSFGRHYGLVGRNGTGKTTFLRHLAMHAIDGIPRNFQILHVEQEV
VGDDTTALQCVLNTDVERTKLLEEEARLLTQQRALEFEDSTEKSNGEVDKDAIGQRLQEI
YKRLEFIDADSAESRAASILAGLSFSPEMQLKPTKAFSGGWRMRIALARALFIEPDLLLL
DEPTNHLDLHAVLWLEAYLVKWPKTCIVVSHAREFLNTVVTDILHLHGQKLNAYKGDYDT
YERTRTELVKNQQKAFEANERSRTHMQSFIDKFRYNAKRAALVQSRIKALDRLGHVDEIV
NDPDYKFEFPTPDDRPGPPIISFSDASFGYPGGPVLFRNLNFGIDLDSRIAMVGPNGIGK
STILKLIAGELQPISGTVFRSAKVRIAVFSQHHVDGLDLSSNPLLYMMRCFPGVPEQKLR
SRLGSFGVSGNLALQSMYTLSGGQKSRVAFAKITFKKPHIILLDEPSNHLDLDAVEALIQ
GLVLFQGGILMVSHDEHLISGSVDELWVVSEGRIQPFHGSFEDYKKILQSS
>AtABCG39
LRWAAIERLPTFDRLRKGMLPQTSANGKIELEDIDLTRLEPKDKKHLMEMILSFVEEDNE
KFLRDLRERTDRVGIEVPKIEVRYENISVEGDVRSASRALPTLFNVTLNTLESILGFFHL
LPSKRKKIQILKDISGIVKPSRMTLLLGPPSSGKTTLLQALAGKLDDTLQMSGRITYCGH
EFREFVPQKTCAYISQHDLHFGEMTVREILDFSGRCLGVGSRYQLMSELSRREKEEGIKP
DPKIDAFMKSIAISGQETSLVTDYVLKILGLDICADILAGDVMRRGISGGQKKRLTTGEM
LVGPARALFMDEISTGLDSSTTFQICKFMRQLVHISDVTMIISLLQPAPETFELFDDIIL
LSEGQIVYQGPRDNVLEFFEYFGFQCPERKGVADFLQEVTSKKDQEQYWNKREQPYNYVS
VSDFSSGFSTFHTGQKLTSEFRVPYDKAKTHSAALVTQKYGISNWELFKACFDREWLLMK
RNSFVYVFKTVQITIMSLITMTVYLRTEMHVGTVRDGQKFYGAMFFSLINVMFNGLAELA
FTVMRLPVFYKQRDFLFYPPWAFALPAWLLKIPLSLIESGIWIGLTYYTIGFAPSAARFF
RQLLAYFCVNQMALSLFRFLGAIGRTEVISNSIGTFTLLIVFTLGGFIIAKDDIRPWMTW
AYYMSPMMYGQTAIVMNEFLDERWSSPNYDTRINAKTVGEVLLKSRGFFTEPYWFWICIV
ALLGFSLLFNLFYILALMYLNPLGNSKATVVEEGKDKQKGENRGTEGSVVELNSSSNKGP
KRGMVLPFQPLSLAFNNVNYYVDMPSEMKAQGVEGDRLQLLRDVGGAFRPGILTALVGVS
GAGKTTLMDVLAGRKTGGYIEGSISISGYPKNQTTFARVSGYCEQNDIHSPHVTVYESLI
YSAWLRLSTDIDIKTRELFVEEVMELVELKPLRNSIVGLPGVDGLSTEQRKRLTIAVELV
ANPSIIFMDEPTSGLDARAAAIVMRTVRNTVDTGRTVVCTIHQPSIDIFESFDELLLMKR
GGQVIYAGSLGHHSQKLVEYFEAVEGVPKINDGYNPATWMLDVTTPSMESQMSLDFAQIF
SNSSLYRRNQELIKDLSTPPPGSKDVYFKTKYAQSFSTQTKACFWKQYWSYWRHPQYNAI
RFLMTVVIGVLFGLIFWQIGTKTENEQDLNNFFGAMYAAVLFLGALNAATVQPAIAIERT
VFYREKAAGMYSAIPYAISQVAVEIMYNTIQTGVYTLILYSMIGCNWTMAKFLWFYYYML
TSFIYFTLYGMMLMALTPNYQIAGICMSFFLSLWNLFSGFLIPRPQIPIWWRWYYWATPV
AWTLYGLITSQVGDKDSMVHISGIGDIDLKTLLKEGFGFEHDFLPVVAVVHIAWILLFLF
VFAYGIKFLNFQRR
>CrTPT2
MLLNSSASANMLSRASDEEEALCLAALQRSPTYIRARTSIFRSISGEISLLDIDKLKNQE
QRQVLDKLINSINEDFDKFFKRVRQRFDAVDLEFPKVEVRFKNLNVDAFVHVGSRALPTI
PNFLFNMTEVFLGQLRVFPGKRKKLSILKNINGILRPSRLTLLLGPPSSGKTTLLLALAG
RLDHGLKMSGRVTYNGHEMNEFVPQRTSAYVSQQDWHMAEMTVRETLEFSGRCQGPGYKH
DMLMELLRKEKDAGIYPDEELDLFIKAVALGEQTSIISEYLMKILGLDICSDTLVGDEML
KGISGGQKKRLTTGEFLMGPSRVLLMDEISTGLDSSTTHQIIKYLRHVTHAFEGTTIVSL
LQPDPETYELFDDIILLSDGEIVYQGPRETALDFFAFMGFRCPSRKNIADFLQEVISEKD
QGQYWMLNSQYRYVPVRNFIEGYQSFGAGKLLAKELSVPFDRRYNHPAALSRNTYGVKRT
DLLKISFFWQMLLLKRNSFVFVFKFMQLFLIVAIMTSVFFRTTMHHKTLDDGGVYLGALY
FAILMILFNGFLEVPMLIAKLPVLYKHRDSRFYPCWIYTLPSWLLTIPISLLESIIWVGV
TYYLVGFDPQITRCLRQLLLYFSLHQMSIALFRVMASLGRNLVVANTFGSFAMLVVMALG
GFILSRDSIPSWWIWGYWFSPLMYAQNAASVNEFRGHSWDKKIGNNTGTSLGETLLRVRS
LFPESYWYWIGVGALLGYTVLFNILFTLFLTYLNPLGNTKVYVVSKDQNTEKNIVQENVQ
ENNNPNISLGEFLQHSHSYNGKVIKSKRGMVLPFVPLSMSFRNINYYIDVPLELKQQGLL
EDRLQLLSNVTGAFRPAVLTALVGVSGAGKTTLMDVLAGRKTGGYIEGDICISGYPKKQE
TFARVSGYCEQNDIHSPCLTVNESLLFSAWLRLSSKVDLRTQRVFVDEVMDLVELTPLRG
AVVGLPGVDGLSTEQRKRLTIAVELVANPSIVFMDEPTSGLDARAAAIVMRTVRNIVDTG

RTIVCTIHQPSIDIFESFDELLLMKRGGKLIYAGPLGNKSCKLIEYFEAIEGVPKIRHGY
NPAAWILEVTSPAEEIRLGVDFAEIYHRSNLFQQNKDLVDLLSKPTINSNELSFPSKYSQ
SFFGQFLACLWKQNRSYWRNPQYTAVRLFYTVVISFMFGTICWRFGAKRETQQEIFNAMG
SMYAAVLFIGITNTTSVQPVLYVERFVSYRERAAGMYSALPFAFAQAVIEFPYVFVQSLI
YCTIFYFLASFELNLWKFVWYIYFMYFTLLYFTFFGMMTTAVTPNQNIAAIIGAPFFMMW
NLFSGFMISRLRIPIWWRWYYWANPIAWTLYGLLTSQYGDMDTFVKLADGVHSVPVKQLL
KEQFGYRHDFLPAASIAVIGFCFVFAVTFGFAIKAFNFQRR
>MtABCG10
MEGTDIYRATNSLRARSSTVWRQSGVEVFSKSSREEDDEEALKWAALEKLPTYNRLRKGLLTASHGGAHE
VDVGDLAFQDKQKLLERLVKVAEEDNEGFLLKVKERVDRVGLDIPTIEVRYNNLKIDAEAFVGSRALPSF
INAATNVIEGVLNFLHIIPTKKRHVAILKDVSGIVKPRRMTLLLGPPGSGKTTLLLALSGKLDPSLQLTG
SVTYNGHGLNEFVPQRTAAYISQHDVHIGEMTVRETLAFSARCQGVGSRYDMLSELSRREKAANIKPDPD
IDVYMKAIATEGQEYSISTDYVLKILGLDICADTMVGDEMLRGISGGQRKRVTTGEMLVGPANIVSSLRQ
YVHIMNGTAVISLLQPAPETYDLFDDIILISDGQVVYHGPREYVLDFFETMGFKCPERKGAADFLQEVTS
KKDQAQYWVRRDQPYRFVTVTQFAEAFQSFHIGRKLAEELSVPFDKTKSHPAALTTKEYGLNKTELLKAN
FSREYLLMKRNSFVYIFKLTQLFIMALIAMTLFFRTEMHRNNQDDAGVYAGALFFTLVTMMFNGMSEISM
TIAKLPVYYKQRDLLFYPSWAYAIPSWILKIPISLVEVSLWVFLTYYVIGFDPNVGRMFKQFLVLFFMSQ
MASGLFRAIASLGRNMIVANTFGSFAVLTLLALGGFILSRKDIKGWWIWGYWISPLMYGQNALMANEFLG
NSWHNATFDLGKNYLDTRGFFPHAYWYWIGVGGLVGFVFLFNAAFGVALAVLGPFDKPSATITEEDSEDD
SSTVQEVELPRIESSGRRDSVTESSHGKKKGMVLPFEPHSITFDDIVYSVDMPAEMKEQGVTEDRLVLLK
GVSGAFRPGVLTALMGVSGAGKTTLMDVLAGRKTGGYIDGDIKVSGYPKKQETFARISGYCEQNDIHSPH
VTVYESLLYSAWLRLPSGVDSNTRKMFIDEVMDLVELNSLRNSLVGLPGVSGLSTEQRKRLTIAVELVAN
PSIIFMDEPTSGLDARAAAIVMRTVRNTVDTGRTVVCTIHQPSIDIFEAFDELFLMKRGGQEIYVGPLGR
HSTHLIKYFESIDGVSKIKDGYNPATWMLEVTTTAQELNLGVDFTDLYKNSDLYRRNKQLIQELSVPAPG
SKDLHFPTQFSQSFLVQCQACLWKQRWSYWRNPPYTAVRFFFTTFIGLMFGTMFWDLGGKHSSRQDLLNA
VGSMYTAVLFLGVQNSSSVQPVVAVERTVFYREKAAGMYSALPYAFSQILVELPYVFAQAVTYGAIVYAM
IGFDWTAEKFLWYLFFMYFTLLYFTFYGMMAVAVTPNHHVASIVAAAFYAIWNLFSGFVVPRPSIPIWWR
WYYWACPVAWTIYGLVASQFGDITTVMSTEGGKDVKTFLDDFFGIQHDFIGWCALVVGGIAVGFAFIFAV
AIKSFNFQKR
>NbPDR1a
MEPANLSNLRGSSLRGSMRGSLRANSNSIWRNNGVEIFSRSSRDEDDEEALKWAALEKLP
TFDRLRKGLLFGSQGAAAEVDINDLGFQERKNLLERLVKVADEDNEKFLLKLKNRIDRVG
IDLPTIEVRYEHLNIDADAYVGSRGLPTFMNFMTNFVETLLNSLHILPSRKRQLTILKDI
SGIIKPCRMTLLLGPPSSGKTTLLLALAGKLDPALKVTGKVSYNGHELHEFVPQRTAAYI
SQHDLHIGEMTVRETLEFSARCQGVGSRFEMLAELSRREKAANIKPDADIDVYMKAAATE
GQEANVVTDYVLKILGLDICADTMVGDDMIRGISGGQKKRVTTGEMLVGPSKALFMDEIS
TGLDSSTTYSIVNSLRQSVQILKGTAVISLLQPAPETYNLFDDIILLSDGYIVYQGPRDD
VLEFFESMGFKCPHRKGVADFLQEVTSKKDQQQYWSKRNEPYRFITSKEFAEAYQSFHVG
RKLGDELATPFDKTKCHPAALTNEKYGIGKKELLKVCTERELLLMKRNSFVYMFKFSQLT
IMALITMTLFFRTEMPRDTTDDGAIYAGALFFVVIMIMFNGMSELAMTIFKLPVFYKQRD
LLFFPSWAYAIPSWILKIPVTLVEVGLWVILTYYVIGFDPNITRFLKQFLLLIVVNQMAS
GLFRFIGAVGRTMGVASTFGSFALLLQFALGGFVLSRDDVKSWWIWGYWTSPMMYSVNSI
LVNEFDGKKWNHIVPGGNETLGATVVKSRGFFPEAYWYWIGVGALVGFTVVFNFCYSVAL
AYLNPFDKPQAVLAEDGENAENVEVSSQITSTDGGDSISESQNNKKGMVLPFEPHSITFD
DVVYSVDMPQEMKEQGAGQDRLVLLKGVSGAFRPGVLTALMGVSGAGKTTLMDVLAGRKT
GGYIDGEIKISGYPKKQETFARISGYCEQNDIHSPYVTVYESLVYSAWLRLPQDVDEKTR
KMFVDEVMELVELGPLRSALVGLPGVNGLSTEQRKRLTIAVELVANPSIIFMDEPTSGLD
ARAAAIVMRTVRNTVDTGRTVVCTIHQPSIDIFEAFDELFLMKRGGQEIYVGPLGRHSCH
LIKYFESNPGVAKIKEGYNPATWMLEVTASAQEMLLGVDFTEVYKNSDLYRSNKALISEL
GVPRPGSKDLHFETQYSQSFWTQCMACLWKQHWSYWRNPAYTAVRFIFTTFIALIFGTMF
WDLGTKVSESQDLLNAMGSMYAAVLFLGVQNASSVQPVVAVERTVFYRERAAGMYSAIPY
AFGQVAIEIPYIFVQSVFYGIIVYAMIGFEWDVGKFFWYLFIMFFTLLYFTFYGMMSVAV
TPNQNVASIVAAFFYGVWNLFSGFIIPRPRMPVWWRWYYWANPVAWTLYGLVASQFGDIQ
TKLSDNETVKQFLRRFFGFRHDFLGVVAAVLSAYVFVFAFTFAFAIKAFNFQRR

>NbPDR1b
MEPANLSNLRGSSLRGSTRGSLRANSNTIWRNNGVEIFSRSSRDEDDEEALKWAALEKLP
TFDRLRKGLLFGSQGAAAEVDIYDLGFQERKNLLERLVKVADEDNEKFLLKLKNRIDRVG
IDLPTIEVRYEHLNIDADAYVGSRGLPTFMNFMTNFVETLLNSLHILPSRKRQLTILKDI
SGIIKPCRMTLLLGPPSSGKTTLLLALAGKLDPALKVTGKVSYNGHELHEFVPQRTAAYI
SQHDLHIGEMTVRETLEFSARCQGVGSRFEMLAELSRREKAANIKPDADIDIYMKAAATE
GQEANVVTDYVLKILGLDICADTMVGDDMIRGISGGQKKRVTTGEMLVGPSKALFMDEIS
TGLDSSTTYSIVNSLRQSVQILKGTAVISLLQPAPETYNLFDDIILLSDGYIVYQGPRDD
VLEFFDSMGFKCPERKGVADFLQEVTSKKDQQQYWSKRNKPYRFITSKEFAEAYQSFHVG
RKLGDELATPFDKTKCHPAALTNEKYGIGKKELLKVCTERELLLMKRNSFVYVFKFSQLT
IMALITMTLFFRTEMPRDTTDDGAIYAGALFFVVIMIMFNGMSELAMTIFKLPVFYKQRD
LLFFPSWAYAIPSWILKIPVTLVEVGLWVILTYYEIGFDPNITRLLKQFLLLIVVNQMAS
GLFRFIGAVGRTMGVASTFGSFALLLQFALGGFVLSRDDVKSWWIWGYWTSPMMYSVNSI
LVNEFDGKKWNHIVPGGNETLGTTVVKSRGFFPEAYWYWIGVGALVGFTVVFNFCYSLAL
AYLNPFDKPQAVLPEDGENAENVEVSSQITSTDGGDSISEGQNNNKKGMVLPFEPHSITF
DDVVYSVDMPQEMKEQGADQDRLVLLKGVSGAFRPGVLTALMGVSGAGKTTLMDVLAGRK
TGGYIDGDIKISGYPKKQETFARISGYCEQNDIHSPYVTVYESLVYSAWLRLPQDVDEKT
RKMFVDEVMELVELGPLRSALVGLPGVNGLSTEQRKRLTIAVELVANPSIIFMDEPTSGL
DARAAAIVMRTVRNTVDTGRTVVCTIHQPSIDIFEAFDELFLMKRGGQEIYVGPLGRHSC
HLIKYFESNPGVAKIKEGYNPATWMLEVTASAQEMMLGVDFTEVYKNSDLYRRNKALISE
LGVPRPGSKDLHFETQYSQSFWTQCMACLWKQHWSYWRNPAYTAVRFIFTTFIALIFGTM
FWDLGTNVSKSQDLLNAMGSMYAAVLFLGVQNASSVQPVVAVERTVFYRERAAGMYSAIP
YAFGQVSIEIPYIFVQSVFYGIIVYAMIGFEWDVGKFFWYLFIMFFTLLYFTFYGMMSVA
VTPNQNVASIVAAFFYGVWNLFSGFIIPRPRMPVWWRWYYWANPVAWTLYGLVASQFGDI
QTKLSDNETVEQFLRRYFGFKHDFLGIVAAVLTAYVFVFAFTFAFAIKAFNFQRR
>NbPDR2a
MEPADLSNLRGRSLRASIRGSMRGSIRENSNSIWRNNGAEVFSHSARDEDDEEALKWAAL
EKLPTYDRLRKGILFGSQGAAAEVDVDDLGVLERKNLLERLVKVADEDNEKFLLKLKNRI
DRVGIDFPSIEVRFEHLNIDADAYVGSRALPTFTNFISNFVEGLLDSIHILPSKKRQVTI
LKDVSGIVKPCRMTLLLGPPGSGKTTLLLALAGKLDSALKVTGKVTYNGHELHEFVPQRT
ATYISQHDLHIGEMTVRETLEFSARCQGVGSRYEMLAELSRREKAANIKPDADIDMFMKA
ASTEGQEAKVVTDYILKILGLDICADTMVGDQMIRGISGGQKKRVTTGEMIVGPSKALFM
DEISTGLDSSTTYSIVNSLKQSVRIMKGTALISLLQPAPETYNLFDDIILLSDGYIVYEG
PRDYVLEFFESMGFKCPERKGAADFLQEVTSKKDQQQYWVRRDEPYRFITSKEFAEAYQS
FHVGRKVRDELATTFDKSKSHPAALTTQKYGIGKRQLLKVCTERELLLMQRNSFVYLFKF
FQLLIIALMTMTIFFRTKMPRDTAEDGGIYSGALFFVVIMIMFNGLSELPMTLYKLPVFY
KQRDFLFYPSWAYAIPSWILKIPVTFAEVGMWVFLTYYVMGFDPNVGRFFKQFLLLLLVN
QMASALFRFIAAVGRTMGVASTFGAFALLLQFALGGFILARKDVKDWWIWGYWTSPLMYS
VNAILVNEFDGQKWKHIVAGGTEPLGAAVVRARGFFPDAYWYWIGVGALAGFTVMFNIAY
SVALAYLNPFDKPQATISDESENNESELSPQITSTQEGDSVSENKKKGMVLPFDPHSITF
DEVVYSVDMPPEMRESGTSDNRLVLLKSVSGAFRPGVLTALMGVSGAGKTTLMDVLAGRK
TGGYIDGSIKISGYPKKQETFARISGYCEQNDIHSPYVTVFESLVYSAWLRLPQDVNEEK
RMMFVEEVMDLVELTPLRSALVGLPGVNGLSTEQRKRLTIAVELVANPSIIFMDEPTSGL
DARAAAIVMRAVRNTVDTGRTVVCTIHQPSIDIFEAFDELFLMKRGGQEIYVGPLGRQSC
HLIKYFESIPGVSKIVEGYNPATWMLEVTASSQEMALGVDFTDLYKKSDLYRRNKALIDE
LSVPRPGTSDLHFDSEFSQPFWTQCMACLWKQHWSYWRNPAYTAVRFIFTTFIALIFGTM
FWDIGTKVSRNQDLINAMGSMYAAVLFLGVQNSSSVQPVVSVERTVFYREKAAGMYSAIP
YAFAQVLIEIPYIFVQATVYGLIVYSMIGFEWTAAKFFWYFFFMFFTFLYFTFFGMMTVA
VTPNQNVASIVAGFFYTVWNLFSGFIVPRPRIPIWWRWYYWGCPIAWTLYGLVASQFGDL
QDPLTDQNQTVEQFLRSNFGFKHDFLGVVAAVIVAFAVVFAFTFALGIKAFNFQRR
>NbPDR2b
MEPADLSNLRGRSLRASMRGSIRENSISIWRNNGADVFSRSARDEDDEEALKWAALERLP
TYDRLRKGILFGSQGAAAEVDVDDLGVLERKNLLERLVKVADEDNEKFLLKLKNRIDRVG
IDFPSIEVRFEHLNIDADAYVGSRALPTFTNFISNFIEGLLDSIHILPSRKRQVTILKDV

SGIVKPCRMSLLLGPPGSGKTTLLLALAGKLDSALKVTGKVTYNGHELHEFVPQRTAAYI
SQHDLHIGEMTVRETLEFSARCQGVGSRYEMLAELSRREKAANIKPDADIDMFMKAASTE
GQEAKVVTDYILKILGLDICADTMVGDQMIRGISGGQKKRVTTGEMIVGPSKALFMDEIS
TGLDSSTTYSIVNSLKQSVRIMKGTALISLLQPAPETYNLFDDIILLSDGYIVYQGPRED
VLNFFESMGFKCPERKGAADFLQEVTSKKDQQQYWIRRDEPYRFITSKEFAEAYQSSHVG
RKVSDELATTFDKSKSHPAALTTQKYGIRKKQLLKVCTERELLLMQRNSFVYLFKFFQLL
VIALMTMTIFFRTKMPRDTAADGNIYSGALFFVVIMIMFNGLSELPMTLYKLPVFYKQRD
FLFYPSWAYAIPSWILKIPVTFAEVGMWVFLTYYVIGFDPNVGRFFKQFLLLLLVNQMAS
ALFRFIAAVGRTMGVASTFGAFALLLQFALGGFILARNDVKDWWIWGYWTSPLMYSVNAI
LVNEFDGKKWKHIVAGGTEPLGAAVVRARGFFPDAYWYWIGIGALAGFTVMFNIAYSVAL
AYLNPFDKQQATISDESENNENTESSPQITSTKEGDSVNENKKKGMVLPFDPHSITFDEV
VYSVDMPPEMRESGTSENRLVLLKSVSGAFRPGVLTALMGVSGAGKTTLMDVLAGRKTGG
YIDGSIKISGYPKKQETFARISGYCEQNDIHSPYVTVFESLVYSAWLRLPQDVDEQKRMM
FVEEVMDLVELTSLRSALVGLPGVNGLSTEQRKRLTIAVELVANPSIIFMDEPTSGLDAR
AAAIVMRAVRNTVDTGRTVVCTIHQPSIDIFEAFDELFLMKRGGQEIYVGPLGRQSCHLI
KYFESIPGVSKIVEGYNPATWMLEVTASSQEMALGVDFTELYKKSDLYRRNKALIDELSM
PQPGTSDLHFDSEFSQPFWTQCMACLWKQHWSYWRNPAYTAVRFIFTTFIALIFGTMFWD
IGTKVSRNQDLINAMGSMYAAVLFLGVQNSSSVQPVVSVERTVFYREKAAGMYSAIPYAF
AQVLIEIPYIFVQAVVYGLIVYSMIGFEWTVAKFFWYFFFMFFTFLYFTFFGMMTVAVTP
NQNVASIVAGFFYTVWNLFSGFIVPRPRIPIWWRWYYWGCPIAWTLYGLVASQFGELQDP
LTDQNETVEQFLRSNFGFKHDFLGVVAAVIVAFAVVFAFTFALGIKAFNFQRR
>NtPDR3
MAQLVGSDEIESFRMDLAEIGRSLRSSFRGQSSSFRSNSALSASQKDDAVDEENMLAWAAIERLPTFDRL
RSSLFEEINGNDANVKRKRVTDVTKLGALERHVFIEKMIKHIEHDNLQLLHKIRKRIDKVGVELPTVEVR
YKNLTIEAECELVHGKPLPTLWNSLKSITMNLARLPGLQSELAKIKILNDVSGVIKPGRMTLLLGPPGCG
KTSLLKALSGNLDKSLKVSGEISYNGYKLEEFVPQKTSAYVSQNDLHIPEMTVRETLDYSSRFQGVGSRA
EIMTDLSRREKEAGVVPDPDIDTYMKAISIEGQKKNLQTDYILKILGLDICADTLVGDAMRRGISGGQKK
RLTTGELIVGPIKALFMDEISNGLDSSTTYQIVACLQQLAHITDATILVSLLQPAPETFDLFDDIILMAE
GKILYHGPRNSALEFFESCGFKCPERKGVADFLQEVTSKKDQAQYWHGTKETYKFVSVDMLSRKFKESPY
RKKLNEELSVPYDNSRSHRNSITFRDYSLPKWELFRACMSREFLLMKRNSFIYIFKTVQLAIIASITMTV
FLRTRMDTDLVHANYYLGALFYALIILLVDGFPELSMTITRLAVFYKQSELCFYPAWAYTIPATILKIPL
SLLESVIWASMTYYVIGFSPEAGRFFRQLLLLFAVHMTSISMFRFLASVCRTIVASTAAGGLSILFVLCF
SGFIIPRPSMPIWLKWGFWISPLTYGEIGLAVNEFLAPRWQKTLPTNTSIGNEVLESRGLNFDGYFYWIS
VCALFGFTILFNIGFTLALTFLKAPGSRAIISTDKYSQIEGSSDSIDKADAAENSKATMDSHERAGRMVL
PFEPLSLVFQDVQYYVDTPAAMTELGFTQKRLQLLSDITGALRPGILTALMGVSGAGKTTLLDVLAGRKT
TGYVEGEIKVGGYPKVQETFARVSGYCEQTDIHSPQITVEESVIFSAWLRLHPQIDSKTKYEFVKEVIET
IELDGIKGMLVGMPGVSGLSTEQRKRLTIAVELVANPSIIFMDEPTTGLDARSAAIVMRAVKNVADTGRT
IVCTIHQPSIDIFEAFDELILLKTGGRMIYWGHLGRNSCKMIEYFEGISCVPKIKNNHNPATWMLEVTST
SSEADISIDFAEVYKNSALHKNNEELVKKLSFPPAGSKDLHFPTRFSQNGWGQFKTCFWKQYWSYWRSPS
YNLMRSLHMLFASLVSGLLFWDKGKKLDNQQSVFSVFGAMFTAVIFCGINNSSSVLPYVTTERSVLYRER
FAGMYASWAYALAQVAIEIPYLLAQALAFTVITYPMIGYYWSAYKVFWYFYSMFCTLLYFTYLGMMLVSM
TPSFPVAAILQSSFYTMFNLFAGFLMPKAQIPKWWIWFYYLTPTSWTLNGMLTSQYGDIEKEITVFQEKK
TVAAFLGDYFGFHHNQLPIVAFVLIAYPLVFASLFAFFIGKLNFQRR
>NpPDR1
MEPADLSNLRGRSLRASIRGSMRGSIRENSNSIWRNNGAEVFSRSARDEDDEEALKWAAL
EKLPTYDRLRKGILFGSQGAAAEVDVDDSGVLERKNLLERLVKVADEDNEKFLLKLKNRI
DRVGIDFPSIEVRFEHLNIDADAYVGSRALPTFTNFISNFVEGLLDSIHILPSKKRQVTI
LKDVSGIVKPCRMTLLLGPPGSGKTTLLLALAGKLDSALKVTGKVTYNGHELHEFVPQRT
AAYISQHDLHIGEMTVRETLEFSARCQGVGSRYEMLAELSRREKAANIKPDADIDMFMKA
ASTEGQEAKVVTDYILKILGLDICADTMVGDQMIRGISGGQKKRVTTGEMIVGPSKALFM
DEISTGLDSSTTYSIVNSLKQSVRIMKGTALISLLQPAPETYNLFDDIILLSDGYIVYEG
PREEVLEFFESMGFKCPERKGAADFLQEVTSKKDQQQYWIRRDEPYRFITSKEFAEAYQS
FHVGRKVSDELKTTFDKSKSHPAALTTQKYGIGKRQLLKVCTERELLLMQRNSFVYLFKF
FQLLIIALMTMTIFFRTKMPRDSAEDGGIYSGALFFVVIMIMFNGLSELPMTLYKLPVFY

KQRDFLFYPSWAYAIPSWILKIPVTFAEVGMWVFLTYYVMGFDPNVGRFFKQFLLLLLVN
QMASALFRFIAAVGRTMGVASTFGAFALLLQFALGGFILARNDVKDWWIWGYWTSPLMYS
VNAILVNEFDGQKWKHIVAGGTEPLGAAVVRARGFFPDAYWYWIGVGALAGFIVMFNIAY
SVALAYLNPFDKPQATISDESENNESESSPQITSTQEGDSASENKKKGMVLPFDPHSITF
DEVVYSVDMPPEMRESGTSDNRLVLLKSVSGAFRPGVLTALMGVSGAGKTTLMDVLAGRK
TGGYIDGSIKISGYPKKQDTFARISGYCEQNDIHSPYVTVFESLVYSAWLRLPQDVNEEK
RMMFVEEVMDLVELTPLRSALVGLPGVNGLSTEQRKRLTIAVELVANPSIIFMDEPTSGL
DARAAAIVMRAVRNTVDTGRTVVCTIHQPSIDIFEAFDELFLMKRGGQEIYVGPLGRQSC
HLIKYFESIPGVSKIVEGYNPATWMLEVTASSQEMALGVDFTDLYKKSDLYRRNKALIDE
LSVPRPGTSDLHFDSEFSQPFWTQCMACLWKQHWSYWRNPAYTAVRLIFTTFIALIFGTM
FWDIGTKVSRNQDLVNAMGSMYAAVLFLGVQNSSSVQPVVSVERTVFYREKAAGMYSAIP
YAFAQVLIEIPYIFVQATVYGLIVYSMIGFEWTVAKFFWDFFFMFFTFLYFTFFGMMTVA
VTPNQNVASIVAGFFYTVWNLFSGFIVPRPRIPIWWRWYYWGCPIAWTLYGLVASQFGDL
QDPLTDQNQTVEQFLRSNFGFKHDFLGVVAAVIVAFAVVFAFTFALGIKAFNFQRR
>PhABCG1
MTNQLPNKVELAEVVPQNGGVFLTWEDLWVTASSVKDGSKAILKGLTGYAMPGELLAIMGPSGSGKSTLL
DTIAGRLGSSTRQSGDILINGRRQTLAYGSSAYVTQDDTLLATLTIKEAVYYSAELQLPNSMSKSEKKEI
ADVTLKGMGLQDAMETRIGGWSGKGISGGQKRRVSICLEILTRPKLLFLDEPTSGLDSAASYYVMKAIAS
QCQGRTIIASIHQPSVDVFSLFHSLCLLSSGRTVYFGPASAANEFFALSGFPCPTLQNPSDHFLKTINSD
FDQDIEEGSTRRKSTEEVIDILIKSYKASDKYNAVQSQVAEICQQEGEMLDKRSHASFITQSLVLTRRSF
INMSRDLGYYWLRLAVYVVIAVGLGSLYYDVGFSAASVQARGSMLMFVASFITFMAIGGFPSFVEDMKVF
QREKLNGHYGSGSFVIANTLSAMPYLLLVSLIPGAIAYFMTGLQNGFEHFIYFALVLFTCMMIVESLMMI
VASMVPNFLMGLIAGAGIQALMLLSGGFFRLPNDLPKPFWKYPLHYVAFHKYAYEGMFKNEFEGLKIHDV
NGEDILRNTWQMNMDYSKWIDLVILLGMLVLYRVLFLLVVKAGEIVKPAIRAFMSHSPNQINSAERPLDV
FDS
>VmABCG1
MIQNFFFKRVRQRFDVVDLEFPKVEVRFQNLSVDAFVHVGSRALPTIPNFIFNMTEVFLRQLRIFPGRRK
KFSILKNINGILRPSRLTLLLGPPSSGKTTLLLALAGRLDHRLKMSGRVTYNGHEMNEFVPQRTSAYISQ
QDWHMGEMTVRETLEFSGNCQGLGYKHDMLMELLRREKSAGILPDEELDLFIKAVALGEQTSLISEYLMK
ILGLDICSDTLVGDEMIKGISGGQKKRLTTGEFLMGATRVLLMDEISTGLDSSTTHQIIKYLRHLTHAFE
GTTIVSLLQPDPETYELFDDIILLSEGQIVYQGPREAALDFFAFMGFRCPSRKNIADFLQEVISEKDQGQ
YWFLNSHYRYVSVQKFIEGYKAFGPGKSLVQELSVPFDRRYNHPAALSRNTYGVKRTELLKISLFWQILL
LKRNSFVFVFKVLQLFLIISIMTSVFFRTTMHHNTLEDGGVYLGALYFSILMILFNGFLEVPMLIAKLPV
LYKHRDSRFYPCWIYTLPSWLLSVPLSLIESSIWVGVTYYLVGFDPQINRCLRQLLLYFCLHQMSIALFR
VMASLGRNLVVANTFGSFAMLVVMALGGFILSRDSIPSWWIWGYWFSPLMYAQNAASVNEFLGHSWDKKA
GNNTDATLGVTLLKARSLFPESYWYWIGIGALLGYTVLFNVLFTLFLTYLNPLGNAQVVVSKEKRIEKIT
VQENVQENTNADISLGEFLQHSHSFNGKVVKNKRGMVLPFEQLSMSFRNISYFIDVPLELKQQGVVEDRL
QLLVNVTGAFRPGVLTALVGVSGAGKTTLMDVLAGRKTGGYIEGDIYISGYPKKQETFARVSGYCEQNDI
HSPCLTVHESLIFSAWLRLSSKVDLKTKRAFVVEVMELVELTPLRGALVGLPGVDGLSTEQRKRLTIAVE
LVANPSIVFMDEPTSGLDARAAAIVMRTVRNIVDTGRTIVCTIHQPSIDIFESFDELLLMKRGGKLIYAG
PLGNKSCKLVEYFEAIEGVPKIRSGYNPAAWILEVTSPAEESRLDVDFAEVYHTSNLFEQNKDLVDLLSM
PKNDYNKLSFPSKYSQSFFGQFLACLWKQNRSYWRNPQYTAVRFFYTVVISLMFGTICWRFGAKRETQQE
IFNAMGSMYAAVLFIGITNTTSVQPVLYVERFVSYRERAAGMYSALPFAFAQVAIEFPYVFVQSLIYSTI
FYFMASFELNFWKLVWYIYFMYFTLLYFTFFGMMTTAVTPNHNIAAIIGAPFFMMWNLFSGFMISRLRIP
IWWRWYYWANPIAWTLYGLLTSQYGDINAFVKLADGVHSMPIKQLLKEQFGYRHEFLATASIVVVGFCCV
FAVAFAFAIKAFNFQRR
>LjABCG1
MEGGDIYRAGHSLRANSTSVWRNSTMEAFSRSSRHEEDDEEALKWAALEKLPTYNRLRKG
LLATSRGAANQIDVSDLGFQERQKLLDKLIKVAEEDNEKFLLKLKERIDRVGIDIPTIEV
RYEHLNVEAEAYVGSRALPSFVNFATNIVESLFTSLHILSTKKRHVTILKDVSGIIKPRR
MTLLLGPPGSGKTTLLLAMSGKLDPNLKVSGRVTYNGHELNEFVPQRTAAYISQHDVHIG
EMTVRETLAFSARCQGVGSRYDLLAELSRREKEANIKPDPYLDVFMKAATTGGQEANIVT
DYVLKILGLDICADTMVGDEMLRGISGGQRKRVTTGEMLVGPANALFMDEISTGLDSSTT
FQIVSSLRQYVHILNGTAVISLLQPAPETFELFDDIILISDGQIVYQGPREFVLDFFETL

GFKCPERKGAADFLQEVTSKKDQEQYWVRRDEPYRFVTVTQFAEAFQAFHVGRKSGDELG
IPFDKSKSHPAALVKKQYGINKKELLKANFSREYLLMKRNSFVYIFKICQLTFMAIMTMT
LFLRTEMHRDSLDDGSVFSGALFFALGTIMFNGMAEISMSIAKLPIFYKQRDLLFYPSWA
YAIPNWILKIPVTFVEVAVWVFLTYYVIGFDPNALRFVKQYALLLLVNQMASGLFRAIAA
LGRNMIVANTFGSFALLMLITLGGFILSRKDIKGWWIWGYWTSPLMYGQNAIMTNEFLGN
SWSHFTKNSNKSLGLQALESRGFFTHAYWYWIGIGALTGFMFLYNIIYTLALTFLNPFDK
AQATINEESEDNTPNGRAPEVELPRIESSGNADSAVDSSHGRKRGMVLPFEPHSIAFDDV
VYSVDMPQEMRDQGVMEDRLVLLKGVSGAFRPGVLTALMGVSGAGKTTLMDVLAGRKTGG
HIDGSVKVSGYPKNQETFARISGYCEQNDIHSPQVTVYESLLYSAWLRLPAEVDSNTRKT
FIEEVIELVELNPLRNSLVGLPGVSGLSTEQRKRLTIAVELVANPSIIFMDEPTSGLDAR
AAAIVMRTVRNTVDTGRTVVCTIHQPSIDIFEAFDELFLMKRGGQEIYVGPLGRHSSKLI
EYFESIEGVNKIKDGYNPATWMLEVTSSAQEVTIGVDFHQTYKNSELYRRNKQLIAELGI
PAPGSNDLYFPTQYSQSFLVQCLACLWKQHWSYWRNPPYTAVRFFFTTFIALIFGTMFWD
LGGKYKNRQDLFNALGSMYTAVLFLGIQNSASVQPVVAVERTVFYRERAAGMYSALPYAL
AQVIIEIPYVFAQALSYGLIVYAMMGFEWTVEKFFWYLFFMFFTLCYFTYYGMMTVAVTP
NHHVASIVAAAFYAIWNLFSGFVVPRPRIPVWWRWYYWACPVAWTIYGMVASQFGDIEHI
LESDDVSVKEFIRSYFGMKHDFIGVCAVVVVGFAVGFAFIFAVSIKVFNFQRR
>GmPDR12
MEGGGSSFRIGSSSIWRNSDAAEIFSNSFHQENDEEALKWAAIQKLPTVARLRKALITSP
DGESNEIDVKKLGLQEKKALLERLVKTAQEDNEKFLLKLKDRIDRVGIDLPTIEVRFENL
SIEAEARAGTRALPTFTNFIVNILEGLLNSLHVLPNRKQHLNILEDVSGIIKPGRMTLLL
GPPSSGKTTLLLALAGKLDPKNKVLWKGTYNGHGVNEFVPQRTAAYVNQNDLHVAELTVR
ETLVFSARVQGVGPRYDLLAELSRREKEANIKPDPDIDAYMKAVASEGQKANMITDYILR
ILGLEVCADTVVGNAMLRGISGGQRKRVTTGEMLVGPAKALFMDEISTGLDSSTTFQIVN
SLKQYVHILKGTTVISLLQPAPETYNLFDDIILLSDSHIVYQGPREHVLEFFELMGFKCP
QRKGVADFCKKLHQGKIRSSTGHTKDHLYRFFTAKEFSEAHKSFHIGRSLVEELATEFDK
SKSHPAALTTKMYGVGKWELLKACLSREYLLMKRNSFVYTFKLCQLAVLAIIAMTIFLRT
EMHRDSVTHGGIYVGALFYGVVVIMFNGLAELSMVVSRLPVFYKQRDYLFFPSWVYALPA
WILKIPLTFVEVGVWVFLTYYAIGFDPYVGRLFRQYLVLVLELVNQMASALFRLVAAVGR
EMTVALTLGSFTLAILFAMSGFVLSKENIKKWWLWGFWISPMMYGQNAMVNNEFLGKRWR
HFLPNSTEALGVEILKSRGFFTQSYWYWIGVGALIGYTLLFNFGYILALTYLNPLGKHQA
VISEEPQINDQSGDSKKGTNVLKNIQRSFSQHSNRVRNGKSLSGSTSPETNHNRTRGMIL
PSETHSITFDDVTYSVDMPVEMRNRGVVEDKLALLKGVSGAFRPGVLTALMGVTGAGKTT
LMDVLAGRKTGGYIGGNITISGYPKKQETFARISGYCEQNDIHSPHVTVYESLLYSAWLR
LSPEINADTRKMFIEEVMELVELKALRNALVGLPGINGLSTEQRKRLTIAVELVANPSII
FMDEPTSGLDARAAAIVMRTVRDTVDTGRTVVCTIHQPSIDIFESFDELLLMKQGGQEIY
VGPLGHHSSHLINYFEGIQGVNKIKDGYNPATWMLEVSTSAKEMELGIDFAEVYKNSELY
RRNKALIKELSTPAPGSKDLYFPSQYSTSFLTQCMACLWKQHWSYWRNPLYTAIRFLYST
AVAAVLGSMFWDLGSKIDKQQDLFNAMGSMYAAVLLIGIKNANAVQPVVAVERTVFYREK
AAGMYSALPYAFAQVLIELPYVLVQAVVYGIIIYAMIGFEWTVTKVFWYQFFMYFTFLTF
TYYGMMSVAVTPNQHISSIVSSAFYAVWNLFSGFIVPRPRIPVWWRWYSWANPVAWSLYG
LVASQYGDIKQSMESSDGRTTVEGFVRSYFGFKHDFLGVVAAVIVAFPVVFALVFAISVK
MFNFQRR
>SpTUR2
MEIAGYRGGSLRGSLQGSLRRSVSAWRSPSTSDVFGRSSREEDDEEALKWAALEKLPTYD
RLRKGIMTGDGGEIQEVDIQGLGFQERKNLLEKLVRNAEEDNERFLLKLRNRMERVGIDN
PTIEVRFEHLNINAEAFVGNRGVPTLVNFFVNKAIWILSALHLMPSGKRPISILHDVSGI
IKPCRMTLLLGPPGAGKTTLLLALAGKLDNTLKVTGNVTYNGHGMHEFVPQRTSAYISQH
DVHIGEMTVRETLAFSSRCQGVGTRYEMLTELSRREKEANIKPDPDVDVYMKAVAVEGQE
SVVTDYILKILGLDICADTMVGDGMIRGISGGQKKRVTTGEMLVGPSKALFMDEISTGLD
SSTTFQIVNSLRQSVHILGGTALIALLQPAPETYDLFDDILLLSDGQIVYQGPRENVLEF
FESMGFKCPERKGVADFLQEVTSRKDQQQYWVRENEPYRFVPVNEFSEAFKSFHVGAKLH
EELSTPFDRSRNHPAALTTSKYGISKMELLKACIDREWLLMKRNSFVYIFKVVQLIVLAL
IAMTVFFRTKLPRNGLEDATIFFGAMFLGLVTHLFNGFAELAMSIAKLPVFYKQRDLLFY

PPWAYALPTWILKIPISFVECGVWIAMTYYVIGFDPNVVRMFRHYLLLVLISQVASGLFR
LLAAVGRDMVVADTFGAFAQLVLLVLGGFIIAREKIKKFWIWGYWSSPLMYAQNAIAVNE
FLGHSWNKLVDATGQTLGERFLRNRGIFVDKNWYWIGVGALIGYMVLFNFLFILFLEWLD
PLGKGQTTVSEEALQEKEANRTGANVELATRGSAATSDGGSVEIRKDGNRKKGMVLPFTP
LSITFDNVKYSVDMPQEMKDRGVTEDKLLLLKGVSGAFRPGVLTALMGVSGRGKTTLMDV
LAGRKTGGYIEGDIRISGYPKNQETFARISGYCEQNDIHSPHVTVYESLLYSAWLRLPAE
VDEKQRKMFVDEVMDLVELNSLRGSLVGLPGVTGLSTEQRKRLTIAVELVANPSIIFMDE
PTSGLDARAAAIVMRAVRNTVDTGRTVVCTIHQPSIDIFEAFDELFLMKRGGEEIYVGPL
GRQSSHLIKYFESIDGVKKIKERYNPATWMLEVTTISQEEILGLNFAEVYRNSDLYKRNK
DLIKELSTPPPGSKDLFFATQFSQSFVMQCLACLWKQHKSYWRNPSYTATRLFFTVVIAL
IFGTIFWDLGKKRSTSLDLINAMGSMYAAVLFIGIQNAQTVQPIVDVERTVFYREKAAGM
YSALPYAYAQVLIEVPHILVQTLLYGLLVYSMIGFDWTAAKFLWYMFFMFFTFLYFTYYG
MMAVAMTPNSDIAAIVAAAFYAIWNIFAGFIIPRPRIPIWWRWYYWACPVAWTLYGLVVS
QFGEYTDTMSDVDETVKDFLRRFLGFRHDFLPVVGVMVVVFTVLFASIFAFSIKTLNFQR
R
>AtABCG21
MMPPNEQESSFPKTPSANRHETSPVQENRFSSPSHVNPCLDDDNDHDGPSHQSRQSSVLR
QSLRPIILKFEELTYSIKSQTGKGSYWFGSQEPKPNRLVLKCVSGIVKPGELLAMLGPSG
SGKTTLVTALAGRLQGKLSGTVSYNGEPFTSSVKRKTGFVTQDDVLYPHLTVMETLTYTA
LLRLPKELTRKEKLEQVEMVVSDLGLTRCCNSVIGGGLIRGISGGERKRVSIGQEMLVNP
SLLLLDEPTSGLDSTTAARIVATLRSLARGGRTVVTTIHQPSSRLYRMFDKVLVLSEGCP
IYSGDSGRVMEYFGSIGYQPGSSFVNPADFVLDLANGITSDTKQYDQIETNGRLDRLEEQ
NSVKQSLISSYKKNLYPPLKEEVSRTFPQDQTNARLRKKAITNRWPTSWWMQFSVLLKRG
LKERSHESFSGLRIFMVMSVSLLSGLLWWHSRVAHLQDQVGLLFFFSIFWGFFPLFNAIF
TFPQERPMLIKERSSGIYRLSSYYIARTVGDLPMELILPTIFVTITYWMGGLKPSLTTFI
MTLMIVLYNVLVAQGVGLALGAILMDAKKAATLSSVLMLVFLLAGGYYIQHIPGFIAWLK
YVSFSHYCYKLLVGVQYTWDEVYECGSGLHCSVMDYEGIKNLRIGNMMWDVLALAVMLLL
YRVLAYLALRNL
>OsABCG43
MKAQGFTESRLQLLSDISGAFRPGVLTALVGVSGAGKTTLMDVLAGRKTSGTIEGDIKLSGYPKKQETFA
RISGYCEQTDIHSPNLTVYESIVYSAWLRLSSEVDKNTRKVFVEEVMSLVELDVLRDALVGLPGVSGLST
EQRKRLTIAVELVANPSIIFMDEPTSGLDARAAAIVMRTVRNTVNTGRTVVCTIHQPSIDIFESFDELLL
LKRGGRVIYAGQLGLHSQILVEYFEAIPGVPKITEGYNPATWMLEVSSSLAEARLDIDFAEVYANSALYR
SNQELIKQLSVPPPGFQDLSFPTKYSQNFLNQCVANTWKQFQSYWKDPPYNAMRYVMTLLYGLVFGTVFW
RRGKNIESVNDLNNLLGATYAAVFFLGAANLLTLLPVVSVERTVFYREKAAGMYSPLSYAFAQGFVEFCY
SAVQGVLYTILIYSMIGYEWKADKFFYFLFFMIAAFAYFTLFSMMLVACTASEMLAAVLVSFVLSSWNNF
AGFIIPRPLIPVWWRWFYWANPVSWTIYGVIASQFADSDRVVTVPGQSTTMVVKDFLEKNMGFKHDFLGY
VVLAHFGYVIIFFFLFGYGIKCLNFQKR
>CsPDR8
MERRTSRNFSRSISRSFSRASWSMEDVFANGNPSRRSSRVDEDEEALRWAAIEKLPTYDR
LRTSILQSVNEPDPRIAGNLPLHKEVDVRKLGVSDRQDFIDRIFKVAEEDNEKFLRKQKN
RIDRVGIRLPTVEVRFEHLTMEADCHVGNRALPTLPNVARNMAESAISLVGVKLAKQTKL
TILKDASGIVKPSRMTLLLGPPSSGKTTLLLALAGKLDPSLKVKGEVSYNGHKLKEFVPQ
KTSAYISQNDVHMGIMTVKETLDFSARCQGVGTRYELLSELARREKDAGIKPEAEVDLFM
KATAMEGVESSLITDYTLKILGLDICKDTIVGDEMIRGISGGQRKRVTTGEMIVGPTKTL
FMDEISTGLDSSTTYQIVKCLQQIVHLTEGTILMSLLQPAPETFDLFDDIILVSEGQIVY
QGPRDHVVEFFESCGFKCPERKGTADFLQEVTSRKDQEQYWADRRKPYRYVPVSEFASRF
KRFHVGLRLENELSISYDKSRGHKAALVFSENVVPKMELLKACFDKEWLLMKRNSFVYIF
KTVQIIIVAIIASTVFLRTRMHTRDQSDGAVFIGALLFSLISNMLNGFSELAMTISRLPV
FYKQRDLKFHPPWTYTIPTVILGIPTSLLESVVWLVVTYYTIGFAPEASRFFKQLLLIFL
VQQMAAGVFRLIAGICRSMIIANTGGSLILLLIFLLGGFIIPRGEIPKWWIWGYWISPLT
YGFNAIAVNEMFAPRWNKLIPNTTVTLGVKVLENFDVFPNKNWYWIGIAAILGFAILFNI
LFTIALTYLNPLTKHQAIMSEETASEMEANQEDSQEPRLRRPMSKKDSFPRSLSASDGNN
TREVNMQRMSSKSEANGVAAKKGMILPFSPLAMSFDTVNYYVDMPPEMKEQGVTEDRLQL

LRGVTGAFRPGILTALMGVSGAGKTTLMDVLAGRKTGGYIEGDVRISGFPKKQETFARIS
GYCEQNDIHSPQVTIRESLIYSAFLRLPKEVSKEEKMVFVDEVMDLVELDNLKDAIVGLP
GVTGLSTEQRKRLTIAVELVANPSIIFMDEPTSGLDARAAAIVMRAVRNTVDTGRTVVCT
IHQPSIDIFEAFDELLLMKRGGQVIYFGPLGRNSQKIIEYFESIPGVPKIKEKYNPATWM
LEVSSVAAEVRLGMDFAEHYKSSSLSKRNKELVTDLSTPPPGAKDLYFESQYSQSTWGQL
KCCLWKQWWTYWRSPDYNLVRYFFTLAAALMIGTVFWKVGTKRDSSTDLTMIIGAMYAAV
LFVGINNCQTVQPIVSVERTVFYRERAAGMYSAFPYVLAQVLVEIPFILVQTTYYTLIVY
SMVSFQWTAPKFFWFYFINFFSFLYFTYYGMMTVSITPNHHVAAIFAAAFYALFNLFSGF
FVPRPRIPKWWVWYYWICPIAWTVYGLIISQYGDVEKKISVPGLSDPISIKSYIESHFGY
DPNFMGPVAGVLVGFAAFFAFMFAYCIKTLNFQLR
>CsPDR12
MDSGEIYRVSSARINSSSIWRNSAMEVFSRSSRDDDDEEALKWASIERLPTYLRVRRGIL
NLDGESAREIDVQNLGLLERRNILERLVKIAEDDNERFLLKLKNRMERVGLDLPAIEVRF
EHLEVEAEAHTAGRALPTMFNFSLNMLEGFLSYFHIIPNRKKQLSILHDVSGIIKPGRMT
LLLGPPSSGKTTLLKTLAGKLGKDLKFSGRVTYNGHGMNEFVPQRTSAYISQQDLHIGEM
TVRETLSFSARCQGVGPRYDMLTELSRREKAANIKPDPDLDIIMKAAALGGQETNVVTDY
VLKILGLEICADTMVGDEMFRGISGGQKKRVTTGEMLVGPSRALFMDEISTGLDSSTTYQ
IVNSMRQYIHILNGTALISLLQPAPETYELFDDIILISDGQVVYQGPRENVLEFFQHMGF
TCPQRKGVADFLQEVTSRKDQEQYWTKRDEVYRFVSVEEFSEAFQSFHVGKKLGDELATP
FDKSKSHPAALTTEKYGASKKELLKACISRELLLMKRNSFVYIFKLIQLILMAFVTMTLF
FRTEMHRRTVDDGSVYMGALFFAIIITMFNGFSELALTILKLPVFYKQRDFLFFPPWAYS
IPTWILKIPITFVEVGIWVVMTYYVVGFDPNAGRFFKHFLMLLFVNQMASALFRLIGALG
RNIIVANTFGSFALLTVLVLGGFVLARDDVHPWWIWGYWISPMMYAQNGIAVNEFLGHKW
RHPAPNSNESLGVLILKSRGIFPQASWYWIGVGATIGYILLFNFLFTIALQYLDPFEKPQ
AIVSKETSTDKSVKKSQDVQELELSSKGKSSSERTENQISLSSRTSSARVGSFSEEANQN
KKRGMVLPFEPHSITFDEIRYAVDMPQEMKSQGVTEDRLELLKGVSGSFRPGVLTALMGV
SGAGKTTLMDVLAGRKTGGYIEGNITISGYPKKQETFARIAGYCEQTDIHSPHVTVYESL
VYSAWLRLPPDVDSATRKMFVEEVMELIELNPLRDAIVGLPGVSGLSTEQRKRLTIAVEL
VANPSIIFMDEPTSGLDARAAAIVMRTVRNTVDTGRTVVCTIHQPSIDIFDAFDELFLLR
RGGEEIYVGPVGRHSSQLIEYFESIEGVPKIKDGYNPATWMLEITTAAQETTLGVNFNTL
YKDSELYRRNKALIKELSVPNENSNELYFPTKYSQSFFIQCIACLWKQHLSYWRNPPYSA
VRFLFTTFIALMFGTIFWDLGSKRGTQQDLFNAMGSMYAAVLFIGVQNATSVQPVVAIER
TVFYRERAAGMYSALPYAFGQVVIELPYIFIQTVVYGVIVYGMIGFEWTAAKFFWYIFFM
YFTLLYFTFYGMMTVAVTPNHNIAAIVSSAFYGFWNLFSGFIVPRTRIPIWWRWYYWICP
VAWTLYGLVTSQFGDINDPMDSNQTVAEFVSNYFGYKYDFLGVVAAVHVGITVLFGFIFA
FSIKVFNFQKR
>StPDR1
MSTRGENGASKNEEDLVLAALQRSPTYIRAQTSIFRGIGGEVALVDVGKMKGEEQKQVLD
VLINAINEDTELFFKRVKERFEKVDLEFPKVKVCFQHLKVDAMVHVGSRALPTIPNFIFN
MTEMSGRVTYNGHDLTEFVPQRTAAYVSQRDSHIAEMTVRETLEFSGRCQGVGFKHDLLM
ELLRREKNAGIIPDQDLDIFIKVICVEKPLHQSHVDVIVFYQAVALGEQTSIVVDYILKI
LGLDICANTLVGDEMLKGISGGQKKRLTTGELLMGAPRVLLMDEISTGLDSSTTFQIIKY
LKYTTRAFDGTTLVSLLQPDPETYSLFDDIILLSEGQIIYQGPRETALEFFEFMGFKCPS
RKNVADFLQELTSEKDQGQYWFLNSQYSYVSVTKFAEGFQSFHVGNALAQELTIPFDKRD
GHPAALSSSTYGVKKSELLKISFDWQLLLLKRNSAVLVFKVTQLFLIILIMMSVFFRSTM
HHDTLEDGAVYLGALYFAILMVLFNGFLELFTIFDRDSIPSWWIWGYWFSPLMYAQNSAS
VNEFRGHSWDKRFRDNISLGQMLLKVRSLFPENYWYWIGVGALIGYVIVFNVLFTLFLTY
LNRNKMQVLWELIMVLQLSAALGSQQAVVSKKNTQNKDKEQESEDNMVPFREFLNHSHSF
TGREIKKRRGMVLPFEPLSMCFKEISYYVDVPMELKLQGLGDKLQLLVNVTGAFRPGVLT
ALVGVSGAGKTTLMDVLAGRKTGGHITGNIYISGHPKKQETFARVSGYCEQNDVHSPCLT
IHESLLFSAWLRLSSQVDVKTQKAFVEEVMELVELTSLRRALVGLPGVDGLSTEQRKRLT
IAVELVANPSIVFMDEPTSGLDARSAAIVMRTVRNIVDTGRTIVCTIHQPSIDIFESFDE
AIQGVHRIRSGQNPAAWVLEVTSSAEENRLGVDFADIYRKSTLFQYFSPSPSVQNEEMVE
SLSKPQEGSAELYFSSKYSQSFFGQFLACLWKQNLSYWRNPQYTAVRFFYTVIISLMFGS

ICWKFGSKRFLS
>StPDR2
MEPSDLSNLRGRSIRGSMRGSMRENSNSIWRNNGVEVFSRSNRDEDDEEALKWAALEKLP
TYDRLRKGILFGSQGVAAEVDVDDLGVQQRKNLLERLVKVADEDNEKFLLKLKNRIDRVG
IDFPSIEVRFEHLNIEADAYVGSRALPTFTNFISNFIESLLDSIHIFPSKKRSVTILKDV
SGYVKPCRMTLLLGPPGSGKTTLLLALAGKLDSDLRVTGKVTYNGHELHEFVPERTAAYI
SQHDLHIGEMTVRETLEFSARCQGVGSRYEMLAELSRREKAANIKPDVDIDMFMKILGLD
ICADTMVGDQMIRGISGGQKKRVTTGEMIVGPSKALFMDEISTGLDSSTTYSIVNSLKQS
VQILKGTALISLLQPAPETYNLFDDIILLSDGYIVYQGPREDVLEFFESMGFKCPDRKGV
ADFLQEVTSKKDQQQYWVRRDEPYRFITSKEFAEAYQSFHVGRKVSNELSTAFDKSKSHP
AALTTEKYGIGKKQLLKVCTEREFLLMQRNSFVYIFKFFQLMVIALMTMTIFFRTEMPRD
TETDGGIYTGALFFTVVMLMFNGLSELPLTLYKLPVFYKQRDFLFYPSWAYAIPSWILKI
PVTLLEVGMWTVLTYYVIGFDPNVGRFFKQFLLLVLVNQMASGLFRFIAAVGRTMGVAST
FGACALLLQFALGGFALARTDVKDWWIWGYWTSPLMFSVNAILVNEFDGEKWKHTAPNGT
EPLGPSVVRSRGFFPDAYWYWIGIGALAGFTILFNIAYSLALAYLNPFGKPQATISEEGE
NNESSGSSPQITSTAEGDSVGENQNKKKGMVLPFEPQSITFDEVVYSVDMPPEMREQGSS
DNRLVLLKGVSGAFRPGVLTALMGVSGAGKTTLMDVLAGRKTGGYIDGSIKISGYPKKQE
TFARISGYCEQNDIHSPYVTVYESLVYSAWLRLPQDVDEHKRMMFVEEVMDLVELTPLRS
ALVGLPGVNGLSTEQRKRLTIAVELVANPSIIFMDEPTSGLDARAAAIVMRAVRNTVDTG
RTVVCTIHQPSIDIFEAFDESMPGVGKIEEGYNPATWMLEVTSSSQEMSLGVDFTDLYKN
SDLCRRNKALITELSVPRPGTSDLHFENQFSQPFWVQCMACLWKQRWSYWRNPAYTAVRF
LFTTFIALIFGSMFWDLGTKVSRPQDLTNAMGSMYAAVLFLGVQNASSVQPVVSVERTVF
YREKAAGMYSAIPYAFAQVFIEIPYVFVQSVVYGLIVYSMIGFEWTVAKFFWYFFFMFFT
FLYFTFFGMMTVAITPNQNVASIVAGFFYTVWNLFSGFIVPRPRIPIWWRWYYWGCPVAW
TLYGLVASQFGDLQDIVNGQTVEEYLRNDYGIKHDFLGVVAGVIVAFAVVFAFTFALGIK
AFNFQKR
>StPDR3
MAQLVSSDDIESIRMDLSEIGRSLRSSFRRQTSILRSNSALSASEKDDVVDEENMLAWAA
IERLPTYDRLRSSVFEEVNGNEANVKTKRVTDVTKLRPVERHVFIEKMIKHIEHDNLQLL
HKIRKRIDKVGVELPTVEVRYKNLTIEAECELVHGKPLPTLWNSLKSTIMNLARLPGLQS
EMAKIKIINDVSGVIKPGRMTLLLGPPGCGKTTLLKALSGNLDNSLKVSGEISYNGYKLE
EFVPQKTSAYISQNDLHIPEMTVRETVDYSSRFQGVGSRADIMIDLSRREKEAGIVPDPD
IDTYMKILGLDICADTLVGDAMRRGISGGQKKRLTTGELIVGPTKALFMDEISNGLDSST
TYQIVACLQQLAHITDATILVALLQPAPETFDLFDDIILMAEGKILYHGPRNSALEFFES
CGFKCPERKGVTSKKDQAQYWHGTKETYKFLSVDTLSRKFKESPYRKKLNDELSVAYDKS
RCHRNSITFHDYSLPKWELFRACMSRELLLMKRNSFIYIFKNVQLVFIAFITMTVFLRTR
MDTDLLHANYYLGALFFALIILLVDGFPELTMTIARLSVFYKQNDLCFYPAWAYAIPAAI
LKIPLSVLESVIWTCLTYYVIGFSPEAGRFFRQLLLLFAVHMTSISMFRFLASVCRTVVA
STAAASMPVWLKWGFWISPLTYGEIGLSVNEFLAPRWQKTLSTNTTIGNEVLESRGLNFD
GYLYWISVCALFGFTILFNIGFTLALTFLKAPGSRAIISRDKYSQIEGNSDSSDKADAEE
NSKTTMDSHEGADITGALRPGVLAALMGVSGAGKTTLLDVLAGRKTSGHVEGEIKVGGYP
KVQETFARVSGYCEQTDIHSPQITVEESVIFSAWLRLHPQIDSKTKYEFVKEVLETIELD
GIKDTMVGMPGVSGLSTEQRKRLTIAVELVANPSIIFMDEPTTGLDARSAAIVMRAVKNV
ADTGRTIVCTIHQPSIDIFEAFDEGISGVPKIKNNYNPATWMLEVTSTSSEAETSIDFAE
VYKNSALHKDDQQSVFSVFGAMFTAVIFCGINNSSSVLPYVTTERSVLYRERFAGMYASW
AYALAQVAIEIPYLLAQALAFTVITYPMIGYYWSAHKVLCCNSSVILLHNVQPLCRILDT
KSSNSKMVDLVLLSSTYIVDVEWDAYFTIWRC
>StPDR4
MEGGENILRVSSARLSGSNVWRNSAMDVFSRSSSREDYDDEEALRWAALEKLPTYRRIRR
GLLLEEEEGQSREVDITKLDLIERRNLLDRLVKIADEDNEKLLMKLKQRIDRVGLDLPTI
EVRFEHLNIDAEARVGSRALPTIFNFTVNILEDFLNYLHILPSRKKPLPILHGVGGIIKP
GRMTLLLGPPSSGKTTLLLALAGKLDNDLKVSGRVTYNGHGMDEFVPQRTSAYISQNDLH
IGEMTVRETLAFSARCQGVGTKYVYYEYAEILAELSRREKEANIKPDPDIDIFMKSAWNE
GQEANVITDYTLKILGLEICADTLVGDEMIRGISGGQRKRLTTGLDKQKPWRVTYNGHGM

DEFVPQRTSAYISQNDLHIGEMTVRETLAFSARCQGVGTKYVVYYEYAEILAELSRREKEA
NIKPDPDIDIFMKSAWNEGQEANVITDYTLKILGLEICADTLVGDEMIRGISGGQRKRLT
TGLDKQKPWEMMVGPARALFMDEISTGLDSSTTYQIVNSIRQSIHILQGTAVISLLQPAP
ETYDLFDDIILLSDGQIVYQGPRENVLEFFEYLGFKCPQRKGVADFLQEVTSRKDQEQYW
SRRDEPYRFITACEFSDVFQSFDVGRKLGDELAVPFDKSKSHPAALTTKRYGISKKELLK
ACTAREYLLMKRNSFVYIFKMVQLTLMASIAMTLFLRTEMHRDTTIDGAIYLGALFYAVI
TIMFNGFSELALSIMKLPSFYKQRDFLFFPAWAYALPTWILKIPITLVEIAIWVCMTYYV
IGFEADVGRFFKQIFLLICLSQMASGLFRFLAALGRNIIVANTFGSCALLIVLVMGGFIL
SRGSYCQSDDVKQWLIWGYWISPMMYAQNAIAVNEFLGKSWAHVPPNSTGTDTLGVSFLK
SRGIFPEARWYWIGAGALFGYVLLFNFLFTVALAYLNPFSKPQAILSEEIVAERNASKRG
EVIELSPIGKSSSDFARSTYGIKAKYAERGNDVPEMKTQGFIEDRLELLKGVSGAFRPGV
LTALMGVSGAGKTTLMDVLAGRKTGGYVEGTISISGYPKQQETFARISGYCEQTDIHSPH
VTVYESLLYSAWLRLPREVDTETRKSFIEEVMELVELTPLREALVGLPGVNGLSTEQRKR
LTVAVELVANPSIIFMDEPTSGLDARAAAIVMRTVRNTVDTGRTVVCTIHQPSIDIFDAF
DEGIDGVLKIRDGYNPATWMLEVTSLAQEAVLGIDFTELYKNSELYSHKTNGFEPNFRGK
QQDILNAIGSMYAAILFLGIINASSVQPVVAIERTVFYRERAAGMYSALPYAFGQVMIEL
PHLFLQTIIYGVIVYAMIGFEWTVTKFFWYLFFMYFTLLYFTLYGMMTVAVTPNHTIASI
VSSAFYTIWNLFCGFVVPKTVILLLSNCITFIQQHMFKLARPT
>OsPDR9
MDAAGEIQKVASMRLGGSMRGDSGSMWRRGDDVFSRSSREEDDEEALRWAALEKLPTYDRVRRAILPLGG
DDGAGDGGGKGVVDVHGLGPRERRALLERLVRVADEDNEKFLLKLKDRVDRVGIDMPTIEVRFEHLEAEA
EVRVGNSGLPTVLNSITNTLEEAGNALGILPNRKQTMPVLHDVSGIIKPRRMTLLLGPPGSGKTTLLLAL
AGRLGKDLKASGKVTYNGHGMEEFVPERTAAYISQHDLHIGEMTVRETLAFSARCQGVGSRFDMLTELSR
REKAANIKPDADIDAFMKAAAMGGQEANVNTDYILKILGLEICADTMVGDEMLRGISGGQRKRVTTGEML
VGPARALFMDEISTGLDSSTTFQIVNSLRQTVHILGGTAVISLLQPAPETYNLFDDIILLSDGQIVYQGP
REDVLEFFESMGFKCPDRKGVADFLQEVTSKKDQRQYWARHDKPYRFVTVKEFVSAFQSFHTGRAIANEL
AVPFDKSKSHPAALATTRYGAPGKELLKANIDREILLMKRNSFVYMFRTFQLMVVSLIAMTLFFRTKMKR
DSVTSGGIYMGALFFGVLMIMFNGFSELALTVFKLPVFFKQRDLLFYPAWSYTIPSWILKIPITFIEVGG
YVFLTYYVIGFDSNVGSFFKQYLLMLAINQMAGSLFRFIGGAARNMIVANVFASFMLLIFMVLGGFILAR
EQVKKWWIWGYWISPMMYAQNAISVNELMGHSWNKIVNSSASNETLGVQVLKSRGVFPEARWYWIGFGAM
IGFTILFNALFTLALTYLRPYGNSRQSVSEEELKEKRANLNGEIVGDVHLSSGSTRRPMGNGTENDSTIV
DDDTEVTQRGMVLPFTPLSLSFDNVRYSVDMPQEMKAQGVADDRLELLKGVSGSFRPGVLTALMGVSGAG
KTTLMDVLAGRKTGGYIEGSINISGYPKKQETFARVSGYCEQNDIHSPQVTVYESLLFSAWLRLPEDVDS
NTRKMFIEEVMELVELKSLRDALVGLPGVNGLSTEQRKRLTIAVELVANPSIIFMDEPTSGLDARAAAIV
MRTVRNTVNTGRTVVCTIHQPSIDIFEAFDELFLMKRGGEEIYAGPLGHHSSELIKYFESIPGVSKIKDG
YNPATWMLEVTTIGQEQALGVDFSDIYKKSELYQRNKALIKDLSQPAPDSSDLYFPTQYSQSSLTQCMAC
LWKQNLSYWRNPPYNAVRFFFTTVIALLFGTIFWDLGGKVTKSQDLFNAMGSMYAAVLFIGVMNCTSVQP
VVAVERTVFYRERAAGMYSAFPYAFGQVVIEIPYTLVQATVYGIIVYAMIGFEWTAAKFFWYLFFMVFTL
LYFTFYGMMAVGLTPNYHIASIVSSAFYAIWNLFSGFVIPRPRVPIWWRWYCWACPVAWTLYGLVVSQFG
DIETPMEDGTPVKVFVENYFGFKHSWLGWVATVVAAFAFLFASLFGFAIMKFNFQKR
>OsABCG50
MSSSSSHHPEFASCTANDDEHHLDEFELELVVQDVQRQQNNGSANTDQHERENLLLLDDS
SKSGALKRRLFFDNLLKNVQDDHIRFLHRQKERIDRVDVKLPAIEVRYNNLSVEAECRTA
NGDHLPSLWNSTKGAFSGLVKLLGLETERAKINVLEDVSGIIKPCRLTLLLGPPGCGKST
LLRALSGKLDKSLKVTGDISYNGYQLDEFVPEKTAAYISQYDLHIPEMTVRETLDFSSRC
QGVGRRPKILKEVSARESAAGIIPDADIDIYMKAISVEASKRSLQTDYILKIMGLEICAD
TMVGDAMIRGLSGGQKKRLTTAEMIVGPARAYFMDEISNGLDSSTTFQIISCFQQLTNIS
EYTMVISLLQPTPEVFDLFDDLILMAEGKIIYHGPRNEALNFFEECGFICPERKEVADFL
QEILSCKDQQQYWSGPNESYRYISPHELSSMFKENHRGRKLEEPIVSPKSELGKEALAFN
KYSLQKLEMFKACGAREALLMKRSMFVYVFKTGQLAIIALVTMSVFLRTRMTTDFTHATY
YMGALFFSILMIMLNGTPEISMQIRRLPSFYKQKSYYFYSSWAYAIPASVLKVPVSILDS
LVWICITYYGIGYTASVSRFFCQFLMLCFVHQSVTSLYRFIASYFQTPTASFFYLFLALT
FFLMFGGFTLPKPSMPGWLNWGFWISPMTYAEIGTVINEFQAPRWQKETIQNITIGNRIL
INHGLYYSWHFYWISIGALFGSIILFYIAFGLALDYITSIEEYHGSRPIKRLCQEQEKDS

NIRKESDGHSNISRAKMTIPVMELPITFHNLNYYIDTPPEMLKQGYPTKRLQLLNNITGA
LRPGVLSALMGVSGAGKTTLLDVLAGRKTGGYIEGDIRIGGYPKVQETFVRILGYCEQAD
IHSPQLTVEESVTYSAWLRLPSHVDKKTRSEFVAEVLETVELDQIKDVLVGTPQKNGLSM
EQRKRLTIAVELVSNPSVILMDEPTTGLDTRSAAIVIRAVKNICKTGRTVVCTIHQPSTK
IFEAFDELILMKNGGKIIYNGPIGERSSKVIEYFEKISGVLKVKSNCNPAAWMMDVTSTS
MEVQHNMDFAILYDESSQHRDIVELVEKLSIPIPNSEILSFSHRFPRNGWIQLKACLWKQ
NLTYWRSPEYNLRRIMLTVISALVYGVLFWKRAKILNDEQDLFNVFGAMYLGSTTIGSYN
HQSIIPFSTTERIVMYREKFAGMYSSWSYSFAQAAIEIPYVFIQVVLYTLIIYPSIGYYW
TTHKFIWFFYTTFCSSLSYIYVGLLLVSLTPNVQVATILASFFNTMQTLFSGFILPAPQI
PKWWVWLYYLTPTSWTLDALLTSQYGNIEKEVRAFGETKSVSIFLNDYFGFHKDKLSLVA
AVLIAFPFVLIILFSFSIEKFNFQKR
>AhAQW44869.1
MASETMVVEIEHDDNEDEKGNKRNEEKEKEGMCMCMTWKDVWVTASMGNKGSKPILSGLT
GYAKPGQLLAIMGPSGSGKSTLLDALAGRLDSNTRQTGEILINGRKEALAYGISAYVTQD
DTLLTTLTVREAVHYSAQLQLPDSMSKAEKRERAEITIREMGLQDAINTRIGGWGIKGIS
GGQKRRVSICIEILTRPRLLFLDEPTSGLDSAASYYVMKRIANLVQKDGIQRTVIASIHQ
PSTEVFQLFHNLCLLSSGTTVYFGPASTATQFFASNGFPCPALQNPSDHLLKTINKDFDQ
DTDMGLSGTRTVPTEEAIRILISAYESSEINQQVQQEVAMLSNQETRSVDHKKRRRAGFL
NQCIVLTKISCVNMFRDLGYYWLRLGIYIALAIATATVFYDLGTSFSTIQDRGALLMFES
SFITFMTIGGFPSFVEVMKVFERERLNGHYGVVAFVIGNTVSSVPYLVLVSVIPGAIAYY
LPHLQRGFDHFVYFICVLFSCLMLVESLMMIVASVVPNYLMGIISGAGIQGIMMLAGGFF
RLPNDLPKPFWRYPMFYVAFHRFAFQGLYKNEFQGLSFATNDGNGSYISGDQILRDTWQV
DMSYSKWVDLGILLGMILVYRVLFFLIIKITEKLKPFFVSFISGSPKRATLVMENNDDNS
ALSHQA
>AtABCA9
MTLREGLPLFHQQFTALFKKNLLLSWRNKRATCLHLFSSFFFILLIFSIEESSKASDLTSTRHKNVTDPK
ALVSLPILPCEDKFFVRLPCFDFVWSGNQSRRVTDIVSAIMANNPGRPIPTNKVQSFTKPEEVDAWFMSH
PSQVTGALHFVEKNATVISYGIQTNSSSEKKRGRREDPTFKFLVPLQIAAEREIARSLIGDPKFSWDFGF
KEFARPAIGGEVIISAFYLMGPVFFLAFSMFGFVLQLGSVVTEKELKLREAMTTMGVYESAYWLSWLIWE
GILTFVSSLFLVLFGMMFQFEFFLKNSFVLVFLLFFLFQFNMIGLAFALSSIISKSSSATTVGFLVFLVG
FITQIVTTAGFPYSSAYSIGSRVIWSLFPPNTFSAGLQLLLEATSSPGDSGISWSERAICAGGESTCVIT
TNKIYIWLVGTFFFWFVLALYFDNIIPNASGVRKSIFYFLKPSYWTGKEGNKVEVPPVEHITPEDEDVLE
EEILVKQQAMDGRVDPNIAVQIHGLAKTYPGTTKLGCCKCTKTSPFHAVKGLWMNIAKDQLFCLLGPNGA
GKTTTISCLTGINPVTGGDAKIYGNSIRSSVGMSNIRKMIGVCPQFDILWDALSSEEHLHLFASIKGLPP
SSIKSIAEKLLVDVKLTGSAKIRAGSYSGGMKRRLSVAIALIGDPKLVFLDEPTTGMDPITRRHVWDIIQ
ESKKGRAIILTTHSMEEADILSDRIGIMAKGRLRCIGTSIRLKSRFGTGFVATVSFIENKKDGAPEPLKR
FFKERLKVEPTEENKAFMTFVIPHDKEQLLKGFFAELQDRESEFGIADIQLGLATLEEVFLNIARRAELE
SATVEGTMVTLELESGIAVEIPVGARFVGIPGTENAENPRGLMVEVYWQQDGSGSMCISGHSAEMRIPEN
VSVIYEPSSQVLGHGQRRVRGIVIDYESNN
>AtABCD1
MPSLQLLQLTERGRGLVASRRKSILLAAGIVAAGGTAVYLKSRVASRRPDSSRLCNGQSDDDETLEKLTA
TDQNAKITTKKKKGGGLKSLQVLTAILLSQMGKMGARDLLALVATVVFRTALSNRLAKVQGFLFRAAFLR
RAPLFLRLISENIMLCFMLSTLHSTSKYITGALSLRFRKILTKIIHSHYFENMVYYKISHVDGRITHPEQ
RIASDVPRFSSELSDLILDDLTAVTDGILYAWRLCSYASPKYIFWILAYVLGAGTAIRNFSPSFGKLMSK
EQQLEGEYRQLHSRLRTHSESIAFYGGETREESHIQQKFKNLVSHMSHVLHDHWWFGMIQDFLLKYLGAT
VAVILIIEPFFSGHLRPDDSTLGRAEMLSNIRYHTSVIISLFQALGTLSISSRRLNRLSGYADRIHELMA
VSRELSGDDKSSFQRNRSRNYLSEANYVEFSDVKVVTPTGNVLVEDLTLRVEQGSNLLITGPNGSGKSSL
FRVLGGLWPLVSGHIVKPGVGSDLNKEIFYVPQRPYMAVGTLRDQLIYPLTSGQESELLTEIGMVELLKN
VDLEYLLDRYQPEKEVNWGDELSLGEQQRLGMARLFYHKPKFAILDECTSAVTTDMEERFAAKVRAMGTS
CITISHRPALVAFHDVVLSLDGEGGWSVHYKRDDSALLTDAEIDSVKSSDTDRQNDAMVVQRAFAAARKE
SATNSKAQSYQTQLIARSPVVDKSVVLPRFPQPQTSQRALPSRVAAMLNVLIPTIFDKQGAQLLAVACLV
VSRTLISDRIASLNGTTVKYVLEQDKAAFVRLIGLSVLQSGASSIIAPSLRHLTQRLALGWRIRLTQHLL
RNYLRNNAFYKVFHMSGNSIDADQRLTRDLEKLTADLSGLLTGMVKPSVDILWFTWRMKLLTGQRGVAIL
YTYMLLGLGFLRRVAPDFGDLAGEEQQLEGKFRFMHERLNTHAESIAFFGGGAREKAMVDKKFRALLDHS

LMLLRKKWLYGILDDFVTKQLPNNVTWGLSLLYALEHKGDRALVSTQGELAHALRYLASVVSQSFMAFGD
ILELHKKFLELSGGINRIFELDEFLDASQSGVTSENQTSRLDSQDLLSFSEVDIITPAQKLMASKLSCEI
VSGKSLLVTGPNGSGKTSVFRVLRDIWPTVCGRLTKPSLDIKELGSGNGMFFVPQRPYTCLGTLRDQIIY
PLSKEEAEKRAAKLYTSGESSTEAGSILDSHLKTILENVRLVYLLERDVGGWDATTNWEDILSLGEQQRL
GMARLFFHRPKFGVLDECTNATSVDVEEQLYRVARDMGVTFITSSQRPALIPFHSLELRLIDGEGNWELR
SIEQTTE
>HvABCD2
MPSLQLLQLTERGRGLLASRRCALAVVSGALVAGGALAYARSGRGQRRRGRPEAAAANDG
GDALGRNGERLGHSGTDGRLAGTTKRRKSALKSLHFLAAILLKKIGPSGTRYLLGLMLTA
VLRTAVGHRLAKVQGFLFKAAFLRRVPTFTRLIIENLILCFLQSAVYQTSKYLTGSLNLR
FKKILTDLVHADYFQNMVYYKISHVDHRISNPEQRIASDIPKFSSELSELVQDDLAAVAE
GLIYTWRLCSYASPKYMLWILAYILVAGGAIRNFSPAFGKMKSTEQHLEGEYRQLHSRLR
THAESVAFYGGEKREEYHIMQRFRALVGHLKHVLHENWWFGMIQDFFLKYFGATVAVVLI
IEPFFSGDLRPDSSTIGRADMLSNLRYHTSVIIALFQSLGTLSISSRRLNILSGYADRIR
ELLDVSRELSGVRDRSLNHSSSVGNYISEANHIEFSGVKVVTPAGNVLVDDLTLRVETGS
NLLITGPNGSGKSSLFRVLGGLWPLVSGHIVKPGVGSDLNKEIFYVPQRPYTAVGTLREQ
LIYPLTADQETEPLTYDGMVDLLKNVDLEYLLERYPLDKEVNWGDELSLGEQQRLGMARL
FYHKPKFAILDECTSAVTIDMEERFCKKVRAMGTSCITISHRPALVAFHNIVLSLDGEGG
WDVQHRRDDSSFYTEDSELTSLETERKSDALIVQRAFMNRAKSNASLRSKDSYSTKVIAN
STKLETGQTVRTPVIPHLQCSPRPLPLRATAMLKILIPKLLDKQGGQLLAVALLIFSRTV
ISDRIASLNGTTVKFVLEQDKAAFVRLVGVSVLQSAANSFVAPSLRTLTSRLALGWRIRL
TNHLLQYYLRRNAFYKVFNMSGKSIDADQRLTLDVDKLTTDLAGLVTGMVKPLVDILWFT
WRMKLLSGRRGVGILYAYMLLGLGFLRAISPDFGHLASEEQELEGTFRFMHSRLRTHAES
IAFFGGGSREKAMVEAKFMKLLNHSKVLLRKQWLYGVVDDFVTKQLPHNVTWGLSLLYAL
EHKGDRALTSTQGELAHALRFLASVVSQSFIAFGDILELHKKFLELSGGINRIFELEEIL
RVAQKDTPVPSSAISAASDEIIEFHEVDIVTPSQKLLARKLSCSVVQGKSLLLTGPNGTG
KSSVFRVLRDLWPAFSGRVTKPSEGMFHVPQSPYTSLGTLRDQIIYPLSREEAEMKILSL
YKSSNRSSAPELLDDHLKTVLVNVRLVYLLEREGWDSTPNWEDVLSLGEQQRLGMVSVNS
TASCANCRNHCLSP
>HvABCD1
MSSLQLLQLTEHGRNLFSSRRRTLAVVSGALLAGGTLAYAQTSRRRKHREENPCNDANVH
SRSEENISQNGVDGKVVKTRKKKNLLKSLHFLAAILLKKIGPSGTNYLLGLMLTAVLRTA
IGHRLAKVQGYLFKSAFLRRVPTFTRLIIENLLLCFLQSTVYQTSKYLTGSLSLRFKKIL
TDLIHADYFENMVYYKISHVDHRISNPEQRIASDIPKFCAGLSDLVQDDLIAVADGLIYI
WRICSYASPKYVLWILAYVLGAGGTIRKFSPSFGKLKAMEQQLEGEYRQVHSRLRTHAES
VAFYGGENREESHIMQRFQALVRHLNVVLHENWWFGMIQDFLLKYLGATVGVILIVEPFF
AGDLKPDTSTLGRAEMLSNLRYHTSVIISLFQSLGTLSTSSRRLNLLSGYADRIHELLDV
SRELSGVRDRSMSRNSSAKNYISEANYIEFSGVKVVTPSGNVLVDDLTLRVESGSNLLIT
GPNGSGKSSLFRVLGGLWPLVSGHIVKPGVGSNLNKEIFYVPQRPYTAVGTLRDQLIYPL
TADQETEPLSYGGMVDLLKNVDLEYLLERYPVDKEVNWGDELSLGEQQRLGMARLFYHKP
KFAILDECTSAVTTDMEERFCNRVRAMGTSCITISHRPALVAFHDVVLSLDGEGGWKVQD
NRNGSFLPTESEFDALKSSETDRKSDALAVQRAFSANTKENTLSGPKDHSYSTQVIATSP
NMEIESTEQPHLIPQLQCSPRPLPVRVAAMSKILVPKVIDKQGAQLLAVALLVLSRTWIS
DRIASLNGTSVKYVLEQDKAAFIRLIGTSVMQSAANSIVAPSLRHLTSKIALGWRIRMTN
HLLAYYLKRNAFYKVFNMTGTDIDADQRITRDVEKLTNDLAGLVTGMVKPSVDILWFTWR
MKLLSGRRGVAILYAYMLLGLGFLRAVSPDFGDLSNQEQELESSFRLVI
>MtABCG20
MARLERDGTNKSLESLMDSHKPGGTTTNLNQLRTQKSIPGYGLEFTNLSYSIIKKQKKDG
VWINKETYLLHDISGQAIKGEIMAIMGPSGAGKSTFLDALAGRIAKGSLQGSVRIDGKPV
TTSYMKMVSSYVMQDDQLFPMLTVFETFMFAAEVRLPPSISRDEKKKRVHELLNKLGLQS
ATHTYIGDEGRRGVSGGERRRVSIGIEIIHKPSLLFLDEPTSGLDSTSAYSVVEKIKDIA
QGGSIVLMTIHQPSFRIQMLLDKITILARGRLIYMGRPDALHTHLSGFGRPVPDGENNIE
YLLDVITEYDQATVGLDPLVQYQHDGHKPDPAAMTPVPKPPRTPYRRNTPASKHMISLRS
QGFTAGTPQPDSSQFGLDDDDNDDDENFDNSLERRSVQTSRNIVTSGVYPRLASQFYQDF

SAKDFSVWLYNGVVGTPRRPPSWTPARTPGWTPGKTPLSGPRSFVSNQHSASYQDPYYIQ
KTNTVVGQSMDYSATSYAPSYEEFEIEEVLDEPDLGPKYANPWLREVAVLSWRTVLNVIR
TPELFASREIVLTVMALVLSTIFKNLGDTTFIDINRLLNFYIFAVCLVFFSSNDAVPSFI
MERFIFIRETSHNAYRASSYVISSLIVYLPFFAVQGLTFAVITKLMLHLKSNLFNFWMIL
FASLITTNAYVMLVSALVPSYITGYAVVIATTALFFLTCGFFLKRTQIPAYWKWLHYISA
IKYPFEGLLINEFKNNRGCYSGNKADLSPGPLGDVKPSKHHNASLPLNCLLGEDVLSTMD
ITMESLWYDILILLAWGVLYRFFFYLVLRFYSKNERK
>MtSTR2
MKTQGLELETVIDIKHKPVSFTGGLEFESLTYTVTKKKKVDGKWSNEDVDLLHDITGYAPKGCITAVMGP
SGAGKSTLLDGLAGRIASGSLKGKVSLDGNSVNASLIKRTSAYIMQEDRLFPMLTVYETLMFAADFRLGP
LSAVDKRQRVEKLIEQLGLSSSRNTYIGDEGTRGVSGGERRRVSIGVDIIHGPSLLFLDEPTSGLDSTSA
LSVIEKLHDIARNGSTVILTIHQPSSRIQLLLDHLIILARGQLMFQGSLKDVGHHLNRMGRKIPKGENPI
ENLIDVIQEYDQCDFVGVEVLAEFARTGMKPPLLSDMEEIISYTNSIAPSPSPLHRGSKYEEKSQDFSYS
SQISRRSLNDEFDHSIRSPYNNTPMSWSASNSAAFLKFTPSRLKNENKVQKPPSHASPGIYTYSSEILPA
TPTPHSSDYVVDENDYLTPTNSSQEHLGPKFANSYIGETWILMRRNFTNIRRTPELFLSRLMVLTFMGVM
MATMFHNPKNTLQGITNRLSFFIFTVCLFFFSSNDAVPAFIQERFIFIRETSHNAYRASCYTIASLITHM
PFLALQALAYAAIVWFALELRGPFIYFFLVLFISLLSTNSFVVFVSSIVPNYILGYAAVIAFTALFFLFC
GYFLSSEDIPLYWRWMNKVSTMTYPYEGLLMNEYQTNETFGSNDGVSITGFDILKSLHIGTEEIKKRNNV
LIMLGWAVLYRILFYIILRFASKNQRS
>AtABCG13
MTTPEGAMYVAWEDLTVVIPNFGEGATKRLLNGVNGCGEPNRILAIMGPSGSGKSTLLDALAGRLAGNVV
MSGKVLVNGKKRRLDFGAAAYVTQEDVLLGTLTVRESISYSAHLRLPSKLTREEISDIVEATITDMGLEE
CSDRTIGNWHLRGISGGEKKRLSIALEVLTKPSLLFLDEPTSGLDSASAFFVVQILRNIASSGKTVVSSI
HQPSGEVFALFDDLLLLSGGETVYFGEAESATKFFGEAGFPCPSRRNPSDHFLRCVNSDFDNVTAALVES
RRINDSSFSLHQLHETTNTLDPLDDIPTAEIRTTLVRKFKCSLYAAASRARIQEIASIVGIVTERKKGSQ
TNWWKQLRILTQRSFINMSRDLGYYWMRIAVYIVLSICVGSIFFNVGRNHTNVMSTAACGGFMAGFMTFM
SIGGFQSFIEEMKVFSRERLNGHYGVAVYTVSNLLSSLPFIILMCLSTSSITIYMVRFQSGGSHFFYNCL
DLICAITTVESCMMMIASVVPNFLMGVMLGAGYIGIMVLSAGFFRFFPDLPMVFWRYPVSYINYGAWALQ
GAYKNEMIGVEYDSPLPLVPKMKGELILQTVLGINPESSKWLDLAVVMMILIGYRIAFFAILKFREKVFP
VIHMLYTKRTLSHIQKRPSFRRMTPFPSRRYPVHHALSSQEGLNSPLH
>AtABCG32
MWNSAENAFSRSTSFKDEIEDEEELRWAALQRLPTYSRIRRGIFRDMVGEPKEIQIGNLEASEQRLLLDR
LVNSVENDPEQFFARVRKRFDAVDLKFPKIEVRFQNLMVESFVHVGSRALPTIPNFIINMAEGLLRNIHV
IGGKRNKLTILDGISGVIRPSRLTLLLGPPSSGKTTLLLALAGRLGTNLQTSGKITYNGYDLKEIIAPRT
SAYVSQQDWHVAEMTVRQTLEFAGRCQGVGFKYDMLLELARREKLAGIVPDEDLDIFMKSLALGGMETSL
VVEYVMKILGLDTCADTLVGDEMIKGISGGQKKRLTTGELLVGPARVLFMDEISNGLDSSTTHQIIMYMR
HSTHALEGTTVISLLQPSPETYELFDDVILMSEGQIIYQGPRDEVLDFFSSLGFTCPDRKNVADFLQEVT
SKKDQQQYWSVPFRPYRYVPPGKFAEAFRSYPTGKKLAKKLEVPFDKRFNHSAALSTSQYGVKKSELLKI
NFAWQKQLMKQNAFIYVFKFVQLLLVALITMTVFCRTTMHHNTIDDGNIYLGSLYFSMVIILFNGFTEVP
MLVAKLPVLYKHRDLHFYPSWAYTLPSWLLSIPTSIIESATWVAVTYYTIGYDPLFSRFLQQFLLYFSLH
QMSLGLFRVMGSLGRHMIVANTFGSFAMLVVMTLGGFIISRDSIPSWWIWGYWISPLMYAQNAASVNEFL
GHNWQKTAGNHTSDSLGLALLKERSLFSGNYWYWIGVAALLGYTVLFNILFTLFLAHLNPWGKFQAVVSR
EELDEREKKRKGDEFVVELREYLQHSGSIHGKYFKNRGMVLPFQPLSLSFSNINYYVDVPLGLKEQGILE
DRLQLLVNITGAFRPGVLTALVGVSGAGKTTLMDVLAGRKTGGTIEGDVYISGFPKRQETFARISGYCEQ
NDVHSPCLTVVESLLFSACLRLPADIDSETQRAFVHEVMELVELTSLSGALVGLPGVDGLSTEQRKRLTI
AVELVANPSIVFMDEPTSGLDARAAAIVMRTVRNIVNTGRTIVCTIHQPSIDIFESFDELLFMKRGGELI
YAGPLGQKSCELIKYFESIEGVQKIKPGHNPAAWMLDVTASTEEHRLGVDFAEIYRNSNLCQRNKELIEV
LSKPSNIAKEIEFPTRYSQSLYSQFVACLWKQNLSYWRNPQYTAVRFFYTVVISLMLGTICWKFGSKRDT
QQQLFNAMGSMYAAVLFIGITNATAAQPVVSIERFVSYRERAAGMYSALPFAFAQVFIEFPYVLAQSTIY
STIFYAMAAFEWSAVKFLWYLFFMYFSIMYFTFYGMMTTAITPNHNVASIIAAPFYMLWNLFSGFMIPYK
RIPLWWRWYYWANPVAWTLYGLLVSQYGDDERSVKLSDGIHQVMVKQLLEDVMGYKHDFLGVSAIMVVAF
CVFFSLVFAFAIKAFNFQRR
>OsABCG31
MVLPFQPLSMCFKNINYYVDVPAELKSQGIVEDRLQLLIDVTGAFRPGILTALVGVSGAGKTTLMDVLAG

RKTGGLIEGSITISGYPKNQETFTRISGYCEQNDVHSPCLTVIESLLYSACLRLPSHVDVNTRRVFVEEV
MELVELNALSGALVGLPGVNGLSTEQRKRLTIAVELVANPSIVFMDEPTSGLDARSAAIVMRTVRNIVNT
GRTIVCTIHQPSIDIFESFDELLFMKRGGQLIYAGPLGSKSRNLVEFFEAIPGVPKIRDGYNPAAWMLEV
TSTQMEQILGVDFAEYYRQSKLFQQTQEMVDILSRPRRESKELTFATKYSQPFFAQYAACLWKQNLSYWR
NPQYTAVRFFYTVIISLMFGTICWKFGSRRETQHDIFNAMGAMYAAVLFIGITNATSVQPVISIERFVSY
RERAAGMYSALPFAFSLVTVEFPYILVQSLIYGTIFYSLGSFEWTAVKFLWYLFFMYFTLLYFTFYGMMT
TAITPNHTVAPIIAAPFYTLWNLFCGFMIPRKRIPAWWRWYYWANPVSWTLYGLLTSQFGDLDQPLLLAD
GITTTTAVDFLRDHFGFRHDFLGVVAGMVAGFCVLFAVVFALAIKYLNFQRR
>HvEIBI1
MWAAEAPFSRSGSWREAEDEQEALRWAALQRLPTVARARRGLLRSPVVAPPGAGGPVEGDDALCEVDVAG
LSSGDRTALVDRLLADSGDAEQFFRRIRARFDAVHIEFPKIEVRYEDLTVDAYVHVGSRALPTIPNFICN
MTEAFLRHLRIYRGGRMKLPILDNINGIIRPSRMTLLLGPPSSGKTTLLLALAGRLGPGLKMSGSITYNG
HHLNEFVPQRTSAYVSQQDWHASEMTVRETLEFAGRCQGVGIKYDMLVELLRREKNAGIKPDEDLDVFMK
ALALEGRQTSLVAEYIMKILGLDICADTIVGDEMVKGISGGQKKRLTTGELLVGSARVLFMDEISTGLDS
ATTYQIIKYLRDSTHALDGTTIISLLQPAPETYELFDDVILISEGQIVYQGPREYAADFFAAMGFKCPER
KNVADFLQEVLSKKDQQQYWCQYDYPYQFVSVTKFAEAFKTFVIGKRLHEDLDRPYNRKHNHPAALSTSN
YGVKRLEILKSNFQWQRLLMKRNSFIYVFKFIQLLLVALITMTVFFRTTMHHDSVDDGIIYLGALYFAIV
MILFNGFTEVSMLVAKLPVLYKHRDLHFYPPWAFTLPSWLLSIPTSLIESGMWTLVTYYVVGYDPQFTRF
LGQFLLLFFLHQTSLALFRVMASLGRNMIVANTFGSFALLVVMILGGFIITKESIPVWWIWGYWISPMMY
AQNAISVNEFHGRSWSKPFADQNITLGEAVLTGYGLFKEKYWFWIGVGALLGYTIVLNALFTLFLTILNP
IGNMQAVVSKDAIRNKDSKRKSDRVALELRSYLHSTSLNGLKLKEQKGMVLPFQPLSMCFKNINYYVDVP
EELKKQGIAEDRLQLLVDVTGAFRPGILTALVGVSGAGKTTLMDVLAGRKTGGLIEGSVSISGYPKNQET
FTRISGYCEQNDVHSPCLTVIESLLYSACLRLPSHVNDDTQRAFVEEVMELVELNPLSGALVGLPGVNGL
STEQRKRLTIAVELVANPSIVFMDEPTSGLDARSAAIVMRTVRNIVNTGRTIVCTIHQPSIDIFESFDEL
LFMKRGGQLIYAGPLGSKSRNLVEFFEGIPGVPKIRDGYNPAAWMLDVTSTQMEQILGVDFAEYYRQSKL
FLQTKEIVEALSKPNSEVKELTFSTKYAQPFCAQFIACLWKQNLSYWRNPQYTAVRFFYTVIISLMFGTI
CWKFGSRRETQHDIFNAMGAMYAAVLFIGITNATSVQPVISIERFVSYRERAAGMYSALPFAFSLVTVEF
PYILVQSLVYGTIFYSLGSFEWTAVKFLWFLFFMYFTLLYFTFYGMMTTAITPNHMVAPIIAAPFYTLWN
LFCGFMIPRKLIPVWWRWYYWANPVSWTLYGLLTSQFGDLDQPLLLADGIRTTTVVAFLEEHFGFRHDFL
GVVATMVVGFCVLFAVVFALAIRNLNFQRR
>OsABCG3
MARAVHDRLPFLATPPPAPARGRNPPLAEMLRLVGAATVDSDAAAAAADEEANALSLPLPRGGVTPPPPG
GRTIQFRLAFTSLTYSVRAARRARPGGGDGGGGFRLPLQNRCDRVTAAAPDAHSSRARVLLDGITGEARE
GEILAVMGASGSGKSTLIDALANRISRDALKGSVTLNGEPLTGNVIKSISAYVMQDDLLFPMLTVAETLS
FAAEFRLPRALPAAKKRTRVLELIEQLGLRAAADTIIGDEGHRGVSGGERRRVSIGTDIIHDPILLFLDE
PTSGLDSTSAFMVVQVLRNIAESGSIVITSIHQPSQRILGLLDRLILLSGGRTVFSGPPSAIPAYFAEFG
YPVPDDENRAEFALDLIREFESLPAGTGQLVSFNKTWQVMHAARHNPNDDPWAPTMSLKEAISASISRGK
LVSGSDVAGEAASMHTYANPFWVEMKVLTKRSAINTRRMPELFLIRLGAVVITGAILATVFYKLDQSPKG
AQERLGFFAFAMSTMFYTCADALPVFLHERYIFLRETAYGAYRRTSYVLSNAIVSFPPLVVLSLAFAFTT
FFAVGLAGGVSGFAFYTLAILASFWAGSGFVTFLSGVIPHVMIGYTVVVAILAYFLLFSGFFINRDRIPD
YWIWFHYLSLVKYPFEGVLQNEFGRGGECYVRGTQMFDNSPLAVLPDTVKTRVLASIGTALGVKIGPNTC
VMTGHNVLREAAVTQLGKWECLLVTAAWGFFFRLLFYFSLVLGSKNKRR
>AtABCG6
MSRVVAADDNMALPFFSPEFGNVSGASSSPTTFAQLLQNVDDSTRRSHHQHHVDVDLASPDQSVPFVLSF
TDLTYSVKVRRKFTWRRSVSSDPGAPSEGIFSSKTKTLLNGITGEARDGEILAVLGASGSGKSTLIDALA
NRIAKGSLKGNVTLNGEVLNSKMQKAISAYVMQDDLLFPMLTVEETLMFAAEFRLPRSLSKSKKSLRVQA
LIDQLGLRNAANTVIGDEGHRGISGGERRRVSIGIDIIHDPILLFLDEPTSGLDSTSALSVIKVLKRIAQ
SGSMVIMTLHQPSYRLLRLLDRLLFLSRGQTVFSGSPAMLPRFFAEFGHPIPEHENRTEFALDLIRELEG
SAGGTRSLVEFNKGFRQRKAEPRSQTGLSLKEAISASISKGKLVSGATTTTHSSGSSPVSTIPTFANPFW
VELAVLAKRSMTNSRRQPELFGIRLGAVLVTGFILATMFWQLDNSPKGVQERLGCFAFAMSTTFYTCADA
LPVFLQERFIFMRETAYNAYRRSSYVLSHSLVALPSLIILSLAFAAITFWGVGLDGGLMGFLFYFLVILA
SFWAGSSFVTFLSGVVPHVMLGYTIVVAILAYFLLFSGFFINRDRIPGYWIWFHYISLVKYPYEAVLLNE
FGDPTKCFVRGVQIFDNTPLVAVPQGMKVRLLATMSKSLGMRITSSTCLTTGYDILQQQGVTDLTKWNCL
WVTVAWGFFFRILFYFSLLLGSKNKRR

>AtABCG12
MELESTSNGRRPPPPAEIGRGAYLAWEDLTVVIPNFSGGPTRRLLDGLNGHAEPGRIMAIMGPSGSGKST
LLDSLAGRLARNVIMTGNLLLNGKKARLDYGLVAYVTQEDILMGTLTVRETITYSAHLRLSSDLTKEEVN
DIVEGTIIELGLQDCADRVIGNWHSRGVSGGERKRVSVALEILTRPQILFLDEPTSGLDSASAFFVIQAL
RNIARDGGRTVVSSIHQPSSEVFALFDDLFLLSSGETVYFGESKFAVEFFAEAGFPCPKKRNPSDHFLRC
INSDFDTVTATLKGSQRIRETPATSDPLMNLATSEIKARLVENYRRSVYAKSAKSRIRELASIEGHHGME
VRKGSEATWFKQLRTLTKRSFVNMCRDIGYYWSRIVIYIVVSFCVGTIFYDVGHSYTSILARVSCGGFIT
GFMTFMSIGGFPSFIEEMKVFYKERLSGYYGVSVYIISNYVSSFPFLVAIALITGSITYNMVKFRPGVSH
WAFFCLNIFFSVSVIESLMMVVASLVPNFLMGLITGAGIIGIIMMTSGFFRLLPDLPKVFWRYPISFMSY
GSWAIQGAYKNDFLGLEFDPMFAGEPKMTGEQVINKIFGVQVTHSKWWDLSAIVLILVCYRILFFIVLKL
KERAEPALKAIQAKRTMKSLKKRPSFKKVPSLSSLSSRRHQPLHSLSSQEGLTSPIN
>AtABCG20
MSGLFGILSPAKRVNGDGLPLFYNIHKSVELQRCHRDTARVSVTLAELLMSVEDEGDDQSRAMDIAVASN
FWSSVPSSRVPSSSPFVLSFKDLTYSVKIKKKFKPFPCCGNSPFDGNDMEMNTKVLLNGISGEAREGEMM
AVLGASGSGKSTLIDALANRISKESLRGDITLNGEVLESSLHKVISAYVMQDDLLFPMLTVEETLMFSAE
FRLPSSLSKKKKKARVQALIDQLGLRNAAKTVIGDEGHRGVSGGERRRVSIGTDIIHDPIILFLDEPTSG
LDSTSAYMVVKVLQRIAQSGSIVIMSIHQPSYRILGLLDKLIFLSRGNTVYSGSPTHLPQFFSEFGHPIP
ENENKPEFALDLIRELEDSPEGTKSLVEFHKQWRAKQTSSQSRRNTNVSLKDAISASISRGKLVSGATNL
RSSFQTFANPFWTEMLVIGKRSILNSRRQPELFGIRLGAVLVTGMILATIFWKLDNSPRGIQERLGFFAF
AMSTTFYTCAEAIPVFLQERYIFMRETAYNAYRRSSYVLAHTIISIPALIILSAAFAASTFSAVGLAGGS
EGFLFFFFTILTAFWAGSSFVTFLSGVVSHVMIGFTVVVAILAYFLLFSGFFISRDRIPLYWIWFHYLSL
VKYPYEGVLQNEFEDPTKCFVRGIQMFDNSPLGQVPTAVKISLLKSMSGVLGINVTAETCVTTGIDILKQ
QGITEISKWNCLWITVAWGFFFRVLFYFTLLIGSKNKRR
>StABCG1
MSRIVAENMLQGGENVQFYDQRVQQAMEMSQASAYSSPTLGQMLKRVGDVRKEVTGDETPVHRILDMSDT
QSISSHSLPFVLSFNNLTYSVKVRRKMSFPAILRQPAAGVSTGDPVAGENLFTNTKFLLNNISGEARDGE
IVAVLGASGSGKSTLIDALANRIAKESLKGTITLNGEPLDSRLLKVISAYVMQDDLLYPMLTVEETLMFA
AEFRLPRSLSKSKKKMRVQALIDQLGLRNAAKTIIGDEGHRGVSGGERRRVSIGIDIIHDPIILFLDEPT
SGLDSTSAYMVVKVLQRIAQSGSIVIMSIHQPSYRILGLLDRMLFLSRGQTVYSGSPMNLPHFFSDFGHP
IPDSENRTEFALDLIRELEGSPGGTKSLVEFNKTWENTKRSNENPEIQTPTHGLSLKEAISASISRGKLV
SGTTSDIHTSPASMVPTYANPFWIEMLVLSKRSFTNSWRVPELFGIRLGAIVVTGFILATMFWQLDDSPK
GVQERLGFFAFAMSTTFYTCADALPVFLQERYIFMRETAYNAYRRSSYCLSHAIVSLPALIFLSFAFAAI
TFWAVGLVGGFSGFLFYFAIILASFWAGNSFVTFLSGVVPSVMLGYTIVVAILAYFLLFSGFFINRDRIP
PYWIWFHYLSLVKYPYEAVLQNEFDDATKCFVKGIQLFDNSPLGNVPNALKEKLLSTMSNTLNVKITSST
CVTTGADILVQQGITDLSKWNCLWITIAWGFFFRVLFYFSLLLGSKNKRR
>ZmGL13
MWAAEAAFSRSGSWREAEDEREALRWAALQRLPTVTRARRGLLRSPAPDGAAAVEGDDVLCEVDVAGLSS
GDRTALVDRLVADSGDSEHFFRRIRSRFDAVQIEFPKIEVRYEDVTVDAYVHVGSRALPTIPNFICNMTE
AFLRHLRIYRGGRVKLPILDNISGVIRPSRMTLLLGPPSSGKTTLLLALAGRLGPGLKMSGNITYNGHHL
NEFVPQRTSAYVSQQDWHASEMTVRETLEFAGRCQGVGIKYDMLVELLRREKNAGIKPDEDLDVFMKALA
LEGKQTSLVAEYIMKILGLDVCADTIVGDEMIKGISGGQKKRLTTGELLVGSARVLFMDEISTGLDSATT
YQIIKYLRNSTHALDGTTIISLLQPAPETYELFDDVILIAEGQIVYQGPREYAVDFFGAMGFRCPERKNV
ADFLQEVLSKKDQQQYWCHYDYPYQFVSVSKFAEAFKTFIIGKRLHQELTVPYNRHHNHPAALCTSSYGV
KRLELLKSNYQWQRLLMKRNSFIYVFKFIQLLLVALITMTVFFRSTMHHDSVDDGIIYLGALYFAIVMIL
FNGFTEVSMLVTKLPVLYKHRDLHFYPPWAYTLPSWLLSIPTSLYESGMWVLVTYYVVGYDPQFTRFLGQ
FLLLFFLHQTSLALFRVMASLGRNMIVANTFGSFALLVVMILGGFIITKESIPVWWIWGYWVSPMMYAQN
AISVNEFHGHSWNKQFANQNITMGEAILTGYGLFKEKYWFWIGVGALFGYAIILNILFTMFLTLLNPIGN
LQAVVAKDQVRHRDSRRKNDRVALELRSYLHSNSLSVLPPAGNLKEQKGMVLPFQPLSMCFRNINYYVDV
PVELKKQGVAEDRLQLLVDVTGAFRPGILTALVGVSGAGKTTLMDVLAGRKTGGLIEGSITISGYPKNQE
TFTRISGYCEQNDVHSPCLTVIESLLYSACLRLPSHVDADTQRVSLDWLYITRAFCPPYLLCSLS
>GhAAP80385.1
MRTSPSNTAMMEIQANKPAGTGMVVGGLSPLSETLWREKTDTELMGDVSARLTWEDLTVMVTLSNGATQK
VLEGLTGYAEPGTLTALMGPSGSGKSTLLDALSSRLAANAFLSGTILLNGRKTKLSFGTAAYVTQDDNLI
GTLTVRETISYSARLRLPDTMPWSAKRDLVEGTIIEMGLQDCADTVIGNWHLRGISGGEKRRVSIALEIL

MRPRLLFLDEPTSGLDSASAFFVTQTLRGLSRDGRTVIASVHQPSSEVFELFDQLYLLSEGKTIYFGQAS
EAYEFFAQAGFPCPALRNPSDHFLRCINSDFDKVKATLKGSMKLRFEASDDPLEKITTTEAIRTLINFYR
TSHQCYAAKEKVDEISKVRGTVLDSGGSQASFLMQSYTLTKRSFVNMSRDFGYYWLRLLIYVVVTVCIGT
IYLNIGTSYNSILARGACASFVFGFVTFMSIGGFPSFVEDMKVFQRERLNGHYGVTAFVIGNTLSAMPFL
IMITFISGTICYFMVRLHPGFEHYMFFVLCLYASVTVVESLMMAIASIVPNFLMGIITGAGIQGIFMLVS
GYFRLPNDIPKPVWRYPMSYISFHFWALQGQYQNDLKGLLFDNQPPELPKIPGEYILENVFQIDVGRSKW
IDLSVIFSMIIIYRIIFFLMIKISEDVTPWIRGLVARRRMQQKNGTQNTMVAPSLSQSPSLRNYVANRAN
GRGKR
>AtABCI14
MMQTCCIHQSFCFPHRVFPRFDASIGIKPPKLCQVGFIGKTQSYGISSPIRQRRLYVNLNANDGHPSMSM
LEEETSTENNAPSQEAELPFSKWSPSKYIWRGLSVPIIAGQVVLRILKGKIHWRNTLQQLERTGPKSLGV
CLLTSTFVGMAFTIQFVREFTRLGLNRSIGGVLALAFSRELSPVITSIVVAGRMGSAFAAELGTMQVSEQ
TDTLRVLGADPIDYLITPRVIASCLALPFLTLMCFTVGMASSALLSDAVYGISINIIMDSAHRALRPWDI
VSAMIKSQVFGAIISVISCSWGVTTTGGAKGVGESTTSAVVMSLVGIFIADFVLSSFFFQGAGDSLKNCV
>AtABCI15
MIGNPVIQVPSSLMPSSSMIACPRVSPNGVPYLPPKPRTRHLVVRAASNSDAAHGQPSSDGGKNPLTVVL
DVPRNIWRQTLKPLSDFGFGKRSIWEGGVGLFIVSGATLLALSWAWLRGFQMRSKFRKYQTVFELSHASG
ICTGTPVRIRGVTVGTIIRVNPSLKNIEAVAEIEDDKIIIPRNSLVEVNQSGLLMETMIDIMPRNPIPEP
SVGPLHPECGKEGLIVCDRQTIKGVQGVSLDELVGIFTRIGREVEAIGVANTYSLAERAASVIEEARPLL
KKIQAMAEDAQPLLSEFRDSGLLKEVECLTRSLTQASDDLRKVNSSIMTPENTELIQKSIYTLVYTLKNV
ESISSDILGFTGDEATRKNLKLLIKSLSRLL
>AtABCI13
MLSLSCSSSSSSLLPPSLHYHGSSSVQSIVVPRRSLISFRRKVSCCCIAPPQNLDNDATKFDSLTKSGGG
MCKERGLENDSDVLIECRDVYKSFGEKHILKGVSFKIRHGEAVGVIGPSGTGKSTILKIMAGLLAPDKGE
VYIRGKKRAGLISDEEISGLRIGLVFQSAALFDSLSVRENVGFLLYERSKMSENQISELVTQTLAAVGLK
GVENRLPSELSGGMKKRVALARSLIFDTTKEVIEPEVLLYDEPTAGLDPIASTVVEDLIRSVHMTDEDAV
GKPGKIASYLVVTHQHSTIQRAVDRLLFLYEGKIVWQGMTHEFTTSTNPIVQQFATGSLDGPIRY

>XP_006338462.1_P_ABCB19_St
MAETTEGKSMPEAEKKKEQSLPFYQLFSFADKYDYLLMTCGSIGAILHGSSMPVFFLLFGEMVNGFGKNQMD
LHKMTHEVSKYALYFVYLGLIVCASSYAEIGCWMYTGERQVSTLRKKYLEAVLKQDVGFFDTDARTGDIVFS
VSTDTLLVQDAISEKVGNFIHYLSTFLAGLVVGFVSAWRLALLSVAVIPGIAFAGGLYAYTLTGLTSKSRESYA
NAGIIAEQAIAQVRTVYSYVGETKALNSYSDAIQNTLKLGYKAGMAKGLGLGCTYGIACMSWALVFWYAGV
FIRNGQSDGGKAFTAIFSAIVGGMSLGQSFSNLGAFSKGKAAGYKLMEIIRQKPTIVQDTLDGKCLSEVSGNIEF
KNVTFSYPSRPDVIIFRDFNIFFPAGKTVAVVGGSGSGKSTVVSLIERFYDPNDGQVLLDNVDIKTLQLRWLRDQ
IGLVNQEPALFATTILENILYGKPDATMAEVEAATCASNAHSFITLLPNGYNTQVGERGVQLSGGQKQRIAIAR
AMLKNPKILLLDEATSALDAGSESIVQEALDRLMVGRTTVVVAHRLSTIRNVDSIAVIQQGQVVETGTHEELIS
KAGAYASLIRFQEMVGNRDFSNPSTRRTRSTRLSHSLSTKSLSLRSGSLRNLSYSYSTGADGRIEMISNAETDRK
NPAPQNYFCRLLKLNAPEWPYSIMGAVGSVLSGFIGPTFAIVMSNMIEVFYYTNPATMERKTKEYVFIYIGAGL
YAVVAYLIQHYFFSIMGENLTTRVRRMMLSAILRNEVGWFDEEENNSSLLAARLATDAADVKSAIAERISVILQ
NMTSLLTSFIVAFIVEWRVSLLILATFPLLVLANFAQQLSLKGFAGDTAKAHAKTSMIAGEGVSNIRTVAAFNA
QEKIISLFSQELRVPQMQSLRRSQMSGLLFGISQLALYGSEALILWYGAHLVNNGVSTFSKVIKVFVVLVITANS
VAETVSLAPEIIRGGEAVGSVFSILDRSTRVDPDDPEADPVESIRGDIELRHVDFAYPSRPDVSVFKDLNLRIRAG
QSQALVGASGSGKSSVIALIERFYDPTGGKVMIDGKDIRRLNLKSLRLKIGLVQQEPALFAASIFENIAYGKEGA
TEAEVIEAARAANVHTFVSGLPEGYKTPVGERGVQLSGGQKQRIAIARAVLKDPSILLLDEATSALDAESECVL
QEALERLMRGRTTVLVAHRLSTIRNVDTIGVVQDGRIVEQGSHSELISRPEGAYSRLLQLQHHRI
>XP_021623402.1_ABCB19-l_Me
MAAESVDTTNNTNTKGSLPEAEKKKEQSLPFYQLFSFADKYDWLLMISGSIGAIIHGSSMPVFFLLFGEMVNGF
GKNQSDLEKMTHEVSKYALYFVYLGLVVCLSSYAEISCWMYTGERQVGTLRKKYLEAVLKQDVGFFDTDAR
TGDIVFSVSTDTLLVQDAISEKVGNFIHYLSTFLAGLVVGFVSAWRLALLSVAVIPGIAFAGGLYAYTLTGLTSK
SRESYAQAGIIAEQAIAQVRTVYSYVGESKALNSYSDAIQNTLKLGYKAGMAKGLGLGCTYGIACMSWALVF
WYAGVFIRNGQTDGGKAFTAIFSAIVGGMSLGQSFSNLGAFSKGKAAGYKLMEIIRQKPTIIQDPSDGKCLPEV
NGNIEFKDVTFSYPSRPDVIIFRDFSIFFPAGKTVAVVGGSGSGKSTVVSLIERFYDPNQGQVLLDNVDIKTLQLR
WLRDQIGLVNQEPALFATTILENILYGKPDATMDEVEAAASAANAHSFITLLPNGYNTQVGERGVQLSGGQKQ
RIAIARAMLKNPKILLLDEATSALDAGSESIVQEALDRLMVGRTTVVVAHRLSTIRNVDTIAVIQQGQVVETGT
HEELISKGGAYASLIRFQEMVRTRDFANPSTRRSRSTRLSHSLSTKSLSLRSGSLRNLSYSYSTGADGRIEMISNA
ETDRKNPAPDGYFCRLLKLNAPEWPYSIMGAIGSVLSGFIGPTFAIVMSNMIEVFYYRNPASMERKTKEYVFIYI
GAGLYAVVAYLIQHYFFSIMGENLTTRVRRMMLAAILRNEVGWFDEEEHNSSLVAARLATDAADVKSAIAERI
SVILQNMTSLLTSFIVAFIVEWRVSLLILATFPLLVLANFAQQLSLKGFAGDTAKAHAKTSMIAGEGVSNIRTVA
AFNAQDKISSLFSYELRVPQLRSLRRSQTSGLLFGLSQLALYASEALILWYGAHLVSKGSSTFSKVIKVFVVLVI
TANSVAETVSLAPEIIRGGEAVGSVFSILDRSTRIDADDPEADSVESIHGEIELRHVDFAYPSRPDVPVFKDLNLRI
RAGQSQALVGASGCGKSSVIALIERFYDPTAGKVMIDGKDIRRLNLKSLRLKIGLVQQEPALFAASIFDNIAYGK
EGATEAEVIEAARAANVHGFVSALPDGYKTPVGERGVQLSGGQKQRIAIARAVLKDPAILLLDEATSALDAES
ECVLQEALERLMRGRTTVLVAHRLSTIRGVDSIGVVQDGRIVEQGSHSELVSRPDGAYSRLLQLQHHHI
>XP_002323847.1_ABCB19_Pt
MAETTEANRPSLPEAEKKKEQSLPFYQLFSFADKYDWLLMISGSIGAIIHGSSMPVFFLLFGEMVNGFGKNQSD
LYKMTHEVSKYALYFVYLGIVVCLSSYAEIACWMYTGERQVSTLRKKYLEAVLKQDVGFFDTDARTGDIVFS
VSTDTLLVQDAISEKVGNFIHYLSTFLAGLVVGFVSAWRLALLSVAVIPGIAFAGGLYAYTLTGLTSKSRESYA
QAGIIAEQAIAQVRTVYSFVGESKALSSYTDAIQNTLKLGYKAGMAKGLGLGCTYGIACMSWALVFWYAGVF
IRNGQTDGGKAFTAIFSAIVGGMSLGQSFSNLGAFSKGKAAGYKLMEIIKQRPSITQDAVDGKCLAEVNGNIEF
KSVTFSYPSRPDVIIFRDFSIFFPAGKTVAVVGGSGSGKSTVVSLIERFYDPNQGQVLLDNVDIKTLQLRWLRDQI
GLVNQEPALFATTILENIRYGKPDATMDEVEAATSAANAHSFITLLPNGYNTQVGERGVQLSGGQKQRIAIARA
MLKNPKILLLDEATSALDASSESIVQEALDRLMIGRTTVVVAHRLSTIRNVDTIAVIQQGLVVETGTHEELIAKA
GAYASLIRFQEMVRNRDFANPSTRRSRSSRLSHSLSTKSLSLRSGSLRNLSYSYSTGADGRIEMISNAETDRKNP
APDGYFCRLLKLNAPEWPYSIMGAVGSVLSGFIGPTFAIVMSNMIEVFYYRNPASMERKTKEYVFIYIGAGLYA
VVAYLIQHYFFSIMGENLTTRVRRMMLAAILRNEVGWFDEEEHNSSLVAARLATDAADVKSAIAERISVILQN
MTSLLTSFIVAFIVEWRVSLLILATFPLLVLANFAQQLSLKGFAGDTAKAHAKTSMIAGEGVSNIRTVAAFNAQ
GKVLSLFCHELRVPQLHSLRRSQTSGLLFGLSQLALYGSEALILWYGAHLVSKGVSTFSKVIKVFVVLVITANS
VAETVSLAPEIIRGGEAVGSVFSILERSTKIDPDDSEAEPVESLRGEIELRHVDFAYPSRPDVPVFKDLNLRIRAG

QSQALVGASGCGKSSVISLIERFYDPMAGKVMIDGKDIRRLNLKSLRLKIGLVQQEPALFAASIFDNIAYGKDG
ATEAEVIEAARAANVHGFVSALPDGYKTPVGERGVQLSGGQKQRIAIARAVLKDPAILLLDEATSALDAESEC
VLQEALERLMRGRTTVLVAHRLSTIRGVDSIGVVQDGRIVEQGSHSELVSRPDGAYFRLLQLQHHHI

>XP_021593643.1_ABCB19_Me
MAAETVDTNTSSCKASLPEAEKKKEQTLPFFQLFSFADKYDWLLMISGSIGAIIHGSSMPVFFLLFGEMVNGFG
KNQSDLPKMTHEVSKYALYFVYLGIVVCLSSYAEIACWMYTGERQVGTLRKKYLEAVLKQDVGFFDTDART
GDIVFSVSTDTLLVQDAISEKVGNFIHYLSTFLAGLVVGFVSAWRLALLSVAVIPGIAFAGGLYAYTLTGLTSKS
RESYAQAGIIAEQAIAQVRTVYSYVGESKALNSYSEAIQNTLKLGYKAGMAKGLGLGCTYGIACMSWALVFW
YAGVFIRNGQTDGGKAFTAIFSAIVGGMSLGQSFSNLGAFSKGKAAGYKLMEIIKQKPTIIQDPSDGKCLSEFNG
NIEFKDVTFSYPSRPDVIIFRDFSIFFPAGKTVAVVGGSGSGKSTVVSLIERFYDPNQGQVLLDNVDIKTLQLRWL
RDQIGLVNQEPALFATTILENILYGKPDATMDEVEAAAASANAHSFITLLPNGYNTQVGERGVQLSGGQKQRIA
IARAMLKNPKILLLDEATSALDAGSESVVQEALDRLMVGRTTVVVAHRLSTIRNVDTIAVIQQGQVVETGTHE
ELIAKGGAYASLIRFQEMVRNRDFANPSTRRSRSSRLSHSLSTKSLSLRSGSLRNLSYSYSTGADGRIEMISNAET
DRKNPAPHGYFCRLLKLNAPEWPYSIMGAIGSVLSGFIGPTFALVMSNMIEVFYYGNPASMERKTKEYVFIYIG
AGLYAVVAYLIQHYFFSIMGENLTTRVRRMMLAAILRNEVGWFDEEEHNSSLVAARLATDAADVKSAIAERIS
VILQNMTSLLTSFIVAFIVEWRVSLLILATFPLLVLANFAQQLSLKGFAGDTAKAHARTSMIAGEGVSNIRTVAA
FNAQGKILSLFCHELRVPQLHSLRRSQTSGLLFGLSQLALYASEALILWYGAHLVSKGASTFSKVIKVFVVLVIT
ANSVAETVSLAPEIIRGGESVGSVFSILDRSTRIDPDDPEADPVESMRGEIELRHVDFAYPSRPDVPVFKDLSLRIR
AGQSQALVGASGCGKSSVIALIERFYDPTAGKVMIDGKDIRRLNLKSLRLKIGLVQQEPALFAASIFDNIAYGKD
GATEAEVIEAARAANVHGFVSALPDGYKTPVGERGVQLSGGQKQRIAIARAVLKDPTILLLDEATSALDAESEC
VLQEALERLMRGRTTVLVAHRLSTIRGVDSIGVVQDGRIVEQGSHSELVSRANGAYSRLLQLQHHQI
>XP_013450956.1_ABCB19_Mt
MAEASDVKASLPEAEKKKEQSLPFFQLFSFADKYDWILMISGSIGAIIHGSSMPVFFLLFGQMVNGFGKNQMDL
KKMTDEVSKYALYFVYLGLVVCISSYAEIACWMYTGERQVSTLRKKYLEAVLKQDVGFFDTDARTGDIVFSV
STDTLLVQDAISEKVGNFIHYLSTFLAGLVVGFVSAWRLALLSVAVIPGIAFAGGLYAYTLTGLTSKSRESYAN
AGIIAEQAIAQVRTVYSYVGESKALNSYSDAIQNTLKLGYKAGMAKGLGLGCTYGIACMSWALVFWYAGVFI
RNGQTDGGKAFTAIFSAIVGGMSLGQSFSNLGAFSKGKAAGYKLMEIIKQKPTIVEDLSDGKCLAEVNGNIEFK
DVSFSYPSRPDVMIFQNFSIFFPAGKTVAVVGGSGSGKSTVVSLIERFYDPNDGQVLLDNVDIKTLQLKWLRDQ
IGLVNQEPALFATTILENILYGKPDATMDEVESATSAANAHSFITLLPNGYNTQVGERGVQLSGGQKQRIAIAR
AMLKNPKILLLDEATSALDAGSESIVQEALDRLMVGRTTVVVAHRLSTIRNVDSIAVIQQRVVVETGTHEELFA
KGGTYASLIRFQEVVGNRDFSNPSTRRNRSSRLSHSLSTKSLSLRSGSLRNLSYQYSTGADGRIEMISNAETDKK
NPAPDGYFFRLLKMNAPEWPYSIMGAVGYVLSGFIGPTFAIVMSNMIEVFYYRNYASMEKKTKEYVFIYIGAGI
YAVGAYLIQHYFFSIMGENLTTRVRRMMLAAILRNEVGWFDEEEHNSSLVAARLATDAADVKSAIAERISVIL
QNMTSLLTSFIVAFIVEWRVSLLILGTFPLLVLANFAQQLSLKGFAGDTAKAHAKTSMIAGEGVSNIRTVAAFN
AQNKMLSIFCHELRVPQSQSLRRSLTSGLLFGLSQLALYASEALILWYGAHLVSKGLSTFSKVIKVFVVLVITAN
SVAETVSLAPEIIRGGEAVGSVFSILDRSTRIDPDDPDAEMVESVRGEIELRHVDFAYPSRPDMMVFKDFSLRIR
AGQSQALVGASGSGKSSVIALIERFYDPLVGKVMIDGKDIRRLNLKSLRLKIGLVQQEPALFASSIFDNIAYGKE
GATEAEVIEAARAANVHGFVSGLPEGYKTPVGERGVQLSGGQKQRIAIARAVLKDPAILLLDEATSALDAESEC
VLQEALERLMRGRTTVLVAHRLSTIRGVDCIGVVQDGRIVEQGSHSELISRPEGAYSRLLQLQHHHI
>XP_002283051.2_P_ABCB19_Vv
MAEGGAEAKALPEAEKKKEQSLPFYQLFSFADKYDWILMVSGSVGAVIHGSSMPVFFLLFGEMVNGFGKNQT
DLSKMTEEVAKYALYFVYLGVVVCISSYAEIACWMYTGERQVSTLRKKYLEAVLKQDVGFFDTDARTGDIVF
SVSTDTLLVQDAISEKVGNFIHYLSTFLAGLVVGFVSAWRLALLSVAVIPGIAFAGGLYAYTLTGLTSKSRESYA
NAGIIAEQAIAQVRTVYSYVGESKALNSYSDAIQNTLKLGYKAGMAKGLGLGCTYGIACMSWALVFWYAGVF
IRNGQTDGGKAFTAIFSAIVGGMSLGQSFSNLGAFSKGKAAGYKLMEIIRQKPSIVQDPSDGKCLAEVNGNIEFK
DVTFSYPSRPDVIIFRDFSIFFPAGKTVAVVGGSGSGKSTVVSLIERFYDPNQGQVLLDNVDIKTLQLRWLRDQI
GLVNQEPALFATTILENILYGKPDATAAEVEAAASAANAHSFITLLPNGYNTQVGERGTQLSGGQKQRIAIARA
MLKNPKILLLDEATSALDAGSESIVQEALDRLMVGRTTVVVAHRLSTIRNVDTIAVIQQGQVVETGTHEELSAK
AGAYASLIRFQEMVRNRDFANPSTRRSRSSRLSHSLSTKSLSLRSGSLRNLSYQYSTGADGRIEMVSNAETDKK
NPAPDGYFYRLLNLNAPEWPYSIMGAVGSVLSGFIGPTFAIVMSNMIEVFYYRNPASMERKTKEYVFIYIGAGL
YAVIAYLIQHYFFSIMGENLTTRVRRMMLAAILRNEVGWFDEEENNSSLLAARLATDAADVKSAIAERISVILQ
NMTSLLTSFIVAFIVEWRVSLLILATFPLLVLANFAQQLSLKGFAGDTAKAHAKTSMIAGEGVSNIRTVAAFNA
QEKILSLFCYELRVPQMQSLRRSQTSGLLFGLSQLALYASEALILWYGSHLVSKGASTFSKVIKVFVVLVITANS

VAETVSLAPEIIRGGEAVGSVFSILDRSTKIDPDDSDAEPVESIRGEIELRHVDFSYPSRSDITVFKDLNLRIRAGQ
SQALVGASGSGKSSVIALIERFYDPTAGKVMIDGKDVRRLNLKSLRLKIGLVQQEPALFAASILDNIAYGKDGA
TEAEVIEAARAANVHGFVSGLPDGYKTPVGERGVQLSGGQKQRIAIARAVLKDPTILLLDEATSALDAESECVL
QEALERLMRGRTTVLVAHRLSTIRGVDSIGVVQDGRIVEQGSHSELISRPEGAYSRLLQLQHHHI
>XP_003554410.1_ABCB19_Gm
MAEAAEPNKALPEAEKKKEQTLPFYKLFSFADKCDWMLMISGSIGAIIHGSSMPVFFLLFGEMVNGFGKNQMN
LKKMTEEVSKYALYFVYLGLVVCISSYAEIACWMYTGERQVSTLRKKYLEAVLKQDVGFFDTDARTGDIVFS
VSTDTLLVQDAISEKVGNFIHYLSTFLAGLVVGFVSAWRLALLSVAVIPGIAFAGGLYAYTLTGLTSKSRESYA
NAGIIAEQAIAQVRTVYSYVGESKALNSYSDAIQNTLKLGYKAGMAKGLGLGCTYGIACMSWALVFWYAGVF
IRNGQTDGGKAFTAIFSAIVGGMSLGQSFSNLGAFSKGKAAGYKLMEIINQKPTIVEDPSEGKCLAEVNGNIEFK
DVTFSYPSRPDMFIFRNFSIFFPAGKTVAVVGGSGSGKSTVVSLIERFYDPNEGQVLLDNVDIKTLQLKWLRDQI
GLVNQEPALFATTILENILYGKPDATMAEVEAATSAANAHSFITLLPNGYNTQVGERGVQLSGGQKQRIAIARA
MLKNPKILLLDEATSALDAGSENIVQEALDRLMVGRTTVVVAHRLSTIRNVDTIAVIQQGQVVETGAHEELIAK
AGTYASLIRFQEMVGNRDFSNPSTRRTRSSRLSHSLSTKSLSLRSGSLRNLSYQYSTGADGRIEMISNAETDKKN
PAPDGYFFRLLKMNAPEWPYSIMGAVGSVLSGFIGPTFAIVMSNMIEVFYFSNYASMERKTKEYVFIYIGAGLY
AVGAYLIQHYFFSIMGENLTTRVRRMMLAAILRNEVGWFDEEEHNSSLVAARLATDAADVKSAIAERISVILQ
NMTSLLTSFIVAFIVEWRVSLLILATFPLLVLANFAQQLSLKGFAGDTAKAHAKTSMIAGEGVSNIRTVAAFNA
QNKMLSVFCHELRVPQSQSLRRSLTSGFLFGLSQLALYASEALILWYGAHLVSKGVSTFSKVIKVFVVLVITAN
SVAETVSLAPEIIRGGEAVGSVFSILDRSTRIDPDDPDADPVESLRGEIELRHVDFAYPSRPDVMVFKDFNLRIRA
GQSQALVGASGSGKSSVIALIERFYDPIAGKVMVDGKDIRKLNLKSLRLKIGLVQQEPALFAASIFENIAYGKEG
ATEAEVIEAARAANVHGFVSGLPEGYKTPVGERGVQLSGGQKQRIAIARAVLKDPTILLLDEATSALDAESECV
LQEALERLMRGRTTVLVAHRLSTIRGVDCIGVVQDGRIVEQGSHSELVSRHEGAYSRLLQLQHHHI

>NP_001169660.1_P_Zm
MAEGDAGKAEAGSCSAAAASGAGGCDAVKKRPEQSVAFHELFGFADPLDWLLMAAGSAGAVVHGAAMPVF
FLLFGELVNGFGKNQHNLRRMTDEVSKYSLYFVYLGLVVCASSYLEIACWMYTGERQVGALRRRYLEAVLR
QDVGFFDTDARTGDVVFSVSTDTLLVQDAIGEKVGNFIHYLATFLAGLVVGFVSAWRLALLSIAVIPGIAFAGG
LYAYTLTGLTSKSRDSYANAGIIAEQAIAQVRTVYSYVGETKALNSYSEAIQNTLKLGYKAGMAKGLGIGCTY
GIACMSWALVFWYAGVFIRNGQTDGGKAFTAIFSAIVGGLSLGQSFSNLGAFSKGKIAGYKLLEVIRQRPTIVQ
DTADGRCLDEVHGNIEFKEVAFSYPSRPDVMIFRDFSLFFPAGKTAAVVGGSGSGKSTVVALIERFYDPNQGQV
LLDNVDIKTLQLKWLRDQIGLVNQEPALFATTILENILYGKPDATMAEVEAAATSANAHSFIALLPNGYNTHV
GDRGLQLSGGQKQRIAIARAMLKNPKLLLLDEATSALDAGSESIVQEALDRLMVGRTTVVVAHRLSTIRCVDM
IAVIQQGQVVETGTHDELLAKGSSGAYAALIRFQETARNRACPSTRKSRSSRLSNSLSTRSLSLRSGSLRNLSYS
YSTGADGRIEMVSNADNDRKYPAPRGYFFKLLKLNAPEWPYTILGAVGSVLSGFIGPTFAIVMSNMIEVFYYRN
PSKMESKTREYVFIYIGTGLYAVVAYLVQHYFFSIMGENLTTRVRRMMLAVILRNDVGWFDQEENNSNLVAA
RLSTDAADVKSAIAERISVILQNMTSLLVSFVVGFIIEWRVALLILVTFPLLVLANFAQQLSMKGFAGDTAKAH
AKTSMIAGEGVSNIRTVAAFNAQDKILSLFCSELRVPQMHSLRRSQISGALFGLSQLSLYASEALILWFGAHLVR
THVSTFSKVIKVFVVLVITANSVAETVSLAPEIVRGGESIRSVFSVLNSRTRIDPDDPDAEQVESVRGEIDFRHVD
FAYPTRPDVMVFKDLSLRIRAGQSQALVGASGSGKSTVIALVERFYDPLAGKVMIDGKDIRRLNLKSLRLRIGL
VQQEPVLFATSILENIAYGRDGATEEEVVEAAKVANVHGFVSALPDGYRTPVGERGVQLSGGQKQRIAIARAV
LKDPAVLLLDEATSALDAESECVLQEALERIMKGRTAVLVAHRLSTIRGVDSIAVVQDGRVVEQGSHGDLVSR
PDGAYSRLLQLQLHHG
>XP_002447959.1_ABCB19_Sb
MAEGDAGKAEAGSCSGAGAGGGCDAVKKRPEQSVAFHELFGFADPLDWLLMAAGSAGAVVHGAAMPVFFL
LFGELVNGFGKNQHNLRRMTDEVSKYSLYFVYLGLVVCASSYLEIACWMYTGERQVGALRRRYLEAVLRQD
VGFFDTDARTGDVVFSVSTDTLLVQDAIGEKVGNFIHYLATFLAGLVVGFVSAWRLALLSIAVIPGIAFAGGLY
AYTLTGLTSKSRDSYANAGIIAEQAIAQVRTVYSYVGETKALNSYSEAIQNTLKLGYKAGMAKGLGIGCTYGI
ACMSWALVFWYAGVFIRNGQTDGGKAFTAIFSAIVGGLSLGQSFSNLGAFSKGKIAGYKLLEVIRQRPTIVQDT
ADGRCLDEVHGNIEFKEVAFSYPSRPDVMIFRDFSLFFPAGKTAAVVGGSGSGKSTVVALIERFYDPNQGQVLL
DNVDIKTLQLKWLREQIGLVNQEPALFATTILENILYGKPDATMAEVEAAATSANAHSFIALLPNGYNTHVGER
GLQLSGGQKQRIAIARAMLKNPKLLLLDEATSALDAGSENIVQEALDRLMVGRTTVVVAHRLSTIRCVDMIAV
IQQGQVVETGTHDELLAKGSSGAYAALIRFQETARNRACPSTRKSRSSRLSNSLSTRSLSLRSGSLRNLSYSYST
GADGRIEMVSNADNDRKYPAPRGYFFKLLKLNAPEWPYTILGAIGSILSGFIGPTFAIVMSNMIEVFYYRNPNK
MESKTREYVFIYIGTGLYAVVAYLVQHYFFSIMGENLTTRVRRMMLAVILRNDVGWFDQEENNSNLVTARLS

TDAADVKSAIAERISVILQNMTSLLVSFVVGFIIEWRVALLILVTFPLLVLANFAQQLSMKGFAGDTAKAHAKT
SMIAGEGVSNIRTVAAFNAQDKILSLFCSELRVPQMHSLRRSQISGALFGASQLSLYASEALILWFGAHLVRTHV
STFSKVIKVFVVLVITANSVAETVSLAPEIVRGGESIRSVFAILNSRTRIDPDDPDAEQVESVRGEIDFRHVDFAYP
TRPDVMVFKDFSLRIRAGQSQALVGASGSGKSTVIALIERFYDPLAGKVMVDGKDIRRLNLKSLRLRIGLVQQE
PVLFAASILENIAYGRDGATEEEVVEAAKVANVHGFVSALPDGYRTPVGERGVQLSGGQKQRIAIARAVLKDP
AVLLLDEATSALDAESECVLQEALERIMKGRTAVLVAHRLSTIRGVDNIAVVQDGRVVEQGSHGDLVSRPDG
AYSRLLQLQLHHG
>XP_002448624.1_ABCB19_Sb
MAETAAADGKADKVANGGGGGGDAAGEGKKRGDQAVAFHELFSFADKWDLMLMAAGSLGALAHGAAMP
FFFLLFGDLINGFGKNQTDLRTMTDEVAKYALYFVYLGLVVCVSSYAEIACWMYTGERQVIALRKAYLDAVL
RQDVGFFDTDARTGDIVFGVSTDTLLVQDAIGEKVGNFMHYIATFLAGLVVGFVSAWRLALLSVAVIPAIAFA
GGLYAYTLTGLTSKSRESYANAGVVAEQAIAQVRTVYSFVGESKALNSYSEAIQNTLKLGYKAGMAKGLGIG
CTYGIACMSWALVFWYAGVFIRNGQTDGGKAFTAIFSAIVGGMSLGQAFSNLGAFSKGKIAGYKLLEVIRQKP
SIVNDHKDGKWLAEVHGNIEFKEVTFSYPSRPDVIIFRDFSLFFPAGKTVAVVGGSGSGKSTVVALIERFYDPNE
GQVLLDNVDIKTLQLRWLRDQIGLVNQEPALFATTILENILYGKPDATIAEVEAAATASNAHGFISLLPNGYNT
MVGERGIQLSGGQKQRIAIARAMLKNPKILLLDEATSALDADSESIVQEALDRLMVGRTTVVVAHRLSTIRNV
NMIAVIQQGQVVETGTHDELLAKGTSGAYASLVRFQETARNRDLAGASTRRSRSIHLTSSLSTKSLSLRSGSLR
NLSYQYSTGADGRIEMISNADNDRKYPAPRGYFFKLLKLNAPEWPYAVLGAIGSVLSGFIGPTFAIVMGEMLD
VFYYRDPNEMEKKTKLYVFIYIGTGIYAVVAYLVQHYFFSIMGENLTTRVRRMMLSAILRNEVGWFDEEENNS
SLVAARLAVDAADVKSAIAERISVILQNMTSLMTSFVVGFIIEWRVAILILATFPLLVLANFAQQLSMKGFAGDT
AKAHAKSSMVAGEGVSNIRTVAAFNAQSKILSLFSHELRVPEQQILRRSQTSGLLFGLSQLCLYSSEALILWYGS
HLVRSHGSTFSKVIKVFVVLVVTANSVAETVSLAPEIIRGGESIRSIFGILNRATRIEPDDPESERVTTIRGDIELRH
VDFSYPARPDIQIFKDFNLKIHAGRSQALVGASGSGKSTVIALIERFYDPCGGKVAIDGKDIRTLNLKSLRLKIGL
VQQEPVLFASSILENIAYGKEGATEEEVIEAAKTANVHGFVSQLPDGYKTAVGERGMQLSGGQKQRIAIARAV
LKDPAILLLDEATSALDAESECVLQEALERLMKGRTTVLVAHRLSTIRGVDRIAVVQDGRIVEHGSHNDLLARP
EGAYSRLLQLQHHRV
>XP_015636531.1_ABCB19_Os
MAESAAAAAGDGKVEKAANGGVNGCDAAGEGKKRADQAVAFHELFTFADKWDLVLMAAGSLGALAHGA
AMPLFFLLFGDLINGFGKNQTDLRTMTDEVSKYALYFVYLGLVVCASSYAEIACWMYTGERQVIALRKAYLD
AVLRQDVGFFDTDARTGDIVFGVSTDTLLVQDAIGEKVGNFIHYIATFLAGLVVGFVAAWRLALLSVAVIPAIA
FAGGLYAYTLTGLTSKSRESYANAGVVAEQAIAQVRTVYSFAGESKALNSYSEAIQNTLKLGYKAGMAKGLG
IGCTYGIACMSWALVFWYAGVFIRNGQTDGGKAFTAIFSAIVGGMSLGQAFSNLGAFSKGKIAGYKLLEVIRQ
KPSIVHDHKDGKLLAEVHGNIEFKDVTFSYPSRPDVMIFRDFSLFFPAAKTVAVVGGSGSGKSTVVALIERFYD
PNEGQVLLDNVDIKTLQLRWLRDQIGLVNQEPALFATTIHENILYGKPDATMAEVEAAATASNAHSFISTLPNG
YNTMVGERGIQLSGGQKQRIAIARAMLKNPKILLLDEATSALDAGSENIVQEALDRLMTGRTTVVVAHRLSTIR
NVNMIAVIQQGQVVETGTHDELLAKGSSGAYASLIRFQEMAQNRDLGGASTRRSRSMHLTSSLSTKSLSLRSG
SLRNLSYQYSTGANGRIEMISNADNDRKYPAPRGYFFKLLKLNAPEWPYAVLGAVGSVLSGFIGPTFAIVMGE
MLDVFYYRDPNEMEKKTKLYVFIYIGTGLYAVVAYLVQHYFFSIMGENLTTRVRRMMLSAILTNEVGWFDEE
ENNSSLVAARLAVDAADVKSAIAERISVILQNMTSLMTSFIVGFIIEWRVALLILATFPLLVLANFAQQLSMKGF
AGDTAKAHAKSSMVAGEGVSNIRTVAAFNAQNKILSLFSYELRIPEQQILRRSQTSGLLFGLSQLCLYSSEALIL
WYGSHLVRSHGSTFSKVIKVFVVLVVTANSVAETVSLAPEIVRGGESIRSIFGILNRATRIEPDDPESERVTNVRG
DIELRHVDFAYPARPDIQIFKDFNLKIQAGRSQALVGASGSGKSTVIALIERFYDPTGGKVTIDGKDIRRLNLKAL
RLKIGLVQQEPVLFAASILENIAYGKDGATEEEVIQAAKTANVHGFVSQLPNGYKTAVGERGVQLSGGQKQRI
AIARAVLKDPAILLLDEATSALDAESECVLQEALERLMKGRTTVLVAHRLSTIRGVDRIAVVQDGRIVEHGSHS
DLVSRPEGAYSRLLQLQHHA
>NP_001309742.1_ABCB19-l_Zm
MAESAATDGKADKVANGGGGGGDAAGEGKKRGDQAVAFHELFSFADKWDLMLMAAGSLGALAHGAAMP
FFFLLFGDLINGFGKNQTDLRTMTDEVAKYALYFVYLGLVVCVSSYAEIACWMYTGERQVIALRKAYLDAVL
RQDVGFFDTDARTGDIVFGVSTDTLLVQDAIGEKVGNFMHYIATFLAGLVVGFVSAWRLALLSVAVIPAIAFA
GGLYAYTLTGLTSKSRESYTNAGVVAEQAIAQVRTVYSFVGESKALNSYSEAIQNTLKLGYKAGMAKGLGIG
CTYGIACMSWALVFWYAGVFIRNGQSDGGKAFTAIFSAIVGGMSLGQAFSNLGAFSKGKIAGYKLLEVIRQKP
SIVNDHKDGKWLAEVHGNIEFKEVTFSYPSRPDVIIFRDFSLFFPAGKTVAVVGGSGSGKSTVVALIERFYDPNE
GQVLLDNVDIKTLQLRWLRDQIGLVNQEPALFATTILENILYGKPDATIAEVEAATTASNAHSFISLLPNGYNT
MVGERGIQLSGGQKQRIAIARAMLKNPKILLLDEATSALDADSESIVQEALDRLMVGRTTVVVAHRLSTIRNV

NMIAVIQQGQVVETGTHDELLAKGTSGAYASLIRFQETARNRDLGGASSRRSRSIHLTSSLSTKSLSLRSGSLRN
LSYQYSTGADGRIEMISNADNDRKYPAPRGYFFKLLKLNAPEWPYAVLGAIGSVLSGFIGPTFAIVMGEMLDVF
YYRDPNEMEKKTKLYVFIYIGTGIYAVVAYLVQHYFFSIMGENLTTRVRRMMLSAILRNEVGWFDEEENNSSL
VAAHLAVDAADVKSAIAERISVILQNMTSLMTSFVVGFIIEWRVAILILATFPLLVLANFAQQLSMKGFAGDTA
KAHAKSSMVAGEGVSNIRTVAAFNAQSKILSLFSHELRVPEQQILRRSQTSGLLFGLSQLCLYSSEALILWYGSH
LVRSHGSTFSKVIKVFVVLVVTANSVAETVSLAPEIIRGGESIRSIFGILNRATRIEPDDPESERVTTIRGDIELRHV
DFSYPARPDIQIFKDFNLKIQAGRSQALVGASGSGKSTIIALIERFYDPCGGKVAIDGKDIRTLNLKSLRRKIGLV
QQEPVLFASSILENIAYGKEGASEEEVVEAAKTANVHGFVSQLPDGYRTAVGERGMQLSGGQKQRIAIARAVL
KDPAILLLDEATSALDAESECVLQEALERLMKGRTTVLVAHRLSTIRGVDRIAVVQDGRVVEHGSHSDLLARP
EGAYSRLLQLQHHRV
>XP_008668793.1_ABCB19_Zm
MAESAAADGKSDKVANGGGGGGDAAGEGKKRGDQAVAFHELFSFADKWDLMLMAAGSMGALAHGAAMP
FFFLLFGDLINGFGKNQTDLRTMTDEVAKYALYFVYLGLVVCVSSYAEIACWMYTGERQVIALRKAYLDAVL
RQDVGFFDTDARTGDIVFGVSTDTLLVQDGIGEKVGNFMHYIATFLAGLVVGFVSAWRLALLSVAVIPAIAFA
GGLYAYTLTGLTSKSRESYANAGVVAEQAIGQVRTVYSFVGESKALNSYSEAIQNTLKLGYKAGMAKGLGIG
CTYGIACMSWALVFWYAGVFIRNGQTDGGKAFTAIFSAIVGGMSLGQAFSNLGAFSKGKIAGYKLLEVIRQKP
SIVNDHKDGKWLAEVHGNIEFKEVTFSYPSRPDVIIFRDFSLFFPAGKTVAVVGGSGSGKSTVVALIERFYDPNE
GQVLLDNVDIKTLQLRWLREQIGLVNQEPALFATTILENILYGKPDATIAEVEAAATASNAHSFISLLPNGYNT
MAGERGIQLSGGQKQRIAIARAMLKNPKILLLDEATSALDADSESIVQEALDRLMVGRTTVVVAHRLSTIRNV
NMIAVIQQGQVVETGTHDELIAKGTSGAYASLVRFQETARNRDLGGASSRRSRSIHLTSSLSTKSLSLRSGSLKN
LSYQYSTGADGRIEMISNADNDRKYPAPRGYFFKLLKLNAPEWPYAVLGAIGSVLSGFIGPTFAIVMGEMLDVF
YYRDPNEIEKKTKLYVFIYIGTGIYAVVAYLVQHYFFSIMGENLTTRVRRMMLSAILRNEVGWFDEEENNSSLV
AARLGVDAADVKSAIAERISVILQNMTSLMTSFVVGFIIEWRVAILILATFPLLVLANFAQQLSMKGFAGDTAK
AHARSSMVAGEAVSNIRTVAAFNAQSKILSLFSHELRVPEQQILRRSQTSGLLFGLSQLCLYSSEALILWYGSHL
VRSHGSTFSKVIKVFVVLVVTANSVAETVSLAPEIIRGGESIRSIFGILNRATRIEPDDPESERVTTIRGDIELRHVD
FSYPARPDIQIFKDFNLKIQAGRSQALVGASGSGKSTVIALIERFYDPCGGKVAIDGKDIRTLNLKSLRLKIGLVQ
QEPVLFASSILENIAYGKEGASEEEVVEAAKTANVHGFVSQLPDGYRTAVGEQGMQLSGGQKQRIAIARAVLK
DPAILLLDEATSALDAESECVLQEALERLMKGRTTVLVAHRLSTIRGVDRIAVVQDGRVVEHGSHSDLLARPE
GAYLRLLQLQHHRV

>XP_024460874.1_ABCB9_Pt
MREDTEGASTNSIANGQKTTNGEDQKVAFHKLFTFADRLDVVLMIVGTLSAIANGLAQPLMTLIFGQLINSFGS
SDRSNVVKEVSKVALNFVYLAIGSGIASLLQVSSWMVTGERQSTRIRSLYLKTILRQDIGFFDSETSTGEVIGRM
SGDTILIQDAMGEKVGKFIQLLATFFGGFAIGFIKGWLLALVLLSSIPPLVIAGGVMALIMTKMSSRGQVAYAEA
GNIVEQTVGAIRTVASFTGEKHAIEKYNSKLKIAYNSAAQQGLASGLGLGTMLFIVFGTYALAIWYGSKLIVEK
GYNGGQVMTVIISIMTGGMSLGQTSPCLNAFASGQAAAYKMFETIERKPKIDPYDTSGMVVEDLDGEIELRDV
YFRYPARPEVQIFSGFSLQVPSGTTTALVGQSGSGKSTVISLVERFYDPDSGEVLIDGVDLKKLKLSWIREKIGL
VSQEPILFATSIKENIAYGKENATDQEIRTAIQLANAAKFIDKMPEGLDTMVGEHGTQLSGGQKQRIAIARAILK
NPKILLLDEATSALDAESERIVQDALVKIMCNRTTLVVAHRLTTIRNADMIAVVHLGKIVEKGSHEELTKDPEG
AYSQLIRLQGGAMDSEESQDIDADMSRSSLDRDRSISSPRSQKHSVQGSISRGSSGSRRSFTLNTVGFGMPGPTS
VHDDEFEQNNERNVKPKEVSIKRLAYLNKPELPVLFLGTVAAVIHGVIFPVFGLLLSKAINMFYEPPKEIRKDSK
FWAVLYLGLGFITFAALPLQYYLFGIAGGKLIERIRSKTFEKVVHQEISWFDDPTNSSGAIGARLSTDASTVRRL
VGDSLSLIVQNISTILSALVIAFSANWMLTLIIIAISPLLFIQGYMQAKFMKGFSADSKMMYEQASQVANDAVGS
IRTVASFCAEKKVMELYQKKCEGPTKQGVRLGFVSGIGYGLSFFILYCTNAFCFYIGAIFVQNGKTTFADVFRV
FFALTIGALGVSQSSGLAPDTAKAKDSAASIFAILDRKPKIDSSRDEGLTLPHVNGDIEIEHVSFKYPMRPHVQIF
RDMSLSIPSGKTVALVGESGSGKSTVISLIERFYDPDSGHVYLDSVEIKKFKLNWLRQQMGLVSQEPILFNETIR
ANIAYGKHGEIAEEEIIEATRASNAHNFISTLPQGYDTKVGERGIQLSGGQKQRIAIARAILKNPKILLLDEATSA
LDAESERIVQEALDRVMVNRTTVVVAHRLATIKGADVIAVVKNGAIAEKGKHDVLMKITDGAYASLVALHMS
AT
>XP_006355579.1_P_ABCB9-l_St
MEDNNNGEKKGDEDQKVSFYKLFSFADKFDVALMIIGTIGAIGNGLTQPLMTLIFGQLVNSFGSSNSDEVVHKI
SKVSIDYVYLAIGAGVASLLQMSCWMVTGERQATRIRGLYLKTILRQDIAFFDTETTTGEVIGRMSGDTILIQDA
LGEKVGKFIQFISTFVGGFVVAFFKGWLLSIVLVSCIPALVIAGGAMALIMSKMSSRGQVAYAQAGNVVEQTIG

AIRTVSAFTGEKLAIDKYDSKLKIACASTVQQGLVSGVGLGTVLLIVFSTYGLAVWYGSKLIIERGYNGGDVIN
VIMAIMTGGMSLGQTTPSLNAFAAGQAAAYKMFETINRKPLIDTSDTNGVVLENIKGEIELKDVYFRYPARPDV
QIFSGFSLIVPNGKTVALVGQSGSGKSTVISLLERFYDPEAGEVLIDGVNLKKFQLKWLRQQMGLVSQEPILFAT
TIKENISYGKENATEDEIKTAIELANAAKFLDKLPQGLDTMVGEHGTQLSGGQKQRLAIARAILKNPRILLLDEA
TSALDAESERIVQEALEKVMANRTTVVVAHRLTTIRNADLIAVVNAGKLIEKGTHTELIQDPNGAYSQLVRMQ
GGNREEENMKNMDLEKVDLTTDLDNNLSRSSSQQLSAMRRSTSQGSSRHSFTLNYTVPGLVGIHEAEIGDEDK
QKEDKGSLKKRKNVSIRRLAGLNKPELPYLLLGSLAAIIHGLIFPLFGLLLSTAIKIFFYPPQKLRSESRFWALMY
FGLGVVTLLVVPFQNYLFGVAGGKLIERIRSLTFKKVVHQEISWFDDPAHSSGAIGARLSTDASTVRTLMGDAL
ALIVQNIATVVAGLVIAFTANWILALIILLVMPLIGVQGFLQTKMYKGFSADAKVMYEEASQIANDAVGSIRTV
ASFCAEEKVMDMYQKKCEGPMKQGVKIGIVSGASLGFGSFILYCTNAFCFYIGSILIQHGLASFGQVFKVFFAL
TLSAVGVTQSTGMAPDASKAKDSIASIFDILDRKPEIDSSSDVGTTLAAVRGDIEFKHVSYRYATRPDVQIFKDL
CLTIPSGKTVALVGESGSGKSTVISLIERFYNPESGSIYLDGVEIRQFKLSWLRQQMGLVSQEPVLFNETIRDNIA
YSRQGHATEEEIIEAAKSANAHNFISSLPQGYDTSVGERGIQLSGGQKQRIAIARAILKDPKILLLDEATSALDAE
SERIVQEALDRVMVNRTTVVVAHRLTTIKGADVIAVVKNGVIAEEGRHDALMNIKDGVYASLVALHMTSA
>XP_004233862.2_ABCB9-l_Sl
MEDNNNNGEKKRDEDQKVSFYKLFSFADKFDIALMIIGTIGAIGNGLTQPLMTLIFGQLVNSFGSSNSDEVVHEI
SKVSIYYVYLAIGAGVASLLQMSCWMVTGERQATRIRGLYLKTILRQDIAFFDTETTTGEVIGRMSGDTILIQDA
LGEKVGKFIQFISTFVGGFIVAFFKGWLLSIVLVSCIPALVIAGGAMALIMSKMSSRGQVAYAQAGNVVEQTIG
AIRTVSAFTGEKLAIDKYDSKLKIACASTVQQGLVSGIGLGTVLLIVFSTYGLAVWYGSKLIIERGYNGGDVINV
IMAIMTGGMSLGQTTPSLNAFAAGQAAAYKMFETINRKPLIDTSDTSGVVLENIKGEIELKDVYFKYPARPDVQ
IFSGFSLVVPSGKTVALVGQSGSGKSTVISLLERFYDPEAGEVLIDGVNLKKFQLKWLRQQMGLVSQEPILFATT
IKENISYGKENATEDEIKTAIELANAAKFLDKLPQGLDTMVGEHGTQLSGGQKQRLAIARAILKNPRILLLDEAT
SALDAESERIVQEALEKVMANRTTVVVAHRLTTIRNADLIAVVNAGKLLEKGTHTELIQDPNGAYSQLVRMQ
GGNREEENMKNIDLEKVDLTTDFDNNLSRSSSQRLSAMRRSTSQGSSRHSFTLNYTVPGLIGIHEAEIGNENKG
KEDKGSSKKRKKVSIRRLAGLNKPELPYLLLGSLAAIIHGLIFPLFGLLLSTAIKIFFYPPQKLRIESRFWALMYFG
LGVVTLLVVPFQNYLFGVAGGKLIERIRSLTFKKVVHQEISWFDDPAHSSGAIGARLSTDASTVRTLMGDALAL
IVQNIATVVAGLVIAFTANWILALIILLVMPLIGVQGFLQTKMYKGFSADAKVMYEEASQIANDAVGSIRTVAS
FCAEEKVMDMYQKKCEGPMKQGVKIGIVSGASLGFGSFILYCTNAFCFYIGSVLIQHGLASFGQVFKVFFALTL
SAVGVTQSTGMAPDANKAKDSIASIFDILDRKPEIDSSSDVGTTLAAVRGDIEFKHVSYRYATRPDVQIFKDLCL
TIPSGKTVALVGESGSGKSTVISLIERFYNPESGSIYLDGVEIRQFKISWLRQQMGLVSQEPVLFNETIRDNIAYSR
QGHATEEEIIEAAKSANAHNFISSLPQGYDTSVGERGIQLSGGQKQRIAIARAILKDPKILLLDEATSALDAESERI
VQEALDRVMVNRTTVVVAHRLTTIKGADVIAVVKNGVIAEEGRHDALMNIKDGVYASLVALHMTSA
>XP_021598602.1_ABCB9_Me
MERDEETATTSQSGGATESKGEDQKVPLYKLFSFADKIDVVLMIVGTVAATANGLSQPLMALIFGQLINSFGSA
DQSSVVHHISKISLRMVFLAIGSGVASLLQVSSWMVTGERQSARIRSLYLKTILRQDIAFFDTETTTGEVIGRMS
GDTVLIQDAMGEKVGKFIQLASTFLGGFVIAFARGWLLALVLLSCIPLLVLVGGFMAILMSKMSSRGQIAYAK
AGNVVEQTIGAIRTVASFTGEKHAIKNYNEKIEIAYKATVQQAVASGLGIGTMLLVIFCTYALAVWYGSKLIMS
KNYNGGQVITVIMSIMTGGMSLGQTSPCLNAFAAGQAAAYKMFETINRVPTIDSYDTSGTVLENINGEIELRDV
YFRYPARPDIQIFNGFSLHIPSGKTAALVGHSGSGKSTVISLVERFYDPDSGEVLIDGVDLKRLRLSWIREQIGLV
SQEPILFATSIKENIAYGKENATDQEIKTAIELANASKFINQMPEGLKTMVGEHGTQLSGGQKQRIAIARAILKNP
KILLLDEATSALDAESERIVQNALDNVMTNRTTVVVAHRLTTVRNADIIAVVHLGKLVEKGTHEELIQNQEGA
YSQLVHLQEGAKESEHSQQVDAEASEIEKSMSRSGSQKMRRSLSRSLSRASSGSRHSFTINTLGLGLLTDVNIHE
TEEQEENIAEEKQQQTIQVPMKRLAYLNKPELPILLVGTIAAAVHGTIFPVFGIVLSTAIKVFYEPPVKLKRDSKF
WAVIYICIGFVALISLSVQNYFFGIAGAKLIQRIRSMTFERVVHQEISWFDDPANSSGAVGARLSTDASTVRSIVG
DALALVVQNVTTILAALIIAFTANWILALIILAISPLLLIQGFIQAKFMKGFSADANLMYEEASQVANDAVGSIRT
VASFCAEKKVMELYQKKCDGPVKKGVRLGLVSGAGFGFSFFILYCTNAFCFYIGSILVQNGKATFVEVFKVFFS
LTIAAVGVSQSSAMSPDSSKAKDSAASIFSIIDRKSKIDSSSDAGTTLAHIRGDIELEHISFKYPLRPHVEIFRDLSL
SIPSGKTVALVGESGSGKSTVIGLLERFYDPDSGRVFIDNVEIKQFKLSWLRQQMGLVGQEPILFNETIRANIAY
GKQGDTTEEEIIAATKAANAHNFISSLPQGYETSVGERGTQLSGGQKQRIAIARAIVKNPKILLLDEATSALDAE
SERVVQDALDKVMVSRTTVVVAHRLTTIKGADIIAVVKNGVIAEKGKHEALMHIANGAYASLVALHMSST

>XP_003593841.3_ABCB9_Mt
MIIGTISAVANGMTQPIMTLILGKIINTFGSIDPHHIVKEVSKVSLLFIYLAAGSGIVSFLQVSCWMVTGERQSARI
RSLYLKTILKQDIAFFDTETNTGEVIGRMSGDTILIQDAMGEKVGKFIQLAATFFGGFAVAFIKGWRLAVVLVA

CIPCVVVVGGFMSMLMAKMSSRGQAAYSEAGNVVDQTVGAIRTVASFTGEKKAIENYNSKLKVAYTTTVQQ
GIASGLGMGTLSLIVFSTYGLAMWYGSKLVLEKGYTGGIVMVVIIALMTGGMSLGQTSPCLDAFAAGQAAAY
KMFETIKRKPKIDAYDTSGTVLKDINGDIELKDVYFSYPARPDVQIFDGFSLFVPSGTTTALVGQSGSGKSTVISL
LERFYDPDAGEVLIDGVNLKNLQLKWIREQIGLVSQEPILFTTTIRENIAYGKEGATDEEITTAITLANAKNFIDK
LPQGLDTMAGQNGTQLSGGQKQRIAIARAILKNPRILLLDEATSALDAESERVVQEALEKVMTQRTTVVVAHR
LTTIRNADLIAVVHQGKIVEKGAHDELIKDDDGAYSQLIRLQEGEKENQKSEADNSSHIFNSEMSRSSNRRISLV
KSISQRSSGRHSQSNIFPLPHESGVQTDEPNIEEGQLDNKKKHKNVSIRRLAYLNKPEVPVLLLGSIAAIVNGAVF
PVFGLVFSSAITMFYEPPKQQRKDARLWSLLYVGLGLVTLVILPLQNYFFGIAGGKLVERIRSLTFAKVVHQEIS
WFDDPANSSGAVGARLSTDASTVKSLVGDTLALIVQNLSTITAGLILAFTSNWILAFIVLAVSPVVLIQGIIQMQF
LKGFSGDAKVMYEEASQVANDAVGSIRTVASFNAESKVMDMYQKKCSGPEKQGVHSGLVSGAGFGFSFVAL
YCMSAFCFYIGSVLVQHGKATFQEVFKVFFSLTITAVGISQSSTLAPDTNKAKDSAASIFEILDSNPTIDSSSNEG
VTLETVTGDIELQHVSFNYPTRPHIQIFKDLCLYIPAGKTVALVGESGSGKSTVISLLERFYNPDSGRILLDGVDI
KTFRLSWLRQQMGLVGQEPILFNESIRANIAYGKEGGAMEDEIIAAAKAANAHNFISSLPNGYDTSVGERGTQL
SGGQKQRIAIARAMLKNPKILLLDEATSALDAESERIVQEALDRVSVNRTTVVVAHRLTTIRGADTIAVIKNGV
VAEKGRHEVLMKITDGVYASLVALHSSAS
>XP_024631391.1_ABCB9_Mt
MIIGFICAVANGLSQPLMTLIFGKLINTFGSTDPSHIVKEVSKVALLFIYLGVGSGIASFLQVACWMVTGERQAA
RIRGLYLKTILKQDISYFDTEATSGEVIGRMSGDTILIQDAMGEKVGKFIQLISSFLGGFVIAFTKGWELTLVLLA
CIPCIVIVGGFMSMMMAKMSSRGQIAYSEAGVVVEQTVGAIRTVASFTGEEKATEKYNNKLRIAYKSTVQQGL
ASGTGMGLLLLIIFGTYALAMWYGSKLIIEKGYDGGSVFNIIIAINTGGMSLGQTTPCINAFATGQVAACKMFET
IKRKPNIDAYDTSGVIMENIKGDIELKDVYFRYPARPDVQIFAGFSFYIPSGTTAALVGQSGSGKSTIISLLERFYD
PEAGEVLIDGVNLKNFQVKWIREQIGLVGQEPVLFTASIKDNIAYGKEGATDEEIATAITLANAKKFIDKLPQGL
DSMVGGHGTQLSGGQKQRIAIARAILKNPRILLLDEATSALDAESERVVQEALEKVMTQRTTVVVAHRLTTIR
NADTIAVVHQGKIVEKGTHDELVKDPCGAYSQLISLQKGAKEAERSNSSEEDKSRNSFNLDTQRTSFARSISQG
SSGSRHSLSLGLTLPYQISGHEYVEGTNGDDESSELDNVKRQKVSVKRLAKLNKPEVPVILLGSIAAAVHGVTL
PIFGLLLSSCIKSFYKPAEQLRKDSEFWSLLFLGLGFVTLVALPVQNYLFGIAGGKLVERIRSLTFKKVVHQEISW
FDHPSNSSGAVSARLATDASTVRTLVGDTLALIVQNIATVAAGLVIAFSANWILSFIILAVSPLMLIQGYIQTKFL
KGFSADAKVEASQVANDAVGSIRTVASFCAEQKVMDMYQKKCSAPEKQGVRLGLVSGIGFGFSFFALYCTNA
FCFYIGSVLMQHGKATFGEVFKVFFCLTITAIGVSQTSALAPDTNKAKDSTASIFEILDSKPTIDSSSNEGATLET
VKGDFELQKVSFRYPTRPNIQIFKDLCLSIPAGKTVALVGESGSGKSTVISLLERFYNPDSGHILLDGLNIKTFKL
SWLRQQMGLVGQEPILFNESIRANIAYGKEGGATEDEIIAAANAANAHNFISSLPGGYNTSVGERGTQLSGGQK
QRIAIARAILKNPRILLLDEATSALDAESERVVQEALDRVSVNRTTVVVAHRLATIKGADIIAVVKNGVIAEKGR
HDLLMKIDGGIYASLVALHISAS
>XP_025981469.1_ABCB9_Gm
MAHNTEVPPSTSSQPHERDKANQKVPFYKLFTLADRLDVALITIGTIGAMANGCSQPLMTLILGKIINTFGSADP
SNTIKEVSNVALLFVYLAIATGIASFLRKITVVLLNGAEVACWMVTGERQAARIRGLYLKTILKQDIAFFDTETT
TGEVIGRMSGDTILIQDAMGEKVGKFIQLASTFIGGFVIGFVRGWRLALVLLACIPCVVLIGGALSMVMTKMAS
RGQAAYAEAGNVVEQTVGAIRTVASFTGEKKAIEKYNTKLNVAYKTMIQQGLASGLGMGALLLTIFCTYALA
MWYGSKLVIEKGYNGGTVITVIVALMTGGMSLGQTSPSLNAFAAGQAAAYKMFETIARKPKIDAYDTNGVVL
EDIKGDIELKNVHFRYPARPDVQIFSGFSLYVPSGTTAALVGQSGSGKSTVISLLERFYDPDAGEVLIDGVNLKN
FQVRWIREQIGLVSQEPVLFATSIRENIAYGKEGATNEEVTTAIKLANAKKFIDKLPQGLETMAGQNGTQLSGG
QKQRIAIARAILKNPRILLLDEATSALDAESEHVVQAALEQAMSKRTTVVVAHRLTTIRNADTIAVVHEGRIVE
QGTHDELIKDVDGAYFQLIRLQKGAKEAEGSHNSEAERSSSSFNLDIHMARSSTQRAVSISRGSSGRHSQSHSFS
LSHQSGVHESGERAGGDAEKPRKVSLRRLAYLNKPEVLVLVLGSIAAIVQGVVFPMFGFLFSSAIAMFYEPPEK
QRKDSSFWALLYVGLGIVTLVIIPVQNYFFGIAGGKLIERIRLLTFKKVVHQEISWFDDPANSSGAVGARLSTDA
STVKSLVGDTLALIVQNISTITAGLVISFTANWILALIIVAVSPLIFIQGVLQMKFLKGFSGDAKAKYEEASQVAN
DAVGSIRTIASFCAESKVMDMYRKKCLEPEKQGVRLGLVSGTGFGFSFLALYCTNAFCFYIGSVLVQHGKATFP
EVFKVFFCLTITAIGISQTSVLAPDTNKAKDSAASIFKILDSKPTIDSSSNEGRTLEAVSGDIELQHVSFNYPTRPHI
QIFKDLCLSIPAGKTVALVGESGSGKSTVISLLERFYNPDSGHILLDGVDIKEFRLSWLRQQMGLVGQEPILFNES
IRANIAYGKEGGATEAEIIAAAEAANAQEFISSLPNGYDTNVGERGTQLSGGQKQRIAIARAMLKDPKILLLDEA
TSALDAESERVVEEALDKVSVDRTTVVVAHRLTTIRDADLIAVMKNGAVAERGRHDALMKITDGVYASLVAL
HMSAA

>XP_019078066.1_P_ABCB9-l_Vv

MNKDGGETTAKRLDQQKVTLYKLFSFADQSDVVLMTVGTISGMANGCSRPLMTVMLGKTINKFGSTDQSQIV
HELSKICLVLLYLAVASGIAGFLRCNRYSTLINTSEYFTETSSWMVTGARQANRIRSLYLDTILRQDIGFFDTETT
TGEVIGRMSGDTILIQDAMGEKVGKFIQLVSNFIGAFVFAFIIGWRLTLVLLPTVPLIIIAGAAMAAVISKMSSYG
QVAYAEAGNVVEQTIGAIRTVAAFTGEKHAMEKYNRRLKVAYAATVKQGLASGFGVGVALLIVFLSYALAIW
YGSKLIIEKGYDGGKIVNVLFCVIGGGMALGQASPCLSAFGAGQAAAYKMFETIKRKPKINAYDTNGVVLEEI
MGEIELKDVYFKYPARPEVQIFSGFSLNIPSGTTAALVGQSGSGKSTVISLLERFYDPEAGEVLIDGVNLKKINLR
WIRGKIGLVSQEPILFAATIKENISYGKEKATDEEIRTAIKLANAAKFIDKMPTGLDTMVGEHGTQLSGGQKQRI
AIARAILKNPRILLLDEATSALDAESERIVQDALQNIMVNRTTVIVAHRLTTIRNADNIAVVHQGKIVEQGTHME
LIRDPDGAYSQLVRLQEGHNQVEDAQSRVSKSSARDNARRSSRSRSLSSQISIISRDSPSVHHSYSLSSGIPDPTGI
IEMEFGGKESSTTQGEAENRKRRKVSLIRLAYLNKPETPVLLLGSIAAGFHGIIYPVFGLLISTAIKIFYEPPNELK
KDSRVWAFMFIGLGVLAFIALPLQNYLFGIAGGKLIQRICSLSFEKVVHQEISWFDDPANSSGSVGARLSTDAST
VRSLVGDTLALVVQNLVTVAAGLVISFTANWILALIILAVLPLMGFQGYLQTRFLKGFSADAKVMYEEASQVA
NDAVSSIRTVASFCAEKKVMEMYQQKCEGPMKHGVRLGLVSGAGLGFSFFSTYCTNAFCFYIGAVLVQHGKA
TFSEVFKVYFALTFLALAISEATAMAPDTNKAKDSTASIFELLDSKPKIDSSSNEGTTLSIVKGDIELQNVSFRYS
TRPDVQIFRDLCLSIPSGKTVALVGESGSGKSTVISLLERFYNPDSGHILLDGMEIQKFKLSWLRQQMGLVNQEP
ALFNETIRANIAYGKQGEAAEEEIIAATRAANAHNFISALPQGYDTSVGERGLQLSGGQKQRIAIARAILKDPRIL
LLDEATSALDAESERVVQDALDRVMVDRTTVVVAHRLTTIKGADVIAVVKNGEIAEKGTHDVLMDIRHGAYA
SLVALHMASST
>XP_003593853.3_ABCB9_Mt
MLFNFADHLDVTLMIIGTISAVANGLASPLMTLFLGNVINAFGSSNPADAIKQVSKVSLLFVYLAIGSGIASFLQ
VTCWMVTGERQAARIRSLYLKTILQQDIAFFDTETNTGEVIGRMSGDTILIQEAMGEKVGKFFQLASNFCGGFV
MAFIKGWRLAIVLLACVPCVAVAGAFMSIVMAKMSSRGQIAYAEAGNVVDQTVGAIRTVASFTGEKKAIEKY
NSKIKIAYTTMVKQGIVSGFGIGMLTFIAFCTYGLAMWYGSKLVIEKGYNGGTVMTVIIALMTGGIALGQTSPS
LQAFAAGQAAAYKMFETIRRKPIIDASDTSGAVLEDIKGDIELRDVSFRYPARPDVQIFDGFSLFVPSGTTTALV
GQSGSGKSTVISLLERFYDPDAGEVLIDGVNLKNLQLRWIREQIGLVSQEPILFTTSIRENIAYGKEGATDEEITT
AITLANAKKFIDKLPQGLDTMAGQNGTQLSGGQKQRIAIARAILKNPKILLLDEATSALDAESERIVQEALEKIIL
KRTTVVVAHRLTTIRNADIIAVVQQGKIVERGTHSGLTMDPDGAYSQLIRLQEGDNEAEGSRKSEADKLGDNL
NIDSHMAGSSTQRTSFVRSISQTSSVSHRHSQSLRGLSGEIVESDIEQGQLDNKKKPKVSIWRLAKLNKPEIPVIL
LGAIAAIVNGVVFPIFGFLFSAVISMFYKPPEQQRKESRFWSLLFVGLGLVTLVILPLQNFFFGIAGGKLIERIRSL
TFEKIVHQEISWFDDPSHSSGAVGARLSIDASTVKSLVGDTMALIVQNISTVIAGLVIAFTANWILAFIVLVLTPM
ILMQGIVQMKFLKGFSADAKVMYEEASQVANDAVSSIRTVASFCAESKVMDMYSKKCLGPAKQGVRLGLVS
GIGFGCSFLVLYCTNAFIFYIGSVLVQHGKATFTEVFRVFFALTMTAIAVSQTTTLAPDTNKAKDSAASIFEIIDS
KPDIDSSSNAGVTRETVVGDIELQHVNFNYPTRPDIQIFKDLSLSIPSAKTIALVGESGSGKSTVISLLERFYDPNS
GRILLDGVDLKTFRLSWLRQQMGLVGQEPILFNESIRANIGYGKEGGATEDEIIAAANAANAHSFISNLPDGYD
TSVGERGTQLSGGQKQRIAIARTMLKNPKILLLDEATSALDAESERIVQEALDRVSVNRTTVVVAHRLTTIRGA
DTIAVIKNGAVAEKGRHDELMRITDGVYASLVALHSSAS

>XP_002271305.1_P_ABCB9-l_Vv
MNGEGGETSKRDEISQQKVAFYRLFSFADGLDIVLMTVGTLGAIADGFTQPLMTLMMGRAIHSFATSDPSHVV
HQVSKVSLMFLYLAAGSGLAAFIQSSSWRVTGARQANSIRSLYLKTILRQDIEFFDTETTAGEVIGRLSGDTILIE
DAMGEKVGKFLQNMSTFVAGFTIAFLKGWRLVLVLLPTIPLVVMAGATMAMMMSKMSSHGQVAYAEAGAV
VEETVGAIRTVASFTGEKHAIENYNKKLKVAYTSTVQQGLASGFAVGAVVVIVFSSYGLAIWYGSKLIIEEGYN
GGTVVNVLLSLMVGGSSLGQASPCLSAFTAGQAAAYKMFETIKRKPKIDTYDTSGIVLEEIRGEIELKDVYFKY
PSRPDVQIFGGFSLHIPSRTTAALVGQSGSGKSTVISLLERFYDPEAGEVLIDGVNLKKLNIRSIREKIGLVSQEPIL
FAGTIKENISYGKKDATNEEIRAAIELSNSARFINKLQRGLDTMVGEHGTQLSGGQKQRIAIARAILKNPRILLLD
EATSALDAQSERIVQDALLNIMADRTTVVVAHRLTTIRNADVIAVVHQGKIVEQGTHVELIRDPNGAYSQLVR
LQEGTNQAADAQKVDKICERENTQKRSRTRSLSYKSVSMDSSSSHHSYSLSFGLPVPIGMDEIEVGREETTQQG
EAENEKSPKVPLRRLAYLNKPEVPVLLLGTIAAAVHGLVFPMFAFLLSTAVKIFYEPPNQLQKDSKFWALFFVG
LGVLALIVGPLQNFLFGVAGGKLIERIRSLSFEKVVHQEITWFDHPGNSSGAVGARLSTDASTVRGLVGDALAL
LVQNLTTIIVGLIISFTANWILALIILGVMPLLGFEGFVQGKFLKGFSAEAKVMYEEASHIVNEALGSIRTVASFC
AEEKVMEMYEQKCEATVKQGIRIGLVSGIGFGSSALALHCTNALVFYIGAILVEHGKATFPQLFKVFFALTISAV
GLSHASAMAPETTKAKDSAASIFHLLDSKPKIDSSIKEGTTLSTVKGDIELQHVSFKYPTRPDVQIFRDLCFSIPSG

KAVALVGESGSGKSTVISLIERFYNPDSGAILLDGMEIHKFKLSWLRQQMGLVGQEPILFNETIRANIAYGKQG
NASEDEIIAATRTANAHDFISALPQGYETTVGERGMQLSGGQKQRIAIARAIIKDPKILLLDEATSALDAESERV
VQEALDRVMVHRTTVVVAHCLTTIRGADMIAVVKNGVIAEMGRHDKLMKIADGAYASMVALHMSSSKGEE
QE
>XP_025884894.1_ABCB9-l_Sl
MEEKSSIVKDEKVPFYKLFLFADRVDIALMTIGTFGAIGEGLTQPLMTLIFGQIINSFGGASSSNEVFHLVSEAAV
YYVYLAIGSGIASFLRMSCWMVTGERQAIRIRGLYLKTILRQDIAFFDTETTTGQVIGTMSGDTFLIQDALGDKV
GKFIQYLSAFVGGFIIAFTKGWLLSLVLVSCIPALVIAGGAMASIMSKMSSRGQMTYAQAGDIVEQTVGAMRT
VAAFNGEKLAMIKYDNTLKIAYAFTVQQGLVSGVGFGTFLLVLFSTYGLAIWYGSKLIIEKGYRGGYVVNVLM
AIMIGGMLSSGNLLLVFLLICRSLGQTTPSLNAFAAAQVAALKIFETISRKPLIDTSDMSGVVLEDIEGEIELKDV
YFRYPSRPDVQIFSGFSLVVPSGKTVALVGQSGSGKSTIISLLERFYDPESGEVLLDGVNLKKYQLKWLRQQMG
LVSQEPILFATTIRENISYGKDNATEEEISAAIELANAANFIDKLPQGLDTMVGEHGTQLSGGQKQRLAIARAIV
KNPKVLLLDEATSALDAESERIVQEALEQVMAKRTTMLVAHRLTTIRNAGLIAVLHDGKLLEQGNHDKLVQD
PNGAYSQLMRMQEDKGGDEEENLIMKNMDSDKVNITMKLDNISWSSNPPLSAAKRSTNQGSPRNSFSPSYPVR
GMIDIHEATIGDVDEKEDDEQSSENRKKIPIRRLAELNKPELPYILLGSLAAIMHGLVMPLFGLLLSEAIKSFFNPP
HKLRNESQFWGLMYVGLGVVIWLVIPFQNYLFGVAGGKLIERIRSLTFKKVVHQEISWFDDPVNSSGALCARL
SIDASTVRTVVGDALALIVQNMATALGGLAIAFTANWILSFIILVVLPLICAPGLFQTKFHKGYSADAKVMYEE
ASQIANDAVGGIRTVASFCAEDKVMDMYQKKCEGPIKKGVKIGIVSGASLGFGSFTLYSSLGFCFFIGSVLIDHR
LATVDQVFKVFFALILAAVGITQSTTMAPNFNKAKDSITSIFDILDRKSIIDSSSDVGTTLAVVHGDIEFRLVSYR
YATRPDVQIFKDLCLIIPSGKTVALVGESGSGKSTVISLIERFYDPESGEIYLDGVEIKQFNLSWLRQQMGLVSQE
PILFNETIRDNIAYSRQGNATEEEIIEAAKSANAHNFISSLPQGYDTSVGERGIQLSGGQKQRIAIARAILKDPKILL
LDEATSALDAESERIVQEALDRVMVNRTTVVVAHRLATIKGADIIAVMKNGVIVEKGRHDVLMNIKDGAYAS
LVALHMTSAY
>XP_021612220.1_ABCB11-l_Me
MAEENGLNGVAKTHEASTSKTHEEKSAINGNSQETEKSKGDEKTNTVPFHKLFSFADSLDILLMIVGTIGAVGN
GISLPLMTIFLGDTINAFGENQNKDVVHVVSKVSLKFVYLAVGSAVASFLQVACWIVTGERQAARIRGLYLQTI
LRQDIAFFDKETNTGEVIGRMSGDTVLIQDAMGEKVGKFLQLVSTFFGGFVVAFIKGWLLTLVLLSSIPLLVLA
GAAMSITIAKMASRGQTAYAKAASVVEQTIGSIRTVASFTGEKQAISNYKKFLVTAYNSGVHEGLATGLGLGV
VMLIVFCSYALAIWFGGKMILEKGYSGGNVINVIIAVLSGSMSLGQASPCMSAFAAGQAAAYKMFETISRKPEI
DAYDTRGKKLDDIRGDIELRDIYFSYPARPDEQIFSGFSLSIPSGTTAALVGQSGSGKSTVVSLIERFYDPQAGEV
LIDGVNLKEFQLKWIREKIGLVSQEPALFTASIRDNIAYGKDGATIEEIRAAAELANAAKFIDKLPQGLDTMAGE
HGTQLSGGQKQRIAIARAILKDPRILLLDEATSALDAESERIVQEALDRIMVNRTTVIVAHRLSTIRNADVIAVIH
RGKLVEKGSHSELLSDPEGAYSQLIRLQEVNKGSEHAAENHKRSDLSSESFRQSSQKISLQRSISRGSSGVGNSS
RHSFSAPFGLPTGINVAENSQEETEVSPSQEKAPEVPISRLAYLNKPEIPVLTLATIAASLNGVIFPIFGILLSRVIKS
FFDPTPHELRKDTKFWAIIFMILGVASFLVLPSQFYFFGVAGNRLIQRIRTICFEKVVHMEVGWFDDPQHSSGAI
GARLSADAALVRALVGDALAQLVQNIATAVAGLVIAFTASWQLAFIILALIPLIGVNGYVQVKFMQGFSADAK
MMYEEASQVANDAVGSIRTVASFCAEEKVMQMYKKKCEGPLKTGVRQGLISGIGFGVSFFLLFSVYATSFYA
GAQLVKHGKTTFSDVFQVFFALTMTALGISQSSSFAPDSSKAKNAAASIFSIIDRKSKIDPSDESGMILENVRGEI
ELRHISFKYPSRPDIQIFRDLSLAIHSGKTVALVGESGSGKSTVISLLQRFYDPDSGHITLDGVEIQRLQVKWLRQ
QMGLVSQEPVLFNDTIRANIAYGKDEDATEAEILAASEMANAHKFISSLQQGYDTIVGERGVQLSGGQKQRVA
IARAIIKSPKILLLDEATSALDAESERVVQDALDRVMVNRTTVVVAHRLSTIKNADVIAVVKNGVVVEKGKHE
TLINIKDGFYASLVALHMTASTA
>XP_021610632.1_ABCB11-l_Me
MAEENGLNGVAGTQEAGTSKTYEEEEEKNPGINGNLQEAKKSKEDEKTNSVPFHKLFSFADSIDILLMIVGTIG
AVGNGISLPLMTIFLGDTINAFGQNQNKDVVHVVSKVSLKFVYLAVASAVASFLQVACWIVTGERQAARIRGL
YLKTILRQDVAFFDKETNTGEVIGRMSGDTVLIQDAMGEKVGKFLQLVSTFIGGFVVAFIKGWLLTIVLLSSIPL
LVLAGAAMSISIARMASRGQNAYAKAATVVEQTIGSIRTVASFTGEKQAISNYKKNLVTAYNSGVNEGLATGL
GLGVLMLIIFCSYALAIWFGGKMILEKGYTGGSVLNVIIAVLSGSMSLGQASPCMSAFAAGQAAAYKMFDTISR
KPEIDAYDTRGKILDDIHGDIELRDIHFSYPARPDEQIFSGFSLFIASGTTTALVGQSGSGKSTVISLIERFYDPQAG
EVLIDGINLKEFQLKWIREKIGLVSQEPVLFTASIRDNIAYGKDGATTEEIRAAAELANAAKFIDKLPQGLDTMA
GEHGTQLSGGQKQRIAIARAILKDPRILLLDEATSALDAESERIVQEALDRIMVNRTTVIVAHRLSTIRNADVIAV
IHRGKMVEKGSHSELLSDPEGAYSQLIRLQEVNKDSEQATEDHKRSNLSSESFRQSSQRISLQRSISRESSGVGNS
SRHSFSVSFGLPTGINVTENSQEKNEVSPPQKEIPEVSIRRLAYLNKPEIPVLTIGTIAACINGIIFPIFGILISRVIKSF
YEPPHELRKDTKFWAFIFMIIGVASFLVLPSQFYFFGVAGNRLIQRIRTICFEKVVHMEVGWFDDPEHSSGAIGA

RLSADAAVVRALVGDALAQLVQNIASAVAGLVIAFTASWQLAFIILVLLPLIGINGYVQVKFMKGFSADAKMM
YEEASQVANDAVGSIRTVASFCAEEKVMQLYKKKCEGPLKTGVRQGLISGIGFGVSFFFLFSVYATSFYAGAQL
VKHGKTTFSDVFQVFFALTMAAIGISQSSSFAPDSAKAKNAAASIFSIIDRKSKIDPSDDSGMTVENVRGEIELRH
VSFKYPSRPDVQIFRDLSLAIHSGKTVALVGESGSGKSTVISLLQRFYDPESGHITLDGVEIQRLQLKWLRQQMG
LVSQEPVLFNYTIRANIAYGKDGDATEAEIIAASEKANAHKFISSLQQGYDTVVGERGVQLSGGQKQRVAIARA
IIKSPKILLLDEATSALDAESERVVQDALDRVMVNRTTVVVAHRLSTIKNADVIAVVKNGVIVEKGKHENLINM
RDGFYASLVALHMSASTA

>XP_024441356.1_ABCB11_Pt
MAVENGRNGDKSMDEASTSKSLEVEEKSSGGRGDQQEPVKSKGDEETKTVPFLKLFSFADSTDILLMILGTIGA
VGNGASFPIMSILFGDLVNSFGQNQNNKDVVDSVTKVALNFVYLGIGSAVAAFLQVACWMVTGERQAARIRG
TYLKTILKQDVAFFDKETNTGEVVGRMSGDTVLIQDAMGEKVGKFIQLVSTFIGGFIIAFVKGWLLTLVMLSSIP
LLVIAGAGLAIIIARMASRGQTAYAKAATVVEQAIGSIRTVASFTGEKQAISNYKKFLATAYSSGVQEGFTAGL
GLGIVMLLIFCSYALAIWFGGKMILEKGYNGGDVINVIVAVLTGSMSLGQASPCMSAFAAGQAAAYKMFETIN
RKPEIDSSDTSGKILDDISGDVELRDVYFTYPARPDEQIFAGFSLFIPSGTTTALVGQSGSGKSTVISLIERFYDPQ
AGEVLIDGTNLKEFQLKWIREKIGLVSQEPVLFASSIKDNIAYGKDGATTEEIRAATELANAAKFIDKLPQGIDT
MVGEHGTQLSGGQKQRIAIARAILKDPRILLLDEATSALDAESERIVQEALDRIMVNRTTVIVAHRLSTVINAD
MIAVIYRGKMVEKGSHSELLKDPEGAYSQLIRLQEVNKESKQETEDPKKSALSAESLRQSSQRISLKRSISRGSS
GVGHSSRNSLSVSFGLPTGFNVPDNPTSELEVSPQKQQTPDVPISRLAYLNKPEVPVLIAGSIAAILNGVIFPIYGL
LLSSVIKTFFEPPDELRKDSKFWALMFMTLGLASFVVYPTQTYLFSVAGCKLIQRIRSMCFEKVVHMEVGWFD
EPEHSSGAIGARLSADAATVRALVGDSLSQLVQNIASAVAGLVIAFTASWQLALVILVLLPLIGLNGFVQIKFM
KGFSADAKKMYEEASQVANDAVGSIRTVASFCAEEKVMQLYRRKCEGPMRTGIRQGMISGTGFGVSFFLLFSV
YATTFYVGAQLVRHGKTNFADVFRVFFALTMAAIGISQSSSFAPDSSKAKGAAASIFAIIDRKSKIDPSDESGTTL
DNVKGEIELRHISFKYPSRPDIEIFRDLSLAIHSGKTVAVVGESGSGKSTVISLLQRFYDPDSGHITLDGIDIQSLQL
KWLRQQMGLVSQEPVLFNETIRANIAYGKEGNATEAEILAASELANAHKFISGLQQGYDTVVGERGTQLSGGQ
KQRVAIARAMVKSPKILLLDEATSALDAESERVVQDALDRVMVSRTTVVVAHRLSTIKNADVIAVVKNGVIVE
KGKHETLIHIKDGFYASLVALHMSASTS
>XP_014618641.1_ABCB21_Gm
MDVENGEERKHDDASTSENRAETSTNGEKEEKSKQQEKPETVPFHKLFAFADSTDILLMAVGTIGAIGNGLGL
PLMTLLFGQMIDSFGSNQRNTNVVEEVSKVSLKFVYLAVGSGLAAFLQVTSWMVTGERQAARIRGLYLKTILR
QDVAFFDKETNTGEVIGRMSGDTVLIQDAMGEKVGKFLQLIATFIGGFVIAFIKGWLLTVVMLSTLPLLALSGA
TMAVIIGRMASRGQTAYAKAAHVVEQTIGSIRTVASFTGEKQAVSSYSKFLVDAYKSGVHEGFIAGAGLGTVM
LVIFCGYALAVWFGAKMIMEKGYNGGTVINVIIAVLTASMSLGEASPSLSAFAAGQAAAYKMFQTIERKPEID
AYDPNGKILEDIQGEIELRDVYFSYPARPEELIFNGFSLHIPSGTTAALVGQSGSGKSTVISLVERFYDPQAGEVLI
DGINLKEFQLRWIRGKIGLVSQEPVLFASSIKDNIAYGKEGATIEEIRSASELANAAKFIDKLPQGLDTMVCEHG
TQLSGGQKQRIAIARAILKNPRILLLDEATSALDAESERVVQEALDRIMVNRTTIVVAHRLSTVRNADMIAVIHR
GKMVEKGTHSELLKDPEGAYSQLIRLQEVSKETEGNADQHDKTELSVESFRQSSQKRSLQRSISRGSSLGNSSR
HSFSVSFGLPTGVNVADPELENSQPKEEAPEVPLSRLASLNKPEIPVIVIGSVAAIANGVIFPIFGVLISSVIKTFYE
PFDEMKKDSEFWALMFMILGLASFLIIPARGYFFSVAGCKLIQRIRLMCFEKVVNMEVSWFDEPENSSGAIGAR
LSADAASVRALVGDALGLLVQNFATALAGLIIAFVASWQLALIILVLIPLIGVNGYVQMKFMKGFSADAKMMY
EEASQVANDAVGSIRTVASFCAEDKVMELYKKKCEGPMKTGIRQGLISGSGFGVSFFLLFCVYATSFYAGARL
MDSGKTTFSDVFQVFFALTMAAIGVSQSSSFAPDSSKAKSATASIFGIIDKKSKIDSSDASGSTLDSIKGEIELRHV
SFKYPSRPDMQIFRDLRLTIHSGKTVALVGESGSGKSTVIALLQRFYDPDSGQITLDGVEIRELQLKWLRQQMG
LVSQEPVLFNESLRANIAYGKGGDATEAEIIAAAELANAHKFISGLQQGYDTIVGERGTQLSGGQKQRVAIARA
IIKSPKILLLDEATSALDAESERVVQDALDKVMVNRTTVVVAHRLSTIKNADVIAVVKNGVIVEKGKHEKLINL
SDGFYASLVQLHTSASTV
>XP_002273987.1_P_ABCB11_Vv
MAEENDLNGKTYMHEATTSSRGALETETVKSSGQNGKQQDSEKSKEEGKPSTVPFHKLFSFADSTDMLLMITG
TIGAAGNGICMPLMAILFGDLIDSFGQNQNNKDVVDIVSKVSLKFVYLAVGAGIAAFFQVACWMVTGERQAA
RIRSLYLKTILRQDVAFFDKETNTGEVIGRMSGDTVLIQDAMGEKVGKFIQLVSTFIGGFIIAFIKGWLLTLVMLS
SIPLLVIAGGAMSLFLSKMATRGQNAYAKAATVVEQTIGSIRTVASFTGEKQAVTKYNQFLVNAYKSGVFEGL
AAGLGLGTVMFIIFASYALAVWFGAKMILEKGYTGGTVLNVIIAVLTGSMSLGQASPCMSAFAAGQAAAFKM
FQTIHRKPEIDVSDTKGKKLEDIQGEIELRDVYFSYPARPDEQIFSGFSLSIPSGTTAALVGQSGSGKSTVISLIERF
YDPLAGEVLIDGINLKEFQLRWIRGKIGLVSQEPVLFTSSIRDNIAYGKEGATIEEIRAAAELANASKFIDKLPQG

LDTMVGEHGTQLSGGQKQRVAIARAILKDPRILLLDEATSALDAESERVVQEALDRIMVNRTTIIVAHRLSTVR
NADMIGVIHRGKMVEKGSHTELLKDPEGAYSQLIRLQEVNKESENQATDSQDRPDGSIEFGRQSSQRMSFLRSI
SRGSSGPGNSSRHSFSVSFGLPTGLGLPDNAIADAEAPRSSEQPPEVPIRRLAYLNKPEIPVLLLGTVAAIVNGTIL
PIFGILISSVIKTFYEPPHQLRKDSNFWALIFLVLGVVSFLAFPARTYLFSVAGCKLIQRVRSMCFEKVVHMEVG
WFDQPEHSSGAIGARLSADAATIRALVGDALAQVVQNAASAIAGLAIAFAASWQLAFIILALIPLIGLNGYVQIK
FLKGFSADAKMMYEEASQVANDAVGSIRTVASFCAEEKVMDLYKKKCEGPMRTGIRQGLVSGIGFGVSFFLLF
CVYALCFYAGARLVEAGKTTFGDVFRVFFALTMATVGISQSSSFSPDSSKAKSAAASIFTIIDRKSTIDPSDESGT
KLENVKGEIELRHISFKYPTRPDIQIFRDLSLTIRSGKTVALVGESGSGKSTVIALLQRFYDPDSGHITLDGVDIQS
LQLRWLRQQMGLVSQEPVLFNDTIRANIAYGKEGHTTEAEVIAASELANAHKFISGLQQGYDTMVGERGIQLS
GGQKQRVAIARAMVKSPKILLLDEATSALDAESERVVQDALDRVMVNRTTVVVAHRLSTIKGADVIAVVKNG
VIVEKGKHETLINIKDGFYASLIALHMSASS
>XP_021612222.1_ABCB11-l_Me
MAMENDINGVTSSHEASTSKSHEEEKISQVNGHPEETGKRKGDEKTNSVPFHRLFSFADFTDIMLMIIGSIGAVG
NGISLPLMTIFLGDMINAFGENQNNKDVLHVVSKVSLKFVYLAVGSGVASFLQVACWMVTGERQAARIRGLY
LKTILRQDIAFFDKETNTGEVIGRMSGDTVLIQDAMGEKVGKFLQLVSTFIGGFVIAFVKGWLLTIVMLSSLPLL
VLAGAAMSIVMARMASQGQNAYAKAATVVEQTIGSIRIVASFTGEKQAISNYEKFLVTAYNAGVREGFFSGLG
LGLFTLIIFCSYGLAIWFGGKMILEKEYTGGEVINVIIAVLTGSTSLGQASPSMTAFAAGQAAAYKMFETINRKS
EIDAYDTRGKILDDIHGNIELREVYFSYPARPNEQIFNGFSLSIPTGTTAALVGQSGSGKSTVISLIERFYDPQAGE
VLIDGINLKEFQLKWIRNKIGLVSQEPVLFTSSIRDNIAYGKDEATTEEIRAAAELANAAKFIDKLPQGLDTMVG
EHGTQLSGGQKQRIAIARAILKDPRILLLDEATSALDAESERIVQEALDRIMVNRTTVIVAHRLSTVRNADMIAV
IHHGKIVQKGSHPELLADPDGAYSQLIRLQEINQDSEHAADENKGSDISSESFRRSSQRNSLQRSISRASNGVGNS
HRHSFSASFGLPTGINASEPEVSPQEKQTSEVPISRLAYLNKPEIPVLVAGSIAAIINGVIFPIFGILISRVIKAFFEPP
HELRKDSKFWAIIFMIIGIVSFLACVTQLYLFSVAGAKLIQRIRSMCFEKVVHMEVGWFDEVEHSSGTIGARLSA
DASILRALVGDTLAQTVQNIASAVAGLVIAFSASWQLAFIILVLIPLIGINGYVQVKFMKGFSADAKMMYEEAS
QVANDAVGSIRTVASFCAEEKVMQLYRKKCQGPLKSGIRQGLISGIGFGVSFFLLFSVYATSFYAGAQLVKHG
KTTFSDVFQVFFALVMAAIGVSQSSSFAPDSSKAKNAAASIFSILDDKSKIDPSDETGITLENVRGEIELVHVSFR
YPSRPDIQIFQDLCLAIHSGKTLALVGESGSGKSTVISLLQRFYDPDSGNITLDGVEIRRLQLKWLRQQMGLVSQ
EPILFNDTIRDNIAYGKGGNATEAEILAASELANAHKFISSLQQGYDTVVGERGIQLSGGQKQRVAIARAIVKNP
KILLLDEATSALDAESERVVQDALDRVMVSRTTVVVAHRLSTIKNADVIAVVKNGVIVEKGKHDSLINIRDGF
YASLVALHMSASTA

>XP_003591310.1_ABCB21_Mt
MGKVNGVDEIENNHDEATTSEKNSTETSSTNVVTNGEKDKTKEKQETVPFHKLFTFADSTDILLMIVGTIGAIG
NGLGLPLMTLLFGQMIDSFGSNQSNTTDVVEQVSKVSLKFVYLAVGSGVAAFLQVSCWMVTGERQAARIRGL
YLKTILRQDVTFFDKETNTGEVVGRMSGDTVLIQDAMGEKVGKFLQLIATFIGGFVIAFTKGWLLTVVMMSTL
PFLVVSGAAMAVIIGRMASKGQTAYAKAAHVVEQTIGSIRTVASFTGEKQAVSSYSKFLVDAYKSGVFEGTIA
GAGLGTVMFVIFCGYALAVWFGAKMIIEKGYNGGTVINVIIAVLTASMSLGQASPSMSAFAAGQAAAYKMFE
TIKRRPEIDAYDPNGKILEDIQGEIELKEVYFSYPARPEELIFNGFSLHISSGTTAALVGQSGSGKSTVISLVERFY
DPQAGEVLIDGINMKELQLRWIRGKIGLVSQEPVLFASSIKDNIAYGKDGATIEEIRSASELANAAKFIDKLPQGL
DTMVGDHGTQLSGGQKQRIAIARAILKNPRILLLDEATSALDAESERVVQEALDRIMVNRTTVVVAHRLSTVR
NADMIAVIHRGKMVEKGTHSELLKDPEGAYSQLIRLQEVNKESEETTDHHGKRELSAESFRQSSQRKSLQRSIS
RGSSIGNSSRHSFSVSFGLPTGVNVADPDLEKVPTKEKEQEVPLRRLASLNKPEIPVLLIGSLAAIANGVILPIFGV
LISSVIKTFYEPFDEMKKDSKFWAIMFMLLGLASLVVIPARGYFFSVAGCKLIQRIRLLCFEKVVNMEVGWFDE
PENSSGAVGARLSADAASVRALVGDALGLLVQNLASALAGLIIAFIASWQLALIILVLIPLIGLNGYVQMKFMK
GFSGDAKMMYEEASQVANDAVGSIRTVASFCAEDKVMELYRKKCEGPMKTGIRQGIISGSGFGVSFFLLFSVY
ATSFYAGARLVKAGNTTFSDVFRVFFALTMAAIGISQSSSFAPDSSKAKSATASIFGMIDKKSKIDPSEESGTTLD
SIKGEIELRHISFKYPSRPDIQIFRDLNLTIHSGKTVALVGESGSGKSTVIALLQRFYDPDSGEITLDGIEIRQLQLK
WLRQQMGLVSQEPVLFNDTIRANIAYGKGGIATEAEIIAAAELANAHRFISGLQQGYDTIVGERGTQLSGGQKQ
RVAIARAIIKSPKILLLDEATSALDAESERVVQDALDKVMVNRTTVVVAHRLSTIKNADVIAVVKNGVIVEKGR
HETLINVKDGFYASLVQLHTSAKTV
>XP_024441397.1_ABCB11_Pt
MMENGLEGDARIHQATTSSPYNDDERHLGNNGIQEEPENSKEDEKSKSVPFFKLFSFSDSTDFLLMFLGTLGAI
GNGLAMPLMTLLLGDVINAFGNNQLSKDMTDLVSKVSLKYVYLAVGSGIAACLQVTCWIVTGERQSSRIRSL
YLKTILRQDIAFFDKETNTGEVVGRMSGDTVLIQDAMGEKVGKFVQLMATFIGGFSVAFYKGWLLAVVMLSA

IPLLVLAGASMALFISKMAARGQNAYAEAANVVEQTIGGIRTVASFTGEKRAINIYNQLLVIAYRSGVQEGIFSG
FGVGVVMLIVFCSYAVAVWFGAKMVLEKGYTGGEVINVIVAVLTGSMSLGQASPCMSAFSAGRAAAYKMFE
TINRQPEIDAYDKRGKVLDDFHGDIELRDVYFSYPARPDEPIFSGFSLSIPRGTTAALVGHSGSGKSTVISLLERF
YDPLSGEVLIDGINIKELQLKWIREKTGLVSQEPVLFASSIKENIAYGKDGATNEEIRAAAELANAAKFIDKLPQ
GFDTMVGEHGTQLSGGQKQRIAIARAILKNPRILLLDEATSALDAESERVVQEALDNIMVDRTTVIVAHRLTTV
RNADMIAVIHRGKMVEKGTHSELLEDPDGAYSQLVRLQEMNKGSEQAALESEITMESFRQSSQRRSIRRSISRG
SSIGSSRHSFTLPFGLPTGFSVRDNVYDEPDDILPPEDAPDVPISRLASLNKPEIPVLIIGTIAACIHGTILPIYGTLM
SKAIKTFFLPPHELRKDSKFWAVMFMVLGVAAFVVIPVRSYFFSVAGCKLIQRIRSMCFEKVVNMEVSWFDEP
QHSSGAIGARLAADASIVRSLVGDQLASTVQNIATVTSAMIIAFTASWQLALVILALIPLIGINGVIQMKFMKGFS
ADAKMMYEEASQVANDAVCSIRTVASFCAEEKVMQLYLGKCRGPMKAGVRLGWVSGIGFGVSSFLLYCFYA
TSFYAGARLVDTGHITFQDVFQVFFALTLASVGISHSSTFTTDTTKAKGAAASVFSIIDRKSKIDPSDESGIILENV
KGEIELRHVSFKYPTRPDIQIFRDINLFMRAGKTVALVGESGSGKSTVVALLQRFYDPDSGHITLDGTEIQKLQL
KWLRQQMGLVGQEPVLFNDTIRANIAYGKGGDATEAEIISAAELANAHKFISGLQQGYNTGVGDRGIQLSGGQ
KQRVAIARAIVKNPKILLLDEATSALDAESERVVQSALERVMVNRTTVVVAHRLSTIRNADLIAVVKNGVIVEK
GRHESLINIKDGYYASLVALHTNAKTA

>XP_024441355.1_ABCB21_Pt
MAIENGRNGDKSMDEASTSKSLEVEEKSSGGRGDQQEPVKSKGDEETKTVPFLKLFSFADSTDILLMILGTIGA
VGNGASFPIMSILFGDLVNSFGQNQNNKDVVDLVTKVSLNFVYLGIGSAVAAFLQVACWMVTGERQAARIRG
TYLKTILKQDVAFFDKETNTGEVVGRMSGDTVLIQDAMGEKVGKFIQLVSTFIGGFIVAFVKGWLLALVMLSSI
PLLVISGAGLAIIIARMASRGQTAYAKAATVVEQTIGSIRTVASFTGEKQAISNYKKFLATAYNSGVQEGFTAGL
GLGIVMLLVFCTYALAIWFGGKMILEKGYTGGDVVNVIIAVLTGSMSLGQASPCMSAFAAGQAAAYKMFETI
NRKPEIDSSDTRGKILDDISGDVELRDVYFTYPARPDEQIFSGFSLFIPSGTTTALVGQSGSGKSTVISLIERFYDP
QAGEVLIDGTNLKEFQLKWIREKIGLVSQEPVLFASSIKDNIAYGKDGATTEEIRAAAELANAAKFIDKLPQGID
TMVGEHGTQLSGGQKQRIAIARAILKDPRVLLLDEATSALDAESERIVQEALDRIMVNRTTVIVAHRLSTVINA
DMIAVIYRGKMVEKGSHSELLKDPEGAYSQLIRLQEVNKESKQETEDPKKSALSAESLRQSSQRISLKRSISRGS
SGVGHSSRHSLSVSFGLPTGFNVPDNPTSELEVSPQKQQTPDVPISRLAYLNKPEVPVLIAGSIAAILNGVILPIYG
ILLSSVIKIFFEPPDELRKDSKFWALMFMTLGLASFVVYPSQTYLFSVAGCKLIQRIRSMCFEKVVHMEVSWFDE
SEHSSGEIGARLSADAAIVRALVGDSLSQLVQNIATAVAGLVIAFSASWQLALVILVLLPLIGLNGFVQVKFMK
GFSADAKKMYEEASQVANDAVSSIRTVASFCAEEKVMQLYRRKCEGPMRTGIRQGMISGTGFGVSFFLLFSVY
ATTFYVGAQLVQHGKTTFAEVFRVFFALTMAAVGISQSSSFSPDSSKAKGAAASIFAIIDRKSKIDPSDESGTTL
DNVKGEIELRHISFKYPSRPDIEIFRDLSLAIHSGKTVALVGESGSGKSTVISLLQRFYDPDSGHITLDGIDIQSLQL
KWLRQQMGLVSQEPVLFNETIRANIAYGKEGNATEAEILAASELANAHKFISGLQQGYDTVVGERGTQLSGGQ
KQRVAIARAMVKSPKILLLDEATSALDAESERVVQDALDRVMVSRTTVVVAHRLSTIKSADVIAVVKNGVIVE
KGKHETLIHIKDGFYASLVALHMSASTS
>XP_021610645.1_ABCB11-l_Me
MMAEENGNPNIQEANSSNSQVVVEEKDSSPSRSKENGKEKPKTVPFLKLFSFADSIDILLMITGTVGAFGNGAS
MPLMSLLMGQMVDSFGKNQADKDILHIVSKVSLKFVYLAIGAAAAAFLQVTCWMVTGERQAARIRSYYLKTI
LRQDIAFFDKETNTGEVVGRMSGDTVLIQDAMGEKVGKFLQLMATFIGGFVIAFAKGWMLALVMLAAIPLLV
LAGATVSILVSRMATRGQNAYAEAATVVEQTIGSIRTVASYTGEKRAISAYNKYLQIAYKSGAHEGFASGVGIG
VVMLVVFSSYGMAVWFGAKMILEKGYSGGQVINVIVAVLTGSMSLGQTSPCMSAFASGQAAAYKMFETINR
KPEIDAYDMSGKVLDDVRGDIELRDVYFSYPARPDEEIFSGFSLSIPSGTTAALVGHSGSGKSTVISLIERFYDPK
SGEVLIDGINIKEFQLKWIREKIGLVSQEPVLFSSSIKDNIAYGKEGATIEEIRSAAELANAAKFIDKLPQGIDTMV
GEHGTQLSGGQKQRIAIARAILKNPRILLLDEATSALDAESERVVQEALDRIMLDRTTVIVAHRLTTVRNADIIA
VIHRGKMVEKGTHSELLEDTDGAYSQLIRLQEVNKESEQAPNDCSRSEISVESFRQSSQRRSLRRSISRGSSRNSS
HHSLSLSFGLPTGLNGPENDLEDIEDLPSKEKYPEVPIRRLAYLNKPELPVLIVGTIAASINGTILPIYGILISKAIKT
FFEPPHELRKDSKFWALMFTTLGVASFLVYPFRTYFFSVAGSKLIQRIRSMCFEKVVHMEIGWFDEPEHSSGAIG
ARLSADAATVRALVGDALAQLVQNIATAVAAMVIAFTASWQLALIILALIPLIGVNGVVQVKFMKGFSADAK
VMYEEASQVANDAVGSIRTVASFCAEEKVMQLYEKKCEGPMWTGVRLGLVSGVGFGLSSFFLFCFYATSFYA
GARLVEGGHITFADVFQVFFALTMAAVGISQSSSMGTDSTKAKAAAASVFAIIDRQSKIDPSDESGTTIENVRGE
IELHHVSFKYPSRPDIQIFRDLSLTIRSGKTVALVGESGSGKSTVIALLQRFYDPNSGHITLDGVEIQKLQLRWLR
QQMGLVSQEPVLFNDTIRANIAYGKEGDATEAEIIAAAELANAHNFISSSQQGYETAVGERGVQLSGGQKQRV
AIARAIVKSPKILLLDEATSALDAESERVVQDALDRVMVNRTTVVVAHRLSTIKNADVIAVVKNGVIVEKGRH
ETLINIKDGFYASLVALHTSAQTA

>XP_021620296.1_ABCB11-l_Me
MAEENGNPNIQEANNSNFQVVVEEKDSSSSGSKENGKEKAKTVPFLKLFSFADSVDISLMITGTVGAFGNGVS
MPLMSLLMGQMVDSFGKNQADKEILHIVSKVSQKFLYLAIGAAAAAFLQVTCWMVTGERQAARIRSYYLKTI
LRQDIAFFDKETNTGEVVGRMSGDTVLIQDAMGEKVGKFLQLMATFIGGFAIAFAKGRMLALDMLAAIPLLVL
AGATGSILVSRMATRGQNAYAEAATAVEQTIGSIRTVASFTGEKRAISTYNKYLQIAYKSGAHEGFASGVVIGT
IILIVFCTYAMAVWFGAKLILEKGYSGGQVINVIVAVLIGSMSLGQASPCMRAFASGQAAAYKMFETINRKPEI
DAYDMSGQVMDDIRGDIELRDVYFSYPARPDEEIFSGFSLSIPSGTTAALVGHSGSGKSTVISLIERFYDPNSGEV
LIDGINIKEFQLKWIRENIGLVSQEPVLFSCSIKDNIAYGKEGATIGEIRSAAELANAAKFIDKLPQGLDTMVGEH
GTQLSGGQKQRIAIARAILKNPRILLLDEATSALDTESERVVQEALDRIMLDQTTVIVAHRLTTVRNADIIAVIHR
GKMVEKGTHSELLEDPDGAYSQLVRLQEVNKESEQAPNDCSRSEISVESFRLSSQRSLRRSISRGSSRNSSHHSL
SLSFGLPTGLDGPENDLENLEEVPSKEKYPEVPILRLAYLNKPELPVLIVGTIAASINGTILPIFGVLISKAIKTFFEP
PHELRKDSKFWALMFMTLGLASFLVHPFRTYFFSVAGSKLIQRIRSMCYEKVVHMEIGWFDEPEHSSGAIGARL
SADAATVRALVGDALAELVQNIATTVTAMVIAFTASWQLAFVILALIPLIGVNGVVQVKFMKGFSADAKAMY
EEASQVANDAVGSIRTVASFCAEEKAMQLYEKKCEGPMWTGVRLGMVSGIGFGLSSFFLFCFYAISFYAGARL
VEGGHITFADVFQVFFSLTMAAIGMSQSSSMGTDSAKAKAAAASVFAIIDRQSKIDPSDESGATIENVRGEIELH
HVSFKYPSRPDIQIFRDLSLTIHSGKTVALVGESGSGKSTVLALLQRFYDPDLGHITLDGVEIQKLQLRWLRQQM
GLVSQEPLLFNDTIRANIAYGKEGEATEAEIIAAAELANAHNFISSLKQGYETAVGERGVQLSGGQKQRVAIAR
AIVKSPKILLLDEATSALDAESERVVQDAMDRVMVNRTTVVVAHRLSTIKNADVIAVVKNGVIVETGRHETLI
NIKDGFYASLVALHTSAQTA

>XP_004240558.1_ABCB21_Sl
MAEGNSNGNSGPNEASSSSGGQNNTSQQDSDKTKQAEKANTVPFYKLFSFADSTDMVLMITGTIAAIGNGLSL
PIMTILFGDLTDSFGQNQNNKDVVRVVSKVSLEFVYLALGCGVASFLQVACWMISGERQASRIRSLYLKTILQQ
DIAFYDKETNTGEVVGRMSGDTVLIQDAMGEKVGKFVQLISTFIGGFVIAFTKGWLLTLVMLSVIPPLVISGGA
MSHVLSKMASSGQDAYAKAATVVEQTIGSIRTVASFTGEKKAVADYNESLVKAYHSGAKEGLATGLGLGSVF
AIIYCSYALAIWYGARLILEKGYTGGKVINIIIAVLTSSMSLGQAAPCMSAFAAGQAAAFKMFETIKRKPEIDAY
DTNGKILDDIRGDIELNDVCFTYPARPDEQIFSGFSLFVSSGTTAALVGQSGSGKSTVISLIERFYDPQSGQVLIDG
INLKDFQLKWIRGKIGLVSQEPVLFTASIKENILYGKYDATAEEIKVATELANAAKFIDKLPQGLDTMVGEHGT
QLSGGQKQRIAIARAILKDPRILLLDEATSALDAESERVVQEALDRIMINRTTVIVAHRLTTVRNADMIAVIHRG
KVVEKGTHGELLKDPEGAYSQLIRLQEVNNKTDKSGLDERDSIEKSMGSGRQSSQRVSLMRSISRSSSGVGNSS
RRSLSISFGLATGLSVPETANTDTETGIQEVAEKRLEVPIRRLAYLNKPEIPVMIIGTVAAIINGSILPIFGILLSSVI
KTFYEPPHELRKDSKFWALMFVLLGGVTFIAFPARTYLFSIAGCKLIRRIRSMCFEKVVRMEVGWFDDSEHSTG
IIGARLSADAAAVRGLVGDALAQMVQDIATSIVGLAIAFEASWQLALIILVMIPLIGLNGYIQIKFMKGFSANAK
VMYEEASQVANDAVGGIRTVASFCAEEKVMEIYKRKCEGPLKAGIKQGLISGIGFGVSFALLFCVYATSFYAG
ARLVQAGQITFSDVFRVFFSLTMAAIGISQSSSLAPDSSKAKSAAASVFAILDRKSKIDPSDESGMTLDTVKGDIE
LKHVSFKYPTRPDVQILRDLCLTIRSGKTVALVGESGCGKSTVISLLQRFYDPDSGQISLDGIEIQKFQVKWLRQ
QMGLVSQEPVLFNDTIRANIAYGKEGNATEAEVLAAAELANAHKFISGLQQSYDTTVGERGTQLSGGQKQRV
AIARAILKNPKILLLDEATSALDAESERIVQDALDRVMVNRTTVVVAHRLSTIKGADVIAVVKNGVIVEKGKHD
TLINIKDGFYSSLVALHTSAS
>XP_024451479.1_ABCB11_Pt
MMAEENGLGGDARIHEATSSSPVNDDGRHLGANENQEKHEKSKEHENTKSVPFFKLFSFADSTDYLLMFLGAI
GAIANGMSMPLMTLLLGDVINAFGSNQFGNDMTSLVSKVSLKFVYLAMGSGVAACFQVTCWIVTGERQASRI
RSTYLKTILRQDIAFFDKDTNTGEVVGRMSGDTVLIQDAMGEKVGKFLQLMATFIGGFAVAFIKGWLLALVML
SAIPLLVLAGASMALFISKMAARGQNAYAEAANVVEQTIGGIRTVASFTGEKRAISIYNDLLLTAYGSGVKEGIF
SGFGVGMVMFIVFCSYSMAVWFGAKMVLEKGYSGGAVINVIVAILTGSMSLGQASPCLSAFAAGRAAAHKM
FETIERKPEIDAYDIKGKVLDDIQGDIELRNVYFSYPARPDEPIFSGFSLSIPSGTTAALVGHSGSGKSTVISLVERF
YDPLAGEVLIDGINIKEFQLKWIREKTGLVSQEPVLFASSIKENIAYGKDGATNEEIRAAAELANAAKFIDKLPQ
GLDTMVGEHGTQLSGGQKQRIAIARAILKNPRILLLDEATSALDAESERVVQDALDKIMVDRTTVIVAHRLTTV
RNADMIAVIHRGKMVEKGTHSQLLGDPDGAYSQLVRLQEINRESGRETEISLESFRQSSQRRSVRRSISRSISRGS
SIGSSRHSFSLPFGLPTGFSVRENAYEDPEDILPPEDAPEVPLSRLASLNKPEIPVLIIGTIAACIHGTILPIYGTLMS
KAIKTFFEPPHVLRKDSKFWALMFMTLGVAAFVVIPVRSYFFSVAGCKLIQRIRSMCFERVINMEVSWFDEPEH
SSGAIGSRLAADAAIVRSLVGDQLAAIVQNIATVTSAMIIAFTASWQLALVILGLIPLIGINGVIQVKFMKGFSAD
AKMMYEEASQVANDAVCSIRTVASFCAEEKVMQLYEGKCRGPMKSGVRLGWVSGVGFGVSSFLLYCFYATS

FYVGARLVDAGHITFQDVFQVFFALTLASVGISHSSTFTTDTTKAKNAAASVFSIIDRKSKIDPSDESGIILENVK
GEIELRHVSFKYPTRPDIQIFRDINLLMRAGKTVALVGESGSGKSTVVALLQRFYDPDSGRITLDGTEIQKLQLK
WFRQQMGLVGQEPVLFNDTIRANIAYGKGGDATEAEIISAAELANAHKFISSLHQGYDTGAGDRGIQLSGGQK
QRVAIARAIVKNPKILLLDEATSALDAESERVVQDALDRVMVNRTTVVVAHRLSTVRNADLIAVVKNGVIVER
GRHESLIKIKDGFYASLVALHTSAKTE

>AtABCB21
MDSVIESEEGLKVDSPNRADAETSNSKIHEEDEKELKTESDLKEEKKKTEKNKQEEDEKTKTVPFHKLFAFADS
FDIILMILGTIGAVGNGLGFPIMTILFGDVIDVFGQNQNSSDVSDKIAKVALKFVYLGLGTLVAALLQVSGWMIS
GERQAGRIRSLYLQTILRQDIAFFDVETNTGEVVGRMSGDTVLIQDAMGEKVGKAIQLVSTFIGGFVIAFTEGW
LLTLVMVSSIPLLVMSGAALAIVISKMASRGQTSYAKAAVVVEQTVGSIRTVASFTGEKQAISNYNKHLVSAYR
AGVFEGASTGLGLGTLNIVIFCTYALAVWYGGKMILEKGYTGGQVLIIIFAVLTGSMSLGQASPCLSAFAAGQA
AAYKMFEAIKRKPEIDASDTTGKVLDDIRGDIELNNVNFSYPARPEEQIFRGFSLSISSGSTVALVGQSGSGKSTV
VSLIERFYDPQSGEVRIDGINLKEFQLKWIRSKIGLVSQEPVLFTSSIKENIAYGKENATVEEIRKATELANASKFI
DKLPQGLDTMVGEHGTQLSGGQKQRIAVARAILKDPRILLLDEATSALDAESERIVQEALDRIMVNRTTVVVA
HRLSTVRNADMIAVIHQGKIVEKGSHSELLRDPEGAYSQLIRLQEDTKQTEDSTDEQKLSMESMKRSSLRKSSL
SRSLSKRSSSFSMFGFPAGIDTNNEAIPEKDIKVSTPIKEKKVSFFRVAALNKPEIPMLILGSIAAVLNGVILPIFGIL
ISSVIKAFFKPPEQLKSDTRFWAIIFMLLGVASMVVFPAQTIFFSIAGCKLVQRIRSMCFEKVVRMEVGWFDETE
NSSGAIGARLSADAATVRGLVGDALAQTVQNLASVTAGLVIAFVASWQLAFIVLAMLPLIGLNGYIYMKFMV
GFSADAKRMYEEASQVANDAVGSIRTVASFCAEEKVMKMYKKKCEGPMRTGIRQGIVSGIGFGVSFFVLFSSY
AASFYAGARLVDDGKTTFDSVFRVFFALTMAAVAISQSSSLSPDSSKASNAAASIFAVIDRESKIDPSDESGRVL
DNVKGDIELRHISFKYPSRPDVQIFQDLCLSIRAGKTIALVGESGSGKSTVIALLQRFYDPDSGQITLDGVEIKTL
QLKWLRQQTGLVSQEPVLFNETIRANIAYGKGGDATETEIVSAAELSNAHGFISGLQQGYDTMVGERGVQLSG
GQKQRVAIARAIVKDPKVLLLDEATSALDAESERVVQDALDRVMVNRTTVVVAHRLSTIKNADVIAVVKNGV
IVEKGKHETLINIKDGVYASLVQLHLSAST

>XP_003609881.1_ABCB11_Mt
MATDIRLEGDFVSVQPVEDHDSNQDSEKSKDKDVTTKTVPLYKLFSFADPSDRLLMLMGTLGAIGNGLSIPLMI
LIFGTMINAFGDSTNSKVVDEVSEVSLKFVYLAAGTFVASFLQLTCWMITGERQSARIRGLYLKTILRQDVSFFD
KETNTGEVVGRMSGDTVLIKDAMGEKVGQFIQFMSTFIGGFVIAFTKGWLLTVVMLSSIPLLILSGSMTSMVIA
KASSTGQAAYSKSAGVVEQTIGSIRTVASFTGEKQATANYNRSLIKVYKTAVQEALASGVGFGTLFFVFICSYG
LAVWFGGKMIIEKGYTGGDVMTVIFAVLIGSTCLGQTSPSLSAFAAGQAAAFKMFETINRKPEIDAYDTSGKKL
DDIRGDIELRDVCFSYPTRPDELIFNGFSLSLPSGTTAALVGQSGSGKSTVVSLIERFYDPTDGEVLIDGINLKEFQ
LKWIRQKIGLVSQEPVLFTCSIKENIAYGKDCATDEEIRVAAELANAAKFIDKLPQGLDTMVGEHGTQLSGGQK
QRVAIARAILKDPRILLLDEATSALDAESERIVQEALNRIMINRTTIVVAHRLSTIRNVDTIAVIHQGKIVERGSHA
ELTNDPNGAYSQLIRLQEMKRSEQNDANDKNKPNSIVHSGRQSSQRSFSLRSISQGSAGNSGRHSFSASYVAPT
TDGFLETEDGGPQASPSKNSSPPEVPLYRLAYFNKPEIPVLLMGTITAVLHGAIMPVIGLLVSKMISTFYKPADEL
RHDSKVWAIVFVAVAVASLLIIPCRFYFFGVAGGKLIQRIRKLCFEKVVHMEVSWFDDVEHSSGALGARLSTD
AASVRALVGDALGLLVQNIATIIVGMVIAFQASWQLAFIVLALAPLLGLNGYVQVKVLKGFSADAKKLYEEAS
QVANDAVGSIRTVSSFCAEEKVMELYKQKCEGPIKKGVRRGIISGLGFGSSFFMLYAVDACVFYAGARLVEDG
KSTFSDVFLVFFALSMAAMGVSQSGTLVPDSTNAKSAAASIFAILDQKSQIDSSDESGMTLEEVKGDIEFNHVSF
KYPTRLDVQIFNDLCLNIRSGKTVALVGESGSGKSTVISLLQRFYDPDSGHITLDGIEIQRMQVKWLRQQMGLV
SQEPILFNDTVRANIAYGKGGDATEAEIVAAAELANAHQFIGSLQKGYDTIVGERGIQLSGGQKQRVAIARAIV
KNPKILLLDEATSALDAESEKVVQDALDRVMVERTTIIVAHRLSTIKGADLIAVVKNGVIAEKGKHEALLHKG
GDYASLVALHTSDSTS
>XP_002275143.2_P_ABCB11_Vv
MAGENGWRGGKYTEQATASTSHSSVMEIEKVPNDTDSKQETDTNREKEESTRTVPFCKLFSFADSWDYLFMF
VGAVAAAANGVSTPLMTILFGDVINSFGKDSNSKDMVHEVSKVSLKFVYLAIGTGVASFLQVTCWMLTGERQ
AARIRSLYLKTILRQDVGFFDKFTNAGEVVGRMSGDTVFIQDAMGEKVGKFIQLMATFLGGFIVAFCKGWLLT
LVMLSCFPPLVIVGAFTTMFITKMASRGQAAYSVAAVVVEQTIGSIRTVASFTGEKQAIAKYNQSLSKAYTSGV
QESVLSGLGFGLFMFVLFASYALAMWFGSKMIIDKGYTGGAVMNIIFSVVAGSMSLGQASPCLSAFGSGQAAA
FKMFETIERKPEIDAYSSDGQKLDDIQGDVELRDVYFSYPTRPDEQVFKGFSLSIPSGTTAALVGESGSGKSTVIS
LIERFYDPQAGEVLIDGINLKEFQLRWIRGKIGLVSQEPVLFTSSIRDNIAYGKDGATIEEIRAAAELANASKFIDK
LPQGLDTLVGEHGTQLSGGQKQRVAIARAILKDPRILLLDEATSALDAESERVVQEALDRVMINRTTIIVAHRLS

TVRNADMIAVIHRGKIVEKGAHSELIKDPDGAYSLLIRLQEISSEQNASHDQEKPEISVDSGRHSSKRMSLLRSIS
RSSSIGQSSRHSFSMSFGVPPDINIIETAPDGQDPAPLEHPPKVPLGRLAYLNKPEIPFLLLGTIAAVVNGAVFPVF
GILISSIIKSFFKPPHELRKDARFWALMFVVLGLVSFSSLSLRSYLFSTAGFKLIKRIRAMCFEKVVYMEVSWFDE
ADHSSGSIGARLSADAAMVRSLVGDALSLLVQNSAAMIAGLVIAFVANWKMSFIILVLLPLFGANGYVQVKFL
KGFTADAKKKYEEASQVANDAVGSIRTVASFCAEEKVMQLYQQKCEGPMNAGIREGLVGGVGYGVSFFLLF
AVYATAFYAGARLVDVGQATFAEVFQVFFVLTLAAVGVSQSSSLAPDTGKAKNAAASIFAILDRESKIDSSDES
GTTLENVKGEIEFHHVSFRYPTRPDIQIFRDLCLAIHSGKTVALVGESGSGKSTAISLLQRFYDPDSGHITLDGVEI
QKLQLKWFRQQMGLVSQEPVLFNETIRANIAYGKEGNATEAEISAAAELANAHKFISGLQQGYDTTVGERGIQ
LSGGQKQRVAIARAIVKDPKILLLDEATSALDAESERVVQDALDRVMVNRTTLVVAHRLSTIKGADLIAVVKN
GAIAEKGKHETLINIKDGIYASLVALHMSASS

>XP_006594002.2_ABCB11_Gm
MLYSMNSLNYDPTIYKLATGFNFPTDNLFFNSFLSCCFLRLVFRPSSQKALIASASCCKRVIGRGVCEQKESFYIE
MEGDISVNGDPNTYSNSNQDSKKSEAKDEPAKTVPLYRLFSFADPLDHLLMFVGTVGAIGNGISLPLMTLIFGN
MINAFGESSNTNEVVDEVSKVSLKFVYLAVGTFFASFLQLTCWMITGDRQAARIRGLYLQTILRQDVSFFDKET
NTGEVVGRMSGDTVLIQDAMGEKVGQFIQLISTFFGGFVVAFIKGWLLTVVMLACIPLLVMSGAMITVIISRAS
SEGQAAYSTAASVVEQTIGSIRTVASFTGERLAIAKYNQSLNKAYKTGVQEALASGLGFGLLYFVFICSYGLAV
WFGAKMIIEKGYTGGKVLTVIFAVLTGSMSLGQASPSLSAFAAGQAAAFKMFETIKRKPEIDAYDTTGRKLEDI
RGDIELREVCFSYPTRPDELIFNGFSLSIPSGTTAALVGQSGSGKSTVVSLIERFYDPQSGAVLIDGINLREFQLKW
IRQKIGLVSQEPVLFTCSIKENIAYGKDGATDEEIRAAAELANAAKFIDKLPQGLDTMVGEHGTQLSGGQKQRV
AIARAILKDPRILLLDEATSALDTESERIVQEALDRIMINRTTVIVAHRLSTIRNADTIAVIHLGKIVERGSHVELT
KDPDGAYSQLIRLQEIKRLEKNVDVREPESIVHSGRHSSKRSSFLRSISQESLGVGNSGRHSFSASFGVPTSVGFIE
PAGEGPQDPPSTAPSPPEVPLYRLAYLNKPEILVLLMGTVSAVITGVILPVFGLLLSKMISIFYEPAHELRKDSKV
WAIVFVGLGAVSFLVYPGRFYFFGVAGGKLIQRIRKMCFEKVVHMEVSWFDEAENSSGAIGARLSTDAASVRA
LVGDALGLLVQNTATAIAGLVIAFESSWQLALIILALVPLLGLNGYLQFKFLKGFSADTKKLYEEASQVANDAV
GSIRTVASFCAEEKVMELYQEKCEGPIKTGKRQGIISGISFGVSFFVLYSVYATSFYAGARLVEDRKATFTDVFR
VFFALSMAAIGISQSGSLVPDSTKAKGAAASIFAILDRKSEIDPSDDTGMTLEEFKGEIELKHVSFKYPTRPDVQI
FRDLSLTIHSGKTVALVGESGSGKSTVISLLQRFYDPDSGHITLDGTEIQRMQVKWLRQQMGLVSQEPVLFNDT
IRANIAYGKADATEAEIITAAELANAHTFISSLQKGYDTLVGERGVQLSGGQKQRVAIARAIVKSPKILLLDEAT
SALDAESEKVVQDALDRVMVDRTTIVVAHRLSTIKGADLIAVVKNGVIAEKGKHEALLNKGGDYASLVALHT
SAST

>XP_015612048.1_ABCB21_Os
MPESWRDAEANASSSASVAAADSSPGNGKGGGGGGGAAVARGERAASASASARVPFHKLFAFADKTDAALM
ALGTLGAVANGAALPFMTVLFGNLIDAFGGAMGIHDVVNRVSMVSLEFIYLAIASAVASFVQVTCWMITGER
QAARIRNLYLKTILRQEIAFFDKYTNTGEVVGRMSGDTVLIQDAMGEKVGKFIQLVVTFLGGFIVAFAQGWLL
TLVMMATIPPLVVAGAVMSNVVAKMASLGQAAYAESSVVVEQTIGSIRTVASFTGEKQAVEKYNKSLKSAYK
SGVREGLAAGLGMGTVMVLLFCGYSLGIWYGAKLILLKGYTGAKVMNVIFAVLTGSLALGQASPSMKAFAG
GQAAAYKMFETINRKPEIDAYSTTGMKPDDIRGDIEFRDVYFSYPTRPDEQIFRGFSLSIPSGTTVALVGQSGSG
KSTVISLIERFYDPQLGDVLIDGVNLKEFQLRWIRSKIGLVSQEPVLFAASIKENIAYGKDNATDQEIRAAAELA
NASKFIDKMPQGLDTSVGEHGTQLSGGQKQRIAIARAILKDPRILLLDEATSALDAESERIVQEALDRVMTNRT
TVIVAHRLSTVRNADTIAVIHQGTLVEKGPHHELLKDPEGAYSQLIKLQEANRQDKSDRKGDSGARSGKQLSIN
QSASRSRRSSRDNSHHSFSVPFGMPLGIDIQDGSSDNLCDGMPQDVPLSRLASLNKPEIPVLILGSIASVISGVIFPI
FAILLSNVIKAFYEPPHLLRKDSQFWSSMFLVFGAVYFLSLPVSSYLFSIAGCRLIKRIRLMTFEKVVNMEIEWFD
HPENSSGAIGARLSADAAKVRGLVGDALQLVVQNTTTLIAGLVIAFVSNWELSLIILALIPLIGLNGWIQMKFIQ
GFSADAKMMYEEASQVANDAVSSIRTVVSFSAEEKVMDLYKKKCEGPLRTGIRTGIISGIGFGVSFFLLFGVYA
ASFYAGARLVEENKTTFPKVFRVFLALAMAAIGVSQSSTLTSDSSKAKSAVSSIFAIVDRKSRIDPSEDAGVTVE
TLHGNIEFQHVSFRYPTRPDVEIFRDLCLTIHSGKTVALVGESGSGKSTAISLLQRFYDPDVGHILLDGVDIQKFQ
LKWLRQQMGLVSQEPALFNDTVRANIAYGKEGEATESEIIEAAKLANAHKFISSSHQGYGTTVGERGAQLSGG
QKQRIAIARAIVKDPKILLLDEATSALDAESERVVQDALDRVMVNRTTVIVAHRLSTIQNADLIAVVKNGVIIEK
GKHDTLMNIKDGAYASLVALHSAASS
>XP_021313046.1_ABCB4_Sb
MEAAGGAKGRDGVEKKEEENGNGHGGGDAVKKVPFTGLFRYADGTDVLLMLLGTVGSVANGVSQPVMTLI
FGQVINAFGDATTDDVLRRVNQAVLNFVYLGIATAVVSFLQVSCWTMTGERQATRIRSLYLKSVLRQEIAFFD

VEMTTGQIVSRMSGDTVLVQDAIGEKVGKFQQLVATFVGGFVIAFVKGWLLSLVMLACIPPVVIAGGIVSKML
AKISTKGQASYSDAGNIVEQTLGSIKTVVSFNGEKQAIALYNKLIHKSYKAAVEEGITNGFGMGSVFFIFFSSYG
LAIWYGGKLILSKGYSGGDIINILFAIMTGAMSLGNATPCMAAFAGGQSAAYRLFTTIKRKPEIDPDDPTGKQLE
DIKGDVDLNDVYFSYPARPEQLVFDGFSLHVSSGTTMAIVGESGSGKSTVISLVERFYDPQAGEVLIDGINIKSL
QLDWIRGKIGLVNQEPLLFMTSIKDNITYGKEDATIEEIKRAAELANAANFIDKLPNGYDTMVGQRGAQLSGG
QKQRIAIARAIIKNPRILLLDEATSALDVESERIVQEALNRIMLDRTTLVVAHRLSTVRNADCISVVQQGKIVEQ
GPHDELIMNPDGAYSQLIRLQESKEEEQKLDHHMSDSRSKSRSLSLKRSISRGSAGNSSRHSLTLPFGMPGSVEL
LEGNDANWEDEKDQARDGEAPKKAPMGRLASLNKPEVPILLLGSLAAGVHGVLFPMFGLMISNAIKTFYEPPH
QLKKDASFWGLMCVVLGIVSILSIPVEYFLFGIAGGKLIERVRAMSFRSIVHQEVAWFDDPKNSSGALGARLSV
DALNVRRLVGDNLALAVQVISTLIAGFVIAFVADWKLTLIILCVMPLSGVQGYAQVKFLKGFSEDAKILYEDAS
QVATDAVSSIRTVASFSAEKRVTTIYEDKCEASKKQGVRTGMVGGLGFGFSFLMMYLTYGLCFYVGAQFVRH
NKSTFGDVFKVFFALMLATIGISQTSALASDSTKAKDSAVSIFALLDRKSKIDSSNDEGSTLHEVKGDIDFRHVS
FKYPSRPDIQIFSDFTLHIPAGKTVALVGESGSGKSTVISLLERFYNPDSGTISLDGVEIKSLKVTWLRDQMGLVS
QEPILFNDTIRANIAYGKHGEVTEEELIKAAKAANAHEFVSSLPQGYDTTVGERGVQLSGGQKQRVAIARAILK
DPRILLLDEATSALDAESERIVQDALDHVMVGRTTVIVAHRLSTIKSADIIAVLKDGVIVEKGRHEALMNIKDGF
YASLVELRSASS
>XP_010321859.1_ABCB21-l_Sl
MGEGNDLNENNTVPFYKLNEASSEEQDNAGGKQDSNMIKQIQTVPFYKLFSFADSTDIVLMIIGTIGAIGNGLSI
PFMTVLFGELTDSFGQNQNNKDVLRLVSKISLKMVYLAVACGVAAFLQVACWMISGERQASRIRSLYLKTILQ
QDIAFYDNETNTGEVVGRMSGDTVLIQDAMGEKVGKCVQLISTFIGGFAIAFTQGWLLTFVMLSIIPLLIISGGV
MSLMLSRMASSGQEAYAKAAGVVEQTIGSIRIVASFTGEKKAIADYNESLIKAYHSGAKEGLASGLGLGSLFAL
MYCSYALAIWYGARLILEKGYTGGQVINIIVAVLTASMSLGQASPCMSAFAAGQAAAFKMFETIERKPEIDAY
DTNGKILNDIRGNIELNDVYFSYPARPDEKIFGGFSLFVPSGTTAALVGQSGSGKSTVISLIERFYDPQSGQVLID
GINLKDFQLKWIRGKIGLVSQEPVLFTASIKENIVYGKYDATPEEIRAAVKLANAAKFLDKLPQGLDTMVGEHG
TQLSGGQKQRIAIARAILKDPRILLLDEATSALDAESERVVQEALDRIMINRTTVIVAHRLTTVRNADMIAVIHR
GKVVEKGTHSELLKDPEGGYSQLIRLQEVNKETEKSGLDERGRLHKSMESGRQSSKRMSLLRSVSRSSSGVGN
SSSRSLSISFSFPNGLSVSETANEDRETGIQEVSGKPLNVPISRLAYLNKPEAPVIIIGTVAAIINGAILPIFGILLATV
IKIFYKPPEELRKDSRFWAEMFVLLAAVTLIAFPARSYLFGIAGCKLVRRIRSLCFEKLVHMEVGWFDEPENSTG
IIGARLSADAAAVRGLVGDALAQMVQDSATALIGLAIAFEASWQLALIVLVMIPLIGLSGYLQMKFMTGFSAD
AKTMYAEASQVANDAVGSIRTVASFCAEEKVMETYRGKCEGPLKAGIKQGLISGMGFGVSNTLMFCVYATSF
YAGALLVQNGKITFADVYRVFFALSTAAIGISQSSSLAPDSTKAKNAAASIFAILDRKSKVDPSDESGKTLENVK
GDIELRHVSFKYPTRPDVQILRDLCLTIRSGQTVALVGESGCGKSTVISLLQRFYDPDSGQISLDGIEIQKFQVKW
LRQQMGLVSQEPVLFNDTIRANIAYGKEGNAIEAEVLAAAELANAHKFISGLQQGYDTTVGERGTQLSGGQKQ
RVAIARAILKNPKILLLDEATSALDAESERIVQDALDRVVVNRTTVVVAHRLSTIKGADVIAVFKNGVIVEKGK
HDTLINIKDGFYSSLVALHTRST
>XP_002314333.2_ABCB4_Pt
MADENGLQGDRKFEQAAATTSHSEIVESEIQAAEKSKEKKESTNVVPYYKLFSFADPTDYLLMFVGTIAAIGNG
ACMPIMTILFGQVVNAFGSTSTNTEEVTHEVSQVALKFVYLGLGAMVAALLQVSCWMVTGERQAARIRNLYL
GAILRQEIGFFDNETHTGEIIGRMSGDTILIQDAMGEKVGKFLQLFTTFTAGFVIAFIKGWKLTLVMASSIPLLVL
SGAVMAITVSKMASRGQTAYSHAANIVDQSIGSIRTVVSFTGEKQAVVQYNKSLTEAVKTGVQEGLAIGVGFG
VVAFIVFSTYALAVWFGAKMILNDGYNGGDVVNVNFAVLTGSMSLGQSSSCLSAFSAGRAAAFKLFEVIDRKS
QIDSYNSNGRTLDDIQGDIELKDIHFSYPARPDEQIFNGFSLAIPPGTTAALVGKSGSGKSTIIGLIERFYDPHAGE
VLIDGVNLKEFQLKWIRQKIGLVSQEPVLFACSIKDNIAYGKDGATSEEIKTASELANAAKFIDKLPQGLDTMV
GENGTQLSGGQKQRIAIARAILKDPRILLLDEATSALDTESERIVQEALDRIMINRTTVVVAHRLSTVRNADAIA
VLHHGKIVEKGSHKELTKDPEGAYYQLIRLQETRTAQNNDVLNNPDGPESLADSDRHLSKRSSFRRSISRGSSL
EHSSRHSFSAAFGVPTGIDLPDTATAEPYILDSEPSEPLPEVPLFRLAYLNKPEIPVLVLAALAAIVAGAILPVFGI
LVSSMIKTFFEPPNKLKKDSEFWALMFVGIGAISLFIQPVKHCFFAVAGCKLIKRIRSMCFEKVIYMEVGWFDQP
EHSSGAIGARLSADAAMVKGLVGDALGMLVQNLGTAVVALFIAFQACWQLAFIMLAVLPLLGVNGFIQQKFM
KGFSADAKKMYEEASQVANDAVRNIRTVASFCSEAKVTGLYQQACKGPLKTGMRQGLVSGIGFGLSFFLLYA
VYAACFYAGSRLVNAGATTFSEVFRVFFALTMASFGISQTSSLGPDIMKAKAAAASVFAILDRNSKIDSTDDSG
TAIENFKGDIEFQHVSFIYPTRPDVQIFRDLCLKIRSGKTVALVGESGSGKSTVISLLQRFYDPDSGYITLDGVEIQ
KLQIKWLRQQMGLVSQEPLLFNDTIRANIAYGKEGIATEAEILAASELANAHKFISSLQQGYDTVVGDRGIQLS
GGQKQRVAIARAIIKAPKILLLDEATSALDAESERVVQDALEKVMVNRTTVIVAHRLSTIKNADVIAVVKNGVI
AEKGRHDTLMNIKDGVYASLVSLHTSASSS

>XP_003609882.2_ABCB11_Mt
MATDISLEGEIVSVQPVVDHDSMQDSDKSKDKDETTNTVPLYKLFSFADPSDRLLMLMGTVGAIGNGLSIPLMI
LIFGTMINAFGDSTNSKVVDEVSEVSLKFVYLAAGTFVASFLQLTCWMITGERQSARIRGLYLKTILRQDVSFFD
KETNTGEVVGRMSGDTFLIKDAMGEKVGQFIQFVATFIGAFVISFTKGWLLTVVMLSSIPLVVLSGAMMSLVIA
KASSTGQAAYSKSASVVEQTIGSIRTVASFTGEKQAITKYNQSLIKVYNTSVQEALASGVGFAALFFVFISSYGL
AVWYGGKLIIEKGYTGGDVMTVIFAVLTGSMCLGQTSPSLSAFAAGQAAAFKMFETIKRMPEIDAYDTSGRKL
DDIHGDIELKNVCFSYPTRPDELIFNGFSLSLPSGTTAALVGQSGSGKSTVVSLIERFYDPTDGEVLIDGINLKEFQ
LKWIRQKIGLVSQEPVLFTCSIKENIAYGKDCATDEEIRVAAELGNAAKFIDKLPQGLDTMVGEHGTQLSGGQK
QRVAIARAILKDPRILLLDEATSALDAESERIVQEALERIMINRTTIVVAHRLSTIRNVETIAVIHHGKIVERGSHA
ELTKYPNGAYSQLIRLQEMKGSEQNDANDKNKSNSIVHSGRQSSQRSFSLRSISQGSSGNSGRHSFSASYVAPA
TDGFLETADGGPQASPSTVSSPPEVPLYRLAYYNKPETAVILMGTIAAVLQGAIMPIFGLLISKMINIFYKPAHEL
RHDSKVWAIVFVAVAVATLLIIPCRFYFFGVAGGKLIQRIRNMCFEKVVHMEVSWFDEAEHSSGALGARLSTD
AASVRALVGDALGLLVQNIATAIAGLVISFQASWQLAFIVLALAPLLGLNGYVQVKVLKGFSADAKKLYEEAS
QVANDAVGSIRTVASFCAEKKVMELYKQKCEGPIKKGVRRGIISGFGFGLSFFMLYAVYACIFYAGARLVEDG
KTTFSDVFLVFFALSMAAMGVSQSGSLLPDSTNAKSATASIFAILDQKSQIDPGDESGMTLEEVKGEIEFNHVSF
KYPTRPDVQIFVDLCLNIHSGKTVALVGESGSGKSTVISLLQRFYDPDSGHITLDGIEIQRMQVKWLRQQMGLV
SQEPVLFNDTVRANIAYGKGGDATEAEIVAAAELANAHQFIGSLQKGYDTIVGERGIQLSGGQKQRVAIARAIV
KNPKILLLDEATSALDAESEKVVQDALDRVMVERTTIIVAHRLSTIKGADLIAVVKNGVIAEKGKHEALLHKG
GDYASLVALHKSASTS
>XP_006355822.2_P_ABCB21-l_St
MGEGNVLNENNTVPFYKLNEVSSEEQDNAGGQQDSNMTKQIQTVPFYKLFSFADSTDIVLMIIGTIGAIGNGLS
LPIMTVLFGELTDSFGQNQNNKDVLRIVTKISLKMVYLALACGVAAFLQVACWMISGERQASRIRSLYLKTILQ
QDIAFYDNETNTGEVVGRMSGDTVLIQDAMGEKVGKCVQLISTFIGGFVIAFTKGWILTFVMLSIIPLLIISGGV
MSLILSRMASSGQEAYAKAATVVEQTIGSIRIVASFTGEKKAIADYNESLIKAYHSGAKEGLASGLGLGSLFAL
MYCSYALAIWYGARLILEKGYTGGQVINIIVAVLTASMSLGQTSPCMSAFAAGKAAAFKMFETIERKPEIDAYD
TNGKILNDIRGNIELNDVYFSYPARPDEKIFGGFSLFVPSGTTAALVGQSGSGKSTVISLIERFYDPQSGQVLIDG
VNLKDFQLKWIRGKIGLVSQEPVLFTASIKENIVYGKYDATPEEIRAAVELANAAKFLDKLPQGLDTMVGEHG
TQLSGGQKQRIAIARAILKDPRILLLDEATSALDAESERVVQEALDKIMINRTTIIVAHRLTTVRNADMIAVIHRG
KVVEKGTHSELLKDPEGGYSQLIRLQEVNKETEKSGLDERGRLDKSMESGRQSSKRMSLLRSVSRSSSGVGNS
SSRSLSISFSFPNGLSVSETANEDTETGIQEVSGKPLNVPISRLAYLNKPEAPVIIIGTVAAIINGAILPIFGVLFATVI
KIFYKPPEELRKDSRFWAEMFVLLAAVTLIAFPARSYLFGIAGCKLVRRIRSMCFEKLVHMEVGWFDEPENSTG
IIGARLSADAAAVRGLVGDALAQMVQDSATAIIGLAVAFEASWQLALIVLAMIPIIGLSGYLQMKFMTGFSADA
KTMYAEASQVANDAVGSIRTVASFCAEEKVMETYRGKCEGPLKAGIKQGLISGMGFGVSNTLMFCVYATSFY
AGALLVQNGKITFADVYRVFFALSTAAIGISQSSSLAPDSTKAKNAAASIFAILDRKSKVDPSDESGKTLDIVKG
DIELRHVSFKYPTRPDVQILRDLCLTIRSGQTVALVGESGCGKSTVISLLQRFYDPDSGQISLDGIEIQKFQVKWL
RQQMGLVSQEPVLFNDTIRANIAYGKEGNAIEAEVLAAAELANAHKFISGLQQGYDTTVGERGTQLSGGQKQ
RVAIARAILKNPKILLLDEATSALDAESERIVQDALDRVVVNRTTVVVAHRLSTIKGADVIAVFKNGVIVEKGK
HNTLINIKDGFYSSLVALHTRSS
>XP_015640224.1_ABCB21_Os
MVDCSLVLRILGLQSMSESSRAFGVNASSSSSGGGGGGEAVAGTTGKNGGGGSVAFHRLFAFADGTDAALML
LGTLGAVANGAALPFMTVLFGGLIDAFGGAAGGDVVARVSEVSLQFIYLAVASAAASFIQVACWMITGERQA
ARIRSLYLRTILRQEVAFFDKHTNTGEVVGRMSGDTVLIQDAMGEKVGKFVQLLVTFLGGFGVAFAQGWLLT
LVMLATIPPLVLSGAVMSNVVARMASLGQAAYADASVVVEQTIGSIRTVASFTGEKQAVAKYSRSLKRAYSS
GVREGLAAGVGMGTVMVLLFCGYSLGIWYGAKLILEKGYTGAQVMNVIFAVLTGSLALGQASPSMKAFAGG
QAAAYKMFETINREPEIDAYSATGRKLDDIQGDIEFRNVYFSYPTRPDEQIFRGFSLAIQSGTTVALVGQSGSGK
STVISLIERFYDPQLGEVLIDGVNLKELQLRWIRSKIGLVSQEPILFAASIIDNIAYGRDNATNQEIRAAAELANAS
KFIDKMPQGFATLVGEHGTQLSGGQKQRIAIARAILKDPRILLLDEATSALDTESERIVQEALDRVMSNRTTVIV
AHRLTTVRNADTIAVIHQGSIVEKGSHHELISDPDGAYSQLIRLQENSHDSEDANYQNKSGKKSDSGIRSGKQSF
SYQSTPQRSSRDNSNNHSFSVSATPLEIDVQGGSPKKIAEETPQEVPLSRLAALNKPEIPVLLLGSVASAVSGVIF
PIFAILLSNVIKAFYEPPQVLKKDAEFWSSMFLVFGAVYFLSLPIGSYLFSVAGCRLIRRIRLMTFEKVVNMEIEW
FDHPENSSGSIGARLSADAAKIRGLVGDALQLVVQNLATLVAGLLIAFISNWELSLIILALIPLIGVNGWIQMKFI
QGFSADAKMMYEEASQVANDAVSSIRTVASFSAEEKVMDLYKMKCEGPLRTGIRTAIISGIGFGVSIFLLFGVY
AASFYAGARLVEDRKTTFPNVFRVFLALTMAAIGVSHTSNLTSDSSKAKSAVSSIFAIVDRKSRIDPSDDAGVSL
EPLRGDIEFQHVSFRYPTRPDVQIFEDLCLTIQSGKTVALVGESGSGKSTAISLLQRFYDPDAGHILLDGVDIQKF

QLRWLRQQMGLVSQEPALFNDTIRANIAYGKEGDATESDIVSSAQLANAHKFISSLHQGYETMVGERGAQLSG
GQKQRIAIARAIVKDPKILLLDEATSALDAESERVVQDALDRVMMNRTTVIVAHRLSTIQGADMIAVVKNGMII
EKGKHDALIGIKDGAYASLVALHVSAAAIS
>XP_002457701.1_ABCB21_Sb
MPESWRPAEANASSQPAAAGTSGPSAPSPGNGAKGGSPGAAEAAATATATRVPFHKLFAFADSTDVALMLLG
ALGAVANGAAMPFMTVLFGNLIDAFGGALSIHDVVNRVSMVSLEFIYLAIASAVASFVQVTCWMITGERQAA
RIRNLYLKTILRQEIAFFDKYTSTGEVVGRMSGDTVLIQDAMGEKVGKFIQLVVTFLGGFIVAFAQGWLLTLVM
MATIPPLVMAGAVMSNVVTKMASLGQAAYAESSVVVEQTIGSIRTVASFTGEKRAVEKYNKSLKNAYKSGVR
EGLATGLGMGTVMVLLFCGYSLGIWYGAKLILEKGYTGAKVMNVIFAVLTGSLALGQASPSMKAFAGGQAA
AYKMFETINRTPEIDAYSTTGRKLEDIRGDIEFRDVYFSYPTRPDEQIFKGFSLTIPSGMTIALVGQSGSGKSTVIS
LIERFYDPQLGDVLIDGVNLKEFQLRWIRSKIGLVSQEPVLFAASIKENIAYGKDNATDLEIRAAAELANAAKFI
DKMPQGFDTSVGEHGTQLSGGQKQRIAIARAILKDPRILLLDEATSALDAESERIVQEALDRVMTNRTTVIVAH
RLSTVRNADTIAVIHQGTLVEKGPHNELLRDPEGAYSQLIRLQEANQQNNRKGDANARPGKQTSINKSASRRSS
RDNSSHHSFSVPFGMPLGIDIQDGSSNKLCDEIPQEVPLSRLASLNKPEIPVLILGSIASVISGVIFPIFAILLSNVIK
AFYEPPHLLRRDSQFWASMFLVFGAVYFLSLPVSSYLFSIAGCRLIRRIRLMTFEKVVNMEIEWFDHPENSSGAI
GARLSADAAKVRGLVGDALQLVVQNSSTLVAGLVIAFVSNWELSLIILALIPLIGLNGWIQMKFIQGFSADAKM
MYEEASQVANDAVSSIRTVASFSAEEKVMDLYKKKCEGPLRTGIRTGIISGIGFGVSFFLLFGVYAASFYAGAR
LVEDRKTTFPKVFRVFLALAMAAIGVSQSSTLTSDSSKAKSAASSIFAIVDRKSRIDPSEDAGVTVETLRGNIEFQ
HVSFRYPTRPDVQIFRDLCLTIHAGKTVALVGESGSGKSTAISLLQRFYDPDVGNILLDGVDIQKFQLRWLRQQ
MGLVSQEPALFNDTIRANIAYGKDGQATESEIISAAELANAHKFISSALQGYDTMVGERGAQLSGGQKQRVAI
ARAIVKDPRILLLDEATSALDAESERIVQDALDRVMVNRTTVIVAHRLSTIQNADLIAVVRNGVIIEKGKHDALI
NIKDGAYASLVALHSAASS
>XP_008673955.1_ABCB21_Zm
MHELGGKEQPYEGEQSREHTARRASSIAQSRGGERFVSAEARIFDAATAAMPESWRPAEANASSQPAAGTSGP
SAQSPGNDAKGRAAGETPGAAEAAATRVPFHKLFAFADSTDVALMLLGALGAVANGAAMPFMTVLFGNLID
AFGGALSIHDVVNRVSMVSLDFVYLAIASAVASFVQVTCWMITGERQAARIRNLYLKTILRQEIAFFDKYTSTG
EVVGRMSGDTVLIQDAMGEKVGKFIQLVVTFFGGFIVAFAQGWLLTLVMMATIPPLVVAGAVMSNVVTKMA
SLGQAAYAESSVVVEQTIGSIRTVASFTGEKRAVEKYNKSLKSAYKSSVREGLATGLGMGTVMLLLFCGYSLG
IWSGAKLILEKGYTGAKVMNVIFAVLTGSLALGQASPSMKAFAGGQAAAYKMFETINRAPEIDAYSTTGRKLE
DIRGEIEFRDVHFSYPTRPDEPIFRGFSLAIPSGTTIALVGQSGSGKSTVISLIERFYDPQLGDVLIDGVNLKEFQLR
WIRSKIGLVSQEPVLFAASIKENIAYGKASATDQEVRAAAELANAAKFIDKMPQGFDTSVGEHGTQLSGGQKQ
RIAIARAILKDPRILLLDEATSALDAESERIVQEALDRVMSNRTTVIVAHRLSTVRNADTIAVIHQGTLVEKGPH
NELLRDPEGAYSQLIKLQEANQQNNRKGDGNARLGKQMSMNKSASRRLSRDNSSHHSFSVPFGMPLGIEIQDG
SSNKLCDEMPQEVPLSRLASLNKPEIPVLVLGSIASVISGVIFPIFAILLSNVIKAFYEPPHLLRRDSQFWASMFLV
FGAVYFLSLPVSSYLFSIAGCRLIRRIRLMTFEKVVNMEVEWFDHPENSSGAIGARLSADAAKVRGLVGDALQL
VVQNSSTLVAGLVIAFVSNWELSLIILALIPLIGLNGWIQMKFIQGFSADSKMMYEEASQVANDAVSSIRTVASF
SAEEKVMDLYKKKCEGPLRTGIRTGIISGIGFGVSFFLLFGVYAASFYAGARLVEDRKTTFPKVFRVFLALAMA
AIGVSQSSTLTSDSSKAKSAASSIFAIVDRKSRIDPSEDAGVTAETLRGNIEFQHVSFRYPTRPDVQIFRDLCLTIH
AGKTVALVGESGSGKSTAISLLQRFYDPDVGHILLDGVDIRKFQLRWLRQQMGLVSQEPALFNDTIRANIAYG
KDGQATESEIVSAAQLANAHKFISSALQGYDTMVGERGAQLSGGQKQRVAIARAIVKDPRILLLDEATSALDA
ESERIVQDALDRVMVNRTTVVVAHRLSTIQNADLIAVVRNGVIIEKGKHDALVNVKDGAYASLVALHSAASS
>XP_004234243.1_ABCB9-l_Sl
MARGGDEKIPFYKLFAFADRNDIILMLFGILGAIASGVSKPLMSLMFGDLIDSYGTSDQSNILDKVSRISLKFVYL
GIGTGIASLLQVACWSITGERQVTRIKCLYLKTILRQDIEFFDTQSATGEVIERMSGDTILLQEAMGEKVGNFVM
HMSTFIGGFVVAFIKEWQLTLVLLATIPAIAISFFCAALVLSKMSGSGQAAYANAGKVVEQTVGGIRTVVSFTG
ENLSIVDYNSKLENAYKPTVNQALASGIGLGTILMFSLFSYGLAIWYGAKLIIDKNYSGGDIVTVIFSAMLGGSSI
GQASPSLSAFSAGQAAAYKIYETIKRTPKIDPYDPRGIQLEDIKGEIELKDVYFKYPARPDVQIFSGFSLYIPSGKT
AALVGQSGSGKSTVISLLQRFYDPEAGEILIDGVDIKKFQLKWLRQQMGLVSQEPVLFATTIRENIMYGKENSS
EEEIRNAIKLANAAKFIDKLPKGLDTMVGGHGTQISGGQKQRIAIARAILKDPRILLLDEATSALDVESERIIQDA
LSNIMINRTTVVVAHRLTTIRNADLIAVVNLGKLVEQGTHDELIKDIDGAYSQLVQMQQNNKHVENTKGKEIE
DSNAQKRLSCSKNPSGRSQKFSISSWKSASKGSSSRYSLAYDLGVTAAIDFHESIRRDDGAESSEYIVDSNRNLS
TQKLMSLAYLNKPEVPIMLVGTIAASINGAVYPVFGLLLSTSIKIFYESHHELRKDSRFWALMFVVIGVVVMIV
APLQNYAFGIAGAKLIQRIRSMTFAKLVYQEISWFDDPANSCGAIGARLSSDASTIRNLAGDALATIVQNISTVA
TGMVIALIANWILALIMLAILPLLVLQGIIQIKLLQESNAEAKVANEEASQVANDAIGSIRTVASFCAEEKVMEM

YQRKSEAPLKQGAKTGLVGGVGLGFSSFVLFSLYALTFYLGAILVKHDKAKFSEVFKVFFALSMASIGLIALGN
LPSDLSKSKGAAASIFEILDSKPRIDSSSSEGIMLDVIEGNIELQHISFKYPTRPDMQIFKDLSLSIPAGKTVALVGE
SGSGKSTVISLIERFYDPDQGYIYLDGVELKKLNLRWLRQQMGLVGQEPILFNETISSNIAYGRQGEVTEEEIISV
AKASNAHNFISSLPNGYKTTVGERGVQLSGGQKQRIAIARAILKDPKILLLDEATSALDTESERIVQEALDRVM
VNRTTVVVAHRLTTIKNADVIAVVKNGVVAEKGTHDVLMNNTQGVYASLVALQTGAT
>XP_014625441.1_ABCB11_Gm
MEGDISVNGDPNINNNHDSKKRDDKDEPAKTVPLYKLFSFADPLDLLLMFVGTVGAIGNGISMPLMTLIFGSLI
NAFGESSNTDEVVDEVSKVSLKFVYLAVGTFFAAFLQLTCWMITGNRQAARIRGLYLKTILRQDVSFFDKETST
GEVVGRMSGDTVLIQDAMGEKVGQFIQLVATFFGGFVVAFIKGWLLTVVMLSCIPLLALSGAMITVIISKASSE
GQAAYSTAAIVVEQTIGSIRTVASFTGERPAIAKYNQSLTKAYKTGVQEALASGLGFGVLYFVLMCSYGLAVW
FGAKMVIEKGYTGGEVVTIIFAVLTGSFSIGQASPSLSAFAAGQAAAFKMFETIKRKPEIDAYGTTGLKINDIRG
DIELKEVCFSYPTRPDELVFNGFSLSIPSGTTAALVGQSGSGKSTVVSLIERFYDPQSGAVLIDGINLREFQLKWI
RQKIGLVSQEPVLFTCSIKENIAYGKDGATDEEIRAAAELANAAKFIDKLPQGLDTMVGEHGTQLSGGQKQRV
AIARAILKDPRILLLDEATSALDAESERIVQEALDRIMINRTTVIVAHRLSTIRNADTIAVIHQGKIVESGSHAELT
KDPDGAYSQLIRLQEIKRSEKNVDNRDKSGSIGHSGRHSSKRSSFLRSISQESLGVGNSGRHSFSASFRVPTSVGF
IEAATGEGPQDPPPTAPSPPEVPLYRLASLNKPEIPVLLMGTVAAVLTGVILPVFSILLTKMISIFYEPHHELRKDS
KVWAIVFVGLGAVSLLVYPGRFYFFGVAGSKLIQRIRKMCFEKVVHMEVSWFDEAEHSSGAIGSRLSTDAASI
RALVGDALGLLVQNIATAIAALIIAFESSWQLALIILALVPLLGLNGYVQLKFLKGFSADTKKLYEEASQVAND
AVGSIRTVASFCAEEKVMELYQEKCEGPIKTGKRQGIISGISFGVSFFMLYAVYATSFYAGARLVEDGKSSFSDV
FRVFFALSMAALGISQSGSLVPDSTKAKGAAASIFAILDRKSEIDPSDDSGMTLEEVKGEIELRHVSFKYPTRPD
VQIFRDLSLTIHTGKTVALVGESGCGKSTVISLLQRFYDPDSGHIILDGKEIQSLQVRWLRQQMGLVSQEPVLFN
DTIRANIAYGKGDATEAEIIAAAELANAHRFISSLQKGYDTLVGERGVQLSGGQKQRVAIARAIVKNPKILLLDE
ATSALDAESEKVVQDALDRVMVDRTTIVVAHRLSTIKGADLIAVVKNGVIAEKGKHEALLDKGGDYASLVAL
HTSASTS
>XP_003591313.2_ABCB21_Mt
MGVEIENDFVDEATTSENNRTETSTNATTNGEKDITKEKQETVPFHKLFSFADSTDILLMIVGTIGAIGNGLGLPI
MTVLLGQMIHSFGSNQTNTEDIVDQVTKVSLKYVYLAVGSGVAAFLQVSCWMVTGERQAARIRGLYLKTILR
QDVTFFDKETNTGEVIGRMSGDTVLIQDAMGEKVGKFLQLIATFVGGFVIAFTRGWLLTVVLMSTLPLLVVSG
AAMAVIIGRMASKGQTAYAKAAHVVEQTIGSIRTVASFTGEKQAVANYSKHLVDGYKSGVFEGFISGVGVGT
FMFLMFLGYALAVWFGAKMVMEKGYNGGTVINVIMVVLTASMSLGQASSGLSAFAAGRAAAYKMFETIKRR
PEIDAYDPNGKILEDIQGEIELKEVYFSYPARPEELIFNGFSLHIPSGTTTALVGQSGSGKSTIISLVERFYDPQAGE
VLIDGINMKEFQVRWIRGKIGLVSQEPVLFASSIKDNISYGKDGATIEEIRSASELANAAKFIDKLPQGLDTMVG
DHGSQLSGGQKQRIAIARAILKNPRILLLDEATSALDAKSERVVQETLDRIMVNRTTVVVAHRLSTVRNADMIA
IIHRGKMVSKGTHTELLKDPEGAYSQLVRLQEINKESEETTDHHIKRELSAKSFRQLSQRKSLQRSISRGSSIGNS
SRHSFSVSSVLPTGINAIDPGLENLPTKEKGQEVPLSRLATLNKPEIPVLLFGCFAAIGNGVIFPIFGILTSSMIKTF
YEPFDEMKKDSKFWAVMFMLLGFASLLVVTAQSYFFSVAGYKLIQRIRLLCFEKVVSMEVGWFDEPENSSGS
VGARLSADAASVRTIVGDALGLLVMNLAAALSGLIIAFVASWQLALIILVLIPLIGLNGYVQMKSMKGFSADAK
MMYEEASQVANDAVGSIRIVASFCAENKVMELYRKKCEVPMKTGIRQGIISGSGFGVSFFLLFCVYALSFYAG
ARLVESGHTKFSDVFRVFFALTMATVGISQSSSFAPDSSKAKSATASIFRMIDKKSKIDPSDESGTTLDSVKGEIE
LRHLSFKYPSRPDIQIFQDLNLTIHSGKTVALVGESGSGKSTVIALLQRFYDPDSGEITLDGIEIRQLQLKWLRQQ
MGLVSQEPVLFNDTIRSNIAYGKGGNATEAEIIAAAELANADRFISGLQQGYDTIVGERGTQLSGGQKQRVAIA
RAIIKSPKILLLDEATSALDAESERVVQDALDKVMVNRTTVVVAHRLSTVKNADVIAVVKNGVIVEKGRHETLI
NV
>XP_020406095.1_ABCB9_Zm
MDAAGGAKGRDGVDKKEVGSGSGNGGGDAGKKVPFTGLFRYADGTDVLLMLLGTVGALANGVSQPVMTVI
FGQVINAFGDATIDDVLNRVNQAVLNFVYLGIATAVVSFLQVSCWTMTGERQATRIRSLYLKSVLRQEIAFFD
VEMTTGQIVSRMSGDTVLVQDAIGEKVGKFQQLVATFIGGFVIAFVKGWLLSLVMLACIPPVVIAGGIVSKML
AKISTKGQASYSDAGNIVEQTLGAIKTVVSFNGEKQAIALYNKLIHKSYKAAVEEGITNGFGMGSVFCIFFSSYG
LAIWYGGKLVLSKGYSGGDVINILFAIMTGAMSLGNATPCMAAFAEGQSAAYRLFTTIKRKPEIDPDDQTGKQ
LEDIKGDVDLNDVYFSYPARPEQLIFDGFSLHVSSGTTMAIVGESGSGKSTVISLVERFYDPQAGEVLIDGVNIK
SLQLDWIRGKIGLVNQEPLLFMTSIKDNITYGKEDATIEEIKRAAELANAANFIDKLPNGYDTMVGQRGAQLSG
GQKQRIAIARAIIKNPRILLLDEATSALDVESERIVQEALNRIMLDRTTLVVAHRLSTVRNADCISVVQQGKIVE
QGPHDELVMNPDGAYSQLIRLQESKEEEEQKPERHVSDSRSKSRSLSLKRSISRGSMGNSSQHFLTLPIGMPGSV
ELLEGNDENWEEDEKEQARDGDREAPKKAPMGRLASLNKPEVPILVLGSLAAGVHGVLFPLFGLMISNAIKTF

YEPPHQLKKDASFWGLMCVVLGIVSILSIPVEYFLFGVAGGKLIERIRALSFRSIVHQEVAWFDDPKNSSGALGA
RLSVDALNVRRLVGDNLALAVQVTSTLIAGFVIAFVADWKLTLIILCVMPLSGVQGYAQVKFLKGFSQDAKIL
YEDASQVATDAVSSIRTVASFCAEKRVTTIYDDKCEASKKQGVRTGMVGGLGFGFSFLMLYLTYGLCFYVGA
QFVRHNKSTFGDVFKVFFALMLATIGVSQTSALASDSTKAKDSAVSIFALLDRKSQIDSSIDEGSTLDEVRGDID
FRHVGFKYPSRPDIQIFSDFTLHIPSGKTVALVGESGSGKSTVISLLERFYNPDSGTISLDGVEIKSLKVNWLRDQ
MGLVGQEPILFNDTIRANIAYGKHGEVTEEELVKVARAANAHEFISSLPQGYDTTVGERGVQLSGGQKQRVAI
ARAILKDPRILLLDEATSALDAESERIVQDALDNVMVGRTTVIVAHRLSTIKSADIIAVLKDGVIVEKGRHEALM
NIKDGFYASLVELRSASS
>XP_015168170.1_P_ABCB9-l_St
MARGGDEKVPFYKLFAFANRNDIILMLLGILGAIASGVSKPLMSLIFGDLVDSYGTSNQSNIRDKVSGYLICVQI
SLKFVYLGIGSGIASVLQVACWVITGERQATRIKCLYLKTILRQDIEFFDTQSATGEVIERMSGDTILLQEAMGE
KVGNFIMHMSTFIGGFVVAFIKEWHLTLVLLTTIPAIAISFICAALVLSKMSGSGQAAYADAGKVVEQTVGGIRT
VVSFTGENLSIIDYNSKLENAYKPTVNQALASGIGLGTTLMVSLFSYGLAIWYGAKLIIDKNYSGGDIITVLFSA
MLGGSSLGQASPSLSAFSAGQAAAYKIYETIKRTPKIDPYDPSGIQLEDIKGEIELKDVYFKYPARPDVQIFSGFS
LYIPSGKTAALVGQSGSGKSTVISLLERFYDPEAGEILIDGVDIKKFQLKWLRQQMGLVSQEPVLFATTIGENIM
YGKENASVEEIRNAIKLANAAKFIDKLPKGLDTMVGGHGTQISGGQKQRIAIARAILKDPRILLLDEATSALDVE
SERIVQDALSNIMINRTTVVVAHRLTTIRNADLIAVVNLGKLVEQGTHDELIKDPDGAYSQLVQMQQKNKHVE
NTKGKEIEGSNAQKRLSCSKNPSGRSRRFSISSRKFASKGASSRFSLAYDLGVTAAVDFHESILRDDGAESSEYI
VNSSRNVSTRKLMSLAYLNKPEVPIMLVGTVAASINGAVYPVFGLLISTAIKIFYESHHELRKDSRFWALMFVVI
GVVIMIVAPLQNYAFGIAGAKLIQRIRSMTFAKLVYQEISWFDDPANSCGAIGARLSSDASTIRNLVGDALATIV
QNISTVATGMVIALIANWILALIMLAIMPLLALQGIIQIKLLQESNAEAKVANEEASQVANDAIGSIRTVASFCAE
EKVMEMYQRKSEAPLKRGAKNGLVGGVGLGFSSFVLFSLYALTFYLGAILVKHDKAKFSDVFKVFFALSMASI
GLSALGNLPSDLSKSKGAAASIFEILDRKPRIDSSSSEGIMLDVIEGNIELQHISFKYPTRPDMQIFRDLSLSIPAGK
TVALVGESGSGKSTVISLIERFYDPDQGYIYLDGVELRKLNLRWLRQQMGLVGQEPILFNETISSNIAYGRQGEV
TEEEIISVAKASNAHNFISSLPNGYKTTVGERGVQLSGGQKQRIAIARAILKDPKILLLDEATSALDTESERIVQE
ALDRVMVNRTTVVVAHRLTTIKNADVIAVVKNGVVAEKGTHDVLMNKTQGVYASLVALQTGAT
>XP_021606229.1_ABCB11-l_Me
MAEESGLVGEVTTEQAKTSMSDPQVVELQEAIEKSKKKKESTNTLPFYKLFSFADSIDYLLMFVGIIAAAGNGV
CMPLLTILFGDSVNAFGDNSVNTKGVVHEVSKVSLKYVYLALGSSVAGFLQVACWMVTGQRQAAKIRSLYLS
TILKQEIGFFDKEIDTGEILGSMSGDMVLIQDAMGEKVGKFLQLILTFISGFVIAFIKGWKLTLVMLSSIPLLVLSG
ALMSIYISKLASRGQTTYSLAATVVEQTIGSIRTVASFNGEKQAIDKYNKSLTKAYKSGVQESMAAGLGFGVVT
FIVFSNYALAVWFGAKLVINEGYKGGDIISIVFVVLTGSLSLGQASPCLTAFSAGQAAAYKMFDVIGRKPQIDA
YDTNGLTLNEIHGDIEFRDIYFSYPARPEEQIFSGFSISIPSGSTAALVGESGSGKSTVISLIERFYDPQAGEVLIDG
VNLKEFQLKWIRQKIGLVSQEPVLFSCSIKENIGYGKENATTEEIVAAAELANAAKFIDKLPQGFDTMVGEHGT
QLSGGQKQRVAIARAILRDPRILLLDEATSALDAESERIVQEALDRIMINRTTVVVAHRLSTVRNADMIAVIHRG
TIVERGSHLELTKDPDGAFSQLIRMQEMSPLPQNIALNDAERTEIIVDSERHSSQLFSILRSLSQGSSGIGNSSRHS
FSVPFGVPTGINVPETALAEPSITLVSASSPPPPKVPLRRLAYLNKPEIPALLLGSLAAATNGVILPLFGVLVSSMI
KTFFEPAEQLRKDSRFWAFMFLGLASMSLLVNPMRSYFFAVGGCKLIRRIRSMCFEKVIYMEVGWFDEPGHSS
GAIGAKLSADAASVRSLVGDALGLLVQNIATAVAGLVIAFEANWQLAFIILIMLPLLGLNGYVQMKFIEGFSAD
AKKMYEEASQIANDAIRSIRTIASFCAEEKVMALYKKICEGPVRTGIREGLISGFGFGLSFFLLYSVYAASFYAG
AQLVEAGNTTFVEVFRAFCALTMAAVGVSQSSSLAPDASKAKGAAASVFAILDQKSKVNSNDDSGIVIEDLKG
QIEFQHVSFRYPIRPDIQIFKDLSLAIQAGKTVALVGESGSGKSTVISLLQRFYDPDSGHITVDGIEIQNLQVKWL
RQQMGMVGQEPVLFNDSIRANIIYGKEGNATEAEILAASELANAHKFISSLRQGYETRVGERGVQLSGGQKQR
VAIARAIVKAPKILLLDEATSALDAESERLVQDALDRVVENRTTVVVAHRLSTIKNADMIAVVKNGVIAEKGK
HETLMNMKEGVYASIVALHTSASSQKAKLT
>XP_003607429.1_ABCB11_Mt
MATGLEGDIASLQPVEDEDRKQDSEKSKDKDEITNTVSLYKLFSFADPLDCLLMLMGTVGAIGNGISLPLMVLI
FGTMINAFGESTTSKVVDEVSKVSLKFVYLAAGSFVASCLQVTCWMITGERQSARIRGLYLKTILRQDVSFFDK
ETNTGEVVGRMAGDTVLIKDAMGEKVGQFIQFVATFIGGFVIAFTKGWLLTVVMLFSIPLLVLSAAVTSKVIAK
ASSTGQAAYSESASLVEQTIGSIRTVASFTGEKQATTKYNHSLIKVYNTTVQEALASGVGFATIFFVFISSYSLAV
WFGGKLIIEKGYTGGDVMTVLFAILTGSMCLGQTSPSLSAFAAGQAAAFKMFETIKRKPEIDAYETTGRKLDDI
RGDIELIEVCFSYPTRPDELIFDGFSLSLQSGTTAALVGQSGSGKSTVISLIERFYDPTNGEVLIDGISLKEFNLKWI
RQKIGLVSQEPVLFTCSIKKNISYGKDGATVEEIRAAAELANAAKFIDKLPQGLDTMVGEHGIQLSGGQKQRVA
IARAILKDPRILLLDEATSALDAESERIVQEALERIMINRTTIVVAHRLSTIRNVDTIAVIRQGKIVERGSHVELTK

DANGAYSQLIRLQEMKGSEQNVANDSNKSNSIMLSEKRSSEISLSSRFIRQVSSGNSGRHSFSASCGAPTTDGFL
ETADGGPQASLSTVSSPPEVPLYRLAYFNKPEISVLLMGTIAAVLNGAIMPTFGLLISKMISIFYKPADELRHDSK
VWAMVFVAVGVASLLVIPCRYYFFGIAGGKLIQRIRKMCFEKVVYMEVNWFDEVEHSSGALGARLSTDAALV
RALVGDALGLLAENIATSITGLVIAFEASWQLAFIVLALAPLLGLDGYVQVKFLKGFSADAKKLYEEASQVAN
DAVGCIRTVSSFCAEEKVMELYEQKCEGPIKKGIRRGIISGLGFGLSCFLLYAVYACCFYAGARLVEDGKSTFSD
VFLVIFALGMAASGVSQLGTLVPDLINAKSATASIFAILDQKSQIDSSDESGMTLEEVKGEIEFNHVSFKYPTRPD
VQIFKDLCLNIHSGKTVALVGESGSGKSTVMSLLQRFYDPNLGHITLDGKEIQRLQLKWLRQQMGLVSQEPVL
FNDTVRANIAYGKGGDATEAEIVAAAELANAHQFISSLQKGYDTIVGERGIQLSGGQKQRVAIARALVKNPKIL
LLDEATSALDAESEKVVQDALDCVMVDRTTIIVAHRLSTIKGADLIAVVKNGVISEKGKHEALLHKGGDYASL
AALHTSASTS
>NP_001346368.1_ABCB21_Zm
MPESWRPAEADALQPAAGTASASGPSARSPGNGAKGRGAGEPPGAAATRVPFHRLFAFADSADVALMLLGAL
GAVANGAALPFMTVLFGNLIDAFGGALSVHDVVSRVSMVSLDFVYLAMASAVASFVQVTCWMITGERQAAR
IRNLYLKTILRQEIAFFDKYTSTGEVVGRMSGDTVLIQDAMGEKVGKFVQLLVTFFGGFIVAFAQGWLLTLVM
MATIPPLVLAGAVMSNVVTKMASLGQAAYAESSVVVEQTIGSIRTVASFTGEKRAVDKYNMSLKNAYKSGVR
EGLATGLGMGTVMVLLFCGYSLGIWYGAKLILEKGYTGAKVMNVIFAVLTGSLALGQASPSMKAFAGGQAA
AHKMFETINRTPEIDAYSTTGRKLEDVRGDIEFRDVYFSYPTRPNEQIFKGFSLAIPSGTTIALVGQSGSGKSTVIS
LIERFYDPQLGDVLIDGVNLKEFQLRWIRSKIGLVSQEPVLFAASIKENIAYGKDNATDQEIRAAAELANAAKFI
DKMPQGFDTSVGEHGTQLSGGQKQRIAIARAILKDPRILLLDEATSALDAESERIVQEALDRVMTNRTTVIVAH
RLSTVRNADTIAVIHQGTLVEKGPHSELLRDPEGAYSQLIRLQEANQQNNGKVDANARPGKQISINKSASRRSS
RDNSSHHSFSVPFGMPHGIDIQDGSSNKLCDEMPQEVPLSRLASLNKAEIPVLILGSIASVISGVIFPIFAILLSNVI
KAFYEPPHLLRRDSQFWASMFLVFGAVYFLSLPVSSYLFSIAGCRLIRRIRLMTFEKVVNMEVEWFDHPENSSG
AIGARLSADAAKVRGLVGDALQLVVQNSSTLVAGLVIAFVSNWELSLIILALIPLIGLNGWIQMKFIHGFSADA
KMMYEEASQVANDAVGSIRTVASFSAEEKVMDLYKKKCEGPLRTGIRTGIISGIGFGVSFFLLFGVYAASFYAG
ARLVEDRKTTFPKVFRVFLALAMAAIGVSQSSTLTSDSSKAKSAASSIFAIVDRKSRIDPSEDAGVTVEALQGNI
VFQHVSFKYPTRPDVQIFRDLCLTIHAGKTVALVGESGSGKSTAISLLQRFYDPDVGHILLDGVDIQKFQLRWL
RQQMGLVSQEPALFNDTIRANIAYGKDGQATESEIISAAELANAHKFISSALQGYDTVVGERGAQLSGGQKQR
VAIARAIVKDPRILLLDEATSALDAESERIVQDALDRVMVNRTTVIVAHRLSTIQNADLIAVVRNGVIIEKGKHD
ALINIKDGAYASLVALHSAASS
>XP_006594001.1_ABCB21_Gm
MAENIDLYGDSDIKQDSKSKVKDESAKTVPLYKLFSFADPLDHLLMFVGAVGAIGNGISMPLMTLIFGNMINAF
GATENSNEVVDEVSKVSLKFVYLAVGTFFASLLQLTCWMITGERQAARIRGLYLQNILRQDVSFFDKETRTGE
VVGRMSGDTVLIQDAMGEKVAQFIQLMTTFVGGFVIAFSRGWLLTLVMLSSIPPLVLCGSMLGLIITKASSRAQ
AAYSIAASIVEQTIGSVRTVASFTGEKQAIDKYNQSIIKAYRAGVQEALATGLGFGSLYFVFNCSYSLATWFGA
KMVIEKGYTGGEVVTVIMAVLTGSMSLGQASPSLSAFAAGQAAAFKMFETIKRKPEIDAYDTTGRQLDDIRGD
IELREVCFSYPTRPDELIFNGFSLSIPSGTTTALVGESGSGKSTVVGLIERFYDPQAGEVLIDSINLKEFKLKWIRQ
KIGLVSQEPVLFTCSIKENIAYGKDGATVEEIRAAAELANAAKFIDKLPQGLDTMVGEHGAQLSGGQKQRVAIA
RAILKDPRILLLDEATSALDAESEKIVQEALNRIMINRTTVIVAHRLSTIRNADSIAVMHQGKIVERGSHAELTRD
PIGAYSQLIRLQEVKRSGQNVANETDKLEGTAHFGRQSSQRSFLQAISQRSSEVGSSGRNSFSESHAVGFLEPAG
GVPQTSPTVSSPPEVPLYRLAYLNKPETPVLLAGSIAAIINGVLLPIVAIFMSKMISIFYEPADELRKDSKLWALLF
VVLGVVSFIMPPCRFYLFGVAGGKLIKRIRKLCFEKVVHMEVSWFDEAEHSSGAIGARLSSDVAAVRALVGDA
LGLLVQNIATAVGGLVIAFEASWQLALIMLALAPLLVLNGYVQFKFLKGFSANSKKLYEEASQVANDAVGSIR
TVASFCSEKKVMKLYQEKCEGPIRTGIRRGIISGISYGVSFFMLYAVYACSFYAGARLIEDGKSTFSDVFRVFFA
LSMTAMGISQSGSLVPDSSNSKSAAASVFAILDQKSQIDPSDDSGLTLEEVKGEIEFNHVSFKYPTRPDVQIFRDL
SLTIHSGKTVALVGESGSGKSTVISLLQRFYDLDSGHITLDRNEIQRMQIKWLRQQMGLVSQEPVLFNDTIRANI
AYGKGGDATEAEIIAAAELANAHNFTCSLQKGYDTIVGERGIQLSGGQKQRVAIARAIVKNPKILLLDEATSAL
DAESEKVVQDALDRVMVDRTTIVVAHRLSTIKGADLIAVVKNGVIAEKGKHEALLNKGGDYASLVALHTSAS
TS
>XP_015626984.1_ABCB9_Os
MAGGGGGGGDGGGGAGAGGGGGGGGAKRVPMRRLFTFADRLDAALMAVGGVAAVANGVAMPFLAFLIGEL
VDAFGAADRAHVVHVVSKISLRFTYVAIGSGIAGFLQVSCWMVTGERQAARIRGLYLEAILRQDITFFDLETST
GEVTERMSSDTVLIQDAIGEKVGKFLQLLSTFLGGFIIAFARGWLLSLVMLSSIPPVALAAAAMSIAISKLANRS
QLAYAEAGKLVEQTIGSIRTVVSFTGERRATDKYNEFLKISYRSAVHQGAAMGLGIGSVMFIVFCSYGLAVWY
GAKLIIEKGYTGGYIINVLMAIMSGAMALGQSSPCLNAFASGQIAAYKMFATINREPEIDASDRSGLVLENFVG

DVEFKDVHFSYPARPEQLIFTGFSISIPSGMTMALVGESGSGKSTVISLVERFYDPQSGEVLLDGVNMKLLNLSR
IRQKIGLVSQEPILFTTTIRENIEYGKKDASEEEIRRAIVLANAAKFIDKLPNGLDTMVGEHGTQLSGGQKQRIAI
ARAILKDPRILLLDEATSALDAESEHVVQDALNNIMVNRTTIIVAHRLSTVRNADTISVLHRGQLVEQGPHAELI
KYSNGAYYQLLQLQEVNARRNGTYELDPNRLSDVANRLSDVANRLSDAANRLSDAGNFVSRHSIRKLSFERS
MSRHSSLGGSRRNSQTYALTEDEIEGCDDTKSGKNVLRRLLHLHKPETAILLLGCIAASANGAILPVFGLLLSSA
INAFYEPPHKLRKDSVFWAEIYVILGVVSIFIIPVQHTLFNMAGGKLIERIRALSFSRVVYQDIGWFDDPLNSSGA
IGARLSADAASVKSIAGDVLSLIVQSISTALVGIVIAMIANWKLAFIVLCFVPCVFAQSYAQSRLMRGFGADAKE
MYEQASTIASDAISNIRTVTSFCVGEKIIESYRNKCKGPVKKGVRQGAISGVGYGFSFALLFCFYAVSFYVGARF
VHNGTADVGEVFKVFFALTMMAVGVSQSSSLARDFSKVQDAAASIFKIIDRKSKIDASSDDGMAPEKIEGNIEF
QHVSFKYPARTDVQIFTNLCLRIPSGKTVALVGESGSGKSTVVALLERFYDPDSGAIFLDGMDLKTLKLTWLRQ
QIGLVGQEPVLFNGTIRANIAYGKQDQVSEEEIVAVAEAANAHRFISSLPHGYDTSVGERGVQLSGGQKQRIAI
ARAILKDPKVLLLDEATSALDSESERIVQEALDRVMVGRTTVIVAHRLSTITGADKIAVIKNGVVAEEGRHGRL
LRLPGGAYASLVALQSSSS
>XP_003601392.3_ABCB11_Mt
MSSNMKIDENLDGESITLESTLSETHAPIAIHETIQRETENQQDSKTSITKGKTTNVVPFYKLFSFADSLDHVLMF
VGTIGAIGNGLATPLMNVVFGNLIDAFGRSTSPGEVVHDVSKVALNFVYLAVGSFVGSFFQVSCWIVTGERQA
SRIRNLYLRAILRQDTSFFDMEETNTGEVVGRMSSDTILIQDAMGEKVGQLIQSVATFIGGFVIAFVKGWLLTLV
LLSSIPPLVFASAVMSIVIAKVASRRQVTYSEAETVVEQTLSSIRTVASFTGEKQAIAKYNQSLAKAYKSGVQEG
LVSGFGIGSVYFIVFCAYGLAIWFGGKLVVEKGYTGGNIMTVIFAIMTGSLSLGQASPSLSALASGRAAAFKMF
ETINRKPDIDAYETTGQQLDDIGGDIELREVSFSYPSRPDQAIFKGFSLSIPRGTTAALVGQSGSGKSTVINLIERL
YDPQAGQVLIDGINVKEFQLKWIRQKIGLVSQEPVLFTGSIKENITYGKDGSTEKEVREAADLANASGFIDKFPQ
GLDTMIGERGMQLSGGQKQRVAIARSILKDPRILLLDEATSALDVESEKIVQEALDKIMINRTTVIVAHRLSTVR
NAATIAVIHQGKLVEKGSHVELTKDPDGAYSKLISLQETEKEAEVQNVATDSDRPENISYSSNQRFSHLQTISQV
GNSGRHSFSVSHALSTTIVPLETSGWEVEVPPLGTSQQPPPPKVPLRRLAYLNKPEIPVLLIGTMAAVVNGAILPL
FGLMIAKMVNTLYEPADELHEDSKFWALIFVVLGVSSFLIFPTRSYFFSIAGEKLVKRVRLLCFEKIIRMEMSWF
DETENSSGALAAKLSTNAATVRGLVGDALGLLVQNIATAIAGLVVAFQANWSLALIILGLLPLLGLNGYLQMK
FIQGFSADAKKLYEEASQVANDAVSNIRTVASFCAEEKVMDLYQKKCEAPIKAGIKQGIISGVGFGMSFLLLFL
VYACSFYAGAKLVGDGKTSFKEVFLVFFTLNMTAVGISQSSSLAPDSAKAKCAAMSILAIIDRKSKIDPSDDSGL
ELEDVKGEVEFHHVSFKYPSRPEVQIFRDFCLTIHSRKTVALVGESGSGKSTVISLLQRFYDLDSGHITVDGIEIQ
KLQVKWLRQKMGLVSQEPVLFNDTVRANIAYGKGKDATEAEIIAAAKMANAHKFISSLQQGYDTVVGERGSR
LSGGQKQRVAIARAILKNPKILLLDEATSALDAESEKVVQDALDRVMVDRTTIIVAHRLSTIKGADLIAVVKNG
VITEKGNHETLINKGGHYASIVDSNH

>XP_002275169.4_P_ABCB11-l_Vv
MAVENSLDGDIYTQQTRASTRQTPAVETVKIPENAGNRQDSEKRKATQGISTSTVPFYKLFSFADSWDYLLML
VGTVTAVGNGMCLPAVALLFGELMDAFGKTVNTNNMLHEVSKLCLKFVYLSSGAAVASFFQVTCWMVTGE
RQATRIRSLYLKTILRQDIAFFDKETKTGEVVGRMSGDTVLIQDAMGEKVGMVIQLAATFIGGFFVAFFKGWIL
VLVLLSCIPPLVASSAVMTILLAKLASQEQTSYSVAASVVEQTIGSIRTVISFTGEKQAIAKYKKSLTKAYDSAV
REGLATGLGLGSVMFIVFCIFALAVWFGAKLIINKGYSGGNVVGVIVAVLTASMSLGQTSPCIKAFAAGQAAAF
KMFETINRKPEIDAYDTKGLKLDDISGDVELRDVYFSYPARPDEQIFSGFSISIPSGTTTALVGQSGSGKSTVISLV
ERFYDPQAGEVLIDGINLKDFQLRWIRQKIGLVNQEPVLFASSIKDNIAYGKDDATIEEIRAAAELANAAKFIHK
LPQGLDTMVGEHGMHLSGGQKQRVAIARAILKDPRILLLDEATSALDLGSERIVQEALDRVMMNRTTIIVAHR
LSTVRNADMIAVIHQGKIVEKGSHTELLRDPHGAYHQLVQLQEISSESEQHDESWESFGARHHNRFPFPFSFGV
SPGINMLETAPAKPNSEPLKHPTEGLVWRLACLNKPEIPVLLLGIVAAIANGLILPAFAVLFSTIIDNFYESADKL
RKESKFWALMFFILGVASLLITPTRTYLFAVAGCKLIKRIRSMCFEKVVHMEVGWFDKAENSSGAIGGRLSAD
AASVRSLVGDALALVVQNIATVIAGLAAAFEANWLLALIILVFLPLIGINGCIQLQFTKGFSGDAKKRYEEASQV
ANEAVGNIRTVASFCAEEKVMQLYQKKCEGPAKTGMTRGLISGLGFGLSFFFVYFIYAVTFYAGARLFRDGKT
TFSKILRVFFALSMVGLGVSQSGSYAPDASKAKSCAASIFAILDQISEIDSSGRSGKRLKNVKGDIKFRHVSFRYP
TRPEIQIFRDLCLTIRSGKTVALVGESGCGKSTVISLLQRFYDPDSGRITLDGADIQKLQLRWLRQQMGLVSQEP
TLFNDTIRANIGYGKEGNATEAEIIAAAELANAHHFISSLQQGYDTAVGERGVQLSGGQKQRVAIARAVVKGP
KILLLDEATSALDAESERVVQDALDRIMVGKTTLVVAHRLSTIKGADLIAVVKNGLIAEKGNHESLMNIKNGR
YASLVALHATASSQ
>XP_002439240.1_ABCB4_Sb
MSESSRAFHLDAPSSSSSSSSTAAAAGDRHHHHGKSAASASTTGGGSVPFHRLFAFADAADAALMSLGTLGAL

ANGAAMPLMTVLFARLIDAFGGAADTRDVVARVSNVSLQFIYLAVASAVASFVQVASWMITGERQAARIRGL
YLGAILRQEVAFFDQRATTGEVVGRMSGDTVLIQDAMGEKVGKCIQLLVAFAGGFAVAFAQGWLLALVMLA
TIPPLVLAGALMSSVVARMASLGQAAYADAAGVVDQTIGSITTVASFTGEQRAVEKYSSSLKRAYSSGVWEGL
AAGVGMGIVMVLLFCGYSLGIWYGAKLILDKGYTGAQVMNVIFAVLTGSLALGQASPSMKAFAGGQAAAYK
MFETINREPEIDAYSTAGRKLDDIQGDIEFRDVYFSYPTRPDEQIFSGFSLTIQSGTTVALVGQSGSGKSTVISLIE
RFYDPQLGEVLIDGVDLREFQLRWIRSKIGLVSQEPVLFTASIRDNIAYGKYNATDEEIRAAAELANASKFIDKM
PQGFATSVGEHGTQLSGGQKQRIAIARAILKDPRILLLDEATSALDTESERIVQEALDRVMTNRTTVIVAHRLST
VRNAGTIAVIHRGSVVEKGSHHDLIRDPEGAYSQLIQLQEASHASEGANYQNKSNRKGDSGIHLGKQMSTNQS
PSQRSPQNNSSNHSFSVSHGVPLEIDVQNSSSKNIDEEIQHEVPLSRLASLNKPEIPVLILGSIASAVSGMIFPIFAIL
LSNVIKAFYEPPRILRKDAEFWSSMFLVFGAVYFLSLPLGSYLFSVAGCKLIRRIRLMTFEKVVNMEIEWFDYPE
NSSGAIGARLSADAAKVRGLVGDALQLVVQNLATLVAGLVIAFVSNWELSLIILALIPLIGLNGWIQMKFIQGFS
ADAKLMYEEASQVATDAVSSIRTVASFSAEEKVMDLYKKKCEGPLRAGIRTGITNGIGFGVSFFLLFGVYAASF
YAGARLVENDKTTFPKVFRVFLALSMAAIGVSHTSTLTSDSSKAKSAVSSIFAIMDRKSRIDPSDDAGVTLEPLS
GNIEFRHVRFRYPTRPDVQIFQDLCLTIQSGKTVALVGESGSGKSTAIALLQRFYDPNAGHILLDGVDIQKFQLR
WLRQQMGLVSQEPSLFNDTIRANIAYGKDGQATELDIVAAARLANAHKFISSLHQGYDTMVGERGAQLSGGQ
KQRVAIARAIIKDPKILLLDEATSALDAESERSVQDALDRVMVNRTTVIVAHRLSTIQGADVIAVVKDGVIVEK
GRHDALIKIEGGAYASLVALHSAAPS
>XP_021311281.1_ABCB11_Sb
MDATASRAGENDDDDKKQGAAPAKKVSLLGLFRYADRLDLLLMAVGTVGALANGVAEPLMTILFGNVIDSF
GDSTSQDIVRSVRKVVLDFVYLGIGAAVVSFLQVSCWTMAGERQSARIRSLYLNAVLRQDIAFFDTELTTGQA
VSRMSSDTLVIQDALGEKAGKLIQLSSAFFGGFIIAFTKGWLLTLVMLTSLPLIAIAGVVSAQFLTNISSKKLTSY
GDAGDTVEQTIGAIRTVVSFNGENKAVAMYKNLIKKAYRTDILEGLINGFGMGSVFCILFSSYGLAFWYGGKLI
ADKGYTGGKIITVLFAVLTGAMSLGNATPSVSSIAQGQSAAYRLFETIERKPEIDSGDTRGVVLEDMKGDVELK
DVHFCYPARPDQLILCGLSLQVASGTTMAIVGESGSGKSTVISLVERFYDPHDGEVLIDGINIKNLRLSCIREKIS
LVSQEPLLFMTSIKDNIMYGKGDTTIEEVKRAAELANAANFIDKLPDGYDTMVGPHGAQLSGGQKQRIAIARAI
LKDPKILLLDEATSALDVESERIVQEALNRIMVERTTLVVAHRLSTVRNVDCITVLRQGKIVEQGPHDVLVKDP
NGAYSQLIRLQETRADERRKTADSGVPDSRSKSTSLSLRRSMNKDSFGNSNRYSFKNPLGLSVELHENRIIGGEE
TEGLSDVVVLKKAPIGRLFKLNMPEVPVLLLGSIAASVHGVVFPLFGILMSGIIKSFYEPPDKMRKDTSFWALIS
VVLGITCLISVPAQYFLFAVAGGKLIERIRALSFQSIVRQEIAWFDNASNSSGALGTRLSVDALNVRRIAGDNLA
LIMQSIATLTTGFVIAFAADWRLALIITCVIPLVGAQGYAQVKFLKGFSEDAKEMYEDASQVATDAVGSIRTVA
SFCAEKRVVATYNEKCEALRKQGIRSGIVGGLGYGFSFLMLYFTYGLCFYVGAQFVRQGKTTFPDVFKVFFAL
VLAAIGVSQASALASDATKARDSAISIFSILDRESKIDSSSDDGMTLENVTGNIDFNNVSFKYPLRPDVQIFSDFT
LRIPSGKTVALVGESGSGKSTIIALLERFYDPDSGRISLDGVEIKSLKISWLRDQMGLVGQEPVLFNDTIRANITY
GKHGDVTEEEVMAVAKAANAHEFISSLPQGYDTMVGEKGIQLSGGQKQRVAIARAIIKDPKILLLDEATSALD
AESERIVQDALDRVMVSRTTIVVAHRLSTIKGADMIAVLKEGKIVEKGRHEVLMRINGGAYAALVELRSKSE

>XP_021606674.1_ABCB21-l_Me
MNKEGAVNSSVPKISNLPDKSNEKVTNTVPHYKLFSFADSLDIMLMFIGTIAAFGNGICMPLMTILLGELIDSIG
KAASMTVVAHNVSEVSLKFVYLAVGSGLASFFQVACWMITGERQAARIRSLYLKTILRQDIAFFDKETSTGEV
VGRMSGDIVLIQNAMGEKVGNLIQLLASFLGGFIIAFTKGWLLTLVMLALIPPIVISGAIMNKVVSKLASRGLTS
YSLAANIVEQTIGSIRTVASFTGEKQAIDKYNKSLIRAYESGMQEGLAAGLGFGTLMFILFCSYGFAVWLGGKM
ILDKGYTGGEVINVIFALLTGSLSLGQTSPCMSAFASGRAAAVKMFEAINRKPEIDVFDTKRLKLKHIHGDIELR
DVYFSYPTRSHEQIFSGFSLSVPSGTTTALVGESGSGKSTVISLIERFYDPQAGEVRIDGVNLKEFQLKWIRENIG
LVSQEPVLFTSSIRDNIAYGKEAASIEEIRAAAQLANAANFINKLPKGLDTLVGEHGIQLSGGQKQRIVIARAVL
KDPRILLLDEATSSLDAESERTVQEALDRVMINRTTVMVAHRLTTVRNADVIAVMQKGNIVQIGSHSDLLKDP
DGAYAQLIQLQEFGEEPEQQVINYPDSSYHSIRVPLSASPRVSISQNVTAKPLGTSTSEKSKLPPQGPLRRLAYLN
SPEIPVLLLGAIAAVANGIILPIFGLLLANIIKTYYEKEDQLQKESRFWGFMFVLLGLVSLLAMPLSTYFFSIAGCR
LIKRIRSMCFEKVVNMEIAWFDEPEHSSGAIGARLSVDAAKMRGLVGDTFCLLIQNSATGIAGLVIAFLANWQI
ALVILSLLPLMGLSGYVQLKSMEGFNANTKKMYEEASQVASDAVSSIRTVAAYCAEEKVLQLYQRKCDGPLK
AGIRRGLISGIGFGLSFFFVFLVYAVSFYVGAYLVDHGKTTFTDVFRVFFALSMAALGISQSNSLAPDASQARSS
AASVFAILDQKSQIDPSDPSGMKIKKLMGIIEFRNVSFRYPTRPDIQIFQDLSITIHSGKVVALVGESGSGKSTLISL
LQRFYNPDSGQITLDGIEIQKLNLKWLRRKMGVVSQEPVLFNDSIRANIAYGKEGNATEAEIMAAAELANAHSF

ISGLEQGYDTTVGERGVQLSGGQKQRVAIARAIVKAPKILLLDEATSALDAESERVVQDALERVMVGRTTLVI
AHRLSTIKGADMIAVMKNGVIVEKGKHDTLMNMKNGIYASLMDPKPNAAQGNSDM
>XP_003541331.1_ABCB21_Gm
MAQDIALNRDSDSKEDSKSKAKDKTVKTVPLYKLFSFADPLDNLLMFLGTVGAIGNGVSIPLTILMFGNMINAF
GGTENSNVVDEVSKVSLKFVYFAVGTFLLSLLQLTCWMVTGERQATRIRGLYLKTILRQDVTFFDKETRTGEV
VGRMSGDTVLIQDAMGEKVGQFLQFIATFIGSFAVAFIKGWLLTVVMLSCIPPLALVGAVLGQVISKASSRGQE
AYSIAATVAEQTIGSIRTVASFTGEKQAIANYNQSLTKAYKAGVQGPLASGLGFGALYFVFTCSYGLATWFGA
KMIIEKGYTGGEVITVIVAVLNGSMSLGQASPSLSAFAAGQAAAFKMFETIKRKPEIDAYDTTGRQLDDIRGDIE
LREVCFSYPTRPDELIFNGFSLSIPSGTTTALVGESGSGKSTVVGLIERFYDPQAGEVLIDSINLKEFKLKWIRQKI
GLVSQEPVLFTCSIKENIAYGKDGATDEEIRAAAELANAAKFIDKLPLGLDTMVGEHGAQLSGGQKQRVAIAR
AILKDPRILLLDEATSALDAESEKIVQEALDRIMINRTTVIVAHRLSTIRNADSIAVIHQGKIVERGSHAELTKDPN
GAYRQLIRLQEIKGSEKNAANDTDKIESIVHSGRQSSQRSSIQSISQRSSGVGSSGCNSFSESHGVPATVGFLEPS
GGRPQAPPSTVSSPPEVPLYRLAYLNKPEIPFLLIGTIAAVGSGVILPILALFISKMISIFYEPVDELHKDSKHWALL
FVALGVVSFVMPPCRFYLFGIAGGKLIKRIRKMCFEKVVHMEVSWFDEAEHSSGAIGARLSSDAAAVRALVGD
ALGLLVQNIATAVAGLVIAFDASWQLALIILALAPLLALNGYVQLKVLKGFSADAKKLYEEASQVANDALGSI
RTVASFCAEKKVMKSYEEKCEGPIRTGIRRGIISGISYGVSFFMLYAVYACSFYAGARLVQDGKATMLDVFRVF
FALNLAAVGISQSGSLVPDSSNSKSAAASVFAILDRKSQIDPSDDSGLTLEEVKGEIEFKHVSFKYPTRPDVQIFR
DLCLTIHNGKTVALVGESGSGKSTVISLLQRFYDPDLGNITLDGTEIQRMQVKWLRQQMGLVSQEPVLFNDTIR
ANIAYGKGGDATEAEIIAAAELANAHNFTCSLQEGYDTIVGERGIQLSGGQKQRVAIARAIVKNPKILLLDEATS
ALDAESEKVVQDALDCVMVDRTTIVVAHRLSTIKGADLIAVVKNGVIAEKGKHEALLNKGGDYASLVALHTT
ASTS

>XP_021311344.1_ABCB11_Sb
MGDDGRGKGAEASAAGDGGGREEGAAAAGSRENSNSKPAAAVGRVPLYRLFAFADRTDALLMAVGAVAAV
ANGMAQPTMTFIFGDVIDAFGSSASPDVLHRVVKVIMNFVYLAIGAGLASTLQVSCWTITGERQAARIRALYL
KAILRQDIAFFDMEMSTGQVVERMAGDTFLIQDAIGEKVGKSLQLLSTFVGGFIIAFVRGWLLALVMLSSIPPIAI
AGAIISKMMTRLSTRMQAKYGDAGNVVEQMLGSIRTVVSYNGEKQAIRTYNKFIRKAYESALQEGAVNGLGL
GSVMAILFCSYGLAVWYGSRLIVERGYNGGMVISVIMAVMIGAMSLGQATPSVTAFAEGQGAAYRMFKTIER
KPDIDIYDTTGVILEDVKGDVELKDVYFSYPTRSEHLVFDGFSLRVPNGTTMALVGESGSGKSTVISLVERFYDP
QAGEVLIDGVDIRKMNVGWIRGKIGLVSQEPVLFSTTIRENIAYGMENLTLEEIKGATELANAAKFIDKLPNGL
DTLVGERGTQLSGGQKQRIAIARAIVKNPRILLLDEATSALDMESERVVQEAMNRVMLERTTIIVAHRLSTVKN
ADVISVLQHGKMVQQGSHVELMKIPEGAYSQLIHLQETRQGADFSSVDPDIIVTNGFGSRSTNSKPRSQRISRQR
STSKGSSSFGHSGRQSFPTPLSVPDPMELDGSPDVEETTDKINRAPKKAPIARLFYLNKPEALVLALGSITAAMH
GVILPIYGILISTAIKVFYEPPEELLKDCRFWASMFVVLGACAFVLIPIEYFLFGLAGGKLVERVRSLTFQSVMRQ
EISWFDKPEHSSGTIGARLSTDAMNLRRLVGDNLALNVQTVSTVISGFTIAVVANWKLALIITVVVPFVGFQGY
AQMKFLKGLNRNAKLKYEEASQVATDAVGGIRTIASFSAEKKVMDAYEKKCEYPIKQGIREGIVGGLGFGFSF
LAFYFTYALCFYVGAKFVQQGKATFPEVFRVFFVLVLATSGISRTSAVGSDSTKANDAAASVFEILDRESKIDY
SCEDGITITSVRGEIGFQNVCFKYPSRPNVQIFKDLSLNIPYGKTVALVGESGSGKSTAIALLERFYDPDSGKILFD
DVELQTLKVSWLRQQVGLVSQEPVLFNDTIRSNIAYGKQGEASEEEIVAAAEAANAHQFISALPDGYNTIVGER
GIQLSGGQKQRVAIARAIIKDPKVLLLDEATSALDAESERVVQEALDHVMVGRTTVVVAHRLSTIRGADIIAVF
KNGAVAEKGRHEELMRIKDGTYASLVELSSSSS

>XP_015638148.1_ABCB4_Os
MAARGSEGGEEAEVEGKAGLHRLFRYADGVDALLMAAGAAGAAASGAAQPLMNLVFGEVVDAFGSGSRDD
VLHRVSKVCLKFFYLAIGSWFACFLQVACWMITGERQAARIRGLYLEAVLRQDIAFFEKEMTTGQVVERMSG
DTILIQDAIGEKVGKFIQLTATFVGGFVVSFAKGWLLSCVMLSSIPPIIIAGATMSWTISKLSTHGQSKYNEAGNV
VEQTIGAIRTVASFNGENRAIALYNKYIHSAYVSAVQESTATGLGFGFIMFMLFCTYGLAAWYGAKLIIDKGYE
GGQVVTVWMAFMTGAMSLGEATPCMSAFASGQAAGYRMMQTIERMPTINSSGTDGAVLENIKGDIELRNVY
FSYPSRPDQLIFDGFSLHVLNGITMAIVGESGSGKSTVINLVERFYDPQAGEVLIDGVNIKTLRLRWIREKIGLVS
QEPLLFATSIRENIVYGREDATTEEIMAATELANAAKFIENLPNGLDTMVGEHGAQLSGGQKQRIAIARAILKNP
KILLLDEATSALDMESERVVQEALNRIMQDKTTIVVAHRLSTIKDADIISVVQHGRVVEQGTHTELLKDPSGAY
SQLIQLQGATEELHKSGVGYQRSISTVRSVMSISKSRGRNASFKRSLSRGTSFGSTSVHLTTAAGMIVPESMHTE
VPSKVLDDNEEHKKVPLCRLISLNKPEIPVLLLGTAAAVVAGVLFPMLGLLISSSIKSFYEPPHQLKKDARFWTL
MYVAAGIVSLVSLPMENFLFGVAGGKLVERIRSLSFKRIVHQEVSWFDNPSNASGTIGARLSVDASNIRRLVGD

SLALIVRSSVTIIAGFIIAMVANWRLALVATVVLPLGGLQGFFQIKFLEGFSADAKVKYEEATQVAHDAVSSIRT
VASFCAENRIMKAYYKKCEAPVRQGIRQGIVSGLGFGISFFVLYSTYALCFYVGAKFMLDGKATFTEIFRVFFA
LLMATIGVSQTSAMGSDSAKAKASASSIFAMIDRESKIDSSSDDGMVLANVAGELELHHVCFSYPSRPDIQIFRN
LSLRIPSGKMVALVGESGCGKSTVIALLERFYDPDSGTVTLDGVDIKNLKVGFLRQQMGLVSQEPVLFNDTVR
ANIAYGKEGDATEEEIVAAARAANAHQFISALPGGYDTCAGERGVQLSGGQKQRVAIARAILKDPRILLLDEAT
SALDAESERAVQAALESVMVGRTTVVVAHRLSTIRGADVIAVLRDGEVVATGRHVELMAKKDGVYASLVEL
RMSSERAGDSKPS
>XP_014624945.1_ABCB4_Gm
MVAEAGLEADMTSTKTTGSTSDHPPAQGPENTQEIEYMQQDCKKNKMKGESNKTVPFYKLFSFADSWDCLL
MVVGAISAVGNGISMPLMTILIGDAIDAFGGNVDNKQAVVHQVSKASLKFASIGAGAFFAAFLQVACWVITGE
RQAARIRGLYLKAILRQDISFFDKDTNSGEVVGRMSGDTVLIQEAMGEKVGKFIQYVACFFGGTVIAFIKGWLL
SLALLSSLPLLVLSGSVMSFAFAKMASRGQTAYSEAATVVERTIGSIRTVASFTGEKQAIAQYNQYLIKAYRVG
VQEGVAGGFGFGLVRLFIYCTYALAVWFGGKMVLEKGYTGGQVISIFFAVLTGSMSLGQASPSLTAFAAGQA
AAFKMFETIKRQPDIDAYDTGGRLLDDISGDIELKEVCFSYPSRPDEQIFNGFSISIPSGTTAALVGQSGSGKSTVI
SLIERFYDPQAGEVLIDGINLREFQLKWIRQKIGLVSQEPVLFACSIKENIAYGKDGATDEEIRAAAELANAAKFI
DKFPHGLDTMVGEHGIQLSGGQKQRISIARAILKDPRILLLDEATSALDAESERVVQETLDRIMINRTTVIVAHR
LSTIRNADVIAVIHHGKVIEKGTHAELTKDPDGAFSQLIRLQKIKRESDQYDANESGKPENFVDSERQLSQRLSF
PQSFSLESSGRGIDSQRSFKISNAMPTSPDLFETSEGGPEVLPSAASNKPQEVSLLRIAYLNKPEIPVLLLGTVAAA
ATGAILPTVGLLLSHMINTFFEPADELRKDSKFWALIFVVLSVAAFIFIPLRSYLFAVAGSKLIKRIRLMCFEKIIQ
MEIGWFDKAENSSGALGARLSTDAASIRTLVGDALGLLVQDISTAITALVIAFDANWQLSLIVLVLVPLVLLNG
NLQMKSMQGFSTNAKKLYEEASQVASDAVGNIRTVAAFGAEEKVMELYQKKCVGPIQTGIRQGLVSGTGFGL
SLFFLFSVYACSFYAGARLVESGKTSISDVFRVFFALSMAAIAMSQSGFMTPAASKAKSSAASVFAILDQKSRID
PSDESGMTLEEVNGEIRFHHVTFKYPTRPNVLIFKDLSLNIHAGETIALVGESGSGKSSVISLLQRFYDPDSGQIT
LDGTEIQKLRIKWFRQQMGLVSQEPVLFNDTIRANIAYGKGDDATETEIIAAAELANAHKFISSLQQGYDTLVG
ERGIQLSGGQKQRVAIARAIVKSPKILLLDEATSALDAESERVVQDALDRVRMDRTTIVVAHRLSTIKDADSIA
VVENGVIAEKGKHETLLNKGGTYASLVALHISASSSS

>XP_021308688.1_ABCB9_Sb
MASASGEENTKAAGRVALHRLFVFADRTDAALMAVGAVAAVANGMAQPLMTLIFGDVIDAFGSGITDGVVH
RVVQVIMNFVYLAIGSGIASTFQVSCWTITGERQAARIRALYLKAILRQDIAFFDMEMSAGQAVERMAGDTFLI
QDAIGEKVGKSIQLLSTFIGGFIIAFTRGWLLALVMLSTVPPIVIAGAIVSKLMTGLSTRMQANYSDAGNVVEQT
LGAIRTVVSFNGENQAITRYNTFIRKAYQSSLQEGAVNGLGFGLIMTILFSSYGLAVWYGSKLIVERGYNGGMV
ISVIMAVIIGAMSLGQTTPSVTAFAEGQGAAYRMFKIIERKPNIDIDDSTGIILEDIKGDVELKDVYFSYPTRPEHL
IFDGFSLQVPSGTTMALVGDSGSGKSTVISLVERFYDPQAGEVLIDGVDIRRMKLGWMRGAIGLVSQEPVLFST
TIRENIAYGTENLTLEGIKRATELANAAKFIDKLPNGLDTMVGEHGTQLSGGQKQRIAIARAIMKNPKILLLDEA
TSALDMESERVVQEALNRIMVERTTIVVAHRLSTVKNADVISVLQHGKMVEQGSHVDLMKIPGGAYSQLIHLH
ETQQEAENVHPDMKVTNSFGFRSIDSKPRSQSISRRSTSKGSFSFGHSIPAPVGSPDPMETSDAPDIGEATDKVTS
SQKKASIGRLFHLNKPETFVLALGSITAVMHGIMFPIYGILISTAIKVFYEPPEELLKDSRFWASMFAVLGACTFV
LIPTEYFLFGLAGGKLVERIRSMTFQSIMRQEINWFDKPEHSSGSICARLSTDALNVKRLVGDNLALNVNTASTII
SGFTIAMVANWKLALIITVVIPFVAFQTYAQMIFLKGLNRNAKLRYEEASQVATDAVGGIRTVASFSAENKVM
DAYEKKCESPRRQGIKEGVVGGLGFGVSFLAFYLTYALCFYVGAKFVQQGTATFPEVFRVFFVLALATGAVSR
TSAVGADSAKASDSAISIFEILDHKSKIDYSSEEGVTITSVRGDIDFQNVCFKYPLRPNVQIFNDLSLRIPSGKTVA
LVGESGSGKSTVIALLERFYDPESGKIFLDDVELQTLKVSWLRQQVGLVAQEPVLFNDTIRANIAYGKQGGVSE
EEIIAAAKAANAHTFIAALPDGYNTIVGERGSQLSGGQKQRVAIARAIIKDPKLLLLDEATSALDAESERVVQEA
LDQVMVGRTTVVVAHRLSTIRGADIIAVLKNGAVLEKGRHEELMLVKDGTYASLVELSSSSA


>XP_006349962.1_P_ABCB11-l_St
MEEENVTGRGIDSMENEVLESSDGSNCARVSDKTEKQKVAAADKVPYYKLFSFADPVDHALMVIGMITAVGS
GICFPLMAVLFGELVDSFGMTVDSEKIVDEVSKVALKFVYLALGSGLATFIQVACWTVTGERQAARIRCLYLK
TVLRQDIGFFDQETNTGVIIESLSSDTLTIQDAIGEKVGKFIQVSATFLGGFVIAFIKGWRLALVLSSSIPPLVISSA
VLVILLAKLASRAQTAYSEAATVVEQTISSIRTVASYTGERRAISEYQNSLNKAYHSGVQEGLASGLGFGVFMFI
LYTSYALGIWYGAKMILEHNYTGGDVMNVIMATLIGSFTLGYASPCLHAFAAGKTAAFKMFQTINRKPVIDPY
DMNGQKPLDISGDIELKNIHFCYPARPQESIFDGFSVSIPKGTTTALVGRSGSGKSTVINLIVRFYDPQAGEVLID

GINIKEFQLRWIRGKIGLVSQEPVLFGSTIKDNIAYGKDDATLEEIKDAVQLANASKFIDKLPQGLDTRVGDHGN
QLSGGQKQRIAIARAILKDPKILLLDEATSALDAESERIVQETLDSVMINRTTVIVAHRLSTVKNADTIAVLQEG
KIVEKGSHLELMRNKEGAYVQLIQLQELSKYSGEQESNELDSEEIIINQQIPVTRSASRGSSRIENSSHHSSSISVS
AAEKAVGECHDPNSTVVLSKDKDNTICRLALMNKPEIPELLFGCIAAMVNALILPIFGVLLSNVIKTFYEPAHEL
RKHSRFWSLLFLGLGLATLLATPLRTFFFAVAGCKLIRRIRLMCFEKIVYMEVSWFDRKENSIGAIGSRLSTDAA
SVRGMVGESLALLVQNTSTAIAGLVIGLEASWQLSLIMIVMVPLIGLNGYLYMKYVSGFGSDAKKLYEDASQV
ASEAIGSIRTVASFSAEEKVVQLYKRKCEGPVRAGIKEGLVSAAGFGFSMFCLYSVYAASFYAGARLIESGKVT
FAEVFRVFYGLSLTATAISQSGGLAPDSTKAKTGASSIFALLDRQSKIDSSDNSGMTLENVMGSIEFRHISFNYPS
RPEVQVLNDLCLAISSGETVALVGESGSGKSTVISLLQRFYDPDSGLITLDGIEIQKLKVKWLREQMGLVSQEPI
LFNDTIRANIAYGKESDATEAEILAAAELANAHNFISGLQQGYETVVGERGIQLSGGQKQRVAIARAIVKCPKIL
LLDEATSALDAESEKVVQDALDRVRSGRTTVVVAHRLSTIKGADVIAVIKDGVIVEKGNHETLVNRQDGIYAS
LVSKSASTMK

>XP_019066857.1_ABCB11-l_Sl
MEEENASGRGTDNIENEVLESSDGLNCARVSDNTEKQKVVAADKVPYYKLFSFADPVDHALMVIGMITAVGS
GICFPLMAVLFGELVDSFGMTVDSEKIVHEVSKVALKFVYLALGSGLATFIQVACWTVTGERQAARIRCLYLK
TVLRQDIGFFDQQTNTGVIIESLSSDTLTIQDAIGEKVGKFIQVSATFLGGLVIAFIKGWRLALVLSSSIPPLVISSA
VLIILLAKLTSRAQTAYSEAATVAEQTISSIRTVASYTGEKRAISEYQNSLNKAYHSGVQEGLASGLGFGVFMFV
FYSSYALAIWYGAKMILEHNYTGGDVMNVIMATLTGSFTLGYASPCLSAFAAGKTAAFKMFQTINRKPIIDPY
DMKGQKPLDISGDIELKNVHFCYPARPQESIFDGFSVSIPKGTTTAIVGRSGSGKSTVISLIVRFYDPQAGEVLID
GINIKEFQLRWIRGKIGLVSQEPVLFGSTIKDNIAYGKDDATLEEIKDAVRLANASKFIDKLPQGLDTRVGDHGN
QLSGGQKQRIAIARAILKNPKILLLDEATSALDAESERIVQETLDSVMINRTTVIVAHRLSTVKNADTIAVLQEG
KIVEKGSHLELMRNKEGAYVQLIQLQELSKYSGEQESNELDSEEIIINQQIPVTRSASRGSARIENSSHHLSSMSV
SAAEKAVGECHDPNSTVVLSKGKENTICRLALMNKREIPELLFGCIAAMVNALILPIFGVLLSNVIKTFYEPAHK
LRKHSRFWSLSFLGLGLASLLATPLRTFFFAVAGCKLIRRIRLMCFEKIVYMEISWFDRKENSIGAIGCRLSTDA
ASVRGMIGESLALLVQNTSTAIAGLVIGLEASWQLSLIMIVMVPLIGLNGYLHMKYVSGFGGDAKKLYEDASQ
VASEAVGSIRTVASFSAEEKVVQLYKRKCEDPVRAGIKEGLVSAAGFGFSMFCLYSVNAASFYAGARFIESGK
VTFAEVFRVFYGLSLTATAISQSGGLAPDSTKAKTGASSIFALLDRQSKIDSSDNSGMTLENVMGNIEFRHISFN
YPSRPEVQVLNDISLAISSGETVALVGESGSGKSTVISLLQRFYDPNSGLITLDGLEIQKLNVKWLREQMGLVSQ
DPILFNDTIRANIAYGTETDATEAEILAAAELANAHNFISGLQQGYETVVGERGIQLSGGQKQRVAIARAIVKCP
KILLLDEATSALDAESEKVVQDALDRVRSGRTTVMVAHRLSTIKGADVIAVIKDGVIVEKGNHETLVNRQDGI
YASLVSKSASTMN

>XP_003603447.2_ABCB11_Mt
MLAKASLDGDITATEMTGSTNHHHLPVSGHENGQEMADMRQDSKKNKVKDQSKKTVPFYKLFSFADSWDYL
LMFVGTIGAVGNGVSMPLLTIIIGDAIDAFGGNVNTNQVVHLVSKVSLKFAIMGAGAFFAAFLQVACWMVTG
ERQAARIRALYLKAILRQDISFFDRETNSVEVVGRISGDTVLIQDAMGEKVGKFIQYVSSFLGGLVVAFIKGWLL
SLVLLSSLPLLVLSGSIMSFAFAKMASRGQAAYSEAATIVDRIIGSIRTVASFTGEKQAITQYNQSLTKSYIIGLQE
GLAIGLGLGLVRLFVYCSYALAVWFGGKMILAKGYTGGEVISVFFAVLTGSLSLGQASPSLTAFAAGQAAAIK
MFEIIKRQPNIDAYDTAGRQLDDISGDIELREVCFGYPSRPNEMIFDALSISISSGTTAALVGQSGSGKSTVISLIER
FYDPQGGEILIDNINLKEFQLKWIRQKIGLVSQEPVLFTCSIKENIAYGKDGATDEEIRAATELAKAAIFIDKFPH
GLDTMVGEHGAQLSGGQKQRIAIARAILKDPRILLLDEATSALDAESERVVQETLERIMINRTMIIVAHRLSTIR
NADIIAVIHQGKVVEKGTHDELTNDPDGAYSQLIRLQEIKKDSSEQHGANDSDKLETFVESGRESRPTALEGVS
EFLPSAAASHKSKTPDVPFLRLAYLNKPEIPALLIGTLAAVIGAMQPILGLLVSKMINTFFEPADELRKDVNFW
ALMFVFFSVASFVFQPLRSYFFAVAGSKLIKRIRLMCFEKIIHMEVGWFDKAENSSGALGARLSTDAASIRTLVG
DALGLLVQDIATVITALVIGFETSWQLSLIILVLLPLLLVNGHLQIKSMQGFSTDARKQYEEASQVANDAVGNIR
TVSAFCAEEKVMELYQKKCVVPVQTGKRQGIVSGVGFGLSIFFMFCVYACSFYAGAQLVKNGKTSISDVFQVF
FSLTMAAVAIAQSGFMAVGASKAKSSVASIFAILDQESKIDSSEESGMTLEDVKGDIEFHHVTFKYPTRPDVHIF
KDLSLTIHSGQTVALVGESGSGKSTVISLLQRFYDPDSGQIKLDGTEIQKLQLRWFRQQMGLVTQEPVLFNDTV
RANIAYGKGGNATEAEIIAAAKLANAHKFISSLQQGYDTIVGERGIQLSGGQKQRVAIARAIVKNPRILLLDEAT
SALDAESEKVVHDALDRLRVDRTTIVVAHRLSTIKGSNSIAVVKNGVIEEKGKHETLLNKSGTYASLVALHTTS
TTKGGTYCNRI

>XP_020398107.1_ABCB11_Zm
MAEESVVKASAGDAVKGGKVEESGEKMVTMAKVPFHSMFKCADRTDVLLMLAGMVGALGNGMSMVVMTI
IFGQMVDAFGGATPDTIVPRVSKAALNFIYLAIGTGFTSFIQISCWTMTGERQATRIRSLYLKSVLRQDMPFFDV
EMTTGQVVSSISADMTLIQGAIGEKVGKFVQLITTFFGGFVLAFIKGWLLTLVMLSTIPPFVVAAGIVAKMISKIS
SEGLASYSDAGDTVEQTIGSIRTVASFNGEKKAIALYNNFIKKAYNGAVKEGIVQGFGMGLLSFIYFSAFGLIIW
YGSKLSLTKGYSGADILNVMFAIMIGARNLGDATPCIASFEEGRVAAYRLFKTIKRRPEIDYGDNTGIVLEDIKG
EVELKDVFFSYPSRPDQLIFDRFSVHVSSGTTMAIVGESGSGKSTVINLVERFYDPQAGEVLIDGMNIKGFKLEW
IRGKIGLVNQEPVLFMTSIRENITYGKEDATLEEIKKAAELANAGFIENLPNGYDTTVGQRGAQLSGGQKQRIAI
ARAILKDPKILLLDEATSALDLESERIVQDALNRIMLGRTTLVVAHRLSTVRKAHCISVVSKGKLVEQGHHDDL
VKDPNGAYSQLIRLQEKQQENGRTSDARLSGSASKRSVSLRRSISRSSAGSSRHSLNLPLGVPGPTELLEYNFGQ
GDRQIENTDSKVPNKAPMGRLINLNKPEVAVLLFGSIVAAIDGAIFPTLGLAMASASKIFYESPDQQRKDSILWA
LLCVGLGAIAMISKIINSFLFAIAGGKLIERIRALTFQSIVHQEVAWFDHPENSSGALNGRLSIDALNVRRLVGDN
LALLVQSTATLTCGIVIAMVADWKLSLVILFVIPLVGLQGYAQVNFLRGFSQDAKIMYEEASQVATEAVGSIRT
VASFCAEKRVMDKYDQKCQASRDQGIRTGIVGGLGFGFSYLMLYSSSALCYYVGAKFVSQGKSTFGDVFKAY
FALVMAMIGVSQTNAMASDSAKANDSAISIFSILDRKSLIDSSSEEGSTLENVKGDIDFKHVGFKYPSRPDVQIFT
DFTLTIPSGKTVALVGQSGCGKSTVISLLERFYEPDNGSILLDRVEISSLKVSWLRDQMGLVSQEPVLFSGTIRDN
IAYGKHEEVTEEDIVTAARAANAHEFISSMPQGYNTTVGERGTQLSGGQKQRIAIARAILKDPRILLLDEATSAL
DAESERIVQDALNGAMVGRTTVIVAHRLSTIQGADMIAVLKDGTIVEKGSHEMLMGIAGGAYASLVELRTM

>XP_008670105.1_ABCB21_Zm
MATGEDEKAAGRVALHRLFVFADRTDAALMAVGAVAAVANGMVQPIMAFIFGDVIDAFGSGATDGVVRRV
VQVIMNFVYLAVGSGVASTLQVSCWTIAGERQAARIRAMYLKAILRQDIAFFDMEMSAGQAVERMAGDTFLI
QDAIGEKVGKAIQLLSTFIGGFIIAFTRGWLLALVMLSSVPPIAIAGAIVSKLMTGLSTRMQANYSDAGNVVEQT
LGAIRTVVSFNGENQAITKYNMFIRKAYRSSLQEGAVNALGFGSVIAILFSSYGLAVWYGSKLIVERGYNGGM
VISVIMSVMIGAMSLGQATRSVTAFAEGQGAAHRMFKIIERKPDIDIDDNTGIILEDVKGDVQLKDVYFSYPTRP
EHLIFDGFSLQVQSGTTMALVGDSGSGKSTVISLVERFYDPQAGEVLIDGVDIRGMKLGWMRGKIGLVNQEPV
LFSTTIRENIAYGTEKPTLEDINRAIQLANAAIFIDKLPNGLDTMVGEHGTQLSGGQKQRIAIARAIIKNPKILLLD
EATSALDMESERVVQEALVRIMVERTTIVVAHRLSTVRNADVISVLQHGKMVEQGSHVDLMKLPGGAYTQLI
RLHETQQEAEDVHPDMEISRRSTSKGSSSFGQSIPAPLGSPDPTEVSGAPDTEEGATDRVKVGRSQKKASIARLF
KLNKPETLVLALGSIAAVAHGTIFPIYGTLISTAIKVFYEPPQELLKGSRFWAGMFALLGACAFVLIPVQYFLFGL
AGGKLVERVRSMTFRSIMRQEISWFDKPEHSSGSICARLSTDALNVKRLVGDNLALNVQTASTIMSGFTIAMVA
NWKLALVITVVIPFVAFQAYAQMLFLKGLNRNAKLRYEEASQVATDAVGGIRTVASFCAESKVMDAYENKCE
SPRRQGMKEGVVGGLGFGFSFVVFYLTYALCFYVGARFVHQGTATFPEVFRVFFVLALATSGVSQTSAAGADS
AKANDSAVSIFAILDRESKIDYSSEEGVTIASVRGDVDFQKVCFKYPLRPNVQIFKDFSLRIPSGKTVALVGESGS
GKSTVIALLERFYDPESGKIFIDGVELGTLKVSWLRQQAGLVAQEPVLFNDTIRANIAYGKQGGVVSEEEVVAA
AEAANAHGFIAALPDGYDTVVGERGSRLSGGQKQRVAIARAVVKDPRLLLLDEATSALDVESERVVQEALDR
VMVGRTTVVVAHRLSTIRGADVIAVLKDGAVLEKGRHEELMQVKDGAYASLLELSSSSA
>XP_021303309.1_ABCB11_Sb
MATGAAGAVANGMAQLLMTLIFGEVVNVFGSSSRNDILHRVSGVCLKFIYLAIGSWFACFLQVASWIITGERQ
AARIRGLYLEALLRQDIAFFDKEMNTGQLVESMSGDTILIQDAIGEKVGKFIQLTATFVGGLVIAFSKGWLLAA
VMMSSVPPVVVAGAAISWTVSKLSSQGQAKYHEAGIVVEQTIGAIKTVASFNGENRAIALYNKYIRNAYVSAV
QEGTFTGLGFGFVMLILFCSHGLTAWYGAKLIIDKGYEGGQVVSVWMAFMTGAMSLGEATPCITAFASGRAA
GYRMMQIIQRKPQIDRNETDGIVLANMKGDIELRDVYFSYPSRRDQLIFDGFSLHVLSGKTMAIVGQSGSGKST
VINLVERFYDPQAGEVSIDGVNIKSLRLGWLRENIGLVSQEPLLFATSIQENIVYGKEDATDEEIKAATKLANAA
NFIDKLPNGLDTMVGEHGAQLSGGQKQRIAITRAILKNPKILLLDEATSALDMESERVVQEALNRIMQGKTTIIV
AHRLSTIKDADTISVIHRGKVVELGTHTELLQDPNGAYSQLIQLQDITGEPDASDVDYQRSTSAVRNVESLSKC
MQAPSLKGSITGGASFGSTSVHLITSANMIVPESTDTEPLPKVWDEGEECRKVDLSRLISLNKPEMPVLLLGTVA
AVISGVMFPILGLLMSSSINSFYEPPHQLQKDSRFWTLMYVASGVASFIILPVENFLFGVAGGKLVERIRSLSFQS
IVCQEISWFDRSSNARFTYFFMSLISMSSGNVGTRLSVDASNIRRLVGDSLALMVQSTVTVIAGFVIAMVANWR
LALVAMVVLPCGGLQGFLQIKFLEGFSTNAKAMYEEATQVATDAVSGIRTIASFCAERKVMKTYYGKRKAPM
QQGTRQGIVSGLGFGVSFFLMYSTYALCFYIGAKFVLDGKATFTEVFRVFFALLLATAGVSQRSALGSDYAKT
KASASTIFALIDRKSKIDPSSDDGMVLVDVAGELELHHICFSYPSRPDIQIFRDLNLRIPSGKTVALVGESGCGKS
TIIALLERFYDPDCGTITLDSVDIKNLKVGWLRRQMGLVSQEPVLFNDTIRANIAYGKEDGEATEEEIAAAAKA
ANAHAFISALPQGYGTVAGERGAQLSGGQKQRVAIARAVLRDPRILLLDEATSALDAESERAVQEALDRAAV

GRTTVVVAHRLSTIRDADVIAVLRNGDVVAQGTHQELMTARDGVYASLVELRMRSERAGVSSSA
>XP_006349874.2_P_ABCB11-l_St
MMEEENATQRGIDNMENKVIVSSDGSSCARVQDKTEKQKVAAAAAEVPYYKLLSFADPTDHALMIIGSIAAV
GTGISFSLMAVLFGEIVDSFGLTVDNDKVVGEVSKVSLKFIYLALGSGLATFVQVTCWTVTGERQAARIRCSYL
KTVLRQDIGFFDQETNTGVIIERLSSDTLTIQDAIGEKVGKFIQILATFLGGLVIAFIKGWRLALVLSSSIPPLVLSS
AVLTILLVKLASRSQTAYSEAATVVEQTISSIRTVASYTGERRAISEYYNSLNKAYYSGVQEGLVSGLSMGVFFF
VFYSSYALAVWYGAKMILDHNYTGGDVMNVMMATLTGSFTLGQASPCLHAFAAGKAAAFKMFQTINRKPVI
DPYDMKGQKLLDISGDIELKNVHFCYPARPQESIFDGFSVSIPKGTTTALVGRSGSGKSTVISLIVRFYDPQAGE
VLIDGINIKEFQLRWIRGKIGLVSQEPVLFGSTIKDNVAYGKDDATLEEIKDAVRLANASEFIDKLPQGLDTRVG
DHGSQLSGGQKQRIAIARAILKDPKILLLDEATSALDAESERIVQETLDSVMINRTTVIVAHRLSTVKNADAIAV
LQEGKIIEKGSHLELMRNKEGAYVQLIQLQELSKYSGEKDSNELDSEEIIINPNNQSNHQIFVTRSTSRHSSEVEN
SSHHPASISVSAAQKAVGECHYPNSTVILRKDKDSTFYRLALMTRPELPELLLGCVAAVVNALILPIFGVLLSYV
IKTFYEPAHELRKHSGFWSLLFLGLGLTSLLAKPLRTFFFAVAGCKLIKRIRLMCFEQLVYMEISWFDRKENSIG
AIGSRLSTDAASVRGMLGESLALLVENTSTAVAGLVIGLEASWQMTLIMIVMVPLIGLHGYLRLKYTNGGGAD
VKKLYDDASRVAHEAVGSIRTVASFSAEEKVVQLYKRKCEGPVRAGIKEGLLSAAGFGFSMFCFYSVYAASFY
AGARLIESGKVTFAEVFRVFYGLSLTATAISQSGGLAPDSSKAKTGASSIFALLDRQSKIDSNNNSGMILDNAKG
NIEFQHVSFNYPSRPEAQVLKDLCLAIRSGETVALVGESGSGKSTVISLLQRFYDPDSGLITLDGLEIQKLNVKW
LREQMGLVSQEPILFNDTIRANIAYGKESDATEAEILAAAELANAHSFISGLQQGYETVVGERGIQLSGGQKQR
VAIARAIVKCPKILLLDEATSALDSESEKVVQDALDRVRSGRTTVVVAHRLSTIKGADVIAVIKDGVIVEKGNH
ETLVNRQDGIYASLVSKSASTMK

>XP_003549468.1_ABCB13_Gm
MAEVELAPDSRLEQNVSSKIDQQTKTESVSFFGLFATADATDCVLMFLGCFGSCVHGAALPVFFILFGRMIDSL
GHLSNDPHKLSSRVSEHALYLVYLGGVVLVSAWMGVAFWMQTGERQTARLRLKYLQAVLKKDINFFDNEAR
DANIIFHISSDAILVQDAIGDKTGHAIRYLSQFIVGFAIGFTSVWQLTLLTLAVVPLIAVAGGAYTIIMSTLSEKGE
AAYAEAGKVAQEVISQVRTVYSFVGEEKAVGSYSKSLDNALKLGKKGGLAKGIGVGFTYGLLFCAWALLLW
YASILVRNHKTNGGKAFTTIINVIFSGFALGQAAPNLGSIAKGRAAAGNIMNMIASTSRNSKKFDDGNVVPQVA
GEIEFCEVCFAYPSRSNMIFEKLSFSVSAGKTIAIVGPSGSGKSTIVSLIQRFYDPTSGKILLDGYDLKNLQLKWL
REQMGLVSQEPALFATTIAGNILFGKEDADMDKVIQAAMAANAHSFIQGLPDGYQTQVGEGGTQLSGGQKQR
IAIARAVLRNPKVLLLDEATSALDAESELIVQQALEKIMSNRTTIVVAHRLSTIRDVDTIVVLKNGQVVESGTHL
ELMSNNGEYVNLVSLQASQNLTNSRSISRSESSRNSSFREPSDNLTLEEQLKLDAAAELQSRDQHLPSKTTSTPSI
LDLLKLNAPEWPYAILGSVGAILAGMEAPLFALGITHILTAFYSPQGSKIKQEVDRVAFIFLGVAVITIPIYLLLH
YFYTLMGERLTARVRLLMFSAILNNEVAWFDKDENNTGSLTAMLAADATLVRSALADRLSTIVQNVALTVTA
FVIGFTLSWKLTAVVVACLPLLIGASITEQLFLKGFGGDYGHAYSRATSLAREAIANIRTVAAFGAEDRVSTQFA
SELNKPNKQALLRGHISGFGYGITQLLAFCSYALGLWYASVLIKKNESNFGDIMKSFMVLIITSLAIAETLALTP
DIVKGSQALGSVFGIIQRRTAITPNDTNSKIVTDVKGEIEFRNVSFKYPMRPDITIFQNLNLRVPAGKSLAVVGQS
GSGKSTVISLVMRFYDPDSGLVLVDECDIKNLNLRSLRLRIGLVQQEPALFSTTVYENIKYGKEEASEIEVMKA
AKAANAHEFISRMPEGYKTEVGERGVQLSGGQKQRVAIARAILKDPSILLLDEATSALDTVSERLVQEALDKL
MEGRTTILVAHRLSTVRDANSIAVLQNGRVAEMGSHERLMAKSGSIYKQLVSLQHETRDQEDH
>XP_003544389.1_ABCB13_Gm
MAEVELAPDSLIEQNVTSKTVQQSKTDSVSFFGLFAAADATDCVLMFLGSVGSCVHGAALPVFFILFGRMIDSL
GHLSNNPHKLSSRISEHALYLVYLGGVVLVSAWMGVAFWMQTGERQTARLRLKYLQAVLKKDINFFDNEAR
DANIIFHISSDAILVQDAIGDKTGHAIRYLSQFIVGFAIGFTSVWQLTLLTLAVVPLIAVAGGAYTIIMSTLSEKGE
AAYAEAGKVAEEVISQVRTVYSFVGEEKAAGSYSKSLDNALKLGKKGGFAKGVGVGFTYGLLFCAWALLLW
YASILVRHHKTNGGKAFTTIINVIFSGFALGQAAPNLGSIAKGRVAAANIMNMIASASRNSKKLDDGNIVPQVA
GEIEFCEVCFAYPSRSNMIFEKLSFSVSAGKTIAVVGPSGSGKSTIVSLIQRFYDPTSGKILLDGYDLKNLQLKWL
REQMGLVSQEPALFATTIAGNILFGKEDADMDKVIQAAMAANAHSFIQGLPDGYQTQVGEGGTQLSGGQKQR
IAIARAVLRNPKVLLLDEATSALDAESELIVQQALEKIMSNRTTIVVAHRLSTIRDVDTIVVLKNGQVVESGTHL
ELMSNNGEYVNLVSLQASQSLTNSRSISCSESSRNSSFREPSDNLTLEEPLKLDTAAELQSRDQHLPSKTTSTPSI
LDLLKLNAPEWPYAILGSVGAILAGMEAPLFALGITHILTAFYSPQGSKIKQEVDWVAFIFLGVAVITIPIYLLLH
YFYTLMGERLTARVRLLMFSAILNNEVAWFDMDEHNTGSLTAMLAADATLVRSALADRLSTIVQNVALTVTA
FVIGFTLSWKLTAVVVACLPLLIGASITEQLFLKGFGGDYGHAYSRATSLAREAIANIRTVAAFGAEDRISIQFAS
ELNKPNKQALLRGHISGFGYGITQLLAFCSYALGLWYASVLIKKNESNFGDIMKSFMVLIITSLAIAETLALTPDI
VKGSQALGSVFGIIQRRTAITPNDPNSKMITDVKGEIEFRNVSFKYPMRPDITIFQNLNLIVPAGKSLAVVGQSGS

GKSTVISLVMRFYDPDLGSVLIDECDIKSLNLRSLRLRIGLVQQEPALFSTTVYENIKYGKEEASEIEVMKAAKA
ANAHEFISRMPEGYKTEVGERGAQLSGGQKQRVAIARAILKDPSILLLDEATSALDTVSERLVQEALDKLMEG
RTTILVAHRLSTVRDADSIAVLQNGRVAEMGSHERLMAKPASIYKQLVSLQHETRDQQDH
>XP_021614081.1_ABCB13-l_Me
MEGMELASDQISDENSHSKVNRERPSISFFGLFSAADRIDYLLMFLGSVGSCIHGAALPVFFIFFGRMIDSLGNLA
VDPHKMSSQVSRHALYLVYLGLVVFASAWIGVAFWMQTGERQTARLRLHYLQSVLKKDMNFFDTEAGDSNII
FHISSDAILVQDAIGDKTGHAMRYLSQFIVGFAIGFASVWQLTLLTLAVVPLIAIAGGAYTIIMSTLSEKGEAAY
AEAGKVADEVISQIRTVYSFVGEDRAIEAYSRSLKNALKLGKKSGIAKGIGVGFTYGLLFCAWAMLLWYASVL
VRHRITSGAKAFTMIINVIFSGFALGQAAPNLAAIAKGRAAASNIISMIETGSNTSKRLKEGSELPKVDGKIEFCN
VCFAYPSRPSKVLENLSFTVSAGKTFAVVGPSGSGKSTIISMVQRFYDPDSGKVLLDGHDLKILRLKWLREQMG
LVSQEPALFASTIADNILLGKEDASMDEIIQAAKAANAHSFIQQLPDGYYTQVGEAGTQLSGGQKQRIAIARAV
VRNPTILLLDEATSALDTESEFIVQQALNKIMSNRTTIIVAHRLSTIRDVDTIIVLKNGQVAESGSHLDLISKGGD
YATLVSLQVAEPLTHSNSIGCSEASRNFSFREVPHSQNNQQDFKSISIREPQSNDDSIPSQNHSPTLSILELIKLNAP
EWPCALLGSVGAILAGMEAPLFALGITHVLTAFYSHDASEMRHEIRRVALIFVGLGVVTIPIYLLQHYFYTLMG
ERLTTRVRLSMFSAMLSNEIGWFDLEENNTGSLTSALSADATLVRSALSDRLSTIVQNVALTVTACVIAFTLSW
RIAAVVVASLPLLVGASIAEQLFLKGFGGDYHAYSRATTVAREALTNIRTVAAFGAEERISIRFASELSKPNKQA
LLRGHISGFGYGLTQLFAFGSYALGLWYASVLITHKDSNFGHIMKSFMVLIITALAIAETLALTPDIVKGSQALG
SVFSVLHRRTAIDPNNLASKVVTDIKGDIEFRNVSFKYPARPDITIFELLNLKVPAGKSLAVVGPSGSGKSTIIALI
LRFYDPISGAILIDGCDIKTMNLKSLRLKIGLVQQEPALFSTTIYENIKYGNENASEVEIMKASKAANAHGFISRM
PEGYQTHVGDRGLQLSGGQKQRVAIARAILKDPSILLLDEATSALDTASEKLVQEALDKLMERRTTILVAHRLS
TIRDADSIAVLQHGRVAEFGSHKQLMGKPGSIYKQLVSLQQEERIQS

>XP_003589516.2_ABCB13_Mt
MDEVELACESSLECKKTKEEGTSKKQSKVESVSFFGLFGAADRTDYVLMFLGSVGSFVHGAALPVSFVLFGRM
IDSLGHLSSNPHKFSSQISQHALYLVYLGVVVLVSAWMGVAFWTQTGERQTAWIRLRYLQSVLKKDIRFFDNE
AKDANIISHISSDAILVQDAIGDKTGHAIRYLSQFIVGFGIGLTSVWQLTLLTLAVVPFIAIAGRTYLTIISTLSEKG
KAAYAEAEKVAEEVISRVRTVYSFAGEEKAVGSYSKSLDKALKLGKKSGFAKGVGVGFTYGLLFCAWALLL
WYASILVIHHKTNGGKAFTTIINAIFSGFALGQAALNIGSIAKGRTAAANIMNMIASVSESSKMLDDGFVLSQVA
GKIDFYEVYFACPSRSKMIFENLSFSVSAGKTVAVVGSSSSGKSTIISLIQRFYDPTSGKVLLDGYDLKNFKLRW
LRKQMGLVSQEPALFATTIAGNILFGKEDASVNEIIHAAKVVNAHSFITGLPQDYNTQVGEGGTQLLGGQKQIIS
LARAVLRNPKILLLDEATSALDAESELIVQQALKKIMLNRTTIIVAHRLSTVRNVDTIIVLKNGQVAESGTHLEL
MSRNGEYVSLQAPQNFTSSSSLFRLGSSRNYSFREIPNNLNNEEVQSSDQGLTSNTASVPSILGLLKLNAPEWPY
AILGSVGAVLAGMEAPLFAIGITHILATFYSAQSPKIKHEVDHVAVIFVVLAVVTIPIYLLKHYFYSLMGDRLTA
RVRLLMFSAILTNEVAWFDINENNTSSLTATQAADATLVRSALADRLSTLVQNIALTVTAFVIAFTMSWKLTLV
VAACLPFLIGAYITEQLFLKGFGGDYSHAYSKANSLARDAIVNIRIVTAFSAEDRMSTQFAYELNKPYKQALLR
GQISGFGYGLTQLFAFCSYALVLWYASILIKKKESTFGDLMKSVVVLIITAIAIVETIALTPDIVKGTQALRSVFSI
LHRKTSINRNDPNSKMISEVKGDVKFQNVCFKYPMRPDITIFQNLNLRVSAGKSLAVVGQSGSGKSTVIALVM
RFYDPTYGSVLIDECDIKSLNLRSLRQKIGLVQQEPALFSTTVYENIKYGKEEATEIEVMKAAKAANAHEFISTM
AEGYKTKVGEKGVQLSRGQKQRVAIARAILKDPSILLLDEATNALDTISERLVLEAIDKLMEGRTMILVAHRLS
TVRNADSIAVLQHGKVAEMGRHEKLMAKPGSIYKQLVSLQQEKHKQEEN

>XP_021602224.1_ABCB15-l_Me
MGTEKKSSMVKKKSNHIGSIRSIFIHADLVDWLLMVLGFIGSVGDGFSTPLVLFVTSKLMNNIGGASSSQSDFSH
NINKNALALCYVACGQWVVCFLEGYCWTRTGERQATRMRARYLKAVLRQEVGYFDLHVTSTEEVITSVSND
SLVIQDVLSEKVPNFLMNASMFFGCYLVGFLMLWRLAIVGFPFIIILVIPGLMYGRTLMGLARKIREEYKKAGTI
AEQAISSIRTVYAFVGESKTIEAYSTALDFSVKLGLRQGLAKGLAIGSNGVVFAIWSFMSYYGSRMVMYHNAR
GGTVFAVGAAIAVGGLALGAGLSNVKYFSEACTAGERIMEVIRRVPKIDLENMEGEVLENVRGEVEFKHAEFA
YPSRPESIIFKDFSLKIPAGRTVALVGSSGSGKSTAIALLQRFYDPLGGEILLDGVAIDKLQLKWLRSQMGLVSQE
PALFATSIKENILFGKEDATLEEVIEAAKASNAHNFICQLPQGYDTQVGERGVQMSGGQKQRIAIARAIIKAPRIL
LLDEATSALDSESERIVQEALDKAAVGRTTIVIAHRLSTIRNADIIAVVQNGQVMETGSHDELMEIEDGLYTTLV
RLQEREKDITNEDDQCYIPSSSLISKIDMNNTSSRRLSMVSRSSSANSMAPSRASVTGEDIQLEEQNFPVPSFRRL
LALNLPEWKQAGFGCLGAILFGGVQPLYAFAMGSMISIYFYTDHDEIKKQIRIYALCFLGLAIFSLIINIVQHYNF
AYMGEHLTKRIRERMLSKILTFEVGWFDQDENSSGAICSRLAKDANVVRSLVGDRMALVVQTVSAVTIACTM
GIVIAWRLAIVMIAVQPIIIVCFYVRRVLLKSMSQKAIKAQDESSKLAAEAVSNLRTITAFSSQDRILRMLEKSQE

GPQRESIRQSLFAGVGLGTSQSLMSCTWALDFWYGGRLISKGYISAKALFETFMVLVSTGRVIADAGSMTTDL
AKGSDAVGSVFAVLDRYTKIEPDDPDGFKPETIMGHVELRDVDFAYPARPDVIIFKSFSIKIEAGKSTALVGQSG
SGKSTIIGLIERFYDPIRGTVKIDGRDIKSYHLRSLRKYIALVSQEPTLFAGTIRENIVYGTSDKNDESEIIEAAKAA
NAHDFITGLKDGYDTWCGDRGVQLSGGQKQRIAIARAILKNPAVLLLDEATSALDGQSEKVVQDALERVMVG
RTSVVVAHRLSTIQNCDLIAVLDKGQVVEQGTHSSLLAKGPTGAYFSLVSLQRTPHYSSNASSHAFN
>XP_021598648.1_ABCB15-l_Me
MVKKKSNHFGSIRSIFMHADRVDWLLMVLGFIGSVGDGFSTPLVLFVTSRLMNNIGGSSSFQSDFSHNVIKNAL
TLCCVACGQWVVCFLEGYCWTRTGERQATRMRARYLKAVLRQDVGYFDLHVTSTAEVITSVSNDSFIIQDVL
SEKVPNFLMNASMFMGCYLVGFLVLWRLAIVGFPFIVMLVIPGLMYGRTLMGLARKIKEEYNKAGAIAEQAIS
SIRTVYAFVGESKTIAAYSEALDFSVKLGLKQGLAKGLAIGSNGIVFAIWSFMCYYGSRMVMYHNARGGTVFV
VGASIAMGGLALGAGLSNVKYFSEACTAGERIMEVIERVPKIDVENMEGETLENVRGEVDFKHVEFAYPSRPE
SIIFKDFSLKFPAGRTVALVGGSGSGKSTVIALLQRFYDPLGGEILLDGVAIDKLQLKWLRSQMGLVSQEPALFA
TSIKENILFGKEDATMEEVVEAAKASNAHNFICQLPQGYNTQVGERGVQMSGGQKQRIAIARAIIKAPRILLLDE
ATSALDSESERIVQQALDKAAVGRTTIIIAHRLSTIRNVDLITVVQNGQVMEMGSHDELMEIKDGHYTTLVRLQ
QTEKGKTNEDDQYHIPSSSLISKIDMNNTSSHRLSMVSRSSSANSMTPSRSSVAAENTLLEEQKFLVPSFQKLLA
LNLPEWKQASFGCLGAILFGGVQPLYAFAMGSMLSVYFYTDHNEIKKQIRVYSLCFLGLAVFSLIINIVQHYNF
ACMGEYLTKRIRERMLTKILTFEVGWFDQDENSSGAICSRLAKDANVVRSLVGDRMALVVQTVSAVTIACTM
GLVIAWRLAVVMIAVQPIIIVCFYTRRVLLKSMSQKAIKAQDESSKLAAEAASNLRTITAFSSQDRILRMLEKAQ
EGPLRESIRQSLFAGVGLGSSQSLMTCTWALDFWYGGKLISKGYISARDLFETFMVLVSTGRVIADAGSMTTDL
AKGSAAIGSVFAVLDRYTKIEPEYPEGFKPETIMGHVELRDVDFAYPARPDVIIFKGFSIKIEAGKSTALVGQSGS
GKSTIIGLIERFYDPIRGTVKIDGRDIKSYHLRSLRKYIALVSQEPTLFAGTIRENIVYGTSDKNDESEIIEAAKAAN
AHDFITGLKDGYDTWCGGRGVQLSGGQKQRIAIARAILKNPAVLLLDEATSALDSQSEKVVQDALERVMVGR
TSVVVAHRLSTIQNCDLIAVLDKGQVVEQGTHSSLLAKGPAGAYFSLVSLQRNSHNSSTTTSYAFN
>XP_002324019.2_ABCB15_Pt
MGTEENSKKSRDHVGSIRSIFMHADRVDWLLMVLGFIGSIGDGFSTPLVLFVTSKLMNNLGGASSSAEAFTHSI
NKNALALCYLACGQWVVSFLEGYCWTRTGERQATRMRARYLKAVLRQDVGYFDLHVTSTAEVITSVSNDSL
VIQDVLSEKVPNFLMNVAMFFGCYIIGFVLLWRLAIVGLPFVVILVIPGLVYGRTLMGIARKTREEYNKSGTIAE
QAISSIRTVFAFVSEAKTIAAYSAALEFSVKLGLRQGLAKGLAIGSNGVVFGIWSFMSYYGSRMVMYHGSAGG
TVFAVGAAIAVGGLALGAGLSNVKYFSEASSAGERIVEMINRVPKIDLENMEGETLENVTGEVEFRHVEFAYPS
RPESMIFKDFCLRIPAGKTVALVGGSGSGKSTVIALLQRFYDPLGGEILVDGIAVDKLQLKWLRSQMGLVSQEP
ALFATTIKENILFGKEDATINEVVEAAKASNAHNFISHLPQEYDTQVGERGVQMSGGQKQRIAIARAIIKAPRIL
LLDEATSALDSESERVVQEALDKAAVGRTTIIIAHRLSTIRNADVIAVVQDGQILESGSHGELIENENGLYTSLVL
LQQTEKEKTNEDASTDISSPSLVSNMDVNNASSRRLSIVSRSSSQNSVTPSRASLTAGENALVEEQQLPVPSFRR
LLALNLPEWKQASIGCLGAIIFGGVQPLYAFTMGSMISIYFLADHNEIKEKIRIYSLCFLGLAFLSLIVNVLQHYN
FAYMGEHLTKRIRERMLSKILTFEVGWFDQDKNSSGAICSRLATDANVVRSLVGDRMALIVQTISAVTIACTM
GLIIAWRLAVVMIAVQPIIIVCFYVRRVLLTSMSQKAIKAQDESTKLAADAVSNLRTITAFSSQDRILKMLGKAQ
EGPRKENIRQSWYAGIGLGTSQSLMSCTWALDFWYGGRLISQGYITAKALFETFMILVSTGRVIADAGSMTTDL
AKGSDSIRSVFAVLDRYTRIEPEDPEGYQPGEIKGHVELCDVDFAYPARPDVRIFKGFSISIEAGKSTALVGQSGS
GKSTIIGLIERFYDPLRGTVKIDGRDIRSYHLRSLRKYIALVSQEPTLFAGTVKENIIYGAANEVSESEVMEAAKA
ANAHDFIAGLKDGYDTWCGDKGVQLSGGQKQRIAIARAILKNPVVLLLDEATSALDSQSEKVVQDALERVMV
GRTSVVVAHRLSTIQNCDLIAVLDKGKVVEKGTHSSLFSKRPTGIYYSFVRLQAQRTTQNSATATTAAFS

>XP_006338500.1_P_ABCB15-l_St
MSCKSKTTIQEKRYGSFRSVFMHADSVDILLMILGFLGAICDGVSMPVMLIVTSKLMNNLGNNDSSSTDSFTHH
INENALALVYLACGQWVACFLEGFCWTRTAERQASRLRISYLKAVLRQDVGYFDLHVASTADVIASVSSDSLV
IQECISEKVPVFLMNVATFIGSYVVGFLMIWKLALVGFPFIIFLVIPGLMYGRALMGIARKIRDEYGKAGIIVEQA
ISSVRTVYSFVGENKTIAEYSNALQGTVDLGLKQGLAKGLAIGSNGIVFAIWSFMSYYGSRMVMYNGEHGGTV
FAVGAAIAIGGLALGSGLSNLKYFSEANAAGERVVQVIKRVPKIDSDNMEGQTLDNVTGEVEFKHVEFAYPSR
PESIILNDFSLKVPTGKTVALVGGSGSGKSTVVALLQRFYDPLGGEILLDGIAIDKLQLKWLRSQMGLVSQEPAL
FATTIKENILFGKEDASMEQVIEAAKASNAHNFICQLPQGYDTQVGERGVQMSGGQKQRIAIARAIIKSPRILLL
DEATSALDSESERVVQEALDKAAVGRTTIIIAHRLSTIRNADLIAVVQNGQVKEIGSHDELIEDVDGLYTSLVRL
QQTENPSDEISIAPTNRNTVFAPSNLNSGFTSDHEVQNTSSRRLSIVSRSSSANSAAQSRRFDQNATISNTPEQVFP
VPSFKRLLAMNLPEWKEATLGCIGAILFGGVQPVYAFAMGSMISVYFLPSHDEIKEKTKIYALCFLGLAFFSLFV
NVLQHYNFAAMGEKLTKRIRERMLSKMLTFEIGWYDKEENSTGAVCSRLAKDANVVRSLIGDRMALLIQTVS

AVTIACTMGLVIAWRLAWVMIAVQPLIIVCYYCKRVLLKNMSKKSIKAQEESSKLAAEAVSNLRTVTAFSSQS
RILQMLKKAQEGPLRESIRQSWFAGIGLGTSNSLMTCTWALDFWYGGKLMAEGLIGAQALFQTFMILVSTGRV
IADAGTMTNDLAKGADAVGSVFAVLDRYSLIEPEDSDGYKPKKITGNVELYDVDFAYPARPNVIIFKGFSIKIEA
GKSTALVGQSGSGKSTIIGLIERFYDPLSGVVKIDGRDVRSYHLRSLRKHIALVSQEPTLFAGTIRQNIGYGASEE
VDESEIIEAAKAANAHDFISALKDGYETWCGDRGLQLSGGQKQRIAIARAILKNPAVLLLDEATSALDSQSEKV
VQDALERVMVGRTSVVVAHRLSTIQNCDTIAVLDKGKIVEKGTHSSLLAKGPSGVYHSLVSLQRAPNSNNTFIS
>XP_014627633.1_ABCB15-l_Gm
MSGDHNSSVSMVGKKKKNGSLRSIFMHADGLDWFLMIFGLFGAIGDGIGTPLVLFITSKIMNNIGGFSSNIGSTF
IHSINENAVVLLYLAGGSFIACFLEGYCWTRTGERQAARMRVRYLKAVLRQEVAYFDLHVTSTSEVITSVSNDS
LVIQDCLSEKVPNFLMNASMFVGSYIVAFALLWRLAIVGFPFVALLVIPGFMYGRTLMGLASKIREEYNKAGTI
AEQAISSIRTVYSFVGESKTIDAFSEALQGSVELGLRQGLAKGLAIGSNGVVFAIWAFMSYYGSRLVMYHGAK
GGTVFAVGAAIALGGLALGAGLSNVKYFSEASTAGERIMEVIKRVPKIDSDSMAEEILENVSGEVEFNHVDFVY
PSRPDSVILNDFCLKIPAGKTVALVGGSGSGKSTVISLLQRFYDPIEGEIFLDGVAIHKLQLKWLRSQMGLVSQE
PALFATSIKENILFGREDATQEEVVEAAKASNAHNFISQLPQGYDTQVGERGVQMSGGQKQRIAIARAIIKKPRI
LLLDEATSALDSESERVVQEALDKAAVGRTTIIIAHRLSTIRNANVIAVVQSGKIMEMGSHHELIQNDNGLYTSL
VRLQQAKNEKEDTIFHPTPPSSISNKDNHNTSSRRLSVVMIRSSSTNSIPRIGGGDDNNIVEEVVEDNKPPLPSFR
RLLALNIPEWKQACLGCLNAVLFGAIQPVYAFAMGSVISVYFLPDHNEIKKKTMIYSLCFLGLAVFSLVVNILQ
HYNFAYIGEYLTKRIRERMFSKILTFEVGWFDQDENSTGAVCSRLAKEANVVRSLVGDRMXLVVQTISAVVIA
FTMGLIIAWRLAIVMIAVQPIIIACFYTRRVLLKSMSSKAIKAQDESSKIAVEAVSNLRTITAFSSQDRILKMLEK
AQEGPSRESIRQSWFAGIGLACSQSLTFCTWALDFWYGGKLVFQGFINAKALFETFMILVSTGRVIADAGSMTN
DLAKGADAVGSVFAILDRYTKIEPDDDIDGYKPEKLTGKIELHDVHFAYPARPNVMIFQGFSIKIDAGRSTALV
GQSGSGKSTIIGLIERFYDPMKGIVTIDGRDIKSYHLRSLRKHIALVSQEPTLFGGTIRENIAYGASNNNNKVDET
EIIEAARAANAHDFIASLKDGYDTSCRDRGVQLSGGQKQRIAIARAILKNPEVLLLDEATSALDSQSEKLVQDA
LERVMVGRTSVVVAHRLSTIQNCDLIAVLDKGKVVEKGTHSSLLAHGPGGAYYSLISLQRRPAN
>XP_004232253.1_ABCB15-l_Sl
MSTSKSKTMIQEKRYGSFQSVFMHADSVDILLMVLGFLGAICDGVSMPVMLIVTSKLMNNLGGNDSSDTFTHH
INENALALVYLACGQWVACFLEGFCWTRTAERQASRLRIRYLKAVLRQDVGYFDLHVASTADVIASVSSDSL
VIQECISEKVPVFLMNVATFTGSYVVGFLMIWKLALVGFPFIIFLVIPGLMYGRALMGIARKIRDEYGKAGIIVE
QAISSVRTVYSFVGENKTLAEYSNALQGTVDLGLKQGLAKGLAIGSNGIVFAIWSFMSYYGSRMVMYNGEHG
GTVFAVGAAIAIGGLSLGSGLSNLKYFSEASAAGERVVQVIKRVPKIDSDNLEGQTLDNVMGEVEFKHIEFAYP
SRPESIILNDFSLKVPTGKTVALVGGSGSGKSTVVALLQRFYDPLGGEILLDGIAIDKLQLKWLRSQMGLVSQEP
ALFATTIKENILFGKEDASMEQVIEAAKASNAHNFICQLPQSYDTQVGERGVQMSGGQKQRIAIARAIIKSPRIL
LLDEATSALDSESERVVQEALDKAAVGRTTIIIAHRLSTIRNADLIAVVQSGQVKEIGSHDELIEDEDGLYTSLVR
LQQTENPSDEISIAPTNRNTVFAPSNLNSGFTSDHEVQNTSSRRLSIVSRSSSANSAAQSCRFDQNATISNTPEQV
FPVPSFKRLLAMNLPEWKEATLGCIGAILFGGVQPVYAFAMGSMISVYFLPSHDEIKEKTKIYALCFLGLAFFSL
FVNVLQHYNFAAMGEKLTKRIRERMLSKMLTFEIGWYDKEENSTGAVCSRLAKDANVVRSLVGDRMALLIQT
VSAVTIACTMGLVIAWRLAWVMIAVQPLIIVCYYFKRVLLKNMSKKSIKAQEESSKLAAEAVSNLRTVTAFSS
QSRILQMLKKAQEGPLRESIRQSWFAGIGLGTSNSLMTCTWALDFWYGGKLMAEGLIGAQALFQTFMILVSTG
RVIADAGTMTNDLAKSADAVGSVFAVLDRYSLIEPEDSDGYKPKKITGNVELCDVDFAYPARPNVIIFKGFSIKI
EAGKSTALVGQSGSGKSTIIGLIERFYDPLRGEVKIDGRDVRSYHLRSLRKHIALVSQEPTLFAGTIRQNIAYGAS
EEVDESEIIEAAKAANAHDFISALKDGYETWCGDRGLQLSGGQKQRIAIARAILKNPAVLLLDEATSALDSQSE
KVVQDALERVMVGRTSVVVAHRLSTIQNCDTIAVLDKGKIVEKGTHSSLLAKGPSGVYHSLVSLQRAPNSNNT
FIS

>XP_024641479.1_ABCB15_Mt
MGGGDQKNVYIVKKKKKNGSFKSIFMHADVLDWFFMVFGLIGSIGDGISVPLLLFIAGRLMNSIGSASGASSNN
FVHDINKNAVLFLYLACASFVACFLEGYCWTRTGERQAARMRVRYLKAILRQDVAYFDLHITSTSEVITSVSN
DSLVIQDVISEKVPNFLMNASMFLGSYIAAFALLWRLAIVGFPFLVLLVIPGFMYGRISMGLARKIREEYNKAGT
IAQQAISSIRTVYSFAGESKTIAAFSNALEGSVKLGLKQGLAKGIGIGSNGLVFAVWSLMSYYGSRMVMYHGA
KGGTVYSVGISITLGGLAFGTSLSNVKYFSEASAAGERIMEVIKRVPKIDSENMEGEIIEKVLGEVEFNHVEFVY
PSRPESVILNDFCLKVPSGKTVALVGGSGSGKSTVVSLLQRFYDPIGGEILLDGVAIHKLQLKWLRSQMGLVSQ
EPALFATSIKENILFGREDATYEEIVDAAKASNAHNFISLLPQGYDTQVGERGVQMSGGQKQRIAIARAIIKMPK
ILLLDEATSALDSESERVVQEALDKAAVGRTTIIIAHRLSTIQNADIIAVVQNGLVMEMGSHDSLMQNDNSLYT
SLVRLQQTRNDQSDDTPSIMNRDHMEITSSRRLVSHSSSFNSMTHGGDDIVNYNNDVEDTNVEVPSFRRLLAM

NGPEWKQACLGCFNAVLFGAIQPVYSFAMGSVISVYFIEDHDEIKKQIRIYGFCFLGLAVISMVINMLQHYSFA
YMGEYLTKRVREKMFSKILTFEVGWFDEDQNSTGSVCSRLAKDANVVRSLVGDRLALVVQTISAVVIAFTMG
LIIAWKLAIVMIAVQPLIIYCFYTRRVLLKNMSSKAIKAQDQCSKIAAEAVSNLRTINAFSSQDRILKMLEKAQQ
GPSHESVRQSWFAGIGLACSQCLNYSTWALDFWYGGKLVSQGYISAKALFKTFMILVSTGRVIADAGSMTSDL
AKGSDAIGSVFAILDRYTKIKPNDLRGYKAEKLIGIIELFDVHFAYPARPNVMIFQGFSIKIDAGKSTALVGESGS
GKSTIIGLIERFYDPLKGIVTIDGRDIKTYNLRSLREHIALVSQEPTLFSGTIRENIAYGAYDDKVDESEIIEASKAA
SAHDFISSLKDGYDTLCGDRGVQLSGGQKQRIAIARAILKNPEVLLLDEATSALDSQSEKLVQDALERVMVGR
TSVVVAHRLSTIQNCDLIAVLDKGIVVEKGTHSNLLSKGPSGAYYSLVSLQRRPNNLISYSSHEIN
>XP_003618412.2_ABCB15_Mt
MGGGDQKNVSINVKKKKNGSFRSIFMHADVLDCFFMAFGLIGAIGDGLMTPLVLFITSRIMNSIGTISGSSSTNF
VHNINENALVLLYLACASFAACFLEGYCWTRTGERQAARMRARYLKAVLRQEVAYFDLHVTSTSEVITSVSN
DSLVIQDVLSEKVPNFLMNASMFIGSYIVAFALLWRLAIVGFPFVVLLVIPGFMYGRTLMGLARKMREEYNQA
GTIAEQAISSIRTVYSFAGESKTIAAFSNALEGSVKLGLKQGLAKGLAIGSNGVVFAIWSFMSFYGSRMVMYHG
AKGGTVFAVGASLALGGLALGAGLSNVKYFSEASVAGERIMEMIKRVPKIDSENIEGEILEKVLGEVEFNHVEF
VYPSRPESVVLNDFCLKVPSGKTVALVGGSGSGKSTVVSLLQRFYDPIGGEILLDGVAIHKLQLKWLRSQMGL
VSQEPALFATSIMENILFGREDATYEEIVDAAKASNAHNFISMLPQGYDTQVGERGVQMSGGQKQRIAIARAII
KMPKILLLDEATSALDSESERVVQEALDKAAVGRTTIIIAHRLSTIQNADIIAVVQNGKIMETGSHESLMQNENS
LYTSLVRLQQTRNDQTDDTPSIMNRGHMQNTSSRRLVSRSSSSFNSMTHGGDDILNYNNVVEDIVNNVVVVD
DRNNHNSINNTKKEKVKVPSFRRLLAMNVPEWKQACLGCINAVLFGAIQPVYSFALGSVVSVYFLEDHDEIKK
QIRIYVFCFLGLAVISLVVNVLQHYSFAYMGEYLTKRVRERMFSKILTFEVGWFDEDRNSTGSVCSRLAKDAN
VVRSLVGDRLALVVQTISAVVIAFTMGLIIAWRLAIVMIAVQPVIICCFYTRRVLLKNMSSKAIKAQDECSKIAA
EAVSNLRTINAFSSQDRILKMLEKAQQGPSHESIRQSWFAGIGLACSQSLNFCTWALDFWYGGKLVSQGYISAK
ALFETFMILVSTGRVIADAGSMTNDLAKGSDAVGSVFAVLDRYTKIEPDDLESYQAEKLIGKIELRDVYFSYPA
RPNVMIFQGFSIKIDAGKSTALVGESGSGKSTIIGLIERFYDPLKGIVTIDGRDIKTYNLRSLRKHIALVSQEPTLFS
GTIRENIAYGAYDDTVDESEIIEASKASNAHDFISSLKDGYDTLCGDRGVQLSGGQKQRIAIARAILKNPEVLLL
DEATSALDSQSEKLVQDALERVMVGRTSVVVAHRLSTIQNCDLIAVLDKGSVVEKGTHSSLLSKGPSGAYYSL
VSLQRRPTNITIDSSHEIN
>XP_003618408.1_ABCB15_Mt
MGGSDQKNVSINVKKKKKNGSFKSIFMHADVLDCFLMAFGLFGAIGDGIMTPLLLFISSKLMNSIGTISGTSSNN
FVHNIYENAIVLLYLACASFVACFLEGYCWTRTGERQAARMRVRYLKAVLRQEVSYFDLHITSTSEVITSVSND
SLVIQDVLSEKVPNLLMNASMFIGSYIVAFTLLWRLAIVGFPFIVLLVIPGFMYRRTSMGLARKISEEYNRAGTIA
EQAISSIRTVYSFTGENKTIAAFSNALEGSVKLGLKQGLAKGFAIGSNGVVFAIASFMTYYGSRMVMYHGAKG
GTVYNVGASLALGGLTLGAVLSNVKYFSEASVAGERIMDVINRVPKIDSENMEGEILEKVLGEVEFNHVEFVY
PSRPESVILNDFCLKVPSGKTVALVGESGSGKSTVVSLLQRFYDPICGEILLDGVAIHKLQLQWLRSQMGLVSQ
EPALFATSIKENILFGREDATYEDVVDAAKVSNAHNFISLLPQGYDTQVGERGVQMSGGQKQRIAIARAIIKMP
KILLLDEATSALDSESERIVQDALDKVAVGRTTIIIAHRLSTIQNADIIAVFQNGKIMETGTHESLAQDENSLYTS
LVRLQQTRNDQNEDPASIMNRGHMQNTSSRRLVSRSSSFNSMTHGGDDINNFVDDIVNNVVIADDHNNNDDK
NNKKKEKVKVSSFQRLLAMNVPEWKQACLGCINAVLFGAIRPVYSFAMGSVISVYFLEDHDEIKRQIRIYAFCF
LGLAVISMVVNVLQHYSFAYMGEYLTKRVRERMFSKILTFEVGWFDEDQNSTGVVCSRLAKEANMVRSLVSD
RLALVVQTISAVVISFTMGLIIAWRLAIVMIAVQPLIICCFYTRRVLLKNMSSKAIKAQDECSKIASEAVTNLRTI
NSFSSQDRILKILGKAQQGPSHESIRQSWFAGIGLACSQSLFLCTWALDFWYGGKLVSQGYISAKALFETFMILI
STGRVIADAGSMTNDLAKGSNAVGSVFAILDRYTTIEPDDFEGYKAKNLIGKIELLDVDFAYPGRPNVMIFQGF
SIKIDAGKSTALVGESGSGKSTIIGLIERFYDPIKGIVTIDGEDIKSYNLRSLRKHIALVSQEPTLFGGTIRENIAYG
AYDDKVDESEIIQASKAANAHDFISSLQDGYDTLCGDRGVQLSGGQKQRIAIARAILKNPKVLLLDEATSALDS
QSEKLVQDALERVMVGRTSVVVAHRLSTIQNCDLIAVLDKGIVVEKGTHSSLLSLGPSGVYYSLVSLQRRPTNT
IVGSSHEIN
>XP_003618404.2_ABCB15_Mt
MNTFWLWWQMCGEKNVSINDKKKKNGSLKSIFMHADVLDWFFMVFGLIGAIGDGLMTPLLLLFLSRLMNSIG
SNSGPSKNYFVRSINENAVVLLYLACASCVACFLEGYCWTRTGERQAARMRVRYLKAVLRQEVAYFDLHVTS
TSEVITSVSNDILVIQDVLSEKVPNFVMNTSIFFGGYIVAFALLWRLAIVGFPFVVLLVIPGFMYGRTMMGLARK
MREEYNKAGTIAEQAISSIRTVYSFAGESKTIAAFSNALEGSVKLGLKQGLAKGLGIGSNGLLFAVWSLMAYY
GSRMVMYHGAKGGTVFAVGYSIALGGSALGAGLSNVKYFSEASVAGERIMEMINRVPKIDSKNMEGEILEKV
SGKVEFNHVEFVYPSRPESVVLNDFCLKVPSGKTVALVGGSGSGKSTVVSLLQRFYDPIGGEILLDGVAIHKLQ
LKWLRSQMGLVSQEPALFATSIKENILFGREDATYEEIVDAAKASNAHNFISLLPQGYDTQVGERGVQMSGGQ

KQRISIARAIIKMPKILLLDEATSALDSESERVVQEALDKATVGRTTIIIAHRLSTIQNADIIAVVQNGMIAETGSH
ESLMQNDNSLYASLVRLQQTKKDQTDDTPSIMNRDHMQNMSGCRLVSPSNSFNSTTRGSDDVFNYNNVVED
VVTKFVVDDDNSKNKKVEVPSFQRLLAMNGPEWKQTCLGCINAILVGAIQPVFSFGLGSVISVYFLENHDEIKK
QIRIYALCFLGLAVISMVVNVLQHYSFAYMGEYLTKRIREKMFSKILTFEVGWFDEDQNSTGSVCSRLAKEAN
VVRSLVGDRLSLVIQTISAVVIAFTMGLLIAWRLAIVMIAVQPIIIYCFYTRFVLLKNMSNKAVKAQDECSKIAA
EAVSNLRTINAFSSQEIILKMLEKSQQGPSHESIRQSWYAGIGLACAQSIKLCSYALSFWYGGKLVLQGYISAKA
LFKTFLILVSTGKVIADAGSMTNDLAKGSDAIASVFTILDRYTKIKPDEIEGHKAIKLIGKIEFCDVYFAYPSRPN
VMIFQGFSIKFDAGKSTALVGKSGSGKSTIIGLIERFYDPLEGIVTIDGRDIKTYNLRSLRKHIALVSQEPTLFGGTI
KENIAYGSYGDQVDESEIIEASKAANAHDFISSLKDGYDTLCGDRGVQLSGGQKQRIAIARAILKNPDVLLLDE
ATSALDSQSEKLVQDTLEKVMVGRTSVVVAHRLSTIQNCDLIAVLDKGSVVENGTHSSLLSKGPSGAYYSLISL
QKRPTNIIVDSPHEIK

>XP_008679898.1_P_MDR_Zm
MGKGGPRPAEAKKSAPALRSLASVFMHADVADVVLMVLGLVGAMGDGMSTPVMLFITSRIFNDLGSGPGLL
QEFSSKINENARNLVFLALGNWLMAFLEGYCWARTAERQASRMRERYLRAVLRQDVEYFDLKVGSTSEVITS
VSNDSLVVQDVLSEKVPNFVMNCSMFLGSYAVGFALLWHLTLVALPSVLLLIIPGFMYGRILIGLARRIREQYT
RPGAIAEQAVSSVRTVYSFVAERSTMAQFSAALQESARLGVKQGLAKGVAIGSNGITFAIWAFNVWYGSRLV
MYHGYQGGTVFAVSAAIVVGGLALGSGLSNVKYFSEASSAAERVQEVILRVPKIDSESSAGDELANVAGEVEF
KNVEFCYPSRPETPIFVSFNLRVPAGRTVALVGGSGSGKSTVIALLERFYDPSAGEVTLDGVDIRRLRLKWLRA
QMGLVSQEPALFATSIRENILFGKEDATGEEIVAAAKAANAHNFISQLPQGYDTQVGERGVQMSGGQKQRIAI
ARAILKSPKILLLDEATSALDTESERVVQEALDLASVGRTTIVIAHRLSTIRNADMIAVMQYGEVKELGSHDDLI
DNENGLYTSLVRLQQTRDSREANQVGGTVSTSAVGQSSSHSMSRRFSAASRSSSGRSMGDAENDNIAEKPKPPI
PSFRRLLMLNAPEWKQALMGSFSAIVFGGIQPAYAYAMGSMISIYFLADHDEIKDKTRTYALIFVALAVLSFLIN
IGQHYNFGAMGEYLTKRVREQMLAKILTFEIGWFDRDENSSGAICSQLAKDANVVRSLVGDRMALVIQTVSA
VLIACTMGLVIAWRLALVMIAVQPLIIVCFYARRVLLKSMSKKSIQAQSESSKLAAEAVSNLRTITAFSSQDRIL
RLFDQAQDGPRKESIRQSWFAGLGLGTSMSLMTCTWALDFWYGGKLMAERHITAKALFQTFMILVSTGRVIA
DAGSMTTDLAKGADAVASVFAVLDRETEIDPDNPEGYKPEKLKGEVDIKGVDFAYPSRPDVIIFKGFSLSIQPG
KSTALVGQSGSGKSTIIGLIERFYDPLRGVVKIDGKDIKTYNLRALRRHIGLVSQEPTLFAGTIRENIVYGTETAT
EAEIENAARSANAHDFISNLKDGYDTWCGERGVQLSGGQKQRIAIARAILKNPAILLLDEATSALDSQSEKVVQ
EALDRVMVGRTSIVVAHRLSTIQNCDQITVLEKGIVVEKGTHASLMAKGPSGTYFGLVSLQQGGNQH
>XP_003618377.1_ABCB15_Mt
MGGGDQKNVSINDKKKKNGSFKSIFMHADVLDWFFMAFGFFGAIGDGMMVPFVLFITSKIMNSVGSASGTSSS
NFVHDVNKNAVVVLYMACASFFVCFLEGYCWTRTGERQAARMRVRYLKAVLRQEVSYFDLHVTSTTDVITS
VSSDSLVIQDVLSDKVPNFLVNASRFLSSNIVAFALLWRLAIVGFPFMVLLVIPGYMYKRISMRLARKIREEYNQ
AGTIAEQAISSIRTVYSFVGESKTLAAFSNALEGSVKLGLKQGLAKGLAIGSNGVVYAIWSLIFYYGSIMVMYH
GAKGGTVFVVGVTLAIGGLAFGTCFSNVRYFAEASVAGERIMEVIKRVPTIDSENMEGEIIEKVLGEVEFNNVE
FVYPSRPESVILNDFCLKVPSGKTVALVGGSGSGKSTVVSLLQRFYDPIGGEILLDGVAIHKLQLKWLRSQMGL
VSQEPALFATSIKENILFGREDATYEEIVDAAKASNAHNFISMLPQGYDTQVGERGIQMSGGQKQRIAIARAIVK
MPKILLLDEATSALDSESERVVQEALDKAVVGRTTIIIAHRLSTIQNADIIAVVQNGKIMETGSHESLMQNDSSIY
TSLVHLQHTKNDQDGDTLSIMNKHHISCRFLSRSSSFNSMTHGGGDVVNYNNVVEDVVNDIDHNTNKKKKKV
KVPSFRRLLAMNAPEWKQVCLGCLSSVLFGAVQPISTFATGAVASVYFLNDRDEMKKQIRMYAFCFLGLALA
SIVFNMLEQYSFAYMGEYLTKRIRERMFSKILTFEVGWFDEDQNSTGVICSRLAKEANVVRSVVGDSLSLVVQ
TISAMVVTCTMGLIITWRLSIVMISVQPITIFCYYTRRVLLNNMSSKAIKAQDDSSKIAAEAVSNLRIITSFSSQNR
ILKMLEKAQQGPRHESIRQSWYAGIGLACSQSLIFCTRALNFWYGGKLVSQGYITKNQFFETIMIWISIGKVIAD
AASSMTNDLAKGSDAVRSVFAILDRYTKIKSDDLEGFRAEKLIGKIVFHDVHFSYPARPNVMVFQGFSIEIDAG
KSTALVGESGSGKSTIIGLIERFYDPLKGIVTVDGRDIKTYNLRSLRKHIALVSQEPTLFGGTIRENIVYGAYDDK
VDESEIIEASKAANAHDFISSLKDGYDTLCGDRGVQLSGGQKQRIAIARAILKNPEVLLLDEATSALDSQSEKLV
QDALEKVMVGRTSVVVAHRLSTIQNCDLIAVLDKGIVVEKGTHSSLLSKGPSGAYYSLVSLQRRPNNIIADSCH
EIN
>XP_002453447.2_P_MDR_Sb
MGKDGPTQAAAAMAKKAPAPVMRWSFASVFMHADATDVVLMVLGLVGTMGDGFSTPVMLFITSRIFNDLG
NGPDVLQEFSSKINENARNLVFLALGCLVMAFLEGYCWARTAERQASRMRERYLRAVLRQDVEYFDLKVGST
SEVITSVSNDSLVVQDVLSEKLPNFVMNCAMFLGSYAVGFALLWHLTLVALPSVLLLIIPGFMYGRILIGLARRI
REQYTRPGAIAEQAVSSVRTVYSFVAERTTMAHFSAALEESARLGIKQGLAKGVAIGSNGITFAIWAFNVWYG

SRLVMYHGYQGGTVFAVSAAIVVGGLALGSGLSNVKYFSEASSAAERVQEVILRVPKIDSESSAGDEVANVAG
DVEFKNVEFCYPSRPETPIFVSFNLRVPAGRTVALVGGSGSGKSTVIALLERFYDPAAGEVTLDGVDIRRLRLK
WLRAQMGLVSQEPALFATSIRENILFGKEDATEEEVVAAAKAANAHNFISQLPQGYDTQVGERGVQMSGGQK
QRIAIARAILKSPKILLLDEATSALDTESERVVQEALDLASVGRTTIVVAHRLSTIRNADMIAVMQYGEVKELGS
HDELIANENGLYTSLVRLQQTRDSREANQVGGTGSTSAAGQSSSHSMSRRFSAASRSSSGRSMGDAENDNITE
KPKLPVPSFRRLLMLNAPEWKQALMGSFSAIVFGGIQPAYSYAMGSMISIYFLADHNEIKDKTRTYTLIFVALA
VLSFLINIGQHYNFGAMGEYLTKRVREQMLAKILTFEIGWFDRDENSSGAICSQLAKDANVVRSLVGDRMALV
IQTVSAVLTACTMGLVIAWRLALVMIAVQPLIILCFYTRRVLLKSMSTKSIQAQSESSRLAAEAVSNLRTITAFSS
QERILRLFDQAQDGPRKESIRQSWFAGLGLGTSMSLMTCTWALDFWYGGKLVAEHHITSKALFQTFMILVSTG
RVIADAGSMTTDLAKGADAVASVFAVLDRETEIDPDNPEGYKPERLKGEVDIRGVDFAYPSRPDVIIFKGFSLSI
QPGKSTALVGQSGSGKSTIIGLIERFYDPLRGVVKIDGKDIKTYNLRGLRRHIGLVSQEPTLFAGTIRENIVYGTE
TATEAEIENAARSANAHDFISNLKDGYDTWCGERGVQLSGGQKQRIAIARAILKNPAILLLDEATSALDSQSEK
VVQEALDRVMVGRTSIVVAHRLSTIQNCDQITVLEKGIVVEKGTHASLMAKGTSGTYFGLVSLQQGGNQH

>XP_003530842.1_ABCB15_Gm
MGRDQKSVVAMVGQERKTNKKNGSLGFRSIFMHADGKDLFLMVLGTIGAVGEGLTTPLVLYISSRMMNNIGS
SSNMDGNTFIHSINKNAVSWLYLAGASFAVCFLEGYCWTRTSERQAARMRCRYLKAVLRQDVEYFDLHVTST
SEIITSVSSDSLVIQDVLSEKVPNFLMNMSLFVGSYIAAFAMLWRLAIVGFPFVVLLVIPGLIYGKTLIGLSSKLRE
EYNQAGTVAEQTISSIRTVFSFVGESKTMNAFSNALQGTVKLGLKQGLAKGLAVGSNGVVFGIWSFMCYYGS
RLVIYHGVKGGTVFAVGAAIAVGGLALGAGLSNVRYFSEAGAAAERIKEVIKRVPKIDSDNKEGEILENIYGEV
EFDRVEFAYPSRPESAILKGLNLRVPAGKRVALVGESGSGKSTVIALLQRFYDPCGGEVRVDGVGIQKLQLKW
LRSCMGLVSQEPALFATSIKDNILFGKEDATQDQVVEAAKAAHAHNFISLLPHGYHTQVGERGIQMSGGQKQR
IAIARAIIKKPRILLLDEATSALDSESERLVQEALDNAAVGCTTIIIAHRLSTIQNADLIAVVGGGKIIEMGSHDELI
KNDTGAYASAFRLQQQMGKDKVEESTEKTVIPGTVLSTTETQDMGLTSVGPTISGGCDDNMATAPSFWRLMA
LSYPEWKHGVFGCLNAMVFGAVQPVYAFTMGSTILLYFNSDHEEIMRRTRFYSFTFLGLFVVSLLSNIGQHYCF
GYMGEYLTKRVRETVLAKILTFEVGWFDLDQNSTASICSRLAKDASVVRSLVGDRMALLVQTFSAVITAYTM
GLIISWRLSIVMIAVQPIIIACFYTRRVLLKSMSNKSMKAQQQSSNIASEAVSNLRTVTAFSSQDRILKMLEEAQQ
RPSLENIRQSWFAGIGLGCSQGLASCIWALDFWYGGKLISYGYITTKTFFESFMVLVSTGRIIADAGSMTTDLAR
GADVVGDIFGIIDRCTKIEPDDPNGYIPERLIGEIEFHEVHFAYPARPNVAIFENFSMKIEAGKSTAMVGQSGSGK
STIIGLIERFYDPLKGMVTIDGMDIKSYNLKSLRKHIALVSQEPTLFGGTIRENIAYGRCESERVDESEIIEAARAA
NAHDFIASLKEGYETWCGDKGVQLSGGQKQRIAIARAILKNPKVLLLDEATSALDGPSEKVVQDTLMRVMRG
RTGVVVAHRLSTIHNCDVIGVLEKGRVVEIGTHSSLLAKGSCGAYYSLVSLQTRHATTPNNTSCTKAGSTHSIN
>XP_015625016.1_P_MDR_Os
MGGGDGGAGKAKARPVFSSFMTVFMHADAADVALMVLGLLGAMGDGISTPVMLLITSRIFNDLGSGADIVKE
FSSKVNVNARNLVFLAAASWVMAFLEGYCWARTAERQASRMRARYLRAVLRQDVEYFDLKKGSTAEVITSV
SNDSLVVQDVLSEKVPNFVMNAAMFAGSYAVGFALLWRLTLVALPSVVLLIIPGFMYGRILVGLARRIREQYT
RPGAIAEQAVSSARTVYSFVAERTTMAQFSAALEESARLGLKQGLAKGIAVGSNGITFAIWAFNVWYGSRLVM
YHGYQGGTVFAVSAAIVVGGLALGSGLSNVKYFSEASSAAERILEVIRRVPKIDSESDTGEELANVTGEVEFRN
VEFCYPSRPESPIFVSFNLRVPAGRTVALVGGSGSGKSTVIALLERFYDPSAGEVMVDGVDIRRLRLKWLRAQM
GLVSQEPALFATSIRENILFGKEEATAEEVVAAAKAANAHNFISQLPQGYDTQVGERGVQMSGGQKQRIAIAR
AILKSPKILLLDEATSALDTESERVVQEALDLASMGRTTIVIAHRLSTIRNADIIAVMQSGEVKELGPHDELIAND
NGLYSSLVRLQQTRDSNEIDEIGVTGSTSAVGQSSSHSMSRRFSAASRSSSARSLGDARDDDNTEKPKLPVPSFR
RLLMLNAPEWKQALMGSFSAVVFGGIQPAYAYAMGSMISVYFLTDHAEIKDKTRTYALIFVGLAVLSFLINIG
QHYNFGAMGEYLTKRIREQMLAKILTFEIGWFDRDENSSGAICSQLAKDANVVRSLVGDRMALVIQTISAVLIA
CTMGLVIAWRLALVMIAVQPLIIVCFYARRVLLKSMSKKSIHAQAESSKLAAEAVSNLRTITAFSSQERILRLFE
QSQDGPRKESIRQSWFAGLGLGTSMSLMTCTWALDFWYGGRLMAEHHISAKELFQTFMILVSTGRVIADAGS
MTTDLAKGADAVASVFAVLDRETEIDPDNPQGYKPEKLKGEVDIRGVDFAYPSRPDVIIFKGFTLSIQPGKSTA
LVGQSGSGKSTIIGLIERFYDPIRGSVKIDGRDIKAYNLRALRRHIGLVSQEPTLFAGTIRENIVYGTETASEAEIE
DAARSANAHDFISNLKDGYDTWCGERGVQLSGGQKQRIAIARAILKNPAILLLDEATSALDSQSEKVVQEALD
RVMIGRTSVVVAHRLSTIQNCDLITVLEKGTVVEKGTHASLMAKGLSGTYFSLVNLQQGGNQQVQH

>XP_015625026.1_P_MDR_Os
MGGDDRSAGKAKPVLGSFMTVFMHADAVDVVLMVLGLLGAVGDGLSMPVLLLITGSVYNNFGGGADNVQE

FSSKVNMNARNLLFLAAGQWVMTFLEGYCWTRTAERQASRMRARYLQAVLRQDVEYFDLKKGSTAEVITSV
ANDSLVVQDVLSEKVPNFVMNAAMFVGNYAFGFALMRQLMLVALPSVVLLIIPTFMYGRVVVDLARRIREQY
TRPGAIAEQAMSSVRTVYSFVAERTTMAQFSAALEESVRLGLKQGLAKGVAIGSNGITFAILAFNVWYGSRLV
MSHGYKGGTVFVVSYAVIQGGLALGSVLSNVKYLSEASSAAERILEVIRRVPKIDSESDTGEELGNVAGEVEFR
NVKFCYPSRPESPIFVSFNLRVPAGRTVALVGGSGSGKSTVIALLERFYDPSAGEVMVDGVDIRRLRLKWLRAQ
MGLVSQEPALFATSIRENILFGKEDATAEEVIAAAKAANAHSFISQLPQGYDTQVGERGVQMSGGQKQRIAIAR
AILKSPKILLLDEATSALDTESESVVQEALDLASMGRTTIVIAHRLSTIRNADIIAVMQSGEVKELGSHDELIANE
NGLYSSLVRLQQTRDSNEIDEIGVIGSTSALGQSSSHSMSRRFSAASRSSSVRSLGDARDADNTEKPKLPVPSFR
RLLMLNAPEWKQALIGSFGAVVFGGIQPAFAYAMGSMISVYFLTDHAEIKDKTRTYALIFVGLAVLSFLINIGQ
HYNFGAMGEYLTKRIREQMLAKILTFEIGWFDRDENSSGAICSQLAKDANVVRSLVGDRMALVIQTISAVLIAC
TMGLVIAWRLALVMIAVQPLIIVCFYARRVLLKSMSKKSIHAQAESSKLAAEAVSNLRTITAFSSQERILRLFDQ
SQDGPRKESIRQSWFAGLGLGTAMSLMACSWTIGFWYSGRLMAEHQITAKEIFQTFIILASTGRVIAEAGSMTT
DLAKGADAVASVFAVLDRETEIDPDNPQGYKPEKLKGEVDIRRVDFAYPSRPDVIIFKGFTLSIQPGKSTALVG
QSGSGKSTIIGLIERFYDPIRGSVKIDGRDIKAYNPRALRRHIGLVSQEPTLFAGTIRENIVYGTETASEAEIEDAA
RSANAHDFISNLKDGYGTWCGERGVQLSGGQKQRIAIARAILKNPAILLLDEATSALDSQSEKVVQEALDRVMI
DRTSVVVAHRLSTIQNCDLITVLEKGIVVEKGTHASLMAKGPSGTYFSLVSMKQRGNQQVQQ
>XP_003622707.2_ABCB15_Mt
MNGSIRSIFMHADGEDWFLMILGTIGAIGEGFNAPLILYICSHMINNIGSSSTMDVDTFIHNINKNALVWLYLAC
ATFLVCFLEGYCWTRTSGRQAARMRYKYLKAVLRQEVAYFDLQVTSTSEIITSVSNDTIVIQDVLSEKVPNFLM
NISLFIGSYIVAFTMLWRMAIVAFPSVILLVIPGIIYGKVLMGLSCKIREEYNQAGTIAEQTISTIRTVYSFVGENK
SMFAFSNALQGIVNLGLKQGLAKGLAIGSNGVVFAIWSFMCYYGSKLVMYHGAKGGTVFAVGASITVGGLGL
GASLLNIKYFSEACSAGERIKRVIERVPKIDSNNTKGEILNNVFGEVEFDHVEFAYPTRPETIILKNLCLKIPAGKT
MALVGESGSGKSTVISLLQRFYDPIGGEIRLDGVAIRNLQIKWLRSMMGLVSQEPALFATSIKENIIFGKEDATE
DEIVEAAKICNAHDFISLLPQGYNTQVGERGIQLSGGQKQRIAIARAIIKKPRIFLLDEATSALDTESEKMVQQAL
ENATNGCTAIIIAHRLSTIQNADIVAVVDDGRVNEIGSQDELLENENGIYSSLVRLQQTNKSKTQSDETVTATFT
NVDTDITCLVDPTSSAEDHISVHQASTSNNKNEEDVKQLNNPVSFWRLLLLNAPEWKQAVLGCLSAMVFGAV
QPVYAFAMGSMISVYFQTDYEELKNKIKIYSLCFLCLSLISLVVNVGQHYNFAYMGEYLTKRVRESMFSKMLT
FEVGWFDREENSSGAICSRLANDANVVRSLVGDRMALLVQAFSAVATAYTMGLIISWRLNLVMIAIQPIIIACF
YTRSVLLKSMSSKSMKAQQQSSKIAAEAVSNHRTITAFSSQDRILKMLETSQQDPIQENFRQSWFAGIGLGFSQF
LLSCSWAMNYWYGAKLVADGNITRKALFESFMVVVSTGRVIGDAGSMTKDLAKGVDVVSSIFAILDRSTKIKP
DNPNGFKPDTLMGHIELYDVHFAYPARPNVAIFQGFSIKIEAGKSTALVGQSGSGKSTIIGLIERFYDPIKGNVTI
DGTNIKSYNLKSLRKHIALVSQEPTLINGTIRDNIAYGTTTCDNIDETEIIEASRVANAHDFIASLKDGYETWCGD
KGVQLSGGQKQRIAIARAMLKNPKVLLLDEATSALDNNSEKVVQDALNKVMVGRTSVVVAHRLSTIHNCDVI
AVLEKGKMVEIGTHKALLDKGPFGAYYSLVSLQTKHA
>XP_006361363.1_P_ABCB15-l_St
MITTIFMHADGVDILLMTLGFLGAVGDGASFPVMLIAIAKLMNIIGGLNTSNVLNFRHNINENVMLLIYVACAK
WIACFLEGFCWTRTAERQASRLRIRYLKAVLRQDVGYFDLHVASTANVIASVSGDSLVIQDCISEKVPLFLRDV
STFIGAYVVGFLMIWRLALIAFPMVFLLMIPSMIYGRALMRISRKMRDEYSKAGSIVEQVISSIRTVYSFVGERK
SIEDYCVALDGCVELGVKQGLAKGLFIGSNGFGFAIRALMSYYGSRLVMYNGAHGGNVFMVTLAISLGGISLS
SGLSNIKDFAEAKVANERVMEIIKRVPKIDSENMEGQTLDKMTGEIEFKHIEFAYPSRPESIVLKDFNLKIPRGKT
VALVGGSGSGKSTVIALLQRFYDPLAGEILLDGVVINRLQPKWLRSQMGLVSQEPALFATTIKENILFGKEDAS
MEQVIEAAKASNAHNFICQLPQGYYTKVGERGIQMSGGQKQRIAIARAIIKSPRILLLDEATSALDTASEIVVQE
ALDNASIGRTTIIVAHRLSTIRNADLIALVQNGQVKEIGSHNELIKNQENRLYASLVRLQQTEKPAGATIVSAQQ
SANRDDSKHTSIPCFSIEAKSTVKNAAVPSTSGEGSFKRLLAMNLPEWKQATLGCIGAILVGGVQPVYSFVMGA
MISVYFSPSHDEIKKKTKIYTLAFLGMTFITLVLNVLQHYNFAVMGERLNKRVRERMLSKILTFEVGWYDKEQ
NSTAAICSRLADEASVVRSLVGDRMSLFIQTIAGMTIACMVGLVIAWRMSLVLFTVQPVIILCLYCKRVLLKSM
SEKSIKAQEESSKLAAEAVTNLRTVTAFSSQARILQMLKEAQEGPLRESIRQSWFAGIVLGTTNSLQSCTWALFF
WYGGYLMAEGNIGAQALFQTFVLLSSNGLVIADLGTMTKDLARGTDAVSSVFATLDRYSLIEPEDSDGYKPRK
ITGHIEMCEVDFAYPARPSVIIFKGFSITIDAGKSTALVGQSGSGKSTIIGLIERFYDPLSGVINIDGRDIRSYHLKSL
RKHIALVSQEPTLFSGTIRENIAYGVLASEEVDESEIIEAAKAANVHSFVSALKDGYDTWCGDRGLQLSGGQKQ
RIAIARAILKNPGVLLLDEATSALDSQSEKLVQDALERVMVRRTSVVVAHRLCTIQNCDAIVVLDKGKVVEKG
THSSLLANRPCGVYYSLVRSVNHHP
>XP_003554864.1_ABCB15_Gm
MREDQNHIGVDTKKKNGSIGSIFMHADGLDWFLMVLGVFGAMGDGFSSPVMMYFIGRIVNNIGDVSKITPSTF

MHNVNKYSLALSYFASASFFTSFLEGYCWTRTSERQAARMRVKYLKAVLRQDVSYFDLHVTSKSEVLTCVSS
DSLVIQEVLSEKVPNFLMNFFRFVGSYIAAFVLLWKLAIVAFPFVVLLVIPGLIYGKTMMGLARRIREESNKAGT
IAEQAIFSIRTVYSFVGESKTINAFSEALQGSVKLGLRQGLAKGLAIGSNGVVFAIWSFMVYYGSRLVMYHGAK
GGTVFAVGSVICIGGSALGASLSELKYITEACVAGERIMEMIKRVPNIDSENMAGVILEKVSGEVEFDHVKFIYP
SRPDNVILNDFCLRIPAGKTLALVGGSGSGKSTVISLLQRFYDPIEGEIRLDGVAYHRLQLKWLRSQMGLVSQEP
TLFATSIKKNILFGREDANEEEIVEAAKAANAHDFISQLPQGYNTQVGEKGVQISGGQKQKIAIARAIIKKPQILL
LDEATSALDSESERKVQEALDKIVLDRTTIIIAHRLSTIRDAHVIIVLENGKIMEMGSHDELIQNNNGYYTSLVHF
QQVEKSKNDAFFHPLISNGDMQNTSSHMARHSVSTNSMAQFSFVDGDNTEKVRDDDQKLPSPSFWRLLSSNL
REWKQTCFGCLSALLFGAIEPLYAFAMGSMVSIFFLSNHDEIKRKIILYSLFFVGLAVLSLVLNIIQHYSFAYMGE
YLTKRLKEKMLSKILNFEIAWFDRDENSTGVVCSRLIKEANIVRSLVGDRMAQLVQTISSVVIACTMGLIIAWR
YAIVIIVVQPIIIACFYTRCVLLKGMSEKAIKAQDKSSKIAIEAISNFRTITSFSSQDHVIKMLKKAQEGPSHESIQQ
SWFVGIGLGCARSLKTLTQALEFWYGGKLVFHGYITSKALFEICLIFANIGRVIADASSLANDIAKGVTVSGLVF
SILDRNTKIEPHETNAYKPQKLTGDIELQDVYFAYPSRPNVMIFQDFSMKIEAGKSTALVGQSGSGKSTIIGLIER
FYDPLEGIVTMDGIDIRSYHLRSLRNYIALVSQEPTLFNGTIRENIAYGAFDKTNEAEIIEAARIANAHDFIASMK
DGYDTWCGDRGLQLSGGQKQRIAIARAVLKNPNVLLLDEATSAIDSQAENVVQNALERVMVGRTSVVVAHR
LNTIKNCNQIVVLDKGRVVEEGNHTSLLAKGPNGVYYSLASLQRSLVTTSVINTE

>XP_021316184.1_P_MDR_Sb
MLTTKFALVFKHADAVDVALMVVGLVGAIGDGMSTPVMLAITSRVFDDAGSGPDHLQQFVPKMNENVRYTL
YLAAAHGITAFLEGYCWTRTAERQAKRMRLRYLRAVLRQDVEYFDLKTGGSTSSEVITSVSNDSLAVQDVLSE
KLPNFLTNVAAFVASYAVAFLLMWRLTLVALPSVLLLIIPGFLYSRVLISLARRIRELHTRPGAIAEQAISSVRTV
YSFVAERSTAARFAAALDESVRLGLKQGLAKGVALGTGGIRIAILAFTVWYGSRLVMYHGYRGGTVYNVALII
VFGGGALGTALSNIKYLSEATSAAERIMELIRRVPKIDSESSAGDVLDNVAGEVEFRNVEFCYPSRPKSPIFVNF
NLHVPAGRTVALVGESGSGKSTVIALLERFYDPSAGEVTLDGVDIRRLRLKWLRAQMGLVSQEPALFATSIRE
NILLGKEDATEEEVVAAANAANAHSFISQLPRGYDTQVGERGIQMSGGQKQRIAIARAILKSPKILLLDEATSAL
DTNSEHVVQEALDLASMGRTTIIIAHRLSTIRNAHLIAFMKSGEVKELGSHDDLIANENGLYSTLVHLQQTRDPT
ETTEVCETRNAYVMLQSNKQSRGFSTASRSITTRSMGDTKDNGNIENPKLPVPSFKRLLMLNAPEWKQGLLGS
FSAIVSGGIQPAFCYIMGSMISIYFSTDHEEIKEKTRTYALISIGLAMLTFMVNIGQHYNFASTGEYLTKRVREQM
LAKYLTFEIGWFDQDDNSSSSICSRLTTDANIVRSLVGDRMSLVIHTVSAVLTAYIMGLVIAWRLAILMIAVQPF
SIICYYTRYVLLKSMSEKSTQAQAECSKLAAEAVSNLRTISAFSSQNRIMCLFDQAQEGPCKESIRQSWFAGIGL
GTSTSLLRCTWALTFWYTGILLDGHHITAKAFFQTFLILISTTLVIADAGSMTADLAKGADAVASVFAILDRETK
IDPDNPEGYKPEKLKGEVDIRGVDFAYPSRPDVIIFKRFSLSIQPAKSTALVGQSGSGKSTIIGLIERFYDPTSGVV
EIDCKDIKAYNLRSLRQHIGLVSQEPTLFAGTIRENIVYGTEAASEEEIENAARSANAHGFISNLKDGYETRCGE
QGVQLSGGQKQRIAIARAILKNPAILLLDEATSALDSQSENVVQEALDRLMVGRTSIVVAHRLSTIQNCNMIVV
LEKGVVVETGTHASLMTKGPAGTYFGLVNLQQGGNQVNNAMALQENPSIPDLQPKITTLD

>XP_002452253.1_P_MDR_Sb
MDAAGRRAKKVDAASLVALGSSVFVHADAAVDVALMVLGLVGAIGDGMATPLRLLVASRIANDLGSGPDHL
QQFTSKINANVIRIVYIACVSWVRAFLEGYCWARTAERQASPMRSRYLQAVLRQDVEFFDLKPGWTSEVVTSV
SNDSLVVQDALSEKLPSFAMYATTFAGSYAVGFALLWRLTLVTLPSALLLVVPGVSYGRALTGLARKIRDQYA
LPGAVAQQAVSSARTVYAFVAEKTTMARFSAALQESARLGLRQGLAKGFALGTNGIAFAIYAFNIWYGGRLV
MYHGYPGGTVFVVSSLIVIGGVSLGAALSNVKYFSEATAAADRILEMIQRVPKIDSESGAGEELANVAGEVEFR
NVDFCHPSRPESPVLANFSLRVPAGHTVALVGPSGSGKSTAIALLERFYDSSAGEVALDGVDIRRLRLKWLRAQ
MGLVSQEPAMFAMSVRENILFGEEDATGEEVVAAAMAANAHSFISQLPQGYDTQVGERGAQMSGGQKQRIAI
ARAILRSPKILLLDEATSALDTESEHVVQEALDAASVGRTTILVAHRLSTVRNADSIAVMQSGSVQELGSHSEL
VAKNGMYSSLVHLQHNRDLNEDTGEDGGTCGASPSAGQCNSNNGKMVSSASRSSSTRSVGDAGDGENADEK
PKPPVPSFGRLLLLNAPEWKFALVGSSCAVLSGAIQPIFAYGMGCTFSIYYSTDHEEIKDKTRMYAFIFLALVAL
SFMLSIGQHYSFAAMGECLTKRIRERMLAKILTFEIGWFDQDNNSTGNICSQLAKEANIVRSLVGDRMALLIQT
GSMVVIAFTVGLVISWRLALVMIALQPFIIACSYARRVLLKNMSMKSIQAQSETSKLAADAVSNLRTITAFSSQG
RILRLFSHAQHGPHKESIRQSWFAGLGLGASVSLTIFSWALNYWYSGKLMAERLIAVEAVFQTSMILVSTGRLI
ADACSMTTDIAKGAEAVSSVFTILDRQTKIDPDNPKGYKPEKLIGDVEIVGVDFAYPSRPDVTIFRGFSLSMMA
GKSTALVGQSGSGKSTIIGLIERFYDPLKGVVNIDGRDIKAYNLQALRRHIGLVSQEPTLFAGTIKENIMLEAEM
ASEAEVEEAARSANAHDFISNLKDGYDTWCGDRGVQLSGGQKQRIAIARAILKNPAILLLDEATSALDSQSEKA

VQEALDRVMVGRTSMVVAHRLSTIQSCDMIAVLDRGVVVEKGTHASLMANGLSGTYFGLVTLQQGGKQH
>XP_006361362.1_P_ABCB15-l_St
MIFMNADGVDILLITLGFLGAVGNGACFSIALIVTTKLMNIIGGADTSNSLNFMHNINENVRLLIYIAGAKWITC
FLEGFCWTRTAKRKVSRLRIRYLKAVLRQDVGFKASVSDDCLVIQDWIGEKVPFFLRDMSNFIGAYVVGFLMI
WRLVLVIFSIFFLLMIPSIIYGRALKRISRKVRDESNKAGNILEQWISSIRTVYSFVGERKSIEDYCVALEGCVELG
VKQSLVKGLFFGSCGFVTAFAITALLSYYGSILVMYNGVHGGNVYMVTLAIFWGGKRLGSGLLNIKDFAEAV
AANKRVMEVMKTVPKIDSENMEGQALDNMTGEIEFKRVKFTYPSGPESIGLKDFSLKISRGKTVALVGGSGSA
VIALLQRFYNPMAGEILLDGVVINKLQPKWLRYQMSLVSNEPALFATTIKENILFGKEDASMEQVIEAAKASNA
HDFICKLPQGYYTKVGEKGIQMSEGQKQRIAIARAIIKSPRILLLDEATSALDTASEIVVQEALDNASIGRTTIIVA
HRLSTTRNADLIAFVQNGQVKEIGSHNELIKNQGLYASLVRLQQTEKPTGATIASSQQSSSIANQDDTKHVSITS
LLTEAKSTEKNATVPSTSGQGSFKRLLAMNLPEWKQATLGCIGAILVGGVLPVYAFLMGAMISVSYSPSHDEIK
KKTKIYTLAFLGMAFITLVLNVLQHYNFAVMGERLTKRVRERMLSKMLTFEVGWYDKEQNSTAAICSRLTDD
ASVVRSLVGDRMSLFIQTIAGMTIACAVGLVIAWRMGLVMIAVQAFIIFSIYCRRVLLKSMLEKSIQAQEESSKL
AAEAVTNLRTVTAFNSQSRILQMLKEAQEGPLRENIQQSWLSGIILGTTISLQLCTWALFFWFGGYFMVEGYIG
AQALFQILVLLISNWRVIADLGTMSKDLDRGTDAVGSVFTTLDRFSLIEPEDSDGYKPKKITGHIEMYEVDFAY
PARPNVIIFKGFSITIDAGKSTAFVGQSGSGKSTIIGLIERFYDPLSGVIKIDGRDIRSYHLKSLRNQIALVSQEPTLF
SGTIRENIAYGVLASEEVDESEIIEAAKAANVHSFISALKDGYDTWCGDKGLQLSRGQKQRIAIARALLKNPGV
VLLDKATSALDSQSEKLVQEALERVMVGRTSVVVTHRLSTIQNCDTIVVLDKGKVVEKGTHSSLLAKRPCGV
YYSLVSSANHHP
>XP_008645788.2_P_MDR_Zm
MAFLEGYCWARTAERQASRMRARYLQAVLRQDVEFFDLKPGSTSEVVTSVSNDSLVVQDALSEKVPNFAMY
VTTFAGSYAVGFALLWRLTLVTLPSALLLIIPGVSYGRVLTGLARRIRERYALPGAVAQQAVSSVRTVYSFGAE
RATMARFSSALEESARLGLRQGLAKGVALGTNGIAFAIYAFNIWYGGRLVMYHGYPGGTVFVVSSLIVIGGVS
LGSALSNVKYFSEATAAADRILEMIRRVPKIDSESAAGEELPNVAGEVEFRNVDFCYPSRPESPVLVDFSLRVPA
GHTVALVGPSGSGKSTAITLLERFYDPSAGEVALDGVDIRRLRLKWLRAQMGLVSQEPALFAMSLRENILFGEE
DATEEEVVAAAMAANAHSFISQLPQGYDTLVGERGAQMSGGQKQRIAIARAILRSPKILLLDEATSALDTESER
VVQEALDVASVGRTTILVAHRLSTVRNADSIAVMQSGAVQELGSHSELIAKNGLYSSLVHLQQNRDSSEDTGE
AAGTRRASPSAGQCSSDDSKMAPSASCRSSSARSIIGDDARDGENTDEKPRPPVPSFGRLLLLNAPEWKHALVG
SSCAVLSGAIQPIFAYGMGCTFSIYYSRDHEEIKDKTEKYAFVFLALVGISFLLNIGQHYSFGAMGECLTKRIRK
QMLAKILTFEIGWFDHDDNSTGNICSQLAKDANIVRSLVGDRMALLIQTASMVVIAFTVGLVISWRLALVMIA
MQPFIIACSYARRVLLKNMSTKSIQAQSETSKLAADAVSNLRTVTAFSSQGRVLRLFGQAQDGPHRESVRQSW
FAGLGLSASVSLTIFSWALNYWYSGKLMAERLITVEAVFQATMILVTTGRVIADACSMTTDIAKGAEAVSSVF
AILDRQTKIDPDSPEGYKPEKLIGEVEAVGVDFAYPSRPDVIIFRGFSLSMVAGKSTALVGQSGSGKSTIIALIERF
YDPLKGVVNIDGRDIKAYNLQALRRHIGLVSQEPTLFAGTIKENIMLEAEAASEAEVEEAARSANAHGFISNLK
DGYDTWCGDRGVQLSGGQKQRVAIARAILKNPAILLLDEATSALDSQSEKAVQEALDRVMVGRTSVVVAHRL
STIQGCDTIAVLDRGVVVEKGTHTSLMASGRSGTYFGLVALQQGGKQH

>XP_024447653.1_ABCB25_Pt
MLVTATAATVASASNKCFRAHNNLHLLRHHHSNYYKYKNNNKNPLLFLCCFNHSLPNHHHTRRCRLLLGPSS
TDSSTALHASFPFLSNTSYSTPPSWNPNAFLSSSSSSSTPFRPPNSMLNGHAFFSTSAPKDKDDDDAANKASSSPS
ITTPAIADKSDQQLADMKILRTLASYLWMKDNPEFRLRVLLALGFLLVAKVLNVQVPFLFKLAVDWLTVATG
NATALASFTAANSTFLALFATPASVLIGYGIARCGSSAFNELRTAVFSKVALRTIRTVSGKVFSHLHELDLRFHL
SRETGGLSRIIDRGSRAINFILSSMVFNVVPTILEISMVSSILAYKFGAPFALITSLSVAAYVTFTLSVTQWRTKFR
KAMNKADNDASTKAIDSLINYETVKYFNNEAYEAEQYDEYLKRYEDAALKTSQSLAFLNFGQNVIFSTALSTA
MVLCSHGIMNGQMTVGDLVMVNGLLFQLSLPLNFLGSVYRETIQSLVDMKSMFHLLEEKPDIRDKDDAKPLM
LKGGGIQFDNVHFSYLEERKILDGVTFSVPAGKSVAIVGTSGSGKSTILRLLFRLFDTISGSIQIDGQDTRDVTLD
SLRRSIGVVPQDTVLFNDTIFHNIHYGRLSATREEVYDAARHAAIHDTVMNFPEKYSTIVGERGLKLSGGEKQR
VALARAFLKAAPIMLCDEATSALDSTTEAEILNALKSLSSNRTSVFIAHRLTTAMQCDEIIVLENGKVVEQGPHE
VLLTKAGRYAQLWAQQNSTVDALDSAIKLEA
>XP_010650796.1_P_ABCB25_X1_Vv
MAGVASCSRLFSGNSKRFLLRYSHRNNSTINICNGRELGIARNLNITRRIHFFRHLNSFLSDSPPSYRPPSPYHRH
PEQSWTLKGYALFSTSTAGDVTATKQVGKKNQAKVVSDEHAADMKILRTLAKYLWSKDNPEFRFRVIMALG
FLVGAKVLNVQVPFLFKLAVDWLTTTTGNATALASFTTANSTALALFVSPAAVLVGYGIARSGASAFNELRTA
VFSKVALRTIRSVSRRVFSHLHDLDLQYHLSRETGALNRIIDRGSRAINFILSSMVFNIVPTILEISMVAGILAYKF

GASFAWITSLSVAAYVAFTLAVTQWRTKFRKIMNKADNDASTRAIDSLINYETVKYFNSEAFEVEKYDELLKR
YEDAALKTQRSLAFLNFGQNLIFSTALSTAMVLCSHGIMNGEMTVGDLVMVNGLLFQLSLPLNFLGSVYRETI
QSLVDMKSMFQLLEERPDIRNADDAKPLKLSGGSIQFSNVHFSYLTERKILDGISFVVPAGKSVAIVGTSGSGKS
TILRLLFRFFDAQCGTICIDGEDIRKVTLESLRKSIGVVPQDTVLFNDTIFHNIQYGRLSATNEEVYDAARRAAIH
DTIMNFPEKYSTVVGERGLKLSGGEKQRVALARAFLKAPAILLCDEATSALDSTTESEILNALKTLANNRTSIFI
AHRLTTAMQCDEIIVLENGTVIEQGPHEVLLSKAGRYAQLWGQQNNSPDGVDPTIKLEA

>XP_003518353.1_ABCB25_Gm
MLAAAASASRHLLQRDATSIGACSGSGNRVRSLLTASAITTTYPIRFRKCYGISFNNPKHRTCPSLFLAVKGFLS
DSSNSNSPHGRVTRPYLPNPVTSSTVDGRALFSTSTKTDDGSQSQKAAASSTIGKSSPPPPPGGQAADVKILRTL
ASYLWMKDNLEFRVRIVAALGLLVGAKILNVQVPFLFKLAVDSLTAATGSVGALASNSSGLAFFATPVAVLIG
YGIARSGASAFNELRTAVFAKVALRTIRLVSRKVFSHLHDLDLRYHLSRETGALSRIIDRGSRGINFILSSMVFNV
VPTILEISMVAGILAYKFGAPFALITSLSVAAYVTFTLTITQWRTKFRKAMNKADNDASTRVIDSLINYETVKYF
NNEVYEADNYDKYLKRYEDAALKTQRSLALLNFGQNVIFSTALSSAMVLCSHGIMDGTMTVGDLVMVNGLL
FQLSLPLNFLGSVYRETIQSLVDMKSMFQLLEERADIRDKENAKPLRFNGGRIQFENVHFSYLTERKILDGISFV
VPAGKSVAIVGTSGSGKSTILRLLFRFFDPHFGSIKIDDQDIREVTFESLRKSIGVVPQDTVLFNDTIFHNIHYGRL
SATEEEVYEAAQQAAIHNTIMKFPDKYSTVVGERGLKLSGGEKQRVALARAFLKAPAILLCDEATSALDSTTE
AEILSALNSVANNRTSIFIAHRLTTAMQCDEIIVLENGKVIEQGPHEVLLSKAGRYAQLWGQQNNSVDAVDTAI
KLGA
>XP_004247015.1_ABCB25_Sl
MQGSIYSRCFRGRELLLHKAQKMILNYIPSRPLPSQLLNPPNYRTHLSTIKRYPYPWDSSRTGFKQIKAFISEFPA
KNPDGKSNGHAMFSTSSAGEKKPLLNKEQTQPATEADQISNARIISTLAKYLWMKDNFEFRFRVIAALTLLVG
AKVVNVQVPFLFKLAVDWLSTATGNASALAEFTTANSTVLALFVSPAAVLIGYGIARSGASAFNELRTAIFSKV
ALRTIRSVSRKVFSHLHELDLQYHLSRETGGLNRVIDRGSRAINFILSSMVFNVFPTILEISMVSGILAYRFGASFA
WITSLSVAAYIAFTLSITQWRTKFRKEMNKADNDASTRAIDSLINYETVKYFNNEVYETEKYDHFLKRYEDAA
LKTQRSLAILNFGQSAIFSIALSAAMVLCSNGIMNGVMTVGDLVMVNGLLFQLSLPLNFLGSVYRETIQSLVDM
KSMFQLLEEKAQIGDAADAKSLNLSGGSIQFENVHFSYLTERKILDGISFIVPAGKSVAIVGTSGSGKSTILRLLF
RFFDTHSGNIKIDGQDIREVTLESLRKSIGVVPQDTVLFNDTIFHNIRYGRLSASEEEVYNAARQAAIHDTIIKFPQ
KYATVVGERGLKLSGGEKQRVALARAFLKAPAILLCDEATSALDSTTESEILSALKSLAINRTAVFIAHRLTTA
MQCDEIIVLENGKVVEQGPHEFLLSRPGRYAQLWSQQNTVDGLDVAIKLNP
>XP_003616083.2_ABCB25_Mt
MLAAASRHARYTPGRNNRVRSLLNASGITTGYSIPTSNGISYSYSYSNLNTRTSPSLLFSIKAFLSDPSSSHNRSL
KVDGRALFSTSAKTDEGSQRNKKTDSNVAKSPPGGADLRILKSLAGYLWMKDNPEFRFRVVAALCLLVGAKV
VNVQVPFLFKLAVDWLNTATGNAGLVASSPTVMTLFATPAAVLIGYGIARSGASAFNELRTALFSKVALRTIRL
VSRKVFSHLHDLDLQYHLSRETGALSRIIDRGSRAINFILSAMVFNVLPTIIEISMVSGILAYKFGAPFAWITSLSV
AAYVAFTLSITQWRTKFRKAMNKADNDATTRAIDSLINYETVKYFNNEGHETDHYDKYLERYEDAALKTQHS
LALLNFGQNAIFSAALSTAMVLCSHGIMNGTMTVGDLVMVNGLLFQLSLPLNFLGSVYRETIQSLIDMKSMFQ
LLEEKADIKDKENAQPLRFNGGKIQFQNVHFSYLTERKILDGISLLVPAGKSVAIVGTSGSGKSTILRMLFRFFDP
HSGSIKIDDQDIRDVTLESLRKSIGVVPQDTVLFNDTIFHNIHYGRLSAAEEEVYEAARKAAIHDTIMSFPEKYAT
VVGERGLKLSGGEKQRVALARAFLKAPAILLCDEATSALDSTTEAEILSALKSLSNNRTSIFIAHRLTTAMQCDK
IIVLENGKVVEHGPHEVLLANAGRYSQLWGQQNNTIDAIDPATKLGA

>XP_015644037.1_ABCB25_Os
MRPTSRILAAGHLLRGSRSRYDPSPVAAAAPIFRRPPTVPRPLPSPLLGGFGPNCWVYPGDGKYAPFGRLSCFM
SDSTYPPPPRDVRGHAFSTSANAVAVGKSSDDKVKKDISKKDVDDQIADTQILKNLGKYLLLNDSPDFRFRLIL
SLGLLVGAKVINVQVPFLFKLAVDWLAALAGAETSLASFTEANATLLALFASPAAVLIGYGIARSGVSACTELR
NAVFSKVTLRAIRSVSSTVFSHLHELDLRYHLSRQTGALNRIIDRGSRAINYILTVMVFNVVPTILEIGMVSSILA
YKFGSTFAWITSVSVATYIAFTLAVTQWRTKFRTAMNKADNASSTVAVDSLLNYETVKYFNNEQFEVEKYDK
YLKKYEDAALKTQSSLAYLNFGQNIIFSSALSTAMVLSSYGVMSGALTVGDLVMVNGLLFQLSLPLNFLGSVY
RESRQSLIDMKSMFQLLEEKPGIKDEPHAQPLQFKGGRIEFENVHFGYVPERKILKGATFTVPAGKSVAIVGTSG
SGKSTILRLLFRFFDSSSGSIRIDGQDIREVTLDSLRKCIGVVPQDTVLFNDTIKHNIQYGRLSATDEEVYDVARR

AAIHDTIMNFPDKYNTVVGERGLKLSGGEKQRVSIARVFLKEPSILLCDEATSALDSTTEASILNSLKTLSVDRT
SIFIAHRLTTAMQCDEIIVLENGEVVEQGPHDFLLSKGGRYAELWSQQNNSDAIDAAAVSLEVS
>XP_021320836.1_ABCB25_Sb
MRPCSRILAAGHLLRGSRFDPSQTTSAAAGFRRLNGANRPCVPKPLFSTLLGGFGSNCGVQRGKGVPFGSLGSF
LPDSAYPLHGARLQRDTRGNAFSTSANAVTVKKPVDDKVQKDASKKDVDEEIADSQILKNLGKYLLLNDSPD
FRFRVALSLGLLVGAKVINVQVPFLFKLAIDWLAALGGAEASLASFTETNATLLALFASPAAVLIGYGIARSGV
SACTELRNAVFSKVTLRAIRSVSRKVFSHLHELDLRYHLSRQTGAINRVIDRGSRAINYILTVMVFNVVPTILEIG
MVSSILAYKFGSTFAWITSASVATYIAFTLAVTQWRTKFRMAMNKADNASSTVAVDSLLNYETVKYFNNEQF
EVEKYDKYLRKYEDAALKTQSSLAYLNFGQNVIFSSALSAAMVLSSYGIMSGALSVGDLVMVNGLLFQLSLPL
NFLGSVYRESRQSLIDMKSLFQLLEEKPGIKDEPHAQPLQFKGGCIEFENVHFGYVPERKILDGATFTVPSGKSV
AIVGSSGSGKSTILRLLFRFFDSTSGSIRIDGQDIRGVTLESLRKCLGVVPQDTVLFNDTIKHNIQYGRLSAKDEE
VYDAARRAAIHDTIMNFPDKYDTVVGERGLKLSGGEKQRVSIARVFLKEPSILLCDEATSALDSTTEASILNSLK
SLSVDRTSIFIAHRLTTAMQCDQIIVLENGKVVEEGPHDFLLSKGGRYAELWYQQNSSDASDSAAVSLEV

>XP_024376246.1_ABCB25-l_Pp
MRSSLIRRSSGAAEMLRRVVRKAVQASRSLPCAENRGSPLLAVLPRSGIDTASENVTGDQWRWHGKDRNFTV
EACGEIGFRGVSVTDLLNKHGADENFTRYLHTRAAQHTVGSSTKPTLNLRGLSSLVDAGKSQPPKNNKNDMS
VKEDAEAVVADARILKSLAAYLWPKDNPEFRRRVTLALSLLVASKVLNVQVPFMFKYAVDALSAAAGATGA
TAAASAATTSTALMFATPTSVLLGYGIARAGASACNELRNAVFAKVAQGTIRNVARKVFMHLHHLDLSYHLS
RQTGSLNRIIDRGTRAINFILSSMVFNVVPTLLEISMVAGILAYKFGAPFAWITSLTVAAYTLFTLTVTQWRTKF
RQDMNKADNAASSRATDSLINYETVKYFNNEEHEAKRYDEFLKKYEGAALKTQTSLSSLNFGQNVIFSAALST
AMIMCSRGILDGTMSIGDLVMVNGLLFQLSLPLNFLGTVYRETRQSLIDMHSMFSLLEVKASVADGPHAKPLQ
LKGGSISFDNVHFGYLTDRTILNGISFDVPAGKSVAIVGTSGSGKSTILRLLYRFFDADSGSVKVDGQDVRDVTL
DSLRKCIGVVPQDTVLFNDTIFYNIHYGRMTASREEVIEAARQAAIHDVIMRFPDQYETKVGERGLKLSGGEKQ
RVALARAFLKASPLLLCDEATSALDSSTEGEILTALRSLANDRTSIFIAHRLTTAMQCDEIVVLEEGRVVERGSH
EVLLGLNGRYAQLWNQQNSTDGGSDVDKLNAAL

>XP_006339381.1_P_ABCB25_St
MQGSIYSRCFRGRELLLHKTQKMILNYIPSRLFPSQLLKSPSYRTHLSTIKRYPWDSSRTTSTGFKQIKAFISDFPA
KNPDGKSNGYAMFSTSSTDDAGTKKPLLNKEQTQPATEADQISNARIISTLAKYLWMKDNFEFRFRVIAALSLL
VGAKVVNVQVPFLFKLAVDWLSTATGNASTLAEFTAANSTVLALFVSPAAVLIGYGIARSGASAFNELRTAIFS
KVALRTIRSVSRKVFSHLHELDLQYHLSRETGGLNRVIDRGSRAINFILSSMVFNVFPTILEISMVSGILAYKFGA
SFAWITSLSVAAYIAFTLSITQWRTKFRKEMNKADNDASTRAIDSLINYETVKYFNNEVYETEKYDHFLKRYED
AALKTQRSLAILNFGQSAIFSIALSAAMVLCSNGIMNGVMTVGDLVMVNGLLFQLSLPLNFLGSVYRETIQSLV
DMKSMFQLLEEKAQIRDAADAKSLNLSGGSIQFENVHFSYLTERKILDGISFIVPAGKSVAIVGTSGSGKSTILRL
LFRFFDTQSGNVRLKACLIAESFFSFLD

>XP_015650392.1_ABCB1_Os
MEEEIKGRVVVLGADAAADPELEAFHLPSADQPPHSHLLHHHHSPQSHPQPDAPAAAAPPPPAPLTPPPPKSPPP
PPHIQTTDLPPPKPLPPAPLRQLFSFADGLDYVLMTLGTLGALVHGCSLPVFLRFFADLVDSFGSHAAHPDTML
RLVVKYAFYFLVVGAAIWASSWAEISCWMWTGERQSTRMRIRYLHAALHQDVSFFDTDVRTSDVIHAINADA
VVVQDAISEKLGNLIHYLATFVSGFVVGFTAAWQLALVTLAVVPLIAVIGGLSAAALAKLSSRSQDALSDASGI
AEQALAQIRIVQSFVGEERVMRAYSAALAVAQRIGYRSGFAKGIGLGGTYFTVFCCYALLLWYGGHLVRRAH
TNGGLAIATMFSVMIGGLALGQSAPSMAAFAKARVAAAKIFRMMEHKPSMEREGGVELEAVTGRVELRDVEF
SYPSRPDVGILRGLSLSVPAGKTIALVGSSGSGKSTVVSLIERFYEPNAGTILLDGHDLRDLNLRWLRRQIGLVS
QEPALFATTIRENLLLGRDGATQEELEEAARVANAHSFIVKLPDAYNTQVGERGLQLSGGQKQRIAIARAMLR
NPAILLLDEATSALDSESEKLVQEALDRFMIGRTTLVIAHRLSTIRKADLVAVLQGGAISEVGTHDELMARGDG
TYARLIRMQEQAHEAALVAARRSSARPSSARNSVSSPIITRNSSYGRSPYSRRLSDADFITGLGLGVDSKQQQQQ
HYFRVQASSFWRLAKMNSPEWGYALVASLGSMVCGSFSAIFAYVLSAVLSVYYAPDAAYMDRQIAKYCYLLI
GMSSAALLFNTVQHLFWDTVGENLTKRVRERMLAAVLRNEIAWFDMEDNSSARIAARLALDAQNVRSAIGD
RISIIVQNSALMLVACTAGFVLQWRLALVLLAVFPLVVAATVLQKMFLKGFSGDLERAHARATQIAGEAVAN

VRTVAAFGSEAKIVGLFEANLAGPLRRCFWKGQIAGSGYGVAQFLLYASYALGLWYAAWLVKHGVSDFSKTI
RVFMVLMVSANGAAETLTLAPDFVKGGRAMQAVFEAMDRRTEIEPDDVDAAAVPERPRGEVELKHVDFAYP
SRPEVQVFRDLSLRARAGRTLALVGASGCGKSSVLALVQRFYEPNSGRVLLDGRDLRKFNLRSLRRAMALVP
QEPFLFAATIHDNIAYGREGATEAEVVEAATAANAHKFISALPEGYGTLVGERGVQLSGGQRQRIAIARALVKQ
APILLLDEATSALDAESERSVQEALASSSGSGRTTIVVAHRLATVRNAHTIAVIDDGKVAEQGSHSHLLNHHPD
GCYARMLQLQRLSHSHVAPGPSSSTTTHGT
>XP_002266505.1_P_ABCB1_Vv
MSQEAVEIKTTIEQWRWSEMQGLELVSPNTDDFKSHPTASRVSKSSAEGGEARDMDGTEPKNQPQPQPQPQA
QAQAHASGSGEKTELVPSSGFGELFRFADGLDYVLMTIGSIGAIVHGSSLPIFLRFFADLVNSFGSNANNIDKMM
QEVLKYAFYFLVVGAAIWASSWAEISCWMWTGERQSTKMRIKYLEAALNQDIQFFDTEVRTSDVVFAVNTDA
VMVQDAISEKLGNFIHYMATFVSGFVVGFTAVWQLALVTLAVVPLIAVIGGIHTATLAKLSAKSQEALSEAGNI
AEQTIVQIRVVFAFVGESRALQAYSAALRISQRLGYKSGFSKGMGLGATYFTVFCCYALLLWYGGYLVRHHY
TNGGLAIATMFSVMLGGLALGQSAPSMSAFAKAKVAAAKIFRIIDHKPNIERNGETGLELESVTGQVELKNVDF
SYPSRPEVRILSDFSLNVPAGKTIALVGSSGSGKSTVVSLIERFYDPTSGQVLLDGHDIKTLKLRWLRQQIGLVSQ
EPALFATTIKENMLLGRPDATLVEIEEAARVANAYSFIVKLPEGFDTQVGERGFQLSGGQKQRIAIARAMLKNP
AILLLDEATSALDSESEKLVQEALDRFMIGRTTLVIAHRLSTIRKADLVAVLQQGSVSEIGTHDELIAKGENGVY
AKLIRMQETAHETALSNARKSSARPSSARNSVSSPIIARNSSYGRSPYSRRLSDFSTSDFSLSLDASHPNYRLEKL
AFKEQASSFWRLAKMNSPEWVYALFGTIGSVVCGSISAFFAYVLSAVLSVYYNQNHAYMSKQIGKYCYLLIGV
SSAALLFNTLQHFFWDVVGENLTKRVREKMLAAVLKNEMAWFDQEENESARIAARLALDANNVRSAIGDRIS
VIMQNSALMLVACTAGFVLQWRLALVLIAVFPVVVAATVLQKMFMQGFSGDLEGAHAKATQLAGEAIANVR
TVAAFNSEAKIVGLFSTNLQTPLRRCFWKGQIAGSGYGIAQFLLYASYALGLWYASWLVKHGISDFSKTIRVF
MVLMVSANGAAETLTLAPDFIKGGRAMRSVFDLLDRKTEIEPDDPDAIPVTDRLRGEVELKHVDFSYPSRPDV
PVFRDLCLRARAGKTLALVGPSGCGKSSVIALVQRFYEPTSGRVMIDGKDIRKYNLKSLRRHIAIVPQEPCLFAT
TIYENIAYGHESATEAEIIEAATLANAHKFVSALPDGYKTFVGERGVQLSGGQKQRIAIARAFLRKAELMLLDE
ATSALDAESERCIQEALERACSGKTTIVVAHRLSTIRNAHTIAVIDDGKVAEQGSHSHLLKNYPDGCYARMIQL
QRFTHGQAVGMASGSSSSTRPRDEEEREG
>XP_021619743.1_ABCB1_Me
MSQDSQEIKTIEQWKWSEMQGLELVSPPHPSSEPFKTNSTSSTPTPTLTINSTQQEQNNQTHQPTSPERREMDDT
APKKDGGGDGGSSSNTCGDGEKPGDVAIVGFGELFRFADGLDYVLMAIGSIGALVHGSSLPLFLRFFADLVNSF
GSNANDVDKMMQEVLKYAFYFLIVGAAIWASSWAEISCWMWTGERQTTRMRIKYLEAALNQDIQYFDTEVR
TSDVVFAVNTDAVMVQDAISEKLGNFLHYMATFVSGFVVGFTAVWQLALVTLAVVPLIAVIAAIHTNTLAKLS
GKSQEALSQAGNIVEQTIVQIRVVLAFVGESRALQGYSSALKVAQRNGYKSGFAKGMGLGATYFVVFCCYAL
LLWYGGYLVRHHYTNGGLAIATMFAVMIGGLALGQSAPSMGAFAKAKVAATKIYRIIDHKPAVDRNSESGLE
LDSVTGLVELKNVDFSYPSRPDVRILNNFTLTVPAGKTIALVGSSGSGKSTVVSLIERFYDPNSGQVLLDGHDIK
ALKLRWLRQQIGLVSQEPALFATTIKENILLGRPDADQIEIEEAARVANAHSFIVKLPDGFDTQVGERGLQLSGG
QKQRIAIARAMLKNPAILLLDEATSALDSESEKLVQEALDRFMIGRTTLVIAHRLSTIRKADLVAVLQQGSVTEI
GTHDELIAKGENGVYAKLIRMQETAHETAMNNARKSSARPSSARNSVSSPIIARNSSYGRSPYSRRLSDFSTSDF
SLSLDATHPNYRLEKLAFKEQASSFWRLAKMNSPEWVYALVGSIGSIVCGSLSAFFAYVLSAVLSVYYNPNHA
YMSREIGKYCYLLIGLSSAALIFNTLQHFFWDIVGENLTKRVREKMLAAVLKNEMAWFDQEENESARISARLA
LDANNVRSAIGDRISVIVQNTALMLVACTAGFVLQWRLALVLIAVFPLVVAATVLQKMFMTGFSGDLEAAHA
KATQLAGEAIANVRTVAAFNSESQIVGLFTSNLQIPLRRCFWKGQIAGSGFGIAQFSLYASYALGLWYASWLV
KHGISDFSKTIRVFMVLMVSANGAAETLTLAPDFIKGGRAMRSVFDLLDRKTEIDPDDPDATPVPDRLRGEVEL
KHVDFSYPTRPDVPIFRDLNLRARAGKTLALVGPSGCGKSSVIALVQRFYEPSSGRVMIDGKDIRKYNLKSLRK
HIAMVPQEPCLFATTIYENIAYGHESATEAEIIEAATLANAHKFISGLPDGYKTFVGERGVQLSGGQKQRIAIAR
ALVRKAELMLLDEATSALDAESERSVQEALDRACSGKTTIVVAHRLSTIRNAHVIAVIDDGKVAEQGSHSHLL
KNYPDGCYARMIQLQRFTHSQVIGITSGSSSSARPKDDEEREG
>XP_003554389.1_ABCB1_Gm
MSKDSEEIKTIEQWKWSEMQGLELVPEEGGAAAPSQHQVPREMNTSEPPNKDVGASSAAVTSNGGGEKKEKE
SVPSVGFGELFRFADGLDYVLMGIGTVGAVVHGCSLPLFLRFFADLVNSFGSNANDVDKMTQEVVKYAFYFL
VVGAAIWASSWAEISCWMWSGERQSTKMRIKYLEAALNQDIQFFDTEVRTSDVVFAINTDAVMVQDAISEKL
GNFIHYMATFVSGFVVGFTAVWQLALVTLAVVPMIAVIGGIHTTTLAKLSGKSQEALSQAGNIVEQTIAQIRVV
LAFVGESRALQAYSSALRVAQKIGYKTGFAKGMGLGATYFVVFCCYALLLWYGGYLVRHHATNGGLAIATM
FAVMIGGLGLGQSAPSMAAFTKARVAAAKIFRIIDHKPSIDQNSESGVELDTVTGLVELKNVDFSYPSRPEVQIL
NDFSLNVPAGKTIALVGSSGSGKSTVVSLIERFYDPTSGQVLLDGHDIKTLRLRWLRQQIGLVSQEPALFATTIR

ENILLGRPDADQVEIEEAARVANAHSFIIKLPDGYETQVGERGLQLSGGQKQRIAIARAMLKNPAILLLDEATSA
LDSESEKLVQEALDRFMIGRTTLIIAHRLSTIRKADLVAVLQQGSVSEIGTHDELFSKGENGVYAKLIKMQEMA
HETAMNNARKSSARPSSARNSVSSPIIARNSSYGRSPYSRRLSDFSTSDFSLSLDASHPSYRLEKLAFKEQASSFW
RLAKMNSPEWLYALIGSIGSVVCGSLSAFFAYVLSAVLSVYYNPDHRYMIREIEKYCYLLIGLSSTALLFNTLQH
FFWDIVGENLTKRVREKMLTAVLKNEMAWFDQEENESARIAARLALDANNVRSAIGDRISVIVQNTALMLVA
CTAGFVLQWRLALVLVAVFPVVVAATVLQKMFMTGFSGDLEAAHAKATQLAGEAIANVRTVAAFNSEKKIV
GLFTTNLQAPLQRCFWKGQISGSGYGVAQFALYASYALGLWYASWLVKHGISDFSKTIRVFMVLMVSANGAA
ETLTLAPDFIKGGRAMRSVFDLLDRRTEIEPDDQDATPVPDRLRGEVELKHVDFSYPTRPDMPVFRDLSLRAKA
GKTLALVGPSGCGKSSVIALIQRFYDPTSGRVMIDGKDIRKYNLKSLRRHISVVPQEPCLFATTIYENIAYGHEST
TEAEIIEAATLANAHKFISGLPDGYKTFVGERGVQLSGGQKQRIAVARAFVRKAELMLLDEATSALDAESERSV
QEALDRASSGKTTIIVAHRLSTIRNANLIAVIDDGKVAEQGSHSQLLKNHPDGIYARMIQLQRFTHSQVIGMASG
SSSSTRPKDDEREG
>XP_021623720.1_ABCB1_Me
MSQESEEIKTIEQWKWSEMQGLELVSPAPPPCSDPFKTNSSSSPPSTSTLTIDSQQLQMDNINTTPKKDGGGSVS
NSGGGGGGEKPGDVATVGFVELFRFADGLDYVLMAIGSVGALVHGSSLPLFLRFFADLVNSFGSNANDMDKM
MQEVLKYAFYFLIVGAAIWASSWAEISCWMWTGERQSTRMRIKYLEAALNQDIQYFDTEVRTSDVVFAINTD
AVMVQDAISEKLGNFLHYMATFVSGFVVGFTAVWQLALVTLAVVPLIAVIAAIHTNTLAKLSGKSQEALSQAG
NIVEQTIVQIRVVLAFVGESRALQGYSSALKVAQRIGYKSGFAKGMGLGATYFVVFCCYALLLWYGGYLVRH
HYTNGGLAISTMFAVMLGGLALGQSAPSMGAFAKAKVAATKIFRMIDHKPAVDRNSESGLELDAVTGLVELK
NVDFSYPSRPEVRILNNFTLNVPAGKTIALVGSSGSGKSTVVSLIERFYDPNSGQVLVDGHDIKTLKLRWLRQQI
GLVSQEPALFATSIKENILLGRPDADQIEIEEAARVANAHSFIVKLPEGFDTQVGERGLQLSGGQKQRIAIARAM
LKNPAILLLDEATSALDSESEKLVQEALDRFMIGRTTLVIAHRLSTIRKADLVAVLQQGSVTEIGTHDELIAKGE
NGVYAKLIRMQEAAHETAMNNARKSSARPSSARNSVSSPIIARNSSYGRSPYSRRLSDFSTSDFSLSLDAAYPN
YRLEKLPFKEQASSFWRLAKMNSPEWVYALVGSIGSVVCGSLSAFFAYVLSAVLSVYYNPNHAYMSREIGKY
CYLLIGLSSAALIFNTLQHFFWDIVGENLTKRVREKMLAAVLKNEMAWFDQEENESARIAARLALDANNVRSA
IGDRISVIVQNTALMLVACTAGFVLQWRLALVLIAVFPLVVAATVLQKMFMTGFSGDLEAAHAKATQLAGEAI
ANVRTVAAFNSESQIVGLFTTNLQIPLRRCFWKGQIAGSGFGVAQFLLYASYALGLWYASWLVKHGISDFSKTI
RVFMVLMVSANGAAETLTLAPDFIKGGRAMRSVFELLDRKTEIEPDEPDATAAPDRLRGEVELKHIDFSYPTRP
DVPIFRDLNLRARAGKTLALVGPSGCGKSSVIALVQRFYEPSSGRVIIDGKDIRKYNLKSLRKHIAMVPQEPCLF
AATIYENIAYGHESATEAEIIEAATLANAHKFISGLPDGYKTFVGERGVQLSGGQKQRIAIARALVRKAELMLL
DEATSALDAESERSVQEALDRACSGKTTIVVAHRLSTIRNAHVIAVIDDGKVAEQGSHSHLLKNYPDGCYARM
IQLQRFTHNQVIGMTSGSSSRSKDNGEREA
>XP_003625677.2_ABCB1_Mt
MSKESEEIKTTEQWKWSEMQGLELVSDDNNSSSSDPFKGNLPTVTEEGVTATVSDSQNQVQVSKEMEEHKKD
GGSKEKVKSAPAVGFGELFRFADGLDYILMTIGTVGAIVHGCSLPLFLRFFADLVNSFGSNANNLDKMTQEVV
KYAFYFLVVGAAIWASSWAEISCWMWTGERQSTKMRIKYLEAALKQDIEFFDTEVRTSDVVFAINTDAVMVQ
DAISEKLGNFIHYMATFVSGFVVGFTAVWQLALVTLAVVPMIAVIGGIHTTTLAKLSSKSQEALSQAGNIVEQT
VVQIRVVLAFVGESRALQGYSSALKVAQKLGYKTGLAKGMGLGATYFVVFCCYALLLWYGGYLVRHHETN
GGLAIATMFAVMIGGIGLGQSAPSMAAFTKARVAAAKIFRIIDHQPGIDRNSESGLELETVTGLVELKNVDFSYP
SRPEVLILNDFSLSVPAGKTIALVGSSGSGKSTVVSLIERFYDPTSGQVMLDGHDIKTLKLKWLRQQIGLVSQEP
ALFATTIRENILLGRPDANQVEIEEAARVANAHSFIIKLPEGFETQVGERGLQLSGGQKQRIAIARAMLKNPAILL
LDEATSALDSESEKLVQEALDRFMIGRTTLVIAHRLSTIRKADLVAVIQQGSVFEIGTHDELFSKGENGVYAKLI
KMQEVAHETAMNNARKSSARPSSARNSVSSPIIARNSSYGRSPYSRRLSDFSTSDFSLSLDASHPNYKHEKLAF
KDQAGSFWRLVKMNSPEWLYALLGSIGSIVCGSLSAFFAYVLSAVLSVYYNPDHKHMIREIDKYCYLLIGLSST
ALIFNTLQHFFWDIVGENLTKRVREKMLTAVLKNEMAWFDQEENESARISARLALDANNVRSAIGDRISVIVQ
NTALMLVACTAGFVLQWRLALVLIAVFPVVVAATVLQKMFMTGFSGDLEAAHAKATQLAGEAIANVRTVAA
FNSESKIVRLFASNLETPLQRCFWKGQISGSGYGIAQFALYASYALGLWYASWLVKHGISDFSKTIRVFMVLMV
SANGAAETLTLAPDFIKGGRAMRSVFDLLDRQTEIEPDDQDATPVPDRLRGEVELKHVDFSYPTRPDMPVFRD
LNLRIRAGKTLALVGPSGCGKSSVIALIQRFYDPTSGRIMIDGKDIRKYNLKSLRRHISVVPQEPCLFATTIYENIA
YGHDSATEAEIIEAATLANAHKFISSLPDGYKTFVGERGVQLSGGQKQRIAVARAFLRKAELMLLDEATSALD
AESERSVQEALDRASTGKTTIIVAHRLSTIRNANVIAVIDDGKVAEQGSHSQLMKNHQDGIYARMIQLQRFTHN
EVIGMASGSSSST
>XP_003520656.1_ABCB1_Gm
MSKDSEEIKTIEQWKWTEMQGLELVPEEGAAAAPSQHHQLPMEMNTSEPPNKDVVGASSSSAAVTNGEKKEK

EKESVPSVGFGELFRFADGLDYVLMGIGTVGAVVHGCSLPLFLRFFADLVNSFGSNANDVDKMTQEVVKYAF
YFLVVGAAIWASSWAEISCWMWSGERQSTTMRIKYLEAALNQDIQFFDTEVRTSDVVFAINTDAVMVQDAISE
KLGNFIHYMATFVSGFVVGFTAVWQLALVTLAVVPMIAVIGGIHTATLAKLSGKSQEALSQAGNIVEQTVAQI
RVVLAFVGESRALQSYSSALRIAQKIGYKTGFAKGMGLGATYFVVFCCYALLLWYGGYLVRHHATNGGLAIA
TMFAVMIGGLGLGQSAPSMAAFTKARVAAAKIFRIIDHKPNIDRNSESGIELDTVTGLVELKNVDFSYPSRPEV
QILNDFSLNVPAGKTIALVGSSGSGKSTVVSLIERFYDPTSGQVLLDGHDIKTLKLRWLRQQIGLVSQEPALFAT
TIRENILLGRPDADQVEIEEAARVANAHSFIIKLPDGYETQVGERGLQLSGGQKQRIAIARAMLKNPAILLLDEA
TSALDSESEKLVQEALDRFMIGRTTLVIAHRLSTIRKADLVAVLQLGSVSEIGTHDELFSKGENGVYAKLIKMQ
EMAHETAVNNARKSSARPSSARNSVSSPIIARNSSYGRSPYSRRLSDFSTSDFSLSLDASHPSYRLEKLAFKEQAS
SFWRLAKMNSPEWLYALIGSIGSVVCGSLSAFFAYVLSAVLSVYYNPDHRYMIREIEKYCYLLIGLSSTALLFNT
LQHFFWDIVGENLTKRVREKMLMAVLKNEMAWFDQEENESARIAARLALDANNVRSAIGDRISVIVQNTALM
LVACTAGFVLQWRLALVLVAVFPVVVAATVLQKMFMTGFSGDLEAAHAKATQLAGEAIANVRTVAAFNSET
KIVGLFTTNLQAPLQRCFWKGQISGSGYGVAQFALYASYALGLWYASWLVKHGISDFSKTIRVFMVLMVSAN
GAAETLTLAPDFIKGGQAMRSVFELLDRRTEIEPDDQDATLVPDRLRGEVELKHVDFSYPTRPDMPVFRDLSLR
ARAGKTLALVGPSGCGKSSIIALIQRFYDPTSGRVMIDGKDIRKYNLKSLRRHISVVPQEPCLFATTIYENIAYGH
ESATEAEIIEAATLANAHKFISGLPDGYKTFVGERGVQLSGGQKQRIAVARAFLRKAELMLLDEATSALDAESE
RSVQEALDRASSGKTTIIVAHRLSTVRNANLIAVIDDGKVAEQGSHSQLLKNHPDGIYARMIQLQRFTHSQVIG
MASGSSSSTRPKDDEREG
>XP_003535149.1_ABCB1_Gm
MSQNSEEIKTLEQWRWSEMQGIELVSSSATVSNSHESNPALEKKREERVIMEEVSSVAKKEEGVPNGVGGEKK
KDGSVASVGFGELFRFSDGLDYILMAIGTVGAFVHGCSLPLFLRFFADLVNSFGSNANDLDKMTQEVVKYAFY
FLVVGAAIWASSWAEISCWMWTGERQSTRMRIRYLEAALDQDIQFFDTEVRTSDVVFAINTDAVMVQDAISE
KLGNFIHYMATFVSGFVVGFTAVWQLALVTLAVVPIIAVIGGIHTTTLAKLSSKSQEALSQAGNIVEQTVVQIR
VVLAFVGETRALQGYSSALRIAQKIGYRTGFAKGMGLGATYFVVFCCYALLLWYGGYLVRHHYTNGGLAIAT
MFSVMIGGLALGQSAPSMAAFTKARVAAAKIFRVIDHKPVIDRRSESGLELESVTGLVELRNVDFSYPSRPEVLI
LNNFSLNVPAGKTIALVGSSGSGKSTVVSLIERFYDPSSGQVLLDGNDVKSFKLRWLRQQIGLVSQEPALFATTI
RENILLGRPDANQVEIEEAARVANAHSFIIKLPEGYETQVGERGLQLSGGQKQRIAIARAMLKNPAILLLDEATS
ALDSESEKLVQEALDRFMIGRTTLVIAHRLSTIRKADLVAVLQQGSVTEIGTHDELFAKGENGVYAKLIRMQE
MAHETSMNNARKSSARPSSARNSVSSPIITRNSSYGRSPYSRRLSDFSTSDFSLSLDASHPNYRLEKLAFKDQAS
SFWRLAKMNSPEWLYALIGSIGSVVCGSLSAFFAYVLSAVLSVYYNPNHRHMIREIEKYCYLLIGLSSAALLFN
TLQHSFWDIVGENLTKRVREKMLAAVLKNEMAWFDQEENESARIAARLSLDANNVRSAIGDRISVIVQNTAL
MLVACTAGFVLQWRLALVLVAVFPVVVAATVLQKMFMTGFSGDLEAAHAKATQLAGEAIANVRTVAAFNS
EKKIVGLFTSNLETPLRRCFWKGQISGSGYGIAQFALYASYALGLWYASWLVKHGISDFSNTIRVFMVLMVSA
NGAAETLTLAPDFIKGGRAMRSVFDLLDRITEIEPDDPDATPVPDRLRGEVELKHVDFSYPTRPDMSVFRDLSL
RARAGKTLALVGPSGCGKSSVIALIQRFYDPTSGRVMIDGKDIRKYNLKSLRRHIAVVPQEPCLFATSIYENIAY
GHDSASEAEIIEAATLANAHKFISSLPDGYKTFVGERGVQLSGGQKQRIAIARAFVRKAELMLLDEATSALDAE
SERSVQEALDRACSGKTTIIVAHRLSTIRNANLIAVIDDGKVAEQGSHSLLLKNYPDGIYARMIQLQRFTNNQVI
GMASGSSSSARPKDDEREG
>NP_001275319.1_MDR_St
MQGVELVVSEDKNSNTPTTTTTTNSHQFQETRMEVKKEEGGDVEKPSSPPPAVGFGELFRFADGLDCVLMIIGS
LGAFVHGCSLPLFLRFFADLVNSFGSYANDVDKMTQEVLKYAFYFLVVGAAIWASSWAEISCWMWTGERQT
TKMRIKYLEAALNQDIQYFDTEVRTSDVVSAINTDAVVVQDAISEKLGNFIHYMATFLSGFVVGFTAVWQLAL
VTLAVVPLIAVIGAIYTVTSAKLSSQSQEALSKAGNIVEQTVVQIRTVLVFVGEAKALQAYTAALRVSQKIGYK
SGFSKGLGLGATYFTVFCCYALLLWYGGYLVRHHFTNGGLAIATMFAVMIGGLALGQSAPSMTAFAKARVA
AAKIFRIIDHKPSVDRNAKTGLELDTVSGQLELKNVEFSYPSRPEIKILNNFNLVVPAGKTIALVGSSGSGKSTVV
SLIERFYDPTSGQLMLDGNDIKTLKLKWLRQQIGLVSQEPALFATSIKENILLGRPDATQIEIEEAARVANAHSFV
IKLPDGFDTQVGERGLQLSGGQKQRIAIARAMLKNPAILLLDEATSALDSESEKLVQEALDRFMIGRTTLVIAHR
LSTIRKADLVAVLQQGSVSEIGSHDELMSKGENGMYAKLIKMQEAAHETALSNARKSSARPSSARNSVSSPIIT
RNSSYGRSPYSRRLSDFSTSDFSLSLDAAYSNYRNEKLAFKDQASSFGRLAKMNSPEWTYALIGSIGSVICGSLS
AFFAYVLSAVLSVYYNPDHAYMSEQIAKYCYLLIGVSSAALIFNTLQHYYWDVVGENLTKRVREKMLAAVLK
MEMAWFDQEENDSSRIAARLSLDANNVRSAIGDRISVIMQNSALMLVACTAGFVLQWRLALVLIGVFPVVVA
ATVLQKMFMKGFSGDLEAAHAKATQLAGEAVANVRTVAAFNSETKIVNLFDSSLQTPLRRCFWKGQIAGSGY
GIAQFLLYSSYALGLWYASWLVKHGISDFSKTIRVFMVLMVSANGAAETLTLAPDFIKGGRAMRSVFELLDRK
TEVEPDDPDATAVPDRLRGEVEFKHVDFSYPTRPDVSIFRDLNLRARAGKTLALVGPSGCGKSSVISLIERFYEP

SSGRVIIDGKDIRKYNLKSLRRHIAVVPQEPCLFATTIYENIAYGHESATEAEITEAATLANAHKFISALPDGYKT
FVGERGVQLSGGQKQRIAIARAFLRKAELMLLDEATSALDAESERCVQEALDRACAGKTTIVVAHRLSTIRNA
HVIAVIDDGKVAEQGSHSHLLKNYSDGIYARMIQLQRFTHGEAVNMATGSTSSSRPKEDQD
>XP_004246624.1_ABCB1_Sl
MSQDSEEIKTIEHWKWSEMQGVELVVSEDKNSNTPTTTTTTTNSHQFQETRMEVKKEEGGDVEKPTSPPPAVG
FGELFRFADGLDYALMIIGSLGAFVHGCSLPLFLRFFADLVNSFGSYANDVDKMTQEVLKYAFYFLVVGAAIW
ASSWAEISCWMWTGERQTTKMRIKYLEAALNQDIQYFDTEVRTSDVVSAINTDAVVVQDAISEKLGNFIHYM
ATFLSGFVVGFTAVWQLALVTLAVVPLIAVIGAIYTMTSAKLSSQSQEALSKAGNTVEQTVVQIRTVLAFVGEA
KAMQAYTAALRVSQKIGYKSGFSKGFGLGATYFTVFCCYALLLWYGGYLVRHHFTNGGLAIATMFAVMIGG
LALGQSAPSMTAFAKARVAAAKIFRIIDHKPSVDRNAKTGLELDTVSGQLELKNVEFSYPSRPEIKILNNFNLVV
PAGKTIALVGSSGSGKSTVVSLIERFYDPTSGQLMLDGNDIKTLKLKWLRQQIGLVSQEPALFATSIKENILLGR
PDATQIEIEEAARVANAHSFIIKLPDGFDTQVGERGLQLSGGQKQRIAIARAMLKNPAILLLDEATSALDSESEK
LVQEALDRFMIGRTTLVIAHRLSTIRKADLVAVLQQGNVSEIGSHDELMSKGENGMYAKLIKMQEAAHETALS
NARKSSARPSSARNSVSSPIITRNSSYGRSPYSRRLSDFSTSDFSLSLDAAYSNYRNEKLAFKDQASSFGRLAKM
NSPEWTYALIGSIGSIICGSLSAFFAYVLSAVLSVYYNPDHAYMSKQIAKYCYLLIGVSSAALIFNTLQHYYWDV
VGENLTKRVREKMLAAVLKMEMAWFDQEENDSSRIAARLSLDANNVRSAIGDRISVIMQNSALMLVACTAGF
VLQWRLALVLIGVFPVVVAATVLQKMFMKGFSGDLEAAHAKATQLAGEAVANVRTVAAFNSETKIVNLFDA
SLQTPLRRCFWKGQIAGSGYGIAQFLLYASYALGLWYASWLVKHGISDFSKTIRVFMVLMVSANGAAETLTLA
PDFIKGGRAMRSVFELLDRKTEVEPDDPDATAAPDRLRGEVEFKHVDFSYPTRPDVSIFRDLNLRARAGKTLAL
VGPSGCGKSSVIALIERFYEPSSGRVIIDGKDIRKYNLKSLRRHIAVVPQEPCLFATTIYENIAYGHESATEAEITE
AATLANAHKFISALPDGYKTFVGERGVQLSGGQKQRIAIARAFLRKAELMLLDEATSALDAESERCVQEALDR
ACAGKTTIIVAHRLSTIRNAHVIAVIDDGKVAEQGSHSHLLKNYSDGIYARMIQLQRFTHGEAVNMATGSTSSS
RPKEDLD
>XP_006381407.1_ABCB1_Pt
MSQESLEIKTIEQWKWSEMQGLELVSEPPPDPSSHSHPFKTTPTRTLNTNSTYQQQESVVERREMESTEPKKDG
TSSNSGGGGNGEKPGEVAVAGFGELFRFADGLDYVLMGIGSMGAFVHGCSLPLFLRFFADLVNSFGSNANNM
DKMMQEVLKYAFYFLIVGAAIWASSWAEISCWMWTGERQSTRMRIKYLEAALNQDIQYFDTEVRTSDVVFAI
NTDAVMVQDAISEKLGNFIHYMATFVSGFVVGFTAVWQLALVTLAVVPLIAVIGAIHTTTLAKLSGKSQEALS
QAGNIVEQTIVQIRVVLAFVGESRALQAYSSALKISQRIGYKSGFSKGMGLGATYFVVFCCYALLLWYGGYLV
RHHYTNGGLAIATMFAVMIGGLGIGQAIPSMGAFAKAKVAAAKIFRIIDHKPAIDRNSESGLELESVTGLVALK
NIDFAYPSRPDARILNNFSLNVPAGKTIALVGSSGSGKSTVVSLIERFYDPNSGQVLLDGHDIKTLKLRWLRQQI
GLVSQEPALFATTIKENILLGRPDADQVEIEEAARVANAHSFIIKLPDGFDTQVGERGLQLSGGQKQRVAIARA
MLKNPAILLLDEATSALDSESEKLVQEALDRFMIGRTTLVIAHRLSTIRKADLVAVLQQGSVSEVGTHDELIAK
GENGVYAKLIRMQEAAHETALNNARKSSARPSSARNSVSSPIIARNSSYGRSPYSRRLSDFSTSDFSLSLDAPFP
NYRLEKLAFKEQASSFWRLAKMNSPEWVYALVGSIGSVICGSLSAFFAYVLSAVLSVYYNPNHDYMSREIAKY
CYLLIGLSSAALIFNTLQHSFWDIVGENLTKRVREKMLTAVLKNEMAWFDQEENESARIAARLALDANNVRSA
IGDRISVIVQNTALMLVACTAGFVLQWRLALVLIAVFPLVVAATVLQKMFMNGFSGDLEAAHSKATQLAGEAI
ANMRTVAAFNSEAKIVGLFSTNLETPLRRCFWKGQIAGSGFGIAQFSLYASYALGLWYASWLVKHGISNFSNTI
RVFMVLMVSANGAAETLTLAPDFIKGGRAMRSVFDLLDRKTEIEPDDPDATPVPDRLRGEVELKHVDFSYPTR
PDIPVFRDLNLRARAGKILALVGPSGCGKSSVIALIQRFYEPSSGRVMIDGKDIRKYNLKSLRKHIAIVPQEPCLF
GTTIYENIAYGNESATEAEIIEAATLANAHKFVSALPDGYKTFVGERGVQLSGGQKQRIAIARALIRKAGLMLL
DEATSALDAESERSVQEALDRACSGKTTIVVAHRLSTIRNAHVIAVIDDGKVAEQGSHSHLLKNYPDGSYARMI
QLQRFTHSEVIGMTSGSSSSTRPKDDDEREG

>XP_013468150.1_ABCB1_Mt
MSQDSDETIKTSEELKWSEMQGEHSLSKEQMEEEASLPKDKSCDVSNGNEKVKNVASVGFCELFRFADGLDY
VLMIIGSLGAVVHGCSLPLFLRFFADLVNSFGSNANNLDKMTQEVAKYALYFLIVGAAIWASSWAEISCWMW
TGERQSTKMRIKYLEAALDQDIQFFDTEVRTSDVVFAINSDAVMVQDAISEKLGNFVHYMATFVSGFAVGFTA
VWQLALVTLAVVPMIAVIGGIHTITLGKLSGKSQEALSQAGNIVEQTIVQIRVVLAFVGETKALQGYSSALRVA
QKIGYRTGFAKGIGLGATYFVVFCCYALLLWYGGYLVRHDYTNGGLAISTMFAVMIGGLALGQSAPSMIAFT
KARVAAAKIFGVIDHKPCIDKKSESGLELETVTGLVELKNVDFSYPSRPEVQILHDFSLSVPSGKTIALVGSSGSG
KSTIVSLIERFYDPSSGQVLLDGHDVKTLKLKWLRQQIGLVSQEPALFATTIRENILLGRPDADQVEIEEAARVA
NAHSFIIKLPDGYETQVGERGLQLSGGQKQRIAIARAMLKNPAILLLDEATSALDSESEKLVQEALDRFMIGRTT
LVIAHRLSTIRKADLVAVLQQGSVSEIGTHDELFAKGENGVYAKLIKMQEMANESSMSNARKSSARPSSARNS

VSSPIITRNSSYGGRSPYSRRLSDFSTSDFSLSFDASHPNFRLEKLAFKDQASSFWRLAKMNSPEWLYALIGSIGSI
VCGSLSAFFAYVLSAVLSVYYNPSHKHMIREIEKYCYLLIGLSSAALVFNTLQHFFWDTVGENLTKRVREKML
SAVLKNEMAWFDREENESARIAARLALDANNVRSAIGDRISVIVQNTALLLVACTAGFVLQWRLALVLVAVFP
VVVAATVLQKMFMTGFSGDLEAAHAKATQLAGEAIANVRTVAAFNSEKKIVGLFASNLETPLRRCFWKGQIS
GSGYGIAQFALYASYALGLWYASWLVKHGISDFSKTIRVFMVLMVSANGAAETLTLAPDFIKGGRAMKSVFD
LLDRRTEIEPDDPDAVIVPDRLRGEVELKHVDFSYPSRPDMPVFRDLSLRARAGKTLALVGPSGCGKSSVIALIQ
RFYDPTSGRVTIDGKDIRKYNLKSLRRHIAVVPQEPCLFATTIYENIAYGHESATEAEIIEAATLSNAHKFISSLPD
GYKTFVGERGVQLSGGQKQRIAIARAFVRKAELMLLDEATSALDAESERSVQEALERACSGKTTIIVAHRLSTI
RNANLIAVIDDGTVEEQGSHSHLLKNHPDGIYARMIQLQRLTNSQVVGTASSSSSSARTKVDEREG

>SlABCB4
maettegksmpeaekkkeqslpfyqlfsfadkydyllmtcgsigailhgssmpvffllfg
emvngfgknqmdlhkmthevskyalyfvylglivcassyaeigcwmytgerqvsalrkky
leavlkqdvgffdtdartgdivfsvstdtllvqdaisekvgnfihylstflaglvvgfvs
awrlallsvavipgiafagglyaytltgltsksresyanagiiaeqaiaqvrtvysyvge
tkalnsysdaiqntlklgykagmakglglgctygiacmswalvfwyagvfirngqsdggk
aftaifsaivggmslgqsfsnlgafskgkaagyklmeiikqkptivqdtldgkclsevsg
niefknvtfsypsrpdviifrdfciffpagktvavvggsgsgkstvvslierfydpndgq
vlldnvdiktlqlrwlrdqiglvnqepalfattilenilygkpdatmaeveaatcasnah
nfitllpngyntqvgergvqlsggqkqriaiaramlknpkillldeatsaldagsesivq
ealdrlmvgrttvvvahrlstirnvdsiaviqqgqvvetgtheeliskagayaslirfqe
mvgnrdfsnpstrrtrstrlshslstkslslrsgslrnlsysystgadgriemisnaetd
rknpapqnyfcrllklnapewpysimgavgsvlsgfigptfaivmsnmievfyytnpatm
erktkeyvfiyigaglyavvayliqhyffsimgenlttrvrrmmlaailrnevgwfdeee
nnssllaarlatdaadvksaiaerisvilqnmtslltsfivafivewrvsllilatfpll
vlanfaqqlslkgfagdtakahaktsmiagegvsnirtvaafnaqekiislfsqelrvpq
mqslrrsqmsgllfgisqlalygsealilwygahlvnngvstfskvikvfvvlvitansv
aetvslapeiirggeavgsvfsildrstrvdpddpegdpvesirgdielrhvdfaypsrp
dvsvfkdlnlriragqsqalvgasgsgkssvialierfydptggkvmidgkdirrlnlks
lrlkiglvqqepalfaasifeniaygkegateaevieaaraanvhtfvsglpegyktpvg
ergvqlsggqkqriaiaravlkdpsillldeatsaldaesecvlqealerlmrgrttvlv
ahrlstirnvdtigvvqdgriveqgshselisrpegaysrllqlqhhri

>AtABCB9
MEEKSSKKNDGGNQKVSFFKLFSFADKTDVVLMTVGTIAAAGNGLTQPFMTLIFGQLINA
FGTTDPDHMVREVWKVAVKFIYLAVYSCVVAFLQVSCWMVTGERQSATIRGLYLKTILRQ
DIGYFDTETNTGEVIGRMSGDTILIQDAMGEKVGKFTQLLCTFLGGFAIAFYKGPLLAGV
LCSCIPLIVIAGAAMSLIMSKMAGRGQVAYAEAGNVVEQTVGAIRTVVAFTGEKQATEKY
ESKLEIAYKTVVQQGLISGFGLGTMLAVIFCSYGLAVWYGAKLIMEKGYNGGQVINVIFA
VLTGGMSLGQTSPSLNAFAAGRAAAFKMFETIKRSPKIDAYDMSGSVLEDIRGDIELKDV
YFRYPARPDVQIFAGFSLFVPNGKTVALVGQSGSGKSTVISLIERFYDPESGQVLIDNID
LKKLQLKWIRSKIGLVSQEPVLFATTIKENIAYGKEDATDQEIRTAIELANAAKFIDKLP
QGLDTMVGEHGTQMSGGQKQRLAIARAILKNPKILLLDEATSALDAESERIVQDALVNLM
SNRTTVVVAHRLTTIRTADVIAVVHQGKIVEKGTHDEMIQDPEGAYSQLVRLQEGSKEEA
TESERPETSLDVERSGSLRLSSAMRRSVSRNSSSSRHSFSLASNMFFPGVNVNQTDEMED
EENNVRHKKVSLKRLAHLNKPEIPVLVLGSIAAMVHGTVFPIFGLLLSSSINMFYEPAKI
LKKDSHFWALIYIALGLTNFVMIPVQNYFFGIAGGKLIKRIRSMCFDKVVHQEISWFDDT
ANSRSLVGDALALIVQNIATVTTGLIIAFTANWILALIVLALSPFIVIQGYAQTKFLTGF
SADAKAMYEEASQVANDAVSSIRTVASFCAEEKVMDLYQQKCDGPKKNGVRLGLLSGAGF
GFSFFFLYCINCVCFVSGAGLIQIGKATFGEVFKVFFALTIMAIGVSQTSAMAPDSNKAK
DSAASIFDILDSTPKIDSSSDEGTTLQNVNGDIEFRHVSFRYPMRPDVQIFRDLCLTIPS
GKTVALVGESGSGKSTVISMIERFYNPDSGKILIDQVEIQTFKLSWLRQQMGLVSQEPIL
FNETIRSNIAYGKTGGATEEEIIAAAKAANAHNFISSLPQGYDTSVGERGVQLSGGQKQR

IAIARAILKDPKILLLDEATSALDAESERVVQDALDRVMVNRTTVVVAHRLTTIKNADVI
AVVKNGVIAEKGRHETLMKISGGAYASLVTLHMSAN

>AtABCB14
MDNIEPPFSGNIHAETEVKKEEKKKMKKESVSLMGLFSAADNVDYFLMFLGGLGTCIHGG
TLPLFFVFFGGMLDSLGKLSTDPNAISSRVSQNALYLVYLGLVNLVSAWIGVACWMQTGE
RQTARLRINYLKSILAKDITFFDTEARDSNFIFHISSDAILVQDAIGDKTGHVLRYLCQF
IAGFVIGFLSVWQLTLLTLGVVPLIAIAGGGYAIVMSTISEKSEAAYADAGKVAEEVMSQ
VRTVYAFVGEEKAVKSYSNSLKKALKLSKRSGLAKGLGVGLTYSLLFCAWALLFWYASLL
VRHGKTNGAKAFTTILNVIYSGFALGQAVPSLSAISKGRVAAANIFKMIGNNNLESSERL
ENGTTLQNVVGKIEFCGVSFAYPSRPNMVFENLSFTIHSGKTFAFVGPSGSGKSTIISMV
QRFYEPRSGEILLDGNDIKNLKLKWLREQMGLVSQEPALFATTIASNILLGKEKANMDQI
IEAAKAANADSFIKSLPNGYNTQVGEGGTQLSGGQKQRIAIARAVLRNPKILLLDEATSA
LDAESEKIVQQALDNVMEKRTTIVIAHRLSTIRNVDKIVVLRDGQVRETGSHSELISRGG
DYATLVNCQDTEPQENLRSVMYESCRSQAGSYSSRRVFSSRRTSSFREDQEKTEKDSKGE
DLISSSSMIWELIKLNAPEWLYALLGSIGAVLAGSQPALFSMGLAYVLTTFYSPFPSLIK
REVDKVAIIFVGAGIVTAPIYILQHYFYTLMGERLTSRVRLSLFSAILSNEIGWFDLDEN
NTGSLTSILAADATLVRSAIADRLSTIVQNLSLTITALALAFFYSWRVAAVVTACFPLLI
AASLTEQLFLKGFGGDYTRAYSRATSLAREAISNIRTVAAFSAEKQISEQFTCELSKPTK
SALLRGHISGFGYGLSQCLAFCSYALGLWYISVLIKRNETNFEDSIKSFMVLLVTAYSVA
ETLALTPDIVKGTQALGSVFRVLHRETEIPPDQPNSRLVTHIKGDIEFRNVSFAYPTRPE
IAIFKNLNLRVSAGKSLAVVGPSGSGKSTVIGLIMRFYDPSNGNLCIDGHDIKSVNLRSL
RKKLALVQQEPALFSTSIHENIKYGNENASEAEIIEAAKAANAHEFISRMEEGYMTHVGD
KGVQLSGGQKQRVAIARAVLKDPSVLLLDEATSALDTSAEKQVQEALDKLMKGRTTILVA
HRLSTIRKADTIVVLHKGKVVEKGSHRELVSKSDGFYKKLTSLQEAV

>AtABCB15
MGKEEEKESGRNKMNCFGSVRSIFMHADGVDWLLMGLGLIGAVGDGFTTPLVLLITSKLM
NNIGGSSFNTDTFMQSISKNSVALLYVACGSWVVCFLEGYCWTRTGERQTARMREKYLRA
VLRQDVGYFDLHVTSTSDVITSVSSDSFVIQDVLSEKLPNFLMSASTFVGSYIVGFILLW
RLAIVGLPFIVLLVIPGLMYGRALISISRKIREEYNEAGFVAEQAISSVRTVYAFSGERK
TISKFSTALQGSVKLGIKQGLAKGITIGSNGITFAMWGFMSWYGSRMVMYHGAQGGTVFA
VAAAIAIGGVSLGGGLSNLKYFFEAASVGERIMEVINRVPKIDSDNPDGHKLEKIRGEVE
FKNVKFVYPSRLETSIFDDFCLRVPSGKTVALVGGSGSGKSTVISLLQRFYDPLAGEILI
DGVSIDKLQVKWLRSQMGLVSQEPALFATTIKENILFGKEDASMDDVVEAAKASNAHNFI
SQLPNGYETQVGERGVQMSGGQKQRIAIARAIIKSPTILLLDEATSALDSESERVVQEAL
ENASIGRTTILIAHRLSTIRNADVISVVKNGHIVETGSHDELMENIDGQYSTLVHLQQIE
KQDINVSVKIGPISDPSKDIRNSSRVSTLSRSSSANSVTGPSTIKNLSEDNKPQLPSFKR
LLAMNLPEWKQALYGCISATLFGAIQPAYAYSLGSMVSVYFLTSHDEIKEKTRIYALSFV
GLAVLSFLINISQHYNFAYMGEYLTKRIRERMLSKVLTFEVGWFDRDENSSGAICSRLAK
DANVVRSLVGDRMALVVQTVSAVTIAFTMGLVIAWRLALVMIAVQPVIIVCFYTRRVLLK
SMSKKAIKAQDESSKLAAEAVSNVRTITAFSSQERIMKMLEKAQESPRRESIRQSWFAGF
GLAMSQSLTSCTWALDFWYGGRLIQDGYITAKALFETFMILVSTGRVIADAGSMTTDLAK
GSDAVGSVFAVLDRYTSIDPEDPDGYETERITGQVEFLDVDFSYPTRPDVIIFKNFSIKI
EEGKSTAIVGPSGSGKSTIIGLIERFYDPLKGIVKIDGRDIRSYHLRSLRRHIALVSQEP
TLFAGTIRENIIYGGVSDKIDEAEIIEAAKAANAHDFITSLTEGYDTYCGDRGVQLSGGQ
KQRIAIARAVLKNPSVLLLDEATSALDSQSERVVQDALERVMVGRTSVVIAHRLSTIQNC
DAIAVLDKGKLVERGTHSSLLSKGPTGIYFSLVSLQTTSG

>AtABCB24
MMRVSQLQLCRTSLSYRLRSGYHHHHHLHHSFFKLIKRNSILESPPTNASHQSPSPITPMVNARVMFFST
STSAPHPEKINRTSSENILRMISSYLWMKDNPKLCFRVISAFACLVGAKFLNVQVPFLFKVAIDWLSSSS
FVDSNPYLVAAFATPSSVLIGYGIARSGSSAFNELRTSVFSKVALRTIRTISRKVLSRLHDLDLRYHLNR
DTGALNRIIDRGSRAINTILSAMVFNIMPTILEISMVSCILAYKFGAVYALITCLSVGSYIAFTLAMTQW

RIKIRKAMNEAENDASTRAIDSLINYETVKYFNNEDYEARKYDQLHENYEDAALQSRKSFALLNFGQSFI
FSTALSTAMVLCSQGIMNGQMTVGDLVMVNGLLFQLSLPLYFLGVVYSDTVQGLVDMKSMFKFLEERSDI
GDKDIDRKLPPLVLKGGSISFENVHFSYLPERKILDGISFEVPAGKSVAIVGSSGSGKSTILRMIFRFFD
VDSGNVKIDGQDIKEVRLESLRSSIGVVPQDTVLFNDTIFHNIHYGNLSATEEEVYNAARRAAIHDTIMK
FPDKYSTAVGERGLMLSGGEKQRVALARAFLKSPAILLCDEATSALDSKTEAEIMKTLRSLASNRTCIFI
AHRLTTAMQCDEILVMEKGKVVEKGTHEVLLGKSGRYAKLWTQQNSKLEV

>OsABCB19
MADESGSCGGGGGGGGGCEAVKKRVDQSVAFHELFGFADPLDWLLMAAGSAGAVVHGAAM
PVFFLLFGELINGFGKNQHSLRRMTDEVSKYSLYFVYLGLVVCASSYLEIACWMYTGERQ
VGALRRRYLEAVLRQDVGFFDTDARTGDVVFSVSTDTLLVQDAIGEKVGNFIHYLSTFLA
GLVVGFVSAWRLALLSIAVIPGIAFAGGLYAYTLTGLTSKSRDSYANAGIIAEQAIAQVR
TVYSYVGESKALNSYSEAIQNTLKLGYKAGMAKGLGIGCTYGIACMSWALVFWYAGVFIR
NGQTDGGKAFTAIFSAIVGGLSLGQSFSNLGAFSKGKIAGYKLLEVIRQRPTIVQDPADG
RCLDEVHGNIEFKEVAFSYPSRPDVMIFRDFSLFFPAGKTAAVVGGSGSGKSTVVALIER
FYDPNQGQVLLDNVDIKTLQLKWLRDQIGLVNQEPALFATTILENILYGKPDATMAEVEA
AATSANAHSFIALLPNGYNTQVGERGLQLSGGQKQRIAIARAMLKNPKILLLDEATSALD
AGSENIVQEALDRLMVGRTTVVVAHRLSTIRCVDMIAVIQQGQVVETGTHDELLAKGSSG
AYAALIRFQEMARNRDFRGPSTRKSRSSRLSNSLSTRSLSLRSGSLRNLSYSYSTGADGR
IEMVSNADNDRKYPAPKGYFFKLLKLNAPEWPYTILGAIGSILSGFIGPTFAIVMSNMIE
VFYFRDPNAMERKTREYVFIYIGTGLYAVVAYLVQHYFFSIMGENLTTRVRRMMLAAILR
NDVGWFDQEENNSSLVAARLSTDAADVKSAIAERISVILQNMTSLLVSFVVGFIIEWRVA
VLILVTFPLLVLANFAQQLSMKGFAGDTAKAHAKTSMIAGEGVSNIRTVAAFNAQDKVLS
LFCTELRVPQMHSLRRSQISGALFGLSQLSLYASEALILWYGAHLVRHHVSTFSKVIKVF
VVLVITANTVAETVSLAPEIVRGGESIRSVFAILNYRTRIDPDEPETEPVESVRGDIDFR
HVDFAYPSRPDVMVFKDFSLRIRAGQSQALVGASGSGKSTVIALIERFYDPLAGKVMIDG
KDIRRLNVRSLRLKIGLVQQEPVLFATSIFENIAYGKDGATEEEVIEAAKVANMHGFVSA
LPEGYKTPVGERGVQLSGGQKQRIAIARAVLKDPAVLLLDEATSALDAESECVLQEALER
IMKGRTAVLVAHRLSTIRGVDSIAVVQDGRVVEQGSHGELVSRPDGAYSRLLQLQLHHG

>ZmABCB1
MSSSDPEEIRARVVVLGSPHADGGDEWARPELEAFHLPSPAHQPPGFLAGQPEAAEQPTLPAPAGRSSSS
SNTPTTSAGGGAAPPPPSSPPPPPASLETEQPPNARPASAGANDSKKPTPPAALRDLFRFADGLDCALML
IGTLGALVHGCSLPVFLRFFADLVDSFGSHADDPDTMVRLVVKYAFYFLVVGAAIWASSWAEISCWMWTG
ERQSTRMRIRYLDAALRQDVSFFDTDVRASDVIYAINADAVVVQDAISEKLGNLIHYMATFVAGFVVGFT
AAWQLALVTLAVVPLIAVIGGLSAAALAKLSSRSQDALSGASGIAEQALAQIRIVQAFVGEEREMRAYSA
ALAVAQRIGYRSGFAKGLGLGGTYFTVFCCYGLLLWYGGHLVRAQHTNGGLAIATMFSVMIGGLALGQSA
PSMAAFAKARVAAAKIFRIIDHRPGISSRDGAEPESVTGRVEMRGVDFAYPSRPDVPILRGFSLSVPAGK
TIALVGSSGSGKSTVVSLIERFYDPSAGQILLDGHDLRSLELRWLRRQIGLVSQEPALFATSIRENLLLG
RDSQSATLAEMEEAARVANAHSFIIKLPDGYDTQVGERGLQLSGGQKQRIAIARAMLKNPAILLLDEATS
ALDSESEKLVQEALDRFMIGRTTLVIAHRLSTIRKADVVAVLQGGAVSEMGAHDELMAKGENGTYAKLIR
MQEQAHEAALVNARRSSARPSSARNSVSSPIMTRNSSYGRSPYSRRLSDFSTSDFTLSIHDPHHHHRTMA
DKQLAFRAGASSFLRLARMNSPEWAYALAGSIGSMVCGSFSAIFAYILSAVLSVYYAPDPRYMKREIAKY
CYLLIGMSSAALLFNTVQHVFWDTVGENLTKRVREKMFAAVLRNEIAWFDADENASARVAARLALDAQNV
RSAIGDRISVIVQNSALMLVACTAGFVLQWRLALVLLAVFPLVVGATVLQKMFMKGFSGDLEAAHARATQ
IAGEAVANLRTVAAFNAERKITGLFEANLRGPLRRCFWKGQIAGSGYGVAQFLLYASYALGLWYAAWLVK
HGVSDFSRTIRVFMVLMVSANGAAETLTLAPDFIKGGRAMRSVFETIDRKTEVEPDDVDAAPVPERPRGE
VELKHVDFSYPSRPDIQVFRDLSLRARAGKTLALVGPSGCGKSSVLALVQRFYEPTSGRVLLDGKDVRKY
NLRALRRVVAVVPQEPFLFAASIHENIAYGREGATEAEVVEAAAQANAHRFIAALPEGYRTQVGERGVQL
SGGQRQRIAIARALVKQAAIVLLDEATSALDAESERCVQEALERAGSGRTTIVVAHRLATVRGAHTIAVI
DDGKVAEQGSHSHLLKHHPDGCYARMLQLQRLTGAAAGPGPSTSCNGAA

>AtABCB4
MASESGLNGDPNILEEVSETKRDKEEEEEVKKTEKKDEEHEKTKTVPFYKLFAFADSFDFLLMILGTLGSIGNG

LGFPLMTLLFGDLIDAFGENQTNTTDKVSKVALKFVWLGIGTFAAAFLQLSGWMISGERQAARIRSLYLKTILR
QDIAFFDIDTNTGEVVGRMSGDTVLIQDAMGEKVGKAIQLLATFVGGFVIAFVRGWLLTLVMLSSIPLLVMAG
ALLAIVIAKTASRGQTAYAKAATVVEQTIGSIRTVASFTGEKQAISNYNKHLVTAYKAGVIEGGSTGLGLGTLF
LVVFCSYALAVWYGGKLILDKGYTGGQVLNIIIAVLTGSMSLGQTSPCLSAFAAGQAAAYKMFETIERRPNIDS
YSTNGKVLDDIKGDIELKDVYFTYPARPDEQIFRGFSLFISSGTTVALVGQSGSGKSTVVSLIERFYDPQAGDVLI
DGINLKEFQLKWIRSKIGLVSQEPVLFTASIKDNIAYGKEDATTEEIKAAAELANASKFVDKLPQGLDTMVGEH
GTQLSGGQKQRIAVARAILKDPRILLLDEATSALDAESERVVQEALDRIMVNRTTVVVAHRLSTVRNADMIAVI
HQGKIVEKGSHTELLKDPEGAYSQLIRLQEEKKSDENAAEEQKMSSIESFKQSSLRKSSLGRSLSKGGSSRGNSS
RHSFNMFGFPAGIDGNVVQDQEEDDTTQPKTEPKKVSIFRIAALNKPEIPVLILGSISAAANGVILPIFGILISSVIK
AFFQPPKKLKEDTSFWAIIFMVLGFASIIAYPAQTFFFAIAGCKLVQRIRSMCFEKVVHMEVGWFDEPENSSGTI
GARLSADAATIRGLVGDSLAQTVQNLSSILAGLIIAFLACWQLAFVVLAMLPLIALNGFLYMKFMKGFSADAK
KMYGEASQVANDAVGSIRTVASFCAEDKVMNMYSKKCEGPMKNGIRQGIVSGIGFGFSFFVLFSSYAASFYVG
ARLVDDGKTTFDSVFRVFFALTMAAMAISQSSSLSPDSSKADVAAASIFAIMDRESKIDPSVESGRVLDNVKGD
IELRHVSFKYPARPDVQIFQDLCLSIRAGKTVALVGESGSGKSTVIALLQRFYDPDSGEITLDGVEIKSLRLKWL
RQQTGLVSQEPILFNETIRANIAYGKGGDASESEIVSSAELSNAHGFISGLQQGYDTMVGERGIQLSGGQKQRV
AIARAIVKDPKVLLLDEATSALDAESERVVQDALDRVMVNRTTIVVAHRLSTIKNADVIAVVKNGVIVEKGKH
DTLINIKDGVYASLVQLHLTAAS
>LjABCB1
MRPENGGTHKHDGTSSNGEKSRQKEKVEIVPYHRLFTFADSTDILLMIVGTIGAIGNGLSIPMMSLLFGQ
MVNSFGNNQFSPDIVNQVSKVSLKFVCLGIGNGVAAFLQVACWMITGERQATRIRCLYLKTILRQNVAFF
DKETNTGEVIGRMSGDTVLIQDAMGEKVGKLLQLIATFVGGYVVAFIKGWLLTVVLLSALPLLVASGAAM
ALLIGKMTSRGQKAYAKAAHVAEQTIGSIKTVASFTGEKQAVSSYRRYLAGAYKSGVYEGFVFGMGHGMI
MLVVFCTFALAVWFGAKMIIEKGYNGGQVINIIIAVLTASMSLGQASPSMSAFAAGQAAAYKMFQTIERK
PEIDAYDPNGKILEDIHGDIDIKDVYFSYPTRPEELVFNGFSIHIPSGTTTALVGESGSGKSTIISLIER
FYDPLAGEVLIDSINMKDFQLRWIRGKIGLVSQEPALFASSIKDNIAYGKEGATIQEIRVALELANAAKF
IDRLPQGLDTMVGDHGTQLSGGQKQRIAIARAILKDPRILLLDEATSALDAQSQRTVQEALDRVMVNRTT
VVVAHRLSTVRNADMIALIHRGKMIEKGTHVELLKDPGGAYSQLIRLQEVNNESKESADNQNKRKLSTES
RSSLGNSSRHTFSVSSGLPTGVDVPKAGNEKLHPKEKSQEVPLLRLASLNKPEIPALLMGCVAAIANGAI
LPIYGVLLSSVIKTLYEPFPDMKKDSKFWSLMFVVLGIASLMAIPARCYFFSVAGSRLIQRIRLVCFEKL
INMEVGWFEEPEHSIGAIGARLSTDAAFVRALVGDALGLLIQSISTALTGLIVAFIASWQLALIVVIIAP
LMGMNGYVQIKFMKGFSADAKMMYEEASQVASDAVGSIRTIASFCAEEKVMELYSKKCEGPVKTGIQQGL
ISGIGFGVSFFLLFSVYATTFHAGARFVDAGMASFSDVFRVFFALTMTAIGISRSSSLAPDSSKGKTATA
SIFEIIDQKSKIDPSDESGGKLDSIKGEIELSHVSFKYPSRPDIQIFRDLSMTIHSGKTVALVGESGSGK
STVIALLQRFYDPDAGQITIDGIEIQKLQLKWLRQQMGLVSQEPILFNDTIRANIAYGKEGNATEAEIIT
AAELANAHRFISGLEQGYDTVVGERGILLSGGQKQRVAIARAIIKSPNILLLDEATSALDVESERVVQDA
LDKVMVNRTTVIVAHRLSTIKSADVIIVLKNGVIVEKGRHETLISIKDGYYASLVQLHTTATTV

>AtABCB1
MDNDGGAPPPPPTLVVEEPKKAEIRGVAFKELFRFADGLDYVLMGIGSVGAFVHGCSLPLFLRFFADLVN
SFGSNSNNVEKMMEEVLKYALYFLVVGAAIWASSWAEISCWMWSGERQTTKMRIKYLEAALNQDIQFFDT
EVRTSDVVFAINTDAVMVQDAISEKLGNFIHYMATFVSGFIVGFTAVWQLALVTLAVVPLIAVIGGIHTT
TLSKLSNKSQESLSQAGNIVEQTVVQIRVVMAFVGESRASQAYSSALKIAQKLGYKTGLAKGMGLGATYF
VVFCCYALLLWYGGYLVRHHLTNGGLAIATMFAVMIGGLALGQSAPSMAAFAKAKVAAAKIFRIIDHKPT
IERNSESGVELDSVTGLVELKNVDFSYPSRPDVKILNNFCLSVPAGKTIALVGSSGSGKSTVVSLIERFY
DPNSGQVLLDGQDLKTLKLRWLRQQIGLVSQEPALFATSIKENILLGRPDADQVEIEEAARVANAHSFII
KLPDGFDTQVGERGLQLSGGQKQRIAIARAMLKNPAILLLDEATSALDSESEKLVQEALDRFMIGRTTLI
IAHRLSTIRKADLVAVLQQGSVSEIGTHDELFSKGENGVYAKLIKMQEAAHETAMSNARKSSARPSSARN
SVSSPIMTRNSSYGRSPYSRRLSDFSTSDFSLSIDASSYPNYRNEKLAFKDQANSFWRLAKMNSPEWKYA
LLGSVGSVICGSLSAFFAYVLSAVLSVYYNPDHEYMIKQIDKYCYLLIGLSSAALVFNTLQHSFWDIVGE
NLTKRVREKMLSAVLKNEMAWFDQEENESARIAARLALDANNVRSAIGDRISVIVQNTALMLVACTAGFV
LQWRLALVLVAVFPVVVAATVLQKMFMTGFSGDLEAAHAKGTQLAGEAIANVRTVAAFNSEAKIVRLYTA
NLEPPLKRCFWKGQIAGSGYGVAQFCLYASYALGLWYASWLVKHGISDFSKTIRVFMVLMVSANGAAETL
TLAPDFIKGGQAMRSVFELLDRKTEIEPDDPDTTPVPDRLRGEVELKHIDFSYPSRPDIQIFRDLSLRAR
AGKTLALVGPSGCGKSSVISLIQRFYEPSSGRVMIDGKDIRKYNLKAIRKHIAIVPQEPCLFGTTIYENI

AYGHECATEAEIIQAATLASAHKFISALPEGYKTYVGERGVQLSGGQKQRIAIARALVRKAEIMLLDEAT
SALDAESERSVQEALDQACSGRTSIVVAHRLSTIRNAHVIAVIDDGKVAEQGSHSHLLKNHPDGIYARMI
QLQRFTHTQVIGMTSGSSSRVKEDDA

>AtABCB19
MSETNTTDAKTVPAEAEKKKEQSLPFFKLFSFADKFDYLLMFVGSLGAIVHGSSMPVFFLLFGQMVNGFG
KNQMDLHQMVHEVSRYSLYFVYLGLVVCFSSYAEIACWMYSGERQVAALRKKYLEAVLKQDVGFFDTDAR
TGDIVFSVSTDTLLVQDAISEKVGNFIHYLSTFLAGLVVGFVSAWKLALLSVAVIPGIAFAGGLYAYTLT
GITSKSRESYANAGVIAEQAIAQVRTVYSYVGESKALNAYSDAIQYTLKLGYKAGMAKGLGLGCTYGIAC
MSWALVFWYAGVFIRNGQTDGGKAFTAIFSAIVGGMSLGQSFSNLGAFSKGKAAGYKLMEIINQRPTIIQ
DPLDGKCLDQVHGNIEFKDVTFSYPSRPDVMIFRNFNIFFPSGKTVAVVGGSGSGKSTVVSLIERFYDPN
SGQILLDGVEIKTLQLKFLREQIGLVNQEPALFATTILENILYGKPDATMVEVEAAASAANAHSFITLLP
KGYDTQVGERGVQLSGGQKQRIAIARAMLKDPKILLLDEATSALDASSESIVQEALDRVMVGRTTVVVAH
RLCTIRNVDSIAVIQQGQVVETGTHEELIAKSGAYASLIRFQEMVGTRDFSNPSTRRTRSTRLSHSLSTK
SLSLRSGSLRNLSYSYSTGADGRIEMISNAETDRKTRAPENYFYRLLKLNSPEWPYSIMGAVGSILSGFI
GPTFAIVMSNMIEVFYYTDYDSMERKTKEYVFIYIGAGLYAVGAYLIQHYFFSIMGENLTTRVRRMMLSA
ILRNEVGWFDEDEHNSSLIAARLATDAADVKSAIAERISVILQNMTSLLTSFIVAFIVEWRVSLLILGTF
PLLVLANFAQQLSLKGFAGDTAKAHAKTSMIAGEGVSNIRTVAAFNAQSKILSLFCHELRVPQKRSLYRS
QTSGFLFGLSQLALYGSEALILWYGAHLVSKGVSTFSKVIKVFVVLVITANSVAETVSLAPEIIRGGEAV
GSVFSVLDRQTRIDPDDADADPVETIRGDIEFRHVDFAYPSRPDVMVFRDFNLRIRAGHSQALVGASGSG
KSSVIAMIERFYDPLAGKVMIDGKDIRRLNLKSLRLKIGLVQQEPALFAATIFDNIAYGKDGATESEVID
AARAANAHGFISGLPEGYKTPVGERGVQLSGGQKQRIAIARAVLKNPTVLLLDEATSALDAESECVLQEA
LERLMRGRTTVVVAHRLSTIRGVDCIGVIQDGRIVEQGSHSELVSRPEGAYSRLLQLQTHRI

>AtABCB21
MDSVIESEEGLKVDSPNRADAETSNSKIHEEDEKELKTESDLKEEKKKTEKNKQEEDEKTKTVPFHKLFAFADS
FDIILMILGTIGAVGNGLGFPIMTILFGDVIDVFGQNQNSSDVSDKIAKVALKFVYLGLGTLVAALLQVSGWMIS
GERQAGRIRSLYLQTILRQDIAFFDVETNTGEVVGRMSGDTVLIQDAMGEKVGKAIQLVSTFIGGFVIAFTEGW
LLTLVMVSSIPLLVMSGAALAIVISKMASRGQTSYAKAAVVVEQTVGSIRTVASFTGEKQAISNYNKHLVSAYR
AGVFEGASTGLGLGTLNIVIFCTYALAVWYGGKMILEKGYTGGQVLIIIFAVLTGSMSLGQASPCLSAFAAGQA
AAYKMFEAIKRKPEIDASDTTGKVLDDIRGDIELNNVNFSYPARPEEQIFRGFSLSISSGSTVALVGQSGSGKSTV
VSLIERFYDPQSGEVRIDGINLKEFQLKWIRSKIGLVSQEPVLFTSSIKENIAYGKENATVEEIRKATELANASKFI
DKLPQGLDTMVGEHGTQLSGGQKQRIAVARAILKDPRILLLDEATSALDAESERIVQEALDRIMVNRTTVVVA
HRLSTVRNADMIAVIHQGKIVEKGSHSELLRDPEGAYSQLIRLQEDTKQTEDSTDEQKLSMESMKRSSLRKSSL
SRSLSKRSSSFSMFGFPAGIDTNNEAIPEKDIKVSTPIKEKKVSFFRVAALNKPEIPMLILGSIAAVLNGVILPIFGIL
ISSVIKAFFKPPEQLKSDTRFWAIIFMLLGVASMVVFPAQTIFFSIAGCKLVQRIRSMCFEKVVRMEVGWFDETE
NSSGAIGARLSADAATVRGLVGDALAQTVQNLASVTAGLVIAFVASWQLAFIVLAMLPLIGLNGYIYMKFMV
GFSADAKRMYEEASQVANDAVGSIRTVASFCAEEKVMKMYKKKCEGPMRTGIRQGIVSGIGFGVSFFVLFSSY
AASFYAGARLVDDGKTTFDSVFRVFFALTMAAVAISQSSSLSPDSSKASNAAASIFAVIDRESKIDPSDESGRVL
DNVKGDIELRHISFKYPSRPDVQIFQDLCLSIRAGKTIALVGESGSGKSTVIALLQRFYDPDSGQITLDGVEIKTL
QLKWLRQQTGLVSQEPVLFNETIRANIAYGKGGDATETEIVSAAELSNAHGFISGLQQGYDTMVGERGVQLSG
GQKQRVAIARAIVKDPKVLLLDEATSALDAESERVVQDALDRVMVNRTTVVVAHRLSTIKNADVIAVVKNGV
IVEKGKHETLINIKDGVYASLVQLHLSAST

>SlABCB4
maettegksmpeaekkkeqslpfyqlfsfadkydyllmtcgsigailhgssmpvffllfg
emvngfgknqmdlhkmthevskyalyfvylglivcassyaeigcwmytgerqvsalrkky
leavlkqdvgffdtdartgdivfsvstdtllvqdaisekvgnfihylstflaglvvgfvs
awrlallsvavipgiafagglyaytltgltsksresyanagiiaeqaiaqvrtvysyvge
tkalnsysdaiqntlklgykagmakglglgctygiacmswalvfwyagvfirngqsdggk
aftaifsaivggmslgqsfsnlgafskgkaagyklmeiikqkptivqdtldgkclsevsg
niefknvtfsypsrpdviifrdfciffpagktvavvggsgsgkstvvslierfydpndgq
vlldnvdiktlqlrwlrdqiglvnqepalfattilenilygkpdatmaeveaatcasnah
nfitllpngyntqvgergvqlsggqkqriaiaramlknpkillldeatsaldagsesivq
ealdrlmvgrttvvvahrlstirnvdsiaviqqgqvvetgtheeliskagayaslirfqe
mvgnrdfsnpstrrtrstrlshslstkslslrsgslrnlsysystgadgriemisnaetd
rknpapqnyfcrllklnapewpysimgavgsvlsgfigptfaivmsnmievfyytnpatm
erktkeyvfiyigaglyavvayliqhyffsimgenlttrvrrmmlaailrnevgwfdeee
nnssllaarlatdaadvksaiaerisvilqnmtslltsfivafivewrvsllilatfpll
vlanfaqqlslkgfagdtakahaktsmiagegvsnirtvaafnaqekiislfsqelrvpq
mqslrrsqmsgllfgisqlalygsealilwygahlvnngvstfskvikvfvvlvitansv
aetvslapeiirggeavgsvfsildrstrvdpddpegdpvesirgdielrhvdfaypsrp
dvsvfkdlnlriragqsqalvgasgsgkssvialierfydptggkvmidgkdirrlnlks
lrlkiglvqqepalfaasifeniaygkegateaevieaaraanvhtfvsglpegyktpvg
ergvqlsggqkqriaiaravlkdpsillldeatsaldaesecvlqealerlmrgrttvlv
ahrlstirnvdtigvvqdgriveqgshselisrpegaysrllqlqhhri

>AtABCB9
MEEKSSKKNDGGNQKVSFFKLFSFADKTDVVLMTVGTIAAAGNGLTQPFMTLIFGQLINA
FGTTDPDHMVREVWKVAVKFIYLAVYSCVVAFLQVSCWMVTGERQSATIRGLYLKTILRQ
DIGYFDTETNTGEVIGRMSGDTILIQDAMGEKVGKFTQLLCTFLGGFAIAFYKGPLLAGV
LCSCIPLIVIAGAAMSLIMSKMAGRGQVAYAEAGNVVEQTVGAIRTVVAFTGEKQATEKY
ESKLEIAYKTVVQQGLISGFGLGTMLAVIFCSYGLAVWYGAKLIMEKGYNGGQVINVIFA
VLTGGMSLGQTSPSLNAFAAGRAAAFKMFETIKRSPKIDAYDMSGSVLEDIRGDIELKDV
YFRYPARPDVQIFAGFSLFVPNGKTVALVGQSGSGKSTVISLIERFYDPESGQVLIDNID
LKKLQLKWIRSKIGLVSQEPVLFATTIKENIAYGKEDATDQEIRTAIELANAAKFIDKLP
QGLDTMVGEHGTQMSGGQKQRLAIARAILKNPKILLLDEATSALDAESERIVQDALVNLM

SNRTTVVVAHRLTTIRTADVIAVVHQGKIVEKGTHDEMIQDPEGAYSQLVRLQEGSKEEA
TESERPETSLDVERSGSLRLSSAMRRSVSRNSSSSRHSFSLASNMFFPGVNVNQTDEMED
EENNVRHKKVSLKRLAHLNKPEIPVLVLGSIAAMVHGTVFPIFGLLLSSSINMFYEPAKI
LKKDSHFWALIYIALGLTNFVMIPVQNYFFGIAGGKLIKRIRSMCFDKVVHQEISWFDDT
ANSRSLVGDALALIVQNIATVTTGLIIAFTANWILALIVLALSPFIVIQGYAQTKFLTGF
SADAKAMYEEASQVANDAVSSIRTVASFCAEEKVMDLYQQKCDGPKKNGVRLGLLSGAGF
GFSFFFLYCINCVCFVSGAGLIQIGKATFGEVFKVFFALTIMAIGVSQTSAMAPDSNKAK
DSAASIFDILDSTPKIDSSSDEGTTLQNVNGDIEFRHVSFRYPMRPDVQIFRDLCLTIPS
GKTVALVGESGSGKSTVISMIERFYNPDSGKILIDQVEIQTFKLSWLRQQMGLVSQEPIL
FNETIRSNIAYGKTGGATEEEIIAAAKAANAHNFISSLPQGYDTSVGERGVQLSGGQKQR
IAIARAILKDPKILLLDEATSALDAESERVVQDALDRVMVNRTTVVVAHRLTTIKNADVI
AVVKNGVIAEKGRHETLMKISGGAYASLVTLHMSAN

>AtABCB14
MDNIEPPFSGNIHAETEVKKEEKKKMKKESVSLMGLFSAADNVDYFLMFLGGLGTCIHGG
TLPLFFVFFGGMLDSLGKLSTDPNAISSRVSQNALYLVYLGLVNLVSAWIGVACWMQTGE
RQTARLRINYLKSILAKDITFFDTEARDSNFIFHISSDAILVQDAIGDKTGHVLRYLCQF
IAGFVIGFLSVWQLTLLTLGVVPLIAIAGGGYAIVMSTISEKSEAAYADAGKVAEEVMSQ
VRTVYAFVGEEKAVKSYSNSLKKALKLSKRSGLAKGLGVGLTYSLLFCAWALLFWYASLL
VRHGKTNGAKAFTTILNVIYSGFALGQAVPSLSAISKGRVAAANIFKMIGNNNLESSERL
ENGTTLQNVVGKIEFCGVSFAYPSRPNMVFENLSFTIHSGKTFAFVGPSGSGKSTIISMV
QRFYEPRSGEILLDGNDIKNLKLKWLREQMGLVSQEPALFATTIASNILLGKEKANMDQI
IEAAKAANADSFIKSLPNGYNTQVGEGGTQLSGGQKQRIAIARAVLRNPKILLLDEATSA
LDAESEKIVQQALDNVMEKRTTIVIAHRLSTIRNVDKIVVLRDGQVRETGSHSELISRGG
DYATLVNCQDTEPQENLRSVMYESCRSQAGSYSSRRVFSSRRTSSFREDQEKTEKDSKGE
DLISSSSMIWELIKLNAPEWLYALLGSIGAVLAGSQPALFSMGLAYVLTTFYSPFPSLIK
REVDKVAIIFVGAGIVTAPIYILQHYFYTLMGERLTSRVRLSLFSAILSNEIGWFDLDEN
NTGSLTSILAADATLVRSAIADRLSTIVQNLSLTITALALAFFYSWRVAAVVTACFPLLI
AASLTEQLFLKGFGGDYTRAYSRATSLAREAISNIRTVAAFSAEKQISEQFTCELSKPTK
SALLRGHISGFGYGLSQCLAFCSYALGLWYISVLIKRNETNFEDSIKSFMVLLVTAYSVA
ETLALTPDIVKGTQALGSVFRVLHRETEIPPDQPNSRLVTHIKGDIEFRNVSFAYPTRPE
IAIFKNLNLRVSAGKSLAVVGPSGSGKSTVIGLIMRFYDPSNGNLCIDGHDIKSVNLRSL
RKKLALVQQEPALFSTSIHENIKYGNENASEAEIIEAAKAANAHEFISRMEEGYMTHVGD
KGVQLSGGQKQRVAIARAVLKDPSVLLLDEATSALDTSAEKQVQEALDKLMKGRTTILVA
HRLSTIRKADTIVVLHKGKVVEKGSHRELVSKSDGFYKKLTSLQEAV

>AtABCB15
MGKEEEKESGRNKMNCFGSVRSIFMHADGVDWLLMGLGLIGAVGDGFTTPLVLLITSKLM
NNIGGSSFNTDTFMQSISKNSVALLYVACGSWVVCFLEGYCWTRTGERQTARMREKYLRA
VLRQDVGYFDLHVTSTSDVITSVSSDSFVIQDVLSEKLPNFLMSASTFVGSYIVGFILLW
RLAIVGLPFIVLLVIPGLMYGRALISISRKIREEYNEAGFVAEQAISSVRTVYAFSGERK
TISKFSTALQGSVKLGIKQGLAKGITIGSNGITFAMWGFMSWYGSRMVMYHGAQGGTVFA
VAAAIAIGGVSLGGGLSNLKYFFEAASVGERIMEVINRVPKIDSDNPDGHKLEKIRGEVE
FKNVKFVYPSRLETSIFDDFCLRVPSGKTVALVGGSGSGKSTVISLLQRFYDPLAGEILI
DGVSIDKLQVKWLRSQMGLVSQEPALFATTIKENILFGKEDASMDDVVEAAKASNAHNFI
SQLPNGYETQVGERGVQMSGGQKQRIAIARAIIKSPTILLLDEATSALDSESERVVQEAL
ENASIGRTTILIAHRLSTIRNADVISVVKNGHIVETGSHDELMENIDGQYSTLVHLQQIE
KQDINVSVKIGPISDPSKDIRNSSRVSTLSRSSSANSVTGPSTIKNLSEDNKPQLPSFKR
LLAMNLPEWKQALYGCISATLFGAIQPAYAYSLGSMVSVYFLTSHDEIKEKTRIYALSFV
GLAVLSFLINISQHYNFAYMGEYLTKRIRERMLSKVLTFEVGWFDRDENSSGAICSRLAK
DANVVRSLVGDRMALVVQTVSAVTIAFTMGLVIAWRLALVMIAVQPVIIVCFYTRRVLLK
SMSKKAIKAQDESSKLAAEAVSNVRTITAFSSQERIMKMLEKAQESPRRESIRQSWFAGF
GLAMSQSLTSCTWALDFWYGGRLIQDGYITAKALFETFMILVSTGRVIADAGSMTTDLAK
GSDAVGSVFAVLDRYTSIDPEDPDGYETERITGQVEFLDVDFSYPTRPDVIIFKNFSIKI

EEGKSTAIVGPSGSGKSTIIGLIERFYDPLKGIVKIDGRDIRSYHLRSLRRHIALVSQEP
TLFAGTIRENIIYGGVSDKIDEAEIIEAAKAANAHDFITSLTEGYDTYCGDRGVQLSGGQ
KQRIAIARAVLKNPSVLLLDEATSALDSQSERVVQDALERVMVGRTSVVIAHRLSTIQNC
DAIAVLDKGKLVERGTHSSLLSKGPTGIYFSLVSLQTTSG

>AtABCB24
MMRVSQLQLCRTSLSYRLRSGYHHHHHLHHSFFKLIKRNSILESPPTNASHQSPSPITPMVNARVMFFST
STSAPHPEKINRTSSENILRMISSYLWMKDNPKLCFRVISAFACLVGAKFLNVQVPFLFKVAIDWLSSSS
FVDSNPYLVAAFATPSSVLIGYGIARSGSSAFNELRTSVFSKVALRTIRTISRKVLSRLHDLDLRYHLNR
DTGALNRIIDRGSRAINTILSAMVFNIMPTILEISMVSCILAYKFGAVYALITCLSVGSYIAFTLAMTQW
RIKIRKAMNEAENDASTRAIDSLINYETVKYFNNEDYEARKYDQLHENYEDAALQSRKSFALLNFGQSFI
FSTALSTAMVLCSQGIMNGQMTVGDLVMVNGLLFQLSLPLYFLGVVYSDTVQGLVDMKSMFKFLEERSDI
GDKDIDRKLPPLVLKGGSISFENVHFSYLPERKILDGISFEVPAGKSVAIVGSSGSGKSTILRMIFRFFD
VDSGNVKIDGQDIKEVRLESLRSSIGVVPQDTVLFNDTIFHNIHYGNLSATEEEVYNAARRAAIHDTIMK
FPDKYSTAVGERGLMLSGGEKQRVALARAFLKSPAILLCDEATSALDSKTEAEIMKTLRSLASNRTCIFI
AHRLTTAMQCDEILVMEKGKVVEKGTHEVLLGKSGRYAKLWTQQNSKLEV

>OsABCB19
MADESGSCGGGGGGGGGCEAVKKRVDQSVAFHELFGFADPLDWLLMAAGSAGAVVHGAAM
PVFFLLFGELINGFGKNQHSLRRMTDEVSKYSLYFVYLGLVVCASSYLEIACWMYTGERQ
VGALRRRYLEAVLRQDVGFFDTDARTGDVVFSVSTDTLLVQDAIGEKVGNFIHYLSTFLA
GLVVGFVSAWRLALLSIAVIPGIAFAGGLYAYTLTGLTSKSRDSYANAGIIAEQAIAQVR
TVYSYVGESKALNSYSEAIQNTLKLGYKAGMAKGLGIGCTYGIACMSWALVFWYAGVFIR
NGQTDGGKAFTAIFSAIVGGLSLGQSFSNLGAFSKGKIAGYKLLEVIRQRPTIVQDPADG
RCLDEVHGNIEFKEVAFSYPSRPDVMIFRDFSLFFPAGKTAAVVGGSGSGKSTVVALIER
FYDPNQGQVLLDNVDIKTLQLKWLRDQIGLVNQEPALFATTILENILYGKPDATMAEVEA
AATSANAHSFIALLPNGYNTQVGERGLQLSGGQKQRIAIARAMLKNPKILLLDEATSALD
AGSENIVQEALDRLMVGRTTVVVAHRLSTIRCVDMIAVIQQGQVVETGTHDELLAKGSSG
AYAALIRFQEMARNRDFRGPSTRKSRSSRLSNSLSTRSLSLRSGSLRNLSYSYSTGADGR
IEMVSNADNDRKYPAPKGYFFKLLKLNAPEWPYTILGAIGSILSGFIGPTFAIVMSNMIE
VFYFRDPNAMERKTREYVFIYIGTGLYAVVAYLVQHYFFSIMGENLTTRVRRMMLAAILR
NDVGWFDQEENNSSLVAARLSTDAADVKSAIAERISVILQNMTSLLVSFVVGFIIEWRVA
VLILVTFPLLVLANFAQQLSMKGFAGDTAKAHAKTSMIAGEGVSNIRTVAAFNAQDKVLS
LFCTELRVPQMHSLRRSQISGALFGLSQLSLYASEALILWYGAHLVRHHVSTFSKVIKVF
VVLVITANTVAETVSLAPEIVRGGESIRSVFAILNYRTRIDPDEPETEPVESVRGDIDFR
HVDFAYPSRPDVMVFKDFSLRIRAGQSQALVGASGSGKSTVIALIERFYDPLAGKVMIDG
KDIRRLNVRSLRLKIGLVQQEPVLFATSIFENIAYGKDGATEEEVIEAAKVANMHGFVSA
LPEGYKTPVGERGVQLSGGQKQRIAIARAVLKDPAVLLLDEATSALDAESECVLQEALER
IMKGRTAVLVAHRLSTIRGVDSIAVVQDGRVVEQGSHGELVSRPDGAYSRLLQLQLHHG

>ZmABCB1
MSSSDPEEIRARVVVLGSPHADGGDEWARPELEAFHLPSPAHQPPGFLAGQPEAAEQPTLPAPAGRSSSS
SNTPTTSAGGGAAPPPPSSPPPPPASLETEQPPNARPASAGANDSKKPTPPAALRDLFRFADGLDCALML
IGTLGALVHGCSLPVFLRFFADLVDSFGSHADDPDTMVRLVVKYAFYFLVVGAAIWASSWAEISCWMWTG
ERQSTRMRIRYLDAALRQDVSFFDTDVRASDVIYAINADAVVVQDAISEKLGNLIHYMATFVAGFVVGFT
AAWQLALVTLAVVPLIAVIGGLSAAALAKLSSRSQDALSGASGIAEQALAQIRIVQAFVGEEREMRAYSA
ALAVAQRIGYRSGFAKGLGLGGTYFTVFCCYGLLLWYGGHLVRAQHTNGGLAIATMFSVMIGGLALGQSA
PSMAAFAKARVAAAKIFRIIDHRPGISSRDGAEPESVTGRVEMRGVDFAYPSRPDVPILRGFSLSVPAGK
TIALVGSSGSGKSTVVSLIERFYDPSAGQILLDGHDLRSLELRWLRRQIGLVSQEPALFATSIRENLLLG
RDSQSATLAEMEEAARVANAHSFIIKLPDGYDTQVGERGLQLSGGQKQRIAIARAMLKNPAILLLDEATS
ALDSESEKLVQEALDRFMIGRTTLVIAHRLSTIRKADVVAVLQGGAVSEMGAHDELMAKGENGTYAKLIR
MQEQAHEAALVNARRSSARPSSARNSVSSPIMTRNSSYGRSPYSRRLSDFSTSDFTLSIHDPHHHHRTMA
DKQLAFRAGASSFLRLARMNSPEWAYALAGSIGSMVCGSFSAIFAYILSAVLSVYYAPDPRYMKREIAKY
CYLLIGMSSAALLFNTVQHVFWDTVGENLTKRVREKMFAAVLRNEIAWFDADENASARVAARLALDAQNV

RSAIGDRISVIVQNSALMLVACTAGFVLQWRLALVLLAVFPLVVGATVLQKMFMKGFSGDLEAAHARATQ
IAGEAVANLRTVAAFNAERKITGLFEANLRGPLRRCFWKGQIAGSGYGVAQFLLYASYALGLWYAAWLVK
HGVSDFSRTIRVFMVLMVSANGAAETLTLAPDFIKGGRAMRSVFETIDRKTEVEPDDVDAAPVPERPRGE
VELKHVDFSYPSRPDIQVFRDLSLRARAGKTLALVGPSGCGKSSVLALVQRFYEPTSGRVLLDGKDVRKY
NLRALRRVVAVVPQEPFLFAASIHENIAYGREGATEAEVVEAAAQANAHRFIAALPEGYRTQVGERGVQL
SGGQRQRIAIARALVKQAAIVLLDEATSALDAESERCVQEALERAGSGRTTIVVAHRLATVRGAHTIAVI
DDGKVAEQGSHSHLLKHHPDGCYARMLQLQRLTGAAAGPGPSTSCNGAA

>AtABCB4
MASESGLNGDPNILEEVSETKRDKEEEEEVKKTEKKDEEHEKTKTVPFYKLFAFADSFDFLLMILGTLGSIGNG
LGFPLMTLLFGDLIDAFGENQTNTTDKVSKVALKFVWLGIGTFAAAFLQLSGWMISGERQAARIRSLYLKTILR
QDIAFFDIDTNTGEVVGRMSGDTVLIQDAMGEKVGKAIQLLATFVGGFVIAFVRGWLLTLVMLSSIPLLVMAG
ALLAIVIAKTASRGQTAYAKAATVVEQTIGSIRTVASFTGEKQAISNYNKHLVTAYKAGVIEGGSTGLGLGTLF
LVVFCSYALAVWYGGKLILDKGYTGGQVLNIIIAVLTGSMSLGQTSPCLSAFAAGQAAAYKMFETIERRPNIDS
YSTNGKVLDDIKGDIELKDVYFTYPARPDEQIFRGFSLFISSGTTVALVGQSGSGKSTVVSLIERFYDPQAGDVLI
DGINLKEFQLKWIRSKIGLVSQEPVLFTASIKDNIAYGKEDATTEEIKAAAELANASKFVDKLPQGLDTMVGEH
GTQLSGGQKQRIAVARAILKDPRILLLDEATSALDAESERVVQEALDRIMVNRTTVVVAHRLSTVRNADMIAVI
HQGKIVEKGSHTELLKDPEGAYSQLIRLQEEKKSDENAAEEQKMSSIESFKQSSLRKSSLGRSLSKGGSSRGNSS
RHSFNMFGFPAGIDGNVVQDQEEDDTTQPKTEPKKVSIFRIAALNKPEIPVLILGSISAAANGVILPIFGILISSVIK
AFFQPPKKLKEDTSFWAIIFMVLGFASIIAYPAQTFFFAIAGCKLVQRIRSMCFEKVVHMEVGWFDEPENSSGTI
GARLSADAATIRGLVGDSLAQTVQNLSSILAGLIIAFLACWQLAFVVLAMLPLIALNGFLYMKFMKGFSADAK
KMYGEASQVANDAVGSIRTVASFCAEDKVMNMYSKKCEGPMKNGIRQGIVSGIGFGFSFFVLFSSYAASFYVG
ARLVDDGKTTFDSVFRVFFALTMAAMAISQSSSLSPDSSKADVAAASIFAIMDRESKIDPSVESGRVLDNVKGD
IELRHVSFKYPARPDVQIFQDLCLSIRAGKTVALVGESGSGKSTVIALLQRFYDPDSGEITLDGVEIKSLRLKWL
RQQTGLVSQEPILFNETIRANIAYGKGGDASESEIVSSAELSNAHGFISGLQQGYDTMVGERGIQLSGGQKQRV
AIARAIVKDPKVLLLDEATSALDAESERVVQDALDRVMVNRTTIVVAHRLSTIKNADVIAVVKNGVIVEKGKH
DTLINIKDGVYASLVQLHLTAAS

>LjABCB1
MRPENGGTHKHDGTSSNGEKSRQKEKVEIVPYHRLFTFADSTDILLMIVGTIGAIGNGLSIPMMSLLFGQ
MVNSFGNNQFSPDIVNQVSKVSLKFVCLGIGNGVAAFLQVACWMITGERQATRIRCLYLKTILRQNVAFF
DKETNTGEVIGRMSGDTVLIQDAMGEKVGKLLQLIATFVGGYVVAFIKGWLLTVVLLSALPLLVASGAAM
ALLIGKMTSRGQKAYAKAAHVAEQTIGSIKTVASFTGEKQAVSSYRRYLAGAYKSGVYEGFVFGMGHGMI
MLVVFCTFALAVWFGAKMIIEKGYNGGQVINIIIAVLTASMSLGQASPSMSAFAAGQAAAYKMFQTIERK
PEIDAYDPNGKILEDIHGDIDIKDVYFSYPTRPEELVFNGFSIHIPSGTTTALVGESGSGKSTIISLIER
FYDPLAGEVLIDSINMKDFQLRWIRGKIGLVSQEPALFASSIKDNIAYGKEGATIQEIRVALELANAAKF
IDRLPQGLDTMVGDHGTQLSGGQKQRIAIARAILKDPRILLLDEATSALDAQSQRTVQEALDRVMVNRTT
VVVAHRLSTVRNADMIALIHRGKMIEKGTHVELLKDPGGAYSQLIRLQEVNNESKESADNQNKRKLSTES
RSSLGNSSRHTFSVSSGLPTGVDVPKAGNEKLHPKEKSQEVPLLRLASLNKPEIPALLMGCVAAIANGAI
LPIYGVLLSSVIKTLYEPFPDMKKDSKFWSLMFVVLGIASLMAIPARCYFFSVAGSRLIQRIRLVCFEKL
INMEVGWFEEPEHSIGAIGARLSTDAAFVRALVGDALGLLIQSISTALTGLIVAFIASWQLALIVVIIAP
LMGMNGYVQIKFMKGFSADAKMMYEEASQVASDAVGSIRTIASFCAEEKVMELYSKKCEGPVKTGIQQGL
ISGIGFGVSFFLLFSVYATTFHAGARFVDAGMASFSDVFRVFFALTMTAIGISRSSSLAPDSSKGKTATA
SIFEIIDQKSKIDPSDESGGKLDSIKGEIELSHVSFKYPSRPDIQIFRDLSMTIHSGKTVALVGESGSGK
STVIALLQRFYDPDAGQITIDGIEIQKLQLKWLRQQMGLVSQEPILFNDTIRANIAYGKEGNATEAEIIT
AAELANAHRFISGLEQGYDTVVGERGILLSGGQKQRVAIARAIIKSPNILLLDEATSALDVESERVVQDA
LDKVMVNRTTVIVAHRLSTIKSADVIIVLKNGVIVEKGRHETLISIKDGYYASLVQLHTTATTV

>AtABCB1
MDNDGGAPPPPPTLVVEEPKKAEIRGVAFKELFRFADGLDYVLMGIGSVGAFVHGCSLPLFLRFFADLVN
SFGSNSNNVEKMMEEVLKYALYFLVVGAAIWASSWAEISCWMWSGERQTTKMRIKYLEAALNQDIQFFDT
EVRTSDVVFAINTDAVMVQDAISEKLGNFIHYMATFVSGFIVGFTAVWQLALVTLAVVPLIAVIGGIHTT
TLSKLSNKSQESLSQAGNIVEQTVVQIRVVMAFVGESRASQAYSSALKIAQKLGYKTGLAKGMGLGATYF
VVFCCYALLLWYGGYLVRHHLTNGGLAIATMFAVMIGGLALGQSAPSMAAFAKAKVAAAKIFRIIDHKPT

IERNSESGVELDSVTGLVELKNVDFSYPSRPDVKILNNFCLSVPAGKTIALVGSSGSGKSTVVSLIERFY
DPNSGQVLLDGQDLKTLKLRWLRQQIGLVSQEPALFATSIKENILLGRPDADQVEIEEAARVANAHSFII
KLPDGFDTQVGERGLQLSGGQKQRIAIARAMLKNPAILLLDEATSALDSESEKLVQEALDRFMIGRTTLI
IAHRLSTIRKADLVAVLQQGSVSEIGTHDELFSKGENGVYAKLIKMQEAAHETAMSNARKSSARPSSARN
SVSSPIMTRNSSYGRSPYSRRLSDFSTSDFSLSIDASSYPNYRNEKLAFKDQANSFWRLAKMNSPEWKYA
LLGSVGSVICGSLSAFFAYVLSAVLSVYYNPDHEYMIKQIDKYCYLLIGLSSAALVFNTLQHSFWDIVGE
NLTKRVREKMLSAVLKNEMAWFDQEENESARIAARLALDANNVRSAIGDRISVIVQNTALMLVACTAGFV
LQWRLALVLVAVFPVVVAATVLQKMFMTGFSGDLEAAHAKGTQLAGEAIANVRTVAAFNSEAKIVRLYTA
NLEPPLKRCFWKGQIAGSGYGVAQFCLYASYALGLWYASWLVKHGISDFSKTIRVFMVLMVSANGAAETL
TLAPDFIKGGQAMRSVFELLDRKTEIEPDDPDTTPVPDRLRGEVELKHIDFSYPSRPDIQIFRDLSLRAR
AGKTLALVGPSGCGKSSVISLIQRFYEPSSGRVMIDGKDIRKYNLKAIRKHIAIVPQEPCLFGTTIYENI
AYGHECATEAEIIQAATLASAHKFISALPEGYKTYVGERGVQLSGGQKQRIAIARALVRKAEIMLLDEAT
SALDAESERSVQEALDQACSGRTSIVVAHRLSTIRNAHVIAVIDDGKVAEQGSHSHLLKNHPDGIYARMI
QLQRFTHTQVIGMTSGSSSRVKEDDA

>AtABCB19
MSETNTTDAKTVPAEAEKKKEQSLPFFKLFSFADKFDYLLMFVGSLGAIVHGSSMPVFFLLFGQMVNGFG
KNQMDLHQMVHEVSRYSLYFVYLGLVVCFSSYAEIACWMYSGERQVAALRKKYLEAVLKQDVGFFDTDAR
TGDIVFSVSTDTLLVQDAISEKVGNFIHYLSTFLAGLVVGFVSAWKLALLSVAVIPGIAFAGGLYAYTLT
GITSKSRESYANAGVIAEQAIAQVRTVYSYVGESKALNAYSDAIQYTLKLGYKAGMAKGLGLGCTYGIAC
MSWALVFWYAGVFIRNGQTDGGKAFTAIFSAIVGGMSLGQSFSNLGAFSKGKAAGYKLMEIINQRPTIIQ
DPLDGKCLDQVHGNIEFKDVTFSYPSRPDVMIFRNFNIFFPSGKTVAVVGGSGSGKSTVVSLIERFYDPN
SGQILLDGVEIKTLQLKFLREQIGLVNQEPALFATTILENILYGKPDATMVEVEAAASAANAHSFITLLP
KGYDTQVGERGVQLSGGQKQRIAIARAMLKDPKILLLDEATSALDASSESIVQEALDRVMVGRTTVVVAH
RLCTIRNVDSIAVIQQGQVVETGTHEELIAKSGAYASLIRFQEMVGTRDFSNPSTRRTRSTRLSHSLSTK
SLSLRSGSLRNLSYSYSTGADGRIEMISNAETDRKTRAPENYFYRLLKLNSPEWPYSIMGAVGSILSGFI
GPTFAIVMSNMIEVFYYTDYDSMERKTKEYVFIYIGAGLYAVGAYLIQHYFFSIMGENLTTRVRRMMLSA
ILRNEVGWFDEDEHNSSLIAARLATDAADVKSAIAERISVILQNMTSLLTSFIVAFIVEWRVSLLILGTF
PLLVLANFAQQLSLKGFAGDTAKAHAKTSMIAGEGVSNIRTVAAFNAQSKILSLFCHELRVPQKRSLYRS
QTSGFLFGLSQLALYGSEALILWYGAHLVSKGVSTFSKVIKVFVVLVITANSVAETVSLAPEIIRGGEAV
GSVFSVLDRQTRIDPDDADADPVETIRGDIEFRHVDFAYPSRPDVMVFRDFNLRIRAGHSQALVGASGSG
KSSVIAMIERFYDPLAGKVMIDGKDIRRLNLKSLRLKIGLVQQEPALFAATIFDNIAYGKDGATESEVID
AARAANAHGFISGLPEGYKTPVGERGVQLSGGQKQRIAIARAVLKNPTVLLLDEATSALDAESECVLQEA
LERLMRGRTTVVVAHRLSTIRGVDCIGVIQDGRIVEQGSHSELVSRPEGAYSRLLQLQTHRI

>XP_020880317.1_ABCB21_X2_Al
MDSVMESEEGLKVDSPNRANVETSNSKIHEEEEKELKTESVKKKTEKNKKEEEDEKTKTVPFHKLFAFADSFDI
VLMILGTIGAVGNGLGFPIMTILFGDVIDVFGQNQNSSDVSDKIAKVALKFVYLGLGTLVAALLQVSGWMISGE
RQAGRIRSLYLQTILRQDIAFFDVETNTGEVVGRMSGDTVLIQDAMGEKVGKAIQLVSTFVGGFVIAFTEGWLL
TLVMVSSIPLLVMTGAALAIVISKMASRGQTSYAKAAVVVEQTVGSIRTVASFTGEKQAISNYNKHLVSAYRA
GVFEGASTGLGLGTLNIVIFCTYALAVWYGGKMILEKGYTGGQVLIIIFAVLTGSMSLGQASPCLSAFAAGQAA
AYKMFETIKRKPEIDASDTTGKVLDDIRGDIELKDVNFSYPARPEEQIFRGFSLSISSGSTVALVGQSGSGKSTVV
SLIERFYDPQSGEVRIDGINLKEFQLKWIRSKIGLVSQEPVLFTSSIKENIAYGKENATIEEIRKATELANASKFIDK
LPQGLDTMVGEHGTQLSGGQKQRIAVARAILKDPRILLLDEATSALDAESERIVQEALDRIMVNRTTVVVAHR
LSTVRNADMIAVIHQGKIVEKGSHSELLRDPEGSYSQLIRLQEDTKQTEDSTDEQKLSMESMKRSSLRKSSLSRS
LSKRSSSFSMFGFPAGIDTNNEAIPEQDIKVSTPIKESETEHKKVSFFRVAALNKPEIPMLILGSIAAVLNGVILPIF
GILISSVIEAFFKPPQQLKSDTRFWAIIFMLLGVASMVVYPAQTIFFSIAGCKLVQRIRSMCFEKVVRMEVGWFD
ETENSSGAIGARLSADAATVRGLVGDALAQTVQNLASVTAGLVIAFVASWQLAFIVLAMLPLIGLNGYIYMKF
MVGFSADAKRMYEEASQVANDAVGSIRTVASFCAEEKVMKMYKKKCEGPMRTGIRQGIVSGIGFGVSFFVLF
SSYAASFYAGARLVDDGKTTFDSVFRVFFALTMAAVAISQSSSLSPDSSKASNAAASIFAVIDRESKIDPSDESG
RVLDNVKGDIELRHISFKYPSRPDVQIFQDLCLSIRAGKTIALVGESGSGKSTVIALLQRFYDPDSGQITLDGVEI

KTLQLKWLRQQTGLVSQEPVLFNETIRANIAYGKGGDASETKIVSAAELSNAHGFISGLQQGYDTMVGERGVQ
LSGGQKQRVAIARAIVKDPKVLLLDEATSALDAESERVVQDALDRVMVNRTTVVVAHRLSTIKNADVIAVVK
NGVIVEKGKHETLINIKDGVYASLVQLHLSAST

>XP_020880316.1_ABCB21_X1_Al
MDQTTMDSVMESEEGLKVDSPNRANVETSNSKIHEEEEKELKTESVKKKTEKNKKEEEDEKTKTVPFHKLFAF
ADSFDIVLMILGTIGAVGNGLGFPIMTILFGDVIDVFGQNQNSSDVSDKIAKVALKFVYLGLGTLVAALLQVSG
WMISGERQAGRIRSLYLQTILRQDIAFFDVETNTGEVVGRMSGDTVLIQDAMGEKVGKAIQLVSTFVGGFVIAF
TEGWLLTLVMVSSIPLLVMTGAALAIVISKMASRGQTSYAKAAVVVEQTVGSIRTVASFTGEKQAISNYNKHL
VSAYRAGVFEGASTGLGLGTLNIVIFCTYALAVWYGGKMILEKGYTGGQVLIIIFAVLTGSMSLGQASPCLSAF
AAGQAAAYKMFETIKRKPEIDASDTTGKVLDDIRGDIELKDVNFSYPARPEEQIFRGFSLSISSGSTVALVGQSG
SGKSTVVSLIERFYDPQSGEVRIDGINLKEFQLKWIRSKIGLVSQEPVLFTSSIKENIAYGKENATIEEIRKATELA
NASKFIDKLPQGLDTMVGEHGTQLSGGQKQRIAVARAILKDPRILLLDEATSALDAESERIVQEALDRIMVNRT
TVVVAHRLSTVRNADMIAVIHQGKIVEKGSHSELLRDPEGSYSQLIRLQEDTKQTEDSTDEQKLSMESMKRSSL
RKSSLSRSLSKRSSSFSMFGFPAGIDTNNEAIPEQDIKVSTPIKESETEHKKVSFFRVAALNKPEIPMLILGSIAAVL
NGVILPIFGILISSVIEAFFKPPQQLKSDTRFWAIIFMLLGVASMVVYPAQTIFFSIAGCKLVQRIRSMCFEKVVRM
EVGWFDETENSSGAIGARLSADAATVRGLVGDALAQTVQNLASVTAGLVIAFVASWQLAFIVLAMLPLIGLNG
YIYMKFMVGFSADAKRMYEEASQVANDAVGSIRTVASFCAEEKVMKMYKKKCEGPMRTGIRQGIVSGIGFGV
SFFVLFSSYAASFYAGARLVDDGKTTFDSVFRVFFALTMAAVAISQSSSLSPDSSKASNAAASIFAVIDRESKIDP
SDESGRVLDNVKGDIELRHISFKYPSRPDVQIFQDLCLSIRAGKTIALVGESGSGKSTVIALLQRFYDPDSGQITL
DGVEIKTLQLKWLRQQTGLVSQEPVLFNETIRANIAYGKGGDASETKIVSAAELSNAHGFISGLQQGYDTMVG
ERGVQLSGGQKQRVAIARAIVKDPKVLLLDEATSALDAESERVVQDALDRVMVNRTTVVVAHRLSTIKNADV
IAVVKNGVIVEKGKHETLINIKDGVYASLVQLHLSAST

>XP_013591523.1_P_ABCB21_Bo
MDGIMESEEGLKVDSPNRTEAETSNSKTPEKEAKTESVRKDEKTKTVPFHKLFAFADSFDIILMILGTIGAVGNG
LGFPIMTILFGDVIDVFGQNQNSTDVSDKIAKVALKFVYLGLGTLVAALLQVSGWMISGERQAGRIRSLYLKTI
LRQDIAFFDVETNTGEVVGRMSGDTVLIQDAMGEKVGKAIQLISTFIGGFVIAFAEGWLLTLVMVSSIPLLVMS
GAALAIVISKMASRGQTSYAKAAVVVEQTVGSIRTVASFTGEKQAISSYNKHLVSAYRAGVFEGASTGIGLGTL
NVVIFCTYALAVWYGGKMILEKGYTGGQVLIIIFAVLTGSMSLGQASPCLSAFAAGQAAAYKMFETIKRKPEID
ASDTTGKVLDDVRGDIELRDVDFSYPARPEEQIFRGFSLSISSGSTVALVGQSGSGKSTVVSLIERFYDPQSGEVR
IDGVNLKEFQLKWIRSKIGLVSQEPVLFTSSIKENIAYGKEDATVEEIRKATELANASKFIDKLPQGLDTMVGEH
GTQLSGGQKQRIAVARAILKDPRILLLDEATSALDAESERIVQEALDRIMVNRTTVVVAHRLSTVRNADMIAVI
HQGKIVEKGSHSELLRDPEGAYSQLIRLQEDNKKSEDSREEQKVSMESMKRSSLRKSSLSRSLSKRSPSFSMFGF
PAGIDATNETKPEIKEEETVHKKVSFLRVAALNKPEIPMLILGSIAAVLNGVILPIFGILISSVIKAFFKPPEQLKSD
TSFWAIIFMLLGVASMVVYPAQTIFFSIAGCKLVQRIRSMCFEKVVHMEVGWFDETENSSGAIGARLSADAATV
RGLVGDALAQTVQNIASVTAGVVIAFVASWQLAFIVLAMLPLIGLNGYIYMKFMVGFSADAKRMYEEASQVA
NDAVGSIRTVASFCAEEKVMKMYKKKCEGPMKTGIRQGIVSGIGFGVSFFVLFASYAASFYAGARLVDDGKTT
FDAVFRVFFALTMAAVAISQSSSLSPDSSKASNAAASIFAVIDRKSKIDPSDESGRVLDNVKGDIELRHVSFKYPS
RPDVQIFQDLCLSIRAGKTIALVGESGSGKSTVIALLQRFYDPDSGQITLDGVEIKTLQLKWLRQQTGLVSQEPV
LFNETIRANIAYGKGGDASETEIISAAELSNAHGFISGLQQGYDTMVGERGVQLSGGQKQRVAIARAIVKDPKV
LLLDEATSALDAESERVVQDALDRVMVNRTTVVVAHRLSTIKNADVIAVVKNGVIVEKGKHDTLINIKDGVY
ASLVQLHLSAST

>XP_022568115.1_ABCB21-l_Bn
MDGIMESEEGLKVDSPNRTEAETSNSKTPEKEAKTESVRKDEKTKTVPFHKLFAFADSFDIILMILGTIGAVGNG
LGFPIMTILFGDVIDVFGQNQNSTDVSDKIAKVALKFVYLGLGTLVAALLQVSGWMISGERQAGRIRSLYLKTI
LRQDIAFFDVETNTGEVVGRMSGDTVLIQDAMGEKVGKAIQLISTFIGGFVIAFAEGWLLTLVMVSSIPLLVMS
GAALAIVISKMASRGQTSYAKAAVVVEQTVGSIRTVASFTGEKQAISSYNKHLVSAYRAGVFEGASTGIGLGTL
NVVIFCTYALAVWYGGKMILEKGYTGGQVLIIIFAVLTGSMSLGQASPCLSAFAAGQAAAYKMFETIKRKPEID
ASNTTGKVLDDVRGDIELRDVDFSYPARPEEQIFRGFSLSISSGSTVALVGQSGSGKSTVVSLIERFYDPQSGEVR
IDGVNLKEFQLKWIRSKIGLVSQEPVLFTSSIKENIAYGKEDATVEEIRKATELANASKFIDKLPQGLDTMVGEH
GTQLSGGQKQRIAVARAILKDPRILLLDEATSALDAESERIVQEALDRIMVNRTTVVVAHRLSTVRNADMIAVI
HQGKIVEKGSHSELLRDPEGAYSQLIRLQEDNKKSEDSREEQKVSMESMKRSSLRKSSLSRSLSKRSPSFSMFGF

PAGIDATNETKPEIKEEETVHKKVSFLRVAALNKPEIPMLILGSIAAVLNGVILPIFGILISSVIKAFFKPPEQLKSD
TSFWAIIFMLLGVASMVVYPAQTIFFSIAGCKLVQRIRSMCFEKVVHMEVGWFDETENSSGAIGARLSADAATV
RGLVGDALAQTVQNIASVTAGVVIAFVASWQLAFIVLAMLPLIGLNGYIYMKFMVGFSADAKRMYEEASQVA
NDAVGSIRTVASFCAEEKVMKMYKKKCEGPMKTGIRQGIVSGIGFGVSFFVLFASYAASFYAGARLVDDGKTT
FDAVFRVFFALTMAAVAISQSSSLSPDSSKASNAAASIFAVIDRKSKIDPSDESGRVLDNVKGDIELRHVSFKYPS
RPDVQIFQDLCLSIRAGKTIALVGESGSGKSTVIALLQRFYDPDSGQITLDGVEIKTLQLKWLRQQTGLVSQEPV
LFNETIRANIAYGKGGDASETEIISAAELSNAHGFISGLQQGYDTMVGERGVQLSGGQKQRVAIARAIVKDPKV
LLLDEATSALDAESERVVQDALDRVMVNRTTVVVAHRLSTIKNADVIAVVKNGVIVEKGKHDTLINIKDGVY
ASLVQLHLSAST

>XP_006402405.1_ABCB21_Es
MNSVMESEEGMKVESTNRAEAEISNLRTNEEEVKTESVQKEEEKKTEKRKEEEENEKTKTVPFYKLFAFADSY
DILLMILGTIGAVGNGLAFPIMTVLFGDVIDVFGQNQNSSDVSDKIAKVALKFVYLGLGTLVAALLQVSGWMIS
GERQAGRIRSLYLKTILRQDIAFFDVETNTGEVVGRMSGDTVLIQDAMGEKVGKAIQLVSTFIGGFVIAFMEGW
LLTLVMVSSIPLLVMSGAALAIVISKMASRGQTSYAKAAVVVEQTVGSIRTVASFTGEKQAISNYNKHLVSAYR
AGVFEGASTGLGLGTLNIVIFCTYALAVWYGGKMILEKGYTGGQVLIIIFAVLTGSMSLGQASPCLSAFAAGQA
AAYKMFETIKRKPEIDASDTTGKVLDDVRGDIELKDVSFSYPARPEEQIFRGFSLSISSGSTVALVGQSGSGKST
VVSLIERFYDPLAGEVRIDGINLKEFQLKWIRSKIGLVSQEPVLFTSSIKENIAYGKENATIEEIRKATELANASKF
IDKLPQGLETMVGEHGTQLSGGQKQRIAVARAILKDPRILLLDEATSALDAESERIVQEALDRIMVNRTTVVVA
HRLSTVRNADMIAVIHQGKIVEKGSHSELLRDPEGAYSQLIRLQEENKQSEDSTDEQKISMESMKRSSLRKSSLS
RSLSKRSSSFSMFGFPAGIDSNNEAKPEQGEASTPIKEEETEHKKVSFLRVAALNKPEIPMLILGSIAAVLNGVILP
IFGILISSVIKAFFKPPEQLKSDTRFWAIIFMLLGVASMVVFPAQTIFFSIAGCKLVQRIRSMCFEKVVHMEVGWF
DETENSSGAIGARLSADAATVRGLVGDALAQTVQNLASVTAGLVIAFVASWQLAFIVLAMLPLIGLNGYIYMK
FMVGFSADSKRMYEEASQVANDAVGSIRTVASFCAEEKVMKMYKKKCEGPMKTGIRQGIVSGIGFGVSFFVL
FASYAASFYAGARLVDDGKTTFDAVFRVFFALTMAAVAISQSSSLSPDSSKASNAAASIFAVIDRESKIDPSDES
GRVLENVKGDIELRHISFKYPSRPDVQIFQDLCLSIRAGKTIALVGESGSGKSTVIALLQRFYDPNSGQITLDGVE
IKTLQLKWLRQQTGLVSQEPVLFNETIRANIAYGKGGDASETEIVSAAELSNAHGFISGLQQGYDTMVGERGV
QLSGGQKQRVAIARAIVKDPKVLLLDEATSALDAESERVVQDALDRVMVNRTTVVVAHRLSTIKNADVIAVV
KNGVIVEKGKHDTLISIKDGVYASLVQLHLSAST

>XP_018445446.1_P_ABCB21_Rs
MDSVIESEKDPKVDSPNIAEAETSNSKPHKEEKEVKRESVLKEEKKKKEDEDEKTKTVPFHKLFAFADSFDIILM
ILGTIGAVGNGLGFPIMTILFGDVIDVFGQNQNSSDVSDKIAKVALKFVYLGLGTLVAALLQVSGWMISGERQA
GRIRSLYLKTILRQDIAFFDVETNTGEVVGRMSGDTVLIQDAMGEKVGKAIQLISTFIGGFVIAFTEGWLLTLVM
VSSIPLLVMSGAALAIVISKMASRGQTSYAKAAVVVEQTVGSIRTVASFTGEKQAISSYNKHLVSAYRAGVFEG
ASTGLGLGTLNIVIFCTYALAVWYGGKMILEKGYTGGQVLVIIFAVLTGSMSLGQASPCLSAFAAGQAAAYKM
FETIKRKPEIDAFDTSGKVLDDVRGDIELRDVSFSYPARPEEQIFRGFSLSISSGSTVALVGQSGSGKSTVVSLIER
FYDPQSGEVRIDGVNLKEFQLKWIRSKIGLVSQEPVLFTSSIKENIAYGKEDATLEEIRMATELANASKFIDKLPQ
GLDTMVGEHGTQLSGGQKQRIAVARAILKDPRILLLDEATSALDAESERIVQEALDRIMVNRTTVVVAHRLST
VRNADMIAVIHQGKIVEKGSHSELLRDPEGAYSQLIRLQEDNKKSEDPTEEQKISMESMKRSSLRKSSLSRSLSK
RSSSFSMFGFPAGIDTTNETKPEQGQDSSPIKKVSFLRVAALNKPEIPMLILGSIAAVLNGVILPIFGILISSVIKAFF
KPPEQLKSDTRFWAIIFMLLGVASMVVYPAQTIFFSIAGCKLVQRIRSMCFEKVVHMEVGWFDETENSSGAIGA
RLSADAATVRGLVGDALAQTVQNLASVTAGVVIAFVASWQLAFIVLAMLPLIGLNGYVYMKFMVGFSADAK
RMYEEASQVANDAVGSIRTVASFCAEEKVMKMYKKKCEGPMKTGIRQGIVSGIGFGVSFFVLFASYAASFYA
GARLVDDGKTTFDSVFRVFFALTMAAVAISQSSSLSPDSSKASNAAASIFAVIDRESKIDPSDESGRVLDNVKGD
IELRHVSFKYPSRPDVQIFQDLCLSIRAGKTIALVGESGSGKSTVIALLQRFYDPDSGQITLDGVEIKTLQLKWLR
QQTGLVSQEPVLFNETIRANIAYGKGGDASENEIVSAAELSNAHGFISGLQQGYETMVGERGVQLSGGQKQRV
AIARAIVKDPKVLLLDEATSALDAESERVVQDALDRVMVNRTTVVVAHRLSTIKNADVIAVVKNGVIVEKGK
HDTLIGIKDGVYASLVQLHLSAST

>XP_013648504.1_ABCB21_Bn
MDGIMESEEGLKVDSPNRTEAETSNSKTPEEEVKTESVLKEEKKKTDEKKKKEEDDEKTKTVPFHKLFAFADS
FDIILMILGTIGAVGNGLGFPIMTILFGDVIDVFGQNQNSSDVSDKIAKVALKFVYLGLGTLVAALLQVSGWMIS
GERQAGRIRSLYLKTILRQDIAFFDVETNTGEVVGRMSGDTVLIQDAMGEKVGKAIQLISTFIGGFVIAFAEGWL

LTLVMVSSIPLLVISGAALAIVISKMASRGQTSYAKAAVVVEQTVGSIRTVASFTGEKQAISSYNKHLVSAYRA
GVFEGASTGLGLGTLNIVIFCTYALAVWYGGKMILEKGYTGGQVLIIIFAVLTGSMSLGQASPCLSAFAAGQAA
AYKMFETIKRKPEINASDTTGKVLDDVRGDIELRDVNFSYPARPEEQIFRGFSLSISSGSTVALVGQSGSGKSTV
VSLIERFYDPQSGEVRIDGVNLKEFQLKWIRSKIGLVSQEPVLFTSSIKENIAYGKEDATVEEIRKATELANASKF
IDKLPQGLDTMVGEHGTQLSGGQKQRIAVARAILKDPRILLLDEATSALDAESERIVQEALDRIMVNRTTVVVA
HRLSTVRNADMIAVIHQGKIVEKGSHSELLRDPEGAYSQLIRLQEDNKKSEDSTEEQKISMESMKRSSLRKSSLS
RSLSKRSPSFSMFGFPAGIDATNETKQEIKEEETEHKKVSFLRVAALNKPEIPMLILGSIAAVLNGVILPIFGILISS
VIKAFFKPPEQLKSDTSFWAIIFMLLGVASMVVYPAQTIFFSIAGCKLVQRIRSMCFEKVVHMEVGWFDETENS
SGAIGARLSADAATVRGLVGDALAQTVQNLASVTAGVVIAFVASWQLAFIVLAMLPLIGLNGYIYMKFMVGF
SADAKRMYEEASQVANDAVGSIRTVASFCAEERVMKMYKKKCEGPMKTGIRQGIVSGIGFGVSFFVLFASYA
ASFYAGARLVDDGKTTFDAVFRVFFALTMAAVAISQSSSLSPDSSKASNAAASIFAVIDRESKIDPSDESGRVLD
DVKGDIELRHVSFKYPSRPDVQIFQDLCLSIRAGKTIALVGESGSGKSTVIALLQRFYDPDSGQITLDGVEIKTLQ
LKWLRQQTGLVSQEPVLFNETIRANIAYGKGGDASETEIISAAELSNAHGFISGLQKGYDTMVGERGVQLSGG
QKQRVAIARAIVKDPKVLLLDEATSALDAESERVVQDALDRVMVNRTTVVVAHRLSTIKNADVIAVVKNGVI
VEKGKHDTLISIKDGVYASLVQLHLSAST

>XP_009104372.1_P_ABCB21_Br
MDGIMESEEGLKVDSPNRTEAETSNSKTPEEEVKTEAVLKEEKKKTDEKKKKEEDDEKTKTVPFYKLFAFADS
FDIILMILGTIGAVGNGLGFPIMTILFGDVIDVFGQNQNSSDVSDKIAKVALKFVYLGLGTLVAALLQVSGWMIS
GERQAGRIRSLYLKTILRQDIAFFDVETNTGEVVGRMSGDTVLIQDAMGEKVGKAIQLISTFIGGFVIAFAEGWL
LTLVMVSSIPLLVISGAALAIVISKMASRGQTSYAKAAVVVEQTVGSIRTVASFTGEKQAISSYNKHLVSAYRA
GVFEGASTGIGLGTLNIVIFCTYALAVWYGGKMILEKGYTGGQVLIIIFAVLTGSMSLGQASPCLSAFAAGQAA
AYKMFETIKRKPEIDASDTTGKVLDDVRGDIELIDVNFSYPARPEEQIFRGFSLSISSGSTVALVGQSGSGKSTVV
SLIERFYDPQSGEVRIDGVNLKEFKLKWIRSKIGLVSQEPVLFTSSIKENIAYGKEDATVEEIRKATELANASKFI
DKLPQGLDTMVGEHGTQLSGGQKQRIAVARAILKDPRILLLDEATSALDAESERIVQEALDRIMVNRTTVVVA
HRLSTVRNADMIAVIHQGKIVEKGSHSELLRDPEGAYSQLIRLQEDNKKSEDSTEEQKISTESMKRSSLRKSSLS
RSLSKRSPSFSMFGFPAGIEATNETKPEIKEDETVHKKVSFLRVAALNKPEIPMLILGSIAAVLNGVILPIFGILISS
VIKAFFKPPEQLKSDTSFWALIFMLLGVASMVVYPAQTIFFSIAGCKLVQRIRSMCFEKVVHMEVGWFDETENS
SGAIGARLSADAATVRGLVGDALAQTVQNLASVTAGVVIAFVASWQLAFIVLAMLPLIGLNGYIYMKFMVGF
SADAKRMYEEASQVANDAVGSIRTVASFCAEERVMKMYKKKCEGPMKTGIRQGIVSGIGFGVSFFVLFASYA
ASFYAGARLVDDGKTTFDAVFRVFFALTMAAVAISQSSSLSPDSSKASNAAASIFAVIDRESKIDPSDESGRVLD
DVKGDIELRHVSFKYPSRPDVQIFQDLCLSIRAGKTIALVGESGSGKSTVIALLQRFYDPDSGQITLDGVEIKTLQ
LKWLRQQTGLVSQEPVLFNETIRANIAYGKGGDASETEIISAAELSNAHGFISGLQQGYDTMVGERGVQLSGG
QKQRVAIARAIVKDPKVLLLDEATSALDAESERVVQDALDRVMVNRTTVVVAHRLSTIKNADVIAVVKNGVI
VEKGKHDTLINIKDGVYASLVQLHLSAST

>XP_010544933.1_P_ABCB21_Th
MASENGVKDDSNVAEASMSKIPEEKGEEVKKESDLNGEKKTEKNKEDEKTKTVPFYKLFAFADSFDVFLMIIG
AIGAVGNGLGFPIMTILFGDVVDAFGQNQNSTDVSNAISKVALKFVYLGIGTLVAAFLQVSGWMISGERQAARI
RSLYLKTILRQDIAFFDVETNTGEVVGRMSGDTVLIQDAMGEKVGKAIQLVSTFIGGFVIAFTEGWLLTLVMLS
SIPLLVMAGAGLAIVIAKMASVGQTAYAKAAVVVEQTIGSIRTVVSFTGEKQAISNYKKHLASAYRAGVFEGA
STGLGLGTLFFVVFCSYALAVWYGGKMILEKGYSGGQVLNVIVAVLTGSMSLGQASPCLSAFAAGQAAAYK
MFETIERKPDIDAYDTRGKVLDDIRGDIELKDVYFSYPARPDEQIFRGFSLFIPSGTTAALVGQSGSGKSTVISLIE
RFYDPQAGEVLIDGVNLKEFQLRWIRSKIGLVSQEPVLFTSSIKENIAYGKEDATIEEIRAAAELANASKFIDKLP
QGLDTMVGEHGTQLSGGQKQRIAVARAILKDPRILLLDEATSALDAESERIVQEALDRVMVNRTTVIVAHRLS
TVRNADTIAVIHQGKIVEQGSHSVLLKDPEGAYSQLIHLQEEKKESVDATPNEQNRSEISMESMKQQSMGRSLG
RSISKGSSRGNSSRHSFSVFGFPAGIDTNTSTLDQEPEAPLPAEEQKKVSLLRIATLNKPEIPVIILGSISAAGNGVI
LPLFGILISSVIKAFFKPADELKDDTRFWAIIFVALGIASIIAYPAQTFFFSIAGCKLVQRIRTMCFEKVVNMEVGW
FDEPQNSSGAIGXXXXXPPATIRGLVGDALAQAVQNLSSVVAGLVIAFTASWQLAFIVLAMLPLIALNGFVYM
KFMKGFSADAKKMYEQASQVANDAVGSIRTVASFCAEERVMKMYTKKCEGPMKTGIRQGLVSGIGFGVSFF
VLFASYATSFYAGGRLVDDGKTTFDSVFRVFFALTMAAMAISQSSSLTPDSSKADVAAASIFAIIDRKSKIDPSD
ESGTVLDNVKGDIELRHVSFRYPSRPDVQIFQDLCLTIRSGKTVALVGESGSGKSTVIALLQRFYDPDSGHITLD
GVEIQRLKLKWLRQQMGLVSQEPVLFNDTIRANIAYGKGGNATESEIISAAELSNAHRFISGLQQGYDTMVGER
GVQLSGGQKQRVAIARAIVKDPKVLLLDEATSALDAESERVVQDALDRVMVNRTTVVVAHRLSTIKNADVIA

VVKNGVIVEKGKHETLINIKDGVYASLVQLHLSAST

>XP_010523879.1_P_ABCB4-l_Th
MDSSIAESSMSKTREEEGEGKQESDLKEEKNEEEDEKIKTVPFYKLFAFADSFDVFLMVTGTIGAVGNGLGLPI
MTILFGDVVDAFGQNQNRSDVSKDISKVALKFIYLGIGTLAAAFLQVSGWMISGERQAARIRSLYLKTILRQDI
AFFDVETNTGEVVGRMSGDTVLIQDAMGEKVGKAIQLVSTFIGGFAIAFSEGWLLTLVMLSSIPPLVMAGAAL
AIVIAKMASRGQTAYAKSAVVVEQTISSIRTVASFTGEKQAINAYKKHLVAAYKAGVFEGTSTGLGLGTLFLV
VFCSYSLSVWYGGKMILEKGYTGGQVVNIIIAVLTGSMSLGQASPCLSAFAAGKAAAYKMFETIERKPEIDSYA
TTGKVLDDVRGDIELKDVTFSYPARPDEQIFHGFSLFLPSGTTAALVGQSGSGKSTVISLIERFYDPQAGQVLID
GVNLKEFKLRWIRTKIGLVSQEPVLFTSSIKENIAYGKEDATIEEIKAAAELANASKFIDKLPQGLDTMVGEHGT
QLSGGQKQRIAVARAILKDPRILLLDEATSALDAESERIVQEALDRIMVNRTTVIVAHRLSTVRNADMIAVIHQ
GKIVEKGSHSELLNDPEGAYSQLIRLQEEKRQSEGPTDEQNRSETSMEPAKQQSLGSLLGRSISRGGSSRGNSSR
HSFSVFGFPAGVDTNNSNLDQEETPLPTEEHKKVSILRIAALNKPEIPAIILGSISAMGNGVILPLFGILMSSVIKAF
YKPPDQLRHDTRFWAIIFLVLGIAAIVAYPAQTFFFSIAGCKLVQRIRTMCFEKVVCMEVGWFDEPQNSSGAIG
ARLSADAATIRGLVGDALAQAVQNLSSVTAGLVIAFTASWQLALIVLATLPLIALNGVLYMKFMKGFSADAEN
MYQQASQVANDAVGSIRTVASFCAEEKVMKMYKKKCEGPMKTGIRQGLVSGIGFGVSFFVLFASYATSFYAG
ARLVDAGKTTFDSVFRVFFALTMAAVAISQSSSLAPDSSKAGISAASIFAIIDRKSRIDPSDESGMMLENVKGDIE
LRHVSFRYPARPDVQIFQDLCLNIRSGKTVALVGESGSGKSTVIALLQRFYDPDSGDITLDGVEIQRLKLKWLR
QQMGLVSQEPVLFNDTIRANIAYGKGGNATEAEIISAAQLSNAHTFISGLLQGYETMVGERGVQLSGGQKQRV
AIARAIVKDPKVLLLDEATSALDAESERVVQDALDRVMVNRTTVVVAHRLSTIKNADVIAVVKNGVIVEKGK
HETLINIFDGVYASLVQLHMTAST

>XP_010518387.1_P_ABCB4_Cs
MASESGVKGDSNIVEEVSETKRESKEEGEAMKTEKQNKEDHDEKTKTVPFYKLFAFADSFDFLLMLLGTLGSI
GNGLGFPIMTLLFGDLVDAFGQNQTNTTDKVSKIALKFVYLGIGTFVAAFLQLSGWMISGERQAARIRSLYLKT
ILRQDITFFDIDTNTGEVVGRMSGDTVLIQDAMGEKVGKAIQLVSTFVGGFVIAFIRGWLLTLVMLSSIPLLVMS
GALLAIVIAKTASRGQTAYAKAAVVVEQTIGSIRTVASFTGEKQAISNYNKHLVSAYKAGVIEGGSTGLGLGTL
FLVIFCSYALAVWYGGKLILEKGYTGGQVLNVIISVLTGSMSLGQASPCLSAFAAGQAAAYKMFETIERKPDID
SYSTDGKVLDDIKGDIELKDVYFTYPARPDEQIFRGFSLFVSSGSTVALVGQSGSGKSTVVSLIERFYDPQAGEV
LIDGINLKEFQLRWIRSKIGLVSQEPVLFTASIKDNIAYGKEDATIEEIRAAAELANASKFVDKLPQGLDTMVGE
HGTQLSGGQKQRIAVARAILKDPRILLLDEATSALDAESERVVQEALDRIMVNRTTVVVAHRLSTVRNADMIA
VIHQGKIVEKGSHTELLQDSEGAYSQLIRLQEEKKSDENASDERKMSSIESFKQSSLRKSSLGRSLSKGGSSRGN
SSRHSFNMFGFPAGIDAQDQDQEEATTEPKTKQKKVSIFRIAALNKPEIPVLILGSISAAANGVILPLFGILLSSVI
DAFFQPPKKLKEDTSFWAVIFMVLGFASIIAYPAQTFFFGIAGCKLVQRIRSMCFEKVVHMEVGWFDEPENSSG
TIGARLSADAATIRGLVGDALAQTVQNLSSILAGLIIAFLACWQLSFVVLAMLPLIALNGFLYMKFMTGFSADA
KKMYGEASQVANDAVGSIRTVASFCAEDKVMKMYTKKCEGPMKTGIRQGIVSGIGFGFSFFVLFASYATSFYV
GARLVDDGKTTFDAVFRVFFALTMAAMAISQSSSLSPDSSKADVAAASIFAIMDRESKIDPSVESGRVLENVKG
DIELRHVSFKYPARPDVQIFQDLCLSIRAGKTVALVGESGSGKSTVIALLQRFYDPDSGEITLDGVEIKSLRLKW
LRQQTGLVSQEPILFNETIRANIAYGKGGDASESEIVSSAELSNAHGFISGLQQGYDTMVGERGIQLSGGQKQRV
AIARAIVKDPKVLLLDEATSALDAESERVVQDALDRVMVNRTTIVVAHRLSTIKNADVIAVVKNGVIVEKGKH
ETLINIQDGVYASLVQLHLTAAS

>XP_010507941.1_P_ABCB4_Cs
MASESGVNGDSNIVEEVSETKRESKVEGEAMKTEEKNKEEHDEKTKTVPFYKLFAFADSFDFLLMLLGTLGSI
GNGLGFPIMTLLFGDLVDAFGQNQTNTTDKVSKIALKFVYLGIGTFVAAFLQLSGWMISGERQAARIRSLYLKT
ILRQDITFFDIDTNTGEVVGRMSGDTVLIQDAMGEKVGKAIQLVSTFVGGFVIAFIRGWLLTLVMLSSIPLLVMS
GALLAIVIAKTASRGQTAYAKAAVVVEQTIGSIRTVASFTGEKQAISNYNKHLVSAYKAGVIEGGSTGLGLGTL
FLVIFCSYALAVWYGGKLILEKGYTGGQVLNVIISVLTGSMSLGQASPCLSAFAAGQAAAYKMFETIERKPDID
SYSTDGKVLDDIKGDIELKDVYFTYPARPDEQIFRGFSLFVSSGSTVALVGQSGSGKSTVVSLIERFYDPQAGEV
LIDGINLKEFQLRWIRSKIGLVSQEPVLFTASIKDNIAYGKEDATIEEIKAAAELANASKFVDKLPQGLDTMVGE
HGTQLSGGQKQRIAVARAILKDPRILLLDEATSALDAESERVVQEALDRIMVNRTTIVVAHRLSTVRNADMIAV
IHQGKIVEKGSHTELLKDSEGAYSQLIRLQEEKKSDENASDERKMSSIESFKQSSLRKSSLGRSLSKGGSSRGNSS
RHSFNMFGFPAGIDPQDQDQEEATTEPKTKQKKVSIFRIAALNKPEIPVLILGSISAAANGVILPLFGILLSSVIDA
FFQPPKKLKEDTSFWAVIFMVLGFASIIAYPAQTFFFGIAGCKLVQRIRSMCFEKVVHMEVGWFDEPENSSGTIG

ARLSADAATIRGLVGDALAQTVQNLSSILAGLIIAFLACWQLSFVVLAMLPLIALNGFLYMKFMTGFSADAKK
MYGEASQVANDAVGSIRTVASFCAEDKVMKMYTKKCEGPMKTGIRQGIVSGIGFGFSFFVLFASYATSFYVGA
RLVDDGKTTFDAVFRVFFALTMAAMAISQSSSLSPDSSKADVAAASIFAIMDRESKIDPSVESGRVLENVKGDIE
LRHVSFKYPARPDVQIFQDLCLSIRAGKTVALVGESGSGKSTVIALLQRFYDPDSGEITLDGVEIKSLRLKWLRQ
ETGLVSQEPILFNETIRANIAYGKGGDASESEIVSSAELSNAHGFISGLQQGYDTMVGERGIQLSGGQKQRVAIA
RAIVKDPKVLLLDEATSALDAESERVVQDALDRVMVNRTTIVVAHRLSTIKNADVIAVVKNGVIVEKGKHESL
INIQDGVYASLVQLHLTAAS

>XP_022557861.1_ABCB4_X1_Bn
MASESGLNGDSNIVEEVSETKRSRDVEEDEEVKKTEKNNNTEEHEKTKTVTFYKLFAFADSFDFLLMTLGTLG
SIGNGLGFPIMTILFGDLVDAFGENQTDSNVADKVSKVALKFVWLGIGTLAAAFLQLSGWMISGERQAARIRS
MYLKTILRQDIAFFDVDTNTGEVVGRMSGDTVLIQDAMGEKVGKAIQLLATFVGGFVIAFIRGWLLTLVMLSSI
PLLVMAGAGLAIVIARTASRGQTAYAKAAVVVEQTIGSIRTVASFTGEKQAISNYNKHLVTAYKAGVIEGGST
GLGLGTLFLVVFCSYALAVWYGGKLILDKGYTGGQVLNIIISVLTGSMSLGQASPCLTAFAAGQAAAYKMFET
IERRPDIDSYSTNGKVLDDIKGDIELKDVYFTYPARPDEQIFRGFSLFISSGTTVALVGQSGSGKSTVVSLIERFYD
PQAGEVLIDGVNLKEFQLKWIRSKIGLVSQEPVLFTSSIKDNIAYGKEDATLEEIKAAAELANASKFVDKLPQGL
DTMVGEHGTQLSGGQKQRIAVARAILKDPRILLLDEATSALDAESERVVQEALDRIMVNRTTVVVAHRLSTVR
NADMIAVIHQGKIVEKGSHTELLKDPEGAYSQLIRLQEDKKPVETQAGERKMSSIESFKQSSFRKSSLGRSLSKG
GSSRGNSSRHSFNMFGFPSGIEGNDVVQDQEEDDTTEPKTKPKKVSIRRIAALNKPEIPVLILGTISAAANGVILPI
FGILIASVIKAFFKPPKELKEDTSFWAIIFMVLGFASVIAYPAQTFFFSIAGCKLVQRIRSMCFEKVVHMEVGWFD
ESEHSSGTIGARLSADAAAIRGLVGDALAQMVQNLSSILAGLIIAFLACWQLAFVVLAMLPLIALNGFLYMKFM
KGFSADAKKMYGEASQVANDAVGSIRTVASFCAEDKVMNMYTKKCEGPMKTGIRQGIVSGIGFGVSFFVLFA
SYATSFYVGAKLVDDGKTTFDSVFRVFFALTMAAVAISQSSSLSPDSSKADIAAASIFGIIDRESKIDPSVESGRV
LDTVKGDIELRHVSFRYPSRPDVQIFQDLCLSIRAGKTVALVGESGSGKSTVIALLQRFYDPDSGEITLDGVEIKT
LRLKWLRQQTGLVSQEPILFNETIRANIAYGKGGDASESEIVSAAELSNAHGFISGLQQGYDTMVGERGIQLSG
GQKQRVAIARAIVKDPKVLLLDEATSALDAESERVVQDALDRVMVNRTTIVVAHRLSTIKNADVIAVVKNGVI
VEKGKHESLINIKDGVYASLVQLHLSAAS

>XP_022557862.1_ABCB4_X2_Bn
MASESGLNGDSNIVEEVSETKRSRDVEEDEEVKKTEKNNNTEEHEKTKTVTFYKLFAFADSFDFLLMTLGTLG
SIGNGLGFPIMTILFGDLVDAFGENQTDSNVADKVSKVALKFVWLGIGTLAAAFLQLSGWMISGERQAARIRS
MYLKTILRQDIAFFDVDTNTGEVVGRMSGDTVLIQDAMGEKVGKAIQLLATFVGGFVIAFIRGWLLTLVMLSSI
PLLVMAGAGLAIVIARTASRGQTAYAKAAVVVEQTIGSIRTVASFTGEKQAISNYNKHLVTAYKAGVIEGGST
GLGLGTLFLVVFCSYALAVWYGGKLILDKGYTGGQVLNIIISVLTGSMSLGQASPCLTAFAAGQAAAYKMFET
IERRPDIDSYSTNGKVLDDIKGDIELKDVYFTYPARPDEQIFRGFSLFISSGTTVALVGQSGSGKSTVVSLIERFYD
PQAGEVLIDGVNLKEFQLKWIRSKIGLVSQEPVLFTSSIKDNIAYGKEDATLEEIKAAAELANASKFVDKLPQGL
DTMVGEHGTQLSGGQKQRIAVARAILKDPRILLLDEATSALDAESERVVQEALDRIMVNRTTVVVAHRLSTVR
NADMIAVIHQGKIVEKGSHTELLKDPEGAYSQLIRLQEDKKPVETQAGERKMSSIESFKQSSFRKSSLGRSLSKG
GSSRGNSSRHSFNMFGFPSGIEGNDVVQDQEEDDTTEPKTKPKKVSIRRIAALNKPEIPVLILGTISAAANGVILPI
FGILIASVIKAFFKPPKELKEDTSFWAIIFMVLGFASVIAYPAQTFFFSIAGCKLVQRIRSMCFEKVVHMEVGWFD
ESEHSSGTIGARLSADAAAIRGLVGDALAQMVQNLSSILAGLIIAFLACWQLAFVVLAMLPLIALNGFLYMKFM
KGFSADAKKMYGEASQVANDAVGSIRTVASFCAEDKVMNMYTKKCEGPMKTGIRQGIVSGIGFGVSFFVLFA
SYATSFYVGAKLVDDGKTTFDSVFRVFFALTMAAVAISQSSSLSPDSSKADIAAASIFGIIDRESKIDPSVESGRV
LDTVKGDIELRHVSFRYPSRPDVQIFQDLCLSIRAGKTVALVGESGSGKSTVIALLQRFYDPDSGEITLDGVEIKT
LRLKWLRQQTGLVSQEPILFNETIRANIAYGKGGDASESEIVSAGELSNAHGFISGLQQGYDTMVGERGIQLSG
GQKQRVAIARAIVKDPKVLLLDEATSALDAESERVVQDALDRVMVNRTTIVVAHRLSTIKNADVIAVVKNGVI
VEKGKHESLINIKDGVYASLVQLHLSAAS

>XP_013631705.1_P_ABCB4_Bo
MASESGLNGDSNIVEEVSETKRSRDVEEDEEVKKTEKNNNTEEHEKTKTVPFYKLFAFADSFDFLLMTLGTLG
SIGNGLGFPIMTILFGDLVDAFGENQTDSNVADKVSKVALKFVWLGIGTLAAAFLQLSGWMISGERQAARIRS
MYLKTILRQDIAFFDVDTNTGEVVGRMSGDTVLIQDAMGEKVGKAIQLLATFVGGFVIAFIRGWLLTLVMLSSI
PLLVMAGAGLAIVIARTASRGQTAYAKAAVVVEQTIGSIRTVASFTGEKQAISNYNKHLVTAYKAGVIEGGST
GLGLGTLFLVVFCSYALAVWYGGKLILDKGYTGGQVLNIIISVLTGSMSLGQASPCLTAFAAGQAAAYKMFET

IERRPDIDSYSTDGKVLDDIKGDIELKDVYFTYPARPDEQIFRGFSLFISSGTTVALVGQSGSGKSTVVSLIERFYD
PQAGEVLIDGVNLKEFQLKWIRSKIGLVSQEPVLFTSSIKDNIAYGKEDATLEEIKAAAELANASKFVDKLPQGL
DTMVGEHGTQLSGGQKQRIAVARAILKDPRILLLDEATSALDAESERVVQEALDRIMVNRTTVVVAHRLSTVR
NADTIAVIHQGKIVEKGSHTELLKDPEGAYSQLIRLQEEKKPEETQAGERKMSSIESFKQSSFRKSSLGRSLSKG
GSSRGNSSRHSFNMFGFPSGIEGNDVVQDQEEDDTTEPKTKPKKVSIRRIAALNKPEIPVLILGTISAAANGVILPI
FGILIASVIKAFFKPPKELKEDTSFWAIIFMVLGFASVIAYPAQTFFFSIAGCKLVQRIRSMCFEKVVHMEVGWFD
ESEHSSGTIGARLSADAAAIRGLVGDALAQMVQNLSSILAGLIIAFLACWQLAFVVLAMLPLIALNGFLYMKFM
KGFSADAKKMYGEASQVANDAVGSIRTVASFCAEDKVMNMYTKKCEGPMKTGIRQGIVSGIGFGVSFFVLFA
SYATSFYVGAKLVDDGKTTFDSVFRVFFALTMAAVAISQSSSLSPDSSKADIAAASIFGIIDRESKIDPSVESGRV
LDTVKGDIELRHVSFRYPSRPDVQIFQDLCLSIRAGKTVALVGESGSGKSTVIALLQRFYDPDSGEITLDGVEIKT
LRLKWLRQQTGLVSQEPILFNETIRANIAYGKGGDASESEIVSAAELSNAHGFISGLQQGYDTMVGERGIQLSG
GQKQRVAIARAIVKDPKVLLLDEATSALDAESERVVQDALDRVMVNRTTIVVAHRLSTIKNADVIAVVKNGVI
VEKGKHESLINIKDGVYASLVQLHLTAAS

>XP_006397891.1_ABCB4_Es
MASENGFNGDVHIVEEVSETKRSREEEGEKEVKKTEKNDEDHEKTKTVPFYKLFAFADSLDFLLMILGTLGSIG
NGLGFPIMTVLFGDLVDAFGQNQANSNVTDKVSKVALKFVWLGIGTFAAAFLQLSGWMISGERQAARIRSMY
LKTILRQDIAFFDVDTNTGEVVGRMSGDTVLIQDAMGEKVGKAIQLLSTFVGGFVIAFIRGWLLTLVMLSSIPLL
VIAGAGLAIVIARTASHGQTAYAKAAIVVEQTIGSIRTVASFTGERQAISNYNKHLVTAYKAGVIEGGSTGLGL
GTLFLVVFCSYALAVWYGGKLILDKGYTGGQVLNIIISVLTGSMSLGQASPCLSAFAAGQAAAYKMFETIERRP
DIDSYSTNGKVLDDIKGDIELKDVYFTYPARPDEQIFRGFSLFISSGTTVALVGQSGSGKSTVVSLIERFYDPQAG
EVLIDGINLKEFQLKWIRSKIGLVSQEPVLFTSSIKDNIAYGKEDATIEEIKAAAELANASKFVDKLPQGLDTMV
GEHGTQLSGGQKQRIAVARAILKDPRILLLDEATSALDAESERVVQEALDRIMVNRTTVVVAHRLSTVRNADM
ISVIHQGKIVEKGSHSELLKDPEGAYSQLIRLQEEKRSDENPEDEQKMSSIESFKQSSLRKSSLGRSLSKGGSSRG
NSSRHSFNMFGFPAGIEGNDVAQDQEEVPKTKQKKVSIRRIAALNKPEIPVLILGAISAAANGVILPIFGILIASVI
KAFFKPPKELKEDTSFWAIIFMVLGFASVIAYPAQTFFFAIAGCKLVQRIRSMCFEKVVHMEVGWFDEPENSSG
TIGARLSADAAAIRGLVGDALAQMVQNLSSILAGLIIAFLACWQLAFVVLAMLPLIALNGFLYMKFMTGFSAD
AKKMYGEASQVANDAVGSIRTVASFCAEDKVMNMYTKKCEGPLKTGIRQGIVSGIGFGFSFFVLFASYATSFY
VGARLVDDGKTNFDSVFKVFFALTMAAIAISQSSSLSPDSSKADIAAASIFAIMDRESKIDPSVESGRVLDNVKG
DIELRHVSFKYPARPDVQIFQDLCLSIRAGKTVALVGESGSGKSTVIALLQRFYDPDSGEITLDGVEIKSLRLKW
LRQQTGLVSQEPILFNETIRANIAYGKGGDASESEIVSAAELSNAHGFISGLQQGYDTMVGERGIQLSGGQKQR
VAIARAIVKDPKVLLLDEATSALDAESERVVQDALDRVMVNRTTIVVAHRLSTIKNADVIAVVKNGVIVEKGK
HETLINITDGVYASLVQLHLSAAS

>XP_018441942.1_P_ABCB4_Rs
MASESGLNGDSNIVEEVSETKRSRNEDKEVKKTEKKEDHEETKTVPFYKLFAFADSFDFLLMILGTLGSIGNGL
GFPIMTVLFGDLVDAFGENQTDSNVADKVSKVSLKFVWLGIGTFAAAFLQLSGWMISGERQAARIRSMYLKTI
LRQDIAFFDVDTNTGEVVGRMSGDTVLIQDAMGEKVGKAIQLLATFVGGFVIAFIRGWLLTLVMLSSIPLLVM
AGAGLAIVIARTASRGQTAYAKAAVVVEQTIGSIRTVASFTGEKQAISNYNKHLVTAYKAGVMEGGSTGLGLG
TLFLVVFCSYALAVWYGGKLILDKGYTGGQVLNIIISVLTGSMSLGQASPCLTAFAAGQAAAYKMFETIERRPD
IDSYSTDGKVLDDIKGDIELKDVYFTYPARPDEQIFRGFSLFISSGTTVALVGQSGSGKSTVVSLIERFYDPQAGE
VLIDGVNLKEFQLKWIRSKIGLVSQEPVLFTSSIKDNIAYGKEDATIEEIKAAAELANASKFVDKLPQGLDTMVG
EHGTQLSGGQKQRIAVARAILKDPRILLLDEATSALDAESERVVQEALDRIMVNRTTVVVAHRLSTVRNADSIA
VIHQGKIVEKGSHTELLKDPEGAYSQLIRLQEEKKHEENPTDEHKMSSVESFKQSSFRKSSLGRSLSKGGSSRGN
SSRHSFNMFGFPSGIEGNDVGQDQEEVDTTEPKTKPKKVSIRRIAALNKPEIPVLILGTISAAANGVILPIFGILIAS
VIKAFFKPPKELKEDTSFWAIIFMVLGFASVIAYPAQTFFFAIAGCKLVQRIRSMCFEKVVHMEVGWFDEAEHS
SGTIGARLSADAAAIRGLVGDALAQMVQNLSSILAGLIIAFLACWQLAFVVLAMLPLIALNGFLYMKFMKGFS
ADAKKMYGEASQVANDAVGSIRTVASFCAEDKVMNMYTKKCEGPMKTGIRQGIVSGIGFGVSFFVLFASYAT
SFYVGAKLVDDGKTTFDSVFRVFFALTMAAIAISQSSSLSPDSSKADIAAASIFGIIDRESKIDPSVESGRVLDTVK
GDIELRHVSFKYPSRPDVQIFQDLCLSIRAGKTVALVGESGSGKSTVIALLQRFYDPDSGEITLDGVEIKTLRLK
WLRQQTGLVSQEPILFNETIRANIAYGKGGDASESEIVSAAELSNAHGFISGLQQGYDTMVGERGIQLSGGQKQ
RVAIARAIVKDPKVLLLDEATSALDAESERVVQDALDRVMVNRTTIVVAHRLSTIKNADVIAVVKNGVIVEKG
KHESLINIKDGVYASLVQLHLSAAS

>XP_020885129.1_ABCB4_Al
MASESGLNGDSNIVEEVSETKRGKEKEEVKKTEKKDEEHEKTKTVPFYKLFAFADSFDFLLMTLGTLGSIGNGL
GFPLMTLLFGDLIDAFGQNQTNTDVTAKVSKVALKFVWLGIGTFAAAFLQLSGWMISGERQAARIRSLYLKTIL
RQDIAFFDIDTNTGEVVGRMSSDTVLIQDAMGEKVGKAIQLLATFVGGFVIAFVRGWLLTLVMLTSIPLLVMA
GALLAIVIAKTASRGQTAYAKAATVVEQTIGSIRTVASFTGEKQAISNYNKHLVTAYKAGVIEGGSTGLGLGTL
FLVVFCSYALAVWYGGKLILDKGYTGGQVLNIIIAVLTGSMSLGQTSPCLSAFAAGQAAAFKMFETIERKPNID
SYSTDGKVLDDIKGDIELKDVYFTYPARPDEQIFRGFSLFISSGTTVALVGQSGSGKSTVVSLIERFYDPQTGEVL
IDGINLKEFQLKWIRSKIGLVSQEPVLFTASIKDNIAYGKEDATIEEIKAAAELANASKFVDKLPQGLDTMVGEH
GTQLSGGQKQRIAVARAILKDPRILLLDEATSALDAESERVVQEALDRIMVNRTTVVVAHRLSTVRNADMIAVI
HQGKIVEKGSHTELLKDPEGAYSQLIRLQEEKKSDETATEEQKMSSIESFKQSSLRKSSLGRSLSKGGSSRGNSS
RHSFNMFGFPAGIDGNVAQDQEDDTTQPKTEPKKVSIFRIAALNKPEIPVLILGSISAAANGVILPIFGILISSVIKA
FFQPPKKLKEDTSFWAIIFMVLGFASIIAYPAQTFFFAIAGCKLVQRIRSMCFEKVVHMEVGWFDEPENSSGTIG
ARLSADAATIRGLVGDSLAQTVQNLSSILAGLIIAFLACWQLAFVVLAMLPLIALNGFLYMKFMKGFSADAKK
MYGEASQVANDAVGSIRTVASFCAEDKVMNMYTKKCEGPMKTGIRQGIVSGIGFGFSFFVLFSSYAASFYVGA
RLVDDGKTTFDSVFRVFFALTMAAMAISQSSSLSPDSSKADVAAASIFAIMDRESKIDPSVESGRVLDNVKGDIE
LRHVSFKYPARPDVQIFQDLCLSIRAGKTVALVGESGSGKSTVIALLQRFYDPDSGEITLDGVEIKSLRLKWLRQ
QTGLVSQEPILFNETIRANIAYGKGGDASESEIVSSAELSNAHGFISGLQQGYDTMVGERGIQLSGGQKQRVAIA
RAIVKDPKVLLLDEATSALDAESERVVQDALDRVMVNRTTIVVAHRLSTIKNADVIAVVKNGVIVEKGKHDTL
INIKDGVYASLVQLHLTAAS

>XP_023640786.1_ABCB4_Cr
MASESGVNGDSNIVEEVSETKRGSKEEGETMKTEKKKKNKEEHDEKTKSVPFYKLFAFADSFDFLLMFLGTLG
SIGNGLGFPIMTLLFGDLIDAFGQNQTDTTDKVSKIALKFVWLGIGTFVAAFLQLSGWMISGERQAARIRSLYLK
TILRQDIAFFDIDTNTGEVVGRMSGDTVLIQDAMGEKVGKAIQLVSTFVGGFVIAFIRGWLLTLVMLSSIPLLVM
AGALLAIVIAKTASRGQTAYAKAAVVVEQTIGSIRTVASFTGEKQAISNYNKHLVSAYKAGVIEGGSTGLGLGT
LFLVIFCSYALAVWYGGKLILDKGYTGGQVLNVIISVLTGSMSLGQASPCLSAFAAGQAAAYKMFETIERKPDI
DSYSTDGKVLDDIKGDIELKDVYFTYPARPEEQIFRGFSLFVSSGTTVALVGQSGSGKSTVVSLIERFYDPQAGE
VLIDGINLKEFQLRWIRSKIGLVSQEPVLFTASIKDNIAYGKEDATIEEIKSAAELANASKFVDKLPQGLDTMVG
EHGTQLSGGQKQRIAVARAILKDPRILLLDEATSALDAESERVVQEALDRIMVNRTTIVVAHRLSTVRNADMIA
VIHQGKIVEKGSHTELLKDTEGAYSQLIRLQEEKKSDENASDEKKMSSIESFKQSSLRKSSLGRSLSKGGSSRGN
SSRHSFNMFGFPAGIDGNDAQDQEESATEPKTKQKKVSIFRIAALNKPEIPVLILGSISAAANGVILPLFGILLSSVI
KAFFQPPKKLKEDTSFWAIIFMVLGFASIIAYPAQTFFFGIAGCKLVQRIRGMCFEKVVHMEVGWFDEPENSSG
TIGARLSADAATIRGLVGDALAQTVQNLSSILAGLIIAFLACWQLSFVVLAMLPLIALNGFLYMKFMTGFSADA
KKMYGEASQVANDAVGSIRTVASFCAEDKVMNMYTKKCEGPMKTGIRQGIVSGIGFGFSFFVLFASYATSFYV
GARLVDDGKTTFDAVFRVFFALTMAAMAISQSSSLSPDSSKADVAAASIFAIMDRESKIDPSVESGRVLDNVRG
DIELRHVSFKYPARPDVQIFQDLCLSIRAGKTVALVGESGSGKSTVIALLQRFYDPDSGEITLDGVEIKSLRLKW
LRQQTGLVSQEPILFNDTIRANIAYGKGGDASESEIVSSAELSNAHGFISGLQQGYDTMVGERGIQLSGGQKQR
VAIARAIVKDPKVLLLDEATSALDAESERVVQDALDRVMVNRTTIVVAHRLSTIKNADVIAVVKNGVIVEKGR
HESLINIQDGVYASLVQLHLTAAS

>XP_013747568.1_ABCB4-l_Bn
MASESGLNGDANIVEEVSETKRSRDEEEKEDKEVKKNNNEDHEKTKTVPFYKLFAFADSFDFLLMTLGTLGSI
GNGLGFPIMTILFGDLVDAFGENQNDSNVADKVSKVSLKFVWLGIGTFAAAFLQLSGWMISGERQAARIRSMY
LKTILRQDIAFFDVDTNTGEVVGRMSGDTVLIQDAMGEKVGKAIQLLATFVGGFVIAFIRGWLLTLVMLSSIPL
LVMAGAGLAIVITRTASRGQTAYAKAAVVVEQTIGSIRTVASFTGEKQAISNYNKHLVTAYKAGVIEGGSTGL
GLGTLFLVFCSYALAVWYGGKLILDKGYTGGQVLNIIISVLTGSMSLGQASPCLTAFAAGQAAAYKMFETIERR
PDIDSYSTDGKVLDDIKGDIELKDVYFTYPARPDEQIFCGFSLFISSGTTVALVGQSGSGKSTVVSLIERFYDPQA
GEVIIDGVNLKEFQLKWIRSKIGLVSQEPVLFTSSIKDNIAYGKEDATLEEIKAAAELANASKFVDKLPQGLDTM
VGEHGTQLSGGQKQRIAVARAILKDPRILLLDEATSALDAESERVVQEALDRIMVNRTTIVVAHRLSTVRNADT
IAVIHQGKIVEKGSHTELLKDPEGAYSQLIRLQEDTKPEETQAGERKMSSIESFKQSSFRNSSLGRSLSKGGSSRG
NSSRHSFNMFGFPAGIEGNDVAQEDQEEDDTTEPKTKPKKVSIRRIAALNKPEIPVLILGTISAAANGVILPIFGILI
ASVIKAFFKPPKELKEDTSFWAIIFMVLGFASVIAYPAQTFFFSIAGCKLVQRIRSMCFEKVVHMEVGWFDESEH

SSGTIGARLSADAAAIRGLVGDALAQMVQNLSSILAGLIIAFLACWQLAFVVLAMLPLIALNGFLYMKFMKGFS
ADAKKMYGEASQVANDAVGSIRTVASFCAEDKVMNMYTKKCEGPMKTGIRQGIVSGIGFGVSFFVLFASYAT
SFYVGAQLVDDGKTTFDSVFRVFFALTMAAIAISQSSSLSPDSSKADIAAASIFGIIDRESKIDPSVESGRVLDTVK
GDIELRHVSFKYPSRPDVQIFQDLCLSIRAGKTVALVGESGSGKSTVIALLQRFYDPDSGEITLDGVEIKTLRLK
WLRQQTGLVSQEPILFNETIRANIAYGKGGDASESEIVSAGELSNAHGFISGLQQGYDTMVGERGIQLSGGQKQ
RVAIARAIVKDPKVLLLDEATSALDAESERVVQDALDRVMVNRTTIVVAHRLSTIKNADVIAVVKNGVIVEKG
KHESLINIKDGVYASLVQLHLSAAS

>XP_018484614.1_P_ABCB4-l_Rs
MASGSGLNRDANIVEEVSETKRSIEEDKEVKKTEKKEDHEKTKTVPFYKLFAFADSFDILLMILGTLGSIGNGLG
FPIMTVLFGDLIDAFGQNQNDSNVSDKVSKVALKFVWLGIGTFAAAFLQLSGWMISGERQAARIRSMYLKTIL
RQDIAFFDVDTNTGEVVGRMSGDTVLIQDAMGEKVGKAIQLLSTFVGGFVIAFLKGWLLTLVMLSSIPLLVMA
GAGLAIVIAKTASRGQTAYANAAVVVEQTIGSIRTVASFTGEKQAISNYNKHLLTAYKAGVIEGGSTGLGLGTL
FLVIFCSYALAVWYGGKLILDKGYTGGQVLNIIISVLTGSMSLGQASPCLSAFAAGQAAAYKMFETIERRPDIDS
YSTDGKVLDDIKGDIELKDVYFTYPARPDEQIFRGFSLFISSGTTVALVGQSGSGKSTVVSLIERFYDPQAGEVLI
DGVNLKEFQLKWIRSKIGLVSQEPVLFTSSIRDNIAYGKEDATTEEIKAAAELANASKFVDKLPQGLDTMVGEH
GTQLSGGQKQRIAVARAILKDPRILLLDEATSALDAESERVVQEALDRIMVNRTTVVVAHRLSTVRNADMIAVI
HQGKIVEKGSHNELLKDPEGAYSQLIRLQEEKKQEENPEGEQKMSSLESFKQSSLRKSSLGRSLSKGGSSRGNS
SRHSFNMFGFPAGIEGNDVAQDQEEGTTEHKTKPKKVSIRRVAALNKPEIPVLILGAISAAANGVILPLFGILIAS
VIKAFFKPPKQLKEDTSFWAIIFMVLGFASIIAYPAQTFFFAIAGCKLVKRIRSMCFEKVVHMEVGWFDESEHSS
GTIGARLSADAAAIRGLVGDALAQMVQNLSSILAGLIIAFLACWQLAFVVLAMLPLIALNGFLYMKFMQGFSA
DAKKMYGEASQLANDAVGSIRTVASFCAEEKVMNMYTKKCEGPMKTGIRQGIVSGIGFGFSFFVLFASYACSF
YVGARLVDDGKTTFDSVFRVFFALTMAAMAISQSSSLSPDSSKADIAAASIFAIIDRESKIDPSVESGRVLDTVK
GDIELRHVSFKYPARPDVQIFQDLCLTIRAGKTVALVGESGSGKSTVIALLQRFYDPDSGEITLDGVEIKSLRLK
WLRQQTGLVSQEPILFNETIRANIAYGKGGDASESEIVSAAELSNAHGFISGLQLGYDTMVGERGIQLSGGQKQ
RVAIARAIVKDPKVLLLDEATSALDAESERVVQDALDRVMVNRTTIVVAHRLSTIKNADVIAVVKNGVIVEKG
KHESLINIKDGVYASLVQLHLSAAS

>XP_013637050.1_P_ABCB4-l_Bo
MAAESGLNGDGNIVEDVSETKRSREEDKEVKKTEKKEEHEKTKTVPFYKLFAFADSFDILLMILGTLGSIGNGL
GFPIMTVLFGDLIDAFGQNQNDSNVSDKVSKVALKFVWLGIGTFAAAFLQLSGWMISGERQAARIRSMYLKTI
LRQDIAFFDVDTNTGEVVGRMSGDTVLIQDAMGEKVGKAIQLLSTFVGGFVIAFLKGWLLTLVMLSSIPLLVM
AGAGLAIVIAKTASRGQTAYANAAVVVEQTIGSIRTVASFTGEKQAISNYNKHLLTAYKAGVIEGGSTGLGLGT
LFLVIFCSYALAVWYGGKLILDKGYTGGQVLNIIISVLTGSMSLGQASPCLSAFAAGQAAAYKMFETIERRPDID
SYSTNGKVMDDIKGDIELKDVYFTYPARPDEQIFRGFSLFISSGTTVALVGQSGSGKSTVVSLIERFYDPQAGEV
LIDGVNLKEFQLKWIRSKIGLVSQEPVLFTSSIKDNIAYGKEDATIEEIKAAAELANASKFVDKLPQGLDTMVGE
HGTQLSGGQKQRIAVARAILKDPRILLLDEATSALDAESERVVQEALDRIMVNRTTVVVAHRLSTVRNADMIA
VIHQGKIVEKGSHTELLKDPEGAYSQLIRLQEEKKSEENPTDEQKLSSIESFKHSSLRKSSLGRSLSKGGSSRGNS
SRHSFNMFGFPAGIEGNDVAQEGTTEAKTKPKKVSIRRVAALNKPEIPVLILGAISAAANGVILPLFGILIASVIK
AFFKPPKELKEDTSFWAIIFMVLGFASIIAYPAQTFFFAIAGCKLVKRIRSMCFEKVVHMEVGWFDESENSSGTI
GARLSADAAAIRGLVGDALAQMVQNLSSILAGLIIAFLACWQLAFVVLAMLPLIALNGFLYMKFMQGFSADA
KKMYGEASQVANDAVGSIRTVASFCAEEKVMNMYAKKCEGPMKTGIRQGIVSGIGFGFSFFVLFASYACSFYV
GARLVDDGKTTFDSVFRVFFALTMAAMAISQSSSLSPDSSKADIAAASIFAIIDRESKIDPSVESGRVLDNVNGDI
ELRHVSFKYPARPDVQIFQDLCLSIRAGKTVALVGESGSGKSTVIALLQRFYDPDSGEITLDGVEIKSLQLKWLR
QQTGLVSQEPILFNETIRANIAYGKGGDASESEIVSAAELSNAHGFISGLQQGYDTMVGERGIQLSGGQKQRVAI
ARAIVKDPKVLLLDEATSALDAESERVVQDALDRVMVNRTTIVVAHRLSTIKNADVIAVVKNGVIVEKGKHES
LINIKDGVYASLVQLHLSAAS

>XP_009142574.1_P_ABCB4_Br
MASESGLNGDANIVEEVSETKRSRDEEEKEDKEVKKNNNEDHEKTKTVPFYKLFAFADSLDFLLMTLGTLGSI
GNGLGFPIMTILFGDLVDAFGENQNDSNVADKVSKVSLKFVWLGIGTFAAAFLQLSGWMISGERQAARIRSMY
LKTILRQDIAFFDVDTNTGEVVGRMSGDTVLIQDAMGEKVGKAIQLLATFVGGFVIAFIRGWLLTLVMLSSIPL
LVMAGAGLAIVIARTASRGQTAYAKAAVVVEQTIGSIRTVASFTGEKQAISNYNKHLVTAYKAGVMEGGSTG
LGLGTLFLVVFCSYALAVWYGGKLILDKGYTGGQVLNIIISVLTGSMSLGQASPCLTAFAAGQAAAYKMFETIE

RRPDIDSYSTDGKVLDDIKGDIELKDVYFTYPARPDEQIFRGFSLFISSGTTVALVGQSGSGKSTVVSLIERFYDP
QAGEVIIDGVNLKEFQLKWIRSKIGLVSQEPVLFTSSIKDNIAYGKEDATLEEIKAAAELANASKFVDKLPQGLD
TMVGEHGTQLSGGQKQRIAVARAILKDPRILLLDEATSALDAESERVVQEALDRIMVNRTTVVVAHRLSTVRN
ADTIAVIHQGKIVEKGSHAELLKDPEGAYSQLIRLQEDKKHEEKKPEELSSIESFKQSSLRKSSLGRSLSKGGSSR
GNSSRHSFNMFGFPSGIEGNDVVQDQEEPKTKPKKVSIRRIAALNKPEIPVLILGTISAAANGVILPIFGILIASVIK
AFFKPPKELKEDTSFWAIIFMVLGFASVIAYPAQTFFFSIAGCKLVQRIRSMCFEKVVHMEVGWFDESEHSSGTI
GARLSADAAAIRGLVGDALAQMVQNLSSILAGLIIAFLACWQLAFVVLAMLPLIALNGFLYMKFMKGFSADA
KKMYGEASQVANDAVGSIRTVASFCAEDKVMNMYTKKCEGPMKTGIRQGIVSGIGFGVSFFVLFASYATSFY
VGAQLVDDGKTTFDSVFRVFFALTMAAIAISQSSSLSPDSSKADIAAASIFGIIDRESKIDPSVESGRVLDTVKGDI
ELRHVSFKYPSRPDVQIFQDLCLSIRAGKTVALVGESGSGKSTVIALLQRFYDPDSGEITLDGVEIKTLRLKWLR
QQTGLVSQEPILFNETIRANIAYGKGGDASESEIVSAAELSNAHGFISGLQQGYDTMVGERGIQLSGGQKQRVAI
ARAIVKDPKVLLLDEATSALDAESERVVQDALDRVMVNRTTIVVAHRLSTIKNADVIAVVKNGVIVEKGKHES
LINIKDGVYASLVQLHLSAAS

>XP_009142463.1_P_ABCB4_Br
MASESGLNGDGNIVEDVSETKRSREEDKEVKKTEKKEEHEKTKTVPFYKLFAFADSFDILLMILGTLGSIGNGL
GFPIMTVLFGDLIDAFGQNQNDSNVSDKVSKVALKFVWLGIGTFAAAFLQLSGWMISGERQAARIRSMYLKTI
LRQDIAFFDVDTNTGEVVGRMSGDTVLIQDAMGEKVGKAIQLLSTFVGGFVIAFLKGWLLTLVMLSSIPLLVM
AGAGLAIVIAKTASRGQTAYANAAVVVEQTIGSIRTVASFTGEKQAINNYNKHLLTAYKAGVIEGGSTGLGLG
TLFLVIFCSYALAVWYGGKLILDKGYTGGQVLNIIISVLTGSMSLGQASPCLSAFAAGQAAAYKMFETIERRPDI
DSYSTNGKVLDDIKGDIELKDVYFTYPARPDEQIFHGFSLFISSGTTVALVGQSGSGKSTVVSLIERFYDPQAGEI
IIDGVNLKEFQLKWIRSKIGLVSQEPVLFTSSIKDNIAYGKEDATIEEIKAAAELANASKFVDKLPQGLDTMVGE
HGTQLSGGQKQRIAVARAILKDPRILLLDEATSALDAESERVVQEALDRIMVNRTTVVVAHRLSTVRNADMIA
VIHQGKIVEKGSHTELLKDPEGAYSQLIRLQEEKKGEENPTDEQKMSSIESFKHSSLRKSSLGRSLSKGGSSRGN
SSRHSFNMFGFPSGVEGNDVTQDQEEGTTEAKTKPKKVSIRRVAALNKPEIPVLILGAISAAANGVILPLFGILIA
SVIKAFFKPPKELKEDTSFWAIIFMALGFASIIAYPAQTFFFAIAGCKLVKRIRSMCFEKVVHMEVGWFDESEHS
SGTIGARLSADAAAIRGLVGDALAQMVQNLSSILAGLIIAFLACWQLAFVVLAMLPLIALNGFLYMKFMQGFS
ADAKKMYGEASQVANDAVGSIRTVASFCAEEKVMNMYTKKCEGPMKTGIRQGIVSGIGFGFSFFVLFASYAC
SFYVGARLVDDGKTTFDSVFRVFFALTMAAMAISQSSSLSPDSSKADIAAASIFAIIDRESKIDPSVESGRVLDTV
KGDIELRHVSFKYPARPDVQIFQDLCLSIRAGKTVALVGESGSGKSTVIALLQRFYDPDSGEITLDGVEIKSLRLK
WLRQQTGLVSQEPILFNETIRANIAYGKGGDASESEIVSAAELSNAHGFISGLQQGYDTMVGERGIQLSGGQKQ
RVAIARAIVKDPKVLLLDEATSALDAESERVVQDALDRVMVNRTTIVVAHRLSTIKNADVIAVVKNGVIVEKG
KHESLINIKDGVYASLVQLHLSAAS

>XP_017980794.1_P_ABCB4_Tc
MAAENGFNGHTDLHEASTSKSQEEPEKVSGVNGENQDSESSKGDEKTNKVPFYKLFAFADSTDILLMIIGTIGA
VGNGVCMPLMTILFGDLVDAFGENQSNDKVVDVVSEVALKFVYLAVGAAAAAFLQVSCWMVTGERQAARI
RGLYLKTILRQDVAFFDVETNTGEVVGRMSGDTVLIQDAMGEKVGKFLQLISTFFGGFIIAFIKGWLLTLVMLS
SIPLLVISGAVMAILISKMASRGQTAYAKAATVVEQTIGSIRTVASFTGEKQAISNYNKFLVTAYRSGVHEGAA
AGLGLGVVMLIIFCSYALAVWFGGKMILEKGYTGGQVLNVIIAVLTGSMSLGQASPCMSAFAAGQAAAFKMF
ETIKRKPEIDSYDTRGKIFEDIRGDIELRDVNFSYPARPDEQIFSGFSLAISSGTTAALVGQSGSGKSTVISLIERFY
DPQAGEVLIDGINLKDFQLRWIRGKIGLVSQEPVLFTSSIRDNIAYGKENATTEEIRAAAELANASKFIDKLPQGL
DTMVGEHGTQLSGGQKQRVAIARAILKDPRILLLDEATSALDAESERVVQEALDRIMGNRTTVIVAHRLSTVR
NADMIAVIHRGKMVEKGSHSELLKDPEGAYSQLIRLQEVNKESEHVADVSDINPESFRQSSLRRSLKRSISRGSS
MGNSSRHSFSVSFGLPTGMNVTDPAMLDTEDPAELSSERAPEVPIRRLAYLNKPEIPVILLGTVAAAANGVILPI
FGILISSVIQTFFKPPDELKKDSRFWALIFMVLGLASLLALPARTYFFSIAGCKLIQRIRSMCFEKVVHMEVGWFD
EPAHSSGSVGARLSADAATIRALVGDALAQMVSNLASAVAGLVIAFVASWQLAFIILALIPLIGVNGYVQVKF
MKGFSADAKMMYEEASQVANDAVGSIRTVASFCAEEKVMQLYKKKCEGPMKTGIRQGLISGSGFGLSFFLLF
CVYATSFYAGAQLVKHGHATFSDVFRVFFALTMAAVGISQSSSFAPDSSKAKTAAASIFAIIDRKSKIDPSDESG
TTLENVKGDIEFRHVSFKYPLRPDIQILRDLSLSIHAGKTVALVGESGSGKSTVISLLQRFYDPDSGRITLDGVEIQ
KLQLKWLRQQMGLVSQEPVLFNDTIRANIAYGKGGNATEAEILAASELANAHKFISSLQQGYDTVVGERGVQ
MSGGQKQRIAIARAIVKSPKILLLDEATSALDAESERVVQDALDRVMVNRTTVVVAHRLSTIKNADVIAVVKN
GVIVEKGKHDALINIKDGFYASLVSLHMSASTA

>XP_013613919.1_P_ABCB4-l_Bo
MASESGLNGDSNIVEEVSETKRSRDVEEDEEVKKTEKNNNTEEHEKTKTVPFYKLFAFADSFDFLLMTLGTLG
SIGNGLGFPIMTILFGDLVDAFGENQTDSNVADKVSKVALKFVWLGIGTLAAAFLQLSGWMISGERQAARIRS
MYLKTILRQDIAFFDVDTNTGEVVGRMSGDTVLIQDAMGEKVGKAIQLLATFVGGFVIAFIRGWLLTLVMLSSI
PLLVMAGAGLAIVIARTASRGQTAYAKAAVVVEQTIGSIRTVASFTGEKQAISNYNKHLVTAYKAGVIEGGST
GLGLGTLFLVVFCSYALAVWYGGKLILDKGYTGGQVLNIIISVLTGSMSLGQASPCLTAFAAGQAAAYKMFET
IERRPDIDSYSTDGKVLDDIKGDIELKDVYFTYPARPDEQIFRGFSLFISSGTTVALVGQSGSGKSTVVSLIERFYD
PQAGEVLIDGVNLKEFQLKWIRSKIGLVSQEPVLFTSSIKDNIAYGKEDATLEEIKAAAELANASKFVDKLPQGL
DTMVGEHGTQLSGGQKQRIAVARAILKDPRILLLDEATSALDAESERVVQEALDRIMVNRTTVVVAHRLSTVR
NADTIAVIHQGKIVEKGSHTELLKDPEGAYSQLIRLQEEKKPEETQAGERKMSSIESFKQSSFRKSSLGRSLSKG
GSSRGNSSRHSFNMFGFPSGIEGNDVVQDQEEDDTTEPKTKPKKVSIRRIAALNKPEIPVLILGTISAAANGVILPI
FGILIASVIKAFFKPPKELKEDTSFWAIIFMVLGFASVIAYPAQTFFFSIAGCKLVQRIRSMCFEKVVHMEVGWFD
ESEHSSGTIGARLSADAAAIRGLVGDALAQMVQNLSSILAGLIIAFLACWQLAFVVLAMLPLIAVNGFLYMKF
MKGFSADAKKMYGEASQVANDAVGSIRTVASFCAEDKVMNMYTKKCEGPMKTGIRQGIVSGIGFSVSFFVLF
ASYGTSFYVGARLVDDNKTTFDSVFRVFFALTMTAIAISQSSSLSPDSSKADIAAASIFGIIDRESKIDPSVESGRV
LDTVKGDIELRPVSFKYPSRPDVQIFQDLCLSIRAGKTVALVGESGSGKSTVIALLQRFYDPDSGEITLDGAEMK
TLRLKWLRQQTGLVSQEPILFNETIRDNIAYGKGGDASESEIVSAAELSNAHGFISGLQQGYDTMVGERGIELSG
GQKQRMAIARAIVKDPKVLLLDEATSALDAESERVVQDALDRVMVHRLRLWWLTGYRRLITRM

>XP_021279616.1_ABCB4-l_Hu
MAAENGFNGHTDLHEASTSKSQEEPEKVSGVNGENQDSESSKGDEKTSTVPFYKLFAFADSTDILLMIIGTIGA
VGNGVCMPLMTILFGDLVDAFGENQSNNKVVDVVSEIALKFVYLAVGSAAAAFLQVSCWMVTGERQAARIR
GLYLKTILRQDVAFFDVETNTGEVVGRMSGDTVLIQDAMGEKVGKFLQLISTFFGGFVIAFIKGWLLTLVMLSS
IPLLAISGGVMAILISKMASRGQTAYAKAATVVEQTIGSIRTVASFTGEKQAISNYNKFLVTAYRSGVHEGTAA
GLGLGVVMLIIFCSYALAIWFGGKMILEKGYTGGQVLNVIIAVLTGSMSLGQASPCMSAFAAGQAAAFKMFET
IKRKPEIDSYDTRGKIFEDIRGDIELRDVYFSYPARPDEQIFSGFSLSISSGTTAALVGQSGSGKSTVISLIERFYDP
QAGEVLIDGINLKDFQLRWIRGKIGLVSQEPVLFTSSIRDNIAYGKENATTEEIRAAAELANASKFIDKLPQGLDT
MVGEHGTQLSGGQKQRVAIARAILKDPRILLLDEATSALDAESERVVQEALDRIMGNRTTVIVAHRLSTVRNA
DMIAVIHRGKMVEKGSHSELLEDPEGAYSQLIRLQEVNKESEHVADVSDINPESFRQSSLRRSLRRSISRGSSIGN
SSRHSFSVSFGLPTGMNVTDPATLDTADHAELSSERAPEVPIRRLAYLNKPEIPVILLGTIAAAVNGVILPIFGILIS
SVIKTFFKPPDELKKDSRFWALIFMVLGLASLLSSPARTYFFAIAGCKLIQRIRSMCFEKVVHMEVGWFDEPDHS
SGSVGARLSADAATIRALVGDALAQMVSNIASAVAGLVIAFVASWQLAFIILALIPLIGVNGYVQVKFMKGFSA
DAKLMYEEASQVANDAVGSIRTVASFCAEEKVMQLYKKKCEGPMKTGIRQGLISGSGFGLSFFLLFCVYATSF
YAGAQLVKHGHATFTDVFQVFFALTMAAVGISQSSSFAPDSSKAKTAAASIFAIIDRKSKIDPSDESGTTLENVK
GDIEFRHVSFKYPLRPDVQILRDLSLSIHAGKTVALVGESGSGKSTVISLLQRFYDPDSGRITLDGVEIQKLQLK
WLRQQMGLVSQEPVLFNDTIRANIAYGKGGNATEAEILAASELANAHKFISALQQGYDTVVGERGVQMSGGQ
KQRIAIARAIVKSPKILLLDEATSALDAESERVVQDALDRVMVNRTTVVVAHRLSTIKNADVIAVVKNGVIVEK
GKHDALINIKDGFYASLVALHMSASTA

>XP_011016204.1_P_ABCB21-l_Pe
MAGENGRSGDKSVDEASTSKSLEVEEKSSGGRGDQQEPVKSKGDEETKTVPFPKLFSFADSTDILLMILGTIGA
VGNGASFPIMSILFGDLVNSFGKNQNNKDVVDSVTKVALNFVYLGIGSAVASFLQVACWMVTGERQAARIRG
TYLKTILKQDVAFFDKETNTGEVVGRMSGDTVLIQDAMGEKVGKFIQLVSTFIGGFIVAFVKGWLLTLVMLSSI
PLLVIAGAGLAIIIARMASRGQTAYAKAAIVVEQAIGSIRTVASFTGEKQAISNYKKFLATAYNSGVQEGFTAGL
GLGIVMLFIFCSYALAIWFGGKMILEKGYTGGDVLNVIVAVLTGSMSLGQASPCMTAFAAGQAAAYKMFETIN
RKPEIDSSDTRGKILDDISGDVELRDVYFTYPARPDEQIFSGFSLFIPSGTTTALVGQSGSGKSTVISLIERFYDPQ
AGEVLIDGTNLKEFQLKWIREKIGLVSQEPVLFASSIKDNIAYGKDGATTDEIRAATELANAAKFIDKLPQGIDT
MVGEHGTQLSGGQKQRIAIARAILKDPRILLLDEATSALDAESERIVQEALDRIMVNRTTVIVAHRLSTVRNAD
MIAVIYRGKMVEKGSHSELLKDPEGAYSQLIRLQEVNKESKQETEDPKKSALSAESLRQSSQRISLKRSISRGSS
GVGHSSRNSLSVSFGLPTGLNVPDNPTSELEVSTQTQQAPDVPISRLAYLNKPEVPVLIAGSIAAILNGVIFPIYGL
LLSSVIKTFFEPPDELRKDSKFWALMFMTLGLASFVVYPTQTYLFSVAGCKLIQRIRSMCFEKVVHMEVGWFD
DPEHSSGAIGARLSADAATVRALVGDSLSQLVQNIASAVAGLVIAFTACWQLAFVILVLLPLIGLNGFVQIKFM
KGFSADAKKMYEEASQVANDAVGSIRTVASFCAEEKVMQLYRRKCEGPMRTGIRQGMISGTGFGVSFFLLFSV
YATTFYVGAQLVRHGKTTFTEVFRVFFALTMAAIGISQSSSFAPDSSKAKGAAASIFAIIDRKSKIDPSDESGRTL

DNVKGEIELRHISFKYPSRPDIEIFRDLSLAIHSGKTVALVGESGSGKSTVISLLQRFYDPDSGHITLDGIDIQSLQL
KWLRQQMGLVSQEPVLFNETIRANIAYGKEGNATEVEILAASELANAHKFISGLQQGYDTVVGERGTQLSGGQ
KQRVAIARAMVKSPKILLLDEATSALDAESERVVQDALDRVMVSRTTVVVAHRLSTIKNADVIAVVKNGVIVE
KGKHEALIHIKDGFYASLVALHMSASTS

>XP_006386686.2_ABCB11_X1_Pt
MAVENGRSRDKSMDEASTSKSQEVEEKSSAGNGDQQKQEKSEGDEETKTVPFIKLFSFADSKDIFLMILGTVG
AIGNGASMPIMSILFGDLINSFGKNQNNKDVVDLVSKVSLKFVYLGVGSAVGSFLQVACWMVTGERQAARIR
GTYLKTILRQDVAFFDKETNSGEVVGRMSGDTVLIQDAMGEKVGKFIQLVSTFIGGFIISFIKGWLLTLVMLSSI
PLLVIAGAGLSIMIARMASRGQTAYSKAASVVEQTIGSIRTVASFTGEKQAISNYKKFLVTAYNSGVQEGLAAG
VGLGIVMLVVFCSYALAVWFGGRMILEKGYTGGDVINVIVAVLTGSMSLGQASPCMSAFASGQAAAYKMFE
AINRKPEIDASDTRGKILDDIRGDIELRDVYFNYPARPDEQIFSGFSLFIPSGSTAALVGQSGSGKSTVISLIERFYD
PQAGEVLIDGINLKEFQLKWIREKIGLVSQEPVLFTSSIKDNIAYGKDMATTEEIRAAAELANAAKFIDKLPQGID
TMVGEHGTQLSGGQKQRIAIARAILKDPRILLLDEATSALDAESERIVQEALDRIMVNRTTVIVAHRLSTVRNA
DMIAVIYRGKMVEKGSHSELLKDPEGAYSQLIRLQEVNKESEQEADDQKKSDISTESLRHSSQKISLKRSISRGS
SDFGNSSRRSFSVTFGLPTGFNAPDNYTEELEASPQKQQTPDVPISRLVYLNKPEVPVLIAGAIAAIINGVIFPIFGI
LISRVIKTFFEPPHELRKDSKFWALMFMTLGLASFVVYPSQTYLFSVAGCKLIQRIRSMCFEKMVHMEVGWFD
EPEHSSGAIGARLSADAATVRGLVGDSLSQLVQNIASAVAGLVIAFVACWQLAFVILVLLPLIGLNGFIQMKFL
KGFSSDAKKMYEEASQVANDAVGSIRTVASFCAEEKVMQLYRKKCEGPMRTGIRQGLISGAGFGVSFFLLFSV
YATSFYVGAQLVQHGKTTFADVFQVFFALTMAAIGISQSSSFAPDSSKAKAAAASIFSIIDRKSQIDSSDESGTTL
DNVKGEIELRHIGFKYPARPDIEIFRDLSLAIHSGKTVALVGESGSGKSTVISLLQRFYDPHSGHITLDGIDIKSLQ
LKWLRQQMGLVSQEPVLFNETIRANIAYGKEGDATEAEILAASELANAHKFISSLQQGYDTVVGERGIQLSGG
QKQRVAIARAIVKSPKILLLDEATSALDAESERVVQDALDRVMVNRTTVVVAHRLSTIKNADVIAVVKNGVIV
EKGKHETLIHIKDGFYASLVALHMSASTS

>XP_022757720.1_ABCB21-l_Dz
MGSENGLDGCTDLPEASTSKSREEPEKVSGVNGENQDEKTNTVSFYKLFGFADSMDILLMIIGTIGAIGNGLCM
PLMTILFGDLVDSFGNNQSNDRIVHVVSKVALRFIYLAVGAAAAAFLQVTCWMVTGERQAARIRGLYLQTILR
QDVAFFDVETNTGEVVGRMSGDTVLIQDAMGEKVGKFIQLVSTFFGGFVIAFIRGWLLTLVMLTSIPLLVISGG
VMALLISKMASRGQSAYAKAAIVVEQTIGSIRTVASFTGEKQAISNYNKFLVISYKSGVHEGAVAGLGLGIVLLI
IFCSYALAVWFGGKMILERGYSGGQVLNVIIAVLTGSMSLGQASPCMSAFAAGQAAAFKMFETIKRKPEIDSY
DAKGKVLEDIRGDVELRDVYFSYPARPGEQIFSGFSLSIPSGTTAALVGQSGSGKSTVISLIERFYDPQAGEVFID
GINLKEFQLRWIRGKIGLVSQEPVLFTSSIRDNIAYGKEGATTEEIRAAAELANAAKFIDKLPQGLDTMVGEHGT
QLSGGQKQRVAIARAILKDPRILLLDEATSALDAESERVVQEALDRIMGNRTTVIVAHRLSTVRNADMIAVIHR
GKMVEKGSHSELLMDPEGAYSQLIRLQEVNKETEQVADVADVTPESFRQSSLRESLKRSISRGSSIGNSSRHSFS
VSFGLPTGMNVTDPAMVNGEDAAEQPSEQAPEVPIWRLAYLNKPEIPVILLGTIFAAVNGVILPIFGVLISNVIKT
FFKPPDELKKDSRFWALIFMALGLASLLASPARTYFFSVAGFKLIQRIRSMCFEKVVRMEVGWFDEPNNSSGSI
GARLSADAALIRALVGDALAQMVSNIASAVAGLVIAFVASWQLAFVILALIPLIGVNGYIQVKSMKGFSADAK
MMYEEASQVANDAVGSIRTVASFCAEEKVMQLYKKKCEGPMKAGIRQGFISGSGFGLSFFLLFSVYATSFYAG
AQLVEHGHATFSDVFQVFFALTMAAVGISQSSSLAPDSSKAKNAAASIFAVIDRQSKIDPSDESGMTLENVKGEI
ELRHVSFKYPSRPDIQILQDLSLSIDAGKTVALVGESGCGKSTVISLLQRFYDPDSGRITLDGVDIQKLQLKWWR
QQMGLVSQEPVLFNDTIRANIAYGKGGNATEAEILAASELANAHKFISALQQGYDTVVGERGVQLSGGQKQR
VAIARAIVKSPKILLLDEATSALDAESEKVVQDALDRVMVNRTTVVVAHRLSTIKNADVIAVVKNCVIVEKGK
HDTLINIRDGFYASLVALHMSASTA

>XP_021612220.1_ABCB11-l_Me
MAEENGLNGVAKTHEASTSKTHEEKSAINGNSQETEKSKGDEKTNTVPFHKLFSFADSLDILLMIVGTIGAVGN
GISLPLMTIFLGDTINAFGENQNKDVVHVVSKVSLKFVYLAVGSAVASFLQVACWIVTGERQAARIRGLYLQTI
LRQDIAFFDKETNTGEVIGRMSGDTVLIQDAMGEKVGKFLQLVSTFFGGFVVAFIKGWLLTLVLLSSIPLLVLA
GAAMSITIAKMASRGQTAYAKAASVVEQTIGSIRTVASFTGEKQAISNYKKFLVTAYNSGVHEGLATGLGLGV
VMLIVFCSYALAIWFGGKMILEKGYSGGNVINVIIAVLSGSMSLGQASPCMSAFAAGQAAAYKMFETISRKPEI
DAYDTRGKKLDDIRGDIELRDIYFSYPARPDEQIFSGFSLSIPSGTTAALVGQSGSGKSTVVSLIERFYDPQAGEV
LIDGVNLKEFQLKWIREKIGLVSQEPALFTASIRDNIAYGKDGATIEEIRAAAELANAAKFIDKLPQGLDTMAGE
HGTQLSGGQKQRIAIARAILKDPRILLLDEATSALDAESERIVQEALDRIMVNRTTVIVAHRLSTIRNADVIAVIH

RGKLVEKGSHSELLSDPEGAYSQLIRLQEVNKGSEHAAENHKRSDLSSESFRQSSQKISLQRSISRGSSGVGNSS
RHSFSAPFGLPTGINVAENSQEETEVSPSQEKAPEVPISRLAYLNKPEIPVLTLATIAASLNGVIFPIFGILLSRVIKS
FFDPTPHELRKDTKFWAIIFMILGVASFLVLPSQFYFFGVAGNRLIQRIRTICFEKVVHMEVGWFDDPQHSSGAI
GARLSADAALVRALVGDALAQLVQNIATAVAGLVIAFTASWQLAFIILALIPLIGVNGYVQVKFMQGFSADAK
MMYEEASQVANDAVGSIRTVASFCAEEKVMQMYKKKCEGPLKTGVRQGLISGIGFGVSFFLLFSVYATSFYA
GAQLVKHGKTTFSDVFQVFFALTMTALGISQSSSFAPDSSKAKNAAASIFSIIDRKSKIDPSDESGMILENVRGEI
ELRHISFKYPSRPDIQIFRDLSLAIHSGKTVALVGESGSGKSTVISLLQRFYDPDSGHITLDGVEIQRLQVKWLRQ
QMGLVSQEPVLFNDTIRANIAYGKDEDATEAEILAASEMANAHKFISSLQQGYDTIVGERGVQLSGGQKQRVA
IARAIIKSPKILLLDEATSALDAESERVVQDALDRVMVNRTTVVVAHRLSTIKNADVIAVVKNGVVVEKGKHE
TLINIKDGFYASLVALHMTASTA

>XP_024441356.1_ABCB11_Pt
MAVENGRNGDKSMDEASTSKSLEVEEKSSGGRGDQQEPVKSKGDEETKTVPFLKLFSFADSTDILLMILGTIGA
VGNGASFPIMSILFGDLVNSFGQNQNNKDVVDSVTKVALNFVYLGIGSAVAAFLQVACWMVTGERQAARIRG
TYLKTILKQDVAFFDKETNTGEVVGRMSGDTVLIQDAMGEKVGKFIQLVSTFIGGFIIAFVKGWLLTLVMLSSIP
LLVIAGAGLAIIIARMASRGQTAYAKAATVVEQAIGSIRTVASFTGEKQAISNYKKFLATAYSSGVQEGFTAGL
GLGIVMLLIFCSYALAIWFGGKMILEKGYNGGDVINVIVAVLTGSMSLGQASPCMSAFAAGQAAAYKMFETIN
RKPEIDSSDTSGKILDDISGDVELRDVYFTYPARPDEQIFAGFSLFIPSGTTTALVGQSGSGKSTVISLIERFYDPQ
AGEVLIDGTNLKEFQLKWIREKIGLVSQEPVLFASSIKDNIAYGKDGATTEEIRAATELANAAKFIDKLPQGIDT
MVGEHGTQLSGGQKQRIAIARAILKDPRILLLDEATSALDAESERIVQEALDRIMVNRTTVIVAHRLSTVINAD
MIAVIYRGKMVEKGSHSELLKDPEGAYSQLIRLQEVNKESKQETEDPKKSALSAESLRQSSQRISLKRSISRGSS
GVGHSSRNSLSVSFGLPTGFNVPDNPTSELEVSPQKQQTPDVPISRLAYLNKPEVPVLIAGSIAAILNGVIFPIYGL
LLSSVIKTFFEPPDELRKDSKFWALMFMTLGLASFVVYPTQTYLFSVAGCKLIQRIRSMCFEKVVHMEVGWFD
EPEHSSGAIGARLSADAATVRALVGDSLSQLVQNIASAVAGLVIAFTASWQLALVILVLLPLIGLNGFVQIKFM
KGFSADAKKMYEEASQVANDAVGSIRTVASFCAEEKVMQLYRRKCEGPMRTGIRQGMISGTGFGVSFFLLFSV
YATTFYVGAQLVRHGKTNFADVFRVFFALTMAAIGISQSSSFAPDSSKAKGAAASIFAIIDRKSKIDPSDESGTTL
DNVKGEIELRHISFKYPSRPDIEIFRDLSLAIHSGKTVAVVGESGSGKSTVISLLQRFYDPDSGHITLDGIDIQSLQL
KWLRQQMGLVSQEPVLFNETIRANIAYGKEGNATEAEILAASELANAHKFISGLQQGYDTVVGERGTQLSGGQ
KQRVAIARAMVKSPKILLLDEATSALDAESERVVQDALDRVMVSRTTVVVAHRLSTIKNADVIAVVKNGVIVE
KGKHETLIHIKDGFYASLVALHMSASTS

>XP_022770726.1_ABCB21-l_X1_Dz
MASENGLKGHTDLHEASTSKSQEEPEKVSDVKGENQDSESSKGDEKTKTVPFYKLFAFADSKDIWLMVIGTIG
AIGNGLCMPLMTILFGDLIDAFGQNQSNDRVVNVVSKVALRFVYLAVGAAVAAFLQVTCWMVTGERQAERIR
GLYLKTILRQDIAFFDVETNTGEVIGRMSGDTVLIQDAMGEKVGKFIQLVSTFFGGFVIAFIRGWLLTLVMLSSI
PLLVISGGVMALLISKMATRGQSAYAKAATVVEQTIGSIRTVASFTGEKQAISNYNKFLVTAYKSGVHEGTAA
GLGLGMVMLIIFCSYALAVWFGGRMILERGYSGGQVINVIIAVLTGSMSLGQASPCMSAFAAGQAAAFKMFET
IKRKPEIDSYDMRGKVLEDIRGDVELRDVYFSYPARPEEQIFSGFSLSIPSGTTAALVGQSGSGKSTVISLIERFYD
PQAGEVLIDGINLKEFQLRWIRGKIGLVSQEPVLFTSSIRDNIAYGKEGATTEEIRAAAELANAAKFIDKLPQGLD
TMVGEHGTQLSGGQKQRVAIARAILKDPRILLLDEATSALDAESERVVQEALDRIMGNRTTVIVAHRLSTVRN
ADMIAVIHRGKMVEKGSHSELLKDPEGAYSQLIRLQEVNKESEQVADVSDITPESFRQSSLRRSLKRSNSRGSS
MGNSSRHSFSVSFGLPTGMNVTEPAMVDTEDPAKRPSEKAPDVPIRRLAYLNKPEIPVILLGTIAAAINGVILPIF
GILISNVIQSFFKPPDELKKDSRFWALIFMALGLASFLASPARTYFFSIAGCKLIQRVRSMCFEKVVRMEVGWFD
EPENSSGSIGARLSADAATIRALVGDALAQMVSNIASAVAGLVIAFVASWQLAFIILAIIPLIGINGYVQVKFMK
GFSADAKMMYEEASQVANDAVGSIRTVASFCAEEKVMQLYKKKCEGPMKTGIRQGLISGSGFGLSFFFLFSVY
ATSFYAGAQLVEHGQTTFPDVFQVFFALTMATVGISQSSSFAPDSSKAKSAAASIFAIIDRESKIDPSDESGMTLE
NVKGDIELRHISFKYPLRPDIQILRDLSLSIHVGKTVALVGESGSGKSTVISLLQRFYDPDSGRITLDGVEIQKLQL
KWLRQQMGLVSQEPVLFNDTIRANIAYGKGGNATEAEILAASELANAHKFISSLQQGYDTVVGERGVQLSGG
QKQRVAIARAIVKSPKILLLDEATSALDAESERVVQDALDRVMVNRTTVVVAHRLSTIKNADVIAVVKNGVIV
EKGKHDSLINIKDGFYASLVALHTSASTA

>XP_012475027.1_P_ABCB11-l_Gr
MDSENGFSDPTDLHEASTSKIQEEPDKVSGSNGDNLESKKVDDEKTNTVPFYKLFAFADSRDTLLMIVGTIGAV
GNGICMPLMTILFGDLIDAFGENQNDDRVVDVVSRVALRFVYLAVGAGVAAFLQVTCWMVTGERQAARIRG

LYLKTILRQDVAFFDVETNTGEVVGRMSGDTVLIQDAMGEKVGKFIQLVSTFIGGFVIAFVQGWLLTLVMLSSI
PPIVISGGVMALIVSKMASRGQSAYAKAASVVEQTIGSIRTVASFTGEKQAISNYNKFLGAAYTSGVHEGFAAG
LGLGVLFLVIFCSYSLAIWFGARMVLDRGYSGGDVINVIFAVLTGSMSLGQASPCVTAFAAGQAAAFKMFETI
KRKPEIDSYDTRGKVLEDIRGDIELRDVYFTYPARPDEQIFSGFSLSIQNGTTVALVGQSGSGKSTVISLIERFYDP
HAGEVLIDGINLKEFQLRWIRGKIGLVSQEPVLFTSSIRDNIAYGKEGATTEEIRAAAELANASKFIDKLPQGLDT
MVGEHGTQLSGGQKQRVAIARAILKDPRILLLDEATSALDAESERVVQEALDRIMGNRTTVIVAHRLSTVRNA
DMIAVIHRGKMVEKGSHSELLQDHEGAYSQLIRLQEVNKESEQATESSDIASESFRRSSLKKSLKRSISRGSSMG
NSNRHSFSASFGLPTGMNAADLAMADAENPAELPSEKAPKVSVRRLAYLNKPEIPVILLGTIAAAANGVIFPIFG
ILISSVIDTFFKPPHELREDSRFWALIFLALGAAAFVVCPAQNYFFSIAGSKLIQRIRSMCFEKVVRMEVGWFDEP
ENSSGAIGARLSADAASIRALVGDALAQLVQNTSSAISGLVIAFVACWQLAFIVLVLLPLIAINGYIQVKFMKGF
SADAKLMYEEASQVANDAVGSIRTVASFCAEEKVMQLYKKKCEGPMKTGIKQGLISGTGFGVSFFFLFSVYAT
SFYAGAQLVEHGYTTFRDVFQVFFALTMAAIGISQSSSFAPDSGKAKSAAASIFAIIDRESKIDPSDESGMKLEN
VKGDIELHHVSFKYPSRPDIQILRDLSLSIRSGKTVALVGESGSGKSTVISLLQRFYDPDSGRISLDGVDIQKLQL
KWLRQQMGLVSQEPVLFNDTIRANIAYGKGGNATEAEILAASELANAHKFISSLQQGYDTVVGERGVQMSGG
QKQRIAIARAIVKSPQILLLDEATSALDAESERVVQAALDRVVVNRTTVVVAHRLSTIKNADVIAVVKNGVVV
EKGKHDTLINIKDGFYASLVALHMSASTA

>XP_016693930.1_P_ABCB11-l_Gh
MDSENGFSDPTDLHEASTSKIQEEPNKVSGSNGDNLESKKVDDEKTNTVPFYKLFAFADSRDTLLMIVGTIGAV
GNGICMPLMTILFGDLIDAFGENQNDDRVVDVVSRVALRFVYLAVGAGVAAFLQVTCWMVTGERQAARIRG
LYLKTILRQDVAFFDVETNTGEVVGRMSGDTVLIQDAMGEKVGKFIQLVSTFIGGFVIAFVQGWLLTLVMLSSI
PPIVISGGVMALIVSKMASRGQNAYAKAASVVEQTIGSIRTVASFTGEKQAISNYNKFLGAAYTSGVHEGFAAG
LGLGVLFLVIFCSYSLAIWFGARMVLDRGYSGGDVINVIFAVLTGSMSLGQASPCVTAFAAGQAAAFKMFETI
KRKPEIDSYDTRGKVLEDIRGDIELRDVYFTYPARPDEQIFSGFSLSIQNGTTVALVGQSGSGKSTVISLIERFYDP
HAGEVLIDGINLKEFQLRWIRGKIGLVSQEPVLFTSSIRDNIAYGKEGATTEEIRAAAELANASKFIDKLPQGLDT
MVGEHGTQLSGGQKQRVAIARAILKDPRILLLDEATSALDAESERVVQEALDRIMGNRTTVIVAHRLSTVRNA
DMIAVIHRGKMVEKGSHSELLQDHEGAYSQLIRLQEVNKESEQATESSDIASESFRRSSLKKSLKRSISRGSSMG
NSNRHSFSASFGLPTGMNAADLAMADAETPAGXTIRKSFRRLAYLNKPEIPVILLGTIAAAANGVIFPIFGILISS
VIDTFFKPPHELREDSRFWALIFLALGAAAFVVCPAQNYFFSIAGSKLIQRIRSMCFEKVVRMEVGWFDEPENSS
GAIGARLSADAASIRALVGDALAQLVQNTSSAISGLVIAFVACWQLAFIVLVLLPLIAINGYIQVKFMKGFSADA
KLMYEEASQVANDAVGSIRTVASFCAEEKVMQLYKKKCEGPMKTGIKQGLISGTGFGVSFFFLFSVYATSFYA
GAQLVEHGYTTFRDVFQVFFALTMAAIGISQSSSFAPDSGKAKSAAASIFAIIDRESKIDPSDESGMKLENVKGDI
ELHHVSFKYPSRPDIQILRDLSLSIRSGKTVALVGESGSGKSTVISLLQRFYDPDSGRISLDGVDIQKLQLKWLRQ
QMGLVSQEPVLFNDTIRANIAYGKGGNATEAEILASSELANAHKFISSLQQGYDTVVGERGVQMSGGQKQRIA
IARAIVKSPQILLLDEATSALDAESERVVQAALDRVVVNRTTVVVAHRLSTIKNADVIAVVKNGVVVEKGKHD
TLINIKDGFYASLVALHMSASTA

>XP_011005954.1_P_ABCB4-l_Pe
MAAENGRSRDKSMDEASTSKGQEVDEKSSAGNGDQQKQKKSEGDEETKTVPFIKLFSFADTKDIFLMILGTVG
AIGNGASLPIMSILFGDLINAFGKNQNNKDVVDLVSKVSLKFVYLGVGSAVGSFLQVACWMVTGERQAARIRG
MYLKTILRQDVAFFDKETNSGEVVGRMSGDTVLIQDAMGEKVGKFIQLVSTFIGGFIISFIKGWLLTLVMLSSIP
LLVIAGAGLSIMISRMASRGQTAYTKAASVVEQTIGSIRTVASFTGEKQAISNYKKFLVTAYNSGVQEGLAAGV
GLGIVMLVVFCSYALAVWFGGRMILEKGYTGGDVINVIVAVLTGSMSLGQASPCMSAFASGQAAAYKMFEAI
NRKPDIDASDTRGKILNDIRGDIELRDVYFNYPARPDEQIFSGFSLFIPSGSTAALVGQSGSGKSTVISLIERFYDP
QAGEVLIDGINLKEFQLKWIREKIGLVSQEPVLFTSSIKDNIAYGKDMATTEEIRAAAELANAAKFIDKLPQGID
TMVGEHGTQLSGGQKQRIAIARAILKDPRILLLDEATSALDAESERIVQEALDRIMVNRTTVIVAHRLSTVRNA
DMIAVIYRGKMVEKGSHSELLEDPEGAYSQLIRLQEVNKESEQEADDQKKSDISTESLRHSSQKISLRRSISRGSS
DFGNSSRRSFSVTFGFPTGFNAPDNYTEELEASPQKQQAPDVPISRLVYLNKPEFPVLIAGAIAAILNGVIFPIFGII
ISRVIKAFFEPPHELRKDSKLWALMFMTLGLASFVVYPSQTYLFSVAGCKLIQRIRSMCFEKMVHMEVGWFDE
PEHSSGAIGARLSADAATVRGLVGDSLSQLVQNIASAIAGLVIAFVACWQLALLILVLLPLIGLNGFIQMKFLKG
FSSDAKKMYEEASQVANDAVGSIRTVASFCAEEKVMQLYRKKCEGPMRTGIRQGLISGAGFGVSFFLLFSVYA
TSFYVGAQLVQHGKTNFTDVFQVFFALTMAAMGISQSSSFAPDSSKAKAAAASIFSIIDRKSKIDSGDESGTTLD
NVKGEIELRHIGFKYPARPDIEIFRDLSLAIHSGKTVALVGESGSGKSTVISLLQRFYDPHSGHITLDGIDIKSLQL
KWLRQQMGLVSQEPVLFNETIRANIAYGKEGEATEAEILAASELANAHKFISSLQQGYDTVVGERGIQLSGGQ

KQRVAIARAIVKSPKILLLDEATSALDAESERVVQDALDRVMVNRTTVVVAHRLSTIKNADVIAVVKNGVIVE
KGKHETLIHIKDGFYASLVALHMSASTS

>XP_021610632.1_ABCB11-l_Me
MAEENGLNGVAGTQEAGTSKTYEEEEEKNPGINGNLQEAKKSKEDEKTNSVPFHKLFSFADSIDILLMIVGTIG
AVGNGISLPLMTIFLGDTINAFGQNQNKDVVHVVSKVSLKFVYLAVASAVASFLQVACWIVTGERQAARIRGL
YLKTILRQDVAFFDKETNTGEVIGRMSGDTVLIQDAMGEKVGKFLQLVSTFIGGFVVAFIKGWLLTIVLLSSIPL
LVLAGAAMSISIARMASRGQNAYAKAATVVEQTIGSIRTVASFTGEKQAISNYKKNLVTAYNSGVNEGLATGL
GLGVLMLIIFCSYALAIWFGGKMILEKGYTGGSVLNVIIAVLSGSMSLGQASPCMSAFAAGQAAAYKMFDTISR
KPEIDAYDTRGKILDDIHGDIELRDIHFSYPARPDEQIFSGFSLFIASGTTTALVGQSGSGKSTVISLIERFYDPQAG
EVLIDGINLKEFQLKWIREKIGLVSQEPVLFTASIRDNIAYGKDGATTEEIRAAAELANAAKFIDKLPQGLDTMA
GEHGTQLSGGQKQRIAIARAILKDPRILLLDEATSALDAESERIVQEALDRIMVNRTTVIVAHRLSTIRNADVIAV
IHRGKMVEKGSHSELLSDPEGAYSQLIRLQEVNKDSEQATEDHKRSNLSSESFRQSSQRISLQRSISRESSGVGNS
SRHSFSVSFGLPTGINVTENSQEKNEVSPPQKEIPEVSIRRLAYLNKPEIPVLTIGTIAACINGIIFPIFGILISRVIKSF
YEPPHELRKDTKFWAFIFMIIGVASFLVLPSQFYFFGVAGNRLIQRIRTICFEKVVHMEVGWFDDPEHSSGAIGA
RLSADAAVVRALVGDALAQLVQNIASAVAGLVIAFTASWQLAFIILVLLPLIGINGYVQVKFMKGFSADAKMM
YEEASQVANDAVGSIRTVASFCAEEKVMQLYKKKCEGPLKTGVRQGLISGIGFGVSFFFLFSVYATSFYAGAQL
VKHGKTTFSDVFQVFFALTMAAIGISQSSSFAPDSAKAKNAAASIFSIIDRKSKIDPSDDSGMTVENVRGEIELRH
VSFKYPSRPDVQIFRDLSLAIHSGKTVALVGESGSGKSTVISLLQRFYDPESGHITLDGVEIQRLQLKWLRQQMG
LVSQEPVLFNYTIRANIAYGKDGDATEAEIIAASEKANAHKFISSLQQGYDTVVGERGVQLSGGQKQRVAIARA
IIKSPKILLLDEATSALDAESERVVQDALDRVMVNRTTVVVAHRLSTIKNADVIAVVKNGVIVEKGKHENLINM
RDGFYASLVALHMSASTA

>XP_021676128.1_ABCB11-l_X1_Hb
MAEENGFNGVAKTHEASTSKTHEEEEEKKPGINGNLQETEKSKGDEKTNSVPFHKLFSFADSLDILLMIVGTIG
AIGNGICLPLMTVFLGDTINAFGENQNKDVVHVVSKVSLKFVYLAVGSAVASFFQVACWIVTGERQAARIRGL
YLKTILRQDVAFFDKETNTGEVIGRMSGDTVLIQDAMGEKVGKFLQLVSTFVGGFVVAFIKGWLLTLVLMSSIP
LLVLAGAAMSMTIARMASRGQNAYAKAASVVEQTIGSIRTVASFTGEKQAINNYKKFLVTAYNSGVREGLAT
GLGLGVVMLIIFCSYALAIWFGGKMILEKGYSGGSVLNVIIAVLSGSMSLGQASPCMSAFAAGRAAAYKMFETI
SRKPEIDAYDTRGKILDDIRGDIELRDIHFSYPARPDEQIFSGFSLSIPSGTTAALVGQSGSGKSTVISLIERFYDPQ
AGEVLIDGVNLKEFQLKWIREKLGLVSQEPVLFTASIRDNIAYGKDGATTEEIRAAAELANAAKFIDKLPQGLD
TMAGEHGTQLSGGQKQRIAIARAILKDPRILLLDEATSALDAESERIVQEALDRIMVNRTTVIVAHRLSTIRNAD
VIAVIHRGKMVEKGSHSELLSDPDGAYSQLIRLQEVNKDSEHAAEDHKRSDLSSESFRQSSQRISLRRSISRGSS
GVGNSSRHSFPVSFGLPTGINVTENSQEETEVSPPQEKAPKVPIRRLAYLNKPEIPMLTIGTIAACINGVIFPIFGILI
SRVIKSFYEPPHELRKDTKFWSIIFMVLGVASFLVLPSQFYFFAVAGSKLIQRIRTICFERVVHMEVGWFDDPEH
SSGAIGARLSADAAIIRALVGDALAQLVQNISSAVAGLVIAFTASWQLAFIILVLIPLIGINGYVQVKFMQGFSAD
AKMMYEEASQVANDAVGSIRTVASFCGEEKVMQLYKKKCEGPLKTGVRQGLISGVGFGVSFFLLFCVYATSF
YAGAQLVQHGKTKFSDVFQVFFALTMAAIGISQSSSFAPDSSKAKNAAASIFSIIDRKSKIDPSDESGMTLENVR
GEIELRHISFKYPSRPDVQIFRDLSLAIHSGKTVALVGESGSGKSTVISLLQRFYDPDSGHITLDGVEIQRLQLKW
LRQQMGLVSQEPVLFNDTIRANIAYGKDEEATEAEILAASEMANAHKFISSLQQGYDTVVGERGVQLSGGQKQ
RVAIARAIIKSPKILLLDEATSALDAESERVVQDALDRVMVNRTTIVVAHRLSTIKNADVIAVVKSGVIVEKGK
HEALINIKDGFYASLVALHMSASTT

>XP_017636209.1_P_ABCB4-l_Ga
MATENGFNGDTNLHKASTSKSQETSKGDEKTNTVPFHKLFVFADSTDILLMIVGTVGAVGNGLCMPLMTILFG
DLVNAFGQNQSNNQVVHVVSKVSLKFVYLAVGAGVAAFLQVSCWMVTGERQAARIRGLYLKTILRQDIAFF
DVETNTGEVVGRMSGDTVLIQDAMGEKVGKVLQLLSTFFGGFTIAFVKGWLLTLVMLSSIPLLVLSGATMAVI
ISKMATRGQTAYAKAATVVEQTIGSIRTVASFTGEKQAISNYNKFLVTAYKSGVHEGTAAGLGLGVVLLIIFCS
YSLAVWFGGKMILEKGYTGGVVVNVIIAVLTGSMSLGQASPCMSAFAAGQAAAFKMFKTINRKPEIDPYDMS
GKVLEDIHGDVELRDVYFSYPARPEEQIFSGFSLSIPCGTTAALVGESGSGKSTVISLIERFYDPQAGEVLIDGINL
KDFQLRWIRGKIGLVSQEPVLFTSSIKDNIAYGKEDATIEEIQAAAELANAAKFIDKLPQGLDTMVGEHGTQLS
GGQKQRVAIARAILKDPRILLLDEATSALDAESERVVQEALDRIMGNRTTVIVAHRLSTVRNADTIAVIHRGKM
VEKGSHSELLKDPEGAYSQLIRLQEVNKESEQVADLSEVTPESFRQSSLRRSMKRSISRGSSIGNSSRHSFSVSFG
LPTGMNVNDSATVDTEDPSKQPLKQPLEVPIRRLAYLNKPEIPVLLLGTIAAVANGVILPIYGLLLSHVIETFFKP

PDELKKDTRFWALIFMALGLASLLASPARTYFFSIAGCKLIQKIRLMCFSKVVHMEVGWFDEPDNSSGSIGARL
SVDAASIRGLVGDALAQMVSNLASAIAGLVIAFVASWQLALIMLGLVPLIGFTGYFQANFIKGFSADAKMMYE
DASQVANDAVGSIRTVASFCAEEKMMQLYSKKCEGPLQTGIKQGLISGSGFGLSFFLMFAVYATNFYAGAQLV
KHGHVTFSDVFQVFFGLTMATIGITQSSSFAPDSSKAKSAAASIFAIIDRESKIDPSDESGTTLENVKGDIELHHVS
FKYPLRPDIQIFRDLSLSIHAGKTIALVGESGSGKSTVISLLQRFYDPDSGHITLDGVEIQNLQLKWLRQQMGLVS
QEPVLFNETIRANIAYGKGGNATEAEILAASELANALKFISSLQQGYDTVVGERGVQLSGGQKQRVAIARAIVK
SPKILLLDEATSALDAESERVVQDALDRVMVNRTTVVVAHRLSTIKNADVIAVVKNGVIVEKGKHDTLINIKD
GIYASLVALHMSASAS

>XP_016708493.1_P_ABCB4-l_Gh
MATENGFNGDTNLHKASTSKSQETSKGDEKTNTVPFHKLFVFADSTDILLMIVGTVGAVGNGLCMPLMTILFG
DLVNAFGQNQSNNQVVHVVSKVSLKFVYLAVGAGVAAFLQVSCWMVTGERQAARIRGLYLKTILRQDIAFF
DVETNTGEVVGRMSGDTVLIQDAMGEKVGKVLQLLSTFFGGFTIAFVKGWLLTLVMLSSIPLLVLSGATMAVI
ISKMATRGQTAYAKAATVVEQTIGSIRTVASFTGEKQAISNYNKFLVTAYKSGVHEGTAAGLGLGVVLLIIFCS
YSLAVWFGGKMILEKGYTGGVVVNVIIAVLTGSMSLGQASPCMSAFAAGQAAAFKMFKTINRKPEIDPYDMS
GKVLEDIHGDVELRDVYFSYPARPEEQIFSGFSLSIPCGTTAALVGESGSGKSTVISLIERFYDPQAGEVLIDGINL
KDFQLRWIRGKIGLVSQEPVLFTSSIKDNIAYGKEDATIEEIQAAAELANAAKFIDKLPQGLDTMVGEHGTQLS
GGQKQRVAIARAILKDPRILLLDEATSALDAESERVVQEALDRIMGNRTTVIVAHRLSTVRNADTIAVIHRGKM
VEKGSHSELLRDPEGAYSQLIRLQEVNKESEQVADLSEVTPESFRQSSLRRSMKRSISRGSSIGNSSRHSFSVSFG
LPTGMNVNDSATVDTEDPSKQPLKQPLEVPIRRLAYLNKPEIPVLLLGTIAAVANGVILPIYGLLLSHVIETFFKP
PDELKKDTRFWALIFMALGLASLLASPARTYFFSIAGCKLIQKIRLMCFSKVVHMEVGWFDEPDNSSGSIGARL
SVDAASIRGLVGDALAQMVSNLASAIAGLVIAFVASWQLALIMLGLVPLIGFTGYFQANFMKGFSADAKMMY
EDASQVANDAVGSIRTVASFCAEEKMMQLYSKKCEGPLQTGIKQGLISGSGFGLSFFFMFAVYATNFYAGAQL
VKHGHVTFSDVFQVFFGLTMATIGITQSSSFAPDSSKAKSAAASIFAIIDRESKIDPSDESGTTLENVKGDIELHH
VSFKYPLRPDIQIFRDLSLSIHAGKTVALVGESGSGKSTVISLLQRFYDPDSGHITLDGVEIQNLQLKWLRQQIGL
VSQEPVLFNETIRANIAYGKGGNATEAEILAASELANALKFISSLQQGYDTVVGERGVQLSGGQKQRVAIARAI
VKSPKILLLDEATSALDAESERVVQDALDRVMVNRTTVVVAHRLSTIKNADVIAVVKNGVIVEKGKHDTLINI
KDGIYASLVALHMSASAS

>XP_010522857.1_P_ABCB11-l_Th
MKLDDGENGYSVSHEASTSKNTKEEKEEKKEMKNGKDEEKTKAVVPFYKLFAFADSMDVFLMIFGTIGAIGN
GVCMPLMTVIFGDLIDSFGENQNNNKDIVDIVSKVCLKFVYLGLGTLAAAFLEVACWMITGERQAARIRSLYL
RTILRQDIAFFDVETNTGEVVGRMSGDTVLIQEAMGEKVGKFIQLISTFIGGFAVAFVKGWLLTLVMLSSIPLLA
MAGAAIAIIVSRTSSRGQAAYAKAATVVEQTIGSIRTVASFTGERQAIRNYNEFILSAYKSSVQQGFSTGFGLGV
LFLVFFGSYALAIWFGGKMILEKGYTGGAVINVIIAVVTGSMSLGQTSPCLSAFAAGQAASYKMFETIERKPLID
SYDVNGKVLEDVRGDIELKDVYFSYPARPDEQIFTGFSLFISSGTTAALVGQSGSGKSTVISLIERFYDPQAGEVL
VDGVNLKDFQLKWIRSKIGLVSQEPVLFSSSIKDNIGYGKEKATMEEIKAAAELANAAKFIDKLPQGFDTMVGE
HGTQLSGGQKQRIAIARAILKDPRILLLDEATSALDAESERVVQEALDRVMVNRTTVVVAHRLSTVRNADMIA
VIHHGKMVEKGTHSELLKDPEGAYSQLIRLQEINKDQNDSNKSEISSGSFRNSSLRKPSLGRSISRGSSVGNSSRH
HSFSILGVPAGLDVGDADQEKATIPREQAPNVSLKRLAALNKPEIPVLLLGTVAAAVQGMIFPIFGILISSVIETFF
KPPRELKKDSRFWAMIFLVLGIASVIASPAQTYLFAVAGGKLIRRIRSMCFEKVVHMEVGWFDEPQNSSGAIGA
RLSADAAMIRALVGDALSLAVQNATSAAAGLIIAFTASWQLALIILVMLPLIGINGYVQVKFLKGFSADAKTKY
EEASQVANDAVGSIRTVASFCAEEKVMRMYKKQCEGPIKAGIKQGVISGLGFGVSFFILFSVYATSFYAGARFV
EDGKTTMNDVFRVFFALTMAAIGISQSSSFAPDSSKAKVAAASIFGIIDRKSKIDPSHESGTVLENVKGDIELRHI
SFVYPARPDIHIFRDLCLSIRAGKTVALVGESGSGKSTVISLLQRFYDPDSGHITLDGVELKKLQLRWLRQQMGL
VGQEPVLFNDTIRANIAYGKGGANATEAEIVAAAELANAHRFISALQQGYETVVGERGIQLSGGQKQRVAIAR
AIVKEPKILLLDEATSALDAESERVVQDALDRVMVNRTTVVVAHRLSTIKNANVIAVVKNGVIAEKGTHDSLI
HIEGGVYASLVQLHLTASH

>XP_017626674.1_P_ABCB11-l_Ga
MDSENGFNDPTDLHEASTSKIQEDPDKVSGSNGDNLESKKVDDEKTYTVPFYKLFAFADSRDTLLMIVGTIGA
VGNGICMPLMTLLFGDLIDAFGENQNDDRVVDVVSRVALRFVYLAVGAGVAAFLQVTCWMVTGERQAARIR
GLYLKTILRQDVAFFDVETNTGEVVGRMSGDTVLIQDAMGEKVGKFIQLVSTFIGGFVIAFVQGWLLTLVMLS
SIPPIVISGGVMALIVSKMASRGQNAYAKAASVVEQTIGSIRTVASFTGEKQAISNYNKFLGAAYTSGVHEGFV

AGLGLGVLFLVIFCSYSLAIWFGARMVLDRGYTGGDVINVIFAVLTGSMSLGQASPCVTAFAAGQAAAFKMFE
TIERKPEIDSYDTRGKVLEDIRGDIELRDVYFSYPARPDEQIFSSFSLSIQNGTTVALVGQSGSGKSTVISLIERFYD
PHAGEVLIDGINLKEFQLRWIRGKIGLVSQEPVLFTSSIRDNIAYGKEGATTEEIRAAAELANASKFIDKLPQGLD
TMVGEHGTQLSGGQKQRVAIARAILKDPRILLLDEATSALDAESERVVQEALDRIMGNRTTVIVAHRLSTVRN
ADMIAVIHRGKMVEKGSHSELLQDPEGAYSQLIRLQEVNKDSEQATESSDIASESFRRSSLKKSLKRSISRGSSM
GNSNRHSFSASFGLPTGMNAADLAMADTENPAELPSEKAPKVSIRRLAYLNKPEIPVILLGTIAAAANGVIFPIF
GILISSVIDAFFKPHELREDSRFWALIFLALGAAAFVVCPAQNYFFSIAGSKLIQRIRSMCFEKVVRMEVGWFDE
PENSSGAIGARLSADAASIRALVGDALAQLVQNTSSAISGLVIAFVACWQLAFIVLVLLPLIAINGYIQVKFMKG
FSADVKLMYEEASQVANDAVGSIRTVASFCAEEKVMQLYKKKCEGPMKTGIKQGLISGTGFGVSFFFLFSVYA
TSFYAGAQLVEHGYTTFRDVFQVFFALTMAAIGISQSSSFAPDSGKAKSAAASIFAIIDRESKIDPNDESGMKLE
NVKGDIELHHVSFKYPLRPDIQILRDLSLSIRSGKTVALVGESGSGKSTVISLLQRFYDPDSGRISLDGLDIQKLQ
LKWLRQQMGLVSQEPVLFNDTIRANIAYGKGGNATEAEILAASELANAHKFISSLQQGYDTVVGERGVQMSG
GQKQRIAIARAIVKSPQILLLDEATSALDAESERVVQAALDRVVVNRTTVVVAHRLSTIKNADVIAVVKNGVV
VEKGKHDTLINIKDGFYASLVALHMSASTA

>XP_018810975.1_P_ABCB11-l_Jr
MAAENGFGGKINADEATTSESYPEAEKTSSTNGDQEDSKKSKGHEKTNTIPFRKLFSFADSTDILMMILGTIGAI
GNGICMPLMTVLFGDLMDSFGDNQNNHEVVEVVSKVSLKFVYLGLGSGVAAFLQVACWMVTGERQAARIRG
LYLKTILRQDVAFFDKETNTGEVIGRMSGDTVLIQDAMGEKVGKFIQLVSTFIGGFVIAFIKGWLLTLVMLSSIP
LLVVSGAVMSTIIAKMASVGQSAYAKAANVVEQTIGSIRTVASFTGEKQAIINYNKFLVKAYKSGVHEGLASG
FGLGVVMLVVFCSYALAIWFGAKMILEKGYSGGAVLTVIIAVLTGSMSLGQASPCMSAFAAGQAAAFKMFETI
ERKPEIDAYDTKGRTLDDIRGDIELRDVFFSYPSRPDEQIFNGFSLCIPSGTTAALVGQSGSGKSTVISLIERFYDP
LAGEVLIDGINLKEYQLKWIRGKIGLVSQEPVLFASSIKDNISYGKDGATIEEIRAATELANAAKFIDKLPQGLDT
MVGEHGTQLSGGQKQRIAIARAILKDPRILLLDEATSALDAESERIVQEALDRIMVNRTTVIVAHRLSTVRNAD
MIAVIHRGKMVEKGSHSVLINDPDGAYSQLIRLQELNKESEQSVDDQNKQEITAESARQSSQRMSILRSISRGSS
GVGNSSRHSFSVSVGLPTGINLPDIALAEKQTPQLPAEEYPNVSLRRIAYLNKPEIPVLIIGAIAAVINGTILPIFGL
LISSVIKTFYEPPNELKKDSKFWAIMFMILGLASFLVIPARSYFFAVAGCKLIQRIRVICFEKVVHMEVGWFDEPE
HSSGAIGARLSADAASVRALVGDALGQVVENAAAAVAGLVIAFVASWQLAFIILVLIPLIGLNGYVQVKFLKG
FSADAKMKYEEASQVANDAVGSIRTVASFCAEEKVMQLYRKKCEGPMKTGIRLGLISGLGFGMSFLLLFCVY
ATSFYAGARLVDDGKATFSDVFRVFFALTMAAIGISQSSSFAPDSSKAKTAAASIFGIIDRKSKIDPSEESGMKLD
DVKGDIELRHLSFKYPSRPDIQILRDLSLAIHSGKTVALVGESGSGKSTVISLLQRFYDPDSGHITLDGIEIQKFQL
KWLRQQMGLVSQEPILFNDSIHANIAYGKEGNATEAEIIAASELANAHKFISSLQQGYDTMVGERGVQLSGGQ
KQRVAIARAIIKSPRILLLDEATSALDAESEKVVQDALDRVMVNRTTIVVAHRLSTIKNADLIAVVKNGVIVEK
GKHDTLIKIKDGFYASLVALHSSASTA

>XP_016735656.1_P_ABCB4-l_Gh
MATENGFNGDTNLHKACTSKSQETSKGDEKTNTVPFHKLFVFADSTDILLMIVGTVGAVGNGLCMPLMTILFG
DLVNAFGQNQSNNQVVHVVSKVSLKFVYLAVGAGVAAFLQVSCWMVTGERQAARIRGLYLKTILRQDIAFF
DVETNTGEVVGRMSGDTVLIQDAMGEKVGKVLQLLSTFFGGFIIAFVKGWLLTLVMLSSIPLLVISGATMAVII
SKMATRGQTAYAQAATIVEQTIGSIRTVASFTGEKQAMSNYNKLLATAYKSGVHEGTAAGLGLGVVLLIIFCS
YSLAVWFGGKMILEKGYTGGEVVNVIIAVLTGSMSLGQASPCMSAFAAGQAAAFKMFKTINRKPEIDPYDMS
GKVLEDIHGDVELRDVYFSYPARPEEQIFSGFSLSIPCGTTAALVGESGSGKSTVISLIERFYDPLAGEVLIDGINL
KDFQLRWIRGKIGLVSQEPVLFTSSIKDNIAYGKEDATIEEIQAAAELANAAKFIDKLPQGLDTMVGEHGTQLS
GGQKQRVAIARAILKDPRILLLDEATSALDAESERVVQEALDRIMGNRTTVIVAHRLSTVRNANTIAVIHRGKM
VEKGSHSELLKDPEGAYSQLIRLQEVNKESEQVADVSEVTPESFRQSSLRRSMKRSISRGSSIGDSSHHSFSVAF
GLPTGMNVNDSSTVDTEDPSKQPLKQPLEVPIRRLAYLNKPEIPVLLLGTIAAVANGVILPIYGLLLSHVIETFFK
PPDELKKDTRFWALIFMALGLASLLASPARTYFFSIAGCKLIQKIRLMCFSKVVHMEVGWFDEPDNSSGSIGAR
LSVDAASIRGLVGDALAQMVSNLASAIAGLVIAFVASWQLALIMLGLVPLIGFTGYFQANFMKGFSADAKMM
YEDASQVANDAVGSIRTVASFCAEEKMMQLYSKKCEGPLQTGIKQGLISGSGFGLSFFLMFSVYATNFYAGAQ
LVKHGHVKFSDVFQVFFGLTMATIGITQSSSFAPDSSKAKSAAASIFAIIDRESKIDPSDESGTTLENVKADIELH
HVSFKYPLRPDIQIFQDLSLSIHAGKTVALVGESGSGKSTVISLLQRFYDPDSGHITLDGVEIRTLQLKWLRQQM
GLVSQEPVLFNETIRANIAYGKGGNATEAEILAASELANAHKFISSLQQGYDTVVGERGVQLSGGQKQRVAIAR
AIVKSPKILLLDEATSALDAESERVVQDALDRIMVNRTTVVVAHRLSTIKNADVIAVVKNGVIVEKGKHDTLIN
IKDGLYASLVALHMSASAS

>XP_016677999.1_P_ABCB11-l_Gh
MDSENGFNDPTDLHEASTSKIQEDPDKVSGSNGDNLESKKVDDEKTNTVPFYKLFAFADSRDTLLMIVGTIGA
VGNGICMPLMTLLFGDLIDAFGENQNDDRVVDVVSRVALRFVYLAVGAGVAAFLQVTCWMVTGERQAARIR
GLYLKTILRQDVAFFDVETNTGEVVGRMSGDTVLIQDAMGEKVGKFIQLVSTFIGGFVIAFVQGWLLTLVMLS
SIPPIVISGGVMALIVSKMASRGQNAYAKAASVVEQTIGSIRTVASFTGEKQAISNYNKFLGAAYTSGVHEGFV
AGLGLGVLFLVIFCSYSLAIWFGARMVLDRGYTGGDVINVIFAVLTGSMSLGQASPCVTAFAAGQAAAFKMFE
TIERKPEIDSYDTRGKVLEDIRGDIELRDVYFSYPARPDEQIFSGFSLSIQNGTTVALVGQSGSGKSTVISLIERFY
DPHAGEVLIDGINLKEFQLRWIRGKIGLVSQEPVLFTSSIRDNIAYGKEGATTEEIRAAAELANASKFIDKLPQGL
DTMVGEHGTQLSGGQKQRVAIARAILKDPRILLLDEATSALDAESERVVQEALDRIMGNRTTVIVAHRLSTVR
NADMIAVIHRGKMVEKGSHSELLQDPEGAYSQLIRLQEVNKESEQATESSDIASESFRRSSLKKSLKRSISRGSS
MGNSNRHSFSASFGLPTGMNAADLAMADAETPAGLPSEKAPKGSVLRRLAYLNKPEIPVILLGTIAAAANGVIF
PIFGILISSVIDAFFKPHELREDSRFWALIFLALGAAAFVVCPAQNYFFSIAGSKLIQRIRSMCFEKVVRMEVGWF
DEPENSSGAIGARLSADAASIRALVGDALAQLVQNTSSAISGLVIAFVACWQLAFIVLVLLPLIAINGYIQVKFM
KGFSADVKLMYEEASQVANDAVGSIRTVASFCAEEKVMQLYKKKCEGPMKTGIKQGLISGTGFGVSFFFLFSV
YATSFYAGAQLVEHGYTTFRDVFQVFFALTMAAIGISQSSSFAPDSGKAKSAAASIFAIIDRESKIDPNDESGMK
LENVKGDIELHHVSFKYPLRPDIQILRDLSLSIRSGKTVALVGESGSGKSTVISLLQRFYDPNSGRISLDGVDIQK
LQLKWLRQQMGLVSQEPVLFNDTIRANIAYGKGGNATEAEILAASELANAHKFISSLQQGYDTVAGERGVQM
SGGQKQRIAIARAIVKSPQILLLDEATSALDAESERVVQAALDRVVVNRTTVVVAHRLSTIKNADVIAVVKNG
VVVEKGKHDTLINIKDGFYASLVALHMSASTA

>XP_002273987.1_P_ABCB11_Vv
MAEENDLNGKTYMHEATTSSRGALETETVKSSGQNGKQQDSEKSKEEGKPSTVPFHKLFSFADSTDMLLMITG
TIGAAGNGICMPLMAILFGDLIDSFGQNQNNKDVVDIVSKVSLKFVYLAVGAGIAAFFQVACWMVTGERQAA
RIRSLYLKTILRQDVAFFDKETNTGEVIGRMSGDTVLIQDAMGEKVGKFIQLVSTFIGGFIIAFIKGWLLTLVMLS
SIPLLVIAGGAMSLFLSKMATRGQNAYAKAATVVEQTIGSIRTVASFTGEKQAVTKYNQFLVNAYKSGVFEGL
AAGLGLGTVMFIIFASYALAVWFGAKMILEKGYTGGTVLNVIIAVLTGSMSLGQASPCMSAFAAGQAAAFKM
FQTIHRKPEIDVSDTKGKKLEDIQGEIELRDVYFSYPARPDEQIFSGFSLSIPSGTTAALVGQSGSGKSTVISLIERF
YDPLAGEVLIDGINLKEFQLRWIRGKIGLVSQEPVLFTSSIRDNIAYGKEGATIEEIRAAAELANASKFIDKLPQG
LDTMVGEHGTQLSGGQKQRVAIARAILKDPRILLLDEATSALDAESERVVQEALDRIMVNRTTIIVAHRLSTVR
NADMIGVIHRGKMVEKGSHTELLKDPEGAYSQLIRLQEVNKESENQATDSQDRPDGSIEFGRQSSQRMSFLRSI
SRGSSGPGNSSRHSFSVSFGLPTGLGLPDNAIADAEAPRSSEQPPEVPIRRLAYLNKPEIPVLLLGTVAAIVNGTIL
PIFGILISSVIKTFYEPPHQLRKDSNFWALIFLVLGVVSFLAFPARTYLFSVAGCKLIQRVRSMCFEKVVHMEVG
WFDQPEHSSGAIGARLSADAATIRALVGDALAQVVQNAASAIAGLAIAFAASWQLAFIILALIPLIGLNGYVQIK
FLKGFSADAKMMYEEASQVANDAVGSIRTVASFCAEEKVMDLYKKKCEGPMRTGIRQGLVSGIGFGVSFFLLF
CVYALCFYAGARLVEAGKTTFGDVFRVFFALTMATVGISQSSSFSPDSSKAKSAAASIFTIIDRKSTIDPSDESGT
KLENVKGEIELRHISFKYPTRPDIQIFRDLSLTIRSGKTVALVGESGSGKSTVIALLQRFYDPDSGHITLDGVDIQS
LQLRWLRQQMGLVSQEPVLFNDTIRANIAYGKEGHTTEAEVIAASELANAHKFISGLQQGYDTMVGERGIQLS
GGQKQRVAIARAMVKSPKILLLDEATSALDAESERVVQDALDRVMVNRTTVVVAHRLSTIKGADVIAVVKNG
VIVEKGKHETLINIKDGFYASLIALHMSASS

>XP_012437899.1_P_ABCB4-l_Gr
MATENGFNGDTNLHKACTSKSQETSKGDEKTNTVPFHKLFVFADSTDILLMIVGTVGAVGNGLCMPLMTILFG
DLVNAFGQNQSNNQVVHVVSKVSLKFVYLAVGAGVAAFLQVSCWMVTGERQAARIRGLYLKTILRQDIAFF
DVETNTGEVVGRMSGDTVLIQDAMGEKVGKVLQLLSTFFGGFIIAFVKGWLLTLVMLSSIPLLVISGATMAVII
SKMATRGQTAYAQAATVVEQTIGSIRTVASFTGEKQAISNYNKLLATAYKSGVHEGTAAGLGLGVVLLIIFCSY
SLAVWFGGKMILEKGYTGGEVVNVIIAVLTGSMSLGQASPCMSAFAAGQAAAFKMFKTINRKPEIDPYDMSG
KVLEDIHGDVELRDVYFSYPARPEEQIFSGFSLSIPCGTTAALVGESGSGKSTVISLIERFYDPLAGEVLIDGINLK
DFQLRWIRGKIGLVSQEPVLFTSSIKDNIAYGKEDATIEEIQAAAELANAAKFIDKLPQGLDTMVGEHGTQLSG
GQKQRVAIARAILKDPRILLLDEATSALDAESERVVQEALDRIMGNRTTVIVAHRLSTVRNANTIAVIHRGKMV
EKGSHSELLKDPEGAYSQLIRLQEVNKESEQVADVSEVTPESFRQSSLRRSMKRSISRGSSIGDSSHHSFSVAFGL
PTGMNVNDSSTVDTEDPSKQPLKQPLEVPIRRLAYLNKPEIPVLLLGTIAAVANGVILPIYGLLLSHVIETFFKPP
DELKKDTRFWALIFMALGLASLLASPARTYFFSIAGCKLIQKIRLMCFSKVVHMEVGWFDEPDNSSGSIGARLS
VDAASIRGLVGDALAQMVSNLASAIAGLVIAFVASWQLALIMLGLVPLIGFTGYFQANFMKGFSADAKMMYE

DASQVANDAVGSIRTVASFCAEEKMMQLYSKKCEGPLQTGIKQGLISGSGFGLSFFLMFSVYATNFYAGAQLV
KHGHVKFSDVFQVFFGLTMATIGITQSSSFAPDSSKAKSAAASIFAIIDRESKIDLSDESGTTLENVKAEIELHHVS
FKYPLRPDIQIFQDLSLSIHAGKTVALVGESGSGKSTVISLLQRFYDPDSGHITLDGVEIRTLQLKWLRQQMGLV
SQEPVLFNETIRANIAYGKGGNATEAEILAASELANAHKFISSLQQGYDTVVGERGVQLSGGQKQRVAIARAIV
KSPKILLLDEATSALDAESERVVQDALDRIMVNRTTVVVAHRLSTIKNADVIAVVKNGVIVEKGKHDTLINIKD
GLYASLVALHMSASAS

>XP_021612222.1_ABCB11-l_Me
MAMENDINGVTSSHEASTSKSHEEEKISQVNGHPEETGKRKGDEKTNSVPFHRLFSFADFTDIMLMIIGSIGAVG
NGISLPLMTIFLGDMINAFGENQNNKDVLHVVSKVSLKFVYLAVGSGVASFLQVACWMVTGERQAARIRGLY
LKTILRQDIAFFDKETNTGEVIGRMSGDTVLIQDAMGEKVGKFLQLVSTFIGGFVIAFVKGWLLTIVMLSSLPLL
VLAGAAMSIVMARMASQGQNAYAKAATVVEQTIGSIRIVASFTGEKQAISNYEKFLVTAYNAGVREGFFSGLG
LGLFTLIIFCSYGLAIWFGGKMILEKEYTGGEVINVIIAVLTGSTSLGQASPSMTAFAAGQAAAYKMFETINRKS
EIDAYDTRGKILDDIHGNIELREVYFSYPARPNEQIFNGFSLSIPTGTTAALVGQSGSGKSTVISLIERFYDPQAGE
VLIDGINLKEFQLKWIRNKIGLVSQEPVLFTSSIRDNIAYGKDEATTEEIRAAAELANAAKFIDKLPQGLDTMVG
EHGTQLSGGQKQRIAIARAILKDPRILLLDEATSALDAESERIVQEALDRIMVNRTTVIVAHRLSTVRNADMIAV
IHHGKIVQKGSHPELLADPDGAYSQLIRLQEINQDSEHAADENKGSDISSESFRRSSQRNSLQRSISRASNGVGNS
HRHSFSASFGLPTGINASEPEVSPQEKQTSEVPISRLAYLNKPEIPVLVAGSIAAIINGVIFPIFGILISRVIKAFFEPP
HELRKDSKFWAIIFMIIGIVSFLACVTQLYLFSVAGAKLIQRIRSMCFEKVVHMEVGWFDEVEHSSGTIGARLSA
DASILRALVGDTLAQTVQNIASAVAGLVIAFSASWQLAFIILVLIPLIGINGYVQVKFMKGFSADAKMMYEEAS
QVANDAVGSIRTVASFCAEEKVMQLYRKKCQGPLKSGIRQGLISGIGFGVSFFLLFSVYATSFYAGAQLVKHG
KTTFSDVFQVFFALVMAAIGVSQSSSFAPDSSKAKNAAASIFSILDDKSKIDPSDETGITLENVRGEIELVHVSFR
YPSRPDIQIFQDLCLAIHSGKTLALVGESGSGKSTVISLLQRFYDPDSGNITLDGVEIRRLQLKWLRQQMGLVSQ
EPILFNDTIRDNIAYGKGGNATEAEILAASELANAHKFISSLQQGYDTVVGERGIQLSGGQKQRVAIARAIVKNP
KILLLDEATSALDAESERVVQDALDRVMVSRTTVVVAHRLSTIKNADVIAVVKNGVIVEKGKHDSLINIRDGF
YASLVALHMSASTA

>XP_023893068.1_ABCB11-l_X2_Qs
MAVENGLGSQTNTDAATISKSNAEEEKTSNMNGDQEDSKKSKGDEKTNTVPFRKLFSFADSTDILLMILGTVG
AVGNGICMPLMTLLFGELTNSFGQNQNSPNMVDTVSKVCLKFVYLALGVAVAAFLQVACWMVTGERQAARI
RGLYLKTILRQDVAFFDKETNTGEVVSRMSGDTVLIQDAMGEKVGKFLQLTSTFIGSFVIAFIKGWLLTLVMLS
SIPLLVASGAVMSIIISKMASRGQSAYAKAANVVEQTIGSIRTVASFTGEKQAIISYKKFVLKAYNSGVQEGMAS
GFGLGTAMLVVFCTYALAVWFGAKMILQKGYNGGQVLTVIIAVLTGSMSLGQASPCMSAFAAGQAAAFKMF
ETIGRKPVIDAYDTKGRTLDDIHGDIELRDVYFSYPSRPDELIFNGFSLSIPSGTTTALVGQSGSGKSTVISLIERFY
DPHAGEVLIDGINLKEFQLKWIRGKIGLVSQEPVLFASSIKDNIAYGKDGATIEEIRAAAELANAAKFIDKLPQG
LDTMVGEHGTQMSGGQKQRIAIARAILKDPRVLLLDEATSALDTESERIVQEALDRIMVNRTTVIVAHRLSTVR
NVDMIAVIHRGKMVEKGSHSELLKDPEGAYSQLIRLQEVNKESEQALDDRNNPEITVESFRHSTQRMSIQRSISR
GSSGVGNSSRHSFSVSFGLPTGVNVPDIARAETESPSVPTEKLPNVPLSRVAYLNKPEIPVLIIGAVAAILNGVILP
VFGLLISSAIKIFFEPNGLKKDSKFWAIMFMLLGLASFLIIPARSYFFAVAGCKLIGRIRVMCFEKVVNMEVSWF
DDPDNSSGAIGARLSADAALVRALVGDALGQMVENMASAVAGLVIAFVACWQLAFIVLVLIPLIGVNAFIQVK
FMKGFSADAKMMYEEASQVANDAVGSIRTVASFCAEENVMELYKRKCEGPMKTGIRQGLISGMGFGLSFFLL
FSVYATSFYAGARLVEAGKTTFSDVFRVFFALTMAAIGISQTSSFAPDSSKAKNAAASIFAILDRKSKIDPSEESG
MKLDDVKGEIELRHVSFKYPSRPDIQIFRDLNLKIHSGKTVALVGESGCGKSTVISLLQRFYDPDSGHITLDEIEI
QKFQLKWLRQQMGLVSQEPILFNDTIRANIAYGKEGDATEAEIISASELANAHKFISSLQQGYDTMVGERGVQL
SGGQKQRVAIARAIVKSPKILLLDEATSALDAESEKIVQDALDRVMVNRTTIVVAHRLSTIKNADLIAVVKNGII
VEKGKHETLINIKDGFYASLVALHTSASTI

>XP_004495862.1_ABCB21-l_Ca
MNMENGVDDEATASEKSPIPIETSGNGEKDREKEKEKEKTETVPFHKLFSFADSTDILLMAAGTIGAVGNGLGLPI
MTLLFGQMIDSFGINQSNTTDVVEQVSKVSLKFVYLAVGSGVAAFLQVTCWMVTGERQAARIRGLYLKTILR
QDVAFFDKETNTGEVVGRMSGDTVLIQDAMGEKVGKFVQLTSTFIGGFVIAFTKGWLLTVVMMSTLPLLALA
GAAMALIIGRMASRGQTAYAKAAHVVEQTIGSIRTVASYTGEKQAVSSYSKYLVDAYQSGVFEGSIAGVGLGT
VMFVVFCGYALAVWFGAKMIMEKGYNGGTVINVIIAVLTASMSLGQASPSLSAFAAGQAAAYKMFETIKRRP
EIDSYDPNGKTLEDIQGEIELKDVYFSYPARPEELIFNGFSLHISSGTTAALVGQSGSGKSTVISLVERFYDPHAG

EVLIDGINLKEFQLRWIRGKIGLVSQEPVLFASSIKDNIAYGKEGATIEEIKSASELANAAKFIDKLPQGLDTMVG
DHGTQLSGGQKQRIAIARAILKNPRILLLDEATSALDAESERVVQEALDRIMVNRTTVVVAHRLSTVRNADMIA
VIHRGKMVEKGTHSELLKDPEGAYSQLVRLQEVNRESEETTDHHNSKSELSAESFRQSSQRKSLQRSISRGSSIG
NSSRQSFSVSFGLPTGVNVADPEPENLPTKEEVQEVPLSRLASLNKPEIPVLLIGCLAAIGNGVLFPIFGILISSVIK
TFYEPFDELKKDSKFWAIMFSLLGLASLVVIPARSYFFSVAGCKLIQRIRLICFEKVLSMEVGWFDEPENSSGAV
GARLSADAASVRALVGDALGLMVQNLATALAGLIIAFVASWKLAFIILVLLPLIGLNGYVQMKFMKGFSADA
KMMYEEASQVANDAVGSIRTVASFCAEDKVMELYGKKCEGPMKTGIRQGVISGAGFGVSFFLLFCVYATSFY
AGSRLVKAGDTTFSDVFRVFFALTMSAIGISQSSSFAPDSSKAKSATASIFGMIDKKSKIDPSDESGTTLDSVKGE
IELRHVSFKYPSRPDIQIFRDLNLAIHSGKTVALVGESGSGKSTVIALLQRFYDPDSGEITLDGIEIRELKLKWLRQ
QMGLVSQEPVLFNESIRANIAYGKGGDATEAEIIASSELANAHRFISGLQQGYDTIVGERGTQLSGGQKQRVAI
ARAIIKSPKILLLDEATSALDAESERVVQDALDKVMVNRTTVVVAHRLSTIKNADVIAVVKNGVIVEKGRHETL
INVKDGFYASLVQLHTSAKTV

>XP_003591310.1_ABCB21_Mt
MGKVNGVDEIENNHDEATTSEKNSTETSSTNVVTNGEKDKTKEKQETVPFHKLFTFADSTDILLMIVGTIGAIG
NGLGLPLMTLLFGQMIDSFGSNQSNTTDVVEQVSKVSLKFVYLAVGSGVAAFLQVSCWMVTGERQAARIRGL
YLKTILRQDVTFFDKETNTGEVVGRMSGDTVLIQDAMGEKVGKFLQLIATFIGGFVIAFTKGWLLTVVMMSTL
PFLVVSGAAMAVIIGRMASKGQTAYAKAAHVVEQTIGSIRTVASFTGEKQAVSSYSKFLVDAYKSGVFEGTIA
GAGLGTVMFVIFCGYALAVWFGAKMIIEKGYNGGTVINVIIAVLTASMSLGQASPSMSAFAAGQAAAYKMFE
TIKRRPEIDAYDPNGKILEDIQGEIELKEVYFSYPARPEELIFNGFSLHISSGTTAALVGQSGSGKSTVISLVERFY
DPQAGEVLIDGINMKELQLRWIRGKIGLVSQEPVLFASSIKDNIAYGKDGATIEEIRSASELANAAKFIDKLPQGL
DTMVGDHGTQLSGGQKQRIAIARAILKNPRILLLDEATSALDAESERVVQEALDRIMVNRTTVVVAHRLSTVR
NADMIAVIHRGKMVEKGTHSELLKDPEGAYSQLIRLQEVNKESEETTDHHGKRELSAESFRQSSQRKSLQRSIS
RGSSIGNSSRHSFSVSFGLPTGVNVADPDLEKVPTKEKEQEVPLRRLASLNKPEIPVLLIGSLAAIANGVILPIFGV
LISSVIKTFYEPFDEMKKDSKFWAIMFMLLGLASLVVIPARGYFFSVAGCKLIQRIRLLCFEKVVNMEVGWFDE
PENSSGAVGARLSADAASVRALVGDALGLLVQNLASALAGLIIAFIASWQLALIILVLIPLIGLNGYVQMKFMK
GFSGDAKMMYEEASQVANDAVGSIRTVASFCAEDKVMELYRKKCEGPMKTGIRQGIISGSGFGVSFFLLFSVY
ATSFYAGARLVKAGNTTFSDVFRVFFALTMAAIGISQSSSFAPDSSKAKSATASIFGMIDKKSKIDPSEESGTTLD
SIKGEIELRHISFKYPSRPDIQIFRDLNLTIHSGKTVALVGESGSGKSTVIALLQRFYDPDSGEITLDGIEIRQLQLK
WLRQQMGLVSQEPVLFNDTIRANIAYGKGGIATEAEIIAAAELANAHRFISGLQQGYDTIVGERGTQLSGGQKQ
RVAIARAIIKSPKILLLDEATSALDAESERVVQDALDKVMVNRTTVVVAHRLSTIKNADVIAVVKNGVIVEKGR
HETLINVKDGFYASLVQLHTSAKTV

>XP_024441355.1_ABCB21_Pt
MAIENGRNGDKSMDEASTSKSLEVEEKSSGGRGDQQEPVKSKGDEETKTVPFLKLFSFADSTDILLMILGTIGA
VGNGASFPIMSILFGDLVNSFGQNQNNKDVVDLVTKVSLNFVYLGIGSAVAAFLQVACWMVTGERQAARIRG
TYLKTILKQDVAFFDKETNTGEVVGRMSGDTVLIQDAMGEKVGKFIQLVSTFIGGFIVAFVKGWLLALVMLSSI
PLLVISGAGLAIIIARMASRGQTAYAKAATVVEQTIGSIRTVASFTGEKQAISNYKKFLATAYNSGVQEGFTAGL
GLGIVMLLVFCTYALAIWFGGKMILEKGYTGGDVVNVIIAVLTGSMSLGQASPCMSAFAAGQAAAYKMFETI
NRKPEIDSSDTRGKILDDISGDVELRDVYFTYPARPDEQIFSGFSLFIPSGTTTALVGQSGSGKSTVISLIERFYDP
QAGEVLIDGTNLKEFQLKWIREKIGLVSQEPVLFASSIKDNIAYGKDGATTEEIRAAAELANAAKFIDKLPQGID
TMVGEHGTQLSGGQKQRIAIARAILKDPRVLLLDEATSALDAESERIVQEALDRIMVNRTTVIVAHRLSTVINA
DMIAVIYRGKMVEKGSHSELLKDPEGAYSQLIRLQEVNKESKQETEDPKKSALSAESLRQSSQRISLKRSISRGS
SGVGHSSRHSLSVSFGLPTGFNVPDNPTSELEVSPQKQQTPDVPISRLAYLNKPEVPVLIAGSIAAILNGVILPIYG
ILLSSVIKIFFEPPDELRKDSKFWALMFMTLGLASFVVYPSQTYLFSVAGCKLIQRIRSMCFEKVVHMEVSWFDE
SEHSSGEIGARLSADAAIVRALVGDSLSQLVQNIATAVAGLVIAFSASWQLALVILVLLPLIGLNGFVQVKFMK
GFSADAKKMYEEASQVANDAVSSIRTVASFCAEEKVMQLYRRKCEGPMRTGIRQGMISGTGFGVSFFLLFSVY
ATTFYVGAQLVQHGKTTFAEVFRVFFALTMAAVGISQSSSFSPDSSKAKGAAASIFAIIDRKSKIDPSDESGTTL
DNVKGEIELRHISFKYPSRPDIEIFRDLSLAIHSGKTVALVGESGSGKSTVISLLQRFYDPDSGHITLDGIDIQSLQL
KWLRQQMGLVSQEPVLFNETIRANIAYGKEGNATEAEILAASELANAHKFISGLQQGYDTVVGERGTQLSGGQ
KQRVAIARAMVKSPKILLLDEATSALDAESERVVQDALDRVMVSRTTVVVAHRLSTIKSADVIAVVKNGVIVE
KGKHETLIHIKDGFYASLVALHMSASTS

>XP_021676132.1_ABCB11-l_Hb

MAVGNGINGVTSSHGASTSKSVDEEKISGVNGHPGETEKRKGDQKSNSVPFHRLFSFADSADTMLMIIGTVGA
VGNGLSLPLMTVFLGDMIDAFGENQNNKDVVHVVSKVSLKFVYLAVGSAVASFLQVACWMVTGERQAARIR
GLYLKTILRQDIAFFDKETNTGEVVGRMSGDTVLIQDAMGEKVGKFVQLVSTFIGGFVIAFVKGWLLTIVMLSS
LPLLVLAGAAMSIMMAKMASRGQNAYAKAATVVEQTIGSIRIVASFTGEKQAISNYEKFLVTAYNAGVHEGFF
SGLGLGLFTLIIFCSYSLAIWFGGKMILEKGYTGGEVINVIIVVLTGSTSLGQASPSMTAFAAGQAAAYKMFETI
NRKSEIDAYDTRGKILDDVQGNIELREVYFSYPARPNEQIFMGFSLSIPSGTTAALVGQSGSGKSTVIGLIERFYD
PQAGAVLIDGINLKEFQLKWIRNKIGLVSQEPVLFTSSIGDNIAYGKDEATIEEIRAAAELANAAKFIDKLPQGLD
TMVGEHGTQLSGGQKQRIAIARAILKDPRILLLDEATSALDAESERIVQEALDRIMVNRTTVVVAHRLSTVRNA
DMIAVIHHGKMVQKGSHPELLVDPDGAYSQLIRLQEINQDSEQAADENKGSEISSESFRRSSQGNSLQRSISRGS
FGVGNSHRHSFSASFGLPTGINVSDPEISLQPKQTPEVPIRRLAYLNKPEIPVLVAGSIAAIINGVIYPLFGILISRVI
KAFFKPAHELRKDSKFWAIIFMIVGIVSFLACITQLYLFSVAGSKLIQRIRSMSFEKVVHMEVGWFDELEHSSGTI
GARLSADASTVRALVGDTLAQMVQNIASAVAGLVIAFTASWQLAFIILALIPLIGINGYVQVKFMKGFSADAK
MMYEEASQVANDAVGSIRTVASFCAEEKVMQLYRKKCEGPMKTGIRQGLISGIGFGVSFFLLFSAYATSFYAG
AQLVKHGKTTFSDIFEVFFALVMAALGISQSSSFAPDSSKAKNAASSIFSILDDKSKIDPSDESGIKLENVRGEIEL
LHVSFRYPSRPDIQIFEDLCLAIHSGKTVALVGESGSGKSTVISLLQRFYDLDSGHITLDGVEIRRLQLKWLRQQ
MGLVSQEPVLFNDTIRANIAYGKGGTATEAEILAASGLANAHNFISSLQQGYDTVVGERGVQLSGGQKQRVAI
ARAIVKNPKILLLDEATSALDAESERLVQDALDRVMVNRTTVVIAHRLSTIKNADVIAVVKNGVIVEKGKHDS
LINIRDGFYASLVALHMSASTA

>XP_021664692.1_ABCB11-l_X1_Hb
MAKENGMNGVPRTQEDITSKPHEEEQEKKPSINENSQETKKSKEDEKTNSVPFHKLFSFADSIDILLMIVGTIGA
VGNGISLPLMTVFLGNTINAFGENQNEYVVHVVSKVSLKFVYLAIGTAVASFLQVACWIVTGERQAARIRFLY
LKTILRQDVAFFDKETNTGEVIGRMSGDTVLIQDAMGEKVGTFLSHVSTFIGGFVVAFLKGWLLTVVLLSSIPL
LVLAGAAMSITIARMASLGQDAYAKAATVVEQTIGSIRTVASFTGEKQALSNYKKFLVTAYNSGVHEGLATGL
GFGVVFLIFFCSYALAVWFGGKMILEKGYKGGSVLNVIVAVLTGSMSLGQASPCMSAFAAGQAAAYKMFETV
SRKPAIDAYDTRGKILDDIRGDIELRDIHFRYPARPDEQIFSGFSLSIASGTTAALVGQSGSGKSTVISLIERFYDPQ
AGDVLIDGINLKEFQLKWIREKIGLVSQEPVLFTASIRDNIAYGKDGATTEEVRAAVELANAAKFIDKLPQGLD
TMAGERGTQLSGGQKQRIAIARAIMKDPRILLLDEATSALDAESERIVQEALDRIMVNRTTVIVAHRLSTIRNAA
VIAVIHRGKMVEKGSHSELLSDPDGAYSQLVRLQEVNKDSERVAESHKRSDLSSESFRQSSQRISLQRAISRASS
GVGNSSRHSFSVSFGLPTGIYVTGNSQEQNDFSPPQEETPEVPLHRLAHLNKPEIPVLAIGTIAACINGVILPILGT
LLSRVIKSFYEPPHELRKDTKFWAIIFMILGVTSFLGFPSQYYFFGVAGNRLIQRIRTICFEKVVHMEVGWFDDP
DNSSGAIGARLSADAALIRALVGDTLAQLVQNIASVVAGLVIAFTASWQLAFIVLVLLPLIGVNGYVQVKLFKG
FSADAKMMYEEASQVANDAVGSIRTVASFCAEEKVMQLYKKKCEGPLKTGVRQGLISGIGFGVSFFLLYSVY
ATSFYAGAQLVQHGKTKFSDVFQVFFALILAAIGISHSSSLAPDSTKAKNAAASIFSIIDRKSKIDPSDKSGMTLE
NVRGEIEFRHVSFKYPLRPDVQIFRNLNLVIHSGKTVALVGESGSGKSTVISLLQRFYDPDSGHITIDGVEIQRLQ
LKWLRQQIGLVGQEPVLFNDTIRANIAYGKDGDATEAEILAASELANSHNFISSLQQGYDTVVGERGVQMSGG
QKQRVAIARAIIKSPKILLLDEATSALDAESERVVQDALDRVMVNRTTVVVAHRLSTIKNADVIAVVKNGVIVE
KGKHETLINISDGFYASLVALHMSASTA

>XP_003518599.1_ABCB4_X1_Gm
MDAENGEERKHHEASTSENSAETSTNGEKREKGKQKEKPETVPFHKLFAFADSTDILLMAVGTIGAIGNGLGL
PLMTLLFGQMIDSFGSNQQNTHVVEEVSKVSLKFVYLAVGSGMAAFLQVTSWMVTGERQAARIRGLYLKTIL
RQDVAFFDKETNTGEVIGRMSGDTVLIQDAMGEKVGKFLQLIATFIGGFVIAFVRGWLLTVVMLSTLPLLALSG
ATMAVIIGRMASRGQTAYAKAAHVVEQTIGSIRTVASFTGEKQAVSSYSKFLVDAYKSGVHEGSTAGAGLGT
VMLVIFCGYALAVWFGAKMIMEKGYNGGTVINVIIAVLTASMSLGQASPSMSAFAAGQAAAYKMFQTIERKP
EIDAYDPNGKILEDIQGEIELRDVDFSYPARPEELIFNGFSLHIPSGTTAALVGQSGSGKSTVISLVERFYDPQAGE
VLIDGINLKEFQLRWIRGKIGLVSQEPVLFASSIKDNIAYGKEGATIEEIRSASELANAAKFIDKLPQGLDTMVGE
HGTQLSGGQKQRIAIARAILKNPRILLLDEATSALDAESERIVQEALDRIMVNRTTIIVAHRLSTVRNADVIAVIH
RGKMVEKGTHIELLKDPEGAYSQLIRLQEVNKETEGNADQHNNSELSVESFRQSSQKRSLQRSISRGSSLGNSS
RHSFSVSFGLPTGVNVADPEHESSQPKEEAPEVPLSRLASLNKPEIPVLVIGSVAAIANGVIFPIFGVLISSVIKTFY
EPFDEMKKDSKFWALMFMILGLASFLIIPARGYFFAVAGCKLIQRIRQMCFEKVVNMEVSWFDEPENSSGAIGA
RLSADAASVRALVGDALGLLVQNFATVLAGLIIAFVASWQLALIILVLIPLIGVNGYVQMKFMKGFSADAKMM
YEEASQVANDAVGSIRTVASFCAEDKVMELYKNKCEGPMKTGIRQGLISGSGFGVSFFLLFCVYATSFYAGAR
LVDAGKATFSDVFRVFFALTMAAIGVSQSSSFAPDSSKAKSATASIFGIIDKKSKIDPGDESGSTLDSVKGEIELR

HVSFKYPSRPDIQIFRDLSLTIHSGKTVALVGESGSGKSTVIALLQRFYNPDSGQITLDGIEIRELQLKWLRQQMG
LVSQEPVLFNETIRANIAYGKGGDATEAEIIAAAEMANAHKFISGLQQGYDTIVGERGTQLSGGQKQRVAIARA
IIKSPKILLLDEATSALDAESERVVQDALDKVMVNRTTVVVAHRLSTIKNADVIAVVKNGVIVEKGKHEKLINV
SGGFYASLVQLHTSASTV

>XP_020538546.1_ABCB11_X1_Jc
MQLKYNTLKREFICGSSIMAKREGVNGLPQGHEASTSTGHEEEEKKSIINGKGNPQDTEQSKGDDKTNSVPFYK
LFSFADPTDIILMIVGTIGAVGNGLSLPIMTILLGDTINAFGQNQNNQDVVEVVSKVSLKFVYMAVGTAVASFL
QVTCWMVTGERQAARIRGLYLKTILRQDIAFFDKETNTGEVIGRMSGDTVLIQDAMGEKVGKFIQLVSTFVGG
FVIAFIKGWLLTLVLLSSIPLLVIAGGAMSITISKMASRGQTAYAKAATVVEQTIGSIRTVASFTGEKQAISNYKK
FLVTAYNSGVHEGFATGFGLGFVLLIIFSTYALAIWSGGKMILHKGYSGGDVLNVMLAVLTGSMSLGQASPCM
SSFAAGQAAAYKMFETINRKPEIDASDLRGKILDDIRGDIEFRNVYFSYPARPDEQIFSGFSLSIPSGTTAALVGQ
SGSGKSTVISLIERFYDPQAGEVLIDGINLKEFQLKWIREKIGLVSQEPVLFTASIRDNIAYGKDGATIDQIRAAAE
LANAAKFIDKLPQGLDTMAGEHGTQLSGGQKQRIAIARAILKDPRILLLDEATSALDAESERVVQEALDRIMVN
RTTVIVAHRLSTIRNADVIAVIHRGKMIEKGSHSELLMDPEGAYSQLIRLQEVNKDSEHAADDHIRPERSSESFR
QSSRRISLHRSISRGSSGVGNNSRHSFSVSFGLPEEQEVSTQEKAPEVPLSRLAYLNKPEIPVLVIGTIAASINGVL
FPIFSILLSRVIKSFYEPPHELRKDTSFWALMFVIIGVVSLLVVPSQYYFFAVAGSRLIQRIRSICFEKVVHMEVGW
FDEPAHSSGAIGARLSADAAKVRALVGDALAQLVQNIASAVAGLVIAFTASWQLALIILVLIPLIGVNGYVQAK
FLQGFSKDAKVLYEEASQVANDAVGSIRTVASFCAEGKVMQLYKKKCEGPMKTGIRTGLVSGTGFGVSFFLM
YSVYAISFYAGAQLVKHGKTTFTDVFQVFFALTMAAVGISQSSSLNPDSAKAKSAAASIFSIIDRKSKIDPSDESG
TISEDVRGEIELRHVSFKYPSRPDIQIFRDLSLAIHSGKTVALVGESGSGKSTVISLLQRFYDPDSGHITLDGVEIQ
SLQIKWLRQQMGLVSQEPVLFNDTIRANIAYGKDGNATEAEIIAASELANAHKFISSLQQGYDTVAGERGVQLS
GGQKQRVAIARAIVKSPKILLLDEATSALDAESERVVQDALDRVMVNRTTVVVAHRLSTIKNADVIAVVKNG
VIAEKGKHETLINIKDGVYASLVALHMSASTA

>XP_012082996.1_ABCB11_X2_Jc
MAKREGVNGLPQGHEASTSTGHEEEEKKSIINGKGNPQDTEQSKGDDKTNSVPFYKLFSFADPTDIILMIVGTIG
AVGNGLSLPIMTILLGDTINAFGQNQNNQDVVEVVSKVSLKFVYMAVGTAVASFLQVTCWMVTGERQAARIR
GLYLKTILRQDIAFFDKETNTGEVIGRMSGDTVLIQDAMGEKVGKFIQLVSTFVGGFVIAFIKGWLLTLVLLSSIP
LLVIAGGAMSITISKMASRGQTAYAKAATVVEQTIGSIRTVASFTGEKQAISNYKKFLVTAYNSGVHEGFATGF
GLGFVLLIIFSTYALAIWSGGKMILHKGYSGGDVLNVMLAVLTGSMSLGQASPCMSSFAAGQAAAYKMFETIN
RKPEIDASDLRGKILDDIRGDIEFRNVYFSYPARPDEQIFSGFSLSIPSGTTAALVGQSGSGKSTVISLIERFYDPQA
GEVLIDGINLKEFQLKWIREKIGLVSQEPVLFTASIRDNIAYGKDGATIDQIRAAAELANAAKFIDKLPQGLDTM
AGEHGTQLSGGQKQRIAIARAILKDPRILLLDEATSALDAESERVVQEALDRIMVNRTTVIVAHRLSTIRNADVI
AVIHRGKMIEKGSHSELLMDPEGAYSQLIRLQEVNKDSEHAADDHIRPERSSESFRQSSRRISLHRSISRGSSGVG
NNSRHSFSVSFGLPEEQEVSTQEKAPEVPLSRLAYLNKPEIPVLVIGTIAASINGVLFPIFSILLSRVIKSFYEPPHEL
RKDTSFWALMFVIIGVVSLLVVPSQYYFFAVAGSRLIQRIRSICFEKVVHMEVGWFDEPAHSSGAIGARLSADA
AKVRALVGDALAQLVQNIASAVAGLVIAFTASWQLALIILVLIPLIGVNGYVQAKFLQGFSKDAKVLYEEASQ
VANDAVGSIRTVASFCAEGKVMQLYKKKCEGPMKTGIRTGLVSGTGFGVSFFLMYSVYAISFYAGAQLVKHG
KTTFTDVFQVFFALTMAAVGISQSSSLNPDSAKAKSAAASIFSIIDRKSKIDPSDESGTISEDVRGEIELRHVSFKY
PSRPDIQIFRDLSLAIHSGKTVALVGESGSGKSTVISLLQRFYDPDSGHITLDGVEIQSLQIKWLRQQMGLVSQEP
VLFNDTIRANIAYGKDGNATEAEIIAASELANAHKFISSLQQGYDTVAGERGVQLSGGQKQRVAIARAIVKSPKI
LLLDEATSALDAESERVVQDALDRVMVNRTTVVVAHRLSTIKNADVIAVVKNGVIAEKGKHETLINIKDGVY
ASLVALHMSASTA

>XP_010474667.1_P_ABCB11-l_X1_Cs
MDRDGAGEGDSVSHEPSTSKSPKEGEEEETTKKEKTDEKTKTVPFYKLFAFADSFDVFLMVCGSIGAIGNGVC
LPLMTLLFGDLIDSFGQNQNNKDIVDVVSKVCLKFVYLGLGTLGAAFLQVACWMITGERQAARIRSTYLKTIL
RQDIGFFDVETNTGEVVGRMSGDTVLIQDAMGEKVGKFIQLVSTFVGGFVLAFIKGWLLTLVMLTSIPLLAMA
GASMALIVTRASSRGQAAYAKAATVVEQTIGSIRTVASFTGERQAVNSYKKFITSAYKSSIQQGFSTGLGLGVM
FFVFFSSYALAIWFGGKMIIEKGYTGGDVINVIIIVVAGSMSLGQTSPCVTAFAAGQPAAYKMFETIKRKPLIDA
YDVNGKVLEDIRGDIELKDVHFSYPARPDEDIFNGFSLFIPSGATAALVGESGSGKSTVISLIERFYDPKAGEVLI
DGVNLKEFQLKWIRSKIGLVSQEPVLFSSSIMENIAYGKDNATLQEIKAATELANAAKFIDKLPQGLDTMVGEH
GTQLSGGQKQRIAIARAILKDPRILLLDEATSALDAESERVVQEALDRVMVNRTTVIVAHRLSTVRNADMIAVI

HRGKMVEKGSHSELLRDAEGAYSQLIRLQEINKDAKPSDISSGSSFRNSNLKKSLEGSVISGGASSVGNSSRHHS
MNVVGLSTGVDLGSSSQEEAGTASKEPLPKVSLTRIAALNKPEIPVLLLGTVAAAINGAIFPLFGILISRVIEAFFK
PADQLKKDSRFWAIIFVALGVTSLIVSPTQMYLFAVAGGKLIRRIRSMCFERAVHMEVGWFDEPQNSSGTMGA
RLSADAALIRALVGDALSLAVQNAASAASGLIIAFTACWELALIILVMLPLIGINGYVQVKFMKGFSADAKSKY
EEASQVANDAVGSIRTVASFCAEEKVMKMYKKQCEGPIKDGIKQGFISGLGFGFSFFILFCVYATSFYAGARLV
EDGKTTFNDVFQVFFALTMAAIGVSQSSSFAPDSSKAKVAAASIFAIIDRKSKIDSSDESGTVLENVKGDIELRHL
SFTYPARPDIQIFRDLCLTIRAGKTVALVGESGSGKSTVISLLQRFYDPDSGHITLDGVELKKLQLKWLRQQMGL
VGQEPVLFNDTIRANIAYGKGSEDAATESEIIAAAELANAHKFISSIQQGYDTVVGERGIQLSGGQKQRVAIARA
IVKEPKILLLDEATSALDAESERVVQDALDRVMVNRTTVVVAHRLSTIKNADVIAVVKNGVIAEKGTHETLIKI
DGGVYASLVQLHMTASN

>XP_010045629.2_P_ABCB11_Eg
MDSSMAIENGAAENSVTSEAATSRSPEVASVKSPAVNENEQDCNKSKGDEKVNSVPFYKLFSFADSTDILLMV
VGSIGAAGNGISTPLMTVLFGTLINTFGENQTDTDVVDLVSKIALKFVYLALGCGAAAFLQVSCWMVTGERQA
ARIRGLYLKTILRQDVAFFDKETNTGEVVGRMSGDTVLIQNATGEKVGTCIQLVSTFVGGFMIAFIKGWLLTLI
MLTMIPLLVIAGGVTSLIISKMASRGQSAYAKAANVVEQTIGSIRMVASFTGEKRAIANYSKFLVDAYRSGVHE
GLAAGLGMGTVMLVIFGGYALAIWCGAKLILDKGYNGGAVINVIMAVLIGSMSLGQASPCMSAFAAGQAAA
YKMFETIQRKPEIDSFDTKGKKLDDIRGDIELRDVYFSYPARPDEQIFNGFSLGIPSGTTAALVGQSGSGKSTVIS
LIERFYDPQDGEVLIDGINLKEFQLKWIRSKIGLVSQEPVLFACSIKDNIAYGKEGATLEEIKAAAELANAAKFID
KLPEGLDTMVGEHGTQLSGGQKQRVAIARAILKDPRILLLDEATSALDTESERIVQEALDRIMGNRTTVIVAHR
LSTVRNANMIAVIHRGKMVEKGSHSELLKDPDGAYSQLIRLQEVNRESEQAPDDQNRSEITEYNRQSNQRMSY
KGSISQRSSIGNSSRHSFSVPFGLPTGLNVADDNVAGPQSPAPGSTEKSPEVSLRRLAHLNKPEAPVLLIGTVAA
VVNGTILPIFGILISSVIKTFYEPPHELREDSKFWALMFLVLGIASFVAFPSRTYLFSVAGCKLIERIRLMCFEKVV
HMEVGWFDEPDHSSGAIGARLSADAASVRALVGDALAQIVQNIASAIAGLVIAFTASWQLALIILALVPLIGVN
GYVQVKFMKGFSADAKMMYEEASQVATDAVGSIRTVASFCAEEKMMQLYKKKCEGPMKTGIRQGLISGIGF
GMSFFLLYCMYATSFYAGAQLVQDGKTTFPDVFRVFFALTMATVGISQSGSIAPDSTKAKAAAASIFAIIDRRS
KIDPSDESGTKLDNVKGEIELRHVSFKYPSRPDIQIFRDLSLAIHFGKTVALVGESGSGKSTVIALLQRFYDPDSG
HITLDGVDIKQLQLKWLRQQMGLVSQEPVLFNETIRANIAYGKDGDATEQEILTASELANAHKFISGLQQGYDT
VVGERGVQLSGGQKQRVAIARAIVKSPKILLLDEATSALDAESEKVVQDALDRVMVNRTTVVVAHRLSTIKN
ADVIAVVKNGVIVEKGNHETLINIKDGFYASLVALHTSASTV

>XP_021664690.1_ABCB11-l_Hb
MAEESSLNVVARTQEASTSKSHEEEEEKKQSINGDSQETKKSKEDQKTNSVPFHKLFSFADSIDILLMIVGTIGA
VGNGISLPLMTVFLGDMIDAFGQNKNKDVVHVVSKVSLKFLYLAVGTAVASFLQVDCWIVTGERQAARIRGL
YLKSILRQDVAFFDKETNTGEVIGRMSGDTVLIQDAMGEKVGKFLQLLSTFVGGFAVAFIKGWLLTIVLLSSIPL
LVLAGASMSITIARMASRGQNAYAKAATVVEQTIGSIRTVASFAGEKQAISNYKKFLVTAYNSGVHEGSATGL
GLGIVMLIIFCSYALAVWFGGKMILEKGYTGGIVINVIIAVLTGSLSLGQASPCMSAFAAGQAAAYKMFETICR
KPAIDAYDTRGKILDDICGDIELRDIHFRYPARPNEQIFSGFSLSIASGTTAALVGQSGSGKSTVISLIERFYDPQA
GEVLIDGINLKEFQLKWIREKIGLVSQEPVLFTVSIRDNIAYGKDGATTEEIRAAAELANAAKFIDKLPQGLDTM
AGEHGTQLSGGQKQRIAIARAILKDPRILLLDEATSALDAESEMIVQEALDRIMVNRTTVIVAHRLSTIRNADVI
AVIHCGKIVEKGSHSELLSDPDGAYSQLIRLQELNKDSERVAEDHKRSDLSPESFRQSSQRISLQRSISQGSSGVG
NSSRHSFSVSFGLPTGINVTENSHEQYDFSPPKGKTPEVPIHRLAYLNKPEIPVLALGTIAACISGVIFPVFGILLSR
VIKSFYEPPHELRKDTKFWSFIFMVLGVTSFFALPSRFYFFGVAGNRLIQRIRTICFEKVVHMEVGWFDDPEHSS
GAIGARLSADAAIVRALVGDALAQLVQNITSAVAGLVIAFTASWQLAFIILVLLPLIGINGYVQVKFLKGFSADA
KMMYEEASQVANDAVGSIRTVASFCAEEKVMQLYKKKCEGPLKTGVRQGLISGIGFGVSFFLMFSVYAASFY
AGAQLVKHGKTTFSDVFQVFFALTMAAIVVSQSSSFAPDSTKAKNAAASIFSIIDRKSKIDPSDESGMTPESVRG
EIEIHHVSFKYPSRSDVQIFRDLNLVIHSGKTMALVGESGSGKSTVISLLQRFYDPDSGHITLDGVEIQRLQLKWL
RQQMGLVSQEPVLFNDTIRANIAYGNGGDATEAEILAASEMANAHKFISSLQQGYDTVVGERGVQLSGGQKQ
RVAIARAIIKSPKILLLDEATSALDAESERVVQDALDRAMVNRTTVVVAHRLSTIKSADVIAVVKNGVIVEKGK
HETLINISDGFYASLVALHMSASTA

>XP_023893066.1_ABCB11-l_X1_Qs
MAVENGLGSQTNTDAATISKSNAEEEKTSNMNGDQEDSKKSKGDEKTNTVPFRKLFSFADSTDILLMILGTVG
AVGNGICMPLMTLLFGELTNSFGQNQNSPNMVDTVSKVCLKFVYLALGVAVAAFLQVACWMVTGERQAARI

RGLYLKTILRQDVAFFDKETNTGEVVSRMSGDTVLIQDAMGEKVGKFLQLTSTFIGSFVIAFIKGWLLTLVMLS
SIPLLVASGAVMSIIISKMASRGQSAYAKAANVVEQTIGSIRTVASFTGEKQAIISYKKFVLKAYNSGVQEGMAS
GFGLGTAMLVVFCTYALAVWFGAKMILQKGYNGGQVLTVIIAVLTGSMSLGQASPCMSAFAAGQAAAFKMF
ETIGRKPVIDAYDTKGRTLDDIHGDIELRDVYFSYPSRPDELIFNGFSLSIPSGTTTALVGQSGSGKSTVISLIERFY
DPHAGEVLIDGINLKEFQLKWIRGKIGLVSQEPVLFASSIKDNIAYGKDGATIEEIRAAAELANAAKFIDKLPQV
LIFSQLDYAVFIIRKLVDLLQYFEFHFQGLDTMVGEHGTQMSGGQKQRIAIARAILKDPRVLLLDEATSALDTES
ERIVQEALDRIMVNRTTVIVAHRLSTVRNVDMIAVIHRGKMVEKGSHSELLKDPEGAYSQLIRLQEVNKESEQ
ALDDRNNPEITVESFRHSTQRMSIQRSISRGSSGVGNSSRHSFSVSFGLPTGVNVPDIARAETESPSVPTEKLPNV
PLSRVAYLNKPEIPVLIIGAVAAILNGVILPVFGLLISSAIKIFFEPNGLKKDSKFWAIMFMLLGLASFLIIPARSYF
FAVAGCKLIGRIRVMCFEKVVNMEVSWFDDPDNSSGAIGARLSADAALVRALVGDALGQMVENMASAVAGL
VIAFVACWQLAFIVLVLIPLIGVNAFIQVKFMKGFSADAKMMYEEASQVANDAVGSIRTVASFCAEENVMELY
KRKCEGPMKTGIRQGLISGMGFGLSFFLLFSVYATSFYAGARLVEAGKTTFSDVFRVFFALTMAAIGISQTSSFA
PDSSKAKNAAASIFAILDRKSKIDPSEESGMKLDDVKGEIELRHVSFKYPSRPDIQIFRDLNLKIHSGKTVALVGE
SGCGKSTVISLLQRFYDPDSGHITLDEIEIQKFQLKWLRQQMGLVSQEPILFNDTIRANIAYGKEGDATEAEIISA
SELANAHKFISSLQQGYDTMVGERGVQLSGGQKQRVAIARAIVKSPKILLLDEATSALDAESEKIVQDALDRV
MVNRTTIVVAHRLSTIKNADLIAVVKNGIIVEKGKHETLINIKDGFYASLVALHTSASTI

>XP_014618641.1_ABCB21_Gm
MDVENGEERKHDDASTSENRAETSTNGEKEEKSKQQEKPETVPFHKLFAFADSTDILLMAVGTIGAIGNGLGL
PLMTLLFGQMIDSFGSNQRNTNVVEEVSKVSLKFVYLAVGSGLAAFLQVTSWMVTGERQAARIRGLYLKTILR
QDVAFFDKETNTGEVIGRMSGDTVLIQDAMGEKVGKFLQLIATFIGGFVIAFIKGWLLTVVMLSTLPLLALSGA
TMAVIIGRMASRGQTAYAKAAHVVEQTIGSIRTVASFTGEKQAVSSYSKFLVDAYKSGVHEGFIAGAGLGTVM
LVIFCGYALAVWFGAKMIMEKGYNGGTVINVIIAVLTASMSLGEASPSLSAFAAGQAAAYKMFQTIERKPEID
AYDPNGKILEDIQGEIELRDVYFSYPARPEELIFNGFSLHIPSGTTAALVGQSGSGKSTVISLVERFYDPQAGEVLI
DGINLKEFQLRWIRGKIGLVSQEPVLFASSIKDNIAYGKEGATIEEIRSASELANAAKFIDKLPQGLDTMVCEHG
TQLSGGQKQRIAIARAILKNPRILLLDEATSALDAESERVVQEALDRIMVNRTTIVVAHRLSTVRNADMIAVIHR
GKMVEKGTHSELLKDPEGAYSQLIRLQEVSKETEGNADQHDKTELSVESFRQSSQKRSLQRSISRGSSLGNSSR
HSFSVSFGLPTGVNVADPELENSQPKEEAPEVPLSRLASLNKPEIPVIVIGSVAAIANGVIFPIFGVLISSVIKTFYE
PFDEMKKDSEFWALMFMILGLASFLIIPARGYFFSVAGCKLIQRIRLMCFEKVVNMEVSWFDEPENSSGAIGAR
LSADAASVRALVGDALGLLVQNFATALAGLIIAFVASWQLALIILVLIPLIGVNGYVQMKFMKGFSADAKMMY
EEASQVANDAVGSIRTVASFCAEDKVMELYKKKCEGPMKTGIRQGLISGSGFGVSFFLLFCVYATSFYAGARL
MDSGKTTFSDVFQVFFALTMAAIGVSQSSSFAPDSSKAKSATASIFGIIDKKSKIDSSDASGSTLDSIKGEIELRHV
SFKYPSRPDMQIFRDLRLTIHSGKTVALVGESGSGKSTVIALLQRFYDPDSGQITLDGVEIRELQLKWLRQQMG
LVSQEPVLFNESLRANIAYGKGGDATEAEIIAAAELANAHKFISGLQQGYDTIVGERGTQLSGGQKQRVAIARA
IIKSPKILLLDEATSALDAESERVVQDALDKVMVNRTTVVVAHRLSTIKNADVIAVVKNGVIVEKGKHEKLINL
SDGFYASLVQLHTSASTV

>XP_006492413.1_ABCB11-l_Csi
MTSETMNGESNSNEASASKSQEEVGKDSSMSGNEHDSEKGKQTEKTESVPFYKLFTFADSADIALMIIGSIGAIG
NGLCLPLMTLLFGDLINTFGDNQNNSETVDKVSKVAVKFVYLGIGSGIASFLQVTCWMITGERQATRIRGLYLK
TILRQDVAFFDNETNTGEVVGRMSGDTVLIQDAMGEKVGKFLQLMATFLGGFLIAFIKGWLLTLVMLSSIPLLA
MSGGVMAIMISKMSSRGQGAYAKAASVVEQTIGSIRTVASFTGEKQAMSNYKKFLVTAYKSGVQEGLAAGIG
LGMVMLIVFCSYALSVWYGGKLILEEGYNGGQVVNVMVAVLTGSMSLGEASPCLSAFGAGQAAAFKMFETI
NRKPEIDAYDTKGKILDDIRGDIELRDVYFSYPARPNEQIFSGFSISISSGTTAALVGQSGSGKSTVISLIERFYDPQ
AGEVLIDGINLKQFQLQWIRKKIGLVSQEPVLFTGSIKDNIAYGKDDATTEEIRVATELANAAKFIDKLPQGIDT
LVGEHGTQLSGGQKQRIAIARAILKDPRILLLDEATSALDAESEKVVQEALDRIMVNRTTVIVAHRLSTVRNAD
MIAVIHRGKIVEKGTHSKLVEDPEGAYSQLIRLQEANKESEQTIDGQRKSEISMESLRHSSHRMSLRRSISRGSSI
GNSSRHSISVSFGLPSGQFADTALGEPAGPSQPTEEVAPEVPTRRLAYLNKPEIPVILAGTIAAMANGVILPIYGL
LISSVIETFFKPPHELKKDSRFWALIYLALGAGSFLLSPAQSYFFAVAGNKLIQRIRSMCFEKVIHMEVSWFDEPE
HSSGAIGARLSADAASVRALVGDALARIVQNISTAAAGLIIAFTASWQLALIILVMLPLIGVSGYTQMKFMKGFS
ADAKMKYEEASQVANDAVGSIRTVASFCAEEKVMQLYKKKCEAPMKTGIRQGMVSGGGFGASFFLLFAFYA
ASFYAGARLVEDGKATFSDVFKVFFSLTMTAIGISQSSSFSSDSNKAKSAAASIFAIIDRESKIDPSDESGTILEDV
KGEIELHHVSFKYPSRPDVQVFRDLNLKIRAGKTVALVGESGSGKSTVVSLLQRFYDPDAGHITLDGVEIQKLQ
LKWLRQQMGLVSQEPVLFNDTIRANIAYGKGGDATEAEIQAASEMANAHKFICSLQQGYDTMVGERGLQLSG

GQKQRVAIARAIVKDPKILLLDEATSALDAESERVVQDALDRVMKNRTTVVVAHRLSTIKNADMIAVVKNGVI
VEKGKHENLINIPDGFYASLIALHSSASTS

>XP_024188759.1_ABCB11-l_X1_Rc
MAEENGVNGDTKNHENHAEEEKSSVPINGDQPGSSDSNGDEKVEKIPFSKLFSFADKTDVILMVVGTIGAIGNG
SCMPLMTVLFGEMIDSFGSNQNSDIVSVVSKVSLKFVYLAVGAAVAAFLQVSCWMVTGERQAARIRGLYLKTI
LRQDVAFFDMETNTGEVVGRMSGDTVLIQDAMGEKVGKVLQLLSTFIGGFIIAFIKGWLLTLVMLSSIPLLVAS
GATMSIIITKMASRGQTAYAKAANVVEQTIGSIRTVASFTGEREAITSYNKYLVDAYKSGVHEGSAAGIGLGLV
MCVVFSSYALAVWFGSKMIRENGYTGGEVLNVIVAVLTGSMSLGQTSPCMSAFAAGQAAAYKMFQTISRKPE
IDAYDEKGKTLGDISGDIELRDVYFSYPARPDEQIFDGFSLCIPSGTTAALVGQSGSGKSTVISLIERFYDPQAGE
VLIDGINLKEFQLKWIRSKIGLVSQEPVLFASSIKENIAYGKDGATSEEIQAAAELANAAKFVDKLPQGLDSLVG
EHGTQLSGGQKQRIAIARAILKDPRILLLDEATSALDAESERVVQEALDRIMVNRTTVVVAHRLSTVRNADMIA
VIHKGKMVEKGSHLNLLKDPEGAYSQLIRLQEVNKESEQTSDDQNKPEITLASLRQSSQRTSSQRLSFARSLSR
NSSVGNSSRHSYSVAFGLPTGLGGIGVQDAAYEETELAPEQPPKVSLRRIAALNKPEIPVLIIGTIAAIINGVILPIF
GVLISRVIKTFYEPPHQQKKDAAFWAIIFMILGLISFVVIPARGYFFSVAGSKLIQRIRLLCFERVVHMEVGWFDE
PENSSGAIGARLSADAASVRALVGDALAQMVQNMAAAVAGLVIAYIACWQLAFIILALLPLIAVNGYVQIKFM
KGFSADAKMMYEEASQVANDAVGSIRTVASFCAEEKVMELYRRKCEGPMKTGIRQGLISGIGFGVSFFFLFCV
YATSFYAGAQLVKAGKTTFTDVFQVFFALTMAATGISQSSSFGPDSGKAKTSAASIFAIIDRPSKIDPSNESGTKI
DGGVKGEIELRHVSFKYPSRPETPIFRDLNLTIRSGKTVALVGESGSGKSTVVALLQRFYDPDSGHITLDGIELG
NYNLKWLRQQMGLVSQEPVLFNDTIRANIAYGKEETATEAEIIAASELANAHKFISTLHQGYDTVVGERGIQLS
GGQKQRVAIARAIIKSPKILLLDEATSALDAESERVVQDALDRVMVNRTTVVVAHRLSTIKNADVIAVVKNGVI
VEKGKHDNLINITDGFYASLVALHMSSSTA

>XP_013666364.1_ABCB11-l_Bn
MDRGEARESDSVSHEPSTSKTPREGEKEDTKKEKNDEKTKTVPFYKLFAFADSIDVFLMICGSVGAMGNGVCL
PLMTLLFGDLIDSFGQNQNNKDIVDVISKVCLKFVYLGLGTLGAAFIQVASWMITGERQAARIRSMYLKTILRQ
DIGFFDVETNTGEVVGRMSGDTVLIQDAMGEKVGKFIQLVSTFVGGFALAFVKGWLLTLVMLTSIPLLAMAG
AAMAIIVTRASSRGQAAYAKAATVVEQTIGSIRTVASFTGEKQAINSYKKFITSAYEQSIKQGFSTGLGLGVMF
MVFFSSYALAIWFGGKMIVEKGYTGGAVINVIIIVVAGSMSLGQTSPCLTAFAAGQAAAYKMFETIKRKPLIDA
YDENGKVLEDIRGDIELKDVHFSYPARPDEDIFNGFSLFIPSGATAALVGESGSGKSTVISLIERFYDPKSGEVLID
GVNLKEFQLKWIRSKIGLVSQEPVLFSSSIRENIAYGKDNATVEEIKAATELANAAKFIDKLPQGLDTMVGEHG
TQLSGGQKQRIAIARAILKDPRILLLDEATSALDAESERVVQEALDRVMVNRTTVIVAHRLSTVRNADMIAVIH
RGKMVEKGSHSELLRDPEGAYSQLIRLQEINKGHDAKTSPGSSFRGSNLKKSMEGGSVISGGTSSVGNSSRHHS
LNVLGLAAGLDLGGGSVSQRVGQEETSQEPAPKVSLTRIAALNKTEIPVLLLGTVAAAINGAIFPLFGILISRVIE
AFFKPADQLKKDSRFWAIIFVALGVTSLIVSPVQTYLFSVAGGKLIRRIRSMCFEKAVHMEVGWFDEPQNSSGT
MGARLSADAALIRALVGDALSLAVQNAASAASGLIIAFTASWELAFIILVMLPLIGINGYIQVKFMKGFTADAK
TKYEDASQVANDAVGSIRTVASFCAEEKVMQMYKKQCEGPIKDGIKQGFISGLGFGVSFFILFSVYATSFYAGA
RLVEAGRTTFNNVFQVFFALTMAAIGISQSSTFAPDSSKAKVAAASIFGIIDRKSKIDSSDESGTVLENVKGDIEL
RHISFTYPARPDIQIFRDLCLSIRAGKTVALVGESGSGKSTVISLLQRFYDPDSGNITLDGVELKSLQLKWLRQQ
MGLVGQEPVLFNDTIRANIAYGKGSEEAATESEIIAAAELANAHKFISSIQQGYDTVVGERGIQLSGGQKQRVAI
AGAIVKEPKILLLDEATSALDAESERVVQDALDRVMVNRTTVVVAHRLSTIKNADVIAVVKNGVIAEKGTHEK
LIKIEGGVYASLVQLHMTASN

>XP_009119625.1_P_ABCB11-l_Br
MDRGEARESDSVSHEPSTSKTPREGEKEDTKKEKNDEKTKTVPFYKLFAFADSIDVFLMICGSVGAMGNGVCL
PLMTLLFGDLIDSFGQNQNNKDIVDVISKVCLKFVYLGLGTLGAAFIQVASWMITGERQAARIRSMYLKTILRQ
DIGFFNVETNTGEVVGRMSGDTVLLQDAMGEKVGKFIQLVSTFVGGFALAFVKGWLLTLVMLTSIPLLAMAG
AAMAIIVTRASSRGQAAYAKAATVVEQTIGSIRTVASFTGEKQAINSYKKFITSAYEQSIKQGFSTGLGLGVMF
MVFFSSYALAIWFGGKMIVEKGYTGGAVINVIIIVVAGSMSLGQTSPCLTAFAAGQAAAYKMFETIKRKPLIDA
YDENGKVLEDIRGDIELKDVHFSYPARPDEDIFDGFSLFIPSGATAALVGESGSGKSTVISLIERFYDPKSGEVLID
GVNLKEFQLKWIRSKIGLVSQEPVLFSSSIRENIAYGKDNATVEEIKAATELANAAKFIDKLPQGLDTMVGEHG
TQLSGGQKQRIAIARAILKDPRILLLDEATSALDAESERVVQEALDRVMVNRTTVIVAHRLSTVRNADMIAVIH
RGKMVEKGSHSELLRDPEGAYSQLIRLQEINKGHDAKTSPGSSFRASNLKKSMEGGSVISGGTSSVGNSSRHHS
LNVLGLAAGLDLGGGSVSQRVGQEETSQEPVPKVSLTRIAALNKTEIPVLLLGTVAAAINGAIFPLFGILISRVIE

AFFKPADQLKKDSRFWAIIFVALGVTSLIVSPVQTYLFSVAGGKLIRRIRSMCFEKAVHMEVGWFDEPQNSSGT
MGARLSADAALIRALVGDALSLAVQNAASAASGLIIAFTASWELAFIILVMLPLIGINGYIQVKFMKGFTADAK
TKYEDASQVANDAVGSIRTVASFCAEEKVMQMYKKQCEGPIKDGIKQGFISGLGFGVSFFILFSVYATSFYAGA
RLVEAGRTTFNNVFQVFFALTMAAIGISQSSTFAPDSSKAKVAAASIFGIIDRKSKIDSSDESGTVLENVKGDIEL
RHISFTYPARPDIQIFRDLCLSIRAGKTVALVGESGSGKSTVISLLQRFYDPDSGNITLDGVELKSLQLKWLRQQ
MGLVGQEPVLFNDAIRANIAYGKGSEEAATESEIIAAAELANAHKFISSIQQGYDTVVGERGIQLSGGQKQRVAI
ARAIVKEPKILLLDEATSALDAESERVVQDALDRVMVNRTTVVVAHRLSTIKNADVIAVVKNGVIAEKGTHEK
LIKIEGGVYASLVQLHMTASN

>XP_006444609.2_ABCB11_X1_Cc
MTSETMNGESNSNEASASKSQEEVGKDSSMSGNEHDSEKGKQTEKTESVPFYKLFTFADSADTALMIIGSIGAI
GNGLCLPLMTLLFGDLINTFGDNQNNSETVDKVSKVAVKFVYLGIGSGIASFLQVTCWMITGERQATRIRGLYL
KTILRQDVAFFDNETNTGEVVGRMSGDTVLIQDAMGEKVGKFLQLMATFLGGFLIAFIKGWLLTLVMLSSIPLL
AMSGGVMAIMISKMSSRGQGAYAKAASVVEQTIGSIRTVASFTGEKQAMSNYKKFLVTAYKSGVQEGLAAGI
GLGMVMLIVFCSYALSVWYGGKLILEEGYNGGQVVNVMVAVLTGSMSLGEASPCLSAFGAGQAAAFKMFET
INRKPEIDAYDTKGKILDDIRGDIELRDVYFSYPARPNEQIFSGFSISISSGTTAALVGQSGSGKSTVISLIERFYDP
QAGEVLIDGINLKEFQLQWIRKKIGLVSQEPVLFTGSIKDNIAYGKDDATTEEIRVATELANAAKFIDKLPQGID
TLVGEHGTQLSGGQKQRIAIARAILKDPRILLLDEATSALDAESEKVVQEALDRIMVNRTTVIVAHRLSTVRNA
DMIAVIHRGKIVEKGTHSKLVEDPEGAYSQLIRLQEANKESEQTIDGQRKSEISMESLRHSSHRMSLRRSISRGSS
IGNSSRHSISVSFGLPSGQFADTALGEPAGPSQPTEEVAPEVPTRRLAYLNKPEIPVILAGTIAAMANGVILPVYG
LLISSVIETFFKPPHELKKDSRFWALIYVALGAGSFLLSPAQSYFFAVAGNKLIQRIRSMCFEKVIHMEVSWFDEP
EHSSGAIGARLSADAASVRALVGDALARIVQNISTAAAGMIIAFTASWELALIVLVMLPLIGVSGYTQMKFMK
GFSADAKMKYEEASQVANDAVGSIRTVASFCAEEKVMQLYKKKCEAPMKTGIRQGMVSGGGFGASFFLLFAF
YAASFYAGARLVEDGKATFSDVFKVFFSLTMTAIGISQSSSFSSDSNKAKSAAASIFAIIDRESKIDPSDESGTILE
DVKGEIELHHVSFKYPSRPDVQVFRDLNLKIRAGKTVALVGESGSGKSTVVSLLQRFYDPDAGHITLDGVEIQK
LQLKWLRQQMGLVSQEPVLFNDTIRANIAYGKGGDATEAEIQAASEMANAHKFICSLQQGYDTMVGERGLQL
SGGQKQRVAIARAIVKDPKILLLDEATSALDAESERVVQDALDRVMKNRTTVVVAHRLSTIKNADMIAVVKN
GVIVEKGKHENLINIPDGFYASLIALHSSASTS

>XP_017980811.1_P_ABCB21_Tc
MATENGLKGDTDFKVASTSKSCKEPEKGCSMNGENKKSETGKPDEKTNTVPFYKLFAFADPTDILLMIVGTIG
AIGNGLSMPLMTILFGELTDSFGENQNNNEVVDVVSEVALKFVHLGVGAAVAGFLQVTCWMVTGERQAARIR
GLYLKTILRQDVAFFDVETNTGEVVGRMSGDTVLIQDALGEKVGKFLQLMSLFIGGFVVAFIKGWLLTLVLLS
SIPFLVIAGAVMSLIIANTASQGQNAYAKAATVVEQTIGSIRTVASFTGEKQAISDYNKFLVSAYKSGVYQGSVS
GLGLGMVMLIMFCSYALAVWFGGKMILEKGYTGGQVVSVIVAIMTGSMSLGQASPCINAFASGQAAAFKMFE
TIERKPNIDAYDTKGKILEDIRGDIDFRDVCFSYPARPEEQIFSGFSLSIPSGTTAALVGQSGSGKSTVISLIERFYD
PQAGEVLIDGINLKDFQLNWIRGKIGLVSQEPVLFTSSIRDNIAYGKEGATSEEIIAAAELANAAKFIDKLPQGLD
TMVGEHGTQLSGGQKQRVAIARAILKDPRILLLDEATSALDAESERTVQEALDRIMVNRTTVIVAHRLSTVRN
AQMIAVVHQGKMVEKGSHSELLKDPDGAYSQLIRLQEVNKQLEQAPDDKNGSDITEKLLRQSSLEISLKKSICR
GSTGLPNGQNVADSVLTDAAAPPTIPSVQASEVSICRLASLNKPEIPVLLIGTIASIVNGLILPTYALLLSEVIKTFY
KPPNELKTDSRFWALIFMALGLASLLAYPTETYLFSIAGCKLIRXVRSMCFEKVVHMEIGWFDEPEHSSGSLGA
RLSADAATLRALVGDALSQMVQSIVSAVAGLIIAFVASWQLALIVLALFPLIGIDGYIQVKFMKGFSADAKMM
YEEASQVANDAVGVFGGIRTISSFCAEEKVMRLYKKKCERPMKTGIRRGLISGIGFGLSFFLMYSVYAASFYAG
AQLVEHGHATFSDVFQVFFALTMAAVAITQSSSFGSDSSKAKTAAASIFSIIDRKSKIDPGDESGVTLENVKGEIE
LCHVSFKYPLRPDIQIFQDLCLAIPAGKTVALVGESGSGKSTVISLLQRFYDLDSGHITLDGVEIQKLQLKWLRQ
QMGLVSQEPVLFNDTIRANIAYGKGGNATEAEILAAAELANAHKFISSLQQGYDTVVGERGVQLSGGQKQRV
AIARAIIKSPKILLLDEATSALDAESEQVVQDALDRVMVNRTTVVVAHRLSTIKNADVIAVVRNGVIVEKGKHE
TLINIKDGSYASLVALHLSASTA

>XP_004152829.2_P_ABCB4-l_Csa
MEIENGVDGKANNIDQPSSSRANETEKSSNKNANQEDLKSKNGDGKTNSVPFYKLFSFADSTDVLLMIFGTIGA
IGNGLSLPLMTIVFGELTDSFGVNQSNTDIVKVVSKVCLKFVYLAIGCGAAAFIQVASWMVTGERQASRIRGLY
LKTILRQDVSFFDMETNTGEVVERMSGDTVLIQDAMGEKVGKCIQLVSTFFGGFIIAFIKGWLLTLVMLSSLPLL
VISGGITSVIITKMTSRGQSAYAKAADVVEQTISSIRTVASFTGEKQAVSNYKKFLVNAYRSGVQEGLAVGVGF

GTIFAVLFFSYSLAIWYGAKLVLDKGYTGGEVLNVVIAVLTGSMSLGQASPCLSAFAAGRAAAFKMFETIKRIP
LIDAYDMKGKTLDDITGDIELKDVHFSYPTRPNENIFNGFSLKIPSGTTAALVGQSGSGKSTVISLIERFYDPSMG
EVLIDGINLKEFQLKWIRSKIGLVSQEPVLFASSIKDNIAYGKDGATMEEIKAAAELANASKFIDKLPQGLDTLV
GAHGTQLSGGQKQRVAIARAILKDPRILLLDEATSALDAESEHVVQEALDRIMVNRTTVIVAHRLSTVRNAEMI
AVIHKGKMVEKGSHTELLKDPEGPYSQLIKLQEVNQESQEAGIDKVKQESISGSFRRYSKGVLMARSISRGSSG
VGNSSRHSFSVSFGLPAGVPITDVPMADESASVDTKERSPPVPLRRLALLNKPEIPILVLGSVAAIINGVILPLFGL
IFANAIETFYKPPDKLKKDSRFWALIMMLLGIASLVAAPARTYFFSVAGCKLIQRIRLLCFQNIVNMEVGWFDR
TENSSGSIGARLSANAATVRALVGDALSQLVENLAAVTAGLVIAFASSWQLAFIVLAMFPLLGLNGYVQMKFL
KGFSADAKLMYEQASQVATDAVGSIRTVASFCAEEKVMLLYKKKCEGPMKAGIRQGLISGTGFGVSFFLLFSV
YAATFFAGAHFVQDGKATFSDIFRVFFALTMAAFAISQSSSLAPDSTKAKEATASIFSMIDRKSEINPSVETGETL
ENFKGEIEFRHVSFKYPSRPDVQILRDLSLTIRSGKTVALVGESGCGKSTVISLLQRFYDPDSGSITLDGIEIHKFQ
VKWLRQQMGLVSQEPILFNDTIRANIAYGKGGDATETEIIAAAELSNAHKFISSLHQGYDSMVGERGAQLSGG
QKQRVAIARAIIKSPKILLLDEATSALDAESERVVQDALDKVMVNRTTIVVAHRLSTVKNADIIAVVKNGVIVE
KGKHDSLINIKDGFYASLVQLHTNASSSAA

>XP_021279931.1_ABCB21-l_X1_Hu
MATENDLKVDTDFKEASTSKSCKEPEKGCRMNGENQKSATGKPDEKTNTVPFYKLFAFADPTDILLMIVGTIG
AIGNGLSMPLMTILFGELIDSFGENQNNNEVVDVVSEVALKFVYLGVGAAVAGFLQVTCWMVTGERQAARIR
GLYLKTILRQDVAFFDVETNTGEVVGRMSGDTVLIQDAMGEKVGKFLQLMSLFIGGFVVAFIKGWLLTLVLLS
SIPFLVIAGAAMSLIIANTASQGQNAYAKAATVVEQTIGSIRTVASFTGEKQAISDYNKFLVSAYKSGVYQGSVS
GLGLGMVMLIMFCSYALAVWFGGKMILEKGYTGGQVVSVILAIMTGSMSLGQASPCINAFASGQAAAFKMFE
TIERKPNIDAYDTKGKILEDIRGDIDFRDVCFSYPARPEEQIFSGFSLSIPSGTTAALVGQSGSGKSTVISLIERFYD
PQAGQVLIDGINLKEFQLNWIRGKIGLVSQEPVLFTSSIRDNIAYGKEGATSEEIIAAAELANAAKYIDKLPQGTG
TMVGEHGTQLSGGQKQRVAIARAILKDPRILLLDEATSALDAESERTVQEALDRIMVNRTTVIVAHRLSTVRN
AQMIAVVHQGKMVEKGSHSELLKDPDGAYSQLIRLQEVNKQLQQVPDDKNGSDITEKLFRQSSLEISLKKSIST
GSTGLPNGQNVADSVLTDAEAPPTIPSVQAPEVSICRLASLNKPEIPVLLMGAIASIVNGFILPTYSLLLSEVIKTF
YKPPNELKTDSRFWALIFMALGLASLLAYPTETYLFSVAGCKLIRRVRSMCFEKVVHLEIGWFDEPEHSSGSLG
ARLSADAATLRALVGDALSQMVQSIVSAVAGLIIAFVASWQLALIVLALFPLIGIDGYIQVKFMKGFSADAKM
MYEEASQVANDAVGGIRTISSFCAEEKVMRLYKKKCERPMKTGIRRGLISGIGFGLSFFLMYSVYAASFYAGA
QLVEHGHATFSDVFQVFFALTIAAVAITQSSSLGSDSSKAKTAAASIFSIIDRKSKIDPGDESGVTLENVKGEIELC
HVSFKYPLRPDIQIFQDLCLAIPAGKTVALVGESGSGKSTVISLLQRFYDLDSGHITLDGVEIQKLQLKWLRQQM
GLVSQEPVLFNDTIRANTAYGKGGNATEAEILAAAELANAHRFISSLQQGYDTVVGERGVQLSGGQKQRVAIA
RAIIKSPKILLLDEATSALDAESEQVVPDALDRVMVNRTTVVVAHRLSTIKNADVIAVVRNGVIVEKGKHETLI
NIKDGSYASLVALHLSTSTA

>XP_013697204.1_ABCB11_Bn
MNRDGAGEGDSVSHEPSTSKTPREGGEEETKKDEKAKTVPFYKLFAFADSYDVLLMICGSVGAMGNGVGLPL
MTLLFGDLIDSFGQNQNNKDIVDVISKVCVKFVYLGIGTLGAAFLQVACWMITGERQAARIRNMYLKTILRQDI
GFFDVETNTGEVVGRMSGDTVLIQDAMGEKVGKFIQLIATFIGGFALAFAKGWLLTLVMLTSIPLLAMAGAAM
AIIVTRASSQGQAAYAKAATVVEQTIGSIRTVASFTGEKEAINKYKKFITSAYKSSIQQGFSTGLGLGIMLFVLFS
SYALAIWFGGKMILEKGYTGGAVINVLIIVVAGAMSLGQTSPCVTAFSAGQSAAYKMFETIERKPLIDAYDLKG
KILEDIRGDIELKDVHFSYPARPDEDIFDGFSLFIPSGATAALVGESGSGKSTVISLIERFYDPKAGQVLIDGVNLK
EFQLKWIRSKIGLVSQEPVLFSSSIMENIAYGKENATIQEIKAATELANAAKFIDKLPQGLDTMVGEHGTQLSGG
QKQRIAIARAILKDPRILLLDEATSALDAESERVVQEALDRVMVNRTTVIVAHRLSTVRNADMIAVIHRGKMVE
KGSHSELLRDPEGAYSQLIRLQEINKDAKTSDAASGSSFRNSSLKKSIEGSSSSVGNSSRHHSLSVVTSGLEHGG
GSSRAGLEDKPGTEAQEPVPKVSLSRIAALNKPEIPVLLLGTVAAAINGAIFPLFGILISRVIEAFFKPAHELRRDS
KFWALIFVALGVVSFIVSPTQMYLFAVAGGKLIRRIRSMCFEKAVHMEVGWFDEPQNSSGTLGARLSADAALI
RALVGDALSLAVQNAASAASGLIIAFTACWELALIILVMLPLIGINGYIQVKFMKGFTADAKSKYEDASQVAND
AVGSIRTVASFCAEEKVMQMYKKQCEGPIKDGIKQGFISGLGFGFSFFILFCVYAASFYAGARLVEAGRTTFND
VFQVFFALTMAAIGISQSSSFAPDSSKAKVAAASIFGIIDRKSKIDSSDETGTVLENVKGDIELRHISFTYPARPDI
QIFRDLCLTIRAGKTVALVGESGSGKSTVISLLQRFYDPDSGHITLDGIELKKMQLKWLRQQMGLVGQEPVLFN
DTIRANIAYGKGSEEAATESEIIAAAELANAHKFISSIQQGYDTVVGERGIQLSGGQKQRVAIARAIVKEPKILLL
DEATSALDAESERVVQDALDRVMVNRTTVVVAHRLSTIKNADVIAVVKNGVIAEKGTHETLIKIEGGVYASLV
QLHMTASN

>XP_027099867.1_ABCB11-l_Car
MTGDEESGSPLSGHPNTNQLPAAATTQGQIHATTAAAANGDLVATTQQHKEEPTTTTTTTNTVPFLKLFSFADS
TDMFLMIIGTIGAIGNGLCLPLMTVFFGELTDSFGQTPNTKDVVRVVSKVSLKFVYLALGSAVAGFLQVSCWMI
TGERQAARIRSLYLKTILRQDVGFFDKETNTGEVVGRMSGDTVLIQDAMGEKVGKFIQLVSTFIGGFVIAFAKG
WLLTLVMLSSIPPLVIAGGLMSLVISRMASHGQEAYAKAAIVVEQTIGSIRTVASFTGEKQAVADYDKSLTKAY
RSGVHEGLATGLGLGSVMCLVFCSYALAIWFGAKMIAEKKNTGGEVLNVIIAVLSGSMSLGQASPCMTAFASG
RAAAFKMFETINRTPEIDAYDASGKTLDDIRGDIELKDVYFSYPARPDEQIFSGLSVFIPSGHTAALVGQSGSGK
STVISLIERFYDPQAGEVLIDGTNLKDFQLKWIREKIGLVSQEPVLFTASIKDNIAYSKERVTIEQIRAAAELANA
AKFIDKLPKGLDTMVGEHGTQLSGGQKQRIAIARAILKDPRILLLDEATSALDAESERIVQEALDRIMVNRTTVI
VAHRLSTVRNADMIAVIHRGKIIEKGTHSELTNDPEGAYSQLIRLQDINRDTEEYIEEKDKSDITIESSRQSSQRM
SLKRSISRGSSVGNSSRRSITVSFGLPTGLTMSEHTVAEPDVNTQDITSKPSKVSMRRLASLNKPEIPVIFVGVIAA
VANGAILPTFGILISSVIKAFYKSPHELKKDSRFWALIFMALGVASLLAYPSRTYLFGVAGCKLIRRIRSMCFEKV
VHMEVGWFDESEHSSGVIGAKLSADAASVRALVGDALAQLVQDTSSTIVGLAIAFDASWQLALIILAMLPLIGL
NGYVQIKFMKGFSADAKMMYEEASQVANDAVGSIRTVASFCAEEKVMELYKKKCEGPMKTGIRQGLISGIGF
GLSFALLFCVYAASFYAGARLVEDGKITFSDVFRVFFALTMAAMAISQSSSIAPDSSKAKGAAASIFAILDRKSK
IDASDESGMTLESVNGEIELQRVSFRYPSRPDVQIFRDLSLKIRSGKTVALVGESGSGKSTVISLLQRFYDPESGH
ITLDGIEIQKFQVKWLRRQMGLVSQEPVLFNDTIRANIAYGKEGNATEGEIIEAAKLANAHKFVSGLQQGYETI
VGERGVQLSGGQKQRVAIARAIVKGPKVVLLDEATSALDAESERIVQDALDQVMVNRTTVVVAHRLSTIKGA
DVIAVVKNGVIVEKGKHDALVNIKDGVYASLVALHMNANANANAPAASSSSS

>XP_022899172.1_ABCB4-l_X1_Oe
MTYSCSCSNKLEAVNTMAVENNEGEASTSGSGSHGGQKDSPKDSTNSIPFYELFTFADSYDKILMIVGTIGAIG
NGLCLPLMSILFGELIDSFGQTQTTNIVSVVSKVALKFVYLALGCGVAAFLQVAFWMITGERQAARIRSLYLKTI
LRQDVAFFDKETNTGEVVGRMSGDTVLIQDAIGEKVGKFIQLVSTFFGGFVIAFIQGWLLTLVMLTSIPLLVISG
AVMSIVLSKMASRGQNAYAKAAIVVEQTIGSIRTVASFTGEKQAVADYDKSLAEAYKSGVNEGWASGLGLGS
VMFIIFCSYALAIWYGAKMILEKGYSGGDVLSVIVAVLTGSMSLGQASPCMSAFAAGQAAAFKMFETINRKPEI
DAYDTKGKILDDICGDIELRDVYFSYPARPNEEIFKGLSLFIPSGTTAALVGQSGSGKSTVISLIERFYDPRAGEV
LVDGINLKEFQLKWIRSKIGLVSQEPVLFTASIKDNIAYGKDGATHEEIRAAAELANASKFIDKLPQGLDTMVG
EHGTQLSGGQKQRVAIARAILKDPRILLLDEATSALDAESERIVQEALDRIMINRTTVVVAHRLSTVRNANMIA
VIHQGKMVEKGTHFDLLQDSEGAYSQLIRLQDIYKDFDQHVDEKDKSDITLESGRRSSQKMSIQYSISRGSSGD
GNSNRHSLSVTFGLPATLSVPETELAVTDTASHKTSEKHSKVPIRRLAYLNKPEVPILIVGAIFAIVNGAIMPTFGI
LLSSVIKTFFETPHKLRKDSKFWALMFVVLGAVSLVAYPARTYLFGVAGNKLIRRIRLLCFEKVVSMEVGWFD
EPEHSSGIIGARLSADAATVRALVGDSLAQIVQDTAAAVVGLGIAFEASWQLALIIVGMVPLIGLNGYVQIKFM
KGFSADAKAMYEEASQVANDAVGSIRTVASFCAEEKVMNMYKYKCEGPMRNGIRQGLISGIGFGLSFSLLFLV
YATSFYAGAQLVEDGKTTFSNVFRVFFALTMAAVAISQSSSFAPDSSKAKTAAASIFAMLDRKSKIDPSDEAGT
TLESVKGEIELKHVSFKYPTRPDIQIFRDLSLAIHSGKTVALVGESGSGKSTVISLLQRFYDPDSGHITLDGIEIQK
FQLKWLRLQMGLVSQEPVLFNDTIRANIAYGKDGNASEAEIIAASELANAHKFISGLQQGYDTIVGERGIQLSG
GQKQRVAIARAIIKSPKIILLDEATSALDAESERIVQDALDRVMVNRTTVIIAHRLSTIKGADLIVVVKNGVIVEK
GKHETLINVKDGFYKSLVSLHMSASS

>XP_019235162.1_P_ABCB11-l_Na
MAEGNGLDGNTGLNGASSSSGNRASQTVADTNAGQQDSDKTKQSESTNTVPFYKLFSFADSTDKVLMIIGTIA
AIGNGLSMPIMTILFGELTDSFGQNQNNKDVLRVVSRVSLKFVYLALGCGAAAFLQVAFWMISGERQAARIRS
LYLKTILQQDIAFYDKETNTGEVVGRMSGDTVLIQDAMGEKVGKFVQLISTFIGGFVISFAKGWLLTLVMLSVI
PLLVISGGVMSLVLSKMASRGQDAYARAATVVEQTIGSIRTVASFTGEKQAVANYNKSLIKAYHSGANEGLAT
GLGLGSLFSIIYCSYALAIWFGARLILEKGYTGGQVLNVIIAVLTASMSLGQASPCMSAFAAGQAAAFKMFETIK
RKPEIDAYDTNGKILDDIRGDIELNDVSFSYPARPDEQIFSGFSLFVPSGTTAALVGQSGSGKSTVISLIERFYDPQ
AGQVLIDGINLKDFQLKWIRGKIGLVSQEPVLFMASIKENIAYGKHDATAEEIKAAVELANAAKFIDKLPQGLD
TMVGEHGTQLSGGQKQRIAIARAILKDPRILLLDEATSALDAESERVVQEALDRIMINRTTIIVAHRLSTIRNAD
MIAVIHRGKVVEKGTHHELLKDPEGAYSQLIRLQEVNKETEQSGLNERDRLDKSMGSGGQSSQRMSLLRSVSR
SSSGIGNSSRHSLSISYGLPTGLSVPETANADTETGIQEVSGKPLKVPIRRLAYLNKPELPVIIIGAVAAIINGTLLPI
FGILFSSVVKTFYEPPHQLRKDSKFWALMFVVLGVVTLIAFPARTYLFSIAGCKLIRRIRSMCFEKVVRMEVGW
FDESEHSSGMIGARLSADAAKVRALVGDSLAQMVQDSATAIAGLAIAFEASWQLALIILAMIPLIGLNGYVQIK

FMKGFSADAKMMYEEASQVANDAVGGIRTVASFCAEEKVMEIYRRKCEGPLKAGIKQGLISGIGFGVSFALLF
LVYATSFYAGAHLVQDGKITFSDVFRVFFALTMAAIGISQSSSLAPDSSKAKDAAASIFAILDRKSKIDPSDDSG
MTLDTVKGDIELQHVSFKYPTRPDIQIFRDLCLTIRSGKTVALVGESGCGKSTVVSLLQRFYDPDSGQVTLDGIE
IQKFQVKWLRQQMGLVSQEPVLFNDTIRANIAYGKEGNATEAEIIAAAELANAHKFISGLQQGYDTTVGERGT
QLSGGQKQRVAIARAIVKNPKILLLDEATSALDAESERLVQDALDRVMVNRTTVVVAHRLSTIKGADVIAVVK
NGVIVEKGKHETLINIKDGFYASLVALHMRAS

>XP_019091644.1_P_ABCB11_X3_Cs
MDRDGAGERDSVSHEPSTSKSPKEGEEEETKKEKTDEKTKTVPFYKLFAFADSFDVFLMVCGSIGAIGNGVCLP
LMTLLFGDLIDSFGKNQNNKDIVDVVSKVCLKFVYLGLGTLGAAFLQVACWMITGERQAVRIRSAYLKTILRQ
DIGFFDVETNTGEVVGRMSGDTVLIQDAMGEKVGKFIQLVSTFVGGFVLAFIKGWLLTLVMLTSIPLLAMAGA
SMALIVTRASSRGQAAYAKAATVVEQTIGSIRTVASFTGEKQAVNSYKKFITSAYKSSVQQGFSTGLGLGVMFF
VFFSSYALAIWFGGKMIIEKGYTGGSVINVIIIVVAGSMSLGQTSPCVTAFAAGQAAAYKMFETIKRKPLIDAYD
VNGKVLEDIRGDIELKDVHFSYPARPDEDIFNGFSLFIPSGATAALVGESGSGKSTVISLIERFYDPKAGEVLIDG
VNLKEFQLKWIRSKIGLVSQEPVLFSSSIMENIAYGKDNATLQEIKAATELANAAKFIDKLPQGLDTMVGEHGT
QLSGGQKQRIAIARAILKDPRILLLDEATSALDAESERVVQEALDRVMVNRTTVIVAHRLSTVRNADMIAVIHR
GRMVEKGSHSELLKDSEGAYSQLIRLQEINKGQDAKPSDISSGSSFRNSNLKKSIEGSVISGGTSSVGNSSRHRSL
NVLGLSAGVDLGSSSQRVGQEEAGTASKEPLPKVSLTRIAALNKPEIPVLLLGTVAAAINGAIFPLFGILISRVIEA
FFKPADELKKDSRFWAIIFVALGVTSLIVSPTQMYLFAVAGGKLIRRIRSMCFERAVHMEVGWFDEPQNSSGTM
GARLSADAALIRALVGDALSLAVQNVASAASGLIIAFTACWELALIILVMLPLIGINGYVQVKFMKGFSADAKS
KYEEASQVANDAVGSIRTVASFCAEEKVMQMYKKQCEGPIKDGIKQGFISGLGFGFSFFILFCVYATSFYAGAR
LVEDGKTTFNDVFQVFFALTMAAIGVSQSSSFAPDSSKAKVAAASIFAIIDRKSKIDSSDESGTVLENVKGDIELR
HLSFTYPARPDIQIFRDLCLTIRAGKTVALVGESGSGKSTVISLLQRFYDPDSGHITLDGVELKKLQLKWLRQQM
GLVGQEPVLFNDTIRANIAYGKGSEDAATESEIIAAAELANAHKFISSLQQGYDTVVGERGIQLSGGQKQRVAI
ARAIVKEPKILLLDEATSALDAESERVVQDALDRVMVNRTTVVVAHRLSTIKNADVIAVVKNGVIAEKGTHET
LIKIDGGVYASLVQLHMTASNCDIYTF

>XP_019441256.1_P_ABCB21-l_La
MASENGLDEATTTNNLPPADTNGEKSKQKEKLETVPFHKLFSFADSTDILLITVGTIGAIGNGLGLPLMTLLFGQ
MIDTFGSNQTTEHVVEEVSKVSLKFVYLAVGSGLAAFLQVSCWMVTGERQAARIRGLYLKTILRQDVAFFDKE
TNTGEVIGRMSGDTVLIQDAMGEKVGKFLQLIATFIGGFVIAFVKGWLLTLVMMSTLPLLVVSGATMAVIIGR
MASRGQTAYAKAAHVVEQTIGSIRTVASFTGEKQAVSSYSKFLVDAYKSGVHEGSIAGAGLGTVMFVIFCGYA
LAVWFGAKMIMEKGYNGGTVINVIIAVLTASMSLGQASPSMSAFAAGQAAAFKMFQTIERKPEIDAYDPNGKT
LEDIKGEIELREVYFRYPARPDELIFNGFSLHIPSGTTAALVGQSGSGKSTVISLVERFYDPQKGEVLIDGINLKEF
QLRWIRGKIGLVSQEPVLFASSIKDNIAYGKEGAKIEEIRIATELANAANFIDKLPQGLDTMVGEHGTQLSGGQK
QRIAIARAILKNPRILLLDEATSALDAESERVVQEALDRIMVNRTTIVVAHRLSTVRNADMIAVIHRGKMVEKG
THSELLGDPEGAYSQLIRLQEVNKETEETADHRNKSELSSESFRQSSQRRSLGRSISRGSSAGNSSHRSFSVSFGL
PTGVNVADTEPESSQAEEKSPEVPLWRLASLNKPEIPVLLMGCAAAVANGVILPIFGLLISSVIKTFYEPFDELKK
DSKFWSIMFMILGLASFVIIPARSYFFSVAGCKLIQRIRHICFEKVVNMEVGWFDESENSSGAIGARLSADAASV
RALVGDALGLLIGNLATALAGLIIAFTASWELALIILVLIPLIGLNGYVQMKFMKGFSADAKMMYEEASQVAN
DAVGSIRTVASFCSEDKVMELYRKKCEGPMKTGIRQGLISGSGFGASFFLLFCVYATSFYAGARLVEAGKTKFS
DVFRVFFALTMAAIGVSQSSSFAPDSSKAKSATASIFGIIDKKSKIDPSDESGCTLDNIKGEIELRHIRFKYPSRPDI
QIFRDLNLTIHSGKTVALVGESGSGKSTVIALLQRFYDPDSGEITLDGVEIRQLQLKWLRQQMGLVSQEPVLFN
ETIRANIAYGKGGNATEAEIIAAAEMANAHRFISGLQQGYDTIVGERGTQLSGGQKQRVAIARAIIKSPKILLLD
EATSALDAESERVVQDALDKVMVNRTTVVVAHRLSTIKNADVIAVVKNGVIVEKGRHETLINVKDGFYASLV
QLHTSAATV

>XP_017229562.1_P_ABCB21-l_Dc
MAAMKGLNGGSTELCIAPTSGVRNLENEAGNSNEDQKKAPEVIKVVPFNKLFSFADSMDVILMIVGTVGSVAN
GLCMPLMSVLIGELTDAFGQNQNNNEVVDKVSQVSLKFVYLAAGAGIASFLQVACWMVTGERQAARIRSLYL
KTILRQDVSFFDVETNTGEVVGRMSGDTVLIQDAMGEKVGKFTQLVATFIAGFAVAFFKGWLLTLVMLSSIPP
LVIAGGMMSLLISKMSSRGQEAYAKAAIVVEQTIGSIRTVASFTGEKQAVADYAKSLINAYKSGVGEGVATGF
GFGTLFSVLFYSYALAVWFGAKMILEKGYTGGDVLNVIVAVLTGSMSLGQASPCLSAFAAGRAAAYKMFETI
NRKPEIDAYDTRGKKLEDIQGNIDLRDVYFSYPARPDEQIFNGFSLSIPSGTTAALVGQSGSGKSTVISLIERFYD

PQAGEVLIDGTNLKEFQLKWIRERIGLVSQEPVLFASSIKDNIAYGKDGATMEEIRAATELANAARFIDKLPQGL
DTMVGEHGTQLSGGQKQRVAIARAILKNPRILLLDEATSALDAESERIVQEALDRIMVNRTTVIVAHRLSTVRN
ANMIAVIHRGKMVEKGSHSELLEDPEGAYSQLIRLQEINTEGAGGKDKSETSADGRSLSQRMSSQRSISRDSAG
LGNSSRRSLSVSFNLHTGPSFTEVTLAEPESPSGKALEQAQKVPLRRLMYLNKPELPILVVGSIAAILNGVILPIFG
IVLASMIKIFYEPPHELRKDSKFWALMFVVLGLATFIAYPSQTYFFSVAGCKLIRRIRHMCFEKVVTMEVGWFD
KPENSSGAIGARLSADAASVRALVGDTLAQVVQNGASAVAGLVIAFAACWQLAFIVLAMLPLIGLNGYVQMK
FMTGFSADAKLMYEEASQVANDAVGSIRTVASFCAEEKVMELYKQKCEGPMRTGIRQGLISGIGFGVSFTLLF
CVYATCFYAGAQLVDSGKTTFNEVFRVFFALTMASVGVSQSSSLTPNSSKAKSATASIFAILDRKSEIDPTNESG
ETLENARGEIELRHVSFKYPTRPDVQIFRDLNLTLRSGKTVALVGESGSGKSTVIALLERFYDPDSGHITLDGVEI
QKLQLKWLRQQMGLVSQEPALFNDTIRANIAYGKEGGATEAEIIAAAEMANAHKFISGLAQGYDTIVGERGTQ
LSGGQKQRVAIARAIVKSPKILLLDEATSALDAESERVVQDALDHVMVNRTTVVVAHRLSTIKNADVIAVVKN
GVIAEKGKHEALININDGVYASLVALHMSAGK

>XP_022899174.1_ABCB4-l_X3_Oe
MAVENNEGEASTSGSGSHGGQKDSPKDSTNSIPFYELFTFADSYDKILMIVGTIGAIGNGLCLPLMSILFGELIDS
FGQTQTTNIVSVVSKVALKFVYLALGCGVAAFLQVAFWMITGERQAARIRSLYLKTILRQDVAFFDKETNTGE
VVGRMSGDTVLIQDAIGEKVGKFIQLVSTFFGGFVIAFIQGWLLTLVMLTSIPLLVISGAVMSIVLSKMASRGQN
AYAKAAIVVEQTIGSIRTVASFTGEKQAVADYDKSLAEAYKSGVNEGWASGLGLGSVMFIIFCSYALAIWYGA
KMILEKGYSGGDVLSVIVAVLTGSMSLGQASPCMSAFAAGQAAAFKMFETINRKPEIDAYDTKGKILDDICGDI
ELRDVYFSYPARPNEEIFKGLSLFIPSGTTAALVGQSGSGKSTVISLIERFYDPRAGEVLVDGINLKEFQLKWIRS
KIGLVSQEPVLFTASIKDNIAYGKDGATHEEIRAAAELANASKFIDKLPQGLDTMVGEHGTQLSGGQKQRVAIA
RAILKDPRILLLDEATSALDAESERIVQEALDRIMINRTTVVVAHRLSTVRNANMIAVIHQGKMVEKGTHFDLL
QDSEGAYSQLIRLQDIYKDFDQHVDEKDKSDITLESGRRSSQKMSIQYSISRGSSGDGNSNRHSLSVTFGLPATL
SVPETELAVTDTASHKTSEKHSKVPIRRLAYLNKPEVPILIVGAIFAIVNGAIMPTFGILLSSVIKTFFETPHKLRK
DSKFWALMFVVLGAVSLVAYPARTYLFGVAGNKLIRRIRLLCFEKVVSMEVGWFDEPEHSSGIIGARLSADAA
TVRALVGDSLAQIVQDTAAAVVGLGIAFEASWQLALIIVGMVPLIGLNGYVQIKFMKGFSADAKAMYEEASQ
VANDAVGSIRTVASFCAEEKVMNMYKYKCEGPMRNGIRQGLISGIGFGLSFSLLFLVYATSFYAGAQLVEDGK
TTFSNVFRVFFALTMAAVAISQSSSFAPDSSKAKTAAASIFAMLDRKSKIDPSDEAGTTLESVKGEIELKHVSFK
YPTRPDIQIFRDLSLAIHSGKTVALVGESGSGKSTVISLLQRFYDPDSGHITLDGIEIQKFQLKWLRLQMGLVSQE
PVLFNDTIRANIAYGKDGNASEAEIIAASELANAHKFISGLQQGYDTIVGERGIQLSGGQKQRVAIARAIIKSPKII
LLDEATSALDAESERIVQDALDRVMVNRTTVIIAHRLSTIKGADLIVVVKNGVIVEKGKHETLINVKDGFYKSL
VSLHMSASS

>XP_009778876.1_P_ABCB21-l_Ns
MAERNGLDGNTGLNGASSSSGSRASQTVADTNAGQQDSDKTKQPESTNTVPFYKLFSFADSTDKVLMIIGTIA
AIGNGLSLPIMTILFGELTDSFGQNQNNKDVLRVVSRVSLKFVYLALGCGAAAFLQVAFWMISGERQAARIRSL
YLKTILQQDIAFYDKETNTGEVVGRMSGDTVLIQDAMGEKVGKFVQLMATFIGGFVISFTKGWLLTLVMLSVI
PLLVISGGVMSVILSKMASRGQDAYARAATVVEQTIGSIRTVASFTGEKQAVANYNKSLVKAYQSGANEGLAS
GLGLGSVFAIIYCSYALAIWFGARLILEKGYTGGQVLNVIIAVLTASMSLGQASPCMTAFAAGQAAAYKMFETI
KRKPEIDAYDTNGKILDDIRGDIELNDVSFSYPARPDEQIFSGFSLFVSSGTTAALVGQSGSGKSTVISLIERFYDP
QAGQVLIDGINLKDFQLKWIRGKIGLVSQEPVLFTASIKENIAYGKHDATAEEIKAAVELANAAKFIDKLPQGL
DTMVGEHGTQLSGGQKQRIAIARAILKDPRILLLDEATSALDAESERVVQEALDRIMINRTTIIVAHRLSTIRNA
DMIAVIHRGKVVEKGTHHELLEDPQGAYSQLIRLQEVNKETEQSGLNERERLDKSMGSGRQSSKTMSLLRSVS
RSSSGIGNSSRHSLSISYGLPTGVSVPETANADTETGIQEVSGKPLKVPIRRLAYLNKPEVPVIIIGAVAAIINGTLL
PIFGILFSSAIKTFYEPPHQLRKDSKFWALMFVVLGAVTLIAFPTRTYLFSIAGCKLIRRIRSMCFEKVVRMEVGW
FDESEHSSGMIGARLSADAAKVRALVGDSLAQMVQDSASAIAGLAIAFEASWQLALIILAMIPLIGLNGYVQIK
FMKGFSADAKMMYEEASQVANDAVGGIRTVASFCAEEKVMKIYRRKCEGPLKAGIKQGLISGIGFGVSFALLF
LVYATSFYAGAHLVQDGKITFSDVFRVFFALTMAAIGISQSSSLAPDSSKAKDAAASIFAILDRKSKIDPSDDSG
MTLDTVKGDIELQHISFKYPTRPDVQIFRDLCLTIRSGKTVALVGESGCGKSTVVSLLQRFYDPDSGQVTLDGIE
IQKFQVKWLRQQMGLVSQEPVLFNDTIRANIAYGKEGNATEAEIIAAAELANAHKFISGLQQGYDTTVGERGT
QLSGGQKQRVAIARAIVKNPKILLLDEATSALDAESERLVQDALDRVMVNRTTVVVAHRLSTIKGADVIAVVK
NGVIVEKGKHETLINIKDGFYASLVALHTRAS

>XP_027182519.1_ABCB11-l_Ce

MTGDEESGSPLSGHPNTNQLPAAATTQGQIHATTAAAANGDLVATTQQHKEEPTTTTTTTNTVPFLKLFSFADS
TDMFLMIIGTIGAIGNGLCLPLMTVFFGELTDSFGQTPNTKDVVRVVSKVSLKFVYLALGSAVAGFLQVSCWMI
TGERQAARIRSLYLKTILRQDVGFFDKETNTGEVVGRMSGDTVLIQDAMGEKVGKFIQLVSTFIGGFVIAFAKG
WLLTLVMLSSIPPLVIAGGLMSLVISRMASHGQEAYAKAAIVVEQTIGSIRTVASFTGEKQAVADYDKSLTKAY
RSGVHEGLATGLGLGSVMCLVFCSYALAIWFGAKMIAEKKNTGGEVLNVIIAVLSGSMSLGQASPCMTAFASG
RAAAFKMFETINRTPEIDAYDASGKTLDDIRGDIELKDVYFSYPARPDEQIFSGLSVFIPSGHTAALVGQSGSGK
STVISLIERFYDPQAGEVLIDGTNLKDFQLKWIREKIGLVSQEPVLFTASIKDNIAYSKERVTIEQIRAAAELANA
AKFIDKLPKGLDTMVGEHGTQLSGGQKQRIAIARAILKDPRILLLDEATSALDAESERIVQEALDRIMVNRTTVI
VAHRLSTVRNADMIAVIHRGKIIEKGTHSELTNDPEGAYSQLIRLQDMNRDTEEYIEEKDKSDITIESSRQSSQR
MSLKRSISRGSSVGNSSRRSITVSFGLPTGLTMSEHTVAEPDVNTQDITSKPSKVSMRRLASLNKPEIPVIFVGVI
AAVANGAILPTFGILISSVIKAFYKSPHELKKDSRFWALIFMALGVASLLAYPSRTYLFGVAGCKLIRRIRSMCFE
KVVHMEVGWFDESEHSSGVIGAKLSADAASVRALVGDALAQLVQDTSSTIVGLAIAFDASWQLALIILAMLPL
IGLNGYVQIKFMKGFSADAKMMYEEASQVANDAVGSIRTVASFCAEEKVMELYKKKCEGPMKTGIRQGLISGI
GFGLSFALLFCVYATSFYAGARLVEDGKITFSDVFRVFFALTMAAMAISQSSSIAPDSSKAKGAAASIFAILDRK
SKIDASDESGMTLESVNGEIELQRVSFRYPSRPDVQIFRDLSLKIRSGKTVALVGESGSGKSTVISLLQRFYDPDS
GHITLDGIEIQKFQVKWLRRQMGLVSQEPVLFNDTIRANIAYGKEGNATEGEIIEAAKLANAHKFVSGLQQGYE
TIVGERGVQLSGGQKQRVAIARAIVKGPKVVLLDEATSALDAESERIVQDALDQVMVSRTTVVVAHRLSTIKG
ADVIAVVKNGVIVEKGKHDALVNIKDGVYASLVALHMNANANANAPAASSSSS

>XP_025012434.1_ABCB4-l_Rco
MDKENGLNGNTSTHEAGTSKSHEEEKEKKSSTNGLPQDTEKSKGDEKTNSVPFHKLFSFADSKDVILMIIGTIA
AIGNGLALPLMTIVLGDIIDAFGQNQNQDVVKVVSKVSLRFVYLAIGAAAASFLRXSKCSNLLSYARKSILYQA
CGLRNSVCCXNTGEVIGRMSGDTVLIQDAMGEKVGKFIQLVSTFLGGFVIAFVKGWLLTFVMLSSIPLLVIAGG
VMSITISKMASRGQTAYAKAATVVEQTIGSIRTVASFTGEKQAISNYKKFLVTAYNSGVHEGIATGVGLGVLM
LVVFCSYSLAIWFGGKMILEKGYTGGQVLNVIIAVLSGSMSLGQASPCMSAFAAGQAAAYKMFETISRMPEID
AYDTRGKILEDIRGDIELRDVYFSYPARPEEQIFSGFSLSIPSGTTTALVGQSGSGKSTVISLIERFYDPQAGEVRID
GINLKEFQLKWIREKIGLVSQEPVLFTASIRDNIAYGKDGATTEEIRSAAELANAAKFIDKLPQGLDTMAGEHGT
QLSGGQKQRIAIARAILKDPRILLLDEATSALDAESERVVQEALDRIMVNRTTVIVAHRLSTIRNADVIAVIHRG
KMVEKGSHSELLMDPDGAYSQLIRLQEVNKDSEQKPEDHKRSDLSSESFRQSSQRISLRRSISRGSSGVGNSSRH
SFSVSFGLPTGINATDNPQEEPTDSPSPENTPEVPIRRLAYLNKPEIPVLIFGAIAACANGVIFPIYGILLSRVIKSFY
EPPHELRKDTNFWALIFMTLGLASFVVIPLQFYFFGVAGSRLIQRIRTICFEKVVHMEVGWFDEPEHSSGAIGAR
LSADAATVRALVGDSLAQMVQNLASAVAGLVIAFTASWQLAFIILALIPLIGVTGYVQVKFMQGFSADAKMM
YEEASQVANDAVGSIRTVASFCAEEKVMQMYKKKCEGPMKTGIRQGVISGMGFGASFFLLFSVYATSFYAGA
QLVKHGKTSFSDVFQVFFALTMAAMGISQSSSLAPDSSKARSAVASIFSIIDRQSKIDPSDESGMTIENVRGEIEL
RRVSFRYPSRPDIQIFRDLNLAIHSGKTVALVGESGSGKSTVISLLQRFYDPDSGHITLDGVEIQRLQLKWLRQQ
MGLVSQEPVLFNDTIRANIAYGKDGDATEAETLAASELANAHKFISSLQQGYDTLVGERGVQLSGGQKQRVAI
ARAIVKSPKILLLDEATSALDAESERVVQDALDRVMVNRTTIVVAHRLSTIQNADVIAVVKNGVIVEKGKHETL
INIKDGFYASLVSLHTTASTA

>XP_010457237.2_P_ABCB11_X1_Cs
MDRDGAGERDSVSHEPSTSKSPKEGEEEETKKEKTDEKTKTVPFYKLFAFADSFDVFLMVCGSIGAIGNGVCLP
LMTLLFGDLIDSFGKNQNNKDIVDVVSKVCLKFVYLGLGTLGAAFLQVACWMITGERQAVRIRSAYLKTILRQ
DIGFFDVETNTGEVVGRMSGDTVLIQDAMGEKVGKFIQLVSTFVGGFVLAFIKGWLLTLVMLTSIPLLAMAGA
SMALIVTRASSRGQAAYAKAATVVEQTIGSIRTVASFTGEKQAVNSYKKFITSAYKSSVQQGFSTGLGLGVMFF
VFFSSYALAIWFGGKMIIEKGYTGGSVINVIIIVVAGSMSLGQTSPCVTAFAAGQAAAYKMFETIKRKPLIDAYD
VNGKVLEDIRGDIELKDVHFSYPARPDEDIFNGFSLFIPSGATAALVGESGSGKSTVISLIERFYDPKAGEVLIDG
VNLKEFQLKWIRSKIGLVSQEPVLFSSSIMENIAYGKDNATLQEIKAATELANAAKFIDKLPQGLDTMVGEHGT
QLSGGQKQRIAIARAILKDPRILLLDEATSALDAESERVVQEALDRVMVNRTTVIVAHRLSTVRNADMIAVIHR
GRMVEKGSHSELLKDSEGAYSQLIRLQEINKGQDAKPSDISSGSSFRNSNLKKSIEGSVISGGTSSVGNSSRHRSL
NVLGLSAGVDLGSSSQRVGQEEAGTASKEPLPKVSLTRIAALNKPEIPVLLLGTVAAAINGAIFPLFGILISRVIEA
FFKPADELKKDSRFWAIIFVALGVTSLIVSPTQMYLFAVAGGKLIRRIRSMCFERAVHMEVGWFDEPQNSSGTM
GARLSADAALIRALVGDALSLAVQNVASAASGLIIAFTACWELALIILVMLPLIGINGYVQVKFMKGFSADAKS
KYEEASQVANDAVGSIRTVASFCAEEKVMQMYKKQCEGPIKDGIKQGFISGLGFGFSFFILFCVYATSFYAGAR
LVEDGKTTFNDVFQVFFALTMAAIGVSQSSSFAPDSSKAKVAAASIFAIIDRKSKIDSSDESGTVLENVKGDIELR

HLSFTYPARPDIQIFRDLCLTIRAGKTVALVGESGSGKSTVISLLQRFYDPDSGHITLDGVELKKLQLKWLRQQM
GLVGQEPVLFNDTIRANIAYGKGSEDAATESEIIAAAELANAHKFISSIQQGYDTVVGERGIQLSGGQKQRVAIA
RAIVKEPKILLLDEATSALDAESERVVQDALDRVMVNRTTVVVAHRLSTIKNADVIAVVKNGVIAEKGTHETLI
KIDGGVYASLVQLHMTASNCDIYTF

>XP_009593037.1_P_ABCB11-l_Nto
MAEGNGLDGNTGLNGASSSSENRAPQTVADTNAGQQDSDKTKQSESTNTVPFYKLFSFADSTDKVLMIIGTIA
AIGNGLSLPIMTILFGELTDSFGQNQNNKDVLRVVSRVSLKFVYLALGCGAAAFLQVAFWMISGERQAARIRSL
YLKTILQQDIAFYDKETNTGEVVGRMSGDTVLIQDAMGEKVGKFVQLISTFIGGFVISFTKGWLLTLVMLSVIP
LLVISGGVMSLILSKMASRGQDAYARAATVVEQTIGSIRTVASFTGEKQAVANYNKSLIKAYQSGASEGLATG
LGLGSLFSIIYCSYALAIWFGARLILEKGYTGGQVLNVIIAVLTASMSLGQASPCMTAFAAGQAAAFKMFETIKR
KPEIDAYDTNGKILDDIRGDIELKDVYFSYPARPDEQIFSGFSLFVPSGTTAALVGQSGSGKSTVISLIERFYDPQA
GQVLIDGINLKDFQLKWIRGKIGLVSQEPVLFTASIKENIAYGKHNATAEEIKAAVELANAAKFIDKLPQGLDT
MVGEHGTQLSGGQKQRIAIARAILKDPRILLLDEATSALDAESERVVQEALDRIMINRTTIIVAHRLSTIRNADMI
AVIHRGKVVEKGTHHELLKDPEGAYSQLIRLQEVNKETEQSGLNERDRLDKSMGSGGQSSQRMSLLRSVSRSS
SGIGNSSRHSLSISYGLPTGLSVPETANADTETGIQEVSGKPLKVPIRRLAYLNKPEVPVIIIGAVAAIINGTLLPIF
GILFSSVIKTFYEPPHQLRKDSKFWALMFVLLGAVTLIAFPARTYLFSIAGCKLIRRIRSMCFEKVVHMEVGWFD
ESEHSSGMIGARLSADAAKVRALVGDSLAQMVQDSASAIAGLAIAFEASWQLALIILAMIPLIGLNGYVQIKFM
KGFSADAKMMYEEASQVANDAVGGIRTVASFCAEEKVMEIYRRKCEGPLKAGMKQGLISGIGFGVSFALLFL
VYATSFYAGAHLVQDGKITFSDVFRVFFALTMAAIGISQSSSLAPDSSKAKDAAASIFAILDRKSKIDPSDDSGM
TLDTVKGDIELQHVSFKYPTRPDVQIFRDLCLTIRSGKTVALVGESGCGKSTVVSLLQRFYDPDSGQVTLDGIEI
QKFQVKWLRQQMGLVSQEPVLFNDTIRANIAYGKEGNATEAEIIAAAELANAHKFISGLQQGYDTTVGERGTQ
LSGGQKQRVAIARAIVKNPKILLLDEATSALDAESERLVQDALDRVMVNRTTVVVAHRLSTIKGADVIAVVKN
GVIVEKGKHETLINIKDGFYASLVALHTRAS

>XP_023546098.1_ABCB11-l_X2_Cp
MEIENGVDGNSNSIDHQPSSSRTNGIEKSSNKNGNQQDLKNKNGDGKTNSVPFYKLFSFADSTDVLLMIVGSIG
AIGNGLSLPLMTILFGELTDSFGGNQNSSDIVKVVSKVCLKFVYLAIGCGVAAFIQVASWMVTGERQASRIRGL
YLKTILRQDVSFFDMETNTGEVVERMSGDTVLIQDAMGEKVGKCIQLVSTFTGGFVIAFIKGWLLTLVMLSSLP
LLVISGGITSIVITKMTSRGQGAYAKAADVVEQTISSIRTVASFTGEKHAVNSYKKYLVDAYRSGVQEGSAVGI
GFGMIFAVLFFSYSLAIWYGAKLILDKGYSGGAVLNVVVAVLTGSMSLGQASPCLSAFAAGRAAAFKMFETIK
RKPLIDAYDTKGKTLDDISGDIELRDVHFSYPTRPDEHIFKGFSLRITSGTTAALVGQSGSGKSTVISLIERFYDPS
MGEVLIDGINLKEFQLKWIRSKIGLVSQEPVLFASSIRDNIAYGKDGATIEEIKAAAELANASKFIDKLPQGLDTL
VGAHGTQLSGGQKQRVAIARAILKDPRILLLDEATSALDAESEHVVQEALDRIMVNRTTVIVAHRLSTVRNAD
MIAVIHKGKMVEKGSHTELLKDPEGPYSQLIRLQEVNQESQEAGIDKVKQESTSGSFRRYSKGASIRRSVSRGSS
GVGNSSRHSFSVSFGLPAAVPITDVPMADESAPENTMERSPPVPLRRLAYLNKPEIPILALGSVAAVINGMILPLF
GLLFANAIETFYKPPDKLKKDSRFWALIMMLLGIASLIVAPAKTYLFSVAGCKLIQRIRLLCFEKIVNNEVGWFD
RTENSSGSIGGRLSANAATVRALVGDALSQLVENLASVTAGLVIAFASSWQLALIVLAMFPLLGMNGYVQMK
FMKGFSADAKLMYEQASQVATDAVGSIRTVSSFCAEEKVMQLYKKKCEGPMKSGIRQGLISGTGFGVSFFLLF
SVYAATFYAGAHFVKDGKATFSDVFRVFFALTMAAFAISQSSSLAPDSSKAKEATASIFSMIDRKSEIDPSVETG
ETLENLKGEIEFRHVSFKYPSRPDVQILRDLSLSIRSGKTVALVGESGCGKSTVISLLQRFYDPDSGSITLDGIEIQ
KFQLKWLRQQMGLVSQEPILFNDTIRANIAYGKSGDATEAEIIAASELSNAHKFISGLQQGYDSTVGERGAQLS
GGQKQRVAIARAIIKSPKILLLDEATSALDAESERVVQDALDKVMVNRTTIVVAHRLSTVKNADIIAVVKNGVI
VEKGRHDTLINIKDGFYASLVQLHTKASTSSA

>XP_018452062.1_P_ABCB11_Rs
MNCEGSGEGDSGSHEPSTSKTPKEGEKEDTKKEKTDEKTKTVPFYKLFAFADSLDVFLMICGSVGAVGNGVCL
PFMTLLFGDLIDSFGKNQNNKDIVDVVSKVCLKFVYLGLGTLGAAFLQVASWMITGERQAARIRSMYLKTILR
QDIGFFDVETNTGEVVGRMSGDTVLIQDAMGEKVGKFIQLVSTFVGGFALAFVKGWLLTLVMLTSIPLLAMA
GASMAIIVTRASSRGQAAYAKAATVVEQTIGSIRTVASFTGEKQAINSYKKFITSAYKQSIQQGFSTGLGLGVMF
MVFFSSYALAIWFGGKMIVEKGYTGGAVINVIIIVVAGSMSLGQTSPCLTAFAAGQAAAYKMFETIKRKPLIDA
YDVNGKVLEDIRGDIELKDVHFSYPARPDEDIFNGFSLFIPSGSTAALVGESGSGKSTVISLIERFYDPKSGEVLID
GVNLKEFQLKWIRSKIGLVSQEPVLFSSSIMENIAYGKENATVEEIKAAAELANAAKFVDKLPQGLDTMVGEH
GTQLSGGQKQRIAIARAILKDPRILLLDEATSALDAESERVVQEALDRVMVNRTTVIVAHRLSTVRNADMIAVI

HRGKMVERGSHSELLRDPEGAYSQLIRLQEINKGQTSSEIASGSSFRASNLKKSMEGSSVLSGGTSSVGNSSRHH
SLNVLGLAAGLDFGGGSVSQRVGQEESVQEPVQKVSLTRIAALNKTEIPVLLLGTVAAAINGAIFPLFGILISRVI
EAFFKPADELKKDSRFWAIIFVALGVTSLIVSPVQTYLFSVAGGKLIRRIRSMCFEKAVHMEVGWFDEPQNSSG
TMGARLSADAALIRALVGDALSLAVQNAASAASGLIIAFTASWELAFIILVMLPLIGINGYIQVKFMKGFTADA
KTKYEDASQVANDAVGSIRTVASFCAEEKVMQMYKKQCEGPIKDGIKQGFISGLGFGVSFFILFSVYATSFYAG
ARLVEAGRTTFNNVFQVFFALTMAAIGISQSSTFAPDSSKARVAAASIFGIIDRKSKIDSSDESGTVLENVKGDIE
LRHISFTYPARPDIQIFRDLCLSIRAGKTVALVGESGSGKSTVISLLQRFYDPDSGNITLDGVELKSLQLKWLRQQ
MGLVGQEPVLFNDTIRANIAYGKGSEEAATESEIIAAAELANAHKFISSIQQGYDTVVGERGIQLSGGQKQRVAI
ARAIVKEPKILLLDEATSALDAESERVVQDALDRVMVNRTTVVVAHRLSTIKNADVIAVVKNGVIAEKGTHEK
LIRIEGGVYASLVQLHMTASN

>XP_013637891.1_P_ABCB11_Bo
MNGGEAREGASVSHEPSTSKTPREGEKEDTKKEKNDEKTKTVPFYKLFAFADSIDVFLMICGSVGAMGNGVCL
PLMTLLFGDLIDSFGQNQNNKDIVDVISKVCLKFVYLGLGTLGAAFLQVASWMITGERQAARIRSMYLKTILRQ
DIGFFDVETNTGEVVGRMSGDTVLIQDAMGEKVGKFIQLVSTFVGGFALAFVKGWLLTLVMLTSIPLLAMAG
AAMAIIITRASSRGQAAYAKAATVVEQTIGSIRTVASFTGEKQAINSYKKYITSAYNQSIKQGFSTGLGLGVMF
MVFFSSYALAIWFGGKMIVEKGYTGGAVINVIIIVVAGSMSLGQTSPCLTAFAAGQAAAYKMFETIKRKPLIDA
YDVNGKVLEDIRGDIELKDVHFSYPARPDEDIFNGFSLFIPSGATAALVGESGSGKSTVISLIERFYDPKSGEVLI
DGVNLKEFQLKWIRSKIGLVSQEPVLFSSSIRENIAYGKDNATVEEIKAATELANAAKFIDKLPQGLDTMVGEH
GTQLSGGQKQRIAIARAILKDPRILLLDEATSALDAESERVVQEALDRVMVNRTTVIVAHRLSTVRNADMIAVI
HRGKMVEKGSHSELLRDPEGAYSQLIRLQEINKGHDAKTSPGSSFRASNLKKSMEGGSVMSGGTSSVGNSSRH
HSLNVLGLAAGLDLGGGGVSQRVGQEETSQEPVPKVSLTRIAALNKTEIPVLLLGTVAAAINGAIFPLFGILISR
VIEAFFKPADQLKKDSRFWAIIFVALGVTSLIVSPVQTYLFSVAGGKLIRRIRSMCFEKAVHMEVGWFDEPQNSS
GTMGARLSADAALIRALVGDALSLAVQNAASAASGLIIAFTASWELAFIILVMLPLIGINGYIQVKFMKGFTAD
AKTKYEDASQVANDAVGSIRTVASFCAEEKVMQMYKKQCEGPIKDGIKQGFISGLGFGVSFFILFSVYATSFYA
GARLVEAGRTTFNDVFQVFFALTMAAIGISQSSTFAPDSSKAKVAAASIFGIIDRKSKIDSSDESGTVLENVKGDI
ELRHISFTYPARPDIQIFRDLCLTIRAGKTVALVGESGSGKSTVISLLQRFYDPDSGNITLDGVELKSLQLKWLRQ
QMGLVGQEPVLFNDTIRANIAYGKGSEEAATESEIIAAAELANAHKFISSIQQGYDTVVGERGIQLSGGQKQRV
AIARAIVKEPKILLLDEATSALDAESERVVQDALDRVMVNRTTVVVAHRLSTIKNADVIAVVKNGVIAEKGTH
EKLIKIEGGVYALLVQLHMTASN

>XP_009111196.1_P_ABCB11_Br
MNRDGAGEGDSVSHEPSTSKTPREGGEEETKKDEKAKTVPFYKLFAFADSYDVLLMICGSVGAMGNGVGLPL
MTLLFGDLIDSFGQNQNNKDIVDVISKVCVKFVYLGIGTLGAAFLQVACWMITGERQAAKIRNMYLKTILRQD
IGFFDVETNTGEVVGRMSGDTVLIQDAMGEKVGKFIQLIATFIGGFALAFAKGWLLTLVMLTSIPLLAMAGAA
MAIIVTRASSQGQAAYAKAATVVEQTIGSIRTVASFTGEKEAINKYKKFITSAYKSSIQQGFSTGLGLGIMLFVLF
SSYALAIWFGGKMILEKGYTGGAVINVLIIVVAGAMSLGQTSPCVTAFSAGQSAAYKMFETIERKPLIDAYDLK
GKILEDIRGDIELKDVHFSYPARPDEDIFDGFSLFIPSGATAALVGESGSGKSTVISLIERFYDPKAGQVLIDGVNL
KEFQLKWIRSKIGLVSQEPVLFSSSIMENIAYGKENATIQEIKAATELANAAKFIDKLPQGLDTMVGEHGTQLSG
GQKQRIAIARAILKDPRILLLDEATSALDAESERVVQEALDRVMVNRTTVIVAHRLSTVRNADMIAVIHRGKM
VEKGSHSELLRDPEGAYSQLIRLQEINKDAKTSDAASGSSFRNSSLKKSIEGSSSSVGNSSRHHSLNVVASGLER
GGGSSRAGLEDKTGTEAQEPVPKVSLTRIAALNKPEIPVLLLGTVAAAINGAIFPLFGILISRVIEAFFKPAHELRR
DSKFWALIFVALGVVSFIVSPTQMYLFAVAGGKLIRRIRSMCFEKAVHMEVGWFDEPQNSSGTLGARLSADAA
LIRALVGDALSLAVQNAASAASGLIIAFTACWELALIILVMLPLIGINGYIQVKFMKGFTADAKSKYEDASQVA
NDAVGSIRTVASFCAEEKVMQMYKKQCEGPIKDGIKQGFISGLGFGFSFFILFCVYAASFYAGARLVEAGRTTF
NDVFQVFFALTMAAIGISQSSSFAPDSSKAKVAAASIFGIIDRKSKIDSSDETGTVLENVKGDIELRHISFTYPARP
DIQIFRDLCLTIRAGKTVALVGESGSGKSTVISLLQRFYDPDSGHITLDGIELKKMQLKWLRQQMGLVGQEPVL
FNDTIRANIAYGKGSEEAATESEIIAAAELANAHKFISSIQQGYETVVGERGIQLSGGQKQRVAIARAIVKEPKIL
LLDEATSALDAESERVVQDALDRVMVNRTTVIVAHRLSTIKNADVIAVVKNGVIAEKGTHETLIKIDGGVYAS
LVQLHMTASN

>XP_002515186.1_ABCB11_Rco
MAVENGVHSDISTHETSTSKGLEEKDKSARANGHPQEMEKSNGEEKTNSVPFHKLFSFADSVDIVLMIIGTIGA
LGNGLSMPLMTIFLGDTIDAFGNNQNNQDVVDIVSKVSLKFVYLGIGSSVASFLQVVCWMVTGERQAARIRGL

YLKTILRQDIAFFDKETNTGEVIGRMSGDTVLIQDAMGEKVGKFLQLLSTFIGGFLIAFVKGWLLTLVMLSSLPL
LVLAGAAMSIMIARTASHGQNAYAKAATVVEQTIGSIRTVASFTGEKQAIRNYEKYLVAAYHSGAHEGLITGL
GLGLFVFILFSSYALAIWYGGKMILEKGYTGGEVINVILVVLTGSTSLGQASPCMSAFAAGQAAAYKMFETIGR
KPEIDAYDTSGKVSDDVHGSIELKEVYFSYPARPDEQIFSGFSLSIPSGMTAALVGQSGSGKSTVISLVERFYDPQ
SGEVLIDGINLKEYQLKWIRGKIGLVSQEPVLFTSSIRDNIAYGKDEATTEEIRAAAELANAAKFIDKLPQGLDT
MVGEHGTQLSGGQKQRIAIARAILKDPRILLLDEATSALDAESERIVQEALDRIMVNRTTVIVAHRLTTIRNAD
MIAVIHRGKIVEKGSHSELLADPDGAYAQLIRLQEVNEDSEEAVDERKRSEISLESLSSQRNSLQRSISRGSSGAG
NSHRHSLSVPSGLRTGLNVSENSLAEPEVSLQKKQTPEVPIRRLAYLNKPEIPELIAGSIGAIIHGVIFPLFGILISRV
IEAFFKPPHELRKDSKFWAIIFVIVAVVSFLACNAQLYFFAVAGSKLIQRIRSMCFEKVVHMEVGWFDVPEHSS
GAIGARLSADAASVRSLVGDSLAQMVQNIASAVAGLVIAFTASWQLAFIILVIVPLTGLNAYVQLEFLKGFSAD
AKMMYEEASQVANDAVGSIRTVASFCAEEKVMQLYRKKCEGPLKTGIRQGLVSGIGFGVSFFLLFSVYATSFY
AGAQLVKHGKATFTDVFQVFFALTVAAMGISQSSSFAPDSSKAKTAVASIFSILDRKSKIDPSDESGMTLENVR
GDIEFQHVTFRYPSRPDIQIFQDLSLSIHSGKTVALVGESGSGKSTAISLLQRFYDPDSGHITLDGVEIQRLQLKW
LRQQMGLVSQEPVLFNETIRANIAYGKDGNASEAEILAASELANSHEFISSLQQGYDTLVGERGVQLSGGQKQR
VAIARAIVKTPKILLLDEATSALDAESERVVQDALDRVMLKRTTVVVAHRLSTIQNADVIAVVKNGAIIEKGKH
ETLIHISNGFYASLVALHVSASTA

>XP_007145097.1_Pv
MGVENDEERKHDDTSTSENRAGTSTNGEREEKSKQKEKPETVPFFKLFAFADSTDILLMVVGTIGAIGNGMGL
PIMTLLFGEMIDSFGSNQQNPNVVEAVSKVSLKFVYLAVGSGMAAFLQVTSWMVTGERQAARIRGLYLKTILR
QDIAFFDKETSTGEVVGRMSGDTVLIQDAMGEKVGKFLQLMATFVGGFTIAFIKGWLLTCVMMATLPLLVLS
GAAMAVIIGKMASRGQTAYAKASHVVEQTIGSIRTVASFTGEKQAVNSYSKFLVDAYRSGVSEGSLAGVGLGT
VMLVIFGGYALAVWFGAKMIMEKGYNGGTVINVIISFLTASMSLGQASPSLSAFAAGQAAAYKMFQTIERKPE
IDAYDPNGKILEDIQGEIDLRDVYFSYPARPEELIFNGFSLHIASGTTAALVGQSGSGKSTVISLVERFYDPQAGE
VLIDGINLKEFQLRWIRGKIGLVSQEPVLFASSIKDNIAYGKEGATIEEIRSASELANAAKFIDKLPQGLNTMVGE
HGTQLSGGQKQRIAIARAILKNPRILLLDEATSALDAESERIVQEALDRIMVNRTTVVVAHRLSTVRNADMIAVI
HRGKMVENGTHSELLKDPEGAYSQLIRLQEISKETEQNADHVGKSELSSESLRQSSQRKSLQRSISRGSSLGNSS
RHSFSVSFGLPTAVNVSDPEHESSMPKEKEVPLHRLASLNKPEIPVLLLGSVAAIINGVILPIFGLLISSAIKTFYEP
FDKMKKDSHFWALMFLTLGIVSFFIIPARGYFFSVAGSKLIQRIRLMCFEKVVNREVGWFDEPENSSGSIGARLS
ADAASVRALVGDALGLLVQNLASAVAGLIIAFVASWQLALIILVLIPLIGVNGYVQMKFMKGFSADAKMMYG
EASQVANDAVGSIRTVASFCAEDNVMELYRKKCEGPMKTGIRQGLISGSGFGVSFFLLFCVYATSFYAGARLV
DAGKTTFSNVFRVFFALTMAAIGISQSSSFAPDSSKARTATASIFGIIDKKSQIDPSDESGTTLDSVKGEIELRHVN
FKYPSRPDVQIFRDLSLTIHSGKTVALVGESGSGKSTVIALLQRFYDPDSGQITLDGIEIRQLQLKWLRQQMGLV
SQEPVLFNETIRANIAYGKGGNATEAEITAAAELANAHKFISGLQQGYDTLVGERGTQLSGGQKQRVAIARAII
KSPKILLLDEATSALDAESEKVVQDALEKVMVNRTTVVVAHRLSTIRNADVIAVVKNGVIVEKGKHEALIKVS
GGFYASLVQLHTSASTV

>XP_023546096.1_ABCB11-l_X1_Cp
MEIENGVDGNSNSIDHQPSSSRTNGIEKSSNKNGNQQDLKNKNGDGKTNSVPFYKLFSFADSTDVLLMIVGSIG
AIGNGLSLPLMTILFGELTDSFGGNQNSSDIVKVVSKVCLKFVYLAIGCGVAAFIQVASWMVTGERQASRIRGL
YLKTILRQDVSFFDMETNTGEVVERMSGDTVLIQDAMGEKVGKCIQLVSTFTGGFVIAFIKGWLLTLVMLSSLP
LLVISGGITSIVITKMTSRGQGAYAKAADVVEQTISSIRTVASFTGEKHAVNSYKKYLVDAYRSGVQEGSAVGI
GFGMIFAVLFFSYSLAIWYGAKLILDKGYSGGAVLNVVVAVLTGSMSLGQASPCLSAFAAGRAAAFKMFETIK
RKPLIDAYDTKGKTLDDISGDIELRDVHFSYPTRPDEHIFNGFSLRIPSGTTAALVGQSGSGKSTVISLIERFYDPS
MGEVLIDGINLKEFQLKWIRSKIGLVSQEPVLFASSIRDNIAYGKDGATIEEIKAAAELANASKFIDKLPQGLDTL
VGAHGTQLSGGQKQRVAIARAILKDPRILLLDEATSALDAESEHVVQEALDRIMVNRTTVIVAHRLSTVRNAD
MIAVIHKGKMVEKGSHTELLKDPEGPYSQLIRLQEVNQESQEAGIDKVKQESTSGSFRRYSKGASIRRSVSRGSS
GVGNSSRHSFSVSFGLPAAVPITDVPMADESAPENTMERSPPVPLRRLAYLNKPEIPILALGSVAAVINGMILPLF
GLLFANAIETFYKPPDKLKKDSRFWALIMMLLGIASLIVAPAKTYLFSVAGCKLIQRIRLLCFEKIVNNEVGWFD
RTENSSGSIGGRLSANAATVRALVGDALSQLVENLASVTAGLVIAFASSWQLALIVLAMFPLLGMNGYVQMK
FMKGFSADAKLMYEQASQVATDAVGSIRTVSSFCAEEKVMQLYKKKCEGPMKSGIRQGLISGTGFGVSFFLLF
SVYAATFYAGAHFVKDGKATFSDVFRVFFALTMAAFAISQSSSLAPDSSKAKEATASIFSMIDRKSEIDPSVETG
ETLENLKGEIEFRHVSFKYPSRPDVQILRDLSLSIRSGKTVALVGESGCGKSTVISLLQRFYDPDSGSITLDGIEIQ
KFQLKWLRQQMGLVSQEPILFNDTIRANIAYGKSGDATEAEIIAASELSNAHKFISGLQQGYDSTVGERGAQLS

GGQKQRVAIARAIIKSPKILLLDEATSALDAESERVVQDALDKVMVNRTTIVVAHRLSTVKNADIIAVVKNGVI
VEKGRHDTLINIKDGFYASLVQLHTKASTSSA

>XP_010091424.1_ABCB11_Mn
MAVENGTRGGGSGGDDINVQEEGTSKVNVEEAQNTPERNGDEQEASESEAEKKKTADQKVPYLKLFSFADRT
DVWLMIFGIIGAVGNGLTMPYMTILLGELINAFGSNQDNNKETVDKVAKVALKFVYLAVAALVVAFLQVAC
WMVTGERQASRLRGLYLKTILRQDVAFFDKETNTGEVVGRMSGDTVLIQDAMGEKVGKFIQLIVTFIGGFSVA
FYKGWLLTLVMMSSIPLMVIAGASMAVFMTKMASRSQNAYAKASTVVEQTIGSIRTVASFTGEKQAISNYTKF
LVSAYKSGVFEGTAAGLGLGMIMLLVFCSYALAIWFGSKMIREKGYTGGDVINVIVAVLTGSMSLGQTSPCLS
AFASGQAAAFKMFETIHRKPEIDAYDDSGKILEDIRGDIELREVNFSYPTRPEEQIFSGFSLSIPSGTTAALVGESG
SGKSTVISLIERFYDPQAGEVLIDGINLKELQLRWIRGKIGLVSQEPVLFASSIKENIAYGKEGATLEEIRAAAELA
NAAKFIDKLPQGLDTMVGEHGTQLSGGQKQRVAIARAILKDPRILLLDEATSALDAESERIVQEALDRIMVNRT
TVIVAHRLSTVRNADMIAVIHRGKMVEKGSHSELVKDPNGAYSQLIRLQEVNKETKQVIGDQNKAEITSTESLR
QSSQRGSFMRSISRGSSVGNSSRHSFSVSFGLPTGIHDTALPDPEATPAKAAEDLPKISLLRLAALNKPEILVLLM
GAVAAIVNGVIMPIFGLLLSTVIKVLYEPADEQEDDANFWSLMFVVLGIVGFLAIPARGYFFAVAGNKLIQRIRV
MCFEKVVNMEVGWFDESDHSSGVIGSRLSADAASVRALVGDALGLLVENSASALAGLIIAFQACWQLAFIVLI
LLPLIGVNGYVQIKFMKGFSADAKAMYEEASQVANDAVGSIRTVSSFCAEEKVMELYKNKCEGPKKTGIRQG
LISGIGFGVSFFLLYTVYATSFYAGSKLMEAGKATFSDVFRVFFALTLAALGVSQSSSFAPDSGKARYAAASIFS
LIDRKSKIDPSDESGETIDDVRGEIQLRHVSFTYPLRPDVQVFKDLSLTIHSGKTVALVGESGSGKSTVVALLQRF
YDPDGGHITLDGVEIQRLKLKWLRQQMGLVSQEPVLFNDAIRANIAYGKEGNATEAEILEASELANAHKFISSL
QQGYDTAVGERGVQLSGGQKQRVAIARAIVKSPKILLLDEATSALDAESERVVQDALDRVMVNRTTVVVAHR
LSTIKNADVIAVVKNGLIVEKGRHETLINIKDGVYASLVQLHTSASTV

>XP_024981326.1_ABCB21-l_Cca
MMNAGSKEKASTSTSGDADADADAVQPQEKQKESTYTVPFYKLFAFADSSDHMLMIAGTLGAIGNGICMPLM
TILFGDLIDAFGQNQNTNDVVHVVSKVSLKFVYLAIGAGVASFLQVAMWMVTGERQAARIRNLYLKTILRQD
VSFFDKETNTGEVVGRMSGDTVLIQDAMGEKVGKFTQLLATFVGGFVIAFIKGWLLTLVMLTSIPPLVISGGV
MSVIISKMASRGQNAYAKAANVVEQTIGSIRTVASFTGEKKAVANYNETLVDAYKSGVHEGLAAGLGLGSM
MLIVFCSYALAVWYGAKMVLERGYTGGTVLTVIFAVLTGSMSLGQASPCLSAFAAGRAAAFKMFETINRKPEI
DAYDTRGKVLSDIRGDVELKDVYFTYPARPDEQIFSGFSLFISSGTTAALVGESGSGKSTVISLIERFYDPQAGEV
LIDNVNLKEFQLKWIREKIGLVSQEPVLFASSIKDNILYGKNGASMDEIRVAVELANAAKFIDKLPQGLDTMVG
EHGTQLSGGQKQRIAIARAILKDPRILLLDEATSALDAESERIVQEALDRIMVNRTTVIVAHRLSTVRNADMIAV
IHRGKMVEKGSHSQLLQDPEGAYSQLIKLQDINNDSRQNGPEDQDKRTSYQRSISRGSSSIGNSSRRSISISFGMP
TQLGVSTAESMEIEASPAKEGSEKPPKVPLRRLAYLNKPEIPVLILGAIAAIINGAVLPVFGILISSMIKTFYEPPDK
MKTDSRFWALMFVVLGVVSFLAFPGRSYFFSVAGSKLIRRIRSLCFEKVINMEVGWFDKPENSSGAIGARLSAD
AASVRGLVGDALAQLVQDSSSAAAGLAIAFAACWQLALIILALVPLIGVNGYVQMKFMKGFSADAKIMYEEA
SQVANDAVGSIRTVASFCAEEKVMELYRNKCEGPKKTGIQQGLISGIGFGVSFFLLFCVYAASFYAGARLVEDG
KTTFSDVFRVFFALTMAAVAVSQSSSFAPDTSKAKSSAVSVFAMLDRKSEIDPSDESGLTLDHVKGEIELRHISF
KYPTRPDVQIFRDLCLTIHSGKTVALVGESGSGKSTVISLLQRFYNPDSGCITLDGTEIQKFQLKWLRLQMGLVS
QEPVLFNDTIRANISYGKDGDATEAEILAASELANAHKFISGLHQGYNTVVGERGVQMSGGQKQRVAIARAIV
KSPKILLLDEATSALDAESERVVQDALDKVMVNRTTVVVAHRLSTIKGADVIAVVKNGVIVEKGKHENLINIK
DGSYASLVALHMTSSK

>XP_020868941.1_ABCB11-l_Al
MNRDGAGEGDSVSHEPSTSKSPKEEEVETKKEEKNDEKAKTVPFYKLFAFADSFDVLLMICGSIGAIGNGVCLP
LMTLLFGDLIDSFGKNQNNKDIVDVVSKVCLKFVYLGLGTLGAAFLQVACWMITGERQAARIRSTYLKTILRQ
DIGFFDLETNTGEVVGRMSGDTVLIQDAMGEKVGKFIQLVSTFVGGFVLAFIKGWLLTLVMLTSIPLLAMAGA
AMAIIVTRASSRGQAAYAKAATVVEQTIGSIRTVASFTGEKQAINSYKKFITSAYKSSIQQGFSTGLGLGVMFFV
FFSSYALAIWFGGKMILEKGYTGGAVINVIIIVVAGSMSLGQTSPCVTAFAAGQAAAYKMFETIKRKPLIDAYD
VNGKVLEDIRGDIELKDVHFSYPARPDEDIFDGFSLFIPSGATAALVGESGSGKSTVISLIERFYDPKAGEILIDGV
NLKEFQLKWIRSKIGLVSQEPVLFSSSIMENIAYGKENATVEEIKAATELANAAKFIDKLPQGLDTMVGEHGTQ
LSGGQKQRIAIARAILKDPRILLLDEATSALDAESERVVQEALDRVMVNRTTVIVAHRLSTVRNADMIAVIHRG
KMVEKGSHSELLKDSEGAYSQLIRLQEINKGNDVKPSDISAGSSFRNPSLKKSIEGSVISGGTSSAGNSSRHHSLN
VLGLSAGLDLSNGSQRVGQEETGTTSQEPLPKVSLTRIAALNKPEIPVLLLGTVAAAINGAIFPLFGILISRVIEAF

FKPADQLKKDSRFWAIIFVALGVTSLIVSPSQMYLFAVAGGKLIRRIRSMCFEKAVXMEVGWFDEPQNSSGTM
GARLSADAALIRALVGDALSLAVQNVASAASGLIIAFTASWELALIILVMLPLIGINGFVQVKFMKGFSADAKS
KYEEASQVANDAVGSIRTVASFCAEEKVMQMYKKQCEGPIKDGIKQGFISGLGFGLSFFILFCVYATSFYAGAR
LVEDGKITFNDVFQVFFALTMAAIGISQSSTFAPDSSKAKVAAASIFAIIDRKSKIDSSDESGTVLENVKGDIELR
HLSFTYPARPDIQIFRDLCLTIRAGKTVALVGESGSGKSTVISLLQRFYDPDSGHITLDGVELKKLQLKWLRQQM
GLVGQEPVLFNDTIRANIAYGKGSEEAATESEIIAAAELANAHKFISSIQQGYDTVVGERGIQLSGGQKQRVAIA
RAIVKEPKILLLDEATSALDAESERVVQDALDRVMVNRTTIVVAHRLSTIKNADVIAVVKNGVIAEKGTHETLI
KIDGGVYASLVQLHMTASN

>XP_011467146.1_P_ABCB11-l_Fv
MADENGVNGDTNSHENHAEEEKSSAPMNKDQPASSDSNGDEKVEKIPFSKLFSFADKTDVILMVVGTIGAVG
NGSCMPLMTVLFGEMIDSFGSNQNKDVVAVVSKVSLKFVYLAVGAGVAAFLQVSCWMVTGERQAARIRGM
YLKTILRQDVAFFDMETNTGEVVGRMSGDTVLIQDAMGEKVGKFLQLMSTFIGGFIIAFIKGWLLTLVMLSSIP
LLVAAGASMSIIITKMASRGQTAYAKAANVVEQTIGSIRTVASFTGEREAITSYSKYLVDAYKSGVHEGSAAGI
GLGLVMCVVFSTYALAVWFGSKMIREKGYTGGEVLNVIVAVLTGSMSLGQASPCMSAFAAGQAAAYKMFLT
ISRKPEIDAYDEKGKILDDISGDIELRDVYFSYPARLDEQIFDGFSLCIPSGTTAALVGQSGSGKSTVISLIERFYDP
RAGEVLIDGINLKEFQLKWIRSKIGLVSQEPVLFASSIKENIAYGKDGATTEEIQAAAELANAAKFVDKLPQGLD
TMVGEHGTQLSGGQKQRIAIARAILKDPRILLLDEATSALDAESERVVQEALDRIMVNRTTVVVAHRLSTVRN
ADMIAVIHKGKMVEKGSHSNLLRDPEGAYSQLIRLQEVNKDSEQTPEDQSKPEITLASLRQSSQKASSQRLSFA
RSLSRNSSAGNSSRHSFSVAFGLPTGLGGIGVQDAAYEETELAPEEPPTVSLRRLAALNKPEIPVLIIGTIAAIING
VILPIFGVLISRVIKTFYEPPNQQKKDAAFWAIIFMILGLISFVVIPARGYFFSVAGSKLIQRIRLLCFERVVHMEV
GWFDEPENSSGSIGARLSADAATVRALVGDALAQMVQNLAAAVSGLVIAYIACWQLAFIILALLPLIAVNGYV
QIKFMKGFSADAKMMYEEASQVANDAVGSIRTVASFCAEEKVMELYRRKCEGPMKTGIRQGLISGIGFGVSFF
FLFCVYATSFYAGAQLVKAGKTTFSDVFQVFFALTMAATGISQSSSFGPDSSKAKSAAASIFAIIDRPSKIDPSNE
SGTKIDGGVKGEIELRHVSFRYPSRPDTPIFRDLNLTIRSGKTVALVGESGSGKSTVVALLQRFYDPDSGRITLDG
IELGDYNLKWLRQQMGLVSQEPVLFNDTIRANIAYGKEETATEAEIIAASELANAHKFISSLHQGYDTIVGERGI
QLSGGQKQRVAIARAIIKSPKILLLDEATSALDAESERVVQDALDRVMVNRTTVVVAHRLSTIKNADVIAVVK
NGVIVEKGKHDNLINITDGFYASLVALHMSSSTA

>XP_006355823.1_P_ABCB11-l_X2_St
MAEGNGLNGNSGINEASSSGGQNNTSQQDSDKTKQAEKANTVPFYKLFSFADSTDMVLMITGTIAAIGNGMSL
PIMTILFGELTDSFGQNQNNKDVLRVVSRVSLKFVYLALGCGVASFLQVACWMISGERQASRIRSLYLKTILQQ
DIAFYDKETNTGEVVGRMSGDTVLIQDAMGEKVGKFVQLISTFIGGFVIAFTKGWLLTLVMLSVIPLLAISGGA
MSHVLSKMASSGQDAYAKAATVVEQTIGSIRTVASFTGEKQAVADYNESLIKAYHSGAKEGLATGLGLGSVF
AIIYCSYALAIWYGARLILEKGYTGGNVINIIIAVLTSSMSLGQAAPCMSAFAAGQAAAFKMFETIKRKPEIDAY
DTNGKILDDIRGDIELNDVCFSYPARPDEQIFSGFSLFVSSGTTAALVGQSGSGKSTVISLIERFYDPQSGQVLIDG
INLKDFQLKWIRGKIGLVSQEPVLFTASIKENILYGKHDATAEEIKAATELANAAKFIDKLPQGLDTMVGEHGT
QLSGGQKQRIAIARAILKDPRILLLDEATSALDAESERVVQEALDRIMINRTTVIVAHRLTTVRNADMIAVIHRG
KVVEKGTHGELLKDPEGAYSQLIRLQEVNNETKKSGLDERDSIDKSMGSGRQSSQRISLMRSISRSSSGVGNSS
RRSLSISLGLATGLSVPETANTDTEMGIPEVAGKRLEVPIRRLAYLNKPEIPVMIIGTVAAIINGAILPIFGILLSSVI
KTFYEPPHELRKDSRFWALMFVLLGAVTLIAFPARTYFFSIAGCKLIRRIRSMCFEKVVHMEVGWFDESEHSTGI
IGARLSADAAAVRGLVGDALAQMVQDTATSIVGLAIAFEASWQLALIVLVMIPLIGLNGYIQIKFMKGFSADAK
MMYEEASQVANDAVGGIRTVASFCAEEKVMEIYRKKCEGPLKAGIKQGLISGIGFGVSFALLFCVYATSFYAG
ARLVQDGKITFSDVFRVFFALTMAAIGISQSSSLAPDSSKAKSAAASVFAILDRKSKIDPSDDSGMTLDTVKGDI
ELKHVSFKYPTRPDVQILRDLCLTIRSGKTVALVGESGCGKSTVISLLQRFYDPDSGQISLDGIEIQKFQVKWLR
QQMGLVSQEPVLFNDTIRANIAYGKEGNATEAEVLAAAELANAHKFISGLQQSYDTTVGERGTQLSGGQKQR
VAIARAILKNPKILLLDEATSALDAESERIVQDALDRVMVNRTTVVVAHRLSTIKGADIIAVVKNGVIVEKGKH
DTLINIKDGFYSSLVALHTSAS

>XP_017629349.1_P_ABCB4-l_Ga
MASENGFNGEKVTDLHEAGTSKSQEESQKVPGDNGENQDPESSKGDEKTNTVPFYKLFAFADSTDILLMIVGT
TAATGNGLSMPLMTILFGNLIDSFGTNQSNGNVVEAVSKVSVRFVYLAMGAAAAAFLQVSCWMITGERQAAR
IRGLYLKTILRQDVAFFDVETNTGEVIGRMSGDTVLIQDAMGEKVGKFSQLISTFIGGFVIAFVKGWLLTLVML
SAIPLLVISGGTIAVIVSKMASRGQNAYAKAATVVEQTIGSIRTVASFTGEKQAINNYNKSLVTAYRSGVHEGT

AAGVGIGLVMLVIFCSYALAVWFGGKMILEKGYTGGEVLNVIMAVLTGSMSLGQASPCMTAFAAGQAAAFK
MFETIKRKPEIDSYDTSGKVLEDIRGDVELRDVYFSYPARPEEQVFSGFSLSIPSGTTVALVGQSGSGKSTAISLIE
RFYDPQAGEVLIDGINLKEFQLRWIRGKIGLVSQEPVLFTSSIRDNIAYGKEDATTEEIRAAAELANAANFIDKLP
QGLDTMVGEHGTQLSGGQKQRVAIARAILKDPRILLLDEATSALDAESERVVQEALDRIMGNRTTVIVAHRLS
TVRNADSIAVIHKGKMVEKGSHSELLKDPEGAYSQLIRLQEVNKGLEQLADVSDVNQELFRQSSLRRSFKRSIS
RGSSRTSSRNSSFSQPFGLPTEMNVTDPAMLDTEDPAEPPLKQAKEVSILRLAYLNKPEIPVILIGTLFAAANGVI
LPLFGILISNMIKTFYQPPDELKKDSRFWALIFLSLGLASFLINPARTYFFSIAGCKLIQRIRSTCFEKVVRMEVAW
FDEPDNSSGSIGARLSADAASIRALVGDALAQLVSSFASALAGLVIAFVASWQMAFIILVLIPLIGVNGYIQAKF
MKGFSADAKMMYEEASQVASDAVGSIRTVASFCAEEKVMQLYKKKCEGPMKTGIRQGLISGSGFGLAFFFLFS
VYATSFYAGAQLVEHGQATFTDVFQVFLALTMAAVGISQSSSFAPDSNKAKIAAASIFAIIDRRSKIDPSDESGM
TLENVKGNMELRDVSFKYPSRPDIQIFQALSLSIHAGKTVALVGESGSGKSTVISLLQRFYDPDSGTITLDGVKIQ
TLQLKWLRQQMGLVSQEPVLFNDTIRANIAYGKGGNATEAEIITTSELANAHTFISALQQGYDTVVGERGLQLS
GGQKQRVAIARAIVKSPKILLLDEATSALDAESEKVVQDALDKVMVNRTTVVVAHRLSTIKNADVIAVVKNG
VIVEKGKHDTLINIKDGFYASLVALHMTASTSQ

>XP_013614658.1_P_ABCB11-l_Bo
MNRDGAGAGNSVSHEPSTSKTPREGGEEETKKDEKAKTVPFYKLFAFADSYDVLLMICGSVGAMGNGVGLPL
MTLLFGDLIDSFGQNQNNKDIVDVISKVCVKFVYLGLGTLGAAFLQVACWMITGERQAARIRNMYLKTILRQD
IGFFDVETNTGEVVGRMSGDTVLIQDAMGEKVGKFIQLIATFIGGFALAFAKGWLLTLVMLTSIPLLAMAGAA
MAIIVTKASSQGQAAYAKAATVVEQTIGSIRTVASFTGEKEAINKYKKFITSAYKSSIQQGFSTGLGLGIMLFVL
FSSYALAIWFGGKMILEKGYTGGAVINVLIIVVAGAMSLGQTSPCVTAFSAGQSAAYKMFETIERKPLIDAYDL
KGKILKDIRGDIELKDVHFSYPARPDEDIFDGFSLFIPSGATAALVGESGSGKSTVISLIERFYDPKAGQVLIDGV
NLKEFQLKWIRSKIGLVSQEPVLFSSSIMGNIAYGKDNATIQEIKAATELANAAKFIDKLPQGLDTMVGEHGTQ
LSGGQKQRIAIARAILKDPRILLLDEATSALDAESERVVQEALDRVMVNRTTVIVAHRLSTVRNADMIAVIHRG
KMVEKGSHSELLRDPEGAYSQLIRLQEINKDAKAASGSSFRNSSLKKSIEGSSSSVGNSSRHHSVNVVTSELEHG
GGGGSRAGQEDKPGTEAQEPVPKVSLTRIAALNKPEIPVLLLGTVAAAINGAIFPLFGILISRVIEAFFKPAHELR
RDSKFWALIFVALGVVSFIVSPTQMYLFAVAGGKLIRRIRSMCFEKAVHMEVGWFDEPQNSSGTLGARLSADA
ALIRALVGDALSLAVQNAASAASGLIIAFTACWELALIILVMLPLIGINGYIQVKFMKGFTADAKSKYEDASQV
ANDAVGSIRTVASFCAEEKVMQMYKKQCEGPIKDGIKQGFISGLGFGFSFFILFCVYAASFYAGARLVEAGRTT
FNDVFQVFFALTMAAIGISQSSSFAPDSSKAKVAAASIFGIIDRKSKIDSSDESGTVLENVKGDIELRHISFTYPAR
PDIQIFRDLCLTIRAGKTVALVGESGSGKSTVISLLQRFYDPDSGHITLDGVELKKMQLKWLRQQMGLVGQEPV
LFNDTIRANIAYGKGSEEAATESEIIAAAELANVHKFISSIQQGYDTVVGERGIQLSGGQKQRVAIARAIVKEPKI
LLLDEATSALDAESERVVQDALDRVMVNRTTVVVAHRLSTIKNADVIAVVKNGVIAEKGTHETLIKMEGGVY
ASLVQLHMTASN

>XP_015168025.1_P_ABCB11-l_X1_St
MPRLGGVRAMAEGNGLNGNSGINEASSSGGQNNTSQQDSDKTKQAEKANTVPFYKLFSFADSTDMVLMITGT
IAAIGNGMSLPIMTILFGELTDSFGQNQNNKDVLRVVSRVSLKFVYLALGCGVASFLQVACWMISGERQASRIR
SLYLKTILQQDIAFYDKETNTGEVVGRMSGDTVLIQDAMGEKVGKFVQLISTFIGGFVIAFTKGWLLTLVMLSV
IPLLAISGGAMSHVLSKMASSGQDAYAKAATVVEQTIGSIRTVASFTGEKQAVADYNESLIKAYHSGAKEGLA
TGLGLGSVFAIIYCSYALAIWYGARLILEKGYTGGNVINIIIAVLTSSMSLGQAAPCMSAFAAGQAAAFKMFETI
KRKPEIDAYDTNGKILDDIRGDIELNDVCFSYPARPDEQIFSGFSLFVSSGTTAALVGQSGSGKSTVISLIERFYDP
QSGQVLIDGINLKDFQLKWIRGKIGLVSQEPVLFTASIKENILYGKHDATAEEIKAATELANAAKFIDKLPQGLD
TMVGEHGTQLSGGQKQRIAIARAILKDPRILLLDEATSALDAESERVVQEALDRIMINRTTVIVAHRLTTVRNA
DMIAVIHRGKVVEKGTHGELLKDPEGAYSQLIRLQEVNNETKKSGLDERDSIDKSMGSGRQSSQRISLMRSISR
SSSGVGNSSRRSLSISLGLATGLSVPETANTDTEMGIPEVAGKRLEVPIRRLAYLNKPEIPVMIIGTVAAIINGAIL
PIFGILLSSVIKTFYEPPHELRKDSRFWALMFVLLGAVTLIAFPARTYFFSIAGCKLIRRIRSMCFEKVVHMEVGW
FDESEHSTGIIGARLSADAAAVRGLVGDALAQMVQDTATSIVGLAIAFEASWQLALIVLVMIPLIGLNGYIQIKF
MKGFSADAKMMYEEASQVANDAVGGIRTVASFCAEEKVMEIYRKKCEGPLKAGIKQGLISGIGFGVSFALLFC
VYATSFYAGARLVQDGKITFSDVFRVFFALTMAAIGISQSSSLAPDSSKAKSAAASVFAILDRKSKIDPSDDSGM
TLDTVKGDIELKHVSFKYPTRPDVQILRDLCLTIRSGKTVALVGESGCGKSTVISLLQRFYDPDSGQISLDGIEIQ
KFQVKWLRQQMGLVSQEPVLFNDTIRANIAYGKEGNATEAEVLAAAELANAHKFISGLQQSYDTTVGERGTQ
LSGGQKQRVAIARAILKNPKILLLDEATSALDAESERIVQDALDRVMVNRTTVVVAHRLSTIKGADIIAVVKNG
VIVEKGKHDTLINIKDGFYSSLVALHTSAS

>MtPDR23
MEGGELRVASGRVGSSSIWRSGAVDVFSGSSRRDDDEQELQWAAIEKLPTYLRMTRGILN
ESQSEQPIEIDINKLGPLQRKNLVERLVKIAEEDNEKFLLKLRQRIDRVGLDFPTIEVRF
EHLNVEAEAHVGSRALPTILNFSINLLEGFLNNLHLIPSRKKPLTVLHDVSGIIKPKRMT
LLLGPPSSGKTTLLLALAGRLSRDLKFSGRVAYNDHGMEEFVPQRTSAYISQTDLHIGEL
TVRETLAFSARCQGIGTRYDMLAELSRREKAENIKPDPDLDIYMKAEALEGQETNIVTDY
IIKILGLDVCADTMVGDDMIRGISGGQKKRVTTGEMLVGPARALFMDEISTGLDSSTTFQ
MINSLRQSIHILNGTALISLLQPTPETYDLFDDIILLSDGQIVYQGPRENVLEFFEHVGF
KCPERKGVADFLQEVTSRKDQEQYWSNKDKPYTFITVREFAEEFQLFHVGQKLGDELGTP
FDASKGHPAVLTKNKYGVSRKELLKACVSRELLLMKRNSFVYIFKMWQLIFTGIVTMTMF
LRTEMHRNTETDGGIYMGALFFILIVIMFNGYSELSMFIMKLPVFYKQRDLLLFPAWAYS
LPTWILKIPITFVEVGIWVVLTYYVIGFDPCFERFIKQYFLLVCINQMASALFRFIGAVG
RNVIVANTVGSFALLAVLVMGGFILSRVDVKKWWLWGYWVSPMMYGQNAIAVNEFLGKSW
SHIPPDSTEPLGVQILKSRGIFPEAYWYWIGVGASIGYMLLFNFLFPLALHYLDSKYPIY
YMWLSAFGKPQALISEEALAERNAATAGSKQIIELSPKLECSSGNASRRSFSSTTLSTKV
GSINAADHTRKRGMVLPFTPLSITFDEIGYAVDMPQEMKAKGIPEDRLELLTGVNGAFRP
GVLTALMGISGAGKTTLMDVLSGRKTTGYVQGQITISGYPKKQETFSRISGYCEQTDIHS
PHVTVYESLVYSAWLRLPPEVDTSTRKMFIEEVMELIELTSIREALVGLPGVNGLSTEQR
KRLTIAVELVANPSIIFMDEPTSGLDARAAAIVMRTVRNTVDTGRTVVCTIHQPSIDIFD
AFDELLLLKRGGEEIYVGPLGRHCSHLINYFEGINGVPKIKNGYNPATWMLEVTSEAQEE
ALGINFAELYKNSDLYRTNKALIRELSTPPEGSKDLYFTTQHSQSFLTQCMACLWKQNLS
YWRNPPYSAVRLLFTTVIAFLFGTIFWNIGSKRERRQDLFNAMGSMYAAVLFIGVQNATS
VQPVVAIERTVFYREKAAGMYSALPYAFGQVAVEIPYILIQSLVYGVIVYTMVGFERTPT
KFFWYLFFMFFTFLYFTFFGMMLVGATPDHNVAAIVSFGFYLLWNLFSGFVIPRTRMPVW
WRWFFWICPISWTLYGLITTQFGDVNERMDTGETVEEFVRSYFGYRDDFKDVAAAVVVSF
SLIFGSAFAFSIKAFNFQKR

>PhPDR1
meggeelfrvssarlsssnvwrnsamdvfsrssreaddeealkwaaleklptylrirrgi
lteeegqsrevditkldlverrnllerlikitdednekfllklkeridrvgldlptievr
fehlsvdaearvgsralptvfnftvniledflnylhilpnrkqplpilhdvsgiikpgrm
tlllgppssgkttlllalagkldkdlkvsgrvtynghdmnefvaqrssayisqydlhige
mtvretlafsarcqgvgakyeilaelsrrekeanikpdpdvdifmkaawnegqeanvvtd
ytlkilgleicadtivgdemvrgisggqrkrlttgemmvgparalfmdeistgldsstty
qivnsirqsihilqgtavisllqpapetydlfddiillsdgqivyqgprenvleffeymg
ficperkgvadflqevtsrkdqeqywarreesykfitvrefseafqafhigrklgdelav
pfdkskshpaalttkrygvskkellkactareyllmkrnsfvyifkmiqltlmasitmtl
flptemhrnttidgavflgalfyalimimfngfselalsimklpsfykhrdllffppway
alptwilkipitlvevaiwvcmtyyvigfeadvgrffkqllllicvnqmasglfrlmgal
grniivantfgsfvlltvlvmggfvlsrddvkkwwiwgywispmmyaqnaiavneflgks
wahvppnststetlgvsflksrgifpdarwywigagaligyvflfnflfavalaylnpfg
kpqavlseetvaernaskrgevielsslgksssekgndvrrsassrsmssrvgsitaadl
skrrgmilpfeplsitfddiryavdmpqemkaqgftedrlellrgvsgafrpgvltalmg
vsgagkttlmdvlagrktggyidgtisisgypkqqetfariagyceqtdihsphvtvyes
lqfsawlrlprevdtatrkmfieevmelieliplrdalvglpgvnglsteqrkrltvave
lvanpsiifmdeptsgldaraaaivmrtvrntvdtgrtvvctihqpsidifdafdellll
krggeeiyvgplgrqsshlikyfegidgvpkikdgynpatwmleitsvaqegalgndfte
lyknselyrrnkalikelsvpascskdlyfptkysqsfftqcmacfwkqhwsywrnppyt
avrimftffialmfgtifwdlgsrrerqqdllnaigsmyiavlflgvqnattvqpviaie
rtvfyreraagmysampyafgqvmielpylflqtiiygvivyamigfewtvakffwylff
myftllyftlygmmtvavtpnqsiaaiissafyavwnlfcgfivpktrmpvwwrwyyyic
piswtlygliasqfgdiqdrldtnetveqfienffdfkhdfvgyvalilvgisvlflfif

afsiktfnfqkr

>NtPDR6
MEGGEDSFRVSSARLSSNVWRNSAMDVFSRREADDEEALKWAALEKLPTYLRRRGLTEEE
GSRVDTKLDLVERRNLLERLVKADEDNEKFLLKLKKRARVGLDLPTEVRFEHVSVDAEAR
GSRALPTFNFTVNLEDFLNYLHLPSRKKPLPLHEVSGKPGRMTLLLGPPSSGKTTLLLAL
AGKLDKDLKVSGRVTYNGHGMDEFVPRSSAYSNDLHGEMTVRETLAFSARCGVGAKYELA
ELSRREKGANKPDPDVDFMKAWNEGEANVVTDYTLKLGLECADTLVGDMRGSGRKRLTTG
EMMVGPARALFMDESTGLDSSTTYVNSRSHLGTAVSLLPAPETYDLFDDLLSDGVYGPRE
NVLEFFEYMGFMCPERKGVADFLEVTSRKDEYWARRDEPYKYTVREFSESFSFHGRKLGD
ELAVPFDKSKSHPAALTTKKYGSKKELLKACTAREYLLMKRNSFVYFKMLTLMASTMTLP
hcLRTEMHRNTTTDGAVFLGALFYAVMMFNGFSLALSMKLPSFYKRDLLFFPAWAYALPT
WLKPVTLVEVAWVCMTYYVGFEADVGRFFKLFLLCVNMASGLFRFGALGRNVVANTFGSC
ALLTVLVMGGFLSRDDVKKWWWGYWSPMMYANAAVNEFLGKSWAHVPPNSTGTETLGVSF
LKSRGFPEARWYWGAGALGYVLLFNFMFTVALAYLNPFGKSAVLSEETVAERNAKRGEVE
LSPEKRSSERGNDVRRSASSRSMSSRGSTEADLNKRRGMLPFEPLSTFDDRYAVDMPEMK
AGVAEDRLELLKGVSGAFRPGVLTALMGVSGAGKTTLMDVLAGRKTGGYDGTSSGYPKET
FARAGYCETDHSPHVTVYESLFSALLRLPREVDTETRKMFVEEVMELVELTPLREALVGL
PGVNGLSTERKRLTVAVELVANPSFMDEPTSGLDARAAAVMRTVRNTVDTGRTVVCTHPS
DFDAFDELLLLKRGGEEYGPLRHSSHLKYFEGDGVPKKDGYNPATWMLETSVAEAARVDF
TELYKNSELYRRNKALKELSVPPPCSKDLYFPTKYSSFFTCKACFWKHWSYWRNPPYTAV
RLMFTFFALMFGTFWDLGSRRKRDLLNAGSMYAAVLFLGVNATSVPVAERTERAAGMYSA
LPYAFGVMELPYLFTYGVVYVMGFEWTVAKFFWYLFFMYFTLLYFTLYGMMTVAVTPNHS
ASSFYAWNLFCGFVVPKTDVFYGSKSDGSNFVLSTVCARAVGVRDTWCWLGAGAWHWVCA
RGTVCAGSAVGNMMVCARSVGTRGTDGRPLGAETDYRWDDRGVCVLVTWRCDHGADRGSR
RWEACRDATGRVGVMGR

>XP_006366078.1_P_PDR1-l_St
MEGGGDILKVSSARLSSSNVWRNSAMDVFSRSSREDYDDEEALKWAALEKLPTYLRIRRGILSEEEGQYREVD
ITKLDLVERRNLLERLVKIADEDNEKFLLKLKKRIDRVGLDLPTIEVRFEHLNVDAEARVGSRALPTIFNFTVNIL
EDFLNYLHILPSRKKPLPILHDVSGIIKPGRMTLLLGPPSSGKTTLLLGLAGKLDKDLKVSGRVTYNGHGMDEF
VPQRTSAYISQNDLHIGEMTVRETLAFSARCQGVGAKYEILAELSRREKEANIKPDPDVDIFMKSAWNDGQEA
NVVTDYTLKILGLEICADTIVGDEMIRGISGGQRKRLTTGEMMVGPARALFMDEISTGLDSSTTYQIVNSIRQSI
HILQGTAVISLLQPAPETYDLFDDIILLSDGQIVYQGPRENVLEFFEYLGFKCPQRKGVADFLQEVTSRKDQEQY
WARRDEPYKFITVREFSEAFQSFHVGRKLGDELAVPFDKSKSHPAALTTKRYGVSKKELLKACTAREYLLMKR
NSFVYIFKMIQLTLMATITMTLFLRTEMHRNTMIDGAVFLGALYYAVIMIMFNGFSELALSIMKLPSFYKQRDL
LFFPAWTYALPTWILKIPITLVEVAIWVCMTYYVIGFEADVGRFFKQLFLLICLNQMASGLFRFLAALGRNIIVA
NTFGSCALLIVLVMGGFILSRDNVKQWLIWGYWISPMMYAQNAIAVNEFLGKSWAHVPPNSTGTDTLGVSFL
KSRGIFPEARWYWIGVGALLGYVLLFNFLFTVALAYLNPFGKPQAVLSEETVAERNASKKGEVIELSPIGKSSSE
RGNDVRRSASSRSMSSRVGNITEGDINKRKGMILPFEPLSITFDDIRYAVDMPQEMKSQGFIEDRLELLKGVSGA
FRPGVLTALMGVSGAGKTTLMDVLAGRKTGGYIEGTISISGYPKQQETFARIAGYCEQTDIHSPHVTVYESLQY
SAWLRLPREVDTETRKRFIEEVMELVELKPLREALVGLPGVNGLSTEQRKRLTVAVELVANPSIIFMDEPTSGL
DARAAAIVMRTVRNTVDTGRTVVCTIHQPSIDIFDAFDELLLLKRGGEEIFVGPLGRHSSHLIKYFEGIDGVPKIR
DGYNPATWMLDITSVAQEAALGVDFTELYRNSELYRRNKALIKELSVPAPGSKDLYFQTKYSQSFFTQSMACF
WKQHWSYWRNPPYTAVRLLFTFFIALMFGTIFWDLGSKRRRQQDILNAIGSMYASVLFLGVQNATSVQPVVAI
ERTVFYRERAAGMYSALPYAFGQIMIELPYIFIQTIIYGVIVYAMIGFEWTVAKFFWYLFFMYFTLLYFTLYGM
MTVAVTPNHSIAAIVSSAFYAVWNLFSGFIVPKTRMPVWWRWYYYICPISWTLYGLIASQFGDLQDKLETKET
VEEFIESFFDFKYDFVGYVAVILVGISVLFLFIFAYSIKAFNFQKR

>XP_004245225.1_PDR1-l_Sl
MEGGGDILKVSSARLGSSTVWRNSGVDVFSRSSREDYDDEEALKWAALEKLPTYLRIRRGILSEEEGQYREVDI
TKLDLVERRNLLERLVKIADEDNEKFLLKLKKRIDRVGLDLPTIEVRFEHLNVDAEARVGSRALPTIFNFTVNIIE
DFLNYLHILPSRKKPLPILHEISGIIKPGRMTLLLGPPSSGKTTLLLGLAGKLDKDLKVSGRVTYNGHGMDEFVP
QRTSAYISQNDLHIGEMTVRETLAFSARCQGVGAKYEILAELSRREKEANIKPDPDVDIFMKSAWNDGQEANV

VTDYTLKILGLEICADTIVGDEMIRGISGGQRKRLTTGEMMVGPARALFMDEISTGLDSSTTYQIVNSIRQSIHIL
QGTAVISLLQPAPETYDLFDDIILLSDGQIVYQGPRENVLEFFEYIGFKCPQRKGVADFLQEVTSRKDQEQYWA
RRDEPYKFITVREFSEAFQSFHVGRKLGDELAVPFDKSKSHPAALTTERYGVSKKELLKACTAREYLLMKRNSF
VYIFKMIQLTLMATITMTLFLRTEMHRDTMIDGAVFLGALYYAVIMIMFNGFSELALSIMKLPSFYKHRDLLFF
PAWTYALPTWILKIPITLVEVAIWVCMTYYVIGFEADVGRFFKQLFLLICLNQMASGLFRFLAALGRNVIVANT
FGSCALLIVLVMGGFILSRDNVKQWLIWGYWISPMMYAQNAIAVNEFLGKSWAHVPPNSTGTDTLGVSFLKS
RGIFPEARWYWIGVGALLGYVLLFNFLFTVALAYLNPFGKPQAVLSEETVAERNASKRGEVIELSPIGKSSSERG
NDVRRSASSRSMSSRVGNIAEGDLNKRKGMILPFEPLSITFDDIRYAVDMPQEMKAQGFTEDRLELLKGVSGAF
RPGVLTALMGVSGAGKTTLMDVLAGRKTGGYIEGTISISGYPKQQATFARIAGYCEQTDIHSPHVTVYESLQYS
AWLRLPREVDTETRKRFIEEVMELVELKPLREALVGLPGVNGLSTEQRKRLTVAVELVANPSIIFMDEPTSGLD
ARAAAIVMRTVRNTVDTGRTVVCTIHQPSIDIFDAFDELLLLKRGGEEIFVGPLGRHSSHLIKYFEGIDGVLKIKD
GYNPATWMLDITSVAQEAALGIDFTELYRNSELYRRNKALIQELSVPAPGSKDLYFETKYSQSFFTQSMACFW
KQHWSYWRNPPYTAVRLMFTFFIALMFGTIFWDLGSKRRRQQDILNAIGSMYAAVLFLGVQNATSVQPVVAIE
RTVFYRERAAGMYSALPYAFGQIMIELPYIFIQTIIYGVIVYAMIGFEWTVAKFIWYLFFMYFTLLYFTLYGMMT
VAVTPNHSIAAIISSAFYAVWNLFSGFIVPKTRMPVWWRWYFYICPISWTLYGLVASQFGDLQDKLETKETVEE
FIESFFDFKYDFVGYVALILVGISVGFLFIFAYSIKAFNFQKR

>XP_006366077.1_P_PDR1-l_X1_St
MEGGENILRVSSARLSGSNVWRNSAMDVFSRSSSREDYDDEEALRWAALEKLPTYRRIRRGLLLEEEEGQSRE
VDITKLDLIERRNLLDRLVKIADEDNEKLLMKLKQRIDRVGLDLPTIEVRFEHLNIDAEARVGSRALPTIFNFTV
NILEDFLNYLHILPSRKKPLPILHGVGGIIKPGRMTLLLGPPSSGKTTLLLALAGKLDNDLKVSGRVTYNGHGM
DEFVPQRTSAYISQNDLHIGEMTVRETLAFSARCQGVGTKYEILAELSRREKEANIKPDPDIDIFMKSAWNEGQ
EANVITDYTLKILGLEICADTLVGDEMIRGISGGQRKRLTTGEMMVGPARALFMDEISTGLDSSTTYQIVNSIRQ
SIHILQGTAVISLLQPAPETYDLFDDIILLSDGQIVYQGPRENVLEFFEYLGFKCPQRKGVADFLQEVTSRKDQEQ
YWSRRDEPYRFITACEFSDVFQSFDVGRKLGDELAVPFDKSKSHPAALTTKRYGISKKELLKACTAREYLLMK
RNSFVYIFKMVQLTLMASIAMTLFLRTEMHRDTTIDGAIYLGALFYAVITIMFNGFSELALSIMKLPSFYKQRDF
LFFPAWAYALPTWILKIPITLVEIAIWVCMTYYVIGFEADVGRFFKQIFLLICLSQMASGLFRFLAALGRNIIVAN
TFGSCALLIVLVMGGFILSRDDVKQWLIWGYWISPMMYAQNAIAVNEFLGKSWAHVPPNSTGTDTLGVSFLKS
RGIFPEARWYWIGAGALFGYVLLFNFLFTVALAYLNPFSKPQAILSEEIVAERNASKRGEVIELSPIGKSSSERGN
DVPVSTSSRSLSTRVGNITEGDLNKRKGMILPFEPLSITFDDIRYAVDMPQEMKTQGFIEDRLELLKGVSGAFRP
GVLTALMGVSGAGKTTLMDVLAGRKTGGYVEGTISISGYPKQQETFARISGYCEQTDIHSPHVTVYESLLYSA
WLRLPREVDTETRKSFIEEVMELVELTPLREALVGLPGVNGLSTEQRKRLTVAVELVANPSIIFMDEPTSGLDA
RAAAIVMRTVRNTVDTGRTVVCTIHQPSIDIFDAFDELLLLKRGGEEIFVGPLGRHSSHLIKYFEGIDGVLKIRDG
YNPATWMLEVTSLAQEAVLGIDFTELYKNSELYRRNKALIQELSVPASGSKDLYFETKYSQSFFTQCMACLWK
QHWSYWRNPPYTAVRLMFTFFVSLMLGTIFWGLGSKRGKQQDILNAIGSMYAAILFLGIINASSVQPVVAIERT
VFYRERAAGMYSALPYAFGQVMIELPHLFLQTIIYGVIVYAMIGFEWTVTKFFWYLFFMYFTLLYFTLYGMMT
VAVTPNHTIASIVSSAFYTIWNLFCGFVVPKTRMPVWWRWYYYICPLSWTLYGLIASQFGDLQDRLDTKETVE
EFLENFFDYKHDFVGYVAVILVGISVVFLFIFAYSIKSFNFQKR

>XP_004245224.1_PDR1-l_Sl
MEGGENLVRVSSARLSGSNVWRNSAMDVFSRSSSREDYDDEEALRWAALEKLPTYSRIRRGLLLEEEGQSREV
DITKLDLIERRNLLDRLVKIADEDNEKLLMKLKQRIDRVGLDLPTIEVRFEHLNVDAEARVGSRALPTIFNFTVN
ILEDFLNYIHILPSRKKPLPILHGVSGIIKPGRMTLLLGPPSSGKTTLLLGLAGKLDKDLKVSGRVTYNGHGMDE
FVPQRTSAYISQNDLHIGEMTVRETLAFSARCQGVGDKYEILAELSRREKEANIKPDPDVDIFMKSAWNEGQEA
NVITDYTLKILGLEICADTLVGDEMIRGISGGQRKRLTTGEMMVGPARALFMDEISTGLDSSTTYQIVNSIRQSIH
ILQGTAVISLLQPAPETYDLFDDIILLSDGKIVYQGPRENVLEFFEYMGFKCPERKGVADFLQEVTSRKDQEQY
WSRRDEPYRFITSCEFSDVFQSFHVGRKLGEELAVPFDKSKSHPAALTTKRYGISKKELLKACAAREYLLMKRN
SFVYIFKMVQLTMMASIAMTLFLRTEMHRDTTIDGAVYLGALFYAVITVMFNGFSELALSIMKLPSFYKQRDL
LFFPAWAYALPTWILKIPITLVEIAIWVCMTYYVIGFEADVGRFFKQLFLLICLNQMASGLFRFLAALGRNIIVA
NTFGSCALLVVLVMGGFILSRDDVKQWLIWGYWTSPMMYAQNAIAVNEFLGKSWSHVPPNSTGTDTLGVSFL
KSRGIFPEARWYWIGAGALIGYVLLFNFLFTVALAYLNPFGKPQAIISEEIVVERIASKRGEVIELSPIGKSSSERG
NDVAISASSRSLSSRVGNITEGDLSKRRGMILPFEPLSITFDDIRYAVDMPQEMKAQGFIEDRLELLKGVSGAFRP
GVLTALMGVSGAGKTTLMDVLAGRKTGGYINGTISISGYPKQQETFARISGYCEQTDIHSPHVTVYESLQYSA
WLRLPREVDTETRKNFIEEVMELVELIPLREALVGLPGVNGLSTEQRKRLTVAVELVANPSIIFMDEPTSGLDAR

AAAIVMRTVRNTVDTGRTVVCTIHQPSIDIFDAFDELLLLKRGGEEIFVGPLGRHSSHLIKYFEGIDGVLKIRDGY
NPATWMLEVTSLAQEAVLGIDFTELYKNSELYRRNKALIQELSVAAPGSKDLYFETEYSQSFFTQCMACLWKQ
HLSYWRNPPYTAVRLMFTFFVSLMLGTIFWGLGSKRGRQQDILNAIGSMYSAILFLGIINATSVQPVVAIERTVF
YRERAAGMYSALPYAFGQVMIELPHLFLQTIIYGVIVYAMIGFEWTVAKFFWYLFFMYFTLLYFTLYGMMTV
AVTPNHTIASIVSSAFYTIWNLFCGFVVPKTRMPVWWRWYYYVCPLSWTLYGLIASQFGDVQDKLDTKETVE
QFLENFFDYKHDFVGYVAVILVGISVAFLFIFAYSIKAFNFQKR

>XP_010648604.1_P_PDR1_X2_Vv
MESSDVYRVNSARLSSSNIWRNSGMEVFSRSSRDEDDEEALKWAAIEKLPTYLRIRRGILAEEEGKAREIDITSL
GLIEKKNLLERLVKIAEEDNEKFLLKLKERIDRVGLDIPTIEVRFEHITVDAEAYIGGRALPTIINFSANMLEGFLN
YLHILPSRKKPLPILHDVSGIIKPGRMTLLLGPPSSGKTTLLLTLAGKLGSDLKLSGRVSYNGHGMDEFVPQRSS
AYISQYDLHIGEMTVRETLAFSARCQGVGTGYDMLAELSRREKVANIKPDPDIDIYMKAAALKGQGGSLITDYI
LKILGLEVCADTIVGDEMVRGISGGQKRRLTTGEMLVGPAKALFMDEISTGLDSSTTFQIVNSIRQSIHILKGTAI
ISLLQPAPETYDLFDDIILLSDGQIVYQGPRENVLEFFEHMGFKCPERKGVADFLQEVTSKKDQEQYWAHRGEP
YSFVTVTEFSEAFQSFHVGRRLGDELAIPFDKAKAHTAALTTKKYGVSKKELLKACISRELLLMKRNSFVYIFK
MSQLILLAFIMMTLFLRTDMPRKTIADGWIFLGSMFFTLMMIMFNGFSELALTIMKLPVFYKQRDLLFYPSWAY
SLPTWILKIPITLVEVAIWVFMTYYVVGFDPNIERFFRQYLLLLCVNQMASGLLRLMAALGRNIIVANTFGSFAL
LAVLVMGGFVLSKDDVKPWWMWGYWISPMMYGQNAIAVNEFLGKSWRHVPENATEPLGVLVLKSRGIFPE
AYWYWLGVGALIGYVFLFNFLFTVALAYLNPYGKHQTVLSEETLTEQSSRGTSCTGGDKIRSGSSRSLSARVG
SFNNADQNRKRGMILPFEPLSITFDEIRYAVDMPQEMKSQGIPENRLELLKGVSGSFRPGVLTALMGVSGAGKT
TLMDVLAGRKTGGYIDGSIKISGYPKNQKTFARISGYCEQTDIHSPHVTVYESLLYSAWLRLPPEVDSATRKMFI
EEVMELVELNSLRQALVGLPGVDGLSTEQRKRLTVAVELVANPSIIFMDEPTSGLDARAAAIVMRTVRNTVDT
GRTVVCTIHQPSIDIFDAFDELFLLKRGGEEIYAGPLGHHSAHLIKYFEGIDGVSKIKDGYNPATWMLEVTSAAQ
EAALGINFTDVYKNSELYRRNKALIKELSTPPPGSKDLYFPTQYSQSFFAQCKTCLWKQHWSYWRNPSYTAVR
LLFTTFIALMFGTIFWDLGSRRQRKQDLFNAMGSMYCAVLFIGAQNATSVQPVVAIERTVFYREKAAGMYSAL
PYAFGQVMIELPYILIQTIIYGVIVYAMIGFDWTMTKFFWYIFFMYFTFLYFTFYGMMAVAVSPNHNIAAIISSAF
YAIWNLFSGFIVPRTRIPVWWRWYYWCCPISWTLYGLIGSQFGDMKDKLDTGETIEDFVRSYFGFRNDFLGIVA
VVIVGITVLFGFTFAYSIRAFNFQKR

>XP_010648603.1_P_PDR1_X1_Vv
MESSDVYRVNSARLSSSNIWRNSGMEVFSRSSRDEDDEEALKWAAIEKLPTYLRIRRGILAEEEGKAREIDITSL
GLIEKKNLLERLVKIAEEDNEKFLLKLKERIDRVGLDIPTIEVRFEHITVDAEAYIGGRALPTIINFSANMLEVIHF
LDYINLCMYTLCSNLLSCKQSHAMIFLSLKGFLNYLHILPSRKKPLPILHDVSGIIKPGRMTLLLGPPSSGKTTLL
LTLAGKLGSDLKLSGRVSYNGHGMDEFVPQRSSAYISQYDLHIGEMTVRETLAFSARCQGVGTGYDMLAELS
RREKVANIKPDPDIDIYMKAAALKGQGGSLITDYILKILGLEVCADTIVGDEMVRGISGGQKRRLTTGEMLVGP
AKALFMDEISTGLDSSTTFQIVNSIRQSIHILKGTAIISLLQPAPETYDLFDDIILLSDGQIVYQGPRENVLEFFEHM
GFKCPERKGVADFLQEVTSKKDQEQYWAHRGEPYSFVTVTEFSEAFQSFHVGRRLGDELAIPFDKAKAHTAAL
TTKKYGVSKKELLKACISRELLLMKRNSFVYIFKMSQLILLAFIMMTLFLRTDMPRKTIADGWIFLGSMFFTLM
MIMFNGFSELALTIMKLPVFYKQRDLLFYPSWAYSLPTWILKIPITLVEVAIWVFMTYYVVGFDPNIERFFRQYL
LLLCVNQMASGLLRLMAALGRNIIVANTFGSFALLAVLVMGGFVLSKDDVKPWWMWGYWISPMMYGQNAI
AVNEFLGKSWRHVPENATEPLGVLVLKSRGIFPEAYWYWLGVGALIGYVFLFNFLFTVALAYLNPYGKHQTV
LSEETLTEQSSRGTSCTGGDKIRSGSSRSLSARVGSFNNADQNRKRGMILPFEPLSITFDEIRYAVDMPQEMKSQ
GIPENRLELLKGVSGSFRPGVLTALMGVSGAGKTTLMDVLAGRKTGGYIDGSIKISGYPKNQKTFARISGYCEQ
TDIHSPHVTVYESLLYSAWLRLPPEVDSATRKMFIEEVMELVELNSLRQALVGLPGVDGLSTEQRKRLTVAVE
LVANPSIIFMDEPTSGLDARAAAIVMRTVRNTVDTGRTVVCTIHQPSIDIFDAFDELFLLKRGGEEIYAGPLGHH
SAHLIKYFEGIDGVSKIKDGYNPATWMLEVTSAAQEAALGINFTDVYKNSELYRRNKALIKELSTPPPGSKDLY
FPTQYSQSFFAQCKTCLWKQHWSYWRNPSYTAVRLLFTTFIALMFGTIFWDLGSRRQRKQDLFNAMGSMYCA
VLFIGAQNATSVQPVVAIERTVFYREKAAGMYSALPYAFGQVMIELPYILIQTIIYGVIVYAMIGFDWTMTKFF
WYIFFMYFTFLYFTFYGMMAVAVSPNHNIAAIISSAFYAIWNLFSGFIVPRTRIPVWWRWYYWCCPISWTLYGL
IGSQFGDMKDKLDTGETIEDFVRSYFGFRNDFLGIVAVVIVGITVLFGFTFAYSIRAFNFQKR

>XP_021606416.1_PDR1-l_Me
MEGGDITSRVSSARLSSSNIWRNTTLEVFSKSSSCNEDDEEALKWAALEKLPTYLRVRRGILTEEEGQSREIDIN
NLGFIEKRNLLERLVKIAEQDNEKFLLKLKNRIEKVGLDMPTIEVRFEHLTVETEAYVGSRALPTMFNFSANMF

EGFLNYLHILPSRKKPLSILNDVSGIIKPRRMTLLLGPPSSGKTTLLLALAGKLGKELKFSGKVTYNGHGMDEFV
PQRTSAYISQYDLHIGEMTVRETLAFSARCQGVGTRYEMLAELARREKAANIKPDPDIDIYMKAAALEGQEAN
VVTDYILKILGLEICADILVGDEMVRGISGGQKKRVTTGEMLVGPARALFMDEISTGLDSSTTFQIVNSLRQSIHI
LNGTALVSLLQPAPETYDLFDDIILLSDGQIVYQGPRENVLEFFEYMGFRCPERKGVADFLQEVTSKKDQEQY
WAFKDQPYSFVSVKEFAEAFQSFHVGRKLGDELATPFDKSKSHPASLTTKKYGVSKKELLKACISREYLLMKR
NSFVYIFKMTQLIIMAFVSMTIFLRTEMRRNTVADGGIYMGALFYTIIIIMFNGFSELAMTIMKLPVFYKQRDLLF
YPAWAYALPTWILKVPVTFVEVAVWVVMTYYVIGFDPNIGRFLKQYFILLITNQTSSALFRLTAALGRSVIVAN
TVGSFALLAVLILGGFIISRDSVKKWWIWGYWFSPMMYVQNGMSVNEFLGNSWNHFPPNSTEPLGVTVMKSR
GLFPEAYWYWIAVGALTGYIFLFNFLFTLALKYLDPFGRPQATISEEAYAEKTANETAEFIEQSSKGESSLEKGS
VSQRSASSRTPSTRVGSFSDVNQNRRRGMILPFQPLSITFDEIKYVVDMPQEMKAQGITEDRLELLKGVSGAFRP
GVLTALMGVSGAGKTTLMDVLAGRKTGGYIHGSISISGYPKKQETFARISGYCEQTDIHSPHVTVYESLLYSAW
LRLPPEVDSDTRKMFIEEVMELVELTNLRVALVGLPGVNGLSIEQRKRLTIAVELVANPSIIFMDEPTSGLDARA
AAIVMRTVRNTVDTGRTVVCTIHQPSIDIFDAFDELLLLKRGGEEIYVGPVGRHACHLIKYFEDIEGIPKVKDGY
NPATWMLEVTTAAQEVSLGINFSDIYKNSELYRRNKALIKELSIPPPGSRDLYFPTQYSQSFFTQCMACLWKQH
WSYWRNPPYSAVRLLFTTFIALMFGTIFWNLGSKRSRKQDLFNAMGSMYAAILFLGFQNSTSVQPVVAIERTV
FYRERAAGMYSELPYAFGQVVIELPYILVQTLIYGGIVYAMLGFEWTASKFLWYLFIMYFTLAYFTFYGMMTV
AITPNHNIAAIISSAFYGIWNLFSGFIIPRTRMPVWWRWNYWACPIAWTLYGLIASQYGDIKEELDSGETVEHFL
RSYFGFRHDFVGIVAIVLVGIAVFFGFTFAFSIKAFNFQHR

>XP_024445693.1_PDR1_Pt
MDGGGDIYRVSSARLSSSSNIWRNSTLDVFSRSSRDEDDEEALKWAAIEKLPTCLRMRRGILTEEEGQAREIDIA
SLGLIEKRNLVERLVKIAEEDNERFLLKLKERIHRVGLDIPTIEVRFEHLSIEAEAYVGGRALPTIFNFSANMLEG
FLSFLHILPSRKQPFPILHDLSGIIKPRRMTLLLGPPSSGKTTLLLALAGKLGKDLKSSGSVTYNGHGMAEFVPQR
TSAYISQYDLHIGEMTVRETLSFSARCQGVGPRYEMLTELSRREREANIKPDPDIDIFMKAAALEGQETTVTTD
YILKILGLDICADTMVGDEMIRGISGGQKKRLTTGEMLVGPARALFMDEISTGLDSSTTFQIANSLRQTTHILNG
TTFISLLQPAPETYDLFDDIILLSEGLIIYQGPRENVLEFFESLGFKCPERKGVADFLQEVTSRKDQEQYWACRDQ
PYSFVSAKEFSEAFQSFHIGRKLGDELATPFDKSKSHPAALTTEKYGVSKKELLKACISREFLLMKRNSFVYIFK
FTQLIILASITMTIFLRTEMHRNTIVDGGIYLGALFFAIIVIMFNGFSELAMTIMKLPIFYKQRDLLFYPPWAYAIPT
WILKIPITFVEVAIWTIMTYYVIGFDPNIGRFFKQYLIFVLANQMSSGLFRMTGALGRNIIVANTFGSFAFLAVLV
LGGFILSRDNVKPWWIWGYWVSPLMYVQNAASVNEFLGHSWRHIPPNSTESLGVVVLKSRGIFPEAHWYWIG
IGALIGYTLLFNFLFTLALKYLNPFGKPQAMLSKEALAERNANRTGELIELSTRGKSSSVRGIDSRRSSSARPPSL
RMHSFGDASQNKRGMVLPFQPLSITFDEIRYSVDMPQEMKAQGILEDRLELLKGVSGAFRPGVLTALMGVSGA
GKTTLMDVLSGRKTGGYIEGRISISGYPKNQQTFARISGYCEQMDIHSPHVTVYESLVYSAWLRLSPDVDSETR
KMFIEEVVELVELNPLREALVGLPGVNGLSTEQRKRLTIAVELVANPSIIFMDEPTSGLDARAAAIVMRTVRNT
VDTGRTVVCTIHQPSIDIFDAFDELFLLKRGGEEIYVGPVGRHACHLIKYLEEIEGVPKIKDGHNPATWMLEVTS
AAQEALLGVDFTDIYKNSELFRRNKALIKELSSPPPGSNDLYFPTQYSHSFFTQCMACLWKQHWSYWRNPPYT
AVRLLFTTFIALMFGTIFWDMGSKRRNRQDIFNSMGSMYAAVLFIGVQNATSVQPVVAIERTVFYRERAAGMY
SALPYAFAQVMIEIPYVLVQTLIYGVIVYTMIGFDWTVSKFFWYIFFMYFTLLYMTFYGMMTVAVTPNHNVAA
IVSSAFYAIWNLFSGFIVPRTRIPIWWRWYFWACPISWTLYGLIASQYGDIKDKLEGDETVEDFVRNYFGFRHDF
VGTCAIVIVGICVLFAFTFAFSIRAFNFQRR

>XP_024460182.1_PDR1_Pt
MDGAGDIYRVSSARLSTSSNKWRNSIPEVFSRSSRDEDDEEALKWAALEKLPTYLRLTRGILTEEEGKAREIDI
MNLGLVEKRDLLERLVKIAEEDNERFLLKLKERIDRVELEIPTIEVRFEHLNVEAEAYVGGRALPTILNFSANML
EGFLSFLHLLPSRKQPFPILRDVSGIIKPRRMTLLLGPPSSGKTTLLMALAGKLGKDLQCSGSVTYNGHGMEEFV
PQRTSAYISQFDLHIGEMTVRETLSFSARCQGVGPRYEMLTELSRREKEANIKPDPDLDIYMKAAALEGQETSV
TTYYILKITGLDICADTMVGDEMIRGISGGQKKRLTTGEMLVGPARALFMDEISTGLDSSTTFQIVNSLRQTTHI
LNGTTLISLLQPAPETYDLFDDVILLSDGLIVYQGPRENVLEFFESLGFKCPERKGVADFLQEVTSRKDQEQYW
ASRDQPYSFVSAKEFSEAFQSFHIGRKLGDELAIPFDKSKSHPSALSTEKYGVSKKELLKACISREFLLMKRNSF
VYIFKFTQLILLASIAMTVFLRTEMHRNTITDGGIYIGALFFAIIVIMFNGFSELVMTIMKLPVFYKQRDLLFYPP
WAYAIPTWILKIPITFVEVAIWTTMTYYAVGFDPNIGRFFKQYLIFVLANQMSSGLFRMMGALGRNVIVANNV
GSFALLAVLVMGGFILSRDNVKSWWIWGYWVSPLMYVQNAVSVNEFLGNSWRHIPPSSTESLGVTLLKSRGV
FPEARWYWIGVGALIGYTLLFNFLFTLALKYLNPFGKPQAILSKEALAERDANRTGNFIELSTRGKSSSERGKDS
KTNSSARAPSLRMPSLGDANQNKRGMVLPFQPLSITFEEIRYSVDMPQEMKAQGIPEDRLELLKGVSGAFRSGV

LTALMGVSGAGKTTLMDVLSGRKTGGYIDGRISISGYAKNQQTFARISGYCEQTDIHSPHVTVYESLVYSAWL
RLSPDVDSETRKMFIEEVMELVELNPLREALVGLPGVDGLSTEQRKRLTIAVELVANPSIIFMDEPTSGLDARAA
AIVMRAVRNTVDTGRTVVCTIHQPSIDIFDAFDELFLLKRGGEEIYVGPVGRHACHLIKYFEEIEGVPKIKDGYN
PATWMLEVTSAAQEAVLNDNFTDIFKNSELYRRNKALIKELSAPPPGSKDLYFPTRYSQSFFTQCMACLWKQH
WSYWRNPPYNAVRLLSTTVIALMFGTIFWNLGSKRNRKQDIFNSMGSMYAAVLFIGVQNATSVQPVVAIERTV
FYRERVAGMYSALPYAFAQVMIEIPYTLVQALIYGVIVYSMIGFEWTAIKFFWYIFFMYFTLLYMTFYGMMNV
AITPNHSIASLVSSAFYAIWNLFSGFIIPRTRVPIWWRWYCWACPFSWTLYGLIASQYGDLEDKLESDETVKDFL
RNYFGFRHDFVGICAIVVVGMSVLFAFTFAFSIRTFNFQRR

>XP_010648606.1_P_PDR1_Vv
MESSDISRVTSGRITASNILRNSSVEVFSRSSREEDDEEALKWAALEKLPTFLRIQRGILTEEKGQTREINIKSLGL
PERKNLIQRLVKIDGHDNEKFLLKLKERIDRVGLDIPTVEVRFEHLTVDAEAYVGSRALPTIFNFSANILEGFLN
YLHILPSRKKPFSILHDVSGIIKPRRMTLLLGPPSSGKTTLLLALAGRLGSDLKVSGRVTYNGHGMDEFVPQRTS
AYTSQYDLHAGEMTVRETLDFSARCQGVGGLSDMLAELSRREKAANIKPDPDIDIYMKAAALEGQKTSVVTE
YMLKILGLEICADTLVGDVMKQGISGGQKKRLTTGEILVGPARALFMDEISTGLDSSTAFQIVNSLRQSIHILNG
TALISLLQPAPETYNLFDDIILLSDGKIVYQGPCENVLEFFGYMGFKCPERKGVADFLQEVTSRKDQEQYWARK
DEPYSYVTVKEFAEAFQSFHIGQKLGDELAVPFDKTKGHPAALTTKKYGISKRELLRACTSREFLLMKRNSFVL
FFLFFQLIIVAFINMTLFLRTEMSRNTVEDGGIFMGALFFAVLMIMFNGFTELPMTIFQLPVFYKQRDLLFFPSWA
YSLPKWILKMPIAFAEVGAWVIMTYYVIGFDPNIERFFKQYLLLLCIHQMASGLLRLMAALGRNIIVANTFGSF
ALLVVMVLGGFVLSKDDVKTWWEWGYWVSPLMYGQNAISVNEFLGNSWRHVPANSTESLGVLVLKARGVF
TEPHWYWLGVGALIGYVLLFNFLFTLALSYLNPFGKSQPILSKETLTEKQANRTEELIELSPETGARIQSGSSRSL
SARVGSITEADQSRKRGMVLPFEPLSISFDEIRYAVDMPQEMKAQGITEDRLELLRGVSGSFRPGILTALMGVTG
AGKTTLMDVLAGRKTSGYIEGIIKVYGYPKKQETFARVLGYCEQTDIHSPHVTVYESLLYSAWLRLPSEVDSAT
RKMFIEEVMELVELNSLREALVGLPSENGLSTEQRKRLTIAVELVANPSIIFMDEPTSGLDARAAAIVMRTVRNT
VDTGRTVVCTIHQPSIDIFDAFDELLLLKRGGEEIYAGPIGRHSSHLIKYFEGINGVSKIKDGYNPSTWMLEVTSA
AQEVALGVNFTEEYKNSELYRRNKALIKELSSPPPGSKDLYFSTQYSQSFFTQCLACLWKQHWSYWRNPAYTA
VRLFFTTFIALMLGTIFWDFGSKRKRQQDLFNAMGSMYAAVISIGIQNASSVQAVVAIERTVFYRERAAGMYSP
FPYAFGQVMIELPHIFIQTIIYGLIVYAMVGFEWTVTKFFWYLFFMYFTFLYFTFYGMMAVAITPNQHISGIVSS
AFYGLWNLFSGFIIPHTRIPVWWKWYFWSCPVSWTLYGLLVTQFGDIKERLESGERVEDFVRSYFGYRNDFVG
VVAGIVVGITVLFGFIFAYSIRAFNFQKR

>XP_003526427.1_PDR1_Gm
MESGELRVASARIGSSGVWRSGSIDVFSGSSRRDDDEQELKWAAIEKLPTYLRMTRGILTETEGQPTEIDINKLC
PLQRKNLVERLVKIAEQDNEKFLFKLRDRIDRVGLEIPTIEIRFEHLNVEAEAHVGSRALPTIFNFCINLFEGFLNS
LHLIPSRKKPFTVLDDVSGIIKPKRMTLLLGPPSSGKTTLLLALAGRLSKDLKFSGRVSYNGHGMEEFVPQRTSA
YISQTDLHIGEMTVRETLAFSARCQGIGTRYEMLAELSRREKAANIKPDPDLDIYMKAAALEGQETNVVTDYI
MKILGLEVCADTMVGDDMIRGISGGQKKRVTTGEMLVGPARALFMDEISTGLDSSTTFQMVNSLRQSIHILNG
TAVISLLQPAPETYELFDDIILLSDGQIVYQGPRENVLEFFEYMGFKCPERKGVADFLQEVTSRKDQEQYWANK
DEPYSFVTVKEFAEAFQSFHAGRKLGDELATPFDMSKGHPAVLTKNKFGVCKKELLKACVSREFLLMKRNSF
VYIFKMWQLILTGFITMTLFLRTEMHRDTETDGGIYMGALFFVLIVIMFNGYSELSMSIMKLPVFYKQRDLLFFP
CWAYSLPTWILKIPITLVEVGIWVVMTYYVIGFDPSIERFIKQYFLLVCINQMASGLFRFMGAVGRNIIVANTVG
SFALLAVMVMGGFILSRVDVKKWWLWGYWFSPMMYGQNALAVNEFLGKSWSHVTPNSTEPLGVKVLKSRG
IFPKAYWYWIGVGASIGYMLLFNFLFPLALHYLDPFGKPQALISEEALAERNAGRNEHIIELSSRIKGSSDKGNES
RRNVSSRTLSARVGGIGASEHNKKRGMVLPFTPLSITFDEIRYSVEMPQEMKSQGILEDRLELLKGVNGAFRPG
VLTALMGVSGAGKTTLMDVLSGRKTAGYIQGQITISGYPKRQETFARIAGYCEQTDIHSPHVTVYESLVYSAW
LRLPPEVDSSTRQMFIEEVMELVELTSLREALVGLPGVNGLSTEQRKRLTIAVELVANPSIIFMDEPTSGLDARA
AAIVMRTVRNTVDTGRTVVCTIHQPSIDIFDAFDELLLLKRGGEEIYVGPLGQHCSHLINHFEGINGVPKIKNGY
NPATWMLEVTSEAQEAALGVNFAEIYKNSDLYRRNKALIRELTTPPTGSKDLYFPTKYSQTFFTQCMACLWKQ
HLSYWRNPPYSAVRLLFTTIIALLFGTIFWDIGSKRQRKQDLFNAMGSMYAAVLFIGIQNATSVQPVVAIERTVF
YRERAAGMYSALPYAFGQVAIEIPYIFIQTLVYGVIVYAMIGFDWTFSKFFWYLFFMFFTFLYFTFYGMMAVGL
TPDHNVAAIVSFGFYMIWNLFSGFVIPRTRMPVWWRWYFWICPVSWTLYGLVTSQFGDIKEPIDTGETVEEFV
RSYFGYRDDFVGVAAAVLVGFTLLFGFTFAFSIKAFNFQKR

>XP_021608284.1_PDR1-l_X1_Me

MEASDTGRVISSRRTHSFNGWTNNNTMEVFSKSSHHLEDDEEALKWAAVERLPTYLRVRRAILDKKEIDVNKI
GFLERRNLLERLVKIAEQDNETFLLKLRDRMERVGLDMPTIEVRFEHLNVEAEAYIGSRSLPTIFNFSINLLEGFL
NCLHIFPSRKKPLPILRDVSGIIKPRRMTLLLGPPSSGKTTLLLALAGKLGKDLKFSGRVTYNGHEMGEFVPQRT
AAYISQYDLHIAEMTVRETLAFSARCQGVGPRYEMLAELSRREKAANIKPDPDIDVYMKAAALEGQEANVVA
DYILKILGLEGCADTMVGDEMIRGISGGEKKRVTTGEMLVGPARALFMDDISTGLDSSTTFQIVNSLRQSIHILS
GTALVSLLQPAPETYDLFDDIILLSDGQIVYQGPRENVLEFFEHMGFKCPERKGVADFLQEVTSRKDQEQYWA
LKDLPYSYVSVKEFAEAFQSFHVGRKLGDELATPFDKSKCHPAALTTKKYGISKKELLKACFSRELLLMKRNSF
IYIFKMTQLVIMALISVTVFLRTEMHRETLTDGGIYLGALFFAIVTLMFNGFTELALTIMKLPVFYKQRDLLFYPS
WAYALPTWILKIPVTFVEVAIWVIITYYVIGFDPNIERFFKQYLILLCTNQMASGLFRLMAALGRNIIVANTVGSF
ALLVVLVLGGFVISRDNINKWWLWGYWISPLTYVQNAISVNEFLGKNWRHVPFLSTEPLGVGLLKSRGIFLEA
HWYWIGVGALIGYILLFNFLYTLALKYLDPFGKPQATLSKEVLAEQNANRTGEFSKSSTSGKSYLERGNESHKS
ISSRTLSARVDSFNDANQNKKRGIVLPFQPLSIAFNEIKYAVDMPKEMQAQGIPEDRLELLKGISGAFRPGVLTA
LIGISGAGKTTLMDVLAGRKTDGYIEGSIFISGYPKKQETFARISGYCEQTDIHSPHVTVYESLLYSAWLRLPPEV
NSHTRKMFIEEIMELVELTSLREALVGLPGVNGLSTEQRKRLTIAIELVANPSIIFMDEPTSGLDARAAAIVMRTV
RNTVDTGRTVVCTIHQPSIDIFDAFDELLLLQRGGEEIYAGPIGRHSCHLIKYFEDIKGVPKIKDGYNPATWMLEI
TSAAQEAALGINFADVYKNSELYRKSKAFIKELSTPQPGSKELYFLSHYSQPFLTQCMACLWKQHWSYWRNPS
YTAVKLLFTTVIALMFGTIFWDLGCKRRRQQDIFNAIGSMYVALIFIGVQNAVSVQPVVAIERTVFYRERAAGM
YSALPYAFGQVMIELPYVFIQTIIYGVIVYAMIGFDWTLSKFFWYIFFMYFTFLYFSFYGMMTTAITPDHNIAAV
VASAFYALWNLFSGFIIPLPRIPVWWKWYYWSCPLAWTLYGLVASQFGDFKDMLETGEPLDLFLRRYFGFRH
DFVRIAAVVTIGISVLFAFIFALAIKSLNFQKR

>XP_015160196.1_P_PDR1-l_X2_St
MDEFVPQRTSAYISQNDLHIGEMTVRETLAFSARCQGVGTKYEILAELSRREKEANIKPDPDIDIFMKSAWNEG
QEANVITDYTLKILGLEICADTLVGDEMIRGISGGQRKRLTTGEMMVGPARALFMDEISTGLDSSTTYQIVNSIR
QSIHILQGTAVISLLQPAPETYDLFDDIILLSDGQIVYQGPRENVLEFFEYLGFKCPQRKGVADFLQEVTSRKDQE
QYWSRRDEPYRFITACEFSDVFQSFDVGRKLGDELAVPFDKSKSHPAALTTKRYGISKKELLKACTAREYLLM
KRNSFVYIFKMVQLTLMASIAMTLFLRTEMHRDTTIDGAIYLGALFYAVITIMFNGFSELALSIMKLPSFYKQRD
FLFFPAWAYALPTWILKIPITLVEIAIWVCMTYYVIGFEADVGRFFKQIFLLICLSQMASGLFRFLAALGRNIIVA
NTFGSCALLIVLVMGGFILSRDDVKQWLIWGYWISPMMYAQNAIAVNEFLGKSWAHVPPNSTGTDTLGVSFL
KSRGIFPEARWYWIGAGALFGYVLLFNFLFTVALAYLNPFSKPQAILSEEIVAERNASKRGEVIELSPIGKSSSER
GNDVPVSTSSRSLSTRVGNITEGDLNKRKGMILPFEPLSITFDDIRYAVDMPQEMKTQGFIEDRLELLKGVSGAF
RPGVLTALMGVSGAGKTTLMDVLAGRKTGGYVEGTISISGYPKQQETFARISGYCEQTDIHSPHVTVYESLLYS
AWLRLPREVDTETRKSFIEEVMELVELTPLREALVGLPGVNGLSTEQRKRLTVAVELVANPSIIFMDEPTSGLD
ARAAAIVMRTVRNTVDTGRTVVCTIHQPSIDIFDAFDELLLLKRGGEEIFVGPLGRHSSHLIKYFEGIDGVLKIRD
GYNPATWMLEVTSLAQEAVLGIDFTELYKNSELYRRNKALIQELSVPASGSKDLYFETKYSQSFFTQCMACLW
KQHWSYWRNPPYTAVRLMFTFFVSLMLGTIFWGLGSKRGKQQDILNAIGSMYAAILFLGIINASSVQPVVAIER
TVFYRERAAGMYSALPYAFGQVMIELPHLFLQTIIYGVIVYAMIGFEWTVTKFFWYLFFMYFTLLYFTLYGMM
TVAVTPNHTIASIVSSAFYTIWNLFCGFVVPKTRMPVWWRWYYYICPLSWTLYGLIASQFGDLQDRLDTKETV
EEFLENFFDYKHDFVGYVAVILVGISVVFLFIFAYSIKSFNFQKR

>XP_021610345.1_PDR1-l_Me
MESADLYRASSSLRRGSFSTWRNNPSDVFTRSSREEDDEEALKWAALEKLPTYDRLRKGIFLSASKGAVNEIDV
DNLGFQERKTLLERLVKVAEEDNEKFLLKLKNRIDRVGIEIPTIEVRFEHLNIEADAYVGSSALPSFINFSVHMLE
GFLNYLHVLPSRKRPLTILKDVSGVIKPSRMTLLLGPPSSGKTTLLLALAGKLDPNLKFSGTVTYNGHGMDEFIP
QRTAAYISQHDVHIGEMTVRETLAFSARCQGVGTQHELLAELSRREKDANIKPDPDIDVFMKAAATEGQETSV
VTDYILKILGLEICADTLVGNEMIRGISGGQRKRVTTGEMLVGPAKALFMDEISTGLDSSTTYQIVNSLKQSIHIL
NGTAVISLLQPAPETYDLFDDIILLSDGQIVYQGPREQVLGFFEYMGFKCPERKGVADFLQEVTSRKDQKQYW
ARRDQPYSFVTVQEFAEAFQSYDVGRRIGDELSTPFDKTKSHPAALSTKKFGVGKMELLKACMSREYLLMKR
NSFVYIFKLTQLTFMAIIMMTLFLRTEMHRDNIMDGGVYLGALFFTVIMVMFNGMAELSMTIAKLPVFYKQRE
LLFYPAWAYSIPTWILKIPVTFVEVAVWVFLTYYVVGFDPNVTRFFKHYFVLLLVNQMASALFRCIAATGRNVI
VANTFGSFSLLTLFALGGFVLSRDEIKKWWIWGYWMSPLMYGQNAIVANEFLGKSWSHIPPNSTESLGVQVM
KGRGFFPDAYWYWLGAGASAGFIIVFNICFALALTFLDPFEKPQAVITEDSQSNDPDDEDGGDILLTNNGSSHK
SSTGAGEEIRQVNHNKKKGMVLPFEPHSVAFDNVVYSVDMPQEMKSQGVLEDKLVLLKGVSGAFRPGVLTAL
MGVSGAGKTTLMDVLAGRKTGGYIEGNITISGFPKKQETFARISGYCEQNDIHSPHVTVYESLVYSAWLRLPPE

VDSKTRKMFVDEVMELVELNPLRQALVGLPGVSGLSTEQRKRLTIAVELVANPSIIFMDEPTSGLDARAAAIV
MRTVRNTVDTGRTVVCTIHQPSIDIFDAFDELFLMKRGGEEIYVGPLGRHSCHLIKYFEGIEGVSKITDGYNPAT
WMLDITSYAQELALNVDFAAIYKNSELYRRNKAMIMELSTPAPGSKDLYFPTQYSQSFLTQCIACIWKQRLSY
WRNPPYTAVRFLFTTFIALMFGTMFWKIGSKLKKQQDLFNAAGAMYAAVLFLGVQNASSVQPVVAIERTVFY
RERAAGMYSAMPYAYAQVLVELPYVFAQAIFYGTITYAMMGFEWTIAKFFWYLFFMYFTLLYFTLYGMMTV
AVTPNHHIAAIVSSAFYGMWNIFAGFILPRTRMPVWWRWYFWICPVAWTLYGLIASQFGDVKDVLANGQTVE
DFIREYYGYKHDFVGVTACVIVGIVVLFAFIFGISIRSFNFQRR

>XP_014630001.1_PDR1_Gm
MESGELRVASARIGSSSVWRSSGGVDVFSGSSRRDDDEQELKWAAIEKLPTYLRMTRGILTEAEGQPTEIDINK
LCPLQRKNLVERLVKIAEQDNEKFLFKLRDRIDSVGLEIPAIEVRFEHLNVEAEAHVGSRALPTIFNFCINLLEGF
LNSLHLIPSRKKPFTVLDDVSGIIKPKRMSLLLGPPSSGKTTLLLALAGRLGKDLKFSGRVSYNGHGMEEFVPQR
TSAYISQTDLHIGEMTVRETLAFSARCQGIGTRNEMLAELSRREKAANIKPDPDLDIYMKAAALEGQETNVVTD
YIMKILGLEICADTMVGDDMIRGISGGQKKRVTTGEMLVGPARALLMDEISTGLDSSTTFQMVNSLRQSIHILN
GTAVISLLQPAPETYELFDDIILLSDGQIVYQGPRENVLEFFEYMGFKCPERKGVADFLQEVTSRKDQEQYWAN
KDEPYSFVTVKEFAEAFQSFHVGRKLGDELATPFDMSKGHPAVLTKNKYGVCKKELLKACVSREFLLMKRNS
FVYIFKMWQLILTGFITMTLFLRTEMHRDTETDGGIYMGALFFVLIVIMFNGYSELSMSIMKLPVFYKQRDLLFF
PCWAYSLPTWILKIPITLVEVGIWVVMTYYVIGFDPSIERFIKQYFLLVCINQMASGLFRFMGAVGRNIIVANTV
GSFALLAVMVMGGFILSRVDVKKWWLWGYWFSPMMYGQNALAVNEFLGKSWSHVPPNSTEPLGVKVLKSR
GIFPEAYWYWIGVGASIGYMLLFNFLFPLALHYLDPFGKPQALISEEALAERNAGRNEHIIELSSRIKGSSDRGNE
SRRNMSSRTLSARVGSIGASEHNKKRGMVLPFTPLSITFDEIRYSVEMPQEMKSQGILEDRLELLKGVNGVFRP
GVLTALMGVSGAGKTTLMDVLSGRKTAGYVQGQITISGYPKKQETFARIAGYCEQTDIHSPHVTVYESLVYSA
WLRLPPEVDSVTRQMFIEEVMELVELTSLREALVGLPGVNGLSTEQRKRLTIAVELVANPSIIFMDEPTSGLDAR
AAAIVMRTVRNTVDTGRTVVCTIHQPSIDIFDAFDELLLLKRGGEEIYVGPLGQCCSQLINYFEGINGVPKIKKG
YNPATWMLEVTSEAQEAALGLNFAEIYKNSDLYRRNKALIRELSTPTTGFKDLYFPTKYSQTFITQCMACLWK
QHLSYWRNPPYSAVRLLFTTIIALLFGTIFWDIGSKRQRKQDLFNAMGSMYAAVLFIGIQNATSVQPVVAIERT
VFYRERAAGMYSALPYAFGQVAIEIPYIFIQTLVYGVIVYAMIGFDWTFSKFFWYLFFMFFTFLYFTFYGMMAV
GLTPDHNVATIVSFGFYMIWNLFSGFVIPRTRMPVWWRWYFWICPVSWTLYGLVTSQFGDIKERIDTGETVEE
FVRSYFGYRDDFVGVAAAVLVGFTLLFGFTFAFSIKAFNFQKR

>XP_010654715.1_P_PDR1-l_Vv
MATGEIYRAGGSLRKDSSSIWRNSGEEVFSRSSRDEDDEEALKWAALEKLPTYNRMRKGLLMGSAGEASEVDI
HNLGFQEKKNLVERLVKIAEEDNEKFLLKLRNRIDRVGIDLPEIEVRFEHLTIDAEAHVGSRALPSFIYSAFNQIE
DILNTLRILPSRKKKLTILHDVSGIIKPRRMTLLLGPPSSGKTTLLLALSGKLDSSLKVTGKVTYNGHGMNEFVP
QRTATYISQHDTHIGEMTVRETLAFSARCQGVGDRYDMLAELSRREKAANIKPDPDIDVFMKAVATEGQKEN
VITDYTLKILGLEVCADTLVGDQMIRGISGGQRKRVTTGEMLVGPSKALFMDEISTGLDSSTTYQIVNSLRQTIH
ILNGTALISLLQPAPETYDLFDDIILLSDSQIVYQGPREDVLDFFESMGFRCPERKGVADFLQEVTSRKDQQQYW
ARKDEPYSFVTVKEFAEAFQSFHIGRKLGHELATPFDKTKSHPAALKTEKYGVRKKELLDACISREYLLMKRN
SFVYIFKLTQLIIMAAISMTIFLRTEMHKNSTDDGSIYTGALFFTVVMIMFNGMSELAMTIAKLPVFYKQRGLLF
YPAWAYALPSWILKIPITFVEVAVWVFMSYYVIGFDPNVGRLFKQYLLLVLVNQMASALFRFIAAAGRNMIVA
NTFGSFSLLLLFALGGFVLSRENVKKWWIWGYWSSPLMYAQNAIVVNEFLGKSWSKNSSTDSTESLGVAVLK
SRGFFTEAYWYWIGAGALLGFILVFNFCYTVALTYLNAFEKPQAVITEESENSKTGGKIELSSHRRGSIDQTAST
ERRDEIGRSISSTSSSVRAEAIAEARRNNKKGMVLPFQPLSITFDDIRYSVDMPEEMKSQGVLEDRLELLKGVSG
AFRPGVLTALMGVSGAGKTTLMDVLAGRKTGGYIEGNINISGYPKKQETFARISGYCEQNDIHSPHVTIHESLL
YSAWLRLPADVDSKTRKMFIEEVMELVELTPLKDSLVGLPGVNGLSTEQRKRLTIAVELVANPSIIFMDEPTSG
LDARAAAIVMRTVRNTVDTGRTVVCTIHQPSIDIFEAFDELLLLKRGGQEIYVGPLGRHSSHLIKYFQGIEGVSKI
KDGYNPATWMLEVTSSAQEFLLGVDFTEIYKNSDLYRRNKDLIKELSQPAPGSKDLYFPTQYSQSFFTQCMAC
LWKQRRSYWRNPPYTAVRFFFTTFIALIFGTMFWDLGTKRKKQQDLSNAMGSMYAAVLFLGVQNSSSVQPVV
AVERTVFYRERAAGMYSAMPYAFAQALVEIPYVFAQAVVYGVIVYAMIGFEWTAAKFFWYLFFMFFTLLYFT
FYGMMAVAATPNQHIAAIVAAAFYGLWNLFSGFIVPRTRIPVWWRWYYWACPVAWTLYGLVTSQFGDIQDR
FEDTGDTVEQYLNDYFGFEHDFLGVVAAVIVGFTVLFLFIFAFAIKAFNFQRR

>XP_021628888.1_PDR1-l_Me

MESGDIYKASSSLRRGSFSTWKHHPSGIFSTSSREEDDEEALKWAALEKLPTYDRLRKGILLSASKGAVNEIDV
DNLGFQERKTLLERLVKVAEEDNEKFLFKLKNRIDRVGIEIPTIEVRFEHLNVEADAFVGSRALPSFINFSVNML
EGFLNYIPIFPSRKRPLTILKDVSGVIKPSRMTLLLGPPSSGKTTLLLALAGKLDPNLKFSGTVTYNGYKMNEFIP
QRTAAYISQHDVHLGEMTVRETLAFSARCQGVGTQHELLAELSRREKAANIKPDPDIDVFMKAAATEGQETSV
VTDYILKILGLEICADILVGDEMVRGISGGQRKRVTTGEMLVGPAKALFMDEISTGLDSSTTYQIVNSLKQSIHIL
NGTAVISLLQPAPETYDLFDDIILLSDGQIVYQGPREQVLGFFEHMGFKCPERKGVADFLQEVTSRKDQKQYW
ARRGQPHRFITVQEFSEAFQSYELGRRIADELSTPFDKTKSHPAALSTKKYGVGKMELLKACISREFLLMKRNS
FVYIFKLTQLTFMASVSMTLFLRTEMHRNNLTDGGIYLGALFFSVIMVMFNGMAELSLTIAKLPVFYKQRDFLF
YPAWAYSLPTWILKTPVTFVEAAVWTVLTYYEIGFDPNVVRFFKHYLVLLLVNQMASSLFRFIAATGRNVIVA
NTFGSFSLLTLFALGGFILSREEIKKWWIWGYWISPLMYGQHAVVVNEFLAKSWRHIPPNSTEPLGVQLLKTRG
FFPHAYWYWLGVGALGGFVLLFNFCFTLALTFLDPFEKPQAVIVEDSQSNEPGDENGKGAGEEISEANHNKKK
GMVLPFEPRSVAFDNVMYSVDMPQEMKSQGVLEDKLVLLKNVSGAFRPGVLTALMGVSGAGKTTLMDVLA
GRKTGGYIEGDIRISGYPKKQETFARVSGYCEQNDIHSPFVTVYESLVYSAWLRLPPEVDSDTRKMFVDEIMEL
VELDPLKQALVGLPGVSGLSTEQRKRLTIAVELVANPSIIFMDEPTSGLDARAAAIVMRTVRNTVDTGRTVVCT
IHQPSIDIFDAFDELFLMKRGGEEIYVGPLGRLSCHLIKYFEGIEGVSKITDGYNPATWMLEVTSSAQELALNVD
FAAIYKNSELYRRNKEMIKELSTPAPGSTDLYFPTKYSQSFFTQCIACLWKQRLSYWRNPPYTAVRFLFTTFIAL
MFGTMFWDLGSKKTKQQDIFNAAGSMYAAVFFLGVQNASSVQPVVAIERTVFYRERAAGMYSAMPYAYAQ
VLVELPYIFAQAIVYAVITYAMMGFEWTGIKFFWYIFFMYFTLLYFTFYGMMTVAVTPNHHIAAIVSSAFYGL
WNLFAGFIVPRTKMPVWWRWYYWGCPVSWTLYGLVASQYGDVKDVLDTNQTVEDFVREYYGFKHDFVGVI
AGGIVGISVLFAFIFGFSIRFFNFQRR

>XP_010654718.1_P_PDR1_Vv
METAEIYTASGRRASGSFRKNSSSIWRNSGAEVFSRSSRDEDDEEALKWAALEKLPTYNRLRKGLLIGSEGEAS
EVDIHNLGPQERKNLVERLVKIAEEDNEKFLLKLKNRMDRVGIDLPEIEVRFEHLTIDAEAHVGSRALPSFINSV
FNQIEDILNTLRILPSRKKKFTILHDVSGIIKPGRMTLLLGPPSSGKTTLLLALSGKLDSSLKVTGRVTYNGHGMN
EFVPQRTAAYISQLDTHIGEMTVRETLAFSARCQGVGDRYDMLVELSRREKAANIKPDPDIDVFMKAAAAEGQ
KENVITDYTLKILGLEICADTMVGDEMVRGISGGQRKRVTTGEMLVGPSKALFMDEISTGLDSSTTYQIVNSLR
QTVHILNGTALISLLQPAPETYDLFDDIILLSDSRIIYQGPREDVLNFFESMGFRCPERKGVADFLQEVTSRKDQE
QYWAHKDEPYSFVTAKEFAEAFQSFHFGRKLGDELATPFDKTKSHPAALKTEKYGVRKKELLDACISREYLL
MKRNSFVYIFKLTQLTIVAMIAMTIFLRTEMHKNTTEDGNIYTGALFFTVMMVMFNGMSELAMTILKLPVFYK
QRGLLFYPAWAYALPSWFLKIPITFVEVGVWVFITYYVIGFDPNVGRLFRQYLLLLLLNQTASSLFRFIAAACRS
MIVANTFGSFALVLPFALGGIVLSRENVKKWWIWGYWSSPMMYAQNAILVNEFLGKSWSKNASTNSTESLGV
AVLKARGFFTEAHWYWIGAGALLGFIFVFNFCYTVALTYLNPFEKPQAVITVESDNAKTEGKIELSSHRKGSID
QTASTESGEEIGRSISSVSSSVRAEAIAEARRNNKKGMVLPFQPLSITFDDIRYSVDMPEEMKSQGVPEDRLELL
KGVSGAFRPGVLTALMGVSGAGKTTLMDVLAGRKTGGYIEGSISISGYPKKQETFARISGYCEQNDIHSPHVTV
HESLLYSAWLRLPPNVDAETRKMFIEEVMELVELTPLRGALVGLPGVNGLSTEQRKRLTIAVELVANPSIIFMD
EPTSGLDARAAAIVMRTVRNTVDTGRTVVCTIHQPSIDIFDAFDELLLLKRGGQEIYMGPLGRHSSHLIKYFEGI
EGVSKIKDGYNPATWMLEVTASAQELILGVDFTEIYEKSDLYRRNKDLIKELSQPTPGSKDLYFPTQYSQSFFTQ
CMACLWKQRLSYWRNPPYTAVRFFFTTFVALMFGTMFWDLGTKRTRQQDISNAMGSMYAAVLFLGFQNGQ
SVQPVVAVERTVFYRERAAGMYSAMPYAFAQALVEIPYVFSQAVVYGVIVYAMIGFEWTAAKFFWYLFFMFF
SLLYFTFYGMMAVAATPNQHIAAIVASSFYTLWNLFSGFIVPRNRIPVWWRWYYWACPVAWSLYGLVTSQFG
DIEDTLLDSNVTVKQYLDDYFGFKHDFLGVVAVVIVGFTVLFLFIFAFAIKAFNFQRR

>XP_010654722.1_P_PDR1_Vv
MATADTYRASGSLRRNGSSIWRSSGADVFSRSSRDEDDEEALKWAALEKLPTYNRLRRGLLMGSEGEASEIDI
HNLGFQEKKNLVERLVKVAEEDNEKFLLKLKNRIDRVGIDVPEIEVRFEHLTIDAEAFVGSRALPSFHNFIFNKL
EGILNAVRILPSKKRKFTILNDVSGIIKPRRLTLLLGPPSSGKTTLLLALAGKLDPNLKVMGRVTYNGHGMNEFV
PQRTAAYISQHDTHIGEMTVRETLAFSARCQGVGDRYDMLAELSRREKAANIKPDPDLDVFMKAAATEGQKE
NVVTDYTLKILGLDICADTMVGDEMIRGISGGQRKRVTTGEMLVGPSKALFMDEISTGLDSSTTFQIINSLKQTI
HILNGTAVISLLQPAPETYNLFDDIILLSDSQIVYQGPREDVLEFFESIGFKCPERKGEADFLQEVTSRKDQAQYW
ARKDVPYSFVTVKEFAEAFQSFHIGRKVADELASPFDRAKSHPAALTTKKYGVRKKELLDANMSREYLLMKR
NSFVYIFKLTQLAVVAVIAMTLFLRTEMNKNSTEDGSIYTGALFFTVVMIMFNGMAELAMTIAKLPVFYKQRD
FLFYPAWAYALPTWVLKIPITFVEVAVWVFITYYVIGFDPNVERLFRQYLLLLLVNQMASGLFRFIAAAGRNMI
VASTFGAFAVLMLMALGGFILSHDNVKKWWIWGYWSSPLMYAQNAIVVNEFLGKSWSKNVTNSTESLGITV

LKSRGFFTDAHWYWIGAGALLGFIFVFNFFYTLCLNYLNPFEKPQAVITEESDNAKTATTERGEHMVEAIAEGN
HNKKKGMVLPFQPHSITFDDIRYSVDMPEEMKSQGALEDRLELLKGVSGAFRPGVLTALMGVSGAGKTTLMD
VLAGRKTGGYIEGNISISGYPKKQETFARISGYCEQNDIHSPHVTVHESLLYSAWLRLPSDVNSETRKMFIEEVM
ELVELTPLRDALVGLPGVNGLSTEQRKRLTIAVELVANPSIIFMDEPTSGLDARAAAIVMRTVRNTVDTGRTVV
CTIHQPSIDIFEAFDELLLMKRGGQEIYVGPLGRHSSHLINYFEGIEGVSKIKDGYNPATWMLEVTTGAQEGTLG
VDFTEIYKNSDLYRRNKDLIKELSQPAPGTKDLYFATQYSQPFFTQFLACLWKQRWSYWRNPPYTAVRFLFTT
FIALMFGLIFWDLGTRRTRQQDLLNAMGSMYAAVLFLGVQNAQSVQPVIVVERTVFYRERAAGMYSALPYAF
GQALVEIPYVFAQAVVYGVIVYGMIGFEWTATKFFWYLFFMFCTLLYFTFYGMMAVAATPNQHIASIIAATFY
TLWNLFSGFIVPRNRIPVWWRWYCWICPVAWTLYGLVASQFGDIQSTLLENNQTVKQFLDDYFGFKHDFLGV
VAAVVVGFVVLFLFIFAYAIKAFNFQKR

>XP_021609606.1_PDR1-l_Me
MDGADLYRASSSIRRSSSIWRNNSADVFSRSSREEDDEEALKWAVLEKLPTYDRLRKGILISGSKGEANEVEIDS
LGFQERKSLLERLVRVTEEDNEKFLLKLKDRIDRVGIEVPTIEVRFEHLNIEAEALVGTSALPTFFNFLINILEGFL
NNLHVFPSRKKPFTILKDISGVIKPSRMTLLLGPPSSGKTTLLLALAGKLDPNLKFSGSVTYNGHGLNEFIAQRT
AAYISQHDLHIGEMTVRETLGFSARCQGVGCLQDMLAELSRREKAANIKPDPDIDVFMKAAATEGQEASLVTD
YILKILGLDVCADTLVGDEMLRGISGGQRKRVTTGEMLVGPSRALFMDEISTGLDSSTTYQIVNSLKQSIHILNG
TAVISLLQPAPETYDLFDDIILLADGQIVYQGPREDVLGFFEYMGFKCPERKGVADFLQEVTSRKDQQQYWAN
RDQPYSFISVQEFSEAFQSYDVGQRLGQELSTPFDKAKSHPAALPTVKYGVGMVELLKACFSREYLLMKRNSF
VYIFKLIQLTTMAIIGMTLFLRTNMHRDNLIDGGIYLGALFFSVVMIMFNGMSELSMTIAKLPVFYKQRDLLFYP
SWAYSLPSWILKIPISFLEVAVWVFITYYVMGFDPNVGRLFKQYILLFLVNQMASALFRFIAAVGRNMIVANTF
GSFALLILFALGGVVLSREEIKKWWIWGYWLSPMMYGQNAIVVNEFLGKSWSHIPPNSTESLGVLLMKSRGFF
PHAYWYWIGVGASAGFVLLFNLCFTLALTFLNPFEKPQAVISDEPETSGRLESRHTTNTENEMSDIDESNHKKK
KGMVLPFEPHSITFDNVIYSVDMPQEMRNQGIAEDKLVLLKGVSGAFRPGVLTALMGVSGAGKTTLMDVLAG
RKTGGYIEGSIKISGYPKKQETFARISGYCEQNDIHSPHVTVYESLIYSAWLRLPPEVDSETRKMFVDEVMELVE
LNPLRQALVGLPGVNGLSTEQRKRLTIAVELVANPSIIFMDEPTSGLDARAAAIVMRTVRNTVDTGRTVVCTIH
QPSIDIFEAFDELFLMKRGGEEIYVGPLGRHSSRLINYFEEINGVSKITDGYNPATWMLEVTSSAQELSLGVDFA
VIYKNSELYRRNKATIEALSTPAPGSKDLYFPTQYSQSFFTQCLACLWKQRLSYWRNPPYTAVRFLFTTFISLMF
GTMFWDLGTKTSKQQDIFNSLGSMYAAVLFIGIQNAASVQPVVAVERTVFYRERAAGMYSAMPYAYAQVLIE
LPYIFIQAAVYGLITYAMIGFEWTAAKFFWYLFFMYFTLLYFTYYGMMTVAVTPNQHIASIISSAFYAIWNLFSG
FIIPRTRMPVWWRWFYWVCPVSWTLYGLIASQFADIKDPIEGGVTVEQFVKAYYGVKHDFLGVVAAMIVGFT
VLFAFIFAVSVRSFNFQKR

>XP_010654716.1_P_PDR1_Vv
MATGEIYRAGGSLRKDSSSIWRNSGEEVSSRSSRDEDDEEALKWAALEKLPTYNRMRKGLLMGSAGEASEVDI
HNLGFQEKKNLVERLVKIAEEDNEKFLLKLRNRIDRVGIDLPEIEVRFEHLTIDAEAHVGSRALPSFINSAFNQIE
DILNTLRILPSRKKKFTILHDVSGIIKPRRMTLLLGPPSSGKTTLLLALSGKLDSSLKVTGKVTYNGHGMNEFVP
QRTATYISQHDTHIGEMTVRETLAFSARCQGVGDRYDMLAELSRREKAANIKPDPDIDVFMKAAATEGQKEN
VITDYTLKILGLEVCADTLVGDQMIRGISGGQRKRVTTGEMLVGPSKALFMDEISTGLDSSTTYQIVNSLRQTIH
ILNGTALISLLQPAPETYDLFDDIILLSDSQIVYQGPREDVLDFFESMGFRCPERKGVADFLQEVTSRKDQQQYW
ARKDEPYSFVTVKQFAEAFQSFHSGRKVGDELATPFDKTKSHPAALKTEKYGVRKKELLDACISREYWLMKR
NSFVYILQLTQLIIMAAISMTIFLRTEMHKNSTDDGSIYMGALFFTVVMIMFNGMSELAMTIAKLPVFYKQRGL
LFYPAWAYALSSWILKIPITFVEVAVWVFMSYYVIGFDPNVGRLFKQYLLLVLVNQMASALFRFIAAAGRNMI
VANTFGSFSLLLLFALGGFVLSRENVKKWWIWGYWSSPLMYAQNAIVVNEFLGKSWSKNSSTNSTESLGVAV
LKSRGFFTEAYWYWIGAGALLGFILVFNFCYTVALTYLNAFEKPQAVITEESENSKTGGKIELSSHRRGSIDQTA
STERRDEIGRSISSTSSSVRAEAIAEARRNTKRGMVLPFQPLSITFDDIRYSVDMPEEMKSQGVLEDRLKLLKGV
SGAFRPGVLTALMGVSGAGKTTLMDVLAGRKTGGYIEGNINISGYPKKQETFTRISGYCEQNDIHSPHVTIHES
LLYSAWLRLPADVDSKTRKMFIEKVMELVELTPLKDSLVGLPGVNGLSTEQRKRLTIAVELVANPSIIFMDEPT
SGLDARAAAIVMRTVRNTVDTGRTVVCTIHQPSIDIFEAFDELLLLKRGGQEIYVGLLGRHSSCLIKYFEGIEGV
SKIKGGYNPATWMLEVTTSAQEFLLGVDFTEIYKNSNLYRRNKDLIKELSQPAPGSKDLYFPTQYSQSFFTQCM
ACLWKQRRSYWRNPPYTAVRFFFTTFIALIFGTMFWDLGTKRTKQQDLSNAMGSMYAAVLFLGVQNSSSVQP
VVAVERTVFYRERAAGIYSAMPYAFAHALVEIPYVFAQAVVYGVIVYAMIGFEWTAAKFFWYLFFMFFTLLY
FTFYGMMAVAATPNQHIAAIVAAAFYGLWNLFSGFIVPRTRIPVWWRWYYWACPVAWTLYGLVTSQFGDIQ
DRFEDTGDTVEQYLNDYFGFEHDFLGVVAAVIVGFTVLFLFIFAFAIKAFNFQRR

>XP_010654717.1_P_PDR1_Vv
MATAEIYRASGSLRKDSSSIWRNSGAEVFSRTSGDEDDEEALKWAALEKLPTYNRMRKGLLMGSEGEANEVD
IHNLGLQERKNLVERLVKIADEDNEKFLLKLKNRIDRVGIDLPEIEVRFEHLTIDAEAYVGSRALPSFINSAFNQI
EDILNALRILPSRKKKFTILHDVSGIIKPRRMTLLLGPPSSGKTTLLLALSGKLDSSLKVMGSVTYNGHGMNEFV
PQRTAAYISQLDTHIGEMTVRETLAFSARCQGVGDRYDMLAELSRREKSANIKPDPDIDVFMKAVAAEGQKEN
VITDYTLKILGLEVCADTMVGDEMVRGISGGQRKRVTTGEMLVGPSKALFMDEISTGLDSSTTYQIVNSLRQNI
HIFKGTALISLLQPAPETYNLFDDIILLSDSQIVYQGPREDVLDFFESMGFRCPERKGVADFLQEVTSRKDQEQY
WICKDEPYSFVTVKEFAEAFQSFHIGRKLGDELATPFDKTKSHPAAMKTEKYGVRKKELLDACIAREYLLMKR
NSFVYIFKLTQLTIMAVIGMTIFLRTEMHKNTTEDGNIYTGALFFIVITVMFNGMSELAMTIVKLPVFYKQRGLL
FYPAWAYALPSWFLKIPITFVEVGVWVFITYYVIGFDPNVGRLFRQYLLLLLLNQVASSLFRFIAAASRNMIIAN
TFGTFALLLLFALGGFVLSRENIKKWWIWVYWSSPLMYAQNAIVVNEFLGKSWSKNASTTSTESLGVTVLKSR
GFFTEAHWCWIGAGALLGFIFVFNFFYTVALTYLNPFEKPQAVITEESDNAKTGGKIELSSHRKGSIDQTASTKR
GGEIGRSISSTFSYVTEEAIAEANHNKKKGMVLPFQPHSITFDDIRYSVDMPEEMKSQGVLEDKLELLKGVSGA
FRPGVLTALMGVSGAGKTTLMDVLAGRKTGGYIEGNISISGYPKKQETFARICGYCEQNDIHSPHVTIHESLLYS
AWLRLSPDVDAETRMMFIEEVMELVELTPLRDALVGLPGVNGLSTEQRKRLTIAVELVANPSIIFMDEPTSGLD
ARAAAIVMRTVRNTVDTGRTVVCTIHQPSIDIFEAFDELLLLKRGGQEIYVGPLGRHSSHLIKYFEGIEGVSKIKD
GYNPATWMLEVTTSAQELILGVDFTEIYKNSDLYRNNKDLLKELSQPTPGSKDLYFPTQYSQSFFTQCMACLW
KQRWSYWRNPPYTAVRFFFTTFIALMFGTMFWDLGTQRTRQQDLSNAMGSMYAAVIFLGFQNGQSVQPVVV
VERTVFYRERAAGMYSAMPYAFAQALVEIPYVFSQAVVYGAIVYAMIGFEWTTAKFFWYIFFTFFSLLYFTFF
GMMAVAATPNQHIAAIIAAAFYALWNLFSGFIIPRTRIPVWWRWYYWACPVAWTLYGLVTSQYGDIEDRLLD
TNVTVKQYLDDYFGFEHDFLGVVAAVIVGFTVLFLFIFAFSIKAFNFQRR

>XP_024460925.1_PDR1_Pt
MESADIYRASSSLRDSFRAGSSAWRNTTVEAFSRSSREEDDEEALKWAAIEKLPTYDRLRKGILTSASKGVANE
VDIEKLGLQERKQLLERLVKVAEEDNEKFLWKLKDRVERVGIDVPTIEVRYDNLNIEAEAYVGSSALPSFAKFT
FNIIEGLLISLNILRNRKKPLTILKDVSGIVKPSRLTLLLGPPSSGKTTLLLALAGKLDPNLKFSGRVTYNGHEMN
EFVPQRTAAYISQHDVHIGEMTVRETLAFSARCQGAGYLHDMLAELSRREKEANIKPDPDVDVFMKAVASQG
DEANVITDYVLKILGLEICADTMVGDEMIRGISGGQRKRVTTGEMLVGPSRALFMDEISTGLDSSTTYQIVNSL
RHTVHILNCTAVISLLQPAPETYDLFDDIILLSDGQIVYQGPRERVLEFFEHMGFKCPERKGVADFLQEVTSRKD
QEQYWARKDQPYRFITANEFAEAFQSFTVGRRTAEELSIPFDKSKNHPAALVTKTHGAGKKDLLKANFSREYL
LMKRNSFVYIFKICQLTIMALISMTLFFRTKMHRDTVRHGGIYTGALFFTAIMIMFNGMSELSMTIAKLPVFYKQ
RDLRFFPSWAYAFPQWILKIPVSFVEVAAWVFLTYYVIGFDPNVERLFKQYLVLLLINQTASALFRFIAAAGRN
MIIANTFGSFALLTLFTLGGFILSREKIKKWWIWGYWSSPLMYGQTAILVNEFLGNSWSHVPENSTEPLGIQVLK
SRGFFTEAYWYWIGAGATIGFILLLNLFFVLALTFLNAFDKPQAFISEEPESDESGRKTERAIQLSNHGSSHGTNT
EGGVGISRASSEVIGGVSNNRKKGMVLPFEPHSITFDDIIYSVDMPQEMKVQGVVQDRLVLLNGVNGAFRPGV
LTALMGVSGAGKTTLMDVLAGRKTGGYIDGEIKISGYPKKQETFARVSGYCEQNDIHSPQVTVYESLLYSAWL
RLPPEVDSETRRMFIEEVMDLVELNPLRHALIGLPGVNGLSTEQRKRLTIAVELVANPSIIFMDEPTSGLDARAA
AIVMRTVRNTVDTGRTVVCTIHQPSIDIFEAFDELFLMKRGGQEIYVGPLGRHSTHLIKYFEAIEGVSKIKDGYN
PATWMLEISSSAQEMALEVDFSNIYKNSDLFRRNKALIVELSTPAPGSTDLYFPTKYSTSFLTQCMACLWKQH
WSYWRNPPYTAVRFIFTTFIALMFGTMFWDLGSKVSSTQDLSNAMGSMYAAVLFLGVQNASSVQPVVAVERT
VFYRERAAGMYSALPYAFAQVLIELPYIFAQAAVYGIIVYAMIGFDWTVAKFFWYLFFMYFTLLYFTYYGMM
AVAVTPNHHIASIVSSAFYGIWNLFSGFIVPRPSIPIWWRWYSWACPVAWTLYGLVVSQFGDIQKKLTETQTVK
EYVKDYFGFHHDFLGVVAAAIVGWTVLFAFIFAFAIKAFNFQRR

>XP_002297807.2_PDR1_Pt
MESADIYRASSSLRGSFRGGSSAWRNTTVEAFSRSSREEDDEEALTWAAIEKLPTYDRLRKGILTSASKGVANE
VDIEKLGVQERKQLLERLVKVAEEDNEKFLWKLKDRVERVGIDVPTIEVRYDNLNIEAEAYVGSSALPSFAKFT
FNIIEGLLISLNILRNRKKPLTILKDVSGIVKPSRLTLLLGPPSSGKTTLLLALAGKLDPNLKFSGRVTYNGHEMN
EFVPQRTAAYISQHDLHIGEMTVRETLAFSARCQGAGYLHDMLAELSRREKEANIKPDPDVDVFMKAVASQG
EEANVITDYVLKILGLEVCADTMVGDEMIRGISGGQRKRVTTGEMLVGPSRALFMDEISTGLDSSTTYQIVNSL
RHTVHILNCTAVISLLQPAPETYDLFDDIILLSDGQIVYQGPRERVLEFFKHMGFECPERKGVADFLQEVTSRKD
QEQYWARKDQPCRFITANEFAEAFQSFSVGRRTAEELSIPFDKSKNHPAALVTKTHGAGKKDLLKANFSREYL
LMKRNSFVYIFKICQLTIMALISMTLFFRTEMHRDTVADGGIYTGALFFTAIMIMFNGMSELSMTIAKLPVFYKQ

RDLRFFPSWAYAIPQWILKIPVAFVEVGVWVFLTYYVIGFDPNVGRLFKQYLLLLLINQMASALFRFIAAAGRN
MIVANTFGSFALLTLFALGGFILSREKIKKWWIWGYWISPLMYGQTAIVVNEFLGNSWSHVPENSTEPLGIQVL
KSRGFFTEAYWYWIGAGATIGFILLLNLFFVLALTFLNAFDKPQAVISDEPESDESGRKTERAIQLSNHGSSHGT
NTEGGVGISRASSEAIGRVSNNRKKGMVLPFEPLSITFDDVIYSVDMPQEMKIQGVVEDRLVLLNGVNGAFRPG
VLTALMGVSGAGKTTLMDVLAGRKTGGYIDGEIKISGYPKKQETFARVSGYCEQNDIHSPQVTVYESLLYSAW
LRLPPEVDSETRRMFIEEVMDLVELNPLRHALVGLPGVNGLSTEQRKRLTIAVELVANPSIIFMDEPTSGLDARA
AAIVMRTVRNTVDTGRTVVCTIHQPSIDIFEAFDELFLMKRGGQEIYVGPLGRHSTHLIKYFEAIEGVSKIKDGY
NPATWMLEISSSAQEMALEVDFSNIYKNSDLFRRNKALIVELSTPAPGSTDLYFPTKYSTSFLTQCMACLWKQH
WSYWRNPPYTAVRFLFTTFIALMFGTMFWDLGSKVDSTQDLFNAMGSMYAAVIFLGVQNASSVQPVVAVER
TVFYRERAAGMYSALPYAFAQVLIELPYIFVQAAVYGIIVYAMIGFEWTVVKFFWYLFFMYFTLLYFTFYGMM
AVAMTPNHHIAAIVSSAFYGIWNLFSGFIIPRPSMPIWWRWYSWACPIAWTLYGLVVSQFGDIQKDLTETQTVK
EYVKDYFGFDHDFLGVVAAAIVGWTVLFAFIFAFAIKAFNFQRR

>XP_021628887.1_PDR1-l_Me
MEGDHYRASTSLRRGSSSAWRNNVLDVFSASSRDEDDEEALKWAALEKLPTYDRLRKGILVSVSKGGANELD
VDNLGFNERKTLLERLVKVAEEDNEKFLLKLKNRLDRVGIEVPTIEVRYEHLNIEAEALVGSNALPSFLNFTISI
AEGLLNYLHIFPSRKRPLTILNDVSGVIKPSRMTLLLGPPSSGKTTLLLALAGKLDPNLKVSGNVTYNGHTLNEF
IPQRTAAYISQHDLHIGEMTVRETLAFSARCQGVGTQHEMLAELSRREKAANIKPDPDLDFFMKAAATEGQET
SVVTDYILKILGLDICADTLVGNEMIRGISGGQRKRVTTGEMLVGPAKALFMDEISTGLDSSTTFSIVNSLRQSIH
ILNGTAVISLLQPAPETYNLFDDIILLSDGQIVYQGPREHVLEFFEYMGFKCPERKGVADFLQEVTSKKDQQQY
WARKDRLYRFITVREFAEAFQSYEVGRKIAGDLKTPFDRRKNHPAALATKHYGVGKMELLKANFSREYLLMK
RNSFVYIFKLSQLIMMATIGMTLFFRTEMKRDDLEDAGVYLGALFFTLITIMFNGMAELSMTIAKLPVFYKQRN
LLFFPAWSYSIPSWILKIPVTFLEVGVWVFLTYYVIGFDPNVGRLFKQYMLLLLVNQMASALFRFIASVGRNMI
VANTFGSFALLTLFALGGFVLSREDIKKWWIWGYWVSPLMYGQNAIVANEYLGHSWSHIPANSNSTDSLGVQ
FMKSRGFFPNAYWYWLGVAASAGYILLFNLAYTVALTFLDSFEKPQAVISDEPEESKSSERAIQLSKLESSHRT
NTESRTSGIDESNHNRKKGMVLPFEPRSITFDNVMYSVDMPQEMKNQGVLDDKLVLLKGVSGAFRPGVLTAL
MGVSGAGKTTLMDVLAGRKTGGYIEGDIRISGYPKKQETFARISGYCEQNDIHSPHVTVYESLVYSAWLRLPQ
EVDSETRRMFVEEVMELVELNPLRQALVGLPGVNGLSTEQRKRLTIAVELVANPSIIFMDEPTSGLDARAAAIV
MRTVRNTVDTGRTVVCTIHQPSIDIFEAFDELFLMKRGGEEIYVGPLGRHSCHLIKYFEGMEGVSKITDGYNPA
TWMLEVTSAAQELALGVDFAEIYRNSDLYRRNKTMIQELSKPAPGTQDLYFPTKYSQPFLTQCLACLWKQSW
SYWRNPPYTGVRFWFTTFIALMFGTIFWDLGSKKGKEGDLSNAMGSMYAAVLFLGVQNASSVQPVVAVERT
VFYREKAAGLYSAMPYAYAQALIELPYIFAQAGVYSVITYAMIGFEWTAAKFFWYLFFLYFTLLYFTFYGMMT
VAVTPNHHIASIVSSAFYAIWNLFSGFIIPRTKMPVWWRWYYWGCPISWTLYGLIASQFADIKTMLGDSGKTVE
EYVNDFYGIKHDFVGVAAGVIVGITVLFAFIFGISIKAFNFQRR

>XP_021627417.1_PDR1-l_Me
MEGADHYRASNSFKRNSFIWRNNSFQNSFVWRTDTCEAFSKSCREEDDEEALKWAALEKLPTYDRLRKGILVS
VSKGGANEIDVDALGFHDRQRLLERLVKVAEEDNEKFLLKLRRRIDRVGIELPKVEVRFENLNVEAEAFVGNR
ALPTFVNFCVNIIEGCLNSLHILPSRKKPLTILKDVSGVIKPSRMTLLLGPPSSGKTTLLLALAGKLDPDLKLSGS
VTYNGHGINEFIPQKTAAYISQHDLHIGEMTVRETLAFSARCQGVGWQHEMLAELSRREKASNIKPDPDIDVF
MKAAAIEGQESSVVTDYILKILGLEVCADTMVGDDMLRGISGGQRKRVTTGEMLVGPSKALFMDEISTGLDSS
TTFQIVNSLRQTVHILNGTAVISLLQPAPETYDLFDDIILLSDGQIVYQGPREDVLGFFEHMGFKCPERKGVADF
LQEVTSRKDQKQYWAHKDQPYSFVTVQEFVEAFQSYAVGRRIGQELSTPFDKSKSHPAALATGKYGVGKMEL
LKACLSREYLLMKRNSFVYFFKLFQLSLMGIIAMTLFLRTNMHRNDLNGGGIYLGALFFTVIMIMFNGMSELS
MTVAKLPVFYKQRDLLFYPSWAYSLPTWILKIPITFFEVAVWVLLTYYPIGFDPNVVRLLKQYILLLFVNQMAS
ALFRFIAAAGRNMIVANTFGSFALLIIFALGGVILSRDDIKKWWIWGYWISPMMYGQNAIVANEFLGKSWSHIP
PNSTESLGVQVLKSRGFFPQAHWFWLGVGALAGFIIVFNICFTLALTYLNPFDKPQAVISDEPEHSERTNTEDGT
SGINEAKHKKKGMVLPFEPHSITFDNVIYSVDMPQEMKNQGVLDDKLVLLKGVSGAFRPGVLTALMGVSGAG
KTTLMDVLAGRKTGGYIEGDIRISGYPKKQETFARISGYCEQNDIHSPHVTVYESLLYSAWLRLPKEVNSETRK
MFVEEVMELVELNPLRQALVGLPGVNGLSTEQRKRLTIAVELVANPSIIFMDEPTSGLDARAAAIVMRTVRNT
VDTGRTVVCTIHQPSIDIFEAFDELFLMKRGGEEIYVGPLGRQSGHLINYFEEIEGVEKIQDGYNPATWMLEVSS
SAQELSLGVDFAAIYKNSELYRRNKATIEELSRPAPGSKDLYFPTQYSQSFFTQCIACLWKQRLSYWRNPPYTA
VRFLFTTIIAMMFGTMFWGLGSKTSKEQDIFNSAGSMYAAVQFLGVQNAGSVQPVVAVERTVFYRERAAGMY
SPLPYAYAQVLVELPYIFVQAVVYGLMTYAMIGYEWTAVKFLWYIFFMYFTLLYFTYYGMMSVAATPNYHIA

SISSSAFYTIWNLFSGFIIPRTRMPVWWRWYYWLNPVSWTLYGLITSQFGDMKHILEGGQTVGEFVRDYYGIN
HHFIGVVAAVVLGFTLLFAFIFAISIRFFNFQRR

>XP_024453096.1_PDR1_X1_Pt
MESAVISRGSDSFRGSSRGVSSVWRNSTVEVFSRSSREEDDEEALKWAALEKLPTYDRLRKGILTSASRGIISEV
DIENLGVQERKQLLERLVKVADEDNEKFLWKLKNRVERVGIEFPTIEVRYENLNIEAEAYVGSSALPSFAKFIFH
IIEGFFIALHVLPSRKKPLTILKDVSGIIKPSRLTLLLGPPNSGKTTLLLAMAGKLDPSLKFSGHVTYNGHEMNEF
VPQRTAAYVSQHDLHIGEMTVRETLEFSARCQGVGHLHEMLAELSRREKEANIKPDQDVDVFMKAVATQGQ
EASVITDYVLKILGLEVCADTLVGDEMIRGISGGQRKRVTTGEMLVGPSRALLMDEISTGLDSSTTYQIVNSLK
QTIHVLNCTAVISLLQPAPETYDLFDDIILLSDGQIVYQGPRENVLGFFEHMGFKCPDRKGVADFLQEVTSKKD
QEQYWAIKDQPYRFVRVNEFSEAFQSFNVGRKIADELSIPFDKTKNHPAALVNKKYGAGKMDLLKANFSREY
LLMKRNSFVYIFKICQLTVVALISMSLFFRTKMHHDTVADGGIYTGALFFTVIIIMFNGMSELSMTIVKLPVFYK
QRELLFFPPWAYSIPPWILKIPVTFVEVAAWVLLTYYVIGFDPNVERLLRQYFLLLLINQMASALFRFIAAAGRN
MIVANTFGSFALLTLFALGGFILSREQIKKWWIWGYWLSPLMYGQNAIVVNEFLGHSWSHIPGTSTEPLGIQVL
KSREFFTEANWYWIGVGATVGFMLLFNICFALALTFLNAFEKPQAFIFEESEREGSVGKTGGAVQLSNHGSSHK
NKTENGDEINRNGFASIGEASDNRKRGMVLPFEPHSITFDDVIYSVDMPQEMKIQGVVEDRLVLLKGVNGAFR
PGVLTTLMGVSGAGKTTLMDVLAGRKTGGYIEGDIKISGYPKKQETFARIAGYCEQNDIHSPHVTVYESLLYSA
WLRLPPEVDSETRKMFIDEVMELVELDSLRNALVGLPGVNGLSTEQRKRLTIAVELVANPSIIFMDEPTSGLDA
RAAAIVMRTVRNTVDTGRTVVCTIHQPSIDIFDAFDELFLMKRGGEEIYVGPLGHHSTHLIKYFEAIEGVSKIKD
GYNPATWMLEVTASSQEMALEVDFANIYKNSDLFRRNKALIAELSTPAPGSKDVHFPTRYSTSFFTQCMACLW
KQHWSYWRNPPYTAVRFLFTTFIALMFGTMFWDLGSKVKTTQDLSNAMGSMYAAVLFLGFQNGTAVQPVV
AVERTVFYRERAAGMYSALPYAFAQALIELPYVFVQAAVYGVIVYAMIGFEWTAAKFFWYLFFMYFTLLYFT
FYGMMAVAVTPNHHIAGIVSTAFYAIWNLFSGFIIPRTRIPIWWRWYYWGCPVSWSLYGLVVSQYGDIQEPITA
TQTVEGYVKDYFGFDHDFLGVVAAVVLGWTVLFAFIFAFSIKAFNFQRR

>XP_002298123.2_PDR1_X1_Pt
MESGYLYRAGSSVRRGNSSGTFSNNAAADHQVFSLSSHGQDDDEEALKWAALEKLPTYDRLRKGILTTSTGA
ASEVEVQNLGFQERKNLVERLVNVAEEDNEKFLLKLKNRIDRVGIHVPTIEVRFEHLNVEAEAYVGSRALPTFF
NYSVNMLEGVLNYLHILSSRKKHMWILKDVSGIIKPSRMTLLLGPPSSGKTTLLLALAGKLDHALKFSGRVTY
NGHEMDEFVPQRTAAYISQHDLHIGEMTVRETLAFSARCQGVGSRYDMLAELSRREKEAGIKPDPDIDVFMKA
AATEGQEDSVVIDYILKVLGLEVCADTLVGDEMLRGISGGQKKRVTTGEMLVGPAKALFMDEISTGLDSSTTY
QIVNSIKQYVQILEGTALISLLQPAPETYDLFDDIILLSDGEIVYQGPREHVLRFFEYMGFKCPARKGVADFLQEV
TSRKDQMQYWARRDVPYRFVTVKEFAEAFYSFHEGKRLGNELAVPFDKSKNHPAALTTKKYGVNKRELCKA
SFSREFLLMKRNSFVYAFKFIQLTIVAVIAMTLFLRTEMHRDSVTDGGIYVGAMFFIVVVIMFNGMAEISMTLA
KLPVFYKQRDLLFFPAWIYALPTWILKIPITFIEVAIMVFITYFVIGFDPNVGRLFKHYLVLLLTNQMASGLFRTI
AAVGRNMVVANTFGSFVLLLLFVLGGFVLSRDDIKKWWIWGFWTSPMMYAQNAVVVNEFLGKSWNHVLPN
STEPLGIEVLKSRGFFTEAYWYWLAVAALFGFTLLYNFLYILALAFLNPLGKPQQAGISEEPQSNNVGRIGEAIH
LMNPGINSSLHTSAESIDEIGRSKSSRFTCNKQRGVIIPFEPHSITFDKVMYSVDMPQEMKSHGVHEDKLVLLKG
VSGAFRPGVLTALMGISGAGKTTMMDVLAGRKTGGYIEGNITISGYPKKQETFARISGYCEQNDIHSPHITVYE
SLLYSAWLRLPTEVDIETRKMFVEEVMELVELNPLRQALVGLPGVDGLSTEQRKRLTIAVELVANPSIIFMDEP
TSGLDARAAAIVMRTVRNTVDTGRTVVCTIHQPSIDIFEAFDELFLLKRGGQEIYVGPLGRLSCHLIKYFEGIEG
VNKIKDGYNPATWMLEVTSTAEELALGVDFAEIYRSSELFRRNRALIKDLSTPAPGSKDLYFSTQYSRSFFTQCL
ACLWKQHWSYWRNPPYTAIRFLSTTVIGLIFGTMFWDIGSKITKRQDLFNAMGSMYTAVLFLGVQNAASVQP
VVAVERTVFYRERAAGMYSALPYAFAQVLIELPYIFVQAAVYGVIVYSMIGFGWTISKFFWYLYFMYFTLLYF
TFYGMMAVAVSPNHQIASVISAAFYGIWNVFSGFVIPRSRMPLWWRWYSWICPVFWTLYGLVASQFGDMKD
RLETGETVEQFVTIYLDFKHDFLGVVAAVILGFTVLFAITFAISIKLFNFQRR

>XP_003546230.1_PDR1_Gm
MEGSDIYRASNSLRSRSSTVWRNSGVEAFSRSSREEDDEEALKWAALEKLPTYNRLRKGLLTASHGVANEIDV
SDLGIQERQKLLERLVKVAEEDNERFLLKLKERIDRVGLDIPTIEVRYEHLNIEAEAFVGSRALPSFINSVTNVVE
GFFNLLHVSTSKKKHVTILKDVSGIIKPRRMTLLLGPPSSGKTTLLLALSGKLDKTLKVSGRVTYNGHELNEFVP
QRTAAYISQHDLHIGEMTVRETLAFSARCQGVGSRYDMLSELSRREKAANIKPDPDLDVYMKATATEGQESNI
VTDYTLKILGLDICADTMVGDEMLRGISGGQRKRVTTGEMLVGPANALFMDEISTGLDSSTTFQIVSSLRHYVH
ILNGTAVISLLQPAPETYDLFDDIILISDGQVVYHGPREYVLDFFESMGFRCPERKGVADFLQEVTSKKDQAQY

WVRRDQPYRFVTVTQFAEAFQSFHIGGKLGEELTVPFDRTKSHPAALTTKKYGINKKELLKANFSREYLLMKR
NSFVYLFKLSQLFIMALVAMTLFLRTEMHHENMDDAGVYAGAVFFMLITVMFNGLAEISMTIAKLPVFYKQR
NLLFYPSWAYAIPSWILKIPVTIVEVAVWVFLTYYVIGFDPNVGRFFKQYLVLLIVSQMASGLFRTIAALGRNMI
VANTFGAFAIITVVALGGFILSKRDIKSWWIWGYWISPLMYGQNALMVNEFLSNSWHNATHNLGVEYLESRAF
FTDSYWYWLGLGALVGFVFLFNVMFGLALEFLGPFDKPQATITEDESSNEGTLADIELPGIESSGRGDSLVESSH
GKKKGMVLPFEPHSITFDEVVYSVDMPQEMKEQGVQEDRLVLLKGVSGAFRPGVLTALMGVSGAGKTTLMD
VLAGRKTGGYIDGSIKISGYPKKQETFARISGYCEQNDIHSPHVTVYESLLYSAWLRLPSSVDSKTRKMFIEEVM
ELVELNPVRNSLVGLPGVSGLSTEQRKRLTIAVELVANPSIIFMDEPTSGLDARAAAIVMRTVRNTVDTGRTVV
CTIHQPSIDIFEAFDELFLMKRGGQEIYVGPLGRHSSHLIKYFESIEGVSKIKDGYNPATWMLEVTATAQELSLG
VDFTDLYKNSDLYRRNKQLIQELGQPAPGSKDLHFPTQYSQSFLVQCQACLWKQRWSYWRNPPYTAVRFFFT
TFIALMFGTIFWDLGGKHSTRGDLLNAIGSMYTAVLFLGVQNASSVQPVVAIERTVFYREKAAGMYSALPYAF
AQILVELPYVFVQAVTYGVIVYAMIGFEWTAEKFFWYLFFMYFTLLYYTFYGMMTVGLTPNHHIASIVAAAFY
AVWNLFSGFVVTRPSIPVWWRWYYWACPVAWTIYGLVASQFGDLTEPMTSEGQKIVKDFLEDYYGIKHDFIG
VSAVVVAGIAVLFALIFAVSIKTFNFQKR

>XP_024460929.1_PDR1_X1_Pt
MESADIYRASSSLRDSLRAGSFVWRNSTVEAFSRSSREEDDEEALKWAALEKLPTYDRLRKGILMSASRGVSSE
VDIEKLGVQERKQLLERLVKAADEDNEKFLWKLKNRIERVGIEFPTIEVRYEHLNIGAEAYVGSGALPSFAKFT
FSIIEDLLIALRIIPSRKKPLTILKDVSGIIKPSRLTLLLGPPSSGKTTLLLALAGKLDPSLKYSGRVTYNGHGMNEF
VPQRTASYTSQQDLHIGEMTVRETLAFSARCQGVGNLHDMLAELSRREKEANIKPDPDIDVFLKAVATQGQEA
NVITDYVLKILGLEVCADTLVGDEMIRGISGGQRKRVTTGEMLVGPSRALFMDEISTGLDSSTTHQIVNSLKQTI
HILNYTAVISLLQPAPETYDLFDDIILLSDGQIVYQGPRENVLGFFEHLGFQCPERKGVADFLQEVTSRKDQEKY
WARKDQPYRFVTVNEFAEAFQSLSVGRRVIEELSIPFDKTKNHPAALVNKKYGAGKMDLLKANFSREFLLMK
RNSFVYIFKIFQLTMMAIISMTLFFRTKMPRDTVEDGGIYAGALFFTAIMIMFNGMAELSMTIAKLPVFYKQREL
LFFPPWTYSIPPWILKIPITFVEVAAWVFLTYYVIGFDPNIGRFFKLYAVLVLINQMASALFRFIAAAGRNMIVAN
TFGSFVLLAVFALGGVILSREQIKKWWIWGYWASPLMYGQNAIVVNEFLGNSWSHIPAGSTESLGIQVLKSREF
FTEAYWYWIGIGATAGFILLFNVCFVVALTVLDAYEKPQAVISEEPESGDSEGAVQLSNRGISHQTNTDSIGEAS
NNRKKGMVLPFEPHSITFDDVIYSVDMPQEMKVQGVAEDRLALLKGVSGAFRPGILTALMGVSGAGKTTLMD
VLAGRKTGGYIEGDIKISGYPKKQETFARISGYCEQNDIHSPQVTVYESLLYSAWLRLPSEVDSETRKMFIEEVM
DLVELNPLRSALVGLPGVNGLSTEQRKRLTIAVELVANPSIIFMDEPTSGLDARAAAIVMRTVRNTVDTGRTVV
CTIHQPSIDIFDAFDELFLMKRGGEEIYVGPVGRHSTHLIKYFEEIEGVSKIKDGYNPATWMLEVTSSSQEMALG
VDFANIYKNSNLLRRNKALIAELSTPAPGSKDIYFSTQYSTSFFTQCMACLWKQHWSYWRNPPYTAVRFLFTTF
IGLMFGTMFWDLGSKVGTAQDLSNAMGSMYAAVLFLGFQNGSAVQPVVAVERTVFYRERAAGMYSALPYA
FAQVLIEIPYVFVQSAVYGVIVYAMIGFEWTAAKFFWYLFFMYFTLLYFTFYGMMSVAVTPNHHIAAIVSTAF
YLIWNLFSGYIVPRPRIPIWWRWYYWACPVSWSLYGLVVSQYGDIQKNLTETETVKQYVKNYFGFDHDFVGV
VAAAVLGWTVLFAFIFAFSIRAFNFQRR

>XP_021610343.1_PDR1-l_Me
MEGDHYRASNSLRRGSSSVWRNNGLDVFSRSSREEDDEEALKWAALEKLPTYDRLRKGILVSVSKGGANEID
VDNLGFQERKTLLERLVKVAEEDNEKFLLKLKNRIDRVGIEVPTIEVRYEHLNVEAEALVGSNALPSFLNFSFSL
VEGLFRYILPNRKRPLTILKDVSGVIKPSRMTLLLGPPSSGKTSLLLALAGKLDPSLKVSGTVTYNGHSLNEFIPQ
RTAAYISQHDLHIGEMTVRETLAFSARCQGVGTQHEMLAELSRREKAANIKPDPDLDVFMKAAATEGQETSV
VTDYVLKILGLDICADTLVGNEMIRGISGGQRKRVTTGEMLVGPAKALFMDEISTGLDSSTTFSIVNSLKQSIHIL
NGTAVISLLQPAPETYNLFDDIILISDGQIVYQGPRENVLEFFEYMGFKCPERKGVADFLQEVTSKKDQQQYWA
RKDQPYRFVTVNEFAEAFQSYEVGRKIAEDLSVPFDRTKNHPAALTTKPYGVGKMELIKANFSREYLLMKRNS
FVYVFKLTQLIVMAIIGMTLFFRTEMKHDSFEDAGVYAGALFFTLITIMFNGMAELSMTIAKLPVFYKQRNLLF
FPAWSYAIPSWILKIPVTFLEVGVWVFITYYVIGFDPNVGRLFKQYMLLLLVNQMASGLFRFIASVGRNMIVAN
TFGSFALLTLFALGGFVLKRSDIKKWWIWGYWVSPLMYGQNAIVANEFLGNSWNHIPANSTSTDSLGVQFLKT
NGFFPHAYWYWLGVGASAGYILVFNLLYTVALTFLDQFEKPQAVISDEPEESNRSGGAIQLSQAESSHRTQTES
GTSGIDESNHNKKKGMVLPFEPHSITFDNVIYSVDMPQEMKSQGVVEDKLVLLKGVSGAFRPGVLTALMGVS
GAGKTTLMDVLAGRKTGGYIEGDIRISGYPKKQETFARISGYCEQNDIHSPYVTVYESLVYSAWLRLPPDVDSE
TRRMFVEEVMELVELNPLRQALVGLPGVNGLSTEQRKRLTIAVELVANPSIIFMDEPTSGLDARAAAIVMRTVR
NTVDTGRTVVCTIHQPSIDIFEAFDELFLMKRGGEEIYVGPLGRHSCHLIKYFEGIGGVSKITDGYNPATWMLEV
SSSAQELTLGVDFANVYRNSDLYRRNKAMIQELSKPAPGTEDLYFPTQYSQPFLTQCMACLWKQSWSYWRNP

PYTGVRFWFTTFIALMFGTIFWDLGSKMEKEGDLTNAMGSMYAAVLFLGVQNSSSVQPVVAVERTVFYREKA
AGMYSAMPYAYAQALIELPYIFAQAGVYSLITYAMIGFEWTAAKFFWYLFFLYFTLLYFTFYGMMTVAVTPN
HHIASIVSSAFYSIWNLFSGFIVPRPKMPVWWRWYYWGCPISWTLYGLFASQFADITKPLGTTGKTVEEYVNET
YGIKHDFLGASAGVIFGIALLFAVIFAVSIKAFNFQRR

>XP_010654719.1_P_PDR1_Vv
METAEIYTASGRRASGSFKKNSSSIWRNSGAEVFSRSSRDEDDEEALKWAALEKLPTYNRLRKGLLIGSEGEAS
EVDIHNLGPQERKNLVERLVKIAEEDNEKFLLKLKNRMDRVGIDLPEIEVRFEHLTIDAEAHVGSRALPSFINSV
FNQIEDILNTLRILPSRKKKFTILHDVSGIIKPGRMTLLLGPPSSGKTTLLLALSGKLDSSLKVTGRVTYNGHGMN
EFVPQRTAAYISQLDTHIGEMTVRETLAFSARCQGVGDRYDMLVELSRREKAANIKPDPDIDVFMKAAAAEGQ
KENVITDYTLKILGLEICADTMVGDEMVRGISGGQRKRVTTGEMLVGPSKALFMDEISTGLDSSTTYQIVNSLR
QTVHILNGTALISLLQPAPETYDLFDDIILLSDSRIIYQGPREDVLNFFESMGFRCPERKGVADFLQEVSANRRSX
QYWAHKDXXXXXXXXXXXAEAFQSFHFGRKLGDELATPFDKTKSHPAALKTEKYGVGKKELLDACISREYL
LMKRNSFVYIFKLTQLTIVAMIAMTIFLRTEMPKNTTEDGIIYTGALFFTVMKVMFNGMSELAMTILKLPVFYK
QRGLLFYPAWAYALPSWFLKIPITFVEVGLWVFITYYVIGFDPNVGRLFRQYLLLLLLNQTASSLFRFIAAACRS
MIVANTFGSFALVLPFALGGFVLSRESVKKWWIWGYWSSPMMYAQNAIVVNEFLGKSWSKNASTNSTESLGV
AVLKARGFFTEAHWYWIGAGALLGFIFVFNFCYTVALTYLNPFEKPRAVITVESDNAKTEGKIELSSHRKGSID
QTASTESGEEIGRSISSVSSSVRAEAIAEARRNNKKGMVLPFQPLSITFDDIRYSVDMPEEMKSQGVPEDRLELL
KGVSGAFRPGVLTALMGVSGAGKSTLMDVLAGRKTGGYIEGSISISGYPKKQETFARISGYCEQNDIHSPHVTV
HESLLYSAWLRLPPNVDAETRKMFIEEVMDLVELTPLRGALVGLPGVNGLSIEQRKRLTIAVELVANPSIIFMDE
PTSGLDARAAAIVMRTVRNTVDTGRTVVCTIHQPSIDIFDAFDELLLLKRGGQEIYMGPLGRHSSHLIKYFEGIE
GVSKIKDGYNPATWMLEVTASAQELILGVDFTEIYEKSDIYRRNKDLIKELSQPTPGSKDLYFPTQYSQSFFTQC
MACLWKQRLSYWRNPPYTAVRFFFTTFVALMFGTMFWDLGTKRTRQQDISNAMGSMYAAVLFLGFQNGQS
VQPVVAVERTVFYRERAAGMYSAMPYAFAQALVEIPYVFSQAVAYGVIVYAMIGFEWTAAKFFWYLFFMFFT
LLYFTFYGMMAVAATPNQHIAAIVALAFYTLWNLFSGFIVPRNRIPVWWRWYYWACPVAWSLYGLVTSQFG
DIEDTLLDSNVTVKQYLDDYLGFKHDFLGVVAVVIVGFTVLFLFIFAFAIKAFNFQRR

>NP_001288053.1_PDR-l_Vv
MATAEIYRAAGSLRRNGSMWRSSGADVFSRSSRDEDDEEALKWAALEKLPTYNRLRKGLLMGSQGAASEVD
VDNLGFQEKQSLMERLVKIAEEDNEKFLLRLRNRIERVGITIPEIEVRFEHLTIDAEAFIGSRALPSFHNFMFNKIE
DALTGLRILRSRRRKFTILHDVSGIIKPQRMTLLLGPPSSGKTTLLLALSGKLDPTLKVTGRVTYNGHGMDEFVP
QRTAAYISQHDTHIGEMTVRETLAFSARCQGVGDRYDMLAELSRREKAANIKPDPDLDVFMKAAATEGQKEN
VVTDYTLKILGLDICADTMVGDEMIRGISGGQRKRVTTGEMLVGPSKALFMDEISTGLDSSTTFQIVNCLKQTI
HILNGTAVISLLQPAPETYNLFDDIILLSDGRIIYQGPREDVLEFFESTGFRCPERKGVADFLQEVTSKKDQQQY
WARKEEPYRFVTVKEFAEAFQSFHTGRKVGDELASPYDKTKSHPAALTTKKYGVNKKELLDANMSREYLLM
KRNSFVYVFKLTQLAIMAVITMTLFLRTEMHKNSVDDGNIYTGALFFTVVMIMFNGMAELAMAIAKLPVFYK
QRDLLFYPAWAYALPTWILKIPITFIEVGVWVFMTYYVIGFDPNVERLFRQYLLLLLVNQMASGLFRLIASAGR
NMIVSNTFGAFVLLMLLALGGFILSHDDVKKWWIWGYWCSPLMYAQNAIVVNEFLGHSWKKNVTGSTESLG
VTVLNNRGFFTEAYWYWIGAGALFGFILLFNFGYTLCLNFLNPFDKPQAVIVEESDNAETGGQIELSQRNSSIDQ
AASTERGEEIGRSISSTSSAVREEAVAGANHNKKKGMVLPFQPYSITFDDIRYSVDMPEEMKSQGVVEDKLELL
KGVSGAFRPGVLTALMGVSGAGKTTLMDVLAGRKTGGYIEGNITISGYPKKQETFARISGYCEQNDIHSPHVT
VYESLLYSAWLRLPSDVKSETRQMFIEEVMELVELTPLRDALVGLPGVSGLSTEQRKRLTIAVELVANPSIIFMD
EPTSGLDARAAAIVMRTVRNTVDTGRTVVCTIHQPSIDIFEAFDELLLLKRGGQEIYVGPLGRYSCHLINYFEGIE
GVSKIKDGYNPATWMLEATTAAQEATLGVDFTEIYKNSDLYRRNKDLIKELSQPPPGTKDLYFRTQFSQPFFTQ
FLACLWKQRWSYWRNPPYTAVRFLFTTFIALMFGTMFWDLGTKWSTQQDLFNAMGSMYAAVLFLGIQNSQS
VQPVVVVERTVFYRERAAGMYSPLSYAFAQALVEIPYIFSQAVVYGLIVYAMIGFQWTAAKFFWYLFFMFFTL
MYFTFYGMMAVAATPNQNIASIVAAAFYGLWNLFSGFIVPRNRIPVWWRWYYWICPVSWTLYGLVTSQFGDI
TEELNTGVTVKDYLNDYFGFKHDFLGVVAAVVVGFVVLFLFIFAYAIKALNFQRR

>NP_001237697.2_PDR-l _Gm
MEGGGSSFRIGSSSIWRNSDAAEIFSNSFHQENDEEALKWAAIQKLPTVARLRKALITSPDGESNEIDVKKLGLQ
EKKALLERLVKTAQEDNEKFLLKLKDRIDRVGIDLPTIEVRFENLSIEAEARAGTRALPTFTNFIVNILEGLLNSL
HVLPNRKQHLNILEDVSGIIKPGRMTLLLGPPSSGKTTLLLALAGKLDPKLKFSGKVTYNGHGMNEFVPQRTA
AYVNQNDLHVAELTVRETLAFSARVQGVGPRYDLLAELSRREKEANIKPDPDIDAYMKAVASEGQKANMITD

YILRILGLEVCADTVVGNAMLRGISGGQRKRVTTGEMLVGPAKALFMDEISTGLDSSTTFQIVNSLKQYVHILK
GTTVISLLQPAPETYNLFDDIILLSDSHIVYQGPREHVLEFFELMGFKCPQRKGVADFLQEVTSRKDQEQYWAH
KDQPYRFVTAKEFSEAHKSFHIGRSLGEELATEFDKSKSHPAALTTKMYGVGKWELLKACLSREYLLMKRNSF
VYTFKLCQLAVLAIIAMTIFLRTEMHRDSVTHGGIYVGALFYGVVVIMFNGLAELSMVVSRLPVFYKQRDYLF
FPSWVYALPAWILKIPLTFVEVGVWVFLTYYAIGFDPYVGRLFRQYLVLVLVNQMASALFRLVAAVGREMTV
ALTLGSFTLAILFAMSGFVLSKENIKKWWLWGFWISPMMYGQNAMVNNEFLGKRWRHFLPNSTEALGVEILK
SRGFFTQSYWYWIGVGALIGYTLLFNFGYILALTYLNPLGKHQAVISEEPQINDQSGDSKKGTNVLKNIQRSFSQ
HSNRVRNGKSLSGSTSPETNHNRTRGMILPSEPHSITFDDVTYSVDMPVEMRNRGVVEDKLALLKGVSGAFRP
GVLTALMGVTGAGKTTLMDVLAGRKTGGYIGGNITISGYPKKQETFARISGYCEQNDIHSPHVTVYESLLYSA
WLRLSPEINADTRKMFIEEVMELVELKALRNALVGLPGINGLSTEQRKRLTIAVELVANPSIIFMDEPTSGLDAR
AAAIVMRTVRNTVDTGRTVVCTIHQPSIDIFESFDELLLMKQGGQEIYVGPLGHHSSHLINYFEGIQGVNKIKDG
YNPATWMLEVSTSAKEMELGIDFAEVYKNSELYRRNKALIKELSTPAPGSKDLYFPSQYSTSFLTQCMACLWK
QHWSYWRNPLYTAIRFLYSTAVAAVLGSMFWDLGSKIDKQQDLFNAMGSMYAAVLLIGIKNANAVQPVVAV
ERTVFYREKAAGMYSALPYAFAQVLIELPYVLVQAVVYGIIIYAMIGFEWTVTKVFWYLFFMYFTFLTFTYYG
MMSVAVTPNQHISSIVSSAFYAVWNLFSGFIVPRPRIPVWWRWYSWANPVAWSLYGLVASQYGDIKQSMESS
DGRTTVEGFVRSYFGFKHDFLGVVAAVIVAFPVVFALVFAISVKMFNFQRR

>XP_004247841.1_PDR1_Sl
MEPANLGNLRGSSLRGSISGSRRGSVSLRANSNSIWRNTGVEIFSRSARDEDDEEALKWAALEKLPTFDRLRKG
LLFGSQGAAAEIDIDDIGLQERKNLLERLVRVADEDNEKFLLKLKNRIDRVGIDLPTIEVRYENLNIEADAYVGS
RGLPTFINFMTNFLETLLNTLHILPSSKRQITILKDISGIIKPCRMTLLLGPPSSGKTTLLLALAGKLDSSLKVTGK
VSYNGHELHEFVPQRTAAYISQHDLHIGEMTVRETLEFSARCQGVGSRYEMLAELSRREKAANIKPDPDIDIYM
KASATEGQEANVVTDYVLKILGLDICADTMVGDEMLRGISGGQKKRVTTGEMLVGPSKALFMDEISTGLDSST
TYSIVNSLRQSVQILKGTAVISLLQPAPETYNLFDDIILISDGYIVYQGPRDDVLQFFESMGFKCPERKGVADFLQ
EVTSKKDQPQYWSRRNEHYRFISSKEFSDAYQSFHVGRKLGDELAIPFDRTKCHPAALTNEKYGIGKKELLKV
CTEREYLLMKRNSFVYVFKFFQLTIMALMTMTLFFRTEMPRDTVDDGGIYAGALFFVVVMIMFNGMSEMAM
TIFKLPVFYKQRDLLFFPSWAYAIPSWILKIPVTLVEVGLWVILTYYVIGFDPNITRFLKQFMLLVLVNQMASGL
FRFMGAVGRTMGVASTFGAFALLLQFALCGFVLSREDVKGWWIWGYWISPLMYSVNSILVNEFDGSKWKHIA
PNGTEPLGVAVVKSRGFFPDAYWYWIGFAALFGFTVVFNFFYSLSLAYLKPYGKSQTVRPEDSGNAENGQAAS
QMTSTDGGDIVSAGQSKKKGMVLPFEPHSITFDDVVYSVDMPQEMKEQGAGEDRLVLLKGVSGAFRPGVLTA
LMGVSGAGKTTLMDVLAGRKTGGYIDGDIKISGYPKKQETFARISGYCEQNDIHSPYVTVYESLVYSAWLRLP
KDVDEKTRKMFVDEVMELVELEPLRSALVGLPGVNGLSTEQRKRLTIAVELVANPSIIFMDEPTSGLDARAAAI
VMRTVRNTVDTGRTVVCTIHQPSIDIFEAFDELFLMKRGGQEIYVGPLGRHSCHLIKYFESIPGVAKIKEGYNPA
TWMLEVTASAQEMMLGVDFTDLYKNSDLYRRNKALITELSVPRPGSKDLYFETQYSQSIWIQCMACLWKQN
WSYWRNPAYTAVRFIFTMFIALVFGTMFWDIGTKVSQSQDLFNAMGSMYAAVLFLGVQNASSVQPVVDVER
TVFYRERAAGMYSAIPYAFGQVFIEIPYVFVQAIVYGIIVYAMIGFEWEAGKFFWYLFIMFTTLLYFTFYGMMS
VAVTPNQNVASIVAAFFYAIWNLFSGFIVPRPRMPIWWRWYYWCCPVAWTLYGLVASQFGDIQSRLTDEETV
EQFLRRYFGFRHDFLPVVAGVLVAYVVVFAFTFAFAIKAFNFQRR

>XP_021610859.1_PDR1-l_Me
MEGPEVYVGGGSFRRGDSSIWRSNAMDTFSKSSREEDDEEALKWAALERLPTYDRLKKGILTTSKGEASEIYV
QDLGFQERRTLVDRLVNVAEEDNERFLLNLKNRIERVGIELPTIEVRFEHLNIETEAHVGNRALPTFINFSIDMVE
GFLNKLHILPSRKKRLSILQDISGIIKPRRMTLLLGPPSSGKTTLLLALAGKLDPKLKFSGRVTFNGHEMNEFVPQ
RTAAYISQYDKHIGEMTVRETLAFSARCQGVGHRYEMLTELLRREKASNIKPDSDLDVFMKAIATEGQETSVIT
DYILKVLGLEVCADIMVGNEMLRGVSGGQRKRVTTGEMLVGPAKALFMDEISTGLDSSTTFQIVNSIKQYIKIL
NGTAVISLLQPAPETYDLFDDIILLSDGWIVYQGPREHVLEFFEFMGFKCPERKGVADFLQEVTSRKDQQQYWT
RKDDPYCFVTVQQFSEAFQSFHVGRNLQAELSTPFDKTKSHPAALTTKKYGVGKMELLKACFSRELLLMKRN
SFVYIFKLSQLTIMAIIAMTLFLRTEMHRESVIDGGIYVGALFYSVVFIMFNGLSEISMTISKLPVFYKQRNLLFYP
AWAFSLPPWIIKIPITLVQVALWVFITYYVIGYDPYVGRLFRQYLLLVLVSQMASALFRFIAAAGRDMIVANTF
GSFALLTLFALGGFILSRDNIKKWWIWGYWISPLMYGQNAIVVNEFLGKSWSRVLPNSSEPLGVEVLKSRGFFT
NAYWYWIGVGALVGFTLLYNLCFTLALTFLGPLQKPQAVISEDSPSDESGSDHQTSAKSGSGSGSSSARAEVRV
NSSHQNKKGMVLPFEPHFITFDEIRYSVDMPQEMKNQGVTEDKLELLRGVSGSFRPGVLTALMGVSGAGKTTL
MDVLAGRKTGGYIEGNITISGYPKKQETFARISGYCEQNDIHSPHVTVYESLLYSAWLRLSPDVSSETRKMFIEQ
VMHLVELEPLRQALVGLPGVSGLSTEQRKRLTIAVELVANPSIIFMDEPTSGLDARAAAIVMRTVRNTVDTGRT

VVCTIHQPSIDIFEAFDELFLMKRGGQEIYVGPLGRHSCHLIKYFEGIEGVPNIKDAYNPATWMLEVTSSAQESV
LGVDFAAVYRNSELYRSNKEMIEKLSTPAPDSKDLYFPSKYSQSFFTQCMACLWKQRWSYWRNPPYTAVRLL
FTTVIALMFGTMFWNLGSKTKKRQDLFNAMGSMYAAIVFLGVQNASSVQPVVAVERSVFYRERAAGMYSPL
PYAFAQVLIELPYIFIQSVVYGLIVYAMIGFEWNAAKFFWYLFFMYFTLLYYTFYGMMSVAATPNQHVGAIVS
SAFYSLWNLFSGFIIPRPRIPVWWRWYAWACPVAYTLYGLISSQFGDLKHTLESGETVEDFVRSYFGFRHELLG
AVAAAVVGFATLFAFIFAICIKFFNYQRR

>XP_006360347.1_P_PDR1_St
MEPANLGNLRGSSLRGSISGSRRGSVSLRANSNSIWRNTGVEIFSRSARDEDDEEALKWAALEKLPTFDRLRKG
LLFGSQGAAAEIDINDIGYQERKNLLERLVRVAEEDNEKFLLKLKNRIDRVGIDLPTIEVRYENLNIEADAYVGS
RGLPTVINFMTNFIETLLNTLHILPSSKRQITILKDISGIIKPCRMTLLLGPPSSGKTTLLLALAGKLDSSLKVTGKV
SYNGHELHEFVPQRTAAYISQHDLHIGEMTVRETLEFSARCQGVGSRYEMLAELSRREKAANIKPDPDIDIYMK
ASATEGQEANVVTDYVLKILGLDICADTMVGDEMLRGISGGQKKRVTTGEMLVGPSKALFMDEISTGLDSSTT
YSIVNSLRQSVQILKGTAVISLLQPAPETYNLFDDIILISDGYIVYQGPRDDVLEFFESMGFKCPERKGVADFLQE
VTSKKDQPQYWSRRNEHYRFISSKEFSDAFQSFHVGRKLGDELAIPFDRTKCHPAALTNEKYGIGKKELLKVCT
EREYLLMKRNSFVYVFKFVQLTIMALMTMTLFFRTEMPRDTVDDGGIYAGALFFVVVMIMFNGMSEMAMTIF
KLPVFYKQRDLLFFPSWAYAIPSWILKIPVTLVEVGLWVILTYYVIGFDPNITRFLKQFLLLVLVNQMASGLFRF
MGAVGRTMGVASTFGAFALLLQFALCGFVLSREDVKGWWIWGYWISPLMYSVNSILVNEFDGNKWKHIAPN
GTEPLGVAVVKSRGFFADAYWYWIGFAALFGFTIVFNFFYSLALAYLKPYGKSQTVRPEDSENAENGQAASQ
MASTDGGDIVSAGQSKKKGMVLPFEPHSITFDDVVYSVDMPQEMKEQGAGEDRLVLLKGVSGAFRPGVLTAL
MGVSGAGKTTLMDVLAGRKTGGYIDGDIKISGYPKKQETFARISGYCEQNDIHSPYVTVYESLVYSAWLRLPK
DVDEKIRKMFVDEVMELVELEPLRSALVGLPGVNGLSTEQRKRLTIAVELVANPSIIFMDEPTSGLDARAAAIV
MRTVRNTVDTGRTVVCTIHQPSIDIFEAFDELFLMKRGGQEIYVGPLGRHSCHLIKYFESIPGVAKIKEGYNPAT
WMLEVTASAQEMMLGVDFTDLYKNSDLYRRNKALITELSVPRPGSKDLYFETQYSQSLWIQCMACLWKQNW
SYWRNPAYTAVRFIFTMFIALVFGTMFWDIGTKVSQSQDLFNAMGSMYAAVLFLGVQNASSVQPVVDVERTV
FYRERAAGMYSAIPYAFGQVFIEIPYVFVQAIVYGIIVYAMIGFEWETGKVFWYLFIMYTTLLYFTFYGMMSVA
VTPNQNVASIVAAFFYAIWNLFSGFIVPRPRMPIWWRWYYWCCPVAWTLYGLVASQFGDIQTKLVDEETVEQ
FLRRYFGFRHDFLPVVAGVLVAYVVVFAFTFAFAIKAFNFQRR

>XP_010654721.1_P_PDR1_Vv
MATADIYRASGSLRRNGSSIWRSSGADIFSRSSRDEDDEEALKWAALEKLPTYNRLRRGLLMGSEGEASEIDIH
NLGFQEKKNLVERLVKVAEEDNEKFLLKLKNRIDRVGIDVPEIEVRFEHLTIDAEAFVGSRALPSFHNFIFSKLE
GILNAVRILPSKKRKFTILNDVSGTIKPRRLTLLLGPPSSGKTTLLLALAGKLDPNLKVMGRVTYNGHGMNEFV
PQRTAAYISQHDTHIGEMTVRETLAFSARCQGVGDRYDMLAELSRREKAANIKPDPDLDVFMKAAATEGQKE
NVVTDYTLKILGLDICADTMVGDEMIRGISGGQRKRVTTGEMLVGPSKALFMDEISTGLDSSTTYQIINSLKQTI
HILNGTAVISLLQPAPETYNLFDDIILLSDSQIVYQGPREDVVEFFESMGFKCPARKGVADFLQEVTSRKDQAQY
WARKDVPYSFVTVKEFAEAFQSFHIGRKVADELASPFDRAKSHPAALTTKKYGVRKKELLDANMSREYLLMK
RNSFVYIFKLTQLAVMAVIAMTLFLRTEMHKNSTDDGNIYTGALFFTVVMIMFNGMAELAMAIAKLPVFYKQ
RDLLFYPAWAYALPTWVLRIPITFVEVGVWVFITYYVIGFDPNVERLFRQYLLLLLVNQMASGLFRFIAAAGRN
MIVANTFGAFALLMLLALGGFILSYDNVKKWWIWGYWSSPLMYAQNAIVVNEFLGKSWSKNVTDSTESLGV
TVLKSRGFFTDAHWYWIGAGALLGFIFVFNIFYTLCLNYLNLFEKPQAVITEESDNAKTATTERGEQMVEAIAE
ANHNKKKGMVLPFQPHSITFDDIRYSVDMPEEMKSQGALEDRLELLKGVSGAFRPGVLTALMGVSGAGKTTL
MDVLAGRKTGGYIEGNITISGYPKKQETFARISGYCEQNDIHSPHVTVHESLLYSAWLRLPSDVNSETRKMFIEE
VMELVELTPLRDALVGLPGVNGLSTEQRKRLTIAVELVANPSIIFMDEPTSGLDARAAAIVMRTVRNTVDTGRT
VVCTIHQPSIDIFEAFDELLLMKRGGQEIYVGPLGRHSSHLINYFEGIEGVSKIKDGYNPATWMLEVTTGAQEGT
LGVDFTEIYKNSDLYRRNKDLIKELSQPAPGTKDLYFATQYSQPFFTQFLACLWKQRWSYWRNPPYTAVRFLF
TTFIALMFGTMFWDLGTERTRQQDLLNAMGSMYAAVLFLGVQNAQSVQPVVVVERTVFYRERAAGMYSALP
YAFGQALVEIPYVFAQAVVYGVIVYAMIGFEWTAAKFFWYLFFMFFTLLYFTFYGMMAVAATPNQHIASIVA
AAFYGLWNLFSGFIVPRNRIPVWWRWYYWICPVAWTLYGLVTSQFGDIQDTLLDKNQTVEQFLDDYFGFKHD
FLGVVAAVVVGFVVLFLFIFAYAIKAFNFQRR

>XP_021627707.1_PDR1-l_Me
MESTEVFMGGGSFRRGDSSIWRSNAMDSFSRSSREEDDEEALKWAALERLPTYDRLKKGILTTSKGEAREIDV
ENLGFQERRSLVDRLVKVAEEDNEKFLLKLRNRIDRVGIELPTIEVRFEHLKIETEAHVGSRALPTFINFSVDIVE

GFLNNLHILPSRKKRLTILQDISGIVKPRRMTLLLGPPSSGKTTLLLALAGKLDPKLKFSGRVIYNGHEMNEFVP
QRTAAYISQYDTHIGEMTVRETLAFSARCQGVGHRYEMLTELLRREKESKIKPDSDIDVFMKAIATQGQETSVI
TDYVLKILRLEVCADIRVGNEMLRGVSGGQRKRVTTGEMLVGPAKALFMDEISTGLDSSTTFQIVNSIKQYIQIL
NGTAVISLLQPAPETYDLFDDIILLSDGQIVYQGPREHVLEFFEFMGFKCPERKGVADFLQEVTSRKDQQQYWA
RKDEPYNFVRIKEFAEAFQSFHVGRHLQNDLSTPFDKAKSHPAALTTKRYGVGKMELLKACFSREILLMKRNS
FVYTFKLTQLAIMALVAMTLFLRTEMHRDSVTDGGVYVGSLFFSVAFNMFNGLSDISMTIAKLPVFYKQKNLL
FYPAWAYSIPPWITRIPLTLAQVSIWVFLTYYVIGYDPNVGRLYRQYLLLVLVSQMASALFRFIAAAGRTMIVA
NTFGSFALLVLFALGGFILSPDDIKKWWTWGYWISPLMYGQNAIVVNEFTSKSWSQVLPNSTEPLGIQVLKSRG
FFTNAYWYWIGVGALVGFTLLFNLCFTLALTFLGPLRKPQAVISEESQCNEPGIGQIKFIRHSESGRGSSTLWAE
AIDNDKHQNKRGMVLPFEPHSITFDEIRYSVDMPQEMKNQGVTEDKLELLRGVSGAFRPGILTALMGVSGAGK
TTLMDVLAGRKTGGYIKGNITISGYPKKQETFARISGYCEQNDIHSPHVTVYESLLCSAWLRLSHEVSSETRKM
FIEEVMQLMELQPLRQALVGLPGVSGLSTEQRKRLTIAVELVANPSIIFMDEPTSGLDARAAAIVMRTVRNTVD
TGRTVVCTIHQPSIDIFEAFDELFLMKKGGQEIYVGPLGPHSCHLIKYFEGIEGVPKIKDGCNPATWMLEVTSSA
QESVFGVDFTAIYGNSELYRRNKGLIERFSTPSPESTELYFPTQYSQSFFTQCMACLCKQHWSYWRNPPYTAVR
LLFTTVIALMFGTMFWDLGSKKTKRQDLFNAMGSMYAAIVFLGIQNASSVQPVVAVERTVFYRERAAGMYSP
LPYAFAQVLIELPYVFIQSVVYGLIVYAMIGFEWTAAKFFWYLFFMYFTLLYYTFYGMMSVAVTPNQHIASIVS
SAFYSIWNLFSGFIIPRPRIPVWWRWYAWACPVAYTLYGLVSSQFGDIKHTLESGETVEDFVRSYFGFKHELLG
AVAVAVFGFATLFAFIFAISIKFFNYQRR

>XP_024632994.1_PDR1_X3_Mt
MEGTDIYRATNSLRARSSTVWRQSGVEVFSKSSREEDDEEALKWAALEKLPTYNRLRKGLLTASHGGAHEVD
VGDLAFQEKQKLLERLVKVAEEDNERFLLKVKERVDRVGLDIPTIEVRYQNLKIDAEAFVGSRALPSFINAATN
VVEGVLNFLHIIPTKKRHVSILKDVSGIVKPRRMTLLLGPPGSGKTTLLLALSGKLDPSLQLTGSVTYNGHGLNE
FVPQRTAAYISQHDVHIGEMTVRETLAFSARCQGVGSRYDMLSELSRREKAANIKPDPDIDVYMKAIATEGQE
YSISTDYVLKILGLDICADTMVGDEMLRGISGGQRKRVTTGEMLVGPANALFMDEISTGLDSSTTFQIVSSLRQ
YVHIMNGTAVISLLQPAPETYDLFDDIILISDGQVVYHGPREYVLDFFETMGFKCPERKGAADFLQEVTSKKDQ
AQYWVRRDQPYRFVTVTQFAEAFQSFHIGRKLAEELSVPFDKTKSHPAALTTKEYGLNKTELLKANFSREYLL
MKRNSFVYIFKLTQLFIMALIAMTLFFRTEMHRNDQDDAGVYAGALFFTLVTMMFNGMSEISMTIAKLPVYYK
QRDLLFYPSWAYAIPSWILKIPVSLMEVSLWVFLTYYVIGFDPNVGRMFKQFVVLFFMSQMASGLFRAIASLGR
NMIVANTFGSFALLTFLSLGGFILSRKDIKGWWIWGYWISPLMYGQNALMANEFLGHSWHNATADLGKDYLD
TRGFFPHAYWYWIGVGGLVGFVFLFNVAFGVALAVLGPFDKPSATITEDSEDDSSTVQEVELPRIESSGRADSV
TESSHGKKKGMVLPFEPHSITFDDIVYSVDMPVEMKEQGVREDRLVLLKGVSGAFRPGVLTALMGVSGAGKT
TLMDVLAGRKTGGYIDGDIKVSGYPKKQETFARISGYCEQNDIHSPHVTVYESLLYSAWLRLPSGVDSNTRKM
FIDEVMDLVELNSLRNSLVGLPGVSGLSTEQRKRLTIAVELVANPSIIFMDEPTSGLDARAAAIVMRTVRNTVD
TGRTVVCTIHQPSIDIFEAFDELFLMKRGGQEIYVGPLGRHSTHLIKYFESIDGVSKIKDGYNPATWMLEVTTTA
QELNLGVDFTDLYKNSDLYRRNKQLIQELSVPAPGSKDLHFPTQFSQSFLVQCQACLWKQRWSYWRNPPYTA
VRFFFTTFIGLMFGTMFWDLGGKHSSRQDLLNAVGSMYTAVLFLGVQNSSSVQPVVAVERTVFYREKAAGM
YSALPYAFSQILVELPYVFAQAVTYGVIVYAMIGFDWTAEKFLWYLFFMYFTLLYFTFYGMMAVAVTPNHHV
ASIVAAAFYAIWNLFSGFVVPRPSIPIWWRWYYWACPVAWTIYGLVASQFGDITTVMTTEGGKDVKTFLDDFF
GIQHDFIGWCALVVGGIAVAFAFIFAVAIKSFNFQKR

>XP_003597818.1_PDR1_X2_Mt
MEGTDIYRATNSLRARSSTVWRQSGVEVFSKSSREEDDEEALKWAALEKLPTYNRLRKGLLTASHGGAHEVD
VGDLAFQEKQKLLERLVKVAEEDNERFLLKVKERVDRVGLDIPTIEVRYQNLKIDAEAFVGSRALPSFINAATN
VVEGVLNFLHIIPTKKRHVSILKDVSGIVKPRRMTLLLGPPGSGKTTLLLALSGKLDPSLQLTGSVTYNGHGLNE
FVPQRTAAYISQHDVHIGEMTVRETLAFSARCQGVGSRYDMLSELSRREKAANIKPDPDIDVYMKAIATEGQE
YSISTDYVLKILGLDICADTMVGDEMLRGISGGQRKRVTTGEMLVGPANALFMDEISTGLDSSTTFQIVSSLRQ
YVHIMNGTAVISLLQPAPETYDLFDDIILISDGQVVYHGPREYVLDFFETMGFKCPERKGAADFLQEVTSKKDQ
AQYWVRRDQPYRFVTVTQFAEAFQSFHIGRKLAEELSVPFDKTKSHPAALTTKEYGLNKTELLKANFSREYLL
MKRNSFVYIFKLTQLFIMALIAMTLFFRTEMHRNDQDDAGVYAGALFFTLVTMMFNGMSEISMTIAKLPVYYK
QRDLLFYPSWAYAIPSWILKIPVSLMEVSLWVFLTYYVIGFDPNVGRMFKQFVVLFFMSQMASGLFRAIASLGR
NMIVANTFGSFALLTFLSLGGFILSRKDIKGWWIWGYWISPLMYGQNALMANEFLGHSWHNATADLGKDYLD
TRGFFPHAYWYWIGVGGLVGFVFLFNVAFGVALAVLGPFDKPSATITEDSEDDSSTVQEVELPRIESSGRADSV
TESSHGKKKGMVLPFEPHSITFDDIVYSVDMPVEMKEQGVREDRLVLLKGVSGAFRPGVLTALMGVSGAGKT

TLMDVLAGRKTGGYIDGDIKVSGYPKKQETFARISGYCEQNDIHSPHVTVYESLLYSAWLRLPSGVDSNTRKM
FIDEVMDLVELNSLRNSLVGLPGVSGLSTEQRKRLTIAVELVANPSIIFMDEPTSGLDARAAAIVMRTVRNTVD
TGRTVVCTIHQPSIDIFEAFDELFLMKRGGQEIYVGPLGRHSTHLIKYFESIDGVSKIKDGYNPATWMLEVTTTA
QELNLGVDFTDLYKNSDLYRRNKQLIQELSVPAPGSKDLHFPTQFSQSFLVQCQACLWKQRWSYWRNPPYTA
VRFFFTTFIGLMFGTMFWDLGGKHSSRQDLLNAVGSMYTAVLFLGVQNSSSVQPVVAVERTVFYREKAAGM
YSALPYAFSQILVELPYVFAQAVIYGVIVYAMIGFDWTAEKFLWYLFFMYFTLLYFTFYGMMAVAVTPNHHV
ASIVAAAFYAIWNLFSGFVVPRPSIPIWWRWYYWACPVAWTIYGLVASQFGDITTVMSTEGGKDVKTFLDDFF
GIQHDFIGWCALVVGGIAVGFAFIFAVAIKSFNFQKR

>XP_014623541.1_PDR1_X2_Gm
MLQESSPVYSLNFSVESLYFTRSNFTYIFMHATLLFHITSQNCTSREKVCCSSIFLTLSLLTMENDSSLRVSSSIRR
DASDIFSPSSFEEDDEEALKWAALDKLPTYNRLKKGLLITSNGEVNEIDVTDMGTQRRKEVLERLVRDAEEDN
EKFLLKLRERIDRVGVSIPTIEARFEHLNVEAEAYVGSRALPTFFNFIVNTVESYLNYLHILSSKKKHVTILKDVS
GIVKPCRMTLLLGPPSSGKTTLLLALAGKLDPDLKVSGRVTYNGHGMNEFVPQRTAAYISQDDVHIGEMTVRE
TLAFSARCQGVGSRYDMLSELSRREIVTDIKPDPNIDIYMKAIASEGQEANQMMTEYVLKILGLEMCADIVVGD
EMLRGISGGQRKRVTTGEMLVGPTNALFMDEISSGLDSSSTVQIIKCLRQMVHILDGTAVISLLQPEPETYELFD
DIILLSDGQIVYQGPREFVLEFFESKGFRCPERKAVADFLQEVTSRKDQQQYWIHKDEPYSFVSVNEFAEAFRCF
HVGRKLGDELAVPFDKTKNHPAALTTKKYGVNKKELLKANFSREYLLMKRNAFVYIFKLSQLALMAVVAMT
VFLRTEMHKDSVDNGGVYTGALFFSIVMILFNGMADISMTVAKLPIFYKQRDLLFYPAWAYAIPGWILKIPITL
AEVVVWVSITYYVIGFDPSVARFFKQYLLLLLLGQMASALFRTIAAIGRNMIIANTFGSFAIVTLLTLGGFILSRE
DVKKWWIWGYWISPIMYEQNAMMVNEFLGQSWSHVLPNSTESLGVEVLKSRGFFTHASWYWIGAGALLGFV
VLLNITFTLALTYLNPPEMSRAVIFKESHGNRNKDRTLDDIRLSLRLTGNAPSSNLEIGNLDDNGTESMSSRSAS
VRPKAAVESSHRRKRGMVLPFEPHSLTFDGITYSVDMPQEMKNQGVVEDRLVLLKGVSGAFRPGVLTALMGV
SGAGKTTLMDVLAGRKTGGYIEGSITISGYPKNQETYAQISGYCEQNDIHSPHVTIYESLLYSAWLRLSPEVNSE
TRKMFIEEVMELVELNLLREALVGLPGVSGLSTEQRKRLTIAVELVANPSIIFMDEPISGLDARAAAIVMRTVRN
IVDTGRTIVCTIHQPSIDIFEAFDELFLLKRGGREIYVGPLGRHSNHLVEYFERIEGVGKIKDGHNPAAWMLEITT
PAREMDLNVDFSDIYKNSVLCRRNKALVAELSKPAPGSKELHFPTQYAQPFFVQCKACLWKQHWSYWRNPPY
TAVRFLFTTFVALMFGTMFWDLGSKTRRKQDLFNAIGSMYNAILFLGIQNALSVQPVVAIERTVFYRERAAGM
YSAIPYALAQVVIELPYIFVQAVTYGIIVYAMIGFEWTASKFFWYLFFMYFTFLYFTFYGMMTVAVTPNQHIASI
VATAFYGIWNLFSGFVVPRPFYILYGDRAFLCGGDGTIGHVLWLGAYMDWLHHSLGI

>XP_003546218.1_PDR1_Gm
MEGSDIYRASNSLRRSSTVWRNSGVEVFSRSSREEDDEEALKWAALEKLPTYNRLRKGLLTASHGVANEIDVS
DLGIQERQKLLERLVKVAEEDNERFLLKLKERIDRVGLDIPTIEVRYEHLNIEAEAFVGSRALPSFINSVTNVVEG
FFNLLHISTSKKKHVTILKDVSGIIKPRRMTLLLGPPSSGKTTLLLALSGKLDKTLKVSGRVTYNGHELNEFVPQ
RTAAYISQHDLHIGEMTVRETLAFSARCQGVGSRYDMLSELSRREKAANIKPDPDLDVYMKATATEGQESSLV
TDYTLKILGLDICADTMVGDEMLRGISGGQRKRVTTGEMLVGPANALFMDEISTGLDSSTTFQIVSFLRQYVHI
LNGTAVISLLQPAPETYDLFDDIILISDGQVVYHGPREYVLDFFESMGFRCPERKGVADFLQEVTSKKDQAQYW
ARRDQPYRFVTVTQFSEAFQSFHIGGKLGEELAVPFDKTKSHPAALTTKKYGINKKELLKANLSREYLLMKRN
SFVYIFKLCQLSIMALMTMTLFLRTELHRNNMDDAGLYAGALFFTLVMIMFNGMAEISMTIAKLPVFYKQRDL
LFYPSWAYAIPSWILKIPVTLLEVAVWVFLTYYVIGFDPNVGRLFKQYLILLFIGQMASALFRAIAALGRNMIVS
NTFGAFAVLTFLTLGGFVMAKSDIKNWWIWGYWISPLMYGQTALMVNEFLSNSWHNSSRNLGVEYLESRGFP
SSAYWYWLGLGAMAGFVLLFNVMFSAALEILGPFDKPQATIAEEESPNEVTVAEVELPRIESSGRGGSVVESSH
GKKKGMVLPFEPHSITFDEVVYSVDMPQEMKEQGVQEDRLVLLKGVSGAFRPGVLTALMGVSGAGKTTLMD
VLAGRKTGGYIDGNIKISGYPKKQETFARISGYCEQNDIHSPHVTVYESLLYSAWLRLPSSVDSQTRKMFIEEV
MELVELNPLRNSLVGLPGVSGLSTEQRKRLTIAVELVANPSIIFMDEPTSGLDARAAAIVMRTVRNTVDTGRTV
VCTIHQPSIDIFEAFDELFLMKRGGQEIYVGPLGRHSSHLIKYFESIEGVSKIKDGYNPATWMLEVTTSAQELSLG
VDFTDLYKNSDLYRRNKQLIQELGQPAPGSKDLYFPTQYSQSFLVQCQACLWKQRWSYWRNPPYTAVRFFFT
TFIALMFGTMFWDLGSRRTTRGDLLNALGSMYTAVLFLGIQNASSVQPVVAVERTVFYREKAAGMYSALPYA
FAQVLVEIPYIFAQAVTYGLIVYAMIGFDWTAEKFFWYLFFSFFSLLYFTFYGMMAVGVTPNHHVAAIVAAAF
YAIWNLFSGFIVVRPKMPVWWRWYYWACPVAWTLYGLIASQFGDITERMPGEDNKMVKDFVEDYFGFKHDF
VGVCAVVVAGIAVAFALIFGVAIKTFNFQKR

>XP_004239864.1_PDR1-l_Sl
MEPVNLNSMRGSSMRGSMRGSLRASTSNSIWRNNGVDAFSRSTRDEDDEEALKWAALEKLPTFDRLRKGLLF
GSQGAANEIDVNDLGYQERKNLLERLVKVADEDNEKFLMKLKNRIDRVGIDMPSIEVRYEHLNIEADAYAGSR
ALPTFINFMTNFVETLLNSLHILPSKKRQITILKDVSGMIKPCRMTLLLGPPSSGKTTLLLALAGKLDPALRVTGN
VTYNGHELHEFVPQRTAVYISQHDLHIGEMTVRETLEFSARCQGVGSRFEMLAELSRREKAANIKPDPDIDIYM
KAAATEGQEANVVTDYVLKILGLDICADTMVGDEMIRGISGGQKKRVTTGEMLVGPSKALFMDEISTGLDSST
TFSIVNSLRQSVQLLKGTAVISLLQPAPETYNLFDDIILLSDGYIVYQGPREAVLDFFESMGFKCPERKGAADFL
QEVTSKKDQQQYWAKRNEPYRFITSKEFSEAYQSFHVGRKLSDELATPYDKTKSHPAALSTKKYGIGTKQLLK
VCAEREFLLMKRNSFVYIFKLTQLAIMALITMSVFFRTKLPRDDMDDGGIYAGALFFVVVMIMFNGMAEIALTI
FKLPVYFKQRDLLFFPSWAYALPTWILKIPITFVECGMWTFLTYYVMGFDPNVSRLFKQFLLLVLVHQMASAL
FRFIGAVGRTMGVASTFGAFALLLQFALGGFVLAREDVKKWWIWGYWTSPLMYSVNSILVNEFDGKNWKHI
APNGTEPLGAAVVRSRGFFPDAYWYWIGCGALFGFTMIFNFFYSIALAYLDPFGKPQAMISEDGEDAVELTERS
ETEGQDKKKGMVLPFEPHSITFDNIVYSVDMPQEMKEQGSAEDRLVLLKGVSGAFRPGVLTALMGVSGAGKT
TLMDVLAGRKTGGYIDGDIKISGYPKKQETFARISGYCEQNDIHSPYVTVYESLVYSAWLRLPQDVDENKRKM
FVDEVMELVELAPLRSALVGLPGVNGLSTEQRKRLTIAVELVANPSIIFMDEPTSGLDARAAAIVMRAVRNTVD
TGRTVVCTIHQPSIDIFEAFDELFLMKRGGQEIYVGPLGRHSCHLIKYFESMPGVGKIKEAYNPATWMLEVTAS
SQEMMLGVDFADLYKNSDLYRRNKALIAELSTPRPATKDLHFETQFSQPFWTQCMACLWKQHWSYWRNPAY
TAVRFIFTTFIALVFGTMFWDLGTKVSRSQDLINAMGSMYAATLFLGVQNSSSVQPVVAVERTVFYREKAAG
MYSAIPYAFGQVVIEIPYVFVQSAFYGVIVYAMIGFEWTAVKFLWYFFFMYCTLLYFTFYGMMTVAVTPNQN
VASIVAAFFYAVWNLFSGFIVPRPRIPIWWRWYYWACPVAWTLYGLVASQFGDIQTPLTDDENVEQYLRRYF
GFKHDFLGVVAAVIVALPVMFALTFALGIKAFNFQRR

>XP_003597816.3_PDR1_X1_Mt
MDWLFNDGQPKEAMKAMKCYESLLNHKFSMSSQPSNSLLKVLFRHDKKTMMLDDHRSVHQYCLEKKNKKH
KLVLRLLNKAKMEGTDIYRATNSLRARSSTVWRQSGVEVFSKSSREEDDEEALKWAALEKLPTYNRLRKGLL
TASHGGAHEVDVGDLAFKEKQKLLERLVRVAEEDNEGFLLKVKERVDRVGLDIPTIEVRYQNLKIDAEAFVGS
RALPSFINAATNVVEGVFNFLHIIPTKKRHVAILRDVSGIIKPRRMTLLLGPPGSGKTTLLLALSGKLDSSFQLSG
NVTYNGHGLNEFVPQRTAAYISQHDVHIGEMTVRETLAFSARCQGVGSRYDMLSELSRREKVANIKPDPDIDV
YMKAIATEGQESSISTDYVLKILGLDICADTMVGDEMLRGISGGQRKRVTTGEMLVGPANALFMDEISTGLDSS
TTFQIVSSLRQYVHIMNGTAVISLLQPAPETYDLFDDIILISDGQVVYHGPREYVLDFFESMGFKCPERKGVADF
LQEVTSKKDQAQYWVRRDQPYRYVTVTQFAEAFQSFHIGGKLAEELSIPFDKTKSHPAALTTKEYGLNKTELL
KANFSREYLLMKRNSFVYIFKLTQLFIMALIAMTLFFRTEMHRDDQDDAGVYAGALFFTLVTMMFNGMSEIS
MTIAKLPVYYKQRDLLFYPSWAYAIPSWILKIPVSLVEVSLWVFLTYYVIGFDPNVGRMFKQFVVLFFMSQMA
SGLFRAIASLGRNMIVANTFGSFAVLTLFALGGFILSRKDIKSWWIWGYWISPMMYGQNALMANEFLANSWH
NATSDLGKDYLDTRGFFPHAYWYWIGVGGLAGFVFLFNAAFGVALAVLGPFDKPSATITDNSEDDSSNYMTA
QEVELPRIESSGRGDSVTVSSHGKKKGMVLPFEPHSITFDDIVYSVDMPAEMKEQGVTEDRLVLLKGVSGAFRP
GVLTALMGVSGAGKTTLMDVLAGRKTGGYIDGDIKVSGYPKKQETFARISGYCEQNDIHSPHVTVYESLLYSA
WLRLPSGVDSNTRKMFIEEVMDLVELNSLRDSLVGLPGVSGLSTEQRKRLTIAVELVANPSIIFMDEPTSGLDA
RAAAIVMRTVRNTVDTGRTVVCTIHQPSIDIFEAFDELFLMKRGGQEIYVGPLGRHSTHLIKYFESIDGVSKIKD
GYNPATWMLEVTTTAQELNLGVDFTDLYKNSDLYRRNKQLIQELGVPAPGSKDLHFPTQFSQSFLVQCQACL
WKQRWSYWRNPPYTAVRFFFTTFIALMFGTMFWDLGGKHSRRQDLLNAVGSMYTAVLFLGVQNSSSVQPVV
AVERTVFNREKAAGMYSALPYAFSQILVELPYVFAQAVTYGVIVYAMIGFDWTAEKFLWYLFFMYFTLLYFTF
YGMMAVAVTPNHHVASIVAAAFYAIWNLFSGFVVPRPSIPIWWRWYYWACPVAWTIYGLVASQFGDITTVM
TTEGGKDVKTFLDDFFGIQHDFIGWCALVVGGIAVAFAFIFAVAIKSFNFQKR

>XP_021626775.1_PDR1-l_Me
MESADLYRAGSSFRRGSSLTRRNNGLEIFAQSFREDDDEESLKWAALEKLPTYDRLRKGIITTMTGGASEIDVH
NIGLQERKNLLERLVRVADEDNEKFLLKLKNRIDRVGIDIPTIEVRFEHLTVEAEAYEGSRALPTFFNYFINMLE
GILNYFHILSSRKKHLHILKDVSGIIKPSRMTLLLGPPSSGKTTLLLALAGKLDPALKVSGWVTYNGHHLHEFIP
QRTAAYISQHDLHIGEMTVRETLAFSARCQGVGSRYDLLAELSRREIAANIKPDSDIDVFMKAAATEGQEANVI
TDYILKVLGLEVCADTMVGDEMLRGISGGQRKRVTTGEMLVGPALALFMDEISTGLDSSTTYQIVNSLRQNVQ
ILKGTALISLLQPAPETYDLFDDIVLLSDGLIVYQGPREQVLRFFEFMGFQCPIRKGVADFLQEVTSRKDQMQY
WARKDEPYRFITAKEFSEAYESFQVGRRLVEELATPFEKANSHPAALTTKKYGVNKKELFKACFSRELLLMKR

NSFFYVFKFSQLTILALITMALFFRTEMHRDSVIDGGIYMGALFFIMLMVLFNGMAEIPMTIAKLPVFYKQRDLR
FYPAWAYALPSWILKIPITFIEVGISVFLTYYVIGFDPNVGRLFRQYLLLLLVNQMASGLFRSIAAVARNMIVAN
TFGSFILLLLFVLGGFILSRDHIKKWWLWAYWTSPTMYGQNAIVVNEFLGHSWSHVLPNSTEPLGVQVLKSRG
FFTEAYWYWLGLGALCGFTILFNFLYTIALTYLNEFSKPQAVTSKEPQDNGTARMEDGVHLSYLGSSSNQQTN
TAVIGDEIIRGKSPKSSQNNHRKGMVLPFEPYSITFDEIVYSVDMPREMKNEGVCEDKLVLLNGVSGAFRPGVL
TALMGVSGAGKTTLMDVLAGRKTGGYIEGSIKISGYPKKQETFARISGYCEQNDIHSPHITVYESLVFSAWLRL
SHEVDNETREMFVEEVMELVELNTLRQALVGLPGVNGLSTEQRKRLTIAVELVANPSIIFMDEPTSGLDARAA
AIVMRTVRNTVDTGRTVVCTIHQPSIDIFEAFDELFLLKRGGHEIYVGPLGRYSCHLIKYFEGIEGVNKIKDGYN
PATWMLEVTTAAQEMALGVDFADIYRNSELYGRNKALIKDLSKSAPGSKDLHFPNKYSESFFGQFSACLWKQ
HLSYWRNPPYTAIRFLFTAFIGLVFGTMFWDLGPKLTKQQDLFNAMGSMYAAVQFLGVQNASSVQPVVAVER
TVFYRERAAGMYSALPYAFGQVVIELPYIFMQAAVYGVTVYAMIGFEWNASKFFWYLYFTYFTLLYFTFYGM
MAVGVSPNHHISSIISSAFYGIWNLFSGFIIPRTRMPAWCSWYYWLNPVSWTLYGLVASQFGDIKEKLETGETV
ELFTRDYFGFRHDFLGLVAAVVFGFVILFAFIFAVSIKMFNFQRR

>XP_006597138.1_PDR1_X1_Gm
MLQESSPVYSLNFSVESLYFTRSNFTYIFMHATLLFHITSQNCTSREKVCCSSIFLTLSLLTMENDSSLRVSSSIRR
DASDIFSPSSFEEDDEEALKWAALDKLPTYNRLKKGLLITSNGEVNEIDVTDMGTQRRKEVLERLVRDAEEDN
EKFLLKLRERIDRVGVSIPTIEARFEHLNVEAEAYVGSRALPTFFNFIVNTVESYLNYLHILSSKKKHVTILKDVS
GIVKPCRMTLLLGPPSSGKTTLLLALAGKLDPDLKVSGRVTYNGHGMNEFVPQRTAAYISQDDVHIGEMTVRE
TLAFSARCQGVGSRYDMLSELSRREIVTDIKPDPNIDIYMKAIASEGQEANQMMTEYVLKILGLEMCADIVVGD
EMLRGISGGQRKRVTTGEMLVGPTNALFMDEISSGLDSSSTVQIIKCLRQMVHILDGTAVISLLQPEPETYELFD
DIILLSDGQIVYQGPREFVLEFFESKGFRCPERKAVADFLQEVTSRKDQQQYWIHKDEPYSFVSVNEFAEAFRCF
HVGRKLGDELAVPFDKTKNHPAALTTKKYGVNKKELLKANFSREYLLMKRNAFVYIFKLSQLALMAVVAMT
VFLRTEMHKDSVDNGGVYTGALFFSIVMILFNGMADISMTVAKLPIFYKQRDLLFYPAWAYAIPGWILKIPITL
AEVVVWVSITYYVIGFDPSVARFFKQYLLLLLLGQMASALFRTIAAIGRNMIIANTFGSFAIVTLLTLGGFILSRE
DVKKWWIWGYWISPIMYEQNAMMVNEFLGQSWSHVLPNSTESLGVEVLKSRGFFTHASWYWIGAGALLGFV
VLLNITFTLALTYLNPPEMSRAVIFKESHGNRNKDRTLDDIRLSLRLTGNAPSSNLEIGNLDDNGTESMSSRSAS
VRPKAAVESSHRRKRGMVLPFEPHSLTFDGITYSVDMPQEMKNQGVVEDRLVLLKGVSGAFRPGVLTALMGV
SGAGKTTLMDVLAGRKTGGYIEGSITISGYPKNQETYAQISGYCEQNDIHSPHVTIYESLLYSAWLRLSPEVNSE
TRKMFIEEVMELVELNLLREALVGLPGVSGLSTEQRKRLTIAVELVANPSIIFMDEPISGLDARAAAIVMRTVRN
IVDTGRTIVCTIHQPSIDIFEAFDELFLLKRGGREIYVGPLGRHSNHLVEYFERIEGVGKIKDGHNPAAWMLEITT
PAREMDLNVDFSDIYKNSVLCRRNKALVAELSKPAPGSKELHFPTQYAQPFFVQCKACLWKQHWSYWRNPPY
TAVRFLFTTFVALMFGTMFWDLGSKTRRKQDLFNAIGSMYNAILFLGIQNALSVQPVVAIERTVFYRERAAGM
YSAIPYALAQVVIELPYIFVQAVTYGIIVYAMIGFEWTASKFFWYLFFMYFTFLYFTFYGMMTVAVTPNQHIASI
VATAFYGIWNLFSGFVVPRPSIPVWWRWYYWACPVAWSLYGLVASQFGDITSAVELNETVKEFLRRYFGYRD
DFVGVAACVVVGFAVLFATIFAFSLKVFNFERR

>XP_003543663.1_PDR1_Gm
MEGSDIYRASNSLRRSSTAWRNSGVEVFSRSSREEDDEEALKWAALEKLPTYNRLRKGLLTASHGVANEIDVS
DLGTQERHKLLERLVKVAEEDNERFLLKLKERIDRVGLDIPTIEVRYEHLNIEAEAFVGSRALPSFINSVTNIIEG
FFNLLHITTSKKKHVTILKDVSGIIKPRRMTLLLGPPSSGKTTLLLALSGKLDKTLKVSGRVTYNGHELNEFVPQ
RTAAYISQHDLHIGEMTVRETLAFSARCQGVGSRYDMLSELSRREKAANIKPDPDLDVYMKATATEGQESSIV
TDYTLKILGLDICADTMVGDEMLRGISGGQRKRVTTGEMLVGPANALFMDEISTGLDSSTTFQIVNSLRQYVHI
LNGTAVISLLQPAPETYDLFDDIILISDGQVVYHGPREYVLDFFESMGFRCPERKGVADFLQEVTSKKDQAQYW
ARRDQPYRFVKVTQFAEAFQSFHIGRKLGEELVVPFDKTKSHPAALTTKKYGINKKELLKANLSREYLLMKRN
SFVYIFKLCQLSIMALMTMTLFLRTELHRNNMDDAGLYSGALFFTLIMIMFNGMAEISMTIAKLPVFYKQRDLL
FYPSWAYAIPSWILKIPVTLLEVAVWVFLTYYVIGFDPNVGRFFKQYLILLFIGQMASALFRAIAALGRNMIVSN
TFGAFAVLTFLTLGGYVMSKNDIKNWWIWGYWISPLMYGQNALMVNEFLSNSWHNTSRNLGVEYLESRGFP
SSSYWYWLGLGAMAGFVLLFNVMFSAALEILGPFDKPQATITEEESPNEGTVAEVELPRIESSGRGDSVVESSH
GKKKGMVLPFEPHSITFDEVIYSVDMPQEMKEQGVQEDRLVLLKGVSGAFRPGVLTALMGVSGAGKTTLMD
VLAGRKTGGYIDGSIKISGYPKKQETFARISGYCEQNDIHSPHVTVYESLLYSAWLRLPSGVDSKTRKMFIEEV
MELVELNPLRNSLVGLPGVSGLSTEQRKRLTIAVELVANPSIIFMDEPTSGLDARAAAIVMRTVRNTVDTGRTV
VCTIHQPSIDIFEAFDELFLMKRGGQEIYVGPLGRHSTHLIKYFESIGGVSKIKDGYNPATWMLEVTTSAQELSL
GVDFTDLYKNSDLYRRNKQLIQELGQPAPGSKDLYFPTQYSQSFLVQCQACLWKQRWSYWRNPPYTAVRFFF

TTFIALMFGTMFWDLGSRRTTRGDLLNALGSMYSAVLFLGIQNASSVQPVVAVERTVFYREKAAGMYSALPY
AFAQVLVEIPYIFAQAVTYGLIVYAMIGFDWTAEKFFWYLFFSFFSLLYFTFYGMMAVGVTPNHHVAAIVAAA
FYAIWNLFSGFIVVRPKMPVWWRWYYWACPVAWTLYGLIASQFGDITERMPGEDNKMVKEFIEDYFGFKHD
FVGICAVVVAGIAVAFALIFGAAIKTFNFQKR

>XP_021626777.1_PDR1-l_Me
MESVDLYRARSSFRRSSPLTRRNSGLEIFAPSFREEDDEESLKWAALEKLPTYERLRKGILTTMTGGVSEIDVHN
IGSQERKNFLERLVKVADKDNEKFLLKLKNRIDRVGIDIPTIEVRFEHLTVEAEAYEGSRALPTFFNYYINMLEG
LLNNFHILSSRKKRLHILKDVSGIIKPSRMTLLLGPPSSGKTTILLALAGKLDPTLKVSGRVTYNGHHLHEFIPQR
TAAYISQHDLHIGEMTVRETLAFSARCQGVGSRYDLLAELSRREIAENIKPDSDIDVFMKAAATEGQEVNVMT
DYILKVLGLEVCADTMVGDEMLRGISGGQRKRVTTGEMLVGPSLALFMDEISTGLDSSTTYQIVESLRQYVQIL
KGTALISLLQPAPETYDLFDDIILLSDGLIVYQGPREQVLQFFEFMGFQCPERKGVADFLQEVTSRKDQMQYWA
RKDEPYRFITAKEFSEAYKSFHVGRGLREELATPFEKANSHPAALTTKKYGVNKKELFKACCSREFLLMKRNSF
FYVFKLCQLTITTLIAMALFFRTEMHRDSVTDGGIYVGALFFIVLVVLFNGMAEISMTIAKLPVFYKQRDLCFYP
AWAYALPTWILKIPITFIEIGISVFMTYYVIGFDPNVGRLFRHYLVLLLVNQMASGLFRSIAAVGRNMIVANTFG
SFVLLLLFVSGGVVLSRDNIKKWWMWSYWTSPMMYGQNAIVVNEFLGHSWSHVLPKSIEPLGIQVLKSRGFF
TEAYWYWLGAGALCGFTIVFNLLYTVALTFLSEYSKPQAVTSKEPQDNGTGRMEDGVRLGYHGNSSNQQTST
VSRDEIIREKSSRSSQNNRKGMVLPFEPHSITFDEIVYSVDMPQAMKNEGVHEDKLVLLNSVSGSFRPSVLTAL
MGVSGAGKTTLMDVLAGRKTGGYIEGSITISGYPKKQETFARISGYCEQNDIHSPHITVYESLLFSAWLRLPCEV
ETREMFIKEVMELVELNTLGQALVGLPGVNGLSTEQRKRLTIAVELVANPSVIFMDEPTSGLDARAAAIVMRT
VRNTVDTGRTVVCTIHQPSIDIFEAFDELFLMKRGGQEIYVGPLGRHSCHLINYFEGIEGVEKIKDGYNPATWM
LEVTSTTQEMALGVDFADIYRNSELYRRNKALIKDLSKSAPGSKDLYFPNQYSLSFFGQYLACLWKQHLSYWR
NPPYTAIRFLFTAFIGLIFGTMFWDLGSKMKKQQDLFNAVGSMYAAVLFLGFIYASAVQPVVSVERTVFYRER
AAGMYSALPYAFGQIVVELPYVFMQAAVYGVTVYAMIGFEWNASKFFWYLYFTYFSLLYFTFYGMMAVGVS
PNHQISSIISFAFFIIWNLFSGFIIPRTMMPAWCSWYYWLNPVSWTLYGLITSQFGDIKETLETGETVEHFTRHYF
GFRHDFLGLVAAMVFVFVILFALSFAVSLKVFNFQKR

>NP_001308682.2_P_Zm
MDAAGDIQKVASMRRGDSGSIWRRGDDVFSRSSREEDDEEALRWAALEKLPTYDRVRRAMVPLGLGADGAE
AAGRKGLVDVDVLSLGPRERRALLERLVRVADEDNERFLLKLKDRVDRVGIDMPTIEVRFQNLEAEAEVRVG
SSGLPTVLNSVVNTIEEAANALHILPSRKRIMPILHDVSGIIKPRRMTLLLGPPGSGKTTLLLALAGRLDKDLKVS
GKVTYNGHEMTEFVPERTAAYISQHDLHIGEMTVRETLAFSARCQGVGSRFDMLTELSRREKAANIKPDADID
AFMKASAMGGQDANVVTDYILKILGLEICADTMVGDEMLRGISGGQRKRVTTGEMLVGPARALFMDEISTGL
DSSTTFQIVNSLRQSIHILGGTAVISLLQPAPETYNLFDDIILLSDGQVVYQGPREEVLEFFESVGFRCPERKGVA
DFLQEVTSKKDQKQYWARPNEPYRFVAVKEFATAFKSSHTGRSITNELAVPFDKSKSHPAALTTTRYGVSGKE
LLKANIDREILLMKRNSFVYMFRTFQLMLMSIIAMTLFFRTKMKHGTVNDGGLYMGALFFGVLMIMFNGFSEL
ALTVFKLPVFFKQRDLLFFPAWSYTIPSWILKVPITFIEVGGYVFLTYYVIGFDPNVGRFFKQYLLLLVVNQMAA
SLFRFIGGVSRNMIVANVFASFMLLVVMVLGGFILVRDKVKKWWIWGYWISPMMYAQNAISVNEMLGHSWD
KILNSTASNETLGVQVLKSRGVFTEAKWYWIGFGAMVGFTILFNALFTVALTYLKPYGNSRPSVSEEELKEKH
ANIKGEVLDGNHLVSASSHRSTGVNPETDSAIMEDDSALTKRGMILPFVPLSLTFDNIKYSVDMPQEMKAQGV
QEDRLELLKGVSGSFRPGVLTALMGVSGAGKTTLMDVLAGRKTGGYIEGDIRISGYPKKQDTFARVSGYCEQN
DIHSPQVTVYESLLFSAWLRLPKDVDSNKRKIFIEEVMELVELKPLRNALVGLPGVNGLSTEQRKRLTIAVELV
ANPSIIFMDEPTSGLDARAAAIVMRTVRNTVDTGRTVVCTIHQPSIDIFEAFDELFLMKRGGEEIYAGPLGHHSS
DLIKYFESLHGVSKIKDGYNPATWMLEVTTTSQEQILGVDFSDIYKKSELYQRNKALIKELSQPAPGSTDLHFPS
KYAQSSITQCVACLWKQNLSYWRNPPYNTVRFFFTTIIALLLGTIFWDLGGKTYTSQDLMNAMGSMYSAVLFI
GVMNCTSVQPVVAVERTVFYRERAAGMYSAFPYAFGQVVIELPYALAQDILYGVIVYSMIGFEWTAAKFFWY
LFFGYFTLLYFTFYGMMAVGLTPNYHIAAIVSSAFYAIWNLFSGFIIPRPKVPIWWRWYCWICPVAWTLYGLVV
SQFGDVMTPMDDGRAVKVFVEDYFDFKHSWLGWVAAVVVAFAVLFATLFGFAIMKLNFQKR

>XP_013454713.1_PDR1_Mt
MEGSDIYKAGNSFRMSSSSTTVWRNSKMEAFSMSSRHGGEDEEALRWAALEKLPTYNRLRKGLLATSRGVAN
EIDILSDLGFQERQKLLDRLINVAEEGNEKFLLKLKERIDRVGIEIPTIEVRYEHLIVDAEAYVGGRALPTLLNSV
MNAVESILTYLHIFTSKKKHMTILKDVSGIVKPRRMTLLLGPPSSGKTTLLLALSGKLDPNLKVSGRVTYNGHG
MDEFVPQRTAAYISQHDVHIGEMTVRETLAFSARCQGVGSRYDLLSELSRREKEAKIKPDPDIDVFMKAVATG

GQQESVVTDYVLKLLGLDVCADTMVGNEMLRGISGGQRKRVTTGEMLVGPANALFMDEISTGLDSSTTFQIV
KSLRQYVHILNGTAVISLLQPAPETYELFDDIILISDGQIVYQGPREHVLDFFESVGFKCPERKGVADFLQEVTSK
KDQEQYWVDREKPYRFLTVTQFAEAFQSYHVGRKTRDELAIPFDKSKNHPAALTTKKYGVNKKELLKANFSR
EYLLMKRNSFVYIFKICQLTLMATVTMTLFLRTEMHRDSLNDGGVYAGAIFFSVVMLMFNGLAELSMTIAKLP
SFYKQRDLLFFPSWAYAIPTWILKIPITFLEVAVWVFLTYYVIGFDPNVTRLLKQYLLLLLINQMASGLFRAIAA
LGRNMIVANTFGSFALLALLTLGGFIMSRRDIKSWWIWGYWISPLMYGQNAIMVNEFLGDSWNHFTPNSNKTL
GIQVLESRGFFTEAYWYWIGIGALTGFMFLFNILFTMALTHLNPFDKPQAKINEESEDSITNGTLQEVELPRIASL
GEYVVSSSNRKKRGMILPFEPHSIIFDQVVYSVDMPQEMKVQGVVEDRLVLLKGVSGAFRPGVLTALMGVSG
AGKTTLMDVLAGRKTGGYIDGTIKISGYLKRQETFARISGYCEQNDIHSPHVTVYESMVYSAWLRLPAEVDSN
SRKMFIEEVMELVELNPLRNSLVGLPGVNGLSTEQRKRLTIAVELVANPSIIFMDEPTSGLDARAAAIVMRTVR
NTVDTGRTVVCTIHQPSIDIFEAFDELLLMKRGGQETYVGPLGRHSNQLIKYFESIEGVSKIKDGYNPATWMLE
VTSSAQEHTLGVDFHDIYKNSELYRRNKQLIVELGKPAPGSKDLHFSAQYSQSFWIQCLACLWKQHWSYWRN
PPYTAVRFFFTTFIALMFGTMFWNLGRKYSNRQDLFNALGSMYTAVLFLGVQNSSSVQPVVAVERSVFYRERA
AGMYSALPYAFAQVIIELPYIFVQATSYGVIVYAMIGFEWTLEKFFWYIFFMYFTLCYFTFYGMMAVAVTPNH
HVASIVASAFYAIWNLFSGFIIPRPRIPVWWRWYYWACPVAWTLYGLVASQFGDINNIMESENKSVQEFIRSYF
DFKHDFIGVCAVVVVGTAVLFACIFAVSIKLFNFQRR

>XP_003545503.1_PDR1_X3_Gm
MEGRNISRVDSARASGSNIWRNNNMDVFSTSEREDDEDALKWAAIERLPTYLRIQRSILNNEDGKGREVDIKQ
LGLTERKILLERLVKIAEEDNERFLLKLRERMDRVGLDIPTIEVRFEHINVEAQVYVGGRALPSMLNFFANVLEG
FLNYLHIIPSPKKPLRILQNISGIIKPRRMTLLLGPPGSGKTTLLLALAGKLGKDLKHSGRVTYNGHELEEFVPQR
TSAYISQYDNHIGEMTVRETLAFSARCQGVGQNYEILAELLRREKQAKIKPDPDIDSYMKAAALGRQRTSVVT
DYILKILGLEVCADIMVGDGMIRGISGGQKKRVTTGEMLVGPIKVLFMDEISTGLDSSTTFQIINSIRQSIHILNGT
ALVSLLQPAPETYELFDDIILLTDGQIVYQGPRENVLEFFESMGFKCPERKGVADFLQEVTSKKDQWQYWVRK
DEPYSFVTVKDFAEAFQLFHIGQNLGEELASPFDRSKSHPNVLTTKKYGVNKKELLRACASREFLLMKRNSFV
YIFKVTQLIYLAIITTTLFLRTKMHRDTVEDGGAYMGALFFAVTVAMFNGISELNMAIMKLPVFYKQRDLLFYP
AWAYSLPPWILKIPITLIEVAIWEGISYYAIGFDPSLVRLLKQYLIILCINQMASSLFRLMAAFGRDVIVANTAGSF
ALLIVLVLGGFVISRENVHKWFLWGYWSSPLMYGQNAIAVNEFLGHSWRKVTPNSNETLGVLILKTRGFFPEA
YWYWIGVGALIGYVFLYNFLFTLALQYLSPFRKDQASGLSQEKLLERNASTAEELIQLPKGNSSSETNIVEEANI
PSRSFSGRISDDKASGSGRRGMVLPFQPLSLTFDEMKYSVDMPQEMKKQGVFEERLELLKGVSGVFRPGVLTA
LMGVSGAGKTTLMDVLAGRKTGGYIEGSITISGYPKRQETFARISGYCEQFDIHSPNVTVYESLLYSAWLRLPR
EVDRATRKMFIEEVMELVELNSIREALVGLPGENGLSTEQRKRLTIAVELVANPSIIFMDEPTSGLDARAAAIVM
RTVRNTVNTGRTVVCTIHQPSIDIFDAFDELLLLKLGGEQIYAGPLGRHCSHLIQYFEAIQGVPKIKEGYNPATW
MLEVTSAGTEASIKVNFTNVYRNSELYGRNKQLIQELSIPPQGSRDLHFDSQYSQTLVTQCKACLWKQHLSYW
RNTSYTAVRLLFTMLIALLFGIIFWDIGLKRSKEQDLFNAMGSMYAAVTFIGVQNGASVQPIIAVERTVFYRER
AAGMYSALPYALAQVIIELPHILVQALMYGIIVYAMMGFDWTTSKFLWYLFFMYFTFLYYTFYGMMTMAITP
NAHVAAILSSAFYAIWSLFSGFVIPLSRIPIWWKWYYWICPVAWTLNGLVASQYGDNRDKLENGQRVEEFVKS
YFGFEHDFLGVVASVVAGFSLLFAFIFAFGIKVLNFQKR

>XP_006596116.1_PDR1_X2_Gm
MEGRNISRVDSARASGSNIWRNNNMDVFSTSEREDDEDALKWAAIERLPTYLRIQRSILNNEDGKGREVDIKQ
LGLTERKILLERLVKIAEEDNERFLLKLRERMDRVGLDIPTIEVRFEHINVEAQVYVGGRALPSMLNFFANVLED
LFFLPKGFLNYLHIIPSPKKPLRILQNISGIIKPRRMTLLLGPPGSGKTTLLLALAGKLGKDLKHSGRVTYNGHEL
EEFVPQRTSAYISQYDNHIGEMTVRETLAFSARCQGVGQNYEILAELLRREKQAKIKPDPDIDSYMKAAALGRQ
RTSVVTDYILKILGLEVCADIMVGDGMIRGISGGQKKRVTTGEMLVGPIKVLFMDEISTGLDSSTTFQIINSIRQSI
HILNGTALVSLLQPAPETYELFDDIILLTDGQIVYQGPRENVLEFFESMGFKCPERKGVADFLQEVTSKKDQWQ
YWVRKDEPYSFVTVKDFAEAFQLFHIGQNLGEELASPFDRSKSHPNVLTTKKYGVNKKELLRACASREFLLMK
RNSFVYIFKVTQLIYLAIITTTLFLRTKMHRDTVEDGGAYMGALFFAVTVAMFNGISELNMAIMKLPVFYKQRD
LLFYPAWAYSLPPWILKIPITLIEVAIWEGISYYAIGFDPSLVRLLKQYLIILCINQMASSLFRLMAAFGRDVIVAN
TAGSFALLIVLVLGGFVISRENVHKWFLWGYWSSPLMYGQNAIAVNEFLGHSWRKVTPNSNETLGVLILKTRG
FFPEAYWYWIGVGALIGYVFLYNFLFTLALQYLSPFRKDQASGLSQEKLLERNASTAEELIQLPKGNSSSEEANI
PSRSFSGRISDDKASGSGRRGMVLPFQPLSLTFDEMKYSVDMPQEMKKQGVFEERLELLKGVSGVFRPGVLTA
LMGVSGAGKTTLMDVLAGRKTGGYIEGSITISGYPKRQETFARISGYCEQFDIHSPNVTVYESLLYSAWLRLPR
EVDRATRKMFIEEVMELVELNSIREALVGLPGENGLSTEQRKRLTIAVELVANPSIIFMDEPTSGLDARAAAIVM

RTVRNTVNTGRTVVCTIHQPSIDIFDAFDELLLLKLGGEQIYAGPLGRHCSHLIQYFEAIQGVPKIKEGYNPATW
MLEVTSAGTEASIKVNFTNVYRNSELYGRNKQLIQELSIPPQGSRDLHFDSQYSQTLVTQCKACLWKQHLSYW
RNTSYTAVRLLFTMLIALLFGIIFWDIGLKRSKEQDLFNAMGSMYAAVTFIGVQNGASVQPIIAVERTVFYRER
AAGMYSALPYALAQVIIELPHILVQALMYGIIVYAMMGFDWTTSKFLWYLFFMYFTFLYYTFYGMMTMAITP
NAHVAAILSSAFYAIWSLFSGFVIPLSRIPIWWKWYYWICPVAWTLNGLVASQYGDNRDKLENGQRVEEFVKS
YFGFEHDFLGVVASVVAGFSLLFAFIFAFGIKVLNFQKR

>XP_013454712.1_PDR1_Mt
MEGSDIYSASNSLRFSMRSSSTTGWRNGTMEAFAKSSRREEDDDEEALKWAALEKLPTYNRLRKGLLATSRG
VANEVDITDLGFQEKQKLLDRLINVAEEGNEKFLLKLKERIDRVGIEIPAIEVRYEHLNVEAEAFVGGRALPTLL
NSVTNTVESILISLHILTSRKKQMTILKDVSGIVKPRRMTLLLGPPSSGKTTLLLALSGKLDPNLKVSGRVTYNG
HGMDEFVPQRTAAYISQHDVHIGEMTVRETLAFSARCQGVGSRYDLLAELSRREKEANIKPDPDVDVFMKAM
ATGGQQESVATDYVLKLLGLDVCADTMVGNEMLRGISGGQRKRVTTGEMLVGPANALFMDEISTGLDSSTTF
QIVRSLQQYVHILNGTTVISLLQPAPETYELFDDIILISDGQIVYQGPREHILEFFESVGFKCPERKGAADFLQEVT
SKKDQEQYWVDREKPYRFVTVTQFAEAFQSYHVGRKTGDELAIPFDKSKNHPAALTTKKYGVNKKELLKANF
SREYLLMKRNSFVYIFKICQLLLMATIAMTLFLRTEMHRDSLNGGGVYSGALFFAVVMIMFNGMAELSMTIAK
LPSFYKQRDLLFFPSWAYAIPTWILKIPITFLEVAAWVFLTYYVIGFDPNVTRLLKQYLLLLLINQMASGLFRAIA
ALGRNMIVANTFGSFALLALLTLGGFVMSRKDIKSWWIWGYWISPLMYGQNAIMVNEFLGDSWNHFTPNSNK
TLGIQVLESRGFFTEAYWYWIGIGALTGFMFLFNILFTVALTYLDPFDKPQATINEESEDSTTNGTTQEVELPRIA
SSGGSNGADPSQRERRGMVLPFEPHSIAFDDVVYSVDMPQEMKVQGVLEDRLVLLKGVSGAFRPGVLTALMG
VSGAGKTTLMDVLAGRKTGGYIDGSIKISGYPKKQETFARISGYCEQNDIHSPHVTVYESLVYSAWLRLPADV
DSNTRKMFIEEVMELVELNPLRNSLVGLPGVNGLSTEQRKRLTIAVELVANPSIIFMDEPTSGLDARAAAIVMR
TVRNTVDTGRTVVCTIHQPSIDIFEAFDELFLMKRGGEEIYVGPLGRHSSQLIKYFESIEGVSKIKDGYNPATWM
LEVSSSAQELTLGIDFHHAYKNSELYRRNKQLIEELGKPAPGSNDLYFSAQYSQSFLVQCLACLWKQHWSYWR
NPPYTSVRFFFTVFIGLMFGTIFWDLGRKYSKRQDLFNALGSMYTAVLFLGVQNSSAVQPVVAVERSVFYRER
AAGMYSALPYAFAQVLIELPYIFVQAASYGVIVYAMIGFEWTVAKFLWYIFFMYCTLCYFTFYGMMAVAITPN
HHVASIVAAAFYAIWNLFSGFIVPRPMIPVWWRWYYWGCPVSWTLYGLIASQFGDITKIMESENESVQEFIRSY
FGMKHDFIGVCAVVVVGTAVLFACIFAVSIKVFNFQRR

>XP_006596115.1_PDR1_X1_Gm
MEGRNISRVDSARASGSNIWRNNNMDVFSTSEREDDEDALKWAAIERLPTYLRIQRSILNNEDGKGREVDIKQ
LGLTERKILLERLVKIAEEDNERFLLKLRERMDRVGLDIPTIEVRFEHINVEAQVYVGGRALPSMLNFFANVLED
LFFLPKGFLNYLHIIPSPKKPLRILQNISGIIKPRRMTLLLGPPGSGKTTLLLALAGKLGKDLKHSGRVTYNGHEL
EEFVPQRTSAYISQYDNHIGEMTVRETLAFSARCQGVGQNYEILAELLRREKQAKIKPDPDIDSYMKAAALGRQ
RTSVVTDYILKILGLEVCADIMVGDGMIRGISGGQKKRVTTGEMLVGPIKVLFMDEISTGLDSSTTFQIINSIRQSI
HILNGTALVSLLQPAPETYELFDDIILLTDGQIVYQGPRENVLEFFESMGFKCPERKGVADFLQEVTSKKDQWQ
YWVRKDEPYSFVTVKDFAEAFQLFHIGQNLGEELASPFDRSKSHPNVLTTKKYGVNKKELLRACASREFLLMK
RNSFVYIFKVTQLIYLAIITTTLFLRTKMHRDTVEDGGAYMGALFFAVTVAMFNGISELNMAIMKLPVFYKQRD
LLFYPAWAYSLPPWILKIPITLIEVAIWEGISYYAIGFDPSLVRLLKQYLIILCINQMASSLFRLMAAFGRDVIVAN
TAGSFALLIVLVLGGFVISRENVHKWFLWGYWSSPLMYGQNAIAVNEFLGHSWRKVTPNSNETLGVLILKTRG
FFPEAYWYWIGVGALIGYVFLYNFLFTLALQYLSPFRKDQASGLSQEKLLERNASTAEELIQLPKGNSSSETNIV
EEANIPSRSFSGRISDDKASGSGRRGMVLPFQPLSLTFDEMKYSVDMPQEMKKQGVFEERLELLKGVSGVFRPG
VLTALMGVSGAGKTTLMDVLAGRKTGGYIEGSITISGYPKRQETFARISGYCEQFDIHSPNVTVYESLLYSAWL
RLPREVDRATRKMFIEEVMELVELNSIREALVGLPGENGLSTEQRKRLTIAVELVANPSIIFMDEPTSGLDARAA
AIVMRTVRNTVNTGRTVVCTIHQPSIDIFDAFDELLLLKLGGEQIYAGPLGRHCSHLIQYFEAIQGVPKIKEGYN
PATWMLEVTSAGTEASIKVNFTNVYRNSELYGRNKQLIQELSIPPQGSRDLHFDSQYSQTLVTQCKACLWKQH
LSYWRNTSYTAVRLLFTMLIALLFGIIFWDIGLKRSKEQDLFNAMGSMYAAVTFIGVQNGASVQPIIAVERTVF
YRERAAGMYSALPYALAQVIIELPHILVQALMYGIIVYAMMGFDWTTSKFLWYLFFMYFTFLYYTFYGMMTM
AITPNAHVAAILSSAFYAIWSLFSGFVIPLSRIPIWWKWYYWICPVAWTLNGLVASQYGDNRDKLENGQRVEEF
VKSYFGFEHDFLGVVASVVAGFSLLFAFIFAFGIKVLNFQKR

>XP_021628885.1_PDR1-l_X2_Me
MEGDLYKASSSLRRGSSSVWRNNGLDVFSRSSREEDDEEALKWAALEKLPTYDRLRKGILVSVSKGGANEIDV
ENLGFQERKALLERLVKVAEEDNEKFLLKLKNRIDRVGIEVPTIEVRYEHLNVEAEALVGSNALPSFLNFSISIV

EGLLNYLHILPNRKRPLTILKDVSGVIKPSRMALLLGPPSSGKTTLLLALAGKLDPNLKVSGNVTYNGHALNEFI
PQRTAAYISQHDLHIGEMTVRETLAFSARCQGVGTQHEMLAELSRREKAANIKPDPDLDVFMKAAATEGQETS
VVTDYVLKILGLDICADTMVGNEMIRGISGGQRKRVTTGEMLVGPAKALFMDEISTGLDSSTTFSIVNSLRQSV
HILNGTAVISLLQPAPETYNLFDDIILLSDGQIVYQGPREHVLEFFENMGFKCPERKGVADFLQEVTSKKDQQQ
YWARKDQPYRFVTVKEFAEAFKSFEVGQTITQVLSTPFDKSKNHPAALTTKPYGVGKMELLKANFSREYLLM
KRNSFVYIFKLSQLIIMAIIGMTLFFRTEMSKDDLEGGGIYIGALFFTLITIMFNGMSELSMTIAKLPVFYKQRNIL
FFPPWAYSIPSWILKIPITFLEVGVWVFLSYYVIGFDPNVGRLFKQYLLLLLVNQMASALFRFIASVGRNMIVAN
TFGSFALLTLFALGGVILSRENIKKWWIWGYWVSPLMYGQNSILANEFLGNSWSHVPANSSSTESLGVQVLKN
GGYFPHAYWYWIGVGASAGYMFLFNFLYTVALTLLDTFEKPQAVISDEPEENDKTRGAIQLSQLESSHRTNTE
SGTSENNESSHNKKKGMVLPFEPHSITFDNVIYSVDMPQEMKHQGVVEDKLMLLKGVSGAFRPGVLTALMGV
SGAGKTTLMDVLAGRKTGGYIEGDVRISGYPKQQETFARISGYCEQNDIHSPHVTVYESLVYSAWLRLPSDVD
SETRKMFVEEVMELVELNPLRQALVGLPGVNGLSTEQRKRLTIAVELVANPSIIFMDEPTSGLDARAAAIVMRT
VRNTVDTGRTVVCTIHQPSIDIFEAFDELFLMKRGGEEIYVGPLGRHSCHLIEYFEGIEGVSKITDGYNPATWML
EVSSSAQELTLGVDFANIYRNSDLYRRNKEMIQELSKPAPGTEDLYFPTQYSQPFLTQCIACLWKQSWSYWRN
PPYTAVRFWFTTFIALMFGTIFWDLGTKTERQSDLSNALGSMYAAVLFLGLQNATSVQPVVAVERTVFYREKA
AGMYSAMPYAYAQALIEIPYIFVQTVVYSVITYAMIGFEWTAAKFFWYLFFLLFTLLYFTYYGMMTVAVTPNH
HIASIVSSAFYSIWSLFSGFMIPRTKMPVWWRWYYWGCPISWTLYGLLGSQFGDVKTMLGNTGQTVEEYVND
YYGIKHDFLGVVAGVVVGITVLFAFTFAISIKAFNFQKR

>XP_003528365.1_PDR1_X1_Gm
MEGSDIYRARNSLRANSSTVWRNSIMEAFSRSSRHEEDNDEEALKWAALEKLPTYNRLRKGLLTTSRGVANEI
DITELGFQERQKLLDRLINVAEEDNETLLLKLKERIDRVGIDIPTIEVRYEHLNVEAEAYVGSRALPTFLNFVTN
MVESFFTSLHILSGKKKHVTILRDVSGIIKPRRMALLLGPPSSGKTTLLLALSGKLDPTLKVSGRVNYNGHEMN
EFVPQRTAAYISQHDVHIGEMTVRETLAFSARCQGVGTRYDLLSELARREKEAKIKPDPDIDVYMKAAATGGQ
EASLVTDYVLKILGLDICADTMMGDEMLRGISGGQRKRVTTGEMLVGPANALFMDEISTGLDSSTTFQIVKSL
RQYVHILNGTAVISLLQPAPETYELFDDIVLISDGQIVYQGPREYVLEFFEYVGFQCPERKGVADFLQEVTSRKD
QEQYWIHRDESYRFVTVTEFAEAFQSFHVGRRIGEELATPFDKSKSHPAALTTKKYGVNKKELLKANFSREYL
LMKRNSFVYIFKLFQLTILAILTMTMFLRTEMHRNSLNDGGVYTGALFFAVVILMFNGLAEISMTIVKLPIFYKQ
RDLLFYPSWAYAIPSWILKIPITFIEAAVWVFLTYYVIGFDPNVGRLLKQYLVLLLINQMSSGLFRAIAALGRNM
IVASTFGSFALLVLFALGGFVLSRNDIKNWWIWGYWISPLMYGQNAIVVNEFLGDSWNHFTPNSNKTLGIQILE
SRGFFTHAYWYWIGIGALIGFMILFNIIYTLALTYLNPYDTPQTTITEESESGMTNGIAESAGRAIAVMSSSHKKK
RGMILPFEPYSITFDQIVYSVDMPLEMKDQGVREDRLVLLKGVSGAFRPGVLTALMGVSGAGKTTLMDVLAG
RKTGGYIEGNIKVSGYPKRQETFARISGYCEQNDIHSPHVTVYESLVYSAWLRLPAEVEAYTRKMFIEEVMELV
ELNPLRNSLVGLPGVNGLSTEQRKRLTIAVELVANPSIIFMDEPTSGLDARAAAIVMRTVRNTVDTGRTVVCTI
HQPSIDIFEAFDELFLMKRGGQEIYVGPLGRHSSQMIKYFESIEGVGKIKDGYNPATWMLEVTTPAQELNLGVD
FHEIYRNSGLCRRNKRLISELGNPAPGSKDLHFPTQYPQSLLVQCLACLWKQHWSYWRNPPYTAVRFLSTTVT
AVLFGTMFWDLGGKYSSRQDLFNAMGSMYNAVLFVGVQNSASVQPVVAIERTVFYRERAAGMYSALPYALA
QVIIELPYVFVQATSYSVIVYAMMGFEWTLQKFFWYVFFMYFTLCYFTFYGMMTVAVTPNHHVASVVASAFY
GIWNLFSGFVIARPSIPVWWRWYYWACPVAWTIYGLVASQFGDITNVMKSENMSVQEFIRSHLGIKHDFVGVS
AIMVSGFAVLFVIIFAVSIKAFNFQRR

>XP_020405525.1_P_X1_Zm
MDAAGDIQKVASMRRGDSGSIWRRGDDVFSRSSREEDDEEALRWAALEKLPTYDRVRRAMVPLGLGADGAE
AAGRKGLVDVDVLSLGPRERRALLERLVRVADEDNERFLLKLKDRVDRVGIDMPTIEVRFQNLEAEAEVRVG
SSGLPTVLNSVVNTIEEAANALHILPSRKRIMPILHDVSGIIKPRRMTLLLGPPGSGKTTLLLALAGRLDKDLKVS
GKVTYNGHEMTEFVPERTAAYISQHDLHIGEMTVRETLAFSARCQGVGSRFDMLTELSRREKAANIKPDADID
AFMKASAMGGQDANVVTDYILKILGLEICADTMVGDEMLRGISGGQRKRVTTGEMLVGPARALFMDEISTGL
DSSTTFQIVNSLRQSIHILGGTAVISLLQPAPETYNLFDDIILLSDGQVVYQGPREEVLEFFESVGFRCPERKGVA
DFLQEVTSKKDQKQYWARPNEPYRFVAVKEFATAFKSSHTGRSITNELAVPFDKSKSHPAALTTTRYGVSGKE
LLKANIDREILLMKRNSFVYMFRTFQLMLMSIIAMTLFFRTKMKHGTVNDGGLYMGALFFGVLMIMFNGFSEL
ALTVFKLPVFFKQRDLLFFPAWSYTIPSWILKVPITFIEVGGYVFLTYYVIGFDPNVGRFFKQYLLLLVVNQMAA
SLFRFIGGVSRNMIVANVFASFMLLVVMVLGGFILVRDKVKKWWIWGYWISPMMYAQNAISVNEMLGHSWD
KILNSTASNETLGVQVLKSRGVFTEAKWYWIGFGAMVGFTILFNALFTVALTYLKPYGNSRPSVSEEELKEKH
ANIKGEVLDGNHLVSASSHRSTGVNPETDSAIMEDDSALTKRGMILPFVPLSLTFDNIKYSVDMPQEMKAQGV

QEDRLELLKGVSGSFRPGVLTALMGVSGAGKTTLMDVLAGRKTGGYIEGDIRISGYPKKQDTFARVSGYCEQN
DIHSPQVTVYESLLFSAWLRLPKDVDSNKRKIFIEEVMELVELKPLRNALVGLPGVNGLSTEQRKRLTIAVELV
ANPSIIFMDEPTSGLDARAAAIVMRTVRNTVDTGRTVVCTIHQPSIDIFEAFDELFLMKRGGEEIYAGPLGHHSS
DLIKYFESLHGVSKIKDGYNPATWMLEVTTTSQEQILGVDFSDIYKKSELYQRNKALIKELSQPAPGSTDLHFPS
KYAQSSITQCVACLWKQNLSYWRNPPYNTVRFFFTTIIALLLGTIFWDLGGKTYTSQDLMNAMGSMYSAVLFI
GVMNCTSVQPVVAVERTVFYRERAAGMYSAFPYAFGQVNGSPGNQFHTRLFFFVLLLFDPTNLSPVSAQVVIE
LPYALAQDILYGVIVYSMIGFEWTAAKFFWYLFFGYFTLLYFTFYGMMAVGLTPNYHIAAIVSSAFYAIWNLFS
GFIIPRPKVPIWWRWYCWICPVAWTLYGLVVSQFGDVMTPMDDGRAVKVFVEDYFDFKHSWLGWVAAVVV
AFAVLFATLFGFAIMKLNFQKR

>XP_024625156.1_PDR1_Mt
MMEGEASFRISSSSIWRNSDAAEIFSNSFRQEDDEEALKWAAIQKLPTFARLRKGLLSLLQGEATEIDVEKLGLQ
ERKDLLERLVRLAEEDNEKFLLKLKDRIDRVGIDLPTIEVRFEHLNIEAEANVGSRSLPTFTNFMVNIVLGLLNS
LHVLPSRKQHLNILREVSGIIKPSRITLLLGPPSSGKTTILLALAGKLDPKLKVSGKVTYNGHEMGEFVPQRTAA
YVDQNDLHIGEMTVRETLAFSARVQGVGPRYDLLAELSRREKHANIMPDPDIDVYMKAIATEGQKANLITDY
VLRILGLEICADTVVGNAMLRGISGGQKKRVTTGEMLVGPTKALFMDEISTGLDSSTTFQIVNSIKQYVHILKGT
AVISLLQPPPETFNLFDEIILLSDSHIIYQGPREHVLEFFESIGFKCPDRKGVADFLQEVTSRKDQEQYWEHKDQP
YRFITAEEFSEAFQSFHVGRRLGDELGTEFDKSKSHPAALTTKKYGVGKWELFKACLSREYLLMKRNSFVYIFK
IFQLCVMAMIAMTIFFRTEMHRDSLTHGGIYVGAIFYGVVTIMFNGMAEISMVVSRLPVFYKQRGYLFFPPWA
YALPEWILKIPLSFVEVAVWVFLTYYVIGFDPYIGRFFRQYLILVLVHQMASALFRFIAAVGRDMTVALTFGSF
ALAILFAMSGFVLSKDSIKKWWIWAFWISPMMYAQNAMVNNEFLGNKWKRVLPNSTEPIGVEVLKSHGFFSE
PYWYWIGVGALIGYTLIFNFGYILALTFLNPLGKHQTVIPEESQIRKRADVLKFIKDSFSQHSNRLRNGKSRSGSI
SPKTNHRRKRGMVLPFEPHSITFDEVSYSVDMPQEMRTRGVVENMLVLLKGLSGAFRPGVLTALMGVTGAGK
TTLMDVLSGRKTGGYIGGNITISGYPKKQDTFARISGYCEQTDIHSPYVTVYESLLYSAWLRLSPDINAETRKMF
IEEVMELVELKPLRNALVGLPGVSSLSTEQRKRLTIAVELVANPSIIFMDEPTSGLDARAAAIVMRTVRNTVDTG
RTVVCTIHQPSIDIFESFDELFLLKQGGQEIYVGPLGHNSSNLISYFEGIKGVSKIKYGYNPATWMLEVTTSSKER
ELGIDFAEVYKNSELYRRNKALIKELSTPAPCSKDLYFTSQYSRSFWTQCMACLWKQHWSYWRNPVYTAIRF
MYSTAVAVMLGTMFWNLGSKIEKVQDLFNAMGSMYSAVLLIGIKNGNAVQPVVSVERTVFYRERAAGMYSA
LPYAFAQVVIELPHVFVQSVVYGFIVYAMIGFEWTLVKFLWCLFFMYFTFLYFTFYGMMSVAMTPNNHISIIVS
SAFYSIWNLFSGFIVPRPRIPVWWRWYSWANPVAWSLYGLVTSQYGDVKQNIETSDGRQTVEDFLRNYFGFK
HDFLGVVALVNVAFPIVFALVFALSIKMFNFQRR

>XP_008656640.1_ABCG36_Zm
MDAAGDIQKVASMRRGDSGSMWRRGDDVFSRSSREEDDEEALRWAALEKLPTYDRIRRAIVPLGLGDEAPGS
KGLVDVDVLSLGPRERRALLERLVRVADEDNERFLLKLKDRIDRVGIDMPTIEVRFQNLEAEAEVRVGSSGLPT
VLNSVVNTVEEAANALHILPSRKQIMPILHDVSGIIKPRRLTLLLGPPGSGKTTLLLALAGRLDKDLKFSGKVTY
NGHEMTEFVPERTAAYISQHDLHIGEMTVRETLAFSARCQGVGSRLDMLTELSRREKAANIKPDADIDAFMKA
AALGGQDANVVTDYILKILGLDICADTMVGDEMLRGISGGQRKRVTTGEMLVGPARALFMDEISTGLDSSTTF
QIVNSLRQSIHILGGTAVISLLQPAPETYNLFDDIILLSDGQVVYQGPREEVVEFFESVGFRCPERKGVADFLQEV
TSKKDQKQYWARPDEPYRFVSVKELATAFKSSHTGRALANELAVPFDKSKSHPAALTTTRYGVSGKELLKANI
DREILLMKRNSFVYMFRTFQLMVMSIIAMTLFFRTKMKHDTVNDGGIYMGALFFGVLMIMFNGLSELALTVFK
LPVFFKQRDLLFFPAWSYTIPAWILKVPITFIEVGGYVFLTYYVIGFDPNVGRFFKQYLLLLAVNQMTAALFRFV
GGVSRNMIVANVFASFMLLVVMVLGGFILQRDKVKKWWIWGYWISPMMYAQNAISVNEMLGHSWDKILNS
TASNETLGVQVLKSRGVFPEAKWYWIGFGAMVGFTILFNALFTLALTYLKPYGNSRPSVSKEELKEKHANIKG
EVVDGNHLVSVNPVTDSAIMEDDSASTKKGMILPFVPLSVTFDNIKYSVDMPQEMKGQGVQEDRLELLKSISG
SFRPGVLTALMGVSGAGKTTLMDVLAGRKTGGYIEGDIRISGYPKKQETFARVSGYCEQNDIHSPQVTVYESL
LFSAWLRLPKDVDSNKRKIFIEEVMELVELKPLRNALVGLPGVNGLSTEQRKRLTIAVELVANPSIIFMDEPTSG
LDARAAAIVMRTVRNTVDTGRTVVCTIHQPSIDIFEAFDELFLMKRGGEEIYAGPLGHNSSELIKYFEEIQGVSKI
KDGYNPATWMLEVTTISQEQILGVDFSDIYKKSELYQRNKALIKELSQPAPGSTDLHFSSKYAQSFNTQCVACL
WKQNLSYWRNPPYNTVRFFFTGIIALLLGTIFWDLGSKVYTSQDLLNAMGSMYSAVLFIGVMNCTSVQPVVA
VERTVFYRERAAGMYSAFPYAFGQVVIELPYALAQDILYAVIVYSMIGFEWTVAKFFWYLFFGYFTLLYFTFY
GMMTVGLTPNYHIAAIVSAAFYAIWNLFSGFVIPRPKVPIWWRWYCWICPVAWTLYGLVVSQYGDIMTEMDD
KRTVKVFVEDYFDFKHSWLGWVAAVVVAFGVLFATLFAFAIMKLNFQKR

>XP_021312973.1_ABCG36_Sb
MDAAGDIQKVASMRRGGSGSVWRRGDDVFSRSSREEDDEEALRWAALEKLPTYDRVRRAIVPLDGDEAAGG
KGLVDVDVLSLGPRERRALLERLVRVADEDNERFLLKLKDRIDRVGIDMPTIEVRFQNLEAEAEVRVGSSGLPT
VLNSVVNTVEEAANALHILPSRKRIMPILHDVSGIIKPRRLTLLLGPPGSGKTSLLLALAGRLDKDLKFSGKVTY
NGHEMTEFVPERTAAYISQHDLHIGEMTVRETLAFSARCQGVGSGFDMLTELSRREKAANIKPDADIDAFMKA
YAMGGQDANVVTDYILKILGLEICADTMVGDEMLRGISGGQRKRVTTGEMLVGPARALFMDEISTGLDSSTTF
QIVNSLRQSIHILGGTAVISLLQPAPETYNLFDDIILLSDGQVVYQGPREEVPEFFESVGFRCPERKGVADFLQEV
TSKKDQKQYWVRPDEPYRFVSVKEFATAFKSFHTGRAIANELAVPFDKSKSHPAALTTTRYGVSGKELLKANI
DREILLMKRNSFVYTFRTFQLILNSIITMTLFFRTKMKHDTVNDGGLYMGAVFFGVVLIMFNGMSELSLTVFKL
PVFFKQRDLLFFPAWSYTLPSWIVKVPITFIEVGGYVFLTYYVIGFDPNVSRFFKQYLLLLAVNQMAAALFRFIS
GASRNMIVANVSASFMLLVVMVLGGFILQKDKIRKWWIWGYWISPMMYAQNAISVNEMLGHSWDKILNSTA
SNETLGVQSLKSRAVFTEAKWYWIGFGAMVGFTILFNALFTLALTYLKPYGNSRPSVSEEQLQEKHANIKGEV
LDANHLVSAFSHRSTDVNTETDLAIMEDDSASSKKGMILPFDPLSLTFDNIKYSVDMPQEMKAQGVQEDRLEL
LKGVSGSFRPGVLTALMGVSGAGKTTLMDVLAGRKTGGYIEGDIRISGYPKKQETFARVSGYCEQNDIHSPQV
TVYESLLFSAWLRLPKDVDSNKRKIFIEEVMELVELKPLRNALVGLPGVNGLSTEQRKRLTIAVELVANPSIIFM
DEPTSGLDARAAAIVMRTVRNTVDTGRTVVCTIHQPSIDIFEAFDELFLMKRGGEEIYAGPLGHHSSELINYFEA
IQGVSKIKDGYNPATWMLEVTTTSQEQILGLDFSDMYKKSELYQRNKALIKELSQPAPGSSDLHFPSKYAQSSIT
QCVACLWKQNMSYWRNPPYNTVRFFFTTIIALLLGTIFWDLGGKVSTQQDLMNAMGSMYSAVLFIGIMNCTS
VQPVVAVERTVFYRERAAGMYSAFPYAFGQVVIELPYALVQDILYGVIVYSMIGFEWTAAKFFWYLFFGYFTL
LYFTFYGMMTVGLTPNYHIASIVSSAFYALWNLFSGFIIPRPKTPIWWRWYCWICPVAWTLYGLVVSQFGDIM
TPMDDNRPVKVFVEDYFDFKHSWLGWVAAVVVAFTVLFATLFAFAIMKLNFQKR

>XP_004239865.1_PDR1-l_Sl
MMEGANLNNFRGSLRASMRGNSSNSIFSRSGRDEDDEEALKWAALEKLPTFDRMRKGLLFGKEGETISEVDTN
DIGHQERKNLLDRLVKVADEDNEKFLLKLKDRIETVGIDLPSIEVRYEHLNIAADAYVGSRALPTFINFMTNSVE
TFLNTIHILPSRKRQITILNDVSGMIKPSRLTLLLGPPSSGKTTLLLALAGKLDPTLKVKGNVTYNGHELHEFVPQ
KTAVYISQHDLHIGEMTVRETLEFSARCQGVGPRYEMLAELSRREKAANIKPDRDIDIYMKASVAKGQEANIV
TDYVLKILGLDICADTMVGDEMLRGISGGQKKRVTTGEMLVGPSKALFMDEISTGLDSSTTFSIVNSLRQSVQL
LKGTAVISLLQPAPETYNLFDDIILLSDAQIVYQGPREDVLDFFESMGFKCPERKGVADFLQEVTSKKDQQQYW
AKKDEPYRFITSKEFAEAYQSFHVGKKLADELKTPYDKTKSHPAALSTKKYGIGMKQLLKVCADREFLLMKR
NSFVFIFKFFQLMVMAFIMMSIFFRTEMPRNNMDDGGMYAGALFFVVVVIMFNGMAEINLTILKLPVYFKQRD
LLFYPSWAYALPTWILKIPITIVEVAIWTFLTYYVMGFDPNVSRLFKQFLLLVLVHQMASGLFRFIGAAGRTMG
VATTFGAFALVLQFALSGFVLSRNDVKKWWIWGYWISPLMYSVNSILVNEFDGKKWDRIAPNGAESLGHAVL
RSRGFFPDPYWYWIGVGALIGYIIIFNLGYSIGLAYLNPFGKPQAILSEDNETEQLIEGSETEGQDKKRGMVLPFE
PHSITFDNIVYSVDMPQEIKDQGSTEDRLVLLKGVSGAFRPGVLTALMGVSGAGKTTLMDVLAGRKTGGYIDG
DIKISGYPKKQATFARISGYCEQNDIHSPYITVYESLVYSAWLRLPQDVDKNKRKMFVEEVMELVELTPLRSAL
VGLPGVNGLSTEQRKRLTIAVELVANPSIIFMDEPTSGLDARAAAIVMRAVRNTVDTGRTVVCTIHQPSIDIFEA
FDELLLMKRGGQEIYVGPLGRYSCHLIKYFESLPGVSKIKEAYNPATWMLEVTAASQEMMLGVDFTDLYKKS
DLYKRNKALIAELSTPRPGTTDLHFETQFSQSFWTQCMACLWKQHLSYWRNPSYTAVRFIFTVILALVFGTLF
WDLGSRLSRSQDLFNAMGSMYAATLFLGVQNSSSAQPVVAVERTVFYRERAAGMYSALPYAFGQVIVEIPYV
FLQAVFYGIIVYAMIGFEWTVAKFFWYLFIMYFTLLYFTFYGMLTVAVSPNQNVASIIAAFFYALWNLFSGFIVP
RPRIPIWWRWYYWLCPVAWTLYGLVASQFGDLQTMLSDDENVEQFLGRYFGFEHDFLGVVAAVIVVWPAVF
AFLFAYAIKAFNFQKR

>XP_025887045.1_PDR1-l_Sl
MEPANLNNFRGSLRASMRADSSRSVFSRSARDEDDEEALKWAALEKLPTFDRMRKGLLFGKEGESATEVDTN
DIGHQERKNLLDRLVKVADEDNEKFLLKLKDRIQTVGIDLPSIEVRYEHLNIVADAYVGSRALPTFINFMTNFV
ETFLNTIHILPSRKRQITILKDVSGMIKPSRMTLLLGPPSSGKTTLLLALAGKLDPTLKVTGKVTYNGHELHEFVP
QKTAVYISQYDLHIGEMTVRETLEFSARCQGVGPRYEMLAELSRREKAANIKPDHDIDIYMKASVTKGQEANI
VTDYVLKILGLDVCADTMVGDEMLRGISGGQKKRVTTGEMLVGPSKALFMDEISTGLDSSTTFSIVNSLRQLV
QLLKGTAVISLLQPAPETYNLFDDIILLSDACIVYQGPREDVLDFFESMGFKCPERKGVADFLQEVTSKKDQQQ
YWAKKDKPYRFITSKEFAEAYQSFHVGKELADELTTPYDKTKSHPAALSTQKYGIGTKELLNVCAEREFLLMK
RNSFVYIFKLFQLMVMAFIMMTVFFRTEMPRDDMDDGGMYAGALFFVVVVIMFNGMAEINLTILKLPVYFKQ
RDLLFYPSWAYALPTWILKIPITFIEVGLWTFLTYYVMGFDPNVSRLFKQFLLLVLVHQMASGLFRFIGAAGRT

MGVATTFGAFALVLQFALSGFVLSRNDVKKWWIWGYWISPLMYSVNSILVNEFDGKKWDHIVPNGAEPLGH
AVVRSRGFFPDAYWYWIGVGALIGYIIIFNLCYSIGLAYLNPFGKPQAIISEDSENVRLIEESETDSQDKKRGMVL
PFEPHSITFDNVVYSVDMPQEIKDQGSTEDRLVLLKGVSGAFRPGVLTALMGVSGAGKTTLMDVLAGRKTGG
YIDGDIKISGYPKKQETFARISGYCEQNDIHSPYITVYESLVYSAWLRLPQDVDKNKRKMFVEEVMELVELTPL
RSALVGLPGVNGLSTEQRKRLTIAVELVANPSIIFMDEPTSGLDARAAAIVMRAVRNTVDTGRTVVCTIHQPSID
IFEAFDELFLMKRGGQEIYVGPLGRHSCHLIKYFESLPGVSKIKEAYNPATWMLEVTAASQEMMLGVDFTDLY
KKSDLYKRNKALISELSMPRPGTKDLHFETQFSQPFWTQCMACLWKQHLSYWRNPSYTAVRFIFTVILALVFG
TLFWDLGSRVSQSQDLFNAMGSMYAATLFLGVQNSSSVQPVVAVERTVFYRERAAGMYSALPYAFGQVIVEI
PYVFVQAAFYGIIVYAMIGFEWTVAKFFWYLFIMYFTLLYFTFYGMMTVAISPNQNVASIVAAFFYAVWNLFS
GFIVPRPRIPIWWRWYYWLCPVAWTLYGLVASQFGDLQTMISNDENVEQFLGRYFGFEHDFLGVVAAVIVVW
PAVFAFLFAYAIKAFNFQKR

>XP_019069398.1_PDR1-l_Sl
MMEPANLNNFRGSMRGSLRADSSNSIFSRSARDEDDEEALKWAALEKLPTFDRMRKGLLFGKEGEAAAEVDT
NDIGHQERKNLLDRLVKVADEDNEKFLLKLKNRIETVGIDLPSIEVRYEHVNIDADAYVGSRALPTFINFMTNF
VESFLNSIHILPSRKRQITILKHVSGMIKPSRMTLLLGPPSSGKTTLLLALAGKLDSTLKVTGNVTYNGHELHEFV
PQKTAVYISQYDLHIGEMTVRETLEFSARCQGVGPRYEMLAELSRREKAANIKPDHDVDIYMKASVTKGQEA
NVVTDYVLKILGLDVCADTMVGDEMLRGISGGQKKRVTTGEMLVGPSKALFMDEISTGLDSSTTFSIVNSLRQ
SVQLLNGTAVISLLQPAPETYNLFDDIILLSDGRIVYQGPREAVLDFFESMGFKCPERKGVADFLQEVTSKKDQ
QQYWAKRDEAYRFITSKEFAEAYESFHVGKKLADELATPYDKTKSHPAALSTQKYGLGTKEMLKVCAEREFL
LMKRNSFVYIFKLFQLVVMALIMMTVFFRTEMPRDNMDDGGMYAGALFFVVVVIMFNGMAEINLTILKLPVY
FKQRDLLFYPSWAYALPTWILKIPITFIEVGLWTFLTYYVMGFDPNVSRLFKQFLLLVLVHQMASGLFRFIGAA
GRTMGVATTFGAFALVLQFALSGFVLSRNDVKKWWIWGYWISPLMYSVNSILVNEFDGKKWDHIAPNGAEPL
GHAVVRSRGFFPDAYWYWVGVVALIGFIIIFNLCYSVGLAYLNPFGKQVMISEDDENDRLIEGSETEGEKKKG
MVLPFEPHSITFDNVVYSVDMPQEIKDQGSTEDRLVLLKGVSGAFRPGVLTALMGVSGAGKTTLMDVLAGRK
TGGYIDGDIKISGYPKKQETFARISGYCEQNDIHSPYITVYESLVYSAWLRLPQDVDKNKRKMFVEEVMELVEL
TPLRSALVGLPGVNGLSTEQRKRLTIAVELVANPSIIFMDEPTSGLDARAAAIVMRAVRNTVDTGRTVVCTIHQ
PSIDIFEAFDELFLMKRGGQEIYVGPLGRYSCHLIKYFESLPGVSKIKEAYNPATWMLEVTAASQEMMLGVDFT
DLYKKSDLYKRNKALIAELSTPRPGTKDLHFETQFSQSFWTQCMACLWKQHLSYWRNPSYTAVRFIFTVILAL
VFGTLFWDLGSRLSRSQDLFNAMGSMYAATLFLGVQNSSSAQPVVAVERTVFYRERAAGMYSALPYAFGQVI
VEIPYVFLQAVFYGIIVYAMIGFEWTVAKFFWYLFIMYFTLLYFTFYGMLTVAVSPNQNVASIIAAFFYALWNL
FSGFIVPRPRIPIWWRWYYWLCPVAWTLYGLVASQFGDLQTMLSDDENVEQFLGRYFGFEHDFLGVVAAIIAA
WPVVFAFLFAFAIKAFNFQKR

>XP_006365806.1_P_PDR1-l_St
MEPTNSRGTSLREKIIRGSSLRGSLSSKRNSTNNSRWSGNDGEIFNRSTRDEDDEEALKWAALEKLPTFDRLRK
GLLLGSQGASAEIDIHDIGFQERNKLLERLVKVADEDNEKLLLKLRQRIDRVGIDLPEIEVRYEHLTIEADAYIGS
RALPTFINFITNFLEDILNPLHILPSRKRKLTILNDVSGIIKPRRLTLLLGPPSSGKTTLLLALAGKLDSALKVTGKV
TYNGHEMNEFVPQRTAAYISQYDLHIGEMTVRETLEFSARCQGVGSSYEMLVELTRREKEAKIKPDPDIDIFMK
ALAAEGQEANFVTEYVLKLLGLDICADTMVGDEMIRGISGGQKKRVTTGEMLVGPSKALFMDEISTGLDSSTT
YSIVNSLRQSVQILHGTAVISLLQPAPETYNLFDDIILLSDGKIVYQGPREDVLGFFESMGFKCPDRKGVADFLQ
EVTSKKDQQQYWVRRDETYRFITSKEFAEAYQSFHVGRKLVDDLAASYDKSKSHPAALSTQKYGIGKKQLLK
VCTEREFLLMKRNSFVYIFKFIQLTIMALISMTLFFRTKMPRDTIEDGVKYVGALFFVVTMIMFNGMAEIALTIY
KLPVFYKQRDLLFYPSWAYAMPTWILKIPITFVEVGLWVFLTYYVIGFDPSPARFFKHFLLLILVNQMASGLFRF
IGATGRTMGVANTFGTFVLLLQFALGGFVLSRDDVKKWWLWGYWSSPMMYSMNSILVNEFGGKRWKQIAPI
GTDSLGVTVVRSRGFFTNAYWYWIGVGALIGFTIVFNICYSLALAYLNPFGKPQGMISEDSDDAKTTSTEKEVS
TSEGQNKKKGMVLPFEPHSITFDEVTYSVDMPQEMKNQGVTEDRLVLLNGVCGAFRPGVLTALMGVSGAGK
TTLLDVLAGRKTGGYIEGSIKISGYPKKQETFARISGYCEQNDIHSPYVTVYESLVYSAWLRLPSDVDEKTRKM
FVDEVMELVELTPLRSALVGLPGVNGLSTEQRKRLTIAVELVANPSIIFMDEPTSGLDARAAAIVMRTVRNTVD
TGRTVVCTIHQPSIDIFEAFDELFLMKRGGKEIYVGPLGHHSCHLIRYFESIPGVSKIQDGYNPATWMLEVTNSA
QEMMLGVDFTDLYKKSDLYRRNKILIRELSVPGPGTKDLHFNNQYSQPFWTQCMACLWKQHWSYWRNPAYT
AVRYIFTIIIALAIGTMFWDLGTKVSKSQDLFNAMGSMYAPVLFLGFQNASSVMPVVAVERTVFYRERAAGMY
SSLPYAFGQAFIEIPYVFVQAVTYGVIIYAMIGFEWTVTKFFWYLFIMYFTLLYFTFYGLMSVAVSPNQNIAQIV
SLFGYAMWNLFSGFMIPRPSMPIWWRWYYWACPVSWTLYGLVASQFGDLQDKLTDSDETAKHFLRRYFGFK

HDFLGVVAFVTVAYAVVFAFTFALAIKVFNFQKR

>XP_002458139.2_ABCG36_Sb
MDVTGELQKVASMRGGSGSMWRRGDDVFSRSSREEDDEEALRWAALEKLPTYDRVRRAIVPLDLGADGAEA
AGGKGLVDVDVLSLGPRERRALLERLVRVADEDNERFLLKLKDRVDRVGIDMPTIEVRFQNLEAEAEVRVGSS
GLPTVLNSIVNTVEEAANALHILPSSKRIMPILHDVSGIIKPRRLTLLLGPPGSGKTTLLLALAGRLDKDLKFSGK
VTYNGHEMTEFVPERTAAYISQHDLHIGEMTVRETLAFSARCQGVGSRFDMLTELSRREKAANIKPDADIDAF
MKASAMGGQDANVVTDYILKILGLEICADTMVGDEMLRGISGGQRKRVTTGEMLVGPSRALFMDEISTGLDS
STTFQIVNSLRQSIHILGGTAVISLLQPAPETYNLFDDIILLSDGQVVYQGPREEVLEFFESVGFRCPERKGVADFL
QEVTSKKDQKQYWARLDAPYRFVSVKEFATAFKSFHTGRAIANELAVPFDKSKGHPAALTTTRYGVSGKELL
KANIDREILLMKRNSFVYIFRTFQLVLMSIIVMTLFFRTKMKHDSVTDGGIYLGAVFFGVLMIMFNGFSELALTV
FKLPVFFKQRDLLFFPAWSYTIPSWILKIPISFIEVGGYVFLTYYVIGFDPNVGRFFKQYLLLLAVNQMAAALFRF
IGGASRNMIVANVFASFMLLVVMVMGGFILVRDKIKKWWIWGYWISPMMYAQNAISVNEMLGHSWDKILNS
AASNETLGLQSLKSRGVFTEPKWYWIGFGALVGFTLLFNALFTLALTYLKPYGNSRPSVSEEELQEKHANIKGG
NHLVSASSHQSTGLNTETDSAIMEDDSASTKKGMILPFDPLSLTFDNIKYSVDMPQEMKAQGVQEDRLELLKG
VSGSFRPGVLTALMGVSGAGKTTLMDVLAGRKTGGYIEGDICISGYPKKQETFARVSGYCEQNDIHSPQVTVY
ESLLFSAWLRLPKDVDSNTRKIFIEEVMELVELKPLRNALVGLPGVNGLSTEQRKRLTIAVELVANPSIIFMDEP
TSGLDARAAAIVMRTVRNTVDTGRTVVCTIHQPSIDIFEAFDELFLMKRGGEEIYAGPLGHHSSDLIKYFEGIQG
VSKIKDGYNPATWMLEVTTTSQEQILGVDFSDIYKKSELYQRNKALIKELSHPVPGSSDLHFASTYAQSSITQCV
ACLWKQNLSYWRNPPYNTVRFFFTTIIALLLGTIFWDLGGKVSTSQDLMNALGSMYAAVIFIGVMNCTSVQPV
VAVERTVFYRERAAGMYSAFPYAFGQVVIELPYALVQDILYGVIVYAMIGFEWTAAKFFWYLFFGYFTLLYFT
FYGMMAVGLTPNYHIASIVSSAFYAIWNLFSGFIIPRPKTPIWWRWYCWICPVAWTLYGLVVSQFGDIMTEMD
DNNRTVVVSQYVEDYFGFKHSWLGWVAAVVVAFAVLFAALFGFAIMKFNFQKR

>XP_024625542.1_PDR1_X4_Mt
MEGGGSFRIGNSSIWRNSDAAEIFSNSFHQEDDEESLKWAAIQKLPTFERLRKGLLTSLQGEATEVDVEKLGLQ
ERKDLLERLVRLAEEDNEKFLLKLKDRMDRVGIDLPTIEVRFERLNINAEARVGSRSLPTFTNFMVNIVEGMLN
SLHVLPSRKQHLNILKDVSGIIKPSRMTLLLGPPSSGKTTLLLALAGKLDQKLKVSGKVTYNGHEMSEFVPQRT
AAYVDQNDLHIGEMTVRETLAFSARVQGVGPRYDLLAELSRREKDANIKPDPDIDVYMKAVATEGQKENLIT
DYVLRVLGLEICADTVVGNAMIRAISGGQKKRLTTGEMLVGPTKALFMDEISTGLDSSTTFQIVNSMRQYVHIL
KGTVVISLLQPPPETYNLFDDIILLSDSHIIYQGPREHVLEFFESIGFKCPNRKGVADFLQEVTSRKDQEQYWEHK
DRPYRFITAEEFSEAFQTFHVGRRLGDELGTEFDKSKSHPAALTTKKYGVGKIELLKACSSREYLLMKRNSFVY
IFKLCQLAIMAMIAMTIFLRTEMHRDSVAHGDIYVGALFYGCIVILFIGVAELSMVVSRLPVFYKQRGYLFFPPW
AYALPAWILKIPLTFVEVAVWVILTYYVIGFDPYIGRFFRQYLILVLVHQMATALFRFIAAVGRDMTVTLTLGSF
ALAILFSMSGFVLTKGSTKKWWIWGFWISPLMYGQNAVVINEFLGNKWKHVLPNSTGSLGVEVLKSRSFFTET
YWYWICVGALIGYTLLFNFGYILALTFLNPLGKHKTVIREDSFSQHSNRLRNGESRSGSISPSTLSDRQETVGVE
TNHRRKRGMVLPFEPHSITFDEVSYSVDMPQEMRNRGVIEDKLVLLKGLSGAFRPGVLTALMGVTGAGKTTL
MDVLSGRKTGGYIGGNITISGYPKKQETFARISGYCEQTDIHSPHVTVYESLLYSAWLRLSPDINAETRKMFIEE
VMELVELKPLRYALVGLPGVSGLSTEQRKRLTVAVELVANPSIIFMDEPTSGLDARAAAIVMRAVRNTVDTGR
TVVCTIHQPSIDIFESFDELLLLKQGGQEIYVGPLGHNSSNLINYFEGVQGVSKIKDGYNPATWMLEVTTSSKEV
ELRIDYAEVYKNSELYRRNKALIKELSAPAPCSKDLYFPSRYSRSFFTQCIACLWKQHWSYWRNPEYNAIRFLY
STAVAVLLGSMFWNLGSKIEKDQDLFNAMGSMYAAVILIGAMNSNSVQPVVGVERTVFYRERAARMYSAFP
YALAQVVIELPYVFVQAVVYGIVVYVMIGFEWTLVKVVWCLFFMYFTFLYFTFYGMMSVAMTPNNHISIIVSS
AFYSVWNLFSGFVVPRPSIPVWWRWYSWANPVAWSLYGLVTSQYGDVKQNIETSDGRQTVEDFLRNYFGFK
HDFLGVVALVNIAFPIVFALVFAIAIKMFNFQRR

>XP_015648337.1_ABCG37_X2_Os
MDREVHRMASLRREGSMWRSGGDVFSRSSSRFQDEDDDEEALRWAALERLPTYDRVRRGILAVSSEDGGAG
GEKVEVDVGRLGARESRALIERLVRAADDDHERFLLKLRERMDRVGIDYPTIEVRFENLEVEADVHVGNRGLP
TLLNSVTNTVEAIGNALHILPNKKQPMTVLHDVSGIIKPRRMTLLLGPPGSGKTTLLLALAGKLDKDLKVSGKV
TYNGHGMHEFVPERTAAYISQHDLHIGEMTVRETLAFSARCQGVGTRYEMLTELARREKAANIKPDHDIDIYM
KASAMGGQESSVVTDYILKILGLDICADTVVGNEMLRGISGGQRKRVTTGEMLVGPARALFMDEISTGLDSST
TYQIVNSLRQTIHILGGTAVISLLQPAPETYNLFDDIILLSDGQVVYQGPREHVLEFFEFMGFRCPARKGVADFL
QEVTSRKDQGQYWCRRDRPYRFVPVKQFADAFRSFHVGRSIQNELSEPFDRTRSHPAALATSKYGVSRKELLK

ATIDRELLLMKRNAFMYIFKAVNLTLMALIVMTTFFRTSMRHDRDYGMIYLGALYFALDTVMFNGFAELAMT
VMKLPVFFKQRDLLFFPAWAYTIPSWILQIPITFLEVGVYVFITYYVIGFDPSVSRFFKQYLLLLALNQMSSALFR
FIAGIGRDMVVSHTFGPLSLLAFAALGGFILARPDVKKWWIWGYWISPLSYAQNAISTNEFLGHSWSQILPGEN
VTLGVSVLKSRGIFTEAKWYWIGLGALLGYTLLFNLLYTVALSVLSPFTDSHASMSEDALKEKHANLTGEVVE
GQKDTKSRKQELELSHIADQNSGINSADSSASRKGMVLPFAPLSISFNDVRYSVDMPEAMKAQGITEDRLLLLK
GVSGSFRPGVLTALMGVSGAGKTTLMDVLAGRKTGGYIEGDIRISGYPKKQETFARISGYCEQNDIHSPHVTVY
ESLVFSAWLRLPSEVDSEARKMFIEEVMDLVELTSLRGALVGLPGVSGLSTEQRKRLTIAVELVANPSIIFMDEP
TSGLDARAAAIVMRTVRNTVNTGRTVVCTIHQPSIDIFEAFDELFLMKRGGEEIYVGPVGQNSSKLIEYFEGIDG
VSRIKDGYNPATWMLEVTSSAQEEMLGVDFSEIYRQSELYQRNKELIEELSTPPPGSTDLNFPTQYSRSFITQCL
ACLWKQNWSYWRNPSYTAVRLLFTIVIALMFGTMFWNLGTRTKKQQDLFNAMGSMYAAVLYIGVQNSGSV
QPVVVVERTVFYRERAAGMYSAFPYAFGQVAIELPYIMVQTLIYGVLVYSMIGFEWTVAKFLWYLFFMYFTLL
YFTFYGMMAVGLTPNESIAAIISSAFYNVWNLFSGYLIPRPNRFKFLYRKSLFGGGGTVGFARLHGLCMD

>XP_015648322.1_ABCG36_X1_Os
MDAAGEIQKVASMRLGGSMRGDSGSMWRRGDDVFSRSSREEDDEEALRWAALEKLPTYDRVRRAILPLGGD
DGAGDGGGKGVVDVHGLGPRERRALLERLVRVADEDNEKFLLKLKDRVDRVGIDMPTIEVRFEHLEAEAEVR
VGNSGLPTVLNSITNTLEEAGNALGILPNRKQTMPVLHDVSGIIKPRRMTLLLGPPGSGKTTLLLALAGRLGKD
LKASGKVTYNGHGMEEFVPERTAAYISQHDLHIGEMTVRETLAFSARCQGVGSRFDMLTELSRREKAANIKPD
ADIDAFMKAAAMGGQEANVNTDYILKILGLEICADTMVGDEMLRGISGGQRKRVTTGEMLVGPARALFMDEI
STGLDSSTTFQIVNSLRQTVHILGGTAVISLLQPAPETYNLFDDIILLSDGQIVYQGPREDVLEFFESMGFKCPDR
KGVADFLQEVTSKKDQRQYWARHDKPYRFVTVKEFVSAFQSFHTGRAIANELAVPFDKSKSHPAALATTRYG
APGKELLKANIDREILLMKRNSFVYMFRTFQLMVVSLIAMTLFFRTKMKRDSVTSGGIYMGALFFGVLMIMFN
GFSELALTVFKLPVFFKQRDLLFYPAWSYTIPSWILKIPITFIEVGGYVFLTYYVIGFDSNVGSFFKQYLLMLAIN
QMAGSLFRFIGGAARNMIVANVFASFMLLIFMVLGGFILAREQVKKWWIWGYWISPMMYAQNAISVNELMG
HSWNKIVNSSASNETLGVQVLKSRGVFPEARWYWIGFGAMIGFTILFNALFTLALTYLRPYGNSRQSVSEEELK
EKRANLNGEIVGDVHLSSGSTRRPMGNGTENDSTIVDDDTEVTQRGMVLPFTPLSLSFDNVRYSVDMPQEMK
AQGVADDRLELLKGVSGSFRPGVLTALMGVSGAGKTTLMDVLAGRKTGGYIEGSINISGYPKKQETFARVSG
YCEQNDIHSPQVTVYESLLFSAWLRLPEDVDSNTRKMFIEEVMELVELKSLRDALVGLPGVNGLSTEQRKRLTI
AVELVANPSIIFMDEPTSGLDARAAAIVMRTVRNTVNTGRTVVCTIHQPSIDIFEAFDELFLMKRGGEEIYAGPL
GHHSSELIKYFESIPGVSKIKDGYNPATWMLEVTTIGQEQALGVDFSDIYKKSELYQRNKALIKDLSQPAPDSSD
LYFPTQYSQSSLTQCMACLWKQNLSYWRNPPYNAVRFFFTTVIALLFGTIFWDLGGKVTKSQDLFNAMGSMY
AAVLFIGVMNCTSVQPVVAVERTVFYRERAAGMYSAFPYAFGQVVIEIPYTLVQATVYGIIVYAMIGFEWTAA
KFFWYLFFMVFTLLYFTFYGMMAVGLTPNYHIASIVSSAFYAIWNLFSGFVIPRPRVPIWWRWYCWACPVAW
TLYGLVVSQFGDIETPMEDGTPVKVFVENYFGFKHSWLGWVATVVAAFAFLFASLFGFAIMKFNFQKR

>XP_015648329.1_ABCG37_X1_Os
MDREVHRMASLRREGSMWRSGGDVFSRSSSRFQDEDDDEEALRWAALERLPTYDRVRRGILAVSSEDGGAG
GEKVEVDVGRLGARESRALIERLVRAADDDHERFLLKLRERMDRVGIDYPTIEVRFENLEVEADVHVGNRGLP
TLLNSVTNTVEAIGNALHILPNKKQPMTVLHDVSGIIKPRRMTLLLGPPGSGKTTLLLALAGKLDKDLKVSGKV
TYNGHGMHEFVPERTAAYISQHDLHIGEMTVRETLAFSARCQGVGTRYEMLTELARREKAANIKPDHDIDIYM
KASAMGGQESSVVTDYILKILGLDICADTVVGNEMLRGISGGQRKRVTTGEMLVGPARALFMDEISTGLDSST
TYQIVNSLRQTIHILGGTAVISLLQPAPETYNLFDDIILLSDGQVVYQGPREHVLEFFEFMGFRCPARKGVADFL
QEVTSRKDQGQYWCRRDRPYRFVPVKQFADAFRSFHVGRSIQNELSEPFDRTRSHPAALATSKYGVSRKELLK
ATIDRELLLMKRNAFMYIFKAVNLTLMALIVMTTFFRTSMRHDRDYGMIYLGALYFALDTVMFNGFAELAMT
VMKLPVFFKQRDLLFFPAWAYTIPSWILQIPITFLEVGVYVFITYYVIGFDPSVSRFFKQYLLLLALNQMSSALFR
FIAGIGRDMVVSHTFGPLSLLAFAALGGFILARPDVKKWWIWGYWISPLSYAQNAISTNEFLGHSWSQILPGEN
VTLGVSVLKSRGIFTEAKWYWIGLGALLGYTLLFNLLYTVALSVLSPFTDSHASMSEDALKEKHANLTGEVVE
GQKDTKSRKQELELSHIADQNSGINSADSSASRKGMVLPFAPLSISFNDVRYSVDMPEAMKAQGITEDRLLLLK
GVSGSFRPGVLTALMGVSGAGKTTLMDVLAGRKTGGYIEGDIRISGYPKKQETFARISGYCEQNDIHSPHVTVY
ESLVFSAWLRLPSEVDSEARKMFIEEVMDLVELTSLRGALVGLPGVSGLSTEQRKRLTIAVELVANPSIIFMDEP
TSGLDARAAAIVMRTVRNTVNTGRTVVCTIHQPSIDIFEAFDELFLMKRGGEEIYVGPVGQNSSKLIEYFEGIDG
VSRIKDGYNPATWMLEVTSSAQEEMLGVDFSEIYRQSELYQRNKELIEELSTPPPGSTDLNFPTQYSRSFITQCL
ACLWKQNWSYWRNPSYTAVRLLFTIVIALMFGTMFWNLGTRTKKQQDLFNAMGSMYAAVLYIGVQNSGSV
QPVVVVERTVFYRERAAGMYSAFPYAFGQVAIELPYIMVQTLIYGVLVYSMIGFEWTVAKFLWYLFFMYFTLL

YFTFYGMMAVGLTPNESIAAIISSAFYNVWNLFSGYLIPRPKIPVWWRWYCWICPVAWTLYGLVASQFGDIQH
VLEGDTRTVAQFVTDYFGFHHNFLWVVAVVHVVFAVTFAFLFSFAIMKFNFQRR

>XP_003625401.2_PDR1_Mt
MEGGGSFRISSSSIWRSSDAAEIFSNSFHQEDDEEALKWAAIQNLPTFARLRKGLLTSLQGEAVEIDIEKLGLQER
KDLLERLVRLAEEDNEKFLLKLKDRMDRVGVDLPTIEVRFEHLNIEAEARVGSRSLPTFTNFMVNIVEGLLNSL
HVLPSRKQHLNILRDVSGILKPSRMTLLLGPPSSGKTTLLLALAGKLDPKLKFSGRVTYNGHEMSEFVPQRTAA
YVDQNDLHIGEMTVRETLAFSARVQGVGPRYDLLAELSRREKDANIKPDPDIDVYMKAVATEGQKANLITDYI
LRVLGLEICADTIVGNAMLRGISGGQKKRLTTGEMLVGPTKALFMDEISTGLDSSTTFQIVNSMRQDVHILNGT
AIISLLQPPPETYNLFDDVILLSDSRIIYQGPREHVLEFFESIGFKCPDRKGVADFLQEVTSRKDQEQYWDHKDQP
YRFVTAEEFSEAFQSFHVGRRLGDELGTEFDKSKSHPAALTTKKYGVGKWELYKACSSREYLLMKRNAFVYIF
KLCQLAVMAMIAMTLFLRTEMHRDSVTHGGIYVGALFYGVVVIMFNGMAELSMVVSRLPVFYKQRGYLFFP
AWAYALPGWILKIPLIFAEVAVWVFLTYYVIGFDPYIERFFRQYLILVLVHQMATALFRFIAAVGRDMTVALTF
GSFAIAILFAMSGFVLSKDSIKNGWIWGFWISPMMYGQNAMVNNEFLGNKWKHVLPNSTEPLGVEVLKSRGF
FTESYWYWIGVGALIGYTLLFNFGYMLALTFLNPLGKHQTVIPDDSQSSEKIGGSRERSNVLRFIKDGFSQITNK
VRNGESRSGSISPIRQEIVASETNHSRKRGMVLPFEPHSITFDEVTYSVDMPQEMRRNLGVVEDKLVLLKGVSG
AFRPGVLTALMGVTGAGKTTLMDVLSGRKTGGYIGGNITISGFPKKQETFARISGYCEQNDIHSPYVTVYESLL
YSAWLRLSPDINAETRKMFVEEVMELVELKPLQNALVGLPGVNGLSTEQRKRLTIAVELVANPSIIFMDEPTSG
LDARAAAIVMRTVRNTVNTGRTVVCTIHQPSIDIFESFDELLLLKQGGQEIYVGPLGHNSSNLINYFEGIHGVSKI
KDGYNPATWMLEVTTSSKERELGIDFAEVYQNSELYRRNKALIKELSTPAPCSKDLYFASQYSRSFWTQCMAC
LWKQHWSYWRNPEYNAIRFLYSTAVAVLFGSMFWDLGSKIEKEQDLFNAMGSMYSAVIVIGIKNANSVQPVV
AVERTVFYRERAAGMYSAFPYAFAQVVIELPYVFVQAVVYGIIVYAMIGFEWSVVKFLWCLFFLFCTFLYFTY
YGLMSVAMTPNNHISIIVSSAFYSIWNLFSGFIVPRPNIPVWWRWYSWANPIAWSLYGLVVSQYGDEKHNIETS
DGRQTVEGFLKNYFDFKHDFLGVVALVNVAFPIGFALVFAISIKMFNFQRR

>XP_006360348.1_P_PDR1-l_X1_St
MEPSDLSNLRGRSIRGSMRGSMRENSNSIWRNNGVEVFSRSNRDEDDEEALKWAALEKLPTYDRLRKGILFGS
QGVAAEVDVDDLGVQQRKNLLERLVKVADEDNEKFLLKLKNRIDRVGIDFPSIEVRFEHLNIEADAYVGSRAL
PTFTNFISNFIESLLDSIHIFPSKKRSVTILKDVSGYVKPCRMTLLLGPPGSGKTTLLLALAGKLDSDLRVTGKVT
YNGHELHEFVPERTAAYISQHDLHIGEMTVRETLEFSARCQGVGSRYEMLAELSRREKAANIKPDVDIDMFMK
AVSTEGQESKVITDYVLKILGLDICADTMVGDQMIRGISGGQKKRVTTGEMIVGPSKALFMDEISTGLDSSTTY
SIVNSLKQSVQILKGTALISLLQPAPETYNLFDDIILLSDGYIVYQGPREDVLEFFESMGFKCPDRKGVADFLQEV
TSKKDQQQYWVRRDEPYRFITSKEFAEAYQSFHVGRKVSNELSTAFDKSKSHPAALTTEKYGIGKKQLLKVCT
EREFLLMQRNSFVYIFKFFQLMVIALMTMTIFFRTEMPRDTETDGGIYTGALFFTVVMLMFNGLSELPLTLYKL
PVFYKQRDFLFYPSWAYAIPSWILKIPVTLLEVGMWTVLTYYVIGFDPNVGRFFKQFLLLVLVNQMASGLFRFI
AAVGRTMGVASTFGACALLLQFALGGFALARTDVKDWWIWGYWTSPLMFSVNAILVNEFDGEKWKHTAPN
GTEPLGPSVVRSRGFFPDAYWYWIGIGALAGFTILFNIAYSLALAYLNPFGKPQATISEEGENNESSGSSPQITST
AEGDSVGENQNKKKGMVLPFEPQSITFDEVVYSVDMPPEMREQGSSDNRLVLLKGVSGAFRPGVLTALMGVS
GAGKTTLMDVLAGRKTGGYIDGSIKISGYPKKQETFARISGYCEQNDIHSPYVTVYESLVYSAWLRLPQDVDE
HKRMMFVEEVMDLVELTPLRSALVGLPGVNGLSTEQRKRLTIAVELVANPSIIFMDEPTSGLDARAAAIVMRA
VRNTVDTGRTVVCTIHQPSIDIFEAFDELFLMKRGGQEIYVGPLGRESCHLIKYFESMPGVGKIEEGYNPATWM
LEVTSSSQEMSLGVDFTDLYKNSDLCRRNKALITELSVPRPGTSDLHFENQFSQPFWVQCMACLWKQRWSYW
RNPAYTAVRFLFTTFIALIFGSMFWDLGTKVSRPQDLTNAMGSMYAAVLFLGVQNASSVQPVVSVERTVFYRE
KAAGMYSAIPYAFAQVFIEIPYVFVQSVVYGLIVYSMIGFEWTVAKFFWYFFFMFFTFLYFTFFGMMTVAITPN
QNVASIVAGFFYTVWNLFSGFIVPRPRIPIWWRWYYWGCPVAWTLYGLVASQFGDLQDIVNGQTVEEYLRND
YGIKHDFLGVVAGVIVAFAVVFAFTFALGIKAFNFQKR

>XP_003625399.2_PDR1_X1_Mt
MEGGGSFRIGSSSIWRNSDAAEIFSNSFHQEDDEEALKWAAIQKLPTFERLRKGLLTSLQGEATEIDVENLGLQE
RKDLLERLVRLAEEDNEKFLLKLKDRIDRVGIDLPTIEVRFEGLNIEAEAHVGNRSLPTFTNFMVNIVEGLLNSL
HVLPSRKQHLNILKDVSGILKPSRMTLLLGPPSSGKTTLLLALAGKLDPKLKFSGKVTYNGHEMNEFVPQRTAA
YVDQNDLHIGEMTVRETLAFSARVQGVGPRYDLLAELSRREKHANIMPDPDIDVYMKAIATEGQKANLITDY
VLRILGLEICADTVVGNAMLRGISGGQKKRVTTGEMLVGPAKALFMDEISTGLDSSTTFQIVNSMKQFVHILKG
TAVISLLQPPPETYNLFDDIILLSDSHIIYQGPREHVLEFFESIGFKCPDRKGVADFLQEVTSRKDQEQYWEHKDQ

PYRFVTAEEFSEAFQSFHVGRRLGDELGTEFDKSKSHPAALTTKKYGVGKWELFKACLSREYLLMKRNSFVYI
FKICQICIMAMIAMTIFFRTEMHRDSVTLGGIYVGALFYGVVVIMFNGMAEISMVVSRLPVFYKQRGYLFFPPW
AYALPAWILKIPLTFVEVAVWVFLTYYVIGFDPYIGRFFRQYLILVLVNQMASALFRFIAAVGRDMTVALTFGS
FALSILFAMSGFVLSKDRIKKWWIWGFWISPMMYGQNAMVNNEFLGNKWKHVLPNSTDPIGVEVLKSRGYFT
ESYWYWIGVGALIGYTLLFNFGYILALTFLNPLGKHQTVIPDESQSDGQIGGGRKRTNVLKFIKDSFSQHSNKV
RNGEIRSGSTSPSTSSDRQERVAAETNHSRKRGMVLPFEPHSITFDEVTYSVDMPQEMRNRGVVEDKLVLLKG
VSGAFRPGVLTALMGVTGAGKTTLMDVLSGRKTGGYIGGNITISGYPKKQDTFARISGYCEQTDIHSPHVTVYE
SLLYSAWLRLSPDINAETRKMFIEEVMELVELKPLQNAIVGLPGVSGLSTEQRKRLTIAVELVANPSIIFMDEPTS
GLDARAAAIVMRTVRNTVDTGRTVVCTIHQPSIDIFESFDELLLLKQGGKEIYVGSLGHNSSNLISYFEGIHGVN
KIKEGYNPATWMLEITNSSKEVDLGIDFAEVYKNSDLYRRNKTLIEELSTPASGSKDLYFTSQYSRSFWTQCMA
CLWKQHWSYWRNPVYTAIRFLYSTSVAVLLGTMFWNLGSNIEKEQDLFNAMGSMYSAVLLIGIKNSNAVQPV
VAVERTVFYRERAAGMYSAFPYAFAQVVIELPHVFVQSVVYGFIVYAMIGFEWSVVKVLWYLFFMYFTFLYF
TFYGMMAVAMTPNNHISTIVSSAFYSVWNLFSGFIVPRPRIPVWWRWYSWANPVAWSLYGLVASQYGDLKQ
NIETSDRSQTVKDFLRNYFGFKHDFLGMVALVNVAFPIAFALVFAIAIKMFNFQRR

>XP_004252847.1_PDR1_Sl
MELTNSRGTSLREKIIRGSSLRGSLSRKRNSTNNSRWNGNDGEIFNRSTRDEDDEEALKWAALEKLPTFDRLRK
GLLFGSQGASAEIDIHDIGFQERNKLLERLVKVADEDNEKLLLKLKQRIDRVGIDLPEIEVRYEHLTIEADAYVG
SRALPTFINFISNFFEDILNSVHILPSRKRKLTILNDVSGIIKPRRLTLLLGPPSSGKTTLLLALAGKLDSALKVTGK
VTYNGHEMNEFVPQRTAAYISQYDLHIGEMTVRETLEFSARCQGVGSSYELLVELSRREKAAKIKPDPDIDIFM
KALATEGQEAVFVTDYVLKLLGLDICADTMVGDEMIRGISGGQKKRVTTGEMLVGPSKALFMDEISTGLDSST
TYSIVNSLRQSVQILHGTAVISLLQPAPETYNLFDDIILLSDEKIVYQGPREDVLGFFESMGFKCPDRKGVADFLQ
EVTSKKDQQQYWVRRDETYRFITSKEFAEAHQSFHVGRKLADKLAASYDKSKSHPAALSTQKYGIGKKQLLK
VCTERELLLMKRNSFVYIFKFIQLTIVALISMTLFFRTKMPRDTIEDGVKYVGALFLVVTQIMFNGMAEIALTIY
KLPVFYKQRDLLFYPSWAYAVPTWILKMPITFAEVGLWVFLTYYVIGFDPSAARFFKQFLLLISLNQMASALFR
FIGAAGRTMGVANTFGTFVLLLQFALGGFVLSRVDVKKWWLWGYWSSPMMYAMNSILVNEFDGKKWKQIA
PNGTDSLGVTVVRSRGFFTNAYWYWIGVGAQIGFTIVFNICYSIALAYLNPFGKPQGMISEDSNDAKTTSTEKE
VSTSEGQNKKKGMVLPFEPHSITFDEVTYSVDMPQEMKNQGVTEDRLVLLNGVSGAFRPGVLTALMGVSGAG
KTTLMDVLAGRKTGGYIEGSIKVSGYPKKQETFARISGYCEQNDIHSPYVTVYESLVYSAWLRLPSDVGEKTR
KMFVDEVMELVELTPLRSALVGLPGVNGLSTEQRKRLTIAVELVANPSIIFMDEPTSGLDARAAAIVMRTVRNT
VDTGRTVVCTIHQPSIDIFEAFDELFLMKRGGNEIYVGPLGHHSCHLIRYFESIPGVSKIRDGYNPATWMLEVTN
SAQEMMLVLDFTDLYKKSDLYRRNKILISELSVPRPGTKDLHFKNQYSQTFWTQCLACLWKQHWSYWRNPT
YTAVRYIFTVIIALAIGTMFWDLGTKVSKSQDLFNAMGSMYAPVLFLGFQNASSVMPVVAVERTVFYRERAA
GMYSSLPYAFGQTFIEIPYVFVQAVTYAVIIYAMIGFEWTVSKFFWYLFIMYFTFLYFTFYGMMSVAVSPNQNI
AQIVSLFGYSMWNLFSGFMIPRPSMPIWWRWYYWADPVAWTLYGLVVSQFGDLQDKITDIDETSKQFLRRYF
GFKHDFLGVVAAVTVAYAVVFAFTFGLAIKFFNFQKR

>XP_024625540.1_PDR1_X1_Mt
MEVGGSFRIGSSSIWRNSDAAEIFSNSFHQGDDEEALKWAAIQILPTFERLRKGLLTSLQGGTIEIDVENLGMQE
KKDLLERLVRLAEEDNEKFLLKLKDRIDRVGIDLPTIEVRFEHLNIEAEARVGSRSLPTFTNFMVNIVERIFNSLL
VLPSRKQHLNILKDVSGIIKPSRMTLLLGPPSSGKTTLLLALAGKLDQKLKFSGRVTYNGHEMSEFVPQRTAAY
VDQNDLHIGELTVRETLAFSARVQGVGPQYDLLAELSRREKDANIKPDPDIDVYMKAVATEGQKANLITDYVL
RVLGLEICADTVVGNAMIRGISGGQKKRLTTGEMLVGPTKALFMDEISTGLDSSTTFQIVNSMKQYVHILKGTA
VISLLQPPPETYNLFDSIILLSDSHIIYQGPREHVLEFFESIGFKCPNRKGVADFLQEVTSVKDQEQFWEHKDQPY
KFVTAEEFSEAFQTFHVGRRLGDELGTEFDKSKSHPAALTTKKYGVGKIELLKACSSREYLLMKRNSFVYIFKL
CQLAVMAMITMTVFLRTEMRKDSVVHGGIYVGALFFGVTVIMFIGMAELSMVVSRLPVFYKQRGCLFFPPWA
YSLPSWILKIPLTCVEVAVWVFLTYYVIGFDPYIGRFFRQYLILVLVHQMAAALFRFIAAVGRDMTVALTFGSF
AIAILFSMSGFVLSKDGIKKWWIWAFWISPLMYGQNAMVNNEFLGNKWKHVLPNSTESLGVEVLKSRSFFTET
YWYWICVGALIGYTLLFNFGYILALTFLNPLGKHQAVIPDESQSNEQIGGSQKRTNALKFIKDGFSKLSNKVKK
GESRRGSISPSRQEIVAAATNHSRKKGMVLPFEPHSITFDEVTYSVDMPQEMRNRGVLEDKLVLLKGVSGAFRP
GVLTALMGITGAGKTTLMDVLSGRKTGGYIGGNIKISGFPKKQETFARISGYCEQTDIHSPHVTVYESLLYSAW
LRLSPDINAETRKMFIEEVMELVELKPLQNAIVGLPGVSGLSTEQRKRLTVAVELVANPSIIFMDEPTSGLDARA
AAIVMRTVRNTVDTGRTVVCTIHQPSIDIFESFDELLLLKQGGQEIYVGPLGHNSSNLINHFEGIQGVSKIKDGY
NPATWMLEVTNSSKEVELGIDFVELYKNSELYRINKALIKELGSPAPCSKDLYFPTQYSRSFFTQCMACLWKQH

WSYWRNPEYNAIRFLYSTAVAVLLGSMFWDLSSKIEKEQDLFNAMGSMYAAVILIGVMNGNSVQPVVAVERT
VFYRERAAGMYSAFPYAFGQVVIELPYVFVQAVVYGIVVYVMIGFEWTLVKVVWCLFFMYFTFLYFTFYGM
MSVAMTPNNHISIIVSSAFYSVWNLFSGFVVPRPSIPVWWRWYSWANPVAWSLYGLVTSQYGDVKQNIETSD
GRQTVEDFLRNYFGFKHDFLGVVALVNIAFPIVFALVFAIAIKMFNFQRR

>XP_015648358.2_ABCG36_X2_Os
MDAAGEIQKVASMRLGGSMRGDSGSMWRRGDDVFSRSSREEDDEEALRWAALEKLPTYDRVRRAILPLGGD
DGAGDGGGKGVVDVHGLGPRERRALLERLVRVADEDNEKFLLKLKDRVDRVGIDMPTIEVRFEHLEVEAEVR
VGNSGIPTVLNSITNKIEEAANALGILPTRKQTLRILHDISGIIKPKRMTLLLGPPGSGKTTFLLALAGRLKDLKFS
GQVTYNGHQMEDFVPQRTAAYISQHDLHIGEMTVRETLSFSARCQGVGSRFDMLTELTRREKAANIKPDADV
DAFMKASAMEGQESNLITDYILKILGLEICADTMVGDDMVRGISGGQRKRVTTGEMLVGPANAFFMDEISTGL
DSSTTFQIVKSLRQTIHILGGTAVISLLQPAPETYDLFDDIILLSDGHIVYQGPRENVLEFFELMGFKCPERKGVA
DFLQEVTSRKDQKQYWAQHDKPYRYVPIKEFASAFQSFHTGRSIANELATPFDKSKSHPAALTTSRYGVSAME
LLKANIDRELLLIKRNSFVYIFRTIQLMTVSAMAMTVFFRTKMHRDSVADGVIFMGALFFAVMMIMLNGLSEL
PLTIFKLPVFFKQRDLLFFPAWTYTIPSWILKSPMSFIEVGGFCFMSYYVIGFDPNVGRFFKQYLLMLAVSQMAA
ALFRFVGGAARNLIVANVFGSFMLLIFMVLGGFILARDKVNKWWIWGYWISPMMYAQNAVSVNEFLGHSWD
KVLNNSLSNETLGVQALMSRGIFPEAKWYWIGFGALLGFIMLFNILFTLALTYLKPDGKSQPSISEEELKEKQAN
INGNVLDVDTMASSNNLAIVGSTGTGSEIADNSQPTQRGMVLPFTPLSLTFEDIKYSVDMPQEMKAHGIVEDRL
ELLKGVSGCFRPGVLTALMGVSGAGKTTLMDVLAGRKTGGYIEGNISISGYPKKQETFARVSGYCEQNDIHSP
QVTVSESLLFSAWLRLPKDVDSNTRKMFIEEVMELVELKPLRDALVGLPGVNGLSIEQRKRLTIAVELVANPSII
FMDEPTSGLDARAAAIVMRTVRNTVNTGRTVVCTIHQPSIDIFEAFDELFLMKRGGEEIYVGPLGHHSSELIKYF
EGIQGVSKITDGYNPATWMLEVTTVSQEQALDVDFCDIYRKSELFQRNKALIQELSTPPPGSSELYFPTQYSQSF
LIQCLACLWKQHLSYWRNPPYNAIRLFFTTVIALIFGTIFWDLGGKMGQSQDLFNAMGSMYAAVLFIGVLNGQ
SVQPVVSVERTVFYRERAAGMYSALPYAFGQVAIEFPYTLVQSVIYSIIVYSMIGFQWTVAKFFWYLFFMFFTL
LYFTFYGMMAVGLTPSYHVASIVSSAFYAIWNLFTGFVISRPATPVWWRWYCWICPVAWTLYGLIVSQYGDIV
TPMDDGIPVNVFVENYFDFKHSWLGFVAVVIVAFTMLFAFLFGFAIMKLNFQKR

>XP_002458135.1_ABCG35_Sb
MDAAAELQKVASMRRDSGGSRSGSSAWWRAPDAFSRSSSRMEEDDEEALRWAALERLPTCDRVRRAILPLG
GNGDGHGHGGGDAATQVVDVLGLGPRERRALLERLVRVADEDNERFLLKLKERVERVGIDMPTIEVRFKHLR
AEADVRVGTSGLPTVLNSITNKLEEVANALHVRRSRKQAMPILHDVSGIVKPRRMTLLLGPPGSGKTTLLLALA
GRLDKDLKVSGKVTYNGHEMDEFVPERTAAYISQHDLHIGEMTVRETLEFSARCQGVGTRFDMLTELSRREK
VGNIKPDADIDAFMKACAMRGQEANVISDYILKILGLEICADTMVGDDMLRGISGGQRKRVTTGEMLVGPAN
ALFMDEISTGLDSSTTFQIIKSLRQAIHILGGTALISLLQPAPETYDLFDDIILLSDGQIVYQGPRESVLEFFLSLGF
KCPERKGVADFLQEVTSRKDQKQYWVWHDKPYRYVSVKEFATAFQCFHVGRAIANELAIPFDKSKNHPAALT
TSKYGVSAWELFKANIDREMLLMKRNSFVYIFRTLQLMTVSIIAMTLFFRTKMHRDSVTDGGIYLGALFFAVIM
IMFNGLSELALTIIKLPVFFKQRDLLFFPAWAYTIPTWILKIPISFVEVGGFVFMAYYVIGIDPNVGRFFKQYLLLL
ALNQMAASLFRFVGGAARNMIVANVFGSFMLLIFMVLGGFILVRDKVKKWWIWGYWISPLMYAQNAISVNE
MLGHSWDKILNSSVSYETLGVQSLKSRGVFPEAKWYWIGLGALLGFVMLFNCLFTLALAYLKPYGKSHPSISE
EELNEKYANLNGNVVAEDNLPPGSSYLAAVDITRSDSATIENHSGTMQRGMVLPFAPLSLTFSNIKYFVDMPQE
MKTHDVVGDRLELLKCVSGSFRPGVLTALMGVSGAGKTTLMDVLAGRKTSGYIEGNISISGYPKKQETFARVS
GYCEQNDIHSPQVTVYESLVFSAWLRLPSDVDLNTRKMFIEEVMELVELKPLRNALVGLPGVNGLSTEQRKRL
TIAVELVANPSIIFMDEPTSGLDARAAAIVMRTVRNTVDTGRTVVCTIHQPSIDIFEAFDELFLMKRGGEEIYVGP
LGHHSSELIKYFEGIHGVKKIKDGYNPATWMLEVTTISQEEILGVDFSDLYKKSELYQRNKALIQELSEPSVGST
DLHFRNQYSQSFFMQCLACLWKQNLSYWRNPAYNAVRLFFTTIIALIFGTIFWDLGGKMGQSQDLFNAMGSM
YAAVMFIGVLNATSVQPVVSVERTVFYRERAAGMYSALPYAFGQVTIELPYTLTQATVYGIIVYSMIGFEWTV
AKFFWYLFFMYFTFLYFTFYGMMAVGLTPSYHVASIVSSAFYGIWNLFSGFIIPRPKVPIWWKWYCWACPVA
WTLYGLVVSQFGDITMPMDNGVPVNVFVENYFGFKHSWLGVVAAVVMAFTIFFASLFGFAIMKLNFQRR

>XP_015626248.1_ABCG39_Os
MDIVRMGSVASGGGSVRRTASSWRGTSGRSDAFGRSVREEDDEEALKWAAIEKLPTYDRMRKGILTAGGVEE
VDIGGLGLQERRNLIERLVRTAEEDNERFLLKLRDRMERVGIDNPTIEVRFENLSIDAEAYVGNRGIPTFTNFFS
NKIMDVLSAMRIVSSGKRPISILHDISGIIRPGRMSLLLGPPGSGKTSLLLALAGKLDSTLKVSGRVTYNGHDMD
EFVPQRTSAYIGQHDLHIGEMTVRETLAFSARCQGVGTRYDMLTELSRREKEASIKPDPDIDVYMKAISVEGQE

SVVTDYILKILGLEICADTMVGDAMIRGISGGQKKRVTTGEMLVGPAKALFMDEISTGLDSSTTYQIVNSLRQS
VHILGGTALIALLQPAPETYDLFDDIVLLSEGQIVYQGPRENILEFFEAMGFKCPERKGVADFLQEVTSRKDQHQ
YWCRRDEPYRYISVNDFSEAFKEFHVGRNLGSELRVPFDRTRNHPAALTTSRYGISKMELTKACFSREWLLMK
RNSFVYIFKILQLIILGSIGMTVFLRTKMHRRSVEDGAIFLGAMFLGLVTHLFNGFAELAMSIAKLPIFYKQRDLL
FYPSWAYALPTWVLKIPISFLECAVWICMTYYVMGFDPNIERFFRHYVLLVLISQMASGLFRLLAALGREMVV
ADTFGSFAQLILLVLGGFLISRENIKKWWIWGYWSSPLMYAQNAIAVNEFLGHSWNKVVDPTQSNDTLGVQV
LKVRGIFVDANWYWIGVGALLGYIMLFNILFILFLEWLDPLGKGQAVVSEEELREKHVNRTGENVELLTLGTD
SQNSPSDGRGEITGADTRKRGMVLPFTPLSITFDNIRYSVDMPQEMKDKGVTEDRLLLLKGVSGAFRPGVLTAL
MGVSGAGKTTLMDVLAGRKTGGYIEGDISISGYPKKQETFARIAGYCEQNDIHSPHVTVYESLLYSAWLRLPSE
VDSEARKMFVEEVMELVELTSLRGALVGLPGVNGLSTEQRKRLTIAVELVANPSIIFMDEPTSGLDARAAAIVM
RTVRNTVDTGRTVVCTIHQPSIDIFEAFDELFLMKRGGEEIYVGPLGHNSCHLINYFEGIQGVRKIKDGYNPATW
MLEVTTLAQEDILGINFAEVYRNSDLYQRNKTLISELSTPPPGSTDLHFPTQFSQPFFTQCMACLWKQHKSYWR
NPSYTATRIFFTTVIALIFGTIFLNLGKKINKRLDLFNSLGSMYAAVLFIGIQNGQTVQPIVDVERTVFYREKAAG
MYSALPYAFAQVLIEIPHIFLQTVVYGLIVYSLIGFDWTVEKFFWYMFFMFFTFMYFTFYGMMAVAMTPNSDI
AAIVSTAFYCIWNIFAGFLIPRPRIPIWWRWYSWACPVAWTLYGLVASQYGDITNSTLEDGEVVQDYIRRYFGF
RHDYLGYVATAVVGFAALFAFVFAFSIKVFNFQRR

>XP_002451754.1_ABCG39_Sb
MDLVRMGSIASGSMRRTASSWRASGRSDAFGRSVREEDDEEALRWAAIEKLPTYDRMRKGILTGAGAGFEEV
DIQGLGMEERKNLIERLVRTAEEDNERFLLKLRDRMERVGIDNPTIEVRFEHLNIDAEAYVGNRGIPTMTNFFS
NKIMDALSAMHIVASGKRPISILHDISGVIRPGRMSLLLGPPGSGKTSLLLALSGKLDSTLKVSGRVTYNGHDM
DEFVPQRTSAYIGQHDIHVGEMTVRETLSFSARCQGVGTRYDMLTELSRREKEANIQPDPDIDVYMKAISVEGQ
ESVVTDYILKILGLEVCADTMVGDSMIRGISGGQKKRVTTGEMLVGPAKALFMDEISTGLDSSTTYQIVNSLRQ
SVHILGGTALIALLQPAPETYELFDDIVLLSEGQIVYQGPRENVLEFFEAMGFKCPERKGVADFLQEVTSRKDQ
HQYWCRRDERYRYISVNDFSEAFKAFHVGRKLGTELKEPFDRTRNHPAALTTSKYGISKMELLKACFSREWLL
MKRNSFVYIFKVVQLIILGTIAMTVFLRTTMHRRGVEDGVIFLGAMFLGLVTHLFNGFAELAMSIAKLPIFYKQ
RDLLFYPSWAYALPTWLLKIPISFLECAVWIGMTYYVIGFDPNIERFFRHYLLLVLISQMASGLFRLLAAVGRE
MVVADTFGSFAQLVLLILGGFLIARDNIKKYWIWGYWSSPLMYAQNAIAVNEFLGHSWQKVVDSTHSNDTLG
VQILKARGIFVDPNWYWIGVGALLGYIMLFNVLFILFLDWLGPLGQGQAVVSEEELREKHVNRTGENVELLAL
GTSSQNSPSDGRGEIAGAETRNRGMALPFTPLSITFDNVKYSVDMPQEMKDKGITEDRLLLLKGVSGAFRPGVL
TALMGVSGAGKTTLMDVLAGRKTGGYIEGDISISGYPKKQETFARIAGYCEQNDIHSPHVTVYESLLYSAWLR
LPHEVDSEARKMFVEQVMELVELTPLRGALVGLPGVNGLSTEQRKRLTIAVELVANPSIIFMDEPTSGLDARAA
AIVMRAVRNTVDTGRTVVCTIHQPSIDIFEAFDELFLMKRGGEEIYVGPLGRNSCHLIDYFEGIEGVKKIKDGYN
PATWMLEVTTLSQEDILGINFAEVYRNSDLYRRNKALISELSIPPPGSRDLYFPTQYSQSFLTQCMACLWKQHK
SYWRNPSYTATRIFFTTVIALIFGTIFLNLGKKIGTRQDLLYALGSMYAAVLFIGIQNGQTVQPIVDVERTVFYRE
KAAGMYSALPYAFAQVLIEIPHIFLQTVVYGLIVYSLIGFEWTAEKFLWYMFFMFFTFMYFTFYGMMAVAMTP
NSDIAAIVSTAFYAIWNIFAGFLIPRPRIPIWWRWYSWACPVAWTLYGLVASQFGDITDVRLEDDEIVKDFVNR
FFGFYHDDLAYVATAVVGFTVLFAFVFAFSIKVFNFQRR

>XP_004247842.1_PDR1_Sl
MEPLDLSNLRGRSIRGSMRENSNSIWRNNGVEVFSRSNRDEDDEEALKWAALEKLPTYDRLRKGILFGSQGVT
AQVDVDDLGVSQRKSLLERLVKVADEDNEKFLLKLKNRIDRVGIDFPSIEVRFEHLNIEADAYVGSRALPTFTN
FISNFIESLLDSIHITPSKKRSVTILKDVSGYVKPCRMTLLLGPPGSGKTTLLLALAGKLDSDLRVTGKVTYNGHE
LHEFVPQRTAAYISQHDLHIGEMTVRETLEFSARCQGVGSRYEMLAELSRREKAANIKPDVDIDMFMKAISTEG
QESKVITDYILKILGLDICADTMVGDQMIRGISGGQKKRVTTGEMIVGPSKALFMDEISTGLDSSTTYSIVNSLK
QSVQILKGTALISLLQPAPETYNLFDDIILLSDGYIVYQGPREDVLEFFESMGFKCPDRKGVADFLQEVTSKKDQ
QQYWVRRDEPYRFITSKEFAEAYQSFHVGRKVSNELSTAFDKSKSHPAALTTEKYGIGKKQLLKVCTEREFLL
MQRNSFVYIFKFFQLMVIALMTMTIFFRTEMPRDTATDGGIYAGALFFTVVMLMFNGLSELPLALYKLPVFYK
QRDFLFYPSWAYAIPSWILKIPVTFLEVGMWTFLTYYVIGFDPNVGRFFKQFLLLVLVNQMASGLFRFIAAVGR
TMGVASTFGACALLLQFALGGFALARTDVKDWWIWGYWTSPLMYSVNAILVNEFDGEKWKHTAPNGTEPLG
PSVVRSRGFFPDAYWYWIGIGALAGFTILFNIAYSLALVYLNPFGKPQATISEEGENNESSGSSSQITSTTEGDSV
DENQNKKKGMVLPFEPHSITFDEVVYSVDMPPEMREQGSSDRLVLLKGVSGAFRPGVLTALMGVSGAGKTTL
MDVLAGRKTGGYIDGSIKISGYPKKQETFARISGYCEQNDIHSPYVTVHESLVYSAWLRLPQDVDEHKRMMFV
EEVMDLVELTPLRSALVGLPGVNGLSTEQRKRLTIAVELVANPSIIFMDEPTSGLDARAAAIVMRAVRNTVDTG

RTVVCTIHQPSIDIFEAFDELFLMKRGGQEIYVGPLGRESCHLIKYFESMPGVGKIEEGYNPATWMLEVTSSSQE
MSLGVDFTELYKNSDLCRRNKALITELSVPRPGTSDLHFENQFSQPFWVQCMACLWKQHWSYWRNPAYTAV
RFLFTTFIALMFGSMFWDLGTKVSRPQDLTNAMGSMYAAVLFLGVQNASSVQPVVSVERTVFYREKAAGMY
SAIPYAFAQVFIEIPYVFVQAVVYGLIVYSMIGFEWTVAKFFWYFFFMFFTFLYFTFFGMMTVAITPNQNVASIV
AGFFYTVWNLFSGFIVPRPRIPIWWRWYYWACPVAWTLYGLIASQFADLQDIVNGQTVEEYLRNDYGIKHDFL
GVVAGVIVAFAVVFAFTFALGIKAFNFQRR

>XP_008679861.1_ABCG39_Zm
MDLVQMGSIAGGSMRRTASSWRASGRSDAFGRSVREEDDEEALRWAAIEKLPTYDRMRKGILTGNAAGAGV
EEVDIQGLGMQERKNLIERLVRTAEEDNERFLLKLRDRMELVGIDNPTIEVRFENLNIDAEAYVGNRGVPTMT
NFFSNKVMDVLSAMHIVSSGKRPVSILHDISGVIRPGRMSLLLGPPGSGKTSLLLALSGKLDSNLKVSGRVTYN
GHDMDEFVPQRTSAYIGQHDVHVGEMTVRETLAFSARCQGVGTRYDMLTELSRREKEANIKPDPDVDVYMK
AISVEGQESVVTDYILKILGLEICADTMVGDSMIRGISGGQKKRVTTGEMLVGPAKALFMDEISTGLDSSTTYQI
VNSLRQSVHILGGTALIALLQPAPETYELFDDIVLLSEGQIVYQGPRENVLEFFEVMGFKCPERKGVADFLQEVT
SRKDQHQYWCRRDEPYRYISVNDFSEAFKAFHVGRKLGSDLKVPFDRTRNHPAALTTSKYGISKMELLRACFS
REWLLMKRNSFVYIFKVVQLIILGTIAMTVFLRTTMHRRGVEDGVIFLGAMFLGLVTHLFNGFAELAMSIAKLP
IFYKQRDLLFYPSWAYASPTWLLKIPISFLECAVWIGMTYYVIGFDPSIERFFRHYLLLVLVSQMASGLFRLLAA
LGREMVVADTFGSFAQLVLLILGGFLIARDNIKKWWIWGYWSSPLMYAQNAVAVNEFLGHSWQMVVDRTHS
NDTLGVQILKARGIFVDPNWYWIGVGALLGYIMLFNVLFVLFLDWLGPLGKGQAVVSEEELREKHVNRTGQN
VELLPLGTASQNPPSDGRGEIAGAESRKRGMVLPFTPLSITFDNIKYSVDMPQEMKDKGITEDRLLLLKGVSGA
FRPGVLTALMGVSGAGKTTLMDVLAGRKTGGHIEGDISISGYPKKQETFARIAGYCEQNDIHSPHVTVYESLLY
SAWLRLPHEVDSEARKMFVEEVMELVELTPLRGALVGLPGVNGLSTEQRKRLTIAVELVANPSIIFMDEPTSGL
DARAAAIVMRTVRNTVDTGRTVVCTIHQPSIDIFEAFDELFLMKRGGEEIYVGPLGRNSCHLINYFEGIEGVKKI
KDGYNPATWMLEVTTLAQEDILGINFAEVYRNSDLYRRNKDLISELSTPPPGSKDLYFPTQYSQSFLTQCMACL
WKQHKSYWRNPSYTATRIFFTTVIALIFGTIFLNLGKKIGTRQDLFNSLGSMYAAVLFIGIQNGQTVQPIVDVER
TVFYREKAAGMYSALPYAFAQVLIEIPHIFLQTVVYGLIVYSLIGFDWTVAKFFWYMFFMFFTFMYFTFYGMM
AVAMTPNSDIAAIVSTAFYAIWNIFAGFLIPRPRIPIWWRWYSWACPVAWTLYGLVASQFGDIADIRLEDDGEL
VKDFVNRFFGFEHDNLGYVATAVVGFTVLFAFVFAFSIKVFNFQRR

>XP_002451753.1_ABCG39_Sb
MDLVRMGSIASGSMRRTASSWRGSGRSDAFGRSVREEDDEEALRWAAIEKLPTYDRMRKGILTGAGAGGGIE
EVDIQGLGMQERQNLIERLVRTAEEDNERFLLKLRDRMERVGIDNPTIEVRFENLNIDAEAYVGNRGVPTMTN
FFSNKVMDALSAMHIVSSGKRPISILHDISGIIRPGRMSLLLGPPGSGKTSLLLALAGKLDSALKVSGRVTYNGH
DMDEFVPQRTSAYIGQHDVHVGEMTVRETLAFSARCQGVGTRYDMLTELSRREKEANIKPDPDIDVYMKAIS
VEGQESVVTDYILKILGLEICADTMVGDSMIRGISGGQKKRVTTGEMLVGPAKALFMDEISTGLDSSTTYQIVN
SLRQSVHILGGTALIALLQPAPETYELFDDIVLLSEGQIVYQGPRENVLEFFEAMGFKCPERKGVADFLQEVTSR
KDQHQYWCRRDERYRYISVNDFSEAFKAFHVGRKLGSELMEPFDRTRNHPAALTTSKYGISKMELLRACFSRE
WLLMKRNSFVYIFKVVQLIILGTIAMTVFLRTTMHRRSVEDGVIFLGAMFLGLVTHLFNGFAELAMSIAKLPIFY
KQRDLLFYPSWAYALPTWLLKIPISFLECAVWIGMTYYVIGFDPNIERFFRHYLLLVLISQMASGLFRLLAALGR
EMVVADTFGSFAQLVLLILGGFLIARDNIKKYWIWGYWSSPLMYAQNAIAVNEFLGHSWQKVVDSTQSNDTL
GVEILKARGIFVDPNWYWIGVGALLGYIMLFNVLFVLFLDWLGPLGQGQAVVSEEELREKHVNRTGENVELLP
LGTASQNSPSDGRGEIAGAETRKRGMVLPFMPLSITFDNVKYSVDMPQEMKDKGITEDRLLLLKGVSGAFRPG
VLTALMGVSGAGKTTLMDVLAGRKTGGYIEGDISISGYPKKQETFARIAGYCEQNDIHSPHVTVYESLLYSAW
LRLPHEVDSEARKMFVEEVMELVELTPLRGALVGLPGVNGLSTEQRKRLTIAVELVANPSIIFMDEPTSGLDAR
AAAIVMRTVRNTVDTGRTVACTIHQPSIDIFEAFDELFLMKRGGEEIYVGPLGRNSCHLIDYFEGIEGVKKIKDG
YNPATWMLEVTTLAQEDILGINFAEVYRNSDLYRRNKALISELSTPPPGSKDLYFPTQYSQSFLTQCMACLWK
QHMSYWRNPSYTATRIFFTTVIALIFGTIFLNLGKKIGTRQDLLYALGSMYAAVLFIGIQNGQTVQPIVDVERTV
FYREKAAGMYSALPYAFAQVLIEIPHIFLQTVVYGLIVYSLIGFEWTAEKFFWYMFFMFFTFMYFTFYGMMAV
AMTPNSDIAAIVSTAFYAIWNIFAGFLIPRPRIPIWWRWYSWACPVAWTLYGLVASQFGDITDVRLEDDEIVKD
FVNRFFGFQHDNLGYVATAVVGFTVLFAFVFAFSIKVFNFQRR

>XP_015650488.1_ABCG44_Os
MDTGEAAFGVASLRLRGSMASASSRRAPSYRDYDVFSIASSSRAEAEDDEEALKWAALEKLPTHARVRKGIVA
AADDGQGSGAAGEVVDVAGLGFQERKHLLERLVRVAEEDHESFLLKLKQRIDRVGLDFPTIEVRYEHLSIDAL

AHVGSRGLPTFLNTTLNSLESLANLLHVVPNKKRPLNILHDVHGVIKPRRMTLLLGPPGSGKTTLLLALAGKLG
SDLKVSGKVTYNGYGMDEFVAQRSAAYISQHDLHIPEMTVRETLAFSARCQGVGTRYDMLTELARREKAANI
KPDPDLDVYMKAISVGGQETNIITDYVLKILGLDICADTIVGNEMLRGISGGQRKRVTTGEMIVGPARAMFMD
EISTGLDSSTTFQIVKSLGQITSILGGTTVISLLQPAPETYNLFDDIILLSDGHIVYQGPREHVLEFFESMGFKCPDR
KGVADFLQEVTSRKDQQQYWARTHQPYRYIPVQEFACAFQSFHVGQTLSDELSHPFDKSTSHPASLTTSTYGA
SKLELLRTCIARELLLMKRNMFVYRFRAFQLLVITIIVMTLFLRTNMHHETRTDGIVYLGALFFAMVAHMFNGF
SELAMATIKLPVFFKQRDYLFFPSWAYTIPTWILKIPISCFEVAITVFLSYYVIGFDPNVGRLFKQYLLLLLVNQM
AAALFRFIAALGRTMVVANTLASFALLVLLVLSGFILSHHDVKKWWIWGYWISPLQYAMNAIAVNEFLGHKW
NRLVQGTNTTLGIEVLKSRGMFTEAKWYWIGVGALFGYVIVFNILFTIALGYLKPSGKAQQILSEEALKEKHAN
ITGETINDPRNSASSGQTTNTRRNAAPGEASENRRGMVLPFAPLAVAFNNIRYSVDMPPEMKAQGVDQDRLLL
LKGVSGSFRPGVLTALMGVSGAGKTTLMDVLAGRKTGGYIEGDISISGYPKKQETFARVSGYCEQNDIHSPNV
TVYESLAYSAWLRLPSDVDSETRKMFIEQVMELVELNPLRDALVGLPGVNGLSTEQRKRLTIAVELVANPSIIF
MDEPTSGLDARAAAIVMRTVRNTVDTGRTVVCTIHQPSIDIFEAFDELFLMKRGGEEIYVGPLGHHSCDLIEYFE
GVEGVSKIKPGYNPATWMLEVTTLAQEDVLGISFTDVYKNSDLYQRNQSLIKGISRPPQGSKDLFFPTQFSQSFS
TQCMACLWKQNLSYWRNPPYTVVRFFFSLIVALMFGTIFWRLGSKRSRQQDLFNAMGSMYAAVLFMGISYSS
SVQPVVAVERTVFYRERAAGMYSALPYAFGQVVVELPYVLVQSAVYGVIVYAMIGFEWEAKKFFWYLYFMY
FTLLYFTFYGMLAVGLTPSYNIASIVSSFFYGIWNLFSGFVIPRPSMPVWWRWYSWACPVSWTLYGLVASQFG
DLKEPLRDTGVPIDVFLREYFGFKHDFLGVVAVAVAGFATLFAVSFSLSIKMLNFQRR

>XP_008656641.1_ABCG37_Zm
MDREIHRVTSLRRDSSLWRRGDDVFSRQSSRFQDEEEDDEEALRWAALERLPTYDRVRRGILALHDAGGEKV
EVDVGRLGARESRALVERLVRAADDDHERFLLKLKERMDRVGIDYPTIEVRYENLHVEAQVHVGDRGLPTLI
NSVTNTIESIGNALHVLPSRKQPMTVLHDVSGIVKPRRMTLLLGPPGSGKTTLLLALAGKLDKDLRVSGKVTY
NGHGMNEFVPERTAAYISQHDLHIGEMTVRETLAFSARCQGVGTRYEMLTELSRREKAANIKPDHDIDIYMKA
SAMGGQESSIVTDYILKILGLEVCADTVVGNEMMRGISGGQRKRVTTGEMLVGPARALFMDEISTGLDSSTTY
QIVNSLRQTIHILGGTAVISLLQPAPETYNLFDDIILLSDGHVVYQGPREHVLEFFEFMGFRCPARKGVADFLQE
VTSRKDQGQYWCRQDRPYRFVPVKKFADAFSTFHVGRSIQNELSEPFDRTRSHPAALATSKFGASRMELLKAT
IDRELLLMKRNAFMYIFKAVNLTVMSFIVMTTFFRTNMKRDASYGSIYMGALFFALDTIMFNGFAELAMTVM
KLPVFFKQRDLLFFPAWAYTIPSWILQIPITFLEVGVYVFTTYYVIGFDPSVIRFFKQYLLLLALNQMSSALFRFIA
GIGRDMVVSHTFGPLALLAFQTLGGFILARPDVKKWWIWGYWISPLSYAQNAISTNEFLGHSWSKIENGTTVGI
RVLRSRGVFTEAKWYWIGLGALVGYALLFNLLYTVALAVLSPFTDSHGSMSEEELKEKHANLTGEVAEGHKE
KKSRRQELELSHSHSVGQNLVHSSEDSSQNRKGMALPFPPLSLTFNDIRYSVDMPEAMKAQGVAEDRLLLLKG
VSGSFRPGVLTALMGVSGAGKTTLMDVLAGRKTGGYIEGDITISGYPKKQETFARISGYCEQNDIHSPHVTVYE
SLLFSAWLRLPSDVNLETRKMFIEEVMDLVELTSLRGALVGLPGVSGLSTEQRKRLTIAVELVANPSIVFMDEP
TSGLDARAAAIVMRTVRNTVNTGRTVVCTIHQPSIDIFEAFDELFLMKRGGEEIYVGPVGQNSSRLIEYFEGIEGI
SNIKDGYNPATWMLEVTSSSQEEILGVDFSEIYRRSELYQRNKALIEELSAPPPGSSDLNFATQYSRSFFTQCLAC
LWKQKKSYWRNPSYTAVRLLFTIVIALMFGTMFWDLGRKTKKQQDLFNAMGSMYAAVIYIGVQNSGSVQPV
VVVERTVFYRERAAGMYSAFPYAFGQVAIEFPYISVQTLIYGVLVYSMIGFEWTAAKFLWYLFFMYFTLLYFT
FYGMMAVGLTPNESIAAIISSAFYNVWNLFSGYLIPRPKMPVWWRWYSWACPVAWTLYGLVASQFGDITEPL
EDSVTGQSVAQFITDYFGFHHDFLWVVAVVHVGLAVFFAFLFSFAIMKFNFQKR

>XP_025882826.1_ABCG36_X3_Os
MSSAGVVEMQKAASFRREGGGSMASMWLSADGNGAFSRSSSSSSRRMRGEEDDEEALRWAALQKLPTYDRV
RAAILPMVEGEGGEAGGGGGGRRVVVDVHSLGPHERRALLERLVRVADDDNERFLLKLKERISRVGIDMPTIE
VRFEHLEVEAEVRVGNSGIPTVLNSITNKIEEAANALGILPTRKQTLRILHDISGIIKPKRMTLLLGPPGSGKTTFL
LALAGRLKDLKFSGQVTYNGHQMEDFVPQRTAAYISQHDLHIGEMTVRETLSFSARCQGVGSRFDMLTELTR
REKAANIKPDADVDAFMKASAMEGQESNLITDYILKILGLEICADTMVGDDMVRGISGGQRKRVTTGEMLVG
PANAFFMDEISTGLDSSTTFQIVKSLRQTIHILGGTAVISLLQPAPETYDLFDDIILLSDGHIVYQGPRENVLEFFEL
MGFKCPERKGVADFLQEVTSRKDQKQYWAQHDKPYRYVPIKEFASAFQSFHTGRSIANELATPFDKSKSHPAA
LTTSRYGVSAMELLKANIDRELLLIKRNSFVYIFRTIQLMTVSAMAMTVFFRTKMHRDSVADGVIFMGALFFA
VMMIMLNGLSELPLTIFKLPVFFKQRDLLFFPAWTYTIPSWILKSPMSFIEVGGFCFMSYYVIGFDPNVGRFFKQ
YLLMLAVSQMAAALFRFVGGAARNLIVANVFGSFMLLIFMVLGGFILARDKVNKWWIWGYWISPMMYAQN
AVSVNEFLGHSWDKVLNNSLSNETLGVQALMSRGIFPEAKWYWIGFGALLGFIMLFNILFTLALTYLKPDGKS
QPSISEEELKEKQANINGNVLDVDTMASSNNLAIVGSTGTGSEIADNSQPTQRGMVLPFTPLSLTFEDIKYSVDM

PQEMKAHGIVEDRLELLKGVSGCFRPGVLTALMGVSGAGKTTLMDVLAGRKTGGYIEGNISISGYPKKQETFA
RVSGYCEQNDIHSPQVTVSESLLFSAWLRLPKDVDSNTRKMFIEEVMELVELKPLRDALVGLPGVNGLSIEQRK
RLTIAVELVANPSIIFMDEPTSGLDARAAAIVMRTVRNTVNTGRTVVCTIHQPSIDIFEAFDELFLMKRGGEEIYV
GPLGHHSSELIKYFEGIQGVSKITDGYNPATWMLEVTTVSQEQALDVDFCDIYRKSELFQRNKALIQELSTPPPG
SSELYFPTQYSQSFLIQCLACLWKQHLSYWRNPPYNAIRLFFTTVIALIFGTIFWDLGGKMGQSQDLFNAMGSM
YAAVLFIGVLNGQSVQPVVSVERTVFYRERAAGMYSALPYAFGQVAIEFPYTLVQSVIYSIIVYSMIGFQWTVA
KFFWYLFFMFFTLLYFTFYGMMAVGLTPSYHVASIVSSAFYAIWNLFTGFVISRPATPVWWRWYCWICPVAW
TLYGLIVSQYGDIVTPMDDGIPVNVFVENYFDFKHSWLGFVAVVIVAFTMLFAFLFGFAIMKLNFQKR

>XP_008675479.1_ABCG37_X1_Zm
MDREIHRVTSLRRDSSLWRRGDDVFSRQSSRFQDDEEDDDEALRWAALERLPTYDRVRRGILALHEGGGGEK
VEVDVGRLGARESRALVERLVRAADDDHERFLLKLRERMDRVGIDYPTIEVRYESLHVEAQVHVGDRGLPTL
VNSVTNTVESIGNALHILPSRKRPMTVLHDVSGIVKPRRMTLLLGPPGSGKTTLLLALAGKLDKDLRVSGKVTY
NGHGMNEFVPERTAAYISQHDLHIGEMTVRETLAFSARCQGVGTRYEMLTELARREKAANIKPDHDIDVYMK
ASAMGGQESSIVTDYTLKILGLEVCADTLVGNEMMRGISGGQRKRVTTGEMLVGPARALFMDEISTGLDSSTT
YQIVNSLRQTIHILGGTAVISLLQPAPETYNLFDDIILLSDGHVVYQGPREHVLEFFEFMGFRCPARKGVADFLQ
EVTSRKDQGQYWYRQDRPYRFVPVKKFADAFSIFHVGRSTQNELSEPFDRTRSHPAALATSKFGASRMELLKA
TIDRELLLMKRNAFMYIFKAVNLTVMSFIVMTTFFRTNMKRDASYGNIYMGALFFALDTIMFNGFAELAMTV
MKLPVFFKQRDLFFFPAWAYTIPSWIVQIPITFLEVGVYVFTTYYVIGFDPNVFRFLKQYLLLLALNQMSSALFR
FIAGIGRDMVVSHTFGPLALLAFQALGGFILARPDVKKWWIWGYWISPLSYAQNAISTNEFLGHSWSKIQNGTT
VGIGVLQSRGVFTEAKWYWIGLGVLVGYALLFNLLYTVALAVLSPFTDSHGSMSEEELKEKHANLTGEVIEVR
KEKTSRRQELELSHSVGQNSVHSSEDSSQNRKGMALPFPPLSLTFNDIRYSVDMPEAMKAQGVTEDRLLLLKG
VSGSFRPGVLTALMGVSGAGKTTLMDVLAGRKTGGYIEGDITISGYPKKQETFARISGYCEQNDIHSPHVTVYE
SLLFSAWLRLPSGINLETRKMFIEEVMDLVELTSLRGALVGLPGVSGLSTEQRKRLTIAVELVANPSIIFMDEPTS
GLDARAAAIVMRTVRNTVNTGRTVVCTIHQPSIDIFEAFDELFLMKRGGEEIYVGPVGQNSSKLIEYFEGIEGIS
QIKDGYNPATWMLEVSSSSQEEILGVDFSEIYRQSELYQRNKALIEELSTPPPGSSDLNFPTQYSRSFFTQCLACF
WKQKKSYWRNPTYTAVRLLFTVVIALMFGTMFWDLGRKTNKQQDLFNAMGSMYAAVVYIGMQNSGSVQP
VVVVERTVFYRERAAGMYSAFPYAFGQVAIEIPYIFVQTLLYGVLVYSMIGFEWTVAKFLWYLFFMYFTLLYF
TFYGMMAVGLTPNETIAVITSSAFYNVWNLFSGYLIPRPKLPVWWRWYSWICPVAWTLYGLVASQFGDIAHP
LEDSPTGQTVAQFITDYFGFHHDFLWVVAGVHVGLTVLFAFLFSFAIMKFNFQNR

>XP_006349934.1_P_PDR1-l_St
MEPIDFGNLRGSSLRGSVKGSFRGSFGSDSNSIFRNNNIFNRSSRDEDDEEALKWAALEKLPTFDRLRKGLLFGA
NEVDIHDLGNQQSKDLVDRLVKVADEDNEKFLLKLRDRIDRVGLDLPTIEVRYEHLKIEADAYVGSSALPTFIN
FVTNFIEPMLNSLHIVPNRKRKLTILDDMSGIIKPCRLTLLLGPPGSGKTTLLLALAGKLDTELKASGKVTYNGH
EMNEFVPQRTAAYISQHDLHIGEMTVRETLEFSARCQGVGSRYEMLAELSRREKTANIKPDPDIDVFMKAAAT
EGQEANVVTDYVLKILGLDICADTMVGDEMVRGISGGQKKRVTTGEMLVGPSKALFMDEISTGLDSSTTYFIV
NSLRQTVQILKGTAVISLLQPAPETYNLFDDIILLSDSVIVYQGPREDIIGFFESMGFKCPERKGVADFLQEVTSK
KDQQQYWVRRDEPYRFITSKEFSEAYQAFHVGRKLGEDLAVSYDKRKSHPAALTTEKYGIGKKQLFEVCKER
EYLLMKRNSFVYIFKFCQLLIMALISMTIFFRTEMKHDTMDDGGIYAGALFFVIIMNMFNGMSELGMIIYKLPVF
FKQRDLLFFPAWAYAIPSWILKIPVTFVETALWVFLTYYVMGFDPHPSRLFKQFLLLIIVSQMASGLFRFIGAVG
RSLGVASIFGSFALLLQFALGGFVLSRDDVKNWWIWGYWTSPMMYSVNAILVNEFDGKRWKHIPPNGTESLG
AAVVRGRGFFPDASWYWIGFGALVGFTIVFNICYTIALAYLKPFGKPQAMIPEDTEDAQTTSAETEDSNSESQN
KKKGMVLPFEPHSITFDDVMYSVNMPQEMKDQGATEDRLVLLKGVSGAFRPGVLTALMGVSGAGKTTLMDV
LAGRKTGGYIEGDIKISGYPKKQDTFARISGYCEQNDIHSPYVTVYESLVYSAWLRLPHNVDTKTRKMFVEQV
MDLVELGPLRSALVGLPGINGLSTEQRKRLTIAVELVANPSIIFMDEPTSGLDARAAAIVMRTVRNTVDTGRTV
VCTIHQPSIDIFEAFDELFLMKRGGQEIYVGPLGRHSFHLIKYFESMPGVSKIKDGYNPATWMLEVTASAQEILF
GVDFTDLYKKSDLYTRNKALISELSVPRPGTKDLHFDTKYSQPFWTQCIACLWKQHWSYWRNPTYTAVRFLF
TTIIALVFGTMFWDIGGKVSKSQDLFNAMGCLYATVLFLGTQNSSSVQPVVAVERTVFYRERAAGMYSALPY
AFGQISIEIPYVFMQSVFCGAIMYAMIGFEWTVAKFFWYLFFLFFTLLYFTFYGMMTVAVTPNVSVAQIVGSFF
YGVWNLFSGFIVPRTRIPIWWRWYYWCCPVAWTLYGLVASQFGDLQNKLTDEETVEQFLRRYFGFKHDFLPI
VAVAIVGYTVLFGFTFAFAIKAFNFQTR

>XP_024460932.1_PDR1_X2_Pt

MESADIYRASSSLRDSLRAGSFVWRNSTVEAFSRSSREEDDEEALKWAALEKLPTYDRLRKGILMSASRGVSSE
VDIEKLGVQERKQLLERLVKAADEDNEKFLWKLKNRIERVGIEFPTIEVRYEHLNIGAEAYVGSGALPSFAKFT
FSIIEDLLIALRIIPSRKKPLTILKDVSGIIKPSRLTLLLGPPSSGKTTLLLALAGKLDPSLKYSGRVTYNGHGMNEF
VPQRTASYTSQQDLHIGEMTVRETLAFSARCQGVGNLHDMLAELSRREKEANIKPDPDIDVFLKAVATQGQEA
NVITDYVLKILGLEVCADTLVGDEMIRGISGGQRKRVTTGEMLVGPSRALFMDEISTGLDSSTTHQIVNSLKQTI
HILNYTAVISLLQPAPETYDLFDDIILLSDGQIVYQGPRENVLGFFEHLGFQCPERKGVADFLQEVTSRKDQEKY
WARKDQPYRFVTVNEFAEAFQSLSVGRRVIEELSIPFDKTKNHPAALVNKKYGAGKMDLLKANFSREFLLMK
RNSFVYIFKIFQLTMMAIISMTLFFRTKMPRDTVEDGGIYAGALFFTAIMIMFNGMAELSMTIAKLPVFYKQREL
LFFPPWTYSIPPWILKIPITFVEVAAWVFLTYYVIGFDPNIGRFFKLYAVLVLINQMASALFRFIAAAGRNMIVAN
TFGSFVLLAVFALGGVILSREQIKKWWIWGYWASPLMYGQNAIVVNEFLGNSWSHIPAGSTESLGIQVLKSREF
FTEAYWYWIGIGATAGFILLFNVCFVVALTVLDAYEKPQAVISEEPESGDSEGAVQLSNRGISHQTNTASGVEIN
RSGSDSIGEASNNRKKGMVLPFEPHSITFDDVIYSVDMPQEMKVQGVAEDRLALLKGVSGAFRPGILTALMGV
SGAGKTTLMDVLAGRKTGGYIEGDIKISGYPKKQETFARISGYCEQNDIHSPQVTVYESLLYSAWLRLPSEVDS
ETRKMFIEEVMDLVELNPLRSALVGLPGVNGLSTEQRKRLTIAVELVANPSIIFMDEPTSGLDARAAAIVMRTV
RNTVDTGRTVVCTIHQPSIDIFDAFDELFLMKRGGEEIYVGPVGRHSTHLIKYFEEIEGVSKIKDGYNPATWMLE
VTSSSQEMALGVDFANIYKNSNLLRRNKALIAELSTPAPGSKDIYFSTQYSTSFFTQCMACLWKQHWSYWRNP
PYTAVRFLFTTFIGLMFGTMFWDLGSKVGTAQDLSNAMGSMYAAVLFLGFQNGSAVQPVVAVERTVFYRER
AAGMYSALPYAFAQVI

>XP_004253003.1_PDR1-l_Sl
MEPIDFGNLRGSSLRGSVKGSSRGSFRSDSNSIFRNNNIFNRSSRDEDDEEALKWAALEKLPTFDRLRKGILFGA
NEIDIHDLGNQQSKDLVDRLVKVADEDNEKFLLKLRDRIDRVGIDLPTIEVRYEHLKIEADAYVGSSALPTFINF
VTNFIEPLLYSLHIVPNRKRKLTILDDVSGIIKPCRLTLLLGPPGSGKTTLLLALAGKLDTELKASGKVTYNGHE
MNEFVPQRTAAYISQHDLHIGEMTVRETLQFSARCQGVGSRYEMLAELSRREKTANIKPDPDIDVFMKAAATE
GQEANVVTDYVLKILGLDICADTMVGDEMVRGISGGQKKRVTTGEMLVGPSKALFMDEISTGLDSSTTYSIVN
SLRQTVQILKGTAVISLLQPAPETYNLFDDIILLSDSVIVYQGPREDVIGFFESMGFKCPERKGVADFLQEVTSKK
DQQQYWVRRDEPYRFITSKEFSEAYQAFHVGRKLGNDLAVSFDKRKSHPAALTTEKYGIGKKQLFEVCKERE
YLLMKRNSFVYIFKFCQLLIMALISMTIFFRTEMKHDTIDDGGIYSGALFFVIIMNMFNGMSELGMIIYKLPVFFK
QRDLLFFPAWAYAIPSWILKIPVTFVETALWVFLTYYVMGFDPHPSRLFKQFLLLIIVSQMASGLFRFIGAVGRS
LGVASIFGSFALLLQFALGGFVLSRDDVKSWWIWGYWTSPMMYSVNAILVNEFDGKRWKHIPPNGTEPLGAA
VVRGRGFFPDASWYWIGFGALVGFTIVFNICYTIALTYLKPFGKPQAMIPEDSEDAQTTSAETEDSNSESQNKK
KGMVLPFEPHSITFDDVMYSVGMPQEMKDQGATEDRLVLLKGVSGAFRPGVLTALMGVSGAGKTTLMDVLA
GRKTGGYIEGDIKISGYPKKQDTFARISGYCEQNDIHSPYVTVYESLVYSAWLRLPHNVDTKTRKMFVEQVMD
LVELGPLRSALVGLPGINGLSTEQRKRLTIAVELVANPSIIFMDEPTSGLDARAAAIVMRTVRNTVDTGRTVVCT
IHQPSIDIFEAFDELFLMKRGGQEIYVGPLGRHSCHLIKYFESMPGVSKIKDGYNPATWMLEVTASAQEILFGVD
FTDLYKKSDLYTRNKALISELSVPRPGTKDLHFDTKYSQPFWTQCIACLWKQHWSYWRNPTYTAVRFLFTTIIA
LVFGTMFWDIGGKVSKSQDLFNAMGCLYATVLFLGTQNSSSVQPVVAVERTVFYRERAAGMYSALPYAFGQI
SIEIPYVFMQSVFCGAIMYAMIGFEWTVAKFFWYLFFLFFTLLYFTFYGMMTVAVTPNVSVAQIVGSFFYGVW
NLFSGFIVPRTRIPIWWRWYYWCCPVAWTLYGLVASQFGDLQNKLTDEETVEQFLRRYFGFKHDFLPIVAVAI
VGYTVLFGFTFAFAIKAFNFQTR

>XP_024625541.1_PDR1_X3_Mt
MMGGGGSSSIWRNSDAAQIFSNSFHQEDDEEALKWAAIQKLPTFERLRKGLLTSLQGEATEVDVEKLGLQVRK
DLLERLVRLAEEDNEKFLLKLKDRMDRVGIDLPTIEVRFEHLNIEAEAHVGSISLPTFTNFMVNIVESLLNSLHV
LPSRKQRLNILKDVSGIIKPSRMTLLLGPPSSGKTTLLLALAGKLDPKLKFSGRVTYNGHEMSEFVPQRTAAYV
DQNDLHIGELTVRETLAFSARVQGVGPQYDLLAELSRREKDANIKPDPDIDVYMKVVAIEGQKENLITDYVLR
VLGLEICADTVVGNAMIRGISGGQKKRLTTGEMLVGPTKALFMDEISTGLDSSTTFQIVNSMKQYVHILKGTAV
ISLLQPPPETYNLFDDIILLSDSHIIYQGPREHVLEFFKSIGFKCPNRKGVADFLQEVTSRKDQEQYWQHKDQQY
RFVTAEEFSEAFQSFHVCRRLGDELGTEFDKSKSHPAALTTKKYGVGKFELLKACSSREYLLMKRNSFVYIFQL
CQLAVMAMIAMTVFLRTEMRKDSVAHGGIYVGALFFGVVVIMFIGMAELSMVVSRLPIFYKQRGCLFFPPWA
YSLPSWILKIPLTCLEVAVWVFLTYYVIGFDPYIGRFFRQYLILVLVHQMAAALFRFVAAVGRDMTVALTFVSF
AIAILFSMSGFVLSKDSIKKWWIWGFWISPLMYGQNAMVINEFLGNKWKHVLPNSTESLGVEVLKSRSFFTETY
WYWICVGALIGYTLLFNFGYILALTFLNPLGKHQTVIPDESQSNEQIGGSRKRTNVLKFIKESFSKLSNKVKKGE
SRSGSISPSRQEIIAAETNHSRKKGMVLPFEPHSITFDEVTYSIDMPQEMKNRGVLEDKLVLLKGVSGAFRPGVL

TALMGITGAGKTTLMDVLSGRKTGGYIGGNITISGYPKKQETFARISGYCEQTDIHSPYVTVYESLLYPTWLRLS
PDINAETRKMFVEEVMELVELKPLRNALVGLPGVCGLSMEQRKRLTVAVELVANPSIIFMDEPTSGLDARAAA
VVMRTVRNTVDTGRTVVCTIHQPSIDIFESFDELLLLKQGGQEIYVGPLGHNSSNLINHFEGIQGVRKIKDGYNP
ATWMLEVTTSSKERELGIDFAELYKNSELYRINKALVKELSAPAPCSKDLYFPSQYSRSFFTQCMACLWKQHW
SYWRNPEYNAIRFLYSTAVAVLLGSMFWDLGSKIEKEQDLFNAMGSMYSAVILIGVMNCNSVQPVVVVERTV
FYRERAAGMYSTFPYAFGQVLIELPYVFVQAVVYGIIVYAMIGLEWSVVKFSYFLFFMYFTFLYYTYYGMMSV
ALTPNNHISIIVSSAFYSIWNLFSGFIVPRPSIPVWWRWYSWANPVAWSLYGLVTSQYGDVKQNIETSDGRQTV
EDFLRNYFGFKHDFLGVVALVNIAFPIVFALVFAIAIKMFNFQRR

>XP_003625363.2_PDR1_X2_Mt
MMGGGGSSSIWRNSDAAQIFSNSFHQEDDEEALKWAAIQKLPTFERLRKGLLTSLQGEATEVDVEKLGLQVRK
DLLERLVRLAEEDNEKFLLKLKDRMDRVGIDLPTIEVRFEHLNIEAEAHVGSISLPTFTNFMVNIVESLLNSLHV
LPSRKQRLNILKDVSGIIKPSRMTLLLGPPSSGKTTLLLALAGKLDPKLKFSGRVTYNGHEMSEFVPQRTAAYV
DQNDLHIGELTVRETLAFSARVQGVGPQYDLLAELSRREKDANIKPDPDIDVYMKVVAIEGQKENLITDYVLR
VLGLEICADTVVGNAMIRGISGGQKKRLTTGEMLVGPTKALFMDEISTGLDSSTTFQIVNSMKQYVHILKGTAV
ISLLQPPPETYNLFDDIILLSDSHIIYQGPREHVLEFFKSIGFKCPNRKGVADFLQEVTSRKDQEQYWQHKDQQY
RFVTAEEFSEAFQSFHVCRRLGDELGTEFDKSKSHPAALTTKKYGVGKFELLKACSSREYLLMKRNSFVYIFQL
CQLAVMAMIAMTVFLRTEMRKDSVAHGGIYVGALFFGVVVIMFIGMAELSMVVSRLPIFYKQRGCLFFPPWA
YSLPSWILKIPLTCLEVAVWVFLTYYVIGFDPYIGRFFRQYLILVLVHQMAAALFRFVAAVGRDMTVALTFVSF
AIAILFSMSGFVLSKDSIKKWWIWGFWISPLMYGQNAMVINEFLGNKWKHVLPNSTESLGVEVLKSRSFFTETY
WYWICVGALIGYTLLFNFGYILALTFLNPLGKHQTVIPDESQSNEQIGGSRKRTNVLKFIKESFSKLSNKVKKGE
SRSGSISPSRQEIIAAETNHSRKKGMVLPFEPHSITFDEVTYSIDMPQEMKNRGVLEDKLVLLKGVSGAFRPGVL
TALMGITGAGKTTLMDVLSGRKTGGYIGGNITISGYPKKQETFARISGYCEQTDIHSPYVTVYESLLYPTWLRLS
PDINAETRKMFVEEVMELVELKPLRNALVGLPGVCGLSMEQRKRLTVAVELVANPSIIFMDEPTSGLDARAAA
VVMRTVRNTVDTGRTVVCTIHQPSIDIFESFDELLLLKQGGQEIYVGPLGHNSSNLINHFEGIQGVRKIKDGYNP
ATWMLEVTTSSKERELGIDFAELYKNSELYRINKALVKELSAPAPCSKDLYFPSQYSRSFFTQCMACLWKQHW
SYWRNPEYNAIRFLYSTAVAVLLGSMFWDLGSKIEKEQDLFNAMGSMYSAVILIGVMNCNSVQPVVVVERTV
FYRERAAGMYSTFPYAFGQVLIELPYVFVQAVVYGIIVYAMIGLEWSVVKFSYFLFFMYFTFLYYTYYGMMSV
ALTPNNHISIIVSSAFYSIWNLFSGFIVPRPSIPVWWRWYSWANPIAWSLYGLVASQYGDVKQNIETSDGRQTVE
EFLRNYFGFKHDFLGVVALVNVAFPIAFALVFAIAIKMFNFQRR

>XP_024625545.1_PDR1_X7_Mt
MEVGGSFRIGSSSIWRNSDAAEIFSNSFHQGDDEEALKWAAIQILPTFERLRKGLLTSLQGGTIEIDVENLGMQE
KKDLLERLVRLAEEDNEKFLLKLKDRIDRVGIDLPTIEVRFEHLNIEAEARVGSRSLPTFTNFMVNIVERIFNSLL
VLPSRKQHLNILKDVSGIIKPSRMTLLLGPPSSGKTTLLLALAGKLDQKLKFSGRVTYNGHEMSEFVPQRTAAY
VDQNDLHIGELTVRETLAFSARVQGVGPQYDLLAELSRREKDANIKPDPDIDVYMKAVATEGQKANLITDYVL
RVLGLEICADTVVGNAMIRGISGGQKKRLTTGEMLVGPTKALFMDEISTGLDSSTTFQIVNSMKQYVHILKGTA
VISLLQPPPETYNLFDSIILLSDSHIIYQGPREHVLEFFESIGFKCPNRKGVADFLQEVTSVKDQEQFWEHKDQPY
KFVTAEEFSEAFQTFHVGRRLGDELGTEFDKSKSHPAALTTKKYGVGKIELLKACSSREYLLMKRNSFVYIFKL
CQLAVMAMITMTVFLRTEMRKDSVVHGGIYVGALFFGVTVIMFIGMAELSMVVSRLPVFYKQRGCLFFPPWA
YSLPSWILKIPLTCVEVAVWVFLTYYVIGFDPYIGRFFRQYLILVLVHQMAAALFRFIAAVGRDMTVALTFGSF
AIAILFSMSGFVLSKDGIKKWWIWAFWISPLMYGQNAMVNNEFLGNKWKHVLPNSTESLGVEVLKSRSFFTET
YWYWICVGALIGYTLLFNFGYILALTFLNPLGKHQAVIPDESQSNEQIGGSQKRTNALKFIKDGFSKLSNKVKK
GESRRGSISPSRQEIVAAATNHSRKKGMVLPFEPHSITFDEVTYSVDMPQEMRNRGVLEDKLVLLKGVSGAFRP
GVLTALMGITGAGKTTLMDVLSGRKTGGYIGGNIKISGFPKKQETFARISGYCEQTDIHSPHVTVYESLLYSAW
LRLSPDINAETRKMFIEEVMELVELKPLQNAIVGLPGVSGLSTEQRKRLTVAVELVANPSIIFMDEPTSGLDARA
AAIVMRTVRNTVDTGRTVVCTIHQPSIDIFESFDELLLLKQGGQEIYVGPLGHNSSNLINHFEGIQGVSKIKDGY
NPATWMLEVTNSSKEVELGIDFVELYKNSELYRINKALIKELGSPAPCSKDLYFPTQYSRSFFTQCMACLWKQH
WSYWRNPEYNAIRFLYSTAVAVLLGSMFWDLSSKIEKEQDLFNAMGSMYAAVILIGVMNGNSVQPVVAVERT
VFYRERAAGMYSAFPYAFGQASIQLPYVFVQAVVYGIIVYAMIGFEWSMVKVLWCLFFLFFTFLYYTYYGMM
SVALTPNNHISIIVSSAFYSIWNLFSGFIVPRPSIPVWWRWYSWANPMAWSLYGLAASQYGDLKKNIESNDGSQ
TVEEFLRNYFGFKPDFLGVVALVNVAFPIAFALVFSIAIKMFNFQRR

>XP_021310826.1_ABCG35_Sb

MDAAAEGVRRPDSSGGGAESSSGRAGVRSATATAGMDAAAELQRVASLRRDSFGSRSSGPSAWWRATDATF
SRSSSRRGEEEEDDEEALRWAAIERLPTCDRVRSAILPLGGDGDGHGHGGGEVVDVLGLGPRDRRALLERLVC
VADEDNERFLLKVKERIQRVGIDLPTIEVRFEHLSAEADVRVGSSGLPTVLNSITNKLEDIANALHLRRSQKQA
MPILHDVSGIVKPCRMTLLLGPPGSGKTTLLLALAGRLHNNLKVSGKVTYNGHEMDEFVPERTAAYISQHDLH
IGEMTVRETLEFSARCQGVGTRFDLLAELSRREKAGNIKPDTDIDAFMKACSMRGQEANVICDYILKILGLEICA
DTMVGDEMLRGISGGQRKRVTTGEMLVGPANALFMDEISTGLDTSTTFQIIKSIRQTIHILGGTALISLLQPAPET
YDLFDDIILLSDGQIVYQGPRESVLEFFLSLGFKCPQRKGVADFLQEVTSRKDQKQYWVWHDKPYRYVSVKEF
ASAFQSFHVGRAVAHELAIPFDKSKNHPGALTTSRYGVSAWELFKANVDRELLLMKRNSFVYIFRTLQLMITTI
IVMTLFFRTNMHRDSVTDGGIYMGALFFSVLLIMLNGFSELALTIMKIPVFFKQRDLLFFPAWAYTIPTWILKIPI
SFIEVGGFVFMAYYVIGFDPNVVRFFKQYLLFLAVNQMAAALFRFIGGAARDMTVANVFGSFVLLIFMVLCGFI
LDREKVKKWWIWGYWISPMMYAQNALSVNEMLGHSWDKILNSSMSNETLGVQSLKSRGIFPEAKWYWIGLA
ALIGFVMLFNCLFTLALAYLKPYGKSHPSISEEELKAKYANINGNVVAEDSLPVGSSHLETVGITRSSSATVENH
SGTMQRGMILPFAPLSLTFSNIKYFVDMPQEMKTHGVVGDRLELLKGISGSFRPGVLTALMGVSGAGKTTLMD
VLAGRKTSGYIEGNISISGYPKKQETFARVSGYCEQNDIHSPHVTVYESLVFSAWLRLPTDVDSNTRKMFIEEV
MELVELKPLRNALVGLPGVNGLSTEQRKRLTIAVELVANPSIIFMDEPTSGLDARAAAIVMRTVRNIVDTGRTI
VCTIHQPSIDIFEAFDELFLMKRGGEEIYVGPLGHHSSELIKYFEGIEGVKKIEDGYNPATWMLEVTAVSQEQILG
VDFSDLYKKSELYQRNRALIQELSEPPAGSSDLHFHSQYAQSFFMQCLACLWKQNLSYWRNPAYNAVRLFFTT
VIALMFGTIFWDLGGKMGQPQDLFNAMGSMYAAVMFIGVLNSTSVQPVVSVERTVFYRERAAGMYSALPYA
FGQVSIELPYILVQAIVYGIIVYSMIGFEWTVAKLFWYLFFMYFTFLYFTFYGMMAVGLTPSYHVAAIVSTLFY
GIWNLFSGFLIPLPKVPIWWKWYCWACPVAWSLYGLVVSQFGDIRTPMDDGVPVNVFVENYFDFKHSWLGV
VAIVVVAFVVLFAFLFGFAIMKLNFQRR

>XP_015622300.1_ABCG32_X1_Os
MAREIHKIASLRRESSLWRRGDDGVYFSRSSTGASSSRFRDEEDDEEALRWAALERLPTRDRVRRGILLQAAEG
NGEKVEVDVGRMGARESRALIARLIRAADDDHALFLLKLKDRMDRVGIDYPTIEVRFEKLEVEAEVHVGNRG
LPTLLNSIINTVQAIGNALHISPTRKQPMTVLHDVSGIIKPRRMTLLLGPPGSGKTTLLLALAGKLEDNLKVSGK
VTYNGHGMDEFVPQRTAAYISQHDLHIGEMTVRETLAFSARCQGVGSRYDMLTELSRREKAENIKPDQDIDVY
MKASAIGGQESSVVTEYILKILGLDICADTVVGNDMLRGVSGGQRKRVTTGEMLVGPARALFMDEISTGLDSS
TTYQIVNSIGQTIRILGGTAVISLLQPAPETYNLFDDIILLSDGQIVYQGAREHVLEFFELMGFRCPQRKGVADFL
QEVTSKKDQEQYWYRNDIPYSFVPVKQFADAFRSFHVGQSIQNELSEPFDRSRSHPASLATSKFGVSWMALLK
ANIDRELLLMKRNSFVYIFKAANLTLTAFLVMTTFLRTKMRHDTTYGTIYMGALYFALDTIMFNGFAELGMTV
MKLPVFFKQRDLLFFPAWTYTIPSWILQIPVTFFEVGVYVFTTYYVVGFDPNVSRFFKQYLLLVALNQMSSSLF
RFIAGIGRDMVVSQTFGPLSLLAFTALGGFILARPDVKKWWIWGYWISPLSYAQNAISTNEFLGRSWNKSFPGQ
NDTVGISILKSRGIFTEAKWYWIGFGALIGYTLLFNLLYTVALSFLKPLGDSYPSVPEDALKEKRANQTGEILDS
CEEKKSRKKEQSQSVNQKHWNNTAESSQIRQGILPFAQLSLSFNDIKYSVDMPEAMTAQGVTEERLLLLKGVS
GSFRPGVLTALMGVSGAGKTTLMDVLAGRKTGGYIEGDITISGYPKKQETFARISGYCEQNDIHSPHVTVYESL
VFSAWMRLPSEVDSETRKMFIEEVMELVELTSLRGALVGLPGVNGLSTEQRKRLTVAVELVANPSIIFMDEPTS
GLDARAAAIVMRTVRKTVDTGRTVVCTIHQPSIDIFEAFDELFLMKRGGEEIYVGPLGQNSSKLIEYFEGIEGISK
IKDGYNPATWMLEVTSTTQEEMLGIDFSEIYKRSELYQRNKELIQDLSTPTPGSTDLHFPTQYSRSFFTQCIACL
WKHKLSYWRNPSYTAVRLLFTIIIALLFGTMFWDLGRKTKKEQDLFNAVGSMYAAVLYIGIQNSGCVQPVVV
VERTVFYRERAAGMYSGFPYAFGQVAIELPYILVQTLVYGVLVYSMIGFEWTVAKFIWYLFFMYFTLLYFTFF
GMMAVGLTPNESIAAIISPAIYNAWNLFSGYLIPRPKIPVWWRWYCWICPVAWTLYGLVASQFGNIQTKLDGK
DQTVAQFITEYYGFHHDLLWLVAVVHVVFTVMFAFLFSFAIMKFNFQRR

>XP_002458133.1_ABCG35_Sb
MNAAAELQKVATSMRGNSGGSGSGRPSAWWRAPDDAFSRSSSRREEEDDEEALRWAALERLPTCDRIHRAIL
PLGGGDCDGGGEAAPQVVDVLGLGPRERRALLERLVRVADEDNERFLLKIKERVERVGIDMPTIEVRFEHLSA
EADVRVGSSGLPTVLNSITNKLEDVANALHVRRSRKQAIPILHDVSGIVKPRRMTLLLGPPRSGKTTLLLALAG
RLDKDLKVSGKVTYNGHEMDEFVPERTAAYISQHDLHIGEMTVRETLEFSARCQGVGTRFDLLAELSRREKAG
NIKPDTDIDAFMKACSMRGQEANVICDYILKILGLEICADTMVGDEMWRGISGGQRKRVTTGEMLVGPANALF
MDEISTGLDSSTTFQIIKSLRQAIHNLGGTALISLLQPAPETYDLFDDIILLSDGQIVYQGPRESVLEFFSSLGFKCP
ERKGVADFLQEVTSRKDQKQYWVRHDKPYQYVSVKDFASAFQSFHVGRAIANELVVPFDKCKNHPSSLTTSR
YGVSSWELLKANIDREILLMKRNSFVYIFKTLQLMMMSIMGMTIFFRNKMHHDSVTDGGIYFGALFFTVITIMF
NGFSELALTVIKLPVFFKQRDLLFFPAWACTIPTWILRIPISFVEVGGFVFMAYYVIGFDPNVGRFFKQYLLLLAF

NQMATSLFRFVGGAARNMIIANVFGGFILLSFMVLGGFILVRDKVKKWWIWGYWISPLMYAQNAISVNEMLG
HSWDKILNSSMSNETLGVQSLKSRGVFPEAKWYWIGLGALIGFVMLFNCLFTLALAYLKPYGKSHPSISEEELK
VKYANLSGNVVAGGNLPLGSSHLETVGITRSGSATVENHSGTTQRGMVLPFARLSLTFNNIKYFVDMPQEMKT
LGVVGDRLELLKGISGSFKPGVLTALMGASGAGKTTLMDVLAGRKTSGYIEGNISISGYPKKQETFARVSGYCE
QNDIHSPQVTVYESLVFSAWLRLPKDVDSNTRKVFIEEVMELVELKPLRNALVGLPGVNGLSTEQRKRLTIAVE
LVANPSIIFMDEPTSGLDARAAAIVMRTVRNTVDTGRTIVCTIHQPSIDIFEAFDELFLMKPGGEEIYVGPLGHHS
SELIKYFEGIDGVKKIKNGYNPATWMLEVTTISQEQILGVDFSDMYKKSELYQRNKALIQKLSEPSAGSSDLHF
RNQYSQSFFMQCVACLWKQNLSYWRNPAYNAIRLFFTTIIALISGTVFWDLGGKMSQSQDLLNTMGSMYAAV
MFIGILNAKSIQPVVFVERTVFYRERAAGMYSALPYAFGQVSIELPYTLAQATIYGVIVYSMIGFKWTVAKFFW
YLFFMYFTFLYFTFYGMMAVGLTPSYPVASIVSSAFYNIWNLFSGFIIPRPKVPIWWNWYCWACPVAWTLYGL
VVSQFGDITTPMDNGVPVNVFVEKYFGFKHSWLGVVAVVVVAFAIFFALLFGFAIMKLNHQRR

>NP_001274820.1_PDR1-l_St
MEPSDLSNLRGRSIRGSMRGSMRENSNSIWRNNGVEVFSRSNRDEDDEEALKWAALEKLPTYDRLRKGILFGS
QGVAAEVDVDDLGVQQRKNLLERLVKVADEDNEKFLLKLKNRIDRVGIDFPSIEVRFEHLNIEADAYVGSRAL
PTFTNFISNFIESLLDSIHIFPSKKRSVTILKDVSGYVKPCRMTLLLGPPGSGKTTLLLALAGKLDSDLRVTGKVT
YNGHELHEFVPERTAAYISQHDLHIGEMTVRETLEFSARCQGVGSRYEMLAELSRREKAANIKPDVDIDMFMK
ILGLDICADTMVGDQMIRGISGGQKKRVTTGEMIVGPSKALFMDEISTGLDSSTTYSIVNSLKQSVQILKGTALIS
LLQPAPETYNLFDDIILLSDGYIVYQGPREDVLEFFESMGFKCPDRKGVADFLQEVTSKKDQQQYWVRRDEPY
RFITSKEFAEAYQSFHVGRKVSNELSTAFDKSKSHPAALTTEKYGIGKKQLLKVCTEREFLLMQRNSFVYIFKFF
QLMVIALMTMTIFFRTEMPRDTETDGGIYTGALFFTVVMLMFNGLSELPLTLYKLPVFYKQRDFLFYPSWAYAI
PSWILKIPVTLLEVGMWTVLTYYVIGFDPNVGRFFKQFLLLVLVNQMASGLFRFIAAVGRTMGVASTFGACAL
LLQFALGGFALARTDVKDWWIWGYWTSPLMFSVNAILVNEFDGEKWKHTAPNGTEPLGPSVVRSRGFFPDAY
WYWIGIGALAGFTILFNIAYSLALAYLNPFGKPQATISEEGENNESSGSSPQITSTAEGDSVGENQNKKKGMVLP
FEPQSITFDEVVYSVDMPPEMREQGSSDNRLVLLKGVSGAFRPGVLTALMGVSGAGKTTLMDVLAGRKTGGY
IDGSIKISGYPKKQETFARISGYCEQNDIHSPYVTVYESLVYSAWLRLPQDVDEHKRMMFVEEVMDLVELTPLR
SALVGLPGVNGLSTEQRKRLTIAVELVANPSIIFMDEPTSGLDARAAAIVMRAVRNTVDTGRTVVCTIHQPSIDI
FEAFDESMPGVGKIEEGYNPATWMLEVTSSSQEMSLGVDFTDLYKNSDLCRRNKALITELSVPRPGTSDLHFEN
QFSQPFWVQCMACLWKQRWSYWRNPAYTAVRFLFTTFIALIFGSMFWDLGTKVSRPQDLTNAMGSMYAAVL
FLGVQNASSVQPVVSVERTVFYREKAAGMYSAIPYAFAQVFIEIPYVFVQSVVYGLIVYSMIGFEWTVAKFFW
YFFFMFFTFLYFTFFGMMTVAITPNQNVASIVAGFFYTVWNLFSGFIVPRPRIPIWWRWYYWGCPVAWTLYGL
VASQFGDLQDIVNGQTVEEYLRNDYGIKHDFLGVVAGVIVAFAVVFAFTFALGIKAFNFQKR

>XP_015638596.1_ABCG35_Os
MDAAAEMQKVVSLRRGGGGSSSRGAASMWWSADNGVFSRSRASSSGEDGEDDEEALRWAALEKLPTYDRV
RRAVLPVVEEGGGGGEAGKKVVDVLSLGPQERRALLERLVRVAEDDNERFLLKLKERIDRVGIDIPTIEVRFEH
LEAEAEVRVGNSGLPTVLNSMTNKLEGAANALGILPNKKQTMPILHDVSGIVKPRRMTLLLGPPGSGKTTLLL
ALAGRLGKDIKFSGQVTYNGHQMEDFVPQRTAAYISQHDLHIGEMTVRETLSFSARCQGVGSRFDMLTELSRR
EKAANIKPDADIDAFMKASAMEGQETNLITDYILKILGLDICADTMVGDDMVRGISGGQRKRVTTGEMLVGPA
NALFMDEISTGLDSSTTFQIVKSLRQAIHILGGTAVISLLQPAPETYDLFDDIILLSDGQIVYQGPREGVLEFFELM
GFKCPERKGVADFLQEVTSRKDQKQYWMQHDKPYRYVPVKDFASAFQSFHTGKSIANELATPFDKSKNHPAA
LTTSRYGVSAMELLKANIDREFLLMKRNSFVYIFRACQLMVVSAIAMTVFFRTKMHRDSVTDGVIFMGALFFS
VMMIMFNGLSELPLTIFKLPVFFKQRDLLFFPAWTYTIPSWILKIPMSFIEVGGFVFMSYYVIGFDPSAGRFFKQY
LLMLAINQMAAALFRFVGGAARNMIVANVFGSFMLLIFMVLGGFILVREKVKKWWIWGYWISPMMYAQNAI
SVNEFLGHSWDKVLNNSLSNETLGVQALRSRGVFPEAKWYWIGFGALLGFIMLFNGLFTLALTYLKPYGKSQP
SVSEEELKEKQANINGNVLDVDTMASSTNLAIVDNTETSSEIADNSQPTQRGMVLPFAPLSLTFDNIKYSVDMP
QEMKAHGIVEDRLELLKGVSGSFRPGVLTALMGVSGAGKTTLMDVLAGRKTGGYIEGNITISGYPKKQETFAR
VSGYCEQNDIHSPQVTVSESLLFSAWLRLPKDVDSNTRKMFIEEVMELVELKPLRDALVGLPGVNGLSTEQRK
RLTIAVELVANPSIIFMDEPTSGLDARAAAIVMRTVRNTVDTGRTVVCTIHQPSIDIFEAFDELFLMKRGGEEIYV
GPLGHQSSELIKYFEGIKGVSRIKDGYNPATWMLEVSTISQEQALGVDFCDIYRKSELFQRNKALIQELSTPPPGS
SELYFPTKYSLSFLNQCLACLWKMHLSYWRNPPYNAIRLFFTTVIALLFGTIFWDLGGKTGKSQDLFNAMGSM
YSAVLFIGVLNSQSVQPVVSVERTVFYRERAAGMYSAFPYAFGQVAIEFPYTLVQSIIYGIIVYSMIGFKWTAAK
FFWYLFFMFFTFLYFTFYGMMAVGLTPSYHVASIVSSAFYGIWNLFSGFIIPRPKVPIWWRWYCWICPVAWTLY
GLVASQFGDIMTPMDDGTPVKIFVENYFDFKHSWLGVVAVVIVAFTMLFAFLFGFAIMKLNFQKR

>XP_013454711.1_PDR1_X1_Mt
MDGSDLYKASSSLRGNTSTFFSRSSRREEDDEEALKWAAIEKLPTYNRLKKGLLASSHGVANEIIDIDKLGVQE
RQKLLNRLIKAAEEDNEKFLLKLKERIDRVGIEIPTIEVRFEHLTIEAEAYVGSRALPSFTNFTIGAVEGLLAFLGII
SHKKKHMTILKDVSGIVKPGRMALLLGPPSSGKTTLLLALTGKLDKALKESGRVTYNGYGMDEFVPQRTAAYI
SQHDVHIGEMTVRETLAFSARCQGVGSRYDLLSELCRREKEAKIIPDPDIDVYMKATSTEGQEESLITDYMLKIL
GLDICADTMVGDEMHRGISGGQRKRLTTGEMLVGPSKVLFMDEISTGLDSSTAFQIVKSLRQYVYILHGTALIS
LLQPAPETYELFDDIILLSDGEIVYQGPRENVLEFFESIGFKCPERKGVADFLQEVTSRKDQEQYWMHRDEPYRF
VTVTQFAEAFKSFHVGKTIKEELEIPFDKSNNHPAALTTKKYGVNKKELLKANISREFLLMKRNSFIYLFKMVQ
VSIMATITMTLFLRTHMHKETVIDGQIHFGALYFSLIMLMFNGTIELTMTIVKLPTFFKQRDHLFYPSWAYAIPS
WIVRAPVTLVDASIWVFLTYYVIGFDPNIWRFLKMYLLLILLNQTASGLFRAIAAFCRDMIIANITGFYTLLIVFT
LGGFVLAKDDIKGWWIWGYWISPLMYAQNAIMVNEFLGNSWNKITPYSNMTLGILSLKSRGFFTHAYWYWIG
VGALIGFIFLTNFLYIIALTYLDPLDKPQATIKEESGGDNAPNDRNQEIELPQLENSRRSTAVADSSRGKERGMV
LPFEPYSITFDEIVYSVDVPQEMKDQGVIEDRLVLLKGVSGAFRPGVLTALMGVSGAGKTTLMDVLAGRKTSG
YIDGSIKISGYPKKQETFARISGYCEQNDIHSPQVTVYESLLYSAWLRLPAEVDSNTRKMFIEEVMELVELNPLR
NSLVGLPGVSGLSTEQRKRLTISVELVANPSIIFMDEPTSGLDARAAAIVMRTVRNTVDTGRTVVCTIHQPSIDIF
ESFDELFLLKSGGREIYAGPLGRYSNQLIKYFESIEGVSKINDGYNPATWMLEVTSPAQEVALGADFHEIYKNSE
LYRRNKQLIEELGKPALGSKDLYFPSQYSQSFLVQCLACLWKQRWSYWRNPLYTAVRFYFATFIALMFGTMF
WDLGRKYTRGLDLSNAMGSMYTAVFFIGVQNSASVQPVVAVERTVFYRERAAGMYSALPYALAQVLIELPYI
LAQTLSYGVIVYAMIGFEWTVPKFFWYMFFMYFTFCYFTFYGMMTVAVTPNYHLAAVLASAFYGSWNLFSG
FVVPRPRMPVWWRWYYWANPVAWSLYGLFASQFGNITDIMEMEDVTVQEYIRNYYGIKHDFVGVSAAVVF
GIAIAFAFTFAVSIKVFNFQHR

>XP_006576269.1_PDR-l _X1_Gm
MEGGGSSFRIGSSSIWRNSDAAEIFSNSFHQENDEEALKWAAIQKLPTVARLRKALITSPDGESNEIDVKKLGLQ
EKKALLERLVKTAQEDNEKFLLKLKDRIDRVGIDLPTIEVRFENLSIEAEARAGTRALPTFTNFIVNILEGLLNSL
HVLPNRKQHLNILEDVSGIIKPGRMTLLLGPPSSGKTTLLLALAGKLDPKLKFSGKVTYNGHGMNEFVPQRTA
AYVNQNDLHVAELTVRETLAFSARVQGVGPRYDLLAELSRREKEANIKPDPDIDAYMKAVASEGQKANMITD
YILRILGLEVCADTVVGNAMLRGISGGQRKRVTTGEMLVGPAKALFMDEISTGLDSSTTFQIVNSLKQYVHILK
GTTVISLLQPAPETYNLFDDIILLSDSHIVYQGPREHVLEFFELMGFKCPQRKGVADFLQEVTSRKDQEQYWAH
KDQPYRFVTAKEFSEAHKSFHIGRSLGEELATEFDKSKSHPAALTTKMYGVGKWELLKACLSREYLLMKRNSF
VYTFKLCQVGLEIGMFHFIVLSPNAIIILLKCFPSFQLAVLAIIAMTIFLRTEMHRDSVTHGGIYVGALFYGVVVI
MFNGLAELSMVVSRLPVFYKQRDYLFFPSWVYALPAWILKIPLTFVEVGVWVFLTYYAIGFDPYVGRLFRQYL
VLVLVNQMASALFRLVAAVGREMTVALTLGSFTLAILFAMSGFVLSKENIKKWWLWGFWISPMMYGQNAM
VNNEFLGKRWRHFLPNSTEALGVEILKSRGFFTQSYWYWIGVGALIGYTLLFNFGYILALTYLNPLGKHQAVIS
EEPQINDQSGDSKKGTNVLKNIQRSFSQHSNRVRNGKSLSGSTSPETNHNRTRGMILPSEPHSITFDDVTYSVDM
PVEMRNRGVVEDKLALLKGVSGAFRPGVLTALMGVTGAGKTTLMDVLAGRKTGGYIGGNITISGYPKKQETF
ARISGYCEQNDIHSPHVTVYESLLYSAWLRLSPEINADTRKMFIEEVMELVELKALRNALVGLPGINGLSTEQR
KRLTIAVELVANPSIIFMDEPTSGLDARAAAIVMRTVRNTVDTGRTVVCTIHQPSIDIFESFDELLLMKQGGQEIY
VGPLGHHSSHLINYFEGIQGVNKIKDGYNPATWMLEVSTSAKEMELGIDFAEVYKNSELYRRNKALIKELSTPA
PGSKDLYFPSQYSTSFLTQCMACLWKQHWSYWRNPLYTAIRFLYSTAVAAVLGSMFWDLGSKIDKQQDLFNA
MGSMYAAVLLIGIKNANAVQPVVAVERTVFYREKAAGMYSALPYAFAQVLIELPYVLVQAVVYGIIIYAMIGF
EWTVTKVFWYLFFMYFTFLTFTYYGMMSVAVTPNQHISSIVSSAFYAVWNLFSGFIVPRPRIPVWWRWYSWA
NPVAWSLYGLVASQYGDIKQSMESSDGRTTVEGFVRSYFGFKHDFLGVVAAVIVAFPVVFALVFAISVKMFNF
QRR

>XP_024637157.1_PDR1_X2_Mt
MDGSDLYKASSSLRGNTSTFFSRSSRREEDDEEALKWAAIEKLPTYNRLKKGLLASSHGVANEIIDIDKLGVQE
RQKLLNRLIKAAEEDNEKFLLKLKERIDRVGIEIPTIEVRFEHLTIEAEAYVGSRALPSFTNFTIGAVEGLLAFLGII
SHKKKHMTILKDVSGIVKPGRMALLLGPPSSGKTTLLLALTGKLDKALKESGRVTYNGYGMDEFVPQRTAAYI
SQHDVHIGEMTVRETLAFSARCQGVGSRYDLLSELCRREKEAKIIPDPDIDVYMKATSTEGQEESLITDYMLKIL
GLDICADTMVGDEMHRGISGGQRKRLTTGEMLVGPSKVLFMDEISTGLDSSTAFQIVKSLRQYVYILHGTALIS
LLQPAPETYELFDDIILLSDGEIVYQGPRENVLEFFESIGFKCPERKGVADFLQEVTSRKDQEQYWMHRDEPYRF
VTVTQFAEAFKSFHVGKTIKEELEIPFDKSNNHPAALTTKKYGVNKKELLKANISREFLLMKRNSFIYLFKMVQ

VSIMATITMTLFLRTHMHKETVIDGQIHFGALYFSLIMLMFNGTIELTMTIVKLPTFFKQRDHLFYPSWAYAIPS
WIVRAPVTLVDASIWVFLTYYVIGFDPNIWRFLKMYLLLILLNQTASGLFRAIAAFCRDMIIANITGFYTLLIVFT
LGGFVLAKDDIKGWWIWGYWISPLMYAQNAIMVNEFLGNSWNKITPYSNMTLGILSLKSRGFFTHAYWYWIG
VGALIGFIFLTNFLYIIALTYLDPLDKPQATIKEESGGDNAPNDRNQEIELPQLGDNSSRGKERGMVLPFEPYSITF
DEIVYSVDVPQEMKDQGVIEDRLVLLKGVSGAFRPGVLTALMGVSGAGKTTLMDVLAGRKTSGYIDGSIKISG
YPKKQETFARISGYCEQNDIHSPQVTVYESLLYSAWLRLPAEVDSNTRKMFIEEVMELVELNPLRNSLVGLPGV
SGLSTEQRKRLTISVELVANPSIIFMDEPTSGLDARAAAIVMRTVRNTVDTGRTVVCTIHQPSIDIFESFDELFLLK
SGGREIYAGPLGRYSNQLIKYFESIEGVSKINDGYNPATWMLEVTSPAQEVALGADFHEIYKNSELYRRNKQLI
EELGKPALGSKDLYFPSQYSQSFLVQCLACLWKQRWSYWRNPLYTAVRFYFATFIALMFGTMFWDLGRKYTR
GLDLSNAMGSMYTAVFFIGVQNSASVQPVVAVERTVFYRERAAGMYSALPYALAQVLIELPYILAQTLSYGVI
VYAMIGFEWTVPKFFWYMFFMYFTFCYFTFYGMMTVAVTPNYHLAAVLASAFYGSWNLFSGFVVPRPRMPV
WWRWYYWANPVAWSLYGLFASQFGNITDIMEMEDVTVQEYIRNYYGIKHDFVGVSAAVVFGIAIAFAFTFAV
SIKVFNFQHR

>XP_004252846.1_PDR1-l_Sl
MEPTNSRGTSLRERIIRGNSLKGNSTNNSRWTSNDGEIFNRSTRDEDDEEALKWAALEKLPTFDRLRKGLLFGS
QGASAEIDIHDIGFQERNKLLERLVKVADEDNEKLLLKLRQRIDRVGIDFPEIEVRYENLTIEADAYIGSRALPTF
TNFITNFLEDMLNSLHILPSRKRNLTILNDVSGIIKPCRLTLLLGPPGCGKTTFLLALAGKLDSALKVTGKVTYN
GHVMNEFVPQRTAAYISQYDLHIGEMTVRETLEFSARCQGVGSRYEMLIELSRREKAAKIKPDPDIDIFMKALA
TEGQEAIFVTDYVLKLLGLDICADTLVGDEMIRGISGGQKKRVTTGEMLVGPSKALFMDEISTGLDSSTTYSIV
NSLRQSVQILHGTAVISLLQPAPETYNLFDDIILLSDGKIVYQGPREDVLGFFESMGFKCPDRKGVADFLQEVTS
KKDQQQYWVRDETYQFIKSNEFAEAYQSFHVGRKLADELAASYDKSKSHPAALSTQKYGIGRKQLLKVCTER
EILLMKRNLFVYIFKFIQNMIIAVITTTLFFRTKMPHDTIEDGGKYAGALFFIVTQIMFSGMIEIGLVIYKLPIFYKQ
RDLLFFPSWAYAMPSWILKIPIAFVEVGLWVLLTYYVIGFDPSPVRLFKHFLLLILVNQMTSGMCRFLGAAGRT
MGVANTYGTFALLLLFGLGGFVLSRDDVKKWWIWGYWSSPLMYSLNSIFVNEFDGKRWKHIAPTGTDSLGV
AIVRSRGFFPNAYWYWIGVGALIGFTIVFNICYSIALAFLNPLGKPQGMISEDSDDAKTTNTGKEVPTSEGQNKK
KGMVLPFEPHSITFNEVTYSVDMPQEMKNQGATEDRLVLLNGVCGAFRPGVLTALMGVSGAGKTTLMDVLA
GRKTGGYIEGSIKISGYPKKQETFARISGYCEQNDIHSPYVTVYESLVYSAWLRLPSDVDEKTRKMFVDEVMEL
VELTPLRSALVGLPGVNGLSTEQRKRLTIAVELVANPSIIFMDEPTSGLDARAAAIVMRTVRNTVDTGRTVVCTI
HQPSIDIFEAFDELFLMKRGGNEIYVGPLGHHSCHLIRYFESIPGVSKIHDGYNPATWMLEVTNLAQETMLGLD
FTDLYKKSDLYRRNKTLISELSMPCPGTKDLHFNNQYSQPFWIQCMACLWKQHWSYWRNPAYTAVRYICTIFI
ALAIGTMFWDLGTKVGKKQDLFNALGSLYTPVFFLGFQNASSVLPVVAVERTVYYRERAAGMYSAIPYAFGQ
TFIEIPYVFVQAVSYGVIVYAMIGFEWTVTKFFWYLFIMFFTLLYFTFYGMMSVAITPNQHVAQIVSVSGYGM
WNLFSGFIVPRPSMPIWWRWYYWADPVAWTLYGLVASQFGDLQNKITDSDETAKQFLRRYFGFKHDFVGVA
AVVTVAYTLVFAFTFALAIKVFNFQKR

>XP_024637158.1_PDR1_X3_Mt
MDGSDLYKASSSLRGNTSTFFSRSSRREEDDEEALKWAAIEKLPTYNRLKKGLLASSHGVANEIIDIDKLGVQE
RQKLLNRLIKAAEEDNEKFLLKLKERIDRVGIEIPTIEVRFEHLTIEAEAYVGSRALPSFTNFTIGAVEGLLAFLGII
SHKKKHMTILKDVSGIVKPGRMALLLGPPSSGKTTLLLALTGKLDKALKESGRVTYNGYGMDEFVPQRTAAYI
SQHDVHIGEMTVRETLAFSARCQGVGSRYDLLSELCRREKEAKIIPDPDIDVYMKATSTEGQEESLITDYMLKIL
GLDICADTMVGDEMHRGISGGQRKRLTTGEMLVGPSKVLFMDEISTGLDSSTAFQIVKSLRQYVYILHGTALIS
LLQPAPETYELFDDIILLSDGEIVYQGPRENVLEFFESIGFKCPERKGVADFLQEVTSRKDQEQYWMHRDEPYRF
VTVTQFAEAFKSFHVGKTIKEELEIPFDKSNNHPAALTTKKYGVNKKELLKANISREFLLMKRNSFIYLFKMVQ
VSIMATITMTLFLRTHMHKETVIDGQIHFGALYFSLIMLMFNGTIELTMTIVKLPTFFKQRDHLFYPSWAYAIPS
WIVRAPVTLVDASIWVFLTYYVIGFDPNIWRFLKMYLLLILLNQTASGLFRAIAAFCRDMIIANITGFYTLLIVFT
LGGFVLAKDDIKGWWIWGYWISPLMYAQNAIMVNEFLGNSWNKITPYSNMTLGILSLKSRGFFTHAYWYWIG
VGALIGFIFLTNFLYIIALTYLDPLDKPQATIKEESGENSRRSTAVADSSRGKERGMVLPFEPYSITFDEIVYSVDV
PQEMKDQGVIEDRLVLLKGVSGAFRPGVLTALMGVSGAGKTTLMDVLAGRKTSGYIDGSIKISGYPKKQETFA
RISGYCEQNDIHSPQVTVYESLLYSAWLRLPAEVDSNTRKMFIEEVMELVELNPLRNSLVGLPGVSGLSTEQRK
RLTISVELVANPSIIFMDEPTSGLDARAAAIVMRTVRNTVDTGRTVVCTIHQPSIDIFESFDELFLLKSGGREIYAG
PLGRYSNQLIKYFESIEGVSKINDGYNPATWMLEVTSPAQEVALGADFHEIYKNSELYRRNKQLIEELGKPALG
SKDLYFPSQYSQSFLVQCLACLWKQRWSYWRNPLYTAVRFYFATFIALMFGTMFWDLGRKYTRGLDLSNAM
GSMYTAVFFIGVQNSASVQPVVAVERTVFYRERAAGMYSALPYALAQVLIELPYILAQTLSYGVIVYAMIGFE

WTVPKFFWYMFFMYFTFCYFTFYGMMTVAVTPNYHLAAVLASAFYGSWNLFSGFVVPRPRMPVWWRWYY
WANPVAWSLYGLFASQFGNITDIMEMEDVTVQEYIRNYYGIKHDFVGVSAAVVFGIAIAFAFTFAVSIKVFNFQ
HR

>XP_002462387.1_ABCG36_Sb
MDPSGEIRKVASMRRDSGGSVWRSGGNDIFSRSSRRDDDMDDEEALRWATLEKLPTRDRVRRAIIFPLPPAGA
AGTTTGQQQGLVDVDVLSLGPGERRALLERLVRVADEDHERFLVKLRERLDRVGIDMPTIEVRFEHLNVEAEV
RVGSSGIPTVLNSITNTLEEAATALRILRSRKRALPILHDVSGIIRPRRMTLLLGPPGSGKTTLLLALAGRLDKDL
KVSGRVSYNGHGMEEFVPQRTAAYISQHDLHIAEMTVRETLAFSARCQGVGSRFDMLMELSRREKAANIKPD
ADIDAFMKASAVGGHEANVVTDYILKILGLELCADTMVGDEMLRGISGGQRKRVTTGEMLVGPARALFMDEI
STGLDTSTTFQIVNSLRQSIHVLGGTAVISLLQPGPETFNLFDDIILLSDGQVVYQGPREDVIEFFESMGFRCPQR
KGVADFLQEVTSKKDQKQYWAWSDKPYRFVPAKEFATAHKLFHTGRALAKDLAMPFNKNKSHPAALTTTRY
GVSGMELLKANIDREILLMKRNSFIYVFRTFQLTLMSIIAMTVFFRTNMKHDSVASGGIYMGAMFFGILMIMYN
GFSELALTVFRLPVFFKQRDLLFYPAWAYTIPSWILKIPISFMEVSGYVFLTYYVIGYDPNVGRFFKQYLIMLAIN
QLAASLFRFIGGAARNMIVANVFAMLVMMAAIILNGFIIIRDKVKKWWIWGYWISPLMYVQNAITVNEMLGH
SWDKVLNRTISNETLGVQVLKSHGVFPEAKWYWIGFGALLGFTILLNVVFTFALTYLKPNGNPKPSISEEELKL
KCSNVNNDIMDANPLASRTTLQLIGNNTETNLEMLEDNSGPSQRGMVLPFPPLSLSFDDIRYSVDMPQEMKAQ
GVVEDRLILLKGISGSFRPGVLTALMGVSGAGKTTLMDVLAGRKTGGYVEGNISISGYLKNQETFARVSGYCE
QNDIHSPQVTVDESLLFSAWLRLPKDVDSNTRKMFIEEVMELVELKPLRDALVGLPGVNGLSTEQRKRLTIAVE
LVANPSIIFMDEPTSGLDARAAAIVMRTVRNTVNTGRTVVCTIHQPSIDIFEQFDELFLMKRGGEVIYAGPLGHN
SLELIKYFEAIEGVSKIKDGYNPATWMLEVTTVSQEHVLGVDFSDIYKKSELYQRNKDLIKELSQPAPGSRDLY
FPTKYSQSSFTQCMACIWKQNMSYWRNPPYNTARFIFTTITALIFGTMFWNLGSKIDKSQDLFNALGSMYLSVI
FLGCTNSISVQPVVAVERTVFYRERAAGMYSAFPYAFGQVVIELPYALVQASIYGVIVYAMIGFEWTAAKFFW
YLFFMYFTLLYFTFYGMMGVGLTPNYQIASIVSTAFYNIWNLFSGFFIPRPKTPIWWRWYCWICPVAWTLYGL
VVSQYGDITTPMEDGRTVNVFLEDYFDFKHSWLGRAAAIVVAFSVFFATLFAFATMKLNFEKR

>XP_003553514.1_PDR1_X2_Gm
MEDGGSVKAGSTTNTMSSFRIGSRSVWSNSGVEIFANSFHQEDDEEALKWAAIQKLPTFARLRTGLMTSPEGV
ANEVNVHQLGLQERRGLLERLVRVAEEDNEKFMLKLRDRIDRVGITIPTIEVRFENMNIGAEVHVGSRALPTFT
NYMVNKVEGLLNFLHVLPSRKQRINILQNVSGIIRPARMTLLLGPPSSGKTTLLLALAGRLDSKLKFTGKVTYN
GHGMNEFVPQRTAAYVSQNDLHIGEMTVRETLAFSARVQGVGARYDLLAEVSRREKEANIKPDPDIDVYMKA
VATEGQKANFITDYILRILGLEVCADTIVGNAMLRGISGGQRKRVTTGEMLVGPAKAVFMDEISTGLDSSTTFQ
VVNSLKHFIHSLKGTAVVSLLQPAPETYNLFDDIILLSDGQIVYQGPREHVLEFFASVGFKCPERKGVADFLQEV
TSRKDQEQYWVHRDQPYRFVTTEEFVEAFQSFHVGRSLADELATQFDKSKSHPAALATKMYGLGKWELLKA
CLSREYLLMKRNSFVHIFQLCQLAIVAFIAMTVFFRTEMHPDSVTSGGIYAGALFYGLLVILLDGFADLTMTVS
KLPVFYKQRDFLFFPSWVYALPAWILKIPMTFAQVGIWVFLTYYVIGFDPYVGRFFRQFLLLLFVNQMASALFR
FIGALGRELTVAFTIGSFVLAILIAMSGFILSKGNMKKWWLWGFWSSPMMYGLNAMINNEFQGKRWRHVLPN
STTPLGVQVLKSRGFFTQSKWYWIGVGALIGYTIVFNIAYILALTYLNPIVQHQAVKSEKSQSNEQDGGSTSARS
SSRRKEADRRRGMALPFEPHSITFDDVTYSVDMPQEMKNQGVLEDRLNLLKGVSGTFRPGVLTALMGSTGAG
KTTLMDVLAGRKTGGYIGGNITISGYPKKQETFARISGYCEQNDIHSPYVTVYESLLYSAWLRLSAEINSETRK
MFIEEVIELVELNPLKHTIVGLPGVNGLSTEQRKRLTISVELVANPSIIFMDEPTSGLDARAAAVVMRAIRKIVDT
GRTVVCTIHQPSIDIFESFDELFLMKRGGQEIYVGPLGHHSYHLISYFEGIKGVRTIEDGYNPATWMLEVTTSAK
EMELGIDFAELYKNSDLYRRNKELIEELSTPAPGSKDLYFSSKYSRSFITQCMACLWKQHWSYWRNNEYTALR
FLFTIAVALLFGSIYWNLGSKIKKQQDLFNAMGSMYAAVLLLGIKNSNSAQPLVAVERTVFYREKAAGMYSAL
AYAFAQVVVELPHVLLQTVVYSAIVYAMIGFEWSVTKFFWYLFFMYFTFLYFTYYGMMSAAMTPNPSLAVIIS
SGFYEVWNLFSGFIIPRPRMPVWWRWYYWANPVAWTLYGLVTSQFGDIQDHIEFNGRSTTVEDFLRNYFGFK
HDFLGVVAAVLIGFAVTFALIFAIAIKMLNFQRR

>XP_025982896.1_PDR1_X1_Gm
MEDGGSVKAGSTTNTMSSFRIGSRSVWSNSGVEIFANSFHQEDDEEALKWAAIQKLPTFARLRTGLMTSPEGV
ANEVNVHQLGLQERRGLLERLVRVAEEDNEKFMLKLRDRIDRVGITIPTIEVRFENMNIGAEVHVGSRALPTFT
NYMVNKVEGLLNFLHVLPSRKQRINILQNVSGIIRPARMTLLLGPPSSGKTTLLLALAGRLDSKLKFTGKVTYN
GHGMNEFVPQRTAAYVSQNDLHIGEMTVRETLAFSARVQGVGARYDLLAEVSRREKEANIKPDPDIDVYMKA
VATEGQKANFITDYILRILGLEVCADTIVGNAMLRGISGGQRKRVTTGEMLVGPAKAVFMDEISTGLDSSTTFQ

VVNSLKHFIHSLKGTAVVSLLQPAPETYNLFDDIILLSDGQIVYQGPREHVLEFFASVGFKCPERKGVADFLQEV
TSRKDQEQYWVHRDQPYRFVTTEEFVEAFQSFHVGRSLADELATQFDKSKSHPAALATKMYGLGKWELLKA
CLSREYLLMKRNSFVHIFQLCQLAIVAFIAMTVFFRTEMHPDSVTSGGIYAGALFYGLLVILLDGFADLTMTVS
KLPVFYKQRDFLFFPSWVYALPAWILKIPMTFAQVGIWVFLTYYVIGFDPYVGRFFRQFLLLLFVNQMASALFR
FIGALGRELTVAFTIGSFVLAILIAMSGFILSKGNMKKWWLWGFWSSPMMYGLNAMINNEFQGKRWRHVLPN
STTPLGVQVLKSRGFFTQSKWYWIGVGALIGYTIVFNIAYILALTYLNREFLHLKKKMFCNQMREANMCTNIIT
NRLAAIVQHQAVKSEKSQSNEQDGGSTSARSSSRRKEADRRRGMALPFEPHSITFDDVTYSVDMPQEMKNQG
VLEDRLNLLKGVSGTFRPGVLTALMGSTGAGKTTLMDVLAGRKTGGYIGGNITISGYPKKQETFARISGYCEQ
NDIHSPYVTVYESLLYSAWLRLSAEINSETRKMFIEEVIELVELNPLKHTIVGLPGVNGLSTEQRKRLTISVELVA
NPSIIFMDEPTSGLDARAAAVVMRAIRKIVDTGRTVVCTIHQPSIDIFESFDELFLMKRGGQEIYVGPLGHHSYH
LISYFEGIKGVRTIEDGYNPATWMLEVTTSAKEMELGIDFAELYKNSDLYRRNKELIEELSTPAPGSKDLYFSSK
YSRSFITQCMACLWKQHWSYWRNNEYTALRFLFTIAVALLFGSIYWNLGSKIKKQQDLFNAMGSMYAAVLLL
GIKNSNSAQPLVAVERTVFYREKAAGMYSALAYAFAQVVVELPHVLLQTVVYSAIVYAMIGFEWSVTKFFWY
LFFMYFTFLYFTYYGMMSAAMTPNPSLAVIISSGFYEVWNLFSGFIIPRPRMPVWWRWYYWANPVAWTLYGL
VTSQFGDIQDHIEFNGRSTTVEDFLRNYFGFKHDFLGVVAAVLIGFAVTFALIFAIAIKMLNFQRR

>XP_014625095.1_PDR1_Gm
MEGSDTPRVDAQRVSSGFNMWRSHCMGVFSTSEREEDEETLKCVAIERLLAKARIIRRRDLNQVEGKGEEVDI
KQLELSERKSLLERLVKIPEEENERFLLKLKERMDRVGLEIPTIEVRFEHLNVEAQVYAGSRAFPTLINFFVNLLE
GFLNSLHTIRSPKKPLHILQNVSGIIKPRRMTLLLGPPSSGKTTLLLALAGRLGKDLKHSGRVTYNGHGLEEFVP
QRTSAYVSQRDNHIGEMTVRETLAFSARCQGIGQNYEILTDLLRREKEANIEPDPDIDAYMKAAALEGKQTSV
VIDYILKVLGLEVCADIMVGDEMIRGISGGQKKRLTTGEMLVGPIRVFFMDEISTGLDSSTTFQIINSIQQSIHILN
GTALVSLLQPAPETYELFDDIILLTDGQIVYQGPRENVLEFFESTGFKCPERKGVADFLQEVTSRKDQWQYWAH
KEEPYSFVTVKNFAEAFQSFHIGQQLGDELANPFDKSKCHPYALTTKNYGVKKKELLKACASREFLLMKRNSF
VYIFKATQLTYLAILTTTLFLRTKMSRNTLADAEAYMGALFFTVTVALFNGISELNMAVMKLPVFYKQRDQLF
YPSWAYSFPPWILKIPITLVEVFIWECIAYYAIGFDPNIGRLLKQYLVIFCINLMASGLFRMMAALGRNIVVANT
AGTFALLAVTAFGGFVISRKDVHKWLLWGYFSSPLMYGQAAIAVNEFLGHSWRKVSPNSNETLGVLILRSHGF
FPEAYWYWIGIGALIGYAFLFNFLFTLALQYLNPFRNYQSGLPQEKLLERNASTAEEFNQLQARKSSSDTKMEE
VGENNKATDRGKRGMVLPFQPLSLTFDEIRYSVDMPQEMKSEGISEDRLELLKGISGAFRPGVLTALMGISGAG
KTTLLDVLAGRKTSGYIEGSITISGYPKNQETFARIAGYCEQFDIHSPNVTVYESLLYSAWLRLSPKVDKATRK
MFIEEVMELVELNSLREALVGLPGETGLSTEQRKRLTIAVELVANPSIIFMDEPTSGLDARAAAIVMRTVRNTV
DTGRTVVCTIHQPSIDIFDAFDELLLLKLGGEQIYDGPIGNNSSNLIQYFEAIQGIPQIKDGYNPATWMLEVTSAA
KEANLKVDFTEVYKNSELHRRNKQLIQELSSPSQGSKDLYFDSQYSQSFVAQFIACLWKQHLSYWRNTSYTAV
RLLFTTMAGLLMGVIFLDVGKKSHRRKEQDVFNAMGSMYAAVTSIGVINGASVQPIVAIERNVFYRERAAGM
YSALPYALAQVIIELPHILAQALIYGLIVYAMMGFEWTTSKVFWYLYYTYFTFLYYTFYGMMTMAITPNPHVA
AILSTSFYAIWCLFSGFIIPLSRIPVWWKWYYWICPVSWTLYGLVASQYGDDMDKLENGQRIDEFVKSYFGFEH
DFLGVVAIVVAGFSVLFALIFAFGIKVFNFQKR

>XP_021628884.1_PDR1-l_X1_Me
MEGDLYKASSSLRRGSSSVWRNNGLDVFSRSSREEDDEEALKWAALEKLPTYDRLRKGILVSVSKGGANEIDV
ENLGFQERKALLERLVKVAEEDNEKFLLKLKNRIDRVGIEVPTIEVRYEHLNVEAEALVGSNALPSFLNFSISIV
EGLLNYLHILPNRKRPLTILKDVSGVIKPSRMALLLGPPSSGKTTLLLALAGKLDPNLKVSGNVTYNGHALNEFI
PQRTAAYISQHDLHIGEMTVRETLAFSARCQGVGTQHEMLAELSRREKAANIKPDPDLDVFMKAAATEGQETS
VVTDYVLKILGLDICADTMVGNEMIRGISGGQRKRVTTGEMLVGPAKALFMDEISTGLDSSTTFSIVNSLRQSV
HILNGTAVISLLQPAPETYNLFDDIILLSDGQIVYQGPREHVLEFFENMGFKCPERKGVADFLQEVTSKKDQQQ
YWARKDQPYRFVTVKEFAEAFKSFEVGQTITQVLSTPFDKSKNHPAALTTKPYGVGKMELLKANFSREYLLM
KRNSFVYIFKLSQVIINLIWIILFSVSKFCMWANDFSLFLRYQLIIMAIIGMTLFFRTEMSKDDLEGGGIYIGALFF
TLITIMFNGMSELSMTIAKLPVFYKQRNILFFPPWAYSIPSWILKIPITFLEVGVWVFLSYYVIGFDPNVGRLFKQ
YLLLLLVNQMASALFRFIASVGRNMIVANTFGSFALLTLFALGGVILSRENIKKWWIWGYWVSPLMYGQNSIL
ANEFLGNSWSHVPANSSSTESLGVQVLKNGGYFPHAYWYWIGVGASAGYMFLFNFLYTVALTLLDTFEKPQA
VISDEPEENDKTRGAIQLSQLESSHRTNTESGTSENNESSHNKKKGMVLPFEPHSITFDNVIYSVDMPQEMKHQ
GVVEDKLMLLKGVSGAFRPGVLTALMGVSGAGKTTLMDVLAGRKTGGYIEGDVRISGYPKQQETFARISGYC
EQNDIHSPHVTVYESLVYSAWLRLPSDVDSETRKMFVEEVMELVELNPLRQALVGLPGVNGLSTEQRKRLTIA
VELVANPSIIFMDEPTSGLDARAAAIVMRTVRNTVDTGRTVVCTIHQPSIDIFEAFDELFLMKRGGEEIYVGPLG

RHSCHLIEYFEGIEGVSKITDGYNPATWMLEVSSSAQELTLGVDFANIYRNSDLYRRNKEMIQELSKPAPGTED
LYFPTQYSQPFLTQCIACLWKQSWSYWRNPPYTAVRFWFTTFIALMFGTIFWDLGTKTERQSDLSNALGSMYA
AVLFLGLQNATSVQPVVAVERTVFYREKAAGMYSAMPYAYAQALIEIPYIFVQTVVYSVITYAMIGFEWTAAK
FFWYLFFLLFTLLYFTYYGMMTVAVTPNHHIASIVSSAFYSIWSLFSGFMIPRTKMPVWWRWYYWGCPISWTL
YGLLGSQFGDVKTMLGNTGQTVEEYVNDYYGIKHDFLGVVAGVVVGITVLFAFTFAISIKAFNFQKR

>XP_015611955.1_ABCG53_Os
MDDAGEIHALGGSLRREASSARSGDAAVFFSRSSSRDEDDEEALRWAALEKLPTYDRARTAVLAMPEGELRE
VNVQRLGPQERHALLQRLAWVGDDHARFLSKFKDRVDRVGIELPTIEVRYENLNVEAEAYVGSRGLPTILNTY
ANVLEGLANTLHITPNRKQKISILHNVSGIIKPHRMTLLLGPPGAGKTTLLLALAGNVPSGLKVSGQITYNGHT
MDEFEPRRSAAYVSQHDLHMGELTVRETVNFSAKCQGIGHRYDLLMELSRREKEENIKPDPEVDIYLKAAATG
EQKAEVVTNHILKVLGLDICADTIVGNNMLRGISGGQKKRVTTAEMIVTPGRALFMDEISTGLDSSTTYNIVDSI
RQTIHIVGGTAVIALLQPAPETYELFDDIILLSDGQVVYNGPREHVLEFFESVGFKCPERKGVADFLQEVTSRKD
QRQYWMHGDETYRYVPVKEFAEAFQSFHVGQAIRSELAIPFDKSRSHPAALKTSKYGASMKELLKANIDREIL
LMKRNSFVYIFKATQLTLMTFIAMTVFIRTNMHHDSITNGGIYMGALFFGILMIMFNGLAEVGLTIAKLPVFFK
QRDLLFYPAWTYSLPSWIIKTPLSLLNVTIWVFITYYVIGFDPNVERLFRQFLLLLVMNETSSGLFRFIAGFARHQ
VVASTMGSFCILIFMLLGGFILSRENVKKWWIWGYWISPLMYAQNAISVNEFLGHSWNKTIPGFREPLGKLVLE
SRGVFPEAKWYWIGVGALLGYVLLFNILYTICLTFLNPFDSNQPTISEETLKIKQANLTGDVIEASSRGRITTNTN
TADDSNDEAISNHATVNSSPGKKGMVLPFVPLSITFEDIRYSVDMPEVIKAQGVTESRLELLKGISGSFRPGVLT
ALMGVSGAGKTTLMDVLAGRKTSGYIEGNITISGYPKKQETFARVSGYCEQNDIHSPNVTVYESLAFSAWLRL
PAEIDSATRKMFIDEVMELVELSPLKDSLVGLPGVSGLSTEQRKRLTIAVELVANPSIIFMDEPTSGLDARAAAIV
MRAIRNTVDTGRTVVCTIHQPSIDIFESFDELFLMKRGGEEIYVGPVGQHSCELIRYFESIEGVSKIKHGYNPSTW
MLEVTSTVQEQITGVNFSEIYKNSELYRRNKSMIKELSSPPDGSSDLSFPTEYSQTFITQCLACLWKQSLSYWRN
PPYTAVKYFYTIVIALLFGTMFWGVGRKRSNQQDLFNAMGSMYASVLFMGVQNSSSVQPVVSVERTVFYRER
AAHMYSPLPYALGQVAIELPYILVQSLIYGVLVYAMIGFEWTAAKFFWYLFFMYFTLSYYTFYGMMSVGLTPS
YNVASVVSTAFYAIWNLFSGFIIPRTRIPIWWRWYYWVCPVAWTLYGLVTSQFGDVTDTFDNGVRISDFVESY
FGYHRDFLWVVAVMVVSFAVLFAFLFGLSIKIFNFQKR

>XP_014629326.1_PDR1_Gm
MEGGSSFRIGSSSIWRVDSDTNIFSNSFHQEDDEEALKWAAIQKLPTVARLRKALLTSSEGEISEIDVKKLGLQE
RRALLERLVRTVEDDNEKFLLKLRNRIDRVGIHLPTVEVRFENLNVEAEVHVGTRASPTFFNFMFNIVEGLLNF
LHILPSRKQHITIIRDVSGIIKPGRMTLLLGPPSSGKTTLLLALAAKLDPKLKFSGKVTYNGHEMNEFVPQRTAA
YVNQNDHHVAELTVRETLAFSARVQGVGTHYDLLAELSRREKEANIRPDPDIDVYMKAVATEGQKANLITDY
VLRILGLETCADTIIGNEMLRGISGGQKKRLTTGEMLVGPTKALFMDEISTGLDSSTTFQIVNSVKQCVHILKGT
AVISLLQPTPETYNLFDDIILLSDSHIVYQGPREHVLEFFKSMGFKCPERKGVADFLQEVTSRKDQEQYWADKD
QPYRFVTSKEFSEAHRSFHVGRSLVEELATEFDKSKSHPAALTTKKYGVGKWELFKACLSREYLLIKRHSFVYT
FKLSQLSVAAFVAMTVFLQTEMHRDSVIDGGIYVGALFYGLVVIMFNGMPELSMAVSRLPVFYKERDNLFFPS
WAYALPAWLLKILMSFVEVGVWVFLTYYVIGFDPYVGRFFRQYLVLVLVKQMTSALYRFVAALGRESTVALT
LGSGTNATLLAMSGFVLSKDNIKKWWLWGFWMSPTMYGQNAMVNNEFLGKRWRHILPNSTEPLGIEVLRSR
GFFTQSYWYWIGVGALIGYTLLFNFGYILALMYLSPPGKHRAVLSEEPQSNEQNGGSKKGTNVLRHIKYSLSQ
HSNKGRKGKRVSGSTSSHTLPASGMVLPFQPHSITFDEVTYAVDMPQEMRDQGVVKDKLVLLKGVSGAFRPG
VLTALMGVTGAGKTTLMDVLAGRKTGGYVGGNIKISGYRKKQETFARISGYCEQNDIHSPHVTVYESLLYSS
WLRLSLDINVETRKMFIEEVMELVELKPLRHVLVGFPGVTGLSTEQRKRLTIAVELVANPSIIFMDEPTSGLDAR
AAAIVMRIVRNTVDTGRTVVCTIHQPSMDIFESFDELFLMKQGGQEIYVGPLGHHSSHLISYFEGIQGVSEIKAG
YNPATWVLEVTNSSKEMELGIDFAEVFKNSELCRRNKELVKELSTPAPGSKDLYFPSQYSTSFFMQCMACLWK
QHRSYWRNTRYTALSFIYSTTLAVLLGSMFWNLGSKIEKQQDLLNALGSMYVAVLLIGIKNAYSVQPVVAAER
IVFYRERAAGMYSALPYAFAQVLIEIPYVLVQAVVYSLIVYAMIGFEWTVAKFFWFLFFMYFNFLCFTYYGMM
SMAVTPNQHISSIVSTGFYSAWNIFSGFIIPRPRIPVWWRWYSWANPIAWSMYGLVASQYGDIKENIESTDGTTT
VEDFVRSYFGYKHDFLGVVATVIAAFAVVFALVFAISMKMFNFQRR

>NP_001292644.1_PDR1_Csa
MDSGEIYRVSSARINSSSIWRNSAMEVFSRSSRDDDDEEALKWASIERLPTYLRVRRGILNLDGESAREIDVQNL
GLLERRNILERLVKIAEDDNERFLLKLKNRMERVGLDLPAIEVRFEHLEVEAEAHTAGRALPTMFNFSLNMLEG
FLSYFHIIPNRKKQLSILHDVSGIIKPGRMTLLLGPPSSGKTTLLKTLAGKLGKDLKFSGRVTYNGHGMNEFVPQ
RTSAYISQQDLHIGEMTVRETLSFSARCQGVGPRYDMLTELSRREKAANIKPDPDLDIIMKAAALGGQETNVVT
DYVLKILGLEICADTMVGDEMFRGISGGQKKRVTTGEMLVGPSRALFMDEISTGLDSSTTYQIVNSMRQYIHIL
NGTALISLLQPAPETYELFDDIILISDGQVVYQGPRENVLEFFQHMGFTCPQRKGVADFLQEVTSRKDQEQYWT
KRDEVYRFVSVEEFSEAFQSFHVGKKLGDELATPFDKSKSHPAALTTEKYGASKKELLKACISRELLLMKRNSF
VYIFKLIQLILMAFVTMTLFFRTEMHRRTVDDGSVYMGALFFAIIITMFNGFSELALTILKLPVFYKQRDFLFFPP
WAYSIPTWILKIPITFVEVGIWVVMTYYVVGFDPNAGRFFKHFLMLLFVNQMASALFRLIGALGRNIIVANTFG
SFALLTVLVLGGFVLARDDVHPWWIWGYWISPMMYAQNGIAVNEFLGHKWRHPAPNSNESLGVLILKSRGIF
PQASWYWIGVGATIGYILLFNFLFTIALQYLDPFEKPQAIVSKETSTDKSVKKSQDVQELELSSKGKSSSERTEN
QISLSSRTSSARVGSFSEEANQNKKRGMVLPFEPHSITFDEIRYAVDMPQEMKSQGVTEDRLELLKGVSGSFRP
GVLTALMGVSGAGKTTLMDVLAGRKTGGYIEGNITISGYPKKQETFARIAGYCEQTDIHSPHVTVYESLVYSA
WLRLPPDVDSATRKMFVEEVMELIELNPLRDAIVGLPGVSGLSTEQRKRLTIAVELVANPSIIFMDEPTSGLDAR
AAAIVMRTVRNTVDTGRTVVCTIHQPSIDIFDAFDELFLLRRGGEEIYVGPVGRHSSQLIEYFESIEGVPKIKDGY
NPATWMLEITTAAQETTLGVNFNTLYKDSELYRRNKALIKELSVPNENSNELYFPTKYSQSFFIQCIACLWKQH
LSYWRNPPYSAVRFLFTTFIALMFGTIFWDLGSKRGTQQDLFNAMGSMYAAVLFIGVQNATSVQPVVAIERTV
FYRERAAGMYSALPYAFGQVVIELPYIFIQTVVYGVIVYGMIGFEWTAAKFFWYIFFMYFTLLYFTFYGMMTV
AVTPNHNIAAIVSSAFYGFWNLFSGFIVPRTRIPIWWRWYYWICPVAWTLYGLVTSQFGDINDPMDSNQTVAE
FVSNYFGYKYDFLGVVAAVHVGITVLFGFIFAFSIKVFNFQKR

>NP_001323077.1_PDR12_At
MEGTSFHQASNSMRRNSSVWKKDSGREIFSRSSREEDDEEALRWAALEKLPTFDRLRKGILTASHAGGPINEIDI
QKLGFQDTKKLLERLIKVGDDEHEKLLWKLKKRIDRVGIDLPTIEVRFDHLKVEAEVHVGGRALPTFVNFISNF
ADKFLNTLHLVPNRKKKFTILNDVSGIVKPGRMALLLGPPSSGKTTLLLALAGKLDQELKQTGRVTYNGHGM
NEFVPQRTAAYIGQNDVHIGEMTVRETFAYAARFQGVGSRYDMLTELARREKEANIKPDPDIDIFMKAMSTAG
EKTNVMTDYILKILGLEVCADTMVGDDMLRGISGGQKKRVTTGEMLVGPSRALFMDEISTGLDSSTTYQIVNS
LRNYVHIFNGTALISLLQPAPETFNLFDDIILIAEGEIIYEGPRDHVVEFFETMGFKCPPRKGVADFLQEVTSKKD
QMQYWARRDEPYRFIRVREFAEAFQSFHVGRRIGDELALPFDKTKSHPAALTTKKYGVGIKELVKTSFSREYLL
MKRNSFVYYFKFGQLLVMAFLTMTLFFRTEMQKKTEVDGSLYTGALFFILMMLMFNGMSELSMTIAKLPVFY
KQRDLLFYPAWVYSLPPWLLKIPISFMEAALTTFITYYVIGFDPNVGRLFKQYILLVLMNQMASALFKMVAAL
GRNMIVANTFGAFAMLVFFALGGVVLSRDDIKKWWIWGYWISPIMYGQNAILANEFFGHSWSRAVENSSETL
GVTFLKSRGFLPHAYWYWIGTGALLGFVVLFNFGFTLALTFLNSLGKPQAVIAEEPASDETELQSARSEGVVEA
GANKKRGMVLPFEPHSITFDNVVYSVDMPQEMIEQGTQEDRLVLLKGVNGAFRPGVLTALMGVSGAGKTTL
MDVLAGRKTGGYIDGNITISGYPKNQQTFARISGYCEQTDIHSPHVTVYESLVYSAWLRLPKEVDKNKRKIFIE
EVMELVELTPLRQALVGLPGESGLSTEQRKRLTIAVELVANPSIIFMDEPTSGLDARAAAIVMRTVRNTVDTGR
TVVCTIHQPSIDIFEAFDELFLLKRGGEEIYVGPLGHESTHLINYFESIQGINKITEGYNPATWMLEVSTTSQEAAL
GVDFAQVYKNSELYKRNKELIKELSQPAPGSKDLYFPTQYSQSFLTQCMASLWKQHWSYWRNPPYTAVRFLF
TIGIALMFGTMFWDLGGKTKTRQDLSNAMGSMYTAVLFLGLQNAASVQPVVNVERTVFYREQAAGMYSAM
PYAFAQVLQKNIIESEIYDFSLRKPQIQTSICSRFSSRSHTFSCKR

>NP_173005.1_PDR12_At
MEGTSFHQASNSMRRNSSVWKKDSGREIFSRSSREEDDEEALRWAALEKLPTFDRLRKGILTASHAGGPINEIDI
QKLGFQDTKKLLERLIKVGDDEHEKLLWKLKKRIDRVGIDLPTIEVRFDHLKVEAEVHVGGRALPTFVNFISNF
ADKFLNTLHLVPNRKKKFTILNDVSGIVKPGRMALLLGPPSSGKTTLLLALAGKLDQELKQTGRVTYNGHGM
NEFVPQRTAAYIGQNDVHIGEMTVRETFAYAARFQGVGSRYDMLTELARREKEANIKPDPDIDIFMKAMSTAG
EKTNVMTDYILKILGLEVCADTMVGDDMLRGISGGQKKRVTTGEMLVGPSRALFMDEISTGLDSSTTYQIVNS
LRNYVHIFNGTALISLLQPAPETFNLFDDIILIAEGEIIYEGPRDHVVEFFETMGFKCPPRKGVADFLQEVTSKKD
QMQYWARRDEPYRFIRVREFAEAFQSFHVGRRIGDELALPFDKTKSHPAALTTKKYGVGIKELVKTSFSREYLL
MKRNSFVYYFKFGQLLVMAFLTMTLFFRTEMQKKTEVDGSLYTGALFFILMMLMFNGMSELSMTIAKLPVFY
KQRDLLFYPAWVYSLPPWLLKIPISFMEAALTTFITYYVIGFDPNVGRLFKQYILLVLMNQMASALFKMVAAL
GRNMIVANTFGAFAMLVFFALGGVVLSRDDIKKWWIWGYWISPIMYGQNAILANEFFGHSWSRAVENSSETL

GVTFLKSRGFLPHAYWYWIGTGALLGFVVLFNFGFTLALTFLNSLGKPQAVIAEEPASDETELQSARSEGVVEA
GANKKRGMVLPFEPHSITFDNVVYSVDMPQEMIEQGTQEDRLVLLKGVNGAFRPGVLTALMGVSGAGKTTL
MDVLAGRKTGGYIDGNITISGYPKNQQTFARISGYCEQTDIHSPHVTVYESLVYSAWLRLPKEVDKNKRKIFIE
EVMELVELTPLRQALVGLPGESGLSTEQRKRLTIAVELVANPSIIFMDEPTSGLDARAAAIVMRTVRNTVDTGR
TVVCTIHQPSIDIFEAFDELFLLKRGGEEIYVGPLGHESTHLINYFESIQGINKITEGYNPATWMLEVSTTSQEAAL
GVDFAQVYKNSELYKRNKELIKELSQPAPGSKDLYFPTQYSQSFLTQCMASLWKQHWSYWRNPPYTAVRFLF
TIGIALMFGTMFWDLGGKTKTRQDLSNAMGSMYTAVLFLGLQNAASVQPVVNVERTVFYREQAAGMYSAM
PYAFAQVFIEIPYVLVQAIVYGLIVYAMIGFEWTAVKFFWYLFFMYGSFLTFTFYGMMAVAMTPNHHIASVVS
SAFYGIWNLFSGFLIPRPSMPVWWEWYYWLCPVAWTLYGLIASQFGDITEPMADSNMSVKQFIREFYGYREGF
LGVVAAMNVIFPLLFAVIFAIGIKSFNFQKR

>XP_001292644.1_PDR1_Csa
MDSGEIYRVSSARINSSSIWRNSAMEVFSRSSRDDDDEEALKWASIERLPTYLRVRRGILNLDGESAREIDVQNL
GLLERRNILERLVKIAEDDNERFLLKLKNRMERVGLDLPAIEVRFEHLEVEAEAHTAGRALPTMFNFSLNMLEG
FLSYFHIIPNRKKQLSILHDVSGIIKPGRMTLLLGPPSSGKTTLLKTLAGKLGKDLKFSGRVTYNGHGMNEFVPQ
RTSAYISQQDLHIGEMTVRETLSFSARCQGVGPRYDMLTELSRREKAANIKPDPDLDIIMKAAALGGQETNVVT
DYVLKILGLEICADTMVGDEMFRGISGGQKKRVTTGEMLVGPSRALFMDEISTGLDSSTTYQIVNSMRQYIHIL
NGTALISLLQPAPETYELFDDIILISDGQVVYQGPRENVLEFFQHMGFTCPQRKGVADFLQEVTSRKDQEQYWT
KRDEVYRFVSVEEFSEAFQSFHVGKKLGDELATPFDKSKSHPAALTTEKYGASKKELLKACISRELLLMKRNSF
VYIFKLIQLILMAFVTMTLFFRTEMHRRTVDDGSVYMGALFFAIIITMFNGFSELALTILKLPVFYKQRDFLFFPP
WAYSIPTWILKIPITFVEVGIWVVMTYYVVGFDPNAGRFFKHFLMLLFVNQMASALFRLIGALGRNIIVANTFG
SFALLTVLVLGGFVLARDDVHPWWIWGYWISPMMYAQNGIAVNEFLGHKWRHPAPNSNESLGVLILKSRGIF
PQASWYWIGVGATIGYILLFNFLFTIALQYLDPFEKPQAIVSKETSTDKSVKKSQDVQELELSSKGKSSSERTEN
QISLSSRTSSARVGSFSEEANQNKKRGMVLPFEPHSITFDEIRYAVDMPQEMKSQGVTEDRLELLKGVSGSFRP
GVLTALMGVSGAGKTTLMDVLAGRKTGGYIEGNITISGYPKKQETFARIAGYCEQTDIHSPHVTVYESLVYSA
WLRLPPDVDSATRKMFVEEVMELIELNPLRDAIVGLPGVSGLSTEQRKRLTIAVELVANPSIIFMDEPTSGLDAR
AAAIVMRTVRNTVDTGRTVVCTIHQPSIDIFDAFDELFLLRRGGEEIYVGPVGRHSSQLIEYFESIEGVPKIKDGY
NPATWMLEITTAAQETTLGVNFNTLYKDSELYRRNKALIKELSVPNENSNELYFPTKYSQSFFIQCIACLWKQH
LSYWRNPPYSAVRFLFTTFIALMFGTIFWDLGSKRGTQQDLFNAMGSMYAAVLFIGVQNATSVQPVVAIERTV
FYRERAAGMYSALPYAFGQVVIELPYIFIQTVVYGVIVYGMIGFEWTAAKFFWYIFFMYFTLLYFTFYGMMTV
AVTPNHNIAAIVSSAFYGFWNLFSGFIVPRTRIPIWWRWYYWICPVAWTLYGLVTSQFGDINDPMDSNQTVAE
FVSNYFGYKYDFLGVVAAVHVGITVLFGFIFAFSIKVFNFQKR

>XP_002297807.2_PDR1_Pt
MESADIYRASSSLRGSFRGGSSAWRNTTVEAFSRSSREEDDEEALTWAAIEKLPTYDRLRKGILTSASKGVANE
VDIEKLGVQERKQLLERLVKVAEEDNEKFLWKLKDRVERVGIDVPTIEVRYDNLNIEAEAYVGSSALPSFAKFT
FNIIEGLLISLNILRNRKKPLTILKDVSGIVKPSRLTLLLGPPSSGKTTLLLALAGKLDPNLKFSGRVTYNGHEMN
EFVPQRTAAYISQHDLHIGEMTVRETLAFSARCQGAGYLHDMLAELSRREKEANIKPDPDVDVFMKAVASQG
EEANVITDYVLKILGLEVCADTMVGDEMIRGISGGQRKRVTTGEMLVGPSRALFMDEISTGLDSSTTYQIVNSL
RHTVHILNCTAVISLLQPAPETYDLFDDIILLSDGQIVYQGPRERVLEFFKHMGFECPERKGVADFLQEVTSRKD
QEQYWARKDQPCRFITANEFAEAFQSFSVGRRTAEELSIPFDKSKNHPAALVTKTHGAGKKDLLKANFSREYL
LMKRNSFVYIFKICQLTIMALISMTLFFRTEMHRDTVADGGIYTGALFFTAIMIMFNGMSELSMTIAKLPVFYKQ
RDLRFFPSWAYAIPQWILKIPVAFVEVGVWVFLTYYVIGFDPNVGRLFKQYLLLLLINQMASALFRFIAAAGRN
MIVANTFGSFALLTLFALGGFILSREKIKKWWIWGYWISPLMYGQTAIVVNEFLGNSWSHVPENSTEPLGIQVL
KSRGFFTEAYWYWIGAGATIGFILLLNLFFVLALTFLNAFDKPQAVISDEPESDESGRKTERAIQLSNHGSSHGT
NTEGGVGISRASSEAIGRVSNNRKKGMVLPFEPLSITFDDVIYSVDMPQEMKIQGVVEDRLVLLNGVNGAFRPG
VLTALMGVSGAGKTTLMDVLAGRKTGGYIDGEIKISGYPKKQETFARVSGYCEQNDIHSPQVTVYESLLYSAW
LRLPPEVDSETRRMFIEEVMDLVELNPLRHALVGLPGVNGLSTEQRKRLTIAVELVANPSIIFMDEPTSGLDARA
AAIVMRTVRNTVDTGRTVVCTIHQPSIDIFEAFDELFLMKRGGQEIYVGPLGRHSTHLIKYFEAIEGVSKIKDGY
NPATWMLEISSSAQEMALEVDFSNIYKNSDLFRRNKALIVELSTPAPGSTDLYFPTKYSTSFLTQCMACLWKQH
WSYWRNPPYTAVRFLFTTFIALMFGTMFWDLGSKVDSTQDLFNAMGSMYAAVIFLGVQNASSVQPVVAVER
TVFYRERAAGMYSALPYAFAQVLIELPYIFVQAAVYGIIVYAMIGFEWTVVKFFWYLFFMYFTLLYFTFYGMM
AVAMTPNHHIAAIVSSAFYGIWNLFSGFIIPRPSMPIWWRWYSWACPIAWTLYGLVVSQFGDIQKDLTETQTVK
EYVKDYFGFDHDFLGVVAAAIVGWTVLFAFIFAFAIKAFNFQRR

>XP_002527332.1_PDR1_Rco
MEGGGLYRAASSSLRRGGSSIWTNNTIPDIFSMSSREEDDEEALKWAALERLPTYDRLRKGILFSASRNGANEI
DVGSLGFHERKLLLERLLRVVEEDNEEFLLKLKNRIDRVGIELPTIEVRFENLNIEAEAFVGSRALPTFVNFSINL
FEGFLNSLHILPSRKKQLTILKDVSGVIKPSRMTLLLGPPSSGKTTLLLALAGKLDPNLKFSGNVTYNGHGMNEF
IPQSTAAYISQHDLHIGEMTVRETLSFSVRCQGVGTRNDMLVELSRREKAANIKPDPDIDVFMKAVATEGQETN
VVTDYVLKILGLEVCADTLVGDEMLRGISGGQRKRVTTGEMLVGPARALFMDEISTGLDSSTTYQIVNSLKQTI
HILDGTAVISLLQPAPETYDLFDDIILLSDGQIVYQGPREHVLEFFEYMGFKCPERKGVADFLQEVTSKNDQKQ
YWVQKDQPYSFVTVQEFSEAFQSYDVGQIIGQELSTPFDKSKSHPAALAARKYGVDKMELLKACFAREYLLM
KRNSFVYIFKLTQLVVMAIISMTLFLRTEMHREDLTDAGVYLGALFFSLIAIMFNGMSELSMTIAKLPVFYKQR
DLQFYPPWAYALPTWILKIPITFFEVGVWVFITYYVIGFDPNVERLFKQYFLLLIVNQMASGLFRFIAAVGRNMI
VANTFGSFALLTVFALGGIVLSRDDIKKWWTWGYWISPMMYGQNALVANEFLGESWNHVPANSTSTDSLGV
QFIKSRGFFPHAYWYWIGIGALTGFTILFNLCFTLALTHLNPYEKPHAVISDEPERSDRTGGAIQLSQNGSSHRTI
TENGVGIRMTDEANQNKKKGMVLPFEPHSITFNDVMYSVDMPQEMKSQGIADDKLVLLKGVSGAFKPGVLT
ALMGVSGAGKTTLMDVLAGRKTGGYIEGDIRISGYPKKQDTFARISGYCEQNDIHSPHVTVYESLIYSAWLRL
APEVDPETRKMFVNEVMELVELNPLRQALVGLPGVNGLSTEQRKRLTISVELVANPSIIFMDEPTSGLDARAAA
IVMRTVRNTVDTGRTVVCTIHQPSIDLFEAFDELFLMKRGGEEIYVGPLGRHSCHMIDYFEVIEGASKVKDGYN
PATWMLEVTSSAQELSLGVDFATIYKNSELYRRNKAIIKELSTSVPGSKDLYFPTQYSQSFLTQCIACLWKQRLS
YWRNPPYTAVRFLFTTFIALMFGTMFWDLGSKTRTQQDIFNSAGSMYAAVVFLGTQNAASVQPVVAIERTVF
YRERAAGMYSALPYAYAQVLVEIPYIFAQAVVYGLLTYSMIGFEWTAAKFFWYIFFMYFTLMYFTYYGMMA
VAVTPNHHIASVVSSAFYGIWNLFSGFIVPRTRMPVWWRWYYWVCPVSWTLYGLIGSQFSDIKDAFEGGSQT
VEDFVREYYGIRHDFLGVVAAVIVGTTVLFAFIFAVSIKSFNFQRR

>XP_002892871.1_ABCG40_Al
MEGTSFQKASNSLRRNSSVWRKDSGMEIFSRSSREEDDEEALRWAALEKLPTFDRLRKGILTASHAGGAINEID
IQKLGFQDTKKLLERLIKVGDDEHEKLLWKLKKRIDRVGIDLPTIEVRFDHLKVEAEVHVGGRALPTFVNFISN
FGDKFLNTLHLVPNRKKKFTILNDVSGIVKPGRMALLLGPPSSGKTTLLLALAGKLDLELKQTGRVTYNGHGM
NEFVPQRTAAYIGQNDVHIGEMTVRETFAYAARFQGVGSRYDMLTELARREKEANIKPDADVDVFMKAMST
AGEKTNVMTDYILKILGLEVCADTMVGDDMLRGISGGQKKRVTTGEMLVGPSRALFMDEISTGLDSSTTYQIV
NSLRNYVHIFNGTALISLLQPAPETFNLFDDIILIAEGEIIYEGPRDYVVEFFETMGFKCPPRKGVADFLQEVTSK
KDQMQYWARRDEPYRFIRVREFAEAFQSFHVGRRIGDELALPFDKTKSHPAALTTKKYGVGIKELVKTSFSRE
YLLMKRNSFVYYFKFGQLLVMAFLTMTLFFRTEMQKKTVVDGSLYTGALFFLLMMLMFNGMSELSMTIAKL
PVFYKQRDLLFYPAWVYSLPPWLLKIPISFIEAALTTFITYYVIGFDPNVGRLFKQYILLVLMNQMASALFKMV
AALGRNMIVANTFGAFAMLVFFALGGVVLSRDDIKKWWIWGYWISPIMYGQNAILANEFFGHSWSRAVPNSS
ETLGVTFLKSRGFLPHAYWYWIGTGALLGFVVLFNFGFTLALTFLNSLGKPQAVIAEEPASDETELQSARTEGV
VEASANKKRGMVLPFEPHSITFDNVVYSVDMPQEMIEQGTQEDRLVLLKGVNGAFRPGVLTALMGVSGAGKT
TLMDVLAGRKTGGYIDGNITISGYPKNQQTFARISGYCEQTDIHSPHVTVYESLVYSAWLRLPKEVDSNKRKIFI
EEVMELVELTPLRQALVGLPGESGLSTDQRKRLTIAVELVANPSIIFMDEPTSGLDARAAAIVMRTVRNTVDTG
RTVVCTIHQPSIDIFEAFDELFLLKRGGEEIYVGPLGHESTHLINYFESIQGINKITEGYNPATWMLEVSNTSQEA
ALGVDFAQLYKNSELYKRNKELIKELSQPAPGSKDLYFPTQYSQSFWTQCMASLWKQHWSYWRNPPYTAVR
FLFTIGIALMFGTMFWDLGGKTKTTQDLSNAMGSMYTAVLFLGLQNAASVQPVVNVERTVFYREQAAGMYS
AMPYAFAQVFIEIPYVFVQAVVYGLIVYAMIGFEWTAVKFFWYLFFMYGSFLTFTFYGMMAVAMTPNHHIAS
VVSSAFYGIWNLFSGFLIPRPSMPVWWEWYYWLCPVAWTLYGLIASQFGDITEPMADGTSVKQFIRDFYGYRE
GFLGVVAAMNVIFPMLFAVIFAVGIKSFNFQKR

>XP_003526427.1_PDR1_Gm
MESGELRVASARIGSSGVWRSGSIDVFSGSSRRDDDEQELKWAAIEKLPTYLRMTRGILTETEGQPTEIDINKLC
PLQRKNLVERLVKIAEQDNEKFLFKLRDRIDRVGLEIPTIEIRFEHLNVEAEAHVGSRALPTIFNFCINLFEGFLNS
LHLIPSRKKPFTVLDDVSGIIKPKRMTLLLGPPSSGKTTLLLALAGRLSKDLKFSGRVSYNGHGMEEFVPQRTSA
YISQTDLHIGEMTVRETLAFSARCQGIGTRYEMLAELSRREKAANIKPDPDLDIYMKAAALEGQETNVVTDYI
MKILGLEVCADTMVGDDMIRGISGGQKKRVTTGEMLVGPARALFMDEISTGLDSSTTFQMVNSLRQSIHILNG
TAVISLLQPAPETYELFDDIILLSDGQIVYQGPRENVLEFFEYMGFKCPERKGVADFLQEVTSRKDQEQYWANK
DEPYSFVTVKEFAEAFQSFHAGRKLGDELATPFDMSKGHPAVLTKNKFGVCKKELLKACVSREFLLMKRNSF
VYIFKMWQLILTGFITMTLFLRTEMHRDTETDGGIYMGALFFVLIVIMFNGYSELSMSIMKLPVFYKQRDLLFFP

CWAYSLPTWILKIPITLVEVGIWVVMTYYVIGFDPSIERFIKQYFLLVCINQMASGLFRFMGAVGRNIIVANTVG
SFALLAVMVMGGFILSRVDVKKWWLWGYWFSPMMYGQNALAVNEFLGKSWSHVTPNSTEPLGVKVLKSRG
IFPKAYWYWIGVGASIGYMLLFNFLFPLALHYLDPFGKPQALISEEALAERNAGRNEHIIELSSRIKGSSDKGNES
RRNVSSRTLSARVGGIGASEHNKKRGMVLPFTPLSITFDEIRYSVEMPQEMKSQGILEDRLELLKGVNGAFRPG
VLTALMGVSGAGKTTLMDVLSGRKTAGYIQGQITISGYPKRQETFARIAGYCEQTDIHSPHVTVYESLVYSAW
LRLPPEVDSSTRQMFIEEVMELVELTSLREALVGLPGVNGLSTEQRKRLTIAVELVANPSIIFMDEPTSGLDARA
AAIVMRTVRNTVDTGRTVVCTIHQPSIDIFDAFDELLLLKRGGEEIYVGPLGQHCSHLINHFEGINGVPKIKNGY
NPATWMLEVTSEAQEAALGVNFAEIYKNSDLYRRNKALIRELTTPPTGSKDLYFPTKYSQTFFTQCMACLWKQ
HLSYWRNPPYSAVRLLFTTIIALLFGTIFWDIGSKRQRKQDLFNAMGSMYAAVLFIGIQNATSVQPVVAIERTVF
YRERAAGMYSALPYAFGQVAIEIPYIFIQTLVYGVIVYAMIGFDWTFSKFFWYLFFMFFTFLYFTFYGMMAVGL
TPDHNVAAIVSFGFYMIWNLFSGFVIPRTRMPVWWRWYFWICPVSWTLYGLVTSQFGDIKEPIDTGETVEEFV
RSYFGYRDDFVGVAAAVLVGFTLLFGFTFAFSIKAFNFQKR

>XP_003528365.1_PDR1_X1_Gm
MEGSDIYRARNSLRANSSTVWRNSIMEAFSRSSRHEEDNDEEALKWAALEKLPTYNRLRKGLLTTSRGVANEI
DITELGFQERQKLLDRLINVAEEDNETLLLKLKERIDRVGIDIPTIEVRYEHLNVEAEAYVGSRALPTFLNFVTN
MVESFFTSLHILSGKKKHVTILRDVSGIIKPRRMALLLGPPSSGKTTLLLALSGKLDPTLKVSGRVNYNGHEMN
EFVPQRTAAYISQHDVHIGEMTVRETLAFSARCQGVGTRYDLLSELARREKEAKIKPDPDIDVYMKAAATGGQ
EASLVTDYVLKILGLDICADTMMGDEMLRGISGGQRKRVTTGEMLVGPANALFMDEISTGLDSSTTFQIVKSL
RQYVHILNGTAVISLLQPAPETYELFDDIVLISDGQIVYQGPREYVLEFFEYVGFQCPERKGVADFLQEVTSRKD
QEQYWIHRDESYRFVTVTEFAEAFQSFHVGRRIGEELATPFDKSKSHPAALTTKKYGVNKKELLKANFSREYL
LMKRNSFVYIFKLFQLTILAILTMTMFLRTEMHRNSLNDGGVYTGALFFAVVILMFNGLAEISMTIVKLPIFYKQ
RDLLFYPSWAYAIPSWILKIPITFIEAAVWVFLTYYVIGFDPNVGRLLKQYLVLLLINQMSSGLFRAIAALGRNM
IVASTFGSFALLVLFALGGFVLSRNDIKNWWIWGYWISPLMYGQNAIVVNEFLGDSWNHFTPNSNKTLGIQILE
SRGFFTHAYWYWIGIGALIGFMILFNIIYTLALTYLNPYDTPQTTITEESESGMTNGIAESAGRAIAVMSSSHKKK
RGMILPFEPYSITFDQIVYSVDMPLEMKDQGVREDRLVLLKGVSGAFRPGVLTALMGVSGAGKTTLMDVLAG
RKTGGYIEGNIKVSGYPKRQETFARISGYCEQNDIHSPHVTVYESLVYSAWLRLPAEVEAYTRKMFIEEVMELV
ELNPLRNSLVGLPGVNGLSTEQRKRLTIAVELVANPSIIFMDEPTSGLDARAAAIVMRTVRNTVDTGRTVVCTI
HQPSIDIFEAFDELFLMKRGGQEIYVGPLGRHSSQMIKYFESIEGVGKIKDGYNPATWMLEVTTPAQELNLGVD
FHEIYRNSGLCRRNKRLISELGNPAPGSKDLHFPTQYPQSLLVQCLACLWKQHWSYWRNPPYTAVRFLSTTVT
AVLFGTMFWDLGGKYSSRQDLFNAMGSMYNAVLFVGVQNSASVQPVVAIERTVFYRERAAGMYSALPYALA
QVIIELPYVFVQATSYSVIVYAMMGFEWTLQKFFWYVFFMYFTLCYFTFYGMMTVAVTPNHHVASVVASAFY
GIWNLFSGFVIARPSIPVWWRWYYWACPVAWTIYGLVASQFGDITNVMKSENMSVQEFIRSHLGIKHDFVGVS
AIMVSGFAVLFVIIFAVSIKAFNFQRR

>XP_003543663.1_PDR1_Gm
MEGSDIYRASNSLRRSSTAWRNSGVEVFSRSSREEDDEEALKWAALEKLPTYNRLRKGLLTASHGVANEIDVS
DLGTQERHKLLERLVKVAEEDNERFLLKLKERIDRVGLDIPTIEVRYEHLNIEAEAFVGSRALPSFINSVTNIIEG
FFNLLHITTSKKKHVTILKDVSGIIKPRRMTLLLGPPSSGKTTLLLALSGKLDKTLKVSGRVTYNGHELNEFVPQ
RTAAYISQHDLHIGEMTVRETLAFSARCQGVGSRYDMLSELSRREKAANIKPDPDLDVYMKATATEGQESSIV
TDYTLKILGLDICADTMVGDEMLRGISGGQRKRVTTGEMLVGPANALFMDEISTGLDSSTTFQIVNSLRQYVHI
LNGTAVISLLQPAPETYDLFDDIILISDGQVVYHGPREYVLDFFESMGFRCPERKGVADFLQEVTSKKDQAQYW
ARRDQPYRFVKVTQFAEAFQSFHIGRKLGEELVVPFDKTKSHPAALTTKKYGINKKELLKANLSREYLLMKRN
SFVYIFKLCQLSIMALMTMTLFLRTELHRNNMDDAGLYSGALFFTLIMIMFNGMAEISMTIAKLPVFYKQRDLL
FYPSWAYAIPSWILKIPVTLLEVAVWVFLTYYVIGFDPNVGRFFKQYLILLFIGQMASALFRAIAALGRNMIVSN
TFGAFAVLTFLTLGGYVMSKNDIKNWWIWGYWISPLMYGQNALMVNEFLSNSWHNTSRNLGVEYLESRGFP
SSSYWYWLGLGAMAGFVLLFNVMFSAALEILGPFDKPQATITEEESPNEGTVAEVELPRIESSGRGDSVVESSH
GKKKGMVLPFEPHSITFDEVIYSVDMPQEMKEQGVQEDRLVLLKGVSGAFRPGVLTALMGVSGAGKTTLMD
VLAGRKTGGYIDGSIKISGYPKKQETFARISGYCEQNDIHSPHVTVYESLLYSAWLRLPSGVDSKTRKMFIEEV
MELVELNPLRNSLVGLPGVSGLSTEQRKRLTIAVELVANPSIIFMDEPTSGLDARAAAIVMRTVRNTVDTGRTV
VCTIHQPSIDIFEAFDELFLMKRGGQEIYVGPLGRHSTHLIKYFESIGGVSKIKDGYNPATWMLEVTTSAQELSL
GVDFTDLYKNSDLYRRNKQLIQELGQPAPGSKDLYFPTQYSQSFLVQCQACLWKQRWSYWRNPPYTAVRFFF
TTFIALMFGTMFWDLGSRRTTRGDLLNALGSMYSAVLFLGIQNASSVQPVVAVERTVFYREKAAGMYSALPY
AFAQVLVEIPYIFAQAVTYGLIVYAMIGFDWTAEKFFWYLFFSFFSLLYFTFYGMMAVGVTPNHHVAAIVAAA

FYAIWNLFSGFIVVRPKMPVWWRWYYWACPVAWTLYGLIASQFGDITERMPGEDNKMVKEFIEDYFGFKHD
FVGICAVVVAGIAVAFALIFGAAIKTFNFQKR

>XP_003546218.1_PDR1_Gm
MEGSDIYRASNSLRRSSTVWRNSGVEVFSRSSREEDDEEALKWAALEKLPTYNRLRKGLLTASHGVANEIDVS
DLGIQERQKLLERLVKVAEEDNERFLLKLKERIDRVGLDIPTIEVRYEHLNIEAEAFVGSRALPSFINSVTNVVEG
FFNLLHISTSKKKHVTILKDVSGIIKPRRMTLLLGPPSSGKTTLLLALSGKLDKTLKVSGRVTYNGHELNEFVPQ
RTAAYISQHDLHIGEMTVRETLAFSARCQGVGSRYDMLSELSRREKAANIKPDPDLDVYMKATATEGQESSLV
TDYTLKILGLDICADTMVGDEMLRGISGGQRKRVTTGEMLVGPANALFMDEISTGLDSSTTFQIVSFLRQYVHI
LNGTAVISLLQPAPETYDLFDDIILISDGQVVYHGPREYVLDFFESMGFRCPERKGVADFLQEVTSKKDQAQYW
ARRDQPYRFVTVTQFSEAFQSFHIGGKLGEELAVPFDKTKSHPAALTTKKYGINKKELLKANLSREYLLMKRN
SFVYIFKLCQLSIMALMTMTLFLRTELHRNNMDDAGLYAGALFFTLVMIMFNGMAEISMTIAKLPVFYKQRDL
LFYPSWAYAIPSWILKIPVTLLEVAVWVFLTYYVIGFDPNVGRLFKQYLILLFIGQMASALFRAIAALGRNMIVS
NTFGAFAVLTFLTLGGFVMAKSDIKNWWIWGYWISPLMYGQTALMVNEFLSNSWHNSSRNLGVEYLESRGFP
SSAYWYWLGLGAMAGFVLLFNVMFSAALEILGPFDKPQATIAEEESPNEVTVAEVELPRIESSGRGGSVVESSH
GKKKGMVLPFEPHSITFDEVVYSVDMPQEMKEQGVQEDRLVLLKGVSGAFRPGVLTALMGVSGAGKTTLMD
VLAGRKTGGYIDGNIKISGYPKKQETFARISGYCEQNDIHSPHVTVYESLLYSAWLRLPSSVDSQTRKMFIEEV
MELVELNPLRNSLVGLPGVSGLSTEQRKRLTIAVELVANPSIIFMDEPTSGLDARAAAIVMRTVRNTVDTGRTV
VCTIHQPSIDIFEAFDELFLMKRGGQEIYVGPLGRHSSHLIKYFESIEGVSKIKDGYNPATWMLEVTTSAQELSLG
VDFTDLYKNSDLYRRNKQLIQELGQPAPGSKDLYFPTQYSQSFLVQCQACLWKQRWSYWRNPPYTAVRFFFT
TFIALMFGTMFWDLGSRRTTRGDLLNALGSMYTAVLFLGIQNASSVQPVVAVERTVFYREKAAGMYSALPYA
FAQVLVEIPYIFAQAVTYGLIVYAMIGFDWTAEKFFWYLFFSFFSLLYFTFYGMMAVGVTPNHHVAAIVAAAF
YAIWNLFSGFIVVRPKMPVWWRWYYWACPVAWTLYGLIASQFGDITERMPGEDNKMVKDFVEDYFGFKHDF
VGVCAVVVAGIAVAFALIFGVAIKTFNFQKR

>XP_003546230.1_PDR1_Gm
MEGSDIYRASNSLRSRSSTVWRNSGVEAFSRSSREEDDEEALKWAALEKLPTYNRLRKGLLTASHGVANEIDV
SDLGIQERQKLLERLVKVAEEDNERFLLKLKERIDRVGLDIPTIEVRYEHLNIEAEAFVGSRALPSFINSVTNVVE
GFFNLLHVSTSKKKHVTILKDVSGIIKPRRMTLLLGPPSSGKTTLLLALSGKLDKTLKVSGRVTYNGHELNEFVP
QRTAAYISQHDLHIGEMTVRETLAFSARCQGVGSRYDMLSELSRREKAANIKPDPDLDVYMKATATEGQESNI
VTDYTLKILGLDICADTMVGDEMLRGISGGQRKRVTTGEMLVGPANALFMDEISTGLDSSTTFQIVSSLRHYVH
ILNGTAVISLLQPAPETYDLFDDIILISDGQVVYHGPREYVLDFFESMGFRCPERKGVADFLQEVTSKKDQAQY
WVRRDQPYRFVTVTQFAEAFQSFHIGGKLGEELTVPFDRTKSHPAALTTKKYGINKKELLKANFSREYLLMKR
NSFVYLFKLSQLFIMALVAMTLFLRTEMHHENMDDAGVYAGAVFFMLITVMFNGLAEISMTIAKLPVFYKQR
NLLFYPSWAYAIPSWILKIPVTIVEVAVWVFLTYYVIGFDPNVGRFFKQYLVLLIVSQMASGLFRTIAALGRNMI
VANTFGAFAIITVVALGGFILSKRDIKSWWIWGYWISPLMYGQNALMVNEFLSNSWHNATHNLGVEYLESRAF
FTDSYWYWLGLGALVGFVFLFNVMFGLALEFLGPFDKPQATITEDESSNEGTLADIELPGIESSGRGDSLVESSH
GKKKGMVLPFEPHSITFDEVVYSVDMPQEMKEQGVQEDRLVLLKGVSGAFRPGVLTALMGVSGAGKTTLMD
VLAGRKTGGYIDGSIKISGYPKKQETFARISGYCEQNDIHSPHVTVYESLLYSAWLRLPSSVDSKTRKMFIEEVM
ELVELNPVRNSLVGLPGVSGLSTEQRKRLTIAVELVANPSIIFMDEPTSGLDARAAAIVMRTVRNTVDTGRTVV
CTIHQPSIDIFEAFDELFLMKRGGQEIYVGPLGRHSSHLIKYFESIEGVSKIKDGYNPATWMLEVTATAQELSLG
VDFTDLYKNSDLYRRNKQLIQELGQPAPGSKDLHFPTQYSQSFLVQCQACLWKQRWSYWRNPPYTAVRFFFT
TFIALMFGTIFWDLGGKHSTRGDLLNAIGSMYTAVLFLGVQNASSVQPVVAIERTVFYREKAAGMYSALPYAF
AQILVELPYVFVQAVTYGVIVYAMIGFEWTAEKFFWYLFFMYFTLLYYTFYGMMTVGLTPNHHIASIVAAAFY
AVWNLFSGFVVTRPSIPVWWRWYYWACPVAWTIYGLVASQFGDLTEPMTSEGQKIVKDFLEDYYGIKHDFIG
VSAVVVAGIAVLFALIFAVSIKTFNFQKR

>XP_003597816.3_PDR1_X1_Mt
MDWLFNDGQPKEAMKAMKCYESLLNHKFSMSSQPSNSLLKVLFRHDKKTMMLDDHRSVHQYCLEKKNKKH
KLVLRLLNKAKMEGTDIYRATNSLRARSSTVWRQSGVEVFSKSSREEDDEEALKWAALEKLPTYNRLRKGLL
TASHGGAHEVDVGDLAFKEKQKLLERLVRVAEEDNEGFLLKVKERVDRVGLDIPTIEVRYQNLKIDAEAFVGS
RALPSFINAATNVVEGVFNFLHIIPTKKRHVAILRDVSGIIKPRRMTLLLGPPGSGKTTLLLALSGKLDSSFQLSG
NVTYNGHGLNEFVPQRTAAYISQHDVHIGEMTVRETLAFSARCQGVGSRYDMLSELSRREKVANIKPDPDIDV
YMKAIATEGQESSISTDYVLKILGLDICADTMVGDEMLRGISGGQRKRVTTGEMLVGPANALFMDEISTGLDSS

TTFQIVSSLRQYVHIMNGTAVISLLQPAPETYDLFDDIILISDGQVVYHGPREYVLDFFESMGFKCPERKGVADF
LQEVTSKKDQAQYWVRRDQPYRYVTVTQFAEAFQSFHIGGKLAEELSIPFDKTKSHPAALTTKEYGLNKTELL
KANFSREYLLMKRNSFVYIFKLTQLFIMALIAMTLFFRTEMHRDDQDDAGVYAGALFFTLVTMMFNGMSEIS
MTIAKLPVYYKQRDLLFYPSWAYAIPSWILKIPVSLVEVSLWVFLTYYVIGFDPNVGRMFKQFVVLFFMSQMA
SGLFRAIASLGRNMIVANTFGSFAVLTLFALGGFILSRKDIKSWWIWGYWISPMMYGQNALMANEFLANSWH
NATSDLGKDYLDTRGFFPHAYWYWIGVGGLAGFVFLFNAAFGVALAVLGPFDKPSATITDNSEDDSSNYMTA
QEVELPRIESSGRGDSVTVSSHGKKKGMVLPFEPHSITFDDIVYSVDMPAEMKEQGVTEDRLVLLKGVSGAFRP
GVLTALMGVSGAGKTTLMDVLAGRKTGGYIDGDIKVSGYPKKQETFARISGYCEQNDIHSPHVTVYESLLYSA
WLRLPSGVDSNTRKMFIEEVMDLVELNSLRDSLVGLPGVSGLSTEQRKRLTIAVELVANPSIIFMDEPTSGLDA
RAAAIVMRTVRNTVDTGRTVVCTIHQPSIDIFEAFDELFLMKRGGQEIYVGPLGRHSTHLIKYFESIDGVSKIKD
GYNPATWMLEVTTTAQELNLGVDFTDLYKNSDLYRRNKQLIQELGVPAPGSKDLHFPTQFSQSFLVQCQACL
WKQRWSYWRNPPYTAVRFFFTTFIALMFGTMFWDLGGKHSRRQDLLNAVGSMYTAVLFLGVQNSSSVQPVV
AVERTVFNREKAAGMYSALPYAFSQILVELPYVFAQAVTYGVIVYAMIGFDWTAEKFLWYLFFMYFTLLYFTF
YGMMAVAVTPNHHVASIVAAAFYAIWNLFSGFVVPRPSIPIWWRWYYWACPVAWTIYGLVASQFGDITTVM
TTEGGKDVKTFLDDFFGIQHDFIGWCALVVGGIAVAFAFIFAVAIKSFNFQKR

>XP_003597818.1_PDR1_X2_Mt
MEGTDIYRATNSLRARSSTVWRQSGVEVFSKSSREEDDEEALKWAALEKLPTYNRLRKGLLTASHGGAHEVD
VGDLAFQEKQKLLERLVKVAEEDNERFLLKVKERVDRVGLDIPTIEVRYQNLKIDAEAFVGSRALPSFINAATN
VVEGVLNFLHIIPTKKRHVSILKDVSGIVKPRRMTLLLGPPGSGKTTLLLALSGKLDPSLQLTGSVTYNGHGLNE
FVPQRTAAYISQHDVHIGEMTVRETLAFSARCQGVGSRYDMLSELSRREKAANIKPDPDIDVYMKAIATEGQE
YSISTDYVLKILGLDICADTMVGDEMLRGISGGQRKRVTTGEMLVGPANALFMDEISTGLDSSTTFQIVSSLRQ
YVHIMNGTAVISLLQPAPETYDLFDDIILISDGQVVYHGPREYVLDFFETMGFKCPERKGAADFLQEVTSKKDQ
AQYWVRRDQPYRFVTVTQFAEAFQSFHIGRKLAEELSVPFDKTKSHPAALTTKEYGLNKTELLKANFSREYLL
MKRNSFVYIFKLTQLFIMALIAMTLFFRTEMHRNDQDDAGVYAGALFFTLVTMMFNGMSEISMTIAKLPVYYK
QRDLLFYPSWAYAIPSWILKIPVSLMEVSLWVFLTYYVIGFDPNVGRMFKQFVVLFFMSQMASGLFRAIASLGR
NMIVANTFGSFALLTFLSLGGFILSRKDIKGWWIWGYWISPLMYGQNALMANEFLGHSWHNATADLGKDYLD
TRGFFPHAYWYWIGVGGLVGFVFLFNVAFGVALAVLGPFDKPSATITEDSEDDSSTVQEVELPRIESSGRADSV
TESSHGKKKGMVLPFEPHSITFDDIVYSVDMPVEMKEQGVREDRLVLLKGVSGAFRPGVLTALMGVSGAGKT
TLMDVLAGRKTGGYIDGDIKVSGYPKKQETFARISGYCEQNDIHSPHVTVYESLLYSAWLRLPSGVDSNTRKM
FIDEVMDLVELNSLRNSLVGLPGVSGLSTEQRKRLTIAVELVANPSIIFMDEPTSGLDARAAAIVMRTVRNTVD
TGRTVVCTIHQPSIDIFEAFDELFLMKRGGQEIYVGPLGRHSTHLIKYFESIDGVSKIKDGYNPATWMLEVTTTA
QELNLGVDFTDLYKNSDLYRRNKQLIQELSVPAPGSKDLHFPTQFSQSFLVQCQACLWKQRWSYWRNPPYTA
VRFFFTTFIGLMFGTMFWDLGGKHSSRQDLLNAVGSMYTAVLFLGVQNSSSVQPVVAVERTVFYREKAAGM
YSALPYAFSQILVELPYVFAQAVIYGVIVYAMIGFDWTAEKFLWYLFFMYFTLLYFTFYGMMAVAVTPNHHV
ASIVAAAFYAIWNLFSGFVVPRPSIPIWWRWYYWACPVAWTIYGLVASQFGDITTVMSTEGGKDVKTFLDDFF
GIQHDFIGWCALVVGGIAVGFAFIFAVAIKSFNFQKR

>XP_004239864.1_PDR1-l_Sl
MEPVNLNSMRGSSMRGSMRGSLRASTSNSIWRNNGVDAFSRSTRDEDDEEALKWAALEKLPTFDRLRKGLLF
GSQGAANEIDVNDLGYQERKNLLERLVKVADEDNEKFLMKLKNRIDRVGIDMPSIEVRYEHLNIEADAYAGSR
ALPTFINFMTNFVETLLNSLHILPSKKRQITILKDVSGMIKPCRMTLLLGPPSSGKTTLLLALAGKLDPALRVTGN
VTYNGHELHEFVPQRTAVYISQHDLHIGEMTVRETLEFSARCQGVGSRFEMLAELSRREKAANIKPDPDIDIYM
KAAATEGQEANVVTDYVLKILGLDICADTMVGDEMIRGISGGQKKRVTTGEMLVGPSKALFMDEISTGLDSST
TFSIVNSLRQSVQLLKGTAVISLLQPAPETYNLFDDIILLSDGYIVYQGPREAVLDFFESMGFKCPERKGAADFL
QEVTSKKDQQQYWAKRNEPYRFITSKEFSEAYQSFHVGRKLSDELATPYDKTKSHPAALSTKKYGIGTKQLLK
VCAEREFLLMKRNSFVYIFKLTQLAIMALITMSVFFRTKLPRDDMDDGGIYAGALFFVVVMIMFNGMAEIALTI
FKLPVYFKQRDLLFFPSWAYALPTWILKIPITFVECGMWTFLTYYVMGFDPNVSRLFKQFLLLVLVHQMASAL
FRFIGAVGRTMGVASTFGAFALLLQFALGGFVLAREDVKKWWIWGYWTSPLMYSVNSILVNEFDGKNWKHI
APNGTEPLGAAVVRSRGFFPDAYWYWIGCGALFGFTMIFNFFYSIALAYLDPFGKPQAMISEDGEDAVELTERS
ETEGQDKKKGMVLPFEPHSITFDNIVYSVDMPQEMKEQGSAEDRLVLLKGVSGAFRPGVLTALMGVSGAGKT
TLMDVLAGRKTGGYIDGDIKISGYPKKQETFARISGYCEQNDIHSPYVTVYESLVYSAWLRLPQDVDENKRKM
FVDEVMELVELAPLRSALVGLPGVNGLSTEQRKRLTIAVELVANPSIIFMDEPTSGLDARAAAIVMRAVRNTVD
TGRTVVCTIHQPSIDIFEAFDELFLMKRGGQEIYVGPLGRHSCHLIKYFESMPGVGKIKEAYNPATWMLEVTAS

SQEMMLGVDFADLYKNSDLYRRNKALIAELSTPRPATKDLHFETQFSQPFWTQCMACLWKQHWSYWRNPAY
TAVRFIFTTFIALVFGTMFWDLGTKVSRSQDLINAMGSMYAATLFLGVQNSSSVQPVVAVERTVFYREKAAG
MYSAIPYAFGQVVIEIPYVFVQSAFYGVIVYAMIGFEWTAVKFLWYFFFMYCTLLYFTFYGMMTVAVTPNQN
VASIVAAFFYAVWNLFSGFIVPRPRIPIWWRWYYWACPVAWTLYGLVASQFGDIQTPLTDDENVEQYLRRYF
GFKHDFLGVVAAVIVALPVMFALTFALGIKAFNFQRR

>XP_004245224.1_PDR1-l_Sl
MEGGENLVRVSSARLSGSNVWRNSAMDVFSRSSSREDYDDEEALRWAALEKLPTYSRIRRGLLLEEEGQSREV
DITKLDLIERRNLLDRLVKIADEDNEKLLMKLKQRIDRVGLDLPTIEVRFEHLNVDAEARVGSRALPTIFNFTVN
ILEDFLNYIHILPSRKKPLPILHGVSGIIKPGRMTLLLGPPSSGKTTLLLGLAGKLDKDLKVSGRVTYNGHGMDE
FVPQRTSAYISQNDLHIGEMTVRETLAFSARCQGVGDKYEILAELSRREKEANIKPDPDVDIFMKSAWNEGQEA
NVITDYTLKILGLEICADTLVGDEMIRGISGGQRKRLTTGEMMVGPARALFMDEISTGLDSSTTYQIVNSIRQSIH
ILQGTAVISLLQPAPETYDLFDDIILLSDGKIVYQGPRENVLEFFEYMGFKCPERKGVADFLQEVTSRKDQEQY
WSRRDEPYRFITSCEFSDVFQSFHVGRKLGEELAVPFDKSKSHPAALTTKRYGISKKELLKACAAREYLLMKRN
SFVYIFKMVQLTMMASIAMTLFLRTEMHRDTTIDGAVYLGALFYAVITVMFNGFSELALSIMKLPSFYKQRDL
LFFPAWAYALPTWILKIPITLVEIAIWVCMTYYVIGFEADVGRFFKQLFLLICLNQMASGLFRFLAALGRNIIVA
NTFGSCALLVVLVMGGFILSRDDVKQWLIWGYWTSPMMYAQNAIAVNEFLGKSWSHVPPNSTGTDTLGVSFL
KSRGIFPEARWYWIGAGALIGYVLLFNFLFTVALAYLNPFGKPQAIISEEIVVERIASKRGEVIELSPIGKSSSERG
NDVAISASSRSLSSRVGNITEGDLSKRRGMILPFEPLSITFDDIRYAVDMPQEMKAQGFIEDRLELLKGVSGAFRP
GVLTALMGVSGAGKTTLMDVLAGRKTGGYINGTISISGYPKQQETFARISGYCEQTDIHSPHVTVYESLQYSA
WLRLPREVDTETRKNFIEEVMELVELIPLREALVGLPGVNGLSTEQRKRLTVAVELVANPSIIFMDEPTSGLDAR
AAAIVMRTVRNTVDTGRTVVCTIHQPSIDIFDAFDELLLLKRGGEEIFVGPLGRHSSHLIKYFEGIDGVLKIRDGY
NPATWMLEVTSLAQEAVLGIDFTELYKNSELYRRNKALIQELSVAAPGSKDLYFETEYSQSFFTQCMACLWKQ
HLSYWRNPPYTAVRLMFTFFVSLMLGTIFWGLGSKRGRQQDILNAIGSMYSAILFLGIINATSVQPVVAIERTVF
YRERAAGMYSALPYAFGQVMIELPHLFLQTIIYGVIVYAMIGFEWTVAKFFWYLFFMYFTLLYFTLYGMMTV
AVTPNHTIASIVSSAFYTIWNLFCGFVVPKTRMPVWWRWYYYVCPLSWTLYGLIASQFGDVQDKLDTKETVE
QFLENFFDYKHDFVGYVAVILVGISVAFLFIFAYSIKAFNFQKR

>XP_004245225.1_PDR1-l_Sl
MEGGGDILKVSSARLGSSTVWRNSGVDVFSRSSREDYDDEEALKWAALEKLPTYLRIRRGILSEEEGQYREVDI
TKLDLVERRNLLERLVKIADEDNEKFLLKLKKRIDRVGLDLPTIEVRFEHLNVDAEARVGSRALPTIFNFTVNIIE
DFLNYLHILPSRKKPLPILHEISGIIKPGRMTLLLGPPSSGKTTLLLGLAGKLDKDLKVSGRVTYNGHGMDEFVP
QRTSAYISQNDLHIGEMTVRETLAFSARCQGVGAKYEILAELSRREKEANIKPDPDVDIFMKSAWNDGQEANV
VTDYTLKILGLEICADTIVGDEMIRGISGGQRKRLTTGEMMVGPARALFMDEISTGLDSSTTYQIVNSIRQSIHIL
QGTAVISLLQPAPETYDLFDDIILLSDGQIVYQGPRENVLEFFEYIGFKCPQRKGVADFLQEVTSRKDQEQYWA
RRDEPYKFITVREFSEAFQSFHVGRKLGDELAVPFDKSKSHPAALTTERYGVSKKELLKACTAREYLLMKRNSF
VYIFKMIQLTLMATITMTLFLRTEMHRDTMIDGAVFLGALYYAVIMIMFNGFSELALSIMKLPSFYKHRDLLFF
PAWTYALPTWILKIPITLVEVAIWVCMTYYVIGFEADVGRFFKQLFLLICLNQMASGLFRFLAALGRNVIVANT
FGSCALLIVLVMGGFILSRDNVKQWLIWGYWISPMMYAQNAIAVNEFLGKSWAHVPPNSTGTDTLGVSFLKS
RGIFPEARWYWIGVGALLGYVLLFNFLFTVALAYLNPFGKPQAVLSEETVAERNASKRGEVIELSPIGKSSSERG
NDVRRSASSRSMSSRVGNIAEGDLNKRKGMILPFEPLSITFDDIRYAVDMPQEMKAQGFTEDRLELLKGVSGAF
RPGVLTALMGVSGAGKTTLMDVLAGRKTGGYIEGTISISGYPKQQATFARIAGYCEQTDIHSPHVTVYESLQYS
AWLRLPREVDTETRKRFIEEVMELVELKPLREALVGLPGVNGLSTEQRKRLTVAVELVANPSIIFMDEPTSGLD
ARAAAIVMRTVRNTVDTGRTVVCTIHQPSIDIFDAFDELLLLKRGGEEIFVGPLGRHSSHLIKYFEGIDGVLKIKD
GYNPATWMLDITSVAQEAALGIDFTELYRNSELYRRNKALIQELSVPAPGSKDLYFETKYSQSFFTQSMACFW
KQHWSYWRNPPYTAVRLMFTFFIALMFGTIFWDLGSKRRRQQDILNAIGSMYAAVLFLGVQNATSVQPVVAIE
RTVFYRERAAGMYSALPYAFGQIMIELPYIFIQTIIYGVIVYAMIGFEWTVAKFIWYLFFMYFTLLYFTLYGMMT
VAVTPNHSIAAIISSAFYAVWNLFSGFIVPKTRMPVWWRWYFYICPISWTLYGLVASQFGDLQDKLETKETVEE
FIESFFDFKYDFVGYVALILVGISVGFLFIFAYSIKAFNFQKR

>XP_004247841.1_PDR1_Sl
MEPANLGNLRGSSLRGSISGSRRGSVSLRANSNSIWRNTGVEIFSRSARDEDDEEALKWAALEKLPTFDRLRKG
LLFGSQGAAAEIDIDDIGLQERKNLLERLVRVADEDNEKFLLKLKNRIDRVGIDLPTIEVRYENLNIEADAYVGS
RGLPTFINFMTNFLETLLNTLHILPSSKRQITILKDISGIIKPCRMTLLLGPPSSGKTTLLLALAGKLDSSLKVTGK

VSYNGHELHEFVPQRTAAYISQHDLHIGEMTVRETLEFSARCQGVGSRYEMLAELSRREKAANIKPDPDIDIYM
KASATEGQEANVVTDYVLKILGLDICADTMVGDEMLRGISGGQKKRVTTGEMLVGPSKALFMDEISTGLDSST
TYSIVNSLRQSVQILKGTAVISLLQPAPETYNLFDDIILISDGYIVYQGPRDDVLQFFESMGFKCPERKGVADFLQ
EVTSKKDQPQYWSRRNEHYRFISSKEFSDAYQSFHVGRKLGDELAIPFDRTKCHPAALTNEKYGIGKKELLKV
CTEREYLLMKRNSFVYVFKFFQLTIMALMTMTLFFRTEMPRDTVDDGGIYAGALFFVVVMIMFNGMSEMAM
TIFKLPVFYKQRDLLFFPSWAYAIPSWILKIPVTLVEVGLWVILTYYVIGFDPNITRFLKQFMLLVLVNQMASGL
FRFMGAVGRTMGVASTFGAFALLLQFALCGFVLSREDVKGWWIWGYWISPLMYSVNSILVNEFDGSKWKHIA
PNGTEPLGVAVVKSRGFFPDAYWYWIGFAALFGFTVVFNFFYSLSLAYLKPYGKSQTVRPEDSGNAENGQAAS
QMTSTDGGDIVSAGQSKKKGMVLPFEPHSITFDDVVYSVDMPQEMKEQGAGEDRLVLLKGVSGAFRPGVLTA
LMGVSGAGKTTLMDVLAGRKTGGYIDGDIKISGYPKKQETFARISGYCEQNDIHSPYVTVYESLVYSAWLRLP
KDVDEKTRKMFVDEVMELVELEPLRSALVGLPGVNGLSTEQRKRLTIAVELVANPSIIFMDEPTSGLDARAAAI
VMRTVRNTVDTGRTVVCTIHQPSIDIFEAFDELFLMKRGGQEIYVGPLGRHSCHLIKYFESIPGVAKIKEGYNPA
TWMLEVTASAQEMMLGVDFTDLYKNSDLYRRNKALITELSVPRPGSKDLYFETQYSQSIWIQCMACLWKQN
WSYWRNPAYTAVRFIFTMFIALVFGTMFWDIGTKVSQSQDLFNAMGSMYAAVLFLGVQNASSVQPVVDVER
TVFYRERAAGMYSAIPYAFGQVFIEIPYVFVQAIVYGIIVYAMIGFEWEAGKFFWYLFIMFTTLLYFTFYGMMS
VAVTPNQNVASIVAAFFYAIWNLFSGFIVPRPRMPIWWRWYYWCCPVAWTLYGLVASQFGDIQSRLTDEETV
EQFLRRYFGFRHDFLPVVAGVLVAYVVVFAFTFAFAIKAFNFQRR

>XP_004486706.1_P_PDR1-l_Ca
MEGTDIYRATNSLRARSSTVWRNSGVEVFSRSSREEDDEEALKWAALEKLPTYNRLRKGLLTTSHGAANEIDV
TDLGYQEKQKLLERLVKVAEEDNEGFLLKLKERVDRVGLDIPTIEVRYEHLKIDAEAFVGGRALPSFINAATNV
VEGLLNVLHIVPSKKRHVAILKDVSGVIKPRRMTLLLGPPGSGKTTLLLALSGKLDTTLQLSGSVTYNGHGLNE
FVPQRTAAYISQHDVHIGEMTVRETLAFSARCQGVGSRYDMLSELSRREKAANIKPDPDVDVYMKAIATEGQE
SSISTDYVMKILGLDICADTMVGDEMLRGISGGQRKRVTTGEMLVGPANALFMDEISTGLDSSTTFQIVSSLRQ
YVHIMNGTAVISLLQPAPETYDLFDDIILISDGQVVYHGPREYVLDFFESMGFKCPERKGVADFLQEVTSKKDQ
AQYWVRRDQPYRFVTVTQFAEAFQSFHIGRKLTEELSVPFDKTKSHPAALTTKEYGLNKKELLKANFSREYLL
MKRNSFVYIFKLCQLFVMAFIAMTLFFRTEMHRDNQDDAGVYAGALFFTLVTIMFNGMSEISMTIAKLPVFYK
QRDLLFYPSWAYAIPSWILKIPITLMEVSLWVFLTYYVIGFDPNVGRFFKQFLVLFFMSQMASGLFRATAALGR
NMIVANTFGSFAVLTLLALGGFILSRKDIKGWWIWGYWISPLMYGQNALMANEFLGNNWHNATFDLGKQYL
DNRGFFPHAYWYWIGVGGLVGFVFFFNALFGVALAVLGPFDKPQATITEDSEEVVEEVELPRIESSGRGDSVTE
SSHGKKKGMVLPFEPHSITFDDIVYSVDMPVEMKEQGVQEDRLVLLKGVSGAFRPSVLTALMGVSGAGKTTL
MDVLAGRKTGGYIDGSIKVSGYPKKQETFARVSGYCEQNDIHSPHVTVYESLLYSAWLRLASGVDSKTRKMFI
EEVMDLVELNSLRNSLVGLPGVSGLSTEQRKRLTIAVELVANPSIIFMDEPTSGLDARAAAIVMRTVRNTVDTG
RTVVCTIHQPSIDIFEAFDELFLMKRGGQEIYVGPLGRHSTHLIKYFESIEGVSKIKDGYNPATWMLEVTTTAQE
HNLGVDFTDYYKNSDLYRRNKQLIQELGQPAPGSKDLHFPTQFSQSFLVQCQACLWKQRWSYWRNPPYTAV
RFFFTTFIAIMFGTMFWDLGGKHSSRQDLLNAVGSMYTAVLFLGVQNSSSVQPVVAVERTVFYREKAAGMYS
ALPYAFSQILVELPYVFAQAVTYGVIVYAMIGFDWTAEKFFWYLFFMYFTLLYFTFYGMMAVAVTPNHHVAS
IVAAAFYAIWNLFSGFVVPRPSIPIWWRWYYWACPVAWTIYGLVASQFGDITTVMQEEHKDVKTFLDDFFGIK
HDFIGECAVVVGGIAVAFAVIFAVAIKTFNFQKR

>XP_004486707.1_P_PDR1-l_Ca
MEGTDIYRATNSLRARSSTVWRNSGVEVFSKSSREEDDEEALKWAALEKLPTYNRLRKGLLTTSHGAANEIDV
TDLDFQEKQKLLERLVKVAEEDNEGFLLKLKERVDRVGLDLPTIEVRYEHLKIDAEAFVGSRALPSFINAATNV
VEGLLNVLHIVPSKKRHVAILKDVSGVIKPRRMTLLLGPPGSGKTTLLLALSGKLDPSLQLSGSVTYNGHGLNE
FVPQRTAAYISQHDVHIGEMTVRETLAFSARCQGVGSRYDMLSELSRREKAANIKPDPDIDVYMKAIATEGQK
YSISTDYVLKILGLDICADTMVGDEMLRGISGGQRKRVTTGEMLVGPANALFMDEISTGLDSSTTFQIVSSLRQ
YVHIMNGTAVISLLQPAPETYDLFDDIILISDGQVVYHGPREYVLDFFESMGFKCPDRKGVADFLQEVTSRKDQ
AQYWVRRDQPYRFVTVTQFAEAFQSFHIGRKLTEELSVPFDKTKSHPAALTTKEYGLNKKELLKANFSREYLL
MKRNSFVYIFKLTQLFIMALIAMTLFFRTEMHRNNQDDAGVYAGALFFTLVTIMFNGMSEISMTIAKLPVFYKQ
RDLLFYPSWAYAIPSWILKIPVTMVEVALWVFLTYYVIGFDPNIGRFFKQFLILFFTSQMASGLFRAIAALGRNM
IVANTFGSFAVLTLLALGGFILSRKDIKSWWIWGYWISPLMYGQNALMANEFLGNNWHNATFDLGKLYLDNR
GFFPHAYWYWIGIGGLVGFVFLFNALFGVALAVLGPFDKPQATITEDSEEVVEEVELPRIESSGRGDSVTESSHG
KKKGMVLPFEPHSITFDDIVYSVDMPAEMKEQGVQEDRLVLLKGVSGAFRPGVLTALMGVSGAGKTTLMDV
LAGRKTGGYIDGSIKVSGYPKKQETFARISGYCEQNDIHSPHVTVYESLLYSAWLRLPSGVDSNTRKMFIDEVM

DLVELNSLRNSLVGLPGVSGLSTEQRKRLTIAVELVANPSIIFMDEPTSGLDARAAAIVMRTVRNTVDTGRTVV
CTIHQPSIDIFEAFDELFLMKRGGQEIYVGPLGRHSTHLIKYFESIEGVSKINNGYNPATWMLEVTTTAQEHNLG
VDFTDYYKNSDLYRRNKQLIQELGQPAPGSKDLHFPTQFSQSFLVQCQACLWKQRWSYWRNPPYTAVRFFFT
TFIALMFGTIFWDLGGKHSSRQDLLNAVGSMYTAVLFLGVQNSSSVQPVVAVERTVFYREKAAGMYSALPYA
FSQILVELPYVFAQAVTYGVIVYAMIGFEWTAEKFFWYLFFMYFTLLYFTFYGMMAVAVTPNHHVASIVAAA
FYAIWNLFSGFVVPRPSIPIWWRWYYWACPVAWTIYGLVASQFGDLTTVMTSEDKDVKTFLDDFFGIKHDFIG
ECAVVVGGIAVSFAFIFAVAIKAFNFQKR

>XP_004501294.1_P_PDR1-l_Ca
MESSELRVGSGRIGSSSIWRSGAVDVFSGSSRREDDEQELQWAAIEKLPTYLRLTRGILTESQDQQPIQIDINKLG
PLQRKNLVERLVKIAEEDNEKFLLKLRKRIDRVGLDIPTIEVRFEHLNVEAEAHVGSRALPTILNFSINLFEGLLN
YLHLIPSRKKPFTVLHDVSGIIKPKRMTLLLGPPSSGKTTLLLALAGRLNKDLKFSGRVVYNGHEMEEFVPQRT
SAYISQTDLHIGEMTVRETLAFSARCQGIGTRYDMLAELSRREKAENIKPDPDLDIYMKAAALEGQETNVVTD
YIIKILGLDACADTMVGDDMIRGISGGQKKRVTTGEMLVGPERALFMDEISTGLDSSTTFQMINSLRQSIHILNG
TALISLLQPAPETYELFDDIILLSDGQIVYQGPRENVLEFFEHIGFKCPERKGVADFLQEVTSRKDQEQYWANKD
ESYTFITVREFAEAFQSFHIARKLGDELATPFDTSKGHPAVLTKNKYGVSKKELLKACVSREFLLMKRNSFVYIF
KMWQLIFTGIVTMTLFLRTEMHRNTETDGGIYMGALFFILIVIMFNGYSELSMFIMKLPVFYKQRDLLFFPAWA
YSLPTWILKIPITFLEVGIWVVFTYYVIGFDPSFERFIKQYFLLVCINQMASALFRFIGAVGRNIIVANTAGSFALI
VVLVMGGFILSRVDVKKWWLWGYWVSPMMYGQNAIAVNEFLGNSWSHVPPDSTEPLGVQILKSRGIFPEAY
WYWIGVGASIAYMLLFNFLFPLALHYLDSYDKPQALISEEALAERNAATAGNRQIIELSPEYECSSAKGNRSRK
SFPSTTASARVGSISAADHSRKHGMVLPFTPLSITFDEIRYEVDMPQEMKAKGVLEDRLELLTSVNGAFRPGVL
TALMGISGAGKTTLMDVLSGRKTTGYIQGRITISGYPKKQETFARISGYCEQTDIHSPHVTVYESMVYSAWLRL
PPEVDSATRKMFIEEVMELIELTSIREALVGLPGVNGLSTEQRKRLTIAVELVANPSIIFMDEPTSGLDARAAAIV
MRTVRNTVDTGRTVVCTIHQPSIDIFDAFDELLLLKRGGEEIYVGPLGQHCSNLINYFEGINGVPKIKNSYNPAT
WMLEVTSEAQEEAIGVNFADLYKNSDLYRRNKALIKELSTPPEGSKDLFFTTQHSQSFLTQCMACLWKQHLSY
WRNPPYSAVRLLFTTVIAFLFGTIFWNIGSKRERRQDLFNAMGSMYAAVLFIGVQNATSVQPVVAIERTVFYRE
RAAGMYSALPYAFGQVAVEIPYILIQSLVYGVIVYTMIGFEMTPAKFFWYLFFMFFTLLYFTFFGMMLVGATPN
HNVAAIVSFGFYLLWNLFSGFVIPRTRMPIWWRWFFWICPISWTLYGLVTTQFGNVKERMETGETVEEFVMTY
FGYRDDFKGVAAAVVVSFSLIFGFTFAFSIKAFNFQKR

>XP_004506879.1_P_PDR1_X1_Ca
MEGSDIYKASNSFRTNSTSVWRNSTMDAFSKSSRHEEDDEEALKWAALEKLPTYNRLRKGLLATSRGVVHEID
ILTDLGFQERQKLLDRLINVAEEDNEKFLLKLKERIDRVGIEIPTIEVRYEHLIIEAEAYVGGRALPTFMNSITNTL
ESIFTSLHILKSKKKHMTILKDVSGIVKPQRMTLLLGPPSSGKTTLLLALSGKLDPNLKVSGRVTYNGHEMEEFV
PQRTAAYISQHDVHIGEMTVRETLAFSARCQGVGSRYDLLSELSRREKEAKIKPDQDIDVFMKAVSTGGQQES
VVTDYVLKLLGLDVCADTMVGDEMLRGISGGQRKRVTTGEMLVGPANALFMDEISTGLDSSTTFQIVRSLKQ
YVHILNGTAVISLLQPAPETYELFDDIVLLSDGEIVYQGPRELVLDFFESLGFKCPDRKGVADFLQEVTSKKDQE
QYWVHKEKPYRFVTVTQFAEAFQSFHVGRRIGDELAIPFDKSKSHPAALNTKKYGVNKKELLKANFSREYLL
MKRNSFVYIFKICQLILMATVTMTLFLRTEMHRDTLNDGGVYAGAIFFTVVMLMFNGLSELSMTIAKLPSFYK
QRDLLFFPPWAYAIPTWILKIPVTFVEVAVWVFLTYYVIGFDPNVARFLKQYILLLLINQMASGLFRAIAALGRN
MIVANTFGSFALLALLTLGGFILSKKDIKGWWIWGYWISPLMYGQNAIMVNEFLGDSWNHFTPNSNKTLGIQIL
ESRGFFTDAYWYWIGIGALTGFMLLFNVAFTVALTYLNPFDKPQATINEESEGSTPNGIAQEVELPQLASSGESY
NVVDSSQKKKRGMVLPFEPHSITFDEVVYSVDMPQEMKVQGVIEDRLVLLKGVNGAFRPGVLTALMGVSGA
GKTTLMDVLAGRKTGGYIDGSIKISGYLKRQETFARISGYCEQNDIHSPHVTVYESLLYSAWLRLSAEVDSNTR
KMFIEEVMELVELNPLRNSLVGLPGVNGLSTEQRKRLTIAVELVANPSIIFMDEPTSGLDARAAAIVMRTVRNT
VDTGRTVVCTIHQPSIDIFEAFDELFLMKRGGQEIYVGPLGRHSSQLIKYFESIEGVHKIKDGYNPATWMLEVTS
SAQELTLGVDFHDIYRNSELYRRNKQLIEELGKPAIGSKDLHFSTQYSQSFLVQCLACLWKQHWSYWRNPPYT
AVRFFFTTFIALMFGTMFWNLGRKYSNRQDLFNALGSMYTSVLFLGVQNSSSVQPVVAVERSVFYRERAAGM
YSALPYAFAQVIIELPYIFAQATSYGVIVYAMIGFEWTLAKFFWYIFFMYFTLCYFTFYGMMAVAVTPNHHVAS
IVASAFYAIWNLFSGFIVPRPMIPVWWRWYYWACPVSWTIYGLVASQFGDINKIMESENKPVQEFLRSYFDFK
HDFIGVCAVVVVGTAVLFACIFAVSIKVFNFQKR

>XP_006360347.1_P_PDR1_St
MEPANLGNLRGSSLRGSISGSRRGSVSLRANSNSIWRNTGVEIFSRSARDEDDEEALKWAALEKLPTFDRLRKG

LLFGSQGAAAEIDINDIGYQERKNLLERLVRVAEEDNEKFLLKLKNRIDRVGIDLPTIEVRYENLNIEADAYVGS
RGLPTVINFMTNFIETLLNTLHILPSSKRQITILKDISGIIKPCRMTLLLGPPSSGKTTLLLALAGKLDSSLKVTGKV
SYNGHELHEFVPQRTAAYISQHDLHIGEMTVRETLEFSARCQGVGSRYEMLAELSRREKAANIKPDPDIDIYMK
ASATEGQEANVVTDYVLKILGLDICADTMVGDEMLRGISGGQKKRVTTGEMLVGPSKALFMDEISTGLDSSTT
YSIVNSLRQSVQILKGTAVISLLQPAPETYNLFDDIILISDGYIVYQGPRDDVLEFFESMGFKCPERKGVADFLQE
VTSKKDQPQYWSRRNEHYRFISSKEFSDAFQSFHVGRKLGDELAIPFDRTKCHPAALTNEKYGIGKKELLKVCT
EREYLLMKRNSFVYVFKFVQLTIMALMTMTLFFRTEMPRDTVDDGGIYAGALFFVVVMIMFNGMSEMAMTIF
KLPVFYKQRDLLFFPSWAYAIPSWILKIPVTLVEVGLWVILTYYVIGFDPNITRFLKQFLLLVLVNQMASGLFRF
MGAVGRTMGVASTFGAFALLLQFALCGFVLSREDVKGWWIWGYWISPLMYSVNSILVNEFDGNKWKHIAPN
GTEPLGVAVVKSRGFFADAYWYWIGFAALFGFTIVFNFFYSLALAYLKPYGKSQTVRPEDSENAENGQAASQ
MASTDGGDIVSAGQSKKKGMVLPFEPHSITFDDVVYSVDMPQEMKEQGAGEDRLVLLKGVSGAFRPGVLTAL
MGVSGAGKTTLMDVLAGRKTGGYIDGDIKISGYPKKQETFARISGYCEQNDIHSPYVTVYESLVYSAWLRLPK
DVDEKIRKMFVDEVMELVELEPLRSALVGLPGVNGLSTEQRKRLTIAVELVANPSIIFMDEPTSGLDARAAAIV
MRTVRNTVDTGRTVVCTIHQPSIDIFEAFDELFLMKRGGQEIYVGPLGRHSCHLIKYFESIPGVAKIKEGYNPAT
WMLEVTASAQEMMLGVDFTDLYKNSDLYRRNKALITELSVPRPGSKDLYFETQYSQSLWIQCMACLWKQNW
SYWRNPAYTAVRFIFTMFIALVFGTMFWDIGTKVSQSQDLFNAMGSMYAAVLFLGVQNASSVQPVVDVERTV
FYRERAAGMYSAIPYAFGQVFIEIPYVFVQAIVYGIIVYAMIGFEWETGKVFWYLFIMYTTLLYFTFYGMMSVA
VTPNQNVASIVAAFFYAIWNLFSGFIVPRPRMPIWWRWYYWCCPVAWTLYGLVASQFGDIQTKLVDEETVEQ
FLRRYFGFRHDFLPVVAGVLVAYVVVFAFTFAFAIKAFNFQRR

>XP_006366077.1_P_PDR1-l_X1_St
MEGGENILRVSSARLSGSNVWRNSAMDVFSRSSSREDYDDEEALRWAALEKLPTYRRIRRGLLLEEEEGQSRE
VDITKLDLIERRNLLDRLVKIADEDNEKLLMKLKQRIDRVGLDLPTIEVRFEHLNIDAEARVGSRALPTIFNFTV
NILEDFLNYLHILPSRKKPLPILHGVGGIIKPGRMTLLLGPPSSGKTTLLLALAGKLDNDLKVSGRVTYNGHGM
DEFVPQRTSAYISQNDLHIGEMTVRETLAFSARCQGVGTKYEILAELSRREKEANIKPDPDIDIFMKSAWNEGQ
EANVITDYTLKILGLEICADTLVGDEMIRGISGGQRKRLTTGEMMVGPARALFMDEISTGLDSSTTYQIVNSIRQ
SIHILQGTAVISLLQPAPETYDLFDDIILLSDGQIVYQGPRENVLEFFEYLGFKCPQRKGVADFLQEVTSRKDQEQ
YWSRRDEPYRFITACEFSDVFQSFDVGRKLGDELAVPFDKSKSHPAALTTKRYGISKKELLKACTAREYLLMK
RNSFVYIFKMVQLTLMASIAMTLFLRTEMHRDTTIDGAIYLGALFYAVITIMFNGFSELALSIMKLPSFYKQRDF
LFFPAWAYALPTWILKIPITLVEIAIWVCMTYYVIGFEADVGRFFKQIFLLICLSQMASGLFRFLAALGRNIIVAN
TFGSCALLIVLVMGGFILSRDDVKQWLIWGYWISPMMYAQNAIAVNEFLGKSWAHVPPNSTGTDTLGVSFLKS
RGIFPEARWYWIGAGALFGYVLLFNFLFTVALAYLNPFSKPQAILSEEIVAERNASKRGEVIELSPIGKSSSERGN
DVPVSTSSRSLSTRVGNITEGDLNKRKGMILPFEPLSITFDDIRYAVDMPQEMKTQGFIEDRLELLKGVSGAFRP
GVLTALMGVSGAGKTTLMDVLAGRKTGGYVEGTISISGYPKQQETFARISGYCEQTDIHSPHVTVYESLLYSA
WLRLPREVDTETRKSFIEEVMELVELTPLREALVGLPGVNGLSTEQRKRLTVAVELVANPSIIFMDEPTSGLDA
RAAAIVMRTVRNTVDTGRTVVCTIHQPSIDIFDAFDELLLLKRGGEEIFVGPLGRHSSHLIKYFEGIDGVLKIRDG
YNPATWMLEVTSLAQEAVLGIDFTELYKNSELYRRNKALIQELSVPASGSKDLYFETKYSQSFFTQCMACLWK
QHWSYWRNPPYTAVRLMFTFFVSLMLGTIFWGLGSKRGKQQDILNAIGSMYAAILFLGIINASSVQPVVAIERT
VFYRERAAGMYSALPYAFGQVMIELPHLFLQTIIYGVIVYAMIGFEWTVTKFFWYLFFMYFTLLYFTLYGMMT
VAVTPNHTIASIVSSAFYTIWNLFCGFVVPKTRMPVWWRWYYYICPLSWTLYGLIASQFGDLQDRLDTKETVE
EFLENFFDYKHDFVGYVAVILVGISVVFLFIFAYSIKSFNFQKR

>XP_006366078.1_P_PDR1-l_St
MEGGGDILKVSSARLSSSNVWRNSAMDVFSRSSREDYDDEEALKWAALEKLPTYLRIRRGILSEEEGQYREVD
ITKLDLVERRNLLERLVKIADEDNEKFLLKLKKRIDRVGLDLPTIEVRFEHLNVDAEARVGSRALPTIFNFTVNIL
EDFLNYLHILPSRKKPLPILHDVSGIIKPGRMTLLLGPPSSGKTTLLLGLAGKLDKDLKVSGRVTYNGHGMDEF
VPQRTSAYISQNDLHIGEMTVRETLAFSARCQGVGAKYEILAELSRREKEANIKPDPDVDIFMKSAWNDGQEA
NVVTDYTLKILGLEICADTIVGDEMIRGISGGQRKRLTTGEMMVGPARALFMDEISTGLDSSTTYQIVNSIRQSI
HILQGTAVISLLQPAPETYDLFDDIILLSDGQIVYQGPRENVLEFFEYLGFKCPQRKGVADFLQEVTSRKDQEQY
WARRDEPYKFITVREFSEAFQSFHVGRKLGDELAVPFDKSKSHPAALTTKRYGVSKKELLKACTAREYLLMKR
NSFVYIFKMIQLTLMATITMTLFLRTEMHRNTMIDGAVFLGALYYAVIMIMFNGFSELALSIMKLPSFYKQRDL
LFFPAWTYALPTWILKIPITLVEVAIWVCMTYYVIGFEADVGRFFKQLFLLICLNQMASGLFRFLAALGRNIIVA
NTFGSCALLIVLVMGGFILSRDNVKQWLIWGYWISPMMYAQNAIAVNEFLGKSWAHVPPNSTGTDTLGVSFL
KSRGIFPEARWYWIGVGALLGYVLLFNFLFTVALAYLNPFGKPQAVLSEETVAERNASKKGEVIELSPIGKSSSE

RGNDVRRSASSRSMSSRVGNITEGDINKRKGMILPFEPLSITFDDIRYAVDMPQEMKSQGFIEDRLELLKGVSGA
FRPGVLTALMGVSGAGKTTLMDVLAGRKTGGYIEGTISISGYPKQQETFARIAGYCEQTDIHSPHVTVYESLQY
SAWLRLPREVDTETRKRFIEEVMELVELKPLREALVGLPGVNGLSTEQRKRLTVAVELVANPSIIFMDEPTSGL
DARAAAIVMRTVRNTVDTGRTVVCTIHQPSIDIFDAFDELLLLKRGGEEIFVGPLGRHSSHLIKYFEGIDGVPKIR
DGYNPATWMLDITSVAQEAALGVDFTELYRNSELYRRNKALIKELSVPAPGSKDLYFQTKYSQSFFTQSMACF
WKQHWSYWRNPPYTAVRLLFTFFIALMFGTIFWDLGSKRRRQQDILNAIGSMYASVLFLGVQNATSVQPVVAI
ERTVFYRERAAGMYSALPYAFGQIMIELPYIFIQTIIYGVIVYAMIGFEWTVAKFFWYLFFMYFTLLYFTLYGM
MTVAVTPNHSIAAIVSSAFYAVWNLFSGFIVPKTRMPVWWRWYYYICPISWTLYGLIASQFGDLQDKLETKET
VEEFIESFFDFKYDFVGYVAVILVGISVLFLFIFAYSIKAFNFQKR

>XP_006416897.1_ABCG40_Es
MEGTSFQKANNSLRRNSSVWRKDSGMEIFSRSSREEDDEEALKWAALEKLPTFDRLRKGILTASHGLNEIDIQK
LGFQDTKKLLERLIKVGDDEHEKLLWKLKNRIDRVGIDLPTIEVRFDHLKVEAEVHVGGRALPTFVNFISNFAD
KLLNTLHLLPNRKKKFTILNNVSGIIKPGRMTLLLGPPSSGKTTLLLALAGKLDRELKETGRVTYNGHGMNEFV
PQRTAAYIGQNDVHIGEMTVRETFAYAARFQGVGSRYDMLTELSRREKEANIKPDPDIDVFMKATSTAGEETN
VMTDYILKILGLEVCADTMVGDDMLRGISGGQKKRVTTGEMLVGPSRALFMDEISTGLDSSTTYQIVNSLRNY
VHIFNGTALISLLQPAPETFNLFDDIILIAEGEIIYEGPRDHVVEFFETMGFKCPPRKGVADFLQEVTSKKDQMQY
WSRSDEPYRFIRVREFSEAFQSFHVGRRLGDELALPFDKTKSHPAALTTKKYGVGVKELVKTSFSREYLLMKR
NSFVYYFKFGQLLVMAFMVMTLFFRTEMQKKTEADGSLYTGALFFILMMLMFNGMSELSMTIAKLPVFYKQR
DLLFYPAWVYSLPPWLLKIPISFIEAALTAFITYYVIGFDPNIGRLFKQYMLLVLMNQMASGLFKMMAALGRN
MIVANTFGAFSLLVFFALGGVVLSREDIKKWWIWGYWISPIMYGQNAIVANEFFGHSWSQAVPNSSDTLGVTF
LKSRGFLPHAYWYWIGTGSLLGFVVLFNLGFTVALTFLNSLGKPQAVLMEEPASDGKNETELQSARTGGAVE
ANKKRGMVLPFEPHSITFDNVIYSVDMPQEMIEQGTNEDRLVLLKGVNGAFRPGVLTALMGVSGAGKTTLMD
VLAGRKTGGYIDGNITISGYPKNQQTFARISGYCEQTDIHSPHVTVYESLVYSAWLRLPKEVDSNTRKMFIEEV
MELVELTPLRQALVGLPGESGLSTEQRKRLTIAVELVANPSIIFMDEPTSGLDARAAAIVMRTVRNTVDTGRTV
VCTIHQPSIDIFEAFDELFLLKRGGEEIYVGPLGHESSHLINYFESIQGISKITEGYNPATWMLEVSTTSQEAALGV
DFAQLYKNSELYKRNKELIKELSKPAPGSKDLYFPTQYSQSFWTQCMASLWKQHWSYWRNPPYTAVRFLFTI
GIALMFGTMFWDLGGKTRTRQDLSNAMGSMYTAVLFLGLQNAASVQPVVNVERTVFYREKAAGMYSAMPY
AFAQVFIEMPYVLVQAVVYGLIVYAMIGFEWTAAKFFWYLFFMYGSLLTFTFYGMMAVAMTPNHHIASVVSS
AFYGIWNLFSGFLIPRPSMPVWWEWYYWLCPVSWTLYGLITSQFGDITEPMTDGTSVKQFIRDFYGFREGFMG
VVAAMNVIFPLVFAIIFAVGIKTFNFQKR

>XP_006450535.2_PDR1_Cc
MQMDAGQASFRISSARLGSSSIWRNNTLDVFARSSREDTYDDDEALTWAAIEKLPTYLRVQRGMLTADEGQA
REVDIKNLGFIERRNLIERLLKIAEEDNEKFLLKLKVRIERVGLDIPTIEVRFEHLNVEAEAYIGSRALPTVFNSCA
NMLEGFLNYLHVLPSRKKPLTILHDVSGIIKPRRLALLLGPPSSGKTTLLLALAGKLGKDLKFSGRVTYNGHGM
EEFVPQRTSAYISQNDLHIGEMTVRETLAFSARCQGVGPRYEVLQELSRREKAANIKPDPDIDLIMKAASLEGQ
EKNVVTDYVLKILGLEVCADTMVGDEMLRGISGGQRKRLTTGEMLVGPARALFMDEISTGLDSSTTYQIVNSL
RQSIHILNGTAVISLLQPAPETYELFDDLILLSDGQIVYQGPRENVLEFFERMGFKCPERKGVADFLQEVTSRKD
QEQYWANKDEPYSFVTAKEFSEVFQSFHIGQKLGDELATPFDKSKSHPAALTTKKYGASKKELLKACFAREYL
LMKRNSFVYFFKMFQIFFSASVAMTLFLRTEMHRSTVEDGGIYMGALFFAVITIMFNGFSELSMTIMKLPVFYK
QRDFLFFPAWAYSLPTWILKIPITFIEVGIWVFMTYYVVGFESNIERFVKQYFLLLCVNQTASGLFRLMGALGRN
IIVANTFGSFANLTVLVLGGFILSRDDVKKWWLWGYWFSPMMYGQNALAVNEFLGKSWGHVPPNSTEPLGV
VILKSRGLFPNAYWYWIGVGALLGYVLLFNFLFTVALKYLDPFGKPQAILSEEALAKKNACKTEEPVELSSGV
QSSYDRGNESHRNVSFRTLSARVRSFNEADQNRKRGMILPFEPHSITFDDIRYALDMPQEMKAQGIPDDRLEFL
KGVSGAFRPGVLTALMGVSGAGKTTLMDVLAGRKTGGYVSGSITISGYPKNQETFARISGYCEQTDIHSPHVT
VYESLVYSAWLRLPPEVDSDTRKMFVEEVMELVELNPIREALVGLPGVSGLSTEQRKRLTIAVELVANPSIIFM
DEPTSGLDARAAAIVMRTVRNTVDTGRTVVCTIHQPSIDIFDAFDELLLMKRGGEEIYVGPLGRHCSQLIKYFE
GIDGVPKIKEGYNPATWMLEVTTPAQEAALGINFAKVYKNSELYKGNKEMIKELSIPPPGSKNLYFQTRYSQSF
FTQCMACLWKQHLSYWRNPPYTAVRLFFTTFIALMFGTIFWDIGSKRANRQDLFNAMGSMYAAILFLGVQNA
TSVQPVVAVERTVFYRERAAGMYSALPYAFGQVVIELPHIFIQAVIYGVIVYAMIGFDWTVSKFLWYLLFMYL
TFLYFTLYGMMTVAVTPNHNIAAIIASAFYVLWNLFSGFIIPRPRMPIWWRWYCWICPVSWTLYGLVASQFGD
VNDTFDSGQKVGDFVKDYFGYDHDMLGVVAVVHGLVVLFGFTFAYSIKAFNFQHR

>XP_006465411.1_PDR1-l_Csi
MESGNKVYKASNSLRIGSTSIWRSNSATLGAFSMSSRGEEDDEEALKWAALEKLPTYNRLKKGILTSSRGEAN
EVDVCNLGPQERQRIIDKLVKVADVDNEEFLLKLKNRIDRVGISLPTIEVRFEHLNVEAEAYVGSRALPTFFNFC
ANIIEGFLNSVNILPSRKKHLAILKDVSGIIRPGRMTLLLGPPASGKTTLLLALAGKLDSSLRVSGRVTYNGHDM
DEFVPQRTAAYISQHDNHIGEMTVRETLAFSARCQGVGSRHEMLSELSRREKAAGIKPDPDIDVFMKAAATEG
QEASVVTDYILKILGLDVCADTMVGDEMLRGISGGQKKRVTTGEMMVGPAQALFMDEISTGLDSSTTFQIVNS
LRQSIHILKGTTLISLLQPAPETYDLFDDIILISDGQIVYQGPREHVLEFFKFMGFECPKRKGVADFLQEVTSRKD
QEQYWVHKEEPYRFVTVKEFADAFQVFYMGQKVGDELRIPFDKRKSHRAALTTKIYGVSKKELLKACMSREL
LLMKRNSFVYIFKLCQLTIMGLVAMTLFFRTKMHRDSITDGVIYTGALFFIVLMIMFNGMAEIPMTIAKLPIFYK
QRDLRFYPSWAYALSTWILKIPISYIEVAVWVFLTYYVIGFDPNVGRLFRQYLLLLFLNQMASALFRLIAATGR
NIVVANTFGSFALLLLFVLGGFVLSREDIKKWWIWAYWCSPLMYAQNAIVVNEFLGNSWRKVLPNTTEPLGV
QVLKSRGFFTDAYWYWLGLGALAGFILLFNFGFTLALSFLNPFGKNQAVISEESQSNEHDNRTGGTIQLSTSEIG
HDIMGTYSSSLIEAEVKANHHKKRGMVLPFKPHSITFDEITYSVDMPQEMMRPGVLEDKLVLLNGVSGAFRPG
VLTALMGVSGAGKTTLMDVLAGRKTGGYISGSIMISGYPKKQETFARISGYCEQNDIHSPNVTVYESLLYSAW
LRLPLEVDSPTQKMFIEEVMELVELKPLRQALVGLPGVSGLSTEQRKRLTIAVELVANPSIIFMDEPTSGLDARA
AAIVMRTVRNTVDTGRTVVCTIHQPSIDIVEAFDELFLLKRGGQEIYVGSLGRHSSHLIKYFEGIRGVSKIKDGY
NPATWMLEVTAPSQETALGIDFADIYKSSELYRRNKALIKDISKPAPGSKDLHFATQYAQSFFTQCMACLWKQ
HWSYWRNPPYSAVRFLFTTIIALAFGTMFWDMGTKTKKQQDLFNAMGSMYTAVLFLGVQNAASVQPVVSIE
RTVFYRERAAGMYSALPYAFAQALIEIPYIFVQSVTYGVIVYAMIGFEWTAAKFLWYQFFMFFTLLYFTYYGM
MAVAMTPNHHISGIVAFAFYGLWNVFSGFIIPRTRIPIWWRWYYWACPVSWTLYGLVASQFGDIQDRLESGET
VEQFLRSFFGFKHDFLGVVAAVVFAFPVLFALIFAVGIKVFNFQKR

>XP_006476214.2_PDR1-l_X1_Csi
MQMDAGQASFRISSARLGSSSIWRNNTLDVFARSSREDTYDDDEALTWAAIEKLPTYLRVQRGMLTEDEGQA
REVDIKNLGFIERRNLIERLLKIAEEDNEKFLLKLKDRIERVGLDIPTIEVRFEHLNVEAEAYIGSRALPTVFNSCA
NMLEGFLNYLHVLPSRKKPLTILHDVSGIIKPQRLTLLLGPPSSGKTTLLLALAGKLGKDLKFSGRVTYNGHGM
EEFVPQRTSAYISQNDLHIGEMTVRETLAFSARCQGVGPRYEVLQELSRREKAANIKPDPDIDLIMKAASLEGQ
EKNVVTDYVLKILGLEVCADTMVGDEMLRGISGGQRKRLTTGEMLVGPARALFMDEISTGLDSSTTYQIVNSL
RQSIHILNGTAVISLLQPAPETYELFDDLILLSDGQIVYQGPRENVLEFFERMGFKCPERKGVADFLQEVTSRKD
QEQYWANKDEPYSFVTAKEFSEVFQSFHIGQKLGDELATPFDKSKSHPAALTTKKYGASKKELLKACFAREYL
LMKRNSFVYFFKMFQIFFSASVAMTLFLRTEMHRSTVEDGGIYMGALFFAVITIMFNGFSELSMTIMKLPVFYK
QRDFLFFPAWAYSLPTWILKIPITFIEVGIWVFMTYYVVGFESNIERFVKQYFLLLCVNQTASGLFRLMGALGRN
IIVANTFGSFANLTVLVLGGFILSRDDVKKWWLWGYWFSPMMYGQNALAVNEFLGKSWGHVPPNSTEPLGV
VILKSRGLFPNAYWYWIGVGALLGYVLLFNFLFTVALKYLDPFGKPQAILSEEALAKKNACKTEEPVELSSGV
QSSYDRGNESHRNVSFRTLSARVRSFNEADQNRKRGMILPFEPHSITFDDIRYALDMPQEMKAQGIPDDRLEFL
KGVSGAFRPGVLTALMGVSGAGKTTLMDVLAGRKTGGYVSGSITISGYPKNQETFARISGYCEQTDIHSPHVT
VYESLVYSAWLRLPPEVDSDTRKMFVEEVMELVELNPIREALVGLPGVSGLSTEQRKRLTIAVELVANPSIIFM
DEPTSGLDARAAAIVMRTVRNTVDTGRTVVCTIHQPSIDIFDAFDELLLMKRGGEEIYVGPLGRHCSQLIKYFE
GIDGVPKIKEGYNPATWMLEVTTPAQEAALGINFAKVYKNSELYKGNKEMIKELSIPPPGSKNLYFQTRYSQSF
FTQCMACLWKQHLSYWRNPPYTAVRLFFTTFIALMFGTIFWDIGSKRANRQDLFNAMGSMYAAILFLGVQNA
TSVQPVVAVERTVFYRERAAGMYSALPYAFGQVVIELPHIFIQAVIYGVIVYAMIGFDWTVSKFLWYLLFMYL
TFLYFTLYGMMTVAVTPNHNIAAIIASAFYVLWNLFSGFIIPRPRMPIWWRWYCWICPVSWTLYGLVASQFGD
VNDTFDSGQKVGDFVKDYFGYDHDMLGVVAVVHVGLVVLFGFTFAYSIKAFNFQHR

>XP_007013791.2_P_PDR1_Tc
MESGTDAFRVSSARIGSSSIWRTNTMEAFSKSSREEDDEEALKWAAIEKLPTYLRVRRGILTEEEGQSREVDIKD
LGFVERRNLLERLVKIAEEDNEKFLLKLKERIERVGLDMPTIEVRFEHLNVEAEAYVGSRALPTMFNFSANILEG
LLSYLRILPSRKKPLPILNDVSGIIKPRRMALLLGPPSSGKTTLLLALAGKLGKDLKFSGRVTYNGHGMEEFVPQ
RTSAYISQYDLHIGEMTVRETLAFSARCQGVGPRYEMLAELSRREKEANIKPDPDIDIYMKAAALEGQEASVVT
DYILKILGLEVCADTMVGNEMIRGISGGQKKRVTTGEMLVGPSRALFMDEISTGLDSSTTYQIVNSLRQSIHILN
GTALISLLQPAPETYDLFDDIILLSDGQIVYQGPRENVLEFFECMGFKCPERKGVADFLQEVTSRKDQEQYWAR
KDEPYSFVSVKEFAEAFQSFHIGQKLGDDLAIPFDKSKSHPAALTKEKYGVSKKELIKACVSREYLLMKRNLFV
YVFKMIQLIIMGFITMTLFLRTEMHRDTMTDGVVFMGALFFILIMIMFNGFAELAMTIMKLPIFYKQRDLLFYPS
WAYSLPAWILKIPISVLEVTIWVFMTYYVIGFDPDAGRFFKHYLILLCLSQMASGLFRLMGGLGRNIIVANTCGS

FALLAVLVMGGFILTRDDVKKWWIWGYWISPLMYGQNAIAVNEFLGKSWRQIPPNSKEPLGVSILKSRGIFPE
AHWYWIGVGALIGYCFLFNFLFTLTLKYLDPFGKPQAIISKETLAEKIASKTGENAELSLRGKGSSERGIESRRSA
SSRSLSAKVGSINEATQNRKRGMVLPFEPLSMSFDEIKYAVDMPQEMKAQGVSEDRLELLKGVSGAFRPGVLT
ALMGISGAGKTTLMDVLAGRKTGGYVEGTIKISGYPKKQETFARISGYCEQTDIHSPHVTVYESLLFSAWLRLP
PEVDSETRMMFIEEVMELVELTSLREALVGLPGVNGLSTEQRKRLTIAVELVANPSIIFMDEPTSGLDARAAAIV
MRTVRNTVDTGRTVVCTIHQPSIDIFDAFDELLLLKRGGEEIYVGPLGRHSCHLIKYFEEITGIPRIKDGYNPATW
MLEVTSAAQEETLGVNLTNIYKNSELYRRNKALVKELSSPAPGSKDLYFLTRYSQSLITQCMACLWKQYWSY
WRNPPYTAVRFLFTTVIALLFGTIFWDLGSKRTRQQDVLNAMGSMYAAVLFIGFQNSASVQPVVAVERTVFYR
ERAAGMYSALPYAFGQVVVELPYVLVQTVIYGVIVYAMIGFDWTAAKFFWYLFFMYFTFLYFTFYGMMTVA
VTPNHNIAAIVSSAFLALWNLFSGFVIPRTRIPVWWRWYYWVCPISWTLYGLIGSQYGDIKDRFDSGETVEHFV
RNYFGFRDEFVGVVAVVTVGICVLFGFIFAFSIKAFNFQKR

>XP_007135719.1_P_Pv
MEGSDIYRARNSLRASSSTAWRRSIMEGFSRSSQHEEGADEEALKWAALEKLPTYNRLKKGLLTTSRGVASEI
DITELGFRERQKLLDRLINVAEEDNEKFLLKLKERIDRVGIDIPTIEVRYEHLHVDAEAYVGSRALPTFVNFVTN
MVESVFTSLHILSGKKKHVTILKDVSGIIKPRRMALLLGPPSSGKTTLLLALSGKLDPNLKASGRVTYNGHGMD
EFVPQRTAAYISQNDVHIGEMTVRETLAFSARCQGVGTRYDLLSELARREKEAKIKPDPDIDVYMKAAATGGQ
EASLVTDYVLKILGLDICADTMMGDEMLRGISGGQRKRVTTGEMLVGPANALFMDEISTGLDSSTTFQIVKSL
RQYVHILDGTAVISLLQPAPETYELFDDIILISDGQIVYQGPREHVLQFFESVGFQCPERKGVADFLQEVTSRKD
QEQYWMHRDEPYRFVTVTEFAEAFQSFHVGRRIGEQLATPFDKSKNHPAALTTERYGVNKKELLKANISREYL
LMKRNSFVYFFKLFQLTTLAILTMTMFLRTEMHRDNMGDGGVYTGALFFAVVILMFNGLAEISMTIVKLPIFY
KQRDLLFYPSWAYAIPSWILKIPITFLEAAVWVFLTYYVIGFDPNVGRFLKQYLVLLLINQMSSGLFRAIAALGR
NMIVANTFGSFALLILFALGGFVLSKNDIKKWWIWGYWISPLMYGQNAIVVNEFLGNSWNHFTPNSNKTLGIQI
LESRGFFTHAYWYWIGIGALIGFIFLFNIMYTLALSYLNPFDKPQATITESEGGMANGRAREEELTCLESCGSAN
SVVSCSRRKNRGMVLPFEPYSITFDQIVYSVDMPQEMKEQGVREEKLVLLKGVSGAFCPGVLTALMGVSGAG
KTTLMDVLAGRKTGGYIEGNIKVSGYPKRQETFARISGYCEQNDIHSPHVTVYESLVYSAWLRLTAEVESKTR
KMFVEEVMELVELNPLRNSLVGLPGVSGLSTEQRKRLTIAVELVANPSIIFMDEPTSGLDARAAAIVMRTVRNT
VDTGRTVVCTIHQPSIDIFEAFDELFLMKRGGQEIYVGPLGRQSSQLIKYFESIEGVSKIKDGYNPATWMLEVTT
PAQELTLGVDFHEIYRNSELYRRNKQLIAELGNPGHGSKDIEFPTQYAQSLLVQCIACLWKQHWSYWRNPPYT
AVRFLSTTVTALMFGTMFWDLGGKYSRRQDLFNALGSMYNAVLFVGVQNSSSVQPVVAIGRTVFYRERAAG
MYSAFPYALAQVLIELPYIFVQASTYSVIVYSMMGFEWTLEKFFWYLFFMYFTLCYFTFYGMMTVAVTPNHH
VASVVASAFYGIWNLFSGFVIARPSIPVWWRWYYWACPVAWTIYGLVASQFGDITNVMKLENVSVQEFLRSY
FGFRHDFIGVSAIMVSGFAGLFAIIFAVSIKAFNFQKR

>XP_007137056.1_P_Pv
MESDELRAASARIGSSSVWRSSGVDVFSGSSRRDDDEEELKWAAIEKLPTYLRMTRGILTEAEGQPPTEIDINKL
CPLQRKNLVERLVKIAEQDNEKFLFKLRDRIDRVGLEIPTIEVRFEHLNVEAEAHVGSRALPTIFNFCINLLEGFL
SSLHLIPSRKKPFTVLDGVSGIIKPRRMTLLLGPPSSGKTTLLLALAGRLGKDLKFSGRVSYNGRGMEEFVPQRT
SAYISQSDLHIGEMTVRETLAFSARCQGIGTRYEMLAELSRREKAENIKPDPDLDIYMKAAALEGQETNVVTDY
IMKILGLEVCADTMVGDDMIRGISGGQKKRVTTGEMLVGPTRALFMDEISTGLDSSTTFQMVNSLRQSIHILNG
TAVISLLQPAPETYELFDDIILLSDGQIVYQGPRENVLEFFEYMGFKCPERKGVADFLQEVTSRKDQEQYWANK
DEPYIFITVREFAEAFQSFHIGRKLGDELATPFDMSKGHPAVLTKNKYGVSKKELLKACVSREFLLMKRNSFVY
IFKMWQLILTGFITMTLFLRTEMHRDTETDGGIFMGALFFVLIVIMFNGFSELSMSIMKLPVFYKQRDLLFFPSW
AYSLPTWILKIPITLVEVGIWVVMTYYVIGFDPSIERFIKQYFLLVCINQMASGLFRFMGAVGRNIIVANTVGSFA
LLAVMVMGGFILSRVDVKKWWLWGYWFSPMMYAQNALAVNEFLGKSWLHVPPNSTEPLGVKVLKSRGIFP
EAYWYWIGVGASIGYMALFNFLFPLALHYLDPFGKPQALISEEALAERNAGRNEHIVELSSRHNGSSDKGNED
RRSVCSRTLSARVGSIGASEHNTKRGMVLPFTPFSITFDDIRYAVEMPQEMKNQGILEDRLELLKGVNGAFRPG
VLTALMGVSGAGKTTLMDVLSGRKSAGYIQGQITISGYPKKQETFARIAGYCEQTDIHSPHVTVYESLVYSAW
LRLPPEVDSATKQMFIEEVMELVELTSLREALVGLPGVNGLSTEQRKRLTIAVELVANPSIIFMDEPTSGLDARA
AAIVMRTVRNTVDTGRTVVCTIHQPSIDIFDAFDELLLLKRGGEEIYVGPLGQHCSELISYFEGINGVPKIKNGY
NPATWMLEVTSEAQEAVLEVNFTEIYKNSDLYGRNKALIRELSTPPAGSKDLYYPTKYSQTFFTQCMACLWKQ
HLSYWRNPPYSAVRLLFTTIIALLFGTIFWDIGSKRQRRQDLFNAMGSMYAAVLFIGIHNATSVQPVVAIERTVF
YRERAAGMYSALPYAFGQVAIEIPYILIQTLVYGVIVYAMIGFDWTLSKFFWYLFFMFFTFLYFTFYGMMAVG
LTPDHNIAAIVSFGFYMIWNLFSGFVIPRTRMPVWWRWYFWICPVSWTLYGLVTSQFGDIKDRIESGETVEEFV

RSYFGYRDDFVGVAAAVLVGFTLLFGFTFAFSIKAFNFQKR

>XP_007150693.1_P_Pv
MDGDIYRASNSMRARSSTVWRNSGVEAFSRSSREEDDEEALKWAALEKLPTYNRLRKGLLTASHGVANEIDV
SDLGFQQKQKLLERLVKVAEEDNERFLLKLRERIDRVGLDIPTIEVRYEHLNIDAEAFEGSRALPSFINSVTNVIE
GLFNILHIIPSKKKHVTILKDVSGIIKPRRMTLLLGPPSSGKTTLLLALSGKLDKSLQVSGKVTYNGHELNEFVPQ
RTAAYISQHDVHIGEMTVRETLAFSARCQGVGSRYDMLSELSRREKAANIKPDPDLDVYMKATATAGQESSIV
TDYTMKILGLDICADTMVGDEMLRGISGGQRKRVTTGEMLVGPANALFMDEISTGLDSSTTFQIVTCLRQYVH
ILDGTAVISLLQPAPETYDLFDDIILISDGQVVYHGPREYVLDFFESMGFKCPERKGTADFLQEVTSKKDQAQY
WVRRDQPYRFVTVTQFAEAFQSFHIGRKIVEDIAVPFDRSKNHPAALTTKQYGINKKELLKANFSREYLLMKR
NSFVYIFKLCQLSVMALIALTLFFRTEMPRDNMDDAGVYGGAIFFTIIMIMFNGMAEISMTIAKLPVFYKQRDLL
FFPSWAYAIPSWILKIPITIVEVALWVFLTYYVIGFDPNVGRFFKQYLILFFVSQMASGLFRAIAALGRNMIVANT
FGSFAVLTLLSLGGFLLSKRDIKSWWIWGFWISPLMYGQNALMINEFLGNNWHNATYNLGLDYLDSRAFFKD
SYWYWIGLGGLIGFVFVLNGMFSFALEFLDPFDKPQASIAEETPEANTPNEGTVAEVELPRRESSGRGGSVVES
GHGKKKGMVLPFEPHSITFDEVVYSVDMPQEMKEQGLQEDRLVLLKGVSGAFRPGVLTALMGVSGAGKTTL
MDVLAGRKTGGYIDGNIKISGYPKKQETFARISGYCEQNDIHSPHVTVYESLVYSAWLRLPSSVDSKARKMFIE
EVMELVELNPLRNSLVGLPGVSGLSTEQRKRLTIAVELVANPSIIFMDEPTSGLDARAAAIVMRTVRNTVDTGR
TVVCTIHQPSIDIFEAFDELFLMKRGGQEIYVGPLGRHSSHLIKYFESIDGVSKIKDGYNPATWMLEVTTTAQEL
SLGVDFTDLYRNSDLYRRNKQLIQELSQPAPGSKDLYFPTQYSQNILVQCQACLWKQRLSYWRNPPYTSVRFF
FTTFIALMFGTMFWDLGTKRSTRGDLMNAIGSMYSAVLFLGVQNSSSVQPVVAVERTVFYREKAARMYSALP
YAFSQILVEIPYIFTQAVTYGVIVYAMIGFEWTAEKFFWYLFFMYFTLLYFTFYGMMAVGVTPNHHVASIVAA
AFYAVWNLFSGFVVARPNIPIWWRWYYWACPVAWTLYGLIGSQFGDVMERMTSESNKTVKAYIEDSYGIEH
DFIGVAAIVVPCIAVLFAFIFAVAIKTFNFQKR

>XP_007150694.1_P_Pv
MEGSDIYRASNSMRARSSTVWRNNSVEVFSRSSREEDDEEALKWAALEKLPTYNRLRKGLLTASHGAANEID
VADLGYQERHKLLERLVKVAEEDNESFLLKLKDRIDRVGLDIPTIEVRYEHLKIEAEAFVGGRALPSFINSATNV
IEGFLNFLHILPSRKKHVTILKDVSGVIKPRRMTLLLGPPSSGKTTLLLALSGKLDKSLQVSGKVTYNGHELNEF
VPQRTAAYISQHDVHIGEMTVRETLAFSARCQGVGSRYDMLSELSRREKAANIKPDPDLDVYMKATATAGQE
SSIVTDYTMKILGLDICADTMVGDEMLRGISGGQRKRVTTGEMLVGPANALFMDEISTGLDSSTTFQIVSCLRQ
YVHILNGTAVISLLQPAPETYDLFDDIILISDGQVVYHGPREYVLDFFESMGFRCPERKGAADFLQEVTSKKDQ
AQYWVRRDQPYRFVTVTQFAEAFQSFHIGRKLGQELAVSFDKTKSHPAALTTKKFGINKKELLKANFSREYLL
MKRNSFVYIFKLCQLFIMALIALTLFFRTEMDHDSLDGAGVYAGAIFFTIITVMFNGMAEISMTIAKLPVFYKQR
DLLFFPSWAYAIPSWILKIPVTIAEVALWVFLTYYVIGYDPNVGRLFKQYLILLLISQMASSLFRAIAALGRNMIV
ANTFGSFSVLTLLTLGGFILSKRDIKKWWIWGFWISPLMYGQNALMTNEFLGNSWHNDTHNLGLDYLDSRAFF
KDAYWYWLGLGALVGFVLFFNVVFGFALDFLGPFDKPQAVIAEEPTHEGSPAEVELPRIETGKDGSVVESSHG
KKKGMVLPFEPHSITFDEVVYSVDMPQEMKEQGVQEDKLVLLKGVSGAFRPGVLTALMGVSGAGKTTLMDV
LAGRKTGGYIEGNIKISGYPKKQETFARISGYCEQNDIHSPHVTVYESLLYSAWLRLPSSVDSKTRKMFIEEVME
LVELNPLRNSLVGLPGVSGLSTEQRKRLTIAVELVANPSIIFMDEPTSGLDARAAAIVMRTVRNTVDTGRTVVC
TIHQPSIDIFEAFDELFLMKRGGQEIYVGPLGRHSSHLIKYFESIDGVSKIKDGYNPATWMLEVTTTAQELGLGV
DFTDLYRNSDLYRRNKQLIQELGQPAPGSKDLYFPTQFSQNFFVQCQACLWKQRWSYWRNPPYTAVRFFFTTF
IAIMFGTMFWDLGGKRSTRGDLMNALGSMYTAVLFLGVQNSSSVQPVVAVERTVFYREKAAGMYSALPYAF
SQILVELPYIFTQAVTYGVIVYAMIGFEWTAEKFFWYLFFMYFTLLYFTFYGMMGVAVTPNHHVASIVSAAFY
AIWNLFSGFVVARPSIPIWWRWYYWACPVAWTLYGLVGSQFGDVMELMEAEGNKTVKAFIEDSYGIKHDFIG
VAAVVVAGIAVLFGFTFAVAIKTFNFQKR

>XP_008392127.1_P_PDR1-l_X1_Md
MDNGSGDIIRVSSARLSSSNIWRNSTMDVFSKSSHDEDDEEALKWAAIEKLPTYLRIRRGILTEEEGKGREIDVK
NLGLLERKNVLERLVKTAEEDNEKFLLKLKDRINRVGLDMPTIEVRYEHLNVEAEAYVGGRALPTIFNFVANIL
EGCLNFVHILPSRKHPLPMLTDVSGIIKPRRMTLLLGPPGSGKTTLLLALAGKLAKDLKFSGRVAYNGHGMEEF
VPERTSAYISQHDLHIGEMTVRETLAFSARCQGVGPRYEMLAELSRREKEANIMPDPDXDIYMKAASLEGQET
SVVTDYILKVLGLEVCADIMVGDEMVRGISGGQKKRVTTGEMLVGPARALFMDEISTGLDSSTTFQIVKSLRES
IHILSGTAVISLLQPAPETYDLFDDIIVLSDGQIVYQGPRENVLEFFEHMGFKCPERKGVADFLQEVTSKKDQEQ
YWADKEEPYNFISSKEFXEAFQSFHIGRKLGDELATPFDKSKGHPAALTTQKYGVSKKELLKACIDREFLLMKR

NSFVYIFKMTQLTLLAFISMTLFLRTEMHKNTVDDGGIFMGAMFFTVIIVMFNGFSELAMTIMKLPVFFKQRDL
LFYPAWAYSLPTWILKIPISFVEVAVWVVMTYYVIGFDPNIERFFRQYLLLLLLNQMASGLFRFMGALGRNIIV
ANTFGSFALLAVLVLGGFVLSRDDVKKWWVWGYWASPMMYGQNAIAVNEFLGDSWSHVPPNSTESLGIMV
LKSRGVFIEPYWYWIGVGATVGYIFLFNFFYTLALKFLNRKYLCTYVFWYFHMNIITNDPNNYFAAFEKPQAV
LSKEALAEKLTDQTGDSVELSSRGKNSSDPRNESRRSVSLRTLSARVGSITEANQNKKRGMVLPFEPLWITFDEI
KYAVDMPQEMKTQGVTEDRLPLLKGVTGAFRPGVLTALMGISGAGKTTLMDVLAGRKTGGFIEGNITISGYP
KKQETFARISGYCEQTDIHSPHVTVYESLVYSASLRLPPEVDSXTRKMFIEEVMELVELTSIREALVGLPGVNGL
STEQRKRLTIAVELVANPSIIFMDEPTSGLDARAAAIVMRTVRNTVDTGRTVVCTIHQPSIDIFDAFDELFLLKRG
GEEIYVGPXGRHSAHLIKYFEDIDGVPKIKDGYNPATWMLEITAAAQEAVLGVNFTEIYRNSELYRRNKAMIKE
LSTPPAGSKDLYFPTQYSQSFFSQCMACLWKQHLSYWRNPPYSAVRLLFTTFIALMFGTIFWDLGSKRRNQQD
LFNAMGSIYAAVLFIGXQNSSSVQPVVAIERTVFYRERAAGMYSALPYAFGQVVIELPYIFVQTIIYGVIVYTMI
GFEWTVSKFLWYIFFMYFTFLYFTFYGMMTVAVTPNHNIGAIVASAFYAIWNLFSGFIVPRTRMPIWWRWYY
WICPVSYTLYGLIASQFGDIKDKFDSGESVEHFVRNYFGYRRDLLGVVAAVHVGICVLFGFTFAFSIKAFNFQK
R

>XP_008392128.1_P_PDR1-l_X2_Md
MDNGSGDIIRVSSARLSSSNIWRNSTMDVFSKSSHDEDDEEALKWAAIEKLPTYLRIRRGILTEEEGKGREIDVK
NLGLLERKNVLERLVKTAEEDNEKFLLKLKDRINRVGLDMPTIEVRYEHLNVEAEAYVGGRALPTIFNFVANIL
EGCLNFVHILPSRKHPLPMLTDVSGIIKPRRMTLLLGPPGSGKTTLLLALAGKLAKDLKFSGRVAYNGHGMEEF
VPERTSAYISQHDLHIGEMTVRETLAFSARCQGVGPRYEMLAELSRREKEANIMPDPDXDIYMKAASLEGQET
SVVTDYILKVLGLEVCADIMVGDEMVRGISGGQKKRVTTGEMLVGPARALFMDEISTGLDSSTTFQIVKSLRES
IHILSGTAVISLLQPAPETYDLFDDIIVLSDGQIVYQGPRENVLEFFEHMGFKCPERKGVADFLQEVTSKKDQEQ
YWADKEEPYNFISSKEFXEAFQSFHIGRKLGDELATPFDKSKGHPAALTTQKYGVSKKELLKACIDREFLLMKR
NSFVYIFKMTQLTLLAFISMTLFLRTEMHKNTVDDGGIFMGAMFFTVIIVMFNGFSELAMTIMKLPVFFKQRDL
LFYPAWAYSLPTWILKIPISFVEVAVWVVMTYYVIGFDPNIERFFRQYLLLLLLNQMASGLFRFMGALGRNIIV
ANTFGSFALLAVLVLGGFVLSRDDVKKWWVWGYWASPMMYGQNAIAVNEFLGDSWSHVPPNSTESLGIMV
LKSRGVFIEPYWYWIGVGATVGYIFLFNFFYTLALKFLNPFEKPQAVLSKEALAEKLTDQTGDSVELSSRGKNS
SDPRNESRRSVSLRTLSARVGSITEANQNKKRGMVLPFEPLWITFDEIKYAVDMPQEMKTQGVTEDRLPLLKG
VTGAFRPGVLTALMGISGAGKTTLMDVLAGRKTGGFIEGNITISGYPKKQETFARISGYCEQTDIHSPHVTVYES
LVYSASLRLPPEVDSXTRKMFIEEVMELVELTSIREALVGLPGVNGLSTEQRKRLTIAVELVANPSIIFMDEPTSG
LDARAAAIVMRTVRNTVDTGRTVVCTIHQPSIDIFDAFDELFLLKRGGEEIYVGPXGRHSAHLIKYFEDIDGVPK
IKDGYNPATWMLEITAAAQEAVLGVNFTEIYRNSELYRRNKAMIKELSTPPAGSKDLYFPTQYSQSFFSQCMA
CLWKQHLSYWRNPPYSAVRLLFTTFIALMFGTIFWDLGSKRRNQQDLFNAMGSIYAAVLFIGXQNSSSVQPVV
AIERTVFYRERAAGMYSALPYAFGQVVIELPYIFVQTIIYGVIVYTMIGFEWTVSKFLWYIFFMYFTFLYFTFYG
MMTVAVTPNHNIGAIVASAFYAIWNLFSGFIVPRTRMPIWWRWYYWICPVSYTLYGLIASQFGDIKDKFDSGE
SVEHFVRNYFGYRRDLLGVVAAVHVGICVLFGFTFAFSIKAFNFQKR

>XP_008394025.1_P_PDR1-l_Md
MDNGNGDIFRVSSARLSSSNIWRNSTMDVFSKSSHDADDEEALKWAAIEKLPTYLRIQRGILTEEEGKGREIDIK
NLGLLERKNVLERLVKTADEDNEKFLLKLKDRINRVGLDIPTIELRFEHLNVEAEAYVGGRALPTIFNFLANILE
GCLNFVHVLPSRKHPLPILADVSGIIKPRRMTLLLGPPSSGKTTLLLALAGKLAKDLKFSGRVEYNGHGMEEFV
PERTSAYISQHDLHIGEMTVRETLAFSARCQGVGPRYEMLAELSRREKEANIIPDPDLDIYMKAASLQGQETNV
VTDYILKILGLEVCADIMVGDEMVRGISGGQKKRLTTGEMLVGPARALFMDEISTGLDSSTTFQIVKSLRESIHI
LSGTAVISLLQPAPETYDLFDDIILLSDGQIVYQGPRENVLEFFEHMGFKCPERKGVADFLQEVTSKKDQEQYW
ADKEEPYSFISSKEFAEAFQSFHIGRKLGDELATPFEKSMGHPAALTTQKYGVSKKELLKACIDREFLLMKRNSF
VYIFKMTQLTLMAFISMTLFCRTDLHKNTVDDGGIFMGAMFFAVVIIMFNGFSELAMTIMKLPVFFKQRDLLFY
PAWAYSLPTWILKIPISFVEVAVWVIMTYYVIGFDPNFERFLKQYLLLLLLNQMASGLFRFMGALGRNIIVANT
FGSFALVAVLVLGGFVLSRDNVMKWWVWGYWASPMMYGQNAIAVNEFLGNSWSHVPPNSTESLGIMVLKS
RGVFLEPYWYWLGVGATIGYIFLFNFFYTLALKFLNPFGKPQAVLSKEALAEKLADKAGDSVELSSRGKNSSD
SRNESRRSVSSRTLSARVGSIAEVNGNKKRGMVLPFEPLWITFDEIKYAVDMPQEMKTQGVTEDRLQLLKGVT
GAFRPGVLTALMGISGAGKTTLMDVLAGRKMGGYIEGNITISGYPKKQETFARISGYCEQTDIHSPHVTVYESL
VYSASLRLPPEVDSTTRKMFIEEVMELVELTSIREALVGLPGVNGLSTEQRKRLTIAVELVANPSIIFMDEPTSGL
DARAAAIVMRTVRNTVDTGRTVVCTIHQPSIDIFDAFDELFLLKRGGEEIFVGPLGRHSSHLIKYFEDIDGVPKIK
DGYNPATWMLEITAAAQEASIGVNFTEIYRNSELYGRNKAMIKELSTPPAGSKDLYFPTQYSQSFFSQCMACL

WKQHLSYWRNPPYSAVRLMFTTFIALMFGTIFWDLGSKRTSKQDLFNAMGSMYAAVLFIGVQNAASVQPVV
AIERTVFYRERAAGMYSALPYAFGQVVIELPYIFVQTIIYGVIVYAMIGFDWTVSKFLWYLFFMYFTFLYFTFYG
MMTVAVTPNHNIAAIVSSAFYAIWNLFSGFIVPRTRMPIWWRWYYWICPVSYTLYGLVASQFGDIKDKFDTGE
SVEHFVRDYFGYRRDFLGAVAAVHVGICVLFGFTFAFSIKVFNFQKR

>XP_008459146.1_P_PDR1-l_Cm
MDSGEIYRVSSARINSSSIWRNSAMEVFSRSSRDEDDEEALKWASIERLPTYLRVRRGILNLDGESAREIDVQNL
GLLERRNILERLVKIAEDDNERFLLKLKNRMERVGLDLPAIEVRFEHLEVEAEAHTAGRALPTMFNFSLNMFEG
FLSYFHIIPNRKKQLSILHDVSGIIKPGRMTLLLGPPSSGKTTLLKTLAGKLGKDLKFSGRVTYNGHGMNEFVPQ
RTSAYISQQDLHIGEMTVRETLSFSARCQGVGPRYDMLTELSRREKAANIKPDPDLDIIMKAAALGGQETNVVT
DYVLKILGLEICADTMVGDEMFRGISGGQKKRVTTGEMLVGPARALFMDEISTGLDSSTTYQIVNSMRQSIHIL
NGTALISLLQPAPETYELFDDIILISDGQVVYQGPRENVLEFFQHMGFTCPQRKGVADFLQEVTSRKDQEQYWT
KRDEVYRFVSVEEFSEAFQSFHVGKKLGDELATPFDKSKSHPAALTTEKYGASKKELLKACISRELLLMKRNSF
VYIFKLTQLILMAFVTMTLFFRTEMRRRTVDDGSVYMGALFFAIIIIMFNGFSELALTILKLPVFYKQRDFLFFPP
WAYSIPTWILKIPITFVEVGIWVVMTYYVVGFDPNAGRFFKHFLLLLCVNQMASALFRLIGALGRNIIVANTFGS
FALLTVLVLGGFVLARDDVHPWWIWGYWTSPMMYAQNGIAVNEFLGHKWRHPGPNSTESLGVMILKSRGIFP
QASWYWIGVGATIGYILLFNFLFTIALKYLDPFEKPQAVVSKETSTDKSVKKSQDVQELELSSKGKSTSERTEN
QISLSSRTSSARVGSFSEEAHQNKKRGMVLPFEPHSIAFDEIRYAVDMPQEMKSQGITENRLELLKGVSGSFRPG
VLTALMGVSGAGKTTLMDVLAGRKTGGYIEGNITISGYPKKQETFARIAGYCEQTDIHSPHVTVYESLVYSAW
LRLPPEVDSATRKMFVEEVMELIELNPLRDAIVGLPGVSGLSTEQRKRLTIAVELVANPSIIFMDEPTSGLDARA
AAIVMRTVRNTVDTGRTVVCTIHQPSIDIFDAFDELFLLRRGGEEIYVGPIGRHSSHLIEYFESIEGVPKIKDGYNP
ATWMLEITTAAQETTLGVNFNTLYKASELYRRNKALIKELSVPNENSNELYFPTKYSQSFFIQCIACLWKQHLS
YWRNPPYSAVRFLFTTFIALMFGTIFWDLGSKRGTQQDLFNAMGSMYAAVLFIGVQNATSVQPVVAIERTVFY
RERAAGMYSALPYAFGQVVIELPYIFIQTVVYGVIVYGMIGFEWTAAKFFWYIFFMYFTLLYFTFYGMMTVAV
TPNHNIAAIVSSAFYGFWNLFSGFIVPRTRIPIWWRWYYWICPVAWTLYGLVTSQFGDINDPMDSNQTVAEFVS
NYFGYEYDFLGVIAAVHVGITVLFGFIFAFSIKVFNFQKR

>XP_009148948.1_P_ABCG40_Br
MEGTSFQKASNSLRRDSSAWKRDSGMEIFSRSSREEDDEEALKWAALEKLPTFDRLRKGILTASHGINQIDIEKL
GFQDTKKLLERLIKVGDDEHEKLLWKLKNRIDRVGIDLPTIEVRFDHLKVEAEVHVGGRALPTFVNFMSNFAD
KLLNSLHLLPNRKKKFTILNDVSGIVKPGRMALLLGPPSSGKTTLLLALAGKLDHELKETGRVTYNGHGMNEF
VPQRAAAYIGQNDVHIGEMTVRETFAYAARFQGVGSRYDMLTELARREKEANIKPDPDIDVFMKATSTAGEE
TNVMTDYILKILGLEVCADTMVGDDMLRGISGGQKKRVTTGEMLVGPSRALFMDEISTGLDSSTTYQIVNSLR
NYVHIFNGTALISLLQPAPETFDLFDDIFLIAEGEIIYEGPREHVVEFFETMGFKCPPRKGVADFLQEVTSKKDQM
QYWARPDEPYRFVRVREFAEAFQSFHVGRRMGDELAVPFDKKKSHPAALTTKKYGVGIKELVNTSFSREYLL
MKRNSFVYYFKFGQLLVMAFATMTLFFRTEMQKKTVVDGSLYTGALFFILMMLMFNGMSELSMTIAKLPVFY
KQRDLLFYPAWVYSLPPWLLKIPISFIEAALTAFITYYVIGFDPNIGRLFKQYILLVLMNQMASALFKMVAALGR
NMIVANTFGAFAMLVFFALGGVVLSKDDIKKWWIWGYWISPIMYGQNAIVANEFFGHSWSRAVPNSSDTLGV
TVLKSRGFLPHAYWYWIGTGALLGFVVLFNFGFTLALTYLNSLGKPQAVLTEDPASNETELLVVEANANKKK
GMVLPFEPHSITFDNVIYSVDMPQEMIEQGTQEDKLVLLKGVNGAFRPGVLTALMGVSGAGKTTLMDVLAGR
KTGGYIDGNITISGYPKNQQTFARISGYCEQTDIHSPHVTVYESLVYSAWLRLPKEVDSNTRKMFIDEVMDLVE
LTPLRQALVGLPGESGLSTEQRKRLTIAVELVANPSIIFMDEPTSGLDARAAAIVMRTVRNTVDTGRTVVCTIHQ
PSIDIFEAFDELFLLKRGGEEIYVGPLGHESSHLINYFESIQGISKITEGYNPATWMLEVSTTSQEAALGVDFAQL
YKNSELYKRNKDLIKELSQPAPGSKDLYFPTQYSQSFWTQCMASLWKQHWSYWRNPPYTAVRFLFTIGIALMF
GTMFWDLGGKTRTQQDLSNAMGSMYTAVLFLGLQNAASVQPVVNVERTVFYREQAAGMYSAMPYAFAQVF
IEMPYVLVQAVVYGLIVYAMIGFEWTAAKFFWYLFFMYGSFLTFTFYGMMAVAMTPNHHIASVVSSAFYGIW
NLFSGFLIPRPSMPVWWEWYYWLCPVSWTLYGLITSQFGDITTPMADGTSVKQFIKDFYGFREGFLGVVAAM
NVIFPLAFAIIFAIGIKSFNFQKR

>XP_009334795.1_P_PDR1-l_Pb
MDNGNGDIFRVSSARLSSSNIWRNSTMDVFSKSSHDADDEEALKWAAIEKLPTYLRIQRGILTEEEGKGREIDIK
NLGLLERKNVLERLVKTADEDNEKFLLKLKDRINRVGLDIPTIEVRFEHLNVEAEAYVGGRALPTIFNFLANILE
GSLNFVHVLPSRKHPLPILADVSGIIKPRRMTLLLGPPSSGKTTLLLALAGKLAKDLKFSGRVEYNGHGMEEFV
PERTSAYISQHDLHIGEMTVRETLAFSARCQGVGPRYEMLAELSRREKEANIIPDPDLDIYMKAASLQGQETNV

VTDYILKILGLEVCSDIMVGDEMVRGISGGQKKRLTTGEMLVGPARALFMDEISTGLDSSTTFQIVKSLRESIHIL
SGTAVISLLQPAPETYDLFDDIILLSDGQIVYQGPRENVLDFFEHMGFKCPERKGVADFLQEVTSKKDQEQYWA
DKEEPYGFISSKEFAEAFQSFHTGRKLGDELATPFEKSMGHPAALTTQKYGVSKKELLKACIDREFLLMKRNSF
VYIFKMTQLTLMAFISMTLFCRTNLHKNTVDDGGIFMGAMFFAVVMIMFNGFSELAMTIMKLPVFFKQRDLLF
YPAWAYSLPTWILKIPISFVEVAVWVIMTYYVIGFDPNFERFLKQYLLLLLLNQMASGLFRFMGALGRNIIVAN
TFGSFALVAVLVLGGFVLSRDNVMKWWVWGYWASPMMYGQNAIAVNEFLGDSWSHVPPNSIESLGIMILKS
HGVFLEPYWYWLGVGATIGYIFLFNFFYTLALKFLNPFGKPQAVLSKEAFAEKLADKAGDSVELSSRGKNSSD
SRNESRRSVSSRTLSARVGSISEVNGNKKRGMVLPFEPLWITFDEINYAVDMPQEMKTQGVTEDRLQLLKGVT
GAFRPGVLTALMGISGAGKTTLMDVLAGRKTGGYIEGNITISGYPKKQETFARISGYCEQTDIHSPHVTVYESL
AYSASLRLPPEVDSTTRRMFIEEVMELVELISIREALVGLPGVNGLSTEQRKRLTIAVELVANPSIIFMDEPTSGL
DARAAAIVMRTVRNTVDTGRTVVCTIHQPSIDIFDAFDELLLLKRGGEEIYVGPLGRHSSHLIKYFEDIDGVPKI
KDGYNPATWMLEITAAAQEASIGVNFTEIYRNSELYGRNKAMIKELSTPPAGSKDLYFPTQYSQSFFSQCMACL
WKQHLSYWRNPPYSAVRLLFTTFIALMFGTIFWDLGSKRTSRQDLFNAMGSMYAAVLFIGVQNAASVQPVVA
IERTVFYRERAAGMYSALPYAFGQVVIELPYIFVQTIIYGVIVYTMIGFDWTVSKFLWYLFFMYFTFLYFTFYG
MMTVAVTPNHNIAAIVSSAFYAIWNLFSGFIVPRTRMPIWWRWYYWICPVSYTLYGLVASQFGDINDKFDTGE
SVEHFVRDYFGYRRDFLGAVAAVHVGICVLFGFTFAFSIKVFNFQKR

>XP_009589545.1_P_PDR1-l_Nto
MEGGEDSFRVSSARLSSSNVWRNSAMDVFSRSSREADDEEALKWAALEKLPTYLRIRRGILTEEEGQSREVDIT
KLDLVERRNLLERLVKIADEDNEKFLLKLKKRIARVGLDLPTIEVRFEHVSVDAEARVGSRALPTIFNFTVNILE
DFLNYLHILPSRKKPLPILHEVSGIIKPGRMTLLLGPPSSGKTTLLLALAGKLDKDLKVSGRVTYNGHGMDEFVP
QRSSAYISQNDLHIGEMTVRETLAFSARCQGVGAKYEILAELSRREKGANIKPDPDVDIFMKSAWNEGQEANV
VTDYTLKILGLEICADTLVGDEMIRGISGGQRKRLTTGEMMVGPARALFMDEISTGLDSSTTYQIVNSIRQSIHIL
QGTAVISLLQPAPETYDLFDDIILLSDGQIVYQGPRENVLEFFEYMGFMCPERKGVADFLQEVTSRKDQEQYW
ARRDEPYKYITVREFSESFQSFHIGRKLGDELAVPFDKSKSHPAALTTKNYGISKKELLKACTAREYLLMKRNS
FVYIFKMIQLTLMASITMTLFLRTEMHRNTTTDGAVFLGALFYAVIMIMFNGFSELALSIMKLPSFYKQRDLLFF
PAWAYALPTWILKIPVTLVEVAIWVCMTYYVIGFEADVGRFFKQLFLLICVNQMASGLFRFIGALGRNVIVANT
FGSCALLTVLVMGGFILSRDDVKKWWIWGYWISPMMYAQNAIAVNEFLGKSWAHVPPNSTGTETLGVSFLKS
RGIFPEARWYWIGAGALLGYVLLFNFMFTVALAYLNPFGKSQAVLSEETVAERNASKRGEVIELSPIEKRSSER
GNDVRRSASSRSMSSRVGSITEADLNKRRGMILPFEPLSITFDDIRYAVDMPQEMKAQGVAEDRLELLKGVSG
AFRPGVLTALMGVSGAGKTTLMDVLAGRKTGGYIDGTISISGYPKQQETFARIAGYCEQTDIHSPHVTVYESLQ
FSALLRLPREVDTETRKMFVEEVMELVELTPLREALVGLPGVNGLSTEQRKRLTVAVELVANPSIIFMDEPTSG
LDARAAAIVMRTVRNTVDTGRTVVCTIHQPSIDIFDAFDELLLLKRGGEEIYVGPLGRHSSHLIKYFEGIDGVPKI
KDGYNPATWMLEITSVAQEAARVIDFTELYKNSELYRRNKALIKELSVPAPCSKDLYFPTKYSQSFFTQCKACF
WKQHWSYWRNPPYTAVRLMFTFFIALMFGTIFWDLGSRRKRQQDLLNAIGSMYAAVLFLGVQNATSVQPVIA
IERTVFYRERAAGMYSALPYAFGQVMIELPYLFIQTIIYGVIVYVMIGFEWTVAKFFWYLFFMYFTLLYFTLYG
MMTVAVTPNHSIAAIISSAFYAIWNLFCGFVVPKTRMPVWWRWYYYICPVSWTLYGLIASQFGDLQDILDTNE
TVEQFIENFFDIKYDFVGYVAVILVGISVVFLFIFAYSIKAFNFQKR

>XP_009775073.1_P_PDR1-l_Ns
MEGGEDIFRVSSARLSSSNVWRNSAMDVFSRSSREADDEEALKWAALEKLPTYLRIRRGILTEEEGQSREVDIT
KLDLVERRNLLERLVKIADEDNEKFLLKLKKRIDRVGLDLPTIEVRFEHVSVDAEARVGSRALPTIFNFTVNILE
DFLNYLHILPSRKKPLPILHEVSGIIKPGRMTLLLGPPSSGKTTLLLALAGKLDKDLKVSGRVTYNGHGMDEFVP
QRSSAYISQNDLHIGEMTVRETLAFSARCQGVGAKYEILAELSRREKEANIKPDPDVDIFMKSAWNEGQEANV
VTDYTLKILGLEICADTLVGDEMIRGISGGQRKRLTTGEMMVGPARALFMDEISTGLDSSTTYQIVNSIRQSIHIL
QGTAVISLLQPAPETYDLFDDIILLSDGQIVYQGPRENVLEFFEYMGFMCPERKGVADFLQEVTSRKDQEQYRA
CRDEPYNFITVREFSESFQSFHIGRKLGDELAVPFDKSKSHPAALTTKRYGVSKKELLKACTAREYLLMKRNSF
VYIFKMIQLTLMASITMTLFLQTEMHRNTTTDGAVFLGALFYAVIMIMFNGFSELALSIMKLPSFYKQRDLLFFP
AWAYALPTWILKIPVTLVEVAIWVCMTYYVIGFEADVGRFFKQLFLLICVNQMASGLFRFIGALGRNVIVANTF
GSCALLTVIVMGGFILSRDDVKKWWIWGYWISPMMYAQNAIAVNEFLGKSWAHVPPNSTGTETLGVSFLKSR
GIFPEARWYWIGAGALLGYVLLFNFLFTVALAYLNPFGKSQAVLSEETVAERNASKRGEVIKLSLIEKHSSERL
NDVRRSASSRSMSSRVGSITEADLNKRRGMILPFEPLSITFDDIRYAVDMPQEMKAQGVAEDRLELLKGVSGAF
RPGVLTALMGVSGAGKTTLMDVLAGRKTGGYIDGTISISGYPKQQETFARIAGYCEQTDIHSPHVTVYESLQFS
AWLRLPHEVDTETRKMFVEEVMELVELTPLREALVGLPGVNGLSTEQRKRLTVAVELVANPSIIFMDEPTSGL

DARAAAIVMRTVRNTVDTGRTVVCTIHQPSIDIFDAFDELLLLKRGGEEIYVGPLGRHSSHLIKYFEGIDGVPKI
KDGYNPATWMLEITSVAQEAARGIDFTELYKNSELYRRNKALIKELSVPAPCSKDLYFPTKYSQSFFTQCMACF
WKQRWSYWRNPPYTAVRLMFTFFIALMFGTIFWDLGSRRKRQQDLLNAIGSMYVAVLFLGVQNATSVQPVIA
IERTVFYRERAAGMYSALPYAFGQVMIELPYLFIQTIIYGVIVYVMIGFEWTVAKFFWYLFFMYFTLLYFTLYG
MMTVAVTPNHSIAAIISSAFYAIWNLFCGFVVPKTRMPVWWRWYYYICPISWTLYGLIASQFGDIQDKLDTNE
TVEEFIESFFDFKYDFVGYVAVILVGISVVFLFIFAFSIKAFNFQKR

>XP_010023520.1_P_PDR1_Eg
MDGGDIYRAGNSLRATSSTSWRNQASMADVFSRSSRDEDDEEALRWAALEKLPTFKRLQKGILTAAGGANQI
DIWNLGFHDRKRLLERLMRVTEEDNEGFLLKLRNRIDRVGINLPTIEVRFEHLKVEAEAYEGSRALPTVINFCTS
ILEGFLNFLHVLPSRKKHSTILQDVSGIIKPGRMTLLLGPPSSGKTTLLLALAGKLDPELKTTGRVTYNGHVMNE
FVPQRTAAYISQHDLHIGEMTVRETLAFSARCQGVGSRYDMLSELSRREKAANIKPDPDIDIFMKAAVTKGQE
ANVVTDYILKILGLEICADAMVGDEMLRGISGGQRKRVTTGEMLVGPAEVFFMDEISTGLDSSTTYQIVNSLKQ
FIHILDGTAVISLLQPAPETYDLFDDIILLSDGQIVYQGPHELVLDFFESMGFKCPRRKCVADFLQEVTSRKDQH
QYWVRKDEPYTFVTVREFAEAFQSFHVGRKLGDELSTPFDKSKNHPAALTTKRFGVGMKDLLKACILREYLL
MKRNSFVYIFKVTQLIIMAFISMTLFLRNKMHRDTVTDGGVYIGALFFTMMTFMFNGMAELSMTIAKLPIFYK
QRDLLFYPAWAYALPSWILKIPITFVEVAAWVFITYYVIGYDPNVGRLFKQYLLLVAINQMASGLFRLIAALGR
NLIVANTFGSFVLLALVALGGVVLSREDVKKWWIWGYWTSPLMYGQNAIVVNELLGSNWNKIPPISSTNEPLG
IQVLKSRGFFTEAYWYWIGLGALFGFLILFNFGFSVALALLNPFGKSHTVKSDDPEGNKNVDRIEGSIQLQSRGS
SLRTGSGRSSKPETAVAANTKRGMVLPFEPHSITFDEITYSVDMPREMENQGAPEERLVLLKGVSGSFRPGVLT
ALMGVSGAGKTTLMDVLAGRKTGGYIEGNITISGYPKNQDTFARISGYCEQNDIHSPHVTVYESLIYSAWLRLP
PGVDDQTRKLFVEEVMELVELNPLRQALVGLPGVDGLSIEQRKRLTIAVELVANPSIIFMDEPTSGLDARAAAI
VMRTVRNTVDTGRTVVCTIHQPSIDIFEAFDELFLMKRGGQEIYVGPLGRHSSHLIEYFEGIQGVSKIKDGYNPA
TWMLEVTSPGQELALGVDFSDLYKNSDLYRRNKALIEELSIPPPNSKDLYFPTKYSQSTFTQLMACLWKQRWS
YWRNPPYTAVRFLFTIIIALMFGTMFWDLGSKTTRSQDLMNAMGSMYAAVVFLGIQNAASVQPVVAVERTVF
YRERAAGMYSALPYALAQVLIEVPYVLVQAISYGIIVYAMIGFKWTVAKFFWYLFFMFFTLLYFTYYGMMAL
GMTPNQHIASIVSSAFYAIWNLFSGFIVPRTRIPIWWRWYYWVCPVSWTLYGLVISQFGDLKNRLEDTGDTVEE
YMRDYFGYRHNFLGAIAAAMIGFTMLFAFIFAISIKILNFQRR

>XP_010023530.1_P_ABCG40_X1_Eg
MDGGDTYRAGNSLRATSSTTWRNHASMVDVFSRSSRDEDDEEALRWAALEKLPTFERLQKGIMTAGGGANQ
IDIWNLGFHDRKRLLERLVRVIEEDNEGFLLKLRNRIDRVGIDLPTIEVRFEHLKVEAEAHEGSRALPTVINFCTS
ILEGFLNFLHVLPSRKKHLTILQDVSGIIKPGRMTLLLGPPSSGKTTLLLALAGKLDPELKMTGRVTYNGHVMN
EFVPQRTAAYISQHDLHIGEMTVRETLAFSARCQGVGSRYDMLLELSRREKAANIKPDPDIDIFMKAAVTEGQE
ANVVTDYILKILGLEICADVMVGDEMLRGISGGQRKRVTTGEMLVGPAEVFLMDEISTGLDSSTTYQIVNFLKQ
FIHILDGTAVISLLQPAPETYDLFDDIILLSDGQIVYQGPRELVLDFFESMGFKCPRRKGVADFLQEVTSRKDQH
QYWVRKDEPYTFVTVREFVEAFQSFHVGRKLGDELSTPFDKSKNHPAALTTKRFGVGMKDLLKACILREYLL
MKRNSFVYIFKVTQLIIMAFISMTLFLRTKMHRDTVTDGGVYIGALFFTVITIMFNGMAEISMTIAKLPIFYKQR
DLLFYPAWAYALPSWILKIPITFVEVAAWVFTTYYVIGYDPNVGRLFKQYLLLVAINQMSSGLFRLIAALGRNL
IVANTFGTFVLLALVAMGGVVLSREDVNKWWIWGYWTSPLMYGQNAIVVNELLGSNWNKIPPNSSTNEPLGI
QVLKSRGFFTEAYWYWIGLGALFGFIILFNFGFSVALALLNPFGKSHTVKSDDPEGNKNVDRIEGSIQLQSRGSS
LRNGSGRSSKLTPPWPETAVAANTKRGMVLPFEPHSITFDEITYSVDMPREMTNQGAPEDRLVLLKGASGSFRP
GVLTALMGVSGAGKTTLMDVLAGRKTGGYIKGNITISGYPKNQDTFARISGYCEQNDIHSPHVTVYESLIYSA
WLRLPPGVDDQTRKLFVEEVMELVELNPLRQALVGLPGVDGLSIEQRKRLTIAVELVANPSIIFMDEPTSGLDA
RAAAIVMRTVRNTVDTGRTVVCTIHQPSIDIFEAFDELFLMKRGGQEIYVGPLGRHSSHLIEYFEGIQGVSKIKD
GYNPATWMLEVTSPAQELALGVDFSDLYKNSDLYRRNKALIEELSISPPNSKDLYFPTKYSQSTFTQLMACLW
KQHWSYWRNPPYTAVRFLFTISIALMFGTMFWDLGSKTTRSQDLFNAMGSMYAALVFLGFQNASSVQPVVA
VERTVFYRERAAGMYSALPYALAQVLIEVPYVLVQAISYGIIVYAMIGFKWTVAKFFWYLFFKFFTLLYFTYY
GMMAVGMTPNHHIASIVSLAFYALWNLFSGFVVPRTKIPIWWRWFYWVCPVSWTLYGLVVSQFGDLTNTLE
DTGDTVEEYMRDYFGYRHDFLGVIAAMVIGFTMLFAFVFAISIKILNFQRR

>XP_010023532.2_P__ABCG40_Eg
MDGGDIYRAGNSLRATSSTTWRNHASIADVFSRSSRDEDDEEALRWAALEKLPTFERLQKGILTAGGGANQIDI
WNLGFHDRKRLLERLVRVTEEDNEGFLLKLRNRIDRVGIDLPTIEVRFEHLKVEAEAHEGSRALPTVINFCTSIL

EGFLNFLHILPSRKKHLTILQDVSGIIKPGRMTLLLGPPSSGKTTLLLALAGKLDPELKTTGRVTYNGHVMNEFV
PQRTAAYISQQDLHIGEMTVRETLAFSARCQGVGSRYDMLSELSRREKAASIKPDPDVDIFMKAAVIEGQEAN
VVIDYILKILGLEICADAMVGDEMLRGISGGQRKRVTAGEMLVGPVEVFLMDEISTGLDSSTTYQIVNFLKQFIH
ILDGTAIISLLQPAPETYDLFDDIILLSDGQIVYQGPRELILDFFKSMGFKCPRRKGVADFLQEVTSRKDQHQYW
VRKDEPYTFVTVREFVEAFQSFHVGRKLGDELSTPFDKSKNHPAALTTKRFGVGMKDLLKACILREYLLMKRN
SFVYIFKVSQLIILAFISVTLFLRTKMHRDTVTDGGVYIGALFFTVITIMFNGMSEISMTIAKLPIFYKQRDLLFYP
AWAYALPSWILKIPITFVEVAAWVFITYYVTGYDPNVGRLFKHYLLLVAINQMASGLFRLIAALGRNLIVANTF
GSFVLLALVALGGVVLSREDVKKWWIWGYWTSPLMYGQNAIVVNELLGSNWNKIPPNSSTNEPLGIQVLKSR
GFFTEAYWYWIGLGALFGFIVLFNFGFSVALALLNPFGKSHTVKSDDPEGNKNVDRIEGSIQLQFRGSRRSSKPT
PAWPETAVAANTKRGMVLPFEPHSITFDEITYSVDMPREMTNQGAPEDRLVLLKGASGSFRPGVLTALMGVSG
AGKTTLMDVLAGRKTGGYIEGNITISGYPKNQDTFARISGYCEQNDIHSPHVTVYESLIYSAWLRLPPGVDDQT
RKLFVEEVMELVELNPLRQALVGLPGVDGLSIEQRKRLTIAVELVANPSIIFMDEPTSGLDARAAAIVMRTVRN
TVDTGRTVVCTIHQPSIDIFEAFDELFLMKRGGQEIYVGPLGRHSSHLIEYFEGIQGVSKIKDGYNPATWMLEVT
SPAQELTLGVDFSDLYKNSDLYRRNKALIEELSIPPPNAKDLYFPTKYSQSTFTQLMACLWKQHWSYWRNPPY
TAVRFLFTISIALMFGTMFWDLGSKTTRSQDLLNAMGSMYAAIFFLGMQNASSVQPVVAVERTVFYRERAAG
MYSALPYALAQVLIEVPYVLVQAISYGIIVYAMIGFKWTVVKFFWYLFFMFFTLLYFTYYGMMAVGMTPNHH
IASIVSFAFYAIWNLFSGFVVPRTRIPIWWRWFYWVCPVSWTLYGLVVSQFGNLTNRLEDTGETVEEYMRDYF
GYRHGFLGMIAAVMIGFTMLFVFVFAISIKILNFXRR

>XP_010049001.1_P_PDR1_Eg
MDTGDALKISSARISSSRLGSARLGSARLSSSNIWRTSGREIFSRSSRDEDDEEALKWAALEKLPTYLRIQRGILA
EEEGQITEVDIWNLGLIERRNLLERLLKTAEEDNEKFLLKLKQRIDRVGLDIPTIEVRFEHLNVEAEAHVGSRAL
PTIFNFTVNIVEGFLNCFHILPSRKKPLPILNDVSGIIKPHRMTLLLGPPGCGKTTLLLALAGKLDKDLKFSGRVT
YNGHEMEEFVPQRTSAYISQHDLHIGEMTVRETLAFSARCQGVGPRYDMLTELSRREKAANIKPDPDIDIFMKA
ASLKGQETNVVTDYILKILGLEICADIMVGDEMIRGISGGQKKRVTTGEMLVGPAKALFMDEISTGLDSSTTFQI
VNSLRQSVHILNGTALISLLQPAPETYDLFDDIILLSDGQIVYQGPRENVLEFFGHMGFRCPERKGVADFLQEVT
SRKDQEQYWANKDSPYRFVTVKEFSEAFQSFHIGRKLGDQLAMPFDKSRSHPAALTTEKYGVSRKELLRACIS
RELLLMKRNSFVYVFKMCQLILVAFITMTVFLRTEMHRDTVTDGGIYFGALFFTLIIIMFNGFSELAMTIMKLPV
FYKQRDFLFFPEWTYALPTWILKIPISIVEVAVWVFMTYYVIGYDPDAGRLFKQYLLLLCLNQMASALFRFIGA
VGRNMIVANTFGSFGLLAIMVLGGFIISRVDIKPWWIWGYWSSPMMYGQNAIAVNEFLGKQWRHIPANATEPL
GVSVLKSRGIFPEARWYWIGVGASIGYIVLFNFLFTVALKYLNPFGKPQAVLSKETLVEKVASKHGEVIELSSR
GSQRSSSKKESGHQGSAASGTLSTRGGSTNDSDPNRKRGMVLPFEPLSIAFDDVRYAVDMPQEMKAQGILEDR
LELLKGVSGAFRPGVLTALMGVSGAGKTTLMDVLAGRKTGGYIDGTITISGYPKQQETFARISGYCEQTDIHSP
HVTVYESLAYSAWLRLPSEVDSETRKMFIEEVMELIELTPLREALVGLPGVNGLSTEQRKRLTIAVELVANPSII
FMDEPTSGLDARAAAIVMRTVRNTVDTGRTVVCTIHQPSIDIFEAFDELFLLKRGGQEIYVGPLGQHSSHLIDYF
EGISGVSKIKDGYNPATWMLEVTAAAQEEILGFNFTEVYRNSELFRRNKTLINELSTPPPGSKDLYFPAQYSQSF
FTQCMACLWKQYWSYWRNPSYTAVRLLFTTFIALMFGTIFWNLGSKRGNQQDLFNAIGSMYAACLFIGFQNS
TSVQPVVAIERTVFYRERGAGMYSALPYAFAQVVIEIPYTFLQTVTYGVIVYSMIGFDWTASKFFWYIFCMFFS
LLYFTYYGMMSVAVTPNQHIAAITSSAFYAIWNLFSGFVVPRPRMPVWWRWYYWADPISWTLYGLVASQFG
DIKHQLDTGETVKDFVRSYFGYKHDFLGVVAAVLVGLPVLFGFIFAFSIRAFNFQKR

>XP_010249929.1_P_PDR1-l_Nn
MDGVDLYKVSSLRRNNSVWRSDAVEVFSRSSREEDDEEALKWAALEKLPTYDRIRKGILTGVGSQPTEVDIGN
LGFQEKKNLIERLVRVAEEDNEKFLLKLKNRIERVGIDLPTIEVRFEHLNINADAYVGSRALPTIFNFTANIVEGI
LNFLHILPSRKKPLCILQDVSGIIKPSRMTLLLGPPSSGKTTLLLALAGKLDPDLKVSGRVTYNGHGMDEFVPQR
TSAYISQHDLHIGEMTVRETLAFSARCQGVGERYDMLTELSRREKAANIKPDPDIDIYMKAATLEGQETSVVTD
YILKILGLDVCADTMVGDEMLRGISGGQRKRVTTGEMLVGPARALFMDEISTGLDSSTTYQIVSSLRQSIHILNG
TAVISLLQPAPETYNLFDDIILLSDGQVIYQGPREHVLDFFESMGFKCPERKGIADFLQEVTSKKDQKQYWARK
LKPYRFVTVKEFAEAFQSFHAGQKISDELSTPFDKTKNHAAALTTKKYGVSKKELFKACLSREILLMKRNSFVY
FFKMAQLAILAFITMTLFLRTEMHQDDSTDGGIYMGALFFALITVMFNGLSEISMTIAKLPVFYKQRDLLFYPA
WAYSLPTWILKIPITFIEVGIWVFITYYVIGFDPNVERFFRQYFLLLLINQMASALFRFIGATGRNMIVANTFGAF
AVLTFVVLGGFILSRNDVKNWWIWGYWISPLMYGQNAISVNEFLGKNWRHVLPNSTEPLGVTVIKSRGFFPEA
HWYWIGVGASIGYMFLFNFLYTVALHYLDPFGKPQAVLPDEGEARTGEDNELSSLEMTSSGHTTSGEQQDGII
RVASESSSARIDEVNQHKKRGMVLPFQPLSITFDEIRYFVDMPQEMKDQGVTEDRLELLKGVSGSFRPGVLTAL

MGVSGAGKTTLMDVLAGRKTGGYIEGNITISGYPKKQETFARISGYCEQNDIHSPHVTVYESLLYSAWLRLPSD
VDSSTRKMFIEEVMELVELNPLRGALVGLPGVNGLSTEQRKRMTIAVELVANPSIIFMDEPTSGLDARAAAIVM
RTVRNTVDTGRTVVCTIHQPSIDIFEAFDELFLMKRGGEEIYVGPLGRQSCHLIKYFEGIEGVSKIKDGYNPATW
MLEVTTMAQEEILGVNFAEVYKNSELHRRNKSLIQELSTPPPGSKDLYFPTMYSQSFFTQCMACLWKQHWSY
WRNPPYIAVRFIFTTFIALLFGTIFWDLGSKRSRQQDLFNAMGSMYAAVLFLGIQNASSVQPVVAVERTVFYRE
RAAGMYSALPYAFGQVMIEIPHIFVQAVVYGVIVYAMIGFEWTAAKFFWYLFFMYFTLLYFTFYGMMAVAVT
PNHNVAAIVSSAFYGIWNLFSGFIVPRTRMPVWWRWYYWICPVAWTLYGLVASQFGDIQDKIETDQTVEEFLR
SYFGFRHDFLGVVSAVIIGFTVLFAFIFAFSIRAFNFQRR

>XP_010249930.1_P_PDR1-l_Nn
MDSVDLYKVSSLRRNNSVWRSNGVEVFSRSSREDDDEEALKWAALEKLPTYDRIRKGILTGVGSQPTEVDIGS
LGYQEKKNLIERLVRVAEEDNEKFLLKLKNRIERVGIDLPTIEVRFEHLNINADAYVGSRALPTIFNFTANIFEGF
LNFLHILPSRKKPLCILHDVSGIIKPSRMTLLLGPPSSGKTTLLLALAGKLDPDLKVSGRVTYNGHGMDEFVPQR
TSAYISQHDLHIGEMTVRETLAFSARCQGVGARYDMLTELSRREKAANIKPDPDIDIYMKAATLEGQEASVVT
DYILKILGLDVCADTMVGDEMLRGISGGQRKRVTTGEMLVGPARALFMDEISTGLDSSTTYQIVSSLRQSIHILN
GTAVISLLQPAPETYNLFDDIILLSDGQVVYQGPREHVLDFFESMGFKCPERKGVADFLQEVTSKKDQKQYWA
RKHEPYRFVTVKEFAEAFQSFHVGQKLGDELSTPFDKTKSHPAALTTKKYGVSKKELFKASMSREILLMKRNS
FVYIFKMTQLAIMAFITMTLFLRTKMHRDDSTDGGIYLGALFFALITLMFNGLSEISMTIAKLPVFYKQRDLLFF
PAWAYSLPTWILKIPITFIEAGIWVFITYYVIGFDPNVERFFRQYFLLLLINQMASALFRFIGATGRNMIVANTFG
AFAVLTFVVLGGFILSRNDVKNWWIWGYWISPLMYGQNAISVNEFLGKNWRHVLPNSTEPLGVTVIKSRGFFP
EAHWYWIGVGATIGYMFLFNILYTVALHYLDPFGKPQAILPEGEARTGEDNELSSLEMTSSGHTTSGEQKDGII
GVASESSSARIDEVSQNRKRGMVLPFQPLSITFDEIRYFVDMPQEMKDQGVTEDRLELLKGVSGSFRPGVLTAL
MGVSGAGKTTLMDVLAGRKTGGYIEGSITISGYPKKQETFARISGYCEQNDIHSPHVTVYESILYSAWLRLPSD
VEPSTRKMFIEEVMELVELNSLRGALVGLPGVNGLSTEQRKRLTIAVELVANPSIIFMDEPTSGLDARAAAIVM
RTVRNTVDTGRTVVCTIHQPSIDIFEAFDELFLMKRGGEEIYVGPLGHNSCHLIKYFEEIEGVSKIKYGYNPATW
MLEVTTVAQEETLGVSFADVYKNSELYRRNKSLIQELSTPPPGSKDLYFPTKYSQSFFTQCMACLWKQHWSY
WRNPPYIAVRFIFTTFIALLFGTIFWDLGSKRSRQQDLFNAMGSMYAAVLFLGIQNASSVQPVVAVERTVFYRE
RAAGMYSALPYAFGQVMIEIPHIFVQAVVYGVIVYAMIGFEWTAAKFFWYLFFMYFTLLYFTFYGMMAVAVT
PNHNVAAIVSSAFYGIWNLFSGFIVPRTRMPVWWRWYYWICPVAWTLYGLVASQFGDIQDKIETDQTVEEFLR
SYFGFRHDFLGVVSAVIIGFTVLFAFIFAFSIRAFNFQRR

>XP_010254256.1_P_PDR1-l_Nn
MESADLYKVSSLRRNSSVWRNSAVEVFSRSSREEDDEEALKWAALEKLPTYNRLRKGILIGAGSELSEIDIGSL
GIQEKKDLLDRLVRVAEEDNEKFLSKLKNRIDRVGIDIPTIEVRFEHLSINADAYVGSRALPTILNFSVNIVEGLL
NHLHLLPSRKKPFSILQDVSGIIKPSRMALLLGPPSSGKTTLLLALAGKLDPDLKVSGRVTYNGHGLDEFVPQRT
SAYISQHDLHIGEMTVRETLAFSARCQGVGTGYEMLVELARREKAANIKPDPDIDVYMKASSLEGQEASVVTD
YILKILGLDICADTMVGDEMLRGISGGQRKRVTTGEMLVGPAKALFMDEISTGLDSSTTFQIVNSLRQSIHILNG
TAVISLLQPAPETYDLFDDIILLSDGEIVYQGPRENVLEFFESMGFKCPERKGVADFLQEVTSKKDQKQYWARK
DVPYSFVSAKEFAEAFHSFHVGKKLEEELATPFDKTKSHPAALSTKKYGVSKKELLKACSSREFLLMKRNSFV
YIFKMTQLTIVALITMTIFLRTKMHRRDDTDAGIFMGALFFTLIMIMFNGFSEMAMTIQKLPVFYKQRDLLFFPA
WAYSLPTWILKIPISFMEVAVWVFITYYVIGFDPNVERLFRQYFLLLLVNQVASALFRFIGSVGRNMIVANTFGS
FSLLAVLVLGGFILSRENVKKWWIWGYWVSPLMYGQNALAVNEFLGKSWRHVPLNKTETLGVSAIKSRGFFA
EARWYWIGVGAMIGYIFLFNFLFTVALTYLNPFEKPQAVISEETLSNKDTSRTREVEGSSSQGMSSTGRTTSGER
QDEIRRSVSSGFSSVRAETIDEVNENTRRGMVLPFQPLSITFDEIRYSVDMPQEMKNQGVTEDRLELLKGVSGA
FRPGVLTALMGVSGAGKTTLMDVLAGRKTGGYIEGNITISGYPKKQETFARISGYCEQNDIHSPYVTVYESLLY
SAWLRLPPDVDSSTRKMFVEEVMELVELNSLREALVGLPGVNGLSTEQRKRLTIAVELVANPSIIFMDEPTSGL
DARAAAIVMRTVRNTVDTGRTVVCTIHQPSIDIFEAFDELFLMKRGGQEIYVGPLGHHSCHLIKYFEGIEGVSKI
KDGYNPATWMLEVTTTAQEEILGVDFTEVYKRSELYRRNKSLIEELSTPSPGSKDLYFPTKYSQPFITQCMACL
WKQHLSYWRNPSYTAVRFLFTTFIALMFGTIFWDLGSKRGRKQDLINAIGSMYAAVLFLGVQNSSAVQPVVA
VERTVFYREKAAGMYSALPYAYAQVMIELPHIFVQASVYGVIVYAMIGFEWTAAKFFWHIFFMYFTLLYFTFY
GMMAVAVTPNHNIAAIVSSAFYLIWNLFSGFIVPRTRIPVWWRWYYWACPVAWTLYGLSVSQFGDIEDRLDT
GETVESYLREYFGFKHDFLGVVAAVIVGFTVLFVFIFAFSIRAFNFQRR

>XP_010254262.1_P_PDR1-l_X2_Nn

MESGELYRVGSSLRIDSSSIRRNSIVDDVFQRSSRHEDDEEALKWAALEKLPTYNRIRKGLLSGVGGETKEIDIE
SLSLQEKKVLLERLLKIADEDNENFLLKLKNRIDRVGLDIPKIEVRFEHLNVDAEAYVGSRALPTIFNYSVNMFE
EFLNYLHILPSRKKPLSILHDVSGIIKPCRMTLLLGPPGSGKTTLLLALAGKLGSDVKFSGRVTYNGHKMNEFVP
QRTAAYISQYDLHIGEMTVRETLAFSARCQGVGTGYEMLAELSRREKETNIKPDPDIDIYMKAAALEGQEASV
VTDYILKILGLEICADTLVGDEMFRGISGGQKKRVTTGEMLVGPARALFMDEISTGLDSSTTFQILNSLRQSIHIL
NGTALISLLQPAPETYELFDDIILLSDGQIVYQGPRENVLEFFESMGFKCPERKGVADFLQEVTSWKDQEQYWA
RKDEPYRFIPVKEFAEAFQSFHVGLKLGDELANPFDKTKSHPASLTTKKYGVSKKELLKACISRELLLMKRNYF
VYIFKMTQLTILGFIAMTLFLRIKMHRDSVIDGGIYIGALFFTLMMIMFNGYSELALTILKLPVFYKQRDLLFYPS
WAYSLPTWILKIPYTFFEVAVWVFMTYYVIGFDPNVGRLFKQYLLLLGVNQMASGLFRFIGAVGRDMIVANTF
GSFSLLAILVMGGFVVSREDVKKWWKWGYWISPMMYGQNAIAVNEFLGNSWSHVPPNSTEPLGVTVLKSRGI
FPKAYWYWLGVGALVGYMFLFNVLFTLALSYLNPFGKPQPVVSEESLNEKWANRTGEFIKLSSREKSSVCQTS
STEEGNEMRSMSSGISPASTETINGNQNRKRGMVLPFQPLSITFDEIRYSVDMPQELKAQGVPEDQLELLKGVS
GSFRPGVLTALMGVSGAGKTTLMDVLAGRKTCGYIDGCITISGYPKKQETFARISGYCEQNDIHSPHVTVYEAL
LYSAWLRLPLEVKSATREMFVEEVMELVELTSLREALVGLPSVNGLSTEQRKRLTIAVELVANPSIIFMDEPTSG
LDARAAAIVMRTVRNTVDTGRTVVCTIHQPSIDIFDAFDELLLLKQGGEEIYVGPLGRHSCHLIKYFEGIQGVGK
INDGYNPATWMLEVTSRAQETVLGVNFTDVYKSSELYRRNKALINELNTPAPGSSDLYFPTRYSQSFFTQCMA
CLWKQHYSYWRNPPYTAVRLLFTTFTALMFGTIFWDLGSRRSKQQDLFNAMGSMYAAVLFLGIQNASSVQPV
VAIERTVFYRERAAGMYSALPYAFGQVMIEVPYIFLQTLIYGVIVYSMIGFEWTVAKFFWHLFFMYMTLLYFT
CYGMMTVAVTPNHNIAAIIATAFYAIWNLFSGFIVPRPRIPIWWRWYYWVCPVSWTLYGLVASQFGDVEEKLD
SGETVEEFLRSYFGFRHDFIGVVAVVVVGFTVLFGFIFAFSIRAFNFQKR

>XP_010459089.1_P_ABCG40_X1_Cs
MEGTSFQKASNSLRRNSSAWRKDSGMEIFSRSSREEDDEEALRWAALEKLPTFDRLRKGILTASHAGGAINEID
IQKLGFQDTKKLLERLIKVGDDEHEKLLWKLKQRTDRVGIDLPTIEVRFDNLKVEAEVHVGGRALPTLVNFISN
FADKLLNTLHLVPNQKKKFTILNDVSGIVKPGRMALLLGPPSSGKTTLLLALAGKLDKELKQTGRVTYNGHG
MNEFVPQRTAAYIGQNDVHIGEMTVRETFAFAARFQGVGSRYDMLTELARREKEANIKPDPDIDVFMKALSTA
GEETNVMTDYILKILGLEVCADTMVGDDMLRGISGGQKKRVTTGEMLVGPSRALFMDEISTGLDSSTTYQIVN
SLRNYVHIFNGTALISLLQPAPETFNLFDDIILIAEGEIIYEGPRDHVVEFFETMGFKCPPRKGVADFLQEVTSKK
DQMQYWSRPDEPYRFIRVREFAEAFQSFHVGRRIGDELALPFDKRKSHPAALTTKKYGVGIKELLKTSFSREYL
LMKRNSFVYYFKFGQLLVMAFLTMTLFFRTELQKKTVVDGSLYTGALFFVLMMLMFNGMSELSMTIAKLPVF
YKQRDLLFYPAWVYSLPPWLLKIPISFIEAALTTFITYYVIGFDPNVGRLFKQYILLVLMNQMASGLFKMVAAL
GRNMIVANTFGAFAMLVFFALGGVVLAREDIKKWWIWGYWISPIMYGQNALMANEFFGHSWSRAVANSSET
LGVTVLKSRGFLPHAYWYWIGTGALLGFVILFNFGFTVALTFLNSLGKPQAVMAEEPASDGTEQELQSARTNG
VAEVGANKKRGMVLPFEPHSITFDNVVYSVDMPQEMIEQGTQEDKLVLLKGVNGAFRPGVLTALMGVSGAG
KTTLMDVLAGRKTGGYIDGNITISGYPKNQQTFARISGYCEQTDIHSPHVTVYESLVYSAWLRLPKEVDSNKRK
MFIDEVMELVELTPLRQALVGLPGESGLSTEQRKRLTIAVELVANPSIIFMDEPTSGLDARAAAIVMRTVRNTV
DTGRTVVCTIHQPSIDIFEAFDELFLLKRGGEEIYVGPLGHESTHLINYFESIQGISKITEGYNPATWMLEVSNTSQ
EAALGVDFAQLYKNSDLYKRNKELIKELSQPAPGSKDLYFPTQYSQSFWTQCMASLWKQHWSYWRNPPYTA
VRFLFTIGIALMFGTMFWDLGGKTKTTQDLSNAMGSMYTAVLFLGLQNAASVQPVVNVERTVFYREQAAGM
YSAMPYAFAQVFIEIPYVFVQAVVYGLIVYAMIGFEWTAVKFFWYLFFMYGSFLTFTFYGMMAVAMTPNHHI
ASVVSSAFYGIWNLFSGFLIPRPSMPVWWEWYYWLCPVAWTLYGLIASQFGDITEPMADGTTVKQFIKDFYGY
REGFLGVVAAMNVIFPLLFAVIFAVGIKSFNFQKR

>XP_010459090.1_P_ABCG40_X2_Cs
MLTELARREKEANIKPDPDIDVFMKALSTAGEETNVMTDYILKILGLEVCADTMVGDDMLRGISGGQKKRVTT
GEMLVGPSRALFMDEISTGLDSSTTYQIVNSLRNYVHIFNGTALISLLQPAPETFNLFDDIILIAEGEIIYEGPRDH
VVEFFETMGFKCPPRKGVADFLQEVTSKKDQMQYWSRPDEPYRFIRVREFAEAFQSFHVGRRIGDELALPFDK
RKSHPAALTTKKYGVGIKELLKTSFSREYLLMKRNSFVYYFKFGQLLVMAFLTMTLFFRTELQKKTVVDGSLY
TGALFFVLMMLMFNGMSELSMTIAKLPVFYKQRDLLFYPAWVYSLPPWLLKIPISFIEAALTTFITYYVIGFDPN
VGRLFKQYILLVLMNQMASGLFKMVAALGRNMIVANTFGAFAMLVFFALGGVVLAREDIKKWWIWGYWISP
IMYGQNALMANEFFGHSWSRAVANSSETLGVTVLKSRGFLPHAYWYWIGTGALLGFVILFNFGFTVALTFLNS
LGKPQAVMAEEPASDGTEQELQSARTNGVAEVGANKKRGMVLPFEPHSITFDNVVYSVDMPQEMIEQGTQED
KLVLLKGVNGAFRPGVLTALMGVSGAGKTTLMDVLAGRKTGGYIDGNITISGYPKNQQTFARISGYCEQTDIH
SPHVTVYESLVYSAWLRLPKEVDSNKRKMFIDEVMELVELTPLRQALVGLPGESGLSTEQRKRLTIAVELVAN

PSIIFMDEPTSGLDARAAAIVMRTVRNTVDTGRTVVCTIHQPSIDIFEAFDELFLLKRGGEEIYVGPLGHESTHLI
NYFESIQGISKITEGYNPATWMLEVSNTSQEAALGVDFAQLYKNSDLYKRNKELIKELSQPAPGSKDLYFPTQY
SQSFWTQCMASLWKQHWSYWRNPPYTAVRFLFTIGIALMFGTMFWDLGGKTKTTQDLSNAMGSMYTAVLFL
GLQNAASVQPVVNVERTVFYREQAAGMYSAMPYAFAQVFIEIPYVFVQAVVYGLIVYAMIGFEWTAVKFFW
YLFFMYGSFLTFTFYGMMAVAMTPNHHIASVVSSAFYGIWNLFSGFLIPRPSMPVWWEWYYWLCPVAWTLY
GLIASQFGDITEPMADGTTVKQFIKDFYGYREGFLGVVAAMNVIFPLLFAVIFAVGIKSFNFQKR

>XP_010476642.1_P_ABCG40-l_Cs
MEGTSFQKASNSLRRNSSAWRKDSGMEIFSRSSREEDDEEALRWAALEKLPTFDRLRKGILTASHAGGAINEID
IQKLGFQDTKKLLERLIKVGDDEHEKLLWKLKQRTDRVGIDLPTIEVRFDNLKVEAEVHVGGRALPTLVNFISN
FADKLLNTLHLVPNQKKKFTILNDVSGIVKPGRMALLLGPPSSGKTTLLLALAGKLDKELKQTGRVTYNGHG
MNEFVPQRTAAYIGQNDVHIGEMTVRETFAFAARFQGVGSRYDMLTELARREKEANIKPDPDIDVFMKALSTA
GEETNVMTDYILKILGLEVCADTMVGDDMLRGISGGQKKRVTTGEMLVGPSRALFMDEISTGLDSSTTYQIVN
SLRNYVHIFNGTALISLLQPAPETFNLFDDIILIAEGEIIYEGPRDHVVEFFETMGFKCPPRKGVADFLQEVTSKK
DQMQYWSRPDEPYRFIRVREFAEAFQSFHVGRRIGDELALPFDKKKSHPAALTTKKYGVGIKELLKTSFSREYL
LMKRNSFVYYFKFGQLLVMAFLTMTLFFRTELQKKTVVDGSLYTGALFFVLMMLMFNGMSELSMTIAKLPVF
YKQRDLLFYPAWVYSLPPWLLKIPISFIEAALTTFITYYVIGFDPNIGRLFKQYILLVLMNQMASGLFKMVAALG
RNMIVANTFGAFAMLVFFALGGVVLAREDIKKWWIWGYWISPIMYGQNALMANEFFGHSWSRAVANSSETL
GVTVLKSRGFLPHAYWYWIGTGALLGFVILFNFGFTVALTFLNSLGKPQAVMAEDPASDGNETELQSARTNGI
AEVGANKKRGMVLPFEPHSITFDNVVYSVDMPQEMIEQGTQEDKLVLLKGVNGAFRPGVLTALMGVXXXXX
XXXXXXLAGRKTGGYIDGNITISGYPKNQQTFARISGYCEQTDIHSPHVTVYESLVYSAWLRLPKEVDSNKRK
MFIDEVMELVELTPLRQALVGLPGESGLSTEQRKRLTIAVELVANPSIIFMDEPTSGLDARAAAIVMRTVRNTV
DTGRTVVCTIHQPSIDIFEAFDELFLLKRGGEEIYVGPLGHESTHLINYFESIQGISKITEGYNPATWMLEVSNTSQ
EAALGVDFAQLYKNSDLYKRNKELIKELSQPAPGSKDLYFPTQYSQSFWTQCMASLWKQHWSYWRNPPYTA
VRFLFTIGIALMFGTMFWDLGGKTKTTQDLSNAMGSMYTAVLFLGLQNAASVQPVVNVERTVFYREQAAGM
YSAMPYAFAQVFIEIPYVFVQAVVYGLIVYAMIGFEWTAVKFFWYLFFMYGSFLTFTFYGMMAVAMTPNHHI
ASVVSSAFYGIWNLFSGFLIPRPSMPVWWEWYYWLCPVAWTLYGLIASQFGDITEPMADGTTVKQFIKDFYGY
REGFLGVVAAMNVIFPLLFAVIFAVGIKSFNFQKR

>XP_010497221.1_P_ABCG40_Cs
MEGTSFQKASNSLRRNSSAWRKDSGMEIFSRSSREEDDEEALRWAALEKLPTFDRLRKGILTASHAGGAINEID
IQKLGFQDTKKLLERLIKVGDDEHEKLLWKLKQRTDRVGIDLPTIEVRFDNLKVEAEVHVGGRALPTLVNFISN
FADKLLNTLHLVPNQKKKFTILNDVSGIVKPGRMALLLGPPSSGKTTLLLALAGKLDKELKQTGKVTYNGHG
MNEFVPQRTAAYIGQNDVHIGEMTVRETFAFAARFQGVGSRYDMLTELARREKEANIKPDPDIDVFMKALSTA
GEETNVMTDYILKILGLEVCADTMVGDDMLRGISGGQKKRVTTGEMLVGPSRALFMDEISTGLDSSTTYQIVN
SLRNYVHIFNGTALISLLQPAPETFNLFDDIILIAEGEIIYEGPRDHVVEFFETMGFKCPPRKGVADFLQEVTSKK
DQMQYWSRPDEPYRFIRVREFAEAFQSFHVGRRIGDELALPFDKKKSHPAALTTKKYGVGIKELLKTSFSREYL
LMKRNSFVYYFKFGQLLVMAFLTMTLFFRTELQKKTVVDGSLYTGALFFVLMMLMFNGMSELSMTIAKLPVF
YKQRDLLFYPAWVYSLPPWLLKIPISFIEAALTTFITYYVIGFDPNVGRLFKQYILLVLMNQMASGLFKMVAAL
GRNMIVANTFGAFAMLVFFALGGVVLAREDIKKWWIWGYWISPIMYGQNALMANEFFGHSWSRAVANSSET
LGVTVLKSRGFLPHAYWYWIGTGALLGFVILFNFGFTVALTFLNSLGKPQAVMAEDPASDGNETELQSARANG
VAEVGANKKRGMVLPFEPHSITFDNVVYSVDMPQEMIEQGTQEDRLVLLKGVNGAFRPGVLTALMGVSGAG
KTTLMDVLAGRKTGGYIDGNITISGYPKNQQTFARISGYCEQTDIHSPHVTVYESLVYSAWLRLPKEVDSNKRK
VIXXEVMELVELTPLRQALVGLPGESGLSTEQRKRLTIAVELVANPSIIFMDEPTSGLDARAAAIVMRTVRNTV
DTGRTVVCTIHQPSIDIFEAFDELFLLKRGGEEIYVGPLGHESTHLINYFESIQGISKITEGYNPATWMLEVSNTSQ
EAALGVDFAQLYKNSDLYKRNKELIKELSQPAPGSKDLYFPTQYSQSFWTQCMASLWKQHWSYWRNPPYTA
VRFLFTIGIALMFGTMFWDLGGKTKTTQDLSNAMGSMYTAVLFLGLQNAASVQPVVNVERTVFYREQAAGM
YSAMPYAFAQVFIEIPYVFVQAVVYGLIVYAMIGFEWTAVKFFWYLFFMYGSFLTFTFYGMMAVAMTPNHHI
ASVVSSAFYGIWNLFSGFLIPRPSMPVWWEWYYWLCPVAWTLYGLIASQFGDITEPMADGTTVKQFIKDFYGY
REGFLGVVAAMNVIFPLLFAVIFAVGIKSFNFQKR

>XP_010532149.1_P_ABCG40-l_X1_Th
MEGTSLQKASSSMRRNSSRWRRDTGMEAFSRSVREENDEEALRWAALERLPTVDRLRKGILTASHGGGVSEV

DIHNLGFQEKKKLLERLMKAGDEDHQKLLQKLRDRIDSVGIEVPTIEVRFEHLTVEAEAHVGGRALPTFVNFIS
NIAEGFLNNLHILPSRKKKLTILKDVSGIIKPSRMTLLLGPPSSGKTTLLLALAGKLGSELKQTGNVTYNGHGMN
EFVPQRTAAYISQHDTHIGEMTVRETFAFAARCQGVGSRYDMLAELSRREKQANVKPDPDIDVFMKAISMEGQ
EASVITDYMLKVLGLEVCADTLVGDEMLRGISGGQKKRVTTGEMLVGPAGALFMDEISTGLDSSTTYQIVNSI
RNYIHIFNGTAVISLLQPAPETYDLFDDIILISDGHIVYEGPRDHVLEFFQSVGFKCPQRKGVADFLQEVTSRKDQ
MQYWARPEEAYRFITVEEFAEAFQSFHVGQSLGDELGLPFDKTKSHPAALTTEKYGVGPKELLKANFSREYLL
MKRNSFVYYFKFGQLFVMSFLTLTLFFRTEMHKRNILDGTVYQGALFFILTMLMFNGMSELSMTIAKLPVFYK
QRDLLFYPAWVYSLPPWLLKVPISFIEAAITVSTTYYVVGFDPNVGRVFKQYLLLVLVNQMASGLFKFIAGVGR
NMIVANTFGSFALLVFFALGGMVLSRDDIKKWWIWGYWISPMMYGQNAIVANEFLGHSWSRLAPNSAESLGV
TILKSRGFFAHAYWYWIGVAATVGFTLLFNFCFALSITFLNAIGKPQAILPEEHASGGNDPGNETELRVGAEGN
GNRKRGMVLPFEPHSLTFDNVTYSVDMPQEMKERGVGEDRLVLLKGVSGAFRPGVLTALMGVSGAGKTTLM
DVLAGRKTGGYIDGNITISGYPKKQETFARISGYCEQTDIHSPHVTVYESLLYSAWLRLPKEVDSEKRKMFIEEV
MELVELTPLRQALVGLPGVNGLSTEQRKRLTIAVELVANPSIIFMDEPTSGLDARAAAIVMRTVRNTVDTGRTV
VCTIHQPSIDIFEAFDELFLLKRGGEEIYVGPLGHHSTHLINYFEGIQGISKIKDGYNPATWMLEVSNIAQEIALG
MNFTEIYKNSELYRRNKEIIKELSQPAPASKDLYFPTQYAQSFMTQCMASLWKQHWSYWRNPPYTSVRFLFTV
GIAVMFGTMFWDLGGKTTKRQDLFNAMGSMYTAVLFLGLQNAASVQPVVAVERTVFYREKAAGMYSAMPY
AFAQVLIEIPYVLVQTVAYGLIVYSMMGFEWTAAKFFWYLFFMCTSLLTFTFYGMMAVAMTPNHHIAAVVSS
AFYALWNLFSGFIVPRPSIPVWWRWYYWMCPVSWTLYGLITSQFGDVDDMMAEGATVKQFVRGYYGFRHDF
LGVVAVVNLAFPLLFAVIFAVGIKTFNFQSR

>XP_010537955.1_P_ABCG40_X1_Th
MEGTSFHKASSSLRRNSSVWRRDTGMEVFSRSSREEDDEEALRWAALEKLPTFDRLRKGILTASHGGVNEIDIH
SLGFQEKKKLLERLMKVGDEDHEKLLWSLKNRIDRVGIEVPTIEVRFDHLTVEAEAHVGGRALPTFVNFISNIV
EGFLNKLHFLPSGKQKFTILRDVSGVIKPSRMTLLLGPPSSGKTTLLLALAGKLDTELKQSGNVTYNGHGMNEF
VPQRTAAYISQHDVHIGEMTVRETFAFAARCQGVGSRYDMLSELARREKQANIKPDPDIDVFMKAMSTEGQE
TSVVTDYMLKILGLEVCADTMVGDEMLRGISGGQKKRVTTGEMLVGPARALFMDEISTGLDSSTTYQIVNSIR
DYIHIFNGTTVISLLQPAPETYGLFDDIILISDGQIVYEGPREHVLEFFESMGFKCPQRKGVADFLQEVTSRKDQM
QYWARTEEPYRFITVKEFAEAFQSFHVGRRLGDELGLPFDKTRSHPAALTTHKFGVGLKELLKANFSREYLLM
KRNSFVYYFKFGQLLVMSFVTLTLFFRTEMHKKTEVDGTIYQGALFFMMMMLMFNGMSELSMTIAKLPVFYK
QRDLLFYPAWVYSLPPWLLKIPITLIEAAITVFTTYYVIGFDPNVGRLFKQYLLLVLINQMASGLFKFMAGIGRN
MIVANTFGAFAMLVFFALGGMVLSRDDIKKWWIWGYWISPIMYGQNAIVANEFLGNSWSRPAPNSTESLGVF
VLKSRGFFTDAYWYWIGVGALLGFIVLFNFFFTLSITFLNPFGKPQANLPEDAATEKENPSNGTQPDISNTGNAA
EANDNRQRGMVLPFEPHSLTFDNVTYSVDMPQEMKEQGFQEDRLVLLKGVSGAFRPGVLTALMGVSGAGKT
TLMDVLAGRKTGGYIDGDVMISGYPKKQETFARISGYCEQNDIHSPHVTVYESLLYSAWLRLPREVDSETRKM
FIEEVMELVELTPLRQALVGLPGVNGLSTEQRKRLTIAVELVANPSIIFMDEPTSGLDARAAAIVMRTVRNTVD
TGRTVVCTIHQPSIDIFEAFDELFLLKRGGEEIYVGPLGHQSGHLINYFESIQGISKIKDGYNPATWMLEVSTTAQ
EIALEVDFAQIYKNSDRYRRNKELIKELSQPAPGSEDLYFPTQYSQSFLTQCMASLWKQHWSYWRNPPYTAVR
FLFTIGIALMFGTMFWDLGGKLTKRQDLFNAMGSMYTAVLFLGLQNAASVQPVVAVERTVFYREQAAGMYS
AMPYAFAQVLIEMPYVLVQAVVYGLIVYAMMGFEWTAAKFFWYLFFMYGSLLTFTFYGMMAVAMTPNHHI
ASVVSSAFYGIWNLFSGFIIPRPSIPVWWRWYYWICPVSWTLYGLVASQFGDITEPLAEGPTVKQFIKDYYGFR
HGFLGVVAAVNVAFPVLFAVIFAVAIKFFNFQKR

>XP_010648603.1_P_PDR1_X1_Vv
MESSDVYRVNSARLSSSNIWRNSGMEVFSRSSRDEDDEEALKWAAIEKLPTYLRIRRGILAEEEGKAREIDITSL
GLIEKKNLLERLVKIAEEDNEKFLLKLKERIDRVGLDIPTIEVRFEHITVDAEAYIGGRALPTIINFSANMLEVIHF
LDYINLCMYTLCSNLLSCKQSHAMIFLSLKGFLNYLHILPSRKKPLPILHDVSGIIKPGRMTLLLGPPSSGKTTLL
LTLAGKLGSDLKLSGRVSYNGHGMDEFVPQRSSAYISQYDLHIGEMTVRETLAFSARCQGVGTGYDMLAELS
RREKVANIKPDPDIDIYMKAAALKGQGGSLITDYILKILGLEVCADTIVGDEMVRGISGGQKRRLTTGEMLVGP
AKALFMDEISTGLDSSTTFQIVNSIRQSIHILKGTAIISLLQPAPETYDLFDDIILLSDGQIVYQGPRENVLEFFEHM
GFKCPERKGVADFLQEVTSKKDQEQYWAHRGEPYSFVTVTEFSEAFQSFHVGRRLGDELAIPFDKAKAHTAAL
TTKKYGVSKKELLKACISRELLLMKRNSFVYIFKMSQLILLAFIMMTLFLRTDMPRKTIADGWIFLGSMFFTLM
MIMFNGFSELALTIMKLPVFYKQRDLLFYPSWAYSLPTWILKIPITLVEVAIWVFMTYYVVGFDPNIERFFRQYL
LLLCVNQMASGLLRLMAALGRNIIVANTFGSFALLAVLVMGGFVLSKDDVKPWWMWGYWISPMMYGQNAI
AVNEFLGKSWRHVPENATEPLGVLVLKSRGIFPEAYWYWLGVGALIGYVFLFNFLFTVALAYLNPYGKHQTV

LSEETLTEQSSRGTSCTGGDKIRSGSSRSLSARVGSFNNADQNRKRGMILPFEPLSITFDEIRYAVDMPQEMKSQ
GIPENRLELLKGVSGSFRPGVLTALMGVSGAGKTTLMDVLAGRKTGGYIDGSIKISGYPKNQKTFARISGYCEQ
TDIHSPHVTVYESLLYSAWLRLPPEVDSATRKMFIEEVMELVELNSLRQALVGLPGVDGLSTEQRKRLTVAVE
LVANPSIIFMDEPTSGLDARAAAIVMRTVRNTVDTGRTVVCTIHQPSIDIFDAFDELFLLKRGGEEIYAGPLGHH
SAHLIKYFEGIDGVSKIKDGYNPATWMLEVTSAAQEAALGINFTDVYKNSELYRRNKALIKELSTPPPGSKDLY
FPTQYSQSFFAQCKTCLWKQHWSYWRNPSYTAVRLLFTTFIALMFGTIFWDLGSRRQRKQDLFNAMGSMYCA
VLFIGAQNATSVQPVVAIERTVFYREKAAGMYSALPYAFGQVMIELPYILIQTIIYGVIVYAMIGFDWTMTKFF
WYIFFMYFTFLYFTFYGMMAVAVSPNHNIAAIISSAFYAIWNLFSGFIVPRTRIPVWWRWYYWCCPISWTLYGL
IGSQFGDMKDKLDTGETIEDFVRSYFGFRNDFLGIVAVVIVGITVLFGFTFAYSIRAFNFQKR

>XP_010648604.1_P_PDR1_X2_Vv
MESSDVYRVNSARLSSSNIWRNSGMEVFSRSSRDEDDEEALKWAAIEKLPTYLRIRRGILAEEEGKAREIDITSL
GLIEKKNLLERLVKIAEEDNEKFLLKLKERIDRVGLDIPTIEVRFEHITVDAEAYIGGRALPTIINFSANMLEGFLN
YLHILPSRKKPLPILHDVSGIIKPGRMTLLLGPPSSGKTTLLLTLAGKLGSDLKLSGRVSYNGHGMDEFVPQRSS
AYISQYDLHIGEMTVRETLAFSARCQGVGTGYDMLAELSRREKVANIKPDPDIDIYMKAAALKGQGGSLITDYI
LKILGLEVCADTIVGDEMVRGISGGQKRRLTTGEMLVGPAKALFMDEISTGLDSSTTFQIVNSIRQSIHILKGTAI
ISLLQPAPETYDLFDDIILLSDGQIVYQGPRENVLEFFEHMGFKCPERKGVADFLQEVTSKKDQEQYWAHRGEP
YSFVTVTEFSEAFQSFHVGRRLGDELAIPFDKAKAHTAALTTKKYGVSKKELLKACISRELLLMKRNSFVYIFK
MSQLILLAFIMMTLFLRTDMPRKTIADGWIFLGSMFFTLMMIMFNGFSELALTIMKLPVFYKQRDLLFYPSWAY
SLPTWILKIPITLVEVAIWVFMTYYVVGFDPNIERFFRQYLLLLCVNQMASGLLRLMAALGRNIIVANTFGSFAL
LAVLVMGGFVLSKDDVKPWWMWGYWISPMMYGQNAIAVNEFLGKSWRHVPENATEPLGVLVLKSRGIFPE
AYWYWLGVGALIGYVFLFNFLFTVALAYLNPYGKHQTVLSEETLTEQSSRGTSCTGGDKIRSGSSRSLSARVG
SFNNADQNRKRGMILPFEPLSITFDEIRYAVDMPQEMKSQGIPENRLELLKGVSGSFRPGVLTALMGVSGAGKT
TLMDVLAGRKTGGYIDGSIKISGYPKNQKTFARISGYCEQTDIHSPHVTVYESLLYSAWLRLPPEVDSATRKMFI
EEVMELVELNSLRQALVGLPGVDGLSTEQRKRLTVAVELVANPSIIFMDEPTSGLDARAAAIVMRTVRNTVDT
GRTVVCTIHQPSIDIFDAFDELFLLKRGGEEIYAGPLGHHSAHLIKYFEGIDGVSKIKDGYNPATWMLEVTSAAQ
EAALGINFTDVYKNSELYRRNKALIKELSTPPPGSKDLYFPTQYSQSFFAQCKTCLWKQHWSYWRNPSYTAVR
LLFTTFIALMFGTIFWDLGSRRQRKQDLFNAMGSMYCAVLFIGAQNATSVQPVVAIERTVFYREKAAGMYSAL
PYAFGQVMIELPYILIQTIIYGVIVYAMIGFDWTMTKFFWYIFFMYFTFLYFTFYGMMAVAVSPNHNIAAIISSAF
YAIWNLFSGFIVPRTRIPVWWRWYYWCCPISWTLYGLIGSQFGDMKDKLDTGETIEDFVRSYFGFRNDFLGIVA
VVIVGITVLFGFTFAYSIRAFNFQKR

>XP_010648606.1_P_PDR1_Vv
MESSDISRVTSGRITASNILRNSSVEVFSRSSREEDDEEALKWAALEKLPTFLRIQRGILTEEKGQTREINIKSLGL
PERKNLIQRLVKIDGHDNEKFLLKLKERIDRVGLDIPTVEVRFEHLTVDAEAYVGSRALPTIFNFSANILEGFLN
YLHILPSRKKPFSILHDVSGIIKPRRMTLLLGPPSSGKTTLLLALAGRLGSDLKVSGRVTYNGHGMDEFVPQRTS
AYTSQYDLHAGEMTVRETLDFSARCQGVGGLSDMLAELSRREKAANIKPDPDIDIYMKAAALEGQKTSVVTE
YMLKILGLEICADTLVGDVMKQGISGGQKKRLTTGEILVGPARALFMDEISTGLDSSTAFQIVNSLRQSIHILNG
TALISLLQPAPETYNLFDDIILLSDGKIVYQGPCENVLEFFGYMGFKCPERKGVADFLQEVTSRKDQEQYWARK
DEPYSYVTVKEFAEAFQSFHIGQKLGDELAVPFDKTKGHPAALTTKKYGISKRELLRACTSREFLLMKRNSFVL
FFLFFQLIIVAFINMTLFLRTEMSRNTVEDGGIFMGALFFAVLMIMFNGFTELPMTIFQLPVFYKQRDLLFFPSWA
YSLPKWILKMPIAFAEVGAWVIMTYYVIGFDPNIERFFKQYLLLLCIHQMASGLLRLMAALGRNIIVANTFGSF
ALLVVMVLGGFVLSKDDVKTWWEWGYWVSPLMYGQNAISVNEFLGNSWRHVPANSTESLGVLVLKARGVF
TEPHWYWLGVGALIGYVLLFNFLFTLALSYLNPFGKSQPILSKETLTEKQANRTEELIELSPETGARIQSGSSRSL
SARVGSITEADQSRKRGMVLPFEPLSISFDEIRYAVDMPQEMKAQGITEDRLELLRGVSGSFRPGILTALMGVTG
AGKTTLMDVLAGRKTSGYIEGIIKVYGYPKKQETFARVLGYCEQTDIHSPHVTVYESLLYSAWLRLPSEVDSAT
RKMFIEEVMELVELNSLREALVGLPSENGLSTEQRKRLTIAVELVANPSIIFMDEPTSGLDARAAAIVMRTVRNT
VDTGRTVVCTIHQPSIDIFDAFDELLLLKRGGEEIYAGPIGRHSSHLIKYFEGINGVSKIKDGYNPSTWMLEVTSA
AQEVALGVNFTEEYKNSELYRRNKALIKELSSPPPGSKDLYFSTQYSQSFFTQCLACLWKQHWSYWRNPAYTA
VRLFFTTFIALMLGTIFWDFGSKRKRQQDLFNAMGSMYAAVISIGIQNASSVQAVVAIERTVFYRERAAGMYSP
FPYAFGQVMIELPHIFIQTIIYGLIVYAMVGFEWTVTKFFWYLFFMYFTFLYFTFYGMMAVAITPNQHISGIVSS
AFYGLWNLFSGFIIPHTRIPVWWKWYFWSCPVSWTLYGLLVTQFGDIKERLESGERVEDFVRSYFGYRNDFVG
VVAGIVVGITVLFGFIFAYSIRAFNFQKR

>XP_010654722.1_P_PDR1_Vv
MATADTYRASGSLRRNGSSIWRSSGADVFSRSSRDEDDEEALKWAALEKLPTYNRLRRGLLMGSEGEASEIDI
HNLGFQEKKNLVERLVKVAEEDNEKFLLKLKNRIDRVGIDVPEIEVRFEHLTIDAEAFVGSRALPSFHNFIFNKL
EGILNAVRILPSKKRKFTILNDVSGIIKPRRLTLLLGPPSSGKTTLLLALAGKLDPNLKVMGRVTYNGHGMNEFV
PQRTAAYISQHDTHIGEMTVRETLAFSARCQGVGDRYDMLAELSRREKAANIKPDPDLDVFMKAAATEGQKE
NVVTDYTLKILGLDICADTMVGDEMIRGISGGQRKRVTTGEMLVGPSKALFMDEISTGLDSSTTFQIINSLKQTI
HILNGTAVISLLQPAPETYNLFDDIILLSDSQIVYQGPREDVLEFFESIGFKCPERKGEADFLQEVTSRKDQAQYW
ARKDVPYSFVTVKEFAEAFQSFHIGRKVADELASPFDRAKSHPAALTTKKYGVRKKELLDANMSREYLLMKR
NSFVYIFKLTQLAVVAVIAMTLFLRTEMNKNSTEDGSIYTGALFFTVVMIMFNGMAELAMTIAKLPVFYKQRD
FLFYPAWAYALPTWVLKIPITFVEVAVWVFITYYVIGFDPNVERLFRQYLLLLLVNQMASGLFRFIAAAGRNMI
VASTFGAFAVLMLMALGGFILSHDNVKKWWIWGYWSSPLMYAQNAIVVNEFLGKSWSKNVTNSTESLGITV
LKSRGFFTDAHWYWIGAGALLGFIFVFNFFYTLCLNYLNPFEKPQAVITEESDNAKTATTERGEHMVEAIAEGN
HNKKKGMVLPFQPHSITFDDIRYSVDMPEEMKSQGALEDRLELLKGVSGAFRPGVLTALMGVSGAGKTTLMD
VLAGRKTGGYIEGNISISGYPKKQETFARISGYCEQNDIHSPHVTVHESLLYSAWLRLPSDVNSETRKMFIEEVM
ELVELTPLRDALVGLPGVNGLSTEQRKRLTIAVELVANPSIIFMDEPTSGLDARAAAIVMRTVRNTVDTGRTVV
CTIHQPSIDIFEAFDELLLMKRGGQEIYVGPLGRHSSHLINYFEGIEGVSKIKDGYNPATWMLEVTTGAQEGTLG
VDFTEIYKNSDLYRRNKDLIKELSQPAPGTKDLYFATQYSQPFFTQFLACLWKQRWSYWRNPPYTAVRFLFTT
FIALMFGLIFWDLGTRRTRQQDLLNAMGSMYAAVLFLGVQNAQSVQPVIVVERTVFYRERAAGMYSALPYAF
GQALVEIPYVFAQAVVYGVIVYGMIGFEWTATKFFWYLFFMFCTLLYFTFYGMMAVAATPNQHIASIIAATFY
TLWNLFSGFIVPRNRIPVWWRWYCWICPVAWTLYGLVASQFGDIQSTLLENNQTVKQFLDDYFGFKHDFLGV
VAAVVVGFVVLFLFIFAYAIKAFNFQKR

>XP_011019747.1_P_PDR1-l_Pe
MDGAGDIYRVSSARLSTSTNIWRNSIPEVFSRSSRDEDDEEALKWAALEKLPTYLRLTRGILTEEEGKAREIDIIN
LGLVEKRDLLERLVKIAEEDNERFLLKLKERIDRVELEMPTIEVRFEHLNVEAEAYVGGRALPTILNFSANMLE
GFLSFLHLLPSRKQTFPILRDVSGIIKPRRMTLLLGPPSSGKTTLLMALAGKLGKDLQCSGSVTYNGHGMEEFVP
QRTSAYISQFDLHIGEMTVRETLSFSARCQGVGPRYEMLTELSRREKEANIKPDPDLDIYMKAAALEGQETSVT
TYYILKITGLDICADTMVGDEMIRGISGGQKKRLTTGEMLVGPARALFMDEISTGLDSSTTFQIVNSLRQTTHIL
NGTTLISLLQPAPETFALFDDVILLSDGLIVYHGPRENVLEFFESLGFKCPERKGVADFLQEVTSRKDQEQYWAS
RDQPYSFVSANEFSEAFQSFHIGRKLGDELAIPFDKSKSHPSALSTEKYGVSKKELLKACISREFLLMKRNSFVYI
FKFTQLILLASITMTIFLRTEMHRNTITDGGIYIGALFFAIIIIMFNGFSELVMTIMKLPVFYKQRDLLFYPSWAYAI
PTWILKIPITFVEVAIWTTMTYYAVGFDPNIGRFFKQYLIFVLANQMSSGLFRLMGALGRNVIVANNVGSFALL
AVLVMGGFILSRDNVKSWWIWGYWVSPLMYVQNAVSVNEFLGNSWRHIPPNSTESLGVTLLKSRGVFPEAR
WYWIGVGALIGYTLLFNFLFTLALKYLNPFGKPQAILSKEALAERDANRTGNFIELSTRGKSSSERGKDSKRNSS
ARVPSLRIPSPGDANQNKRGMILPFQPLSITFEEIRYSVDMPQEMKAQGIPEDRLELLKGVSGAFRPGVLTALMG
VSGAGKTTLMDVLSGRKTGGYIDGRISISGYPKNQQTFARISGYCEQTDIHSPHVTVYESLVYSAWLRLSPDVD
SETRKMFIEEVMELVELNPLRESLVGSPGVDGLSTEQRKRLTIAVELVANPSIIFMDEPTSGLDARAAAIVMRAV
RNTVDTGRTVVCTIHQPSIDIFDAFDELFLLKRGGEEIYVGPVGRHACHLIKYFEEIEGVPKIKDGYNPATWMLE
VTSATQEAVLKDNFTDIFKNSEPYRRNKALIKELSAPQPGSKDLYFPTRYSQSFFAQCMACLWKQHWSYWRNP
PYNAVRLLFTTFIAVMFGTIFWNLGSKRNRKQDIFNSMGSMYAAVLFIGIQNATSVQPVVAIERTVFYRERAAG
MYSALPYAFAQVMIEIPYTLVQALIYGVIVYSMIGFEWTPIKFFWYIFFMYFTLLYMTFYGMMNVAITPNHNIA
SLVSSAFYAIWNLFSGFIIPRTRVPIWWRWYCWACPFSWTLYGLIASQYGDLEDTVEGDETVKDFVRNYFGFR
HDFVGICAIVVVGMSVLFAFTFAFSIKAFNFQRR

>XP_011034492.1_P_PDR1-l_Pe
MESADIYRASSSLRSSSRAGSSAWRNTTVEAFSRSSREEDDEEALKWAAIEKLPTYDRLRKGILTSASKGVANE
VDIEKLGVQERKQLLERLVKVAEEDNEKFLWKLKDRVERVGIDVPTIEVRYDNLNIEAEAYVGSGALPSFAKF
TFNIIEGLLISLNILRNRKKPFTILKDVSGIVKPSRLTLLLGPPSSGKTTLLLALAGKLDPNLKFSGRVTYNGHEM
NEFVPQRTAAYISQHDLHIGEMTVRETLAFSARCQGVGYLHDMLAELSRREKEANIKPDPDVDVFMKAVASQ
GDEANVITDYVIKILGLEICADTMVGDEMIRGISGGQRKRVTTGEMLVGPSRALFMDEITSGLDSSTTYQIVNSL
RHTVHILNCTAVISLLQPAPETYDLFDDIILLSDGQIVYQGPRERVLDFFEHMGFECPERKGVADFLQEVTSRKD
QEQYWARKDQPYRFITANEFAEAFQSFNVGRRTAEELSIPFDKSKNHPAALVTKTHGAGKKDLLKANFSREYL
LMKRNSFVYIFKISQLTIMALISMTLFFRTEMHRDTVTDGGIYTGALFFTAIMIMFNGMSELSMTIAKLPVFYKQ
RDLRFFPSWAYAIPQWILKIPVAFVEVGAWVFLTYYVIGFDPNVERLFKQYFLLLLINQMASALFRFIAAAGRN

MIVANTFGSFALLTLFALGGFILSREKIKKWWIWGYWISPLMYGQTAIVVNEFLGNSWSHVPENSTEPLGIQVL
KSRGFFTEAYWYWIGVGATIGFILLLNLFFVLALTFLNAFDKPQAVISEEPESDESGRKTERAIQLSNHGSSHGT
NTEGGVGISRASSEAIGGVSNNRKKGMVLPFEPHSITFVDVIYSVDMPQEMKVQGVVEDRLVLLNGVNGAFRP
GVLTALMGVSGAGKTTLMDVLAGRKTGGYIEGEIKISGYPKKQESFARVSGYCEQNDIHSPQVTVYESLLYSA
WLRLPPEVDSETRRMFIEEVMDLVELNPLRHALVGLPGVNGLSTEQRKRLTIAVELVANPSIIFMDEPTSGLDA
RAAAIVMRTVRNTVDTGRTVVCTIHQPSIDIFEAFDELFLMKRGGQEIYVGPLGRHSTHLIKYFEAIEGVSKIKD
GYNPATWMLEISSSAQEMALEVDFSNIYKNSDLFRRNKALIAELSTPAPGSTDLYFPTKYSTSFLTQCMACLWK
QHWSYWRNPPYTAVRFLFTTFIALMFGTMFWDLGSKVNSTQDLLNAMGSMYAAVLFLGVQNASSVQPVVAV
ERTVFYRERAAGMYSALPYAFAQVVIELPYIFVQAAVYGIIVYAMIGFEWTVVKFFWYLFFMYFTLLYFTFYG
MMAVAMTPNHHIAAIVSSAFYGIWNLFSGFIVPRPSIPIWWRWYYWACPVSWTLYGLVVSQFGDIQKELSETQ
TVEEYVKDYFGFDHDFVGVVAAAVVGWTVLFAFIFAFAIKAFNFQRR

>XP_011081908.1_PDR1-l_X2_Si
MMEGGDIFRVSSARLSSSNIWRNTGMEAFSRSSREADDEEALKWAALEKLPTNLRIRRGIFTEEEGKTREIDIKN
LGLVERKNLVERLLRIAEEGNEKFLLKLKERIQRVGLDFPTIEVRYEHLNVDAEAYVGSRALPTVFNFTVNILEG
FLSYLHVLPSRKKPLPILHDVSGIIKPGRMTLLLGPPCSGKTTLLLALAGKLDPELKVSGRVTYNGHEMEEFVPQ
RTSAYISQNDLHIGELTVRETLAFSARCQGVGARYEMLAELSRREKEANIKPDPDLDIFMKAASIEGQDASVVT
DYIIKILGLEVCADTLVGDEMVRGISGGQRKRLTTGEMLVGPARALFMDEISTGLDSSTTFQIVNSIRQSIHILQG
TAVISLLQPAPETYNLFDDIILLSDGEIVYQGPREHVLEFFEFMGFRCPERKGVADFLQEVTSKKDQEQYRIHKD
EPYSYVSSKEFSEAFQSFHVGRKLGDELAVPFDKEKSHRDALTTVKYGVSKKELLKACVAREFLLMKRNSFVY
IFKLIQLILMGSISMTVFLRTEMPKNTVTDGGIYMGALFYALIMIMFNGFSELALSILKLPVFYKQRDLLFFPPWA
YSLPAWILKIPITLVEVAIWVGLTYYPTGYDSDAGRFFKQLLLLIVINQMAGGLFRLVGALGRNIIVANTFGSCA
LLTVLVLGGFILSRDNIKSWWIWGYWFSPLMYGQNAIAVNEFLGKSWSHIPPGATEPVGVSILKARGIFPEARW
YWIGLGAIIGYAFLFNFLVTLALTYLNPLGKPQAILSEETLAERTASKRIEPIELSSRGKRTSGREDDVQRSVSAR
SMSSRVGSISNADPSRKRGMILPFEPLSMTFDDIHYAVDMPQEMKAHGIQEERLELLKGVSGAFRPGVLTALM
GISGAGKTTLMDVLSGRKTGGYIDGRITISGYPKKQETFARIAGYCEQTDIHSPHVTVYESLQFSAWLRLPPEVD
AATRTMFIEEVMELVELTPLRDALVGLPGVDGLSTEQRKRLTIAVELVANPSIIFMDEPTSGLDARAAAIVMRT
VRNTVDTGRTVVCTIHQPSIDIFDAFDELLLLKRGGEEIYVGPLGRHSSQLIKFFEGIDGISKIKDGYNPATWMLE
VTSEAQEAALGVNFAEVYRNSELYRRNKALIKELSTPAPGSKDLYFQTHYSQSFWTQCMACLWKQHWSYWR
NPPYTAVRLLFTTFIAIMFGTIFWDLGSKRKKQQDIFNAMGSMYAAVLFLGVQNATSVQPVVAIERTVFYRER
AAGMYSALPYAFGQAVVELPNLLIQTLVYGVIVYAMIGFEWTVAKFLWYLFFMYFTLLYFTLYGMMTVAVTP
NHNIAAIVSSAFYALWNLFSGFIIPKTRIPVWWRWYYYVCPISWTLYGLVASQFGDIKAQLDDNDQTVEEFVR
NYFGFRHDFVGYVAVIIAGIAVLFAFIFAFSIRVFNFQKR

>XP_011459223.1_P_PDR1-l_Fv
MESSSGDVYRVSSARLSSSNIWRNSTMDVFSKSSHDEEDEEALKWAAIEKLPTYLRIRRGIFTEEEGEAREIDIK
NLGLLERKNVLERLVKVADEDNERFLMKLKNRIDRVGLEFPTIEVRFEHLSVEAEAYVGGRALPTIFNFSANIL
EGFLSFCRIIPTRKHPFPILDDVSGIIKPKRMTLLLGPPSSGKTTLLLALAGKLAKDLKSSGRVVYNGHGMEEFVP
ERTSAYISQYDLHIGEMTVRETLAFSARCQGVGGRYEMLAELSRREKEANIMPEKDLDIYMKAASLEGQEASV
VTDYILKILGLEVCADTMVGDEMFRGISGGQKKRVTTGEMLVGPARALFMDEISTGLDSSTTFQIVNSLRQSIHI
LNGTALISLLQPAPETYDLFDDIILLSDGQIVYQGPRENVLEFFEYMGFKCPERKGVADFLQEVTSKKDQEQYW
FQKEEPYNFISSKEFSEAFQSFHIGRKLGDELATPFDKSKGHPAALTTKKYGVSKKELLKACIAREYLLMKRNSF
VYIFKMTQLTLLAFISMTLFFRTEMPRHTVEDGGIFMGAMFFTIIIIMFNGFSELALTIMKLPVFFKQRDLLFYPA
WAYSLPTWILKIPITFVEVAIWVFMTYYVIGFDPNPGRFFKQYLLLLFVNQMASGLFRFMGALGRNIIVANTFG
SFALLAVLVMGGFILSRDNVQAWWLWGYWVSPMMYSQNAIAVNEFLGDSWSHVPPNSNESLGIMVLKSRGV
FVEAHWYWIGVGATIGYIFLFNFFYTVALQYLDPFGKPQAVLSKEALAEKTAATAGGNLELSSRGNNSSEAKN
DSRRSVSSRTQSARVGSFNEANENRKKGMVLPFEPLWITFDEIKYSVDMPQEMKTQGITEERLPLLKGVTGAFR
PGVLTALMGISGAGKTTLMDVLAGRKTGGYIEGDITISGYPKKQETFARISGYCEQTDIHSPHVTVYESLVYSA
WLRLPPEVDSATRKMFVEEVMELVELTSIRESLVGLPGVNGLSTEQRKRLTIAVELVANPSIIFMDEPTSGLDAR
AAAIVMRTVRNTVDTGRTVVCTIHQPSIDIFDAFDELFLLKRGGEEIYVGPLGRHSSQLIKYFEEIEGVPKIKDGY
NPATWMLEVTSAAQEVVLGVNFTDIYRSSELYRRNKDLIKELSTPPQGTQDLYFSTKYSQSFYYQCMACLWK
QHLSYWRNPPYSAVRLLFTTFIALMFGTIFWDLGSKRKNQQDVFNAMGSMYAAVLFIGVQNATSVQPVVAVE
RTVFYRERAAGMYSALPYAFGQVVIELPYIFVQTVIYGVIVYAMIGFDWTVSKFLWYLFFMYFTFLYFTFYGM
MTVAVTPNHNIAAIVSSAFYAIWNLFSGFIIPRTRMPIWWRWYYWICPVSYTLYGLVASQFGDIKEVMESGESV

EDFVRNYFGYRHDLVGIIAVVLVGISVLFGFTFAFSIKAFNFQKR

>XP_012073069.1_PDR1_Jc
MEGTDIHRASSSIRGMSSSAWRNTGVDVFSRSSREDDDEEALKWAALEKLPTYDRLRKGILVSVSKGGVNEID
VGDLGFQERKSLLERLVRVAEEDNEKFLLKLKNRIDRVGIEIPTIEVRFERLNIEGEAFVGNRALPTFVNFSVNIV
EGFLNSLHILPGRKKRFTILKDVSGIIKPSRMTLLLGPPSSGKTTLLLALAGKLDPNLKFSGSVTYNGHGMNEFIP
QRTAAYISQHDLHIGEMTVRETLAFSARCQGVGTQQEMLAELSRREKAANIKPDPDIDFFMKAAAIEGQETSV
VTDYILKILGLEICADTLVGAEMLRGISGGQRKRVTTGEMLVGPARALFMDEISTGLDSSTTYQIVNSLKQSINIF
NGTAVISLLQPATETYDLFDDIILLSDGQIVYQGPRDDVLGFFEYMGFKCPERKGVADFLQEVTSKKDQQQYW
ARKDHPYSFVTVPEFSRAFQSYEIGRKIEHDLSTPFDKTKSHPAALATGKYGAGKMELLKSCFSREYLLMKRNS
FVYIFKLSQLTIMAIITMTVFLRTNMHRDDVTDGGIYLGALFFTMVAVMFNGMSEISLTIVKLPVFYKQRDLLF
YPAWAYSLPTWIIKIPITILEVSVWVFITYYVIGFDPNVGRLFKQYLLLLLVNQMSSALFRLIASVGRNMIVANTF
GSFALLALFALGGVILSREDIKKWWIWGYWISPMMYGQNAIIVNEFLGKSWSHIPENSGEPLGIQVMKTRGFFP
QAYWYWLGLGALAGFILLFNLCFTLALHFLNPFEKPQAVISEVPEPRDRIEGATAIQLSQAECSHRTKTEIGTSG
NDEVNYNKKKGMVLPFEPHCLTFDNVVYSVDMPQEMKSQGVLEDKLVLLKGVSGAFRPGVLTALMGVSGA
GKTTLMDVLAGRKTGGYIEGSIPISGYPKQQETCARISGYCEQKDIHSPHVTVYESLLFSAWLRLPPEIDSGTRD
MFVDEIMELVELNPLRHALVGLPGVNGLSTEQRKRLTIAVELVANPSIIFMDEPTSGLDARAAAIVMRTVRNTV
DTGRTVVCTIHQPSIDIFEAFDELFLMKRGGEEIYVGPLGGHSYHLIKYFEEIEGVSKIKDGYNPATWMLEVTSS
AQEMALGVDFSVIYKNSELYRRNKAIIKELSVPAPGSKDLYFPAQYSQSFLTQCIACLWKQRLSYWRNPPYTA
VRFLFTTFMALMFGTIFWNLGSKIGKQQDLINSTGSLYAAVLFLGVQNAASVQPVVAIERTVFYRERAAGMYS
ALPYAYAQVIIELPYIFCQAIVYGLITYAMIGFEWTSAKFFWYIFFMYFTLLYFTYYGMMTVAVSPNHHIASIISS
AFYAIWNLFSGFVIPRTRMPVWWRWYYWACPLSWTLYGLIASQYGDIKDPLEDGARQPVQDYVREYYGVKH
DFLGAVAAVIPGIAVLFAFIFAVSIRSFNFQRR

>XP_012073071.1_PDR1_Jc
MEGGDIYRASSSLRRKGSFSMWRNNNPNDVFSRSSRDEDDEEALKWAALEKLPTYDRLRKGILLSASKGVASE
IDVDNLGFQERKLLLERLIKVAEEDNEKFLLKLKNRIDRVGIEIPTIEVRFEHLNVEAEAYSASRALPSFVNFTFGI
LEGFLNYLHIIPSRTRPLHILKDVSGIIKPSRMALLLGPPSSGKTTLLLALAGKLDPNLKFSGTVTYNGHGMNEFI
PQRTAAYISQHDEHIGEMTVRETLAFSARCQGVGTQHDLLAELSRREKAANIKPDPDIDVFMKAAATEGQETS
VITDYFLKILGLEVCADTLVGDEMIRGISGGQRKRVTTGEMLVGPAKALFMDEISTGLDSSTTYQIVNSLRQSIH
ILNGTALISLLQPAPETYDLFDDIILLSDGQIVYQGPREQVLGFFEYMGFKCPERKGVADFLQEVTSKKDQQQY
WARRDQPYRFITAQEFSEAFQSYEVGRKIGDELSTPFDTTKSHPAALTTKRYGVGKMELLKACISREYLLMKR
NSFVYIFKLTQLIIMALVTMTLFLRTKMHRDTVDDGGIYLGALFFATILIMFNGMSELAMTIAKLPVFYKQRNL
LFYPAWAYSLPTWILKIPITFVEVAVWIVLTYYVIGFDPNVGRFFKQYLVLIIVNQMASGLFRFIAATGRNMIVA
NTFGSFALLALFALGGFILSKDDIKKWWIWGYWVSPLMYGQNAIVVNEFLGHSWSHIPAGTTKPLGIQLLEAR
GFFPHAYWYWLGFGACVGFMLLFNFLFTVALTYLDPFEKPQAVISEDSETNESGNQTRGSIQLTNYGSSHKSSI
DGADENSEVNHIKKKGMVLPFAPHSITFDDIIYSVDMPQEMKSQGVLEDKLVLLKGVSGAFRPGVLTALMGVS
GAGKTTLMDVLAGRKTGGYIEGSITISGYPKKQETFARISGYCEQNDIHSPHVTVHESIVYSAWLRLPPEVDKET
RTMFVEEVMDLVELNPIRNALVGLPGVNGLSTEQRKRLTIAVELVANPSIIFMDEPTSGLDARAAAIVMRTVRN
TVDTGRTVVCTIHQPSIDIFEAFDELFLMKRGGEEIYVGPLGRHSCHLIKYFEGIEGVRKITDGYNPATWMLEVT
GPAQELAMDVDFASIYRNSEVYRRNKAMIKELSVPAPGSKDLYFPTQYATSFLTQCIACFWKQRWSYWRNPP
YTAVRFLFTTFIALMFGTLFWNLGSKSTKQQDLFNAMGSLYAATLFLGVQNSGAVQPVVAIERTVFYRERAAG
MYSAMPYALAQVMIEIPYIFCQAIVYGLITYAMIGFHWTVAKFLWYMYFMYLTLLYFTFYGMMAVAVTPNH
HIAAIVSSAFYGLWNLFAGFIVPRTRIPVWWRWYYWANPIAWTLYGIIGSQFGDIKDILDGDQTVGEFVREYYG
YKHDFVPVVAAVVLALTVGFAFIFGLSIKAFNFQRR

>XP_012073073.1_PDR1_Jc
MDGTDIYRASSSIRGGGSSARRNNGVDVFSRSSREEDDEEALKWAALEKLPTYDRLRKGILVSVSKGEMHEIDI
DDLGFQERKTLVDRLVRVAEEDNEKFLLKLKNRIDRVGIEIPTIEVRFEHLNIEAEVHVGSRALPTFVNFSANIV
EGFLNSLHILPSRKKQLTILKDVSGVIKPSRMTLLLGPPSSGKTTLLLALAGKLDPNLKFSGSVTYNGHGMNEFI
PQRTAAYISQHDLHIGEMTVRETLAFSARCQGVGTQHDMLAELSRREKIANIKPDPDIDVFMKAAATEGQDTN
VVTDYILKILGLEICADTLVGDEMLRGISGGQRKRVTTGEMLVGPAKALFMDEISTGLDSSTTYQIVNSLKQSIH
ILNGTAVVSLLQPAPETYDLFDDIILLSDGQIVYQGPRDDVLGFFEYMGFGCPERKGVADFLQEVTSRKDQQQY
WTCKDQPYSFVTVPEFTRAFQSYEIGQKIEHDLSIPFDKTRSHPAALATRKYGVGKIELLKACFSREYLLMKRN

SFVYIFKIGQLTVMAIITMTLFLRTNMHRDDVTDGGIYLGALFFTVVMVLFNGMSEISMTIVKLPVFYKQRDFL
FYPSWAYSLPIWILKIPITFLEIAVWVFITYYAIGFDPNVGRLFKQYLLLLLFTQMSSALFRFIASVGRNMIVANTF
GSFALLTLVALGGVILSREDVKKWWIWGYWMSPVMYGQNAVVVNEFLGKSWSHIPAKSEEPLGIQVMKSRG
FFPEAHWYWLGLGASAGFILVFNICSTLALHFLNPFEKPQAFISEESAPRDKTEGAIQLSQAESSDRTKTEIEASG
NDEVNHNKKKGMVLQIEPHCLTFDNVVYSVDMPQEMKSQGVLEDKLVLLKGVSGAFRPGVLTALMGVSGA
GKTTLMDVLAGRKTGGYIEGSITISGYPKKQETFARISGYCEQNDIHAPHVTVYESLLFSAWLRLAPEIDSSTRD
MFVDEIMELVELNPLRHALVGLPGVNGLSTEQRKRLTIAVELVANPSIIFMDEPTSGLDARAAAIVMRTVRNTV
DTGRTVVCTIHQPSIDIFEAFDELFLLKRGGEEIYVGPLGRHSYHLIKYFEEVEGVSKIKDGYNPATWMLEVTSS
AQELALGVDFSAIYKNSELYRRNIAVINELSTPAPGSKDLYFSTQYSQSFLTQCIACLWKQRLSYWRNPPYTAIR
FLFTIFIALMFGTIFWNLGSKTGKQQDLSNSTGSLYAAVLFLGVQNGSSVQPVVAVERTVFYRERAAGMYSAL
PYAYAQVIVELPYIFCQAFVYGLITYAMIGFEWTAAKFFWYIFFMYFTLLYFTYYGMMAVAVTPNYHIASIVSS
AFYAIWNIFSGFVIPRPRMPVWWRWYYWGCPVAWTLYGLLASQYGDIKDPLEDGDQQPVQDFLREYYGIKH
DFLGKVAVVIPGIAVLFAFIFAVSIRSFNFQRR

>XP_012076643.1_PDR1_Jc
MESGDITRVSSARLSSAKVSSARLSSSNIWRNTTLDVFSKSSCNDDDEEALKWAALEKLPTHLRVRRGILTEEE
GQSREIDINSLGLIEKRNLLERLVKIAEQDNEKFLLKLKERIIKVGLEMPTIEVRFEHLSVDAQAYIGSRGLPTMF
NFSANMFEGFLNYLHIFPSRKKPLSILQDVSGIIKPQRMTLLLGPPSSGKTTLLLALAGKLSKELKISGNVTYNGH
GMDEFVPQRTSAYISQYDLHIGEMTVRETLAFSARCQGVGTRYEMLTELARREKEANIKPDPDIDIYMKAAAL
EGQETNVVTDYILKILGLEVCADIMVGDEMIRGISGGQKKRVTTGEMLVGPARALFMDEISTGLDSSTTFQMV
NSLRQSIHILNGTAVISLLQPAPETYDLFDDIILLSDGQIVYQGPRENVLEFFEYIGFRCPERKGVADFLQEVTSRK
DQEQYWARRDEPYSFVSVKEFAEAFQSFHIGRKLGDELATPFDKSKSHLAALTTKKHGASKKELLKACISREY
LLMKRNSFVYIFNMSQLIILAFITMTVFLRTEMHRNTETDGGIYLGALFFTVITTMFNGFSELAMTIMKLPVFYK
QRDILFYPSWAYALPSWILKIPIAFVEVAVWVVMTYYVIGFDPNIGRFFKQYLILMITSQMASALFRLTAALGR
NIIVANTIGCFALLAVLVLGGFVISRDNVKKWWIWGYWFSPMMYVQNGISVNEFLGKSWRHLAHNSTEPLGIT
VLRSRGLFPEAYWYWIAVGALTGYIFLFNFLFILALKYLNPFGRSQPILSEDVYAQRNATRTGQVIELSSKGKSF
QEKGSVSERNASSRTQSIRVSIFNDDHQKRKRGMVLPFQPLFITFDEIRYAVDMPQVMKAEGITEDRLELLKGV
NGAFRPGVLTALMGVSGAGKTTLMDVLAGRKTGGYIEGNITISGYPKNQETFARISGYCEQTDIHSPHVTVYES
LLYSAWLRLPSKVNSSTREMFIEEVMELVELNTIREALVGLPGVNGLSIEQRKRLTIAVELVANPSIIFMDEPTSG
LDARAAAIVMRTVRNTVDTGRTVVCTIHQPSIDIFDAFDELFLLKRGGEEIYVGPVGRHACHLIKYFEDIEGTPK
IKDGYNPATWMLEVTTAAQEVALGVNFSDIYKNSELYRRNKALIKELSKPPPGSEDLSFPTQYSQSFLTQCMAC
LWKQHWSYWRNPSYSAVRLLFTTFIALMFGTIFWNLGSKRGSRQDILNAMGSMYAAVLFLGFQNSTSVQPVV
AIERTVFYRERAAGMYSALPYAFGQVMIELPYILVQTITYGVIVYAMVGFEWSVSKFFWYIFFMYFTLLYFTFY
GMMSVAITPNPHIAAIISSAFYAIWNIFSGFIVPRPRIPVWWRWYYWACPVAWTLYGLVASQYGDIKEPLDSGE
TVQDFLRSYFGFHHDFLGIVALVLVGIPVLFGFIFAFSIKAFNFQIR

>XP_012076649.2_PDR1_Jc
MKKLLANVSTNEGTEERKVQTLVKMEGAGDRVSSERISSFSIWRNSTMEAFSKSSRHEEDDEEALKWAALEKL
PTYLRVRRGILTGQEGQSREIDVKKLGFIEKRNILERLVKIAEEDNENFLLKLKERIDKVGLDMPTIEVRFEHLRI
EAEAYVGSRALPTMFNFSVNLFEGFLNYLHILPSRKISLPILQDVSGIIKPRRMTLLLGPPSSGKTTLLLALAGKL
SKDLKFSGRVTYNGHGMEEFVPQRTSAYISQYDLHIGEMTVRETLSFSARCQGVGTRYEMLAELARREKAANI
KPDPDIDIYMKAAALEGQDRNLVTDYILKILGLEVCADTMVGDEMVRGISGGQKKRVTTGEMLVGPARALFM
DEISTGLDSSTTFQIVNSLRQSIHILNGTALISLLQPAPETYDLFDDIILLSDGQVVYQGPRENVLEFFEYMGFKCP
ERKGVADFLQEVTSRKDQEQYWAHKDEPYSFVPANEFAEAFQSFHIGRKLGDELATPFDKSKSHPASLTTKKY
GVSKKELLKACISREFLLMKRNSFAYIFKMLQLILMASVTMTIFVRTEMHRNTVEDGQVYFGALFFAVMTIFFN
GLSEIPMTVIKLPVFYKQRDLLFYPSWSYALPTWILKIPISFLEVAIWVILTYYVIGFDPNIERFFKQYLILLLTNQ
MASSLFRLVAALGRNMILANTLAIFSLLAVLVLGGFVLSRDDVKKLWIWGYWFSPLMYAQNGISVNEFLGKR
WNHFPPNSTEALGVTFLKSHGLFPEAYWYWIAAGGLTGYVFLFNFLFTLALKYLDPFKKPQAILSEEAFAEKN
ANRTGEFGIELSARRKNPLGRWNSSHRSSSFRMPSARVGSSSGADKDRKRGMVLPFQPLSITFDEIRYAVDMPR
EMKAQGITEDRLELLKGVSGAFRPGVLTALMGASGAGKTTLMDVLAGRKTGGYIEGNITISGYPKKQETFARI
SGYCEQTDIHSPHVTVYESVIYSAWLRLPTEVNSNTRKMYVEEVMELVELTSLKEALVGLPGVSGLSIEQRKRL
TIAVELVSNPSIIFMDEPTSGLDARAAAIVMRTVRNTVDTGRTVVCTIHQPSIDIFDAFDELILLRRGGQEIYVGP
VGRQACHLIRYFEEIEGVPKIKDGYNPATWMLEVTTAAQEEALGVNFTDIYKNSELYRRNKALINELSKPPPGS
KDLCFPTQYSQPFLTQCMACLWKQHWSLWRNPPYSAVRLLFTTFIALMMGTIFWDIGSKRSRQQDILNAMGS

MYIAVLFLGFLNASSVQPVAAIERTVFYRERAAGMYSALPYAFGQVVIELPHILVQTIIYGVIVYAMIGFEWTA
NKFFWYIFYMYFTFLYFTLYGMMVVATTPNHSIAAIVSTAFYAIWNLFSGFVVPRTRIPVWWRWYYWCCPIA
WTLYGLIASQYGDVKETLDTGETVEHFLRSYFGFRHDFIGIVAIVLVGITTLFGFIFAFSIKIFNFQKR

>XP_012457161.1_P_PDR1-l_Gr
MQGADIYRASGSLRRSPSAWRNNDLEVFSRSSREEDDEEALKWAALEKLPTVSRLRKGILTSSEGGASEINIHD
LGWVERKALLERLVKVAEEDNEKFLLKLKNRVARVGIDFPTIEVRFEHLNIEAQAFVGTNALPTILNFITSLFEG
FLIDMGILSSRKKKLTILNDVSGIIKPSRLTLLLGPPSSGKTTLLLALAGKLDPALKCSGKVTYNGHGLDEFVPQR
TVAYISQHDLHIGEMTVRETLAFSARCQGVGDRYELLAELSRREKQANIKPDPDMDVYMKAVATEGQEANVI
TDYVLKVLGLEVCADIMVGDEMLRGISGGQRKRVTTGEMLVGPAKVLFMDEISTGLDSSTTFQIVNSLKQTVH
ILNGTAIISLLQPAPETYNLFDDIILLSDGQIVYQGPREHVLSFFESMGFKCPERKGVADFLQEVTSRKDQQQYW
VHKDQPYRFVTANEFSEAFQSFHVGKELRDELGVPYEKTKSHPAALTTKKYGVGRKELLKACISREYLLMKR
NSFVYIFKFIQLTIMAFITMTLFLRTEMRNDSIEGGGIYMGALFFGLIAIMFNGMSEISMTIAKLPVFFKQRDLLFF
PSWAYALPTCILKIPISFLEVSLWVFLTYYVIGFDPNVERLFRQYLILVLVTQMSSGLFRFIAAAGRNMVVANTF
GAFALLILFALGGFVLSRDDIKKWWIWGYWISPMMYGQNALMVNEFLGNQWRRILPGSNEPLGITILKSRGFF
QDPRWYWFGVGGLLGFIVLFNFFYTMALAYLKPFEKIQAVISEELEEKEQAESSSEVQDDTKTSISSKSSSMDE
VTTGNKKKGMVLPFEPHSITFDDIIYSVDMPQEIKEQGVNEDKLVLLKGVSGSFRPGVLTALMGVSGAGKTTL
MDVLAGRKTSGYIEGNITVSGFPKKQETFARVSGYCEQNDIHSPHVTVYESLLYSAWLRLPEKVNAETRKMFI
DEVMDLVELNPLRQAQVGLPGVNGLSTEQRKRLTIAVELVANPSIIFMDEPTSGLDARAAAIVMRTVRNTVDT
GRTVVCTIHQPSIDIFEAFDELFLMKRGGQEIYVGPLGYHSKHLIDYFEGIQGVSKIKDGYNPATWMLEVSTTA
QELALGVDFADIYKKSELYKRNKTLIKDLSRPAPGSKELYFPTQFSQPFLTQCAACLWKQRWSYWRNPAYTAV
RLLFTTVIALMFGTLFWDLGTKTKKRQDLANAMGSMYAAVLFLGIQNAASVQPVVAVERTVFYREKAAGMY
SAMPYAIAQVLIEIPYIFVQAVVYGLIVYVMIGFEWTAAKFLWYLFFMYFTLLYFTFYGMMAVAVTPNHHIAGI
VSSAFYGIWNVFSGFIIPRPRIPIWWRWYYYICPVSWTLYGLVVSQFGDIQDVLENGETVEQYLRNYLGFKHEFI
GIVAVIIIAFAILFGAIFTVSIRLFNFQIR

>XP_012465762.1_P_PDR1-l_Gr
MENVEAFRVGSARIGSSSIWRNNAMEAFSMSAREEDDEEDLKWAAIEKLPTYLRVRRGIFTEGEGQSREIDIKN
LGFIERRNLLERLVRIAEDDNERFLLKLKQRIDRVGLDMPTIEVRFEHLNVEAEAYVGSRALPTIFNFSANILEGL
LSYLHILPNRKKPLPILNDISGIIRPRRMTLLLGPPSSGKTTLLLSLAGKLGKDLKFAGRVTYNGHGMKEFVPQR
TSAYISQYDVHIGEMTVRETLAFSARCQGVGPRYEMLKELSRREKEANIKPDPDIDIYMKAAALEGQEANVVT
DYILKILGLEVCADTFVGDEMRRGISGGQKKRVTTGEMLVGPARALFMDEISTGLDTSTTFHIVNSLRQSIHILN
GTALISLLQPAPETYDLFDDIILLSDGHVVYQGPRENVLEFFEYMGFKCPERKGVADFLQEVTSRKDQEQYWA
RKDEPYSFISVKELAEAFQSFHIGQKLGDDLAVPFDKSKSHPAALTKDKYGVSKKELLKACVSREYLLMKRNL
FVYYVFKMIQLIFIGVITMTIFIRTEMHRDTITDGGIFMGALFFILIMIMFNGFAELAMTILKLPVFYKQRDLLFYPS
WAYSLPALILKTPISILEVTVWVFMSYYVIGFDPDVGSFFKNYLVLLCLSQMASGLFRLMGGLGRNIIVANTCG
SFALLTVLVMGGFILTRDDVKKWWKWGYWISPLMYAQNAIAVNEFLGKSWRHVPPNSTEPLGVLVMKSRGIF
PEPHWYWIGVGALIGYCFLFNFLFTLALKYLDPFGKPQAVISKETLAERIASKTGENIELSSRGRDSSERRTGASS
RSLSWKVTSVNEANQKRKRGMVLPFEPLSMSFDEIKYALDMPQEMKAQGISEERLELLKGVSGAFRPGVLTAL
MGVSGAGKTTLMDVLAGRKTGGYVEGTIKISGYPKKQETFARISGYCEQTDIHSPHVTVYESLVFSAWLRLPP
EVNSETRMMFIEEVMELVELSSLRDALVGLPGVNGLSTEQRKRLTIAVELVANPSIIFMDEPTSGLDARAAAIV
MRTVRNTVDTGRTVVCTIHQPSIDIFDAFDELLLLKRGGEEIYVGPLGRHSCHLIKYFEEINGIPKIKDGYNPAT
WMLEVTSGAQEEAIGVNFTNIYKNSELYRRNKALVKELSNPAPGSKDLYFQTRYSQSLLTQCIACLWKQYWS
YWRNPPYTAVRFLFTTFIALMFGTIFWDLGSKRTRRQDVFNSMGSMYAAVLFIGFQNAASVQPVVAVERTVFY
RERAAGMYSALPYAFGQVVIELPYILVQTVIYGIIVYAMIGFEWTSDKFFWYLFFMYFTFLYFTFYGMMTVAV
TPDHNIAGIISSAFFALWNLFSGFIIPRTRIPVWWRWYYWVCPISWSLYGLIASQYGDVQDKFGSGETVQHFVR
NYFDFREEFVGVVAIVVVGICVLFGFIFAFSIKAFNFQKR

>XP_012474406.1_P_PDR1-l_Gr
MESGTDAFGVSSARIGSSSICRNNVREAFSMSSHEEDDENALKWAAIQKLPTYLRVRRGILTEQDGQSREIDIKN
LGFVERRNLLERLVRIAEEDNERFLLKLKERIDRVGLDMPTIEVRFEHLNVEAEAYVGNRALPTMFNFSVNIVE
GLLSNLHILPSRKKPFPILNDVSGIIKPRRMTLLLGPPSSGKTTLLLALAGKLGKDLKFSGRVTYNGHGMEEFVP
QRTSAYISQYDLHIGEMTVRETLAFSARCQGVGPRYEMLAELSRREKEANIKPDPDIDIYMKAAALEGQEAGV
VTDYILKILGLEVCADTMVGDEMIRGISGGQKKRVTTGEMLVGPARALFMDEISTGLDSSTTFQIVNSLRQSIHI

LNGTALISLLQPAPETYQLFDDVILLSDGQIVYQGPRENVLDFFKYMGFKCPKRKGVADFLQEVTSKKDQEQY
WTRIDEPYSFISVKEFAEAFQSFHIGQKLGDDLAIPFDKSKSHPDALSKDKYGVPKKELLKACLSRELLLMKRN
LFVYVFKMFQLIFIGFITVTIFLRIEMHRDTITDGGIFMGALFFILVTVMFNGFAELTLTILKLPVFYKQRDLLFYP
SWAYSLPTWILKIPISILDATLWVLMSYYVIGFDPNVGRFFKQYLLLLCLSQMASALFRFMGGLGRNIIVANTC
GSFAMLAVLVMGGFVLTRDAVKKWWIWGYWISPLMYGQNAIAVNEFLGKSWRQVPPNSTEPLGVLILKSRGI
FPEARWYWIGVGALIGYCFLFNFLFTLALKYLDPFGKPQAVISKETLAEKIASKAREKVDLSSRGEGSSGGGNE
SQRSVSFRSLSAKVGSVNDANQSRKRGMVLPFEPLSMSFDEIRYAVDMPQEMKAQGISEDRLELLKGICGAFRP
GVLTALMGISGAGKTTLMDVLAGRKSGGYVKGTIKISGYPKKQETFARISGYCEQPDIHSPHVTVYESLLFSAW
LRLPPEVNPETKTMFIEEVMELVELTSLREALVGLPGVNGLSTEARKRLTIAVELVANPSIIFMDEPTSGLDARA
AAVVMRTVRNTVDTGRTVVCTIHQPSIDIFDAFDELLLLKRGGEEIYMGPLGHHSCYLIKYFEEINGIPKIKDGY
NPATWMLEITSAAQEEALGVNFADIYKNSELYRRNKALVKELSSPAPGSKDLYFQTRYSQSLLTQCMACLWK
QYWSYWRNPPYNAVRFLFTTVIGLLFGTIFWDIGSKRTREQDVFNSMGSMYAAVLFIGFQNCASVQPVVAVER
TVFYRERATGMYSALPYAFGQVVIELPYVLVQTAIYGVIVYAMIGFQWTAAKFFWYLFFMYFTFLYFTFYGM
MAVAVTPNHNIAAIVSSAFFAIWNLFSGFIIPRTRIPIWWRWYYWACPVSWTIYGLIASQYGDINEKFDSGETVE
HFVRNYFDFRNEFVEIVAMVVVGICVLFGSIFAVSIKAFNFQKR

>XP_012855955.1_P_PDR1-l_Egu
MEGGDIFRVSSARLGSSSIWRNTGMESFSRSEKDAEDDEEALKWAALEKLPTYLRIRRGMLVEDEGKTREVDI
KNLGLSERRNLVERLLKIAEEGNEKFLLKLKERIQRVGLDFPTIEVRFENLNVDADAYAGGRALPTIFNFTVNIL
EGFLSYLRILPNRKKPLPILHDVSGIIKPGRMTLLLGPPSSGKTTLLLALAGKLDPELKISGRVTYNGHEMNEFVP
QRTSAYISQHDLHIGELTVRETLAFSARCQGVGARYEMLTELSRREKEANIKPDPDLDIYMKASSIGGQEASVV
TDYIIKILGLEVCADTLVGDEMFRGISGGQRKRLTTGEMMVGPARALFMDEISTGLDSSTTFQIVNSIRQSIHILQ
GTTIISLLQPAPETYDLFDDIILLSDGQIVYQGPRENVLEFFEHMGFRCPERKGVADFLQEVTSKKDQEQYRIQK
DETYEYVSVQEFSEAFRSFRIGQELGDELAVPFDKEKSHPTALTTDKYGVSKKELLKACVAREYLLMKRNSFV
YIFKMLQLIIVASISLTLFLRTEMPKNNVTDGGIYLGALFFALIMIMFNGFSELALSIMKLPVFFKQRDLLFFPPW
AYTLPSWILKIPITLVEVAIWVGMTYYATGYDSDAGRFFRQLLLLICVNQMASALFRLMGALGRNLIVANTFGS
CALLTVFVLGGFILSRDDIKKWWIWGFWTSPLMYGQNAIAVNEFLGKSWSHVPAGSTESVGLTVLKARGLFSE
ARWYWIGVGAIIGYVFLFNFLVTVALTYLNPFGKPQAILPEEAVAERNAGRGDDVRRSSSSGSMSSRVGTVIGN
ADPNRKRGMILPFEPLSITFCDIRYAVDMPQEMKAQGIQENRLELLKGVSGAFRPGVLTALMGISGAGKTTLM
DVLAGRKTGGYINGTITISGYPKKQETFARIAGYCEQTDIHSPHVTVHESLQFSAWMRLPPEVDTATRKMFIEE
VMELVELTPLREALVGLPGVNGLSTEQRKRLTIAVELVANPSIIFMDEPTSGLDARAAAIVMRTVRNTVDTGRT
VVCTIHQPSIDIFDAFDELLLLKRGGEEIYVGPLGRHSSQLIKYFEEIDGISKIKDGYNPATWMLDITSTAQEAAL
GVDFAELYKNSELCRKNKALIEELTVPIPGSKDLYFSKRYSQSFSTQCMACLWKQHWSYWRNPPYTAVRLLFT
TFIALLFGTIFWGIGSKRERQQDVFNAMGSMYVAVLFLGIQNATSVQPVVAIERTVFYRERAAGMYSALPYAF
GQAVIELPYLLIQTLIYGVIVYAMIGFEWTVVKFFWYIFFMYFTLMYFTFYGMMTVAVTPNHNIAAIVSSAFYG
LWNLFSGFIIPKTRIPVWWRWYYYACPIAWTLYGLVASQFGDVQDRLDTDETVAEFVHSYFGFQHDFVGYVAI
IITGLAVLFGFIFAFSIRAFNFQKR

>XP_013454712.1_PDR1_Mt
MEGSDIYSASNSLRFSMRSSSTTGWRNGTMEAFAKSSRREEDDDEEALKWAALEKLPTYNRLRKGLLATSRG
VANEVDITDLGFQEKQKLLDRLINVAEEGNEKFLLKLKERIDRVGIEIPAIEVRYEHLNVEAEAFVGGRALPTLL
NSVTNTVESILISLHILTSRKKQMTILKDVSGIVKPRRMTLLLGPPSSGKTTLLLALSGKLDPNLKVSGRVTYNG
HGMDEFVPQRTAAYISQHDVHIGEMTVRETLAFSARCQGVGSRYDLLAELSRREKEANIKPDPDVDVFMKAM
ATGGQQESVATDYVLKLLGLDVCADTMVGNEMLRGISGGQRKRVTTGEMLVGPANALFMDEISTGLDSSTTF
QIVRSLQQYVHILNGTTVISLLQPAPETYELFDDIILISDGQIVYQGPREHILEFFESVGFKCPERKGAADFLQEVT
SKKDQEQYWVDREKPYRFVTVTQFAEAFQSYHVGRKTGDELAIPFDKSKNHPAALTTKKYGVNKKELLKANF
SREYLLMKRNSFVYIFKICQLLLMATIAMTLFLRTEMHRDSLNGGGVYSGALFFAVVMIMFNGMAELSMTIAK
LPSFYKQRDLLFFPSWAYAIPTWILKIPITFLEVAAWVFLTYYVIGFDPNVTRLLKQYLLLLLINQMASGLFRAIA
ALGRNMIVANTFGSFALLALLTLGGFVMSRKDIKSWWIWGYWISPLMYGQNAIMVNEFLGDSWNHFTPNSNK
TLGIQVLESRGFFTEAYWYWIGIGALTGFMFLFNILFTVALTYLDPFDKPQATINEESEDSTTNGTTQEVELPRIA
SSGGSNGADPSQRERRGMVLPFEPHSIAFDDVVYSVDMPQEMKVQGVLEDRLVLLKGVSGAFRPGVLTALMG
VSGAGKTTLMDVLAGRKTGGYIDGSIKISGYPKKQETFARISGYCEQNDIHSPHVTVYESLVYSAWLRLPADV
DSNTRKMFIEEVMELVELNPLRNSLVGLPGVNGLSTEQRKRLTIAVELVANPSIIFMDEPTSGLDARAAAIVMR

TVRNTVDTGRTVVCTIHQPSIDIFEAFDELFLMKRGGEEIYVGPLGRHSSQLIKYFESIEGVSKIKDGYNPATWM
LEVSSSAQELTLGIDFHHAYKNSELYRRNKQLIEELGKPAPGSNDLYFSAQYSQSFLVQCLACLWKQHWSYWR
NPPYTSVRFFFTVFIGLMFGTIFWDLGRKYSKRQDLFNALGSMYTAVLFLGVQNSSAVQPVVAVERSVFYRER
AAGMYSALPYAFAQVLIELPYIFVQAASYGVIVYAMIGFEWTVAKFLWYIFFMYCTLCYFTFYGMMAVAITPN
HHVASIVAAAFYAIWNLFSGFIVPRPMIPVWWRWYYWGCPVSWTLYGLIASQFGDITKIMESENESVQEFIRSY
FGMKHDFIGVCAVVVVGTAVLFACIFAVSIKVFNFQRR

>XP_013586280.1_P_ABCG40_Bo
MEGTSFQKASNSLRRDSSAWKRDSGMEIFSRSSREEDDEEALKWAALEKLPTFDRLRKGILTASHGINQIDIEKL
GFQDTKKLLERLIKVGDDEHEKLLWKLKNRIDRVGIDLPTIEVRFDHLKVEAEVHVGGRALPTFVNFISNFADK
LLNSLHLLPNRKKKFTILNDVSGIVKPGRMALLLGPPSSGKTTLLLALAGKLDHELKETGRVTYNGHGMNEFV
PQRAAAYIGQNDVHIGEMTVRETFAYAARFQGVGSRYDMLTELARREKEANIKPDPDIDVFMKATSTTGEETN
VMTDYILKILGLEVCADTMVGDDMLRGISGGQKKRVTTGEMLVGPSRALFMDEISTGLDSSTTYQIVNSLRNY
VHIFNGTALISLLQPAPETFDLFDDIFLIAEGEIIYEGPREHVVEFFETMGFKCPPRKGVADFLQEVTSKKDQMQY
WARPDEPYRFIRVREFAEAFQSFHVGRRMGDELAVPFDKKKSHPAALTTKKYGVGIKELVNTSFSREYLLMKR
NSFVYYFKFGQLLVMAFATMTLFFRTEMQKKTVVDGSLYTGALFFILMMLMFNGMSELSMTIAKLPVFYKQR
DLLFYPAWVYSLPPWLLKIPISFIEAALTAFITYYVIGFDPNIGRLFKQYILLVLMNQMASALFKMVAALGRNMI
VANTFGAFAMLVFFALGGVVLSKDDIKKWWIWGYWISPIMYGQNAIVANEFFGHSWSRAVPNSSDTLGVTVL
KSRGFLPHAYWYWIGTGALLGYVVLFNFGFTLALTYLNSLGKPQAVLTEEPASNETELLVVEANANKKKGMV
LPFEPHSITFDNVIYSVDMPQEMIEQGTQEDRLVLLKGVNGAFRPGVLTALMGVSGAGKTTLMDVLAGRKTG
GYIDGNITISGYPKNQQTFARISGYCEQTDIHSPHVTVYESLVYSAWLRLPKEVDSNTRKMFIDEVMDLVELTPL
RQALVGLPGESGLSTEQRKRLTIAVELVANPSIIFMDEPTSGLDARAAAIVMRTVRNTVDTGRTVVCTIHQPSID
IFEAFDELFLLKRGGEEIYVGPLGHESSHLINYFESIQGISKITEGYNPATWMLEVSTTSQEAALGVDFAQLYKNS
ELYTRNKDLIKELSQPAPGSKDLYFPTQYSQSFWTQCMASLWKQHWSYWRNPPYTAVRFLFTIGIALMFGTMF
WDLGGKTRTQQDLSNAMGSMYTAVLFLGLQNAASVQPVVNVERTVFYREQAAGMYSAMPYAFAQVFIEMP
YVLVQAVVYGLIVYAMIGFEWTAAKFFWYLFFMYGSFLTFTFYGMMAVAMTPNHHIASVVSSAFYGIWNLFS
GFLIPRPSMPVWWEWYYWLCPVSWTLYGLITSQFGDITEPMADGTSVKQFIKDFYGFREGFLGVVAAMNVIFP
LAFAIIFAIGIKSFNFQKR

>XP_013641491.1_ABCG40_Bn
MEGTSFQKASNSLRRDSSAWKRDSGMEIFSRSSREEDDEEALKWAALEKLPTFDRLRKGILTASHGINQIDIEKL
GFQDTKKLLERLIKVGDDEHEKLLWKLKNRIDRVGIDLPTIEVRFDHLKVEAEVHVGGRALPTFVNFMSNFAD
KLLNSLHLLPNRKKKFTILNDVSGIVKPGRMALLLGPPSSGKTTLLLALAGKLDLELKETGRVTYNGHGMNEF
VPQRAAAYIGQNDVHIGEMTVRETFAYAARFQGVGSRYDMLTELARREKEANIKPDPDIDVFMKATSTAGEE
TNVMTDYILKILGLEVCADTMVGDDMLRGISGGQKKRVTTGEMLVGPSRALFMDEISTGLDSSTTYQIVNSLR
NYVHIFNGTALISLLQPAPETFDLFDDIFLIAEGEIIYEGPREHVVEFFETMGFKCPPRKGVADFLQEVTSKKDQM
QYWARPDEPYRFIRVREFAEAFQSFHVGRRMGDELAVPFDKKKSHPAALTTKKYGVGIKELVNTSFSREYLLM
KRNSFVYYFKFGQLLVMAFATMTLFFRTEMQKKTVVDGSLYTGALFFILMMLMFNGMSELSMTIAKLPVFYK
QRDLLFYPAWVYSLPPWLLKIPISFIEAALTAFITYYVIGFDPNIGRLFKQYILLVLMNQMASALFKMVAALGRN
MIVANTFGAFAMLVFFALGGVVLSKDDIKKWWIWGYWISPIMYGQNAIVANEFFGHSWSRAVPNSSDTLGVT
VLKSRGFLPHAYWYWIGTGALLGFVVLFNFGFTLALTYLNSLGKPQAVLTEEPASNETELLVVEANANKKKG
MVLPFEPHSITFDNVIYSVDMPQEMIEQGTQEDKLVLLKGVNGAFRPGVLTALMGVSGAGKTTLMDVLAGRK
TGGYIDGNITISGYPKNQQTFARISGYCEQTDIHSPHVTVYESLVYSAWLRLPKEVDSNTRKMFIDEVMDLVEL
TPLRQALVGLPGESGLSTEQRKRLTIAVELVANPSIIFMDEPTSGLDARAAAIVMRTVRNTVDTGRTVVCTIHQP
SIDIFEAFDELFLLKRGGEEIYVGPLGHESSHLINYFESIQGISKITEGYNPATWMLEVSTTSQEAALGVDFAQLY
KNSELYKRNKELIKELSQPAPGSKDLYFPTQYSQSFWTQCMASLWKQHWSYWRNPPYTAVRFLFTIGIALMFG
TMFWDLGGKTRTQQDLSNAMGSMYTAVLFLGLQNAASVQPVVNVERTVFYREQAAGMYSAMPYAFAQVFI
EMPYVLVQAVVYGLIVYAMIGFEWTAAKFFWYLFFMYGSFLTFTFYGMMAVAMTPNHHIASVVSSAFYGIW
NLFSGFLIPRPSMPVWWEWYYWLCPVSWTLYGLITSQFGDITTPMADGTSVKQFIKDFYGFREGFLGVVAAM
NVIFPLAFAIIFAIGIKSFNFQKR

>XP_013749948.1_ABCG40-l_Bn
MEGTSFQKASNSLRRDSSGWKRDSGMEIFSRSSREEDDEEALKWAALEKLPTFDRLRKGILTASHGINQIDIEKL
GFQDTKKLLERLIKVGDDEHEKLLWKLKNRIDRVGIDLPTIEVRFDHLKVEAEVHVGGRALPTFVNFISNFADK

LLNSLHLLPNRKKKFTILNDVSGIVKPGRMALLLGPPSSGKTTLLLALAGKLDHELKETGRVTYNGHGMNEFV
PQRAAAYIGQNDVHIGEMTVRETFAYAARFQGVGSRYDMLTELARREKEANIKPDPDIDVFMKATSTTGEETN
VMTDYILKILGLEVCADTMVGDDMLRGISGGQKKRVTTGEMLVGPSRALFMDEISTGLDSSTTYQIVNSLRNY
VHIFNGTALISLLQPAPETFDLFDDIFLIAEGEIIYEGPREHVVEFFETMGFKCPPRKGVADFLQEVTSKKDQMQY
WARPDEPYRFIRVREFAEAFQSFHVGRRMGDELAVPFDKKKSHPAALTTKKYGVGIKELVNTSFSREYLLMKR
NSFVYYFKFGQLLVMAFATMTLFFRTEMQKKTVVDGSLYTGALFFILMMLMFNGMSELSMTIAKLPVFYKQR
DLLFYPAWVYSLPPWLLKIPISFIEAALTAFITYYVIGFDPNIGRLFKQYILLVLMNQMASALFKMVAALGRNMI
VANTFGAFAMLVFFALGGVVLSKDDIKKWWIWGYWISPIMYGQNAIVANEFFGHSWSRAVPNSSDTLGVTVL
KSRGFLPHAYWYWIGTGALLGYVVLFNFGFTLALTYLNSLGKPQAVLTEEPASNETELLVVEANANKKKGMV
LPFEPHSITFDNVIYSVDMPQEMIEQGTQEDRLVLLKGVNGAFRPGVLTALMGVSGAGKTTLMDVLAGRKTG
GYIDGNITISGYPKNQQTFARISGYCEQTDIHSPHVTVYESLVYSAWLRLPKEVDSNTRKMFIDEVMDLVELTPL
RQALVGLPGESGLSTEQRKRLTIAVELVANPSIIFMDEPTSGLDARAAAIVMRTVRNTVDTGRTVVCTIHQPSID
IFEAFDELFLLKRGGEEIYVGPLGHESSHLINYFESIQGISKITEGYNPATWMLEVSTTSQEAALGVDFAQLYKNS
ELYKRNKDLIKELSQPAPGSKDLYFPTQYSQSFWTQCMASLWKQHWSYWRNPPYTAVRFLFTIGIALMFGTM
FWDLGGKTRTQQDLSNAMGSMYTAVLFLGLQNAASVQPVVNVERTVFYREQAAGMYSAMPYAFAQVFIEM
PYVLVQAVVYGLIVYAMIGFEWTAAKFFWYLFFMYGSFLTFTFYGMMAVAMTPNHHIASVVSSAFYGIWNLF
SGFLIPRPSMPVWWEWYYWLCPVSWTLYGLITSQFGDITEPMEDGTSVKQFIKDFYGFREGFLGVVAAMNVIF
PLAFAIIFAIGIKSFNFQKR

>XP_014500773.1_ABCG40_Vr
MDNSDIYRASNSMRSRSSTVWRNNSMEVFSRSTREEDDEEALKWAALEKLPTYNRLRKGLLTASHGAANEID
VADLGFQDKQKLLERLVKVAEEDNERFLLKLKERIDRVGLDIPTIEVRYEHLKIEAEAFVGSRALPSFINSATNV
IEGFFNALHIVPSRKKHVTILKDVSGIIKPRRMTLLLGPPSSGKTTLLLALSGKLDKSLQVSGKVTYNGHELNEF
VPQRTAAYISQHDVHIGEMTVRETLAFSARCQGVGSRYDMLSELSRREKAANIKPDPDLDVYMKATATAGQE
SSIVTDYTMKILGLDICADTMVGDEMLRGISGGQRKRVTTGEMLVGPANALFMDEISTGLDSSTTFQIVSSLRQ
YVHILNGTAVISLLQPAPETYDLFDDIILISDGQVVYHGPREYILDFFESMGFRCPERKGAADFLQEVTSKKDQA
QYWVRRDQPYRFVTVTQFAEAFQSFHIGRKLGQELAVPFDKTKSHPAALTTKKFGINKKELLKANFSREYLLM
KRNSFVYIFKLCQLFIMALIALTLFFRTEMHHDDLDDAGVYAGAIFFTIMTIMFNGMAEISMTIAKLPVFYKQRD
LLFFPSWAYAIPSWILKIPVTIAEVALWVFLTYYVIGFDPNVGRLFKQYLILLFISQMASGLFRAIAALGRNMIVA
NTFGSFAVLTLLTLGGFILSKRDIKKWWIWGFWISPLMYGQNALMTNEFLGNSWHNATHNLGLEYLDSRAFF
KDSYWFWLGFGALVGFVLLFNVLFGLALQYLDPFDKPQAFIAEDPDTEASATEVELPRLESSGRDGSVVESSH
GKKKGMVLPFEPHSITFDEIVYSVDMPQEMKDQGIQEDRLVLLKGVSGAFRPGVLTALMGVSGAGKTTLMDV
LAGRKTGGYIDGSIKISGYPKKQETFARISGYCEQNDIHSPHVTVYESLVYSAWLRLPSSVDSKTRKMFIEEVME
LVELNPLRNSLVGLPGVSGLSTEQRKRLTIAVELVANPSIIFMDEPTSGLDARAAAIVMRTVRNTVDTGRTVVC
TIHQPSIDIFEAFDELFLMKRGGQEIYVGPLGRHSSHLIKYFESIDGVSKIKDGYNPATWMLEVTTTAQELSLGV
DFTDLYKNSDLYRRNKQLIQELGEPAPGSKDLYFPTQYSQSFLVQCQACLWKQRWSYWRNPPYTSVRFFFTTF
IAIMFGTIFWDLGGKHSTRGDLMNALGSMYTAVLFLGVQNSSSVQPVVAVERTVFYREKAAGMYSALPYAFS
QILVELPYIFFQAVTYGVIVYAMIGFDWTAEKFFWYLFFMYFTLLYFTFYGMMAVAVTPNHHVAAIVAAAFY
AIWNLFSGFVVSRPSIPIWWRWYYWACPVAWTLYGLVGSQFGDIMENMPFEHKTVKDFVEDSYGIKHDFIGV
AAVVVAGIAVLFAFTFAVAIKTFNFQKR

>XP_014500774.1_ABCG40-l_X1_Vr
MEGSDIYRASNSMRARSSTVWRNSSVEVFSRSSREEDDEEALKWAALEKLPTYNRLRKGLLTASHGAANEIDV
ADLGFQDRQKLLERLVKVAEEDNERFLLKLRERIDRVGLDIPTIEVRYEHLKIEAEAFVGGRALPSFINSATNVI
EGFLNALHILPSRKKHVTILKDVSGIIKPRRMTLLLGPPSSGKTTLLLALSGKLDKSLQVSGKVTYNGHEMNEFV
PQRTAAYISQHDVHIGEMTVRETLAFSARCQGVGSRYDMLSELSRREKAANIKPDPDLDVYMKATATAGQESS
IVTDYTMKILGLDICADTMVGDEMLRGISGGQRKRVTTGEMLVGPANALFMDEISTGLDSSTTFQIVSSLRQYV
HILNGTAVISLLQPAPETYDLFDDIILISDGQVVYHGPREYILDFFESMGFRCPERKGAADFLQEVTSKKDQAQY
WVRRDQPYRFVTVTQFAEAFQSFHIGRKLGQELAVPFDKTKSHPAALTTKKFGINKKELLKANFSREYLLMKR
NSFVYIFKLCQLFIMALIALTLFFRTEMHHDNLDDAGVYAGAIFFTIVTVMFNGMAEISMTIAKLPVFYKQRDL
LFFPSWAYAIPSWILKIPVTIVEVALWVFLTYYVIGFDPNVGRLFKQYLILLFISQMASSLFRAIAALGRNMIVAN
TFGSFAVLTLLTLGGFILSKRDIKKWWIWGFWISPLMYGQNALMGNEFLGNSWHNATHNLGLEYLDSRAFFK
DSYWYWLGFGALVGFVLLFNVLFGLALEYLDPFDKPQAVIAEDPTTEASATEVELPRIESSGRDGSVVESCHG
KKKGMVLPFEPHSITFDEIVYSVDMPQEMKEQGVQEDRLVLLKGVSGAFRPGVLTALMGVSGAGKTTLMDVL

AGRKTGGYIDGSIKISGYPKKQETFARISGYCEQNDIHSPHVTVYESLVYSAWLRLPSSVDSKTRKMFIEEVMEL
VELNPLRNSLVGLPGVSGLSTEQRKRLTIAVELVANPSIIFMDEPTSGLDARAAAIVMRTVRNTVDTGRTVVCTI
HQPSIDIFEAFDELFLMKRGGQEIYVGPLGRHSSHLIKYFESIDGVSKIKDGYNPATWMLEVTTTAQELSLGVDF
TDLYKNSDLYRRNKQLIQELGQPAPGSKDLYFPTQYSQSFLVQCQACLWKQRWSYWRNPPYTSVRFFFTTFIAI
MFGTMFWDLGGKHSTRGDLMNALGSMYTAVLFLGVQNSSSVQPVVAVERTVFYREKAAGMYSALPYAFSQI
LVELPYIFFQAVTYGVIVYAMIGFEWTAEKFFWYLFFMYFTLLYFTFYGMMGVAVTPNHHVASIVAAAFYAI
WNLFSGFVVSRPSIPIWWRWYYWACPVAWTLYGLVGSQFGDILEVMPSENKTVKAFIEDSYGIKHDFIGVAAV
VVAGIAVLFAFTFAVAIKTFNFQKR

>XP_014501298.1_PDR1_Vr
MEGDIYRASNSLRARSSTVWRNSGVEAFSRSSREEDDEEALKWAALEKLPTYNRLRKGLLTASHGVANEIDVT
DLGFQQKQKLLERLVKVAEEDNERFLLKLRERIDRVGLDLPTIEVRYEHLNIDAEAFAGSRALPSFINSVTNVLE
GFLNLLHIVPSKKKHVTILKDVSGVIKPRRMTLLLGPPSSGKTTLLLALSGKLDESLKVSGKVTYNGHELNEFVP
QRTAAYISQHDVHIGEMTVRETLAFSARCQGVGSRYDMLSELSRREKAANIKPDPDLDVYMKATATAGQESSI
VTDYTMKILGLDICADTMVGDEMLRGISGGQRKRVTTGEMLVGPANALFMDEISTGLDSSTTFQIVSSLRQYV
HILNGTAVISLLQPAPETYDLFDDIILISDGQVVYHGPREYILDFFESMGFRCPERKGAADFLQEVTSKKDQAQY
WVRRDQPYRFVTVTQFAEAFQSFHIGRKLGQELAVPFDKTKSHPAALTTKRFGINKKELLKANFSREYLLMKR
NSFVYIFKLCQLFIMALIALTLFFRTEMHHDDIDDAGVYAGAIFFTIMTVMFNGMADISMTIAKLPVFYKQRNL
LFYPSWAYAIPSWILKIPVTLAEVSVWVFMTYYVIGFDPNVGRFFKQFLILFFISQMASGLFRAIAALGRNMIVA
NTFGSFAVLTLLALGGFILSKRDIKNWWIWGFWISPLMYGQNALMINEFLGNNWHNATYDLGVAYLDSRAFF
TDSYWYWIGFAGLVGFVLVLNGLFAFALEFLGPFDKPQASVTEEEPEANNGGTVAEVELPRRESSGRDGSVVE
SSHGKKKGMVLPFEPHSITFDEIVYSVDMPQEMKDQGVQEDRLVLLKGVSGAFRPGVLTALMGVSGAGKTTL
MDVLAGRKTGGYIDGSIKISGYPKKQETFARISGYCEQNDIHSPHVTVYESLVYSAWLRLPSSVDSKTRKMFIE
EVMELVELNPLRNSLVGLPGVSGLSTEQRKRLTIAVELVANPSIIFMDEPTSGLDARAAAIVMRTVRNTVDTGR
TVVCTIHQPSIDIFEAFDELFLMKRGGQEIYVGPLGRHSSHLIKYFESIDGVSKIKDGYNPATWMLEVTTTAQEL
SLGVDFTDLYKNSDLYRRNKQLIQELSQPAPGSKDLHFPSRYSQSFLVQCQACLWKQRWSYWRNPPYTSVRFF
FTTFIALMFGTMFWDLGGKHSTRGDLMNAIGSMYTAVLFLGVQNSSSVQPVVAVERSVFYREKAAGMYSALP
YAFSQILVELPYIFAQAVTYGLIVYAMIGFEWTAEKFFWYLFFMYFTLLYFTFYGMMAVGVTPNHHVASIVAA
AFYAVWNLFSGFVVARPSIPIWWRWYYWACPVAWTLYGLIGSQFGDLTEFMSEEGMNVKDFIEHSYGIEHDFI
GVAAVVVAGIAVLFAFIFAVAIKTFNFQKR

>XP_014502148.1_ABCG39_Vr
MESDELRAASARIGSSSVWRSSGVDVFSGSSRREEDEQELKWAAIEKLPTYLRMTRGILTEAEGQPPTEIHINKL
GPLQRKNLVERLVKIAEQDNEKFLFKLRDRIDRVGLEIPTIEVRFEHLNVEAEAHVGSRALPTIFNFCIDLLEGFL
NSLYLIPSRKKPFTVLDDVSGIIKPKRMTLLLGPPSSGKTTLLLALAGRLGKDLKSSGRVLYNGRGMEEFVPQRT
SAYISQTDLHIGEMTVRETLAFSARCQGIGTRYEMLAELSRREKAENIKPDPDLDIYMKAALEGQETNVVTDY
IMKILGLEVCADTMVGDDMIRGISGGQKKRVTTGEMLVGPARALFMDEISTGLDSSTTFQMVNSLRQSIHILNG
TAVISLLQPAPETYELFDDIILLSDGQIVYQGPRENVLEFFEYMGFKCPERKGVADFLQEVTSRKDQEQYWANK
DEPYIFVTVREFAEAFQSFHIGRKLGDELATPFDMSKGHPAVLTKNKYGVSKKELLKACVSREFLLMKRNSFV
YIFKMWQLILTGFITMTLFLRTEMHRDTETDGGIFMGALFFVLIVIMFNGYSELSMSIMKLPVFYKQRDLLFFPS
WAYSLPTWILKIPITLVEVGIWVVMTYYVIGFDPSIERFIKQYFLLVCINQMASGLFRFMGAVGRNIIVANTVGS
FALLAVMVMGGFILSRVDVKRWWLWGYWFSPMMYAQNALAVNEFLGKSWLHVAPNSTEPLGVKVLKSRGI
FPEAYWYWIGVGASIGYMLLFNFLFPLALQYLDPFGKPQALISEEALAERNAGRKEHIIELSSRFKGSSDKGNEN
RRSVCSGTLSARVGSIGASEHNTKRGMVLPFTPLSITFDEIRYAVEMPQEMKNQGILEDRLELLKGVNGAFRPG
VLTALMGVSGAGKTTLMDVLSGRKSTGYIQGQITISGYPKKQETFARIAGYCEQTDIHSPHVTVYESLVYSAW
LRLPLEVDSATRQMFIEEVMELVELTSLREALVGLPGVNGLSTEQRKRLTIAVELVANPSIIFMDEPTSGLDARA
AAIVMRTVRNTVDTGRTVVCTIHQPSIDIFDAFDELLLLKRGGEEIYVGPLGQHCSQLISYFEGINGVPKIKTGY
NPATWMLEVTSEAQEAALGVNFTEIYKNSDLYRRNKALIRELSTPPAGSKDLYFPTKYSQTFFTQCMACLWKQ
HLSYWRNPPYSAVRLLFTTIIALLFGTIFWDIGSKRQRRQDIFNAMGSMYAAVLFIGIQNSTSVQPVVAIERTVF
YRERAAGMYSALPYAFGQVAIEIPYILIQTLVYGVIVYAMIGFDWTLSKFFWYLFFMFFTFLYFTLYGMMAVG
LTPDHNVAAIVSFGFYMIWNLFSGFVIPRTRMPVWWRWYFWICPVSWTLYGLVTSQFGDIKERIENGESVEEF
VRSYFGYRDDFVGVAAAVLVGFSLLFGFTFAFSIKAFNFQKR

>XP_014516644.1_ABCG40_Vr

MEGSDIYRASNSLRASSSTAWRRSIMEGFSRSSNHEEGTDEEALKWAALEKLPTYNRLKKGLLTTSRGVANEID
ITELGFRERQKLLDRLINVAEEDNERFLLKLKGRIDRVGIDIPTIEVRYEHLNVEAETYVGSRALPTFVNFVTNIV
ESVFTSLHILSGKKKHVTILKDVSGIIKPRRMTLLLGPPSSGKTTLLLALSGKLDPNLKASGRVTYNGHGFDEFV
PQRTAAYISQNDVHIGEMTVRETLAFSARCQGVGTRYDLLSELARREKEAKIKPDPDIDVYMKAAVTGGQEAS
LVTDYVLKILGLDICADTMMGDEMLRGISGGQRKRVTTGEMLVGPANALFMDEISTGLDSSTTFQIVKCLRQY
VHILDGTAVISLLQPAPETYELFDDIILISDGQIVYQGPREHVLEFFESVGFQCPERKGVADFLQEVTSRKDQEQY
WMHRNEPYRFVTVTQFTEAFQSFHVGRRIGEELATPFDKSKNHPAALTTKRYGVNKKELIKANISREFLLMKR
NSFVYIFKLFQLTTLAILTMTMFLRTEMHRNNLGDGGVYTGALFFAVVILMFNGLAEISMTIVKLPIFYKQRDL
LFYPSWAYAIPSWILKIPITFLEAAVWVFLTYYVIGFDPNVSRFLKQYLVLLLINQMSSGLFRAIAALGRNMIVA
NTFGSFALLILFALGGFILSRNDIKDWWIWGYWISPLMYGQNAIVVNEFLGNSWNHFTPNSNKTLGIQILESRGF
FTHAYWYWIGIGALIGFIFLFNIMYTLALTYLNPFDKPQTTITEESEGGVANGRAREEGLTCLESSGSCSRRKNR
GMVLPFEPYSITFDQIVYSVDMPQEMKDQGVREDKLVLLKGVSGAFCPGVLTALMGVSGAGKTTLMDVLAG
RKTGGYIEGNIKVSGYPKRQETFARISGYCEQNDIHSPHVTVYESLVYSAWLRLTAEVESNTRKMFIEEVMELV
ELNPLRNSLVGLPGVSGLSTEQRKRLTIAVELVANPSIIFMDEPTSGLDARAAAIVMRTVRNTVDTGRTVVCTIH
QPSIDIFEAFDELFLMKRGGQEIYVGPLGRHSSHLIKYFESIEGVSKIKDGYNPATWMLEVTTPAQELTLGVDFH
EVYRNSELYRRNKQLIAELGNPGHGSKDIHFPTQYAQSLLVQCLACLWKQHWSYWRNPPYTAVRFLSTTVIA
VMFGTMFWDLGGKYSSRQDLFNAMGSMYNAVLFVGIQNSASVQPVLAIGRTVFYRERAAGMYSAFPYALAQ
VLIELPYIFVQATTYSVIVYSMMGFEWTLEKFFWYMFFMYFTLCYFTFYGMMTVAVTPNHHVASVVAAAFYG
IWNLFSGFVIPRPSMPLWWRWYYWACPVAWTIYGLLASQFGDITDVMKLENISVQEFLRSYFGIRHDFIGVSAI
MVSGFAGLFAVIFAVSIKAFNFQKR

>XP_014630001.1_PDR1_Gm
MESGELRVASARIGSSSVWRSSGGVDVFSGSSRRDDDEQELKWAAIEKLPTYLRMTRGILTEAEGQPTEIDINK
LCPLQRKNLVERLVKIAEQDNEKFLFKLRDRIDSVGLEIPAIEVRFEHLNVEAEAHVGSRALPTIFNFCINLLEGF
LNSLHLIPSRKKPFTVLDDVSGIIKPKRMSLLLGPPSSGKTTLLLALAGRLGKDLKFSGRVSYNGHGMEEFVPQR
TSAYISQTDLHIGEMTVRETLAFSARCQGIGTRNEMLAELSRREKAANIKPDPDLDIYMKAAALEGQETNVVTD
YIMKILGLEICADTMVGDDMIRGISGGQKKRVTTGEMLVGPARALLMDEISTGLDSSTTFQMVNSLRQSIHILN
GTAVISLLQPAPETYELFDDIILLSDGQIVYQGPRENVLEFFEYMGFKCPERKGVADFLQEVTSRKDQEQYWAN
KDEPYSFVTVKEFAEAFQSFHVGRKLGDELATPFDMSKGHPAVLTKNKYGVCKKELLKACVSREFLLMKRNS
FVYIFKMWQLILTGFITMTLFLRTEMHRDTETDGGIYMGALFFVLIVIMFNGYSELSMSIMKLPVFYKQRDLLFF
PCWAYSLPTWILKIPITLVEVGIWVVMTYYVIGFDPSIERFIKQYFLLVCINQMASGLFRFMGAVGRNIIVANTV
GSFALLAVMVMGGFILSRVDVKKWWLWGYWFSPMMYGQNALAVNEFLGKSWSHVPPNSTEPLGVKVLKSR
GIFPEAYWYWIGVGASIGYMLLFNFLFPLALHYLDPFGKPQALISEEALAERNAGRNEHIIELSSRIKGSSDRGNE
SRRNMSSRTLSARVGSIGASEHNKKRGMVLPFTPLSITFDEIRYSVEMPQEMKSQGILEDRLELLKGVNGVFRP
GVLTALMGVSGAGKTTLMDVLSGRKTAGYVQGQITISGYPKKQETFARIAGYCEQTDIHSPHVTVYESLVYSA
WLRLPPEVDSVTRQMFIEEVMELVELTSLREALVGLPGVNGLSTEQRKRLTIAVELVANPSIIFMDEPTSGLDAR
AAAIVMRTVRNTVDTGRTVVCTIHQPSIDIFDAFDELLLLKRGGEEIYVGPLGQCCSQLINYFEGINGVPKIKKG
YNPATWMLEVTSEAQEAALGLNFAEIYKNSDLYRRNKALIRELSTPTTGFKDLYFPTKYSQTFITQCMACLWK
QHLSYWRNPPYSAVRLLFTTIIALLFGTIFWDIGSKRQRKQDLFNAMGSMYAAVLFIGIQNATSVQPVVAIERT
VFYRERAAGMYSALPYAFGQVAIEIPYIFIQTLVYGVIVYAMIGFDWTFSKFFWYLFFMFFTFLYFTFYGMMAV
GLTPDHNVATIVSFGFYMIWNLFSGFVIPRTRMPVWWRWYFWICPVSWTLYGLVTSQFGDIKERIDTGETVEE
FVRSYFGYRDDFVGVAAAVLVGFTLLFGFTFAFSIKAFNFQKR

>XP_015076369.1_P_PDR1-l_Sp
MEPVNLNSMRGSSMRGSMRGSLRASTSNSIWRNNGVDAFSRSTRDEDDEEALKWAALEKLPTFDRLRKGLLF
GSQGATNEIDVNDLGYQERKNLLERLVKVADEDNEKFLMKLKNRIDRVGIDMPSIEVRYEHLNIEADAYAGSR
ALPTFINFMTNFVETLLNSLHILPSKKRQITILKDVSGMIKPCRMTLLLGPPSSGKTTLLLALAGKLDPALRVTGN
VTYNGHELHEFVPQRTAVYISQHDLHIGEMTVRETLEFSARCQGVGSRFEMLAELSRREKAANIKPDPDIDIYM
KAAATEGQEANVVTDYVLKILGLDICADTMVGDEMIRGISGGQKKRVTTGEMLVGPSKALFMDEISTGLDSST
TFSIVNSLRQSVQLLKGTAVISLLQPAPETYNLFDDIILLSDGYIVYQGPREAVLDFFESMGFKCPERKGAADFL
QEVTSKKDQQQYWAKRNEPYRFVTSKEFAEAYQSFHVGRKLSDELATPYDKTKSHPAALSTKKYGIGTKQLL
KVCAEREFLLMKRNSFVYIFKLTQLAIMALITMSVFFRTKLPRDDMDDGGIYAGALFFVVVMIMFNGMAEIAL
TIFKLPVYFKQRDLLFFPSWAYALPTWILKIPITFVECGMWTFLTYYVMGFDPNVSRLFKQFLLLVLVHQMASA
LFRFIGAVGRTMGVASTFGAFALLLQFALGGFVLAREDVKKWWIWGYWTSPLMYSVNSILVNEFDGKNWKH

VAPNGTEPLGAAVVRSRGFFPDAYWYWIGCGALFGFTMIFNFFYSIALAYLDPFGKPQAMISEDGEDAVELTE
RSETEGQDKKKGMVLPFEPHSITFDNVIYSVDMPQEMKEQGSAEDRLVLLKGVSGAFRPGVLTALMGVSGAG
KTTLMDVLAGRKTGGYIDGDIKISGYPKKQETFARISGYCEQNDIHSPYVTVYESLVYSAWLRLPQDVDENKR
KMFVDEVMELVELAPLRSALVGLPGVNGLSTEQRKRLTIAVELVANPSIIFMDEPTSGLDARAAAIVMRAVRN
TVDTGRTVVCTIHQPSIDIFEAFDELFLMKRGGQEIYVGPLGRHSCHLIKYFESMPGVGKIKEAYNPATWMLEV
TASSQEMMLGVDFADLYKNSDLYRRNKALIAELSTPRPGTKDLHFETQFSQPFWTQCMACLWKQHWSYWRN
PAYTAVRFIFTTFIALVFGTMFWDLGTKVSRSQDLINAMGSMYAATLFLGVQNSSSVQPVVAVERTVFYREKA
AGMYSAIPYAFGQVVIEIPYVFVQSAFYGLIVYAMIGFEWTAVKFLWYFFFMYCTLLYFTFYGMMTVAVTPNQ
NVASIVAAFFYAVWNLFSGFIVPRPRIPIWWRWYYWACPVAWTLYGLVASQFGDIQTPLTDDENVEQYLRRY
FGFKHDFLGVVAAVIVALPVMFALTFALGIKAFNFQRR

>XP_015083661.1_P_PDR1-l_X1_Sp
MEGGENLVRVSSARLSGSNVWRNSAMDVFSRSSSREDYDDEEALRWAALEKLPTYSRIRRGLLLEEEGQSREV
DIKKLDLIERRNLLDRLVKIADEDNEKLLMKLKQRIDRVGLDLPTIEVRFEHLNIEAEARVGSRALPTIFNFTVNI
LEDFLNYLHILPSRKKPLPILHGVSGIIKPGRMTLLLGPPSSGKTTLLLGLAGKLDKDLKVSGRVTYNGHGMDEF
VPQRTSAYISQNDLHIGEMTVRETLAFSARCQGVGDKYEILAELSRREKEANIKPDPDVDIFMKSAWNEGQEA
NVITDYTLKILGLEICADTLVGDEMIRGISGGQRKRLTTGEMMVGPARALFMDEISTGLDSSTTYQIVNSIRQSIH
ILQGTAVISLLQPAPETYDLFDDIILLSDGEIVYQGPRENVLEFFEYMGFKCPERKGVADFLQEVTSRKDQEQYW
SRRHEPYRFITSCEFSDVFQSFHVGRKLGEELAVPFDKSKSHPAALTTKRYGISKKELLKACTAREYLLMKRNS
FVYIFKMVQLTMMASIAMTLFLRTEMHRDTTIDGAVYLGALFYAVITVMFNGFSELALSIMKLPSFYKQRDLL
FFPAWAYALPTWILKIPITLVEIAIWVCMTYYVIGFEADVGRFFKQLFLLICLNQMASGLFRFLAALGRNIIVAN
TFGSCALLVVLVMGGFILSRDDVKQWLIWGYWISPMMYAQNAIAVNEFLGKSWSHVPPNSTGTDTLGVSFLK
SRGIFPEARWYWIGAGALIGYVLLFNFLFTVALAYLNPFGKPQAIISEEIVAEKNASKRGEVIELSPIGKSSSERG
NDVPISASSRSLSSRVGNITEGDLSKRRGMILPFEPLSITFDDIRYAVDMPQEMKAQGFIEDQLELLKGVSGAFRP
GVLTALMGVSGAGKTTLMDVLAGRKTGGYINGTISISGYPKQQETFARISGYCEQTDIHSPHVTVYESLQYSA
WLRLPREVDTETRKSFIEEVMELVELIPLREALVGLPGVNGLSTEQRKRLTVAVELVANPSIIFMDEPTSGLDAR
AAAIVMRTVRNTVDTGRTVVCTIHQPSIDIFDAFDELLLLKRGGEEIFVGPLGRHSSHLIKYFEGIDGVLKIRDGY
NPATWMLEVTSLAQEAVLGIDFTELYKNSELYRRNKALIQELSVAAPGSKDLYFETKYSQSFFTQCMACLWKQ
HLSYWRNPPYTAVRLMFTFFVSLMLGTIFWGLGSKRGRQQDILNAIGSMYSAILFLGVINATSVQPVVAIERTV
FYRERAAGMYSALPYAFGQVMIELPHLFLQTIIYGVIVYAMIGFEWTVTKFFWYLFFMYFTLLYFTLYGMMTV
AVTPNHTIASIVSSAFYTIWNLFCGFVVPKTRMPVWWRWYYYICPLSWTLYGLIASQFGDVQDRLDTKETVEQ
FLENFFDYKHDFVGYVAVILVGISVAFLFIFAYSIKSFNFQKR

>XP_015083662.1_P_PDR1-l_X2_Sp
MEGGENLVRVSSARLSGSNVWRNSAMDVFSRSSSREDYDDEEALRWAALEKLPTYSRIRRGLLLEEEGQSREV
DIKKLDLIERRNLLDRLVKIADEDNEKLLMKLKQRIDRVGLDLPTIEVRFEHLNIEAEARVGSRALPTIFNFTVNI
LEDFLNYLHILPSRKKPLPILHGVSGIIKPGRMTLLLGPPSSGKTTLLLGLAGKLDKDLKVSGRVTYNGHGMDEF
VPQRTSAYISQNDLHIGEMTVRETLAFSARCQGVGDKYEILAELSRREKEANIKPDPDVDIFMKSAWNEGQEA
NVITDYTLKILGLEICADTLVGDEMIRGISGGQRKRLTTGEMMVGPARALFMDEISTGLDSSTTYQIVNSIRQSIH
ILQGTAVISLLQPAPETYDLFDDIILLSDGEIVYQGPRENVLEFFEYMGFKCPERKGVADFLQEVTSRKDQEQYW
SRRHEPYRFITSCEFSDVFQSFHVGRKLGEELAVPFDKSKSHPAALTTKRYGISKKELLKACTAREYLLMKRNS
FVYIFKMVQLTMMASIAMTLFLRTEMHRDTTIDGAVYLGALFYAVITVMFNGFSELALSIMKLPSFYKQRDLL
FFPAWAYALPTWILKIPITLVEIAIWVCMTYYVIGFEADVGRFFKQLFLLICLNQMASGLFRFLAALGRNIIVAN
TFGSCALLVVLVMGGFILSRDDVKQWLIWGYWISPMMYAQNAIAVNEFLGKSWSHVPPNSTGTDTLGVSFLK
SRGIFPEARWYWIGAGALIGYVLLFNFLFTVALAYLNPFGKPQAIISEEIVAEKNASKRGEVIELSPIGKSSSERG
NDVPISASSRSLSSRVGNITEGDLSKRRGMILPFEPLSITFDDIRYAVDMPQEMKAQGFIEDQLELLKGVSGAFRP
GVLTALMGVSGAGKTTLMDVLAGRKTGGYINGTISISGYPKQQETFARISGYCEQTDIHSPHVTVYESLQYSA
WLRLPREVDTETRKSFIEEVMELVELIPLREALVGLPGVNGLSTEQRKRLTVAVELVANPSIIFMDEPTSGLDAR
AAAIVMRTVRNTVDTGRTVVCTIHQPSIDIFDAFDELLLLKRGGEEIFVGPLGRHSSHLIKYFEGIDGVLKIRDGY
NPATWMLEVTSLAQEAVLGIDFTELYKNSELYRRNKALIQELSVAAPGSKDLYFETKYSQSFFTQCMACLWKQ
HLSYWRNPPYTAVRLMFTFFVSLMLGTIFWGLGSKRGRQQDILNAIGSMYSAILFLGVINATSVQPVVAIERTV
FYRERAAGMYSALPYAFGQVMIELPHLFLQTIIYGVIVYAMIGFEWTVTKFF

>XP_015083790.1_P_PDR1-l_Sp

MEGGGDILKVSSARLGSSTVWRNSGVDVFSRSSREDYDDEEALKWAALEKLPTYLRIRRGILSEEEGQYREVDI
TKLDLVERRNLLERLVKIADEDNEKFLLKLKKRIDRVGLDLPTIEVRFEHLNVDAEARVGSRALPTIFNFTVNIIE
DFLNYLHILPSRKKPLPILHEVSGIIKPGRMTLLLGPPSSGKTTLLLGLAGKLDKDLKVSGRVTYNGHGMDEFVP
QRTSAYISQNDLHIGEMTVRETLAFSARCQGVGAKYEILAELSRREKEANIKPDPDVDIFMKSAWNDGQEANV
VTDYTLKILGLDICADTIVGDEMIRGISGGQRKRLTTGEMMVGPARALFMDEISTGLDSSTTYQIVNSIRQSIHIL
QGTAVISLLQPAPETYDLFDDIILLSDGQIVYQGPRENVLEFFEYTGFKCPQRKGVADFLQEVTSRKDQEQYWA
RRDEPYKFITVREFSEAFQSFHVGRKLGDEIAVPFDKSKSHPAALTTERYGVSKKELLKACTAREYLLMKRNSF
VYIFKMIQLTLMATITMTLFLRTEMHRDTMIDGAVFLGALYYAVIMIMFNGFSELALSIMKLPSFYKHRDLLFF
PAWTYALPTWILKIPITLVEVAIWVCMTYYVIGFEADVGRFFKQLFLLICLNQMASGLFRFLAALGRNVIVANT
FGSCALLIVLVMGGFILSRDNVKQWLIWGYWISPMMYAQNAIAVNEFLGKSWAHVPPNSTGTDTLGVSFLKS
RGIFPEARWYWIGVGALLGYVLLFNFLFTVALAYLNPFGKPQAVLSEETVAERNASKRGEVIELSPIGKSSSERG
NDVRRSASSRSMSSRVGNIAEGDLNKRKGMILPFEPLSITFDDIRYAVDMPQEMKAQGFTEDRLELLKGVSGAF
RPGVLTALMGVSGAGKTTLMDVLAGRKTGGYIEGTISISGYPKHQATFARIAGYCEQTDIHSPHVTVYESLQYS
AWLRLPREVDTETRKRFIEEVMELVELKPLREALVGLPGVNGLSTEQRKRLTVAVELVANPSIIFMDEPTSGLD
ARAAAIVMRTVRNTVDTGRTVVCTIHQPSIDIFDAFDELLLLKRGGEEIFVGPLGRHSSHLIKYFEGIDGVAKIR
DGYNPATWMLDITSVAQEAALGIDFTELYRNSELYRRNKALIQELSVPAPGSKDLYFETKYSQSFFTQSMACF
WKQHWSYWRNPPYTAVRLMFTFFIALMFGTIFWDLGSKRRRQQDILNAIGSMYAAVLFLGVQNATSVQPVVA
IERTVFYRERAAGMYSALPYAFGQIMIELPYIFIQTIIYGVIVYAMIGFEWTVAKFFWYLFFMYFTLLYFTLYGM
MTVAVTPNHSIAAIISSAFYAVWNLFSGFIVPKTRMPVWWRWYFYICPISWTLYGLVASQFGDLQDKLETKET
VEEFIESFFDFKYDFVGYVAVILVGISVGFLFIFAYSIKAFNFQKR

>XP_015088126.1_P_PDR1_Sp
MEPANLGNLRGSSLRGSISGSRRGSVSLRANSNSIWRNTGVEIFSRSARDEDDEEALKWAALEKLPTFDRLRKG
LLFGSQGAAAEIDIDDIGLQERKNLLERLVRVADEDNEKFLLKLKNRIDRVGIDLPTIEVRYENLNIEADAYVGS
RGLPTFINFMTNFLETLLNTLHILPSSKRQITILKDISGIIKPCRMTLLLGPPSSGKTTLLLALAGKLDSSLKVTGK
VSYNGHELHEFVPQRTAAYISQHDLHIGEMTVRETLEFSARCQGVGSRYEMLAELSRREKAANIKPDPDIDIYM
KASATEGQEANVVTDYVLKILGLDICADTMVGDEMLRGISGGQKKRVTTGEMLVGPSKALFMDEISTGLDSST
TYSIVNSLRQSVQILKGTAVISLLQPAPETYNLFDDIILISDGYIVYQGPRDDVLEFFESMGFKCPERKGVADFLQ
EVTSKKDQPQYWSRRNEHYRFISSKEFSDAYQSFHVGRKLGDELAIPFDRTKCHPAALTNEKYGIGKKELLKV
CTEREYLLMKRNSFVYVFKFFQLTIMALMAMTLFFRTEMPRDTVDDGGIYAGALFFVVVMIMFNGMSEMAM
TIFKLPVFYKQRDLLFFPSWAYAIPSWILKIPVTLVEVGLWVILTYYVIGFDPNITRFLKQFLLLVLVNQMASGLF
RFMGAVGRTMGVASTFGAFALLLQFALCGFVLSREDVKGWWIWGYWISPLMYSVNSILVNEFEGSKWKHIAP
NGTEPLGVAVVKSRGFFPDAYWYWIGFAALFGFTVVFNFFYSLALAYLKPYGKSQTVRPEDSENAENGQAAS
QMTSTDGGDIVSAGQSKKKGMVLPFEPHSITFDDVVYSVDMPQEMKEQGAGEDRLVLLKGVSGAFRPGVLTA
LMGVSGAGKTTLMDVLAGRKTGGYIDGDIKISGYPKKQETFARISGYCEQNDIHSPYVTVYESLVYSAWLRLP
KDVDEKTRKMFVDEVMELVELEPLRSALVGLPGVNGLSTEQRKRLTIAVELVANPSIIFMDEPTSGLDARAAAI
VMRTVRNTVDTGRTVVCTIHQPSIDIFEAFDELFLMKRGGQEIYVGPLGRHSCHLIKYFESIPGVAKIKEGYNPA
TWMLEVTASAQEMMLGVDFTDLYKNSDLYRRNKALITELSVPRPGSKDLYFETQYSQSMWIQCMACLWKQN
WSYWRNPAYTAVRFIFTMFIALVFGTMFWDIGTKVSQSQDLFNAMGSMYAAVLFLGVQNASSVQPVVDVER
TVFYRERAAGMYSAIPYAFGQVFIEIPYVFVQAIVYGIIVYAMIGFEWEAGKFFWYLFIMFTTLLYFTFYGMMS
VAVTPNQNVSSIVAAFFYAIWNLFSGFIVPRPRMPIWWRWYYWCCPVAWTLYGLVASQFGDIQSRLTDEETVE
QFLRRYFGFRHDFLPVVAGVLVAYVVVFAFTFAFAIKAFNFQRR

>XP_015570748.1_PDR1_Rco
MEGTELYIAGGSLRRGESSIWRSNAMEGFSKSSRGDEDDDEEALKWAAIERLPTYDRLKKGLLTTSKGEANEI
DVKNLGFHEKRTLLDRLVKVAEEDNELFLLKLKNRIDRVGIELPMIEVRFEHLNVETEAHVGSRALPTFFNFSID
IVEGFLNFLHILPSGKKSLSILQDVSGIIKPKRMTLLLGPPSSGKTTLLLALAGKLDPKLKFSGRVTYNGHEMNEF
VPQRTAAYISQHDTHIGEMTVRETLAFAARCQGVGHRYEMISELLRREKASNIKPDPDIDVFMKAMATEGQEA
NVVTDYILKILGLEVCADIMVGNEMLRGVSGGQRKRVTTGEMLVGPAKALFMDEISTGLDSSTTYQIVNSIKQ
YIHILNGTAVISLLQPPPETYNLFDDIILLSDGQIVYQGPRENVLEFFEYMGFKCPERKGVADFLQEVTSRKDQA
QYWADKDKPYSFVTVREFAEAFQSFLVGRRLEAELSTPFDKSKSHPAALTTKKYGVGKMELLKACFSREILLM
KRNSFVYIFKLTQLTIMAMVAMTLFLRTEMHRDSVTNGGIYVGALFFSVVFIMFNGLSEISLTIAKLPVFYKQRS
LLFYPPWAFSLPPWITKIPITLVQVAIWVFLTYYVIGFDPNVGRFFKQYLLLALVSQMASGLFRFIAAAGRNMIV
ANTFGSFALLALFALGGFILSRDNIKKWWIWGYWISPLMYGQNAIVVNEFLGNSWNKKVLPDTTETLGIQVLE

SRGFFTHAYWYWIGVGALVGFTLLYNFFFTLALTFLGPLQKPQAVISEDSASNTSGKTGEVIQLSSVRTELIVEE
NHQKQKGMVLPFEPHSITFNDIRYSVDMPQEMKRQGATEDRLELLRGVSGAFRPGVLTALMGVSGAGKTTLM
DVLAGRKTGGYIEGDIRISGFPKKQETFARISGYCEQNDIHSPHVTVYESLLYSSWLRLPPEVNSETRKMFIEEV
MELVELTPLRQALVGLPGVSGLSTEQRKRLTIAVELVANPSIIFMDEPTSGLDARAAAIVMRTVRNTVDTGRTV
VCTIHQPSIDIFEAFDELLLMKRGGQEIYVGPLGRHSCQLIKYFEAIEGVPDIKDGYNPATWMLEVSSSAQEMVL
GLDFAAIYKNSELYRRNKALIEELSTPPLGSNDLYFPTQYSQSFFTQCMACLWKQHWSYWRNPPYTAVRFLFT
TVIALMFGTMFWDLGSKTTKRQDLFNAMGSMYAAIVFLGIQNASSVQPVVAVERTVFYRERAAGMYSPLPYA
FAQVVIELPYIFLQAAVYGLIVYAMIGFEWSAAKFFWYLFFMYFTLLFYTYYGMMAVAVTPNQQVASIVSSAF
YSIWNLFSGFIIPRPRIPVWWRWYAWTCPVAYTLYGLVSSQFGDIKHTLESGETVEDFVRSYFDFKHELLGAVA
AAVFGFATLFAFTFAFSIKFFNFQRR

>XP_015579753.1_PDR1_Rco
MDGDIFRASNSLRRGSSSIYRNSGVDVFSRSSREEDDEEALRWAALEKLPTYDRLRKGILVSVSKGGANEIDVD
NLGFEERKTLLERLVKVAEEDNEKFLLKLKNRLDRVGIEIPTIEVRFERLNVEAQAFVGTSGLPTFANFSISAIEG
ILNALHVLPNRKRPLTILKDVNGVIKPRRMTLLLGPPSSGKTTLLLALAGKLDPNLKFSGNVTYNGHAMNEFIP
QRTAAYISQHDLHIGEMTVKETLAFSARCQGVGTQHEMLAELSRREKAANIKPDPDIDVFMKAAATEGQETSV
VTDYVLKILGLEVCADTLVGNEMIRGISGGQKKRVTTGEMLVGPAKALFMDEISTGLDSSTTYQIVNSLRQSIHI
LNGTAVISLLQPAPETYNLFDDIILISDGQIVYQGPREHVLDFFEYMGFKCPERKGVADFLQEVTSKKDQQQYW
ARKEQPYTYVPVKEFAETFQSYDLGRRIGEELSTPYDKTKSHPAALSTKRYGVGKMELFKACFAREYLLMKRN
SFVFIFKLCQLLVMAFIGTTVFLRTEMSKDTVTDGNIYTGALFFSLITVMFNGMSELSMTIAKLPVFYKQRDLLF
FPPWAYSIPSWILKIPITFLEVGVWVFITYYVMGFDPNVERLFRQFFLLLLVNQMASGLFRFIASVGRNMIIANTF
GSFALLTLFALGGFVLSREDIKKWWIWGFWVSPLMYGQNAILVNEFLGHSWSHLPANSTSNDSLGVQVLSSRG
FFTESKWYWLGVIASAGYMVLFNILYTIALTVLGSFEKPTAVIADDHESSDVTGGAIQLSQVESSRRSNTESGTS
RHDEANQSKKKGMVLPFEPHSLTFDNVIYSVDMPQEMRNQGVLEDKLVLLKGVSGAFRPGVLTALMGVSGA
GKTTLMDVLAGRKTGGYIEGNITISGYPKKQETFARISGYCEQNDIHSPHVTVYESLVYSAWLRLPAEVDSDTR
KMFVEEVIDLVELNAQRNSLVGLPGVNGLSTEQRKRLTIAVELVANPSIIFMDEPTSGLDARAAAIVMRTVRNT
VDTGRTVVCTIHQPSIDIFEAFDELFLMKRGGEEIYVGPLGRHSCHLINYFEGLEGVSKVTDGYNPATWMLEVT
SSAQELTLGVDFANLYRNSDLYRRNKAMIQELSKPAPGTKDLYFPTQYSQSFLTQCMACLWKQYWSYWRNPP
YTAVRFWFTTFIALMFGTIFWDLGSKTSEPQDLTNAMGSMYAAVLFLGVQNSSSVQPVVAVERTVFYRERAA
GMYSAMPYAYAQALIEVPYIFVQSAAYSIITYAMIGFEWDAAKFLWYLFFLYFTLMYFTFYGMMAVAFTPNH
HIASIVSSAFYSIWNVFAGFIVPRTRLPVWWRWYYWGCPISWTLYGLIASQYGDVKTLIGSDGQTVEEYVEEFY
GMKHDFLGVTAAVIVGITIGFAFIFAVSIKAFNFQRR

>XP_015933410.1_PDR1_Ad
MEGTDIYRASNSLRRSSSVWRNSGVEVFSRSSREENDEEALKWAALEKLPTYNRLRKGLLTASQGPASEVDVT
DLGYQERQKLLERLVKVAEEDNEKFLRKLKQRIDRVGLEIPTVEVRYEHLNIDAEAFVGSRALPSFINSVFNVV
EGFLNMIHVLPSKKKHVTILKNVSGIVKPQRMTLLLGPPGSGKTTLLLALSGKLDKGLQVSGKVTYNGHEMNE
FVPQRTAAYISQHDVHIGEMTVRETLAFSARVQGVGSRYDMLSELARREKAANIKPDPDIDVYMKAVSTAGQ
ESSIATDYTLKILGLDICADTMVGDEMLRGISGGQRKRVTTGEMLVGPTNALFMDEISTGLDSSTTFQIVSSLRQ
YVHIMNGTAVISLLQPAPETYELFDDIILISDGQVVYHGAREYVLDFFESMGFRCPERKGVADFLQEVTSKKDQ
AQYWSRRDEPYRFVTVTQFAEAFQSFHVGRKLNEEIAVPFDKSKSHPAALTTKKYGLNKKELLKANFAREYLL
MKRNSFVYVFKLVQLTIMALIAMTLFFRTKMHHRNQDDAGVYAGALFFTLITIMFNGMAEISMSIAKLPVFYK
QRDLLFYPSWAYAIPSWILKIPVTIVEVAVWVFLTYYVIGFDPNVGRLFKQYLLLLFVHQLASGLFRAIAALGR
NMIVANTFGSFAILAFLSLGGFLLSRKNIKDWWIWGYWCSPLMYGQTGLMVNEFLGHNWNKSSNPNFGVTFL
ESRGFFTDSYWYWLSLGACVGYVFLFNGVFAAALSILGPFDKPQPTITEESEDDAQEVELPRIENSRSAEDAVV
ESSHGKKKGMVLPFEPYAITFDEVVYSVDMPQEMKEQGVQEDKLVLLKGVSGAFRPGVLTALMGVSGAGKT
TLMDVLAGRKTGGYIEGSIKVSGYPKKQETFARVSGYCEQNDIHSPHVTVYESLLYSAWLRLSSSVDSKTRKM
FIEEVMDLVELTPLRNSLVGLPGVSGLSTEQRKRLTIAVELVANPSIIFMDEPTSGLDARAAAIVMRTVRNTVDT
GRTVVCTIHQPSIDIFEAFDELFLMKRGGQEIYVGPLGHHSSHLIKYFESIEGVSKITDGYNPATWMLEVTTTAQ
ELNLGVDFTDLYKNSDLYRRNKQLIQELGQPAPGSKDLHFPTQFSQSFLVQCQACLWKQRLSYWRNPPYTAV
RFFFTTFIALMIGSMFWGLGDKTKKRQDLFNAVGSMYSAVLFLGIQNASSVQPVVAVERTVFYREKAAGMYS
ALPYAFAQILVEIPYLFTQSVTYGLIIYAMLQFDWTAAKFFWYLYFMFFTLCYFTFYGMMAVAVTPNHHVAAI
VAAAFYAIWNLFSGFVVPRPSIPIWWRWYYWGCPVAWSLYGLIGSQFGDITTLMDDEGGKNVGQFIHSYFGIK

HDFIGYAAVVVAGIAVLFAFIFASAIKAFNFQKR

>XP_015937455.1_PDR1_Ad
MENDELRVASARIGSSSIWRSSGAVDVFSGSSRRDDDEEQLKWAAIEKLPTYLRLTRGILTESQGEYTEIDINKL
GPLQRKNLVERLVKIAEQDNEKFLLKLRRRIDRVGLDIPTIEVRFEHLNVEAEAHVGSRALPTNFNFCINLLEGF
LNSLCLLPSRKKPFTVLHDVSGIIKPRRMTLLLGPPSSGKTTLLLALAGRLGKDLKFSGRVSYNGHGMEEFVPQ
RTSAYISQTDLHIGELTVRETLAFSARCQGIGMRYDMLAELSRREKAENIKPDPDLDIYMKAAALEGQETNVVT
DYIMKILGLEVCADTMVGDEMIRGISGGQKKRVTTGEMLVGPARALFMDEISTGLDTSTTFQMVNSLRQSIHIL
NGTAVISLLQPAPETYELFDDVILLSDGQIVYQGPRENVLEFFEYMGFKCPERKGVADFLQEVTSRKDQEQYW
ANKDEPYTFISVREFAEAFQSFHIGRKLGDELATPFDKTKGHPAVLTKNKYGVSKKELLRACVSREFLLMKRNS
FVYIFKMWQLILTGLITMTLFLRTEMHRNTVSDGGIYMGALFFVLIVIMFNGFSELSMTIAKLPVFYKQRDLLFY
PSWAYSLPTWILKIPITLVEVGIWVVMTYYVIGFDPSFERFIKQYFLLACINQMASALFRFMGAVGRNLIVANTF
GSFALLAVMVMGGFILSRVDVRKWWLWGYWISPMMYGQNAIAVNEFLGNSWKHVPPNSTEPLGVKVLKAR
GIFPEAHWYWIGVGASIGYMLLFNLLFPLALHFLDPFGKPQALISEEALAERNSVRNDHIIELSSGRNGASDKSN
GRSVSSRTLSARVGAINGTDRNRKRGMVLPFTPLSITFDEIRYTLDMPQEMKAQGIVEDKLELLKGISGAFRPG
VLTALMGVSGAGKTTLMDVLSGRKTAGYIQGQITISGYPKKQETFARISGYCEQTDIHSPHVTVYESLVYSAW
LRLPPEVDSSTREMFIEEVMELVELNSLKDALVGLPGVNGLSTEQRKRLTIAVELVANPSIIFMDEPTSGLDARA
AAIVMRTVRNTVNTGRTVVCTIHQPSIDIFDAFDELLLLKRGGEEIYVGPLGRHSSHLISYFEGINGVPKIKNGYN
PATWMLEVTSEAQEEALGINFAELYKNSDLYRGNKALISELSTPPSASKDLYFTTKYSQSFITQCKACLWKQNL
SYWRNPPYSAVRFLFTTFIALLFGTIFWDIGSKRKRAQDVFNAMGSMYAAVLFIGVQNATSVQPVVAIERTVFY
REKAAGMYSALPYAFGQVAIEIPYILLQTLVYGVIVYAMIGFEWTAMKFFWYLFFMFFTFLYFTLYGMMAVG
ATPDHHVAGIVSFGFYLIWNLFSGFVIPRTRMPVWWRWYFWICPVSWTMYGLVTSQFGDVTERIDTGETVGD
FVKRYFGYRDDFIGIAAAVVVGFALLFGFTFAFSIKAFNFQKR

>XP_016169934.1_PDR1_Ai
MEGTDIYRASNSLRRSSSVWRNSGVEVFSRSSREENDEEALKWAALEKLPTYNRLRKGLLTASQGPASEVDVT
DLGYQERQKLLERLVKVAEEDNEKFLRKLKQRIDRVGLEIPTVEVRYEHLNIDAEAFVGSRALPSFINSVFNVV
EGFLNIIHVLPSKKKHVTILKNVSGIVKPQRMTLLLGPPGSGKTTLLLALSGKLDKGLQVAGKVTYNGHEMNEF
VPQRTAAYISQHDVHIGEMTVRETLAFSARVQGVGSRYDMLSELARREKAANIKPDPDIDVYMKAVSTAGQE
SSIATDYTLKILGLDICADTMVGDEMLRGISGGQRKRVTTGEMLVGPTNALFMDEISTGLDSSTTFQIVSSLRQY
VHIMNGTAVISLLQPAPETYELFDDIILISDGQVVYHGAREYVLDFFESMGFRCPERKGVADFLQEVTSKKDQA
QYWSRRDEPYRFVTVTQFAEAFQSFHVGRKLNEEIAVPFDKSKSHPAALTTKKFGLNKKELLKANFAREYLLM
KRNSFVYVFKLVQLTIMALIAMTLFFRTKMHHRNQDDAGVYAGALFFTLITIMFNGMAEISMSIAKLPVFYKQ
RDLLFYPSWAYAIPSWILKIPVTIVEVGVWVFLTYYVIGFDPNVGRLFKQYLLLLFVHQLASGLFRAIAALGRN
MIVANTFGSFAILAFLSLGGFLLSRKNIKDWWIWGYWCSPLMYGQTGLMVNEFLGHSWNKSSNPNFGVTFLES
RGFFTDSYWYWIGLAACVGYVFLFNGVFAAALNILGPFDKPQPTITEESEDDAQEVELPRIENSRSAEDAVVES
SHGKKKGMVLPFEPYAITFDEVVYSVDMPQEMKEQGVQEDKLVLLKGVSGAFRPGVLTALMGVSGAGKTTL
MDVLAGRKTGGYIEGSIKVSGYPKKQETFARVSGYCEQNDIHSPHVTVYESLLYSAWLRLSSSVDSKTRKMFIE
EVMDLVELTPLRNSLVGLPGVSGLSTEQRKRLTIAVELVANPSIIFMDEPTSGLDARAAAIVMRTVRNTVDTGR
TVVCTIHQPSIDIFEAFDELFLMKRGGQEIYVGPLGHHSSHLIKYFESIEGVSKIKDGYNPATWMLEVTTTAQEL
NLGVDFTDLYKNSDLYRRNKQLIQELGQPAPGSKDLHFPTQFSQSFLVQCQACLWKQRLSYWRNPPYTAVRFF
FTTFIALMIGSMFWGLGDKTKKRQDLFNAVGSMYSAVLFLGIQNASSVQPVVAVERTVFYREKAAGMYSALP
YAFAQILVEIPYLFTQSVTYGLIIYAMLQFDWTAAKFFWYLYFMFFTLCYFTFYGMMAVAVTPNHHVAAIVAA
AFYAIWNLFSGFVVPRPSIPIWWRWYYWACPVAWSLYGLIGSQFGDITTPMDGEGGKNVGQFIHSYFGIKHDFI
GYAAVVVAGIAVLFAFIFASAIKAFNFQKR

>XP_016170247.1_PDR1_Ai
MENDELRVASARIGSSSIWRSSGAVDVFSGSSRRDDDEEQLKWAAIEKLPTYLRLTRGILTESQGEYTEIDINKL
GPLQRKNLVERLVKIAEQDNEKFLLKLRRRIDRVGLDIPTIEVRFEHLNVEAEAHVGSRALPTNLNFCINLLEGF
LNSLCLLPSRKKPFTVLHDVSGIIKPRRMTLLLGPPSSGKTTLLLALAGRLGKDLKFSGRVSYNGHGMEEFVPQ
RTSAYISQTDLHIGELTVRETLAFSARCQGIGMRYDMLAELSRREKAENIKPDPDLDIYMKAAALEGQETNVVT
DYIMKILGLEICADTMVGDEMIRGISGGQKKRVTTGEMLVGPARALFMDEISTGLDTSTTFQMVNSLRQSIHIL
NGTAVISLLQPAPETYELFDDVILLSDGQIVYQGPRENVLEFFEYMGFKCPERKGVADFLQEVTSRKDQEQYW
ANKDEPYTFISVREFADAFQSFHIGRKLGDELATPFDKTKGHPAVLTKNKYGVSKKELLRACVSREFLLMKRN

SFVYIFKMWQLILTGLITMTLFLRTEMHRNTVSDGGIYMGALFFVLIVIMFNGFSELSMTIAKLPVFYKQRDLLF
YPSWAYSLPTWILKIPITLVEVGIWVVMTYYVIGFDPSFERFIKQYFLLACINQMASALFRFMGAVGRNLIVANT
FGSFALLAVMVMGGFILSRVDVRKWWLWGYWISPMMYGQNAIAVNEFLGNSWKHVPPNSTEPLGVKVLKA
RGIFPEAHWYWIGVGASIGYMLLFNLLFPLALHFLDPFGKPQALISEEALAERNSVRNDHIIELSSGRSGASDKS
NGRSVSSRTLSARVGAINGTDRNRKRGMVLPFTPLSITFDEIRYTVDMPQEMKAQGIVEDKLELLKGISGAFRP
GVLTALMGVSGAGKTTLMDVLSGRKTAGYIQGQITISGYPKKQETFARISGYCEQTDIHSPHVTVYESLVYSA
WLRLPPEVDSSTREMFIEEVMELVELNSLRDALVGLPGVNGLSTEQRKRLTIAVELVANPSIIFMDEPTSGLDAR
AAAIVMRTVRNTVNTGRTVVCTIHQPSIDIFDAFDELLLLKRGGEEIYVGPLGRHSSHLISYFEGINGVPKIKNGY
NPATWMLEVTSEAQEEALGINFAELYKNSDLYRGNKALISELSTPPSASKDLYFTTKYSQSFITQCKACLWKQN
LSYWRNPPYSAVRFLFTAFIALLFGTIFWDIGSKRKRAQDVFNAMGSMYAAVLFIGVQNATSVQPVVAIERTVF
YREKAAGMYSALPYAFGQVAIEIPYILLQTLVYGVIVYAMIGFEWTAMKFFWYLFFMFFTFLYFTLYGMMAV
GATPDHHVAGIVSFGFYLIWNLFSGFVIPRTRMPVWWRWYFWICPVSWTMYGLVTSQFGDVTERIDTGETVG
DFVKSYFGYRDDFIGIAAAVVVGFALLFGFTFAFSIKAFNFQKR

>XP_016457560.1_P_PDR1-l_Nt
MEGGEDIFRVSSARLSSSNVWRNSAMDVFSRSSREADDEEALKWAALEKLPTYLRIRRGILTEEEGQSREVDIT
KLDLVERRNLLERLVKIADEDNEKFLLKLKKRIDRVGLDLPTIEVRFEHVSVDAEARVGSRALPTIFNFTVNILE
DFLNYLHILPSRKKPLPILHEVSGIIKPGRMTLLLGPPSSGKTTLLLALAGKLDKDLKVSGRVTYNGHGMDEFVP
QRSSAYISQNDLHIGEMTVRETLAFSARCQGVGAKYEILAELSRREKEANIKPDPDVDIFMKSAWNEGQEANV
VTDYTLKILGLEICADTLVGDEMIRGISGGQRKRLTTGEMMVGPARALFMDEISTGLDSSTTYQIVNSIRQSIHIL
QGTAVISLLQPAPETYDLFDDIILLSDGQIVYQGPRENVLEFFEYMGFMCPERKGVADFLQEVTSRKDQEQYRA
CRDEPYNFITVREFSESFQSFHIGRKLGDELAVPFDKSKSHPAALTTKRYGVSKKELLKACTAREYLLMKRNSF
VYIFKMIQLTLMASITMTLFLQTEMHRNTTTDGAVFLGALFYAVIMIMFNGFSELALSIMKLPSFYKQRDLLFFP
AWAYALPTWILKIPVTLVEVAIWVCMTYYVIGFEADVGRFFKQLFLLICVNQMASGLFRFIGALGRNVIVANTF
GSCALLTVIVMGGFILSRDDVKKWWIWGYWISPMMYAQNAIAVNEFLGKSWAHVPPNSTGTETLGVSFLKSR
GIFPEARWYWIGAGALLGYVLLFNFLFTVALAYLNPFGKSQAVLSEETVAERNASKRGEVIKLSPIEKHSSERG
NDVRRSASSRSMSSRVGSITEADLNKRRGMILPFEPLSITFDDIRYAVDMPQEMKAQGVAEDRLELLKGVSGAF
RPGVLTALMGVSGAGKTTLMDVLAGRKTGGYIDGTISISGYPKQQETFARIAGYCEQTDIHSPHVTVYESLQFS
AWLRLPHEVDTETRKMFVEEVMELVELTPLREALVGLPGVNGLSTEQRKRLTVAVELVANPSIIFMDEPTSGL
DARAAAIVMRTVRNTVDTGRTVVCTIHQPSIDIFDAFDELLLLKRGGEEIYVGPLGRHSSHLIKYFEGIDGVPKI
KDGYNPATWMLEITSVAQEAARGIDFTELYKNSELYRRNKALIKELSVPAPCSKDLYFPTKYSQSFFTQCMACF
WKQRWSYWRNPPYTAVRLMFTFFIALMFGTIFWDLGSRRKRQQDLLNAIGSMYVAVLFLGVQNATSVQPVIA
IERTVFYRERAAGMYSALPYAFGQVMIELPYLFIQTIIYGVIVYVMIGFEWTVAKFFWYLFFMYFTLLYFTLYG
MMTVAVTPNHSIAAIISSAFYAIWNLFCGFVVPKTRMPVWWRWYYYICPISWTLYGLIASQFGDIQDKLDTNE
TVEEFIESFFDFKYDFVGYVAVILVGISVVFLFIFAFSIKAFNFQKR

>XP_016492264.1_P_PDR1-l_Nt
MEGGEDSFRVSSARLSSSNVWRNSAMDVFSRSSREADDEEALKWAALEKLPTYLRIRRGILTEEEGQSREVDIT
KLDLVERRNLLERLVKIADEDNEKFLLKLKKRIARVGLDLPTIEVRFEHVSVDAEARVGSRALPTIFNFTVNILE
DFLNYLHILPSRKKPLPILHEVSGIIKPGRMTLLLGPPSSGKTTLLLALAGKLDKDLKVSGRVTYNGHGMDEFVP
QRSSAYISQNDLHIGEMTVRETLAFSARCQGVGAKYEILAELSRREKGANIKPDPDVDIFMKSAWNEGQEANV
VTDYTLKILGLEICADTLVGDEMIRGISGGQRKRLTTGEMMVGPARALFMDEISTGLDSSTTYQIVNSIRQSIHIL
QGTAVISLLQPAPETYDLFDDIILLSDGQIVYQGPRENVLEFFEYMGFMCPERKGVADFLQEVTSRKDQEQYW
ARRDEPYKYITVREFSESFQSFHIGRKLGDELAVPFDKSKSHPAALTTKKYGISKKELLKACTAREYLLMKRNS
FVYIFKMIQLTLMASITMTLFLRTEMHRNTTTDGAVFLGALFYAVIMIMFNGFSELALSIMKLPSFYKQRDLLFF
PAWAYALPTWILKIPVTLVEVAIWVCMTYYVIGFEADVGRFFKQLFLLICVNQMASGLFRFIGALGRNVIVANT
FGSCALLTVLVMGGFILSRDDVKKWWIWGYWISPMMYAQNAIAVNEFLGKSWAHVPPNSTGTETLGVSFLKS
RGIFPEARWYWIGAGALLGYVLLFNFMFTVALAYLNPFGKSQAVLSEETVAERNASKRGEVIELSPIEKRSSER
GNDVRRSASSRSMSSRVGSITEADLNKRRGMILPFEPLSITFDDIRYAVDMPQEMKAQGVAEDRLELLKGVSG
AFRPGVLTALMGVSGAGKTTLMDVLAGRKTGGYIDGTISISGYPKQQETFARIAGYCEQTDIHSPHVTVYESLQ
FSALLRLPREVDTETRKMFVEEVMELVELTPLREALVGLPGVNGLSTEQRKRLTVAVELVANPSIIFMDEPTSG
LDARAAAIVMRTVRNTVDTGRTVVCTIHQPSIDIFDAFDELLLLKRGGEEIYVGPLGRHSSHLIKYFEGIDGVPKI
KDGYNPATWMLEITSVAQEAARVIDFTELYKNSELYRRNKALIKELSVPPPCSKDLYFPTKYSQSFFTQCKACF
WKQHWSYWRNPPYTAVRLMFTFFIALMFGTIFWDLGSRRKRQQDLLNAIGSMYAAVLFLGVQNATSVQPVIA

IERTVFYRERAAGMYSALPYAFGQVMIELPYLFIQTIIYGVIVYVMIGFEWTVAKFFWYLFFMYFTLLYFTLYG
MMTVAVTPNHSIAAIISSAFYAIWNLFCGFVVPKTRMPVWWRWYYYICPVSWTLYGLIASQFGDLQHILDTNE
TVEQFIENFFDFKYDFVGYVAVILVGISVVFLFIFAYSIKAFNFQKR

>XP_016537945.1_P_PDR1-l_Can
MPNWRKMEGGEDIFRVSSARLSSSNVWRNSAMDLFSRSSKEDYDDEEALKWAAIEKLPTYLRIRRGILTEDEG
QCREVDITKLDLVERRNLLERLVKIADEDNEKFLLKLKKRIDRVGLDLPTIEVRFEHLGVDAEARVGSRALPTIF
NFTVNILEDFLNYLHILPSRKKPLPVLHDVSGIIKPGRMTLLLGPPSSGKTTLLLGLAGKLDKDLKVSGRVTYNG
HGMDEFVPQRTSAYISQNDLHIGEMTVRETLAFSARCQGVGAKYEILAELARREKEANIKPDPDVDIFMKSAW
NEGQEANVVTDYTLKILGLEICADTLVGDEMIRGISGGQRKRLTTGEIMVGPARALFMDEISTGLDSSTTYQIV
NSIRQSIHILQGTAVISLLQPAPETYDLFDDIVLLSDGQIVYQGPRENVLEFFEYMGFKCPERKGVADFLQEVTSR
KDQEQYWARRDETYKFISVREFSEAFQSFHVGRKLGDELAVPFDKSKSHPAALTTKRYGVSKKELLKACTARE
YLLMKRNSFVYIFKIIQLTLMASITMTLFLRTEMHRNTIVDGAVFLGALYYAVIMIMFNGFSELALSIMKLPSFY
KQRDLLFFPAWAYALPTWILKIPITLVEVAIWVCMTYYVIGFEADTGRFFKQLFLLICLNQMASGLFRFIAALGR
NVIIANTFGSCALLIVLVMGGFILSRDNVKQWLIWGYWISPMMYAQNAIAVNEFLGKSWAHVPPNSTGTETLG
VSFLKSRGIFPEARWYWIGAGALFGYVLLFNLLFTVALAYLNPFGKPQAVLSEESVAERNASKRGEVIELSPIGK
SSSERGNDVRRSASSRSMSSRVGSITETDLNKRRGMILPFEPLSITFDDIRYAVDMPQEMKAQGVGEDQLELLK
GVSGAFRSGVLTALMGVSGAGKTTLMDVLAGRKTGGYIEGTISISGYPKKQETFARIAGYCEQTDIHSPHVTVY
ESLQYSAWLRLPREVDTETRKMFIEEVMELEELTPLREALVGLPGVNGLSTEQRKRLTVAVELVANPSIIFMDE
PTSGLDARAAAIVMRTVRNTVDTGRTVVCTIHQPSIDIFDAFDELLLLKRGGEEIYVGPLGRHSSNLIKYFEGID
GVAKIKDGYNPATWMLEITSVAQEAALGIDFTDMYKNSEMYRRNKALIKELSVPVPGSKDLYFQTKYSQSFFT
QCMACFWKQHWSYWRNPPYTAVRLMFTFFIALMFGTIFWDLGSKRRRQQDILNAIGSMYAAVLFLGVQNAT
SVQPVVAIERTVFYNLMLLGSYSYSSTEYFFFMQVMIELPYLLIQTIIYGVIVYAMIGFEWTVTKFFWYLFFMYF
TLLYFTLYGMMTVAVTPNHSIAAIISSAFFAVWNLFCGFIIPKTRMPVWWRWYYYICPISWTLYGLIASQFGDL
QDKLDTNETVEEFMETFFDFKYDFVGYVAVILVGISVTFLFIFAYSIKAFNFQKR

>XP_016566101.1_P_PDR1_Can
MEPANLSNLRGSSLRGSISGSSMRGSIRANSNNSIWRNTGLEVFSRSGRDEDDEEALKWAALEKLPTFDRLRKG
ILFGSQGTSGTEIDVDDLGFQQRKNLLDRLVKVADEDNEKFLLKLKNRIDRVGIDLPTIEVRYENLNIEADAYV
GSRGLPTFINFMTNYLEALLNTLHILPSSKRRVTILKDISGIIKPCRMTLLLGPPSSGKTTLLLALAGKLDKALKV
TGKVSYNGHELHEFVPQRTAAYISQHDLHIGEMTVRETLEFSARCQGVGSRYEMLAELSRREKAANIKPDPDID
IYMKASATEGQEANVVTDYVLKILGLDICADTMVGDEMLRGISGGQKKRVTTGEMLVGPSKALFMDEISTGL
DSSTTYSIVNSLRQSVQILKGTAVISLLQPAPETYNLFDDIILISDGYIVYQGPRDDVLEFFESMGFKCPDRKGVA
DFLQEVTSKKDQQQYWCRRNEPYRFITSKEFSDAYQSFHVGRKLGDEIATPFDRSKCHPAALTNEKYGIGKKD
LLKVCTEREFLLMKRNSFVYIFKFSQLTIMALITMTLFFRTEMPRDTVDDGGIYAGALFFVVVMIMFNGMSEM
AMTIIKLPVFYKQRDLLFFPSWAYALPSWILKIPVTLVEVGLWVILTYYVIGFDPNVTRFLKQFLILILVNQMAS
GLFRFMGAMGRTMGVAMTFGSFALLLQFALGGFVLSRDDVKSWWIWGYWISPLMYSMNAVMVNEFDGKK
WKHLAPNGTESLGATVVKSRGFFPDASWYWIGLGALFGFTILFNFCFSLSLAFLNPYGKSQSVLPEDSENAENG
QAASQITSTESGEIISAGQNKKKGMVLPFEPHSITFDEVIYSVDMPQEMKEQGATEDRLVLLKGVSGAFRPGVL
TALMGVSGAGKTTLMDVLAGRKTGGYIDGDIKISGYPKKQETFARISGYCEQNDIHSPYVTVYESLVYSAWLR
LPKDVDEKTRKMFVDEVMELVELEPLRSALVGLPGVNGLSTEQRKRLTIAVELVANPSIIFMDEPTSGLDARAA
AIVMRTVRNTVDTGRTVVCTIHQPSIDIFEAFDELFLMKRGGQEIYVGPLGRHSCHLIKYFESLPGVTKIKEGYN
PATWMLEVTASAQEMMLGVDFTDLYKNSDLYRRNKALISELSVPRPGSKDLYFETQYSQSFLIQCMACLWKQ
HWSYWRNPAYTAVRFIFTTFIALVFGTMFWDLGTKVSKSQDLFNAMGSMYAATLFLGVQNASSVQPVVDIER
TVFYRERAAGMYSAIPYAFGQVFIEIPYVLVQSVFYGIIVYAMIGFEWDAGKFFWYLFIMFTTLLYFTYYGMMS
VAVTPNQNVASIVAAFFYAIWNLFSGFIVPRPRMPVWWRWYYWACPVAWTLYGLVASQFGDIKTALTDDET
VEQFLRRYFGFRHDFVGVVAGVLVAYVLVFAFTFAFAIKAFNFQRR

>XP_016573617.1_P_PDR1-l_Can
MEPVNLNNMRGSSMRGSIRGSSMRGSLRASTNNSIWRNNGVEVFSRSARDEDDEEALKWAALEKLPTFDRLR
KGLLFGSQSGGAINEVDINDLGYQQRKSLLERLVKVADEDNEKFLMKLKNRVDRVGIDLPSIEVRYEHLNIEA
DAYAGSRALPTFLNFMTNFVETLLNSLHILPNKKRQITILKDVSGIIKPCRMTLLLGPPSSGKTTLLLALAGKLDP
ALKVTGKVTYNGHELHEFVPQRTAVYISQHDLHIGEMTVRETLEFSARCQGVGSRFEMLAELSRREKAANIKP
DPDIDIYMKAAATEGQEANVVTDYVLKILGLDICADTMVGDEMIRGISGGQKKRVTTGEMLVGPSKALFMDEI

STGLDSSTTFSIVNSLRQTVQLLKGTAVISLLQPAPETYNLFDDIILLSDGYIVYQGPREDVLDFFESMGFKCPER
KGAADFLQEVTSKKDQQQYWAKRNEPYRFVTSKEFAEAYQSFHVGRKLSAELATPYDKSKSHPAALSTQKYG
IGTKQLLKVCAEREFLLMKRNSFVYIFKLSQLAVMALITMSVFFRTKLPRDDMDDGGIYAGALFFVVVMIMFN
GMAEIALTIFKLPVYFKQRDLLFYPSWAYALPTWILKIPITFVECGMWTFLTYYVMGFDPNVSRLFKQFLLLVL
VHQMASALFRFIGAMGRTMGVASTFGAFALLLQFALGGFVLAREDVKKWWIWGYWTSPLMYSVNSILVNEF
DGKNWKHIAPNGTEPLGAAVVRARGFFPDAYWYWIGCGALLGFTMVFNIFYSISLAYLDPFGKPQAMISEDDE
IADNVELTERSETEGQDKKKGMVLPFEPHSITFDNVVYSVDMPQEMKEQGSAEDRLVLLKGVSGAFRPGVLTA
LMGVSGAGKTTLMDVLAGRKTGGYIDGDIKISGYPKKQETFARISGYCEQNDIHSPYVTVYESLVYSAWLRLP
QDVDENKRKMFVDEVMELVELTPLRSALVGLPGVNGLSTEQRKRLTIAVELVANPSIIFMDEPTSGLDARAAAI
VMRAVRNTVDTGRTVVCTIHQPSIDIFEAFDELFLMKRGGQEIYVGPLGRHSCHLIKYFESMHGVSKIKEAYNP
ATWMLEVTASSQEMMLGVDFADLYKNSDLYKRNKALIAELSTPRPGTKDLHFETQFSQPFWTQCMACLWKQ
HWSYWRNPAYTAVRFIFTTFIAIVFGTMFWDLGTKVSRSQDLINAMGSMYAATLFLGVQNSSSVQPVVAVERT
VFYREKAAGMYSAIPYAFGQVFIEIPYVFVQSVFYGVIVYSMIGFEWTAAKFFWYFFFMYCTLLYFTFYGMMT
VAITPNQNVASIVAAFFYAAWNLFSGFIVPRPRIPIWWRWYYWACPVAWTLYGLVASQFGDIQTKLSDDETVE
QYLRRYFGFKHDFLGIVAAVIVALPVMFALTFALSIKAFNFQRR

>XP_016575874.1_P_PDR1-l_Can
MEGGEDLIRVSSARLSGSNIWRNSAMDVFSRSSREDYDDEEALKWAALERLPTYHRIRRGILTEEEGQSREVDI
TRLDLVERRNLLERLVKITDEDNEKFLLKLKKRIDRVGLDLPTIEVRFEHLSVDAEARVGSRALPTIFNFTVNIV
EDFLNYLHILPSRKKPLPILHDVSGIIKPGRMTLLLGPPSSGKTTLLLGLAGKLDKDLKVSGRVTYNGHGMDEF
VPQRTSAYISQNDLHIGEMTVRETLAFSARCQGVGAKYEMLAELSRREKEANIKPDPDVDIFMKSAWNEGQEA
NVITDYTLKILGLEICADTLVGDEMIRGISGGQRKRLTTGEMMVGPARALFMDEISTGLDSSTTYQIVNSIRQSIH
ILQGTAVISLLQPAPETYDLFDDIILLSDGKIVYQGPRENVLEFFECMGFKCPERKGVADFLQEVTSRKDQEQY
WTRRDEPYRFITVSEFSEAFQSFDVGRQLGDELAVPFDKSTSHPAALTTKSYGISKKELLKACTAREYLLMKRN
SFVYIFKMIQLTLMASIAMTLFLRTEMHRNTTTDGAVFLGALFYAVITIMFNGFSELALSILKLPSFYKQRDLLFF
PAWAYALPTWILKIPVTLVEIAIWVCMTYYVIGFDADVERFFKQLFLLVCLNQMASGLFRFLAALGRNIIVANT
FGSCALLIVLVMGGFILSRDNVKQWLIWGYWISPMMYAQNAIAVNEFLGKSWAHVPPNSTSTDTLGVSFLKSR
GIFPEARWYWIGAGALLGYIFLFNFLFTVALAYLNPFGKPQAILSEETVAERNASKRGMVIELSPVGRSSSERGN
DVRLSASSRSLSSRAGSITEEDLNKRRGMVLPFEPLSITFDDIGYAVDMPQEMKAQGFIEDRLELLKGVSGAFRP
GVLTALMGVSGAGKTTLMDVLAGRKTGGYIEGAISISGYPKKQETFARIAGYCEQTDIHSPHVTVYESLQFSA
WLRLPPEVDTESRKMFIEEVMELVELIPLREALVGLPGVNGLSTEQRKRLTVAVELVANPSIIFMDEPTSGLDAR
AAAIVMRTVRNTVDTGRTVVCTIHQPSIDIFDAFDELLLLKRGGEEIYVGPLGRHSSHLIKYFEGIDGVPKIKNG
YNPATWMLEITSVAQEAALGIDFTELYKNSELYRRNKALIEELSVPAPGSKDLYFQTKYSQSFFTQCMACFWK
QHWSYWRNPPYTAVRLMFTFFVSLMLGTIFWGLGSKRKRQQDIFNAVGSMYAAILFLGIINATSVQPVVAIER
TVFYRERAAGMYSALPYAFGQVMIELPHLFIQTILYGVVVYAMIGFEWTFIKFFWYLFFMYFTLLYFTLYGMM
TVAVTPNHTIASVVSSAFYTIWNLFCGFVVPKTRMPVWWRWYYYLCPVAWTFYGLITSQFGDLQDRLDTNET
VEEFLESFFDFKHDFLGYVAVILVGISVLFLFIFAYSIKAFNFQKR

>XP_016702966.1_P_PDR1-l_Gh
MESGTDAFGVSSARIGSSSICRNNVREAFSMSSHEEDDENALKWAAIQKLPTYLRVRRGILTEQDGQSREIDITN
LGFVERRNLLERLVRIAEEDNERFLLKLKERIDRVGLDMPTIEVRFEHLNVEAEAYVGNRALPTMFNFSVNIVE
GLLSNLHILPSRKKPFPILNDVSGVIKPRRMTLLLGPPSSGKTTLLLALAGKLGKDLKFSGRVTYNGYGMEEFVP
QRTSAYISQYDLHIGEMTVRETLAFSARCQGVGPRYEMLAELSRREKEANIKPDPDIDIYMKAAALEGQEAGV
VTDYILKILGLEVCADTMVGDEMIRGISGGQKKRVTTGEMLVGPARALFMDEISTGLDSSTTFQIVNSLRQSIHI
LNGTALISLLQPAPETYQLFDDVILLSDGQIVYQGPRENVLEFFKYMGFKCPKRKGVADFLQEVTSKKDQEQY
WTRIDEPYNFISVKEFAEAFQSFHIGQKLGDDLAIPFDKSKSHPDALSKDKYGVPKKELLKACLSRELLLMKRN
LFVYVFKMFQLIFIGFITVTIFLRIEMHPDTTTDGGIFMGALFFILVTVMFNGFAELTLTILKLPVFYKQRDLLFYP
SWAYSLPTWILKIPISILDATLWVLMSYYVIGFDPNVGRFFKQYLLLLCLSQMASALFRFMGGLGRNIIVANTC
GSFAMLAVLVMGGFVLTRYAVKKWWIWGYWISPLMYGQNAIAVNEFLGKSWRQVPPNSTEPLGVLILKSRGI
FPEARWYWIGVGALIGYCFLFNFLFTLALKYLDPFGKPQAVISKETLAEKIASKAREKVDLSSRGEGSSGGGNE
SQRSVSFRSLSAKVGSVNDANQSRKRGMVLPFEPLSMSFDEIRYAVDMPQEMKAQGISEDRLELLKGICGAFRP
GVLTALMGISGAGKTTLMDVLAGRKSGGYVKGTIKISGYPKKQETFARISGYCEQTDIHSPHVTVYESLLFSAW
LRLPPEVNPETKTMFIEEVMELVELTSLREALVGLPGVNGLSTEARKRLTIAVELVANPSIIFMDEPTSGLDARA
AAVVMRTVRNTVDTGRTVVCTIHQPSIDIFDAFDELLLLKRGGEEIYMGPLGHHSCYLIKYFEEINGIPKIKDGY

NPATWMLEITSAAQEEALGVNFADIYKNSELYRRNKALVKELSSPAPGSKDLYFQTRYSQSLLTQCMACLWK
QYWSYWRKPPYNAVRFLFTTVIGLLFGTIFWDIGSKRTREQDVFNSMGSMYAAVLFIGFQNCASVQPVVAVER
TVFYRERATGMYSALPYAFGQVVIELPYVLVQTAIYGVIVYAMIGFQWTAAKFFWYLFFMYFTFLYFTFYGM
MAVAVTPNHNIAAIVSSAFFAIWNLFSGFIIPRTRIPIWWRWYYWACPVSWTIYGLIASQYGDINEKFDSGETVE
HFVRNYFDFRNEFVEIVAMVVVGICVLFGSIFAVSFKAFNFQKR

>XP_016742277.1_P_PDR1-l_Gh
MENVEAFRVGSARIGSSSIWRNNAMEAFSMSAREEDDEEDLKWAAIEKLPTYLRVRRGIFTEGEGQSREIDIEN
LGFIERRNLLERLVRIAEDDNERFLLKLKERIDRVGLDMPTIEVRFEHLNVEAEAYVGSRALPTIFNFSANILEGL
LSYLHILPNRKKPLPILNDISGIIRPRRMTLLLGPPSSGKTTLLLSLAGKLGKDLKFAGRVTYNGHGMEEFVPQRT
SAYISQYDVHIGEMTVRETLAFSARCQGVGPRYEMLKELSRREKEANIKPDPDIDIYMKAAALEGQEANVVTD
YILKILGLEVCADTFVGDEMRRGISGGQKKRVTTGEMLVGPARALFMDEISTGLDTSTTFHIVNSLRQSIHILNG
TALISLLQPAPETYDLFDDIILLSDGDVVYQGPRENVLEFFEYMGFQCPERKGVADFLQEVTSRKDQEQYWAR
KDEPYSFISVKELAEAFQSFHIGQKLGDDLAVPFDKSKSHPAALTKDKYGVSKKELLKACVSREYLLMKRNLF
VYVFKMIQLIFIGVITMTIFIRTEMHRDTITDGGIFMGALFFILIMIMFNGFAELAMTILKLPVFYKQRDLLFYPSW
AYSLPALILKTPISILEVTVWVFMSYYVIGFDPDVGSFFKNYLVLLCLSQMASGLFRLMGGLGRNIIVANTCGSF
ALLTVLVMGGFILTRDDVKKWWKWGYWISPLMYAQNAITVNEFLGKSWRHVPPNSTEPLGVLVMKSRGIFPE
PHWYWIGVGALIGYCFLFNFLFTLALKYLDPFGKPQAVISKETLAERIASKTGGNIELSSRGRDSSERRTGASSR
SLSWKVTSVNEANQKRKRGMVLPFEPLSMSFDEIKYAVDMPQEMKAQGISEERLELLKGVSGAFRPGVLTAL
MGVSGAGKTTLMDVLAGRKTGGYVEGTIKISGYPKKQETFARISGYCEQTDIHSPHVTVYESLVFSAWLRLPP
EVNSETRMMFIEEVMELVELSSLRDALVGLPGVNGLSTEQRKRLTIAVELVANPSIIFMDEPTSGLDARAAAIV
MRTVRNTVDTGRTVVCTIHQPSIDIFDAFDELLLLKRGGEEIYVGPLGRHSCHLIKYFEEINGIPKIKDGYNPAT
WMLEVTSAPQEEAIGVNFTNIYKNSELYRRNKALVKELSNPAPGSKDLYFQTRYSQSLLTQCIACLWKQYWSY
WRNPPYTAVRFLFTTFIALMFGTIFWDLGSKRTRRQDVFNSMGSMYAAVLFIGFQNAASVQPVVAVERTVFYR
ERAAGMYSALPYAFGQVVIELPYILVQTVIYGIIVYAMIGFEWASHKFFWYLFFMYFTFLYFTFYGMMTVAVT
PDHNIAGIISSAFFALWNLFSGFIIPRTRIPVWWRWYYWVCPISWSLYGLIASQYGDVQENFESGETVQHFVRNY
FDFREEFVGVVAIVVVGICVLFGFIFAFSIKAFNFQKR

>XP_017227475.1_P_PDR1-l_Dc
MNMEGSDIFRVSSARISSSNIWRSSGRDIFSRTSVEQDDEEALTWAAIEKLPTYLRIRRGLLTEKEGEAREIDIKS
LGIAEKRSLLERLVKVAEEDNERFLLKLKERIDRVGLEIPAIEVRFEHLSVDAEAYVGGRALPTIFNFSANILEDT
LNYLHLLPSRKKPLPILHDVSGIIKPGRMALLLGPPSSGKTTLLLALAGKLGSDLQVSGRVTYNGAGMDEFVPQ
RTSAYISQHDLHIGEMTVRETLAFSARCQGVGARYETLLELSRREKEANIKPDPDIDIYMKATSLEGQESSVVTD
YTLKILGLEACADTIVGDEMFRGISGGQKKRLTTGEMMVGPAKALFMDEISTGLDSSTTFQIVNSIRQSIHILQG
TAVISLLQPAPETYDLFDDIILLSDGHIVYQGPCENVLEFFESMGFVCPERKGVADFLQEVTSRKDQEQYWLHK
NQPYHFISARELAEAFQSFHVGVKLGDNLAIPFDKAKGHPAALTTEKYGVSKKELLKACIAREFLLMRRNSFV
YTFKMTQMIFVGSIAMTVFLRTEMAKRTLDDGQIYLGALFFGIITLMFNGFSELALSIIKLPVFFKQRDLLFFPA
WAYSLPTWILKIPITIVEALVWVCMTYYVMGFEPDAGRFFKQYLLLVVINQMASGLFRSIGALGRNIIVANTFG
SCALLTVLVLGGFVMSRNDIKAWWIWGYWFSPLMYAQNAVAVNEFLGKRWAHVLPNATEPLGVSVMKARG
LFPQAYWYWIGVGALVGYVFLFNFIFTLALTYLNPFGKSRAVLTEEILAERSGQYIELSSKDNLEKGNQDRRSV
SSRSMSARVGIINEDNQKKHGMVLPFQPLSITFDDISYRVDMPQEMKAQGVTEERLELLKGVSGAFRPGVLTAL
MGVSGAGKTTLMDVLAGRKTGGYIEGTITISGYPKKQETFARIAGYCEQTDIHSPHVTVYESLQYSAWLRLPPE
VDATTKQMFVEEVMELVELSPLREALVGLPGIDGLSTEQRKRLTIAVELVANPSIIFMDEPTSGLDARAAAIVM
RTVRNTVDTGRTVVCTIHQPSIDIFDAFDELLLLKRGGEEIYVGPLGRLSCHLIEYFEGINGVNRIKDGQNPATW
MLDITSESREATKGVDFAQVYKKSELYRRNKELIKEASTPIPGSKDLYFPTQYSQSFYTQCMACLWKQNWSYW
RNPPYNAVRFLFTVFIALLFGTIFWNVGSSRGTKQNLFDSMGSMYAAVIFLGIQSASSVQPVVGVERTVFYRER
AAGMYSALPYAVGQVLIELPYSFIQTILYGLIVYAMLGFEWTFVKFFWHLFFMYFTLLYFTFYGMMTVAVTPN
HNIAAIVSSAFYALWNLFSGFIVPKTRIPIWWRWYYYICPIAWTLYGLVASQFGDLQDELDTGETVQHFIEDYF
GFDYDFVGYVAIIISGFAVLFGFIFAYSIRTFNFQNR

>XP_017407416.1_P_PDR1-l_Va
MEGSDIYRASNSLRASSSTAWRRSIMEGFSRSSHHEEGIDEEALKWAALEKLPTYNRLKKGLLTTSRGVANEID
ITELGFRERQKLLDRLINVAEEDNERFLLKLKGRIDRVGIDIPTIEVRYEHLNVEAETYVGSRALPTFVNFVTNM
VESVFTSLHILSGKKKHVTILKDVSGIIKPRRMTLLLGPPSSGKTTLLLALSGKLDPNLKASGRVTYNGHGFDEF

VPQRTAAYISQNDVHVGEMTVRETLAFSARCQGVGTRYDLLSELARREKEAKIKPDPDIDVYMKAAVTGGQE
ASLVTDYVLKILGLDICADTMMGDEMLRGISGGQRKRVTTGEMLVGPANALFMDEISTGLDSSTTFQIVKCLR
QYVHILDGTAVISLLQPAPETYELFDDIILISDGQIVYQGPREHVLEFFESVGFQCPERKGVADFLQEVTSRKDQE
QYWMHRDEPYRFVTVTKFTEAFQSFHVGRRIGEELATPFDKSKSHPAALTTKRYGVNKKDLVKANISRELLLM
KRNSFVYIFKLFQLTILAILTMTMFLRTEMHRDNLGDGGVYTGALFFAVVILMFNGLAEISMTIVKLPIFYKQRD
LLFYPSWAYAIPSWILKIPITFLEAAVWVFLTYYVIGFDPNVSRFLKQYLVLLLINQMSSGLFRAIAALGRNMIV
ANTFGSFALLILFALGGFILSRNDIKDWWIWGYWISPLMYGQNAIVVNEFLGNSWNHFTPNSNKTLGIQILESR
GFFTHAYWYWIGIGALIGFIFLFNIMYTLALTYLNPFDKPQTTITEESEGGVANGRAGEEELTCLESYGSANSVV
SCSRRKNRGMVLPFEPYSITFDQIVYSVDMPQEMKDQGVREDKLVLLKGVSGAFCPGVLTALMGVSGAGKTT
LMDVLAGRKTGGYIEGNIKVSGYPKRQETFARISGYCEQNDIHSPHVTVYESLVYSAWLRLTAEVESKTRKMFI
EEVMELVELNPLRNSLVGLPGVSGLSTEQRKRLTIAVELVANPSIIFMDEPTSGLDARAAAIVMRTVRNTVDTG
RTVVCTIHQPSIDIFEAFDELFLMKRGGQEIYVGPLGRHSSQLIKYFESFEGVSKIKDGYNPATWMLEVTTPAQE
LTLGVDFHEIYKNSELYRRNKQLIAELGNPGHGSKDIHFPTRYAQSLLVQCLACLWKQHWSYWRNPPYTAVR
FLSTTVIAVMFGTMFWDLGGKYSSRQDLFNAMGSMYNAVLFVGIQNSASVQPVVAIGRTVFYRERAAGMYS
AFPYALAQVIIELPYIFVQATTYSVIVYSMMGFEWTLEKFFWYMFFMYFTLCYFTFYGMMTVAVTPNHHVAS
VVAAAFYGIWNLFSGFVIPRPNMPVWWRWYYWACPVAWTIYGLLASQFGDITDVMKLENISVQEFLRSYFGI
RHDFIGVSAIMVSGFAGLFAVIFAVSIKAFNFQKR

>XP_017424314.1_P_PDR1-l_Va
MEGDIYRASNSLRARSSTVWRNSGVEAFSRSSREEDDEEALKWAALEKLPTYNRLRKGLLTASHGAANEIDVT
DLGFQQKQKLLERLVKVAEEDNERFLLKLRERIDRVGLDLPTIEVRYEHLNIDAEAFAGSRALSSFINSVTNVIE
GFLNLLHIVPSKKKHVTILKDVSGIIKPRRMTLLLGPPSSGKTTLLLALSGKLDESLKVSGKVTYNGHELNEFVP
QRTAAYISQHDVHIGEMTVRETLAFSARCQGVGSRYDMLSELSRREKAANIKPDPDLDVYMKATATAGQESSI
VTDYTMKILGLDICADTMVGDEMLRGISGGQRKRVTTGEMLVGPANALFMDEISTGLDSSTTFQIVSSLRQYV
HILNGTAVISLLQPAPETYDLFDDIILISDGQVVYHGPREYILDFFESMGFRCPERKGAADFLQEVTSKKDQAQY
WVRRDQPYRFVTVTQFAEAFQSFHIGRKIGQELAVPFDKTKSHPASLTTKRFGINKTELLKANFSREYLLMKRN
SFVYIFKLCQLFIMALIALTLFFRTEMHHDDIDDAGVYAGAIFFTIMTVMFNGMADISMTIAKLPVFYKQRNLLF
YPSWAYAIPSWILKIPVTLAEVSVWVFMTYYVIGFDPNVGRFFKQFLILFFISQMASGLFRAIAALGRNMIVANT
FGSFAVLTLLALGGFILSKRDIKNWWIWGFWISPLMYGQNALMINEFLGSNWHNATYDLGVAYLDSRAFFTES
YWYWIGFAGLVGFVLVLNGLFAFALEFLGPFDKPQASITEEEPEANNGGTVAEVELPRRESSGRDGSIVESSHG
KKKGMVLPFEPHSITFDEIVYSVDMPQEMKDQGVQEDRLVLLKGVSGAFRPGVLTALMGVSGAGKTTLMDV
LAGRKTGGYIDGTIKISGYPKKQETFARISGYCEQNDIHSPHVTVYESLVYSAWLRLPSSVDSKTRKMFIDEVM
ELVELNPLRNSLVGLPGVSGLSTEQRKRLTIAVELVANPSIIFMDEPTSGLDARAAAIVMRTVRNTVDTGRTVV
CTIHQPSIDIFEAFDELFLMKRGGQEIYVGPLGRHSSHLIKYFESIDGVSKIKDGYNPATWMLEVTTTAQELSLG
VDFTDLYKNSDLYRRNKQLIQELGQPAPGSKDLHFPTQYSQSFLVQCQACLWKQRWSYWRNPPYTSVRFFFT
TFIALMFGTMFWDLGGKHSTRGDLMNAIGSMYTAVLFLGVQNSSSVQPVVAVERSVFYREKAAGMYSALPY
AFSQILVELPYIFAQAVTYGLIVYAMIGFEWTAEKFFWYLFFMYFTLLYFTFYGMMAVGVTPNHHVASIVAAA
FYAVWNLFSGFVVARPSIPIWWRWYYWACPVAWTLYGLIGSQFGDLTEFMSEEGMTVKDFIEHSYGIEHDFIG
VAAVVVAGIAVLFAFIFAVAIKTFNFQKR

>XP_017617058.1_P_PDR1-l_Ga
MENGEAFRVGSARIGSSSIWRNNAMEAFSMSAREEDDEEDLKWAAIEKLPTYLRVRRGIFTEGEGQSREIDIEN
LGFIERRNLLERLVRIAEDDNERFLLKLKERIDRVGLDMPTIEVRFEHLNVEAEAYVGSRALPTIFNFSANILEGL
LSYLHILPNRKKPLPILNDISGIIRPRRMTLLLGPPSSGKTTLLLSLAGKLGKDLKFAGRVTYNGHGMEEFVPQRT
SAYISQYDVHIGEMTVRETLAFSARCQGVGPRYEMLKELSRREKEANIKPDPDIDIYMKAAALEGQEANVVTD
YILKILGLEVCADTFVGDEMRRGISGGQKKRVTTGEMLVGPARALFMDEISTGLDTSTTFHIVNSLRQSIHILNG
TALISLLQPAPESYDLFDDIILLSDGHVVYQGPRENVLEFFEYMGFQCPERKGVADFLQEVTSRKDQEQYWAR
KDEPYSFISVKELAEAFQSFHIGQKLGDDLAVPFDKSKSHPAALTKDKYGVSKKELLKACVSREYLLMKRNLF
VYVFKMIQLIFIGVITMTIFIRTEMHRDTITDGGIFMGALFFILIMIMFNGFAELAMTILKLPVFYKQRDLLFYPSW
AYSLPALILKTPISILEVTVWVFMSYYVIGFDPDVGSFFKNYLVLLCLSQMASGLFRLMGGLGRNIIVANTCGSF
ALLTVLVMGGFILTRDDVKKWWKWGYWISPLMYAQNAITVNEFLGKSWRHVPPNSTEPLGVLVMKSRGIFPE
PHWYWIGVGALIGYCFLFNFLFTLALKYLDPFGKPQAVISKETLAERIASKTGGNIELSSRGRDSSERRTGASSR
SLSWKVTSVNEANQKRKRGMVLPFEPLSMSFDEIKYAVDMPQEMKAQGISEERLELLKGVSGAFRPGVLTAL
MGVSGAGKTTLMDVLAGRKTGGYVEGTIKISGYPKKQETFARISGYCEQTDIHSPHVTVYESLVFSAWLRLPP

EVNSETRMMFIEEVMELVELSSLRDALVGLPGVNGLSTEQRKRLTIAVELVANPSIIFMDEPTSGLDARAAAIV
MRTVRNTVDTGRTVVCTIHQPSIDIFDAFDELLLLKRGGEEIYVGPLGRHSCHLIKYFEEINGIPKIKDGYNPAT
WMLEVTSAPQEEAIGVNFTNIYKNSELYRRNKALVKELSNPAPGSKDLYFQTRYSQSLLTQCIACLWKQYWSY
WRNPPYTAVRFLFTTFIALMFGTIFWDLGSKRTRRQDVFNSMGSMYAAVLFIGFQNAASVQPVVAVERTVFYR
ERAAGMYSALPYAFGQVVIELPYILVQTVIYGIIVYAMIGFEWASHKFFWYLFFMYFTFLYFTFYGMMTVAVT
PDHNIAGIISSAFFALWNLFSGFIIPRTRIPVWWRWYYWVCPISWSLYGLIASQYGDVQENFESGETVQHFVRNY
FDFREEFVGVVAIVVVGICVLFGFIFAFSIKAFNFQKR

>XP_017639259.1_P_PDR1-l_X2_Ga
MEGGDIYKASTSLRRSIRSGSSSIWRNNGIEVFSRSSRDEDDEEALKWAALEKLPTVARLRKGILASSQGGANEI
DVFDIGWQERKALLERLVKVAEEDNEKFLLKLKNRIQRVGIEVPTIEVRFQHLNIDAQAYVGSNALPTVINFVT
NIFESLLVEMGILSSRKKKLTILKDVSGIIKPGRMTLLLGPPSSGKTTLLLALAGKLDSALKCSGTVTYNGHEMN
EFVPQRTAAYISQYDLHIGEMTVRETLAFSARCQGVGDRYDMLAELSRREKQANIKPDPDIDVFMKAAATEGQ
EVNVITDYILKVLGLEVCADTMVGDEMLRGISGGQKKRVTTGEMLVGPAKALFMDEISTGLDSSTTFQIVNSL
KQTVHILNGTAVISLLQPAPETYDLFDDIILLSDGRIVYQGPREHVLSFFESMGFRCPERKGVADFLQEVTSRKD
QMQYWARRDQPYRFVTADEFAEAFQSFHVGLQLEDELRTPFEKAKSHPAALTTKKYGVGKWELLKANILREF
LLMKRNSFVYIFIFMQLSFMAIVSMTLFLRTEMNRDSTVDGGIYMGAIFFGLIMVMFNGMPEISMTIAKLPVFFK
QRDLLFFPAWTYALPRWILKIPITFVEVAIWVFLTYYVIGFDPNVERLFRQYFILLLVNQMSSGLFRFIAASARN
MIVANTFGSFALLILFALSGFILARDDIKSWWIWGYWISPLMYGQSALMVNEFRGNQWSHSLPGSTEPAGIEVL
KSRDFFHEPKWYWIGAGGLAGFVILLNFFFTVALTYLKPFGSSRAVISEQTESNEQTNGIGGSIQLTNNESSSNQI
QEEVQRSISSKSSSVTEATVGAIAIKKKGMVLPFEPHCLTFDNVVYSVDMPQEMKQQGITEDRLVLLKGVSGAF
RPGVLTALMGVSGAGKTTLMDVLAGRKTGGYIDGNITVSGFQKKQETFARISGYCEQNDIHSPHVTVYESLLY
SAWLRLSADVKAETRKMFIEEVMELVELNPLRQALVGLPGVNGLSTEQRKRLTIAVELVANPSIIFMDEPTSGL
DARAAAIVMRTVRNTVDTGRTVVCTIHQPSIDIFEAFDELFLMKRGGEEIYVGPLGHHSKHLIEYFEGIQGVSKI
KEGYNPATWMLEVTTTAQELALGVDFADIYKNSDLHRRNKALIEDLSKPAAGSKELYFPTQYSQSFLIQCAAC
LWKQHWSYWRNPPYTAVRFLFTTVIALVFGTMFWDLGSKMNKGQDLTNAMGSMYAAVLFLGIQNASSVQP
VVAVERTVFYRERAAGMYSAMPYAISQVIIEMPYIFIQAASYGILVYAMIGFEWTAAKFFWYLFFMYFTLLYFT
FYGMMAVAVTPNYHIASIVSAAFYGLWNLFSGFIIPRPSMPVWWRWYYWVCPVAWTLYGLFVSQFGDVETPL
EDGDFIGQTVEQYLRSRYGFRHEFLGVVVAVILGFTVLFASIFTVSIKVFNFQRR

>XP_018435982.1_P_ABCG40_Rs
MEGTSFQKASNSLRRDSSVWKRDSGMEIFSRSSREEDDEEALKWAALEKLPTFDRLRKGILTASHGINEIDIEKL
GFQDTKKLLERLIKVGEDEHEKLLWKLKDRIDRVGIDLPTIEVRFDHLKVEAEVHVGGRALPTFVNFMSNFVD
KLLNSLHLLPNRKKKFTILNDVNGIVKPGRMALLLGPPSSGKTTLLLALAGKLDHELKETGRVSYNGHGMNEF
VPQRAAAYIGQNDVHIGEMTVRETFAYAARFQGVGSRYDMLTELARREKEANIKPDPDIDVFMKATSTAGEE
TNVMTDYILKILGLEVCADTMVGDDMLRGISGGQKKRVTTGEMLVGPSRALFMDEISTGLDSSTTYQIVNSLR
NYVHIFNGTALISLLQPAPETFNLFDDIFLIAEGEIIYEGPREHVVEFFETMGFKCPPRKGVADFLQEVTSKKDQM
QYWSRPEEPYRFVRVREFAEAFQSFHVGRRLGDELAVPFDKKKSHPAALTTKKFGVGIKELVNTSFSREYLLM
KRNSFVYYFKFGQLLVMAFATMTLFFRTEMQKKTVIDGSLYTGALFFILMMLMFNGMSELSMTIAKLPVFYK
QRDLLFYPAWVYSLPPWLLKIPISFIEAALTTFITYYVIGFDPNIGRLFKQYILLVLMNQMASALFKMMAALGRN
MIVANTFGAFSMLVFFALGGVVLSKDDIKKWWIWGYWISPIMYGQNAIVANEFFGHSWSQAVPNSSDTLGVT
VLKSRGFLPHAYWYWIGTGALLGFVVLFNFGFTLALTYLNSLGKPQAVLTEDPASNETEELLVVEANANKKR
GMVLPFEPHSITFDNVIYSVDMPQEMIEQGTQEDRLVLLKGVNGAFRPGVLTALMGVSGAGKTTLMDVLAGR
KTGGYIDGNITISGYPKNQQTFARISGYCEQTDIHSPHVTVYESLVYSAWLRLPKEVDSNTRKMFIDEVMELVE
LTPLRQALVGLPGESGLSTEQRKRLTIAVELVANPSIIFMDEPTSGLDARAAAIVMRTVRNTVDTGRTVVCTIHQ
PSIDIFEAFDELFLLKRGGEEIYVGPLGHESSHLINYFESIQGISKITEGYNPATWMLEVSTTSQEAALGVDFAQL
YKNSELYKRNKELIKELSQPAPGSKDLYFPTQYSQSFWVQCMASLWKQHWSYWRNPPYTAVRFLFTIGIALMF
GTMFWDLGGKTRTQQDLSNAMGSMYTAVLFLGLQNAASVQPVVNVERTVFYREQAAGMYSAMPYAFAQVF
IEMPYVLVQAVVYGLIVYAMIGFEWTAAKFFWYLFFMYGSFLTFTFYGMMAVAMTPNHHIASVVSSAFYGIW
NLFSGFLIPRPSMPVWWEWYYWLCPVSWTLYGLITSQFGDITEPMADGTSVKQFIKDFYGFREGFLGVVAAM
NVIFPLAFAIIFAIGIKSFNFQKR

>XP_018715618.1_P_ABCG40_Eg
MEGGDIYRASNSLRATSSTMQMHHATTMDVFSRSSCDEDDEESLRWAALEKLPTFERLRKGILTAGGGANEID
IRNLGFHERKRLIERLVRVTEEDNESFLLKLRNRIDRVGIELPTIEVRFEHLNVEVEAHEGSRALPTVINFCTSILE
GFLNFMHILPRKKYLTILQDVSGIIKPGRMTLLLGPPNSGKTTLLLALASKLDPKLETTGRVTYNGHVMNEFVP
QRTAAYISQHNLHIGEMTVRETLAFSTRCQGVGSRYDMLSELSRREKAANIKPDPDIDIFMKAAATEGQEANV
VTDYILKMLGLEKCADTMVGDEMLRGISGGEQKRVTIGEMLVGPAKVFFMDEISTGLDSSTTYQIVNSLKKFIH
ILDGTAVISLLQPAPETYDLFDDIIILFDGQMVYQGPRELVLDFFESMGFKCPRRKGVADFLQEVTSRKDQHQY
WVRKDEPYTFVTVQEFAEAFQSFHVGRKLGDELSTPFDKSKNHPAALTTKRFGVGMKDLLKAWILREYLLMK
RNSFVYIFKVTQLIIMAFISMTLFLRTKMHRDTVTDGGVYIGALFFTMITIMFNGMAELSMTIAKLPIFYKQRDL
LFYPAWAYALPTWILKIPITFVEVAVWVFITYYVIGYDPNVGRLFKQYLLLVAVNQMASGLFRLIAALGRNLIV
ANTFGSFVLFALFALGGVVLSREEVKKWWIWAYWISPLMYGQNAIVVNELLGSNWNKIPPNSRTNEPLGIQVL
KSRGFFTEAYWYWIGVGALFGFIILFNFGFSVALALLNPFGKSHTVKSDDPEGNKNVNRIEGSIQLQSQGSSLIN
GSGRSSEPTPPWPETAVAANRKRGMVLPFKQHSITFDEITYSVDMPREMKNQGALEDKLVLLEGVSGAFRPGV
LTALMGVSGAGKTTLMDVLAGRKTGGYIKGNITISGYPKNQDTFARISGYCEQNDIHSPHITVYESLIYSAWLR
LPPGVDDQTRKLFVEEVMELVELNPLRQALVGLPGVDGLSIEQRKRLTIAVELVANPSIIFMDEPTSGLDARAA
AIVMRTVRNTVDTGRTVVCTIHQPSIDIFEAFDELFLMKRGGQEIYVGPLGRHSSHLIEYFEGIQGVGKIKDGYN
PATWMLEVTSPTQELALGVDFSDLYKNSDLYRRNNALIEELSIPPPNFKDLYFPTKYSQSTFTQLMACLWKQR
WSYWRNPPYTAVRFLFTIVTALMFGTMFWDLGSKTTRSQDLINAMGSMYGALFLGVQNAASVQPVVAVERT
VFYRERAAGMYSALPYALAQVLIEVPYVLVQAISYGIIVYAMIGFKWTVAKFFWYLFFMCFTLLYFTYYGMM
AVGMTPNHHVASIVSFAFYAIWNLFSGFILPRTRIPIWWRWYYWLCPVSWTLYGLVISQFGDLTNRLEDTGGT
VEEYMRDYFGYRHDFLGVIAVVMVGFTMLFAFVFAISIKILNFQRR

>XP_018716726.1_P_ABCG40_X2_Eg
MDGGDTYRAGNSLRATSSTTWRNHASMVDVFSRSSRDEDDEEALRWAALEKLPTFERLQKGIMTAGGGANQ
IDIWNLGFHDRKRLLERLVRVIEEDNEGFLLKLRNRIDRVGIDLPTIEVRFEHLKVEAEAHEGSRALPTVINFCTS
ILEGFLNFLHVLPSRKKHLTILQDVSGIIKPGRMTLLLGPPSSGKTTLLLALAGKLDPELKMTGRVTYNGHVMN
EFVPQRTAAYISQHDLHIGEMTVRETLAFSARCQGVGSRYDMLLELSRREKAANIKPDPDIDIFMKAAVTEGQE
ANVVTDYILKILGLEICADVMVGDEMLRGISGGQRKRVTTGEMLVGPAEVFLMDEISTGLDSSTTYQIVNFLKQ
FIHILDGTAVISLLQPAPETYDLFDDIILLSDGQIVYQGPRELVLDFFESMGFKCPRRKGVADFLQEVTSRKDQH
QYWVRKDEPYTFVTVREFVEAFQSFHVGRKLGDELSTPFDKSKNHPAALTTKRFGVGMKDLLKACILREYLL
MKRNSFVYIFKLIIMAFISMTLFLRTKMHRDTVTDGGVYIGALFFTVITIMFNGMAEISMTIAKLPIFYKQRDLLF
YPAWAYALPSWILKIPITFVEVAAWVFTTYYVIGYDPNVGRLFKQYLLLVAINQMSSGLFRLIAALGRNLIVAN
TFGTFVLLALVAMGGVVLSREDVNKWWIWGYWTSPLMYGQNAIVVNELLGSNWNKIPPNSSTNEPLGIQVLK
SRGFFTEAYWYWIGLGALFGFIILFNFGFSVALALLNPFGKSHTVKSDDPEGNKNVDRIEGSIQLQSRGSSLRNG
SGRSSKLTPPWPETAVAANTKRGMVLPFEPHSITFDEITYSVDMPREMTNQGAPEDRLVLLKGASGSFRPGVLT
ALMGVSGAGKTTLMDVLAGRKTGGYIKGNITISGYPKNQDTFARISGYCEQNDIHSPHVTVYESLIYSAWLRLP
PGVDDQTRKLFVEEVMELVELNPLRQALVGLPGVDGLSIEQRKRLTIAVELVANPSIIFMDEPTSGLDARAAAI
VMRTVRNTVDTGRTVVCTIHQPSIDIFEAFDELFLMKRGGQEIYVGPLGRHSSHLIEYFEGIQGVSKIKDGYNPA
TWMLEVTSPAQELALGVDFSDLYKNSDLYRRNKALIEELSISPPNSKDLYFPTKYSQSTFTQLMACLWKQHWS
YWRNPPYTAVRFLFTISIALMFGTMFWDLGSKTTRSQDLFNAMGSMYAALVFLGFQNASSVQPVVAVERTVF
YRERAAGMYSALPYALAQVLIEVPYVLVQAISYGIIVYAMIGFKWTVAKFFWYLFFKFFTLLYFTYYGMMAV
GMTPNHHIASIVSLAFYALWNLFSGFVVPRTKIPIWWRWFYWVCPVSWTLYGLVVSQFGDLTNTLEDTGDTV
EEYMRDYFGYRHDFLGVIAAMVIGFTMLFAFVFAISIKILNFQRR

>XP_018716731.1_P__ABCG40_Eg
MDGGDIYRAGNSLRATSSTTWTNHASIADVFSRSSRDEDDEEAPRWAALEELPTFERLQKGILTAGGGANQIDI
WNLGFHDRKRLLERLVXVTEEDNEGFLLKLRNRIDRVGIDLPTIEVRFDHLKVEAEAHEGSRALPTVINFCTSIL
EGFLNFLHVLPRRKKHLTILQDVSGIIKPGRMTLLLGPPSSGKTTLLLALAGKLDPELKTTGRVTYNGHVVNEF
VPQRTAAYISQHDLHIGEMTVRETLAFSARCQGVGSRYAMLSKLSRREKAANIKPDPDIDIFMKAVVTEGQEA
NVVIDYILKILGLEICDDVMVGDEMLRGISGGQRKRVTTGEMLVGPAEVFLMDEISTGLDSSTTYQIVNFLKQFI
HILDGTAVISLLQPAPETYDLFDDIILLSDGQIVYQGPRELVLDFFKSMGFKCPRRKGVADFLQEVTSRKDQHQY
WVRKDEPYTFVTVREFVEAFQSFHVGRKLGDELSTPFDKSKNHPAALTTKRFGVGMKDLLKACILREYLLMK
RNSFVYIFKVSQLIIMAFISTTLFLRTKMHRDIVTDGGVYIGALFFTVITIMFNGMSEISMTIPKLPIFYKQRDLLF
YPAWAYALPSWILKIPITFVEVAAWVFITYYVTGYHPNVGRLFKQYLLLVAINQMASGLFRLIAALGRNLIVAN

TFGSFVLLALVALGGVVLSREDVKKWWIWGYWTSPLMYGQNAIVVNEFLGSNWNKIPTNSSTNEPLGIQVLK
SRGFFTEAYWYWIGLGALFGFIILFNFGFSVALALLNPFGKSHKVKSDDPEGNKNVDRIEGSIQLQSRGSSLRNG
SGRSSKPTPPWPETAVAANTKRGMVLPFEPHSITFDKITYSVDMPREMKNQGAPEERLVLLKGVSGAFRPGVL
TALMGISGAGKTTLMDVLAGRKTGGYIKGNITISGYPKNQDTFARISGYCEQNDIHSPHVTVYESLIYSAWLRL
PPGVDDQTRKLFVEEVMELVELNPLRQALVGLPGVDGLSIEQRKRLTIAVELVANPSIIFMDEPTSGLDARAAAI
VMRTVRNTVDTGRTVVCTIHQPSIDIFESFDELFLMKRGGQGIYVGPLGRHSSHLIEYFEGIQGVSKIKDGYNPA
TWMLEVTSPAQELALGVDFSDLYKNSDLYRRNKALIEELSIPPPNSKDLYFPTKYSQSTFTQLMACLWKQHWS
YWRNPPYTAVRFLFTISIAIMFGTMFWDLGSKTTRSQDLFNAMGSMYAALVFLGIQNASSVQPVVAVERTVFY
RERAAGMYSALPYALAQVLIEVPYVLMQAISYGIIVYVMIGFKWTVAKFFWYLFFMFFTLLYFTYYGMMAVG
MTPNHHIASIVSFAFYAIWNLFSGFVVPRTKIPIWWRWFYWVCPVSWTLYGLVVSQFGDLTNTLEDTGDTVEE
YMRDYFGYRHDFLGVIAAVMIGFTMLFAFVFAVSIKILNFQRR

>XP_018811186.1_P_PDR1-l_Jr
MDASGDIYKASGSLRGSLRLRGSSAWRNNAMDVFSKSSREEDDEEALKWAALEKLPTFDRLRKGILTTSKGEA
SEVNIQNLGFEERKKLLERLVKLAEEDNEKFLLKVRNRIDRVGIDLPAIEVRFEHVNIDAEVFVGSRALPSCFNF
CISIVEGFLNFAHILSNKKKHLSILKDASGVIKPRRMTLLLGPPSSGKTTLLLALAGKLDPDLKFSGRVTYNGHD
MNEFVPQRTAAYISQFDLHIGEMTVRETLAFSARCQGIGSRYDMLAELSRREKEANIKPDQDVDIYMKAATTE
GQEADVATDYILKVLGLEACADTMVGDEMLRGISGGQRKRVTTGEMMVGPAKALFMDEISTGLDSSTTFQIV
NSLRQYVHILDGTAVISLLQPAPETYNLFDDIILLSDGYIVYQGPREDVLEFFECMGFKCPDRKGVADFLQEVTS
RKDQEQYWARKDEPYSFVTTQEFAEAFQSFHVGRRIGDELATPYDKTKSHPAALTTKKYGVSQKELLKASFSR
EYLLMKRNSFVYVFKLSQLFFMALISMTIFLRTKMPREDTTDGGIYTSALFFTVIMIMFNGMAELSMTIIKLPVF
YKQRDLLFYPSWVYAIPQWILKIPITFLEVAVWVFMTYYVIGFDPNVGRLFKQYLLLVLVNQMASGLFRFMAG
LGRSMIVANTFGTFAFLLLFALGGFILAKEDIKKWWIWGYWISPLMYGQNALVVNEFLGNNWKKVLPNTNET
LGVTVLKSRDFVTDANWYWIGAGATVGFILLFNIGFPIALAYLNPFGKSQSTTSDEPESNEQGKRTGGGIQLTQ
RENSSSQRGSTSSAIEASRNGKRGMVLPFEQHSITFDEIIYSVDMPQEMKEQGVLEDKLELLKGVSGAFRPGVL
TALMGVSGAGKTTLMDVLAGRKTGGYIEGNITISGYPKKQETFARISGYCEQNDIHSPHVTVYESLVYSAWLR
LSSGVDSETRKMFIEEVMELVELNPLRNALVGLPGVNGLSTEQRKRLTIAVELVANPSIIFMDEPTSGLDARAA
AIVMRTVRNTVDTGRTVVCTIHQPSIDIFEAFDELFLMKRGGQEIYVGPLGHHSNHLVQYFESTEGVKKITDGY
NPATWMLEVTSQGEEIALGVDFTDVYRNSELYRRNKALIKELSKPAPGSKDLYFLTQYSQPFWTQCMACLWK
QRWSYWRNPLYTAVRFLFTVFIALAFGTMFWDLGSKTKRIQDLTNAMGSMYAAVLFLGVQNASSVQPVVAV
ERTVFYRERAAGMYSALPYAFGQVAIEIPYIFVQAAVYGLIVYAMIGFEWTAAKFFWYLFFMFFTLLYFTFYG
MMSVAATPNHHIAAIISSAFYGVWNLFSGFIIPRPRIPIWWRWYTWACPVAWTLYGLLVSQFGDIKEVLTDSEIF
DETVEAYMRRYFGFKHDFLAVVAVVVAGFAVLFGFIFAFSIKVFNFQRR

>XP_018827627.1_P_PDR1-l_Jr
MDSSDLYRATGSLRLRNSSVWRNNAMDVFSMSSREEDDEEALKWAALEKLPTFDRLRKGILTTSRGEAIEVDL
QNLGLDERKKLLERLVKLADEDNEKFLLKVRNRIDRVGIDLPTIEVRFEHVNVEAEAHVGGRALPTFFNFSVN
MLEGFLNFFHILSSQKKHLSILKDVTGVIKPRRMTLLLGPPSSGKTTLLLALAGKLDPTLKVSGRVTYNGHDMR
EFVPQRTAAYISQYDLHIGEMTVRETLAFSARCQGVGSRYDMLAELSRREKEANIKPDPDVDIYMKAATTEGQ
EADVATDYILKVLGLEVCADTMVGDEMVRGISGGQRKRVTTGEMLVGPAKALFMDEISTGLDSSTTFQIVNSL
RQYVQILDGTAVISLLQPAPETYDLFDDIILLSDGYIVYQGPRENVLEFFESMGFKCPDRKGVADFLQEVTSRKD
QEQYWARRDAPYSFVTVREFAEAFQSSHVGRRIGDELSAPYDKTKSHPAALTTKTYGVGKKELLKASFSREYL
LMKRNSFVYVFKLSQLFLMALIAMTIFLRTKMPRRDSTDGTIYLGALFFTVVMIMFNGMSELSMTIIKLPVFYK
QRDLLFYPSWVYALPAWILKIPVTFLEVAVWVFMTYYVIGFDPNVGRLFRQYFLLVLVNQMASALFRFIAAM
GRNMIVANTFGSFALLLLFALGGFILSRDDVKKWWIWGYWMSPLMYGQNAILVNEFLGNNWKKVLPNTNEP
LGVTVMKSRGFFPQAYWYWIGVGASFGFIILFNIGFTFSLAYLNPFGKSQTVKSDEPESDEQGNRTGEGVELTR
RENSSSHHRRAIGSGRDSRRGSTSTRSSFEIEASRNGKRGMVLPFEQHSITFDEIMYSVDMPQEMKEQGVLQDK
LVLLKGVSGAFRPGVLTALMGVSGAGKTTLMDVLAGRKTGGYIEGNITISGYPKKQETFARISGYCEQNDIHSP
HVTVYESLVYSAWLRLSSDVDSETRKMFIEEVMELVELNPLRNALVGLPGVNGLSTEQRKRLTIAVELVANPSI
IFMDEPTSGLDARAAAIVMRTVRNTVDTGRTVLCTIHQPSIDIFEAFDELFLMKRGGQEIYVGPLGHHSSHLIKY
FENMEGVRKIKDGYNPATWMLEVTTPAEELTLGVDFTTLYKNSDLYRRNKALIAELSKPAPASKDLYFPTQFS
QSFRTQCMACLWKQRLSYWRNPPYTAVRFLFTVFIALTFGTMFWDLGSKTKRTIDLFNAMGSMYAAVLFLGV
QNSSSVQPVVAVERTVFYRERAAGMYSALPYAFGQVAIELPYVFMQSAVYGIIVYAMIGFEWTAAKFFWYLFF
MYFTLLYFTFYGMMSVAFTPNHHIAAIVSSAFYAIWNLFSGFIVPRTRIPIWWRWYYWACPVAWTLYGLVVSQ

FGDVQDRLEDNDQTVEEYLKSYLGFRHDFLGVVGVVVAGFAVLFGFIFAFSIKAFNFQRR

>XP_018834768.1_P_PDR1-l_Jr
MEGSDILKVSSARLSSSSIWRNSGMEGFSRSSLDENDEEALKWAAIEKLPTYLRIRRGILTEEEGQAREIDIKNLG
LLDRKNLIERLLKIAEEDNENFLLKLKDRIERVGLEFPTIEVRFEHLYVEAEAYVGSRALPTIFNFCVNMLEGFLS
CLHILPSRKKPLPILHDLSGIIKPRRMTLLLGPPSSGKTTLLLALAGKLGKELKASGRVTYNGHGMEEFVPQRTS
AYISQHDVHIGEMTVRETLAFSARCQGVGPRYEMLAELSRREKAANIKPDPDLDIYMKASALEGPEASVETHYI
LKILGLEVCADTMVGDEMLRGISGGQKKRVTTGEMLVGPARALFMDEISTGLDSSTTFQIVNSLRQSIHILNGT
AVISLLQPAPETYDLFDDIILLSDGQIVYQGPRENVLEFFECMGFKCPERKGVADFLQEVTSRKDQEQYWANKD
EPYNFVSVKEFAESFQSFHIGRKLGDELATPFDKSKGHPAALTTKKYGVSKKELFKACASREFLLMKRNSFVYI
FKMFQLILSAFITMTLFIRTEMRRDTTTDGGIYMGAMFFTIVMIMFNGFSELAMTIMKLPVFYKQRDLLFYPSW
AYSLPTWILKIPITLVEVAIWVLMTYYVIGFDPNIQRFFKQFLLLLCINQMASGLFRLMGALGRNIIVANTFGSFA
LLAVLVMGGFVISREDVQKWWLWGYWVSPMMYGQNAIAVNEFLGNSWRHIPPNATEPLGILILKSRGVFTEA
YWYWIGVGATIGYIFLFNFLFTLALKYLDPFGKPQAVQSREALAEKIANRTGEVIELSSRRRSSFEKGNESKRSV
SSRTLSARVSSINEAKQHNKRGMVLPFEPLSITFDEIRYAIDMPQEMKAQGVPEDRLELLKGVSGAFRPGVLTA
LMGVSGAGKTTLLDVLAGRKTGGYIEGTITISGYPKKQETFARISGYCEQTDIHSPHVTVYESLLYSAWLRLPPE
VDSATRKMFLEEVMELVELTTLREALVGLPGINGLSTEQRKRLTIAVELVANPSIIFMDEPTSGLDARAAAIIMR
TVRNTVDTGRTVVCTIHQPSIDIFDAFDELFLLKRGGEEIYVGPLGRHSSHLINHFEGIDGVPKIKEKYNPATWM
LEVTSAGQETALGVNFTEIYKNSELYRRNKALIKELSTATLGSKELYFPTQYSQSFFTQCMACLWKQHLSYWR
NPPYSAVRLLFTTFIALMFGTIFWDLGSKRRNRQDLFNAMGSMYAAVLFIGVQNASSVQPIVAIERTVFYRERA
AGMYSALPYAFGQVVIELPYIFVQTIIYGIIVYAMIGFDWTLSKFLWYIFFMYFTFLYFTFYGMMTVAVTPNHNI
AAIVSSAFYAIWNLFSGFIVPRTRMPIWWRWYFWVCPVSWTLYGLIASQFGDIKDKLDTGETVEDFLRNYFGY
RNEFLGVVAVAIVGISVLFGFTFAYSIKAFNFQKR

>XP_019166107.1_P_PDR1-l_In
MEAADLGIIRGGSIRGSLRGSLRGSSRANSNSIWRNTGVEVFSRSSRDEDDEEALKWAALEKLPTFDRLRKGLL
FGAEGPATEVDINNLGFHERQTLLQRLVNVADEDNEKFLMKLRNRIDRVGIDLPTIEVRYEHLKVEADAYAGS
RALPTFINFTTNFIEDLLNALHITRTKKRNLTILNDVSGIIKPCRMTLLLGPPSSGKTTLLLALAGKLDKALRVGG
RVTYNGHGLDEFVPQRTAAYISQHDLHIGEMTVRETLEFSARCQGVGSRYEMLAELSRREKAANIKPDPDIDV
YMKAAATEGQEANVVTDYILKILGLEVCADTMVGDEMIRGISGGQKKRVTTGEMIVGPSKALFMDEISTGLDS
STTYSIVNSLKQYVHILKGTAVISLLQPAPETYNLFDDIILLSDGYLVYQGPREHILDFFESMGFRCPDRKGVADF
LQEVTSKKDQQQYWIRRDEPYRFVKAKEFAEAYQSFHVGRRSAEELSAPYDKTKSHPAALTNEKYGIGSKQLL
KVCTEREYLLMQRNSFAYVFKFCQLTIMALITMTLFFRTEMHKNNETDGGIYVGALFFAVVMIMFNGMSELP
MTIYKLPVFYKQRDLLFFPPWAYALPSWILKVPVTFVEVGLWVFVTYYVMGFDPNVGRLFKQFLILIMVNQM
ASSLFRFIAAMGRTMGVANTFGSFALLLQFALGGVVLSRDDVKKWWIWGYWTSPLMYAQNGILVNEFNGHS
WSKPVVNSSETLGARVVTSRGFFSESKWYWIGLGALFGFTIVFNLCYSIALQYLNPFGKPQALISDDDDDKETA
SVQTVGTNGGNGISEAPPETQKRGMVLPFEPHSLTFDDVVYSVDMPQEMRDQGAAENRLVLLKGVSGAFRPG
VLTALMGVSGAGKTTLMDVLAGRKTGGYIDGDIKISGYPKKQETFARISGYCEQNDIHSPFVTVYESLVYSAW
LRLPEDVDARTRKMFVDEVMELVELVPLRSALIGLPGVNGLSTEQRKRLTIAVELVANPSIIFMDEPTSGLDAR
AAAIVMRTVRNTVDTGRTVVCTIHQPSIDIFEAFDELFLMKRGGQEIYVGPLGRNSCHLIKYFESIEGVAKIKDG
YNPATWMLEVTTSAQEMLLGVDFTDAYKNSDLYRRNKALISELSTPRPGSKDLYFRTQYSQSFLSQCMACLW
KQHWSYWRNTSYTAVRFIFTSFIALAFGTMFWDLGTKVDRKQDVFNAMGSMYAAVLFLGVQNSSSVQPVVA
VERTVFYREKAAGMYSALPYAFAQALIEIPYILLQSAVYGVIVYAMIGFEWTVAKFFFYFFVMFFSLLYFTFYG
MMSVAVTPNQNVASIVAAFFYGIWNLFSGFIIPRPSMPVWWRWYFWACPVSWTLYGLVASQFGDIESKLRDS
DQTVKEFVEDYLGFKHDFLGVVAAVTVAFPVLFGFIFAFAIKAFNFQKR

>XP_019175624.1_P_PDR1-l_In
MEVFSSRGEGENEEEALKWAAIEKLPTYLRVRRGILTEEEGQCKEVDVKGLGLVEKKNLLERLVRVAEEDNEK
FLLKLKQRIDRVDIDLPTIEVRFEHLNVDAEAYVGSRALPTIFNFTVNMLEGFLNSIHILPNRKKPLPILHEVSGII
KPGRMTLLLGPPSSGKTTLLLALAGKLGSDLKVSGKVTYNGHEMTEFVPQRTSAYISQYDLHIGELTVRETLAF
SARCQGIGPRYEMLAELSRREKELNIKPDPDIDIFMKAASIEGEEASVVTDYVLKILGLEGCADILVGDEMIRGIS
GGQKKRVTTGEMMVGPTRVLFMDEISTGLDSSTTFQIVNSIRQSIHILEGTAVISLLQPAPETYDLFDDIILLSDG
QIVYQGPRENVVEFFEHMGFKCPERKGVADFLQEVTSRKDQEQYWARRHEQYKFITAREFSEAFQCFHVGRK
LGDELAVPFDKAKSHPAALTTKKYGVNKKELLKACISREFLLMKRNSFVYIFKMMQLTLMAFISMTLFLRTKM

KRDSATDGTIFMGALFFAVITTMYNGYSELFLSILKLPVFYKQRDFLFFPSWAYALPTWILKIPITLVEVAIWVC
MTYYVIGFDPDFGRFFKQLFLLICVNQMASALFRFIAALGRNIIVANTFGSFALLTVLVMGGFVLSRNDVKKW
WVWGYWFSPMMYAQNAIAVNEYLGKSWRHIPSGSSEPLGVTVLKSRAIFPEARWYWIGVGALIGYVILFNFLF
TVALTYLNPFGKPQAVLSEETLAERNASKRREPIESISERRNDVLKSASSRSISSRVGSINEANPKRRRGMVLPFE
PHSITFDEIKYAVDMPQEMKAQGFTEDRLELLKGVSGAFRPGVLTALMGVSGAGKTTLMDVLAGRKTGGYIE
GDISISGYPKKQETFARIAGYCEQTDIHSPHVTVLESLQYSAWLRLPPEVDTEARKMFVEEVMELVELTPLRGA
LVGLPGVNGLSTEQRKRLTVAVELVANPSIIFMDEPTSGLDARAAAIVMRTVRNTVDTGRTVVCTIHQPSIDIFD
AFDELLLLKRGGQEIYVGPIGRHSSQLIEHFEAIPGVPRIKDGYNPATWMLEVTSAAQETLIATDFAQVYRNSEL
FRRNKALIKELMSKPALGSKDLHFPTKYSQSFYTQCLACLWKQHWSYWRNPPYTAVRLMFTTFIALMFGTIFW
DLGSRRRKQQDVFNAMGSMYAAILFLGVQNGSSVQPVVSVERTVFYRERAAGMYSALPYAIAQVVIELPYLL
VQTIIYGVIVYAMIGFEWTVTKFLWYLFFMYFTLLYFTLYGMMTVAVTPNHNIAGIVSSAFYAIWNLFSGFIVP
KTRIPVWWRWYYYICPISWTLYGMVASQFGDVKDKLVDTDQTVEEFIKTYFDFDQDFVGFVGVILVGLSLLFA
FIFAFSIKVFNFQKR

>XP_019179480.1_P_PDR1-l_In
MEGGEGGLRVSSARLSSSNVWRSSGVEVFSRSSREAAEDDEEALKWAAIEKLPTYLRIRRGILAAEEEGGGPKE
IDVEKLGVAEKRSLLERLVRIAQEDNESFLLKLKHRTDRVGIDLPTIEVRFEHLNVDAEAYVGSRALPTIFNFTA
NILEAFMNYLHILPNRKKPLPILHDVSGIIKPARMTLLLGPPSSGKTTLLLALAGKLDSDLKVSGRVTYNGHEMT
EFVPRRTSAYINQHDLHIGEMTVRETLAFSARCQGVGHRYEMLAELSRREKELNIKPDPDIDVYMKAISIEGQE
ASVVTDYILKILGLEVCADILVGDEMIRGISGGQRKRLTTGEMLVGPARALFMDEISTGLDSSTTFQIVNSIRQSI
HILQGTAVISLLQPAPETYELFDDIILLSDGQVVYQGPRDNVLEFFQHMGFKCPERKGVADFLQEVTSRKDQEQ
YWMRREEAYEFITVGEFSEAFQSFHVGKKLGDELAVPFDKSKSHPAALSNKKYGVNKKELLKACASREFLLM
KRNSFVYIFKMIQLVLMAFVAMTVFLRTEMPKNSIADGTVFMGALFFAVIMTMFNGFSELALSILKLPAFYKQR
DLFFFPPWAYALPTWILKIPVTLVEVAIWVCMTYYAIGFDPDAGRFFKQLFLLICVNQMASALFRLTAAAGRSV
IVANTFGSCAMILVLVLGGFILSRDDVKKWWIWGYWISPLMYAQNAISVNEYLGKSWRHVPSGTSETLGVTVL
RSRGIFPEAHWYWIGVGALVGYVFLLNFLFCLALTYLNPLGKPQAMLSEETLAERNACKRGEPIELELKGGSTS
EKGNVVRRSVSSRSLSSRVGSVNVADPNMKRGMVLPFEPLCITFDEIKYAVDMPTEMKAQGLTEDRLVLLKGI
NGAFRPGVLTALMGISGAGKTTLMDVLAGRKTGGYIEGSITISGYPKRQETFARIAGYCEQTDIHSPHVTVYES
LVYSAWLRLPPEIDTATREMFVEEVMDLVELTSLREALVGLPGVNGLSTEQRKRLTIAVELVANPSIIFMDEPTS
GLDARAAAIVMRTVRNTVDTGRTVVCTIHQPSIDIFDAFDELLLLKRGGEEIYVGPLGRHSSQLIRYFETINGIQR
IRDGYNPATWMLEITSEAQEAALGIDFAEVYRNSELYRRNKALIQELSKPAPGSKDLYFPTKYSQSFFTQCMAC
LWKQHWSYWRNPPYTAVRLLFTTFNALLFGTMFWGIGPKRRTQQDLFNAMGCMYAAVVFLGVQNATSVQP
VVAIERTVFYRERAAGMYSALPYAFGQVVIELPHLFVQTLIYGVIVYAMIGFEWTAAKFLWFIFFMYFTLLYFT
LYGMMTVAVTPNHNIAAIISSAFYGFWNLFSGFIIPKTRIPVWWRWYYYICPVSWTLYGLIASQFGDVKEKLDT
NETVKDFVKSFFDFDHDFVGYVATIIVGICVMFGFIFAFSIKAFNFQTR

>XP_019234268.1_P_PDR1-l_Na
MEGGEDIFRVSSARLNSSNVWRNSAMDVFSRSSREADDEEALKWAALEKLPTYLRIRRGILTEEEGQSREVDIT
KLDLVERRNLLERLVKIADEDNEKFLLKLKKRIDRVGLDLPTIEVRFEHVSVDAEARVGSRALPTIFNFTVNILE
DFLNYLHILPSRKKPLPILHEVSGIIKPGRMTLLLGPPSSGKTTLLLALAGKLDKDLKVSGKVTYNGHGMDEFVP
QRSSAYISQNDLHIGEMTVRETLAFSARCQGVGAKYEILAELSRREKEANIKPDPDVDIFMKSAWNEGQEANV
VTDYTLKILGLDICADTLVGDEMIRGISGGQRKRLTTGEMMVGPARALFMDEISTGLDSSTTYQIVNSIRQSIHIL
QGTAVISLLQPAPETYDLFDDIILLSDGQIVYQGPRENVLEFFEYMGFMCPERKGVADFLQEVTSRKDQEQYW
ARRDEPYKFITVREFSESFQSFHIGRKLGDELAVPFDKSKSHPAALTTKRYGVSKKELLKACTAREYLLMKRNS
FVYIFKMIQLTLMASITMTLFLRTEMHRNTTTDGAVFLGALFFAVIMIMFNGFSELALSIMKLPSFYKQRDLLFF
PAWAYALPTWILKIPVTLVEVAIWVCMTYYVIGFEADVGRFFKQLFLLICVNQMASGLFRFLGALGRNVIVAN
TFGSCALLTVLVMGGFIVSRDDVKKWWIWGYWISPMMYAQNAIAVNEFLGKSWAHVPPNSTGTETLGVSFL
KSRGIFPEARWYWIGAGALLGYVLLFNFLFTVALAYLNPFGKSQAVLSEETVAERNASKRGEVIELAPIEKPSSE
RGNDIRRSASSRSMSSRVGSITEADLKKRRGMILPFEPLSITFDDIRYTVDMPQEMKAQGVAEDRLELLKGVSG
AFRPGVLTALMGVSGAGKTTLMDVLAGRKTGGYIDGTISISGYPKQQETFARIAGYCEQTDIHSPHVTVYESLQ
FSASLRLPREVDTETRKMFVEEVMELVELTPLREALVGLPGVNGLSTEQRKRLTVAVELVANPSIIFMDEPTSG
LDARAAAIVMRTVRNTVDTGRTVVCTIHQPSIDIFDAFDELLLLKRGGEEIYVGPLGRHSSHLIKYFEGIDGVPKI
KDGYNPATWMLEITSAAQEAARGIDFTELYKNSELYRRNKALIKELSVPAPCSKDLYFPTKYSQSFFTQCMACF
WKQHLSYWRNPPYTAVRLMFTFFIALMFGTIFWDLGSIRKRQQDLLNAIGSMYVAVLFLGVQNATSVQPVIAI

ERTVFYRERAAGMYSALPYAFGQVMIELPYLFIQTIIYGVIVYVMIGFEWTVAKFFWYLFFMYFTLLYFTLYGM
MTVAVTPNHSIAAIISSAFYAIWNLFCGFVVPKTRMPVWWRWYYYICPVSWTLYGLIASQFGDLQDKLDTNET
VEEFIENFFDFKYDFVGYVAVILVGISVVFLFIFAYSIKAFNFQKR

>XP_019425122.1_P_PDR1-l_La
MEGSDIYRATNSLRVNSSTVWRNSAMEVFSKSSREENDEDALKWAALEKLPTYNRLKKGLLTTSCGVSNEIDV
TDIGFQERQKLLDRLVKVAEEDNEKFLLKLKKRIDRVGLDIPTIEVRFQHLNVEAEAYVGSRSLPSFLNFGTNIV
ESFFTSLHILKSKKKHMTILKDVSGIIKPRRMTLLLGPPSSGKTTLLLALSGKLDPNLKVSGRVTYNGYGMDEFV
PQRTAAYISQHDVHIGEMTVRETLAFSARCQGVGTRYDLLSELSRREIEAKIKPDPDIDVYMKATAAIGQEESL
VTDYALKILGLDICADTLVGDEMLRGISGGQRKRVTTGEMLVGPANALFMDEISTGLDSSTTFQIVSSLRQYVH
ILNGTAVISLLQPAPETYELFDDIILISDGKIVYQGPCEYVLDFFESMGFKCPERKGVADFLQEVTSVKDQEQYW
AHRDIPYRFITVMQFAEAFQSFHIGRRMGDEIAVQFDKSKNHPAALTTNKYGVNKKELLKANFSREYLLMKRN
SFVYIFKICQLTLMALIAMTLFLRTEMHRDSLSDGGVYAGALFFTVVMIMFNGMAELSMTIAKLPIFYKQRDLL
FYPSWAYAIPSWILKIPITLIEVAVWVFLTYYVIGFDPNVERLLKQYFLLVLINQMASGLFRAIAALGRNIIVAST
FGSFALLTFLTLGGFVLSKKDIKRWWIWGYWISPLMYGQNAIVVNEFLGNSWNKLTPTSNKTIGIQILESRGFFT
QAYWYWIGIGALIGFMIIFNIVYTLALTYFNPFDKPQATMTQESEGGQEVELPFIGNSGRANAVMDSSHGKKRG
MVLPFEPHSLTFDQIVYSVDMPQEMKDQGAIEDRLVLLKSVSGAFRPGVLTALMGVSGAGKTTLMDVLAGRK
TGGYIEGSIKVSGYPKKQETFARISGYCEQNDIHSPHVTVYESLVYSAWLRLPAEVDSNARKMFIEEVMELVEL
NPLRNSLVGLPGVSGLSTEQRKRLTIAVELVANPSIIFMDEPTSGLDARAAAIVMRTVRNTVDTGRTVVCTIHQP
SIDIFEAFDELFLMKRGGQEIYVGPLGRHSSQLINYFESIDGVSKIKDGYNPATWMLEVTTSAQELNLGVDFHD
MYKNSELYRRNKQVIAELSNLALGSKDLYFATQYSQSLLVQCLACLWKQHWSYWRNPPYTAVRFLFTTFIAL
MFGTMFWNLGGKTSNRQDLFNAIGSMYTAVLFLGVQNSASVQPVVAVERTVFYRERAAGMYSALPYAIAQVI
IELPYVFAQAVSYGGIVYSMIGFEWTLEKFFWYIFFMYFTLCYFTFYGMMAVAVTPNHHVASIVAAAFYAIWN
LFSGFVVPRPRIPVWWRWYYWACPVAWTIYGLVASQFGDITTVMKSEDMSVQEFLRSYFGIKHDFVGVCAVV
VSGTAILFAFTFAVSIKVFNFQKR

>XP_019437694.1_P_PDR1-l_X1_La
MEGGDELRVASARIGSSRIWRSNGFDGFSGSSRREEDEEEELKWAAIEKLPTFLRMSRGILSESEGQPGTEIHINK
LGSLQRKDLVERLVKIAEEDNEKFLLKLRERIDRVGLDIPTIEVRFEHLNVEAEAHVGSRALPTNFNFCINLLEG
SLNDLRLLPSRKKQFTVLHDVSGIIKPRRMTLLLGPPSSGKTTLLLALAGRLSKDLKLSGRVAYNGHGMEEFVP
QRTSAYISQYDLHIGEMTVRETLAFSARCQGIGSRYEMLAELSRREKASNIKPDPDIDIYMKAAALEGQEANVV
TDYILKILGLEVCADTMVGDDMIRGISGGQKKRVTTGEMLVGPARALFMDEISTGLDSSTTFQMINSLRQSIHIL
NGTAVISLLQPAPETYELFDDIILLSDGQIVYQGPRDNVLEFFEFMGFKCPERKGVADFLQEVTSRKDQEQYWA
NKDEPYSFITVKEFSDAFQSFHIGQKLGDELATPFDTSKGHPAVLTKNKYGVSKKELLKACVSREFLLMKRNSF
VYIFKMWQLFFMAFITMTLFLRTEMHRDTVNDGGIYMGALFFTLIVIMFNGSAEINMTIVKLPVFYKQRELLFY
PSWAYSLPTWILKIPITIIEVGIWVIMTYYVIGFDPNFERFIKQYFLLVCINQTASGLFRFMGAVGRNIIVANTFGS
FALLALMALGGFVLSRGELLIHFIACFKEFYYGNLTKVCSFALVDVKKWWLWGYWVSPMMYAQNALAVNEF
LGKSWAHVPPNSTQSLGVQVLKARGFFPEAYWYWIGVGACIGYMLLFNFLFPLALQYLNPFDKPQALISEEAL
AERNAVRKDHIIELSSTYKGSSDISRRNVSSRTLSARVGSFSAVHHSKKRGMVLPFTPLSITFDEIRYAVDMPQE
MKAQGFPEDQLELLKGVNGAFRPGVLTALMGVSGAGKTTLMDVLSGRKTTGYVQGQITISGHPKKQETFARI
SGYCEQTDIHSPHVTVYESLVYSAWLRLPPEVDSSTRQMFIEEVMELVELTSLREALVGLPGVNGLSTEQRKRL
TVAVELVANPSIIFMDEPTSGLDARAAAIVMRTVRNTVDTGRTVVCTIHQPSIDIFDAFDELILLKRGGEEIYVGP
LGQHCSHLINYFEGINGVPKIRNGYNPATWMLEITSEAQEAALGVNFAEIYKNSDLYRRNKDLIRELSTPLPGSK
DLYFPTTYSQTFLTQCMACLWKQRLSYWRNPPYSGVRFLFTTFIALMFGTIFWDIGSKSGRIQDLFNAMGSMY
AAVLFIGVSNATSVQPIVAIERTVFYRERAAGMYSALPYAFGQIAIEIPYIFIQTAVYGIIVYAMIGFEWTASKFF
WYLFFMFFTFLYFTFYGMMAVGATPDQNIAAITSFAFYLIWNLFSGFIIPQKRMPIWWRWYFWICPVSWTLYG
LVTSQYGDVQEHLETGETVQEFLFNYFGYRHDFIGVAASVLVGFTLLFGFTFAFSIKTFNFQKR

>XP_019437695.1_P_PDR1-l_X2_La
MEGGDELRVASARIGSSRIWRSNGFDGFSGSSRREEDEEEELKWAAIEKLPTFLRMSRGILSESEGQPGTEIHINK
LGSLQRKDLVERLVKIAEEDNEKFLLKLRERIDRVGLDIPTIEVRFEHLNVEAEAHVGSRALPTNFNFCINLLEG
SLNDLRLLPSRKKQFTVLHDVSGIIKPRRMTLLLGPPSSGKTTLLLALAGRLSKDLKLSGRVAYNGHGMEEFVP
QRTSAYISQYDLHIGEMTVRETLAFSARCQGIGSRYEMLAELSRREKASNIKPDPDIDIYMKAAALEGQEANVV
TDYILKILGLEVCADTMVGDDMIRGISGGQKKRVTTGEMLVGPARALFMDEISTGLDSSTTFQMINSLRQSIHIL

NGTAVISLLQPAPETYELFDDIILLSDGQIVYQGPRDNVLEFFEFMGFKCPERKGVADFLQEVTSRKDQEQYWA
NKDEPYSFITVKEFSDAFQSFHIGQKLGDELATPFDTSKGHPAVLTKNKYGVSKKELLKACVSREFLLMKRNSF
VYIFKMWQLFFMAFITMTLFLRTEMHRDTVNDGGIYMGALFFTLIVIMFNGSAEINMTIVKLPVFYKQRELLFY
PSWAYSLPTWILKIPITIIEVGIWVIMTYYVIGFDPNFERFIKQYFLLVCINQTASGLFRFMGAVGRNIIVANTFGS
FALLALMALGGFVLSRVDVKKWWLWGYWVSPMMYAQNALAVNEFLGKSWAHVPPNSTQSLGVQVLKARG
FFPEAYWYWIGVGACIGYMLLFNFLFPLALQYLNPFDKPQALISEEALAERNAVRKDHIIELSSTYKGSSDISRR
NVSSRTLSARVGSFSAVHHSKKRGMVLPFTPLSITFDEIRYAVDMPQEMKAQGFPEDQLELLKGVNGAFRPGV
LTALMGVSGAGKTTLMDVLSGRKTTGYVQGQITISGHPKKQETFARISGYCEQTDIHSPHVTVYESLVYSAWL
RLPPEVDSSTRQMFIEEVMELVELTSLREALVGLPGVNGLSTEQRKRLTVAVELVANPSIIFMDEPTSGLDARA
AAIVMRTVRNTVDTGRTVVCTIHQPSIDIFDAFDELILLKRGGEEIYVGPLGQHCSHLINYFEGINGVPKIRNGYN
PATWMLEITSEAQEAALGVNFAEIYKNSDLYRRNKDLIRELSTPLPGSKDLYFPTTYSQTFLTQCMACLWKQRL
SYWRNPPYSGVRFLFTTFIALMFGTIFWDIGSKSGRIQDLFNAMGSMYAAVLFIGVSNATSVQPIVAIERTVFYR
ERAAGMYSALPYAFGQIAIEIPYIFIQTAVYGIIVYAMIGFEWTASKFFWYLFFMFFTFLYFTFYGMMAVGATPD
QNIAAITSFAFYLIWNLFSGFIIPQKRMPIWWRWYFWICPVSWTLYGLVTSQYGDVQEHLETGETVQEFLFNYF
GYRHDFIGVAASVLVGFTLLFGFTFAFSIKTFNFQKR

>XP_019448108.1_P_PDR1-l_X2_La
MDGSDIYRASNSLRARSSTVWRDSGVEVFSKSTREEDDEEALKWAALEKLPTYNRLRKGLLTTSHGAASEIVV
TDLAHQERQKLLDRLVKVAEEDNEKFLLKLRERVDRVGLDIPTIEVRYEHMKIEAEAFVGGRALPSFINSATNV
IEGLLNLLHIIPTKKKHVTILKDVSGIIKPRRMTLLLGPPGSGKTTLLLALSGKLDPSLQVTGKVTYNGHEMNEF
VPQRTAAYISQHDVHIGEMTVRETLAFSARCQGVGSRYDMLSELSRREKAANIKPDPDIDVYMKATATAGQES
SIVTDYILKILGLDICADTMVGDEMLRGISGGQRKRVTTGEMLVGPANALFMDEISTGLDSSTTFQIVSSLRQYV
HILNGTAVISLLQPAPETYDLFDDIILISDGYVVYHGPREYVLDFFESMGFKCPERKGVADFLQEVTSRKDQAQ
YWVRKDQPYRFVNEVQFAEAFQSFHIGRKLADELAVPFDKSKNHPAALTTEKYGINKKELLKANLSREYLLM
KRNSFVYIFKLCQIFVMAFIAFTLFFRTEMHHGNVTDAGVYTGALFFTLVTIMFNGMAEISMTIAKLPVFYKQR
DLLFYPSWAYAIPSWILKIPVTMAEVAVWVFLTYYVIGFDPNVGRFFKQYLILFFLSQMASGLFRAIAALGRNM
IVANTFGSFAVLTFLSLGGFVISKKDIKSWWIWGYWISPLMYGQNALMVNEFLGNSWHNSTHDIGVDLLESRG
FFTRSYWYWIGFGALVGFVFLFNMLFGVALEWLGPFDKPQATISEEPEETTEVEQEVELPRIESSGRGVVVEGS
SHGKKKGMVLPFEPHSITFDEITYSVDMPQEMKEQGVQEDKLVLLNGVSGAFRPGVLTALMGVSGAGKTTLM
DVLAGRKTGGYIDGSIKISGYPKKQETFARISGYCEQNDIHSPHVTVYESLLYSAWLRLSSSVDSNKRKMFIEEV
MELVELKPLRNSLVGLPGVSGLSTEQRKRLTIAVELVANPSIIFMDEPTSGLDARAAAIVMRTVRNTVDTGRTV
VCTIHQPSIDIFEAFDELFLMKRGGREIYVGPLGRHSTHLIKYFESIEGVSKIKDGYNPATWMLEVTSTAQELSLG
ADFTEIYQNSDLYRRNKQLIQELSQPAPGSKDLYFPTQFSQSFLVQCQACLWKQRWSYWRNPPYTAVRFFFTT
FIALMFGTMFWDIGTKTANMQDLLNAVGSMYSAVLFLGVQNSASVQPVVAVERTVFYREKAAGMYSALPYA
FSQILVELPYIFAQAVTYDLIVYAMIGFEWTVAKFFWSLFFMYFTLCYFTFYGMMAVAVTPNHHVASIVAAAF
YAVWNLFSGFVVTRPNIPVWWRWYYWACPVAWTIYGLIASQFGDIEDFMESEQKSVKDFVESYFGIKHDFIG
VTAVVVPGIAVLFAFIFAVGIKVFNFQKR

>XP_020205457.1_PDR1-l_X1_Ccaj
MESGELRVASARIGSSSMWRSSVVDVFSGSSRRDDDEEELKWAAIEKLPTYLRMTRGILTESEGQLTEIDINKL
GPLQRKNLVERLVKIAEQDNENFLLKLRNRIDRVGLEIPTIEVRFEHLNVEAEAHVGSRALPTIFNFCINLLEGFL
NSLHLIPSRKKPFAVLHDVSGIIKPKRMTLLLGPPSSGKTTLLLALAGRLGKDLKFSGRVLYNGHGMKEFVPQR
TSAYISQADLHIGEMTVRETLAFSARCQGIGSRYEMLAELSRREKAANIKPDPDIDMYMKAAALEGQETNVVT
DYIMKILGLEVCADTMVGDDMIRGISGGQKKRVTTGEMLVGPARALFMDEISTGLDSSTTFQMVNSLRQSIHIL
NGTAVISLLQPAPETYELFDDIILLSDGQIVYQGPRENVLEFFEFMGFKCPDRKGVADFLQEVTSRKDQEQYWA
KQDESYTFVTVKEFAEAFQSFHVGRKLGDELATPFDTSKGHPALLTKNKYGVCKKELLKACVSREFLLMKRN
SFVYIFKMWQLILTGFVTMTLFLRTEMHRDTETDGGIFMGALFFVLIVIMFNGYSELSMSIMKLPVFYKQRDLL
FFPSWAYSLPTWILKIPITLVEVGIWVVMTYYVIGFDPSIERFLKQYFLLVCINQMASALFRFMGAVGRNIIVAN
TVGSFALLAVMVMGGFILSRVDVKKWWLWGYWFSPMMYAQNAIAVNEFLGKSWSHALPNSTEPLGVKVLK
SRGIFPEAYWYWIGVGASIGYMLLFNLLLPLALHYLDRKYLTVHVFATAYGKPQAIISEESLAERNAGKNEHIM
ELSSRFKGSSGNESRRSVSSRTLSARVGSIGARDHSRKRGMVLPFTPLSITFDEIRYAVDMPQEMKNQGILEDRL
ELLKGVNGAFRPGVLTALMGVSGAGKTTLMDVLSGRKTAGYILGQITISGYPKKQETFARIAGYCEQTDIHSPH
VTVYESLVYSAWLRLPPEVDSATRQMFIEEVMELVELTSLREALVGLPGVNGLSTEQRKRLTIAVELVANPSIIF
MDEPTSGLDARAAAIVMRTVRNTVDTGRTVVCTIHQPSIDIFDAFDELLLLKRGGEEIYVGPLGQHCSHLINYFE

GINGVPKIKNGYNPATWMLEVTSEAEEEALGVNFAELYKNSDLYRRNKALIRELSTPPAGSKDLYFPTTYSQTF
FTQCMACLWKQHLSYWRNPPYSAVRLLFTTTIALLFGTIFWDIGSKRQRKQDLFNAMGSMYAAVLFIGIQNST
SVQPVVAIERTVFYRERAAGMYSALPYAFGQVAIEIPYILIQTLVYGIIVYAMIGFEWTFIKFFWYLFFMFFTFLY
FTFYGMMAVGLTPDHNIAAIVSFGFYLIWNLFSGFVIPRTRMPVWWRWYFWICPVSWTLYGLVTSQFGDIKER
IETGETVDEFVRSYFGYRDDFLGLAAVVVVGFSLLFGFTFAFSIKAFSFQKR

>XP_020205458.1_PDR1-l_X2_Ccaj
MESGELRVASARIGSSSMWRSSVVDVFSGSSRRDDDEEELKWAAIEKLPTYLRMTRGILTESEGQLTEIDINKL
GPLQRKNLVERLVKIAEQDNENFLLKLRNRIDRVGLEIPTIEVRFEHLNVEAEAHVGSRALPTIFNFCINLLEGFL
NSLHLIPSRKKPFAVLHDVSGIIKPKRMTLLLGPPSSGKTTLLLALAGRLGKDLKFSGRVLYNGHGMKEFVPQR
TSAYISQADLHIGEMTVRETLAFSARCQGIGSRYEMLAELSRREKAANIKPDPDIDMYMKAAALEGQETNVVT
DYIMKILGLEVCADTMVGDDMIRGISGGQKKRVTTGEMLVGPARALFMDEISTGLDSSTTFQMVNSLRQSIHIL
NGTAVISLLQPAPETYELFDDIILLSDGQIVYQGPRENVLEFFEFMGFKCPDRKGVADFLQEVTSRKDQEQYWA
KQDESYTFVTVKEFAEAFQSFHVGRKLGDELATPFDTSKGHPALLTKNKYGVCKKELLKACVSREFLLMKRN
SFVYIFKMWQLILTGFVTMTLFLRTEMHRDTETDGGIFMGALFFVLIVIMFNGYSELSMSIMKLPVFYKQRDLL
FFPSWAYSLPTWILKIPITLVEVGIWVVMTYYVIGFDPSIERFLKQYFLLVCINQMASALFRFMGAVGRNIIVAN
TVGSFALLAVMVMGGFILSRVDVKKWWLWGYWFSPMMYAQNAIAVNEFLGKSWSHALPNSTEPLGVKVLK
SRGIFPEAYWYWIGVGASIGYMLLFNLLLPLALHYLDPYGKPQAIISEESLAERNAGKNEHIMELSSRFKGSSVG
SIGARDHSRKRGMVLPFTPLSITFDEIRYAVDMPQEMKNQGILEDRLELLKGVNGAFRPGVLTALMGVSGAGK
TTLMDVLSGRKTAGYILGQITISGYPKKQETFARIAGYCEQTDIHSPHVTVYESLVYSAWLRLPPEVDSATRQM
FIEEVMELVELTSLREALVGLPGVNGLSTEQRKRLTIAVELVANPSIIFMDEPTSGLDARAAAIVMRTVRNTVDT
GRTVVCTIHQPSIDIFDAFDELLLLKRGGEEIYVGPLGQHCSHLINYFEGINGVPKIKNGYNPATWMLEVTSEAE
EEALGVNFAELYKNSDLYRRNKALIRELSTPPAGSKDLYFPTTYSQTFFTQCMACLWKQHLSYWRNPPYSAVR
LLFTTTIALLFGTIFWDIGSKRQRKQDLFNAMGSMYAAVLFIGIQNSTSVQPVVAIERTVFYRERAAGMYSALP
YAFGQVAIEIPYILIQTLVYGIIVYAMIGFEWTFIKFFWYLFFMFFTFLYFTFYGMMAVGLTPDHNIAAIVSFGFY
LIWNLFSGFVIPRTRMPVWWRWYFWICPVSWTLYGLVTSQFGDIKERIETGETVDEFVRSYFGYRDDFLGLAA
VVVVGFSLLFGFTFAFSIKAFSFQKR

>XP_020223558.1_PDR1-l_Ccaj
MEGSDIYRASNSLRARSSTVWRNSGVEVFSKSSREEDDEEALKWAALEKLPTYNRLRKGLLTASHGVANEIDV
TDLDYQEKQKLLERLVKVAEEDNEKFLLKLKERIDRVGLDIPTIEVRYEHLNIEAEAFVGSRALPSFINSITNVVE
GFLNLLHIIPTKKKHVTILKDVSGIIKPRRMTLLLGPPSSGKTTLLLALSGKLDKSLKVSGKVTYNGHGMNEFVP
RRTAAYISQHDVHIGEMTVRETLAFSARCQGVGSRYDMLSELSRREKAANIKPDPNLDVYMKATATAGQESSI
VTDYTMKILGLDICADTMVGDEMLRGISGGQRKRVTTGEMLVGPANALFMDEISTGLDSSTTFQIVSSLRQYV
HILNGTAVISLLQPAPETYDLFDDIILVSDGQVVYHGPREYVLDFFESMGFKCPERKGVADFLQEVTSKKDQAQ
YWARRDQPYRFVTVTQFAEAFQSFHIGRKLAEELTVPFDKTKSHPAALTTEKYGINKKELLKANFSREYLLMK
RNSFVYIFKLCQLFVIALIAMTLFFRTEMHHENQDDAGVYAGALFFTLITIMFNGMAEISMTIAKLPVFYKQRDL
LFYPSWAYAIPSWILKIPITVVEVALWVFLTYYVIGFDPNVGRLFKQYLVLLFMSQMASGLFRAIAALGRNMIV
ANTFGSFVVLTLVALGGFILSKKDIKNWWIWGFWISPMMYGQNAMMVNEFLGNSWHNATRNLGVEFLVTRS
FFTDSYWYWLGFGALVGFVFLFNVMFGVALELLGPFDKPQATITEEEPEANTSNEGIELPRIESSGKADSITESS
HGKKKGMVLPFEPHSITFDEIVYSVDMPQEMKEQGVQEDKLVLLKGVSGAFRPGVLTALMGVSGAGKTTLM
DVLAGRKTGGYIEGSIKISGYPKKQETFARISGYCEQNDIHSPHVTVYESLLYSAWLRLPSSVDSKTRKMFIEEV
MELVELNPLRNSLVGLPGVSGLSTEQRKRLTIAVELVANPSIIFMDEPTSGLDARAAAIVMRTVRNTVDTGRTV
VCTIHQPSIDIFEAFDELFLMKRGGQEIYVGPLGRHSSHLIKYFESIKGVSKIKDGYNPATWMLEVTTTAQELSL
GVDFTDLYKNSDLHRRNKQLIQELGQPAPGSKDLYFPTQFSQSFLVQCQACLWKQRWSYWRNPPYTAVRFFF
TTFIALMFGTMFWDLGGKHSSRQDLLNALGSMHTAVLFLGIQNSSSVQPVVAVERTVFYREKAAGMYSALPY
AFAQILVELPYVFAQAVTYGLIVYAMIGFEWTAEKFFWYLFFMYFTFLYFTFYGMMSVAVTPNHHVASIVAA
AFYAIWNLFSGFVVTRPKIPIWWRWYYWACPVAWTIYGLVASQFGDIMEPMSSENDKLVKYFIEDYFGIKHDF
VGVCAVVVAGIAVLFAFIFAVAIKTFNFQKR

>XP_020985459.1_PDR1-l_Ad
MENGELRVASARLGSSRVWSSGAVDVFSGSSRRDDDEEQLKWAAIEKLPTYRRLTRGILTESQGEYTEIDINEL
GPLQRKKLVERLVKIAEQDNEKFLLKLRRRIDRVGLDIPMIEVRFEHLNVEAEAHVGSRALPTNLNFCINLLEGI

LNSFRILPSRKKPFTVLHDVSGIIKPRRMTLLLGPPSSGKTTLLLALAGRLGKDLKVRTCDVSKFSGRVSYNGNG
MEEFVPQRTSAYISQTDLHIGELTVRETLAFSARCQGIGTRYEMLAELSRREKAQSIKPDPDLDIYMKAAALEG
QETNVVTDYIMKILGLEVCADTMVGDEMIRGISGGQKKRVTTGEMLVGPARALFMDEISTGLDTSTTFQMVNS
LRQSIHILNGTAVISLLQPAPETYELFDDVILLSDGQIVYQGPRENVLEFFEYMGFKCPNRKGEADFLQEVTSRK
DQEQYWANNDKPYTFVSVREFADAFRSFHIGRKLGNELATPFDKTRGHPAVLTKNKYGVSKRELLRACVSRE
FLLMKRNSFVYIFKMCQLTLTGIITMTLFLRTEMHRNTVSDGGIYMGALFFGLIMIMFNGFSELSMTIAKLPVFY
KQRDLLFYPSWAYSLPLWILKIPITLVEVGIWVVMTYYVIGFDPNIERFIKQYLLLACINQMAAALFRFMGAVG
RNLVVANAFGIFALIAIMVMGGFVLSRVDVRKWWLWGYWTSPMMYGQNALAVNEFLGNSWKHVPPNATEP
LGVQVLKARGLFPEAYWYWIGVGASIGYMLLFNLLFPLALHYLDPFGKPQALISEEALAERNSVKNDHIIELSS
GRNGASDKSNGRSVSSRTLSARVVAINETDRYRKCGMVLPFTPLSITFDEIRYAVDMPQEMTAQGIVEDKVELL
KGISGAFRPGVLTALMGVSGAGKTTLMDVLSGRKTAGYIQGQITISGYPKKQETFARISGYCEQTDIHSPHVTV
YESLVYSAWLRLPPEVDSSTREMFIEEVMELVELNSLRDALVGLPGVNGLSTEQRKRLTIAVELVANPSIIFMDE
PTSGLDARAAAIVMRTVRNTVNTGRTVVCTIHQPSIDIFDVFDELLLLKRGGEEIYVGPLGRHASHLISYFEGIN
GVPKIKNGYNPATWMLEVTSEAQEEALGINFAELYKNSDLYRGNKALISELSIPPSDSKDLYFPTKYSQSFIAQC
KACLWKQNLSYWRNPPYTAVRLLFTTFLALLFGTIFWDFGSKRKRAQDIFNAMGSMYAAVLSTGVQNASSVQ
PVVAIERTVFYREKAAGMYSALPYAFGQVAIEIPYILLQTLAYGVIVYAMIGFEWAAAKFFWYLFFMFFTFFYY
TLYGMMAVGITPDHHIAAIVSLGFYLIWNLFSGFVIPRTRMPVWWRWYFWICPVAWTMYGLVTSQFGDVTER
LETGETVGDFVERYFGYRDDFIGIAAAVVVGFTLLFGFTFAFSIKAFNFQKR

>XP_021277311.1_PDR1-l_X1_Hu
MESGTDAFRVSSARIGSSSIWRNNTMEAFSKSSREEDDEEALKWAAIEKLPTYLRVRRGILTEEEGQSREVDIKD
LGFIERRNLLERLVKIAEEDNEKFLLKLKERIERVGLDMPTIEVRFEHLNVEVEAYVGSRALPTMFNFSANILEG
LLSYLRILPSRKKPLPILNDVSGIIKPRRMTLLLGPPSSGKTTLLLALAGKLGKDLKFSGRVTYNGHGMEEFVPQ
RTSAYISQYDLHIGEMTVRETLAFSARCQGVGPRYGMLAELSRREKEANIKPDPDIDIYMKAAALEGQEASVV
TDYILKILGLEVCADTMVGNEMIRGISGGQKKRVTTGEMLVGPARALFMDEISTGLDSSTTYQIVNSLRQSIHIL
NGTALISLLQPAPETYDLFDDIILLSDGQIVYQGPRENVLEFFETMGFKCPERKGVADFLQEVTSRKDQEQYWA
RKDEPYSFVSVKEFAEAFQSFHIGQKLGDDLAIPFDKSKSHPAALTKEKYGVSKKDLIKACVSREYLLMKRNLF
VYVFKMIQLIIMGLITMTLFLRTKMHRDTMTDGAIFMGALFFILIMIMFNGFAELAMTIMKLPIFYKQRDLLFYP
SWAYSLPAWILKIPISVLEVTIWVFMTYYVIGFDPDAGRFFKHYLILLCLSQMASGLFRLMGGLGRNIIVANTCG
SFALLAVLVMGGFILTRDNVKKWWIWGYWISPLMYGQNAIAVNEFLGKSWRLIPPNSTEPLGVSILKSRGIFPE
AHWYWIGVGALIGYCFLFNFLFTLTLKYLDPFGKPQAVISKETLAEKIVSKTGENAELSLRGKGSSERRIESRRS
ASSRSLSSKVGSINEANQNRKRGMVLPFEPLSMSFDEIKYAVDMPQEMKAQGVSEDRLELLKGVSGAFRPGVL
TALMGVSGAGKTTLMDVLAGRKTGGYVEGTIKISGYPKKQETFARISGYCEQTDIHSPHVTVYESLLFSAWLR
LPPEVDSETRMMFIEEVMELVELTSLREALVGLPGVNGLSTEQRKRLTIAVELVANPSIIFMDEPTSGLDARAAA
IVMRTVRNTVDTGRTVVCTIHQPSIDIFDAFDELLLLKRGGEEIYVGPLGRHSCHLIKYFEEITGIPRIKDGYNPA
TWMLEVTSAAQEETIGVNFTNIYKNSELYRRNKALVKELSSPAPGSKDLYFLTRYSQSLITQCMACLWKQYWS
YWRNPPYTAVRFLFTTVIALLFGTIFWDLGSKRTRQQDVLNSMGSMYAAVLFIGFQNSAAVQPVVAVERTVFY
RERAAGMYSALPYAFGQVVVELPYVLVQTVIYGVIVYAMIGFEWTAAKFFWYLFFMYFTFLYFTFYGMMTV
AVTPNHNIAAIVSSAFFALWNLFSGFIIPRTRIPVWWRWYYWVCPISWTLYGLIASQYGDIKETFDSGETVEHFV
RNYFGFRDEFVGVVAIVVVGICVLFGFIFAFSIKAFNFQKR

>XP_021277312.1_PDR1-l_X2_Hu
MESGTDAFRVSSARIGSSSIWRNNTMEAFSKSSREEDDEEALKWAAIEKLPTYLRVRRGILTEEEGQSREVDIKD
LGFIERRNLLERLVKIAEEDNEKFLLKLKERIERVGLDMPTIEVRFEHLNVEVEAYVGSRALPTMFNFSANILEG
LLSYLRILPSRKKPLPILNDVSGIIKPRRMTLLLGPPSSGKTTLLLALAGKLGKDLKFSGRVTYNGHGMEEFVPQ
RTSAYISQYDLHIGEMTVRETLAFSARCQGVGPRYGMLAELSRREKEANIKPDPDIDIYMKAAALEGQEASVV
TDYILKILGLEVCADTMVGNEMIRGISGGQKKRVTTGEMLVGPARALFMDEISTGLDSSTTYQIVNSLRQSIHIL
NGTALISLLQPAPETYDLFDDIILLSDGQIVYQGPRENVLEFFETMGFKCPERKGVADFLQEVTSRKDQEQYWA
RKDEPYSFVSVKEFAEAFQSFHIGQKLGDDLAIPFDKSKSHPAALTKEKYGVSKKDLIKACVSREYLLMKRNLF
VYVFKMIQLIIMGLITMTLFLRTKMHRDTMTDGAIFMGALFFILIMIMFNGFAELAMTIMKLPIFYKQRDLLFYP
SWAYSLPAWILKIPISVLEVTIWVFMTYYVIGFDPDAGRFFKHYLILLCLSQMASGLFRLMGGLGRNIIVANTCG
SFALLAVLVMGGFILTRDNVKKWWIWGYWISPLMYGQNAIAVNEFLGKSWRLIPPNSTEPLGVSILKSRGIFPE
AHWYWIGVGALIGYCFLFNFLFTLTLKYLDPFGKPQAVISKETLAEKIVSKTGENAELSLRGKGSSGGRIESRRS
ASSRSLSSKVGSINEANQNRKRGMVLPFEPLSMSFDEIKYAVDMPQEMKAQGVSEDRLELLKGVSGAFRPGVL

TALMGVSGAGKTTLMDVLAGRKTGGYVEGTIKISGYPKKQETFARISGYCEQTDIHSPHVTVYESLLFSAWLR
LPPEVDSETRMMFIEEVMELVELTSLREALVGLPGVNGLSTEQRKRLTIAVELVANPSIIFMDEPTSGLDARAAA
IVMRTVRNTVDTGRTVVCTIHQPSIDIFDAFDELLLLKRGGEEIYVGPLGRHSCHLIKYFEEITGIPRIKDGYNPA
TWMLEVTSAAQEETIGVNFTNIYKNSELYRRNKALVKELSSPAPGSKDLYFLTRYSQSLITQCMACLWKQYWS
YWRNPPYTAVRFLFTTVIALLFGTIFWDLGSKRTRQQDVLNSMGSMYAAVLFIGFQNSAAVQPVVAVERTVFY
RERAAGMYSALPYAFGQVVVELPYVLVQTVIYGVIVYAMIGFEWTAAKFFWYLFFMYFTFLYFTFYGMMTV
AVTPNHNIAAIVSSAFFALWNLFSGFIIPRTRIPVWWRWYYWVCPISWTLYGLIASQYGDIKETFDSGETVEHFV
RNYFGFRDEFVGVVAIVVVGICVLFGFIFAFSIKAFNFQKR

>XP_021606416.1_PDR1-l_Me
MEGGDITSRVSSARLSSSNIWRNTTLEVFSKSSSCNEDDEEALKWAALEKLPTYLRVRRGILTEEEGQSREIDIN
NLGFIEKRNLLERLVKIAEQDNEKFLLKLKNRIEKVGLDMPTIEVRFEHLTVETEAYVGSRALPTMFNFSANMF
EGFLNYLHILPSRKKPLSILNDVSGIIKPRRMTLLLGPPSSGKTTLLLALAGKLGKELKFSGKVTYNGHGMDEFV
PQRTSAYISQYDLHIGEMTVRETLAFSARCQGVGTRYEMLAELARREKAANIKPDPDIDIYMKAAALEGQEAN
VVTDYILKILGLEICADILVGDEMVRGISGGQKKRVTTGEMLVGPARALFMDEISTGLDSSTTFQIVNSLRQSIHI
LNGTALVSLLQPAPETYDLFDDIILLSDGQIVYQGPRENVLEFFEYMGFRCPERKGVADFLQEVTSKKDQEQY
WAFKDQPYSFVSVKEFAEAFQSFHVGRKLGDELATPFDKSKSHPASLTTKKYGVSKKELLKACISREYLLMKR
NSFVYIFKMTQLIIMAFVSMTIFLRTEMRRNTVADGGIYMGALFYTIIIIMFNGFSELAMTIMKLPVFYKQRDLLF
YPAWAYALPTWILKVPVTFVEVAVWVVMTYYVIGFDPNIGRFLKQYFILLITNQTSSALFRLTAALGRSVIVAN
TVGSFALLAVLILGGFIISRDSVKKWWIWGYWFSPMMYVQNGMSVNEFLGNSWNHFPPNSTEPLGVTVMKSR
GLFPEAYWYWIAVGALTGYIFLFNFLFTLALKYLDPFGRPQATISEEAYAEKTANETAEFIEQSSKGESSLEKGS
VSQRSASSRTPSTRVGSFSDVNQNRRRGMILPFQPLSITFDEIKYVVDMPQEMKAQGITEDRLELLKGVSGAFRP
GVLTALMGVSGAGKTTLMDVLAGRKTGGYIHGSISISGYPKKQETFARISGYCEQTDIHSPHVTVYESLLYSAW
LRLPPEVDSDTRKMFIEEVMELVELTNLRVALVGLPGVNGLSIEQRKRLTIAVELVANPSIIFMDEPTSGLDARA
AAIVMRTVRNTVDTGRTVVCTIHQPSIDIFDAFDELLLLKRGGEEIYVGPVGRHACHLIKYFEDIEGIPKVKDGY
NPATWMLEVTTAAQEVSLGINFSDIYKNSELYRRNKALIKELSIPPPGSRDLYFPTQYSQSFFTQCMACLWKQH
WSYWRNPPYSAVRLLFTTFIALMFGTIFWNLGSKRSRKQDLFNAMGSMYAAILFLGFQNSTSVQPVVAIERTV
FYRERAAGMYSELPYAFGQVVIELPYILVQTLIYGGIVYAMLGFEWTASKFLWYLFIMYFTLAYFTFYGMMTV
AITPNHNIAAIISSAFYGIWNLFSGFIIPRTRMPVWWRWNYWACPIAWTLYGLIASQYGDIKEELDSGETVEHFL
RSYFGFRHDFVGIVAIVLVGIAVFFGFTFAFSIKAFNFQHR

>XP_021609606.1_PDR1-l_Me
MDGADLYRASSSIRRSSSIWRNNSADVFSRSSREEDDEEALKWAVLEKLPTYDRLRKGILISGSKGEANEVEIDS
LGFQERKSLLERLVRVTEEDNEKFLLKLKDRIDRVGIEVPTIEVRFEHLNIEAEALVGTSALPTFFNFLINILEGFL
NNLHVFPSRKKPFTILKDISGVIKPSRMTLLLGPPSSGKTTLLLALAGKLDPNLKFSGSVTYNGHGLNEFIAQRT
AAYISQHDLHIGEMTVRETLGFSARCQGVGCLQDMLAELSRREKAANIKPDPDIDVFMKAAATEGQEASLVTD
YILKILGLDVCADTLVGDEMLRGISGGQRKRVTTGEMLVGPSRALFMDEISTGLDSSTTYQIVNSLKQSIHILNG
TAVISLLQPAPETYDLFDDIILLADGQIVYQGPREDVLGFFEYMGFKCPERKGVADFLQEVTSRKDQQQYWAN
RDQPYSFISVQEFSEAFQSYDVGQRLGQELSTPFDKAKSHPAALPTVKYGVGMVELLKACFSREYLLMKRNSF
VYIFKLIQLTTMAIIGMTLFLRTNMHRDNLIDGGIYLGALFFSVVMIMFNGMSELSMTIAKLPVFYKQRDLLFYP
SWAYSLPSWILKIPISFLEVAVWVFITYYVMGFDPNVGRLFKQYILLFLVNQMASALFRFIAAVGRNMIVANTF
GSFALLILFALGGVVLSREEIKKWWIWGYWLSPMMYGQNAIVVNEFLGKSWSHIPPNSTESLGVLLMKSRGFF
PHAYWYWIGVGASAGFVLLFNLCFTLALTFLNPFEKPQAVISDEPETSGRLESRHTTNTENEMSDIDESNHKKK
KGMVLPFEPHSITFDNVIYSVDMPQEMRNQGIAEDKLVLLKGVSGAFRPGVLTALMGVSGAGKTTLMDVLAG
RKTGGYIEGSIKISGYPKKQETFARISGYCEQNDIHSPHVTVYESLIYSAWLRLPPEVDSETRKMFVDEVMELVE
LNPLRQALVGLPGVNGLSTEQRKRLTIAVELVANPSIIFMDEPTSGLDARAAAIVMRTVRNTVDTGRTVVCTIH
QPSIDIFEAFDELFLMKRGGEEIYVGPLGRHSSRLINYFEEINGVSKITDGYNPATWMLEVTSSAQELSLGVDFA
VIYKNSELYRRNKATIEALSTPAPGSKDLYFPTQYSQSFFTQCLACLWKQRLSYWRNPPYTAVRFLFTTFISLMF
GTMFWDLGTKTSKQQDIFNSLGSMYAAVLFIGIQNAASVQPVVAVERTVFYRERAAGMYSAMPYAYAQVLIE
LPYIFIQAAVYGLITYAMIGFEWTAAKFFWYLFFMYFTLLYFTYYGMMTVAVTPNQHIASIISSAFYAIWNLFSG
FIIPRTRMPVWWRWFYWVCPVSWTLYGLIASQFADIKDPIEGGVTVEQFVKAYYGVKHDFLGVVAAMIVGFT
VLFAFIFAVSVRSFNFQKR

>XP_021610343.1_PDR1-l_Me
MEGDHYRASNSLRRGSSSVWRNNGLDVFSRSSREEDDEEALKWAALEKLPTYDRLRKGILVSVSKGGANEID
VDNLGFQERKTLLERLVKVAEEDNEKFLLKLKNRIDRVGIEVPTIEVRYEHLNVEAEALVGSNALPSFLNFSFSL
VEGLFRYILPNRKRPLTILKDVSGVIKPSRMTLLLGPPSSGKTSLLLALAGKLDPSLKVSGTVTYNGHSLNEFIPQ
RTAAYISQHDLHIGEMTVRETLAFSARCQGVGTQHEMLAELSRREKAANIKPDPDLDVFMKAAATEGQETSV
VTDYVLKILGLDICADTLVGNEMIRGISGGQRKRVTTGEMLVGPAKALFMDEISTGLDSSTTFSIVNSLKQSIHIL
NGTAVISLLQPAPETYNLFDDIILISDGQIVYQGPRENVLEFFEYMGFKCPERKGVADFLQEVTSKKDQQQYWA
RKDQPYRFVTVNEFAEAFQSYEVGRKIAEDLSVPFDRTKNHPAALTTKPYGVGKMELIKANFSREYLLMKRNS
FVYVFKLTQLIVMAIIGMTLFFRTEMKHDSFEDAGVYAGALFFTLITIMFNGMAELSMTIAKLPVFYKQRNLLF
FPAWSYAIPSWILKIPVTFLEVGVWVFITYYVIGFDPNVGRLFKQYMLLLLVNQMASGLFRFIASVGRNMIVAN
TFGSFALLTLFALGGFVLKRSDIKKWWIWGYWVSPLMYGQNAIVANEFLGNSWNHIPANSTSTDSLGVQFLKT
NGFFPHAYWYWLGVGASAGYILVFNLLYTVALTFLDQFEKPQAVISDEPEESNRSGGAIQLSQAESSHRTQTES
GTSGIDESNHNKKKGMVLPFEPHSITFDNVIYSVDMPQEMKSQGVVEDKLVLLKGVSGAFRPGVLTALMGVS
GAGKTTLMDVLAGRKTGGYIEGDIRISGYPKKQETFARISGYCEQNDIHSPYVTVYESLVYSAWLRLPPDVDSE
TRRMFVEEVMELVELNPLRQALVGLPGVNGLSTEQRKRLTIAVELVANPSIIFMDEPTSGLDARAAAIVMRTVR
NTVDTGRTVVCTIHQPSIDIFEAFDELFLMKRGGEEIYVGPLGRHSCHLIKYFEGIGGVSKITDGYNPATWMLEV
SSSAQELTLGVDFANVYRNSDLYRRNKAMIQELSKPAPGTEDLYFPTQYSQPFLTQCMACLWKQSWSYWRNP
PYTGVRFWFTTFIALMFGTIFWDLGSKMEKEGDLTNAMGSMYAAVLFLGVQNSSSVQPVVAVERTVFYREKA
AGMYSAMPYAYAQALIELPYIFAQAGVYSLITYAMIGFEWTAAKFFWYLFFLYFTLLYFTFYGMMTVAVTPN
HHIASIVSSAFYSIWNLFSGFIVPRPKMPVWWRWYYWGCPISWTLYGLFASQFADITKPLGTTGKTVEEYVNET
YGIKHDFLGASAGVIFGIALLFAVIFAVSIKAFNFQRR

>XP_021610345.1_PDR1-l_Me
MESADLYRASSSLRRGSFSTWRNNPSDVFTRSSREEDDEEALKWAALEKLPTYDRLRKGIFLSASKGAVNEIDV
DNLGFQERKTLLERLVKVAEEDNEKFLLKLKNRIDRVGIEIPTIEVRFEHLNIEADAYVGSSALPSFINFSVHMLE
GFLNYLHVLPSRKRPLTILKDVSGVIKPSRMTLLLGPPSSGKTTLLLALAGKLDPNLKFSGTVTYNGHGMDEFIP
QRTAAYISQHDVHIGEMTVRETLAFSARCQGVGTQHELLAELSRREKDANIKPDPDIDVFMKAAATEGQETSV
VTDYILKILGLEICADTLVGNEMIRGISGGQRKRVTTGEMLVGPAKALFMDEISTGLDSSTTYQIVNSLKQSIHIL
NGTAVISLLQPAPETYDLFDDIILLSDGQIVYQGPREQVLGFFEYMGFKCPERKGVADFLQEVTSRKDQKQYW
ARRDQPYSFVTVQEFAEAFQSYDVGRRIGDELSTPFDKTKSHPAALSTKKFGVGKMELLKACMSREYLLMKR
NSFVYIFKLTQLTFMAIIMMTLFLRTEMHRDNIMDGGVYLGALFFTVIMVMFNGMAELSMTIAKLPVFYKQRE
LLFYPAWAYSIPTWILKIPVTFVEVAVWVFLTYYVVGFDPNVTRFFKHYFVLLLVNQMASALFRCIAATGRNVI
VANTFGSFSLLTLFALGGFVLSRDEIKKWWIWGYWMSPLMYGQNAIVANEFLGKSWSHIPPNSTESLGVQVM
KGRGFFPDAYWYWLGAGASAGFIIVFNICFALALTFLDPFEKPQAVITEDSQSNDPDDEDGGDILLTNNGSSHK
SSTGAGEEIRQVNHNKKKGMVLPFEPHSVAFDNVVYSVDMPQEMKSQGVLEDKLVLLKGVSGAFRPGVLTAL
MGVSGAGKTTLMDVLAGRKTGGYIEGNITISGFPKKQETFARISGYCEQNDIHSPHVTVYESLVYSAWLRLPPE
VDSKTRKMFVDEVMELVELNPLRQALVGLPGVSGLSTEQRKRLTIAVELVANPSIIFMDEPTSGLDARAAAIV
MRTVRNTVDTGRTVVCTIHQPSIDIFDAFDELFLMKRGGEEIYVGPLGRHSCHLIKYFEGIEGVSKITDGYNPAT
WMLDITSYAQELALNVDFAAIYKNSELYRRNKAMIMELSTPAPGSKDLYFPTQYSQSFLTQCIACIWKQRLSY
WRNPPYTAVRFLFTTFIALMFGTMFWKIGSKLKKQQDLFNAAGAMYAAVLFLGVQNASSVQPVVAIERTVFY
RERAAGMYSAMPYAYAQVLVELPYVFAQAIFYGTITYAMMGFEWTIAKFFWYLFFMYFTLLYFTLYGMMTV
AVTPNHHIAAIVSSAFYGMWNIFAGFILPRTRMPVWWRWYFWICPVAWTLYGLIASQFGDVKDVLANGQTVE
DFIREYYGYKHDFVGVTACVIVGIVVLFAFIFGISIRSFNFQRR

>XP_021610859.1_PDR1-l_Me
MEGPEVYVGGGSFRRGDSSIWRSNAMDTFSKSSREEDDEEALKWAALERLPTYDRLKKGILTTSKGEASEIYV
QDLGFQERRTLVDRLVNVAEEDNERFLLNLKNRIERVGIELPTIEVRFEHLNIETEAHVGNRALPTFINFSIDMVE
GFLNKLHILPSRKKRLSILQDISGIIKPRRMTLLLGPPSSGKTTLLLALAGKLDPKLKFSGRVTFNGHEMNEFVPQ
RTAAYISQYDKHIGEMTVRETLAFSARCQGVGHRYEMLTELLRREKASNIKPDSDLDVFMKAIATEGQETSVIT
DYILKVLGLEVCADIMVGNEMLRGVSGGQRKRVTTGEMLVGPAKALFMDEISTGLDSSTTFQIVNSIKQYIKIL
NGTAVISLLQPAPETYDLFDDIILLSDGWIVYQGPREHVLEFFEFMGFKCPERKGVADFLQEVTSRKDQQQYWT
RKDDPYCFVTVQQFSEAFQSFHVGRNLQAELSTPFDKTKSHPAALTTKKYGVGKMELLKACFSRELLLMKRN
SFVYIFKLSQLTIMAIIAMTLFLRTEMHRESVIDGGIYVGALFYSVVFIMFNGLSEISMTISKLPVFYKQRNLLFYP

AWAFSLPPWIIKIPITLVQVALWVFITYYVIGYDPYVGRLFRQYLLLVLVSQMASALFRFIAAAGRDMIVANTF
GSFALLTLFALGGFILSRDNIKKWWIWGYWISPLMYGQNAIVVNEFLGKSWSRVLPNSSEPLGVEVLKSRGFFT
NAYWYWIGVGALVGFTLLYNLCFTLALTFLGPLQKPQAVISEDSPSDESGSDHQTSAKSGSGSGSSSARAEVRV
NSSHQNKKGMVLPFEPHFITFDEIRYSVDMPQEMKNQGVTEDKLELLRGVSGSFRPGVLTALMGVSGAGKTTL
MDVLAGRKTGGYIEGNITISGYPKKQETFARISGYCEQNDIHSPHVTVYESLLYSAWLRLSPDVSSETRKMFIEQ
VMHLVELEPLRQALVGLPGVSGLSTEQRKRLTIAVELVANPSIIFMDEPTSGLDARAAAIVMRTVRNTVDTGRT
VVCTIHQPSIDIFEAFDELFLMKRGGQEIYVGPLGRHSCHLIKYFEGIEGVPNIKDAYNPATWMLEVTSSAQESV
LGVDFAAVYRNSELYRSNKEMIEKLSTPAPDSKDLYFPSKYSQSFFTQCMACLWKQRWSYWRNPPYTAVRLL
FTTVIALMFGTMFWNLGSKTKKRQDLFNAMGSMYAAIVFLGVQNASSVQPVVAVERSVFYRERAAGMYSPL
PYAFAQVLIELPYIFIQSVVYGLIVYAMIGFEWNAAKFFWYLFFMYFTLLYYTFYGMMSVAATPNQHVGAIVS
SAFYSLWNLFSGFIIPRPRIPVWWRWYAWACPVAYTLYGLISSQFGDLKHTLESGETVEDFVRSYFGFRHELLG
AVAAAVVGFATLFAFIFAICIKFFNYQRR

>XP_021627417.1_PDR1-l_Me
MEGADHYRASNSFKRNSFIWRNNSFQNSFVWRTDTCEAFSKSCREEDDEEALKWAALEKLPTYDRLRKGILVS
VSKGGANEIDVDALGFHDRQRLLERLVKVAEEDNEKFLLKLRRRIDRVGIELPKVEVRFENLNVEAEAFVGNR
ALPTFVNFCVNIIEGCLNSLHILPSRKKPLTILKDVSGVIKPSRMTLLLGPPSSGKTTLLLALAGKLDPDLKLSGS
VTYNGHGINEFIPQKTAAYISQHDLHIGEMTVRETLAFSARCQGVGWQHEMLAELSRREKASNIKPDPDIDVF
MKAAAIEGQESSVVTDYILKILGLEVCADTMVGDDMLRGISGGQRKRVTTGEMLVGPSKALFMDEISTGLDSS
TTFQIVNSLRQTVHILNGTAVISLLQPAPETYDLFDDIILLSDGQIVYQGPREDVLGFFEHMGFKCPERKGVADF
LQEVTSRKDQKQYWAHKDQPYSFVTVQEFVEAFQSYAVGRRIGQELSTPFDKSKSHPAALATGKYGVGKMEL
LKACLSREYLLMKRNSFVYFFKLFQLSLMGIIAMTLFLRTNMHRNDLNGGGIYLGALFFTVIMIMFNGMSELS
MTVAKLPVFYKQRDLLFYPSWAYSLPTWILKIPITFFEVAVWVLLTYYPIGFDPNVVRLLKQYILLLFVNQMAS
ALFRFIAAAGRNMIVANTFGSFALLIIFALGGVILSRDDIKKWWIWGYWISPMMYGQNAIVANEFLGKSWSHIP
PNSTESLGVQVLKSRGFFPQAHWFWLGVGALAGFIIVFNICFTLALTYLNPFDKPQAVISDEPEHSERTNTEDGT
SGINEAKHKKKGMVLPFEPHSITFDNVIYSVDMPQEMKNQGVLDDKLVLLKGVSGAFRPGVLTALMGVSGAG
KTTLMDVLAGRKTGGYIEGDIRISGYPKKQETFARISGYCEQNDIHSPHVTVYESLLYSAWLRLPKEVNSETRK
MFVEEVMELVELNPLRQALVGLPGVNGLSTEQRKRLTIAVELVANPSIIFMDEPTSGLDARAAAIVMRTVRNT
VDTGRTVVCTIHQPSIDIFEAFDELFLMKRGGEEIYVGPLGRQSGHLINYFEEIEGVEKIQDGYNPATWMLEVSS
SAQELSLGVDFAAIYKNSELYRRNKATIEELSRPAPGSKDLYFPTQYSQSFFTQCIACLWKQRLSYWRNPPYTA
VRFLFTTIIAMMFGTMFWGLGSKTSKEQDIFNSAGSMYAAVQFLGVQNAGSVQPVVAVERTVFYRERAAGMY
SPLPYAYAQVLVELPYIFVQAVVYGLMTYAMIGYEWTAVKFLWYIFFMYFTLLYFTYYGMMSVAATPNYHIA
SISSSAFYTIWNLFSGFIIPRTRMPVWWRWYYWLNPVSWTLYGLITSQFGDMKHILEGGQTVGEFVRDYYGIN
HHFIGVVAAVVLGFTLLFAFIFAISIRFFNFQRR

>XP_021627707.1_PDR1-l_Me
MESTEVFMGGGSFRRGDSSIWRSNAMDSFSRSSREEDDEEALKWAALERLPTYDRLKKGILTTSKGEAREIDV
ENLGFQERRSLVDRLVKVAEEDNEKFLLKLRNRIDRVGIELPTIEVRFEHLKIETEAHVGSRALPTFINFSVDIVE
GFLNNLHILPSRKKRLTILQDISGIVKPRRMTLLLGPPSSGKTTLLLALAGKLDPKLKFSGRVIYNGHEMNEFVP
QRTAAYISQYDTHIGEMTVRETLAFSARCQGVGHRYEMLTELLRREKESKIKPDSDIDVFMKAIATQGQETSVI
TDYVLKILRLEVCADIRVGNEMLRGVSGGQRKRVTTGEMLVGPAKALFMDEISTGLDSSTTFQIVNSIKQYIQIL
NGTAVISLLQPAPETYDLFDDIILLSDGQIVYQGPREHVLEFFEFMGFKCPERKGVADFLQEVTSRKDQQQYWA
RKDEPYNFVRIKEFAEAFQSFHVGRHLQNDLSTPFDKAKSHPAALTTKRYGVGKMELLKACFSREILLMKRNS
FVYTFKLTQLAIMALVAMTLFLRTEMHRDSVTDGGVYVGSLFFSVAFNMFNGLSDISMTIAKLPVFYKQKNLL
FYPAWAYSIPPWITRIPLTLAQVSIWVFLTYYVIGYDPNVGRLYRQYLLLVLVSQMASALFRFIAAAGRTMIVA
NTFGSFALLVLFALGGFILSPDDIKKWWTWGYWISPLMYGQNAIVVNEFTSKSWSQVLPNSTEPLGIQVLKSRG
FFTNAYWYWIGVGALVGFTLLFNLCFTLALTFLGPLRKPQAVISEESQCNEPGIGQIKFIRHSESGRGSSTLWAE
AIDNDKHQNKRGMVLPFEPHSITFDEIRYSVDMPQEMKNQGVTEDKLELLRGVSGAFRPGILTALMGVSGAGK
TTLMDVLAGRKTGGYIKGNITISGYPKKQETFARISGYCEQNDIHSPHVTVYESLLCSAWLRLSHEVSSETRKM
FIEEVMQLMELQPLRQALVGLPGVSGLSTEQRKRLTIAVELVANPSIIFMDEPTSGLDARAAAIVMRTVRNTVD
TGRTVVCTIHQPSIDIFEAFDELFLMKKGGQEIYVGPLGPHSCHLIKYFEGIEGVPKIKDGCNPATWMLEVTSSA
QESVFGVDFTAIYGNSELYRRNKGLIERFSTPSPESTELYFPTQYSQSFFTQCMACLCKQHWSYWRNPPYTAVR
LLFTTVIALMFGTMFWDLGSKKTKRQDLFNAMGSMYAAIVFLGIQNASSVQPVVAVERTVFYRERAAGMYSP
LPYAFAQVLIELPYVFIQSVVYGLIVYAMIGFEWTAAKFFWYLFFMYFTLLYYTFYGMMSVAVTPNQHIASIVS

SAFYSIWNLFSGFIIPRPRIPVWWRWYAWACPVAYTLYGLVSSQFGDIKHTLESGETVEDFVRSYFGFKHELLG
AVAVAVFGFATLFAFIFAISIKFFNYQRR

>XP_021628885.1_PDR1-l_X2_Me
MEGDLYKASSSLRRGSSSVWRNNGLDVFSRSSREEDDEEALKWAALEKLPTYDRLRKGILVSVSKGGANEIDV
ENLGFQERKALLERLVKVAEEDNEKFLLKLKNRIDRVGIEVPTIEVRYEHLNVEAEALVGSNALPSFLNFSISIV
EGLLNYLHILPNRKRPLTILKDVSGVIKPSRMALLLGPPSSGKTTLLLALAGKLDPNLKVSGNVTYNGHALNEFI
PQRTAAYISQHDLHIGEMTVRETLAFSARCQGVGTQHEMLAELSRREKAANIKPDPDLDVFMKAAATEGQETS
VVTDYVLKILGLDICADTMVGNEMIRGISGGQRKRVTTGEMLVGPAKALFMDEISTGLDSSTTFSIVNSLRQSV
HILNGTAVISLLQPAPETYNLFDDIILLSDGQIVYQGPREHVLEFFENMGFKCPERKGVADFLQEVTSKKDQQQ
YWARKDQPYRFVTVKEFAEAFKSFEVGQTITQVLSTPFDKSKNHPAALTTKPYGVGKMELLKANFSREYLLM
KRNSFVYIFKLSQLIIMAIIGMTLFFRTEMSKDDLEGGGIYIGALFFTLITIMFNGMSELSMTIAKLPVFYKQRNIL
FFPPWAYSIPSWILKIPITFLEVGVWVFLSYYVIGFDPNVGRLFKQYLLLLLVNQMASALFRFIASVGRNMIVAN
TFGSFALLTLFALGGVILSRENIKKWWIWGYWVSPLMYGQNSILANEFLGNSWSHVPANSSSTESLGVQVLKN
GGYFPHAYWYWIGVGASAGYMFLFNFLYTVALTLLDTFEKPQAVISDEPEENDKTRGAIQLSQLESSHRTNTE
SGTSENNESSHNKKKGMVLPFEPHSITFDNVIYSVDMPQEMKHQGVVEDKLMLLKGVSGAFRPGVLTALMGV
SGAGKTTLMDVLAGRKTGGYIEGDVRISGYPKQQETFARISGYCEQNDIHSPHVTVYESLVYSAWLRLPSDVD
SETRKMFVEEVMELVELNPLRQALVGLPGVNGLSTEQRKRLTIAVELVANPSIIFMDEPTSGLDARAAAIVMRT
VRNTVDTGRTVVCTIHQPSIDIFEAFDELFLMKRGGEEIYVGPLGRHSCHLIEYFEGIEGVSKITDGYNPATWML
EVSSSAQELTLGVDFANIYRNSDLYRRNKEMIQELSKPAPGTEDLYFPTQYSQPFLTQCIACLWKQSWSYWRN
PPYTAVRFWFTTFIALMFGTIFWDLGTKTERQSDLSNALGSMYAAVLFLGLQNATSVQPVVAVERTVFYREKA
AGMYSAMPYAYAQALIEIPYIFVQTVVYSVITYAMIGFEWTAAKFFWYLFFLLFTLLYFTYYGMMTVAVTPNH
HIASIVSSAFYSIWSLFSGFMIPRTKMPVWWRWYYWGCPISWTLYGLLGSQFGDVKTMLGNTGQTVEEYVND
YYGIKHDFLGVVAGVVVGITVLFAFTFAISIKAFNFQKR

>XP_021628887.1_PDR1-l_Me
MEGDHYRASTSLRRGSSSAWRNNVLDVFSASSRDEDDEEALKWAALEKLPTYDRLRKGILVSVSKGGANELD
VDNLGFNERKTLLERLVKVAEEDNEKFLLKLKNRLDRVGIEVPTIEVRYEHLNIEAEALVGSNALPSFLNFTISI
AEGLLNYLHIFPSRKRPLTILNDVSGVIKPSRMTLLLGPPSSGKTTLLLALAGKLDPNLKVSGNVTYNGHTLNEF
IPQRTAAYISQHDLHIGEMTVRETLAFSARCQGVGTQHEMLAELSRREKAANIKPDPDLDFFMKAAATEGQET
SVVTDYILKILGLDICADTLVGNEMIRGISGGQRKRVTTGEMLVGPAKALFMDEISTGLDSSTTFSIVNSLRQSIH
ILNGTAVISLLQPAPETYNLFDDIILLSDGQIVYQGPREHVLEFFEYMGFKCPERKGVADFLQEVTSKKDQQQY
WARKDRLYRFITVREFAEAFQSYEVGRKIAGDLKTPFDRRKNHPAALATKHYGVGKMELLKANFSREYLLMK
RNSFVYIFKLSQLIMMATIGMTLFFRTEMKRDDLEDAGVYLGALFFTLITIMFNGMAELSMTIAKLPVFYKQRN
LLFFPAWSYSIPSWILKIPVTFLEVGVWVFLTYYVIGFDPNVGRLFKQYMLLLLVNQMASALFRFIASVGRNMI
VANTFGSFALLTLFALGGFVLSREDIKKWWIWGYWVSPLMYGQNAIVANEYLGHSWSHIPANSNSTDSLGVQ
FMKSRGFFPNAYWYWLGVAASAGYILLFNLAYTVALTFLDSFEKPQAVISDEPEESKSSERAIQLSKLESSHRT
NTESRTSGIDESNHNRKKGMVLPFEPRSITFDNVMYSVDMPQEMKNQGVLDDKLVLLKGVSGAFRPGVLTAL
MGVSGAGKTTLMDVLAGRKTGGYIEGDIRISGYPKKQETFARISGYCEQNDIHSPHVTVYESLVYSAWLRLPQ
EVDSETRRMFVEEVMELVELNPLRQALVGLPGVNGLSTEQRKRLTIAVELVANPSIIFMDEPTSGLDARAAAIV
MRTVRNTVDTGRTVVCTIHQPSIDIFEAFDELFLMKRGGEEIYVGPLGRHSCHLIKYFEGMEGVSKITDGYNPA
TWMLEVTSAAQELALGVDFAEIYRNSDLYRRNKTMIQELSKPAPGTQDLYFPTKYSQPFLTQCLACLWKQSW
SYWRNPPYTGVRFWFTTFIALMFGTIFWDLGSKKGKEGDLSNAMGSMYAAVLFLGVQNASSVQPVVAVERT
VFYREKAAGLYSAMPYAYAQALIELPYIFAQAGVYSVITYAMIGFEWTAAKFFWYLFFLYFTLLYFTFYGMMT
VAVTPNHHIASIVSSAFYAIWNLFSGFIIPRTKMPVWWRWYYWGCPISWTLYGLIASQFADIKTMLGDSGKTVE
EYVNDFYGIKHDFVGVAAGVIVGITVLFAFIFGISIKAFNFQRR

>XP_021628888.1_PDR1-l_Me
MESGDIYKASSSLRRGSFSTWKHHPSGIFSTSSREEDDEEALKWAALEKLPTYDRLRKGILLSASKGAVNEIDV
DNLGFQERKTLLERLVKVAEEDNEKFLFKLKNRIDRVGIEIPTIEVRFEHLNVEADAFVGSRALPSFINFSVNML
EGFLNYIPIFPSRKRPLTILKDVSGVIKPSRMTLLLGPPSSGKTTLLLALAGKLDPNLKFSGTVTYNGYKMNEFIP
QRTAAYISQHDVHLGEMTVRETLAFSARCQGVGTQHELLAELSRREKAANIKPDPDIDVFMKAAATEGQETSV
VTDYILKILGLEICADILVGDEMVRGISGGQRKRVTTGEMLVGPAKALFMDEISTGLDSSTTYQIVNSLKQSIHIL
NGTAVISLLQPAPETYDLFDDIILLSDGQIVYQGPREQVLGFFEHMGFKCPERKGVADFLQEVTSRKDQKQYW

ARRGQPHRFITVQEFSEAFQSYELGRRIADELSTPFDKTKSHPAALSTKKYGVGKMELLKACISREFLLMKRNS
FVYIFKLTQLTFMASVSMTLFLRTEMHRNNLTDGGIYLGALFFSVIMVMFNGMAELSLTIAKLPVFYKQRDFLF
YPAWAYSLPTWILKTPVTFVEAAVWTVLTYYEIGFDPNVVRFFKHYLVLLLVNQMASSLFRFIAATGRNVIVA
NTFGSFSLLTLFALGGFILSREEIKKWWIWGYWISPLMYGQHAVVVNEFLAKSWRHIPPNSTEPLGVQLLKTRG
FFPHAYWYWLGVGALGGFVLLFNFCFTLALTFLDPFEKPQAVIVEDSQSNEPGDENGKGAGEEISEANHNKKK
GMVLPFEPRSVAFDNVMYSVDMPQEMKSQGVLEDKLVLLKNVSGAFRPGVLTALMGVSGAGKTTLMDVLA
GRKTGGYIEGDIRISGYPKKQETFARVSGYCEQNDIHSPFVTVYESLVYSAWLRLPPEVDSDTRKMFVDEIMEL
VELDPLKQALVGLPGVSGLSTEQRKRLTIAVELVANPSIIFMDEPTSGLDARAAAIVMRTVRNTVDTGRTVVCT
IHQPSIDIFDAFDELFLMKRGGEEIYVGPLGRLSCHLIKYFEGIEGVSKITDGYNPATWMLEVTSSAQELALNVD
FAAIYKNSELYRRNKEMIKELSTPAPGSTDLYFPTKYSQSFFTQCIACLWKQRLSYWRNPPYTAVRFLFTTFIAL
MFGTMFWDLGSKKTKQQDIFNAAGSMYAAVFFLGVQNASSVQPVVAIERTVFYRERAAGMYSAMPYAYAQ
VLVELPYIFAQAIVYAVITYAMMGFEWTGIKFFWYIFFMYFTLLYFTFYGMMTVAVTPNHHIAAIVSSAFYGL
WNLFAGFIVPRTKMPVWWRWYYWGCPVSWTLYGLVASQYGDVKDVLDTNQTVEDFVREYYGFKHDFVGVI
AGGIVGISVLFAFIFGFSIRFFNFQRR

>XP_021650398.1_PDR1-l_Hb
MEGVEIYIPAGSLRRGDSSIWTSNAMEGFSKSSREEDDEEALKWAALERLPTYDRLKKGILTTSKGTNEIDVHA
LGFHERRALVDRLVKVADQDNERFLLKLRDRIDRVGIEFPTIEVRFEHLTVETEAHVGSRALPSFSNFFVDTVEG
FLNYLHILPSIKKRFSILQDISGIIRPTRMTLLLGPPSSGKTTLLLALAGKLDPKLKFSGSVTYNGHGMNEFVPRRT
AAYISQYDTHIGEMTVRETLAFSARCQGVGHRIEVLTELLRREKESNIKPDPDVDVFMKGIAAEGQETNVITDYI
LKILGLDVCADIMVGNEMLRGVSGGQRKRVTTGEMLVGPAKALFMDEISTGLDSSTTFQIVNCIKQFIKFLNGT
AVISLLQPAPETYDLFDDIILLSDGQIVYQGPREHVLKFFEHMGFRCPERKGVADFLQEVTSRKDQQQYWAHK
DVPYSFITVKEFAEAFQSFHVGQGLSGELSTPFDKAKSHPAALATKKYGVSNIQLLKACFSRELLLMKRNSFVY
IFKLTQLTIMAMVAMTLFFRTEMHRDSITDGGIYAGSLFFSMIFIMFNGLSEMSMTISKLPVFYKQRKLLFYPAW
VYSLPPWIIKIPITLVQVALWVFITYYVIGYDPNAGRLFRQYLLLVLVSQMASALFRFIAAAGRNMIVAQTFGAF
ALVLLFALGGFVLSRENIKKWWIWGYWISPLMYGQNAIVVNEFFGKSWSHVLPNSTEPLGVQVLNSRGFFPHA
YWYWIGTGALVGFTLLYNFCFILALAFLEPLQKPQAVISEDTTSNEPGRTAGAIQLSSQGSNHKTTTESGRGSSL
RISSERIEVTTEGNHERKKGMVLPFEPHSITFSDIKYSVDMPQEMKNQGVTEDKLVLLRGVSGAFRPGVLTALM
GSSGAGKTTLMDVLSGRKTGGYIEGNITVSGYPKKQETFARISGYCEQNDIHSPHVTVYESLLYSAWLRLPAEV
NSETRKMFIEEVMQLVELTPLRQALVGLPGISGLSTEQRKRLTIAVELVANPSIIFMDEPTSGLDARAAAIVMRA
VRNTVDTGRTVVCTIHQPSIDIFEAFDELFLMKRGGQEIYVGPLGRHSCHLIKYFEGIEGVANIKDGYNPATWM
LEVTTSAQETVLGIDFAVAYRNSEQYRRNKALIEELSTPAPGSKDMYFPTQYSQSFLVQCMACLWKQHLSYW
RNPPYTAVRFLFTTVIALTFGTMFWDLGSKTTKRQDLFNAMGSMYAAIVFLGIQNASSVQPVVSVERTVFYRE
RAAGMYSPLPYAFAQVVIELPYIFFQAVVYGLIVYAMIGFEWTAAKFFWYLFFMYFTLLYYTFYGMMSVAAT
PNQHVASIVSSAFYSLWNLFSGFIIPRPRIPVWWRWYAWACPVAYTLYGLVSSQFGDIKHKLESGDTVEDFVRS
YFDFKHELLGAIAAAVVGFGALFAFMFAICIKFLNFQRR

>XP_021655960.1_PDR1-l_Hb
MEGADLYRASSSLRRGSFSTWRNNPSDVFSRSSRDEDDEEALKWAALEKLPTFDRLRKGILLSASKGVVSEIDV
DSLGLQERKTLLERLVKVAEEDNEKFLLKLKNRIDRVGIEIPTIEVRFEHLNIEAEAYVGGRALPSFINFSVNMLE
GFLNYLHILPSRKRPLTILKDVSGVIKPSRMTLLLGPPSSGKTTLLLALAGKLDPNLKFSGSVTYNGHGMNEFIP
QRTAAYISQHDVHIGEMTVRETLAFSARCQGVGTQHDLLAELSRREKDANIKPDPDIDVFMKAAATEGQEASV
VTDYVLKILGLEICADTVVGNEMIRGISGGQRKRVTTGEMLVGPAKALFMDEISTGLDSSTTFQIVNSLKQSIHI
LNGTAVISLLQPAPETYDLFDDIILLSDGQIVYQGPREQVLGFFEHMGFKCPERKGVADFLQEVTSRKDQKQYW
ARRDQPYSFVTVQEFAEAFQSCDVGRRIGDELSIPFDKTKSHPAALTTKKFGIGKMELFKACISRDYLLMKRNS
FVYIFKLTQLTLMAIITMTLFLRTKMHRENIADGGVYLGALFFTVVMVMFNGMAELPMTISKLPVFYKQRELL
FYPAWAYSLPTWILKIPVTFVEVAVWVVLTYYVIGFDPNVSRFFKHYFVLILVNQMASALFRFIAATGRNVIVA
NTFGSFALLTLFALGGFVLSRDDIKKWWIWGYWISPLMYGQNAIVVNEFLGKSWSHIPPNSTEPLGVQLLASR
GFFPDAYWYWLGVGALGGFILLFNICFALALTFLNPFEKPQAVIIEDSQSNDPGDENGGAIQLTNKGSSHKSSTG
IGEEISEVNHNKKKGMVLPFEPHSVAFDNVVYSVDMPQEMKSQGVLEDKLVLLKGVSGAFRPGVLTALMGVS
GAGKTTLMDVLAGRKTGGYIEGNITISGYPKKQETFARISGYCEQNDIHSPHVTVYESLVYSAWLRLPHEVDSN
TRKMFVDEVMELVELNPLRQALVGLPGVSGLSTEQRKRLTIAVELVANPSIIFMDEPTSGLDARAAAIVMRTVR
NTVDTGRTVVCTIHQPSIDIFDAFDELFLMKRGGEEIYVGPLGRHSCHLIKYFEGIEGVHKITDGYNPATWMLE
VTSSAQELALDVDFAAIYKNSELYRRNKAMIMELSTPAPGSKDLYFPAKYSQPFFTQCIACLWKQRLSYWRNP

PYTAVRFLFTTFIALMFGTMFWKTGSQSKKQQDIFNAAGAMYAAVLFLGVQNASSVQPVVAIERTVFYRERA
AGMYSAMPYAYAQVLVEIPYIFAQAIVYGAITYAMMAFEWTVAKFFWYIFFMYFTLLYFTLYGMMTVAVTP
NHHIAAVVSSAFYAIWNLFSGFIVPRTRIPVWWRWYYWVCPVSWTLYGLIASQFADRKDVLDNGQTIEDFIRQ
FYDFKHDFVGVVAGVIVGITVLFAFIFGISIKSFNFQRR

>XP_021655961.1_PDR1-l_Hb
MEGDHYRASTSLRRGSSSVFRNNGLDVFSRSSRDEDDEEALKWAALEKLPTYDRLRKGILVSVSKGGANEIDV
DNLGFQERKTLLERLVKVAEEDNEKFLLKLKNRIDRVGIEIPTIEVRYEHLNIEAEALVGSNALPSFLNFTMSIAE
VFLNCLHVLPNRKRPLTILKDVSGVIKPSRMTLLLGPPSSGKTTLLLALAGKLDPNLKVSGNVTYNAHTLNEFIP
QRTAAYISQHDLHIGEMTVRETLAFSARCQGVGTQHEMLAELSRREKAANIKPDPDLDVFMKAAATEGQETS
VVTDYVLKILGLDICADTLVGNEMIRGISGGQRKRVTTGEMLVGPAKALFMDEISTGLDSSTTFSIINSLRQSIHI
LNGTAVISLLQPAPETYNLFDDIILLSDGQVVYQGPREQVLGFFEYMGFRCPERKGVADFLQEVTSKKDQQQY
WARKDQPYRFVTVNEFAEAFQSYEVGRRIAEDLSVPFDRTKNHPAALTTKHYGVGKMELLKANLSREYLLMK
RNSFVYIFKLTQLIVMATIGMTLFFRTEMKRDTLEDAGVYLGALFFSLITIMFNGMAELSMTIAKLPVFYKQRN
LLFFPAWSYSIPSWILKIPVTFVEVGVWVFITYYVIGFDPNVGRLFKQYMLLLLVNQMASALFRFIASVGRNMIV
ANTFGSFALLTLFALGGFILKRPEIKKWWIWGYWISPLMYGQNAIVANEFLGHSWNHIPANSNSTESLGVQFIK
NNGFFPHAYWYWLGLGASAGYIFVFNILYTVALTFLDQFEKPQAIISDEPEERERSGGAIQLSQVESSHRNNTES
GTSGIDESNHNKKKGMVLPFEPHSITFDNVIYSVDMPQEMKNQGVVEDKLVLLKGVSGAFRPGVLTALMGVS
GAGKTTLMDVLAGRKTGGYIEGDIRISGYPKKQETFARISGYCEQNDIHSPHVTVYESLVYSAWLRLPSDVNSE
TRKMFVEEVMELVELNPLRQALVGLPGVNGLSTEQRKRLTIAVELVANPSIIFMDEPTSGLDARAAAIVMRTV
RNTVDTGRTVVCTIHQPSIDIFEAFDELFLMKRGGEEIYVGPLGRHSCHLIKYFEGIEGVSKITDGYNPATWMLE
VSSSAQELSLGVDFANIYRNSDLYRRNKAMIQELSKPAPGTKDLYFPTQYSQPFLTQCMACLWKQSWSYWRN
PPYTGVRFWFTTFIALMFGTIFWDLGSKLEKEGDLSNAMGSMYVAVLFLGVQNSSSVQPVVAIERTVFYREKA
AGMYSAMPYAYAQALIELPYIFTQAGVYSLITYAMIGFEWTAAKFFWYLFFLYFTLLYFTYYGMMAVAVTPN
HHIASIISSAFYAIWNLFAGFIVPRPKMPVWWRWYYWGCPISWTLYGLFASQFGDITKPLGETGKTVEQYVSDF
YGIKHNFLGACAGVIIGIDTLFAVIFAISIKAFNFQRR

>XP_021665656.1_PDR1-l_X1_Hb
MEGGDITSRVSSARLSSSNIWRNTTLEVFSKSSSQIEDDEEALKWAALEKLPTYLRVRRGILTEEEGQSREIDVN
SLGLIEKRNLLERLVKIAEQDNEKFLLKLKDRIEKVGLDMPTIEVRFEHLSVETEAYVGSRALPTMFNFSAKMF
EGFLNYLHVLPSRKKPLSILNDVSGIIKPRRMTLLLGPPSSGKTTLLLAFAGKLGKELKFSGRVKYNGHGMGEF
VPQRTSAYISQYDLHIGEMTVRETLAFSARCQGVGIGYEMLAELARREKAANIKPDTDIDIYMKLRGSALEGQE
ANVVTDYILKILGLEVCADILVGDEMVRGISGGQKKRVTTGEMLVGPARALFMDEISTGLDSSTTFQIVNSLRQ
SIHIFNGTAFVSLLQPAPETYDLFDDIILLSDGQIVYQGPRENVLEFFEYRGFRCPQRKGVADFLQEVTSKKDQE
QYWAHKEEPYSFVAVKEFAEAFQSFHVGRKLGDELAIPFDITKAHPDSLTTKKYGVSKKELLKACISREYLLM
KRNSFVYIFKMTQLIIMAFVTMTIFLRTEMHRNTETDGGIYLGALFYTIIVTVFNGFSELAMTIMKLPVFYKQRD
LLFYPAWAYALPTWILKIPVSFVEVAVWVVMTYYVIDFDPNIGRFFKQYLILLVTSQMASAMFRLTAALGRSVI
VANTVGSFALLAVLILGGFIISRDSVKKWWIWGYWFSPMMYVQNGISVNEFLGNSWNHFLPNSTEALGVAFM
KSRGLFPEAYWYCIAVGALTGYIFLFNFLFTLALKYLDPFGRPHALISEEAYAEKNAAKTGESIELSSKGKSSLE
RGSASQRSASSRTPSTRVSSFSDANQNRKRGMILPFQPLSITFDEVRYAVDMPQEMKAQGITEDRLELLKGVSG
AFRPGVLTALMGVSGAGKTTLMDVLAGRKTGGYIDGSISISGYPKKQETFARISGYCEQTDIHSPHVTVYESLL
YSAWLRLPPEVDSDTRKMFIEEVMELVELTNLRVALVGLPGVNGLSIEQRKRLTIAVELVANPSIIFMDEPTSGL
DARAAAIVMRTVRNTVDTGRTVVCTIHQPSIDIFDAFDELFLLKRGGEEIYVGPVGRHACHLIKYFEDIEGIPKIK
DGYNPATWMLEVTTAAQEAALGINFSDIYKNSELYRRNKALINELSKPSPGSKDLYFPTQYSQSFFTQCMACL
WKQHWSYWRNPPYSAVRLLFTVFIALMFGTIFWNLGSKSTRKQDLFNAMGSMYAAVLFLGFQNSTSVQPVV
AIERTVFYRERAAGMYSELPYAFGQVMIELPYILVQTITYGGIVYAMLGFEWTISKFLWYIFIMYFTLAYFTFYG
MMTVAITPNHHIAAIVSSFFYGIWNIFSGFIIPRTRIPVWWRWNYWACPIAWTLYGLVASQYGDKKEPLESGET
VEHFLRSYFGFRHYFVGIVAIVIVGIAVLFGFIFAFSIKVFNFQHR

>XP_021665657.1_PDR1-l_X2_Hb
MEGGDITSRVSSARLSSSNIWRNTTLEVFSKSSSQIEDDEEALKWAALEKLPTYLRVRRGILTEEEGQSREIDVN
SLGLIEKRNLLERLVKIAEQDNEKFLLKLKDRIEKVGLDMPTIEVRFEHLSVETEAYVGSRALPTMFNFSAKMF
EGFLNYLHVLPSRKKPLSILNDVSGIIKPRRMTLLLGPPSSGKTTLLLAFAGKLGKELKFSGRVKYNGHGMGEF
VPQRTSAYISQYDLHIGEMTVRETLAFSARCQGVGIGYEMLAELARREKAANIKPDTDIDIYMKLRGSALEGQE

ANVVTDYILKILGLEVCADILVGDEMVRGISGGQKKRVTTGEMLVGPARALFMDEISTGLDSSTTFQIVNSLRQ
SIHIFNGTAFVSLLQPAPETYDLFDDIILLSDGQIVYQGPRENVLEFFEYRGFRCPQRKGVADFLQEVTSKKDQE
QYWAHKEEPYSFVAVKEFAEAFQSFHVGRKLGDELAIPFDITKAHPDSLTTKKYGVSKKELLKACISREYLLM
KRNSFVYIFKMTQLIIMAFVTMTIFLRTEMHRNTETDGGIYLGALFYTIIVTVFNGFSELAMTIMKLPVFYKQRD
LLFYPAWAYALPTWILKIPVSFVEVAVWVVMTYYVIDFDPNIGRFFKQYLILLVTSQMASAMFRLTAALGRSVI
VANTVGSFALLAVLILGGFIISRDSVKKWWIWGYWFSPMMYVQNGISVNEFLGNSWNHFLPNSTEALGVAFM
KSRGLFPEAYWYCIAVGALTGYIFLFNFLFTLALKYLDPFGRPHALISEEAYAEKNAAKTGEGSASQRSASSRTP
STRVSSFSDANQNRKRGMILPFQPLSITFDEVRYAVDMPQEMKAQGITEDRLELLKGVSGAFRPGVLTALMGV
SGAGKTTLMDVLAGRKTGGYIDGSISISGYPKKQETFARISGYCEQTDIHSPHVTVYESLLYSAWLRLPPEVDSD
TRKMFIEEVMELVELTNLRVALVGLPGVNGLSIEQRKRLTIAVELVANPSIIFMDEPTSGLDARAAAIVMRTVR
NTVDTGRTVVCTIHQPSIDIFDAFDELFLLKRGGEEIYVGPVGRHACHLIKYFEDIEGIPKIKDGYNPATWMLEV
TTAAQEAALGINFSDIYKNSELYRRNKALINELSKPSPGSKDLYFPTQYSQSFFTQCMACLWKQHWSYWRNPP
YSAVRLLFTVFIALMFGTIFWNLGSKSTRKQDLFNAMGSMYAAVLFLGFQNSTSVQPVVAIERTVFYRERAAG
MYSELPYAFGQVMIELPYILVQTITYGGIVYAMLGFEWTISKFLWYIFIMYFTLAYFTFYGMMTVAITPNHHIA
AIVSSFFYGIWNIFSGFIIPRTRIPVWWRWNYWACPIAWTLYGLVASQYGDKKEPLESGETVEHFLRSYFGFRH
YFVGIVAIVIVGIAVLFGFIFAFSIKVFNFQHR

>XP_021674585.1_PDR1-l_Hb
MEGDYYRASNSLRSGSSSLWRNNGLDVFSRSSREEDDEEALKWAALEKLPTYDRLRKGILVSVSKGGANEIDV
DNLGFQERKTLLERLVKVAEEDNEKFLMKLKNRIDRVGIEIPTIEVRYEHLNVEAEALVGSNALPSFLNFTVSIA
EVLLSYLHIFPNRKRPLTILKDVSGVIKPSRMTLLLGPPSSGKTTLLLALAGKLDRNLKVSGNVTYNGHTLNEFI
PQRTAAYISQHDLHIGEMTVRETLAFSARCQGVGTQHEMLAELSRREKAANIKPDPDLDVYMKAAATEGQET
SVVTDYVLKILGLDICADTLVGNEMIRGISGGQRKRVTTGEMLVGPAKALFMDEISTGLDSSTTFSIVNSLRQSI
HILNGTAVISLLQPAPETYNLFDDIILLSDGQIVYQGPREHVLEFFEYMGFKCPERKGVADFLQEVTSKKDQQQ
YWARKDQPYRFITVNEFAEAFQSYEVGRKIVEDLSTPFDRTKNHPAALAIKHYGVGKMELLKANFSREYLLM
KRNSFVYIFKLFQLIMMATISMTLFLRTEMKRDDFGDAGVYLGALFFTLIMIMFNGMSELPMTIANLPVFYKQR
DLLFFPAWSYSIPSWILKIPVTFLEVGVWVFLTYYVIGFDPNAGRLFKQYILLLLVNQMASALFRLIASVGRNMI
VANTFGSFALLTLLALGGFVLSREDIKKWWIWAYWVSPLMYGQNAIVANEFLGHSWSHIPANSNSSDSLGVQF
MKSRGFFPNAYWYWLGVGALAGYILLFNLAYAVALTFLDPFEKPQAVISDEPEESTRTGGAIQLSQLESSHRTN
TESGTSGIDESNHNKKKGMVLPFEPYSITFDNVIYSVDMPQEMKNQGVVEDKLVLLKGVSGAFRPGVLTALM
GVSGAGKTTLMDVLAGRKTGGYIEGDIRISGYPKKQETFARISGYCEQNDIHSPHVTVYESLVYSAWLRLPQEV
DSETRKMFVEEVMELVELNPLRQALVGLPGVNGLSTEQRKRLTIAVELVANPSIIFMDEPTSGLDARAAAIVMR
TVRNTVDTGRTVVCTIHQPSIDIFEAFDELFLMKRGGEEIYVGPLGRRSCHLIKYFEGIKGVSKITDGYNPATWM
LEVTSSAQELTLGVDFSDIYRNSDLYRRNKTMIQELSKPAPGTKDLYFPTQYSQPFLTQCMACLWKQSWSYWR
NPPYTGVRFWFTTFIALMFGTIFWDLGSKRGKEGDLSNAMGSMYVAVLFLGVQNASSVQPVVAVERTVFYRE
KAAGMYSAMPYAYAQALIELPYIFAQAGVYSVITYAMIGFEWTAAKFFWYLFFLYFTLLYFTFYGMMTVAVT
PNHHIASIVSSAFYAIWNLFSGFIVPRTKMPVWWRWYYWACPVSWTLYGLFASQFADIKTEIGNTGQTVEQYV
NDLYGIKHDFLGVSAGVIVGITLLFAFIFAISIKAFNFQRR

>XP_021800496.1_PDR1-l_Pa
MESGGGGDIYRVSSARLSSSNIWRNSAMDVFSKSSHDEDDEEALKWAAIEKLPTYLRIRRGILTEGEGQAREIDI
KNLGLLERKSVLERLVKTADEDNEKFLLKLKDRINRVGLDIPTIEVRFEHLSVEAEAYVGGRALPTIFNFCVNIL
EGFLNFVHVLPSRKQPLPILDDVSGIIKPRRMTLLLGPPSSGKTTLLLALAGKLAKDLKFSGRVAYNGHGMEEF
VPERTSAYISQNDLHIGEMTVRETLAFSARCQGVGPRYEMLAELSRREKAANIMPDADLDIYMKAASLEGQET
NVVTDYILKILGLEVCADIMVGDEMVRGISGGQKKRVTTGEMLVGPARALFMDEISTGLDSSTTFQIVNSLRQS
IHILGGTALISLLQPAPETYDLFDDIILLSDGQIVYQGPRENVLEFFEHMGFKCPERKGVADFLQEVTSKKDQEQ
YWAQKEEPYNFISSKEFAEAFQSFHIGRKLGDELATPFDKSKSHPAALTTMKYGVSKKELLKACISREYLLMKR
NSFVYIFKMTQLTLMAFISMTLFLRTKMHRDTVADGGIYLGAMFYTIIIIMFNGFSELAMTIMKLPVFFKQRDLL
FYPSWAYSLPTWILKIPITFIECAVWVVMTYYVIGFDPNIERFFKQYLLLLCLNQMASGLFRFMGALGRNIIVAN
TFGSFALLAVLVMGGFILSREDVQKWWLWGYWVSPMMYGQNAIAVNEFLGKSWSHVPPNSTESLGIMVLKS
RGVFIEPYWYWIGVGATIGYIFLFNFFFTLALQYLDPFGKPQAILSKEALAEKTSDRTGDSIELSSRGKNSSDSRN
ESRRSVSSRTLSARVGSITEANENRKRGMVLPFEPLWITFDEITYAVDMPEEMKTQGVTEDRLQLLKGVTGAFR
PGVLTALMGISGAGKTTLMDVLAGRKTGGYIEGNITISGHPKKQETFARISGYCEQTDIHSPHVTVYESLVYSA
WLRLPPEVDSSTRKMFVEEVMELVELTSIREALVGLPGVNGLSTEQRKRLTIAVELVANPSIIFMDEPTSGLDAR

AAAIVMRTVRNTVDTGRTVVCTIHQPSIDIFDAFDELFLLKRGGEEIYVGPLGRHSTHLIKYFEEIDGVPKIKDG
YNPATWMLEITAAAQEAALGVNFAEIYKNSELYGRNKALIKDLSTPPAGSKDLFFPTQYSQSFFSQCMACLWK
QHLSYWRNPPYSAVRLLFTTFIALMFGTIFWDLGSKRRSQQDLFNAMGSMYAAVLFIGVQNASSVQPVVAIER
TVFYRERAAGMYSALPYAFGQVVIELPYIFVQTIIYGVIVYAMIGFDWTVSKFLWYLFFMYFTFLYFTFYGMM
TVAVTPNHNIAAIVSSAFYAIWNLFSGFIIPRTRMPIWWRWYYWVCPVSYTLYGLVASQFGDIKEIFDSGESAG
KSVEHFVKDYFGYRQDFLGVVAAVHVGICVLFGFTFAFSIKVFNFQKR

>XP_021817956.1_PDR1-l_Pa
MEGGEIYRAGNSLQLGSSMRWRNNGVEVFSRSSREEDDEAALKWAALQKLPTYNRLRKGILTSPAGEANEVD
IPNLDFQERKELIERFLKGAEEDNERFLLKLKNRIHRVGIDLPTIEVRYEHLKVEAEAYVGSRALPTLFNFIVNIL
EGILNRLHIFSNRKKNLSILHSVSGIIKPRRMTLLLGPPSSGKTTLLLALAGKLDPDLKLSGRVTYNGHEMNEFV
PQKTAAYISQHDLHVGEMTVRETLAFSARCQGVGTRYDMLSELCRREKAANIKPDPDIDVFMKAIATEGQEVN
VVTDYILKILGLEVCADTIVGDEMLRGISGGQRKRVTTGEMLVGPANALFMDEISTGLDSSTTFQIVNSIKQYV
HILNGTVVISLLQPAPETYELFDDIILLSDGQIVYQGSREHVLEFFESMGFKCPERKGIADFLQEVTSRKDQEQY
WACKDEPYRFITVKEFNEAFQSFHAGQKIGDELSVPFDKSKNHPAALTTKEYGLKKGELLKACFSREYLLAKR
NAFVYIFKLIQLTVVALISMTLFLRTKMPRDSVNDGGVYAGALFFTMIMVMFNGMSELPMTIIKLPVFYKQRDL
FFYPAWTYALPTWILKIPITIVEVALWVFTTYYVIGFDPSIERLLRQYILLILVSQMASALFRLIAAACRNLVLAN
TLGSFALLIMFTLGGFVLSRDNIRKWWKWGYWISPMMYGQNAVVVNELLGKNWRHVLPNSTVSLGVEVLKS
RGFFPHAYWYWIGVGAMAGFVLLFNSCYIVALSYLNPLGKPQAVRSEDSQSNENDGRTERMGILPRNQNSSSE
RSKTESKGQNRTEVSLQTIHNTKRGMVLPFEPHSITFDEIIYSVDMPQEMKIQGVMQDKLVLLKGVSGAFRPGV
LTALMGVSGAGKTTLMDVLAGRKTGGYIEGDIKISGYPKKQETFARISGYCEQNDIHSPHVTVHESLIYSAWLR
LPPEVKSETRKMFIEEVMELVELTSLRQALVGLPGVNGLSTEQRKRLTIAVELVANPSIIFMDEPTSGLDARAAA
IVMRTVRNTVDTGRTVVCTIHQPSIDIFEGFDELLLLKRGGEEIYVGPLGRHSFHLIKYFEGIEGISKIKDGYNPAT
WMLEVTTSAQELDLKIDFAQVYKTSELYRRNKQLIKDFSTPAPSSKDLYFPTQYTQSFLTQTIACLWKQHWSY
WRNPLYTAVKFLFTIVIALMFGTIFWKLGSKTKRQQDLFNAMGSMYTAVLFLGVQNATSVQPVVAVERTVFY
REKAAGMYSALPYAFAQVLIELPYILVQAVVYGVITYTLIGFKMTLVKFFWYLFFMYFTLLYFTLYGMMTVA
VTPNHHIASIVSSAFYGIWNLFSGFIVPRPRIPIWWRWYYWACPVAWTLYGLVASQFGDLNHVLDNGETVKQF
LDDYFGFKHDFVGVVAAVHVGITVLFGFIFAFSIRAFNFQTR

>XP_022000956.1_PDR1-l_Ha
MEGGDVFRVSSARISSSNIWRNSGRDIFSRSSVEEDDEEALKWAAIEKLPTNLRLERGILTEETTGQPREIDIKNL
DLVEKRNLLERLVKIAEEDNEKFLLKLKNRIDRVGLQLPTIEVRFEHLNVDAEAYVGGRALPTILNFLVNILEGS
LNYLHLVPSRKIPLPVLHDVSGIIKPGRMTLLLGPPSSGKTTLLLALAGKLGSGLKVSGRVTYNGHEMNEFVPQ
RSSAYISQNDLHIGEMTVRETLAFSARCQGVGASYEMLAELSRREKEANIKPDPDLDIYMKAASLEGQEASVV
TEYVIKILGLEVCADTIVGDEMFRGISGGQKKRVTTGEMMVGPARALFMDEISTGLDSSTTFQIVNSIRQSIHILQ
GTSVISLLQPAPETYDLFDDIILLSDGQVVYQGPRENVLEFFEYMGFKCPERKGVADFLQEVTSMKDQEQYWT
KRDQPYSFITSREFAEAFQSFHIGKQLGDELSTPFDRSKSHPHALTTKKFGVSKKELLKACISREYLLMKRNSFV
YIFKMTQLIFMASITMTLFLRTEMPKKTFVDGTIFMGALFFTLVMITFNGFSELSLSILKLPVFYKHRDLLFFPAW
AYSLPTWVLKIPVTIVEVATWVIMTYYVIGFDSDPARFFKQMVLLICIHQMSSALFRFIGSLGRNIIVANTFGSFG
LLTIFVLGGFILARDDIRGWWIWCYWFSPMMYGQNGMAVNEFLGDSWNKVLPNSTDTIGVSVLKSRGIFPEAY
WYWIAVGVSIGYMFLFNICFTLALEYLNPFGNPQAVLSEESMAARKAMKTGHPIELSARSSSERKSEVRMSSRV
GSINGSNQNKKRGMVLPFVPLSITFDNIRYAVDMPQEMKAQGVAQDRLELLKGVSGAFRPGVLTALMGISGA
GKTTLMDVLAGRKTGGYIEGAITISGYPKKQETFARIAGYCEQTDIHSPHVTVYESLQYSAWLRLPPEVDSATR
EMFVEEVMELVELVPLKEALVGLPGVNGLSTEQRKRLTIAVELVANPSIIFMDEPTSGLDARAAAIVMRTVRNT
VDTGRTVVCTIHQPSIDIFDAFDELFLLKRGGEEIYVGPLGRHSSHLIKYFEGIDGVSKIKDGYNPATWMLEVTS
TGQEAALGVNFAEVYKNSELYKRNKELINESSKPIPGSSDIHFRSQYSQSFWTQCMACLWKQNWSYWRNPPY
TAVRFLFTTVIALMFGTIFWDMGSKRGKQQDLFNAMGSMYAAILFIGVQNATSVQPVVSIERTVFYRERAAGM
YSALPYAFGQVMIEIPYIFVQTIVYGIIVYAMIGFEWTAVKFCWFLFFMYFTFLYFTFYGMMTVAVTPNHNFAA
IISSAFYAIWNLFSGFIVPRTRIPIWWRWYYYICPIAWTLYGLVASQFGDITDKLDTGETVAEFVHNFYGFEYDFI
KYVAVIIVGFTVLFGFIFAYSIKAFNFQKR

>XP_022131319.1_PDR1-l_Mc
MEVFSRSSCGDDDEEALKWAAIEKLPTYLRIRRGILKEEQGEAREIDIRNLGLLERRHVLERLVKIAEDDNEKFL
LKLRDRIERVGLEIPTTEVRFEHLNVEAEVYVGRRALPTMFNFSLNILEGLLNYLHILPSRKRPLSILHDVSGIIKP

KRMTLLLGPPSSGKTTLLSALAGKLGKDLKFSGKVSYNGHGMEEFVPQRTSAYISQHDLHIGEMTVRETLAFS
ARCQGVGPRYEMLAELSRREKAANIKPDPDLDIYMKAEALEGQETSIITDYILKILGLEMCADTMVGDEMMRG
ISGGQKKRLTTGEMLVGPARALFMDEISNGLDSSTTYQIVNSIRQSIHILNGTAVISLLQPAPETFDLFDDIILLSE
GHIVYQGPREDVLNFFAHMGFKCPERKGVADFLQEVTSRKDQEQYWGSRDEPYRFVSVEQFSEAFQSFHIGRK
LGDELAAAFDRSKCHPATLTTKKYGVSKKEVLRACISRELLLMKRNSFVYIFKMFQLIIMALITMTLFLRTELH
RDTEIDGGIYMGALFFALIVIMFNGFSELSMTVVKLPVFYKQRDLLFYPSWAYALPTWILKIPITCLEVGLWVL
MTYYVIGFDPNIERFFKQFVLLFCINQMASGLFRLAAALGRDVIVATTCATLALLAVMALGGFVVAREDVHPW
WLWGYWVSPMMYGQNAIAVNEFLGNSWRHVPPNSSEPLGVSVLKSRGIFPKAYWYWIGVGATIGYVLLFNF
LFTLALHYLDPFSKPQAVLSEDTLAETNANRTGKFEPPIKANSSFGGSSNSNLYNAERERESQSISSRTLSIRIGST
SENQNDNRGMVLPYKPHSITFDEIRYAVDMPQEMKAQGVVEDKLELLKGVSGAFRPAVLTALMGVSGAGKT
TLMDVLAGRKTGGYTEGTITISGFPKRQETFARISGYCEQTDIHSPHVTVYESLLYSAWLRLPPDVDSATKNMFI
KEVMELMELTPLRDSLVGLPGVNGLTTEQRKRLTIAVELVANPSIIFMDEPTSGLDARAAAIVMRTVRNTVDT
GRTVVCTIHQPSIDIFDAFDELFLLKRGGEAIYVGPIGRHSSHLIEYFEGIEGVPKIQDGYNPATWMLEITSPAHE
AALGVDFTDIYRNSELYRRNKALIKELSMPSPGSKDLSFPTQYSQSFLNQCMACLWKQHLSYWRNPPYTAVRL
MFATFVAILFGTIFWDLGSRRKTRQDLFNAIGSMYAAVLFIGTQNSSSVQPVVAIERTVFYRERAAGMYSALPY
AFGQIVIELPYVFIQTIIYSVIVYAMIGFEWTLTKFFWYLFFMYFTLLYFTFYGMMAVAITPNHQISSIVSASFYAI
WNVFSGFLIPLTRIPIWWRWYYWACPVSWTLYGLVASQFGDIKDTLHSGETVEEFVRNYFGYSQDFLGVVAV
VHIGMPLLFGFIFAFSIKAFNFQHR

>XP_022148619.1_PDR1-l_Mc
MDSGEIYRVSSARINSSSIWRNSAMEVFSRSSRDEDDEEALKWASIEKLPTYLRVRRGILSLEGESAREIDVKNL
GLLERRNVLERLVKIAEEDNERFLLKLKDRMERVGLDLPAIEVRFEHLEVEAEAHTAGRALPTMFNFSLNMLE
GFLNYFHIVRSRKNQLSILHDVSGIIKPGRMTLLLGPPSSGKTTLLKALAGKLGKDLKFSGRVTYNGHGMNEFV
PQRTSAYISQQDLHIGEMTVRETLSFSARCQGVGPRYDMLTELSRREKAANIKPDPDLDIIMKAAALGGQETNV
VTDYVLKILGLEICADTMVGDEMFRGISGGQKKRVTTGEMLVGPARALFMDEISTGLDSSTTYQIVNSMRQSIH
ILNGTALISLLQPAPETYDLFDDIILISDGQIVYQGPRENVLEFFQHMGFTCPQRKGVADFLQEVTSRKDQEQYW
ANRDEPYSFISVEEFSEAFQSFHVGKKLGDELATPFDKSKSHPAALTTKKYGASKKELLKACISRELLLMKRNS
FVYIFKLTQLILMAFLTMTLFFRTEMHRKTVDDGAVYMGALFFAIIIIMFNGFSELALTILKLPVFYKQRDFLFFP
SWAYSLPTWVLKIPITFVEVGIWVVMTYYVVGFDPNAGRFFKQFLLLLLVNQMASALFRLIGALGRNIIVANTF
GSFALLTVLVLGGFVLARGDVHPWWIWGYWISPMMYAQNALAVNEFLGHKWRHLAPNSTEFLGVSVLKSRG
IFPQASWYWIGVGATIGYILLFNFLFTIALKYLDPFEKPQAVLSKESSTDKSVKKSQEVQELELSSKGKSSSERRT
ENQLSLSSRTSSARVGSISEADPNKKRGMVLPFEPHSITFDEIRYAVDMPQEMKSQGITEDRLELLKGVSGSFRP
GVLTALMGVSGAGKTTLMDVLAGRKTGGYIEGSITISGYPKKQETFARISGYCEQTDIHSPHVTVYESLVYSA
WLRLPPEVDSATRKMFVEEVMELIELNPLRDAIVGLPGVNGLSTEQRKRLTIAVELVANPSIIFMDEPTSGLDAR
AAAIVMRTVRNTVDTGRTVVCTIHQPSIDIFDAFDELFLLRRGGEEIYVGPIGRHSSQLIEYFESIEGVPKIKDGY
NPATWMLEVTTAGQEATLGVNFNTLYKDSELYRRNKALIKELSVATENSKELYFPTKYSQSFVIQCMACLWK
QHLSYWRNPPYSAVRFLFTTFIALMFGTIFWDLGSKRGTQQDLFNAMGSMYAAVLFIGVQNATSVQPVVAIER
TVFYRERAAGMYSALPYAFGQVAIELPYIFIQTIVYGVIVYGMIGFEWTAAKFFWYIFFMYFTLLYFTFYGMMT
VAVTPNHNIAAIVSSAFYGFWNLFSGFIVPRTRIPIWWRWYYWICPVAWTLYGLVTSQFGDISEQMDTNQTVA
EFVSSYFGYKHDFLGVVAAVHVGITVLFGFIFAFSIKVFNFQKR

>XP_022721376.1_PDR1-l_X2_Dz
MEGGDIYRASSSLRRSLRSGSSSIWRNNGVEIFSRSSRDENDEEALKWAALEKLPTVARLRKGILTSSHGGANEI
DVYDLGWQERRTLLERLVKVAEEDNEKFLSKLKNRIDTVGIDLPTIEVRFERLNVEAEAFVGNSALPTVLNFTT
RMVEGFLNDMGILSSRKKQLTILNDVSGIVKPGRMTLLLGPPSSGKTSLLLALAGKLDPALKCSGRVTYNGHG
MDEFVPQRTAAYISQHDLHIGEMTVRETLAFSARCQGVGSRFEMLAELSRREKQANIKPDPDVDVFMKATATE
GQEANVITDYILKVLGLEVCADTMVGNEILRGISGGQRKRVTTGEMLVGPAKALFMDEISTGLDSSTTFQIVNS
LKQSVHILNGTAVISLLQPAPETYNLFDDIILLSDGRIVYQGPREHVLTFFESMGFRCPERKGAADFLQEVTSRK
DQMQYWIRRDQTYRFITAKEFAEAFQSFHVGMKLGDELGTQFDKTKSHPAALTTRKYGVRKKELLKACISRE
YLLMKRNSFVYIFKFIQLTMMALITMTLFLRTEMSRDSVERGGIYMGALFFGLVMIMFNGMAELSMTIAKLPV
FYKQRDLLFFPSWAYALPTWILKIPITFVEVGIWVFLTYYVIGFDPNVERLFRQYLVLILVNQMASGLFRFIAAS
GRNMIVANTFGPFALLVLFALGGFVLSREDIKKWWIWGYWISPMMYGQNALMVNEFLGNQWKHVLPNSNES
LGIQVLKSRGFFQDPHWYWIGAGGLIGFTLLFNICFTLALTYLNPFEKPQAVISDEPESNEQTNGVDVGGSIQLS
NYESSSSRVTKSGIRTHLYFCQRGTVGALANKKKGMVLPFEPHSITFDDIVYSVDMPQEMKEQGVTEDRLLLL

KGVSGAFRPGVLTALMGVSGAGKTTLMDVLAGRKTGGYIDGNITVSGFPKKQETFARVSGYCEQNDIHSPHV
TVYESLLYSAWLRLPAEVDAEARKMFIEEVMELVELNPLRQSLVGLPGVNGLSTEQRKRLTIAVELVANPSIIF
MDEPTSGLDARAAAIVMRTVRNTVDTGRTVVCTIHQPSIDIFEAFDELFLMKRGGQEIYVGPLGHHSKHLIKYF
EGIQGVSKIKDGYNPATWMLEVTTSAQELALGVDFADVYKNSELYRRNKALIEDLSKPAPGSKELYFSTQYSQ
PFLTQCAACLWKQHWSYWRNPPYTAVRFLFTTIIALMFGTLFWDLGSKTKKRQDLANAMGSMYAAVLFLGV
QNSASVQPVVAVERTVFYRERAAGMYSAMPYAIAQVLIEIPYIFIQAVVYGIIVYAMIGFEWTAAKFFWYIFFM
YFTFLYFTFYGMMAVAVTPNYHIASIVSSAFYGIWNVFSGFIIPRLSIPVWWRWYYWICPVSWTLYGLVVSQFG
DLQDTLEDGNGETVEQYLRNFFGFRHEFLGVVAAVILGFTVLFGAIFTVSIKVFNFQRR

>XP_022755318.1_PDR1-l_X1_Dz
MEGGDSYRVSSARIGSSSIWRNNPMEAAFSKSFREEDDEQALQWAAIERLPTYLRVRRGILTEEEGQSREVDIK
NLGFIERRNLLERLVKIAEDDNERFLLKLKERIDRVGLDMPTIEVRFEHLTVEAEAYVGSRALPTMFNFCVNILE
GLLSYLHILPSRKKPLPILNDISGIIRPRRMTLLLGPPSSGKTTLLLSLAGKLDKDLKFSGRVTYNGHGMEEFVPQ
RTSAYISQYDLHIGEMTVRETLAFSARCQGVGPRYEMLAELSRREKEANIKPDPDIDIYMKAAALEGQEASVVT
DYILKILGLDVCADTMVGDEMIRGISGGQRKRVTTGEMLVGPARALFMDEISTGLDSSTTFQIVNSLRQSIHILN
GTALISLLQPAPETYDLFDDIILLSDGQIAYQGPRENVLEFFEYMGFKCPERKGVADFLQEVTSRKDQEQYWAH
KDEPYSFISVKEVAEAFQSFHIGQKLGDDIAIPFDKSKSHPAALTKDKYGVSKKELLKACVSREFLLMKRNIFVY
VFKTIQLIFVGLITMTIFLRTEMHRDTTADGGIFMGALFFILIMIMFNGFAELAMTILKLPVFYKQRDLLFYPPWA
YSLPAWILKIPISILEVTIWVFSSYYVIGFDPDSGRFFKYYLLLICLSQMASGLFRLMGGLGRNIIVANTCGSFALL
AVLVMGGFILTRDDVKKWWIWGYWISPLMYGQNAIAVNEFLGKSWSHVPSNSTEPLGVSILKSRGIFPEAHW
YWIGVGALIGYCFLFNLLFTLALKYLDPFGKPQAVISKETLAEKIASKTGENVELSSRGRGSSERGNESRRSASS
RSLSAKVGSINESDQNRKRGMVLPFEPLSMTFDEIKYAVDMAQEMKAQGISEDRLELLKGVSGAFRPGVLTAL
MGISGAGKTTLMDVLAGRKTGGYVEGTIKISGYAKKQETFARISGYCEQTDIHSPHVTVYESLLFSAWLRLPPE
VNSETRMMFIEEVMELVELTSLRDALVGLPGVNGLSTEQRKRLTIAVELVANPSIIFMDEPTSGLDARAAAIVM
RTVRNTVDTGRTVVCTIHQPSIDIFDAFDELLLLKRGGEEIYVGPLGRHSCHLIKYFEEINGIPRIKDGYNPATW
MLEVTSAAQEKALRVNFTDIYKNSELYRRNKQLVKELSSPAPGSKDLQFQTRYSQSLLTQCIACLWKQYWSY
WRNPPYTAVRFLFTTVIALLFGTIFWDLGSKRTRQQDVLNSMGSMYAAVVFIGFQNGSSVQPVVAIERTVFYR
ERAAGMYSALPYAFGQVVIELPYILIQTVIYGVIVYAMIGFEWTPTKFFWYLFFMYFTFLYFTFYGMMTVAVTP
NHNIAAIVSSAFYALWNLFSGFIVPRTRIPVWWRWYYWACPISWTLYGLIASQYGDIQHAFESGETVEHFVRN
YFGFRQEFVGVVAIVVVGICVLFGFMFAFSIKAFNFQKR

>XP_022929578.1_PDR1-l_Cmo
MEVFSRSSCGDDDEEALKWAAIEKLPTYLRIRRGILKEEQGEAREIDIRNLGLLERRHVLERLVKIAEEDNEKFL
LKLRDRIERVGLEIPTIEVRFEHLNVEAEVYVGRRALPTMFNFSLNIFEGLLNYLHILPSRKKPLSILHDVSGIIKP
KRMTLLLGPPSSGKTTLLLALAGKLGKDLKFSGKVSYNGHGMEEFVPQRTSAYISQHDLHIGEMTVRETLAFS
ARCQGVGSRYEMLAELSRREKAANIKPDPDLDIYMKAEALEGQETSIITDYILKILGLEMCADTMVGDDMIRGI
SGGQRKRLTTGEMLVGPARALFMDEISNGLDSSTTYQMVNSLRQSIHILNGTAVISLLQPAPETFDLFDDIILLSN
GHIVYQGPREDVLSFFAHMGFKCPERKGVADFLQEVTSRKDQEQYWAIREEPYRFVSVKEFSEAFQSFHIGRQL
GDELATPFNRSKSHPATLTTKKYGVSKKEVLKACISREILLMKRNSFVYIFKMFQLIIMALVTMTLFLRTELHRD
TELDGGIYMGALFFTLIVIMFNGFSELAMTVVKLPVFYKQRDLLFYPSWTYALPTWILKIPITCLEVGIWVVMT
YYVIGFDPNIERFLKQFLLLFCINQMASGLFRLAAGLGRNVVVATTCATLALPVVMVLGGFIVAREDVHPWWL
WGYWVSPMMYGQNAIAVNEFLGDSWRHVLPNSSEPLGVTILKSRGIFPEAYWYWIGVGATIGYVLLFNFLFIL
ALHYLDPFGKPQAVLSEDTLAEKNANKTGEFEPPTKTNIFFEREKESQNISSRTLSTRVGSTSEFNHNNNRGMV
LPYEPHSITFDEIRYAVDMPQEMKAQGVVEDKLELLKGVSGAFRPGVLTALMGVSGAGKTTLMDVLAGRKTG
GYTEGTITISGFPKRQETFARISGYCEQTDIHSPHVTVYESLVYSAWLRLPPDVDSGTKNMFIMEVMELMELTPL
RDALVGLPGVNGLTTEQRKRLTIAVELVANPSIIFMDEPTSGLDARAAAIVMRTVRSTVDTGRTVVCTIHQPSID
IFDAFDELFLLKRGGEAIFVGPIGRHSSHLIDYFEGVEGVPKIKDGYNPATWMLEITSPAQEAALGVNFTDIYRN
SELYRRNKALIKELSMPPPGSKDLLFPTQYSQSFLNQCMACLWKQHLSYWRNPPYTIVRLLFATFVAILFGTIF
WDLGSRRKTRQDLFNAIGSMYVAVLFIGTQNSSSVQPVVAIERTVFYRERAAGMYSALPYAFGQIMIELPYVFI
QTTIYSVIVYAMIGFEWTATKFFWYLFFMYFTLLYFTFYGMLAVAITPNDQISSIVSASFYTIWNVFSGFLIPRTR
IPIWWRWYYWACPVSWTLYGLVASQFGDIKDTLQSGETVEEFIRNYFGYRQEFLGVVAVVHIGMPLLFGFIFA
FSIKAFNFQKR

>XP_022943928.1_PDR1-l_Cmo

MDNGEIYRVSSTRMNSSTIWRNSAMDVFSKSSRDEDDEEALKWASIERLPTYLRVRRGILSLEGESAREIDVQN
LGLLERRNILERLVKIAEEDNERFLLKLKNRMERVGLDLPAIEVRFEHLNVEAEAHTADRALPTIFNFSLNMLE
GFLSYCHIIPNRKKHLSILHDVSGIIKPGRISLLLGPPSSGKTTLLRALSGKLGNDLKVSGRVTYNGHGMNEFVPQ
RTSAYISQQDLHIGEMTVRETLSFSARCQGVDQRYDMLTELSRREKAANIKPDQDLDIIMKAAALGGQETNVV
TDYVLKILGLEICADTMVGDEMFRGISGGQKKRVTTGEMLVGPARALFMDEISTGLDSSTTYQIVNSLRQSIQIL
NGTALISLLQPAPETYELFDDIILISDGQVVYQGPRENVLEFFEHMGFTCPQRKGVADFLQEVTSRKDQEQYWA
KRDEPYRFIDVGEFSEAFQSFHVGKKLGDELAIPFDKSKSHPAALTTKTYGATKKELLKACISREFLLMKRNSF
VYIFKLTQLIIMSFVAMTLFFRTKMHRRTTDDGSIYMGAMFFAILIIMFNGFSELALTILKLPVFYKQRDLLFFPA
WAYSLPTWILKIPITIVEVGIWVAMTYYVIGFDPNVERFFKQFLLLLCVNQMASALFRLIASLGRNLIVSNTFGSF
ALLTVLVLGGFIIARENIHPWWIWGYWISPMMYAQNAIGVNEFLGHKWSHLAPNSTESLGVVVLKSRGIFPEA
RWYWIGVAATIGYILLFNFLFTIALKYLDPFGKPQAILSSKESSTEKSVKKSEDVQEGNSSYNAERSENEVSLSS
RTSSARVGSINEADRNKKRGMVLPFEPHTITFDEIRYAVDMPQEMKSEGVTEDRLELLKGVSGSFRPGVLTAL
MGVSGAGKTTLMDVLAGRKTGGYIDGNITISGYPKNQETFARISGYCEQTDIHSPHVTVYESLVYSAWLRLPP
DVDNATRKMFVEEVMNLIELNPIRDSIVGLPGVNGLSTEQRKRLTIAVELVANPSIIFMDEPTSGLDARAAAIVM
RTVRNTVDTGRTVVCTIHQPSIDIFDAFDELFLLRRGGEEIYVGPIGRHSIHLIDYFESIEGVPKIKDGYNPATWM
LEVTTASQEANLGVNFNTLYKKSELFRRNKDLIKQLSVPSENSKELYFPTKYSQSFVVQCIACLWKQHLSYWR
NPSYSAVRFLFTTIIALMFGTIFWDLGSKRDNQQDLFNAMGSMYAAVLFVGVQNATSVQPVVAIERTVFYREK
AAGMYSALPYAFGQVVIELPYIFIQTIVYGVIVYGMIGFEWTAAKFFWYIFFMYFTLLYFTFYGMMTVAVTPN
HNIAAIVSSAFYGFWNLFSGFIIPRTKIPIWWRWYYWICPVAWTLYGLVTSQFGDIEDRMDSNQTVAEFVRSYF
GYKYDFLGIVAAVHVGFTVLFGFIFAFSIKVFNFQNR

>XP_022986442.1_PDR1-l_Cma
MDNGEIYRVSSTRMNSSTIWRNSAMDVFSKSSRDEDDEEALKWASIERLPTYLRVRRGILSLEGESAREIDVQN
LGLLERRNILERLVKIAEEDNERFLLKLKNRMERVGLDLPAIEVRFEHLNVEAEAHTADRALPTVFNFSLNMLE
GFLSYFHIIPNRKKHLSILHDVSGIIKPGRISLLLGPPSSGKTTLLRALSGKLGNDLKVSGRVTYNGHGMNEFVPQ
RTSAYISQQDLHIGEMTVRETLSFSARCQGVDQRYDMLTELSRREKAANIKPDQDLDIIMKAAALGGQETNVV
TDYVLKILGLEICADTMVGDEMFRGISGGQKKRVTTGEMLVGPARAMFMDEISTGLDSSTTYQIVNSLRQSIQI
LNGTALISLLQPAPETYDLFDDIILISDGQVVYQGPRENVLEFFEHMGFSCPQRKGVADFLQEVTSRKDQEQYW
AKRDEPYRFIDVGEFSEAFQSFHVGKKLGDELAIPFDKSRSHPAALTTKRYGASKKELLKACISREFLLMKRNSF
VYIFKLVQLIIMSFVAMTLFLRTEMHRRTTDDGLAYMGAMFFAILIIMFNGFSELALTILKLPVFYKQRDLLFFP
AWAYSLPTWILKIPISIVEVGIWVAMTYYVIGFDPNVERFFKQFLLLLCVNQMASALFRLIASLGRNLIVSNTFG
SFALLTVLVLGGFILSRENIHPWWIWGYWISPMMYAQNAIGVNEFLGHKWSHLAPNSTESLGVVVLKARGIFP
EARWYWIGVAATIGYILLFNFLFTIALKYLDPFGKPQAVLSSKESSTEKSVKKSEDVQEGNSTYNAERSENEVS
LSSRTSSARVGSINEADRNKKRGMVLPFEPHTITFDEIRYAVDMPQEMKSQGVTEDRLELLKGVSGSFRPGVLT
ALMGISGAGKTTLMDVLAGRKTGGYIDGNITISGYPKNQETFARISGYCEQTDIHSPHVTVYESLVYSAWLRLP
PNVDNATRKMFVEEVMNLIELNPIRDSIVGLPGVNGLSTEQRKRLTIAVELVANPSIIFMDEPTSGLDARAAAIV
MRTVRNTVDTGRTVVCTIHQPSIDIFDAFDELFLLRRGGEEIYVGPIGRHSIHLIDYFERIEGVPKIKDGYNPATW
MLEVTTASQEANLGVNFNTLYKESELFRRNKDLIKQLSVPSENSKELYFPTKYSQSFVVQCIACLWKQHLSYW
RNPSYSAVRFLFTTIIALMFGTIFWDLGSKRDTQQDLFNAMGSMYTAVLFVGVQNATSVQPVVAIERTVFYRE
KAAGMYSALPYAFGQVVIELPYIFIQTIVYGVIVYGMIGFEWTAAKFFWYIFFMYFTLLYFTFYGMMTVAVTP
NHNIAAIVSSAFYGFWNLFSGFIIPRTKIPIWWRWYYWICPVAWTLYGLVTSQFGDIEDRMDSNQTVAEFVRSY
FGYKHDFLGIVAAVHVGFTVLFGFIFAFSIKFFNFQNR

>XP_023521772.1_PDR1-l_Cp
MDSGEIYRVSSTRMNSSTIWRNSAMDVFSKSSRDEDDEEALKWASIERLPTYLRVRRGILSLEGESAREIDVQN
LGLMERRNILERLVKIAEEDNERFLLKLKNRMERVGLDLPAIEVRFEHLNVEAEAHTADRALPTIFNFSLNMLE
GFLSYFHIIPNRKKHLSILHDVSGIIKPGRISLLLGPPSSGKTTLLRALSGKLGNDLKVSGRVTYNGHGMNEFVPQ
RTSAYISQQDLHIGEMTVRETLSFSARCQGVDQRYDMLTELSRREKAANIKPDQDLDIIMKAAALGGQETNVV
TDYVLKILGLEICADTMVGDEMFRGISGGQKKRVTTGEMLVGPARALFMDEISTGLDSSTTYQIVNSLRQSIQIL
NGTALISLLQPAPETYDLFDDIILISDGQVVYQGPRENVLEFFEHMGFTCPQRKGVADFLQEVTSRKDQEQYWA
KRDEPYRFIDVGEFSEAFQSFHVGKKLGDELAIPFDKSRSHPAALTTKTYGATKKELLKACISREFLLMKRNSFV
YIFKLVQLIIMSFVAMTLFFRTKMHRRTTDDGSIYMGAMFFAILIIMFNGFSELALTILKLPVFYKQRDLLFFPA
WAYSLPTWILKIPITIVEVGIWVAMTYYVIGFDPNVERFFKQFLLLLCVNQMASALFRLIASLGRNLIVSNTFGSF
ALLTVLVLGGFIIARENIHPWWIWGYWISPMMYAQNAIGVNEFLGHKWSHLAPNSTESLGVVVLKSRGIFPEA

RWYWIGVAATIGYILLFNFLFTIALKYLDPFGKPQAILSSKESSTEKSVKKSEDIQEGNSTYNAERSENEVSLSSR
TSSARVGSINEADRNKKRGMVLPFEPHTITFDEIRYAVDMPQEMKSEGVTEDRLELLKGVSGSFRPGVLTALM
GVSGAGKTTLMDVLAGRKTGGYIDGNITISGYPKNQETFARISGYCEQTDIHSPHVTVYESLVYSAWLRLPPNV
DTATRKMFVEEVMNLIELNPIRDSIVGLPGVNGLSTEQRKRLTIAVELVANPSIIFMDEPTSGLDARAAAIVMRT
VRNTVDTGRTVVCTIHQPSIDIFDAFDELFLLRRGGEEIYVGPIGRHSIHLIDYFESIEGVPKIKDGYNPATWMLE
VTTASQEANLGVNFNTLYKKSELFRRNKDLIKQLSVPSENSKELYFPTKYSQSFVVQCIACLWKQHLSYWRNP
SYSAVRFLFTTIIALMFGTIFWDLGSKRDNQQDLFNAMGSMYAAVLFVGVQNATSVQPVVAIERTVFYREKAA
GMYSALPYAFGQVVIELPYIFIQTIVYGVIVYGMIGFEWTAAKFFWYIFFMYFTLLYFTFYGMMTVAVTPNHNI
AAIVSSAFYGFWNLFSGFIIPRTKIPIWWRWYYWICPVAWTLYGLVTSQFGDIEDRMDSNQTVAEFVRSYFGYK
YDFLGIVAAVHVGFTVLFGFIFAFSIKVFNFQNR

>XP_023644801.1_ABCG40_Cr
MEGTSFQKASNSLRRNSSVWRKDSGMEIFSRSSREEDDEEALRWAALEKLPTFDRLRKGILTASHAGGAINEID
IQKLGFQDTKKLLERLIKVGDDEHEKLLWKLKKRIDRVGIDLPTIEVRFENLKIEAEVHVGGRALPTFVNFMSN
FADKFLNTLHLVPNRKKKFTILNDVNGIVKPGRMALLLGPPSSGKTTLLLALAGKLDQELKQTGKVTYNGHG
MNEFVPQRTAAYIGQNDVHIGEMTVRETFAYAARFQGVGSRYDMLSELARREKEANIKPDPDIDVFMKALST
AGEETNVMTDYTLKILGLEVCADTMVGDDMLRGISGGQKKRVTTGEMLVGPSRALFMDEISTGLDSSTTYQIV
NSLRNYVHIFNGTALISLLQPAPETFNLFDDIILIAEGEIIYEGPRDHVVEFFETMGFKCPPRKGVADFLQEVTSK
KDQMQYWAQPDKPYRFIRVREFAEAFQSFHVGRRLGDELALPFDKTKSHPAALTTKKYGVGIKELVKTSFSRE
YLLMKRNSFVYYFKFGQLLVMAFLTMTLFFRTELQKKTEVDGNLYTGALFFILMMLMFNGMSELSMTIAKLP
VFYKQRDLLFYPAWVYSLPPWLLKIPISFIEAALTTFITYYVIGFDPNVGRLFKQYILLVLMNQMASALFKLVAA
LGRNMIVANTFGAFAMLVFFALGGVVLAREDIKKWWIWGYWISPIMYGQNAIMANEFFGHSWSRAVQNSNQ
TLGVTVLKSRGFLPHAYWYWIGTGALLGFVILFNFGFTLALTFLNSLGKPQAVITEEPASDGNETELQSAQSDG
VVEAGANKKRGMVLPFEPHSITFDNVVYSVDMPQEMIEQGTHEDRLVLLKGVNGAFRPGVLTALMGVSGAG
KTTLMDVLAGRKTGGYIDGNITISGYPKNQKTFARISGYCEQTDIHSPHVTVYESLVYSAWLRLPKDVDANKR
KMFIEEVMELVELTPLRQALVGLPGESGLSTEQRKRLTIAVELVANPSIIFMDEPTSGLDARAAAIVMRTVRNT
VDTGRTVVCTIHQPSIDIFEAFDELFLLKRGGEEIYVGPLGHESTHLINYFESIQGINKITEGYNPATWMLEVSNT
SQEAALGVDFAQLYKNSDLYKRNKELIKELSQPAPGSKDLYFPTQYSQSFWTQCMASLWKQHWSYWRNPPY
TAVRFLFTIGIALMFGTMFWDLGGKTKTRQDLSNAMGSMYTAVLFLGLQNAASVQPVVNVERTVFYREQAA
GMYSAMPYAFAQVFIEIPYVLVQAVVYGLIVYAMIGFEWTAAKFFWYLFFMYGSFLTFTFYGMMAVAMTPN
HHIASVVSSAFYGIWNLFSGFLIPRPSMPVWWEWYYWLCPVAWTLYGLIVSQFGDITETMADSETTVKQYIRE
FYGYREGFLGVVAAMNVIFPLLFAVIFAVGIKSFNFQKR

>XP_023729882.1_PDR1-l_Ls
MEGSDVFRVSSARISSSNIWRNSGRDIFSRSSLDEDDEEALKWAAIEKLPTNLRLERGILTEEKGHPREIDIKNLG
LVEKRNLLERLVKIAEEDNEKFLLKLKNRIDRVGLQLPTIEVRFEHLNIDAEAYIGGRALPTIFNFLVNIFEGSLN
YLHLFPSRKIPLPILHDVSGIIKPGRMALLLGPPSSGKTTLLLALAGKLGTDLQVSGRVTYNGHGMDEFVPQRSS
AYISQHDLHIGEMTVRETLAFSARCQGVGASYEMLAELSRREKEANIKPDPDLDIYMKAASLEGQEASVVTEY
VIKILGLEVCADTVVGDEMFRGISGGQKKRVTTGEMMVGPARALFMDEISTGLDSSTTFQIVNSIRQSIHILQGT
SVISLLQPAPETYELFDDIILLSDGQIVYQGPRENVLEFFEQMGFKCPERKGVADFLQEVTSRKDQEQYWTRRD
HSYNFVTSREFAEAFQLFHVGRSLGDELSTPFDRSKSHPAALTKKKFGVSKKELLKACTSREYLLMKRNSFVYI
FKMTQLIFMATITLTLFLRTEMPKNTLVDGTIFMGALFFILVMITFNGFSELALSIMKLPVFYKQRDLLFFPAWA
YSIPTWVLKIPVTIVEVATWVIMTYYVIGFDSDPGRFFKQMFLLICIQQMSSALFRFMGALGRNIIVANTFGSFGL
LTIFVLGGFILARDDIKGWWIWCYWFSPMMYGQNGVAVNEFLGKSWSKVLPNTTETIGVTILKSRGIFPEARW
YWIAVGVSIAYVFIFNICFTLALEYLNPFGSSQTVLSEESMAARKDMKTIQPIESSSRAPSSMPMSSRVGSINDID
QNKKRGMVLPFVPLSITFDDIKYAVDMPQEMKAEGVAQDRLELLKGVSGAFRPGVLTALMGISGAGKTTLMD
VLAGRKTGGYIEGSITISGYPKKQETFARIAGYCEQTDIHSPHVTIYESLQYSAWLRLPPEVDLATRQMFIEEVM
ELVELVPLREALVGLPGVNGLSTEQRKRLTIAVELVANPSIIFMDEPTSGLDARAAAIVMRTVRNTVDTGRTVV
CTIHQPSIDIFDAFDELFLLKRGGEEIYVGPLGRHSSHLIKYFEEIDGVHKIKDGYNPATWMLEVTSASQEAILGV
DFAEVYKNSELYKSNKEMIKESSKPIPGSSDIQFHTQYSQSFWTQCMACLWKQHWSYWRNPPYTAVRFLFTTF
IALMFGTIFWDMGSKREKKQDLFNAMGSMYAAILFIGVQNATSVQPVVSIERTVFYRERAAGMYSALPYAFG
QVVIEIPYIFVQTIVYGIIVYAMIGFEWTIVKFFWYLFFMYFTFLYFTFYGMMTVAVTPNHNFAAIISSAFYAIWN
LFSGFIVPKTRIPIWWRWYYYICPIAWTLYGLVASQFGDITDRLDSGETVGEFIHDFYGFEYDFIKYVAVIIVGFT
VLFGFIFAYSIKAFNFQRR

>XP_023870369.1_PDR1-l_X1_Qs
MEGGGGGEIFRVSSARLSSSNIWRNTGLEVFSKSSLEEDDEEALKWAAIEKLPTYLRIRRGILTEAEGQAREIDIE
SLGLLERKNLLERLVKIAEEDNEKFLLKLKERIERVGLDMPTIEVRFEHLDVEAEAYVGGRALPTIFNFSVNMLE
GFLNYLHILPSRKKPLPILHDVSGIIKPRRMTLLLGPPSSGKTTLLLALAGKLGKELKSSGRVTYNGHGMEEFVP
QRTSAYISQHDVHIGEMTVRETLAFSARCQGVGPRYEMLAELSRREKTANIKPDPDLDIYMKAASLEGQETNVI
TDYILKILGLEVCADTMVGDEMLRGISGGQRKRLTTGEMLVGPARALFMDEISTGLDSSTTFQIVNSIRQSIHIL
NGTAVISLLQPAPETYDLFDDIILLSDGQIVYQGPRENVLEFFEYMGFRCPERKGVADFLQEVTSRKDQEQYWA
HTDEAYSYVSVKEFSEAFQSFHIGRKIGDELATPFDKSSSHPAALTTKKYGVNKKELFKACISRELLLMKRNSF
VYFFKMFQLISTAFITMTLFIRTEMHRDTVTDGGIYMGAMFFIIIIIMFNGFSELAMTIMKLPVFYKQRDLLFYPS
WAYSLPTWILKIPITFLEVAIWVVMTYYVIGFDPNIGRFFKQFLLLLFINQMASGLFRLMAALGRNVIVANTFGS
FALLAVLVLGGFVLSRDNVQKWWLWGYWVSPMMYAQNAIAVNEFLGKSWRHVPPNSTEPLGVSVLKSRGV
FTEAYWYWIGVGASIGYIFVFNFLFTLALEYLDPFGKPQAVISKEALAEKNANRTGESIELPSRGKSSSGGSSSSK
IYKRGNESRRSVSSRTLSARVSGINDASNKSNRGMVLPFEPLSITFDDIRYAVDMPQEMKVQGVLEDRLELLKG
VSGTFRPGVLTALMGVSGAGKTTLLDVLAGRKTGGYIEGSITISGHPKKQETFARISGYCEQTDIHSPHVTVYES
LVYSAWLRLPPEADSATRKMFTEEVMELVELTSLREALVGLPGVNGLSTEQRKRLTIAVELVANPSIIFMDEPT
SGLDARAAAIVMRTVRNTVDTGRTVVCTIHQPSIDIFDAFDELFLLKRGGEEIYVGPLGRHSSHLIDYFEGIHGV
PKIKDKYNPATWMLEVTSAGQEAAFGVNFTEIYKSSELYRRNKALINELCTPPPGSKDLYFPTQYSQSFFSQCM
ACLWKQHLSYWRNPPYSAVRLLFTTFIALMFGTIFWDLGSKRRNRQDLFNAMGSIYAAVLFIGVQNASSVQPV
VAIERTVFYRERAAGMYSALPYAFGQVFIELPYIFVQTVIYGIIVYAMIGFDWTVSKFFWYIFFMYFTFLYFTFY
GMMTVAVTPNHNIAAIISSAFYAIWNLFSGFIVPRTRMPIWWRWYYWACPVSWTLYGLIASQFGDIKDTFDSG
ETVEDFLRSYFGYRNDFVGIVAIVIVGISVLFAFTFAYSIKAFNFQKR

>XP_023870370.1_PDR1-l_X2_Qs
MEGGGGGEIFRVSSARLSSSNIWRNTGLEVFSKSSLEEDDEEALKWAAIEKLPTYLRIRRGILTEAEGQAREIDIE
SLGLLERKNLLERLVKIAEEDNEKFLLKLKERIERVGLDMPTIEVRFEHLDVEAEAYVGGRALPTIFNFSVNMLE
GFLNYLHILPSRKKPLPILHDVSGIIKPRRMTLLLGPPSSGKTTLLLALAGKLGKELKSSGRVTYNGHGMEEFVP
QRTSAYISQHDVHIGEMTVRETLAFSARCQGVGPRYEMLAELSRREKTANIKPDPDLDIYMKAASLEGQETNVI
TDYILKILGLEVCADTMVGDEMLRGISGGQRKRLTTGEMLVGPARALFMDEISTGLDSSTTFQIVNSIRQSIHIL
NGTAVISLLQPAPETYDLFDDIILLSDGQIVYQGPRENVLEFFEYMGFRCPERKGVADFLQEVTSRKDQEQYWA
HTDEAYSYVSVKEFSEAFQSFHIGRKIGDELATPFDKSSSHPAALTTKKYGVNKKELFKACISRELLLMKRNSF
VYFFKMFQLISTAFITMTLFIRTEMHRDTVTDGGIYMGAMFFIIIIIMFNGFSELAMTIMKLPVFYKQRDLLFYPS
WAYSLPTWILKIPITFLEVAIWVVMTYYVIGFDPNIGRFFKQFLLLLFINQMASGLFRLMAALGRNVIVANTFGS
FALLAVLVLGGFVLSRDNVQKWWLWGYWVSPMMYAQNAIAVNEFLGKSWRHVPPNSTEPLGVSVLKSRGV
FTEAYWYWIGVGASIGYIFVFNFLFTLALEYLDRKYSTHIILTFGKPQAVISKEALAEKNANRTGESIELPSRGKS
SSGNESRRSVSSRTLSARVSGINDASNKSNRGMVLPFEPLSITFDDIRYAVDMPQEMKVQGVLEDRLELLKGVS
GTFRPGVLTALMGVSGAGKTTLLDVLAGRKTGGYIEGSITISGHPKKQETFARISGYCEQTDIHSPHVTVYESLV
YSAWLRLPPEADSATRKMFTEEVMELVELTSLREALVGLPGVNGLSTEQRKRLTIAVELVANPSIIFMDEPTSG
LDARAAAIVMRTVRNTVDTGRTVVCTIHQPSIDIFDAFDELFLLKRGGEEIYVGPLGRHSSHLIDYFEGIHGVPKI
KDKYNPATWMLEVTSAGQEAAFGVNFTEIYKSSELYRRNKALINELCTPPPGSKDLYFPTQYSQSFFSQCMAC
LWKQHLSYWRNPPYSAVRLLFTTFIALMFGTIFWDLGSKRRNRQDLFNAMGSIYAAVLFIGVQNASSVQPVVA
IERTVFYRERAAGMYSALPYAFGQVFIELPYIFVQTVIYGIIVYAMIGFDWTVSKFFWYIFFMYFTFLYFTFYGM
MTVAVTPNHNIAAIISSAFYAIWNLFSGFIVPRTRMPIWWRWYYWACPVSWTLYGLIASQFGDIKDTFDSGETV
EDFLRSYFGYRNDFVGIVAIVIVGISVLFAFTFAYSIKAFNFQKR

>XP_023908150.1_PDR1-l_X1_Qs
MEGGDIYKASSSLRVNSSSRWRNSSVEVFNRSSREEDDEEALKWAALEKLPTFDRLRKGILTTSKGEANEINIES
LGFEERKQLLERLVKVAEEDNEKFLMKVRNRIDRVGIDLPTIEVRFEHLNVEAEAYVGGRALPTFFNFSVNIVE
GFLNSLNILPSKKKHLSILKNVSGIIKPRRMTLLLGPPSSGKTTLLLALAGKLDPDLKLSGRVTYNGHGMNEFVP
QRTAAYISQYDLHIGEMTVRETLCFSARCQGVGSRYDMLAELSRREKEANIKPDPDIDIYMKAATTEGQEATV
VTDYTLKVLGLDVCADTMVGDEMIRGISGGQRKRVTTGEMLVGPAKAMFMDEISTGLDSSTTFQIVNSLKQY
VHILDGTAVISLLQPAPETYNLFDDIILLSDGYIVYQGPRELVLDFFESMGFKCPDRKGVADFLQEVTSRKDQEQ
YWARKDEPYSFVTVQEFAEAFQSFHVGRSIGDELSVPYDKSKSHPAALTTNKYGVNKKELLKANFSREYLLM
KRNSFVYIFKLTQLFMMALIAMTLFLRTKMPRESVTDGSIYTGALFFTVIMIMFNGMAELSMTIVKLPVFYKQR

DLLFYPSWVYALPTWILKIPVTFLEVGVWVFMTYYVIGFDPSPGRLFKQYLLLLLVNQMASALFRSIGAMGRN
MIIANTFGSFALLLLFALGGFILSKDDIKKWWIWGYWISPLMYGQNAIVVNEFLGGNWRNGSTTEALGISVLKS
RGFFTQAYWYWIGVGALVGFLLVFNIIFTLALTYLNPFGKAQSVKSDEPESNEQGQRTGEGIQSTLRGNSSSHH
SNIGSRRRSSSSARSGAEIEASRNGKRGMVLPFDQHSITFDDIIYSVDMPQEMKSQGVIEDKLVLLKGVSGAFRP
GVLTALMGVSGAGKTTLMDVLAGRKTGGYIEGNITISGFPKNQETFARISGYCEQNDIHSPHVTVYESLLYSA
WLRLSSEVDSETRKMFIEEVMELVELNPLRNALVGLPGVNGLSTEQRKRLTIAVELVANPSIIFMDEPTSGLDA
RAAAIVMRTVRNTVDTGRTVVCTIHQPSIDIFEAFDELFLMKRGGQEIYVGPLGHHSSHLIKYFESIEGIRKIKDG
YNPATWMLEVTSSAEEITLGVDFTNVYRNSDLYRRNKALIQELSKPAPNKKELYFPTQYAQSFFTQCMACLWK
QHWSYWRNPPYTAVRFLFTVFIALIFGTMFWDLGSKTKRQQDLFNAMGSMYAAVLFLGVQNASSVQPVVAV
ERTVFYRERAAGMYSALPYAFAQVTIELPYVFAQAVFYGIIVYAMIGFEWTVAKFFWYLFFMYFTLLYFTFYG
MMTVAVTPNHHIASIISSAFYAIWNLFSGFIIPRTRIPVWWRWYYWACPVAWTLYGLVVSQFGDIEELMNDGN
NESVKEFVKSYFGYKHDFLGVVAVVVAGVAVLFAFIFAVSIKIFNFQRR

>XP_023910843.1_PDR1-l_Qs
MEGGDIYKASSSLRVNSSSRWRNSSVEVFNRSSREEDDEEALKWAALEKLPTFDRLRKGILTTSKGEANEINIES
LGFEERKQLLERLVKVAEEDNEKFLMKVRNRIDRVGIDLPTIEVRFEHLNVEAEAYVGGRALPTFFNFSVNIVE
GFLNSLNILPSKKKHLSILKNVSGIIKPRRMTLLLGPPSSGKTTLLLALAGKLDPDLKLSGRVTYNGHGMNEFVP
QRTAAYISQYDLHIGEMTVRETLCFSARCQGVGSRYDMLAELSRREKEANIKPDPDIDIYMKAATTEGQEATV
VTDYTLKVLGLDVCADTMVGDEMIRGISGGQRKRVTTGEMLVGPAKAMFMDEISTGLDSSTTFQIVNSLKQY
VHILDGTAVISLLQPAPETYNLFDDIILLSDGYIVYQGPRELVLDFFESMGFKCPDRKGVADFLQEVTSRKDQEQ
YWARKDEPYSFVTVQEFAEAFQSFHVGRSIGDELSVPYDKSKSHPAALTTNKYGVNKKELLKANFSREYLLM
KRNSFVYIFKLTQLFMMALIAMTLFLRTKMPRESVTDGSIYTGALFFTVIMIMFNGMAELSMTIVKLPVFYKQR
DLLFYPSWVYALPTWILKIPVTFLEVGVWVFMTYYVIGFDPSPGRLFKQYLLLLLVNQMASALFRSIGAMGRN
MIIANTFGSFALLLLFALGGFILSKDDIKKWWIWGYWISPLMYGQNAIVVNEFLGGNWRNGSTTEALGISVLKS
RGFFTQAYWYWIGVGALVGFLLVFNIIFTLALTYLNPFGKAQTVKSDEPESNEQGQRTGEGIQSTLRGNSSSHH
SNIGSRRRSSSSARSGAEIEASRNGKRGMVLPFDQHSITFDDIIYSVDMPQEMKSQGVIEDKLVLLKGVSGAFRP
GVLTALMGVSGAGKTTLMDVLAGRKTGGYIEGNITISGFPKNQETFARISGYCEQNDIHSPHVTVYESLLYSA
WLRLSSEVDSETRKMFIEEVMELVELNPLRNALVGLPGVNGLSTEQRKRLTIAVELVANPSIIFMDEPTSGLDA
RAAAIVMRTVRNTVDTGRTVVCTIHQPSIDIFEAFDELFLMKRGGQEIYVGPLGHHSSHLIKYFESIEGIRKIKDG
YNPATWMLEVTSSAEEITLGVDFTNVYRNSDLYRRNKALIQELSKPAPNKKELYFPTQYAQSFFTQCMACLWK
QHWSYWRNPPYTAVRFLFTVFIALIFGTMFWDLGSKTKRQQDLFNAMGSMYAAVLFLGVQNASSVQPVVAV
ERTVFYRERAAGMYSALPYAFAQVTIELPYVFAQAVFYGIIVYAMIGFEWTVAKFFWYLFFMYFTLLYFTFYG
MMTVAVTPNHHIASIISSAFYAIWNLFSGFIIPRTRIPVWWRWYYWACPVAWTLYGLVVSQFGDIEELMNDGN
NESVKEFVKSYFGYKHDFLGVVAVVVAGVAVLFAFIFAVSIKIFNFQRR

>XP_024035984.1_PDR1_Cc
MESGNKVYKASNSLRIGSTSIWRSNSATLGAFSMSSRGEEDDEEALKWAALEKLPTYNRLKKGILTSSRGEAN
EVDVCNLGPQERQRLIDKLVKVADVDNEEFLLKLKNRIDRVGISLPTIEVRFEHLNVEAEAYVGSRALPTFFNF
CANIIEGFLNSVNILPSRKKHLTILKDVSGIIRPGRMTLLLGPPASGKTTLLLALAGKLDSSLRVSGRVTYNGHD
MDEFVPQRTAAYISQHDNHIGEMTVRETLAFSARCQGVGSRYEMLSELSRREKAAGIKPDPDIDVFMKAAATE
GQEASVVTDYILKILGLDVCADTMVGDEMLRGISGGQKKRVTTGEMMVGPAQALFMDEISTGLDSSTTFQIVN
SLRQSIHILKGTTLISLLQPAPETYDLFDDIILISDGQIVYQGPREHVLEFFKFMGFECPKRKGVADFLQEVTSRKD
QEQYWVHKEEPYRFVTVKEFADAFQVFYMGQKVGDELRIPFDKRKSHRAALTTKIYGVSKKELLKACMSREL
LLMKRNSFVYIFKLCQLTIMGLVAMTLFFRTKMHRDSITDGVIYTGALFFIVLMIMFNGMAEIPMTIAKLPIFYK
QRDLRFYPSWAYALSTWILKIPISYIEVAVWVFLTYYVIGFDPNVGRLFRQYLLLLFLNQMASALFRLIAATGR
NIVVANTFGSFALLLLFVLGGFVLSREDIKQWWIWAYWCSPLMYAQNAIVVNEFLGNSWRKVLPNTTEPLGV
QVLKSRGFFTDAYWYWLGLGALAGFILLFNFGFTLALSFLNPFGKNQAVISQESQSNEHDNRTGGTIQLSTSEIG
HDIMSTYSSSLIEAEVKANHHKKRGMVLPFKPHSITFDEIAYSVDMPQEMMRPGVLEDKLVLLNGVSGAFRPG
VLTALMGVSGAGKTTLMDVLAGRKTGGYISGSIMISGYPKKQETFARISGYCEQNDIHSPNVTVYESLLYSAW
LRLPLEVDSPTRKMFIEEVMELVELNPLRQALVGLPGVSGLSTEQRKRLTIAVELVANPSIIFMDEPTSGLDARA
AAIVMRTVRNTVDTGRTVVCTIHQPSIDIVEAFDELFLLKRGGQEIYVGPLGRHSSHLIKYFEGIRGVCKIKDGY
NPATWMLEVTAPSQETALGIDFADIYKSSELYRRNKALIKDISKPAPGSKDLHFATQYAQSFFTQCMACLWKQ
HWSYWRNPPYSAVRFLFTTIIALAFGTMFWDMGTKTKKQQDLFNAMGSMYTAVLFLGVQNAASVQPVVSIE
RTVFYRERAAGMYSALPYAFAQALIEIPYIFVQSVTYGVIVYAMIGFEWTAAKFLWYQFFMFFTLLYFTYYGM

MAVAMTPNHHISGIVAFAFYGLWNVFSGFIIPRTRIPIWWRWYYWACPVSWTLYGLVASQFGDIQDRLESGET
VEQFLRSFFGFKHDFLGVVAAVVFAFPVLFALIFAVGIKVFNFQKR

>XP_024163520.1_PDR1-l_Rc
MESSSGDVYRVSSARLSSSNIWRNSTMDVFSKSSHDEDDEEALKWAAIEKLPTYLRIRRGILTEEEGEAREIDIK
NLGLLERKNVLERLVKVADEDNERFLMKLKDRIDRVGLDIPTIEVRFEHLNVEAEAYVGGRALPTLYNFSVNIL
EGFLSFCRIIPTRKHPLPILDDVSGIIKPKRMTLLLGPPNSGKTTLLLALAGKLAKDLKSSGRVAYNGHGMEEFV
PERTSAYISQYDLHIGEMTVRETLAFSARCQGVGGRYEMLAELSRREKAANIMPEKDLDIYMKAASLEGQEAN
VVTDYILKILGLEVCADTMVGDEMFRGISGGQKKRVTTGEMLVGPARALFMDEISTGLDSSTTFQIVNSLRQTI
HILNGTALISLLQPAPETYDLFDDIILLSDGQIVYQGPRENVLEFFEYMGFKCPERKGVADFLQEVTSKKDQEQY
WIQKEEPYNFISSKEFAEAFQSFHIGRKLGDDLATPFDKSKGHPAALTTKKYGVSKRQLLKACIAREYLLMKRN
SFVYIFKMTQLTLLAFISMTLFFRTKMHRDTVEDGGIYMGAMFFTVIIIMFNGFSELALTIMKLPVFFKQRDLLF
YPAWAYSLPAWILKIPITFVEVAIWVFMTYYVIGFDPNPGRFFKQYLLLLCINQMASGLFRFMGALGRNIIVAN
TFGSFALLAVLVMGGFILSRENVQAWWLWGYWISPMMYGQNAIAVNEFLGDSWSHIPPDSTESLGIMVLKSR
GVFVQAYWYWIGVGATIGYIFLFNFFYTVALHYLDPFGKPQAVLSKEALADKNAARAGGNIELSSGGKNSSDA
KNDSRRSISSRTLSARVGSFNEANENRKRGMVLPFEPLWITFNEIKYSVDMPEEMKTQGITEERLPLLKGVTGA
FRPGVLTALMGISGAGKTTLMDVLAGRKTGGYIEGDITISGYPKKQETFARISGYCEQTDIHSPHVTVYESLVYS
AWLRLPPEVDSATRKMFVEEVMELVELTSIRESLVGLPGVNGLSTEQRKRLTIAVELVANPSIIFMDEPTSGLDA
RAAAIVMRTVRNTVDTGRTVVCTIHQPSIDIFDAFDELFLLKRGGEEIYVGPLGRHSSQLIKYFEEIDGVPKIKDG
YNPATWMLEVTSAAQEVVLGINFTDIYRSSELYRRNKDLIKELSTPPQGTKDLYFSTKYSQSFYYQCMACLWK
QHLSYWRNPPYSAVRLLFTTFIALMFGTIFWDLGSKRKNQQDLFNAMGSMYAAVLFIGVQNASSVQPVVAIER
TVFYRERAAGMYSALPYAFGQVVIELPYIFVQTVIYGVIVYAMIGFDWTVSKFLWYLFFMYFTFLYFTFYGMM
TVAVTPNHNIAAIVSSAFYAIWNLFSGFIVPRTRMPIWWRWYYWICPVSYTLYGLVASQFGDIKEVMDSGERV
EDFVRNYFGYRHDFVGIVAVVLVGISVLFGFTFAFSIKAFNFQKR

>XP_024445693.1_PDR1_Pt
MDGGGDIYRVSSARLSSSSNIWRNSTLDVFSRSSRDEDDEEALKWAAIEKLPTCLRMRRGILTEEEGQAREIDIA
SLGLIEKRNLVERLVKIAEEDNERFLLKLKERIHRVGLDIPTIEVRFEHLSIEAEAYVGGRALPTIFNFSANMLEG
FLSFLHILPSRKQPFPILHDLSGIIKPRRMTLLLGPPSSGKTTLLLALAGKLGKDLKSSGSVTYNGHGMAEFVPQR
TSAYISQYDLHIGEMTVRETLSFSARCQGVGPRYEMLTELSRREREANIKPDPDIDIFMKAAALEGQETTVTTD
YILKILGLDICADTMVGDEMIRGISGGQKKRLTTGEMLVGPARALFMDEISTGLDSSTTFQIANSLRQTTHILNG
TTFISLLQPAPETYDLFDDIILLSEGLIIYQGPRENVLEFFESLGFKCPERKGVADFLQEVTSRKDQEQYWACRDQ
PYSFVSAKEFSEAFQSFHIGRKLGDELATPFDKSKSHPAALTTEKYGVSKKELLKACISREFLLMKRNSFVYIFK
FTQLIILASITMTIFLRTEMHRNTIVDGGIYLGALFFAIIVIMFNGFSELAMTIMKLPIFYKQRDLLFYPPWAYAIPT
WILKIPITFVEVAIWTIMTYYVIGFDPNIGRFFKQYLIFVLANQMSSGLFRMTGALGRNIIVANTFGSFAFLAVLV
LGGFILSRDNVKPWWIWGYWVSPLMYVQNAASVNEFLGHSWRHIPPNSTESLGVVVLKSRGIFPEAHWYWIG
IGALIGYTLLFNFLFTLALKYLNPFGKPQAMLSKEALAERNANRTGELIELSTRGKSSSVRGIDSRRSSSARPPSL
RMHSFGDASQNKRGMVLPFQPLSITFDEIRYSVDMPQEMKAQGILEDRLELLKGVSGAFRPGVLTALMGVSGA
GKTTLMDVLSGRKTGGYIEGRISISGYPKNQQTFARISGYCEQMDIHSPHVTVYESLVYSAWLRLSPDVDSETR
KMFIEEVVELVELNPLREALVGLPGVNGLSTEQRKRLTIAVELVANPSIIFMDEPTSGLDARAAAIVMRTVRNT
VDTGRTVVCTIHQPSIDIFDAFDELFLLKRGGEEIYVGPVGRHACHLIKYLEEIEGVPKIKDGHNPATWMLEVTS
AAQEALLGVDFTDIYKNSELFRRNKALIKELSSPPPGSNDLYFPTQYSHSFFTQCMACLWKQHWSYWRNPPYT
AVRLLFTTFIALMFGTIFWDMGSKRRNRQDIFNSMGSMYAAVLFIGVQNATSVQPVVAIERTVFYRERAAGMY
SALPYAFAQVMIEIPYVLVQTLIYGVIVYTMIGFDWTVSKFFWYIFFMYFTLLYMTFYGMMTVAVTPNHNVAA
IVSSAFYAIWNLFSGFIVPRTRIPIWWRWYFWACPISWTLYGLIASQYGDIKDKLEGDETVEDFVRNYFGFRHDF
VGTCAIVIVGICVLFAFTFAFSIRAFNFQRR

>XP_024453096.1_PDR1_X1_Pt
MESAVISRGSDSFRGSSRGVSSVWRNSTVEVFSRSSREEDDEEALKWAALEKLPTYDRLRKGILTSASRGIISEV
DIENLGVQERKQLLERLVKVADEDNEKFLWKLKNRVERVGIEFPTIEVRYENLNIEAEAYVGSSALPSFAKFIFH
IIEGFFIALHVLPSRKKPLTILKDVSGIIKPSRLTLLLGPPNSGKTTLLLAMAGKLDPSLKFSGHVTYNGHEMNEF
VPQRTAAYVSQHDLHIGEMTVRETLEFSARCQGVGHLHEMLAELSRREKEANIKPDQDVDVFMKAVATQGQ
EASVITDYVLKILGLEVCADTLVGDEMIRGISGGQRKRVTTGEMLVGPSRALLMDEISTGLDSSTTYQIVNSLK
QTIHVLNCTAVISLLQPAPETYDLFDDIILLSDGQIVYQGPRENVLGFFEHMGFKCPDRKGVADFLQEVTSKKD

QEQYWAIKDQPYRFVRVNEFSEAFQSFNVGRKIADELSIPFDKTKNHPAALVNKKYGAGKMDLLKANFSREY
LLMKRNSFVYIFKICQLTVVALISMSLFFRTKMHHDTVADGGIYTGALFFTVIIIMFNGMSELSMTIVKLPVFYK
QRELLFFPPWAYSIPPWILKIPVTFVEVAAWVLLTYYVIGFDPNVERLLRQYFLLLLINQMASALFRFIAAAGRN
MIVANTFGSFALLTLFALGGFILSREQIKKWWIWGYWLSPLMYGQNAIVVNEFLGHSWSHIPGTSTEPLGIQVL
KSREFFTEANWYWIGVGATVGFMLLFNICFALALTFLNAFEKPQAFIFEESEREGSVGKTGGAVQLSNHGSSHK
NKTENGDEINRNGFASIGEASDNRKRGMVLPFEPHSITFDDVIYSVDMPQEMKIQGVVEDRLVLLKGVNGAFR
PGVLTTLMGVSGAGKTTLMDVLAGRKTGGYIEGDIKISGYPKKQETFARIAGYCEQNDIHSPHVTVYESLLYSA
WLRLPPEVDSETRKMFIDEVMELVELDSLRNALVGLPGVNGLSTEQRKRLTIAVELVANPSIIFMDEPTSGLDA
RAAAIVMRTVRNTVDTGRTVVCTIHQPSIDIFDAFDELFLMKRGGEEIYVGPLGHHSTHLIKYFEAIEGVSKIKD
GYNPATWMLEVTASSQEMALEVDFANIYKNSDLFRRNKALIAELSTPAPGSKDVHFPTRYSTSFFTQCMACLW
KQHWSYWRNPPYTAVRFLFTTFIALMFGTMFWDLGSKVKTTQDLSNAMGSMYAAVLFLGFQNGTAVQPVV
AVERTVFYRERAAGMYSALPYAFAQALIELPYVFVQAAVYGVIVYAMIGFEWTAAKFFWYLFFMYFTLLYFT
FYGMMAVAVTPNHHIAGIVSTAFYAIWNLFSGFIIPRTRIPIWWRWYYWGCPVSWSLYGLVVSQYGDIQEPITA
TQTVEGYVKDYFGFDHDFLGVVAAVVLGWTVLFAFIFAFSIKAFNFQRR

>XP_024460182.1_PDR1_Pt
MDGAGDIYRVSSARLSTSSNKWRNSIPEVFSRSSRDEDDEEALKWAALEKLPTYLRLTRGILTEEEGKAREIDI
MNLGLVEKRDLLERLVKIAEEDNERFLLKLKERIDRVELEIPTIEVRFEHLNVEAEAYVGGRALPTILNFSANML
EGFLSFLHLLPSRKQPFPILRDVSGIIKPRRMTLLLGPPSSGKTTLLMALAGKLGKDLQCSGSVTYNGHGMEEFV
PQRTSAYISQFDLHIGEMTVRETLSFSARCQGVGPRYEMLTELSRREKEANIKPDPDLDIYMKAAALEGQETSV
TTYYILKITGLDICADTMVGDEMIRGISGGQKKRLTTGEMLVGPARALFMDEISTGLDSSTTFQIVNSLRQTTHI
LNGTTLISLLQPAPETYDLFDDVILLSDGLIVYQGPRENVLEFFESLGFKCPERKGVADFLQEVTSRKDQEQYW
ASRDQPYSFVSAKEFSEAFQSFHIGRKLGDELAIPFDKSKSHPSALSTEKYGVSKKELLKACISREFLLMKRNSF
VYIFKFTQLILLASIAMTVFLRTEMHRNTITDGGIYIGALFFAIIVIMFNGFSELVMTIMKLPVFYKQRDLLFYPP
WAYAIPTWILKIPITFVEVAIWTTMTYYAVGFDPNIGRFFKQYLIFVLANQMSSGLFRMMGALGRNVIVANNV
GSFALLAVLVMGGFILSRDNVKSWWIWGYWVSPLMYVQNAVSVNEFLGNSWRHIPPSSTESLGVTLLKSRGV
FPEARWYWIGVGALIGYTLLFNFLFTLALKYLNPFGKPQAILSKEALAERDANRTGNFIELSTRGKSSSERGKDS
KTNSSARAPSLRMPSLGDANQNKRGMVLPFQPLSITFEEIRYSVDMPQEMKAQGIPEDRLELLKGVSGAFRSGV
LTALMGVSGAGKTTLMDVLSGRKTGGYIDGRISISGYAKNQQTFARISGYCEQTDIHSPHVTVYESLVYSAWL
RLSPDVDSETRKMFIEEVMELVELNPLREALVGLPGVDGLSTEQRKRLTIAVELVANPSIIFMDEPTSGLDARAA
AIVMRAVRNTVDTGRTVVCTIHQPSIDIFDAFDELFLLKRGGEEIYVGPVGRHACHLIKYFEEIEGVPKIKDGYN
PATWMLEVTSAAQEAVLNDNFTDIFKNSELYRRNKALIKELSAPPPGSKDLYFPTRYSQSFFTQCMACLWKQH
WSYWRNPPYNAVRLLSTTVIALMFGTIFWNLGSKRNRKQDIFNSMGSMYAAVLFIGVQNATSVQPVVAIERTV
FYRERVAGMYSALPYAFAQVMIEIPYTLVQALIYGVIVYSMIGFEWTAIKFFWYIFFMYFTLLYMTFYGMMNV
AITPNHSIASLVSSAFYAIWNLFSGFIIPRTRVPIWWRWYCWACPFSWTLYGLIASQYGDLEDKLESDETVKDFL
RNYFGFRHDFVGICAIVVVGMSVLFAFTFAFSIRTFNFQRR

>XP_024460925.1_PDR1_Pt
MESADIYRASSSLRDSFRAGSSAWRNTTVEAFSRSSREEDDEEALKWAAIEKLPTYDRLRKGILTSASKGVANE
VDIEKLGLQERKQLLERLVKVAEEDNEKFLWKLKDRVERVGIDVPTIEVRYDNLNIEAEAYVGSSALPSFAKFT
FNIIEGLLISLNILRNRKKPLTILKDVSGIVKPSRLTLLLGPPSSGKTTLLLALAGKLDPNLKFSGRVTYNGHEMN
EFVPQRTAAYISQHDVHIGEMTVRETLAFSARCQGAGYLHDMLAELSRREKEANIKPDPDVDVFMKAVASQG
DEANVITDYVLKILGLEICADTMVGDEMIRGISGGQRKRVTTGEMLVGPSRALFMDEISTGLDSSTTYQIVNSL
RHTVHILNCTAVISLLQPAPETYDLFDDIILLSDGQIVYQGPRERVLEFFEHMGFKCPERKGVADFLQEVTSRKD
QEQYWARKDQPYRFITANEFAEAFQSFTVGRRTAEELSIPFDKSKNHPAALVTKTHGAGKKDLLKANFSREYL
LMKRNSFVYIFKICQLTIMALISMTLFFRTKMHRDTVRHGGIYTGALFFTAIMIMFNGMSELSMTIAKLPVFYKQ
RDLRFFPSWAYAFPQWILKIPVSFVEVAAWVFLTYYVIGFDPNVERLFKQYLVLLLINQTASALFRFIAAAGRN
MIIANTFGSFALLTLFTLGGFILSREKIKKWWIWGYWSSPLMYGQTAILVNEFLGNSWSHVPENSTEPLGIQVLK
SRGFFTEAYWYWIGAGATIGFILLLNLFFVLALTFLNAFDKPQAFISEEPESDESGRKTERAIQLSNHGSSHGTNT
EGGVGISRASSEVIGGVSNNRKKGMVLPFEPHSITFDDIIYSVDMPQEMKVQGVVQDRLVLLNGVNGAFRPGV
LTALMGVSGAGKTTLMDVLAGRKTGGYIDGEIKISGYPKKQETFARVSGYCEQNDIHSPQVTVYESLLYSAWL
RLPPEVDSETRRMFIEEVMDLVELNPLRHALIGLPGVNGLSTEQRKRLTIAVELVANPSIIFMDEPTSGLDARAA
AIVMRTVRNTVDTGRTVVCTIHQPSIDIFEAFDELFLMKRGGQEIYVGPLGRHSTHLIKYFEAIEGVSKIKDGYN
PATWMLEISSSAQEMALEVDFSNIYKNSDLFRRNKALIVELSTPAPGSTDLYFPTKYSTSFLTQCMACLWKQH

WSYWRNPPYTAVRFIFTTFIALMFGTMFWDLGSKVSSTQDLSNAMGSMYAAVLFLGVQNASSVQPVVAVERT
VFYRERAAGMYSALPYAFAQVLIELPYIFAQAAVYGIIVYAMIGFDWTVAKFFWYLFFMYFTLLYFTYYGMM
AVAVTPNHHIASIVSSAFYGIWNLFSGFIVPRPSIPIWWRWYSWACPVAWTLYGLVVSQFGDIQKKLTETQTVK
EYVKDYFGFHHDFLGVVAAAIVGWTVLFAFIFAFAIKAFNFQRR

>XP_024460929.1_PDR1_X1_Pt
MESADIYRASSSLRDSLRAGSFVWRNSTVEAFSRSSREEDDEEALKWAALEKLPTYDRLRKGILMSASRGVSSE
VDIEKLGVQERKQLLERLVKAADEDNEKFLWKLKNRIERVGIEFPTIEVRYEHLNIGAEAYVGSGALPSFAKFT
FSIIEDLLIALRIIPSRKKPLTILKDVSGIIKPSRLTLLLGPPSSGKTTLLLALAGKLDPSLKYSGRVTYNGHGMNEF
VPQRTASYTSQQDLHIGEMTVRETLAFSARCQGVGNLHDMLAELSRREKEANIKPDPDIDVFLKAVATQGQEA
NVITDYVLKILGLEVCADTLVGDEMIRGISGGQRKRVTTGEMLVGPSRALFMDEISTGLDSSTTHQIVNSLKQTI
HILNYTAVISLLQPAPETYDLFDDIILLSDGQIVYQGPRENVLGFFEHLGFQCPERKGVADFLQEVTSRKDQEKY
WARKDQPYRFVTVNEFAEAFQSLSVGRRVIEELSIPFDKTKNHPAALVNKKYGAGKMDLLKANFSREFLLMK
RNSFVYIFKIFQLTMMAIISMTLFFRTKMPRDTVEDGGIYAGALFFTAIMIMFNGMAELSMTIAKLPVFYKQREL
LFFPPWTYSIPPWILKIPITFVEVAAWVFLTYYVIGFDPNIGRFFKLYAVLVLINQMASALFRFIAAAGRNMIVAN
TFGSFVLLAVFALGGVILSREQIKKWWIWGYWASPLMYGQNAIVVNEFLGNSWSHIPAGSTESLGIQVLKSREF
FTEAYWYWIGIGATAGFILLFNVCFVVALTVLDAYEKPQAVISEEPESGDSEGAVQLSNRGISHQTNTDSIGEAS
NNRKKGMVLPFEPHSITFDDVIYSVDMPQEMKVQGVAEDRLALLKGVSGAFRPGILTALMGVSGAGKTTLMD
VLAGRKTGGYIEGDIKISGYPKKQETFARISGYCEQNDIHSPQVTVYESLLYSAWLRLPSEVDSETRKMFIEEVM
DLVELNPLRSALVGLPGVNGLSTEQRKRLTIAVELVANPSIIFMDEPTSGLDARAAAIVMRTVRNTVDTGRTVV
CTIHQPSIDIFDAFDELFLMKRGGEEIYVGPVGRHSTHLIKYFEEIEGVSKIKDGYNPATWMLEVTSSSQEMALG
VDFANIYKNSNLLRRNKALIAELSTPAPGSKDIYFSTQYSTSFFTQCMACLWKQHWSYWRNPPYTAVRFLFTTF
IGLMFGTMFWDLGSKVGTAQDLSNAMGSMYAAVLFLGFQNGSAVQPVVAVERTVFYRERAAGMYSALPYA
FAQVLIEIPYVFVQSAVYGVIVYAMIGFEWTAAKFFWYLFFMYFTLLYFTFYGMMSVAVTPNHHIAAIVSTAF
YLIWNLFSGYIVPRPRIPIWWRWYYWACPVSWSLYGLVVSQYGDIQKNLTETETVKQYVKNYFGFDHDFVGV
VAAAVLGWTVLFAFIFAFSIRAFNFQRR

>XP_024953227.1_PDR1-l_X2_Csi
MQMDAGQASFRISSARLGSSSIWRNNTLDVFARSSREDTYDDDEALTWAAIEKLPTYLRVQRGMLTEDEGQA
REVDIKNLGFIERRNLIERLLKIAEEDNEKFLLKLKDRIERVGLDIPTIEVRFEHLNVEAEAYIGSRALPTVFNSCA
NMLEGFLNYLHVLPSRKKPLTILHDVSGIIKPQRLTLLLGPPSSGKTTLLLALAGKLGKDLKFSGRVTYNGHGM
EEFVPQRTSAYISQNDLHIGEMTVRETLAFSARCQGVGPRYEVLQELSRREKAANIKPDPDIDLIMKAASLEGQ
EKNVVTDYVLKILGLEVCADTMVGDEMLRGISGGQRKRLTTGEMLVGPARALFMDEISTGLDSSTTYQIVNSL
RQSIHILNGTAVISLLQPAPETYELFDDLILLSDGQIVYQGPRENVLEFFERMGFKCPERKGVADFLQEVTSRKD
QEQYWANKDEPYSFVTAKEFSEVFQSFHIGQKLGDELATPFDKSKSHPAALTTKKYGASKKELLKACFAREYL
LMKRNSFVYFFKMFQIFFSASVAMTLFLRTEMHRSTVEDGGIYMGALFFAVITIMFNGFSELSMTIMKLPVFYK
QRDFLFFPAWAYSLPTWILKIPITFIEVGIWVFMTYYVVGFESNIERFVKQYFLLLCVNQTASGLFRLMGALGRN
IIVANTFGSFANLTVLVLGGFILSRDDVKKWWLWGYWFSPMMYGQNALAVNEFLGKSWGHVPPNSTEPLGV
VILKSRGLFPNAYWYWIGVGALLGYVLLFNFLFTVALKYLDPFGKPQAILSEEALAKKNACKTEEPVELSSGV
QSSYDRGNESHRNVSFRTLSARVRSFNEADQNRKRGMILPFEPHSITFDDIRYALDMPQEMKAQGIPDDRLEFL
KGVSGAFRPGVLTALMGVSGAGKTTLMDVLAGRKTGGYVSGSITISGYPKNQETFARISGYCEQTDIHSPHVT
VYESLVYSAWLRLPPEVDSDTRKMFVEEVMELVELNPIREALVGLPGVSGLSTEQRKRLTIAVELVANPSIIFM
DEPTSGLDARAAAIVMRTVRNTVDTGRTVVCTIHQPSIDIFDAFDELLLMKRGGEEIYVGPLGRHCSQLIKYFE
GIDGVPKIKEGYNPATWMLEVTTPAQEAALGINFAKVYKNSELYKGNKEMIKELSIPPPGSKNLYFQTRYSQSF
FTQCMACLWKQHLSYWRNPPYTAVRLFFTTFIALMFGTIFWDIGSKRANRQDLFNAMGSMYAAILFLGVQNA
TSVQPVVAVERTVFYRERAAGMYSALPYAFGQVVIELPHIFIQAVIYGVIVYAMIGFDWTVSKFLWYLLFMYL
TFLYFTLYGMMTVAVTPNHNIAAIIASAFYVLWNLFSGFIIPRPFNQRGLCFFCNRGCQFGGDGTVGYAPSRGH
CMDLLLPNLEM

>XP_024964757.1_PDR1-l_X1_Cca
MMLEGEEIFRVSSARISSSNIWRNSGRDFFSRSSFEEEDDEEALKWAAIQKLPTNLRLERGILTEEKGLLTEINIK
NLGLSEKKNLLDRLVRIAEEDNEKFLLKLKERIDRVGLQLPTIEVRFEHLNVDAEAYVGGRALPTIVNFLVNILQ
GSLNFLHLLPSRKKTFPILHDVSGIVKPGRMTLLLGPPSSGKTTLLLALAGKLGSDLQVSGRVTYNGHEMGEFV

PQRTSAYISQYDLHIGEMTVRETLAFSARCQGVGANYEMLAELSRREKEANIKPDPDLDIYMKAASLEGQEAS
VVTEYVMKILGLEICADIVVGDEMFRGISGGQKKRVTTGEMMVGPARALFMDEISTGLDSSTTFQIVNSIRQSIH
ILQGTSMISLLQPAPETYDLFDDIILLSDGQVVYQGPRENVLEFFEHMGFKCPERKAVADFLQEVTSKKDQEQY
WARISEPYSFVTVQEFTEAFQLFHVGRRLGDDLAAPFDRTKSHPAALTTKKYGVTRKELLKACVSREYLLMKR
NSFVYIFKMIQLILLATTSMTLFLRTEMPKKTIVDGTVYMGALFFTILTMTFNGFSELALSIMKLPVFYKQRDLL
FFPPWVYSLPTWILKIPVTVVEAATWVIMTYYVVGYDPDVGRFFRQLLVLICIQQMASAVFRFIAALGRNIIVA
NTIGSFSFLAILVLGGFILSRDDIKKWWAWGYWLSPLMYGQNGMAVNEFLGKSWRQVLPNSTESLGVAVLKS
RGLFPEAKWYWISVGALVGYVFLFNIFFILALTYLNPLGKPQAIQSEETVAARNALKKGDPIELSVRASSEMKN
EVRTSRSMSSRVGSIIEAHKKKKHGMVLPFVPLSLTFDEIRYAVDMPQEMKAEGITQQQLELLKGVGGCFRPG
VLTALMGISGAGKTTLMDVLAGRKTGGYTKGRITISGYPKKQETFARIAGYCEQTDIHSPHVTVSESLQYSAW
LRLPAEVDSATREMFIEEVMELVELTSLSEALVGLPGVNGLSTEQRKRLTIAVELVANPSIIFMDEPTSGLDARA
AAIVMRTVRNTVDTGRTVVCTIHQPNIDIFDAFDELFLLKRGGEQIYFGPLGRHCSQLIKYFEDIDGVNKIKDGY
NPATWMLEVTSEAQEAALGVNFGDVYRNSELYKRNKELIKENSQPIPGSSDIHFRTQYSQSFWTQCMACLWK
QHWSYWRNPPYSAVRFLFTTFIALMFGTIFWDMGSKRDKQQDLFNAMGSMYAAVTFIGVQNAASVQPVVSIE
RTVFYRERAAGMYSAFPYAFGQIMIEVPYIFVQTIVYGIIVYAMIGFEWTLVKFFWYLFFMYFTLLYFTFYGMM
TVAVTPNHNIAAIISSAFYALWNLFSGFIVPRTRIPIWWRWYYYICPVAWTLYGLVASQFGDMDDELESGQTVS
EFVSSYFGFEHDFIGYVALIVVGFAVLFGFIFAYSIRAFNFQTR

>XP_024964758.1_PDR1-l_X2_Cca
MMLEGEEIFRVSSARISSSNIWRNSGRDFFSRSSFEEEDDEEALKWAAIQKLPTNLRLERGILTEEKGLLTEINIK
NLGLSEKKNLLDRLVRIAEEDNEKFLLKLKERIDRVGLQLPTIEVRFEHLNVDAEAYVGGRALPTIVNFLVNILQ
GSLNFLHLLPSRKKTFPILHDVSGIVKPGRMTLLLGPPSSGKTTLLLALAGKLGSDLQVSGRVTYNGHEMGEFV
PQRTSAYISQYDLHIGEMTVRETLAFSARCQGVGANYEMLAELSRREKEANIKPDPDLDIYMKAASLEGQEAS
VVTEYVMKILGLEICADIVVGDEMFRGISGGQKKRVTTGEMMVGPARALFMDEISTGLDSSTTFQIVNSIRQSIH
ILQGTSMISLLQPAPETYDLFDDIILLSDGQVVYQGPRENVLEFFEHMGFKCPERKAVADFLQEVTSKKDQEQY
WARISEPYSFVTVQEFTEAFQLFHVGRRLGDDLAAPFDRTKSHPAALTTKKYGVTRKELLKACVSREYLLMKR
NSFVYIFKMIQLILLATTSMTLFLRTEMPKKTIVDGTVYMGALFFTILTMTFNGFSELALSIMKLPVFYKQRDLL
FFPPWVYSLPTWILKIPVTVVEAATWVIMTYYVVGYDPDVGRFFRQLLVLICIQQMASAVFRFIAALGRNIIVA
NTIGSFSFLAILVLGGFILSRDDIKKWWAWGYWLSPLMYGQNGMAVNEFLGKSWRQVLPNSTESLGVAVLKS
RGLFPEAKWYWISVGALVGYVFLFNIFFILALTYLNPLGKPQAIQSEETVAARNALKKGDPIELSVRASSGGSSR
TNVHYLSMSSRVGSIIEAHKKKKHGMVLPFVPLSLTFDEIRYAVDMPQEMKAEGITQQQLELLKGVGGCFRPG
VLTALMGISGAGKTTLMDVLAGRKTGGYTKGRITISGYPKKQETFARIAGYCEQTDIHSPHVTVSESLQYSAW
LRLPAEVDSATREMFIEEVMELVELTSLSEALVGLPGVNGLSTEQRKRLTIAVELVANPSIIFMDEPTSGLDARA
AAIVMRTVRNTVDTGRTVVCTIHQPNIDIFDAFDELFLLKRGGEQIYFGPLGRHCSQLIKYFEDIDGVNKIKDGY
NPATWMLEVTSEAQEAALGVNFGDVYRNSELYKRNKELIKENSQPIPGSSDIHFRTQYSQSFWTQCMACLWK
QHWSYWRNPPYSAVRFLFTTFIALMFGTIFWDMGSKRDKQQDLFNAMGSMYAAVTFIGVQNAASVQPVVSIE
RTVFYRERAAGMYSAFPYAFGQIMIEVPYIFVQTIVYGIIVYAMIGFEWTLVKFFWYLFFMYFTLLYFTFYGMM
TVAVTPNHNIAAIISSAFYALWNLFSGFIVPRTRIPIWWRWYYYICPVAWTLYGLVASQFGDMDDELESGQTVS
EFVSSYFGFEHDFIGYVALIVVGFAVLFGFIFAYSIRAFNFQTR

>XP_024986321.1_PDR1-l_X1_Cca
MEGGDVFRVSSARISSSNIWRTSGRDIFSRSSVEEDDEEALKWAAIEKLPTNLRLERGILTEEKGQPREIDIKNLG
LVEKRNLLERLVKIAEEDNEKFLLKLKDRIDRVGIQLPTIEVRFEHLNIDAEAYVGGRALPTVFNFIVNIMEGSL
NYMHLLPSRKIPLPILHDVSGIIKPGRMTLLLGPPSSGKTTLLLALAGKLGSDLKVSGRVTYNGHEMHEFVPQRS
SAYISQHDLHIGEMTVRETLAFSARCQGVGASYEMLAELSRREKEANIKPDPDLDIYMKAASLEGQEASVVTE
YVIKILGLEVCADTIVGDEMFRGISGGQKKRVTTGEMLVGPARALFMDEISTGLDSSTTFQIVNSIRQSIHILQGT
SIISLLQPAPETYDLFDDIILLSDGQIVYQGPRENVLEFFEYMGFKCPERKGVADFLQEVTSKKDQEQYWIRRED
PYSFITSKEFAEAFQSFHVGRRLGDELSTPFDRTKSHPAALTTKKFGVNKKELLKACISREYLLMKRNSFVYIFK
MTQLIFMATITMTLFLRTEMPKNTLIDGTIFMGALFFTLVMITFNGFSELALSIIKLPVFYKQRDLLFFPAWAYSI
PTWILKIPVTIVEVATWVIMTYYVIGFDPDAGRFFKQLMLLICIHQMSSALFRFIGALGRNIIVANTFGSFGLLTIF
VLGGFILARDDIQKWWIWCYWFSPMMYGQNGMAVNEFLGKSWNKVLPNSTETIGVSVLKSRGIFPEAKWYW
IAVAASVGYIFLFNICFTLALEYLNPFGNPQAVLSEESLAERTAMKTEPMHALITESVVTLSILKNAERKSEVRR
NASSMSMSSRVGSINGTDQTKKRGMVLPFVPLSITFDDIKYAVDMPQEMKAQGVSRDRLELLKGVSGAFRPG
VLTALMGISGAGKTTLMDVLAGRKTGGYIDGRISISGYPKKQETFARIAGYCEQTDIHSPHVTIYESLQYSAWL

RLPPEVDSATREMFVEEVMELVELIPLREALVGLPGVNGLSTEQRKRLTIAVELVANPSIIFMDEPTSGLDARAA
AIVMRTVRNTVDTGRTVVCTIHQPSIDIFDAFDELFLLKRGGEEIYVGPLGRHSSQLITYFEEIEGVNKIKDGYNP
ATWMLEVTSAAHEATLGVNFAEVYKNSELYKRNKETIKESSRPIPGSADIQFRTQYSQSFWTQCMACLWKQH
WSYWRNPPYTAVRFLFTTFIALMFGTIFWDMGSKREKQQDLFNAMGSMYAAILFIGVQNATSVQPVVSIERTV
FYRERAAGMYSALPYAFGQVMIEIPYIFVQTIVYGIIVYAMIGFEWTAVKFFWYLFFMYFTFLYFTFYGMMTV
AVTPNHNFAAIISSAFYAIWNLFSGFIVPRTRMPIWWRWYYYICPIAWTLYGLVASQFGDITDKLDTGETVAEF
VHSFYGFEYDFIRYVAVIIVGFTVLFGFIFAYSIKAFNFQKR

>XP_024986322.1_PDR1-l_X2_Cca
MEGGDVFRVSSARISSSNIWRTSGRDIFSRSSVEEDDEEALKWAAIEKLPTNLRLERGILTEEKGQPREIDIKNLG
LVEKRNLLERLVKIAEEDNEKFLLKLKDRIDRVGIQLPTIEVRFEHLNIDAEAYVGGRALPTVFNFIVNIMEGSL
NYMHLLPSRKIPLPILHDVSGIIKPGRMTLLLGPPSSGKTTLLLALAGKLGSDLKVSGRVTYNGHEMHEFVPQRS
SAYISQHDLHIGEMTVRETLAFSARCQGVGASYEMLAELSRREKEANIKPDPDLDIYMKAASLEGQEASVVTE
YVIKILGLEVCADTIVGDEMFRGISGGQKKRVTTGEMLVGPARALFMDEISTGLDSSTTFQIVNSIRQSIHILQGT
SIISLLQPAPETYDLFDDIILLSDGQIVYQGPRENVLEFFEYMGFKCPERKGVADFLQEVTSKKDQEQYWIRRED
PYSFITSKEFAEAFQSFHVGRRLGDELSTPFDRTKSHPAALTTKKFGVNKKELLKACISREYLLMKRNSFVYIFK
MTQLIFMATITMTLFLRTEMPKNTLIDGTIFMGALFFTLVMITFNGFSELALSIIKLPVFYKQRDLLFFPAWAYSI
PTWILKIPVTIVEVATWVIMTYYVIGFDPDAGRFFKQLMLLICIHQMSSALFRFIGALGRNIIVANTFGSFGLLTIF
VLGGFILARDDIQKWWIWCYWFSPMMYGQNGMAVNEFLGKSWNKVLPNSTETIGVSVLKSRGIFPEAKWYW
IAVAASVGYIFLFNICFTLALEYLNPFGNPQAVLSEESLAERTAMKTGKELLVRAPSVTLSILKNAERKSEVRRN
ASSMSMSSRVGSINGTDQTKKRGMVLPFVPLSITFDDIKYAVDMPQEMKAQGVSRDRLELLKGVSGAFRPGVL
TALMGISGAGKTTLMDVLAGRKTGGYIDGRISISGYPKKQETFARIAGYCEQTDIHSPHVTIYESLQYSAWLRLP
PEVDSATREMFVEEVMELVELIPLREALVGLPGVNGLSTEQRKRLTIAVELVANPSIIFMDEPTSGLDARAAAIV
MRTVRNTVDTGRTVVCTIHQPSIDIFDAFDELFLLKRGGEEIYVGPLGRHSSQLITYFEEIEGVNKIKDGYNPAT
WMLEVTSAAHEATLGVNFAEVYKNSELYKRNKETIKESSRPIPGSADIQFRTQYSQSFWTQCMACLWKQHWS
YWRNPPYTAVRFLFTTFIALMFGTIFWDMGSKREKQQDLFNAMGSMYAAILFIGVQNATSVQPVVSIERTVFY
RERAAGMYSALPYAFGQVMIEIPYIFVQTIVYGIIVYAMIGFEWTAVKFFWYLFFMYFTFLYFTFYGMMTVAV
TPNHNFAAIISSAFYAIWNLFSGFIVPRTRMPIWWRWYYYICPIAWTLYGLVASQFGDITDKLDTGETVAEFVHS
FYGFEYDFIRYVAVIIVGFTVLFGFIFAYSIKAFNFQKR

>XP_025012266.1_PDR1_X1_Rco
MENADTPRVGSARLSSSDIWRNTTLEIFSKSSRDEDDEEALKWAALEKLPTYLRIRRGILIEQGGQSREIDINSLG
LIEKRNLLERLVKIAEEDNEKFLLKLKDRIDKVGLDMPTIEVRFEHLSVEAEAYVGSRALPTMFNFSVNMFEAF
LNYLHILPSRKKPLSILNDVSGIIKPRRMTLLLGPPSSGKTTLLLALAGKLTKDLKFSGRVTYNGHGMEEFVPQR
TSAYISQYDIHIGEMTVRETLAFSARCQGVGSRYEMLMELARREKEANIKPDPDIDIYMKAAALEGQEANVVT
DYILKILGLELCADTLVGDEMARGISGGQKKRVTTGEMLVGPAKALFMDEISTGLDSTTTFQIVNSLRQSVHILS
GTALIALLQPAPETFELFDDIILLSDGQIVYQGPRENVLDFFEYMGFKCPERKGVADFLQEVTSRKDQEQYWAH
KDQPYLFVSVNEFSEAFQSFHIGRKLGDELATPFDKSKAHPDSLTTKKYGVSKKELFKACISREYLLMKRNSFV
YIFKMTQLIILGFITMTLFLRTEMHRNTETDGGVYLGALFFTVTTIMFNGFSELAMTILKLPVFYKQRDLLFYPS
WAYALPTWILKIPITFVEVAVWVVMTYYVIGFDPNIQRFFKQYLILLITNQMASALFRLTAALGRNIIVANTVG
AFAMLTALVLGGFVISRDNVKKWWIWGYWFSPMMYVQNAISVNEFLGSSWNHFPPNSTKPLGVTLLKSRGLF
PEAYWYWIGFGALTGYIFLFNFLFTLALKYLDPFGKPQAIISKEAYSEKTAVRTGEFIELSSKEKNFQGGNNANH
YKHNAERGSASHRVASSRTSSARVSSLSNAFENSKRGMVLPFQPLSITFADVRYAVQMPQEMKTQGITEDRLE
LLKGVSGAFRPGVLTALMGVSGAGKTTLMDVLAGRKTGGYIEGNITISGYPKKQETFARISGYCEQTDIHSPHV
TVYESLLYSAWLRLPPEVDSDTRNMFVEEVMELVELTSLREALVGLPGVNGLSVEQRKRLTVAVELVANPSIIF
MDEPTSGLDARAAAIVMRTVRNTVDTGRTVVCTIHQPSIDIFDAFDELLLLKRGGEEIYVGPVGRHACHLIKYF
EDIEGIPKIKDGYNPATWMLEVTTTAQEVALGVDFSDIYKNSELYRKNKALIKELSRPLPGSKDLYFPTQYSKSF
TTQCMACLWKQHWSYWRNPPYTAVRLVFATFIALMFGTIFWKLGTKRSRRQDIFNAMGSMYAAVLFLGFHN
STAVQPVVAIERTVFYRERAAGMYSALAYAFGQVMIEVPYILIQTIIYGVIVYAMVGFEWTISKFFWYLFFMYF
TLLYFTFYGMMNVAITPNHNIAAIVSSAFYAIWNIFSGFIVPRTRIPIWWRWYYWACPIAWTLYGLVASQFGDI
KEELDTGETVEHFLRSYFGFQHDFVGIVAVVLVGICVLFGFLFAFSIRTFNFQRR

>XP_025012267.1_PDR1_X2_Rco
MENADTPRVGSARLSSSDIWRNTTLEIFSKSSRDEDDEEALKWAALEKLPTYLRIRRGILIEQGGQSREIDINSLG

LIEKRNLLERLVKIAEEDNEKFLLKLKDRIDKVGLDMPTIEVRFEHLSVEAEAYVGSRALPTMFNFSVNMFEAF
LNYLHILPSRKKPLSILNDVSGIIKPRRMTLLLGPPSSGKTTLLLALAGKLTKDLKFSGRVTYNGHGMEEFVPQR
TSAYISQYDIHIGEMTVRETLAFSARCQGVGSRYEMLMELARREKEANIKPDPDIDIYMKAAALEGQEANVVT
DYILKILGLELCADTLVGDEMARGISGGQKKRVTTGEMLVGPAKALFMDEISTGLDSTTTFQIVNSLRQSVHILS
GTALIALLQPAPETFELFDDIILLSDGQIVYQGPRENVLDFFEYMGFKCPERKGVADFLQEVTSRKDQEQYWAH
KDQPYLFVSVNEFSEAFQSFHIGRKLGDELATPFDKSKAHPDSLTTKKYGVSKKELFKACISREYLLMKRNSFV
YIFKMTQLIILGFITMTLFLRTEMHRNTETDGGVYLGALFFTVTTIMFNGFSELAMTILKLPVFYKQRDLLFYPS
WAYALPTWILKIPITFVEVAVWVVMTYYVIGFDPNIQRFFKQYLILLITNQMASALFRLTAALGRNIIVANTVG
AFAMLTALVLGGFVISRDNVKKWWIWGYWFSPMMYVQNAISVNEFLGSSWNHFPPNSTKPLGVTLLKSRGLF
PEAYWYWIGFGALTGYIFLFNFLFTLALKYLDPFGKPQAIISKEAYSEKTAVRTGEFIELSSKENESSNNANHYK
HNAERGSASHRVASSRTSSARVSSLSNAFENSKRGMVLPFQPLSITFADVRYAVQMPQEMKTQGITEDRLELLK
GVSGAFRPGVLTALMGVSGAGKTTLMDVLAGRKTGGYIEGNITISGYPKKQETFARISGYCEQTDIHSPHVTVY
ESLLYSAWLRLPPEVDSDTRNMFVEEVMELVELTSLREALVGLPGVNGLSVEQRKRLTVAVELVANPSIIFMD
EPTSGLDARAAAIVMRTVRNTVDTGRTVVCTIHQPSIDIFDAFDELLLLKRGGEEIYVGPVGRHACHLIKYFEDI
EGIPKIKDGYNPATWMLEVTTTAQEVALGVDFSDIYKNSELYRKNKALIKELSRPLPGSKDLYFPTQYSKSFTT
QCMACLWKQHWSYWRNPPYTAVRLVFATFIALMFGTIFWKLGTKRSRRQDIFNAMGSMYAAVLFLGFHNST
AVQPVVAIERTVFYRERAAGMYSALAYAFGQVMIEVPYILIQTIIYGVIVYAMVGFEWTISKFFWYLFFMYFTL
LYFTFYGMMNVAITPNHNIAAIVSSAFYAIWNIFSGFIVPRTRIPIWWRWYYWACPIAWTLYGLVASQFGDIKE
ELDTGETVEHFLRSYFGFQHDFVGIVAVVLVGICVLFGFLFAFSIRTFNFQRR

>XP_025615641.1_PDR1-l_Ai
MENDELRVASARIGSSSIWRGSGAVDVFSGSSRRDDDEEQLKWAAIEKLPTYLRLTRGILTESQGEYTEIDINKL
GPLQRKNLVERLVKIAEQDNEKFLLKLRRRIDRVGLDIPTIEVRFEHLNVEAEAHVGSRALPTNLNFCINLLEGF
LNSLCLLPSRKKPFTVLHDVSGIIKPRRMTLLLGPPSSGKTTLLLALAGRLGKDLKFSGRVSYNGHGMEEFVPQ
RTSAYISQTDLHIGELTVRETLAFSARCQGIGMRYDMLAELSRREKAENIKPDPDLDIYMKAAALEGQETNVVT
DYIMKILGLEVCADTMVGDEMIRGISGGQKKRVTTGEMLVGPARALFMDEISTGLDTSTTFQMVNSLRQSIHIL
NGTAVISLLQPAPETYELFDDVILLSDGQIVYQGPRENVLEFFEYMGFKCPERKGVADFLQEVTSRKDQEQYW
ANKDEPYTFISVREFAEAFQSFHIGRKLGDELATPFDKTKGHPAVLTKNKYGVSKKELLRACVSREFLLMKRNS
FVYIFKMWQLILTGLITMTLFLRTEMHRNTVSDGGIYMGALFFVLIVIMFNGFSELSMTIAKLPVFYKQRDLLFY
PSWAYSLPTWILKIPITLVEVGIWVVMTYYVIGFDPSFERFIKQYFLLACINQMASALFRFMGAVGRNLIVANTF
GSFALLAVMVMGGFILSRVDVRKWWLWGYWISPMMYGQNAIAVNEFLGNSWKHVPPNSTEPLGVKVLKAR
GIFPEAHWYWIGVGASIGYMLLFNLLFPLALHFLDPFGKPQALISEEALAERNSVRNDHIIELSSGRNGASDKSN
GRSVSSRTLSARVGAFNGTDRNRKRGMVLPFTPLSITFDEIRYTLDMPQEMKAQGIVEDKLELLKGISGAFRPG
VLTALMGVSGAGKTTLMDVLSGRKTAGYIQGQITISGYPKKQETFARISGYCEQTDIHSPHVTVYESLVYSAW
LRLPPEVDSSTREMFIEEVMELVELNSLKDALVGLPGVNGLSTEQRKRLTIAVELVANPSIIFMDEPTSGLDARA
AAIVMRTVRNTVNTGRTVVCTIHQPSIDIFDAFDELLLLKRGGEEIYVGPLGRHSSHLISYFEGINGVPKIKNGYN
PATWMLEVTSEAQEEALGINFAELYKNSDLYRGNKALISELSTPPSASKDLYFTTKYSQSFITQCKACLWKQNL
SYWRNPPYSAVRFLFTTFIALLFGTIFWDIGSKRKRAQDVFNAMGSMYAAVLFIGVQNATSVQPVVAIERTVFY
REKAAGMYSALPYAFGQVAIEIPYILLQTLVYGVIVYAMIGFEWTVMKFFWYLFFMFFTFLYFTLYGMMAVG
ATPDHHVAGIVSFGFYLIWNLFSGFVIPRTRMPVWWRWYFWICPVSWTMYGLVTSQFGDVTERIDTGETVGD
FVKRYFGYRDDFIGIAAAVVVGFALLFGFTFAFSIKAFNFQKR

>AtABCG40
MEGTSFHQASNSMRRNSSVWKKDSGREIFSRSSREEDDEEALRWAALEKLPTFDRLRKGI
LTASHAGGPINEIDIQKLGFQDTKKLLERLIKVGDDEHEKLLWKLKKRIDRVGIDLPTIE
VRFDHLKVEAEVHVGGRALPTFVNFISNFADKFLNTLHLVPNRKKKFTILNDVSGIVKPG
RMALLLGPPSSGKTTLLLALAGKLDQELKQTGRVTYNGHGMNEFVPQRTAAYIGQNDVHI
GEMTVRETFAYAARFQGVGSRYDMLTELARREKEANIKPDPDIDIFMKAMSTAGEKTNVM
TDYILKILGLEVCADTMVGDDMLRGISGGQKKRVTTGEMLVGPSRALFMDEISTGLDSST
TYQIVNSLRNYVHIFNGTALISLLQPAPETFNLFDDIILIAEGEIIYEGPRDHVVEFFET
MGFKCPPRKGVADFLQEVTSKKDQMQYWARRDEPYRFIRVREFAEAFQSFHVGRRIGDEL
ALPFDKTKSHPAALTTKKYGVGIKELVKTSFSREYLLMKRNSFVYYFKFGQLLVMAFLTM
TLFFRTEMQKKTEVDGSLYTGALFFILMMLMFNGMSELSMTIAKLPVFYKQRDLLFYPAW

VYSLPPWLLKIPISFMEAALTTFITYYVIGFDPNVGRLFKQYILLVLMNQMASALFKMVA
ALGRNMIVANTFGAFAMLVFFALGGVVLSRDDIKKWWIWGYWISPIMYGQNAILANEFFG
HSWSRAVENSSETLGVTFLKSRGFLPHAYWYWIGTGALLGFVVLFNFGFTLALTFLNSLG
KPQAVIAEEPASDETELQSARSEGVVEAGANKKRGMVLPFEPHSITFDNVVYSVDMPQEM
IEQGTQEDRLVLLKGVNGAFRPGVLTALMGVSGAGKTTLMDVLAGRKTGGYIDGNITISG
YPKNQQTFARISGYCEQTDIHSPHVTVYESLVYSAWLRLPKEVDKNKRKIFIEEVMELVE
LTPLRQALVGLPGESGLSTEQRKRLTIAVELVANPSIIFMDEPTSGLDARAAAIVMRTVR
NTVDTGRTVVCTIHQPSIDIFEAFDELFLLKRGGEEIYVGPLGHESTHLINYFESIQGIN
KITEGYNPATWMLEVSTTSQEAALGVDFAQVYKNSELYKRNKELIKELSQPAPGSKDLYF
PTQYSQSFLTQCMASLWKQHWSYWRNPPYTAVRFLFTIGIALMFGTMFWDLGGKTKTRQD
LSNAMGSMYTAVLFLGLQNAASVQPVVNVERTVFYREQAAGMYSAMPYAFAQVFIEIPYV
LVQAIVYGLIVYAMIGFEWTAVKFFWYLFFMYGSFLTFTFYGMMAVAMTPNHHIASVVSS
AFYGIWNLFSGFLIPRPSMPVWWEWYYWLCPVAWTLYGLIASQFGDITEPMADSNMSVKQ
FIREFYGYREGFLGVVAAMNVIFPLLFAVIFAIGIKSFNFQKR

>MtPDR23
MEGGELRVASGRVGSSSIWRSGAVDVFSGSSRRDDDEQELQWAAIEKLPTYLRMTRGILN
ESQSEQPIEIDINKLGPLQRKNLVERLVKIAEEDNEKFLLKLRQRIDRVGLDFPTIEVRF
EHLNVEAEAHVGSRALPTILNFSINLLEGFLNNLHLIPSRKKPLTVLHDVSGIIKPKRMT
LLLGPPSSGKTTLLLALAGRLSRDLKFSGRVAYNDHGMEEFVPQRTSAYISQTDLHIGEL
TVRETLAFSARCQGIGTRYDMLAELSRREKAENIKPDPDLDIYMKAEALEGQETNIVTDY
IIKILGLDVCADTMVGDDMIRGISGGQKKRVTTGEMLVGPARALFMDEISTGLDSSTTFQ
MINSLRQSIHILNGTALISLLQPTPETYDLFDDIILLSDGQIVYQGPRENVLEFFEHVGF
KCPERKGVADFLQEVTSRKDQEQYWSNKDKPYTFITVREFAEEFQLFHVGQKLGDELGTP
FDASKGHPAVLTKNKYGVSRKELLKACVSRELLLMKRNSFVYIFKMWQLIFTGIVTMTMF
LRTEMHRNTETDGGIYMGALFFILIVIMFNGYSELSMFIMKLPVFYKQRDLLLFPAWAYS
LPTWILKIPITFVEVGIWVVLTYYVIGFDPCFERFIKQYFLLVCINQMASALFRFIGAVG
RNVIVANTVGSFALLAVLVMGGFILSRVDVKKWWLWGYWVSPMMYGQNAIAVNEFLGKSW
SHIPPDSTEPLGVQILKSRGIFPEAYWYWIGVGASIGYMLLFNFLFPLALHYLDSKYPIY
YMWLSAFGKPQALISEEALAERNAATAGSKQIIELSPKLECSSGNASRRSFSSTTLSTKV
GSINAADHTRKRGMVLPFTPLSITFDEIGYAVDMPQEMKAKGIPEDRLELLTGVNGAFRP
GVLTALMGISGAGKTTLMDVLSGRKTTGYVQGQITISGYPKKQETFSRISGYCEQTDIHS
PHVTVYESLVYSAWLRLPPEVDTSTRKMFIEEVMELIELTSIREALVGLPGVNGLSTEQR
KRLTIAVELVANPSIIFMDEPTSGLDARAAAIVMRTVRNTVDTGRTVVCTIHQPSIDIFD
AFDELLLLKRGGEEIYVGPLGRHCSHLINYFEGINGVPKIKNGYNPATWMLEVTSEAQEE
ALGINFAELYKNSDLYRTNKALIRELSTPPEGSKDLYFTTQHSQSFLTQCMACLWKQNLS
YWRNPPYSAVRLLFTTVIAFLFGTIFWNIGSKRERRQDLFNAMGSMYAAVLFIGVQNATS
VQPVVAIERTVFYREKAAGMYSALPYAFGQVAVEIPYILIQSLVYGVIVYTMVGFERTPT
KFFWYLFFMFFTFLYFTFFGMMLVGATPDHNVAAIVSFGFYLLWNLFSGFVIPRTRMPVW
WRWFFWICPISWTLYGLITTQFGDVNERMDTGETVEEFVRSYFGYRDDFKDVAAAVVVSF
SLIFGSAFAFSIKAFNFQKR

>PhPDR1
meggeelfrvssarlsssnvwrnsamdvfsrssreaddeealkwaaleklptylrirrgi
lteeegqsrevditkldlverrnllerlikitdednekfllklkeridrvgldlptievr
fehlsvdaearvgsralptvfnftvniledflnylhilpnrkqplpilhdvsgiikpgrm
tlllgppssgkttlllalagkldkdlkvsgrvtynghdmnefvaqrssayisqydlhige
mtvretlafsarcqgvgakyeilaelsrrekeanikpdpdvdifmkaawnegqeanvvtd
ytlkilgleicadtivgdemvrgisggqrkrlttgemmvgparalfmdeistgldsstty
qivnsirqsihilqgtavisllqpapetydlfddiillsdgqivyqgprenvleffeymg
ficperkgvadflqevtsrkdqeqywarreesykfitvrefseafqafhigrklgdelav
pfdkskshpaalttkrygvskkellkactareyllmkrnsfvyifkmiqltlmasitmtl
flptemhrnttidgavflgalfyalimimfngfselalsimklpsfykhrdllffppway
alptwilkipitlvevaiwvcmtyyvigfeadvgrffkqllllicvnqmasglfrlmgal

grniivantfgsfvlltvlvmggfvlsrddvkkwwiwgywispmmyaqnaiavneflgks
wahvppnststetlgvsflksrgifpdarwywigagaligyvflfnflfavalaylnpfg
kpqavlseetvaernaskrgevielsslgksssekgndvrrsassrsmssrvgsitaadl
skrrgmilpfeplsitfddiryavdmpqemkaqgftedrlellrgvsgafrpgvltalmg
vsgagkttlmdvlagrktggyidgtisisgypkqqetfariagyceqtdihsphvtvyes
lqfsawlrlprevdtatrkmfieevmelieliplrdalvglpgvnglsteqrkrltvave
lvanpsiifmdeptsgldaraaaivmrtvrntvdtgrtvvctihqpsidifdafdellll
krggeeiyvgplgrqsshlikyfegidgvpkikdgynpatwmleitsvaqegalgndfte
lyknselyrrnkalikelsvpascskdlyfptkysqsfftqcmacfwkqhwsywrnppyt
avrimftffialmfgtifwdlgsrrerqqdllnaigsmyiavlflgvqnattvqpviaie
rtvfyreraagmysampyafgqvmielpylflqtiiygvivyamigfewtvakffwylff
myftllyftlygmmtvavtpnqsiaaiissafyavwnlfcgfivpktrmpvwwrwyyyic
piswtlygliasqfgdiqdrldtnetveqfienffdfkhdfvgyvalilvgisvlflfif
afsiktfnfqkr

>NtPDR6
MEGGEDSFRVSSARLSSNVWRNSAMDVFSRREADDEEALKWAALEKLPTYLRRRGLTEEE
GSRVDTKLDLVERRNLLERLVKADEDNEKFLLKLKKRARVGLDLPTEVRFEHVSVDAEAR
GSRALPTFNFTVNLEDFLNYLHLPSRKKPLPLHEVSGKPGRMTLLLGPPSSGKTTLLLAL
AGKLDKDLKVSGRVTYNGHGMDEFVPRSSAYSNDLHGEMTVRETLAFSARCGVGAKYELA
ELSRREKGANKPDPDVDFMKAWNEGEANVVTDYTLKLGLECADTLVGDMRGSGRKRLTTG
EMMVGPARALFMDESTGLDSSTTYVNSRSHLGTAVSLLPAPETYDLFDDLLSDGVYGPRE
NVLEFFEYMGFMCPERKGVADFLEVTSRKDEYWARRDEPYKYTVREFSESFSFHGRKLGD
ELAVPFDKSKSHPAALTTKKYGSKKELLKACTAREYLLMKRNSFVYFKMLTLMASTMTLP
hcLRTEMHRNTTTDGAVFLGALFYAVMMFNGFSLALSMKLPSFYKRDLLFFPAWAYALPT
WLKPVTLVEVAWVCMTYYVGFEADVGRFFKLFLLCVNMASGLFRFGALGRNVVANTFGSC
ALLTVLVMGGFLSRDDVKKWWWGYWSPMMYANAAVNEFLGKSWAHVPPNSTGTETLGVSF
LKSRGFPEARWYWGAGALGYVLLFNFMFTVALAYLNPFGKSAVLSEETVAERNAKRGEVE
LSPEKRSSERGNDVRRSASSRSMSSRGSTEADLNKRRGMLPFEPLSTFDDRYAVDMPEMK
AGVAEDRLELLKGVSGAFRPGVLTALMGVSGAGKTTLMDVLAGRKTGGYDGTSSGYPKET
FARAGYCETDHSPHVTVYESLFSALLRLPREVDTETRKMFVEEVMELVELTPLREALVGL
PGVNGLSTERKRLTVAVELVANPSFMDEPTSGLDARAAAVMRTVRNTVDTGRTVVCTHPS
DFDAFDELLLLKRGGEEYGPLRHSSHLKYFEGDGVPKKDGYNPATWMLETSVAEAARVDF
TELYKNSELYRRNKALKELSVPPPCSKDLYFPTKYSSFFTCKACFWKHWSYWRNPPYTAV
RLMFTFFALMFGTFWDLGSRRKRDLLNAGSMYAAVLFLGVNATSVPVAERTERAAGMYSA
LPYAFGVMELPYLFTYGVVYVMGFEWTVAKFFWYLFFMYFTLLYFTLYGMMTVAVTPNHS
ASSFYAWNLFCGFVVPKTDVFYGSKSDGSNFVLSTVCARAVGVRDTWCWLGAGAWHWVCA
RGTVCAGSAVGNMMVCARSVGTRGTDGRPLGAETDYRWDDRGVCVLVTWRCDHGADRGSR
RWEACRDATGRVGVMGR

>AtABCC10
MIENYWTSFCGNHHTSSNCTVRFLQICFGITLSFLTLCICLFHKEPPKRIHQFFCLRLVSALFNGIIGSLDLVLGIW
VLRENHSKPLILWLVILIQGFTWLFINLIICVRGTRIRKSSLRLLSIFSFFYGLVSSCLSVNNAVFGDELAVRTILDV
LLLPGSVLLLLSAYKGYRFDESGESSLYEPLNAGDSNGFSEKADFDNRVSQFAKAGLFSTLSFWWLNSLIKRGN
VKDLEEEDIPELRKEERAETCYSLFEENLIEQKRRLGSSCQPSILKVTVLCVWRELLTSGFFAFMKIVAVSAGPL
LLNAFILVAEGNASFRYEGLVLAVLLFFSKMIESLSQRQWYFRCRIVGLRVRSLLTAAINKKQLRLNNSSRLIHS
GSEIMNYATVDAYRIGEFPYWFHQLWTTSFQLLIALGILFHSVGVATFSALAVIILTVLCNAPIAKLQNKFQSEL
MTSQDERLKACNESLVNMKVLKLYAWESHFKKVIEKLRNIELKSLKAVQMRKAYNAVLFWSSPVFVSAATFA
TCYFLDIPLRASNVFTFVATLRLVQDPVRMIPDVIGVTIQAKVAFSRIATFLEAPELQGGERRRKQRSEGNQNAII
IKSASFSWEEKGSTKPNLRNVSLEVKFGEKVAVCGEVGSGKSTLLAAILGETPCVSGTIDFYGTIAYVSQTAWIQ
TGTIRDNILFGGVMDEHRYRETIQKSSLDKDLELLPDGDQTEIGERGVNLSGGQKQRIQLARALYQDADIYLLD
DPFSAVDAHTASSLFQEYVMDALAGKAVLLVTHQVDFLPAFDSVLLMSDGEITEADTYQELLARSRDFQDLV
NAHRETAGSERVVAVENPTKPVKEINRVISSQSKVLKPSRLIKQEEREKGDTGLRPYIQYMNQNKGYIFFFIASL
AQVTFAVGQILQNSWMAANVDNPQVSTLKLILVYLLIGLCSVLCLMVRSVCVVIMCMKSSASLFSQLLNSLFR
APMSFYDSTPLGRILSRVSSDLSIVDLDVPFGLIFVVASSVNTGCSLGVLAIVTWQVLFVSVPMVYLAFRLQKY
YFQTAKELMRINGTTRSYVANHLAESVAGAITIRAFDEEERFFKKSLTLIDTNASPFFHSFAANEWLIQRLETVS
AIVLASTAFCMILLPTGTFSSGFIGMALSYGLSLNMGLVYSVQNQCYLANWIISVERLNQYTHLTPEAPEVIEET
RPPVNWPVTGRVEISDLQIRYRRESPLVLKGISCTFEGGHKIGIVGRTGSGKTTLISALFRLVEPVGGKIVVDGVD
ISKIGVHDLRSRFGIIPQDPTLFNGTVRFNLDPLCQHSDAEIWEVLGKCQLKEVVQEKENGLDSLVVEDGSNWS
MGQRQLFCLGRAVLRRSRVLVLDEATASIDNATDLILQKTIRREFADCTVITVAHRIPTVMDCTMVLSISDGRIV
EYDEPMKLMKDENSLFGKLVKEYWSHYNSADSR

>AtABCC11
MGFEALNWYCKPIAEGFWEKTPDGAFGAYTPCAIDSLVMIVSNSVLLGLCFYRIWITLYNAKAQIYVLRKMYY
HCVLWILACCCVVEPVLRLVMGISLFDMGDETDLPPFEVASLMVEAFAWFAMLVLIGLETKQYVKEFRWYVR
FGVVYVLVADAVLLDLVLPLKNSINRTALYLCISSRCCQALFGILLLVYIPELDLYPDYHILNNESLDNVEYDAL
PGGVNICPERYASIFSGIYFSWMTPLMQLGYRKPITERDVWQLDQWDQTETLIKRFQRCWTEESRRPKPWLLR
ALNNSLGRRFWLGGIFKVGHDLSQFVGPVILSHILQSMIEGDPAWVGYVYAFLIFFGVTFGVLCQSQYFQHVGR
VGFRLRSTLVAAIFHKSLRLTNKARKNFASGKVTNMITTDANALQLIAEQLHGLWSAPFRIIVSMVLLYQQLGV
ASIFGSLILFLLIPFQTLIVRKMRKLTKEGLQWTDKRVGIIYEILASMDIVKCYAWEKSFESRIQGIRNEELSWFR
KAQLLSAFNSFILNSTPVVVTLVSFGVYVLLGGDLTPARAFTSLSLFAVLRSPLSTLPNLISQAVNANVSLQRIEE
LLLSEERILAQNPPLQPGAPAISIKNGYFSWDSKTSKPTLSDINLEIPVGSLVAIVGGTGEGKTSLISAMLGELSHA
ETSSVDIRGSVAYVPQVSWIFNATLRENILFGSDFESERYWRAIDVTALQHDLDLFPGRDRTEIGERGVNISGGQ
KQRVSMARAVYSNSDIYIFDDPFSALDAHVAHQVFDSCVKHELKGKTRVLVTNQLHFLPLMDRIILVSEGMIK
EEGNFAELSKSGTLFKKLMENAGKMDATQEVNTNDENISKLGPTVTIDVSERSLGSIQQGKWGRSMLVKQEER
ETGIISWDVVMRYNKAVGGLWVVMILLVCYLTTEVLRVLSSTWLSIWTDQSTPKSYSPGFYIVVYALLGFGQV
AVTFTNSFWLISSSLHAAKRLHDAMLNSILRAPMLFFETNPTGRVINRFSKDIGDIDRNVANLMNMFMNQLWQ
LLSTFALIGIVSTISLWAIMPLLILFYATYIYYQSTSREVRRLDSVTRSPIYALFGEALNGLSSIRAYKAYDRMAKI
NGKSMDNNIRFTLASTSSNRWLTIRSESLGGVMIWLTATFAVLRYGNAENQAVFASTMGLLLSYTLNITTLLSG
VLRQASKAENSLNSVERVGNYIDLPSEATAIIENNRPVSGWPSRGSIQFEDVHLRYRPGLPPVLHGLSFFVYPSE
KVGVVGRTGAGKSSMLNALYRIVELEKGRILIDDYDVAKFGLTDLRRVLSIIPQSPVLFSGTVRFNIDPFSEHND
ADLWEALERAHIKDVIDRNPFGLDAEVSEGGENFSVGQRQLLSLARALLRRSKILFLDEATASVDVRTDSLIQR
TIREEFKSCTMLIIAHRLNTIIDCDKILVLSSGQVLEYDSPQELLSRDTSAFFKMVHSTGPENGQYLSNLVFERRG
NGMSQGG

>AtABCC12
MGFEALNWYCKPVADGFWEKAVDGAFGAYTPCAIDSLVMLVSHFVLLGLCFYRIWIIFHNTKAQIYVLRKKY
YNCVLGLLACYCVVEPVLRLVMGISLFDMDEETDFPPFEVASLMVEAFAWFSMLVLIGLETKQYVKEFRWYV
RFGVLYVLVADAVLLDLVLPLKNSINRTALYLFISSRCSQALFGILLLIYIPELDPYPGYHIVNNEPLDNVEYDAL
RGGEHICPERHASIFSRIYFGWITPLMQLGYRKPITEKDVWQLDKWDQTETLIKRFQRCWTEESRRPKPWLLRA
LNNSLGGRFWLAGIFKIGNDLSQFVGPVILSHLLRSMQEGDPAWVGYVYAFIIFVGVTLGVLCEAQYFQNVWR
VGFRLRSTLVAAIFHKSLRLTHEARKNFASGKVTNMITTDANALQQISQQLHGLWSAPFRIIVSMILLYQQLGV

ASLFGSLILFLLIPLQTLIISKMRKLTKEGLQWTDKRVGITNEILSSMDTVKCYAWEKSFESRIQGIRNEELSWFR
KAQLLSAFNSFILNSIPVVVTVVSFGVFVLLGGDLTPARAFTSLSLFAVLRFPLNMLPNLLSQVVNANVSLQRIE
ELLLSEERILAQNPPLQPGTPAISIKNGYFSWDSKTTKPTLSDINLEIPVGTLVAIVGGTGEGKTSLISAMLGELSH
AETTSVVIRGSVAYVPQVSWIFNATVRENILFGSDFESERYWRAIDATALQHDLDLLPGRDLTEIGERGVNISGG
QKQRVSMARAVYSNSDVYIFDDPLSALDAHVAHQVFDSCMKDELRGKTRVLVTNQLHFLPLMDKIILVSEGM
IKEEGTFVELSKSGILFKKLMENAGKMDATQEVNTNDENILKLGPTVTVDVSERNLGSTKQGKRRRSVLIKQEE
RETGIISWNVLMRYKEAVGGLWVVMILLACYLATEVLRVSSSTWLSIWTDQSTSKNYSPGFYIVVYALLGFGQ
VAVTFTNSFWLITSSLHAARRLHDAMLSSILRAPMLFFHTNPTGRVINRFSKDIGDIDRNVANLMNMFMNQLW
QLLSTFALIGTVSTISLWAIMPLLILFYAAYLYYQSTSREVRRLDSVTRSPIYAQFGEALNGLSSIRAYKAYDRM
AKINGKSMDNNIRFTLANTSSNRWLTIRLETLGGVMIWLTATFAVLQNGNTNNQAGFASTMGLLLSYTLNITS
LLSGVLRQASRAENSLNSVERVGNYIDLPSEATDIIENNRPVCGWPSGGSIKFEDVHLRYRPGLPPVLHGLTFFV
SPSEKVGVVGRTGAGKSSMLNALFRIVEVEKGRIMIDDCDVAKFGLTDVRRVLSIIPQSPVLFSGTVRFNIDPFS
EHNDAGLWEALHRAHIKDVISRNPFGLDAEVCEGGENFSVGQRQLLSLARALLRRSKILVLDEATASVDVRTD
SLIQRTIREEFKSCTMLVIAHRLNTIIDCDKILVLSSGQVLEYDSPQELLSRDTSAFFRMVHSTGPANAQYLSNLV
FERRENGMSVGG

>AtABCC13
MAITLTNFTFLYMDANLKRLGDIVLGFGANVVTLLLILILTITRRNGRCNRRKSYIEKCLLYVTPALGACLSCVD
LVLLVRTNRRREVILCFVPLSGFVMWIAVILSLKFACCACHVFTSQILCFWWIFRFLTDALHLNMIFTLQRVQEI
CLIMLDIAFGISINVLRIKQAHPKIIPLEDPLIEDDDDQKRIVRRLFLEKNGSWWDLFTFGYIGSIMKHGSVKQLE
LENLLTLPPEMDPFTCCENLLRCWQLQECNNYSTPSLIWSIYGVYGWPYFRLGLLKVFNDCIGFAGPLLLNRLI
KSFLDTQYTFRLSKLKLKLRSSIMSVIYRKCLWVNTANRSGFSEGEIQTFMSVDADRIVNLCNSLHDLWSLPLQI
GIALYLLYTQVKFAFLSGLAITILLIPVNKWISVLIASATEKMMKLKDERIRKTGELLTNIRTLKMYGWDNWFA
DWLKETRATEVTHLATRKYLDAWCVFFWATTPTLFSLCTFGLFALMGHQLDAATVFTCLALFNSLISPLNSFP
WVINGLIDAFISTRRVSKFLCCLEHSRDFSIDSGFTSEDLAVCVEDASCTWSSNVEEDYNLTIKQVSLRVPKGSF
VAVIGEVGSGKTSLLNSLLGEMRCVHGSILLNGSVAYVPQVPWLLSGTVRENILFGKPFDSKRYFETLSACALD
VDISLMVGGDMACIGDKGLNLSGGQRARFALARAVYHGSDMYLLDDVLSAVDSQVGCWILQRALLGPLLNK
KTRVMCTHNIQAISCADMIVVMDKGKVNWSGSVTDMPKSISPTFSLTNEFDMSSPNHLTKRKETLSIKEDGVD
EISEAAADIVKLEERKEGRVEMMVYRNYAVFSGWFITIVILVSAVLMQGSRNGNDLWLSYWVDKTGKGVSHY
STSFYLMVLCIFCIINSILTLVRAFSFAFGGLKAAVHVHNALISKLINAPTQFFDQTPSGRILNRFSSDLYTIDDSLP
FILNILLANFVGLLGIIVVLSYVQVLFLLLLLPFWYIYSKLQVFYRSTSRELRRLDSVSRSPIYASFTETLDGSSTIR
AFKSEEHFVGRFIEHLTLYQRTSYSEIIASLWLSLRLQLLGSMIVLFVAVMAVLGSGGNFPISFGTPGLVGLALSY
AAPLVSLLGSLLTSFTETEKEMVSVERVLQYMDVPQEEVSGPQSLSDKWPVHGLVEFHNVTMRYISTLPPALT
QISFTIQGGMHVGVIGRTGAGKSSILNALFRLTPVCSGEILVDGKNISHLPIRELRSCLAVVPQSPFLFQGSLRDN
LDPLGLSEDWRIWEILDKCKVKAAVESVGGLDSYVKESGCSFSVGQRQLLCLARALLKSSKILCLDECTANIDV
HTASLLHNTISSECKGVTVITIAHRISTVVDLDSILILDRGILVEQGKPQHLLQDDSSTFSSFVRASQ

>AtABCC14
MRWLSSTWLSDLSCSSSSVIEPSSSLPAPIQWLRFILLSPCPQRLLSSTVDVLFLLILFFFAIQKLCSSSSSRTNGEA
DITKPLLGRRTRTRTTGLFKTTVVVTIVLSFCSLVLCVSAFFTTRTKLKLVDTLFWLIHAVTNVVIAVLVLHLKR
FASPSHPLTLRIYWVFNFVVTTLFTVSGILHLLSDDPAAASLRADDVASFISFPLTAVLLLVSIKGSTGVVVTTSN
VTAPAKSNDVVVEKSENVSLYASASFISKTFWLWMNPLLRKGYKSPLNLDQVPTLSPEHRAEKLATLFESKWP
KPQENSRNPVRTTLIRCFWKEIAFTAVLAIIRLSVIYVGPVLIQSFVDFTSGKRSSPSQGYYLVLILLIAKFVEVLS
THQFNFNSQKLGMLIRSTLITALYKKGLKLTGSARQNHGVGQIVNYMAVDAQQLSDMMLQLHAIWLMPLQV
AAAIVLLYNTLGPSVVTTVIGLTGIFVFILLGTKRNNRYQFSLMMNRDSRMKATNEMLNYMRVIKFQAWEDHF
NERILKFREMEFGWLSKFLYSIAGNIIVLWSTPVLISALTFTTAVFLGVKLDAGTVFTTTTIFKILQEPIRTFPQSMI
SLSQAMISLGRLDAYMMSRELSEETVERSQGCDGNVAVEIKDGSFSWDDEDDEPAIENINFEVKKGELAAIVGT
VGSGKSSLLASVLGEMHKLSGKVRVCGTTAYVAQTSWIQNGTVQDNILFGLPMNRSKYNEVLKVCCLEKDM
QIMEFGDQTEIGERGINLSGGQKQRIQLARAVYQESDVYLLDDVFSAVDAHTGSDIFKKCVRGALKGKTILLVT
HQVDFLHNVDRILVMRDGMIVQSGKYDELVSSGLDFGELVAAHETSMELVEAGSASATAANVPMASPITQRSI
SIESPRQPKSPKVHRTTSMESPRVLRTTSMESPRLSELNDESIKSFLGSNIPEDGSRLIKEEEREVGQVSFQVYKLY
STEAYGWWGMILVVFFSVAWQASLMASDYWLAYETSAKNEVSFDATVFIRVYVIIAAVSIVLVCLRAFYVTH
LGLKTAQIFFKQILNSLVHAPMSFFDTTPSGRILSRASTDQTNVDIFIPFMIGLVATMYTTLLSIFIVTCQYAWPTV
FFIIPLGWLNIWYRGYYLASSRELTRLDSITKAPVIHHFSESIAGVMTIRAFKKQPMFRQENVKRVNANLRMDF

HNNGSNEWLGFRLELIGSWVLCISALFMVMLPSNIIKPENVGLSLSYGLSLNGVLFWAIYLSCFIENKMVSVERI
KQFTDIPAEAKWEIKESRPPPNWPYKGNIRLEDVKVRYRPNTPLVLKGLTIDIKGGEKIGVVGRTGSGKSTLIQV
LFRLVEPSGGKIIIDGIDICTLGLHDLRSRFGIIPQEPVLFEGTVRSNIDPTEKYSDEEIWKSLERCQLKDVVASKPE
KLDSLVADNGENWSVGQRQLLCLGRVMLKRSRILFLDEATASVDSQTDAMIQKIIREDFSDCTIISIAHRIPTVM
DCDRVLVIDAGKAKEYDSPVRLLERQSLFAALVQEYALRSAGI

>AtABCC15
MSVDVQRITDFIWYVNSIWMLPIQIFSAIYILQKHLGLGALAALVTTLMVMACNYPLTRLQRNYQSDIMNAKD
DRMKATSEILKNMKILKLQAWDNQFLNKVKTLRKKEYDCLWKSLRLQDFTTFILWGAPSLISVVTFVTCMLM
GVKLTAGAVLSALATFQMLQSPIFGLPDLLSALVQSKVSADRIASYLQQSETQKDAVEYCSNDHTEFSVEIENG
AFSWEPESSRPTLDDIELKVKSGMKVAICGAVGSGKSSLPSSILGEIQKLKGTVRVSGKQAYVPQSPWILSGTIR
DNILFGSIYESEKYERTVKACALIKDFELFSNGDLTEIGERGINMSGGQKQRIQIARAVYQNADIYLLDDPFSAV
DAHTGRELFEDCLMGILKDKTVLYVTHQVEFLPAADLILVMQNGRVMQAGKFEELLKQNIGFEVLTQCDSEH
NISTENKKKEAKLVQDEETEKGVIGKEVYLTYLTTVKGGLLVPFIILAQSCFQMLQIASNYWMAWTAPPTAESI
PKLGMGRILLVYALLAAGSSLCVLARTILVAIGGLSTAETFFSRMLCSIFRAPMSYFDSTPTGRILNRASTDQSVL
DLEMAVKLGWCAFSIIQIVGTIFVMSQVAWQVCVIFIPVAVACVFYQRYYTPTERELSRMSGVERAPILHHFAE
SLAGATTIRAFDQRDRFISSNLVLIDSHSRPWFHVASAMEWLSFRLNLLSHFVFAFSLVLLVTLPEGVINPSIAGL
GVTYGLSLNVLQATVIWNICNAENKMISVERILQHSKIPSEAPLVIDDQRPLDNWPNVGSIVFRDLQVRYAEHF
PAVLKNITCAFPGGKKIGVVGRTGSGKSTLIQALFRIVEPSHGTIVIDNVDITKIGLHDLRSRLGIIPQDNALFDGT
IRLNLDPLAQYTDREIWEALDKCQLGDVIRAKDEKLDATVVENGENWSVGQRQLVCLGRVLLKKSNILVLDE
ATASVDSATDGVIQKIINQEFKDRTVVTIAHRIHTVIESDLVLVLSDGRIAEFDSPAKLLQREDSFFSKLIKEYSLR
SNHFAGSNDLLS

>AtABCC4
MWLLSSSPWLSELSCSYSAVVEHTSSVPVPIQWLRFVLLSPCPQRALFSAVDFIFLLCFALHKLFSSPSSSSEING
HAEIRKPLIGIRGRTPTRTTAWFKTTVAVTVLLSFCSVVLCVLAFTGKRRTQRPWNLIDPLFWLIHAVTHLVIAV
LVLHQKRFAALNHPLSLRIYWISSFVLTSLFAVTGIFHFLSDAATSLRAEDVASFFSFPLTAFLLIASVRGITGLVT
AETNSPTKPSDAVSVEKSDNVSLYASASVFSKTFWLWMNPLLSKGYKSPLTLEQVPTLSPEHKAERLALLFESS
WPKPSENSSHPIRTTLLRCFWKEILFTAILAIVRLGVMYVGPVLIQSFVDFTSGKRSSPWQGYYLVLILLVAKFV
EVLTTHQFNFDSQKLGMLIRSTLITALYKKGLKLTGSARQNHGVGQIVNYMAVDAQQLSDMMLQLHAIWLM
PLQVTVALVLLYGSLGASVITAVIGLTGVFVFILLGTQRNNGYQFSLMGNRDSRMKATNEMLNYMRVIKFQA
WENHFNKRILKFRDMEFGWLSKFLYSIAGNIIVLWSTPVLISALTFATALALGVKLDAGTVFTTTTIFKILQEPIR
TFPQSMISLSQAMISLGRLDSYMMSKELSEDAVERALGCDGNTAVEVRDGSFSWDDEDNEPALSDINFKVKKG
ELTAIVGTVGSGKSSLLASVLGEMHRISGQVRVCGSTGYVAQTSWIENGTVQDNILFGLPMVREKYNKVLNVC
SLEKDLQMMEFGDKTEIGERGINLSGGQKQRIQLARAVYQECDVYLLDDVFSAVDAHTGSDIFKKCVRGALK
GKTVLLVTHQVDFLHNVDCILVMRDGKIVESGKYDELVSSGLDFGELVAAHETSMELVEAGADSAAVATSPR
TPTSPHASSPRTSMESPHLSDLNDEHIKSFLGSHIVEDGSKLIKEEERETGQVSLGVYKQYCTEAYGWWGIVLV
LFFSLTWQGSLMASDYWLAYETSAKNAISFDASVFILGYVIIALVSIVLVSIRSYYVTHLGLKTAQIFFRQILNSIL
HAPMSFFDTTPSGRILSRASTDQTNVDILIPFMLGLVVSMYTTLLSIFIVTCQYAWPTAFFVIPLGWLNIWYRNY
YLASSRELTRMDSITKAPIIHHFSESIAGVMTIRSFRKQELFRQENVKRVNDNLRMDFHNNGSNEWLGFRLELV
GSWVLCISALFMVLLPSNVIRPENVGLSLSYGLSLNSVLFFAIYMSCFVENKMVSVERIKQFTDIPSESEWERKE
TLPPSNWPFHGNVHLEDLKVRYRPNTPLVLKGITLDIKGGEKVGVVGRTGSGKSTLIQVLFRLVEPSGGKIIIDGI
DISTLGLHDLRSRFGIIPQEPVLFEGTVRSNIDPTEQYSDEEIWKSLERCQLKDVVATKPEKLDSLVVDNGENWS
VGQRQLLCLGRVMLKRSRLLFLDEATASVDSQTDAVIQKIIREDFASCTIISIAHRIPTVMDGDRVLVIDAGKAK
EFDSPARLLERPSLFAALVQEYALRSAGI

>AtABCC6
MENPIDSLLLQPIYLSVLSFFLNLVLLLILFGSWLFKKRVACEDTDAIMNEEFKHISFSYNKLVLICCVSLSVFYS
VLSLLSCLHWHTNGWPFLDLLLAALTWGSISVYLFGRYTNSCEQKVLFLLRVWWVFFFVVSCYHLVVDFVLY
KKQEMVSVHFVISDLVGVCAGLFLCCSCLWKKGEGERIDLLKEPLLSSAESSDNEEVTAPFSKAGILSRMSFSW
MSPLITLGNEKIIDIKDVPQLDRSDTTESLFWIFRSKLEWDDGERRITTFKLIKALFLSVWRDIVLSALLAFVYTV
SCYVAPYLMDNFVQYLNGNRQYKNQGYVLVTTFFVAKLVECQTQRQWFFRGQKAGLGMRSVLVSMIYEKG
LTLPCHSKQGHTSGEIINLMAVDADRISAFSWFMHDPWILVLQVSLALWILYKSLGLGSIAAFPATILVMLANY
PFAKLEEKFQSSLMKSKDNRMKKTSEVLLNMKILKLQGWEMKFLSKILELRHIEAGWLKKFVYNSSAINSVLW

AAPSFISATAFGACLLLKIPLESGKILAALATFRILQGPIYKLPETISMIVQTKVSLNRIASFLCLDDLQQDVVGRL
PSGSSEMAVEISNGTFSWDDSSPIPTLRDMNFKVSQGMNVAICGTVGSGKSSLLSSILGEVPKISGNLKVCGRKA
YIAQSPWIQSGKVEENILFGKPMEREWYDRVLEACSLNKDLEILPFHDQTVIGERGINLSGGQKQRIQIARALYQ
DADIYLFDDPFSAVDAHTGSHLFKEVLLGLLRHKTVIYVTHQVEFLPEADLILVMKDGKITQAGKYHEILDSGT
DFMELVGAHTEALATIDSCETGYASEKSTTDKENEVLHHKEKQENGSDNKPSGQLVQEEEREKGKVGFTVYK
KYMALAYGGAVIPLILVVQVLFQLLSIGSNYWMTWVTPVSKDVEPPVSGFTLILVYVLLAVASSFCILIRALLV
AMTGFKMATELFTQMHLRIFRASMSFFDATPMGRILNRASTDQSVADLRLPGQFAYVAIAAINILGIIGVIVQVA
WQVLIVFIPVVAACAWYRQYYISAARELARLAGISRSPVVHHFSETLSGITTIRSFDQEPRFRGDIMRLSDCYSR
LKFHSTGAMEWLCFRLELLSTFAFASSLVILVSAPEGVINPSLAGLAITYALNLNTLQATLIWTLCDLENKMISV
ERMLQYTNIPSEPPLVIETTRPEKSWPSRGEITICNLQVRYGPHLPMVLHGLTCTFPGGLKTGIVGRTGCGKSTLI
QTLFRIVEPAAGEIRIDGINILSIGLHDLRSRLSIIPQDPTMFEGTIRSNLDPLEEYTDDQIWEALDNCQLGDEVRK
KELKLDSPVSENGQNWSVGQRQLVCLGRVLLKRSKLLVLDEATASIDTATDNLIQETLRHHFADCTVITIAHRI
SSVIDSDMVLLLDQGLIKEHDSPARLLEDRSSLFSKLVAEYTTSSESKSKRS

>AtABCC7
MKQSYAMDNPIVFFLLESNYFPMFSIFFNLLLLLVMFGSCVYKKRLGWENSDAFTNERFKDMSLTYNKLVVIC
CETLSALNSVLLLLSCFNLHKNGWDRSELMILLDLLFTALSWGAISFYIRSQFTYSHDQKFPILLRVWWVLYFM
FSCYRLLVDIALYKKQELVSVHLLLSDVLAVSVGLFLCYSCLQKQGQGERINLLLEEPLLNGAESSAATSVQLD
KAEDDEVVTPFSNAGFLSHVSFSWMSPLIVLGNEKIIDSEDVPQVDNSDRAEKLFWIFRSKLEWDDGERRITTY
KLIKALFFSVWRDILLSTLFAFVYTVSCYVAPYLMDTFVQYLNGQRQYSNQGVVLVTTFFVAKLVECQARRN
WYFRLQKAGIGMRSVLVSMIYEKGLTLPCYSKQGHTSGEIINLMTVDAERISAFSWYMHDPWILVLQISLALLI
LYRSLGLGSIAAFAATFLVMLGNIPLAKLEEKFQGNLMESKDNRMKKTSEALLNMRILKLQGWEMKFLHKILD
LRGIEAGWLKKFVYNSAAISSVLWAAPSFVSATAFGACMLLKIPLESGKIIAALATFRILQTPIYKLPDTISMIVQ
TKVSLDRIATFLCLDDLQQDGMERLPSGSSKMDVEVSNGAFSWDDSSPIPTLKDIRFKIPHGMNIAICGTVGSGK
SSLLSSILGEVPKISGNLKVCGRKAYIAQSPWIQSGKVEENILFGKPMQREWYQRVLEACSLNKDLEVFPFRDQ
TVIGERGINLSGGQKQRIQIARALYQDADIYLFDDPFSAVDAHTGSHLFKEVLLGLLRNKTVIYVTHQLEFLPEA
DLILVMKDGRITQAGKYNEILESGTDFMELVGAHTDALAAVDSYEKGSASAQSTTSKESKVSNDEEKQEEDLP
SPKGQLVQEEEREKGKVGFTVYQKYMKLAYGGALVPIILVVQILFQVLNIGSNYWMAWVTPVSKDVKPLVSG
STLILVYVFLATASSFCILVRAMLSAMTGFKIATELFNQMHFRIFRASMSFFDATPIGRILNRASTDQSAVDLRLP
SQFSNLAIAAVNILGIIGVMGQVAWQVLIVFIPVIAACTWYRQYYISAARELARLSGISRSPLVQHFSETLSGITTI
RSFDQEPRFRTDIMRLNDCYSRLRFHAISAMEWLCFRLDLLSTVAFALSLVILVSVPEGVINPSFAGLAVTYALN
LNSLQATLIWTLCDLENKMISVERMLQYIDIPSEPSLVIESTRPEKSWPCRGEITICNLQVRYGPHLPMVLRGLTC
TFRGGLKTGIVGRTGCGKSTLIQTLFRIVEPAAGEIRIDGINILTIGLHDLRSRLSIIPQEPTMFEGTVRSNLDPLEE
YADDQIWEALDKCQLGDEIRKKELKLDSPVSENGQNWSVGQRQLVCLGRVLLKRSKVLILDEATASVDTATD
TLIQETLRQHFSGCTVITIAHRISSVIDSDMVLLLDQGLIEEHDSPARLLEDKSSSFSKLVAEYTASSDSRFKRSSM
KTN

>AtABCC8
MAAFIGSLPWCDVELNLASSCFQRTAIAFVNLLFLCIFYLFLIASCVSTHFIVRGRKKGWIFVAVAICCAITSFIFL
GVGLNSLIHGGNDVTEISWVACFVEGIIWVSLAVSLLVNGSKWVNILVSVWWVSFALLDLVAKSGILLQGNGI
RILDILTLPMSLLLLLCSWMNLRSSSAAAQDCSVTGLSDPLLTKNPRKESARLATAGFFSILSFSWMNPLLSLGF
KKPLSPEDIPSVVPEDEAQLAYKKFSQAWDTLLGDESSTKERNLVFRAVVKVYFKENIFIAVFAFLRTFAVVSLP
LMLYVFVDYANSDHRDLRNGFFNLACLVMLKLVESLTMRHWYFASRRSGMRIRSALMVAAYKKQLKLSSLG
RKRHSSGEIVNYIAVDAYRMGEFLWWFHSGWSLSLQLLLSTAVLFGVVGAGAFPGLILLLLCGLLNLPFAKML
QNCQTQFMIAQDKRLRSTSEILNSMKVIKLQSWEDEFKKKIESCRDDEFTWLAKAQLTKAFGSFLYWMSPTIVS
SVVFLGCALLKSAPLNASTIFTVLATLRVMSEPVKIIPDAISAIIQGNVSFQRLNNFLLDDELKMDEIERSGLDAS
GTAVDIQVGNFGWEPETKIPTLRNIHLEIKHGQKVAVCGPVGAGKSSLLHAVLGEIPKVSGTVKVFGSIAYVSQ
TSWIQSGTIRDNILYGKPMESRRYNAAIKACALDKDMNGFGHGDLTEIGQRGINLSGGQKQRIQLARAVYADA
DVYLLDDPFSAVDAHTAGVLFHKCVEDSLKEKTVILVTHQVEFLSEVDQILVMEEGTITQSGKYEELLMMGTA
FQQLVNAHNDAVTVLPLASNESLGDLRKEGKDREIRNMTVVEKIEEEIEKTDIPGVQLTQEEEKESGYVGMKPF
LDYIGVSRGWCLLWSSVLGQVGFVVFQAASTYWLAFAIGIPKITNTMLIGVYSIISTLSAGFVYARAITTAHLGL
KASKAFFSGFTNAVFKAPMLFFDSTPVGRILTRASSDLNVLDYDVPFAFIFVVAPAVELTAALLIMTYVTWQVII
IALLALAATKVVQDYYLASARELIRINGTTKAPVMNYAAETSLGVVTIRAFGTAERFFKNYLNLVDADAVLFF
LSNAAMEWVILRIETLQNVTLFTCALLLILIPKGYIAPGLVGLSLSYALTLTQTQVFLTRWYCTLSNSIISVERIKQ

YMNIPEEPPAIIDDKRPPSSWPSNGTIHLQELKIRYRPNAPLVLKGISCTFREGTRVGVVGRTGSGKSTLISALFRL
VEPASGCILIDGIDISKIGLKDLRMKLSIIPQEPTLFRGCIRTNLDPLGVYSDDEIWKALEKCQLKTTISNLPNKLD
SSVSDEGENWSVGQRQLFCLGRVLLKRNKILVLDEATASIDSATDAIIQRIIREEFADCTVITVAHRVPTVIDSDM
VMVLSFGDLVEYNEPSKLMETDSYFSKLVAEYWASCRGNSSQNLQL

>AtABCC9
MFKPFGFAAETGSHLLTTQWLQLGNSLCLKERISIAMQVTFLAFFLIHLALKWFGVVRNRGSNDVEEDLKKQSI
TVKQSFSYNISLLCSVSILGTHCFILLLLFRDSVVSRCDSSVSVFSAEVSQSFSWLFVSVVVVKIRERRLVKFPWM
LRSWWLCSFILSFSFDAHFITAKHEPLEFQDYADLTGLLASLFLLAVSIRGKTGFHLLESSGNTEPLLLGDQTEQ
NKKDSYSSSSPYGNATLFQRITFSWINPLFSLGYKRPLEKDDVPDIDVKDSARFCSHAFDQKLKTTKEKEGPGN
AFFYNSVLRYVWRKAAINAVFAVVNASTAYIGPYLINDFVEFLSEKQSQSLNHGYLLALGFLTAKIVETVTQRQ
WIFGARQLGLRLRAALISHIYQKGLVLSSQSRQSHTSGEIINYMSVDVQRITDFIWYVNNIWMLPIQIFSAIYILQ
KHLGLGALAALVTTLMVMACNYPLTRLQRNYQSDIMNAKDDRMKATSEILKNMKILKLQAWDNQFLNKVK
TLRKKEYDCLWKSLRLQAFTTFILWGAPSLISVVTFVTCMLMGVKLTAGAVLSALATFQMLQSPIFGLPDLLSA
LVQSKVSADRIASYLQQSETQKDAVEYCSKDHTELSVEIENGAFSWEPESSRPTLDDIELKVKSGMKVAVCGA
VGSGKSSLLSSILGEIQKLKGTVRVSGKQAYVPQSPWILSGTIRDNILFGSMYESEKYERTVKACALIKDFELFSN
GDLTEIGERGINMSGGQKQRIQIARAVYQNADIYLLDDPFSAVDAHTGRELFEDCLMGILKDKTVLYVTHQVE
FLPAADLILVMQNGRVMQAGKFEELLKQNIGFEVLVGAHNEALDSILSIEKSSRNFKEGSKDDTASIAESLQTH
CDSEHNISTENKKKEAKLVQDEETEKGVIGKEVYLAYLTTVKGGLLVPFIILAQSCFQMLQIASNYWMAWTAP
PTAESIPKLGMGRILLVYALLAAGSSLCVLARTILVAIGGLSTAETFFSRMLCSIFRAPMSFFDSTPTGRILNRAST
DQSVLDLEMAVKLGWCAFSIIQIVGTIFVMSQVAWQVCVIFIPVAVACVFYQRYYTPTARELSRMSGVERAPIL
HHFAESLAGATTIRAFDQRDRFISSNLVLIDSHSRPWFHVASAMEWLSFRLNLLSHFVFAFSLVLLVTLPEGVIN
PSIAGLGVTYGLSLNVLQATVIWNICNAENKMISVERILQYSKIPSEAPLVIDGHRPLDNWPNVGSIVFRDLQVR
YAEHFPAVLKNITCEFPGGKKIGVVGRTGSGKSTLIQALFRIVEPSQGTIVIDNVDITKIGLHDLRSRLGIIPQDPA
LFDGTIRLNLDPLAQYTDHEIWEAIDKCQLGDVIRAKDERLDATVVENGENWSVGQRQLVCLGRVLLKKSNIL
VLDEATASVDSATDGVIQKIINQEFKDRTVVTIAHRIHTVIESDLVLVLSDGRIAEFDSPAKLLQREDSFFSKLIKE
YSLRSNHFAGSNDLLS

>XP_002265012.1_ABCC4_Vv
MSSASWITTLSCSSSVIASSGETPFSLILQWLRFIFLSPCPQRALLSSIDLLFLLTLIAFSVQKLYSRFISNGRSSSAIN
KPLIRNNRARLRTTLWFKLTLTATALLAVCHGFLCILAFARGAQMPWKLIDALFWLVEAITHFLITILIAHGKRF
QAVTYPLSLRIFWVVSFIISSLFTTSGIIRIFFVEGFEASNLRLDDIVTLVTFPLSVVLLLVGIRGSTGITVDRESEPV
MDVEEKLYEPLLGKSNVTGFASASILSKALWLWMNPLLGKGYKSPLKIDEIPSLSPEHRAERMSELFESNWPKP
HEKLNHPVRTTLFRCFWREVAFTAFLAIVRLCVIYVGPLLIQRFVDFTSGKRSSPYEGYYLVLILLIAKTVEVLTS
HHFNFNSQKLGMLIRSTLITSLYRKGLRLSCSARQDHGVGQIVNYMAVDAQQLSDMMLQLHAIWLMPLQVTV
ALVLLYNELGGAMITAVIGIFAVLLFVLMGTRRNNRFQHNVMKNRDLRMKATNEMLNYMRVIKFQAWEEHF
NKRIQSFRESEFGWLTKFMYSISGNIIVMWSTPLMISAFTFATAIMLGVQLDAGTVFTTTSIFKILQEPIRAFPQSM
ISISQAMISLARLDKYMTSRELVESSVEREESCDGRIAVEVKDGVFSWDDEGKEEVLRNLNFEIKKGELAAIVGT
VGSGKSSLLASVLGEMHKISGQVRLCGTTAYVAQTSWIQNGTIQENILFGLPMNTEKYREVIRVCCLEKDLEM
MEYGDQTEIGERGINLSGGQKQRIQLARAVYQDCDVYLLDDVFSAVDAHTGTDIFKECVRGALRNKTILLVTH
QVDFLHNVDLILVMRDGMIVQSGKYNDLLESGMDFKALVAAHETSMELVEEAGPAITSENSPKLPQSPQPFSN
HGEANGVDKSGDQSKSNKESSKLIKDEERETGKVSFQVYKQYCTEAYGWSGLAGVLLLSLAWQGSLMASDY
WLAYETSEKHAKSFNASLFITNYSIIAAVSVLLIVIRSFTVTKLGLKTAQIFFSQILHSILHAPMSFFDTTPSGRILS
RASTDQTNVDLFVPFFMAVTLAMYITLLSIIIITCQYAWPTIFLLIPLGWLNVWYRGYFIASSREITRLDSITKAPV
IHHFSESISGVTTIRCFRKQIGFTQENVHRVDKNLRMDFHNNGSNEWLGFRLELIGSFIMCLSTMFMILLPSSIIKP
ENVGLSLSYGLSLNSVLFWAIYMSCFVENKMVSVERIKQFTNIPSEAAWQIKDRLPPPNWPTHGNVELKDLQV
RYRPNSPLVLKGITLNIRGKEKIGVVGRTGSGKSTLVQVFFRLVEPSGGKIIIDGIDIGMLGLHDLRSRFGIIPQEP
VLFEGTVRSNVDPVGQYSDEEIWQSLEHCQLKEVVAGKPDKLDSLVVDNGDNWSVGQRQLLCLGRVMLKRS
RILFLDEATASVDSQTDAVIQRIIREDFANCTIISIAHRIPTVMDCDRVLVIDAGRAKEFDKPSRLLERHSLFGALV
QEYANRSAGM

>XP_002280819.1_ABCC2_Vv
MAFGPLVWYCRPVTNGVWAKLVDNAFGVYTPCATDTLVISISHFILLSLCFYRIWRIKKDFKVQRFCLRSNYY
NYMLALLAGYCTAEPLFRLIMGISVFNLDGQAGLAPFEVVSLIIKAFTWCSMLVLIGIETKVYIREFRWYLRFGV

LYTLIGEAVMLNLILSVKELYDRSILYLYISEVVLQVLFGILLLFYVPDLDPYPGYTPMWTGSVDDAEYEEIPGG
EQICPERHVNIFSRITFGWMNPIMQLGSKRPITEKDVWKLDSWDQTETLNNNFQRCWAEEALRPKPWLLRALN
RSLGGRFWWGGFWKIGNDLSQFVGPLILNQLLQSMQQGDPAWIGYIYAFSIFVGVVFGVLFEAQYFQNVMRV
GFRVRSTLVAAVFRKSLKLTHEGRRQFASGKITNLMTTDAEALQQICQSLHTLWSAPFRIIIAMVLLYQQLGVA
SLLGALMLVLLFPIQTVVISRMQKLSKEGLQRTDKRIGLMNEILAAMDTVKCYAWENSFQSKVQSVRNEELSW
FRKASFLGAFNVFMLNSIPVVVIVISFGMFTLLGGDLTPARAFTSLSLFAVLRFPLFMLPNIITQAVNANVSLKRL
EELFLAEERILLPNPPLEPGLPAISIKNGYFSWDSKADRPTLSNVNLDIPVGGLVAIVGGTGEGKTSLVSAMLGEL
PPMSDASAVIRGTVAYVPQVSWIFNATVRGNILFGSPFEAARYEKAIDVTALQHDLDLLPGGDLTEIGERGVNIS
GGQKQRVSMARAVYSNSDVYIFDDPLSALDAHVGRQVFDRCIKGELRGKTRVLVTNQLHFLSQVDRIILVHEG
MVKEEGTFEELSNNGMMFQKLMENAGKMEEYVEENGAEENIDDKTSKPVANGVVDKLPNNSSNTSKPKEGK
SVLIKQEERETGVVSWKVLVRYKNALGGLWVVMILFMCYILTETLRVSSSTWLSQWTDQGGSRTHGPGYYNL
IYAMLSFGQVLVTLANSYWLIMSSLYAAKRLHDAMLGSILRAPMLFFHTNPIGRIINRFAKDLGDIDRNVAVFV
NMFLGQISQLLSTFVLIGIVSTMSLWAIMPLLVLFYSAYLYYQNTAREVKRLDSITRSPVYAQFGEALNGLSTIR
AYKAYDRMADINGQSMDNNIRYTLVNMSSNRWLAIRLEALGGLMIWLTATFAVMQNERAENQQAFASTMG
LLLSYALNITSLLTGVLRLASLAENSLNSVERVGSYIELPSEAPLVIESNRPPPAWPSSGSIKFEDVVLRYRPELPP
VLHGLSFTISPSDKVGIVGRTGAGKSSMLNALFRIVELERGRILIDDCDISKFGLRDLRKVLGIIPQSPVLFSGTVR
FNLDPFNEHNDADLWEALERAHLKDVIRRNSLGLDAEVSEAGENFSVGQRQLLSLARALLRRSKILVLDEATA
AVDVRTDALIQKTIREEFKSCTMLIIAHRLNTIIDCDRVLLLDAGRVLEYDTPEELLSNDRSAFSKMVQSTGAAN
AEYLRSLVLGGEGENKLGREDNRRLDGQRRWLASSRWTAAAQFALAVSLTSSQNDLQQLEIEDENSILKKTK
DAVITLQGVLEGKHDKVIEETLNQYQVSRDGWWSSLYRMIEGLAVMSRLARNRLQSENGFEDRSIDWDRIEM

>XP_002281070.1_ABCC12_Vv
MAFEPLVWYCQPVANGVWAKAAESAFGPYTPCAVDSIVVCISHLVLLGLCCYRIWLIKMDFKVQRFCLQSNY
YNYMLGLLACYCTAEPLFRLVMGVSIFDLDEQTGLAPYEIVSLIIEAATWCSMLVMIGVETKIYIRQFRWYVRF
GVIYLLVGDAVMLNLILSLKDSYSRSVLYPPISSVLCQVLFGICLLVHVPNLNPYVGYTPMQSDSLENTKYEVLP
GGDQICPEKHANMFSRIYFGWMTPLMQQGYKKPITEKDIWKLDTWDQTETLSRRFQKCWIEESQRSKPRLLRA
LNCSLGGRFWRGGFFKIGNDLSQFVGPVLLNHLLQSMQRGDPAWIGYIYAFSIFIGVSLGVLCEAQYFQNVMR
VGFRLRSTLVAAIFRKSLRLTHEGRKNFPSGKITNMMTTDANALQQICQQLHALWSAPFRIIIAMVLLYQQLGV
ASLLGSLMLLLMLPIQTFIISKMRKLSKEGLQRTDKRVSLMNEILAAMDTVKCYAWEKSFQSKVQSMRNDELS
WFRKAQLLSACNSFILNSIPVIVTVTSFGAFTLLGGDLTPARAFTSLSLFAVLRFPLNMLPNLITQVVTAHVSIQR
LEQLFLTEERVLAPNPTLEPGLPAISIKDGYFSWDSKVEKPTLSNINLDIPVGSLVAVVGGTGEGKTSLISAMLGE
LPPLSDASVVIRGTVAYVPQISWIFNATVRGNILFGSDFEPARYWKAIDVTELQHDLDLLPGHDLTEIGERGVNI
SGGQKQRVSMARAVYSNSDVYIFDDPLSALDAHVAQQVFSNCIKEELKGKTRVLVTNQLHFLPHVDRIILVSD
GTVKEDGTFDDLSKNSKLFQKLMENAGKMEEQVEENECRENLSNNKSKPTTNGEVNELPKNAIHSNKGKEGK
SVLIKQEERETGIVSWKVLMRYKDALGGLWVVTLLFACYVLTEVLRVLSSTWLSVWTDQSMSKDYRPGYYN
LIYALLSFGQVMVTLGNSFWLITSSLHAAKILHNVMLNSILRAPMVFFHTNPIGRIINRFAKDLGDIDRNVAPSA
NMFLGQVWQLLSTFVLIAIVSTISLWAIMPLLILFYAAYLYYQSTSREVKRLDSITRSPVYAQFGEALNGLSTIRA
YKAYDRMASINGKSMDNNIRFTLANISSNRWLTIRLETLGGLMICLTATFAVMENSREENPAAFASTMGLLLSY
TLNITSLLSGVLRQASRAENSFNAVERVGTYVDLPSEAPTIIESNRPPPGWPSSGSIRFEDVVLRYRPELPPVLHGI
SFKISPSEKLGIVGRTGAGKSSMINALFRIVELERGRIWIDEYDIAKFGLTDLRKVLSIIPQSPVLFSGTVRFNLDPF
NEHNDADLWEALERAHLKDVIRRNSFGLDAEVAEGGENFSVGQRQLLSLARALLRRSKILVLDEATAAVDVR
TDALIQKTIREEFKTCTMLVIAHRLNTIIDCDRILVLDAGQVVEYDTPEELLQDEGSSFSRMVRSTGAANAQYLR
SLVFGEDGQKKSGREEAKQLDRQKRWLASSRWAAATQFALSISLTSSQNGLQFLDVEDEMNILKKTNDAVLT
LRGVLEGTHDEVIEEMLKEYQVPRDRWWSALYKMVEGLAVMNRLARHRFQQSEHDFEDTTLDWDLTEM

>XP_002448550.1_ABCC2_Sb
MGFDPLEWYCQPVKNGVWSLVVENAFGAYTPCGTDTLVVCISYLALFGVCFYRIWRTTRDYTVQRYKLRSPY
YNYLLGLLVVYCIAEPLYRIATGTSIMNLDGQPGLAPFEIVSLIIESAAWCCMLVMILLETRIYINEFRWYIRFVVI
YVMVGEAAMFNLVLSVRQYYSSSSIFYLYCSEIACQLLFGILMVVYLPSVDPYPGYTPIRNEVLVDNTDYEPLP
GGEQICPERHVNVFARIFFSWMTPLMQQGFRRPITDKDIWKLDSWDETETLYSQFQKCWNDELRKPKPWLLR
ALHSSLWGRFWLGGFFKIGNDASQFVGPLVLNLLLESMQKGDPSWSGYIYAFSIFAGVSLGVLAEAQYFQNV
MRVGFRLRSTLIAAVFRKSLRLTNESRRKFASGRITNLISTDAESLQQVCQQLHSLWSAPFRIVISMVLLYAQLG
PAALVGALMLVLLFPIQTVIISKMQKLTKEGLQRTDKRISLMNEVLAAMDTVKCYAWEQSFQSKVQDIRDDEL
SWFRRAQLLAALNSFILNSIPVVVTVVSFGVYSLLGGDLTPAKAFTSLSLFAVLRFPLFMLPNLITQVVNCKVSL

KRLEDLLLAEERLLLPNPPIDPDLPAISIKNGYFSWESEAQRPTLSNVNLDVPVGSLVAIVGSTGEGKTSLISAML
GEIPPVSGSGTSVVIRGSVAYVPQVSWIFNATVRDNILFGSPFQPPRYEKAIDVTSLRHDLDLLPGGDLTEIGERG
VNISGGQKQRVSMARAVYSDSDVYIFDDPLSALDAHVGRQVFDKCIKGELQHKTRVLVTNQLHFLPYVDKILL
IHDGVIKEEGTFDELSNSGELFKKLMENAGKMEEQVEEDESKPKDVAKQTENGDVIIADEGSQKSQDSSSKTKP
GKSVLIKQEERETGVVSANVLSRYKNALGGMWVVSILFFCYALTEVLRISSSTWLSIWTDQGSLKIHGPGYYNL
IYGILSFGQVLVTLSNSYWLIISSLRAAKRLHDAMLRSILRAPMVFFHTNPLGRIINRFSKDLGDIDRNVAVFVN
MFMAQISQLLSTFVLIGFVSTMSLWAIMPLLILFYAAYLYYQATSREVKRLDSITRSPVYAQFSEALNGLSTIRA
YKAYDRMANINGRSMDNNIRFTLVNMSANRWLAIRLETLGGIMIWFTATFAVMQNQRAENQKAFASTMGLL
LTYTLNITNLLTAVLRLASLAENSLNAVERVGTYIELPSEAPPVIEDHRPPPGWPSSGVIKFEDVVLRYRPELPPV
LHGISFLINGSEKVGIVGRTGAGKSSMLNALFRIVELERGRILIDDCDTSKFGIWDLRKVLGIIPQAPVLFSGSVRF
NLDPFNEHNDADLWEALERAHLKDVIRRNPLGLDAEVSEAGENFSVGQRQLLSLARALLRRAKILVLDEATAA
VDVRTDALIQKTIREEFKSCTMLIIAHRLNTVIDCDRLLILSAGQVLEFDSPENLLSNEESAFSKMVQSTGPSNAE
YLKSLVFGSGEERSRREEIKLQDIQRRWVASNRWAEAAQFALARSLTSSHSDLLALEAAEGNNILRRTKDAVIT
LQSVLEGKHNTEIDESLTLYQVPADRWWSSLYKVVEGLATMSRLARNRLQQPAYNFENNGSIDWDQM

>XP_003527527.1_ABCC12_Gm
MGFQPLNWYCRPAENSIWAKAVDSAFGSYTPCAINTLVISISNLVLVGLCLYRIWLITCNAKAQRFCLSSNCYS
YLMGMLAAYCAVQPILRLLTGNSAFNLNGETEFAPVEITTLIVEALTWSSMITLILLETKVYIRQFRWLVRFGVI
YVLVGDIVMLNLLLPVKDYCSRSALFLYISSFICQVLFGTLLFVYIPDLVPYSGHTTMQAELPDHGEYEPLCGDD
QVCPERHANIFSRICFGWITPLMKQGYRKPITEKDVWKLDEWDRTETLTEKFQKCWMLEFQSSNPWLLRALNS
SLGKRFWMGGIFKIGNDLSQFVGPILLNHLLDSMQRGDPSWIGYIYAFSIFVGVAVGVLCEAQYFQNVLRVGFR
LRSTLVAAIFRKSLRLTNDGRKNFPSGRLMNMITSDANALQQICQQLHGLWSAPFRITVAIVLLYQQLGVASLI
GSLMLVLIIPLQTFVISKMRKLTKEGLQQTDKRVGLMNEILAAMDTVKCYAWETSFQSRILSIRDNELSWFRKA
QLLYALNSFILNSIPVLVTVTSFGMFTLLGGELTPARAFTSLSLFSVLRFPLNMLPNLLSQVANANVSLQRLEELF
LAEERNLKQNPPIEPGLPAISIENGYFSWDRKEEKPTLSDINVEIPVGSLVAIIGGTGEGKTSLISAMIGELPPLAN
GNATIRGTVAYVPQISWIYNATVRENILFGSKFEYEQYRKVIDMTALQHDLNLLPGRDFTEIGERGVNISGGQK
QRVSIARAVYSNSDIYIFDDPLSALDAHIAQEVFRNCIKEGLRGKTRVLVTNQLHFLPQVDKIILVSEGMIKEQG
TFEELSKSGPLFQKLMENAGKMEQADNNEDRESHGTDNDLPMNNEAIEELPSDASYEKKGKLRKSVLIKKEER
ETGVVSWKVVMRYKSALGGLWVVSILFSCYTLTEVLRISSSTWLSVWTSQDSTADYDPTYFLLIYALFSFGQV
SVALANSYWLIICSLRAAKNLHDAMLDKILRAPMVFFQTNPVGRIINRFAKDTGDIDTNVFNLVNMFLGQVWQ
LLSTFVLIGTVSTISLWAIMPLLIFFYAAYLYYQSTAREVKRMDSITRSPVYAHFGESLNGLSSIRAYKAYDRMA
HINGKFMDKNIRFTLVNISSNRWLTIRLETLGGLMIWLIATSAVLQNARAANQAMFASTMGLLLSYTLNITNLL
SGVLRQASRAENSLNSVERVDTYINLETEAPGVIETHRPPPGWPTSGSIEFEDVVLRYRPELPPVLHGLSFTVPPT
EKIGIVGRTGAGKSSMLNALFRIVELQKGKIIIDGCDISTFGLEDVRKVLTIIPQSPVLFSGTVRFNLDPFNEHNDA
DLWQALERAHLKDVIRRNTFGLDAKVSEGGDNFSVGQRQLLSLARALLRRSKVLVLDEATAAVDVRTDALIQ
KTIRQEFQSCTMLIIAHRLNTIIDCNQILLLDAGRVLEYSSPEELLQNEGTAFYKMVQSTGPENAQYLCSLVFGK
TENNSNEYNKELEHHVRQLASSHWTSSTQFAIASTLSSLHQHLQEPSSEENKDILHKTTAAVTTLQEVLVGKHD
EDIEETLYKYHIPTDRWWSTLYKVIEGLSLLKRLPLDNIQQLELDFEGRSFD

>XP_003542944.1_ABCC2_Gm
MTFEPLDWYCRPVANGVWTRSVENAFGAYTPCAVDSLVISVSNLILLGLCIYRIWLIKKDFTVKRFHLRSNLYN
YILGLLALYCVAEPLYRLILGISVLNLDGQTQFAPFEIVSLIIEALAWCSILILIGIETKVYIREFRWFVRFGLIYAIV
GDAVMFNLIISVKELYSSSVLYLYISEVVGQVLFGILLLVYVPTLDPYPGYTPIGSDMITDAAYDELPGGDMICP
ERNANILSKIMFSWMNPIMKLGYQRPLTEKDIWKLDTWERTETLINKFQKCWVEESRKPKPWLLRALNASLGG
RFWWGGFCKIGNDISQFLGPLILNQLLQSMQNGDPSWTGYAYAFSIFVGVVFGVLCEAQYFQNVMRVGYRLR
STLVAAVFRKSLRLTHEARKQFATGKITNLMTTDAEALQQICQSLHTLWSAPFRIVVAMVLLYQQLGVASLLG
ALMLVLMFPLQTFIISRMQKFSKEGLQRTDKRIGLMNEILAAMDTVKYYAWESSFQSKVQIVRNDELSWFRKA
SLLGACNAFILNSIPVFVTVITFGVFTLLGGDLTPARAFTSLSLFSVLRFPLFMLPNTITQVVNANVSLKRLEDLL
LAEERILLSNPPLEPGLPAISIKNGYFSWDTKAERATLSNINLDIPVGCLVAVVGSTGEGKTSLVSAMLGELPPM
ADSTVVLRGTVAYVPQVSWIFNATVRDNVLFGSVFDPTRYERAINVTELQHDLELLPGGDHTEIGERGVNISGG
QKQRVSMARAVYSNSDVYIFDDPLSALDAHVARQVFDKCIKGDLREKTRVLVTNQLHFLSQVDRIILVHEGM
VKEEGTFEELSNHGLLFQKLMENAGKMEEYEEEEKVVTETTDQKPSSEPVANGSVNDHAKSGSKPKEGKSVLI
KQEERETGVVSWNVLLRYKNALGGFWVVFVLFACYVSTETLRISSSTWLSHWTDQSATKGYNPAFYNMIYAA
LSFGQVLVTLTNSYWLIISSLYAARRLHEAMLSSILRAPMVFFQTNPLGRVINRFAKDLGDIDRNVAPFVNMFL

GQVSQLLSTFILIGIVSTMSLWAILPLLVLFYVAYLYYQSTAREVKRLDSISRSPVYAQFGEALNGLSTIRAYKA
YDRMADINGKSMDNNIRFTLVNISGNRWLAIRLETLGGLMIWLTATFAVMQNGRAENQQEFASTMGLLLSYA
LNITSLLTGVLRLASLAENSLNAVERIGTYIDLPSEAPSIIDDNRPPPGWPSSGSIRFEDVVLRYRAELPPVLHGLS
FTIFPSDKVGIVGRTGAGKSSMLNALFRIVELERGRILIDDYDVAKFGLADLRKVLGIIPQSPVLFSGTVRFNLDP
FNEHNDADLWEALERAHLKDVIRRNSLGLDAEVSEAGENFSVGQRQLLSLSRALLRRSKILVLDEATAAVDVR
TDALIQKTIREEFKSCTMLIIAHRLNTIIDCDRILLLDGGKVLEYDTPEELLSNEGSAFSKMVQSTGAANAQYLRS
LALGGDKSEREENEHLDGKRKWLASSRWAAAAQFALAVSLTSSHNDLQRLEVEDENSILKKTKDALITLQGV
LERKYDKEIEESLNQRQVSPEGWWSSLYKMIEGLAMMSRLAKNRLHQSDFGFEDRSINFDQVDM

>XP_003546076.1_ABCC2_X1_Gm
MAFEPLNWYCRPVANGVWTKSVENAFGAYTPCAVDSLVISVSNLILLGLCIYRIWLIMKDFTVKRFCLRSNLY
NYILGLLALYCVAEPLYRLIMGISVLNLDGQTQLAPFEIISLIIEALAWCSILILIGIETKVYIREFRWFVRFGLIYAI
VGDAVMFNLIISAKEFYSSSVLYFYISEVVGQVLFGILLLVYVPTLDPYPGYTPIGTEMITDATYDELPGGDMICP
ERSANILSRIMFSWMNPIMKLGYERPLTEKDIWKLDTWERTETLINKFQKCWVEESRKSKPWLLRALNASLGG
RFWWGGFCKIGNDISQFMGPLILNQLLQSMQNGEPSWTGYVYAFSIFVGVVFGVLCEAQYFQNVMRVGYRLR
STLVAAVFRKSLRLTHEARKQFATGKITNLMTTDAEALQQICQSLHTLWSAPVRIVVAMVLLYQQLGVASLLG
ALMLVLMFPLQTFIISRMQKLSKEGLQRTDKRIGLMNEILAAMDTLKYYAWESSFQSKVQIVRDDELSWFRKA
SLLGACNGFILNSIPVFVTVITFGVFTLLGGDLTPARAFTSLSLFSVLRFPLFMLPNTITQVVNANVSLKRLEDLL
LAEERVLLPNPPIEPGLPAISIKNGYFSWDAKAERASLSNINLDIPVGCLVAVVGSTGEGKTSLVSAMLGELPPM
ADSSVVLRGTVAYVPQVSWIFNATVRDNILFGSVFDPARYQRAINVTELQHDLELLPGGDLTEIGERGVNISGG
QKQRVSMARAVYSNSDVYIFDDPLSALDAHVARQVFDKCIKGDLRGKTRVLVTNQLHFLSQVNRIILVHEGM
VKEEGTFEELSNHGPLFQKLMENAGKMEEYEEEKVDTETTDQKPSSKPVANGAINDHAKSGSKPKEGKSVLI
KQEERATGVVSLNVLTRYKSALGGFWVVFVLFACYVSTETLRISSSTWLSHWTDQSATEGYNPVFYNMIYAA
LSFGQVLVTLTNSYWLIISSLYAARRLHEAMLSSILRAPMVFFQTNPLGRVINRFAKDLGDIDRNVAPFVNMFL
GQVSQLLSTFILIGIVSTMSLWAILPLLVLFYVAYLYYQSTAREVKRLDSISRSPVYAQFGEALNGLSTIRAYKA
YDRMADINGKSMDNNIRFTLVNMSGNRWLAIRLETLGGLMIWLTATFAVMQNGRAENQQEFASTMGLLLSY
ALNITSLLTGVLRLASLAENSLNAVERIGTYIDLPSEAPSVIDNNRPPPGWPSLGSIRFEDVVLRYRPELPPVLHG
LSFTIFPSDKVGIVGRTGAGKSSMLNALFRIVELEQGRILIDDYDVAKFGLADLRKVLGIIPQSPVLFSGTVRFNL
DPFNEHNDADLWEALERAHLKDVIRRNSLGLDAEVSEAGENFSVGQRQLLSLSRALLRRSKILVLDEATAAVD
VRTDALIQKTIREEFKSCTMLIIAHRLNTIIDCDRILLLDGGKVLEYDTPEELLSNEGSAFSKMVQSTGAANSQYL
RSLALGGDKSEREENKHLDARRKWLASSRWAAAAQFALAVSLTSSHNDLQRLEVEDENSILKKTKDALITLQ
GVLERKHDKEIEESLEQRQISPDGWWSSLYKMIEGLAIMSRLTVNRFHQSDFGFEDRSINFDQVDM

>XP_003593890.2_ABCC2_Mt
MAFDPLVWYCQPVENGVWTRTVQNAFGAYTPCAVDSLVIGISHLVILGLCIYRIWLIQKDFSVKRFRLRSNVY
NYVLGVLAAYCVAEPLYRLIMGVSVLNLDGQSQLAPFEITSLIIEALAWCSMLILLGIETKVYIYEFRWFVRFGL
IYAAVGGAVLFNFIISVQELYSRSVLYLYISEVVCQVLFGILLLVYVPTLDPYPGYTPIASEIVNDAAYDELPEGE
LICPERRAGLWSKMVFSWMNPIMKLGYERPLTEKDIWKLDTWERTEALQNKFQKCWAEESQKSKPWLLRAL
NASLGGRFWFGGIFKIGNDLSQFTGPLILNQLLQSMQNGDPAGMGYIYAFAIFVGVVFGVLSEAQYFQNVMRV
GYRLRSTLVAAVFRKSLRLTHEARKQFASGKITNLMTTDAESLQQICQSLHTLWSAPFRITIAMVLLYQELGVA
SLLGALLLVLMFPLQTVIISRMQKLSKEGLQRTDKRIGLMNEILAAMDTVKCYAWESSFQSRVVNVRNDELSW
FRKASLLGACNSFILNSIPVFVTVISFGVFTLLGGDLTPARAFTSLSLFAVLRFPLFMLPNIITQVVNANVSLKRLE
ELLLAEERILLPNPPLEPGLPAISIRNGYFSWDAKAERATLSNINLDIPVGSLVAVVGSTGEGKTSLVSAMLGELP
PIADSTVVLRGTVAYVPQVSWIFNATVRDNVLFGSVFDPIRYERAINVTELRHDLELLPGGDLTEIGERGVNISG
GQKQRVSMARAVYSNSDVLVFDDPLSALDAHVARQVFDKCIKGELRGKTRVLVTNQLHFLSQVDRIILVHEG
MVKEEGTFEELSSQGLLFQKLMENAGKMEEYEEEKVDIEATDQKSSSKPVVNGAVNDNAKSESKPKGGKSILI
KQEERETGVVSLNVLIRYKNALGGTWVILVLFACYFSTEALRVSSSTWLSHWTDQSAVDGYNPAFYNLVYAA
LSFGQVFVSLINSYWLIISSLYAARRLHEAMLHSILRAPMVFFHTNPLGRVINRFAKDLGDIDRNVAPFVSMFLG
QISQLLSTFILIGIVSTMSLWAIMPLLVLFYGAYLYYQSTAREVKRLDSISRSPVYAQFGEALNGLSTIRAYKAYD
RMADINGRSMDNNIRYTLVNISANRWLAIRLETLGGLMIWFTATFAVMQNGRAENQQEFASTMGLLLSYALNI
TSLLTGVLRLASLAENSLNSVERVGTYIDLPSEAPSVIDDNRPPPGWPSSGSIKFDEVVLRYRPELPPVLHGLSFTI
FPSDKVGIVGRTGAGKSSMLNALFRIVELEKGRILIDDRDIAKFGLADLRKVLGIIPQSPVLFSGTVRFNLDPFTE
HNDADLWEALERAHLKDVIRRNSLGLDAEVSEAGENFSVGQRQLLSLARALLRRSKILVLDEATAAVDVRTD
ALIQKTIREEFKSCTMLIIAHRLNTIIDCDRVLLLDGGKVLEYNTPEELLSNEGSAFSKMVQSTGAANAQYLRSL

VHGGDKTEREENQHLDGQRKWLASSRWAAAAQYALAVSLTSSQNDLQRLEVEDENSILKKTKDALITLQGVL
ERKHDKEIEESLNQRQISSEGWWSSLYKMIEGLAMMSRLARNRLHQSDFGFEDTSINFDQIDM

>XP_004244532.1_ABCC2_X1_Sl
MAFKPSDWYCQPVANGVWSKAVENAFGAYTPCGTNTLVISVSYLVLLALCLNRVWKMMKDLSVQRFRLRS
NYYNYLLGLLAAYCTAEPLFRLVMQISALNIDGQPGLAPYEIISLTIEVLAWFSILVMTVVETKVYIREGRWSVR
FAVIYCLVGDVVMLNLILPVREYYNESVLYLYISEVAVQVLFGLLLLFYVPDVDPYPGYSPLRSDSFDNTAYEE
LPEGEQICPERHANILSQILFSWMNPLMQLGYKRPLTEKDVWKLDTWDRTETLNNSFQKSWAEESQRPKPWLL
RALNRSLGGRFWWGGFWKIGNDASQFIGPLILNQLLQSMQRGDPAWIGYIYAVAIFIGVVVGVLCEAQYFQNV
MRVGYRLRSTLIAAVFRKSLRLTHESRKNFASGKITNLMTTDSEALQQICQSLHTIWSAPLRIVVALVLLYQLL
GVAALIGALLLVLMFPIQTFVISKMQKLTKEGLQRTDKRIGLMNEVLAAMDTVKSYAWEDSFQSKVQDVRNE
ELSWYRKAQLLGALNSFILNSIPVVVIVISFGVFSLLGGDLTPARAFTSLSLFAVLRFPLFMLPNIITQVVNANVS
LKRLEELLLAEERILLPNPPLEPGLPAISIKNGCFSWDSKAEKPTLSNINLDIPVGSLVAIVGGTGEGKTSLISAML
GEVPAITDSMVVVRGTVAYVPQVSWIFNATVRENILFGSAIDAARYDRAIDVTSLRHDLELLPGGDLTEIGERG
VNISGGQKQRVSMARAVYSDSDVCIFDDPLSALDADVGRQVFERCIKGELKGKTRVLVTNQLHFLSQVDKIIL
VHDGMVKEEGTFEYLSNNGILFQKLMENAGKMEEYTEEKENDDDDNANDKSSKPIVNGETNGVAKEDGKGK
KEGKSVLIKQEERETGVVSSNVLMRYKNALGGSWVVLVLFMCYFLIEALRVGSSTWLSFWTDQSSSTRYSAGF
YNLIYSLLSLGQVMVTLMNSFWLITSSLYAAKMLHDAMLNSILRAPMVFFHTNPLGRIINRFAKDIGDIDRSVA
PFVSMFLGQVFQLISTFVLIGIVSTMSLWAIMPLLVLFYGAYLYYQSTAREVKRLDSISRSPVYAQFGEALNGLA
TIRAYKAYDRMANINGKSVDNNIRFTLVNMSGNRWLAIRLETVGGLMIWLTATFAVMQNGRAENQEAFAST
MGLLLSYALNITSLLTAVLRLASLAENSLNAVERVGTYIELPSEGPSIIEGSRPPPGWPSAGSIQFENVVLRYRPE
LPPVLHGISFTISPSDKVGVVGRTGAGKSSMFNALFRLVELERGRILIDDYDVSKFGLTDLRKVLGIIPQAPVLFS
GTVRFNLDPFNEHNDADLWESLERAHLKDVIRRNSLGLDAEVSEAGENFSVGQRQLLSLARALLRRSKILVLD
EATAAVDVRTDALIQKTIREEFKSCTMLIIAHRLNTIIDCDRILLLDSGQVLEYDTPEVLLEKEESAFSRMVQSTG
AANAEYLRSLVIGGGEGNSVVKDKQLDGKRRWLASSRWSAAAQYAISFTLSSSQNDLVNSEIVDEDNILKKTK
NAVITLQGVLEGKHDKEIEETLEQYQVSRDRWWSSFYRMVEGLSVMSKLTRKRFHPEYRAEDPNIEERTIHWD
RAEM

>XP_006354989.1_ABCC12-l_X1_St
MIFNALVWYCKPVANGIWAKETDSTFGAYTPCAIESADCISNVVLFVLCVYRLWLVRMDHNIRRFQLRSKYY
NYILILLGSCCAAEPLLRLFMGISIFNLDAETDLAPFEMVSLSIEALAWMSIIVMNLVETKVYIKEFRWYVRFGV
MYVLVGELVILNFVFSMQSFYSRFTLYIYYSSVICQIVFGALLLVHLPHLNPFPGYIPLRSESVDDKNDETILGED
HICPERYASILSRISFGWITPLLRQGYDRPITEKDVWKLDSWDKTETLSARFQRCWTEESWRKKPWLLRALNCS
LGGRFWYGGLFKVGSDLCQFVGPLLLNRLLESLERGDPAWIGHLYAFLIFVGVSFGVLCEAQYFQNVMRVGF
RMRSTLVAAIFRKAVRLTLEDRKQFPSGKITNMITTDANALQQVCQQLHVLWSAPFRIVIAMVLLYQQLGLAS
LLGALMLVLMIPMQTIIVSYMRKLSKEGLQYTDKRVGLTNEILAAMDVVKCYAWEKSFQSKVQGLRNGELS
WFRKAQLLAAFNNFMLNSIPVLVTVISFGGFTLLGGNLTASRAFSSLSLFAILRFPLNMLPNIITQVVNSNVSLQR
MEELFLAEERVLLPNPPLEPGLPAISIRGGFFSWDSKPEHPTLSNINLDIPVGELVAIVGGTGEGKTSLISAILGELP
PLGNASVTIRGSIAYVPQVSWIFNATVRENILFGSNFEPTRYWKAIDVTALDHDLELLPGRDLTEIGERGVNISG
GQKQRVSMARAVYKKSDIYIFDDPLSALDAHVSHEVFKNCIKEELQGKTRVLVTNQLHFLPQVDRIILVSEGM
VKEDGTFEKLLEHGTLFPKLMENAGKMESYGVETEYDPNFDSKSSQSSNIRHELQKDLTSVTKRKAGKSVLIR
QEERETGIINWSVLMRYKDALGGLWVVIFLFGCYTLTEILRILSSTWLGVWTKASSSKSNGAGFYILVYAILSFS
QVFVTLANSFWLIISSLNAAKKIHDTMLHSILRAPMVFFHTNPSGRIINRFAKDLGDIDRNVANIGNMCLSQLW
QLLSTFVLIGVVSTISLWAIMPLLILFYAAYLYYQNTSREVKRLDSITRSPVYAQFGEAINGLSTIRAYKAHDQL
ATINGKSMDNNIRFTLANTSTNRWLTIRLETLGGIMIWLTATFAVIQNGRADDKVAVAATMGLLLSYSLNITTL
LSSTLRQASRAENSLNAVERVGTYIDLPSEAQNVISSQPPPDWPSSGFIKFEDVVLRYRPGLPPVLHGLSFEISSG
QKVGIVGRTGAGKSSMLNALFRIVELERGRILIDDCDVANIELTDLRSALSIIPQSPVLFSGTVRFNLDPFNEHND
ADLWEALERAHLKDVISRNTFGLDAEVSEGGENFSVGQRQLLSLARAILRRSKILVLDEATAAVDVRTDALIQR
TIREEFKTCTMLIIAHRLNTIIHTNCILVLDAGKVVEYDTPQNLLLNEGSVFSNIVQSTGAANAQYLRNLVLNKE
RDDMFMEEELMHINGMSR

>XP_006354991.1_ABCC12-l_X2_St
MIFNALVWYCKPVANGIWAKETDSTFGAYTPCAIESADCISNVVLFVLCVYRLWLVRMDHNIRRFQLRSKYY
NYILILLGSCCAAEPLLRLFMGISIFNLDAETDLAPFEMVSLSIEALAWMSIIVMNLVETKVYIKEFRWYVRFGV

MYVLVGELVILNFVFSMQSFYSRFTLYIYYSSVICQIVFGALLLVHLPHLNPFPGYIPLRSESVDDKNDETILGED
HICPERYASILSRISFGWITPLLRQGYDRPITEKDVWKLDSWDKTETLSARFQRCWTEESWRKKPWLLRALNCS
LGGRFWYGGLFKSLERGDPAWIGHLYAFLIFVGVSFGVLCEAQYFQNVMRVGFRMRSTLVAAIFRKAVRLTL
EDRKQFPSGKITNMITTDANALQQVCQQLHVLWSAPFRIVIAMVLLYQQLGLASLLGALMLVLMIPMQTIIVSY
MRKLSKEGLQYTDKRVGLTNEILAAMDVVKCYAWEKSFQSKVQGLRNGELSWFRKAQLLAAFNNFMLNSIP
VLVTVISFGGFTLLGGNLTASRAFSSLSLFAILRFPLNMLPNIITQVVNSNVSLQRMEELFLAEERVLLPNPPLEP
GLPAISIRGGFFSWDSKPEHPTLSNINLDIPVGELVAIVGGTGEGKTSLISAILGELPPLGNASVTIRGSIAYVPQVS
WIFNATVRENILFGSNFEPTRYWKAIDVTALDHDLELLPGRDLTEIGERGVNISGGQKQRVSMARAVYKKSDIY
IFDDPLSALDAHVSHEVFKNCIKEELQGKTRVLVTNQLHFLPQVDRIILVSEGMVKEDGTFEKLLEHGTLFPKL
MENAGKMESYGVETEYDPNFDSKSSQSSNIRHELQKDLTSVTKRKAGKSVLIRQEERETGIINWSVLMRYKDA
LGGLWVVIFLFGCYTLTEILRILSSTWLGVWTKASSSKSNGAGFYILVYAILSFSQVFVTLANSFWLIISSLNAAK
KIHDTMLHSILRAPMVFFHTNPSGRIINRFAKDLGDIDRNVANIGNMCLSQLWQLLSTFVLIGVVSTISLWAIMP
LLILFYAAYLYYQNTSREVKRLDSITRSPVYAQFGEAINGLSTIRAYKAHDQLATINGKSMDNNIRFTLANTSTN
RWLTIRLETLGGIMIWLTATFAVIQNGRADDKVAVAATMGLLLSYSLNITTLLSSTLRQASRAENSLNAVERVG
TYIDLPSEAQNVISSQPPPDWPSSGFIKFEDVVLRYRPGLPPVLHGLSFEISSGQKVGIVGRTGAGKSSMLNALFR
IVELERGRILIDDCDVANIELTDLRSALSIIPQSPVLFSGTVRFNLDPFNEHNDADLWEALERAHLKDVISRNTFG
LDAEVSEGGENFSVGQRQLLSLARAILRRSKILVLDEATAAVDVRTDALIQRTIREEFKTCTMLIIAHRLNTIIHT
NCILVLDAGKVVEYDTPQNLLLNEGSVFSNIVQSTGAANAQYLRNLVLNKERDDMFMEEELMHINGMSR

>XP_006362512.1_ABCC2-l_St
MAFKPLDWYCQPVANGVWSKAVENAFGAYTPCGTNTLVISVSYLVLLALCLNRVWKMMKDLSVQRFCLRS
NYYNYFLGLLAAYCTAEPLFRLVMQISALNLDGQPGLAPYEIISLTIEVLAWFSILVMTVVETKVYIREARWSV
RFAVIYCLVGDVVMLNLIPTVREYYNESVLYLYISEVAVQVLFGLLLLFYVPDVDPYPGYSPLRSESFDNTAYE
ELPEGEQICPERHANILSQILFSWMNPLMQLGYKRPLTEKDVWKLDTWDRTETLNNSFQKSWAEESQRPKPWL
LRALNRSLGGRFWWGGFWKIGNDASQFIGPLILNQLLQSMQRGDPAWIGYIYAVAIFIGVVVGVLCEAQYFQN
VMRVGYRLRSTLIAAVFRKSLRLTHESRKNFASGKITNLMTTDSEALQQICQSLHTIWSAPLRIIVALVLLYQLL
GIAALIGALLLVLMFPIQTFIISKMQKLTKEGLQRTDKRIGLMNEVLAAMDTVKSYAWENSFQSKVQDVRNEE
LSWYRKAQLLGALNSFILNSIPVVVIVISFGVFSLLGGDLTPARAFTSLSLFAVLRFPLFMLPNIITQVVNANVSL
KRLEELLLAEERILLPNPPLEPGLPAISIKNGCFSWESKAEKPTLSNINLDIPVGSLVAIVGGTGEGKTSLISAMLG
EVPAITDSMVVVRGTVAYVPQVSWIFNATVRENILFGSAIDAARYDRAIDVTSLQHDLELLPGGDLTEIGERGV
NISGGQKQRVSMARAVYSDSDVCIFDDPLSALDADVGRQVFERCIKGELKGKTRVLVTNQLHFLSQVDKIILV
HDGMVKEEGTFEYLSNNGILFQKLMENAGKMEEYTEEKENDDDDNDKSSKPVVNGETNGVAKEVGKDKKE
GKSVLIKQEERETGVVSSNVLMRYKNALGGSWVVLVLFMCYFLIEALRVGSSTWLSFWTDQSSSTRYSAGFY
NLIYSLLSLGQVMVTLMNSFWLITSSLYAAKMLHDAMLNSILRAPMVFFHTNPLGRIINRFAKDIGDIDRSVAPF
VSMFLGQVFQLISTFVLIGIVSTMSLWAIMPLLVLFYGAYLYYQSTAREVKRLDSISRSPVYAQFGEALNGLATI
RAYKAYDRMANINGKSVDNNIRFTLVNMSGNRWLAIRLETVGGVMIWLTATFAVMQNGRAENQEAFASTM
GLLLSYALNITSLLTAVLRLASLAENSLNAVERVGTYIELPSEGPSIIEGSRPPPGWPSAGSIQFENVVLRYRPELP
PVLHGISFTISPSDKVGVVGRTGAGKSSMFNALFRLVELERGRILIDGFDVSKFGLTDLRKVLGIIPQAPVLFSGT
VRFNLDPFNEHNDADLWESLERAHLKDVIRRNSLGLDAEVSEAGENFSVGQRQLLSLARALLRRSKILVLDEA
TAAVDVRTDALIQKTIREEFKSCTMLIIAHRLNTIIDCDRILLLDSGQVLEYDTPEVLLEKEGSAFSRMVQSTGA
ANAEYLRSLVIGGGEGNSVAKDKQLDGKRRWLASTRWSAAAQYAIAFTLSSSQNDLVNSEIVDEDNILKKTK
NAVITLQGVLEGKHDKEIEETLDQYQVSRDRWWSSFYRMVEGLSVMSKLTRKRFHPEYRPEDPNIEERTIHWD
RAEM

>XP_006388279.1_ABCC12_X1_Pt
MGLEALVWYCRPMANGVWAKEVDNAFGAYTPCAIDSIVICISHLVLLGLCLYRIWLIIDNNTKVQKYCLRTNY
YNYMLGFLAAYCTVQPLLRLFMDVSIFNLDGQTSLAPFELVSLIVEALAWCSTLIMIGLETKIYIRQFRWYVRF
GVIYVLVGEAAMLNLILSVSDYYSRFTLYMYISTVFCQVLLGILLLVYIPNLDPYPDYVMMESESLDNCEYEAL
PGREQICPERHANLFSRIFFGWLTPLMKQGHKRPITEKDVWKLDTWDQTETLIKKFQTCWVEESKRPKPRLLR
ALNNSLGGRFWLGGFFKIGYDLSQFVGPIVLSHLLQSMQRGDPAWIGYIYAFIIFLGVLFGALCESQFFQNVMR
VGFQLRSTLVAAIFRKSLKLTHEGRKNFPSGKITNMITTDANALQQICQQLHGLWSAPFRITISMVLLYQQLGV
ASLFGSLMLVLMVPTQTILMSKMRKLTKEGLHRTDKRVSLMNEILAAMDAVKCYAWEKSFQSRIQSVRDDEL
SWFRSAQLLSAFNSFILNSIPVIVTLVSFGTFTLLGGDLTPAKAFTSLSLFQVLRSPLNMLPNLLSQVVNANISLQ
RLEELFLAEERILAPNLPLKLGIPAISIENGNFLWDSKLEKPTLSDINLKIQVGSLVAIVGGTGEGKTSLISAMLGE

LPPMEDASVVIRGTVAYVPQVSWIFNATVRDNILFGSEYEPSRYWKAIDVTALQHDLDLLPGHDLTEIGERGVN
ISGGQKQRVSMARAVYSNSDVYIFDDPLSALDAHVGRQVFNSCIKDELQGKTRVLVTNQLHFLPQVDKIILLSE
GMIKEEGTFEELSKNGKLFQKLMENAGKMDELVEEKNSENLDYKSSKPAANRGNDLPQKAGYKMKVKGGKS
VLIKQEERETGVVSWNVLIRYNNALGGIWVVLIIFLCYLLTEVLRVSRSTWLSFWTNQSTLESYRPGYYIFVYA
LLSFGQVIVTLVNSYWLISSSLHAAKRLHDAMLDSILRAPMLFFHTNPSGRIINRFAKDLGEIDRNVANFANNFL
NQAWQLFSTFVLIGIVSTISLWAVMPLLILFYSAYLYYQSTSREVKRLDSITRSPVYAQFGEALNGLSSIRAYKA
YDWMAIINGKSMDNNIRFTLVNMSSNRWLTIRLVTLGGIMIWLIATFAVLGNGRTENHVEFASVMGLLLSYTL
NITDLLSNVLRQASRAENSLNSVERVGTYMDLPSEAPAIVETNRPPPAWPSSGSIKFRDVVLRYRPELPPVLHHL
SFEVSPSEKLGIVGRTGAGKSSMLNALFRIVELERGEITIDGCDVAKFGLTDLRKILSIIPQSPVLFSGTVRFNLDP
FSEHNDADLWEALERAHLKDAIRNNSFGLDAEVFEGGENFSVGQRQLLSLARALLRRSKILVLDEATASVDVR
TDALIQKTIREEFRSCTMLVIAHRLNTIIDCDRILVLEAGQVLEHGTPEELLLPNEGSAFSRMVQSTGPANAQYL
YSLVFESKENKLSKRKNDHRWIDSSRWAAAAQLALVVSLTSSENGLPMLDVGDEDNILRKTKDAVIRLQDVL
VGKYDEAICDTLQQSQVPQDGWWSAFYRMIEGLAVMGRLSRGRHQQYDYENEPLDWDDLKI

>XP_006588591.1_ABCC4_Gm
MSSAAASTWITSFSCSPNATPNLPHWLRFIFLSPCPQRALLSGVDILLLLTLFVFALIKLYSRFTSIGNHNSELDKP
LIRNNRVSNRTTAWFKLTLTTTAVWTILYTVACILVFTSSTDGTWKQTDGFFWLLQAITQLVLAVLIIHEKKFQ
AVVHPLSLRIYWIANFILVSLFTASGVIRLVSVGVEDGKHFSFLVDDTVSFISLPLSLFLLCVAVKGSTGIVSGEE
TQPLIDEETKLYDKSNVTGFASASAISKAFWIWINPLLSKGYKSPLKIDEIPYLSPQHRAERMSVIFESKWPKSDE
RSKHPVRTTLLRCFWREIAFTAFLAVIRLSVMFVGPVLIQSFVDFTAGKGSSVYEGYYLVLILLCAKFVEVLTTH
HFNFNSQKLGMLIRCTLITSLYKKGLRLTGSARQDHGVGPIVNYMAVDSQQLSDMMLQLHAVWMMPFQVGI
GLFLLYNCLGASVITALLGLLAVIVFAVVSTRKNKRYQFNAMMSRDSRMKAVNEMLNYMRVIKFQAWEEHF
NGRILGFRKSEFQWLSKFMYSICGVIIVLWSTPLLISTLTFGTALLLGVRLDAGTVFTTTTVFKILQEPIRTFPQSM
ISLSQALVSLGRLDRYMSSRELMDDSVEREEGCGGHTAVEVKDGTFSWDDDGQLKDLKNINLKINKGELTAIV
GTVGSGKSSLLASILGEMHKISGKVQVCGSTAYVAQTSWIQNGTIEENIIFGLPMNRQKYNEVVRVCSLEKDLE
MMEHGDQTEIGERGINLSGGQKQRIQLARAVYQDSDIYLLDDVFSAVDAHTGTEIFKECVRGALKGKTVILVT
HQVDFLHNVDLIVVMRDGMIVQSGKYDDLLASGMDFSALVAAHDTSMELVEQGAVMTGENLNKPLKSPKA
ASNNREANGESNSLDQPKSGKEGSKLIKEEERETGKVSLHIYKLYCTEAFGWWGIIAVISLSVLWQASMMASD
YWLAYETSEERAQLFNPSMFISIYAIIAVVSVVLIVLRSYSVTVLGLKTAQIFFSQILHSILHAPMSFFDTTPSGRIL
SRASTDQTNVDVFIPLFINFVVAMYITVISIFIITCQNSWPTAFLLIPLAWLNIWYRGYFLASSRELTRLDSITKAP
VIHHFSESISGVMTIRAFRKQKEFCGENIKRVNANLRMDFHNFSSNAWLGFRLELLGSLVFCLSAMFMIMLPSSI
IKPENVGLSLSYGLSLNAVMFWAIYMSCFIENKMVSVERIKQFTNIPSEASWNIKDRLPPANWPGEGHVDIKDL
QVRYRPNTPLVLKGITLSINGGEKIGVVGRTGSGKSTLIQVFFRLVEPTGGKIIIDGIDISALGLHDLRSRFGIIPQE
PVLFEGTVRSNIDPTGQYTDEEIWKSLERCQLKDAVASKPEKLDTSVVDNGDNWSVGQRQLLCLGRVMLKQS
RLLFMDEATASVDSQTDAVIQKIIREDFAARTIISIAHRIPTVMDCDRVLVVDAGRAKEFDSPANLLQRPSLFGA
LVQEYANRSSGL

>XP_010661444.1_P_ABCC15_Vv
MLFEDMFDSKSPNFKQEFQTAWLQLSSPCLWEDVSIVLQLGFLGIFLLHLVQKIVGHLWKHRTTVTDKGIEMY
PNEAKASFSCKASIICSSILLGIHVIVLLMPPNGSEGNCKSPILVLSSEVMQVMIWLITLIAVCKISTKKYVKFPWI
LRTYWLCSFLLSVIHTAFDVHFLVTNNGHLRMQDYTDFLGLLASTCLFGISIRGKTGTVLISQNGLADPLLNGK
TDNHSEGKTESPYGKATLFQLITFSWLNPLFAVGIKKPLAQDEIPDVDVKDSAEFTSHYFDECLKHVRERDGTT
NPSIYKAIFLFIWKKAAINALFAMISAAASYVGPYLIDDFVNFLSMKKTRSLESGYLLALAFLSAKTVETIAQRQ
WIFGARQLGLRLRAALISHIYKKGLVLSSQSRQSHTSGEIINYMGVDIQRMTDFIWYMNTIWMLPIQISLAICVL
NMNIGLGSLAALAATLMVMACNIPLTRIQKRYQSKIMEAKDERMKATSEVLRNIKTLKLQAWDSQFLHKLESL
RKIEYNWLWKSLRLGALSAFIFWGSPTFISVVTFGACLLMGIELTSGRVLSALATFRMLQDPIFNLPDLLSVIAQ
GKVSVDRVASFLQEDEVQSDTIEFVPKDQTEFEVEIDNGKFSWNPDSSSPTLDKIQLKVKRGMKVAICGTVGSG
KSSLLSCILGEIKKLSGTVKIGGTKAYVPQSPWILTGNVKENILFGNRYDSVKYDETVKACALTKDFELFPCGDL
TEIGERGINMSGGQKQRIQIARAVYEDADIYLLDDPFSAVDAHTGTQLFKDCLMGILKNKTILYVTHQVEFLPA
ADFILVMQDGRIAQAGRFEQLLKQNIGFEVLVGAHNQALESILTVENSSRTSKDPVPENESNKDPTSNSEMIHT
QHDSEHNISLEITEKQGRLTQDEEREKGSIGKEVYMSYLTIVRGGALVPIIILAQSMFQVLQVASNYWMAWASP
PTSESRPKMGLDYILFVYILLAVGSSLFVLLRASLVAITGLSTAQKLFVKMLQSVVRAPMAFFDSTPTGRILNRA
SIDQSVLDMEMANRLGWCAFSVIQILGTIAVMSQVAWEVFVIFIPVTAICIWYQQYYIPTARELGRLASIQQSPIL
HHFSESLSGAATIRAFDQEDRFIHANLDLVDNFSRPWFHNVSAMEWLSFRLNVLSNFVFAFSLVLLVSLPEGIIN

PSIAGLAVTYGINLNVLQASVIWNICNAENKMISVERILQYSKIKSEAPLVIEECRPENNWPQVGTICFQNLQIRY
AEHLPSVLKNISCTFPGGMKIGVVGRTGSGKSTLIQAIFRIVEPREGSIIIDGVDISKIGLHDLRSRLSIIPQDPAMFE
GTVRGNLDPLDQHPDGQVWEALDKCQLGDLVRAKEEKLDSSVVENGENWSVGQRQLVCLGRALLKRSSILV
LDEATASVDSATDGVIQKIISQEFKDRTVVTIAHRIHTVIDSDLVLVLSEGRIAEYDTPAKLLERDDSFFSKLIKE
YSKRSKGFGKLAIST

>XP_015158397.1_P_ABCC15_St
MEPQTHLSIRPFTYLEERKQQSMHCLQLLVQDHLNVGPYLIDDFVNFLNKKKIRGLQSGYLLALAFLGAKMVE
TIAQKQWIFGARQLGLRLRGALISHIYQKGLLLSSQSRQSYTSGEIINYMSVDVQRITDFVWYLNTIWMLPIQISL
AIYILHMNLGMGALVALGTTVIVMTGNIPLIRTLKGYQTKIMESKDERMKSTSEILRNMKTIKLQAWDSYYLH
KVEMLRKVEHNWLWKSLRLSALSAFIFWGSPTFISVATFSGCVMMGIPLTAGRVLSALATFRMLQDPIFNLPDL
LSAIAQGKVSADRIASYLQEDEIQPDAVEFVPKDETPFGVEIKSGTFSWDTESRIPTLDGIELQAKKGKKVAICGT
VGSGKSSLLSCVLGEMPKLSGIVKISGEVAYVPQSPWILTGNIKENVLFGKPYESVKYDTTVEACALKKDFELF
PAGDLTEIGERGINMSGGQKQRIQIARAVYQDADIYLLDDPFSAVDAHTGTHLFQECLMRILKGKTILYVTHQV
EFLPAADLILVMQNGRIAQAGTFEELLKQNIGFEVLVGAHNQALESILTVENSSRESEDAVTDGDLDTDSNVNA
EFPHTKQDSEHNLCIEITEKDGRLVQDEEREKGSIGKEIYISYLTIVKGGAFIPIILLAQSSFQLLQIASNYWMAWS
CPTGDAAPIAEKMNFILLVYVLLAVGSSLCVLVRSSFVAITGLRTAEKLFRHMLHSIFRAPMSFFDSTPTGRILNR
ASTDQSVLDLEMANKLGWCAFSIIQLLGTIAVMSQAAWEVFVIFIPVTAICIWYQQYYIPTARELARLSGVQRA
PILHHFAESLAGAATIRAFNQKDRFAHANLCLIDGHSRPWFYNASAMEWLSFRLNQLANFVFAFFLVLLVTLPE
GIINPSIAGLAVTYGINLNVLQASVIWNICNAENKMISVERILQYSNLASEAPLVIENSRPSSTWPETGTISFQNLQ
IRYAEHLPSVLKNITCTLPGSKKVGVVGRTGSGKSTLIQALFRIVEPQEGSIIIDDVDICKIGLYDLRSRLSIIPQDP
TMFEGTVRGNLDPLAQHSDTEIWEALDKCQLGDIIRAKPEKLESTVVENGENWSVGQRQLFCLGRALLKKSSI
LVLDEATASVDAATDAVLQKIISQEFRNRTVVTIAHRIHTVIDSDLVLVLNEGRIAEYDTPAKLLEREDSFFSKLI
KEYSMRSKSFNSLAILQT

>XP_021592546.1_ABCC10_Me
MEDLWKMFCGKSGSSESGGKPDGSFFELLSQPSCANHILIICLDFLLLLMLLFNLIQKSTSKTCQIPPRFRGFSCL
QISSAIFNGCLGLVYLCLGIWILEEKLRETHTALPLNRWFLVLFQGITWLSVGLTISLRGKHLPRTPSRLLSVLAF
LFAGIICVLSLYAAVLGKSISVKGGLDVLSFPGAILLLFSLYKGRNEEEIDESEAGLYAPLNGHEANDVSKTDFV
VPVTPFAKAGFFSGMSFWWLNSLMKKGREKTLVDDDMPKLRQADRAESCYLLFLEQLNKQKQAESSSQPSLL
WTIISCHWKEILISGFFALLKIITLSAGPLLLNAFILVAEGKGSFKYEGYILALTLFISKNLESLSQRQWYFRSRLIG
LKVRSLLTAAVYKKQLRLSNAGRLIHTGGEIMNYVTVDAYRIGEFPFWFHQTWTTSLQLCFSLVILFNAVGLA
TIAALVVIIITVLCNTPLAKLQHEFQSKLMVAQDERLRACTESLVNMKVLKLYAWETHFKNVIENLRKEEYKW
LSAVQLRKAYNGFLFWSSPVLVSAATFGACYFLKIPLHANNVFTFVATLRLVQDPIRAIPDVIGVVIQANVAFSR
IVKFLEAPELQSGNVRQRQKQKRNMENHAISIKGANFSWEENSAKPTLRNVNLEIRPGEKVAVCGEVGSGKST
LLAAILGEVPNTQGTIQVSGRIAYVSQTAWIQTGTIQENILFGSALDSQRYQDTLERCSLVKDLELLPYGDHTEI
GERGVNLSGGQKQRIQLARALYQDADIYLLDDPFSAVDAHTATSLFNEYVMGALARKAVLLVTHQVDFLPAF
DSVLLMSDGEIVQAAPYHQLLASSHEFQDLVNAHKETAGSERLTEIATPQKRGSSAMEIKKTCEENQLKVSKG
DQLIKQEEREVGDTGLKPYIQYLNQNKGYLYFSLAALSHLTFVIGQISQNSWMAANVDKPNVSPLWLIAVYLII
GIVSTLFLLSRSLSTVILGLESSKSLFSQLLNSLFRAPMSFYDSTPLGRILSRVSSDLSIVDLDVPFGLIFAVGATTN
AYANLGVLAVVTWQVLFVSIPMLYLAIRLQRYYFSSAKELMRINGTTKSLVANHLAESVAGAMTIRAFEEEER
FFAKNLQLIDTNASPFFHSFAANEWLIQRLETLSATVLASAALCMVLLPPGTFSSGFIGMAISYGLSLNMSLVFSI
QNQCTIANYIISVERLNQYMHVPSEAPEVIEDNRPPPNWPAIGRVDICDLQIRYRPDTPLVLRGISCTFVGGHKIG
IVGRTGSGKTTLIGALFRLVEPARGKIIVDGIDISKIGLHDLRSRFGIIPQDPTLFNGTVRYNLDPLSQHSDHEIWE
VLGKCQLQEAVQEKEQGLDSLVVEDGSNWSMGQRQLFCLGRALLRRSRILVLDEATASIDNATDLILQKTIRT
EFEDCTVITVAHRIPTVMDCTMVLSISDGKLVEYDEPMKLMKRESSLFGQLVKEYWSHYHSAESH

>XP_021611523.1_ABCC4-l_Me
MSSPPWITSLSCSSSVVQSSGDTSLPLIFQWLRFIFLSPCPQRALLSSVDLLFLLILLVFAVQKLFSRFTSNAHSTSD
IHKPLIGNNRVHATTTIWFKLCLITTILLAFGYTVICILAFSGSKQLPWKLVDGFFWLVHAITHAVIAILIIHEKRF
QAVTHPRTLRSYWVANFIIVTLFMSSGIIRLVAQETSLIVDDIVSIVSFPLCLILLSVAIGGSTGVTVNGESERVKD
DDETTLYEPLLGKSNVSAFASASHISKAFWLWMNPLLSKGYKSTLKLDDVPTLSPQHRAEKMSQLFASNWPKP
QEKCKHPVRTTLLRCFWKEIAFTAFLAIVRLCVMYVGPILIQSFVDFTSGKRSSPYEGYYLVLTLLAAKFVEVLS
VHQFNFNSQKLGMLIRSTLITSLYKKGLRLSCSARQAHGVGQIVNYMAVDAQQLSDMMLQHSIWLMPLQVG

VALVLLYNALGVSVIAALVGIIGVVIFIVFGTRRNNRFQFNLMTNRDSRMKATNEMLNYMRVIKFQAWEEHFN
KRIQNFRESEYGWLSKFMYSISGNIIMMWCTPLLISTVTFGVALLLGVPLDAGTVFTTTSIFKILQEPIRTFPQSMI
SLSQAMISLGRLDKYMLSKELAEQSVERMEGCGGRIAVEVKDGAFSWDDESEDQVLKNINLEIKKGELTSIVG
TVGSGKSSLLASILGEMHKIQGQVRVCGTTAYVAQTSWIQNGTIQANILFGLPMDKEKYNEVIRVCCLEKDLE
MMDYGDQTEIGERGINLSGGQKQRIQLARAVYQDCDIYLLDDVFSAVDAHTGSDIFRECVRGALKGKTILLVT
HQVDFLHNVDLIMVMRDGMIVQSGKYNDLMESGMDFGALVAAHETAMELVEEAGATMPGENSPKPPKPPH
APSNVEEANGENKNQDQPRVKGSSKLIEEEERETGKVGLHVYKQYCTAAFGWWGVTAAFLLSILWQASIMAG
DYWLAYETSEERSRVFDPSAFISVYAIIAAISLVLLTMRAFFITIMGLKTAQIFFWGILNSILHAPMSFFDTTPSGRI
LSRASTDQANVDLFIPFVLGLTVAMYITLLSIIIITCQYAWPTVFLLIPLGWLNIWYRGYFLSTSRELTRLDSITKA
PIIHHFSESISGVMTIRSFNKQEKFCQENVNRVNENLRMDFHNNGSNEWLGFRLELIGSFILCISAMFLILLPSSIIR
PENVGLSLSYGLSLNGVLFWAIYMSCFVENRMVSVERIKQFTNIPSEAAWKITDRVPPPSWPAHGNVDLKDLQ
VKYRPNTPLVLKGITLSIYGGEKIGVVGRTGSGKSTLIQVFFRLVEPTGGKIIIDGIDICRLGLHDLRSRFGIIPQEP
VLFEGTVRSNIDPIGQHTDEEIWKSLERCQLKDIVAAKLEKLDAPVVDNGDNWSVGQRQLLCLGRVMLKRSRL
LFMDEATASVDSKTDGVIQKIIREDFAACTIISIAHRIPTVMDCDRVLVIDAGRAKEFDKPSRLLERPSLFGALVQ
EYANRSAGL

>XP_021612770.1_P_ABCC15_X1_Me
MRAELKLLIKMAWPELNSPCLWEHVNIPIHLGFLGILLVLFLRKCVGIACQRRRSIGPDQSMHKYSIGVKFSTAY
KATIVCSILLLGVHFLGLLMLLNGQENRCNSKLFAFSSQIMQLGSSAITLIVVYRILHDKYAKFPWLLRVWWFC
SFLQSIICASLDTYFSITNHGRLRVRDYADFLGVLSSTFLLGVSIHGKTGIVFNSSTAITEPLLHEKTSKQLEGRSE
SPYGKANLLQLITFSWLNPLFATGIKKPLEQDEIPDVDTKDSAEYLSPVFQECLNQVKEKDAATNPSIYKTIFFFV
RKKAAINALFAVTSAAASYVGPYLIDDFVNFLTERGTESLGRGYLLALAFLCAKMIETIAQRQWIFGARQLGLR
LRAALISHIYKKGLLLSSQSRQTHSSGEIINYMSVDIQRITDFMWYVNIIWMLPIQISLAIYILKTNLGLGSLAALA
ATLMVMFCNIPITRIQKGFQSQIMEAKDNRMKATSEVLRNMKILKLQAWDSQFLRKVESLRTIEYNCLWKSLR
LSAISAFVFWGSPTFISVVTFGSCMLMGIQLTAGRVLSALATFRMLQDPIFNLPDLLSVIAQGKVSADRVASYLQ
EGEIQHDAIKYVPKDQTEIDVEIDAGKFSWDPESSTPTLDGIQLKVKRGMKVAICGTVGSGKSSLLSCILGEIQK
QSGTVKISGTKAYVPQSPWILTGNIRENILFGNPYDSAKYYSTVKACALTKDFELFSCGDLTEIGERGINMSGGQ
KQRIQIARAVYQDADIYLLDDPFSAVDAHTGTQLFRECLMGILKDKTILYVTHQVEFLPAADLILVMQNGRIAQ
AGTFDELLNQNTGFGILVGAHSQALESVLKVENSKRMSQTPASDAKSNTDSTSNAELSSTRHDSDHDLSAEIKE
KGGKLVQDEEREKGSIGKEVYWSYLTTVKHGALVPVILLAQSSFQVLQIASNYWMAWASPPTSESEPVIGMKII
LLVYIVLAVGSSFCVLIRAMLVAVVGLSTAQKLFTNMLHSVFRSPMAFFDSTPTGRILNRASTDQSVLDLEIAM
RLGWCAFSVIQIIGTIAVMSQVAWEVFVIFIPVTAICMWYQRYYIPTARELARLAGIQRAPILHHFAESLAGAATI
RAFDQEDRFIEANLDLIDSHSRPWFHNVSAMEWLSFRLNLLSNFVFAFSLVLLVTLPEGVINPSIAGLAVTYGIN
LNVLQATVIWNICNAENKMISVERILQYSNLTSEAPHVVEDCRPSNNWPVIGTICFRDLEIRYAEHLPSVLKNIN
CTFPGRKKVGVVGRTGSGKSTLIQAIFRIVEAKEGSILIDNVDISKIGLQDLRSRLSIIPQDPTMFEGTVRGNLDPL
EQYSDHEVWEALAKSQLGDLVRSKIEKLDAPVVENGENWSVGQRQLLCLGRALLKKSSILVLDEATASVDSA
TDAVIQKIISQEFKDRTVVTIAHRIHTVIDSDLVLVLSDGRIAEYDTPARLLEREDSFFSKLIKEYSMRSQSFNSLA
NVVAD

>XP_021612771.1_P_ABCC15_X2_Me
MAWPELNSPCLWEHVNIPIHLGFLGILLVLFLRKCVGIACQRRRSIGPDQSMHKYSIGVKFSTAYKATIVCSILLL
GVHFLGLLMLLNGQENRCNSKLFAFSSQIMQLGSSAITLIVVYRILHDKYAKFPWLLRVWWFCSFLQSIICASL
DTYFSITNHGRLRVRDYADFLGVLSSTFLLGVSIHGKTGIVFNSSTAITEPLLHEKTSKQLEGRSESPYGKANLLQ
LITFSWLNPLFATGIKKPLEQDEIPDVDTKDSAEYLSPVFQECLNQVKEKDAATNPSIYKTIFFFVRKKAAINALF
AVTSAAASYVGPYLIDDFVNFLTERGTESLGRGYLLALAFLCAKMIETIAQRQWIFGARQLGLRLRAALISHIY
KKGLLLSSQSRQTHSSGEIINYMSVDIQRITDFMWYVNIIWMLPIQISLAIYILKTNLGLGSLAALAATLMVMFC
NIPITRIQKGFQSQIMEAKDNRMKATSEVLRNMKILKLQAWDSQFLRKVESLRTIEYNCLWKSLRLSAISAFVF
WGSPTFISVVTFGSCMLMGIQLTAGRVLSALATFRMLQDPIFNLPDLLSVIAQGKVSADRVASYLQEGEIQHDA
IKYVPKDQTEIDVEIDAGKFSWDPESSTPTLDGIQLKVKRGMKVAICGTVGSGKSSLLSCILGEIQKQSGTVKISG
TKAYVPQSPWILTGNIRENILFGNPYDSAKYYSTVKACALTKDFELFSCGDLTEIGERGINMSGGQKQRIQIARA
VYQDADIYLLDDPFSAVDAHTGTQLFRECLMGILKDKTILYVTHQVEFLPAADLILVMQNGRIAQAGTFDELL
NQNTGFGILVGAHSQALESVLKVENSKRMSQTPASDAKSNTDSTSNAELSSTRHDSDHDLSAEIKEKGGKLVQ
DEEREKGSIGKEVYWSYLTTVKHGALVPVILLAQSSFQVLQIASNYWMAWASPPTSESEPVIGMKIILLVYIVL
AVGSSFCVLIRAMLVAVVGLSTAQKLFTNMLHSVFRSPMAFFDSTPTGRILNRASTDQSVLDLEIAMRLGWCA

FSVIQIIGTIAVMSQVAWEVFVIFIPVTAICMWYQRYYIPTARELARLAGIQRAPILHHFAESLAGAATIRAFDQE
DRFIEANLDLIDSHSRPWFHNVSAMEWLSFRLNLLSNFVFAFSLVLLVTLPEGVINPSIAGLAVTYGINLNVLQA
TVIWNICNAENKMISVERILQYSNLTSEAPHVVEDCRPSNNWPVIGTICFRDLEIRYAEHLPSVLKNINCTFPGRK
KVGVVGRTGSGKSTLIQAIFRIVEAKEGSILIDNVDISKIGLQDLRSRLSIIPQDPTMFEGTVRGNLDPLEQYSDHE
VWEALAKSQLGDLVRSKIEKLDAPVVENGENWSVGQRQLLCLGRALLKKSSILVLDEATASVDSATDAVIQKI
ISQEFKDRTVVTIAHRIHTVIDSDLVLVLSDGRIAEYDTPARLLEREDSFFSKLIKEYSMRSQSFNSLANVVAD

>XP_021615716.1_ABCC12-l_Me
MALEPLAWYCRPARNGVWAKEADSAFGAYTPCAIDSLVICISHLVLLGLCFYRIWLIKKNSKAEKYCLRTNYY
NYMLALLAGLCTAEPLFRLVMGISIFNLDGQISLAPFEIVSLFIEAFAWFSMLLMVGLETKIYIRQFRWYVRFGVI
YLLVGEAAMLNIIFSMSDYYSRFVLYTHISAVFCQVLFGLLLLVYIPNLDPYPGYTILQPDCPENGEYEVLPGAE
QTCPERHVNLLSRIYFGWMTPLMQQGYRKPITENDVWKLDTWDQTETLIKNFQKCWVEESQKPKPRLLRALN
NSLGKRFWLGGVFKIGNDLSQFVGPVLLNHLLKSMQRGDPAWIGYVYAFSILLGVSIGVLCESQYFQNVMRV
GFRLRSTLVAAIFRKSLRLTHESRKNFPSGKITNMITTDANALQQICQQLHGLWSAPFRITLSMVLLYQQLGVAS
LLGSLILVLMVPVQTFVISKMRKLTKEGLQRTDKRVSLMNEILAAMDTVKCYAWEKSFQSKVQSIRNDELSWF
RNAQLLSAFNSFILNSIPVVVTLVSFGTFTLLGGDLTPARAFTSLSLFQVLRFPLNMLPNLLSQVVNANISLQRLE
ELFLAEERILVPNPPLEPGLPAISIKDGCFSWDSKAEKPTLSNINLDIPVGSLVAIVGGTGEGKTSLISAMLGELPS
LANTSVIVRGTVAYVPQVSWIFNATVRDNILFGSEFQPTRYWQAIDVTALHHDLDLLPGRDLTEIGERGVNISG
GQKQRVSMARAVYSNSDVYIFDDPLSALDAHVGRKVFNSCIKEALRGKTRVLVTNQLHFLPQVDRIILVSEGTI
KEEGTFEELSKSGKLFQKLMENAGKMEEMEQAAEGKEDSTRLDLKNSQPATDELNEFSQNGGYTKKGKGRKS
VLVKQEERETGVVSWKVLMRYNNSLGGTWVVMILFVFYLSTEVLRVSSSSWLSFWTDQSTTEGYRPGFYIFV
YALLSLGQVTVTLLNSFWLISSSLRAAKRLHDSMLDSILRAPMLFFHTNPTGRIINRFAKDLGEIDRNVAGFAN
MFLNQVWQLLSTFVLIGIVSTISLWAIMPLLILFYAAYLYYQTTSREVKRLDSITRSPVYAQFGEALNGLSSIRAY
KAYDRMANISGKSMDNNIRFTLVNISSNRWLTIRLESLGGIMIWLTATFAVLQNSRTDNQVAFASTMGLLLSYT
LNITNLLSGVLRQASRAENSFNSVERVGTYIDLPSEAPTIIESNRPPSAWPASGSIKFRDIVLRYRPELPPVLHGLS
FAVSPSEKLGIVGRTGAGKSSMFNALFRIVELERGEIIIDGCDVSKFGLTDLRKALSIIPQSPVLFSGTVRFNLDPF
SEHNDADLWEALERAHLKEVIRKNPFGLDAEVAEGGENFSVGQRQLLSLARALLRRSKILVLDEATAAVDVRT
DALIQKTIREEFKSCTMLVIAHRLNTIIDCDRILVLDAGQVLEHATPEELLQNEGSAFSRMVQSTGPANAQYLRS
LVFEAKEKLNGEVINRLNHQRRWVASSRWAAAAQFALAVSLSQNDLQSLVSAEENNILNKTKDAVITLQQVL
EGKHNEEIDDTLQQYQVPRERWWLSLHRIIEGLSIMSRLAYNRLQELEFDHENESLDWDNIGS

>XP_021616215.1_ABCC2-l_Me
MAFGPLVWYCRPVANGLWTRAVENAFGAYTPCATDTLVVVISHLALLALCLYRIWLIKKDFKVQRFRLRSKW
YNYFLGLLSGYSTAEPLFRLIMGISVLNINGQTGLAPYEIVSLTIEALAWCSVFVMIGVETKVYIRDFRWFVRFG
VLYTLVGDAVILNLILTVKEFYNSSVLYLYISEVFVQVLFGILLLVYVPDLDPYPGYTPIRAESVEDAEYEELPG
GEYICPEQHVNIFSKTIFAWMNPIMKLGYKRPLTEKDIWKLDTWDRTETLNNRFQKCWAEESQRPKPWLLRAL
NSSLGGRFWWGGFWKIGNDVSQFVGPLLLNQLLQSMQEGDPAWIGYIYAFSIFAGVVFGVLFEAQYFQNVMR
VGYRLRSTLIAAVFRKSLRLTHESRQKFASGKITNLMTTDAEALQQICQSLHTLWSAPFRIIIAMVLLFQQLGVA
SLLGALLLVLLFPIQTFVISRMQKLSKEGLQRTDKRIGLMNEILAAMDTVKCYAWENSFQAKVQTVRDDELSW
FRKASLLGACNGFILNSIPVVVTVISFGMFTLLGGDLTPARAFTSLSLFAVLRFPLFMLPNIITQVVNANVSLKRL
EELLLAEERILLPNPPLEPGQPAISIKNGYFSWDSKAERPTLSNINVDIPIGSLVAIVGSTGEGKTSLISAMLGELPA
ISDTSAVIRGSVAYVPQVSWIFNATVRDNILFGSPFDHARYERAIDVTSLQHDLDLLPGGDLTEIGERGVNISGG
QKQRVSMARAVYSNSDVYIFDDPLSALDAHVGQQVFDKCIKGELSRKTRVLVTNQLHFLSQVDRIILVHEGMV
KEEGTFEELSNNGVLFQKLMENAGKMEEYVEDKENGETVDLKPSSKPVANGEMNDLPKNATETKKRKEGKSI
LIKQEERETGVVSWKVLMRYKNALGGAWVVMILFMCYVMTEVLRVSSSTWLSNWTDQGTTKIHGPIYYNLI
YSLLSIGQVLVTLLNSYWLIISSLYAARRLHDAMLNSILRAPMVFFHTNPLGRIINRFAKDLGDIDRNVAVFVNM
FLGQVSQLLSTFVLIGIVSTMSLWAIMPLLVLFYGAYLYYQSTAREVKRMDSISRSPVYAQFGEALNGLSTIRA
YKAYDRMADINGRSMDNNIRFTLVNMSANRWLAIRLETLGGVMIWLTATFAVMQNGRAENQQAFASTMGLL
LSYALNITGLLTAVLRLASLAENSLNAVERVGTYIDLPSEAPSIVEGNRPPPGWPSSGSIKFEDVVLRYRPELPPV
LHGLSFVVSPSDKVGIVGRTGAGKSSMLNALFRIVELERGRILIDGCDIAKFGLMDLRKVLGIIPQSPVLFSGTVR
FNLDPFDEHNDADLWEALERAHLKDVIRRNSLGLDAQVSESGENFSVGQRQLLSLARALLRRSKILVLDEATA
AVDVRTDALIQKTIREEFKSCTMLIIAHRLNTIIDCDRILLLDSGQVLEYDTPEELLSNEASAFSKMVQSTGAAN
AQYLRSLVLGEEGENRFRTQEKEQLSGQKKWLASSRWAAAAQFALAVSLTSSHNDLQRLEIEDEDSILKKTKD
AVVTLQGVLEGKHDKVIDESLNKYQISRDGWWSALYKMVEGLAMMSRLGRNRLHQSESFEDRSIDWDHVEM

>XP_021621186.1_ABCC4-l_Me
MSSSPWITSLSCSSSVIQSSGEASLPLIFQWLRFIFLSPCPQRALLSSVDILFLLVLLVFLCQKLFSRFTFSGHSSSDI
DKPLIGNNRVHIMTTIWFKLSLIATALLAFGYTVICILALSGSTQFPWKLVAGLFWLVQAITHAAIAILIIHEKRF
QAVTHPFSLRIYWVANFIIITLFTASGIIRLVAQETNLLVDDIVSVISFPLSILLLSVAIEGSTGITVKRETEQVIDDA
ETKLCETLSGKSNVSAFATASHISKAFWLWMNPLLSKGYKSPLKLADVPTLSPDHRAEKMSLLFASNWPKPHE
QSKHPVRTTLLRCFRKEIAFTASLAIVRLCVMYVGPILIQSFVDFTAGKRTSPYEGYYLVLTLLAAKFVEVLSLH
QFNFNSQKLGMLIRSTLITSLYKKGLRLSCSARQAHGVGQIVNYMAVDAQQLSDMMLQLHSIWLMPLQVAVA
LVLLYNALGVSVIAALIGIIGVIVFVVFGTRRNNRFQFNVMINRDSRMKATNEMLNYMRVIKFQAWEEHFNKRI
LNFRESEFGWLSKFMYSVSGNIIVMWCTPLLISSVTFGVALLLGVPLDAGTVFTTTSIFKILQEPIRNFPQSMISLS
QAMVSLDRLDRYMLSKELEEKSVERVEGCDGRIAVEIKDACFNWDDENDDLVLKKINLEIKKGELTSIVGTVG
SGKSSLLASILGEMHKISGKVTVCGTTAYVAQTSWIQNGTIQENILFGLPMDKEKYNEVIRVCCLEKDLEMMD
YGDRTEIGERGINLSGGQKQRIQLARAVYQDCDIYLLDDVFSAVDAHTGSDIFKECVRGALKGKTILLVTHQV
DFLHNVDLIMVMRDGMIVQAGKYNDLMESGMDFGALVAAHETAMELVEEAGATIPGESSPNPPKSPQAPSSV
GESNSENKHLDQPKSDKGTSKLIEEEERETGKVGLHVYKQYCTAAFGWWGIVAVLFLSIIWQASLMAGDYWL
AYETSEERSRVFDPSLFISIYTIIAAVSVVLLTVRSFFVTIMGLQTAQIFFTGILNSILHAPMSFFDTTPSGRILSRAS
TDQSNVDLFIPLILGVTVAMYITLLSIIIITCQYAWPTVILLIPLCWLNIWYRGYFLATSRELTRLDSITKAPIIHHFS
ESISGVMTIRSYNKQESFCKENVNRVNANLRMDFHNNGSNEWLGFRLELIGSFILCLSALFLTVLPSSIIRPENVG
LSLSYGLSLNGVLFWAIYMSCFVENRMVSVERIKQFTNIPSEAAWKIQDQIPPPSWPAHGNVDLKDLQVKYRP
NTPLVLKGITLSFYGGEKIGIVGRTGSGKSTLIQVFFRLVEPTGGKIIIDGIDICRLGLHDLRSRFGIIPQEPVLFEGT
VRSNIDPIGQHTDEDIWKSLERCQLKDVVAAKPEKLDAPVVDNGDNWSVGQRQLLCLGRVMLKRSRLLFMDE
ATASVDSKTDGVIQKIIREDFAACTIISIAHRIPTVMDCDRVLVIDAGRAKEFDKPSRLLERPSLFGALVQEYANR
SAGL

>XP_024437176.1_ABCC12_X1_Pt
MGLEALVWYCRPVPNGVWATKVDNAFGAYTPCVVDSLVICISHLVLLGLCLYRIWLITDKNSKAQHYCLRTN
YYNYSLGLLAAYCTVQPLFRLFMNVSIFNLDGQTALAPFEMVSLIVEALSWCSTLIMIGLETRIYIQQFRWYVRF
GVIYVLVGEAAMLNLILSVSDNYDSRFIFYMYLSTVFCQVLFGIHLLVYIPNLDPCSDYVMMEPESPDNSAYEA
LPGREQICPERNATLFSRIFYWWLTPLMKQAHKRPISEKDVWKLDTWDQTETSMNNSCRFQTCWVEESQRPKP
CLLRALNNSLGGRFWLGGFFKIGYDLSEFVGPVVFSHLLQVHSFIGINLFYSMQRGDPAWIGYVVYAFVIFLGML
FSALCESRYYQNVLRVGFRLRSTLVAGIFRKSLKLTHEGQKNFPSGKITNMITTDADVLQQICLLLHGLWSAPF
CITMSMVLLYQQLGVASLFGSLVLVIMVPTQAILLNRMTRLTKEGLHRTDKRVSLMNEILAAMDTVKCYAWE
KSFQFRVQSVRNDELSLFRSAQLLFAFNSFMVNSIPVVVTLVSFGTFTLLGGDLTPAKAFTSLSLFQVLRYPLNM
LPNLLSQVVNANISLQRLEELFLAEERILAPNPPLEPGIPAISIENGNFSWDLKLENPTLTNIKLNIQVGSLVAIVG
GTGEGKTSLISAMLGELPPMEDACVVIRGTVAYAPQVPWIFNATVRDNILFGSKYEPSRYGKAIDVTALQHDL
DLFAGHDLTEIGERGVNISGGQKQRISMARAFYSNSDIYIFDDPLSALDAHVARQVFNSCIKEGLQGKTRVLVT
NQLHFLPQVEKIILLSEGMIKEEGTFEELFKNSELFQKLMENAGKMEEQVKEKEKSDNLDHKSSKAEANWENE
LPQKAASTMKGKEGKSILIKQEERERGVVSWNVLIRYNNALGGVWVVSILFLCYLLTEVFRVSRSTWLSFWTN
QSTLESYRPGYFIFVYGLLSFGQVTVTLANSYWLISSSLHASKRLHDAMLDSILRTPMLFFHTNPTGRIINRFAK
DVGEIDRNVANSANNFLNLAWQLLSTFVLIGTVSTISLWAIMPLLILFYSAYLYYQNTSREVKRLDSITRSPVYA
QFGEALNGLSSIRAYKAYDWMSIINGKYMDNNIRFSLVTISSDGWLAIRLVTLGGMMIWLIASFSVLGNGRTEN
HVGFASIMGLLLSYTSNITDLLSNVLRQASKAENSLNSVERVSTYIDLPSEAPAIDKNNRPPSSWPLSGLIKFTDV
VLRYRPELPPVLHGLSFAVSPSEKLGIVGRTGAGKSSMLNALFRIVELERGEITIDGCDITKFGLTDLRRALSIIPQ
SPVLFSGTVRFNLDPFSEHNDADLWKALERAHLKDAVRNSSFGLDAQVFGGESFSVGQRQLLSLARALLRRSK
ILVLDEATSSVDVRIDALIQKTIREEFRSCTMLIIAHRLNTIIDCDRILVLEAGQVLEHSTPEELLSNEGSAFSRMV
QSTGPANAQYLHSLVFESKENKLMFIHS

>XP_024437177.1_ABCC12_X2_Pt
MGLEALVWYCRPVPNGVWATKVDNAFGAYTPCVVDSLVICISHLVLLGLCLYRIWLITDKNSKAQHYCLRTN
YYNYSLGLLAAYCTVQPLFRLFMNVSIFNLDGQTALAPFEMVSLIVEALSWCSTLIMIGLETRIYIQQFRWYVRF
GVIYVLVGEAAMLNLILSVSDNYDSRFIFYMYLSTVFCQVLFGIHLLVYIPNLDPCSDYVMMEPESPDNSAYEA
LPGREQICPERNATLFSRIFYWWLTPLMKQAHKRPISEKDVWKLDTWDQTETSMNNSCRFQTCWVEESQRPKP
CLLRALNNSLGGRFWLGGFFKIGYDLSEFVGPVVFSHLLQSMQRGDPAWIGYVVYAFVIFLGMLFSALCESRYY
QNVLRVGFRLRSTLVAGIFRKSLKLTHEGQKNFPSGKITNMITTDADVLQQICLLLHGLWSAPFCITMSMVLLY

QQLGVASLFGSLVLVIMVPTQAILLNRMTRLTKEGLHRTDKRVSLMNEILAAMDTVKCYAWEKSFQFRVQSV
RNDELSLFRSAQLLFAFNSFMVNSIPVVVTLVSFGTFTLLGGDLTPAKAFTSLSLFQVLRYPLNMLPNLLSQVVN
ANISLQRLEELFLAEERILAPNPPLEPGIPAISIENGNFSWDLKLENPTLTNIKLNIQVGSLVAIVGGTGEGKTSLIS
AMLGELPPMEDACVVIRGTVAYAPQVPWIFNATVRDNILFGSKYEPSRYGKAIDVTALQHDLDLFAGHDLTEI
GERGVNISGGQKQRISMARAFYSNSDIYIFDDPLSALDAHVARQVFNSCIKEGLQGKTRVLVTNQLHFLPQVEK
IILLSEGMIKEEGTFEELFKNSELFQKLMENAGKMEEQVKEKEKSDNLDHKSSKAEANWENELPQKAASTMKG
KEGKSILIKQEERERGVVSWNVLIRYNNALGGVWVVSILFLCYLLTEVFRVSRSTWLSFWTNQSTLESYRPGYF
IFVYGLLSFGQVTVTLANSYWLISSSLHASKRLHDAMLDSILRTPMLFFHTNPTGRIINRFAKDVGEIDRNVANS
ANNFLNLAWQLLSTFVLIGTVSTISLWAIMPLLILFYSAYLYYQNTSREVKRLDSITRSPVYAQFGEALNGLSSIR
AYKAYDWMSIINGKYMDNNIRFSLVTISSDGWLAIRLVTLGGMMIWLIASFSVLGNGRTENHVGFASIMGLLL
SYTSNITDLLSNVLRQASKAENSLNSVERVSTYIDLPSEAPAIDKNNRPPSSWPLSGLIKFTDVVLRYRPELPPVL
HGLSFAVSPSEKLGIVGRTGAGKSSMLNALFRIVELERGEITIDGCDITKFGLTDLRRALSIIPQSPVLFSGTVRFN
LDPFSEHNDADLWKALERAHLKDAVRNSSFGLDAQVFGGESFSVGQRQLLSLARALLRRSKILVLDEATSSVD
VRIDALIQKTIREEFRSCTMLIIAHRLNTIIDCDRILVLEAGQVLEHSTPEELLSNEGSAFSRMVQSTGPANAQYL
HSLVFESKENKLMFIHS

>XP_024437178.1_ABCC12_X3_Pt
MGLEALVWYCRPVPNGVWATKVDNAFGAYTPCVVDSLVICISHLVLLGLCLYRIWLITDKNSKAQHYCLRTN
YYNYSLGLLAAYCTVQPLFRLFMNVSIFNLDGQTALAPFEMVSLIVEALSWCSTLIMIGLETRIYIQQFRWYVRF
GVIYVLVGEAAMLNLILSVSDNYDSRFIFYMYLSTVFCQVLFGIHLLVYIPNLDPCSDYVMMEPESPDNSAYEA
LPGREQICPERNATLFSRIFYWWLTPLMKQAHKRPISEKDVWKLDTWDQTETSMNKFQTCWVEESQRPKPCLL
RALNNSLGGRFWLGGFFKIGYDLSEFVGPVVFSHLLQSMQRGDPAWIGYVYAFVIFLGMLFSALCESRYYQNV
LRVGFRLRSTLVAGIFRKSLKLTHEGQKNFPSGKITNMITTDADVLQQICLLLHGLWSAPFCITMSMVLLYQQL
GVASLFGSLVLVIMVPTQAILLNRMTRLTKEGLHRTDKRVSLMNEILAAMDTVKCYAWEKSFQFRVQSVRND
ELSLFRSAQLLFAFNSFMVNSIPVVVTLVSFGTFTLLGGDLTPAKAFTSLSLFQVLRYPLNMLPNLLSQVVNANI
SLQRLEELFLAEERILAPNPPLEPGIPAISIENGNFSWDLKLENPTLTNIKLNIQVGSLVAIVGGTGEGKTSLISAM
LGELPPMEDACVVIRGTVAYAPQVPWIFNATVRDNILFGSKYEPSRYGKAIDVTALQHDLDLFAGHDLTEIGER
GVNISGGQKQRISMARAFYSNSDIYIFDDPLSALDAHVARQVFNSCIKEGLQGKTRVLVTNQLHFLPQVEKIILL
SEGMIKEEGTFEELFKNSELFQKLMENAGKMEEQVKEKEKSDNLDHKSSKAEANWENELPQKAASTMKGKE
GKSILIKQEERERGVVSWNVLIRYNNALGGVWVVSILFLCYLLTEVFRVSRSTWLSFWTNQSTLESYRPGYFIF
VYGLLSFGQVTVTLANSYWLISSSLHASKRLHDAMLDSILRTPMLFFHTNPTGRIINRFAKDVGEIDRNVANSA
NNFLNLAWQLLSTFVLIGTVSTISLWAIMPLLILFYSAYLYYQNTSREVKRLDSITRSPVYAQFGEALNGLSSIRA
YKAYDWMSIINGKYMDNNIRFSLVTISSDGWLAIRLVTLGGMMIWLIASFSVLGNGRTENHVGFASIMGLLLS
YTSNITDLLSNVLRQASKAENSLNSVERVSTYIDLPSEAPAIDKNNRPPSSWPLSGLIKFTDVVLRYRPELPPVLH
GLSFAVSPSEKLGIVGRTGAGKSSMLNALFRIVELERGEITIDGCDITKFGLTDLRRALSIIPQSPVLFSGTVRFNL
DPFSEHNDADLWKALERAHLKDAVRNSSFGLDAQVFGGESFSVGQRQLLSLARALLRRSKILVLDEATSSVDV
RIDALIQKTIREEFRSCTMLIIAHRLNTIIDCDRILVLEAGQVLEHSTPEELLSNEGSAFSRMVQSTGPANAQYLHS
LVFESKENKLMFIHS

>XP_024437180.1_ABCC12_X4_Pt
MGLEALVWYCRPVPNGVWATKVDNAFGAYTPCVVDSLVICISHLVLLGLCLYRIWLITDKNSKAQHYCLRTN
YYNYSLGLLAAYCTVQPLFRLFMNVSIFNLDGQTALAPFEMVSLIVEALSWCSTLIMIGLETRIYIQQFRWYVRF
GVIYVLVGEAAMLNLILSVSDNYDSRFIFYMYLSTVFCQVLFGIHLLVYIPNLDPCSDYVMMEPESPDNSAYEA
LPGREQICPERNATLFSRIFYWWLTPLMKQAHKRPISEKDVWKLDTWDQTETSMNNSCRFQTCWVEESQRPKP
CLLRALNNSLGGRFWLGGFFKSMQRGDPAWIGYVYAFVIFLGMLFSALCESRYYQNVLRVGFRLRSTLVAGIF
RKSLKLTHEGQKNFPSGKITNMITTDADVLQQICLLLHGLWSAPFCITMSMVLLYQQLGVASLFGSLVLVIMVP
TQAILLNRMTRLTKEGLHRTDKRVSLMNEILAAMDTVKCYAWEKSFQFRVQSVRNDELSLFRSAQLLFAFNSF
MVNSIPVVVTLVSFGTFTLLGGDLTPAKAFTSLSLFQVLRYPLNMLPNLLSQVVNANISLQRLEELFLAEERILA
PNPPLEPGIPAISIENGNFSWDLKLENPTLTNIKLNIQVGSLVAIVGGTGEGKTSLISAMLGELPPMEDACVVIRG
TVAYAPQVPWIFNATVRDNILFGSKYEPSRYGKAIDVTALQHDLDLFAGHDLTEIGERGVNISGGQKQRISMAR
AFYSNSDIYIFDDPLSALDAHVARQVFNSCIKEGLQGKTRVLVTNQLHFLPQVEKIILLSEGMIKEEGTFEELFKN
SELFQKLMENAGKMEEQVKEKEKSDNLDHKSSKAEANWENELPQKAASTMKGKEGKSILIKQEERERGVVS
WNVLIRYNNALGGVWVVSILFLCYLLTEVFRVSRSTWLSFWTNQSTLESYRPGYFIFVYGLLSFGQVTVTLANS
YWLISSSLHASKRLHDAMLDSILRTPMLFFHTNPTGRIINRFAKDVGEIDRNVANSANNFLNLAWQLLSTFVLIG

TVSTISLWAIMPLLILFYSAYLYYQNTSREVKRLDSITRSPVYAQFGEALNGLSSIRAYKAYDWMSIINGKYMD
NNIRFSLVTISSDGWLAIRLVTLGGMMIWLIASFSVLGNGRTENHVGFASIMGLLLSYTSNITDLLSNVLRQASK
AENSLNSVERVSTYIDLPSEAPAIDKNNRPPSSWPLSGLIKFTDVVLRYRPELPPVLHGLSFAVSPSEKLGIVGRT
GAGKSSMLNALFRIVELERGEITIDGCDITKFGLTDLRRALSIIPQSPVLFSGTVRFNLDPFSEHNDADLWKALER
AHLKDAVRNSSFGLDAQVFGGESFSVGQRQLLSLARALLRRSKILVLDEATSSVDVRIDALIQKTIREEFRSCTM
LIIAHRLNTIIDCDRILVLEAGQVLEHSTPEELLSNEGSAFSRMVQSTGPANAQYLHSLVFESKENKLMFIHS

>XP_024437181.1_ABCC12_X5_Pt
MGLEALVWYCRPVPNGVWATKVDNAFGAYTPCVVDSLVICISHLVLLGLCLYRIWLITDKNSKAQHYCLRTN
YYNYSLGLLAAYCTVQPLFRLFMNVSIFNLDGQTALAPFEMVSLIVEALSWCSTLIMIGLETRIYIQQFRWYVRF
GVIYVLVGEAAMLNLILSVSDNYDSRFIFYMYLSTVFCQVLFGIHLLVYIPNLDPCSDYVMMEPESPDNSAYEA
LPGREQICPERNATLFSRIFYWWLTPLMKQAHKRPISEKDVWKLDTWDQTETSMNKFQTCWVEESQRPKPCLL
RALNNSLGGRFWLGGFFKSMQRGDPAWIGYVYAFVIFLGMLFSALCESRYYQNVLRVGFRLRSTLVAGIFRKS
LKLTHEGQKNFPSGKITNMITTDADVLQQICLLLHGLWSAPFCITMSMVLLYQQLGVASLFGSLVLVIMVPTQA
ILLNRMTRLTKEGLHRTDKRVSLMNEILAAMDTVKCYAWEKSFQFRVQSVRNDELSLFRSAQLLFAFNSFMV
NSIPVVVTLVSFGTFTLLGGDLTPAKAFTSLSLFQVLRYPLNMLPNLLSQVVNANISLQRLEELFLAEERILAPNP
PLEPGIPAISIENGNFSWDLKLENPTLTNIKLNIQVGSLVAIVGGTGEGKTSLISAMLGELPPMEDACVVIRGTVA
YAPQVPWIFNATVRDNILFGSKYEPSRYGKAIDVTALQHDLDLFAGHDLTEIGERGVNISGGQKQRISMARAFY
SNSDIYIFDDPLSALDAHVARQVFNSCIKEGLQGKTRVLVTNQLHFLPQVEKIILLSEGMIKEEGTFEELFKNSEL
FQKLMENAGKMEEQVKEKEKSDNLDHKSSKAEANWENELPQKAASTMKGKEGKSILIKQEERERGVVSWNV
LIRYNNALGGVWVVSILFLCYLLTEVFRVSRSTWLSFWTNQSTLESYRPGYFIFVYGLLSFGQVTVTLANSYWL
ISSSLHASKRLHDAMLDSILRTPMLFFHTNPTGRIINRFAKDVGEIDRNVANSANNFLNLAWQLLSTFVLIGTVS
TISLWAIMPLLILFYSAYLYYQNTSREVKRLDSITRSPVYAQFGEALNGLSSIRAYKAYDWMSIINGKYMDNNIR
FSLVTISSDGWLAIRLVTLGGMMIWLIASFSVLGNGRTENHVGFASIMGLLLSYTSNITDLLSNVLRQASKAENS
LNSVERVSTYIDLPSEAPAIDKNNRPPSSWPLSGLIKFTDVVLRYRPELPPVLHGLSFAVSPSEKLGIVGRTGAGK
SSMLNALFRIVELERGEITIDGCDITKFGLTDLRRALSIIPQSPVLFSGTVRFNLDPFSEHNDADLWKALERAHLK
DAVRNSSFGLDAQVFGGESFSVGQRQLLSLARALLRRSKILVLDEATSSVDVRIDALIQKTIREEFRSCTMLIIAH
RLNTIIDCDRILVLEAGQVLEHSTPEELLSNEGSAFSRMVQSTGPANAQYLHSLVFESKENKLMFIHS

>XP_024440643.1_P_ABCC15_X1_Pt
MLDLGAAAAAAAAAAAANLKLLIRMDWPQLQSPCLREHITIGVQLGFLGILLLHLLRKCADLAFNGGTKTTDQ
GKENYHIGLKFSNSYKASMVCSTCLLGVHISMLLVLLNGQETSCNSIVRVFSAEVLQMISWAITLVAVFRIFPSR
RYVKFPWIIRAWWLCSFMLSIVCTSLDINFKITNHGHLRLRDYAELFALLPSTFLLAISFRGKTGIVFNAFNGVT
DPLLHEKSDKDSDTKRESPYGKATLLQLITFSWLTPLFAVGYKKPLEQDEIPDVYIKDSAGFLSSSFDENLNQVK
EKDRTANPSIYKAIFLFIRKKAAINALFAVTSAAASYVGPYLIDDFVNFLTEKKTRSLQSGYLLALGFLGAKTVE
TIAQRQWIFGARQLGLRLRASLISHIYKKGLLLSSQSRQSHTSGEIINYMSVDIQRITDFIWYLNYIWMLPVQITL
AIYILHTTLGLGSMAALTATLAVMACNIPITRFQKRYQTKIMEAKDKRMKATSEVLRNMKILKLQAWDTQFLH
KIESLRKIEYNCLWKSLRLSAISAFVFWGSPTFISVVTFGACMLMGIQLTAGRVLSALATFRMLQDPIFNLPDLL
SVIAQGKVSADRVASFLQEGEIQHDATEHVPKDQAEYAISIDDGRFCWDSDSSNPTLDEIRLKVKRGMKVAICG
TVGSGKSSLLSCILGEIQKLSGTVKISGAKAYVPQSPWILTGNIRENILFGNPYDSVRYYRTVKACALLKDFELFS
SGDLTDIGERGINMSGGQKQRIQIARAVYQDADIYLFDDPFSAVDAHTGSQLFQECLMGILKDKTIIYVTHQVE
FLPAADIILVMQNGRIAEAGTFSELLKQNVGFEALVGAHSQALESVLTVENSRRTSQDPEPDSESNTESTSNSNC
LSHYESDHDLSVEITEKGGKFVQDEEREKGSIGKEVYWSYLTTVKGGALVPCIILAQSLFQILQIVSNYWMAWS
SPPTSDTAPVYGMNFILLVYTLLSISSSLCVLVRATLVAIAGLSTAQKLFTNMLRSLLRAPMAFFDSTPTGRILNR
ASMDQSVIDMEIAQRLGWCAFSIIQILGTIAVMSQVAWEVFVIFIPVTAVCIWYQQYYTPTARELARLAGIQQAP
ILHHFSESLAGAATIRAFDQQERFYCSNLDLIDNHSRPWFHNVSAMEWLSFRLNLLSNFVFAFSLVLLVSLPEGV
ISPSIAGLAVTYGINLNVLQASVIWNICNAENKMISIERVLQYSSITSEAPLVLEQSRPPNKWPEVGAICFKDLQIR
YAEHLPSVLKNINCAFPGRKKVGVVGRTGSGKSTLIQAIFRIVEPREGSIIIDDVDISKIGLQDLRSRLSIIPQDPTM
FEGTVRGNLDPLGQYSDYEIWEALEKCQLGDLVRGKDEKLDSPVVENGENWSVGQRQLFCLGRALLKKSRIL
VLDEATASVDSATDGVIQKIISQEFKDRTVVTIAHRIHTVIDSDLVLVLSDGRVAEFDTPARLLEREESFFSKLIK
EYSMRSQSFNNLTNVHA

>XP_024440644.1_P_ABCC15_X2_Pt
MLDLGAAAAAADLKLLIRMDWPQLQSPCLREHITIGVQLGFLGILLLHLLRKCADLAFNGGTKTTDQGKENYH

IGLKFSNSYKASMVCSTCLLGVHISMLLVLLNGQETSCNSIVRVFSAEVLQMISWAITLVAVFRIFPSRRYVKFP
WIIRAWWLCSFMLSIVCTSLDINFKITNHGHLRLRDYAELFALLPSTFLLAISFRGKTGIVFNAFNGVTDPLLHEK
SDKDSDTKRESPYGKATLLQLITFSWLTPLFAVGYKKPLEQDEIPDVYIKDSAGFLSSSFDENLNQVKEKDRTA
NPSIYKAIFLFIRKKAAINALFAVTSAAASYVGPYLIDDFVNFLTEKKTRSLQSGYLLALGFLGAKTVETIAQRQ
WIFGARQLGLRLRASLISHIYKKGLLLSSQSRQSHTSGEIINYMSVDIQRITDFIWYLNYIWMLPVQITLAIYILHT
TLGLGSMAALTATLAVMACNIPITRFQKRYQTKIMEAKDKRMKATSEVLRNMKILKLQAWDTQFLHKIESLR
KIEYNCLWKSLRLSAISAFVFWGSPTFISVVTFGACMLMGIQLTAGRVLSALATFRMLQDPIFNLPDLLSVIAQG
KVSADRVASFLQEGEIQHDATEHVPKDQAEYAISIDDGRFCWDSDSSNPTLDEIRLKVKRGMKVAICGTVGSG
KSSLLSCILGEIQKLSGTVKISGAKAYVPQSPWILTGNIRENILFGNPYDSVRYYRTVKACALLKDFELFSSGDLT
DIGERGINMSGGQKQRIQIARAVYQDADIYLFDDPFSAVDAHTGSQLFQECLMGILKDKTIIYVTHQVEFLPAA
DIILVMQNGRIAEAGTFSELLKQNVGFEALVGAHSQALESVLTVENSRRTSQDPEPDSESNTESTSNSNCLSHYE
SDHDLSVEITEKGGKFVQDEEREKGSIGKEVYWSYLTTVKGGALVPCIILAQSLFQILQIVSNYWMAWSSPPTS
DTAPVYGMNFILLVYTLLSISSSLCVLVRATLVAIAGLSTAQKLFTNMLRSLLRAPMAFFDSTPTGRILNRASMD
QSVIDMEIAQRLGWCAFSIIQILGTIAVMSQVAWEVFVIFIPVTAVCIWYQQYYTPTARELARLAGIQQAPILHH
FSESLAGAATIRAFDQQERFYCSNLDLIDNHSRPWFHNVSAMEWLSFRLNLLSNFVFAFSLVLLVSLPEGVISPSI
AGLAVTYGINLNVLQASVIWNICNAENKMISIERVLQYSSITSEAPLVLEQSRPPNKWPEVGAICFKDLQIRYAE
HLPSVLKNINCAFPGRKKVGVVGRTGSGKSTLIQAIFRIVEPREGSIIIDDVDISKIGLQDLRSRLSIIPQDPTMFEG
TVRGNLDPLGQYSDYEIWEALEKCQLGDLVRGKDEKLDSPVVENGENWSVGQRQLFCLGRALLKKSRILVLD
EATASVDSATDGVIQKIISQEFKDRTVVTIAHRIHTVIDSDLVLVLSDGRVAEFDTPARLLEREESFFSKLIKEYS
MRSQSFNNLTNVHA

>XP_024454768.1_ABCC12_X2_Pt
MGLEALVWYCRPMANGVWAKEVDNAFGAYTPCAIDSIVICISHLVLLGLCLYRIWLIIDNNTKVQKYCLRTNY
YNYMLGFLAAYCTVQPLLRLFMDVSIFNLDGQTSLAPFELVSLIVEALAWCSTLIMIGLETKIYIRQFRWYVRF
GVIYVLVGEAAMLNLILSVSDYYSRFTLYMYISTVFCQVLLGILLLVYIPNLDPYPDYVMMESESLDNCEYEAL
PGREQICPERHANLFSRIFFGWLTPLMKQGHKRPITEKDVWKLDTWDQTETLIKKFQTCWVEESKRPKPRLLR
ALNNSLGGRFWLGGFFKSMQRGDPAWIGYIYAFIIFLGVLFGALCESQFFQNVMRVGFQLRSTLVAAIFRKSLK
LTHEGRKNFPSGKITNMITTDANALQQICQQLHGLWSAPFRITISMVLLYQQLGVASLFGSLMLVLMVPTQTIL
MSKMRKLTKEGLHRTDKRVSLMNEILAAMDAVKCYAWEKSFQSRIQSVRDDELSWFRSAQLLSAFNSFILNSI
PVIVTLVSFGTFTLLGGDLTPAKAFTSLSLFQVLRSPLNMLPNLLSQVVNANISLQRLEELFLAEERILAPNLPLK
LGIPAISIENGNFLWDSKLEKPTLSDINLKIQVGSLVAIVGGTGEGKTSLISAMLGELPPMEDASVVIRGTVAYVP
QVSWIFNATVRDNILFGSEYEPSRYWKAIDVTALQHDLDLLPGHDLTEIGERGVNISGGQKQRVSMARAVYSN
SDVYIFDDPLSALDAHVGRQVFNSCIKDELQGKTRVLVTNQLHFLPQVDKIILLSEGMIKEEGTFEELSKNGKLF
QKLMENAGKMDELVEEKNSENLDYKSSKPAANRGNDLPQKAGYKMKVKGGKSVLIKQEERETGVVSWNVLI
RYNNALGGIWVVLIIFLCYLLTEVLRVSRSTWLSFWTNQSTLESYRPGYYIFVYALLSFGQVIVTLVNSYWLISS
SLHAAKRLHDAMLDSILRAPMLFFHTNPSGRIINRFAKDLGEIDRNVANFANNFLNQAWQLFSTFVLIGIVSTIS
LWAVMPLLILFYSAYLYYQSTSREVKRLDSITRSPVYAQFGEALNGLSSIRAYKAYDWMAIINGKSMDNNIRFT
LVNMSSNRWLTIRLVTLGGIMIWLIATFAVLGNGRTENHVEFASVMGLLLSYTLNITDLLSNVLRQASRAENSL
NSVERVGTYMDLPSEAPAIVETNRPPPAWPSSGSIKFRDVVLRYRPELPPVLHHLSFEVSPSEKLGIVGRTGAGK
SSMLNALFRIVELERGEITIDGCDVAKFGLTDLRKILSIIPQSPVLFSGTVRFNLDPFSEHNDADLWEALERAHLK
DAIRNNSFGLDAEVFEGGENFSVGQRQLLSLARALLRRSKILVLDEATASVDVRTDALIQKTIREEFRSCTMLVI
AHRLNTIIDCDRILVLEAGQVLEHGTPEELLLPNEGSAFSRMVQSTGPANAQYLYSLVFESKENKLSKRKNDHR
WIDSSRWAAAAQLALVVSLTSSENGLPMLDVGDEDNILRKTKDAVIRLQDVLVGKYDEAICDTLQQSQVPQD
GWWSAFYRMIEGLAVMGRLSRGRHQQYDYENEPLDWDDLKI

>XP_024454769.1_ABCC12_X3_Pt
MGLEALVWYCRPMANGVWAKEVDNAFGAYTPCAIDSIVICISHLVLLGLCLYRIWLIIDNNTKVQKYCLRTNY
YNYMLGFLAAYCTVQPLLRLFMDVSIFNLDGQTSLAPFELVSLIVEALAWCSTLIMIGLETKIYIRQFRWYVRF
GVIYVLVGEAAMLNLILSVSDYYSRFTLYMYISTVFCQVLLGILLLVYIPNLDPYPDYVMMESESLDNCEYEAL
PGREQICPERHANLFSRIFFGWLTPLMKQGHKRPITEKDVWKLDTWDQTETLIKKFQTCWVEESKRPKPRLLR
ALNNSLGGRFWLGGFFKIGYDLSQFVGPIVLSHLLQSMQRGDPAWIGYIYAFIIFLGVVAAIFRKSLKLTHEGRK
NFPSGKITNMITTDANALQQICQQLHGLWSAPFRITISMVLLYQQLGVASLFGSLMLVLMVPTQTILMSKMRKL
TKEGLHRTDKRVSLMNEILAAMDAVKCYAWEKSFQSRIQSVRDDELSWFRSAQLLSAFNSFILNSIPVIVTLVS
FGTFTLLGGDLTPAKAFTSLSLFQVLRSPLNMLPNLLSQVVNANISLQRLEELFLAEERILAPNLPLKLGIPAISIE

NGNFLWDSKLEKPTLSDINLKIQVGSLVAIVGGTGEGKTSLISAMLGELPPMEDASVVIRGTVAYVPQVSWIFN
ATVRDNILFGSEYEPSRYWKAIDVTALQHDLDLLPGHDLTEIGERGVNISGGQKQRVSMARAVYSNSDVYIFD
DPLSALDAHVGRQVFNSCIKDELQGKTRVLVTNQLHFLPQVDKIILLSEGMIKEEGTFEELSKNGKLFQKLMEN
AGKMDELVEEKNSENLDYKSSKPAANRGNDLPQKAGYKMKVKGGKSVLIKQEERETGVVSWNVLIRYNNAL
GGIWVVLIIFLCYLLTEVLRVSRSTWLSFWTNQSTLESYRPGYYIFVYALLSFGQVIVTLVNSYWLISSSLHAAK
RLHDAMLDSILRAPMLFFHTNPSGRIINRFAKDLGEIDRNVANFANNFLNQAWQLFSTFVLIGIVSTISLWAVMP
LLILFYSAYLYYQSTSREVKRLDSITRSPVYAQFGEALNGLSSIRAYKAYDWMAIINGKSMDNNIRFTLVNMSS
NRWLTIRLVTLGGIMIWLIATFAVLGNGRTENHVEFASVMGLLLSYTLNITDLLSNVLRQASRAENSLNSVERV
GTYMDLPSEAPAIVETNRPPPAWPSSGSIKFRDVVLRYRPELPPVLHHLSFEVSPSEKLGIVGRTGAGKSSMLNA
LFRIVELERGEITIDGCDVAKFGLTDLRKILSIIPQSPVLFSGTVRFNLDPFSEHNDADLWEALERAHLKDAIRNN
SFGLDAEVFEGGENFSVGQRQLLSLARALLRRSKILVLDEATASVDVRTDALIQKTIREEFRSCTMLVIAHRLNT
IIDCDRILVLEAGQVLEHGTPEELLLPNEGSAFSRMVQSTGPANAQYLYSLVFESKENKLSKRKNDHRWIDSSR
WAAAAQLALVVSLTSSENGLPMLDVGDEDNILRKTKDAVIRLQDVLVGKYDEAICDTLQQSQVPQDGWWSA
FYRMIEGLAVMGRLSRGRHQQYDYENEPLDWDDLKI

>XP_024454770.1_ABCC12_X4_Pt
MGLEALVWYCRPMANGVWAKEVDNAFGAYTPCAIDSIVICISHLVLLGLCLYRIWLIIDNNTKVQKYCLRTNY
YNYMLGFLAAYCTVQPLLRLFMDVSIFNLDGQTSLAPFELVSLIVEALAWCSTLIMIGLETKIYIRQFRWYVRF
GVIYVLVGEAAMLNLILSVSDYYSRFTLYMYISTVFCQVLLGILLLVYIPNLDPYPDYVMMESESLDNCEYEAL
PGREQICPERHANLFSRIFFGWLTPLMKQGHKRPITEKDVWKLDTWDQTETLIKKFQTCWVEESKRPKPRLLR
ALNNSLGGRFWLGGFFKSMQRGDPAWIGYIYAFIIFLGVVAAIFRKSLKLTHEGRKNFPSGKITNMITTDANAL
QQICQQLHGLWSAPFRITISMVLLYQQLGVASLFGSLMLVLMVPTQTILMSKMRKLTKEGLHRTDKRVSLMNE
ILAAMDAVKCYAWEKSFQSRIQSVRDDELSWFRSAQLLSAFNSFILNSIPVIVTLVSFGTFTLLGGDLTPAKAFT
SLSLFQVLRSPLNMLPNLLSQVVNANISLQRLEELFLAEERILAPNLPLKLGIPAISIENGNFLWDSKLEKPTLSDI
NLKIQVGSLVAIVGGTGEGKTSLISAMLGELPPMEDASVVIRGTVAYVPQVSWIFNATVRDNILFGSEYEPSRY
WKAIDVTALQHDLDLLPGHDLTEIGERGVNISGGQKQRVSMARAVYSNSDVYIFDDPLSALDAHVGRQVFNS
CIKDELQGKTRVLVTNQLHFLPQVDKIILLSEGMIKEEGTFEELSKNGKLFQKLMENAGKMDELVEEKNSENL
DYKSSKPAANRGNDLPQKAGYKMKVKGGKSVLIKQEERETGVVSWNVLIRYNNALGGIWVVLIIFLCYLLTE
VLRVSRSTWLSFWTNQSTLESYRPGYYIFVYALLSFGQVIVTLVNSYWLISSSLHAAKRLHDAMLDSILRAPML
FFHTNPSGRIINRFAKDLGEIDRNVANFANNFLNQAWQLFSTFVLIGIVSTISLWAVMPLLILFYSAYLYYQSTSR
EVKRLDSITRSPVYAQFGEALNGLSSIRAYKAYDWMAIINGKSMDNNIRFTLVNMSSNRWLTIRLVTLGGIMIW
LIATFAVLGNGRTENHVEFASVMGLLLSYTLNITDLLSNVLRQASRAENSLNSVERVGTYMDLPSEAPAIVETN
RPPPAWPSSGSIKFRDVVLRYRPELPPVLHHLSFEVSPSEKLGIVGRTGAGKSSMLNALFRIVELERGEITIDGCD
VAKFGLTDLRKILSIIPQSPVLFSGTVRFNLDPFSEHNDADLWEALERAHLKDAIRNNSFGLDAEVFEGGENFSV
GQRQLLSLARALLRRSKILVLDEATASVDVRTDALIQKTIREEFRSCTMLVIAHRLNTIIDCDRILVLEAGQVLE
HGTPEELLLPNEGSAFSRMVQSTGPANAQYLYSLVFESKENKLSKRKNDHRWIDSSRWAAAAQLALVVSLTSS
ENGLPMLDVGDEDNILRKTKDAVIRLQDVLVGKYDEAICDTLQQSQVPQDGWWSAFYRMIEGLAVMGRLSR
GRHQQYDYENEPLDWDDLKI

>XP_024454771.1_ABCC12_X1_Pt
MGLEALVWYCRPMANGVWAKEVDNAFGAYTPCAIDSLVICISHLVLLGLCLYRIWLIIDNNTKVQKYCLRTN
YYNYMLGFLAAYCTVQPLLRLFMDVSIFNLDGQTSLSPFELVSLIVEALAWCSTLIMIGLETKIYIRQFRWYVRF
GVIYVLVGEAAMLNLILSVSDYYSRFTLYMYISTVFCQVLLGILLLVYIPNLDPYPDYVMMESESLDNCEYEAL
PGREQICPERHANLFSRIFFGWLTPLMKQGHKRPITEKDVWKLDTWDQTETLIKNYCRFQTCWVEESKRPQPR
LLRALNNSLGGRFWLGGFFKIGNDLSQFVGPIVLSHLLQSMQRGDPAWIGYIYAFIIFLGVLFGALCESQFFQNV
MRVGFQLRSTLVAAIFRKSLKLTHEGRKNFPSGKITNMITTDANALQQICQQLHGLWSAPFRITISMVLLYQQL
GVASLFGSLMLVLMVPTQTILMSKMRKLTKEGLHRTDKRVSLMNEILAAMDVMKCYAWEKSFQSRIQSVRD
DELSWFRGAQLLSAFNSFILNIIPVIVTLVSFGTFTLLGGDLTPARAFTSLSLFQVLRSPLNMLPNLLSQVVNANIS
LQRLEELFLAEERILAPNLPLKLGIPAISIENGNFLWDSKLEKPTLSDINLKIQVGSLVAIVGGTGEGKTSLISAML
GELPPMEDASVVIRGTVAYVPQVSWIFNATVRDNILFRSEYEPSRYWKAIDVTALQHDLDLLPGHDLTEIGERG
VNISGGQKQRVSMARAVYSNSDVYIFDDPLSALDAHVGRQVFNSCIKDELQGKTRVLVTNQLHFLPQVDKIIL
LSEGMIKEEGTFEELSENGKLFQKLMENAGKMEELVEEKNSENLDCKSLKPAANGGNDLPQKAGYKMKVKG
GKSVLIKQEERETGVVSWNVLIRYNNALGGILVVLIIFLCYLLTEVLRVSRSTWLSVWTNQSTLESYKPGYYIFV
YALLSFGQVIVTLVNSYWLISSSLHAAKRLHDAMLDSILRAPMLFFHTNPSGRIINRFAKDLGEIDRNVANIANN

FLNQAWQLFSTFVLIGIVSTISLWAVMPLLILFYSAYLYYQSTSREVKRLDSITRSPVYAQFGEALNGLSSIRAYK
AYDWMAIINGKSMDNNIRFTLVNMSSNHWLTIRLVTLGGIMIWLIATFAVLGNGRTENHVEFASEMGLLLSYT
LNITGLLSNVLRHASRAENSLNSVERVGTYMDLPSEAPAIVETNRPPPAWPSSGSIKFRDVVLRYRPELPPVLHH
LSFEVSPSEKLGIVGRTGAGKSSMLNALFRIVELERGEITIDGCDVAKFGLTDLRKILSIIPQSPVLFSGTVRFNLD
PFSEHNDADLWEALERAHLKDAIRNNSFGLDAEVFEGGENFSVGQRQLLSLARALLRRSKILVLDEATASVDV
RTDALIQKTIREEFRSCTMLVIAHRLNTIIDCDRILVLEAGQVLEHGTPEELLLPNEGSAFSRMVQSTGPANAQY
LYSLVFESKENKLSKRKNDHRWIDSSRWAAAAQLALVVSLTSSENGLPMSDVEDEDNILRKTKDAVIRLQDVL
VGKYDEAICDTQQQSQVPQDGWWSAFYRMIEGLAVMGRLSRGRHQQYDYENESLD

>XP_024454772.1_ABCC12_X2_Pt
MGLEALVWYCRPMANGVWAKEVDNAFGAYTPCAIDSLVICISHLVLLGLCLYRIWLIIDNNTKVQKYCLRTN
YYNYMLGFLAAYCTVQPLLRLFMDVSIFNLDGQTSLSPFELVSLIVEALAWCSTLIMIGLETKIYIRQFRWYVRF
GVIYVLVGEAAMLNLILSVSDYYSRFTLYMYISTVFCQVLLGILLLVYIPNLDPYPDYVMMESESLDNCEYEAL
PGREQICPERHANLFSRIFFGWLTPLMKQGHKRPITEKDVWKLDTWDQTETLIKKFQTCWVEESKRPQPRLLR
ALNNSLGGRFWLGGFFKIGNDLSQFVGPIVLSHLLQSMQRGDPAWIGYIYAFIIFLGVLFGALCESQFFQNVMR
VGFQLRSTLVAAIFRKSLKLTHEGRKNFPSGKITNMITTDANALQQICQQLHGLWSAPFRITISMVLLYQQLGV
ASLFGSLMLVLMVPTQTILMSKMRKLTKEGLHRTDKRVSLMNEILAAMDVMKCYAWEKSFQSRIQSVRDDEL
SWFRGAQLLSAFNSFILNIIPVIVTLVSFGTFTLLGGDLTPARAFTSLSLFQVLRSPLNMLPNLLSQVVNANISLQR
LEELFLAEERILAPNLPLKLGIPAISIENGNFLWDSKLEKPTLSDINLKIQVGSLVAIVGGTGEGKTSLISAMLGEL
PPMEDASVVIRGTVAYVPQVSWIFNATVRDNILFRSEYEPSRYWKAIDVTALQHDLDLLPGHDLTEIGERGVNI
SGGQKQRVSMARAVYSNSDVYIFDDPLSALDAHVGRQVFNSCIKDELQGKTRVLVTNQLHFLPQVDKIILLSE
GMIKEEGTFEELSENGKLFQKLMENAGKMEELVEEKNSENLDCKSLKPAANGGNDLPQKAGYKMKVKGGKS
VLIKQEERETGVVSWNVLIRYNNALGGILVVLIIFLCYLLTEVLRVSRSTWLSVWTNQSTLESYKPGYYIFVYAL
LSFGQVIVTLVNSYWLISSSLHAAKRLHDAMLDSILRAPMLFFHTNPSGRIINRFAKDLGEIDRNVANIANNFLN
QAWQLFSTFVLIGIVSTISLWAVMPLLILFYSAYLYYQSTSREVKRLDSITRSPVYAQFGEALNGLSSIRAYKAY
DWMAIINGKSMDNNIRFTLVNMSSNHWLTIRLVTLGGIMIWLIATFAVLGNGRTENHVEFASEMGLLLSYTLNI
TGLLSNVLRHASRAENSLNSVERVGTYMDLPSEAPAIVETNRPPPAWPSSGSIKFRDVVLRYRPELPPVLHHLSF
EVSPSEKLGIVGRTGAGKSSMLNALFRIVELERGEITIDGCDVAKFGLTDLRKILSIIPQSPVLFSGTVRFNLDPFS
EHNDADLWEALERAHLKDAIRNNSFGLDAEVFEGGENFSVGQRQLLSLARALLRRSKILVLDEATASVDVRTD
ALIQKTIREEFRSCTMLVIAHRLNTIIDCDRILVLEAGQVLEHGTPEELLLPNEGSAFSRMVQSTGPANAQYLYSL
VFESKENKLSKRKNDHRWIDSSRWAAAAQLALVVSLTSSENGLPMSDVEDEDNILRKTKDAVIRLQDVLVGK
YDEAICDTQQQSQVPQDGWWSAFYRMIEGLAVMGRLSRGRHQQYDYENESLD

>XP_024467220.1_ABCC2_X1_Pt
MGFEALDWYCKPVRDGVWTKAVQNAFGAYTPCATDTLVVSLSYLVLMALCFYKIWLTKKDFKLQRFCLRSK
WYAYLLALLALYSTAEPLYRLVMGISVLNLDGQTGLAPFEIGSLIIEALAWCSLLVMIVVEIKVYIREFRWFVRF
GVIYTLVGDAVMLNLILTVKEFYNNAVLHLYISEVIVQGLFGILLLVYVPDLDPYPGYTPMQIESVDDAEYEEL
PGGEYICPERHANIISKIVFGWMSPLMKLGYRRPITEKDVWKLDTWDRTETLNDRFQKCWAEELRKPKPWLLR
ALHSSLGGRFWWGGFWKIGNDASQFVGPLVLNQLLKSMQEGDPAWIGYVYAFSIFAGVVFGVLCEAQYFQN
VMRVGYRLRATLVAAVFRKSLRLTHEGRRKFASGKITNLMTTDAEALQQICQSLHTLWSAPFRIIVAMVLLYQ
QLNVASLLGALMLVLLFPIQTFVISRMQKLSKEGLQRTDKRIGLMNEILAAMDTVKCYAWESSFQAKVQGVR
DDELSWFRKASLLGACNSFILNSIPVMVTVISFGMYTLLGGNLTPARAFTSLSLFAVLRFPLFMLPNMITQVVNA
NVSLKRLEELFLAEERILLPNPLLDPCLPAVSIKNGYFSWDSKAERPTLSNINLDVPIGSLVAVVGSTGEGKTSLV
SAMLGELPATSDASVVIRGTVAYVPQVSWIFNATVRDNILFGSPFDSARYEKAIDVTALQHDLDLLPGGDLTEI
GERGVNISGGQKQRVSMARAVYSNSDVYIFDDPLSALDAQVGRQVFDKCIKGELSKKTRILVTNQLHFLSQVD
RIILVHEGMVKEEGTFEDLSNNGMLFQKLMENAGKMEEYEEQENNEIVDHKTSSKQVANGVMNNLPKNVSG
TKKPKEGKSVLIKQEERETGVVNLKVLIRYKNALGGAWVVMVLFMCYLMTEVLRVSSSTWLSNWTNQGTSK
RHGPLYYNLIYSFLSIGQVSVTLLNSYWLITSSLYAAKRLHDAMLNSILRAPMVFFHTNPLGRIINRFAKDLGDI
DRNVAIFVNMFMGQISQLLSTFVLIGIVSTMSLWAIMPLLVLFYGAYLYYQSTAREVKRLDSITRSPVYAQFGE
ALNGLSTIRAYKAYDRMASINGKSMDNNVRYTLVNMGANRWLAIRLETLGGIMIWFTATFAVMQNGRADNQ
QAFASTMGLLLSYALNITSLLTAVLRLASLAENSLNSVERVGTYIELPSEAPLVIESNRPPPGWPSSGAIKFEDVV
LRYRPELPPVLHGLSFTIFPSDKVGIVGRTGAGKSSMLNALFRIVELERGRILIDDCNISKFGLMDLRKVLGIIPQA
PVLFSGTVRFNLDPFSEHNDADLWEALERAHLKDVIRRNSLGLDSEVTEAGDNFSVGQRQLLSLARALLRRSKI
LVLDEATAAVDVRTDALIQKTIREEFRSCTMLIIAHRLNTIIDCDRVILLDSGRVLEYDTPEELLSNENSAFSKMV

QSTGAANAQYLRSLVMGGERESRSGREENKQLDGPRRWLASSRWAAAAQFALAVSLTSSQNDLQQLEIEDEN
SVLKKTKDAVVTLQRVLEGKHDKVIDESLNQYQISRDGWWSALYKMVEGLAMMSRLGRNRLHQSDYGLED
KTIDWNHVEM

>XP_024635181.1_ABCC12_Mt
MGFEPLIWYCKPEPNSIWSKTVDSAFGSYTPCAINTLVISTSNLVLMGLCLYRIWLIIFNAKAQRFCLKSNYYNY
ILAMLASYCAFQPLLRLWTVNSVFNLNEEADFAPFEIMSLIIESVTWFSMIILILLETKIYIRQFRWLVRFGVIYVL
VGDIVMFDLLLSVKDYSSRSSLYLYISTIICQVLFGTLLLVYIPNLVPYSGHATFQADIPDNGEYEPLCGDDQVCP
EMRANFLSRLSYGWITPLMKQGYRKPITEKDVWKLDKWDQTETLNENFQKCWTSEFQSSNPWLLRALNSSLG
KRFWFGGIFKIGNDLSQFVGPILLNHLLDSMQNGDPSWIGYIYAFSIFVGVSAGVVCEAQYFQNVMRVGFRLRS
TLVAAIFRKSLKLTHESRKKFSMGKLMNMITTDANALQQICQQLHGLWSAPFRIIIAMVLLYQQLGVASLIGSL
LLVLIIPLQTFVISKMRKLTKEGLQQTDKRVGLMNEILSAMDTVKCYAWETSFQSRIQTIRHNELSWFRKAQLL
YALNSFILNSIPVLVTVTSFGVFTLLGGELTPARAFTSLSLFSVLRFPLNMLPNLLSQVANANVSLQRLEELFSAE
ERNLQQNPPIVPGLPAISIKNGFFSWDPKEEKNPTLSNINVEIPVGSLVAIIGGTGEGKTSLISAMLGELPLVSDGN
AIIRGTVAYVPQISWIYNATVRENILFGSKFDHGRYSKAIDVTSLEHDLNFLPGRDFTEIGERGVNISGGQKQRV
SLARAVYSNSDVYIFDDPLSALDAHIAQEVFKNCIKEGLQGKTRVLVTNQLHFLPQVDKIILVSEGMIKEQGTFE
ELSKCGPLFQKLMENAGKMEQEVDSNKDSDNVTPLSDEAIVELPNDASYEKKGKLRKSVLVKKEERETGVVS
WKVLTRYTSALGGLWVVAILFACYTLTEALRISSSTWLSVWTSQDSTAASRAGYFLFIYAMFSFGQVSVALAN
SYWLIISSLRAAKRLHDAMLDKILHAPMVFFQTNPVGRIINRFAKDTGDIDTNVFNLMNMFLGQVWQLLSTFV
LIGTVSTISLWAIMPLLIFFYIAYIYYQSTAREVKRMDSITRSPVYAHFGESLNGLSSIRAYKVYDRMSNINGKFM
DNNIRFTLVNISSNRWLTIRLESLGGLMIWLIATFAVLQNARSENPTLIASTMGLLLSYTLNITNLLSGVLRQASR
AENSLNSVERVDTYINLETEGQSIIETNRPPPGWPTKGSIEFENVVLSYRPELPPVLHGLSFVVPSTEKIGVVGRT
GAGKSSMLNALFRIVELQSGRIIIDGCDISTFGLVDLRRVLTIIPQSPVLFSGTVRFNLDPFNEHSDADLWEALER
AHLKDVIRRNSFGLDAQVSEGGDNFSVGQRQLLSLARALLRRSKVLVLDEATAAVDVRTDALIQKTIRQEFHS
CTMLIIAHRLNTIIDCNRILLLDAGKVLEYNSPEKLLQNEETAFYKMVQSTGPANAEYLCSLVFGRKENNSNEF
NKESENSTRQLASTNWAAATQFAIASTLSSLHQHLQSPNTNDNKDILNRTKDAVVTLQEVLEGKHDDTIEETLT
QYHVPTDRWWSTLYKVIEGLAVLIRLPQDNNYNQLEPDFEGRSFD

>XP_024635182.1_ABCC12_Mt
MGFEPLVWYCKPEPNSIWSKTVDSAFGSYTPCAINTLVISTSNLVLMGLCLYRIWLIIFNAKAQRFCLKTNYYS
YILAMLASYCAFQPLLRLWTGNSAFNLNEETVFAPFEVTGLIIESLTWFSMIILILLETKVYIRQFRWLVRFGVIY
VLVGDIVMLELVLSVKDYSSRSALYLYISTVICQVLFGILLIVYIPNLVPYSGHTTFQADIPNNGEYEPLCGDDQV
CPEMRASFLSRLSFGWITPLMKQGYRKPITEKDVWKLDKWDQTDTLSEKFQKCWVSEFQSSNPWLLRALNNS
LGKRFWFGGIYKIGYDLSQLVAPILLNHLLDSMQNGDPSWIGYVCAFSIFVGVSVGILCETQYFQNVMRVGFRL
RSTLVAAIFRKSLRLTHESRKKFSYGKLMIMIATDANALQQICQQLHGLWSAPFRIIIAMVLLYQQLGVASLVG
SLMLVLIIPLQTFVIGKMKKLTKEGLQQTDKRVGLMNEILSTMDTVKCYAWETSFQSRIQSIRHEELSWFRKAY
LLYALNSFILNSIPVLVTVTSFGMFTLLGGELTPARAFTSLSLFTVLRSPLNSLPNLLNQVANANVSLQRLEELFL
AEERNLKQNPPIVPGLPAISIKNGYFSWDPKEEKKPTLSNINVEIPVGSLVAIIGGTGEGKTSLISAMLGELPLVSD
GNATIRGTAAYVPQISWIYNATVRDNILFGSNFDHGRYLKAIDVTSLEHDLNFLPGRDFTEIGERGINISGGQKQ
RVSLARAVYSNSDVYIFDDPLSALDAHIAQEVFRNCIKEGLQGKTRVLVTNQLHFLPQVDKIILVSEGMIKEQG
TFEELLKCGPLFQKLMENAGKMEQEVDGQDTNDVLPLDNGTIVELANDLSYGKKGKFQKSVLVKQEERETGV
VSWKVLMRYTSALGGIWVVSILLACYTLTEALRISSSTWLSVWTSQDSTAASRAGYFLFIYAIFSFGQVSVALA
NSYWLITASLRAAKRLHDAMLDKVLRAPMIFFQTNPVGRMINRFAKDTGDIDSNVYNLVNIVLGQLWQLLSTF
VLIGTVSTISLWAIMPLLIFFYVAYIYYQSTSREVKRMDSITRSPVYAHFGESMNGVSSIRAYKAYDRILHDNGK
FMDNNIRFTLANISTNRWLTIRLESLGGLMIWLIATFAVLQNARSENPTLVASTMGLLLSYTLNITSIMSSTLRQ
ASKAENSLNSVERVGTYIDLEAEGQSIIETNRPPPGWPTKGSIEFENVVLSYRPELPPVLHGLSFVVSSMEKIGVV
GRTGAGKSSMLNALFRIVELQSGRIIIDGCDISTFGLADLRRVLTIIPQSPVLFSGTVRFNLDPFNEYNDVDIWEA
LERAHMKDVIRRNQFGLDAQVSEGGDNFSVGQRQLLSLARALLRRSKVLVLDEATASVDVRTDALIQKTIRQE
FNSCTMLIIAHRLNTVVDCNRILLLDAGKVLEYNSPKELLQNEETAFYKMVQSTGPANAEYLCSLVFGRKENN
SNEYNKESENGMRQLASTDWTAATQFAIASTLSSLHQHLQSPNTKDDKDILNRT

>XP_025884378.1_ABCC12-l_X1_Sl
MIFDALVWYCKPEANGIWAKETDSAFGAYTPCAIESADCISNVVLFVLCAYRLWLVRMDHEVRRFQLTSKCY
NYILILLGSCCAAEPLLRLFMGISIFNLDAETDLAPFEMVSLSIETLAWMSIIVMNLVETKVYIKEFRWYVRFGVI

YALVGELVILNFVFSMQSFYSRFTLYIYYSSVICQIVFGALLLVHLPHLNPFPGYIPLRSESVDDKTDETILGEDHI
CPERYACILSRISFGWITPLLRQGYNRPITEKDVWKLDSWDKTETLSARFQRCWTEESRRKKPWLLRALNCSLG
GRFWYGGLFKVGSDLCQFVGPLLLNRLLESLERRDPAWIGHLYAFLIFVGVSFGVLCEAQYFQNVMRVGFRM
RSTLVAAIFRKSVRLTLEDHKQFPSGKITNMITTDANTLQQVCQQLHVLWSAPFRIVIAMVLLYQQLGLASLLG
ALMLVVMIPMQTIIVSYMRKLSKEGLQYTDKRVGLTNEILAAMAVVKCYAWEKSFQSKVQGLRNGELSWFR
KAQLLAAFNNFMLNSIPVLVTVISFGGFTLLGGNLTAARAFSSLSLFAILRFPLNMLPNIITQVVNSNVSLQRME
ELFLAEERVLLPNPPLEPGLPTISIRDGFFSWDSKAEHPTLSNINLDIPVGELVAIVGGTGEGKTSLISAILGEFPPL
GNASVTIRGSIAYVPQVSWIFNATVRENILFGSNFEPTRYWKAINVTALDHDLELLPGGDLTEIGERGVNISGGQ
RQRVSMARAVYKNSDIYIFDDPLSALDAHVSHQVFKNCIKEELQGKTRVLVTNQLHFLPQVDRIILVSEGMVK
EDGTFEKLLEHGTLFPKLMENAGKMESYGVETEYDPNFDSESSQSSSTRQEHQQDVTSVTKRKAGKSVLIRQE
ERETGIINWSVLMRYKDALGGLWVVMILFGCYTLTEILRILSSSWLSVWTKASASQSNGAGFYILVYAILSFSQ
VIVTLANSFWLIISSLNAAKGIHDTMLHSILRAPMVFFHTNPSGRIINRFAKDLGDIDRNVANIGNTCLSQLWQL
LSTFVLIGVVSSISLWAIMPLLILFYAAYLYYQNTSREVKRLDSITRSPVYAQFGEAITGLSTIRAYKAHDQLAAI
NGKSMDNNIRFTLANTSTNRWLTIRLETLGGIMIWLTATFAVIQNGRADDKVAVAATMGLLLSYSLNITTLLSN
TLRQASRAENSLNAVERVGTYIDLPSEAQNVICSQPPPHWPSSGFIKFEDVVLRYRPGLPPVLHGLSFEISSGQK
VGIVGRTGAGKSSMLNALFRIVELERGRILIDDCDVANIELTDLRSALSIIPQSPVLFSGTVRFNLDPFNEHNDAD
LWEALERAHLKDVIRRSTFGLDAEVSEGGENFSVGQRQLLSLARAILRRSKILVLDEATAAVDVRTDALIQRTI
REEFKTCTMLIIAHRLNTIIHTNCILVLDAGKVVEYDTPQNLFLNERSVFSNIVQSTGAANAQYLRNLVFYKERD
DMFMEEELMHINGMSR

>AtABCG40
MEGTSFHQASNSMRRNSSVWKKDSGREIFSRSSREEDDEEALRWAALEKLPTFDRLRKGILTASHAGGPINEIDI
QKLGFQDTKKLLERLIKVGDDEHEKLLWKLKKRIDRVGIDLPTIEVRFDHLKVEAEVHVGGRALPTFVNFISNF
ADKFLNTLHLVPNRKKKFTILNDVSGIVKPGRMALLLGPPSSGKTTLLLALAGKLDQELKQTGRVTYNGHGM
NEFVPQRTAAYIGQNDVHIGEMTVRETFAYAARFQGVGSRYDMLTELARREKEANIKPDPDIDIFMKAMSTAG
EKTNVMTDYILKILGLEVCADTMVGDDMLRGISGGQKKRVTTGEMLVGPSRALFMDEISTGLDSSTTYQIVNS
LRNYVHIFNGTALISLLQPAPETFNLFDDIILIAEGEIIYEGPRDHVVEFFETMGFKCPPRKGVADFLQEVTSKKD
QMQYWARRDEPYRFIRVREFAEAFQSFHVGRRIGDELALPFDKTKSHPAALTTKKYGVGIKELVKTSFSREYLL
MKRNSFVYYFKFGQLLVMAFLTMTLFFRTEMQKKTEVDGSLYTGALFFILMMLMFNGMSELSMTIAKLPVFY
KQRDLLFYPAWVYSLPPWLLKIPISFMEAALTTFITYYVIGFDPNVGRLFKQYILLVLMNQMASALFKMVAAL
GRNMIVANTFGAFAMLVFFALGGVVLSRDDIKKWWIWGYWISPIMYGQNAILANEFFGHSWSRAVENSSETL
GVTFLKSRGFLPHAYWYWIGTGALLGFVVLFNFGFTLALTFLNSLGKPQAVIAEEPASDETELQSARSEGVVEA
GANKKRGMVLPFEPHSITFDNVVYSVDMPQEMIEQGTQEDRLVLLKGVNGAFRPGVLTALMGVSGAGKTTL
MDVLAGRKTGGYIDGNITISGYPKNQQTFARISGYCEQTDIHSPHVTVYESLVYSAWLRLPKEVDKNKRKIFIE
EVMELVELTPLRQALVGLPGESGLSTEQRKRLTIAVELVANPSIIFMDEPTSGLDARAAAIVMRTVRNTVDTGR
TVVCTIHQPSIDIFEAFDELFLLKRGGEEIYVGPLGHESTHLINYFESIQGINKITEGYNPATWMLEVSTTSQEAAL
GVDFAQVYKNSELYKRNKELIKELSQPAPGSKDLYFPTQYSQSFLTQCMASLWKQHWSYWRNPPYTAVRFLF
TIGIALMFGTMFWDLGGKTKTRQDLSNAMGSMYTAVLFLGLQNAASVQPVVNVERTVFYREQAAGMYSAM
PYAFAQVFIEIPYVLVQAIVYGLIVYAMIGFEWTAVKFFWYLFFMYGSFLTFTFYGMMAVAMTPNHHIASVVS
SAFYGIWNLFSGFLIPRPSMPVWWEWYYWLCPVAWTLYGLIASQFGDITEPMADSNMSVKQFIREFYGYREGF
LGVVAAMNVIFPLLFAVIFAIGIKSFNFQKR

>GmPDR12
MEGGGSSFRIGSSSIWRNSDAAEIFSNSFHQENDEEALKWAAIQKLPTVARLRKALITSPDGESNEIDVKKLGLQ
EKKALLERLVKTAQEDNEKFLLKLKDRIDRVGIDLPTIEVRFENLSIEAEARAGTRALPTFTNFIVNILEGLLNSL
HVLPNRKQHLNILEDVSGIIKPGRMTLLLGPPSSGKTTLLLALAGKLDPKNKVLWKGTYNGHGVNEFVPQRTA
AYVNQNDLHVAELTVRETLVFSARVQGVGPRYDLLAELSRREKEANIKPDPDIDAYMKAVASEGQKANMITD
YILRILGLEVCADTVVGNAMLRGISGGQRKRVTTGEMLVGPAKALFMDEISTGLDSSTTFQIVNSLKQYVHILK
GTTVISLLQPAPETYNLFDDIILLSDSHIVYQGPREHVLEFFELMGFKCPQRKGVADFCKKLHQGKIRSSTGHTK
DHLYRFFTAKEFSEAHKSFHIGRSLVEELATEFDKSKSHPAALTTKMYGVGKWELLKACLSREYLLMKRNSFV
YTFKLCQLAVLAIIAMTIFLRTEMHRDSVTHGGIYVGALFYGVVVIMFNGLAELSMVVSRLPVFYKQRDYLFFP
SWVYALPAWILKIPLTFVEVGVWVFLTYYAIGFDPYVGRLFRQYLVLVLELVNQMASALFRLVAAVGREMTV
ALTLGSFTLAILFAMSGFVLSKENIKKWWLWGFWISPMMYGQNAMVNNEFLGKRWRHFLPNSTEALGVEILK
SRGFFTQSYWYWIGVGALIGYTLLFNFGYILALTYLNPLGKHQAVISEEPQINDQSGDSKKGTNVLKNIQRSFSQ
HSNRVRNGKSLSGSTSPETNHNRTRGMILPSETHSITFDDVTYSVDMPVEMRNRGVVEDKLALLKGVSGAFRP
GVLTALMGVTGAGKTTLMDVLAGRKTGGYIGGNITISGYPKKQETFARISGYCEQNDIHSPHVTVYESLLYSA
WLRLSPEINADTRKMFIEEVMELVELKALRNALVGLPGINGLSTEQRKRLTIAVELVANPSIIFMDEPTSGLDAR
AAAIVMRTVRDTVDTGRTVVCTIHQPSIDIFESFDELLLMKQGGQEIYVGPLGHHSSHLINYFEGIQGVNKIKDG
YNPATWMLEVSTSAKEMELGIDFAEVYKNSELYRRNKALIKELSTPAPGSKDLYFPSQYSTSFLTQCMACLWK
QHWSYWRNPLYTAIRFLYSTAVAAVLGSMFWDLGSKIDKQQDLFNAMGSMYAAVLLIGIKNANAVQPVVAV
ERTVFYREKAAGMYSALPYAFAQVLIELPYVLVQAVVYGIIIYAMIGFEWTVTKVFWYQFFMYFTFLTFTYYG
MMSVAVTPNQHISSIVSSAFYAVWNLFSGFIVPRPRIPVWWRWYSWANPVAWSLYGLVASQYGDIKQSMESS
DGRTTVEGFVRSYFGFKHDFLGVVAAVIVAFPVVFALVFAISVKMFNFQRR

>LjABCG1
MEGGDIYRAGHSLRANSTSVWRNSTMEAFSRSSRHEEDDEEALKWAALEKLPTYNRLRKGLLATSRGAANQI
DVSDLGFQERQKLLDKLIKVAEEDNEKFLLKLKERIDRVGIDIPTIEVRYEHLNVEAEAYVGSRALPSFVNFATN
IVESLFTSLHILSTKKRHVTILKDVSGIIKPRRMTLLLGPPGSGKTTLLLAMSGKLDPNLKVSGRVTYNGHELNE
FVPQRTAAYISQHDVHIGEMTVRETLAFSARCQGVGSRYDLLAELSRREKEANIKPDPYLDVFMKAATTGGQE
ANIVTDYVLKILGLDICADTMVGDEMLRGISGGQRKRVTTGEMLVGPANALFMDEISTGLDSSTTFQIVSSLRQ
YVHILNGTAVISLLQPAPETFELFDDIILISDGQIVYQGPREFVLDFFETLGFKCPERKGAADFLQEVTSKKDQEQ
YWVRRDEPYRFVTVTQFAEAFQAFHVGRKSGDELGIPFDKSKSHPAALVKKQYGINKKELLKANFSREYLLM

KRNSFVYIFKICQLTFMAIMTMTLFLRTEMHRDSLDDGSVFSGALFFALGTIMFNGMAEISMSIAKLPIFYKQRD
LLFYPSWAYAIPNWILKIPVTFVEVAVWVFLTYYVIGFDPNALRFVKQYALLLLVNQMASGLFRAIAALGRNM
IVANTFGSFALLMLITLGGFILSRKDIKGWWIWGYWTSPLMYGQNAIMTNEFLGNSWSHFTKNSNKSLGLQAL
ESRGFFTHAYWYWIGIGALTGFMFLYNIIYTLALTFLNPFDKAQATINEESEDNTPNGRAPEVELPRIESSGNAD
SAVDSSHGRKRGMVLPFEPHSIAFDDVVYSVDMPQEMRDQGVMEDRLVLLKGVSGAFRPGVLTALMGVSGA
GKTTLMDVLAGRKTGGHIDGSVKVSGYPKNQETFARISGYCEQNDIHSPQVTVYESLLYSAWLRLPAEVDSNT
RKTFIEEVIELVELNPLRNSLVGLPGVSGLSTEQRKRLTIAVELVANPSIIFMDEPTSGLDARAAAIVMRTVRNTV
DTGRTVVCTIHQPSIDIFEAFDELFLMKRGGQEIYVGPLGRHSSKLIEYFESIEGVNKIKDGYNPATWMLEVTSS
AQEVTIGVDFHQTYKNSELYRRNKQLIAELGIPAPGSNDLYFPTQYSQSFLVQCLACLWKQHWSYWRNPPYTA
VRFFFTTFIALIFGTMFWDLGGKYKNRQDLFNALGSMYTAVLFLGIQNSASVQPVVAVERTVFYRERAAGMYS
ALPYALAQVIIEIPYVFAQALSYGLIVYAMMGFEWTVEKFFWYLFFMFFTLCYFTYYGMMTVAVTPNHHVASI
VAAAFYAIWNLFSGFVVPRPRIPVWWRWYYWACPVAWTIYGMVASQFGDIEHILESDDVSVKEFIRSYFGMK
HDFIGVCAVVVVGFAVGFAFIFAVSIKVFNFQRR

>Lr34
MEGLARETNPSSHHQDFTACASDERPDESELELASRQRQNGAANTEHVSENMLLDSSKLGALKRREFFDNLLK
NLEDDHLRFLRGQKERIDRVDVKLPAIEVRYNNLFVEAECRVTKGNHLPSLWNSTKGAFSGLVKLLGFETERA
KTNVLEDVSGIIKPCRLTLLLGPPGCGKSTLLRALAGKLDKSLKVTGDISYNGYELHEFVPEKTAVYINQHDLHI
AEMTVRETLDFSAQCQGVGRRPKILKEVNTRESVAGIIPDADIDLYMKVVAVEASERSLQTDYILKIMGLEICA
DTMVGDAMRRGISGGQKKRLTTAEMIVGPASAYFMDEISNGLDSSTTFQIINCFQQLTNISEYTMVISLLQPTPE
VFDLFDDLILMAEGKIIYHGPRNEALNFFEECGFICPERKAAADFLQEILSWKDQQQYWLGPHESYRYISPHELS
SMFRENHRGRKLHEQSVPPKSQLGKEALAFNKYSLQKLEMFKACGAREALLMKRNMFVYVFKTGQLAIIALV
TMSVFLRTRMTISFTHANYYMGALFFSIMIMLNGIPEMSMQIGRLPSFYKQKSYYFYSSWAYAIPASVLKVPISI
LDSLVWISITYYGIGYTPTVSRFFCQFLILCLLHHSVTSQHRFIASYFQTPIVSFFYLFLALTVFLTFGGFILPKTSM
PGWLNWGFWISPMTYAEISIVINEFLAPRWQKESIQNITIGNQILVNHGLYYSWHYYWISFGALLGSILLFYIAFG
LALDYRTPTEEYHGSRPTKSLCQQQEKDYTIQNESDDQSNISKAKVTIPVMHLPITFHNLNYYIDTPPEMLKQG
YPTRRLRLLNNITGALRPGVLSALMGVSGAGKTTLLDVLAGRKTGGYIEGDIRIGGYPKVQETFVRILGYCEQV
DIHSPQLTVEESVTYSAWLRLPSHVDEQTRSKFVAEVLETVELDQIKDVLVGSPQKNGLSMEQRKRLTIAVELV
SNPSIILMDEPTTGLDTRSAAIVIRAVKNICETGRTVVCTIHQPSTEIFEAFDELILMKSGGKTIYSGPIGERSCKVI
EYFEKISGVPKIKSNCNPATWMMDVTSTSMEVQHNMDFAILYEESSLHREAEDLVEQLSIPLPNSENLCFSHSF
AQNGWIQLKACLWKQNITYWRSPQYNLRRIMMTVISALIYGILFWKHAKVLNNEQDMLSVFGAMYLGFTTIG
AYNDQTIIPFSTTERIVMYRERFAGMYSSWSYSFAQAFIEIPYVFIQVVLYTLIVYPSTGYYWTAHKFLWFFYTT
FCSILSYVYVGLLLVSITPNVQVATILASFFNTMQTLFSGFILPAPQIPKWWTWLYYLTPTSWALNALLTSQYGN
IEKEVKAFGETKSVSIFLNDYFGFHQDKLSVVAAVLVAFPFVLIILFSLSIEKLNFQKR

>MtABCG10
MEGTDIYRATNSLRARSSTVWRQSGVEVFSKSSREEDDEEALKWAALEKLPTYNRLRKGLLTASHGGAHEVD
VGDLAFQDKQKLLERLVKVAEEDNEGFLLKVKERVDRVGLDIPTIEVRYNNLKIDAEAFVGSRALPSFINAATN
VIEGVLNFLHIIPTKKRHVAILKDVSGIVKPRRMTLLLGPPGSGKTTLLLALSGKLDPSLQLTGSVTYNGHGLNE
FVPQRTAAYISQHDVHIGEMTVRETLAFSARCQGVGSRYDMLSELSRREKAANIKPDPDIDVYMKAIATEGQE
YSISTDYVLKILGLDICADTMVGDEMLRGISGGQRKRVTTGEMLVGPANIVSSLRQYVHIMNGTAVISLLQPAP
ETYDLFDDIILISDGQVVYHGPREYVLDFFETMGFKCPERKGAADFLQEVTSKKDQAQYWVRRDQPYRFVTVT
QFAEAFQSFHIGRKLAEELSVPFDKTKSHPAALTTKEYGLNKTELLKANFSREYLLMKRNSFVYIFKLTQLFIM
ALIAMTLFFRTEMHRNNQDDAGVYAGALFFTLVTMMFNGMSEISMTIAKLPVYYKQRDLLFYPSWAYAIPSW
ILKIPISLVEVSLWVFLTYYVIGFDPNVGRMFKQFLVLFFMSQMASGLFRAIASLGRNMIVANTFGSFAVLTLLA
LGGFILSRKDIKGWWIWGYWISPLMYGQNALMANEFLGNSWHNATFDLGKNYLDTRGFFPHAYWYWIGVG
GLVGFVFLFNAAFGVALAVLGPFDKPSATITEEDSEDDSSTVQEVELPRIESSGRRDSVTESSHGKKKGMVLPFE
PHSITFDDIVYSVDMPAEMKEQGVTEDRLVLLKGVSGAFRPGVLTALMGVSGAGKTTLMDVLAGRKTGGYID
GDIKVSGYPKKQETFARISGYCEQNDIHSPHVTVYESLLYSAWLRLPSGVDSNTRKMFIDEVMDLVELNSLRNS
LVGLPGVSGLSTEQRKRLTIAVELVANPSIIFMDEPTSGLDARAAAIVMRTVRNTVDTGRTVVCTIHQPSIDIFEA
FDELFLMKRGGQEIYVGPLGRHSTHLIKYFESIDGVSKIKDGYNPATWMLEVTTTAQELNLGVDFTDLYKNSD
LYRRNKQLIQELSVPAPGSKDLHFPTQFSQSFLVQCQACLWKQRWSYWRNPPYTAVRFFFTTFIGLMFGTMFW
DLGGKHSSRQDLLNAVGSMYTAVLFLGVQNSSSVQPVVAVERTVFYREKAAGMYSALPYAFSQILVELPYVF
AQAVTYGAIVYAMIGFDWTAEKFLWYLFFMYFTLLYFTFYGMMAVAVTPNHHVASIVAAAFYAIWNLFSGF

VVPRPSIPIWWRWYYWACPVAWTIYGLVASQFGDITTVMSTEGGKDVKTFLDDFFGIQHDFIGWCALVVGGIA
VGFAFIFAVAIKSFNFQKR

>NbPDR1_a
MEPANLSNLRGSSLRGSMRGSLRANSNSIWRNNGVEIFSRSSRDEDDEEALKWAALEKLPTFDRLRKGLLFGS
QGAAAEVDINDLGFQERKNLLERLVKVADEDNEKFLLKLKNRIDRVGIDLPTIEVRYEHLNIDADAYVGSRGL
PTFMNFMTNFVETLLNSLHILPSRKRQLTILKDISGIIKPCRMTLLLGPPSSGKTTLLLALAGKLDPALKVTGKVS
YNGHELHEFVPQRTAAYISQHDLHIGEMTVRETLEFSARCQGVGSRFEMLAELSRREKAANIKPDADIDVYMK
AAATEGQEANVVTDYVLKILGLDICADTMVGDDMIRGISGGQKKRVTTGEMLVGPSKALFMDEISTGLDSSTT
YSIVNSLRQSVQILKGTAVISLLQPAPETYNLFDDIILLSDGYIVYQGPRDDVLEFFESMGFKCPHRKGVADFLQ
EVTSKKDQQQYWSKRNEPYRFITSKEFAEAYQSFHVGRKLGDELATPFDKTKCHPAALTNEKYGIGKKELLKV
CTERELLLMKRNSFVYMFKFSQLTIMALITMTLFFRTEMPRDTTDDGAIYAGALFFVVIMIMFNGMSELAMTIF
KLPVFYKQRDLLFFPSWAYAIPSWILKIPVTLVEVGLWVILTYYVIGFDPNITRFLKQFLLLIVVNQMASGLFRFI
GAVGRTMGVASTFGSFALLLQFALGGFVLSRDDVKSWWIWGYWTSPMMYSVNSILVNEFDGKKWNHIVPGG
NETLGATVVKSRGFFPEAYWYWIGVGALVGFTVVFNFCYSVALAYLNPFDKPQAVLAEDGENAENVEVSSQI
TSTDGGDSISESQNNKKGMVLPFEPHSITFDDVVYSVDMPQEMKEQGAGQDRLVLLKGVSGAFRPGVLTALM
GVSGAGKTTLMDVLAGRKTGGYIDGEIKISGYPKKQETFARISGYCEQNDIHSPYVTVYESLVYSAWLRLPQD
VDEKTRKMFVDEVMELVELGPLRSALVGLPGVNGLSTEQRKRLTIAVELVANPSIIFMDEPTSGLDARAAAIV
MRTVRNTVDTGRTVVCTIHQPSIDIFEAFDELFLMKRGGQEIYVGPLGRHSCHLIKYFESNPGVAKIKEGYNPAT
WMLEVTASAQEMLLGVDFTEVYKNSDLYRSNKALISELGVPRPGSKDLHFETQYSQSFWTQCMACLWKQHW
SYWRNPAYTAVRFIFTTFIALIFGTMFWDLGTKVSESQDLLNAMGSMYAAVLFLGVQNASSVQPVVAVERTVF
YRERAAGMYSAIPYAFGQVAIEIPYIFVQSVFYGIIVYAMIGFEWDVGKFFWYLFIMFFTLLYFTFYGMMSVAV
TPNQNVASIVAAFFYGVWNLFSGFIIPRPRMPVWWRWYYWANPVAWTLYGLVASQFGDIQTKLSDNETVKQF
LRRFFGFRHDFLGVVAAVLSAYVFVFAFTFAFAIKAFNFQRR

>NbPDR1_b
MEPANLSNLRGSSLRGSTRGSLRANSNTIWRNNGVEIFSRSSRDEDDEEALKWAALEKLPTFDRLRKGLLFGSQ
GAAAEVDIYDLGFQERKNLLERLVKVADEDNEKFLLKLKNRIDRVGIDLPTIEVRYEHLNIDADAYVGSRGLPT
FMNFMTNFVETLLNSLHILPSRKRQLTILKDISGIIKPCRMTLLLGPPSSGKTTLLLALAGKLDPALKVTGKVSY
NGHELHEFVPQRTAAYISQHDLHIGEMTVRETLEFSARCQGVGSRFEMLAELSRREKAANIKPDADIDIYMKA
AATEGQEANVVTDYVLKILGLDICADTMVGDDMIRGISGGQKKRVTTGEMLVGPSKALFMDEISTGLDSSTTY
SIVNSLRQSVQILKGTAVISLLQPAPETYNLFDDIILLSDGYIVYQGPRDDVLEFFDSMGFKCPERKGVADFLQE
VTSKKDQQQYWSKRNKPYRFITSKEFAEAYQSFHVGRKLGDELATPFDKTKCHPAALTNEKYGIGKKELLKV
CTERELLLMKRNSFVYVFKFSQLTIMALITMTLFFRTEMPRDTTDDGAIYAGALFFVVIMIMFNGMSELAMTIF
KLPVFYKQRDLLFFPSWAYAIPSWILKIPVTLVEVGLWVILTYYEIGFDPNITRLLKQFLLLIVVNQMASGLFRFI
GAVGRTMGVASTFGSFALLLQFALGGFVLSRDDVKSWWIWGYWTSPMMYSVNSILVNEFDGKKWNHIVPGG
NETLGTTVVKSRGFFPEAYWYWIGVGALVGFTVVFNFCYSLALAYLNPFDKPQAVLPEDGENAENVEVSSQIT
STDGGDSISEGQNNNKKGMVLPFEPHSITFDDVVYSVDMPQEMKEQGADQDRLVLLKGVSGAFRPGVLTALM
GVSGAGKTTLMDVLAGRKTGGYIDGDIKISGYPKKQETFARISGYCEQNDIHSPYVTVYESLVYSAWLRLPQD
VDEKTRKMFVDEVMELVELGPLRSALVGLPGVNGLSTEQRKRLTIAVELVANPSIIFMDEPTSGLDARAAAIV
MRTVRNTVDTGRTVVCTIHQPSIDIFEAFDELFLMKRGGQEIYVGPLGRHSCHLIKYFESNPGVAKIKEGYNPAT
WMLEVTASAQEMMLGVDFTEVYKNSDLYRRNKALISELGVPRPGSKDLHFETQYSQSFWTQCMACLWKQH
WSYWRNPAYTAVRFIFTTFIALIFGTMFWDLGTNVSKSQDLLNAMGSMYAAVLFLGVQNASSVQPVVAVERT
VFYRERAAGMYSAIPYAFGQVSIEIPYIFVQSVFYGIIVYAMIGFEWDVGKFFWYLFIMFFTLLYFTFYGMMSV
AVTPNQNVASIVAAFFYGVWNLFSGFIIPRPRMPVWWRWYYWANPVAWTLYGLVASQFGDIQTKLSDNETV
EQFLRRYFGFKHDFLGIVAAVLTAYVFVFAFTFAFAIKAFNFQRR

>NbPDR2a
MEPADLSNLRGRSLRASIRGSMRGSIRENSNSIWRNNGAEVFSHSARDEDDEEALKWAALEKLPTYDRLRKGI
LFGSQGAAAEVDVDDLGVLERKNLLERLVKVADEDNEKFLLKLKNRIDRVGIDFPSIEVRFEHLNIDADAYVG
SRALPTFTNFISNFVEGLLDSIHILPSKKRQVTILKDVSGIVKPCRMTLLLGPPGSGKTTLLLALAGKLDSALKVT
GKVTYNGHELHEFVPQRTATYISQHDLHIGEMTVRETLEFSARCQGVGSRYEMLAELSRREKAANIKPDADID
MFMKAASTEGQEAKVVTDYILKILGLDICADTMVGDQMIRGISGGQKKRVTTGEMIVGPSKALFMDEISTGLD
SSTTYSIVNSLKQSVRIMKGTALISLLQPAPETYNLFDDIILLSDGYIVYEGPRDYVLEFFESMGFKCPERKGAAD

FLQEVTSKKDQQQYWVRRDEPYRFITSKEFAEAYQSFHVGRKVRDELATTFDKSKSHPAALTTQKYGIGKRQL
LKVCTERELLLMQRNSFVYLFKFFQLLIIALMTMTIFFRTKMPRDTAEDGGIYSGALFFVVIMIMFNGLSELPMT
LYKLPVFYKQRDFLFYPSWAYAIPSWILKIPVTFAEVGMWVFLTYYVMGFDPNVGRFFKQFLLLLLVNQMAS
ALFRFIAAVGRTMGVASTFGAFALLLQFALGGFILARKDVKDWWIWGYWTSPLMYSVNAILVNEFDGQKWK
HIVAGGTEPLGAAVVRARGFFPDAYWYWIGVGALAGFTVMFNIAYSVALAYLNPFDKPQATISDESENNESEL
SPQITSTQEGDSVSENKKKGMVLPFDPHSITFDEVVYSVDMPPEMRESGTSDNRLVLLKSVSGAFRPGVLTALM
GVSGAGKTTLMDVLAGRKTGGYIDGSIKISGYPKKQETFARISGYCEQNDIHSPYVTVFESLVYSAWLRLPQDV
NEEKRMMFVEEVMDLVELTPLRSALVGLPGVNGLSTEQRKRLTIAVELVANPSIIFMDEPTSGLDARAAAIVM
RAVRNTVDTGRTVVCTIHQPSIDIFEAFDELFLMKRGGQEIYVGPLGRQSCHLIKYFESIPGVSKIVEGYNPATW
MLEVTASSQEMALGVDFTDLYKKSDLYRRNKALIDELSVPRPGTSDLHFDSEFSQPFWTQCMACLWKQHWSY
WRNPAYTAVRFIFTTFIALIFGTMFWDIGTKVSRNQDLINAMGSMYAAVLFLGVQNSSSVQPVVSVERTVFYR
EKAAGMYSAIPYAFAQVLIEIPYIFVQATVYGLIVYSMIGFEWTAAKFFWYFFFMFFTFLYFTFFGMMTVAVTP
NQNVASIVAGFFYTVWNLFSGFIVPRPRIPIWWRWYYWGCPIAWTLYGLVASQFGDLQDPLTDQNQTVEQFLR
SNFGFKHDFLGVVAAVIVAFAVVFAFTFALGIKAFNFQRR

>NbPDR2b
MEPADLSNLRGRSLRASMRGSIRENSISIWRNNGADVFSRSARDEDDEEALKWAALERLPTYDRLRKGILFGSQ
GAAAEVDVDDLGVLERKNLLERLVKVADEDNEKFLLKLKNRIDRVGIDFPSIEVRFEHLNIDADAYVGSRALP
TFTNFISNFIEGLLDSIHILPSRKRQVTILKDVSGIVKPCRMSLLLGPPGSGKTTLLLALAGKLDSALKVTGKVTY
NGHELHEFVPQRTAAYISQHDLHIGEMTVRETLEFSARCQGVGSRYEMLAELSRREKAANIKPDADIDMFMKA
ASTEGQEAKVVTDYILKILGLDICADTMVGDQMIRGISGGQKKRVTTGEMIVGPSKALFMDEISTGLDSSTTYSI
VNSLKQSVRIMKGTALISLLQPAPETYNLFDDIILLSDGYIVYQGPREDVLNFFESMGFKCPERKGAADFLQEVT
SKKDQQQYWIRRDEPYRFITSKEFAEAYQSSHVGRKVSDELATTFDKSKSHPAALTTQKYGIRKKQLLKVCTE
RELLLMQRNSFVYLFKFFQLLVIALMTMTIFFRTKMPRDTAADGNIYSGALFFVVIMIMFNGLSELPMTLYKLP
VFYKQRDFLFYPSWAYAIPSWILKIPVTFAEVGMWVFLTYYVIGFDPNVGRFFKQFLLLLLVNQMASALFRFIA
AVGRTMGVASTFGAFALLLQFALGGFILARNDVKDWWIWGYWTSPLMYSVNAILVNEFDGKKWKHIVAGGT
EPLGAAVVRARGFFPDAYWYWIGIGALAGFTVMFNIAYSVALAYLNPFDKQQATISDESENNENTESSPQITST
KEGDSVNENKKKGMVLPFDPHSITFDEVVYSVDMPPEMRESGTSENRLVLLKSVSGAFRPGVLTALMGVSGA
GKTTLMDVLAGRKTGGYIDGSIKISGYPKKQETFARISGYCEQNDIHSPYVTVFESLVYSAWLRLPQDVDEQKR
MMFVEEVMDLVELTSLRSALVGLPGVNGLSTEQRKRLTIAVELVANPSIIFMDEPTSGLDARAAAIVMRAVRN
TVDTGRTVVCTIHQPSIDIFEAFDELFLMKRGGQEIYVGPLGRQSCHLIKYFESIPGVSKIVEGYNPATWMLEVT
ASSQEMALGVDFTELYKKSDLYRRNKALIDELSMPQPGTSDLHFDSEFSQPFWTQCMACLWKQHWSYWRNP
AYTAVRFIFTTFIALIFGTMFWDIGTKVSRNQDLINAMGSMYAAVLFLGVQNSSSVQPVVSVERTVFYREKAA
GMYSAIPYAFAQVLIEIPYIFVQAVVYGLIVYSMIGFEWTVAKFFWYFFFMFFTFLYFTFFGMMTVAVTPNQNV
ASIVAGFFYTVWNLFSGFIVPRPRIPIWWRWYYWGCPIAWTLYGLVASQFGELQDPLTDQNETVEQFLRSNFGF
KHDFLGVVAAVIVAFAVVFAFTFALGIKAFNFQRR

>NP_001237697.2_PDR-l_Gm
MEGGGSSFRIGSSSIWRNSDAAEIFSNSFHQENDEEALKWAAIQKLPTVARLRKALITSPDGESNEIDVKKLGLQ
EKKALLERLVKTAQEDNEKFLLKLKDRIDRVGIDLPTIEVRFENLSIEAEARAGTRALPTFTNFIVNILEGLLNSL
HVLPNRKQHLNILEDVSGIIKPGRMTLLLGPPSSGKTTLLLALAGKLDPKLKFSGKVTYNGHGMNEFVPQRTA
AYVNQNDLHVAELTVRETLAFSARVQGVGPRYDLLAELSRREKEANIKPDPDIDAYMKAVASEGQKANMITD
YILRILGLEVCADTVVGNAMLRGISGGQRKRVTTGEMLVGPAKALFMDEISTGLDSSTTFQIVNSLKQYVHILK
GTTVISLLQPAPETYNLFDDIILLSDSHIVYQGPREHVLEFFELMGFKCPQRKGVADFLQEVTSRKDQEQYWAH
KDQPYRFVTAKEFSEAHKSFHIGRSLGEELATEFDKSKSHPAALTTKMYGVGKWELLKACLSREYLLMKRNSF
VYTFKLCQLAVLAIIAMTIFLRTEMHRDSVTHGGIYVGALFYGVVVIMFNGLAELSMVVSRLPVFYKQRDYLF
FPSWVYALPAWILKIPLTFVEVGVWVFLTYYAIGFDPYVGRLFRQYLVLVLVNQMASALFRLVAAVGREMTV
ALTLGSFTLAILFAMSGFVLSKENIKKWWLWGFWISPMMYGQNAMVNNEFLGKRWRHFLPNSTEALGVEILK
SRGFFTQSYWYWIGVGALIGYTLLFNFGYILALTYLNPLGKHQAVISEEPQINDQSGDSKKGTNVLKNIQRSFSQ
HSNRVRNGKSLSGSTSPETNHNRTRGMILPSEPHSITFDDVTYSVDMPVEMRNRGVVEDKLALLKGVSGAFRP
GVLTALMGVTGAGKTTLMDVLAGRKTGGYIGGNITISGYPKKQETFARISGYCEQNDIHSPHVTVYESLLYSA
WLRLSPEINADTRKMFIEEVMELVELKALRNALVGLPGINGLSTEQRKRLTIAVELVANPSIIFMDEPTSGLDAR
AAAIVMRTVRNTVDTGRTVVCTIHQPSIDIFESFDELLLMKQGGQEIYVGPLGHHSSHLINYFEGIQGVNKIKDG
YNPATWMLEVSTSAKEMELGIDFAEVYKNSELYRRNKALIKELSTPAPGSKDLYFPSQYSTSFLTQCMACLWK

QHWSYWRNPLYTAIRFLYSTAVAAVLGSMFWDLGSKIDKQQDLFNAMGSMYAAVLLIGIKNANAVQPVVAV
ERTVFYREKAAGMYSALPYAFAQVLIELPYVLVQAVVYGIIIYAMIGFEWTVTKVFWYLFFMYFTFLTFTYYG
MMSVAVTPNQHISSIVSSAFYAVWNLFSGFIVPRPRIPVWWRWYSWANPVAWSLYGLVASQYGDIKQSMESS
DGRTTVEGFVRSYFGFKHDFLGVVAAVIVAFPVVFALVFAISVKMFNFQRR

>NP_001274820.1_PDR1_-l_St
MEPSDLSNLRGRSIRGSMRGSMRENSNSIWRNNGVEVFSRSNRDEDDEEALKWAALEKLPTYDRLRKGILFGS
QGVAAEVDVDDLGVQQRKNLLERLVKVADEDNEKFLLKLKNRIDRVGIDFPSIEVRFEHLNIEADAYVGSRAL
PTFTNFISNFIESLLDSIHIFPSKKRSVTILKDVSGYVKPCRMTLLLGPPGSGKTTLLLALAGKLDSDLRVTGKVT
YNGHELHEFVPERTAAYISQHDLHIGEMTVRETLEFSARCQGVGSRYEMLAELSRREKAANIKPDVDIDMFMK
ILGLDICADTMVGDQMIRGISGGQKKRVTTGEMIVGPSKALFMDEISTGLDSSTTYSIVNSLKQSVQILKGTALIS
LLQPAPETYNLFDDIILLSDGYIVYQGPREDVLEFFESMGFKCPDRKGVADFLQEVTSKKDQQQYWVRRDEPY
RFITSKEFAEAYQSFHVGRKVSNELSTAFDKSKSHPAALTTEKYGIGKKQLLKVCTEREFLLMQRNSFVYIFKFF
QLMVIALMTMTIFFRTEMPRDTETDGGIYTGALFFTVVMLMFNGLSELPLTLYKLPVFYKQRDFLFYPSWAYAI
PSWILKIPVTLLEVGMWTVLTYYVIGFDPNVGRFFKQFLLLVLVNQMASGLFRFIAAVGRTMGVASTFGACAL
LLQFALGGFALARTDVKDWWIWGYWTSPLMFSVNAILVNEFDGEKWKHTAPNGTEPLGPSVVRSRGFFPDAY
WYWIGIGALAGFTILFNIAYSLALAYLNPFGKPQATISEEGENNESSGSSPQITSTAEGDSVGENQNKKKGMVLP
FEPQSITFDEVVYSVDMPPEMREQGSSDNRLVLLKGVSGAFRPGVLTALMGVSGAGKTTLMDVLAGRKTGGY
IDGSIKISGYPKKQETFARISGYCEQNDIHSPYVTVYESLVYSAWLRLPQDVDEHKRMMFVEEVMDLVELTPLR
SALVGLPGVNGLSTEQRKRLTIAVELVANPSIIFMDEPTSGLDARAAAIVMRAVRNTVDTGRTVVCTIHQPSIDI
FEAFDESMPGVGKIEEGYNPATWMLEVTSSSQEMSLGVDFTDLYKNSDLCRRNKALITELSVPRPGTSDLHFEN
QFSQPFWVQCMACLWKQRWSYWRNPAYTAVRFLFTTFIALIFGSMFWDLGTKVSRPQDLTNAMGSMYAAVL
FLGVQNASSVQPVVSVERTVFYREKAAGMYSAIPYAFAQVFIEIPYVFVQSVVYGLIVYSMIGFEWTVAKFFW
YFFFMFFTFLYFTFFGMMTVAITPNQNVASIVAGFFYTVWNLFSGFIVPRPRIPIWWRWYYWGCPVAWTLYGL
VASQFGDLQDIVNGQTVEEYLRNDYGIKHDFLGVVAGVIVAFAVVFAFTFALGIKAFNFQKR

>NP_001288053.1_PDR-l_Vv
MATAEIYRAAGSLRRNGSMWRSSGADVFSRSSRDEDDEEALKWAALEKLPTYNRLRKGLLMGSQGAASEVD
VDNLGFQEKQSLMERLVKIAEEDNEKFLLRLRNRIERVGITIPEIEVRFEHLTIDAEAFIGSRALPSFHNFMFNKIE
DALTGLRILRSRRRKFTILHDVSGIIKPQRMTLLLGPPSSGKTTLLLALSGKLDPTLKVTGRVTYNGHGMDEFVP
QRTAAYISQHDTHIGEMTVRETLAFSARCQGVGDRYDMLAELSRREKAANIKPDPDLDVFMKAAATEGQKEN
VVTDYTLKILGLDICADTMVGDEMIRGISGGQRKRVTTGEMLVGPSKALFMDEISTGLDSSTTFQIVNCLKQTI
HILNGTAVISLLQPAPETYNLFDDIILLSDGRIIYQGPREDVLEFFESTGFRCPERKGVADFLQEVTSKKDQQQY
WARKEEPYRFVTVKEFAEAFQSFHTGRKVGDELASPYDKTKSHPAALTTKKYGVNKKELLDANMSREYLLM
KRNSFVYVFKLTQLAIMAVITMTLFLRTEMHKNSVDDGNIYTGALFFTVVMIMFNGMAELAMAIAKLPVFYK
QRDLLFYPAWAYALPTWILKIPITFIEVGVWVFMTYYVIGFDPNVERLFRQYLLLLLVNQMASGLFRLIASAGR
NMIVSNTFGAFVLLMLLALGGFILSHDDVKKWWIWGYWCSPLMYAQNAIVVNEFLGHSWKKNVTGSTESLG
VTVLNNRGFFTEAYWYWIGAGALFGFILLFNFGYTLCLNFLNPFDKPQAVIVEESDNAETGGQIELSQRNSSIDQ
AASTERGEEIGRSISSTSSAVREEAVAGANHNKKKGMVLPFQPYSITFDDIRYSVDMPEEMKSQGVVEDKLELL
KGVSGAFRPGVLTALMGVSGAGKTTLMDVLAGRKTGGYIEGNITISGYPKKQETFARISGYCEQNDIHSPHVT
VYESLLYSAWLRLPSDVKSETRQMFIEEVMELVELTPLRDALVGLPGVSGLSTEQRKRLTIAVELVANPSIIFMD
EPTSGLDARAAAIVMRTVRNTVDTGRTVVCTIHQPSIDIFEAFDELLLLKRGGQEIYVGPLGRYSCHLINYFEGIE
GVSKIKDGYNPATWMLEATTAAQEATLGVDFTEIYKNSDLYRRNKDLIKELSQPPPGTKDLYFRTQFSQPFFTQ
FLACLWKQRWSYWRNPPYTAVRFLFTTFIALMFGTMFWDLGTKWSTQQDLFNAMGSMYAAVLFLGIQNSQS
VQPVVVVERTVFYRERAAGMYSPLSYAFAQALVEIPYIFSQAVVYGLIVYAMIGFQWTAAKFFWYLFFMFFTL
MYFTFYGMMAVAATPNQNIASIVAAAFYGLWNLFSGFIVPRNRIPVWWRWYYWICPVSWTLYGLVTSQFGDI
TEELNTGVTVKDYLNDYFGFKHDFLGVVAAVVVGFVVLFLFIFAYAIKALNFQRR

>NP_001308682.2_P_Zm
MDAAGDIQKVASMRRGDSGSIWRRGDDVFSRSSREEDDEEALRWAALEKLPTYDRVRRAMVPLGLGADGAE
AAGRKGLVDVDVLSLGPRERRALLERLVRVADEDNERFLLKLKDRVDRVGIDMPTIEVRFQNLEAEAEVRVG
SSGLPTVLNSVVNTIEEAANALHILPSRKRIMPILHDVSGIIKPRRMTLLLGPPGSGKTTLLLALAGRLDKDLKVS
GKVTYNGHEMTEFVPERTAAYISQHDLHIGEMTVRETLAFSARCQGVGSRFDMLTELSRREKAANIKPDADID
AFMKASAMGGQDANVVTDYILKILGLEICADTMVGDEMLRGISGGQRKRVTTGEMLVGPARALFMDEISTGL

DSSTTFQIVNSLRQSIHILGGTAVISLLQPAPETYNLFDDIILLSDGQVVYQGPREEVLEFFESVGFRCPERKGVA
DFLQEVTSKKDQKQYWARPNEPYRFVAVKEFATAFKSSHTGRSITNELAVPFDKSKSHPAALTTTRYGVSGKE
LLKANIDREILLMKRNSFVYMFRTFQLMLMSIIAMTLFFRTKMKHGTVNDGGLYMGALFFGVLMIMFNGFSEL
ALTVFKLPVFFKQRDLLFFPAWSYTIPSWILKVPITFIEVGGYVFLTYYVIGFDPNVGRFFKQYLLLLVVNQMAA
SLFRFIGGVSRNMIVANVFASFMLLVVMVLGGFILVRDKVKKWWIWGYWISPMMYAQNAISVNEMLGHSWD
KILNSTASNETLGVQVLKSRGVFTEAKWYWIGFGAMVGFTILFNALFTVALTYLKPYGNSRPSVSEEELKEKH
ANIKGEVLDGNHLVSASSHRSTGVNPETDSAIMEDDSALTKRGMILPFVPLSLTFDNIKYSVDMPQEMKAQGV
QEDRLELLKGVSGSFRPGVLTALMGVSGAGKTTLMDVLAGRKTGGYIEGDIRISGYPKKQDTFARVSGYCEQN
DIHSPQVTVYESLLFSAWLRLPKDVDSNKRKIFIEEVMELVELKPLRNALVGLPGVNGLSTEQRKRLTIAVELV
ANPSIIFMDEPTSGLDARAAAIVMRTVRNTVDTGRTVVCTIHQPSIDIFEAFDELFLMKRGGEEIYAGPLGHHSS
DLIKYFESLHGVSKIKDGYNPATWMLEVTTTSQEQILGVDFSDIYKKSELYQRNKALIKELSQPAPGSTDLHFPS
KYAQSSITQCVACLWKQNLSYWRNPPYNTVRFFFTTIIALLLGTIFWDLGGKTYTSQDLMNAMGSMYSAVLFI
GVMNCTSVQPVVAVERTVFYRERAAGMYSAFPYAFGQVVIELPYALAQDILYGVIVYSMIGFEWTAAKFFWY
LFFGYFTLLYFTFYGMMAVGLTPNYHIAAIVSSAFYAIWNLFSGFIIPRPKVPIWWRWYCWICPVAWTLYGLVV
SQFGDVMTPMDDGRAVKVFVEDYFDFKHSWLGWVAAVVVAFAVLFATLFGFAIMKLNFQKR

>NpPDR1
MEPADLSNLRGRSLRASIRGSMRGSIRENSNSIWRNNGAEVFSRSARDEDDEEALKWAALEKLPTYDRLRKGIL
FGSQGAAAEVDVDDSGVLERKNLLERLVKVADEDNEKFLLKLKNRIDRVGIDFPSIEVRFEHLNIDADAYVGS
RALPTFTNFISNFVEGLLDSIHILPSKKRQVTILKDVSGIVKPCRMTLLLGPPGSGKTTLLLALAGKLDSALKVTG
KVTYNGHELHEFVPQRTAAYISQHDLHIGEMTVRETLEFSARCQGVGSRYEMLAELSRREKAANIKPDADIDM
FMKAASTEGQEAKVVTDYILKILGLDICADTMVGDQMIRGISGGQKKRVTTGEMIVGPSKALFMDEISTGLDSS
TTYSIVNSLKQSVRIMKGTALISLLQPAPETYNLFDDIILLSDGYIVYEGPREEVLEFFESMGFKCPERKGAADFL
QEVTSKKDQQQYWIRRDEPYRFITSKEFAEAYQSFHVGRKVSDELKTTFDKSKSHPAALTTQKYGIGKRQLLK
VCTERELLLMQRNSFVYLFKFFQLLIIALMTMTIFFRTKMPRDSAEDGGIYSGALFFVVIMIMFNGLSELPMTLY
KLPVFYKQRDFLFYPSWAYAIPSWILKIPVTFAEVGMWVFLTYYVMGFDPNVGRFFKQFLLLLLVNQMASALF
RFIAAVGRTMGVASTFGAFALLLQFALGGFILARNDVKDWWIWGYWTSPLMYSVNAILVNEFDGQKWKHIV
AGGTEPLGAAVVRARGFFPDAYWYWIGVGALAGFIVMFNIAYSVALAYLNPFDKPQATISDESENNESESSPQI
TSTQEGDSASENKKKGMVLPFDPHSITFDEVVYSVDMPPEMRESGTSDNRLVLLKSVSGAFRPGVLTALMGVS
GAGKTTLMDVLAGRKTGGYIDGSIKISGYPKKQDTFARISGYCEQNDIHSPYVTVFESLVYSAWLRLPQDVNEE
KRMMFVEEVMDLVELTPLRSALVGLPGVNGLSTEQRKRLTIAVELVANPSIIFMDEPTSGLDARAAAIVMRAV
RNTVDTGRTVVCTIHQPSIDIFEAFDELFLMKRGGQEIYVGPLGRQSCHLIKYFESIPGVSKIVEGYNPATWMLE
VTASSQEMALGVDFTDLYKKSDLYRRNKALIDELSVPRPGTSDLHFDSEFSQPFWTQCMACLWKQHWSYWR
NPAYTAVRLIFTTFIALIFGTMFWDIGTKVSRNQDLVNAMGSMYAAVLFLGVQNSSSVQPVVSVERTVFYREK
AAGMYSAIPYAFAQVLIEIPYIFVQATVYGLIVYSMIGFEWTVAKFFWDFFFMFFTFLYFTFFGMMTVAVTPNQ
NVASIVAGFFYTVWNLFSGFIVPRPRIPIWWRWYYWGCPIAWTLYGLVASQFGDLQDPLTDQNQTVEQFLRSN
FGFKHDFLGVVAAVIVAFAVVFAFTFALGIKAFNFQRR

>NtPDR1
MEPANLSNLRGSSLRGSTRGSLRANSNSIWRNNGVEIFSRSSRDEDDEEALKWAALEKLPTFDRLRKGLLFGSQ
GAAAEVDINDLGFQERKNLLERLVKVADEDNEKFLLKLKNRIDRVGIDLPTIEVRYEHLNIDADAYVGSRSLPT
FMNFMTNFVETLLNSLHILSSRKRQLTILKDISGIIKPCRMTLLLGPPSSGKTTLLLALAGKLDPALKVTGKVSY
NGHELHEFVPQRTAAYISQHDLHIGEMTVRETLEFSARCQGVGSRFEMLAELSRREKAANIKPDADIDIYMKA
AATEGQEANVVTDYVLKILGLDICADTMVGDDMIRGISGGQKKRVTTGEMLVGPSKALFMDEISTGLDSSTTY
SIVNSLRQSVQILKGTAVISLLQPAPETYNLFDDIILLSDGYIVYQGPRDDVLEFFESMGFKCPQRKGVADFLQE
VTSKKDQQQYWSKRNEPYRFITSKEFAEAYQSFHVGRKLGDELATPFDKTKCHPAALTNEKYGIGKKELLKVC
TERELLLMKRNSFVYMFKFSQLTIMALITMTLFFRTEMPRDTTDDGGIYAGALFFVVIMIMFNGMSELAMTIFK
LPVFYKQRDLLFFPSWAYAIPSWILKIPVTLVEVGLWVILTYYVIGFDPNITRFLKQFLLLIVVNQMASGMFRFIG
AVGRTMGVASTFGSFALLLQFALGGFVLSRDDVKSWWIWGYWISPMMYSVNSILVNEFDGKKWNHIVPGGN
ETLGSTVVKSRGFFPEAYWYWIGVGALVGFTVVFNFCYSLALAYLNPFDKPQAVLPEDGENAENGEVSSQIPS
TDGGDSISESQNNKKGMVLPFEPHSITFDDVVYSVDMPQEMKEQGAGEDRLVLLKGVSGAFRPGVLTALMGV
SGAGKTTLMDVLAGRKTGGYIDGEIKISGYPKKQETFARISGYCEQNDIHSPYVTVYESLVYSAWLRLPQDVD
EKTRKMFVDEVMELVELGPLRSALVGLPGVNGLSTEQRKRLTIAVELVANPSIIFMDEPTSGLDARAAAIVMRT
VRNTVDTGRTVVCTIHQPSIDIFEAFDELFLMKRGGQEIYVGPLGRHSCHLIKYFESNPGVAKIKEGYNPATWM

LEVTASAQEMMLGIDFTEVYKNSDLYRRNKALISELGVPRPGSKDLHFETQYSQSFWTQCVACLWKQHWSY
WRNPAYTAVRFIFTTFIALIFGTMFWDLGTKVSKSQDLLNAMGSMYAAVLFLGVQNASSVQPVVAIERTVFYR
ERAAGMYSAIPYAFGQVSIEIPYIFVQSVFYGIIVYAMIGFEWDVGKFFWYLFIMFFTLLYFTFYGMMGVAVTP
NQNVASIVAAFFYGVWNLFSGFIIPRPRMPVWWRWYYWANPVAWTLYGLVASQFGDIQTKLSDNETVEQFLR
RYFGFKHDFLGVVAAVLTAYVFMFAFTFAFAIKAFNFQRR

>OsABCG50
MSSSSSHHPEFASCTANDDEHHLDEFELELVVQDVQRQQNNGSANTDQHERENLLLLDDSSKSGALKRRLFFD
NLLKNVQDDHIRFLHRQKERIDRVDVKLPAIEVRYNNLSVEAECRTANGDHLPSLWNSTKGAFSGLVKLLGLE
TERAKINVLEDVSGIIKPCRLTLLLGPPGCGKSTLLRALSGKLDKSLKVTGDISYNGYQLDEFVPEKTAAYISQY
DLHIPEMTVRETLDFSSRCQGVGRRPKILKEVSARESAAGIIPDADIDIYMKAISVEASKRSLQTDYILKIMGLEIC
ADTMVGDAMIRGLSGGQKKRLTTAEMIVGPARAYFMDEISNGLDSSTTFQIISCFQQLTNISEYTMVISLLQPTP
EVFDLFDDLILMAEGKIIYHGPRNEALNFFEECGFICPERKEVADFLQEILSCKDQQQYWSGPNESYRYISPHELS
SMFKENHRGRKLEEPIVSPKSELGKEALAFNKYSLQKLEMFKACGAREALLMKRSMFVYVFKTGQLAIIALVT
MSVFLRTRMTTDFTHATYYMGALFFSILMIMLNGTPEISMQIRRLPSFYKQKSYYFYSSWAYAIPASVLKVPVSI
LDSLVWICITYYGIGYTASVSRFFCQFLMLCFVHQSVTSLYRFIASYFQTPTASFFYLFLALTFFLMFGGFTLPKP
SMPGWLNWGFWISPMTYAEIGTVINEFQAPRWQKETIQNITIGNRILINHGLYYSWHFYWISIGALFGSIILFYIA
FGLALDYITSIEEYHGSRPIKRLCQEQEKDSNIRKESDGHSNISRAKMTIPVMELPITFHNLNYYIDTPPEMLKQG
YPTKRLQLLNNITGALRPGVLSALMGVSGAGKTTLLDVLAGRKTGGYIEGDIRIGGYPKVQETFVRILGYCEQA
DIHSPQLTVEESVTYSAWLRLPSHVDKKTRSEFVAEVLETVELDQIKDVLVGTPQKNGLSMEQRKRLTIAVELV
SNPSVILMDEPTTGLDTRSAAIVIRAVKNICKTGRTVVCTIHQPSTKIFEAFDELILMKNGGKIIYNGPIGERSSKV
IEYFEKISGVLKVKSNCNPAAWMMDVTSTSMEVQHNMDFAILYDESSQHRDIVELVEKLSIPIPNSEILSFSHRF
PRNGWIQLKACLWKQNLTYWRSPEYNLRRIMLTVISALVYGVLFWKRAKILNDEQDLFNVFGAMYLGSTTIG
SYNHQSIIPFSTTERIVMYREKFAGMYSSWSYSFAQAAIEIPYVFIQVVLYTLIIYPSIGYYWTTHKFIWFFYTTFC
SSLSYIYVGLLLVSLTPNVQVATILASFFNTMQTLFSGFILPAPQIPKWWVWLYYLTPTSWTLDALLTSQYGNIE
KEVRAFGETKSVSIFLNDYFGFHKDKLSLVAAVLIAFPFVLIILFSFSIEKFNFQKR

>PhPDR2
MEPVNLGNLRAASLRGSARGSLSGSLRANSNSIWRNDNVFTRSSRDENDEEALKWAALEKLPTFDRLRKGLLF
GSEGTAPSQIDIHDIGFQERQGLLDRLVKDPDEDNEKFLLKLRDRIDRVGLDLPTIEVRYEHLHVVADAYIGGR
ALPTFTNFVTNFLESLLTSLHILPSKKRKLTILNDVSGIIKPCRLTLLLGPPGSGKTTFLLALAGKLDPELKVTGKV
TYNGHEMTEFVPQRTAAYISQHDLHIGEMTVRETLEFSARCQGIGTRYEMLAELSRREKAANIKPDPDIDIYMK
ASATEGQEANVVTDYVLKILGLDICADTLVGDDMVRGISGGQKKRVTTGEMLVGPSKALFMDEISTGLDSSTT
YSIVNSLRQTVQILKETAVISLLQPAPETYNLFDDIILLTDGLVVYQGPREDVLAFFESMGFKCPDRKGVADFLQ
EVTSKKDQQQYWARRDEPYRFITSKEFAEAYQSFHVGRKQLDELGAHLTEQSHPAALSNQKYGIGKKQLLKV
CTEREYLLMKRNSFLFIFKFFQLLIMAILTMTMFLRTEMHHNTEEDGGTYVGALFFVIVMIMFNGMTELGMVL
FKLPVFYKQRDLFFYPSWAYAIPSWILKIPITFVEVALWVFLTYYVIGFDPNPERLFKQFFLLIIVNQMASGLFRFI
GAAGRTLGIAATFGAFALLLQFALGGFVLSRDMMKKWWIWGYWTSPMMYSVNAILVNEFHGKRWRRIAPN
GTEPLGDAVVRGRGFFPDASWYWIGVGALIGFTVLFNILYSLALAYLNPIGKPQAMMPEDSEDAKTTSTEKEG
YNSEGQNKKRGMVLPFEPHSITFDDVIYSVDMPQEMKDQGASEDRLVLLNGVSGAFRPGVLTALMGVSGAGK
TTLMDVLAGRKTGGYIEGRIHISGYPKKQETFARISGYCEQNDIHSPYVTVYESLVYSAWMRLPHDVDERTRK
MFVEEVMDLVELRPIRSALVGLPGVDGLSTEQRKRLTIAVELVANPSIIFMDEPTSGLDARAAAIVMRAVRNTV
DTGRTVVCTIHQPSIDIFEAFDELFLMKRGGQEIYVGPLGRNSCHLIKYFESMPGVSKIKRWLHPATWMLEVTT
PGQETMFGVDFTDLYKKSDLYGRNKALITELSVPRPGTKDLHFDTQYSQPFWTQCMACLWKQHWSYWRNPA
YTAVRFLFTVMISLVFGTMFWDLGSKVSRAQDLSNAMGCLYAAVLFIGTQNASSVQPVVAVERTVFYRERAA
GMYSALPYAFAQAFIEIPYIFVQATFCGTIIYAMIGFEWTVEKYFWYLFFMFFTLMYYTYYGMMTVAITPNVN
VAQVVSAFFYGLWNLFSGFIVPRPRMAIWWRWYYWICPTAWTLYGLIASQFGDYQNKLTDDETVEQYLRRFF
GFKHEFLPVVGVVTAGFTVLFAFTFAFGIKAFNFQTR

>StPDR2
MEPSDLSNLRGRSIRGSMRGSMRENSNSIWRNNGVEVFSRSNRDEDDEEALKWAALEKLPTYDRLRKGILFGS
QGVAAEVDVDDLGVQQRKNLLERLVKVADEDNEKFLLKLKNRIDRVGIDFPSIEVRFEHLNIEADAYVGSRAL
PTFTNFISNFIESLLDSIHIFPSKKRSVTILKDVSGYVKPCRMTLLLGPPGSGKTTLLLALAGKLDSDLRVTGKVT
YNGHELHEFVPERTAAYISQHDLHIGEMTVRETLEFSARCQGVGSRYEMLAELSRREKAANIKPDVDIDMFMK

ILGLDICADTMVGDQMIRGISGGQKKRVTTGEMIVGPSKALFMDEISTGLDSSTTYSIVNSLKQSVQILKGTALIS
LLQPAPETYNLFDDIILLSDGYIVYQGPREDVLEFFESMGFKCPDRKGVADFLQEVTSKKDQQQYWVRRDEPY
RFITSKEFAEAYQSFHVGRKVSNELSTAFDKSKSHPAALTTEKYGIGKKQLLKVCTEREFLLMQRNSFVYIFKFF
QLMVIALMTMTIFFRTEMPRDTETDGGIYTGALFFTVVMLMFNGLSELPLTLYKLPVFYKQRDFLFYPSWAYAI
PSWILKIPVTLLEVGMWTVLTYYVIGFDPNVGRFFKQFLLLVLVNQMASGLFRFIAAVGRTMGVASTFGACAL
LLQFALGGFALARTDVKDWWIWGYWTSPLMFSVNAILVNEFDGEKWKHTAPNGTEPLGPSVVRSRGFFPDAY
WYWIGIGALAGFTILFNIAYSLALAYLNPFGKPQATISEEGENNESSGSSPQITSTAEGDSVGENQNKKKGMVLP
FEPQSITFDEVVYSVDMPPEMREQGSSDNRLVLLKGVSGAFRPGVLTALMGVSGAGKTTLMDVLAGRKTGGY
IDGSIKISGYPKKQETFARISGYCEQNDIHSPYVTVYESLVYSAWLRLPQDVDEHKRMMFVEEVMDLVELTPLR
SALVGLPGVNGLSTEQRKRLTIAVELVANPSIIFMDEPTSGLDARAAAIVMRAVRNTVDTGRTVVCTIHQPSIDI
FEAFDESMPGVGKIEEGYNPATWMLEVTSSSQEMSLGVDFTDLYKNSDLCRRNKALITELSVPRPGTSDLHFEN
QFSQPFWVQCMACLWKQRWSYWRNPAYTAVRFLFTTFIALIFGSMFWDLGTKVSRPQDLTNAMGSMYAAVL
FLGVQNASSVQPVVSVERTVFYREKAAGMYSAIPYAFAQVFIEIPYVFVQSVVYGLIVYSMIGFEWTVAKFFW
YFFFMFFTFLYFTFFGMMTVAITPNQNVASIVAGFFYTVWNLFSGFIVPRPRIPIWWRWYYWGCPVAWTLYGL
VASQFGDLQDIVNGQTVEEYLRNDYGIKHDFLGVVAGVIVAFAVVFAFTFALGIKAFNFQKR

>VvABCG44
MATAEIYRAAGSLRRNGSMWRSSGADVFSRSSRDEDDEEALKWAALEKLPTYNRLRKGLLMGSQGAASEVD
VDNLGFQEKQSLMERLVKIAEEDNEKFLLRLRNRIERVGITIPEIEVRFEHLTIDAEAFIGSRALPSFHNFMFNKIE
DALTGLRILRSRRRKFTILHDVSGIIKPQRMTLLLGPPSSGKTTLLLALSGKLDPTLKVTGRVTYNGHGMDEFVP
QRTAAYISQHDTHIGEMTVRETLAFSARCQGVGDRYDMLAELSRREKAANIKPDPDLDVFMKAAATEGQKEN
VVTDYTLKILGLDICADTMVGDEMIRGISGGQRKRVTTGEMLVGPSKALFMDEISTGLDSSTTFQIVNCLKQTI
HILNGTAVISLLQPAPETYNLFDDIILLSDGRIIYQGPREDVLEFFESTGFRCPERKGVADFLQEVTSKKDQQQY
WARKEEPYRFVTVKEFAEAFQSFHTGRKVGDELASPYDKTKSHPAALTTKKYGVNKKELLDANMSREYLLM
KRNSFVYVFKLTQLAIMAVITMTLFLRTEMHKNSVDDGNIYTGALFFTVVMIMFNGMAELAMAIAKLPVFYK
QRDLLFYPAWAYALPTWILKIPITFIEVGVWVFMTYYVIGFDPNVERLFRQYLLLLLVNQMASGLFRLIASAGR
NMIVSNTFGAFVLLMLLALGGFILSHDDVKKWWIWGYWCSPLMYAQNAIVVNEFLGHSWKKNVTGSTESLG
VTVLNNRGFFTEAYWYWIGAGALFGFILLFNFGYTLCLNFLNPFDKPQAVIVEESDNAETGGQIELSQRNSSIDQ
AASTERGEEIGRSISSTSSAVREEAVAGANHNKKKGMVLPFQPYSITFDDIRYSVDMPEEMKSQGVVEDKLELL
KGVSGAFRPGVLTALMGVSGAGKTTLMDVLAGRKTGGYIEGNITISGYPKKQETFARISGYCEQNDIHSPHVT
VYESLLYSAWLRLPSDVKSETRQMFIEEVMELVELTPLRDALVGLPGVSGLSTEQRKRLTIAVELVANPSIIFMD
EPTSGLDARAAAIVMRTVRNTVDTGRTVVCTIHQPSIDIFEAFDELLLLKRGGQEIYVGPLGRYSCHLINYFEGIE
GVSKIKDGYNPATWMLEATTAAQEATLGVDFTEIYKNSDLYRRNKDLIKELSQPPPGTKDLYFRTQFSQPFFTQ
FLACLWKQRWSYWRNPPYTAVRFLFTTFIALMFGTMFWDLGTKWSTQQDLFNAMGSMYAAVLFLGIQNSQS
VQPVVVVERTVFYRERAAGMYSPLSYAFAQALVEIPYIFSQAVVYGLIVYAMIGFQWTAAKFFWYLFFMFFTL
MYFTFYGMMAVAATPNQNIASIVAAAFYGLWNLFSGFIVPRNRIPVWWRWYYWICPVSWTLYGLVTSQFGDI
TEELNTGVTVKDYLNDYFGFKHDFLGVVAAVVVGFVVLFLFIFAYAIKALNFQRR

>XP_002297807.2_PDR1_Pt
MESADIYRASSSLRGSFRGGSSAWRNTTVEAFSRSSREEDDEEALTWAAIEKLPTYDRLRKGILTSASKGVANE
VDIEKLGVQERKQLLERLVKVAEEDNEKFLWKLKDRVERVGIDVPTIEVRYDNLNIEAEAYVGSSALPSFAKFT
FNIIEGLLISLNILRNRKKPLTILKDVSGIVKPSRLTLLLGPPSSGKTTLLLALAGKLDPNLKFSGRVTYNGHEMN
EFVPQRTAAYISQHDLHIGEMTVRETLAFSARCQGAGYLHDMLAELSRREKEANIKPDPDVDVFMKAVASQG
EEANVITDYVLKILGLEVCADTMVGDEMIRGISGGQRKRVTTGEMLVGPSRALFMDEISTGLDSSTTYQIVNSL
RHTVHILNCTAVISLLQPAPETYDLFDDIILLSDGQIVYQGPRERVLEFFKHMGFECPERKGVADFLQEVTSRKD
QEQYWARKDQPCRFITANEFAEAFQSFSVGRRTAEELSIPFDKSKNHPAALVTKTHGAGKKDLLKANFSREYL
LMKRNSFVYIFKICQLTIMALISMTLFFRTEMHRDTVADGGIYTGALFFTAIMIMFNGMSELSMTIAKLPVFYKQ
RDLRFFPSWAYAIPQWILKIPVAFVEVGVWVFLTYYVIGFDPNVGRLFKQYLLLLLINQMASALFRFIAAAGRN
MIVANTFGSFALLTLFALGGFILSREKIKKWWIWGYWISPLMYGQTAIVVNEFLGNSWSHVPENSTEPLGIQVL
KSRGFFTEAYWYWIGAGATIGFILLLNLFFVLALTFLNAFDKPQAVISDEPESDESGRKTERAIQLSNHGSSHGT
NTEGGVGISRASSEAIGRVSNNRKKGMVLPFEPLSITFDDVIYSVDMPQEMKIQGVVEDRLVLLNGVNGAFRPG
VLTALMGVSGAGKTTLMDVLAGRKTGGYIDGEIKISGYPKKQETFARVSGYCEQNDIHSPQVTVYESLLYSAW
LRLPPEVDSETRRMFIEEVMDLVELNPLRHALVGLPGVNGLSTEQRKRLTIAVELVANPSIIFMDEPTSGLDARA
AAIVMRTVRNTVDTGRTVVCTIHQPSIDIFEAFDELFLMKRGGQEIYVGPLGRHSTHLIKYFEAIEGVSKIKDGY

NPATWMLEISSSAQEMALEVDFSNIYKNSDLFRRNKALIVELSTPAPGSTDLYFPTKYSTSFLTQCMACLWKQH
WSYWRNPPYTAVRFLFTTFIALMFGTMFWDLGSKVDSTQDLFNAMGSMYAAVIFLGVQNASSVQPVVAVER
TVFYRERAAGMYSALPYAFAQVLIELPYIFVQAAVYGIIVYAMIGFEWTVVKFFWYLFFMYFTLLYFTFYGMM
AVAMTPNHHIAAIVSSAFYGIWNLFSGFIIPRPSMPIWWRWYSWACPIAWTLYGLVVSQFGDIQKDLTETQTVK
EYVKDYFGFDHDFLGVVAAAIVGWTVLFAFIFAFAIKAFNFQRR

>XP_002298123.2_PDR1_X1_Pt
MESGYLYRAGSSVRRGNSSGTFSNNAAADHQVFSLSSHGQDDDEEALKWAALEKLPTYDRLRKGILTTSTGA
ASEVEVQNLGFQERKNLVERLVNVAEEDNEKFLLKLKNRIDRVGIHVPTIEVRFEHLNVEAEAYVGSRALPTFF
NYSVNMLEGVLNYLHILSSRKKHMWILKDVSGIIKPSRMTLLLGPPSSGKTTLLLALAGKLDHALKFSGRVTY
NGHEMDEFVPQRTAAYISQHDLHIGEMTVRETLAFSARCQGVGSRYDMLAELSRREKEAGIKPDPDIDVFMKA
AATEGQEDSVVIDYILKVLGLEVCADTLVGDEMLRGISGGQKKRVTTGEMLVGPAKALFMDEISTGLDSSTTY
QIVNSIKQYVQILEGTALISLLQPAPETYDLFDDIILLSDGEIVYQGPREHVLRFFEYMGFKCPARKGVADFLQEV
TSRKDQMQYWARRDVPYRFVTVKEFAEAFYSFHEGKRLGNELAVPFDKSKNHPAALTTKKYGVNKRELCKA
SFSREFLLMKRNSFVYAFKFIQLTIVAVIAMTLFLRTEMHRDSVTDGGIYVGAMFFIVVVIMFNGMAEISMTLA
KLPVFYKQRDLLFFPAWIYALPTWILKIPITFIEVAIMVFITYFVIGFDPNVGRLFKHYLVLLLTNQMASGLFRTI
AAVGRNMVVANTFGSFVLLLLFVLGGFVLSRDDIKKWWIWGFWTSPMMYAQNAVVVNEFLGKSWNHVLPN
STEPLGIEVLKSRGFFTEAYWYWLAVAALFGFTLLYNFLYILALAFLNPLGKPQQAGISEEPQSNNVGRIGEAIH
LMNPGINSSLHTSAESIDEIGRSKSSRFTCNKQRGVIIPFEPHSITFDKVMYSVDMPQEMKSHGVHEDKLVLLKG
VSGAFRPGVLTALMGISGAGKTTMMDVLAGRKTGGYIEGNITISGYPKKQETFARISGYCEQNDIHSPHITVYE
SLLYSAWLRLPTEVDIETRKMFVEEVMELVELNPLRQALVGLPGVDGLSTEQRKRLTIAVELVANPSIIFMDEP
TSGLDARAAAIVMRTVRNTVDTGRTVVCTIHQPSIDIFEAFDELFLLKRGGQEIYVGPLGRLSCHLIKYFEGIEG
VNKIKDGYNPATWMLEVTSTAEELALGVDFAEIYRSSELFRRNRALIKDLSTPAPGSKDLYFSTQYSRSFFTQCL
ACLWKQHWSYWRNPPYTAIRFLSTTVIGLIFGTMFWDIGSKITKRQDLFNAMGSMYTAVLFLGVQNAASVQP
VVAVERTVFYRERAAGMYSALPYAFAQVLIELPYIFVQAAVYGVIVYSMIGFGWTISKFFWYLYFMYFTLLYF
TFYGMMAVAVSPNHQIASVISAAFYGIWNVFSGFVIPRSRMPLWWRWYSWICPVFWTLYGLVASQFGDMKD
RLETGETVEQFVTIYLDFKHDFLGVVAAVILGFTVLFAITFAISIKLFNFQRR

>XP_002451753.1_ABCG39_Sb
MDLVRMGSIASGSMRRTASSWRGSGRSDAFGRSVREEDDEEALRWAAIEKLPTYDRMRKGILTGAGAGGGIE
EVDIQGLGMQERQNLIERLVRTAEEDNERFLLKLRDRMERVGIDNPTIEVRFENLNIDAEAYVGNRGVPTMTN
FFSNKVMDALSAMHIVSSGKRPISILHDISGIIRPGRMSLLLGPPGSGKTSLLLALAGKLDSALKVSGRVTYNGH
DMDEFVPQRTSAYIGQHDVHVGEMTVRETLAFSARCQGVGTRYDMLTELSRREKEANIKPDPDIDVYMKAIS
VEGQESVVTDYILKILGLEICADTMVGDSMIRGISGGQKKRVTTGEMLVGPAKALFMDEISTGLDSSTTYQIVN
SLRQSVHILGGTALIALLQPAPETYELFDDIVLLSEGQIVYQGPRENVLEFFEAMGFKCPERKGVADFLQEVTSR
KDQHQYWCRRDERYRYISVNDFSEAFKAFHVGRKLGSELMEPFDRTRNHPAALTTSKYGISKMELLRACFSRE
WLLMKRNSFVYIFKVVQLIILGTIAMTVFLRTTMHRRSVEDGVIFLGAMFLGLVTHLFNGFAELAMSIAKLPIFY
KQRDLLFYPSWAYALPTWLLKIPISFLECAVWIGMTYYVIGFDPNIERFFRHYLLLVLISQMASGLFRLLAALGR
EMVVADTFGSFAQLVLLILGGFLIARDNIKKYWIWGYWSSPLMYAQNAIAVNEFLGHSWQKVVDSTQSNDTL
GVEILKARGIFVDPNWYWIGVGALLGYIMLFNVLFVLFLDWLGPLGQGQAVVSEEELREKHVNRTGENVELLP
LGTASQNSPSDGRGEIAGAETRKRGMVLPFMPLSITFDNVKYSVDMPQEMKDKGITEDRLLLLKGVSGAFRPG
VLTALMGVSGAGKTTLMDVLAGRKTGGYIEGDISISGYPKKQETFARIAGYCEQNDIHSPHVTVYESLLYSAW
LRLPHEVDSEARKMFVEEVMELVELTPLRGALVGLPGVNGLSTEQRKRLTIAVELVANPSIIFMDEPTSGLDAR
AAAIVMRTVRNTVDTGRTVACTIHQPSIDIFEAFDELFLMKRGGEEIYVGPLGRNSCHLIDYFEGIEGVKKIKDG
YNPATWMLEVTTLAQEDILGINFAEVYRNSDLYRRNKALISELSTPPPGSKDLYFPTQYSQSFLTQCMACLWK
QHMSYWRNPSYTATRIFFTTVIALIFGTIFLNLGKKIGTRQDLLYALGSMYAAVLFIGIQNGQTVQPIVDVERTV
FYREKAAGMYSALPYAFAQVLIEIPHIFLQTVVYGLIVYSLIGFEWTAEKFFWYMFFMFFTFMYFTFYGMMAV
AMTPNSDIAAIVSTAFYAIWNIFAGFLIPRPRIPIWWRWYSWACPVAWTLYGLVASQFGDITDVRLEDDEIVKD
FVNRFFGFQHDNLGYVATAVVGFTVLFAFVFAFSIKVFNFQRR

>XP_002451754.1_ABCG39_Sb
MDLVRMGSIASGSMRRTASSWRASGRSDAFGRSVREEDDEEALRWAAIEKLPTYDRMRKGILTGAGAGFEEV
DIQGLGMEERKNLIERLVRTAEEDNERFLLKLRDRMERVGIDNPTIEVRFEHLNIDAEAYVGNRGIPTMTNFFS
NKIMDALSAMHIVASGKRPISILHDISGVIRPGRMSLLLGPPGSGKTSLLLALSGKLDSTLKVSGRVTYNGHDM

DEFVPQRTSAYIGQHDIHVGEMTVRETLSFSARCQGVGTRYDMLTELSRREKEANIQPDPDIDVYMKAISVEGQ
ESVVTDYILKILGLEVCADTMVGDSMIRGISGGQKKRVTTGEMLVGPAKALFMDEISTGLDSSTTYQIVNSLRQ
SVHILGGTALIALLQPAPETYELFDDIVLLSEGQIVYQGPRENVLEFFEAMGFKCPERKGVADFLQEVTSRKDQ
HQYWCRRDERYRYISVNDFSEAFKAFHVGRKLGTELKEPFDRTRNHPAALTTSKYGISKMELLKACFSREWLL
MKRNSFVYIFKVVQLIILGTIAMTVFLRTTMHRRGVEDGVIFLGAMFLGLVTHLFNGFAELAMSIAKLPIFYKQ
RDLLFYPSWAYALPTWLLKIPISFLECAVWIGMTYYVIGFDPNIERFFRHYLLLVLISQMASGLFRLLAAVGRE
MVVADTFGSFAQLVLLILGGFLIARDNIKKYWIWGYWSSPLMYAQNAIAVNEFLGHSWQKVVDSTHSNDTLG
VQILKARGIFVDPNWYWIGVGALLGYIMLFNVLFILFLDWLGPLGQGQAVVSEEELREKHVNRTGENVELLAL
GTSSQNSPSDGRGEIAGAETRNRGMALPFTPLSITFDNVKYSVDMPQEMKDKGITEDRLLLLKGVSGAFRPGVL
TALMGVSGAGKTTLMDVLAGRKTGGYIEGDISISGYPKKQETFARIAGYCEQNDIHSPHVTVYESLLYSAWLR
LPHEVDSEARKMFVEQVMELVELTPLRGALVGLPGVNGLSTEQRKRLTIAVELVANPSIIFMDEPTSGLDARAA
AIVMRAVRNTVDTGRTVVCTIHQPSIDIFEAFDELFLMKRGGEEIYVGPLGRNSCHLIDYFEGIEGVKKIKDGYN
PATWMLEVTTLSQEDILGINFAEVYRNSDLYRRNKALISELSIPPPGSRDLYFPTQYSQSFLTQCMACLWKQHK
SYWRNPSYTATRIFFTTVIALIFGTIFLNLGKKIGTRQDLLYALGSMYAAVLFIGIQNGQTVQPIVDVERTVFYRE
KAAGMYSALPYAFAQVLIEIPHIFLQTVVYGLIVYSLIGFEWTAEKFLWYMFFMFFTFMYFTFYGMMAVAMTP
NSDIAAIVSTAFYAIWNIFAGFLIPRPRIPIWWRWYSWACPVAWTLYGLVASQFGDITDVRLEDDEIVKDFVNR
FFGFYHDDLAYVATAVVGFTVLFAFVFAFSIKVFNFQRR

>XP_002458135.1_ABCG35_Sb
MDAAAELQKVASMRRDSGGSRSGSSAWWRAPDAFSRSSSRMEEDDEEALRWAALERLPTCDRVRRAILPLG
GNGDGHGHGGGDAATQVVDVLGLGPRERRALLERLVRVADEDNERFLLKLKERVERVGIDMPTIEVRFKHLR
AEADVRVGTSGLPTVLNSITNKLEEVANALHVRRSRKQAMPILHDVSGIVKPRRMTLLLGPPGSGKTTLLLALA
GRLDKDLKVSGKVTYNGHEMDEFVPERTAAYISQHDLHIGEMTVRETLEFSARCQGVGTRFDMLTELSRREK
VGNIKPDADIDAFMKACAMRGQEANVISDYILKILGLEICADTMVGDDMLRGISGGQRKRVTTGEMLVGPAN
ALFMDEISTGLDSSTTFQIIKSLRQAIHILGGTALISLLQPAPETYDLFDDIILLSDGQIVYQGPRESVLEFFLSLGF
KCPERKGVADFLQEVTSRKDQKQYWVWHDKPYRYVSVKEFATAFQCFHVGRAIANELAIPFDKSKNHPAALT
TSKYGVSAWELFKANIDREMLLMKRNSFVYIFRTLQLMTVSIIAMTLFFRTKMHRDSVTDGGIYLGALFFAVIM
IMFNGLSELALTIIKLPVFFKQRDLLFFPAWAYTIPTWILKIPISFVEVGGFVFMAYYVIGIDPNVGRFFKQYLLLL
ALNQMAASLFRFVGGAARNMIVANVFGSFMLLIFMVLGGFILVRDKVKKWWIWGYWISPLMYAQNAISVNE
MLGHSWDKILNSSVSYETLGVQSLKSRGVFPEAKWYWIGLGALLGFVMLFNCLFTLALAYLKPYGKSHPSISE
EELNEKYANLNGNVVAEDNLPPGSSYLAAVDITRSDSATIENHSGTMQRGMVLPFAPLSLTFSNIKYFVDMPQE
MKTHDVVGDRLELLKCVSGSFRPGVLTALMGVSGAGKTTLMDVLAGRKTSGYIEGNISISGYPKKQETFARVS
GYCEQNDIHSPQVTVYESLVFSAWLRLPSDVDLNTRKMFIEEVMELVELKPLRNALVGLPGVNGLSTEQRKRL
TIAVELVANPSIIFMDEPTSGLDARAAAIVMRTVRNTVDTGRTVVCTIHQPSIDIFEAFDELFLMKRGGEEIYVGP
LGHHSSELIKYFEGIHGVKKIKDGYNPATWMLEVTTISQEEILGVDFSDLYKKSELYQRNKALIQELSEPSVGST
DLHFRNQYSQSFFMQCLACLWKQNLSYWRNPAYNAVRLFFTTIIALIFGTIFWDLGGKMGQSQDLFNAMGSM
YAAVMFIGVLNATSVQPVVSVERTVFYRERAAGMYSALPYAFGQVTIELPYTLTQATVYGIIVYSMIGFEWTV
AKFFWYLFFMYFTFLYFTFYGMMAVGLTPSYHVASIVSSAFYGIWNLFSGFIIPRPKVPIWWKWYCWACPVA
WTLYGLVVSQFGDITMPMDNGVPVNVFVENYFGFKHSWLGVVAAVVMAFTIFFASLFGFAIMKLNFQRR

>XP_002458139.2_ABCG36_Sb
MDVTGELQKVASMRGGSGSMWRRGDDVFSRSSREEDDEEALRWAALEKLPTYDRVRRAIVPLDLGADGAEA
AGGKGLVDVDVLSLGPRERRALLERLVRVADEDNERFLLKLKDRVDRVGIDMPTIEVRFQNLEAEAEVRVGSS
GLPTVLNSIVNTVEEAANALHILPSSKRIMPILHDVSGIIKPRRLTLLLGPPGSGKTTLLLALAGRLDKDLKFSGK
VTYNGHEMTEFVPERTAAYISQHDLHIGEMTVRETLAFSARCQGVGSRFDMLTELSRREKAANIKPDADIDAF
MKASAMGGQDANVVTDYILKILGLEICADTMVGDEMLRGISGGQRKRVTTGEMLVGPSRALFMDEISTGLDS
STTFQIVNSLRQSIHILGGTAVISLLQPAPETYNLFDDIILLSDGQVVYQGPREEVLEFFESVGFRCPERKGVADFL
QEVTSKKDQKQYWARLDAPYRFVSVKEFATAFKSFHTGRAIANELAVPFDKSKGHPAALTTTRYGVSGKELL
KANIDREILLMKRNSFVYIFRTFQLVLMSIIVMTLFFRTKMKHDSVTDGGIYLGAVFFGVLMIMFNGFSELALTV
FKLPVFFKQRDLLFFPAWSYTIPSWILKIPISFIEVGGYVFLTYYVIGFDPNVGRFFKQYLLLLAVNQMAAALFRF
IGGASRNMIVANVFASFMLLVVMVMGGFILVRDKIKKWWIWGYWISPMMYAQNAISVNEMLGHSWDKILNS
AASNETLGLQSLKSRGVFTEPKWYWIGFGALVGFTLLFNALFTLALTYLKPYGNSRPSVSEEELQEKHANIKGG
NHLVSASSHQSTGLNTETDSAIMEDDSASTKKGMILPFDPLSLTFDNIKYSVDMPQEMKAQGVQEDRLELLKG
VSGSFRPGVLTALMGVSGAGKTTLMDVLAGRKTGGYIEGDICISGYPKKQETFARVSGYCEQNDIHSPQVTVY

ESLLFSAWLRLPKDVDSNTRKIFIEEVMELVELKPLRNALVGLPGVNGLSTEQRKRLTIAVELVANPSIIFMDEP
TSGLDARAAAIVMRTVRNTVDTGRTVVCTIHQPSIDIFEAFDELFLMKRGGEEIYAGPLGHHSSDLIKYFEGIQG
VSKIKDGYNPATWMLEVTTTSQEQILGVDFSDIYKKSELYQRNKALIKELSHPVPGSSDLHFASTYAQSSITQCV
ACLWKQNLSYWRNPPYNTVRFFFTTIIALLLGTIFWDLGGKVSTSQDLMNALGSMYAAVIFIGVMNCTSVQPV
VAVERTVFYRERAAGMYSAFPYAFGQVVIELPYALVQDILYGVIVYAMIGFEWTAAKFFWYLFFGYFTLLYFT
FYGMMAVGLTPNYHIASIVSSAFYAIWNLFSGFIIPRPKTPIWWRWYCWICPVAWTLYGLVVSQFGDIMTEMD
DNNRTVVVSQYVEDYFGFKHSWLGWVAAVVVAFAVLFAALFGFAIMKFNFQKR

>XP_003526427.1_PDR1_Gm
MESGELRVASARIGSSGVWRSGSIDVFSGSSRRDDDEQELKWAAIEKLPTYLRMTRGILTETEGQPTEIDINKLC
PLQRKNLVERLVKIAEQDNEKFLFKLRDRIDRVGLEIPTIEIRFEHLNVEAEAHVGSRALPTIFNFCINLFEGFLNS
LHLIPSRKKPFTVLDDVSGIIKPKRMTLLLGPPSSGKTTLLLALAGRLSKDLKFSGRVSYNGHGMEEFVPQRTSA
YISQTDLHIGEMTVRETLAFSARCQGIGTRYEMLAELSRREKAANIKPDPDLDIYMKAAALEGQETNVVTDYI
MKILGLEVCADTMVGDDMIRGISGGQKKRVTTGEMLVGPARALFMDEISTGLDSSTTFQMVNSLRQSIHILNG
TAVISLLQPAPETYELFDDIILLSDGQIVYQGPRENVLEFFEYMGFKCPERKGVADFLQEVTSRKDQEQYWANK
DEPYSFVTVKEFAEAFQSFHAGRKLGDELATPFDMSKGHPAVLTKNKFGVCKKELLKACVSREFLLMKRNSF
VYIFKMWQLILTGFITMTLFLRTEMHRDTETDGGIYMGALFFVLIVIMFNGYSELSMSIMKLPVFYKQRDLLFFP
CWAYSLPTWILKIPITLVEVGIWVVMTYYVIGFDPSIERFIKQYFLLVCINQMASGLFRFMGAVGRNIIVANTVG
SFALLAVMVMGGFILSRVDVKKWWLWGYWFSPMMYGQNALAVNEFLGKSWSHVTPNSTEPLGVKVLKSRG
IFPKAYWYWIGVGASIGYMLLFNFLFPLALHYLDPFGKPQALISEEALAERNAGRNEHIIELSSRIKGSSDKGNES
RRNVSSRTLSARVGGIGASEHNKKRGMVLPFTPLSITFDEIRYSVEMPQEMKSQGILEDRLELLKGVNGAFRPG
VLTALMGVSGAGKTTLMDVLSGRKTAGYIQGQITISGYPKRQETFARIAGYCEQTDIHSPHVTVYESLVYSAW
LRLPPEVDSSTRQMFIEEVMELVELTSLREALVGLPGVNGLSTEQRKRLTIAVELVANPSIIFMDEPTSGLDARA
AAIVMRTVRNTVDTGRTVVCTIHQPSIDIFDAFDELLLLKRGGEEIYVGPLGQHCSHLINHFEGINGVPKIKNGY
NPATWMLEVTSEAQEAALGVNFAEIYKNSDLYRRNKALIRELTTPPTGSKDLYFPTKYSQTFFTQCMACLWKQ
HLSYWRNPPYSAVRLLFTTIIALLFGTIFWDIGSKRQRKQDLFNAMGSMYAAVLFIGIQNATSVQPVVAIERTVF
YRERAAGMYSALPYAFGQVAIEIPYIFIQTLVYGVIVYAMIGFDWTFSKFFWYLFFMFFTFLYFTFYGMMAVGL
TPDHNVAAIVSFGFYMIWNLFSGFVIPRTRMPVWWRWYFWICPVSWTLYGLVTSQFGDIKEPIDTGETVEEFV
RSYFGYRDDFVGVAAAVLVGFTLLFGFTFAFSIKAFNFQKR

>XP_003528365.1_PDR1_X1_Gm
MEGSDIYRARNSLRANSSTVWRNSIMEAFSRSSRHEEDNDEEALKWAALEKLPTYNRLRKGLLTTSRGVANEI
DITELGFQERQKLLDRLINVAEEDNETLLLKLKERIDRVGIDIPTIEVRYEHLNVEAEAYVGSRALPTFLNFVTN
MVESFFTSLHILSGKKKHVTILRDVSGIIKPRRMALLLGPPSSGKTTLLLALSGKLDPTLKVSGRVNYNGHEMN
EFVPQRTAAYISQHDVHIGEMTVRETLAFSARCQGVGTRYDLLSELARREKEAKIKPDPDIDVYMKAAATGGQ
EASLVTDYVLKILGLDICADTMMGDEMLRGISGGQRKRVTTGEMLVGPANALFMDEISTGLDSSTTFQIVKSL
RQYVHILNGTAVISLLQPAPETYELFDDIVLISDGQIVYQGPREYVLEFFEYVGFQCPERKGVADFLQEVTSRKD
QEQYWIHRDESYRFVTVTEFAEAFQSFHVGRRIGEELATPFDKSKSHPAALTTKKYGVNKKELLKANFSREYL
LMKRNSFVYIFKLFQLTILAILTMTMFLRTEMHRNSLNDGGVYTGALFFAVVILMFNGLAEISMTIVKLPIFYKQ
RDLLFYPSWAYAIPSWILKIPITFIEAAVWVFLTYYVIGFDPNVGRLLKQYLVLLLINQMSSGLFRAIAALGRNM
IVASTFGSFALLVLFALGGFVLSRNDIKNWWIWGYWISPLMYGQNAIVVNEFLGDSWNHFTPNSNKTLGIQILE
SRGFFTHAYWYWIGIGALIGFMILFNIIYTLALTYLNPYDTPQTTITEESESGMTNGIAESAGRAIAVMSSSHKKK
RGMILPFEPYSITFDQIVYSVDMPLEMKDQGVREDRLVLLKGVSGAFRPGVLTALMGVSGAGKTTLMDVLAG
RKTGGYIEGNIKVSGYPKRQETFARISGYCEQNDIHSPHVTVYESLVYSAWLRLPAEVEAYTRKMFIEEVMELV
ELNPLRNSLVGLPGVNGLSTEQRKRLTIAVELVANPSIIFMDEPTSGLDARAAAIVMRTVRNTVDTGRTVVCTI
HQPSIDIFEAFDELFLMKRGGQEIYVGPLGRHSSQMIKYFESIEGVGKIKDGYNPATWMLEVTTPAQELNLGVD
FHEIYRNSGLCRRNKRLISELGNPAPGSKDLHFPTQYPQSLLVQCLACLWKQHWSYWRNPPYTAVRFLSTTVT
AVLFGTMFWDLGGKYSSRQDLFNAMGSMYNAVLFVGVQNSASVQPVVAIERTVFYRERAAGMYSALPYALA
QVIIELPYVFVQATSYSVIVYAMMGFEWTLQKFFWYVFFMYFTLCYFTFYGMMTVAVTPNHHVASVVASAFY
GIWNLFSGFVIARPSIPVWWRWYYWACPVAWTIYGLVASQFGDITNVMKSENMSVQEFIRSHLGIKHDFVGVS
AIMVSGFAVLFVIIFAVSIKAFNFQRR

>XP_003543663.1_PDR1_Gm
MEGSDIYRASNSLRRSSTAWRNSGVEVFSRSSREEDDEEALKWAALEKLPTYNRLRKGLLTASHGVANEIDVS

DLGTQERHKLLERLVKVAEEDNERFLLKLKERIDRVGLDIPTIEVRYEHLNIEAEAFVGSRALPSFINSVTNIIEG
FFNLLHITTSKKKHVTILKDVSGIIKPRRMTLLLGPPSSGKTTLLLALSGKLDKTLKVSGRVTYNGHELNEFVPQ
RTAAYISQHDLHIGEMTVRETLAFSARCQGVGSRYDMLSELSRREKAANIKPDPDLDVYMKATATEGQESSIV
TDYTLKILGLDICADTMVGDEMLRGISGGQRKRVTTGEMLVGPANALFMDEISTGLDSSTTFQIVNSLRQYVHI
LNGTAVISLLQPAPETYDLFDDIILISDGQVVYHGPREYVLDFFESMGFRCPERKGVADFLQEVTSKKDQAQYW
ARRDQPYRFVKVTQFAEAFQSFHIGRKLGEELVVPFDKTKSHPAALTTKKYGINKKELLKANLSREYLLMKRN
SFVYIFKLCQLSIMALMTMTLFLRTELHRNNMDDAGLYSGALFFTLIMIMFNGMAEISMTIAKLPVFYKQRDLL
FYPSWAYAIPSWILKIPVTLLEVAVWVFLTYYVIGFDPNVGRFFKQYLILLFIGQMASALFRAIAALGRNMIVSN
TFGAFAVLTFLTLGGYVMSKNDIKNWWIWGYWISPLMYGQNALMVNEFLSNSWHNTSRNLGVEYLESRGFP
SSSYWYWLGLGAMAGFVLLFNVMFSAALEILGPFDKPQATITEEESPNEGTVAEVELPRIESSGRGDSVVESSH
GKKKGMVLPFEPHSITFDEVIYSVDMPQEMKEQGVQEDRLVLLKGVSGAFRPGVLTALMGVSGAGKTTLMD
VLAGRKTGGYIDGSIKISGYPKKQETFARISGYCEQNDIHSPHVTVYESLLYSAWLRLPSGVDSKTRKMFIEEV
MELVELNPLRNSLVGLPGVSGLSTEQRKRLTIAVELVANPSIIFMDEPTSGLDARAAAIVMRTVRNTVDTGRTV
VCTIHQPSIDIFEAFDELFLMKRGGQEIYVGPLGRHSTHLIKYFESIGGVSKIKDGYNPATWMLEVTTSAQELSL
GVDFTDLYKNSDLYRRNKQLIQELGQPAPGSKDLYFPTQYSQSFLVQCQACLWKQRWSYWRNPPYTAVRFFF
TTFIALMFGTMFWDLGSRRTTRGDLLNALGSMYSAVLFLGIQNASSVQPVVAVERTVFYREKAAGMYSALPY
AFAQVLVEIPYIFAQAVTYGLIVYAMIGFDWTAEKFFWYLFFSFFSLLYFTFYGMMAVGVTPNHHVAAIVAAA
FYAIWNLFSGFIVVRPKMPVWWRWYYWACPVAWTLYGLIASQFGDITERMPGEDNKMVKEFIEDYFGFKHD
FVGICAVVVAGIAVAFALIFGAAIKTFNFQKR

>XP_003546218.1_PDR1_Gm
MEGSDIYRASNSLRRSSTVWRNSGVEVFSRSSREEDDEEALKWAALEKLPTYNRLRKGLLTASHGVANEIDVS
DLGIQERQKLLERLVKVAEEDNERFLLKLKERIDRVGLDIPTIEVRYEHLNIEAEAFVGSRALPSFINSVTNVVEG
FFNLLHISTSKKKHVTILKDVSGIIKPRRMTLLLGPPSSGKTTLLLALSGKLDKTLKVSGRVTYNGHELNEFVPQ
RTAAYISQHDLHIGEMTVRETLAFSARCQGVGSRYDMLSELSRREKAANIKPDPDLDVYMKATATEGQESSLV
TDYTLKILGLDICADTMVGDEMLRGISGGQRKRVTTGEMLVGPANALFMDEISTGLDSSTTFQIVSFLRQYVHI
LNGTAVISLLQPAPETYDLFDDIILISDGQVVYHGPREYVLDFFESMGFRCPERKGVADFLQEVTSKKDQAQYW
ARRDQPYRFVTVTQFSEAFQSFHIGGKLGEELAVPFDKTKSHPAALTTKKYGINKKELLKANLSREYLLMKRN
SFVYIFKLCQLSIMALMTMTLFLRTELHRNNMDDAGLYAGALFFTLVMIMFNGMAEISMTIAKLPVFYKQRDL
LFYPSWAYAIPSWILKIPVTLLEVAVWVFLTYYVIGFDPNVGRLFKQYLILLFIGQMASALFRAIAALGRNMIVS
NTFGAFAVLTFLTLGGFVMAKSDIKNWWIWGYWISPLMYGQTALMVNEFLSNSWHNSSRNLGVEYLESRGFP
SSAYWYWLGLGAMAGFVLLFNVMFSAALEILGPFDKPQATIAEEESPNEVTVAEVELPRIESSGRGGSVVESSH
GKKKGMVLPFEPHSITFDEVVYSVDMPQEMKEQGVQEDRLVLLKGVSGAFRPGVLTALMGVSGAGKTTLMD
VLAGRKTGGYIDGNIKISGYPKKQETFARISGYCEQNDIHSPHVTVYESLLYSAWLRLPSSVDSQTRKMFIEEV
MELVELNPLRNSLVGLPGVSGLSTEQRKRLTIAVELVANPSIIFMDEPTSGLDARAAAIVMRTVRNTVDTGRTV
VCTIHQPSIDIFEAFDELFLMKRGGQEIYVGPLGRHSSHLIKYFESIEGVSKIKDGYNPATWMLEVTTSAQELSLG
VDFTDLYKNSDLYRRNKQLIQELGQPAPGSKDLYFPTQYSQSFLVQCQACLWKQRWSYWRNPPYTAVRFFFT
TFIALMFGTMFWDLGSRRTTRGDLLNALGSMYTAVLFLGIQNASSVQPVVAVERTVFYREKAAGMYSALPYA
FAQVLVEIPYIFAQAVTYGLIVYAMIGFDWTAEKFFWYLFFSFFSLLYFTFYGMMAVGVTPNHHVAAIVAAAF
YAIWNLFSGFIVVRPKMPVWWRWYYWACPVAWTLYGLIASQFGDITERMPGEDNKMVKDFVEDYFGFKHDF
VGVCAVVVAGIAVAFALIFGVAIKTFNFQKR

>XP_003546230.1_PDR1_Gm
MEGSDIYRASNSLRSRSSTVWRNSGVEAFSRSSREEDDEEALKWAALEKLPTYNRLRKGLLTASHGVANEIDV
SDLGIQERQKLLERLVKVAEEDNERFLLKLKERIDRVGLDIPTIEVRYEHLNIEAEAFVGSRALPSFINSVTNVVE
GFFNLLHVSTSKKKHVTILKDVSGIIKPRRMTLLLGPPSSGKTTLLLALSGKLDKTLKVSGRVTYNGHELNEFVP
QRTAAYISQHDLHIGEMTVRETLAFSARCQGVGSRYDMLSELSRREKAANIKPDPDLDVYMKATATEGQESNI
VTDYTLKILGLDICADTMVGDEMLRGISGGQRKRVTTGEMLVGPANALFMDEISTGLDSSTTFQIVSSLRHYVH
ILNGTAVISLLQPAPETYDLFDDIILISDGQVVYHGPREYVLDFFESMGFRCPERKGVADFLQEVTSKKDQAQY
WVRRDQPYRFVTVTQFAEAFQSFHIGGKLGEELTVPFDRTKSHPAALTTKKYGINKKELLKANFSREYLLMKR
NSFVYLFKLSQLFIMALVAMTLFLRTEMHHENMDDAGVYAGAVFFMLITVMFNGLAEISMTIAKLPVFYKQR
NLLFYPSWAYAIPSWILKIPVTIVEVAVWVFLTYYVIGFDPNVGRFFKQYLVLLIVSQMASGLFRTIAALGRNMI
VANTFGAFAIITVVALGGFILSKRDIKSWWIWGYWISPLMYGQNALMVNEFLSNSWHNATHNLGVEYLESRAF
FTDSYWYWLGLGALVGFVFLFNVMFGLALEFLGPFDKPQATITEDESSNEGTLADIELPGIESSGRGDSLVESSH

GKKKGMVLPFEPHSITFDEVVYSVDMPQEMKEQGVQEDRLVLLKGVSGAFRPGVLTALMGVSGAGKTTLMD
VLAGRKTGGYIDGSIKISGYPKKQETFARISGYCEQNDIHSPHVTVYESLLYSAWLRLPSSVDSKTRKMFIEEVM
ELVELNPVRNSLVGLPGVSGLSTEQRKRLTIAVELVANPSIIFMDEPTSGLDARAAAIVMRTVRNTVDTGRTVV
CTIHQPSIDIFEAFDELFLMKRGGQEIYVGPLGRHSSHLIKYFESIEGVSKIKDGYNPATWMLEVTATAQELSLG
VDFTDLYKNSDLYRRNKQLIQELGQPAPGSKDLHFPTQYSQSFLVQCQACLWKQRWSYWRNPPYTAVRFFFT
TFIALMFGTIFWDLGGKHSTRGDLLNAIGSMYTAVLFLGVQNASSVQPVVAIERTVFYREKAAGMYSALPYAF
AQILVELPYVFVQAVTYGVIVYAMIGFEWTAEKFFWYLFFMYFTLLYYTFYGMMTVGLTPNHHIASIVAAAFY
AVWNLFSGFVVTRPSIPVWWRWYYWACPVAWTIYGLVASQFGDLTEPMTSEGQKIVKDFLEDYYGIKHDFIG
VSAVVVAGIAVLFALIFAVSIKTFNFQKR

>XP_003588699.2_PDR1_Mt
MESNEVSRVDSLRRASSSNIWRNNSMNVFSTSEREDDEEALKWAAIERLPTYLRIRRSIINNEEGEGREIDIKKL
GLTERKVLLERLVKIAEEDNEKFLLKLKERIERVGLDIPIVEVRFEHINVEAQVYVGGRALPSLLNFYANVLEGF
LNYLHIIPSPKKPLHILQNVSGIIKPQRMTLLLGPPGSGKTTLLLALAGKLAKDLKQSGRVTYNGKGLDEFVPQR
TSAYISQHDNHIGEMTVRETLAFSARCQGVGHNYDMLTELLRREKEAKIKPDPDVDAYMKAAALEGQEASVV
TDYILKILGLEICADIMVGDGMIRGISGGQKKRVTTGEMLVGPIRVLFMDEISTGLDSSTTFQIISSIRQSIHILNGT
ALVSLLQPAPETYELFDDIILLTDGQIVYQGPRENVLEFFESMGFKCPERKGVADFLQEVTSRKDQWQYWANK
DEPYSFVTVKDFAEAFQIFHIGQKLGDELANPFDKSKCHASVLTTKKYGVNKKELLKACASREFLLMKRNSFV
HIFKVTQLIYLAIMTTTLFLRTKMHKDTVEDGGAYMGALFFTVTVAMFNGISELNMTLMKLPVFYKQRDLLFY
PSWAYSLPPWILKIPIALIEAVIWEAITYYAIGYDPSFVRLLKQYLVILLINQMATSLFRLMAALGRDVIVASTVG
SFALLVVLVLGGFVISREDVHKWFLWGYWSSPLMYGQNAIAVNEFLGHSWRKVTHNSNETLGVLVMKTRGF
FPQAYWYWIGVGALIGYVFLFNFLFTLALQYLNPFRKDQAGLSEEELLERDASTAVEFTQLPTRKRISETKIAEE
GLMPSRSFSARVSKDKTSISGRRGMVLPFQPLSLTFDEIRYAVDMPQEMKNQGVSEDRLELLKGINGAFRPGVL
TALMGVSGAGKTTLMDVLAGRKTGGYIDGNITISGYPKNQKTFARISGYCEQFDIHSPNVTVYESLLYSAWLR
LPPEVDQATRKMFIEEVMELVELNSLREALVGLPGETGLSTEQRKRLTIAVELVANPSIIFMDEPTSGLDARAAA
IVMRTVRNTVDTGRTVVCTIHQPSIDIFDAFDELLLMKLGGEQIYSGPLGRHCAHLIHYFEAIEGVPKIKDGYNP
ATWMLEVTSAGSEANLKVNFTNVYRNSELYRRNKQLIQELSIPPQDSKELYFDSQYTQTMLSQCKACLWKQH
LSYWRNTSYTAVRLLFTTLIAFLFGIIFWNIGLKRRKEQDLFNAMGSMYASVIFIGVQNGASVQPVIAVERTVFY
RERAAGMYSALPYAAAQVIIELPHILVQTLVYGIIVYAMMGFEWTASKFFWYIFFNYFTFLYYTFYGMMTMAI
TPNPHVAAILSSSFYAIWNLFSGFIIPLSKIPIWWKWFYWVCPVAWTLYGLVTSQYGDNMQKLENGQRVEEFV
KSYFGFEHDFLGVVAIVVVSFSVFFALIFTFGIKAFNFQKR

>XP_003625399.2_PDR1_X1_Mt
MEGGGSFRIGSSSIWRNSDAAEIFSNSFHQEDDEEALKWAAIQKLPTFERLRKGLLTSLQGEATEIDVENLGLQE
RKDLLERLVRLAEEDNEKFLLKLKDRIDRVGIDLPTIEVRFEGLNIEAEAHVGNRSLPTFTNFMVNIVEGLLNSL
HVLPSRKQHLNILKDVSGILKPSRMTLLLGPPSSGKTTLLLALAGKLDPKLKFSGKVTYNGHEMNEFVPQRTAA
YVDQNDLHIGEMTVRETLAFSARVQGVGPRYDLLAELSRREKHANIMPDPDIDVYMKAIATEGQKANLITDY
VLRILGLEICADTVVGNAMLRGISGGQKKRVTTGEMLVGPAKALFMDEISTGLDSSTTFQIVNSMKQFVHILKG
TAVISLLQPPPETYNLFDDIILLSDSHIIYQGPREHVLEFFESIGFKCPDRKGVADFLQEVTSRKDQEQYWEHKDQ
PYRFVTAEEFSEAFQSFHVGRRLGDELGTEFDKSKSHPAALTTKKYGVGKWELFKACLSREYLLMKRNSFVYI
FKICQICIMAMIAMTIFFRTEMHRDSVTLGGIYVGALFYGVVVIMFNGMAEISMVVSRLPVFYKQRGYLFFPPW
AYALPAWILKIPLTFVEVAVWVFLTYYVIGFDPYIGRFFRQYLILVLVNQMASALFRFIAAVGRDMTVALTFGS
FALSILFAMSGFVLSKDRIKKWWIWGFWISPMMYGQNAMVNNEFLGNKWKHVLPNSTDPIGVEVLKSRGYFT
ESYWYWIGVGALIGYTLLFNFGYILALTFLNPLGKHQTVIPDESQSDGQIGGGRKRTNVLKFIKDSFSQHSNKV
RNGEIRSGSTSPSTSSDRQERVAAETNHSRKRGMVLPFEPHSITFDEVTYSVDMPQEMRNRGVVEDKLVLLKG
VSGAFRPGVLTALMGVTGAGKTTLMDVLSGRKTGGYIGGNITISGYPKKQDTFARISGYCEQTDIHSPHVTVYE
SLLYSAWLRLSPDINAETRKMFIEEVMELVELKPLQNAIVGLPGVSGLSTEQRKRLTIAVELVANPSIIFMDEPTS
GLDARAAAIVMRTVRNTVDTGRTVVCTIHQPSIDIFESFDELLLLKQGGKEIYVGSLGHNSSNLISYFEGIHGVN
KIKEGYNPATWMLEITNSSKEVDLGIDFAEVYKNSDLYRRNKTLIEELSTPASGSKDLYFTSQYSRSFWTQCMA
CLWKQHWSYWRNPVYTAIRFLYSTSVAVLLGTMFWNLGSNIEKEQDLFNAMGSMYSAVLLIGIKNSNAVQPV
VAVERTVFYRERAAGMYSAFPYAFAQVVIELPHVFVQSVVYGFIVYAMIGFEWSVVKVLWYLFFMYFTFLYF
TFYGMMAVAMTPNNHISTIVSSAFYSVWNLFSGFIVPRPRIPVWWRWYSWANPVAWSLYGLVASQYGDLKQ
NIETSDRSQTVKDFLRNYFGFKHDFLGMVALVNVAFPIAFALVFAIAIKMFNFQRR

>XP_003625401.2_PDR1_Mt
MEGGGSFRISSSSIWRSSDAAEIFSNSFHQEDDEEALKWAAIQNLPTFARLRKGLLTSLQGEAVEIDIEKLGLQER
KDLLERLVRLAEEDNEKFLLKLKDRMDRVGVDLPTIEVRFEHLNIEAEARVGSRSLPTFTNFMVNIVEGLLNSL
HVLPSRKQHLNILRDVSGILKPSRMTLLLGPPSSGKTTLLLALAGKLDPKLKFSGRVTYNGHEMSEFVPQRTAA
YVDQNDLHIGEMTVRETLAFSARVQGVGPRYDLLAELSRREKDANIKPDPDIDVYMKAVATEGQKANLITDYI
LRVLGLEICADTIVGNAMLRGISGGQKKRLTTGEMLVGPTKALFMDEISTGLDSSTTFQIVNSMRQDVHILNGT
AIISLLQPPPETYNLFDDVILLSDSRIIYQGPREHVLEFFESIGFKCPDRKGVADFLQEVTSRKDQEQYWDHKDQP
YRFVTAEEFSEAFQSFHVGRRLGDELGTEFDKSKSHPAALTTKKYGVGKWELYKACSSREYLLMKRNAFVYIF
KLCQLAVMAMIAMTLFLRTEMHRDSVTHGGIYVGALFYGVVVIMFNGMAELSMVVSRLPVFYKQRGYLFFP
AWAYALPGWILKIPLIFAEVAVWVFLTYYVIGFDPYIERFFRQYLILVLVHQMATALFRFIAAVGRDMTVALTF
GSFAIAILFAMSGFVLSKDSIKNGWIWGFWISPMMYGQNAMVNNEFLGNKWKHVLPNSTEPLGVEVLKSRGF
FTESYWYWIGVGALIGYTLLFNFGYMLALTFLNPLGKHQTVIPDDSQSSEKIGGSRERSNVLRFIKDGFSQITNK
VRNGESRSGSISPIRQEIVASETNHSRKRGMVLPFEPHSITFDEVTYSVDMPQEMRRNLGVVEDKLVLLKGVSG
AFRPGVLTALMGVTGAGKTTLMDVLSGRKTGGYIGGNITISGFPKKQETFARISGYCEQNDIHSPYVTVYESLL
YSAWLRLSPDINAETRKMFVEEVMELVELKPLQNALVGLPGVNGLSTEQRKRLTIAVELVANPSIIFMDEPTSG
LDARAAAIVMRTVRNTVNTGRTVVCTIHQPSIDIFESFDELLLLKQGGQEIYVGPLGHNSSNLINYFEGIHGVSKI
KDGYNPATWMLEVTTSSKERELGIDFAEVYQNSELYRRNKALIKELSTPAPCSKDLYFASQYSRSFWTQCMAC
LWKQHWSYWRNPEYNAIRFLYSTAVAVLFGSMFWDLGSKIEKEQDLFNAMGSMYSAVIVIGIKNANSVQPVV
AVERTVFYRERAAGMYSAFPYAFAQVVIELPYVFVQAVVYGIIVYAMIGFEWSVVKFLWCLFFLFCTFLYFTY
YGLMSVAMTPNNHISIIVSSAFYSIWNLFSGFIVPRPNIPVWWRWYSWANPIAWSLYGLVVSQYGDEKHNIETS
DGRQTVEGFLKNYFDFKHDFLGVVALVNVAFPIGFALVFAISIKMFNFQRR

>XP_004239864.1_PDR1_-l_Sl
MEPVNLNSMRGSSMRGSMRGSLRASTSNSIWRNNGVDAFSRSTRDEDDEEALKWAALEKLPTFDRLRKGLLF
GSQGAANEIDVNDLGYQERKNLLERLVKVADEDNEKFLMKLKNRIDRVGIDMPSIEVRYEHLNIEADAYAGSR
ALPTFINFMTNFVETLLNSLHILPSKKRQITILKDVSGMIKPCRMTLLLGPPSSGKTTLLLALAGKLDPALRVTGN
VTYNGHELHEFVPQRTAVYISQHDLHIGEMTVRETLEFSARCQGVGSRFEMLAELSRREKAANIKPDPDIDIYM
KAAATEGQEANVVTDYVLKILGLDICADTMVGDEMIRGISGGQKKRVTTGEMLVGPSKALFMDEISTGLDSST
TFSIVNSLRQSVQLLKGTAVISLLQPAPETYNLFDDIILLSDGYIVYQGPREAVLDFFESMGFKCPERKGAADFL
QEVTSKKDQQQYWAKRNEPYRFITSKEFSEAYQSFHVGRKLSDELATPYDKTKSHPAALSTKKYGIGTKQLLK
VCAEREFLLMKRNSFVYIFKLTQLAIMALITMSVFFRTKLPRDDMDDGGIYAGALFFVVVMIMFNGMAEIALTI
FKLPVYFKQRDLLFFPSWAYALPTWILKIPITFVECGMWTFLTYYVMGFDPNVSRLFKQFLLLVLVHQMASAL
FRFIGAVGRTMGVASTFGAFALLLQFALGGFVLAREDVKKWWIWGYWTSPLMYSVNSILVNEFDGKNWKHI
APNGTEPLGAAVVRSRGFFPDAYWYWIGCGALFGFTMIFNFFYSIALAYLDPFGKPQAMISEDGEDAVELTERS
ETEGQDKKKGMVLPFEPHSITFDNIVYSVDMPQEMKEQGSAEDRLVLLKGVSGAFRPGVLTALMGVSGAGKT
TLMDVLAGRKTGGYIDGDIKISGYPKKQETFARISGYCEQNDIHSPYVTVYESLVYSAWLRLPQDVDENKRKM
FVDEVMELVELAPLRSALVGLPGVNGLSTEQRKRLTIAVELVANPSIIFMDEPTSGLDARAAAIVMRAVRNTVD
TGRTVVCTIHQPSIDIFEAFDELFLMKRGGQEIYVGPLGRHSCHLIKYFESMPGVGKIKEAYNPATWMLEVTAS
SQEMMLGVDFADLYKNSDLYRRNKALIAELSTPRPATKDLHFETQFSQPFWTQCMACLWKQHWSYWRNPAY
TAVRFIFTTFIALVFGTMFWDLGTKVSRSQDLINAMGSMYAATLFLGVQNSSSVQPVVAVERTVFYREKAAG
MYSAIPYAFGQVVIEIPYVFVQSAFYGVIVYAMIGFEWTAVKFLWYFFFMYCTLLYFTFYGMMTVAVTPNQN
VASIVAAFFYAVWNLFSGFIVPRPRIPIWWRWYYWACPVAWTLYGLVASQFGDIQTPLTDDENVEQYLRRYF
GFKHDFLGVVAAVIVALPVMFALTFALGIKAFNFQRR

>XP_004239865.1_PDR1_-l_Sl
MMEGANLNNFRGSLRASMRGNSSNSIFSRSGRDEDDEEALKWAALEKLPTFDRMRKGLLFGKEGETISEVDTN
DIGHQERKNLLDRLVKVADEDNEKFLLKLKDRIETVGIDLPSIEVRYEHLNIAADAYVGSRALPTFINFMTNSVE
TFLNTIHILPSRKRQITILNDVSGMIKPSRLTLLLGPPSSGKTTLLLALAGKLDPTLKVKGNVTYNGHELHEFVPQ
KTAVYISQHDLHIGEMTVRETLEFSARCQGVGPRYEMLAELSRREKAANIKPDRDIDIYMKASVAKGQEANIV
TDYVLKILGLDICADTMVGDEMLRGISGGQKKRVTTGEMLVGPSKALFMDEISTGLDSSTTFSIVNSLRQSVQL
LKGTAVISLLQPAPETYNLFDDIILLSDAQIVYQGPREDVLDFFESMGFKCPERKGVADFLQEVTSKKDQQQYW
AKKDEPYRFITSKEFAEAYQSFHVGKKLADELKTPYDKTKSHPAALSTKKYGIGMKQLLKVCADREFLLMKR
NSFVFIFKFFQLMVMAFIMMSIFFRTEMPRNNMDDGGMYAGALFFVVVVIMFNGMAEINLTILKLPVYFKQRD
LLFYPSWAYALPTWILKIPITIVEVAIWTFLTYYVMGFDPNVSRLFKQFLLLVLVHQMASGLFRFIGAAGRTMG

VATTFGAFALVLQFALSGFVLSRNDVKKWWIWGYWISPLMYSVNSILVNEFDGKKWDRIAPNGAESLGHAVL
RSRGFFPDPYWYWIGVGALIGYIIIFNLGYSIGLAYLNPFGKPQAILSEDNETEQLIEGSETEGQDKKRGMVLPFE
PHSITFDNIVYSVDMPQEIKDQGSTEDRLVLLKGVSGAFRPGVLTALMGVSGAGKTTLMDVLAGRKTGGYIDG
DIKISGYPKKQATFARISGYCEQNDIHSPYITVYESLVYSAWLRLPQDVDKNKRKMFVEEVMELVELTPLRSAL
VGLPGVNGLSTEQRKRLTIAVELVANPSIIFMDEPTSGLDARAAAIVMRAVRNTVDTGRTVVCTIHQPSIDIFEA
FDELLLMKRGGQEIYVGPLGRYSCHLIKYFESLPGVSKIKEAYNPATWMLEVTAASQEMMLGVDFTDLYKKS
DLYKRNKALIAELSTPRPGTTDLHFETQFSQSFWTQCMACLWKQHLSYWRNPSYTAVRFIFTVILALVFGTLF
WDLGSRLSRSQDLFNAMGSMYAATLFLGVQNSSSAQPVVAVERTVFYRERAAGMYSALPYAFGQVIVEIPYV
FLQAVFYGIIVYAMIGFEWTVAKFFWYLFIMYFTLLYFTFYGMLTVAVSPNQNVASIIAAFFYALWNLFSGFIVP
RPRIPIWWRWYYWLCPVAWTLYGLVASQFGDLQTMLSDDENVEQFLGRYFGFEHDFLGVVAAVIVVWPAVF
AFLFAYAIKAFNFQKR

>XP_004245224.1_PDR1_-l_Sl
MEGGENLVRVSSARLSGSNVWRNSAMDVFSRSSSREDYDDEEALRWAALEKLPTYSRIRRGLLLEEEGQSREV
DITKLDLIERRNLLDRLVKIADEDNEKLLMKLKQRIDRVGLDLPTIEVRFEHLNVDAEARVGSRALPTIFNFTVN
ILEDFLNYIHILPSRKKPLPILHGVSGIIKPGRMTLLLGPPSSGKTTLLLGLAGKLDKDLKVSGRVTYNGHGMDE
FVPQRTSAYISQNDLHIGEMTVRETLAFSARCQGVGDKYEILAELSRREKEANIKPDPDVDIFMKSAWNEGQEA
NVITDYTLKILGLEICADTLVGDEMIRGISGGQRKRLTTGEMMVGPARALFMDEISTGLDSSTTYQIVNSIRQSIH
ILQGTAVISLLQPAPETYDLFDDIILLSDGKIVYQGPRENVLEFFEYMGFKCPERKGVADFLQEVTSRKDQEQY
WSRRDEPYRFITSCEFSDVFQSFHVGRKLGEELAVPFDKSKSHPAALTTKRYGISKKELLKACAAREYLLMKRN
SFVYIFKMVQLTMMASIAMTLFLRTEMHRDTTIDGAVYLGALFYAVITVMFNGFSELALSIMKLPSFYKQRDL
LFFPAWAYALPTWILKIPITLVEIAIWVCMTYYVIGFEADVGRFFKQLFLLICLNQMASGLFRFLAALGRNIIVA
NTFGSCALLVVLVMGGFILSRDDVKQWLIWGYWTSPMMYAQNAIAVNEFLGKSWSHVPPNSTGTDTLGVSFL
KSRGIFPEARWYWIGAGALIGYVLLFNFLFTVALAYLNPFGKPQAIISEEIVVERIASKRGEVIELSPIGKSSSERG
NDVAISASSRSLSSRVGNITEGDLSKRRGMILPFEPLSITFDDIRYAVDMPQEMKAQGFIEDRLELLKGVSGAFRP
GVLTALMGVSGAGKTTLMDVLAGRKTGGYINGTISISGYPKQQETFARISGYCEQTDIHSPHVTVYESLQYSA
WLRLPREVDTETRKNFIEEVMELVELIPLREALVGLPGVNGLSTEQRKRLTVAVELVANPSIIFMDEPTSGLDAR
AAAIVMRTVRNTVDTGRTVVCTIHQPSIDIFDAFDELLLLKRGGEEIFVGPLGRHSSHLIKYFEGIDGVLKIRDGY
NPATWMLEVTSLAQEAVLGIDFTELYKNSELYRRNKALIQELSVAAPGSKDLYFETEYSQSFFTQCMACLWKQ
HLSYWRNPPYTAVRLMFTFFVSLMLGTIFWGLGSKRGRQQDILNAIGSMYSAILFLGIINATSVQPVVAIERTVF
YRERAAGMYSALPYAFGQVMIELPHLFLQTIIYGVIVYAMIGFEWTVAKFFWYLFFMYFTLLYFTLYGMMTV
AVTPNHTIASIVSSAFYTIWNLFCGFVVPKTRMPVWWRWYYYVCPLSWTLYGLIASQFGDVQDKLDTKETVE
QFLENFFDYKHDFVGYVAVILVGISVAFLFIFAYSIKAFNFQKR

>XP_004245225.1_PDR1_-l_Sl
MEGGGDILKVSSARLGSSTVWRNSGVDVFSRSSREDYDDEEALKWAALEKLPTYLRIRRGILSEEEGQYREVDI
TKLDLVERRNLLERLVKIADEDNEKFLLKLKKRIDRVGLDLPTIEVRFEHLNVDAEARVGSRALPTIFNFTVNIIE
DFLNYLHILPSRKKPLPILHEISGIIKPGRMTLLLGPPSSGKTTLLLGLAGKLDKDLKVSGRVTYNGHGMDEFVP
QRTSAYISQNDLHIGEMTVRETLAFSARCQGVGAKYEILAELSRREKEANIKPDPDVDIFMKSAWNDGQEANV
VTDYTLKILGLEICADTIVGDEMIRGISGGQRKRLTTGEMMVGPARALFMDEISTGLDSSTTYQIVNSIRQSIHIL
QGTAVISLLQPAPETYDLFDDIILLSDGQIVYQGPRENVLEFFEYIGFKCPQRKGVADFLQEVTSRKDQEQYWA
RRDEPYKFITVREFSEAFQSFHVGRKLGDELAVPFDKSKSHPAALTTERYGVSKKELLKACTAREYLLMKRNSF
VYIFKMIQLTLMATITMTLFLRTEMHRDTMIDGAVFLGALYYAVIMIMFNGFSELALSIMKLPSFYKHRDLLFF
PAWTYALPTWILKIPITLVEVAIWVCMTYYVIGFEADVGRFFKQLFLLICLNQMASGLFRFLAALGRNVIVANT
FGSCALLIVLVMGGFILSRDNVKQWLIWGYWISPMMYAQNAIAVNEFLGKSWAHVPPNSTGTDTLGVSFLKS
RGIFPEARWYWIGVGALLGYVLLFNFLFTVALAYLNPFGKPQAVLSEETVAERNASKRGEVIELSPIGKSSSERG
NDVRRSASSRSMSSRVGNIAEGDLNKRKGMILPFEPLSITFDDIRYAVDMPQEMKAQGFTEDRLELLKGVSGAF
RPGVLTALMGVSGAGKTTLMDVLAGRKTGGYIEGTISISGYPKQQATFARIAGYCEQTDIHSPHVTVYESLQYS
AWLRLPREVDTETRKRFIEEVMELVELKPLREALVGLPGVNGLSTEQRKRLTVAVELVANPSIIFMDEPTSGLD
ARAAAIVMRTVRNTVDTGRTVVCTIHQPSIDIFDAFDELLLLKRGGEEIFVGPLGRHSSHLIKYFEGIDGVLKIKD
GYNPATWMLDITSVAQEAALGIDFTELYRNSELYRRNKALIQELSVPAPGSKDLYFETKYSQSFFTQSMACFW
KQHWSYWRNPPYTAVRLMFTFFIALMFGTIFWDLGSKRRRQQDILNAIGSMYAAVLFLGVQNATSVQPVVAIE
RTVFYRERAAGMYSALPYAFGQIMIELPYIFIQTIIYGVIVYAMIGFEWTVAKFIWYLFFMYFTLLYFTLYGMMT
VAVTPNHSIAAIISSAFYAVWNLFSGFIVPKTRMPVWWRWYFYICPISWTLYGLVASQFGDLQDKLETKETVEE

FIESFFDFKYDFVGYVALILVGISVGFLFIFAYSIKAFNFQKR

>XP_004247841.1_PDR1_Sl
MEPANLGNLRGSSLRGSISGSRRGSVSLRANSNSIWRNTGVEIFSRSARDEDDEEALKWAALEKLPTFDRLRKG
LLFGSQGAAAEIDIDDIGLQERKNLLERLVRVADEDNEKFLLKLKNRIDRVGIDLPTIEVRYENLNIEADAYVGS
RGLPTFINFMTNFLETLLNTLHILPSSKRQITILKDISGIIKPCRMTLLLGPPSSGKTTLLLALAGKLDSSLKVTGK
VSYNGHELHEFVPQRTAAYISQHDLHIGEMTVRETLEFSARCQGVGSRYEMLAELSRREKAANIKPDPDIDIYM
KASATEGQEANVVTDYVLKILGLDICADTMVGDEMLRGISGGQKKRVTTGEMLVGPSKALFMDEISTGLDSST
TYSIVNSLRQSVQILKGTAVISLLQPAPETYNLFDDIILISDGYIVYQGPRDDVLQFFESMGFKCPERKGVADFLQ
EVTSKKDQPQYWSRRNEHYRFISSKEFSDAYQSFHVGRKLGDELAIPFDRTKCHPAALTNEKYGIGKKELLKV
CTEREYLLMKRNSFVYVFKFFQLTIMALMTMTLFFRTEMPRDTVDDGGIYAGALFFVVVMIMFNGMSEMAM
TIFKLPVFYKQRDLLFFPSWAYAIPSWILKIPVTLVEVGLWVILTYYVIGFDPNITRFLKQFMLLVLVNQMASGL
FRFMGAVGRTMGVASTFGAFALLLQFALCGFVLSREDVKGWWIWGYWISPLMYSVNSILVNEFDGSKWKHIA
PNGTEPLGVAVVKSRGFFPDAYWYWIGFAALFGFTVVFNFFYSLSLAYLKPYGKSQTVRPEDSGNAENGQAAS
QMTSTDGGDIVSAGQSKKKGMVLPFEPHSITFDDVVYSVDMPQEMKEQGAGEDRLVLLKGVSGAFRPGVLTA
LMGVSGAGKTTLMDVLAGRKTGGYIDGDIKISGYPKKQETFARISGYCEQNDIHSPYVTVYESLVYSAWLRLP
KDVDEKTRKMFVDEVMELVELEPLRSALVGLPGVNGLSTEQRKRLTIAVELVANPSIIFMDEPTSGLDARAAAI
VMRTVRNTVDTGRTVVCTIHQPSIDIFEAFDELFLMKRGGQEIYVGPLGRHSCHLIKYFESIPGVAKIKEGYNPA
TWMLEVTASAQEMMLGVDFTDLYKNSDLYRRNKALITELSVPRPGSKDLYFETQYSQSIWIQCMACLWKQN
WSYWRNPAYTAVRFIFTMFIALVFGTMFWDIGTKVSQSQDLFNAMGSMYAAVLFLGVQNASSVQPVVDVER
TVFYRERAAGMYSAIPYAFGQVFIEIPYVFVQAIVYGIIVYAMIGFEWEAGKFFWYLFIMFTTLLYFTFYGMMS
VAVTPNQNVASIVAAFFYAIWNLFSGFIVPRPRMPIWWRWYYWCCPVAWTLYGLVASQFGDIQSRLTDEETV
EQFLRRYFGFRHDFLPVVAGVLVAYVVVFAFTFAFAIKAFNFQRR

>XP_004247842.1_PDR1_Sl
MEPLDLSNLRGRSIRGSMRENSNSIWRNNGVEVFSRSNRDEDDEEALKWAALEKLPTYDRLRKGILFGSQGVT
AQVDVDDLGVSQRKSLLERLVKVADEDNEKFLLKLKNRIDRVGIDFPSIEVRFEHLNIEADAYVGSRALPTFTN
FISNFIESLLDSIHITPSKKRSVTILKDVSGYVKPCRMTLLLGPPGSGKTTLLLALAGKLDSDLRVTGKVTYNGHE
LHEFVPQRTAAYISQHDLHIGEMTVRETLEFSARCQGVGSRYEMLAELSRREKAANIKPDVDIDMFMKAISTEG
QESKVITDYILKILGLDICADTMVGDQMIRGISGGQKKRVTTGEMIVGPSKALFMDEISTGLDSSTTYSIVNSLK
QSVQILKGTALISLLQPAPETYNLFDDIILLSDGYIVYQGPREDVLEFFESMGFKCPDRKGVADFLQEVTSKKDQ
QQYWVRRDEPYRFITSKEFAEAYQSFHVGRKVSNELSTAFDKSKSHPAALTTEKYGIGKKQLLKVCTEREFLL
MQRNSFVYIFKFFQLMVIALMTMTIFFRTEMPRDTATDGGIYAGALFFTVVMLMFNGLSELPLALYKLPVFYK
QRDFLFYPSWAYAIPSWILKIPVTFLEVGMWTFLTYYVIGFDPNVGRFFKQFLLLVLVNQMASGLFRFIAAVGR
TMGVASTFGACALLLQFALGGFALARTDVKDWWIWGYWTSPLMYSVNAILVNEFDGEKWKHTAPNGTEPLG
PSVVRSRGFFPDAYWYWIGIGALAGFTILFNIAYSLALVYLNPFGKPQATISEEGENNESSGSSSQITSTTEGDSV
DENQNKKKGMVLPFEPHSITFDEVVYSVDMPPEMREQGSSDRLVLLKGVSGAFRPGVLTALMGVSGAGKTTL
MDVLAGRKTGGYIDGSIKISGYPKKQETFARISGYCEQNDIHSPYVTVHESLVYSAWLRLPQDVDEHKRMMFV
EEVMDLVELTPLRSALVGLPGVNGLSTEQRKRLTIAVELVANPSIIFMDEPTSGLDARAAAIVMRAVRNTVDTG
RTVVCTIHQPSIDIFEAFDELFLMKRGGQEIYVGPLGRESCHLIKYFESMPGVGKIEEGYNPATWMLEVTSSSQE
MSLGVDFTELYKNSDLCRRNKALITELSVPRPGTSDLHFENQFSQPFWVQCMACLWKQHWSYWRNPAYTAV
RFLFTTFIALMFGSMFWDLGTKVSRPQDLTNAMGSMYAAVLFLGVQNASSVQPVVSVERTVFYREKAAGMY
SAIPYAFAQVFIEIPYVFVQAVVYGLIVYSMIGFEWTVAKFFWYFFFMFFTFLYFTFFGMMTVAITPNQNVASIV
AGFFYTVWNLFSGFIVPRPRIPIWWRWYYWACPVAWTLYGLIASQFADLQDIVNGQTVEEYLRNDYGIKHDFL
GVVAGVIVAFAVVFAFTFALGIKAFNFQRR

>XP_004252846.1_PDR1_-l_Sl
MEPTNSRGTSLRERIIRGNSLKGNSTNNSRWTSNDGEIFNRSTRDEDDEEALKWAALEKLPTFDRLRKGLLFGS
QGASAEIDIHDIGFQERNKLLERLVKVADEDNEKLLLKLRQRIDRVGIDFPEIEVRYENLTIEADAYIGSRALPTF
TNFITNFLEDMLNSLHILPSRKRNLTILNDVSGIIKPCRLTLLLGPPGCGKTTFLLALAGKLDSALKVTGKVTYN
GHVMNEFVPQRTAAYISQYDLHIGEMTVRETLEFSARCQGVGSRYEMLIELSRREKAAKIKPDPDIDIFMKALA
TEGQEAIFVTDYVLKLLGLDICADTLVGDEMIRGISGGQKKRVTTGEMLVGPSKALFMDEISTGLDSSTTYSIV
NSLRQSVQILHGTAVISLLQPAPETYNLFDDIILLSDGKIVYQGPREDVLGFFESMGFKCPDRKGVADFLQEVTS
KKDQQQYWVRDETYQFIKSNEFAEAYQSFHVGRKLADELAASYDKSKSHPAALSTQKYGIGRKQLLKVCTER

EILLMKRNLFVYIFKFIQNMIIAVITTTLFFRTKMPHDTIEDGGKYAGALFFIVTQIMFSGMIEIGLVIYKLPIFYKQ
RDLLFFPSWAYAMPSWILKIPIAFVEVGLWVLLTYYVIGFDPSPVRLFKHFLLLILVNQMTSGMCRFLGAAGRT
MGVANTYGTFALLLLFGLGGFVLSRDDVKKWWIWGYWSSPLMYSLNSIFVNEFDGKRWKHIAPTGTDSLGV
AIVRSRGFFPNAYWYWIGVGALIGFTIVFNICYSIALAFLNPLGKPQGMISEDSDDAKTTNTGKEVPTSEGQNKK
KGMVLPFEPHSITFNEVTYSVDMPQEMKNQGATEDRLVLLNGVCGAFRPGVLTALMGVSGAGKTTLMDVLA
GRKTGGYIEGSIKISGYPKKQETFARISGYCEQNDIHSPYVTVYESLVYSAWLRLPSDVDEKTRKMFVDEVMEL
VELTPLRSALVGLPGVNGLSTEQRKRLTIAVELVANPSIIFMDEPTSGLDARAAAIVMRTVRNTVDTGRTVVCTI
HQPSIDIFEAFDELFLMKRGGNEIYVGPLGHHSCHLIRYFESIPGVSKIHDGYNPATWMLEVTNLAQETMLGLD
FTDLYKKSDLYRRNKTLISELSMPCPGTKDLHFNNQYSQPFWIQCMACLWKQHWSYWRNPAYTAVRYICTIFI
ALAIGTMFWDLGTKVGKKQDLFNALGSLYTPVFFLGFQNASSVLPVVAVERTVYYRERAAGMYSAIPYAFGQ
TFIEIPYVFVQAVSYGVIVYAMIGFEWTVTKFFWYLFIMFFTLLYFTFYGMMSVAITPNQHVAQIVSVSGYGM
WNLFSGFIVPRPSMPIWWRWYYWADPVAWTLYGLVASQFGDLQNKITDSDETAKQFLRRYFGFKHDFVGVA
AVVTVAYTLVFAFTFALAIKVFNFQKR

>XP_004252847.1_PDR1_Sl
MELTNSRGTSLREKIIRGSSLRGSLSRKRNSTNNSRWNGNDGEIFNRSTRDEDDEEALKWAALEKLPTFDRLRK
GLLFGSQGASAEIDIHDIGFQERNKLLERLVKVADEDNEKLLLKLKQRIDRVGIDLPEIEVRYEHLTIEADAYVG
SRALPTFINFISNFFEDILNSVHILPSRKRKLTILNDVSGIIKPRRLTLLLGPPSSGKTTLLLALAGKLDSALKVTGK
VTYNGHEMNEFVPQRTAAYISQYDLHIGEMTVRETLEFSARCQGVGSSYELLVELSRREKAAKIKPDPDIDIFM
KALATEGQEAVFVTDYVLKLLGLDICADTMVGDEMIRGISGGQKKRVTTGEMLVGPSKALFMDEISTGLDSST
TYSIVNSLRQSVQILHGTAVISLLQPAPETYNLFDDIILLSDEKIVYQGPREDVLGFFESMGFKCPDRKGVADFLQ
EVTSKKDQQQYWVRRDETYRFITSKEFAEAHQSFHVGRKLADKLAASYDKSKSHPAALSTQKYGIGKKQLLK
VCTERELLLMKRNSFVYIFKFIQLTIVALISMTLFFRTKMPRDTIEDGVKYVGALFLVVTQIMFNGMAEIALTIY
KLPVFYKQRDLLFYPSWAYAVPTWILKMPITFAEVGLWVFLTYYVIGFDPSAARFFKQFLLLISLNQMASALFR
FIGAAGRTMGVANTFGTFVLLLQFALGGFVLSRVDVKKWWLWGYWSSPMMYAMNSILVNEFDGKKWKQIA
PNGTDSLGVTVVRSRGFFTNAYWYWIGVGAQIGFTIVFNICYSIALAYLNPFGKPQGMISEDSNDAKTTSTEKE
VSTSEGQNKKKGMVLPFEPHSITFDEVTYSVDMPQEMKNQGVTEDRLVLLNGVSGAFRPGVLTALMGVSGAG
KTTLMDVLAGRKTGGYIEGSIKVSGYPKKQETFARISGYCEQNDIHSPYVTVYESLVYSAWLRLPSDVGEKTR
KMFVDEVMELVELTPLRSALVGLPGVNGLSTEQRKRLTIAVELVANPSIIFMDEPTSGLDARAAAIVMRTVRNT
VDTGRTVVCTIHQPSIDIFEAFDELFLMKRGGNEIYVGPLGHHSCHLIRYFESIPGVSKIRDGYNPATWMLEVTN
SAQEMMLVLDFTDLYKKSDLYRRNKILISELSVPRPGTKDLHFKNQYSQTFWTQCLACLWKQHWSYWRNPT
YTAVRYIFTVIIALAIGTMFWDLGTKVSKSQDLFNAMGSMYAPVLFLGFQNASSVMPVVAVERTVFYRERAA
GMYSSLPYAFGQTFIEIPYVFVQAVTYAVIIYAMIGFEWTVSKFFWYLFIMYFTFLYFTFYGMMSVAVSPNQNI
AQIVSLFGYSMWNLFSGFMIPRPSMPIWWRWYYWADPVAWTLYGLVVSQFGDLQDKITDIDETSKQFLRRYF
GFKHDFLGVVAAVTVAYAVVFAFTFGLAIKFFNFQKR

>XP_004253003.1_PDR1_-l_Sl
MEPIDFGNLRGSSLRGSVKGSSRGSFRSDSNSIFRNNNIFNRSSRDEDDEEALKWAALEKLPTFDRLRKGILFGA
NEIDIHDLGNQQSKDLVDRLVKVADEDNEKFLLKLRDRIDRVGIDLPTIEVRYEHLKIEADAYVGSSALPTFINF
VTNFIEPLLYSLHIVPNRKRKLTILDDVSGIIKPCRLTLLLGPPGSGKTTLLLALAGKLDTELKASGKVTYNGHE
MNEFVPQRTAAYISQHDLHIGEMTVRETLQFSARCQGVGSRYEMLAELSRREKTANIKPDPDIDVFMKAAATE
GQEANVVTDYVLKILGLDICADTMVGDEMVRGISGGQKKRVTTGEMLVGPSKALFMDEISTGLDSSTTYSIVN
SLRQTVQILKGTAVISLLQPAPETYNLFDDIILLSDSVIVYQGPREDVIGFFESMGFKCPERKGVADFLQEVTSKK
DQQQYWVRRDEPYRFITSKEFSEAYQAFHVGRKLGNDLAVSFDKRKSHPAALTTEKYGIGKKQLFEVCKERE
YLLMKRNSFVYIFKFCQLLIMALISMTIFFRTEMKHDTIDDGGIYSGALFFVIIMNMFNGMSELGMIIYKLPVFFK
QRDLLFFPAWAYAIPSWILKIPVTFVETALWVFLTYYVMGFDPHPSRLFKQFLLLIIVSQMASGLFRFIGAVGRS
LGVASIFGSFALLLQFALGGFVLSRDDVKSWWIWGYWTSPMMYSVNAILVNEFDGKRWKHIPPNGTEPLGAA
VVRGRGFFPDASWYWIGFGALVGFTIVFNICYTIALTYLKPFGKPQAMIPEDSEDAQTTSAETEDSNSESQNKK
KGMVLPFEPHSITFDDVMYSVGMPQEMKDQGATEDRLVLLKGVSGAFRPGVLTALMGVSGAGKTTLMDVLA
GRKTGGYIEGDIKISGYPKKQDTFARISGYCEQNDIHSPYVTVYESLVYSAWLRLPHNVDTKTRKMFVEQVMD
LVELGPLRSALVGLPGINGLSTEQRKRLTIAVELVANPSIIFMDEPTSGLDARAAAIVMRTVRNTVDTGRTVVCT
IHQPSIDIFEAFDELFLMKRGGQEIYVGPLGRHSCHLIKYFESMPGVSKIKDGYNPATWMLEVTASAQEILFGVD
FTDLYKKSDLYTRNKALISELSVPRPGTKDLHFDTKYSQPFWTQCIACLWKQHWSYWRNPTYTAVRFLFTTIIA
LVFGTMFWDIGGKVSKSQDLFNAMGCLYATVLFLGTQNSSSVQPVVAVERTVFYRERAAGMYSALPYAFGQI

SIEIPYVFMQSVFCGAIMYAMIGFEWTVAKFFWYLFFLFFTLLYFTFYGMMTVAVTPNVSVAQIVGSFFYGVW
NLFSGFIVPRTRIPIWWRWYYWCCPVAWTLYGLVASQFGDLQNKLTDEETVEQFLRRYFGFKHDFLPIVAVAI
VGYTVLFGFTFAFAIKAFNFQTR

>XP_006349934.1_P_PDR1_-l_St
MEPIDFGNLRGSSLRGSVKGSFRGSFGSDSNSIFRNNNIFNRSSRDEDDEEALKWAALEKLPTFDRLRKGLLFGA
NEVDIHDLGNQQSKDLVDRLVKVADEDNEKFLLKLRDRIDRVGLDLPTIEVRYEHLKIEADAYVGSSALPTFIN
FVTNFIEPMLNSLHIVPNRKRKLTILDDMSGIIKPCRLTLLLGPPGSGKTTLLLALAGKLDTELKASGKVTYNGH
EMNEFVPQRTAAYISQHDLHIGEMTVRETLEFSARCQGVGSRYEMLAELSRREKTANIKPDPDIDVFMKAAAT
EGQEANVVTDYVLKILGLDICADTMVGDEMVRGISGGQKKRVTTGEMLVGPSKALFMDEISTGLDSSTTYFIV
NSLRQTVQILKGTAVISLLQPAPETYNLFDDIILLSDSVIVYQGPREDIIGFFESMGFKCPERKGVADFLQEVTSK
KDQQQYWVRRDEPYRFITSKEFSEAYQAFHVGRKLGEDLAVSYDKRKSHPAALTTEKYGIGKKQLFEVCKER
EYLLMKRNSFVYIFKFCQLLIMALISMTIFFRTEMKHDTMDDGGIYAGALFFVIIMNMFNGMSELGMIIYKLPVF
FKQRDLLFFPAWAYAIPSWILKIPVTFVETALWVFLTYYVMGFDPHPSRLFKQFLLLIIVSQMASGLFRFIGAVG
RSLGVASIFGSFALLLQFALGGFVLSRDDVKNWWIWGYWTSPMMYSVNAILVNEFDGKRWKHIPPNGTESLG
AAVVRGRGFFPDASWYWIGFGALVGFTIVFNICYTIALAYLKPFGKPQAMIPEDTEDAQTTSAETEDSNSESQN
KKKGMVLPFEPHSITFDDVMYSVNMPQEMKDQGATEDRLVLLKGVSGAFRPGVLTALMGVSGAGKTTLMDV
LAGRKTGGYIEGDIKISGYPKKQDTFARISGYCEQNDIHSPYVTVYESLVYSAWLRLPHNVDTKTRKMFVEQV
MDLVELGPLRSALVGLPGINGLSTEQRKRLTIAVELVANPSIIFMDEPTSGLDARAAAIVMRTVRNTVDTGRTV
VCTIHQPSIDIFEAFDELFLMKRGGQEIYVGPLGRHSFHLIKYFESMPGVSKIKDGYNPATWMLEVTASAQEILF
GVDFTDLYKKSDLYTRNKALISELSVPRPGTKDLHFDTKYSQPFWTQCIACLWKQHWSYWRNPTYTAVRFLF
TTIIALVFGTMFWDIGGKVSKSQDLFNAMGCLYATVLFLGTQNSSSVQPVVAVERTVFYRERAAGMYSALPY
AFGQISIEIPYVFMQSVFCGAIMYAMIGFEWTVAKFFWYLFFLFFTLLYFTFYGMMTVAVTPNVSVAQIVGSFF
YGVWNLFSGFIVPRTRIPIWWRWYYWCCPVAWTLYGLVASQFGDLQNKLTDEETVEQFLRRYFGFKHDFLPI
VAVAIVGYTVLFGFTFAFAIKAFNFQTR

>XP_006360347.1_P_PDR1_St
MEPANLGNLRGSSLRGSISGSRRGSVSLRANSNSIWRNTGVEIFSRSARDEDDEEALKWAALEKLPTFDRLRKG
LLFGSQGAAAEIDINDIGYQERKNLLERLVRVAEEDNEKFLLKLKNRIDRVGIDLPTIEVRYENLNIEADAYVGS
RGLPTVINFMTNFIETLLNTLHILPSSKRQITILKDISGIIKPCRMTLLLGPPSSGKTTLLLALAGKLDSSLKVTGKV
SYNGHELHEFVPQRTAAYISQHDLHIGEMTVRETLEFSARCQGVGSRYEMLAELSRREKAANIKPDPDIDIYMK
ASATEGQEANVVTDYVLKILGLDICADTMVGDEMLRGISGGQKKRVTTGEMLVGPSKALFMDEISTGLDSSTT
YSIVNSLRQSVQILKGTAVISLLQPAPETYNLFDDIILISDGYIVYQGPRDDVLEFFESMGFKCPERKGVADFLQE
VTSKKDQPQYWSRRNEHYRFISSKEFSDAFQSFHVGRKLGDELAIPFDRTKCHPAALTNEKYGIGKKELLKVCT
EREYLLMKRNSFVYVFKFVQLTIMALMTMTLFFRTEMPRDTVDDGGIYAGALFFVVVMIMFNGMSEMAMTIF
KLPVFYKQRDLLFFPSWAYAIPSWILKIPVTLVEVGLWVILTYYVIGFDPNITRFLKQFLLLVLVNQMASGLFRF
MGAVGRTMGVASTFGAFALLLQFALCGFVLSREDVKGWWIWGYWISPLMYSVNSILVNEFDGNKWKHIAPN
GTEPLGVAVVKSRGFFADAYWYWIGFAALFGFTIVFNFFYSLALAYLKPYGKSQTVRPEDSENAENGQAASQ
MASTDGGDIVSAGQSKKKGMVLPFEPHSITFDDVVYSVDMPQEMKEQGAGEDRLVLLKGVSGAFRPGVLTAL
MGVSGAGKTTLMDVLAGRKTGGYIDGDIKISGYPKKQETFARISGYCEQNDIHSPYVTVYESLVYSAWLRLPK
DVDEKIRKMFVDEVMELVELEPLRSALVGLPGVNGLSTEQRKRLTIAVELVANPSIIFMDEPTSGLDARAAAIV
MRTVRNTVDTGRTVVCTIHQPSIDIFEAFDELFLMKRGGQEIYVGPLGRHSCHLIKYFESIPGVAKIKEGYNPAT
WMLEVTASAQEMMLGVDFTDLYKNSDLYRRNKALITELSVPRPGSKDLYFETQYSQSLWIQCMACLWKQNW
SYWRNPAYTAVRFIFTMFIALVFGTMFWDIGTKVSQSQDLFNAMGSMYAAVLFLGVQNASSVQPVVDVERTV
FYRERAAGMYSAIPYAFGQVFIEIPYVFVQAIVYGIIVYAMIGFEWETGKVFWYLFIMYTTLLYFTFYGMMSVA
VTPNQNVASIVAAFFYAIWNLFSGFIVPRPRMPIWWRWYYWCCPVAWTLYGLVASQFGDIQTKLVDEETVEQ
FLRRYFGFRHDFLPVVAGVLVAYVVVFAFTFAFAIKAFNFQRR

>XP_006360348.1_P_PDR1_-l_X1_St
MEPSDLSNLRGRSIRGSMRGSMRENSNSIWRNNGVEVFSRSNRDEDDEEALKWAALEKLPTYDRLRKGILFGS
QGVAAEVDVDDLGVQQRKNLLERLVKVADEDNEKFLLKLKNRIDRVGIDFPSIEVRFEHLNIEADAYVGSRAL
PTFTNFISNFIESLLDSIHIFPSKKRSVTILKDVSGYVKPCRMTLLLGPPGSGKTTLLLALAGKLDSDLRVTGKVT
YNGHELHEFVPERTAAYISQHDLHIGEMTVRETLEFSARCQGVGSRYEMLAELSRREKAANIKPDVDIDMFMK
AVSTEGQESKVITDYVLKILGLDICADTMVGDQMIRGISGGQKKRVTTGEMIVGPSKALFMDEISTGLDSSTTY

SIVNSLKQSVQILKGTALISLLQPAPETYNLFDDIILLSDGYIVYQGPREDVLEFFESMGFKCPDRKGVADFLQEV
TSKKDQQQYWVRRDEPYRFITSKEFAEAYQSFHVGRKVSNELSTAFDKSKSHPAALTTEKYGIGKKQLLKVCT
EREFLLMQRNSFVYIFKFFQLMVIALMTMTIFFRTEMPRDTETDGGIYTGALFFTVVMLMFNGLSELPLTLYKL
PVFYKQRDFLFYPSWAYAIPSWILKIPVTLLEVGMWTVLTYYVIGFDPNVGRFFKQFLLLVLVNQMASGLFRFI
AAVGRTMGVASTFGACALLLQFALGGFALARTDVKDWWIWGYWTSPLMFSVNAILVNEFDGEKWKHTAPN
GTEPLGPSVVRSRGFFPDAYWYWIGIGALAGFTILFNIAYSLALAYLNPFGKPQATISEEGENNESSGSSPQITST
AEGDSVGENQNKKKGMVLPFEPQSITFDEVVYSVDMPPEMREQGSSDNRLVLLKGVSGAFRPGVLTALMGVS
GAGKTTLMDVLAGRKTGGYIDGSIKISGYPKKQETFARISGYCEQNDIHSPYVTVYESLVYSAWLRLPQDVDE
HKRMMFVEEVMDLVELTPLRSALVGLPGVNGLSTEQRKRLTIAVELVANPSIIFMDEPTSGLDARAAAIVMRA
VRNTVDTGRTVVCTIHQPSIDIFEAFDELFLMKRGGQEIYVGPLGRESCHLIKYFESMPGVGKIEEGYNPATWM
LEVTSSSQEMSLGVDFTDLYKNSDLCRRNKALITELSVPRPGTSDLHFENQFSQPFWVQCMACLWKQRWSYW
RNPAYTAVRFLFTTFIALIFGSMFWDLGTKVSRPQDLTNAMGSMYAAVLFLGVQNASSVQPVVSVERTVFYRE
KAAGMYSAIPYAFAQVFIEIPYVFVQSVVYGLIVYSMIGFEWTVAKFFWYFFFMFFTFLYFTFFGMMTVAITPN
QNVASIVAGFFYTVWNLFSGFIVPRPRIPIWWRWYYWGCPVAWTLYGLVASQFGDLQDIVNGQTVEEYLRND
YGIKHDFLGVVAGVIVAFAVVFAFTFALGIKAFNFQKR

>XP_006365806.1_P_PDR1_-l_St
MEPTNSRGTSLREKIIRGSSLRGSLSSKRNSTNNSRWSGNDGEIFNRSTRDEDDEEALKWAALEKLPTFDRLRK
GLLLGSQGASAEIDIHDIGFQERNKLLERLVKVADEDNEKLLLKLRQRIDRVGIDLPEIEVRYEHLTIEADAYIGS
RALPTFINFITNFLEDILNPLHILPSRKRKLTILNDVSGIIKPRRLTLLLGPPSSGKTTLLLALAGKLDSALKVTGKV
TYNGHEMNEFVPQRTAAYISQYDLHIGEMTVRETLEFSARCQGVGSSYEMLVELTRREKEAKIKPDPDIDIFMK
ALAAEGQEANFVTEYVLKLLGLDICADTMVGDEMIRGISGGQKKRVTTGEMLVGPSKALFMDEISTGLDSSTT
YSIVNSLRQSVQILHGTAVISLLQPAPETYNLFDDIILLSDGKIVYQGPREDVLGFFESMGFKCPDRKGVADFLQ
EVTSKKDQQQYWVRRDETYRFITSKEFAEAYQSFHVGRKLVDDLAASYDKSKSHPAALSTQKYGIGKKQLLK
VCTEREFLLMKRNSFVYIFKFIQLTIMALISMTLFFRTKMPRDTIEDGVKYVGALFFVVTMIMFNGMAEIALTIY
KLPVFYKQRDLLFYPSWAYAMPTWILKIPITFVEVGLWVFLTYYVIGFDPSPARFFKHFLLLILVNQMASGLFRF
IGATGRTMGVANTFGTFVLLLQFALGGFVLSRDDVKKWWLWGYWSSPMMYSMNSILVNEFGGKRWKQIAPI
GTDSLGVTVVRSRGFFTNAYWYWIGVGALIGFTIVFNICYSLALAYLNPFGKPQGMISEDSDDAKTTSTEKEVS
TSEGQNKKKGMVLPFEPHSITFDEVTYSVDMPQEMKNQGVTEDRLVLLNGVCGAFRPGVLTALMGVSGAGK
TTLLDVLAGRKTGGYIEGSIKISGYPKKQETFARISGYCEQNDIHSPYVTVYESLVYSAWLRLPSDVDEKTRKM
FVDEVMELVELTPLRSALVGLPGVNGLSTEQRKRLTIAVELVANPSIIFMDEPTSGLDARAAAIVMRTVRNTVD
TGRTVVCTIHQPSIDIFEAFDELFLMKRGGKEIYVGPLGHHSCHLIRYFESIPGVSKIQDGYNPATWMLEVTNSA
QEMMLGVDFTDLYKKSDLYRRNKILIRELSVPGPGTKDLHFNNQYSQPFWTQCMACLWKQHWSYWRNPAYT
AVRYIFTIIIALAIGTMFWDLGTKVSKSQDLFNAMGSMYAPVLFLGFQNASSVMPVVAVERTVFYRERAAGMY
SSLPYAFGQAFIEIPYVFVQAVTYGVIIYAMIGFEWTVTKFFWYLFIMYFTLLYFTFYGLMSVAVSPNQNIAQIV
SLFGYAMWNLFSGFMIPRPSMPIWWRWYYWACPVSWTLYGLVASQFGDLQDKLTDSDETAKHFLRRYFGFK
HDFLGVVAFVTVAYAVVFAFTFALAIKVFNFQKR

>XP_006366077.1_P_PDR1_-l_X1_St
MEGGENILRVSSARLSGSNVWRNSAMDVFSRSSSREDYDDEEALRWAALEKLPTYRRIRRGLLLEEEEGQSRE
VDITKLDLIERRNLLDRLVKIADEDNEKLLMKLKQRIDRVGLDLPTIEVRFEHLNIDAEARVGSRALPTIFNFTV
NILEDFLNYLHILPSRKKPLPILHGVGGIIKPGRMTLLLGPPSSGKTTLLLALAGKLDNDLKVSGRVTYNGHGM
DEFVPQRTSAYISQNDLHIGEMTVRETLAFSARCQGVGTKYEILAELSRREKEANIKPDPDIDIFMKSAWNEGQ
EANVITDYTLKILGLEICADTLVGDEMIRGISGGQRKRLTTGEMMVGPARALFMDEISTGLDSSTTYQIVNSIRQ
SIHILQGTAVISLLQPAPETYDLFDDIILLSDGQIVYQGPRENVLEFFEYLGFKCPQRKGVADFLQEVTSRKDQEQ
YWSRRDEPYRFITACEFSDVFQSFDVGRKLGDELAVPFDKSKSHPAALTTKRYGISKKELLKACTAREYLLMK
RNSFVYIFKMVQLTLMASIAMTLFLRTEMHRDTTIDGAIYLGALFYAVITIMFNGFSELALSIMKLPSFYKQRDF
LFFPAWAYALPTWILKIPITLVEIAIWVCMTYYVIGFEADVGRFFKQIFLLICLSQMASGLFRFLAALGRNIIVAN
TFGSCALLIVLVMGGFILSRDDVKQWLIWGYWISPMMYAQNAIAVNEFLGKSWAHVPPNSTGTDTLGVSFLKS
RGIFPEARWYWIGAGALFGYVLLFNFLFTVALAYLNPFSKPQAILSEEIVAERNASKRGEVIELSPIGKSSSERGN
DVPVSTSSRSLSTRVGNITEGDLNKRKGMILPFEPLSITFDDIRYAVDMPQEMKTQGFIEDRLELLKGVSGAFRP
GVLTALMGVSGAGKTTLMDVLAGRKTGGYVEGTISISGYPKQQETFARISGYCEQTDIHSPHVTVYESLLYSA
WLRLPREVDTETRKSFIEEVMELVELTPLREALVGLPGVNGLSTEQRKRLTVAVELVANPSIIFMDEPTSGLDA
RAAAIVMRTVRNTVDTGRTVVCTIHQPSIDIFDAFDELLLLKRGGEEIFVGPLGRHSSHLIKYFEGIDGVLKIRDG

YNPATWMLEVTSLAQEAVLGIDFTELYKNSELYRRNKALIQELSVPASGSKDLYFETKYSQSFFTQCMACLWK
QHWSYWRNPPYTAVRLMFTFFVSLMLGTIFWGLGSKRGKQQDILNAIGSMYAAILFLGIINASSVQPVVAIERT
VFYRERAAGMYSALPYAFGQVMIELPHLFLQTIIYGVIVYAMIGFEWTVTKFFWYLFFMYFTLLYFTLYGMMT
VAVTPNHTIASIVSSAFYTIWNLFCGFVVPKTRMPVWWRWYYYICPLSWTLYGLIASQFGDLQDRLDTKETVE
EFLENFFDYKHDFVGYVAVILVGISVVFLFIFAYSIKSFNFQKR

>XP_006366078.1_P_PDR1_-l_St
MEGGGDILKVSSARLSSSNVWRNSAMDVFSRSSREDYDDEEALKWAALEKLPTYLRIRRGILSEEEGQYREVD
ITKLDLVERRNLLERLVKIADEDNEKFLLKLKKRIDRVGLDLPTIEVRFEHLNVDAEARVGSRALPTIFNFTVNIL
EDFLNYLHILPSRKKPLPILHDVSGIIKPGRMTLLLGPPSSGKTTLLLGLAGKLDKDLKVSGRVTYNGHGMDEF
VPQRTSAYISQNDLHIGEMTVRETLAFSARCQGVGAKYEILAELSRREKEANIKPDPDVDIFMKSAWNDGQEA
NVVTDYTLKILGLEICADTIVGDEMIRGISGGQRKRLTTGEMMVGPARALFMDEISTGLDSSTTYQIVNSIRQSI
HILQGTAVISLLQPAPETYDLFDDIILLSDGQIVYQGPRENVLEFFEYLGFKCPQRKGVADFLQEVTSRKDQEQY
WARRDEPYKFITVREFSEAFQSFHVGRKLGDELAVPFDKSKSHPAALTTKRYGVSKKELLKACTAREYLLMKR
NSFVYIFKMIQLTLMATITMTLFLRTEMHRNTMIDGAVFLGALYYAVIMIMFNGFSELALSIMKLPSFYKQRDL
LFFPAWTYALPTWILKIPITLVEVAIWVCMTYYVIGFEADVGRFFKQLFLLICLNQMASGLFRFLAALGRNIIVA
NTFGSCALLIVLVMGGFILSRDNVKQWLIWGYWISPMMYAQNAIAVNEFLGKSWAHVPPNSTGTDTLGVSFL
KSRGIFPEARWYWIGVGALLGYVLLFNFLFTVALAYLNPFGKPQAVLSEETVAERNASKKGEVIELSPIGKSSSE
RGNDVRRSASSRSMSSRVGNITEGDINKRKGMILPFEPLSITFDDIRYAVDMPQEMKSQGFIEDRLELLKGVSGA
FRPGVLTALMGVSGAGKTTLMDVLAGRKTGGYIEGTISISGYPKQQETFARIAGYCEQTDIHSPHVTVYESLQY
SAWLRLPREVDTETRKRFIEEVMELVELKPLREALVGLPGVNGLSTEQRKRLTVAVELVANPSIIFMDEPTSGL
DARAAAIVMRTVRNTVDTGRTVVCTIHQPSIDIFDAFDELLLLKRGGEEIFVGPLGRHSSHLIKYFEGIDGVPKIR
DGYNPATWMLDITSVAQEAALGVDFTELYRNSELYRRNKALIKELSVPAPGSKDLYFQTKYSQSFFTQSMACF
WKQHWSYWRNPPYTAVRLLFTFFIALMFGTIFWDLGSKRRRQQDILNAIGSMYASVLFLGVQNATSVQPVVAI
ERTVFYRERAAGMYSALPYAFGQIMIELPYIFIQTIIYGVIVYAMIGFEWTVAKFFWYLFFMYFTLLYFTLYGM
MTVAVTPNHSIAAIVSSAFYAVWNLFSGFIVPKTRMPVWWRWYYYICPISWTLYGLIASQFGDLQDKLETKET
VEEFIESFFDFKYDFVGYVAVILVGISVLFLFIFAYSIKAFNFQKR

>XP_006576269.1_PDR-l_X1_Gm
MEGGGSSFRIGSSSIWRNSDAAEIFSNSFHQENDEEALKWAAIQKLPTVARLRKALITSPDGESNEIDVKKLGLQ
EKKALLERLVKTAQEDNEKFLLKLKDRIDRVGIDLPTIEVRFENLSIEAEARAGTRALPTFTNFIVNILEGLLNSL
HVLPNRKQHLNILEDVSGIIKPGRMTLLLGPPSSGKTTLLLALAGKLDPKLKFSGKVTYNGHGMNEFVPQRTA
AYVNQNDLHVAELTVRETLAFSARVQGVGPRYDLLAELSRREKEANIKPDPDIDAYMKAVASEGQKANMITD
YILRILGLEVCADTVVGNAMLRGISGGQRKRVTTGEMLVGPAKALFMDEISTGLDSSTTFQIVNSLKQYVHILK
GTTVISLLQPAPETYNLFDDIILLSDSHIVYQGPREHVLEFFELMGFKCPQRKGVADFLQEVTSRKDQEQYWAH
KDQPYRFVTAKEFSEAHKSFHIGRSLGEELATEFDKSKSHPAALTTKMYGVGKWELLKACLSREYLLMKRNSF
VYTFKLCQVGLEIGMFHFIVLSPNAIIILLKCFPSFQLAVLAIIAMTIFLRTEMHRDSVTHGGIYVGALFYGVVVI
MFNGLAELSMVVSRLPVFYKQRDYLFFPSWVYALPAWILKIPLTFVEVGVWVFLTYYAIGFDPYVGRLFRQYL
VLVLVNQMASALFRLVAAVGREMTVALTLGSFTLAILFAMSGFVLSKENIKKWWLWGFWISPMMYGQNAM
VNNEFLGKRWRHFLPNSTEALGVEILKSRGFFTQSYWYWIGVGALIGYTLLFNFGYILALTYLNPLGKHQAVIS
EEPQINDQSGDSKKGTNVLKNIQRSFSQHSNRVRNGKSLSGSTSPETNHNRTRGMILPSEPHSITFDDVTYSVDM
PVEMRNRGVVEDKLALLKGVSGAFRPGVLTALMGVTGAGKTTLMDVLAGRKTGGYIGGNITISGYPKKQETF
ARISGYCEQNDIHSPHVTVYESLLYSAWLRLSPEINADTRKMFIEEVMELVELKALRNALVGLPGINGLSTEQR
KRLTIAVELVANPSIIFMDEPTSGLDARAAAIVMRTVRNTVDTGRTVVCTIHQPSIDIFESFDELLLMKQGGQEIY
VGPLGHHSSHLINYFEGIQGVNKIKDGYNPATWMLEVSTSAKEMELGIDFAEVYKNSELYRRNKALIKELSTPA
PGSKDLYFPSQYSTSFLTQCMACLWKQHWSYWRNPLYTAIRFLYSTAVAAVLGSMFWDLGSKIDKQQDLFNA
MGSMYAAVLLIGIKNANAVQPVVAVERTVFYREKAAGMYSALPYAFAQVLIELPYVLVQAVVYGIIIYAMIGF
EWTVTKVFWYLFFMYFTFLTFTYYGMMSVAVTPNQHISSIVSSAFYAVWNLFSGFIVPRPRIPVWWRWYSWA
NPVAWSLYGLVASQYGDIKQSMESSDGRTTVEGFVRSYFGFKHDFLGVVAAVIVAFPVVFALVFAISVKMFNF
QRR

>XP_006597138.1_PDR1_X1_Gm
MLQESSPVYSLNFSVESLYFTRSNFTYIFMHATLLFHITSQNCTSREKVCCSSIFLTLSLLTMENDSSLRVSSSIRR
DASDIFSPSSFEEDDEEALKWAALDKLPTYNRLKKGLLITSNGEVNEIDVTDMGTQRRKEVLERLVRDAEEDN

EKFLLKLRERIDRVGVSIPTIEARFEHLNVEAEAYVGSRALPTFFNFIVNTVESYLNYLHILSSKKKHVTILKDVS
GIVKPCRMTLLLGPPSSGKTTLLLALAGKLDPDLKVSGRVTYNGHGMNEFVPQRTAAYISQDDVHIGEMTVRE
TLAFSARCQGVGSRYDMLSELSRREIVTDIKPDPNIDIYMKAIASEGQEANQMMTEYVLKILGLEMCADIVVGD
EMLRGISGGQRKRVTTGEMLVGPTNALFMDEISSGLDSSSTVQIIKCLRQMVHILDGTAVISLLQPEPETYELFD
DIILLSDGQIVYQGPREFVLEFFESKGFRCPERKAVADFLQEVTSRKDQQQYWIHKDEPYSFVSVNEFAEAFRCF
HVGRKLGDELAVPFDKTKNHPAALTTKKYGVNKKELLKANFSREYLLMKRNAFVYIFKLSQLALMAVVAMT
VFLRTEMHKDSVDNGGVYTGALFFSIVMILFNGMADISMTVAKLPIFYKQRDLLFYPAWAYAIPGWILKIPITL
AEVVVWVSITYYVIGFDPSVARFFKQYLLLLLLGQMASALFRTIAAIGRNMIIANTFGSFAIVTLLTLGGFILSRE
DVKKWWIWGYWISPIMYEQNAMMVNEFLGQSWSHVLPNSTESLGVEVLKSRGFFTHASWYWIGAGALLGFV
VLLNITFTLALTYLNPPEMSRAVIFKESHGNRNKDRTLDDIRLSLRLTGNAPSSNLEIGNLDDNGTESMSSRSAS
VRPKAAVESSHRRKRGMVLPFEPHSLTFDGITYSVDMPQEMKNQGVVEDRLVLLKGVSGAFRPGVLTALMGV
SGAGKTTLMDVLAGRKTGGYIEGSITISGYPKNQETYAQISGYCEQNDIHSPHVTIYESLLYSAWLRLSPEVNSE
TRKMFIEEVMELVELNLLREALVGLPGVSGLSTEQRKRLTIAVELVANPSIIFMDEPISGLDARAAAIVMRTVRN
IVDTGRTIVCTIHQPSIDIFEAFDELFLLKRGGREIYVGPLGRHSNHLVEYFERIEGVGKIKDGHNPAAWMLEITT
PAREMDLNVDFSDIYKNSVLCRRNKALVAELSKPAPGSKELHFPTQYAQPFFVQCKACLWKQHWSYWRNPPY
TAVRFLFTTFVALMFGTMFWDLGSKTRRKQDLFNAIGSMYNAILFLGIQNALSVQPVVAIERTVFYRERAAGM
YSAIPYALAQVVIELPYIFVQAVTYGIIVYAMIGFEWTASKFFWYLFFMYFTFLYFTFYGMMTVAVTPNQHIASI
VATAFYGIWNLFSGFVVPRPSIPVWWRWYYWACPVAWSLYGLVASQFGDITSAVELNETVKEFLRRYFGYRD
DFVGVAACVVVGFAVLFATIFAFSLKVFNFERR

>XP_008656640.1_ABCG36_Zm
MDAAGDIQKVASMRRGDSGSMWRRGDDVFSRSSREEDDEEALRWAALEKLPTYDRIRRAIVPLGLGDEAPGS
KGLVDVDVLSLGPRERRALLERLVRVADEDNERFLLKLKDRIDRVGIDMPTIEVRFQNLEAEAEVRVGSSGLPT
VLNSVVNTVEEAANALHILPSRKQIMPILHDVSGIIKPRRLTLLLGPPGSGKTTLLLALAGRLDKDLKFSGKVTY
NGHEMTEFVPERTAAYISQHDLHIGEMTVRETLAFSARCQGVGSRLDMLTELSRREKAANIKPDADIDAFMKA
AALGGQDANVVTDYILKILGLDICADTMVGDEMLRGISGGQRKRVTTGEMLVGPARALFMDEISTGLDSSTTF
QIVNSLRQSIHILGGTAVISLLQPAPETYNLFDDIILLSDGQVVYQGPREEVVEFFESVGFRCPERKGVADFLQEV
TSKKDQKQYWARPDEPYRFVSVKELATAFKSSHTGRALANELAVPFDKSKSHPAALTTTRYGVSGKELLKANI
DREILLMKRNSFVYMFRTFQLMVMSIIAMTLFFRTKMKHDTVNDGGIYMGALFFGVLMIMFNGLSELALTVFK
LPVFFKQRDLLFFPAWSYTIPAWILKVPITFIEVGGYVFLTYYVIGFDPNVGRFFKQYLLLLAVNQMTAALFRFV
GGVSRNMIVANVFASFMLLVVMVLGGFILQRDKVKKWWIWGYWISPMMYAQNAISVNEMLGHSWDKILNS
TASNETLGVQVLKSRGVFPEAKWYWIGFGAMVGFTILFNALFTLALTYLKPYGNSRPSVSKEELKEKHANIKG
EVVDGNHLVSVNPVTDSAIMEDDSASTKKGMILPFVPLSVTFDNIKYSVDMPQEMKGQGVQEDRLELLKSISG
SFRPGVLTALMGVSGAGKTTLMDVLAGRKTGGYIEGDIRISGYPKKQETFARVSGYCEQNDIHSPQVTVYESL
LFSAWLRLPKDVDSNKRKIFIEEVMELVELKPLRNALVGLPGVNGLSTEQRKRLTIAVELVANPSIIFMDEPTSG
LDARAAAIVMRTVRNTVDTGRTVVCTIHQPSIDIFEAFDELFLMKRGGEEIYAGPLGHNSSELIKYFEEIQGVSKI
KDGYNPATWMLEVTTISQEQILGVDFSDIYKKSELYQRNKALIKELSQPAPGSTDLHFSSKYAQSFNTQCVACL
WKQNLSYWRNPPYNTVRFFFTGIIALLLGTIFWDLGSKVYTSQDLLNAMGSMYSAVLFIGVMNCTSVQPVVA
VERTVFYRERAAGMYSAFPYAFGQVVIELPYALAQDILYAVIVYSMIGFEWTVAKFFWYLFFGYFTLLYFTFY
GMMTVGLTPNYHIAAIVSAAFYAIWNLFSGFVIPRPKVPIWWRWYCWICPVAWTLYGLVVSQYGDIMTEMDD
KRTVKVFVEDYFDFKHSWLGWVAAVVVAFGVLFATLFAFAIMKLNFQKR

>XP_008679861.1_ABCG39_Zm
MDLVQMGSIAGGSMRRTASSWRASGRSDAFGRSVREEDDEEALRWAAIEKLPTYDRMRKGILTGNAAGAGV
EEVDIQGLGMQERKNLIERLVRTAEEDNERFLLKLRDRMELVGIDNPTIEVRFENLNIDAEAYVGNRGVPTMT
NFFSNKVMDVLSAMHIVSSGKRPVSILHDISGVIRPGRMSLLLGPPGSGKTSLLLALSGKLDSNLKVSGRVTYN
GHDMDEFVPQRTSAYIGQHDVHVGEMTVRETLAFSARCQGVGTRYDMLTELSRREKEANIKPDPDVDVYMK
AISVEGQESVVTDYILKILGLEICADTMVGDSMIRGISGGQKKRVTTGEMLVGPAKALFMDEISTGLDSSTTYQI
VNSLRQSVHILGGTALIALLQPAPETYELFDDIVLLSEGQIVYQGPRENVLEFFEVMGFKCPERKGVADFLQEVT
SRKDQHQYWCRRDEPYRYISVNDFSEAFKAFHVGRKLGSDLKVPFDRTRNHPAALTTSKYGISKMELLRACFS
REWLLMKRNSFVYIFKVVQLIILGTIAMTVFLRTTMHRRGVEDGVIFLGAMFLGLVTHLFNGFAELAMSIAKLP
IFYKQRDLLFYPSWAYASPTWLLKIPISFLECAVWIGMTYYVIGFDPSIERFFRHYLLLVLVSQMASGLFRLLAA
LGREMVVADTFGSFAQLVLLILGGFLIARDNIKKWWIWGYWSSPLMYAQNAVAVNEFLGHSWQMVVDRTHS
NDTLGVQILKARGIFVDPNWYWIGVGALLGYIMLFNVLFVLFLDWLGPLGKGQAVVSEEELREKHVNRTGQN

VELLPLGTASQNPPSDGRGEIAGAESRKRGMVLPFTPLSITFDNIKYSVDMPQEMKDKGITEDRLLLLKGVSGA
FRPGVLTALMGVSGAGKTTLMDVLAGRKTGGHIEGDISISGYPKKQETFARIAGYCEQNDIHSPHVTVYESLLY
SAWLRLPHEVDSEARKMFVEEVMELVELTPLRGALVGLPGVNGLSTEQRKRLTIAVELVANPSIIFMDEPTSGL
DARAAAIVMRTVRNTVDTGRTVVCTIHQPSIDIFEAFDELFLMKRGGEEIYVGPLGRNSCHLINYFEGIEGVKKI
KDGYNPATWMLEVTTLAQEDILGINFAEVYRNSDLYRRNKDLISELSTPPPGSKDLYFPTQYSQSFLTQCMACL
WKQHKSYWRNPSYTATRIFFTTVIALIFGTIFLNLGKKIGTRQDLFNSLGSMYAAVLFIGIQNGQTVQPIVDVER
TVFYREKAAGMYSALPYAFAQVLIEIPHIFLQTVVYGLIVYSLIGFDWTVAKFFWYMFFMFFTFMYFTFYGMM
AVAMTPNSDIAAIVSTAFYAIWNIFAGFLIPRPRIPIWWRWYSWACPVAWTLYGLVASQFGDIADIRLEDDGEL
VKDFVNRFFGFEHDNLGYVATAVVGFTVLFAFVFAFSIKVFNFQRR

>XP_010648603.1_P_PDR1_X1_Vv
MESSDVYRVNSARLSSSNIWRNSGMEVFSRSSRDEDDEEALKWAAIEKLPTYLRIRRGILAEEEGKAREIDITSL
GLIEKKNLLERLVKIAEEDNEKFLLKLKERIDRVGLDIPTIEVRFEHITVDAEAYIGGRALPTIINFSANMLEVIHF
LDYINLCMYTLCSNLLSCKQSHAMIFLSLKGFLNYLHILPSRKKPLPILHDVSGIIKPGRMTLLLGPPSSGKTTLL
LTLAGKLGSDLKLSGRVSYNGHGMDEFVPQRSSAYISQYDLHIGEMTVRETLAFSARCQGVGTGYDMLAELS
RREKVANIKPDPDIDIYMKAAALKGQGGSLITDYILKILGLEVCADTIVGDEMVRGISGGQKRRLTTGEMLVGP
AKALFMDEISTGLDSSTTFQIVNSIRQSIHILKGTAIISLLQPAPETYDLFDDIILLSDGQIVYQGPRENVLEFFEHM
GFKCPERKGVADFLQEVTSKKDQEQYWAHRGEPYSFVTVTEFSEAFQSFHVGRRLGDELAIPFDKAKAHTAAL
TTKKYGVSKKELLKACISRELLLMKRNSFVYIFKMSQLILLAFIMMTLFLRTDMPRKTIADGWIFLGSMFFTLM
MIMFNGFSELALTIMKLPVFYKQRDLLFYPSWAYSLPTWILKIPITLVEVAIWVFMTYYVVGFDPNIERFFRQYL
LLLCVNQMASGLLRLMAALGRNIIVANTFGSFALLAVLVMGGFVLSKDDVKPWWMWGYWISPMMYGQNAI
AVNEFLGKSWRHVPENATEPLGVLVLKSRGIFPEAYWYWLGVGALIGYVFLFNFLFTVALAYLNPYGKHQTV
LSEETLTEQSSRGTSCTGGDKIRSGSSRSLSARVGSFNNADQNRKRGMILPFEPLSITFDEIRYAVDMPQEMKSQ
GIPENRLELLKGVSGSFRPGVLTALMGVSGAGKTTLMDVLAGRKTGGYIDGSIKISGYPKNQKTFARISGYCEQ
TDIHSPHVTVYESLLYSAWLRLPPEVDSATRKMFIEEVMELVELNSLRQALVGLPGVDGLSTEQRKRLTVAVE
LVANPSIIFMDEPTSGLDARAAAIVMRTVRNTVDTGRTVVCTIHQPSIDIFDAFDELFLLKRGGEEIYAGPLGHH
SAHLIKYFEGIDGVSKIKDGYNPATWMLEVTSAAQEAALGINFTDVYKNSELYRRNKALIKELSTPPPGSKDLY
FPTQYSQSFFAQCKTCLWKQHWSYWRNPSYTAVRLLFTTFIALMFGTIFWDLGSRRQRKQDLFNAMGSMYCA
VLFIGAQNATSVQPVVAIERTVFYREKAAGMYSALPYAFGQVMIELPYILIQTIIYGVIVYAMIGFDWTMTKFF
WYIFFMYFTFLYFTFYGMMAVAVSPNHNIAAIISSAFYAIWNLFSGFIVPRTRIPVWWRWYYWCCPISWTLYGL
IGSQFGDMKDKLDTGETIEDFVRSYFGFRNDFLGIVAVVIVGITVLFGFTFAYSIRAFNFQKR

>XP_010648606.1_P_PDR1_Vv
MESSDISRVTSGRITASNILRNSSVEVFSRSSREEDDEEALKWAALEKLPTFLRIQRGILTEEKGQTREINIKSLGL
PERKNLIQRLVKIDGHDNEKFLLKLKERIDRVGLDIPTVEVRFEHLTVDAEAYVGSRALPTIFNFSANILEGFLN
YLHILPSRKKPFSILHDVSGIIKPRRMTLLLGPPSSGKTTLLLALAGRLGSDLKVSGRVTYNGHGMDEFVPQRTS
AYTSQYDLHAGEMTVRETLDFSARCQGVGGLSDMLAELSRREKAANIKPDPDIDIYMKAAALEGQKTSVVTE
YMLKILGLEICADTLVGDVMKQGISGGQKKRLTTGEILVGPARALFMDEISTGLDSSTAFQIVNSLRQSIHILNG
TALISLLQPAPETYNLFDDIILLSDGKIVYQGPCENVLEFFGYMGFKCPERKGVADFLQEVTSRKDQEQYWARK
DEPYSYVTVKEFAEAFQSFHIGQKLGDELAVPFDKTKGHPAALTTKKYGISKRELLRACTSREFLLMKRNSFVL
FFLFFQLIIVAFINMTLFLRTEMSRNTVEDGGIFMGALFFAVLMIMFNGFTELPMTIFQLPVFYKQRDLLFFPSWA
YSLPKWILKMPIAFAEVGAWVIMTYYVIGFDPNIERFFKQYLLLLCIHQMASGLLRLMAALGRNIIVANTFGSF
ALLVVMVLGGFVLSKDDVKTWWEWGYWVSPLMYGQNAISVNEFLGNSWRHVPANSTESLGVLVLKARGVF
TEPHWYWLGVGALIGYVLLFNFLFTLALSYLNPFGKSQPILSKETLTEKQANRTEELIELSPETGARIQSGSSRSL
SARVGSITEADQSRKRGMVLPFEPLSISFDEIRYAVDMPQEMKAQGITEDRLELLRGVSGSFRPGILTALMGVTG
AGKTTLMDVLAGRKTSGYIEGIIKVYGYPKKQETFARVLGYCEQTDIHSPHVTVYESLLYSAWLRLPSEVDSAT
RKMFIEEVMELVELNSLREALVGLPSENGLSTEQRKRLTIAVELVANPSIIFMDEPTSGLDARAAAIVMRTVRNT
VDTGRTVVCTIHQPSIDIFDAFDELLLLKRGGEEIYAGPIGRHSSHLIKYFEGINGVSKIKDGYNPSTWMLEVTSA
AQEVALGVNFTEEYKNSELYRRNKALIKELSSPPPGSKDLYFSTQYSQSFFTQCLACLWKQHWSYWRNPAYTA
VRLFFTTFIALMLGTIFWDFGSKRKRQQDLFNAMGSMYAAVISIGIQNASSVQAVVAIERTVFYRERAAGMYSP
FPYAFGQVMIELPHIFIQTIIYGLIVYAMVGFEWTVTKFFWYLFFMYFTFLYFTFYGMMAVAITPNQHISGIVSS
AFYGLWNLFSGFIIPHTRIPVWWKWYFWSCPVSWTLYGLLVTQFGDIKERLESGERVEDFVRSYFGYRNDFVG
VVAGIVVGITVLFGFIFAYSIRAFNFQKR

>XP_010654715.1_P_PDR1_-l_Vv
MATGEIYRAGGSLRKDSSSIWRNSGEEVFSRSSRDEDDEEALKWAALEKLPTYNRMRKGLLMGSAGEASEVDI
HNLGFQEKKNLVERLVKIAEEDNEKFLLKLRNRIDRVGIDLPEIEVRFEHLTIDAEAHVGSRALPSFIYSAFNQIE
DILNTLRILPSRKKKLTILHDVSGIIKPRRMTLLLGPPSSGKTTLLLALSGKLDSSLKVTGKVTYNGHGMNEFVP
QRTATYISQHDTHIGEMTVRETLAFSARCQGVGDRYDMLAELSRREKAANIKPDPDIDVFMKAVATEGQKEN
VITDYTLKILGLEVCADTLVGDQMIRGISGGQRKRVTTGEMLVGPSKALFMDEISTGLDSSTTYQIVNSLRQTIH
ILNGTALISLLQPAPETYDLFDDIILLSDSQIVYQGPREDVLDFFESMGFRCPERKGVADFLQEVTSRKDQQQYW
ARKDEPYSFVTVKEFAEAFQSFHIGRKLGHELATPFDKTKSHPAALKTEKYGVRKKELLDACISREYLLMKRN
SFVYIFKLTQLIIMAAISMTIFLRTEMHKNSTDDGSIYTGALFFTVVMIMFNGMSELAMTIAKLPVFYKQRGLLF
YPAWAYALPSWILKIPITFVEVAVWVFMSYYVIGFDPNVGRLFKQYLLLVLVNQMASALFRFIAAAGRNMIVA
NTFGSFSLLLLFALGGFVLSRENVKKWWIWGYWSSPLMYAQNAIVVNEFLGKSWSKNSSTDSTESLGVAVLK
SRGFFTEAYWYWIGAGALLGFILVFNFCYTVALTYLNAFEKPQAVITEESENSKTGGKIELSSHRRGSIDQTAST
ERRDEIGRSISSTSSSVRAEAIAEARRNNKKGMVLPFQPLSITFDDIRYSVDMPEEMKSQGVLEDRLELLKGVSG
AFRPGVLTALMGVSGAGKTTLMDVLAGRKTGGYIEGNINISGYPKKQETFARISGYCEQNDIHSPHVTIHESLL
YSAWLRLPADVDSKTRKMFIEEVMELVELTPLKDSLVGLPGVNGLSTEQRKRLTIAVELVANPSIIFMDEPTSG
LDARAAAIVMRTVRNTVDTGRTVVCTIHQPSIDIFEAFDELLLLKRGGQEIYVGPLGRHSSHLIKYFQGIEGVSKI
KDGYNPATWMLEVTSSAQEFLLGVDFTEIYKNSDLYRRNKDLIKELSQPAPGSKDLYFPTQYSQSFFTQCMAC
LWKQRRSYWRNPPYTAVRFFFTTFIALIFGTMFWDLGTKRKKQQDLSNAMGSMYAAVLFLGVQNSSSVQPVV
AVERTVFYRERAAGMYSAMPYAFAQALVEIPYVFAQAVVYGVIVYAMIGFEWTAAKFFWYLFFMFFTLLYFT
FYGMMAVAATPNQHIAAIVAAAFYGLWNLFSGFIVPRTRIPVWWRWYYWACPVAWTLYGLVTSQFGDIQDR
FEDTGDTVEQYLNDYFGFEHDFLGVVAAVIVGFTVLFLFIFAFAIKAFNFQRR

>XP_010654716.1_P_PDR1_Vv
MATGEIYRAGGSLRKDSSSIWRNSGEEVSSRSSRDEDDEEALKWAALEKLPTYNRMRKGLLMGSAGEASEVDI
HNLGFQEKKNLVERLVKIAEEDNEKFLLKLRNRIDRVGIDLPEIEVRFEHLTIDAEAHVGSRALPSFINSAFNQIE
DILNTLRILPSRKKKFTILHDVSGIIKPRRMTLLLGPPSSGKTTLLLALSGKLDSSLKVTGKVTYNGHGMNEFVP
QRTATYISQHDTHIGEMTVRETLAFSARCQGVGDRYDMLAELSRREKAANIKPDPDIDVFMKAAATEGQKEN
VITDYTLKILGLEVCADTLVGDQMIRGISGGQRKRVTTGEMLVGPSKALFMDEISTGLDSSTTYQIVNSLRQTIH
ILNGTALISLLQPAPETYDLFDDIILLSDSQIVYQGPREDVLDFFESMGFRCPERKGVADFLQEVTSRKDQQQYW
ARKDEPYSFVTVKQFAEAFQSFHSGRKVGDELATPFDKTKSHPAALKTEKYGVRKKELLDACISREYWLMKR
NSFVYILQLTQLIIMAAISMTIFLRTEMHKNSTDDGSIYMGALFFTVVMIMFNGMSELAMTIAKLPVFYKQRGL
LFYPAWAYALSSWILKIPITFVEVAVWVFMSYYVIGFDPNVGRLFKQYLLLVLVNQMASALFRFIAAAGRNMI
VANTFGSFSLLLLFALGGFVLSRENVKKWWIWGYWSSPLMYAQNAIVVNEFLGKSWSKNSSTNSTESLGVAV
LKSRGFFTEAYWYWIGAGALLGFILVFNFCYTVALTYLNAFEKPQAVITEESENSKTGGKIELSSHRRGSIDQTA
STERRDEIGRSISSTSSSVRAEAIAEARRNTKRGMVLPFQPLSITFDDIRYSVDMPEEMKSQGVLEDRLKLLKGV
SGAFRPGVLTALMGVSGAGKTTLMDVLAGRKTGGYIEGNINISGYPKKQETFTRISGYCEQNDIHSPHVTIHES
LLYSAWLRLPADVDSKTRKMFIEKVMELVELTPLKDSLVGLPGVNGLSTEQRKRLTIAVELVANPSIIFMDEPT
SGLDARAAAIVMRTVRNTVDTGRTVVCTIHQPSIDIFEAFDELLLLKRGGQEIYVGLLGRHSSCLIKYFEGIEGV
SKIKGGYNPATWMLEVTTSAQEFLLGVDFTEIYKNSNLYRRNKDLIKELSQPAPGSKDLYFPTQYSQSFFTQCM
ACLWKQRRSYWRNPPYTAVRFFFTTFIALIFGTMFWDLGTKRTKQQDLSNAMGSMYAAVLFLGVQNSSSVQP
VVAVERTVFYRERAAGIYSAMPYAFAHALVEIPYVFAQAVVYGVIVYAMIGFEWTAAKFFWYLFFMFFTLLY
FTFYGMMAVAATPNQHIAAIVAAAFYGLWNLFSGFIVPRTRIPVWWRWYYWACPVAWTLYGLVTSQFGDIQ
DRFEDTGDTVEQYLNDYFGFEHDFLGVVAAVIVGFTVLFLFIFAFAIKAFNFQRR

>XP_010654717.1_P_PDR1_Vv
MATAEIYRASGSLRKDSSSIWRNSGAEVFSRTSGDEDDEEALKWAALEKLPTYNRMRKGLLMGSEGEANEVD
IHNLGLQERKNLVERLVKIADEDNEKFLLKLKNRIDRVGIDLPEIEVRFEHLTIDAEAYVGSRALPSFINSAFNQI
EDILNALRILPSRKKKFTILHDVSGIIKPRRMTLLLGPPSSGKTTLLLALSGKLDSSLKVMGSVTYNGHGMNEFV
PQRTAAYISQLDTHIGEMTVRETLAFSARCQGVGDRYDMLAELSRREKSANIKPDPDIDVFMKAVAAEGQKEN
VITDYTLKILGLEVCADTMVGDEMVRGISGGQRKRVTTGEMLVGPSKALFMDEISTGLDSSTTYQIVNSLRQNI
HIFKGTALISLLQPAPETYNLFDDIILLSDSQIVYQGPREDVLDFFESMGFRCPERKGVADFLQEVTSRKDQEQY
WICKDEPYSFVTVKEFAEAFQSFHIGRKLGDELATPFDKTKSHPAAMKTEKYGVRKKELLDACIAREYLLMKR
NSFVYIFKLTQLTIMAVIGMTIFLRTEMHKNTTEDGNIYTGALFFIVITVMFNGMSELAMTIVKLPVFYKQRGLL
FYPAWAYALPSWFLKIPITFVEVGVWVFITYYVIGFDPNVGRLFRQYLLLLLLNQVASSLFRFIAAASRNMIIAN

TFGTFALLLLFALGGFVLSRENIKKWWIWVYWSSPLMYAQNAIVVNEFLGKSWSKNASTTSTESLGVTVLKSR
GFFTEAHWCWIGAGALLGFIFVFNFFYTVALTYLNPFEKPQAVITEESDNAKTGGKIELSSHRKGSIDQTASTKR
GGEIGRSISSTFSYVTEEAIAEANHNKKKGMVLPFQPHSITFDDIRYSVDMPEEMKSQGVLEDKLELLKGVSGA
FRPGVLTALMGVSGAGKTTLMDVLAGRKTGGYIEGNISISGYPKKQETFARICGYCEQNDIHSPHVTIHESLLYS
AWLRLSPDVDAETRMMFIEEVMELVELTPLRDALVGLPGVNGLSTEQRKRLTIAVELVANPSIIFMDEPTSGLD
ARAAAIVMRTVRNTVDTGRTVVCTIHQPSIDIFEAFDELLLLKRGGQEIYVGPLGRHSSHLIKYFEGIEGVSKIKD
GYNPATWMLEVTTSAQELILGVDFTEIYKNSDLYRNNKDLLKELSQPTPGSKDLYFPTQYSQSFFTQCMACLW
KQRWSYWRNPPYTAVRFFFTTFIALMFGTMFWDLGTQRTRQQDLSNAMGSMYAAVIFLGFQNGQSVQPVVV
VERTVFYRERAAGMYSAMPYAFAQALVEIPYVFSQAVVYGAIVYAMIGFEWTTAKFFWYIFFTFFSLLYFTFF
GMMAVAATPNQHIAAIIAAAFYALWNLFSGFIIPRTRIPVWWRWYYWACPVAWTLYGLVTSQYGDIEDRLLD
TNVTVKQYLDDYFGFEHDFLGVVAAVIVGFTVLFLFIFAFSIKAFNFQRR

>XP_010654718.1_P_PDR1_Vv
METAEIYTASGRRASGSFRKNSSSIWRNSGAEVFSRSSRDEDDEEALKWAALEKLPTYNRLRKGLLIGSEGEAS
EVDIHNLGPQERKNLVERLVKIAEEDNEKFLLKLKNRMDRVGIDLPEIEVRFEHLTIDAEAHVGSRALPSFINSV
FNQIEDILNTLRILPSRKKKFTILHDVSGIIKPGRMTLLLGPPSSGKTTLLLALSGKLDSSLKVTGRVTYNGHGMN
EFVPQRTAAYISQLDTHIGEMTVRETLAFSARCQGVGDRYDMLVELSRREKAANIKPDPDIDVFMKAAAAEGQ
KENVITDYTLKILGLEICADTMVGDEMVRGISGGQRKRVTTGEMLVGPSKALFMDEISTGLDSSTTYQIVNSLR
QTVHILNGTALISLLQPAPETYDLFDDIILLSDSRIIYQGPREDVLNFFESMGFRCPERKGVADFLQEVTSRKDQE
QYWAHKDEPYSFVTAKEFAEAFQSFHFGRKLGDELATPFDKTKSHPAALKTEKYGVRKKELLDACISREYLL
MKRNSFVYIFKLTQLTIVAMIAMTIFLRTEMHKNTTEDGNIYTGALFFTVMMVMFNGMSELAMTILKLPVFYK
QRGLLFYPAWAYALPSWFLKIPITFVEVGVWVFITYYVIGFDPNVGRLFRQYLLLLLLNQTASSLFRFIAAACRS
MIVANTFGSFALVLPFALGGIVLSRENVKKWWIWGYWSSPMMYAQNAILVNEFLGKSWSKNASTNSTESLGV
AVLKARGFFTEAHWYWIGAGALLGFIFVFNFCYTVALTYLNPFEKPQAVITVESDNAKTEGKIELSSHRKGSID
QTASTESGEEIGRSISSVSSSVRAEAIAEARRNNKKGMVLPFQPLSITFDDIRYSVDMPEEMKSQGVPEDRLELL
KGVSGAFRPGVLTALMGVSGAGKTTLMDVLAGRKTGGYIEGSISISGYPKKQETFARISGYCEQNDIHSPHVTV
HESLLYSAWLRLPPNVDAETRKMFIEEVMELVELTPLRGALVGLPGVNGLSTEQRKRLTIAVELVANPSIIFMD
EPTSGLDARAAAIVMRTVRNTVDTGRTVVCTIHQPSIDIFDAFDELLLLKRGGQEIYMGPLGRHSSHLIKYFEGI
EGVSKIKDGYNPATWMLEVTASAQELILGVDFTEIYEKSDLYRRNKDLIKELSQPTPGSKDLYFPTQYSQSFFTQ
CMACLWKQRLSYWRNPPYTAVRFFFTTFVALMFGTMFWDLGTKRTRQQDISNAMGSMYAAVLFLGFQNGQ
SVQPVVAVERTVFYRERAAGMYSAMPYAFAQALVEIPYVFSQAVVYGVIVYAMIGFEWTAAKFFWYLFFMFF
SLLYFTFYGMMAVAATPNQHIAAIVASSFYTLWNLFSGFIVPRNRIPVWWRWYYWACPVAWSLYGLVTSQFG
DIEDTLLDSNVTVKQYLDDYFGFKHDFLGVVAVVIVGFTVLFLFIFAFAIKAFNFQRR

>XP_010654719.1_P_PDR1_Vv
METAEIYTASGRRASGSFKKNSSSIWRNSGAEVFSRSSRDEDDEEALKWAALEKLPTYNRLRKGLLIGSEGEAS
EVDIHNLGPQERKNLVERLVKIAEEDNEKFLLKLKNRMDRVGIDLPEIEVRFEHLTIDAEAHVGSRALPSFINSV
FNQIEDILNTLRILPSRKKKFTILHDVSGIIKPGRMTLLLGPPSSGKTTLLLALSGKLDSSLKVTGRVTYNGHGMN
EFVPQRTAAYISQLDTHIGEMTVRETLAFSARCQGVGDRYDMLVELSRREKAANIKPDPDIDVFMKAAAAEGQ
KENVITDYTLKILGLEICADTMVGDEMVRGISGGQRKRVTTGEMLVGPSKALFMDEISTGLDSSTTYQIVNSLR
QTVHILNGTALISLLQPAPETYDLFDDIILLSDSRIIYQGPREDVLNFFESMGFRCPERKGVADFLQEVSANRRSX
QYWAHKDXXXXXXXXXXXXAEAFQSFHFGRKLGDELATPFDKTKSHPAALKTEKYGVGKKELLDACISREYL
LMKRNSFVYIFKLTQLTIVAMIAMTIFLRTEMPKNTTEDGIIYTGALFFTVMKVMFNGMSELAMTILKLPVFYK
QRGLLFYPAWAYALPSWFLKIPITFVEVGLWVFITYYVIGFDPNVGRLFRQYLLLLLLNQTASSLFRFIAAACRS
MIVANTFGSFALVLPFALGGFVLSRESVKKWWIWGYWSSPMMYAQNAIVVNEFLGKSWSKNASTNSTESLGV
AVLKARGFFTEAHWYWIGAGALLGFIFVFNFCYTVALTYLNPFEKPRAVITVESDNAKTEGKIELSSHRKGSID
QTASTESGEEIGRSISSVSSSVRAEAIAEARRNNKKGMVLPFQPLSITFDDIRYSVDMPEEMKSQGVPEDRLELL
KGVSGAFRPGVLTALMGVSGAGKSTLMDVLAGRKTGGYIEGSISISGYPKKQETFARISGYCEQNDIHSPHVTV
HESLLYSAWLRLPPNVDAETRKMFIEEVMDLVELTPLRGALVGLPGVNGLSIEQRKRLTIAVELVANPSIIFMDE
PTSGLDARAAAIVMRTVRNTVDTGRTVVCTIHQPSIDIFDAFDELLLLKRGGQEIYMGPLGRHSSHLIKYFEGIE
GVSKIKDGYNPATWMLEVTASAQELILGVDFTEIYEKSDIYRRNKDLIKELSQPTPGSKDLYFPTQYSQSFFTQC
MACLWKQRLSYWRNPPYTAVRFFFTTFVALMFGTMFWDLGTKRTRQQDISNAMGSMYAAVLFLGFQNGQS
VQPVVAVERTVFYRERAAGMYSAMPYAFAQALVEIPYVFSQAVAYGVIVYAMIGFEWTAAKFFWYLFFMFFT
LLYFTFYGMMAVAATPNQHIAAIVALAFYTLWNLFSGFIVPRNRIPVWWRWYYWACPVAWSLYGLVTSQFG

DIEDTLLDSNVTVKQYLDDYLGFKHDFLGVVAVVIVGFTVLFLFIFAFAIKAFNFQRR

>XP_010654721.1_P_PDR1_Vv
MATADIYRASGSLRRNGSSIWRSSGADIFSRSSRDEDDEEALKWAALEKLPTYNRLRRGLLMGSEGEASEIDIH
NLGFQEKKNLVERLVKVAEEDNEKFLLKLKNRIDRVGIDVPEIEVRFEHLTIDAEAFVGSRALPSFHNFIFSKLE
GILNAVRILPSKKRKFTILNDVSGTIKPRRLTLLLGPPSSGKTTLLLALAGKLDPNLKVMGRVTYNGHGMNEFV
PQRTAAYISQHDTHIGEMTVRETLAFSARCQGVGDRYDMLAELSRREKAANIKPDPDLDVFMKAAATEGQKE
NVVTDYTLKILGLDICADTMVGDEMIRGISGGQRKRVTTGEMLVGPSKALFMDEISTGLDSSTTYQIINSLKQTI
HILNGTAVISLLQPAPETYNLFDDIILLSDSQIVYQGPREDVVEFFESMGFKCPARKGVADFLQEVTSRKDQAQY
WARKDVPYSFVTVKEFAEAFQSFHIGRKVADELASPFDRAKSHPAALTTKKYGVRKKELLDANMSREYLLMK
RNSFVYIFKLTQLAVMAVIAMTLFLRTEMHKNSTDDGNIYTGALFFTVVMIMFNGMAELAMAIAKLPVFYKQ
RDLLFYPAWAYALPTWVLRIPITFVEVGVWVFITYYVIGFDPNVERLFRQYLLLLLVNQMASGLFRFIAAAGRN
MIVANTFGAFALLMLLALGGFILSYDNVKKWWIWGYWSSPLMYAQNAIVVNEFLGKSWSKNVTDSTESLGV
TVLKSRGFFTDAHWYWIGAGALLGFIFVFNIFYTLCLNYLNLFEKPQAVITEESDNAKTATTERGEQMVEAIAE
ANHNKKKGMVLPFQPHSITFDDIRYSVDMPEEMKSQGALEDRLELLKGVSGAFRPGVLTALMGVSGAGKTTL
MDVLAGRKTGGYIEGNITISGYPKKQETFARISGYCEQNDIHSPHVTVHESLLYSAWLRLPSDVNSETRKMFIEE
VMELVELTPLRDALVGLPGVNGLSTEQRKRLTIAVELVANPSIIFMDEPTSGLDARAAAIVMRTVRNTVDTGRT
VVCTIHQPSIDIFEAFDELLLMKRGGQEIYVGPLGRHSSHLINYFEGIEGVSKIKDGYNPATWMLEVTTGAQEGT
LGVDFTEIYKNSDLYRRNKDLIKELSQPAPGTKDLYFATQYSQPFFTQFLACLWKQRWSYWRNPPYTAVRFLF
TTFIALMFGTMFWDLGTERTRQQDLLNAMGSMYAAVLFLGVQNAQSVQPVVVVERTVFYRERAAGMYSALP
YAFGQALVEIPYVFAQAVVYGVIVYAMIGFEWTAAKFFWYLFFMFFTLLYFTFYGMMAVAATPNQHIASIVA
AAFYGLWNLFSGFIVPRNRIPVWWRWYYWICPVAWTLYGLVTSQFGDIQDTLLDKNQTVEQFLDDYFGFKHD
FLGVVAAVVVGFVVLFLFIFAYAIKAFNFQRR

>XP_010654722.1_P_PDR1_Vv
MATADTYRASGSLRRNGSSIWRSSGADVFSRSSRDEDDEEALKWAALEKLPTYNRLRRGLLMGSEGEASEIDI
HNLGFQEKKNLVERLVKVAEEDNEKFLLKLKNRIDRVGIDVPEIEVRFEHLTIDAEAFVGSRALPSFHNFIFNKL
EGILNAVRILPSKKRKFTILNDVSGIIKPRRLTLLLGPPSSGKTTLLLALAGKLDPNLKVMGRVTYNGHGMNEFV
PQRTAAYISQHDTHIGEMTVRETLAFSARCQGVGDRYDMLAELSRREKAANIKPDPDLDVFMKAAATEGQKE
NVVTDYTLKILGLDICADTMVGDEMIRGISGGQRKRVTTGEMLVGPSKALFMDEISTGLDSSTTFQIINSLKQTI
HILNGTAVISLLQPAPETYNLFDDIILLSDSQIVYQGPREDVLEFFESIGFKCPERKGEADFLQEVTSRKDQAQYW
ARKDVPYSFVTVKEFAEAFQSFHIGRKVADELASPFDRAKSHPAALTTKKYGVRKKELLDANMSREYLLMKR
NSFVYIFKLTQLAVVAVIAMTLFLRTEMNKNSTEDGSIYTGALFFTVVMIMFNGMAELAMTIAKLPVFYKQRD
FLFYPAWAYALPTWVLKIPITFVEVAVWVFITYYVIGFDPNVERLFRQYLLLLLVNQMASGLFRFIAAAGRNMI
VASTFGAFAVLMLMALGGFILSHDNVKKWWIWGYWSSPLMYAQNAIVVNEFLGKSWSKNVTNSTESLGITV
LKSRGFFTDAHWYWIGAGALLGFIFVFNFFYTLCLNYLNPFEKPQAVITEESDNAKTATTERGEHMVEAIAEGN
HNKKKGMVLPFQPHSITFDDIRYSVDMPEEMKSQGALEDRLELLKGVSGAFRPGVLTALMGVSGAGKTTLMD
VLAGRKTGGYIEGNISISGYPKKQETFARISGYCEQNDIHSPHVTVHESLLYSAWLRLPSDVNSETRKMFIEEVM
ELVELTPLRDALVGLPGVNGLSTEQRKRLTIAVELVANPSIIFMDEPTSGLDARAAAIVMRTVRNTVDTGRTVV
CTIHQPSIDIFEAFDELLLMKRGGQEIYVGPLGRHSSHLINYFEGIEGVSKIKDGYNPATWMLEVTTGAQEGTLG
VDFTEIYKNSDLYRRNKDLIKELSQPAPGTKDLYFATQYSQPFFTQFLACLWKQRWSYWRNPPYTAVRFLFTT
FIALMFGLIFWDLGTRRTRQQDLLNAMGSMYAAVLFLGVQNAQSVQPVIVVERTVFYRERAAGMYSALPYAF
GQALVEIPYVFAQAVVYGVIVYGMIGFEWTATKFFWYLFFMFCTLLYFTFYGMMAVAATPNQHIASIIAATFY
TLWNLFSGFIVPRNRIPVWWRWYCWICPVAWTLYGLVASQFGDIQSTLLENNQTVKQFLDDYFGFKHDFLGV
VAAVVVGFVVLFLFIFAYAIKAFNFQKR

>XP_013454711.1_PDR1_X1_Mt
MDGSDLYKASSSLRGNTSTFFSRSSRREEDDEEALKWAAIEKLPTYNRLKKGLLASSHGVANEIIDIDKLGVQE
RQKLLNRLIKAAEEDNEKFLLKLKERIDRVGIEIPTIEVRFEHLTIEAEAYVGSRALPSFTNFTIGAVEGLLAFLGII
SHKKKHMTILKDVSGIVKPGRMALLLGPPSSGKTTLLLALTGKLDKALKESGRVTYNGYGMDEFVPQRTAAYI
SQHDVHIGEMTVRETLAFSARCQGVGSRYDLLSELCRREKEAKIIPDPDIDVYMKATSTEGQEESLITDYMLKIL
GLDICADTMVGDEMHRGISGGQRKRLTTGEMLVGPSKVLFMDEISTGLDSSTAFQIVKSLRQYVYILHGTALIS
LLQPAPETYELFDDIILLSDGEIVYQGPRENVLEFFESIGFKCPERKGVADFLQEVTSRKDQEQYWMHRDEPYRF
VTVTQFAEAFKSFHVGKTIKEELEIPFDKSNNHPAALTTKKYGVNKKELLKANISREFLLMKRNSFIYLFKMVQ

VSIMATITMTLFLRTHMHKETVIDGQIHFGALYFSLIMLMFNGTIELTMTIVKLPTFFKQRDHLFYPSWAYAIPS
WIVRAPVTLVDASIWVFLTYYVIGFDPNIWRFLKMYLLLILLNQTASGLFRAIAAFCRDMIIANITGFYTLLIVFT
LGGFVLAKDDIKGWWIWGYWISPLMYAQNAIMVNEFLGNSWNKITPYSNMTLGILSLKSRGFFTHAYWYWIG
VGALIGFIFLTNFLYIIALTYLDPLDKPQATIKEESGGDNAPNDRNQEIELPQLENSRRSTAVADSSRGKERGMV
LPFEPYSITFDEIVYSVDVPQEMKDQGVIEDRLVLLKGVSGAFRPGVLTALMGVSGAGKTTLMDVLAGRKTSG
YIDGSIKISGYPKKQETFARISGYCEQNDIHSPQVTVYESLLYSAWLRLPAEVDSNTRKMFIEEVMELVELNPLR
NSLVGLPGVSGLSTEQRKRLTISVELVANPSIIFMDEPTSGLDARAAAIVMRTVRNTVDTGRTVVCTIHQPSIDIF
ESFDELFLLKSGGREIYAGPLGRYSNQLIKYFESIEGVSKINDGYNPATWMLEVTSPAQEVALGADFHEIYKNSE
LYRRNKQLIEELGKPALGSKDLYFPSQYSQSFLVQCLACLWKQRWSYWRNPLYTAVRFYFATFIALMFGTMF
WDLGRKYTRGLDLSNAMGSMYTAVFFIGVQNSASVQPVVAVERTVFYRERAAGMYSALPYALAQVLIELPYI
LAQTLSYGVIVYAMIGFEWTVPKFFWYMFFMYFTFCYFTFYGMMTVAVTPNYHLAAVLASAFYGSWNLFSG
FVVPRPRMPVWWRWYYWANPVAWSLYGLFASQFGNITDIMEMEDVTVQEYIRNYYGIKHDFVGVSAAVVF
GIAIAFAFTFAVSIKVFNFQHR

>XP_013454712.1_PDR1_Mt
MEGSDIYSASNSLRFSMRSSSTTGWRNGTMEAFAKSSRREEDDDEEALKWAALEKLPTYNRLRKGLLATSRG
VANEVDITDLGFQEKQKLLDRLINVAEEGNEKFLLKLKERIDRVGIEIPAIEVRYEHLNVEAEAFVGGRALPTLL
NSVTNTVESILISLHILTSRKKQMTILKDVSGIVKPRRMTLLLGPPSSGKTTLLLALSGKLDPNLKVSGRVTYNG
HGMDEFVPQRTAAYISQHDVHIGEMTVRETLAFSARCQGVGSRYDLLAELSRREKEANIKPDPDVDVFMKAM
ATGGQQESVATDYVLKLLGLDVCADTMVGNEMLRGISGGQRKRVTTGEMLVGPANALFMDEISTGLDSSTTF
QIVRSLQQYVHILNGTTVISLLQPAPETYELFDDIILISDGQIVYQGPREHILEFFESVGFKCPERKGAADFLQEVT
SKKDQEQYWVDREKPYRFVTVTQFAEAFQSYHVGRKTGDELAIPFDKSKNHPAALTTKKYGVNKKELLKANF
SREYLLMKRNSFVYIFKICQLLLMATIAMTLFLRTEMHRDSLNGGGVYSGALFFAVVMIMFNGMAELSMTIAK
LPSFYKQRDLLFFPSWAYAIPTWILKIPITFLEVAAWVFLTYYVIGFDPNVTRLLKQYLLLLLINQMASGLFRAIA
ALGRNMIVANTFGSFALLALLTLGGFVMSRKDIKSWWIWGYWISPLMYGQNAIMVNEFLGDSWNHFTPNSNK
TLGIQVLESRGFFTEAYWYWIGIGALTGFMFLFNILFTVALTYLDPFDKPQATINEESEDSTTNGTTQEVELPRIA
SSGGSNGADPSQRERRGMVLPFEPHSIAFDDVVYSVDMPQEMKVQGVLEDRLVLLKGVSGAFRPGVLTALMG
VSGAGKTTLMDVLAGRKTGGYIDGSIKISGYPKKQETFARISGYCEQNDIHSPHVTVYESLVYSAWLRLPADV
DSNTRKMFIEEVMELVELNPLRNSLVGLPGVNGLSTEQRKRLTIAVELVANPSIIFMDEPTSGLDARAAAIVMR
TVRNTVDTGRTVVCTIHQPSIDIFEAFDELFLMKRGGEEIYVGPLGRHSSQLIKYFESIEGVSKIKDGYNPATWM
LEVSSSAQELTLGIDFHHAYKNSELYRRNKQLIEELGKPAPGSNDLYFSAQYSQSFLVQCLACLWKQHWSYWR
NPPYTSVRFFFTVFIGLMFGTIFWDLGRKYSKRQDLFNALGSMYTAVLFLGVQNSSAVQPVVAVERSVFYRER
AAGMYSALPYAFAQVLIELPYIFVQAASYGVIVYAMIGFEWTVAKFLWYIFFMYCTLCYFTFYGMMAVAITPN
HHVASIVAAAFYAIWNLFSGFIVPRPMIPVWWRWYYWGCPVSWTLYGLIASQFGDITKIMESENESVQEFIRSY
FGMKHDFIGVCAVVVVGTAVLFACIFAVSIKVFNFQRR

>XP_013454713.1_PDR1_Mt
MEGSDIYKAGNSFRMSSSSTTVWRNSKMEAFSMSSRHGGEDEEALRWAALEKLPTYNRLRKGLLATSRGVAN
EIDILSDLGFQERQKLLDRLINVAEEGNEKFLLKLKERIDRVGIEIPTIEVRYEHLIVDAEAYVGGRALPTLLNSV
MNAVESILTYLHIFTSKKKHMTILKDVSGIVKPRRMTLLLGPPSSGKTTLLLALSGKLDPNLKVSGRVTYNGHG
MDEFVPQRTAAYISQHDVHIGEMTVRETLAFSARCQGVGSRYDLLSELSRREKEAKIKPDPDIDVFMKAVATG
GQQESVVTDYVLKLLGLDVCADTMVGNEMLRGISGGQRKRVTTGEMLVGPANALFMDEISTGLDSSTTFQIV
KSLRQYVHILNGTAVISLLQPAPETYELFDDIILISDGQIVYQGPREHVLDFFESVGFKCPERKGVADFLQEVTSK
KDQEQYWVDREKPYRFLTVTQFAEAFQSYHVGRKTRDELAIPFDKSKNHPAALTTKKYGVNKKELLKANFSR
EYLLMKRNSFVYIFKICQLTLMATVTMTLFLRTEMHRDSLNDGGVYAGAIFFSVVMLMFNGLAELSMTIAKLP
SFYKQRDLLFFPSWAYAIPTWILKIPITFLEVAVWVFLTYYVIGFDPNVTRLLKQYLLLLLINQMASGLFRAIAA
LGRNMIVANTFGSFALLALLTLGGFIMSRRDIKSWWIWGYWISPLMYGQNAIMVNEFLGDSWNHFTPNSNKTL
GIQVLESRGFFTEAYWYWIGIGALTGFMFLFNILFTMALTHLNPFDKPQAKINEESEDSITNGTLQEVELPRIASL
GEYVVSSSNRKKRGMILPFEPHSIIFDQVVYSVDMPQEMKVQGVVEDRLVLLKGVSGAFRPGVLTALMGVSG
AGKTTLMDVLAGRKTGGYIDGTIKISGYLKRQETFARISGYCEQNDIHSPHVTVYESMVYSAWLRLPAEVDSN
SRKMFIEEVMELVELNPLRNSLVGLPGVNGLSTEQRKRLTIAVELVANPSIIFMDEPTSGLDARAAAIVMRTVR
NTVDTGRTVVCTIHQPSIDIFEAFDELLLMKRGGQETYVGPLGRHSNQLIKYFESIEGVSKIKDGYNPATWMLE
VTSSAQEHTLGVDFHDIYKNSELYRRNKQLIVELGKPAPGSKDLHFSAQYSQSFWIQCLACLWKQHWSYWRN
PPYTAVRFFFTTFIALMFGTMFWNLGRKYSNRQDLFNALGSMYTAVLFLGVQNSSSVQPVVAVERSVFYRERA

AGMYSALPYAFAQVIIELPYIFVQATSYGVIVYAMIGFEWTLEKFFWYIFFMYFTLCYFTFYGMMAVAVTPNH
HVASIVASAFYAIWNLFSGFIIPRPRIPVWWRWYYWACPVAWTLYGLVASQFGDINNIMESENKSVQEFIRSYF
DFKHDFIGVCAVVVVGTAVLFACIFAVSIKLFNFQRR

>XP_014629326.1_PDR1_Gm
MEGGSSFRIGSSSIWRVDSDTNIFSNSFHQEDDEEALKWAAIQKLPTVARLRKALLTSSEGEISEIDVKKLGLQE
RRALLERLVRTVEDDNEKFLLKLRNRIDRVGIHLPTVEVRFENLNVEAEVHVGTRASPTFFNFMFNIVEGLLNF
LHILPSRKQHITIIRDVSGIIKPGRMTLLLGPPSSGKTTLLLALAAKLDPKLKFSGKVTYNGHEMNEFVPQRTAA
YVNQNDHHVAELTVRETLAFSARVQGVGTHYDLLAELSRREKEANIRPDPDIDVYMKAVATEGQKANLITDY
VLRILGLETCADTIIGNEMLRGISGGQKKRLTTGEMLVGPTKALFMDEISTGLDSSTTFQIVNSVKQCVHILKGT
AVISLLQPTPETYNLFDDIILLSDSHIVYQGPREHVLEFFKSMGFKCPERKGVADFLQEVTSRKDQEQYWADKD
QPYRFVTSKEFSEAHRSFHVGRSLVEELATEFDKSKSHPAALTTKKYGVGKWELFKACLSREYLLIKRHSFVYT
FKLSQLSVAAFVAMTVFLQTEMHRDSVIDGGIYVGALFYGLVVIMFNGMPELSMAVSRLPVFYKERDNLFFPS
WAYALPAWLLKILMSFVEVGVWVFLTYYVIGFDPYVGRFFRQYLVLVLVKQMTSALYRFVAALGRESTVALT
LGSGTNATLLAMSGFVLSKDNIKKWWLWGFWMSPTMYGQNAMVNNEFLGKRWRHILPNSTEPLGIEVLRSR
GFFTQSYWYWIGVGALIGYTLLFNFGYILALMYLSPPGKHRAVLSEEPQSNEQNGGSKKGTNVLRHIKYSLSQ
HSNKGRKGKRVSGSTSSHTLPASGMVLPFQPHSITFDEVTYAVDMPQEMRDQGVVKDKLVLLKGVSGAFRPG
VLTALMGVTGAGKTTLMDVLAGRKTGGYVGGNIKISGYRKKQETFARISGYCEQNDIHSPHVTVYESLLYSS
WLRLSLDINVETRKMFIEEVMELVELKPLRHVLVGFPGVTGLSTEQRKRLTIAVELVANPSIIFMDEPTSGLDAR
AAAIVMRIVRNTVDTGRTVVCTIHQPSMDIFESFDELFLMKQGGQEIYVGPLGHHSSHLISYFEGIQGVSEIKAG
YNPATWVLEVTNSSKEMELGIDFAEVFKNSELCRRNKELVKELSTPAPGSKDLYFPSQYSTSFFMQCMACLWK
QHRSYWRNTRYTALSFIYSTTLAVLLGSMFWNLGSKIEKQQDLLNALGSMYVAVLLIGIKNAYSVQPVVAAER
IVFYRERAAGMYSALPYAFAQVLIEIPYVLVQAVVYSLIVYAMIGFEWTVAKFFWFLFFMYFNFLCFTYYGMM
SMAVTPNQHISSIVSTGFYSAWNIFSGFIIPRPRIPVWWRWYSWANPIAWSMYGLVASQYGDIKENIESTDGTTT
VEDFVRSYFGYKHDFLGVVATVIAAFAVVFALVFAISMKMFNFQRR

>XP_014630001.1_PDR1_Gm
MESGELRVASARIGSSSVWRSSGGVDVFSGSSRRDDDEQELKWAAIEKLPTYLRMTRGILTEAEGQPTEIDINK
LCPLQRKNLVERLVKIAEQDNEKFLFKLRDRIDSVGLEIPAIEVRFEHLNVEAEAHVGSRALPTIFNFCINLLEGF
LNSLHLIPSRKKPFTVLDDVSGIIKPKRMSLLLGPPSSGKTTLLLALAGRLGKDLKFSGRVSYNGHGMEEFVPQR
TSAYISQTDLHIGEMTVRETLAFSARCQGIGTRNEMLAELSRREKAANIKPDPDLDIYMKAAALEGQETNVVTD
YIMKILGLEICADTMVGDDMIRGISGGQKKRVTTGEMLVGPARALLMDEISTGLDSSTTFQMVNSLRQSIHILN
GTAVISLLQPAPETYELFDDIILLSDGQIVYQGPRENVLEFFEYMGFKCPERKGVADFLQEVTSRKDQEQYWAN
KDEPYSFVTVKEFAEAFQSFHVGRKLGDELATPFDMSKGHPAVLTKNKYGVCKKELLKACVSREFLLMKRNS
FVYIFKMWQLILTGFITMTLFLRTEMHRDTETDGGIYMGALFFVLIVIMFNGYSELSMSIMKLPVFYKQRDLLFF
PCWAYSLPTWILKIPITLVEVGIWVVMTYYVIGFDPSIERFIKQYFLLVCINQMASGLFRFMGAVGRNIIVANTV
GSFALLAVMVMGGFILSRVDVKKWWLWGYWFSPMMYGQNALAVNEFLGKSWSHVPPNSTEPLGVKVLKSR
GIFPEAYWYWIGVGASIGYMLLFNFLFPLALHYLDPFGKPQALISEEALAERNAGRNEHIIELSSRIKGSSDRGNE
SRRNMSSRTLSARVGSIGASEHNKKRGMVLPFTPLSITFDEIRYSVEMPQEMKSQGILEDRLELLKGVNGVFRP
GVLTALMGVSGAGKTTLMDVLSGRKTAGYVQGQITISGYPKKQETFARIAGYCEQTDIHSPHVTVYESLVYSA
WLRLPPEVDSVTRQMFIEEVMELVELTSLREALVGLPGVNGLSTEQRKRLTIAVELVANPSIIFMDEPTSGLDAR
AAAIVMRTVRNTVDTGRTVVCTIHQPSIDIFDAFDELLLLKRGGEEIYVGPLGQCCSQLINYFEGINGVPKIKKG
YNPATWMLEVTSEAQEAALGLNFAEIYKNSDLYRRNKALIRELSTPTTGFKDLYFPTKYSQTFITQCMACLWK
QHLSYWRNPPYSAVRLLFTTIIALLFGTIFWDIGSKRQRKQDLFNAMGSMYAAVLFIGIQNATSVQPVVAIERT
VFYRERAAGMYSALPYAFGQVAIEIPYIFIQTLVYGVIVYAMIGFDWTFSKFFWYLFFMFFTFLYFTFYGMMAV
GLTPDHNVATIVSFGFYMIWNLFSGFVIPRTRMPVWWRWYFWICPVSWTLYGLVTSQFGDIKERIDTGETVEE
FVRSYFGYRDDFVGVAAAVLVGFTLLFGFTFAFSIKAFNFQKR

>XP_015626248.1_ABCG39_Os
MDIVRMGSVASGGGSVRRTASSWRGTSGRSDAFGRSVREEDDEEALKWAAIEKLPTYDRMRKGILTAGGVEE
VDIGGLGLQERRNLIERLVRTAEEDNERFLLKLRDRMERVGIDNPTIEVRFENLSIDAEAYVGNRGIPTFTNFFS
NKIMDVLSAMRIVSSGKRPISILHDISGIIRPGRMSLLLGPPGSGKTSLLLALAGKLDSTLKVSGRVTYNGHDMD
EFVPQRTSAYIGQHDLHIGEMTVRETLAFSARCQGVGTRYDMLTELSRREKEASIKPDPDIDVYMKAISVEGQE
SVVTDYILKILGLEICADTMVGDAMIRGISGGQKKRVTTGEMLVGPAKALFMDEISTGLDSSTTYQIVNSLRQS

VHILGGTALIALLQPAPETYDLFDDIVLLSEGQIVYQGPRENILEFFEAMGFKCPERKGVADFLQEVTSRKDQHQ
YWCRRDEPYRYISVNDFSEAFKEFHVGRNLGSELRVPFDRTRNHPAALTTSRYGISKMELTKACFSREWLLMK
RNSFVYIFKILQLIILGSIGMTVFLRTKMHRRSVEDGAIFLGAMFLGLVTHLFNGFAELAMSIAKLPIFYKQRDLL
FYPSWAYALPTWVLKIPISFLECAVWICMTYYVMGFDPNIERFFRHYVLLVLISQMASGLFRLLAALGREMVV
ADTFGSFAQLILLVLGGFLISRENIKKWWIWGYWSSPLMYAQNAIAVNEFLGHSWNKVVDPTQSNDTLGVQV
LKVRGIFVDANWYWIGVGALLGYIMLFNILFILFLEWLDPLGKGQAVVSEEELREKHVNRTGENVELLTLGTD
SQNSPSDGRGEITGADTRKRGMVLPFTPLSITFDNIRYSVDMPQEMKDKGVTEDRLLLLKGVSGAFRPGVLTAL
MGVSGAGKTTLMDVLAGRKTGGYIEGDISISGYPKKQETFARIAGYCEQNDIHSPHVTVYESLLYSAWLRLPSE
VDSEARKMFVEEVMELVELTSLRGALVGLPGVNGLSTEQRKRLTIAVELVANPSIIFMDEPTSGLDARAAAIVM
RTVRNTVDTGRTVVCTIHQPSIDIFEAFDELFLMKRGGEEIYVGPLGHNSCHLINYFEGIQGVRKIKDGYNPATW
MLEVTTLAQEDILGINFAEVYRNSDLYQRNKTLISELSTPPPGSTDLHFPTQFSQPFFTQCMACLWKQHKSYWR
NPSYTATRIFFTTVIALIFGTIFLNLGKKINKRLDLFNSLGSMYAAVLFIGIQNGQTVQPIVDVERTVFYREKAAG
MYSALPYAFAQVLIEIPHIFLQTVVYGLIVYSLIGFDWTVEKFFWYMFFMFFTFMYFTFYGMMAVAMTPNSDI
AAIVSTAFYCIWNIFAGFLIPRPRIPIWWRWYSWACPVAWTLYGLVASQYGDITNSTLEDGEVVQDYIRRYFGF
RHDYLGYVATAVVGFAALFAFVFAFSIKVFNFQRR

>XP_015648322.1_ABCG36_X1_Os
MDAAGEIQKVASMRLGGSMRGDSGSMWRRGDDVFSRSSREEDDEEALRWAALEKLPTYDRVRRAILPLGGD
DGAGDGGGKGVVDVHGLGPRERRALLERLVRVADEDNEKFLLKLKDRVDRVGIDMPTIEVRFEHLEAEAEVR
VGNSGLPTVLNSITNTLEEAGNALGILPNRKQTMPVLHDVSGIIKPRRMTLLLGPPGSGKTTLLLALAGRLGKD
LKASGKVTYNGHGMEEFVPERTAAYISQHDLHIGEMTVRETLAFSARCQGVGSRFDMLTELSRREKAANIKPD
ADIDAFMKAAAMGGQEANVNTDYILKILGLEICADTMVGDEMLRGISGGQRKRVTTGEMLVGPARALFMDEI
STGLDSSTTFQIVNSLRQTVHILGGTAVISLLQPAPETYNLFDDIILLSDGQIVYQGPREDVLEFFESMGFKCPDR
KGVADFLQEVTSKKDQRQYWARHDKPYRFVTVKEFVSAFQSFHTGRAIANELAVPFDKSKSHPAALATTRYG
APGKELLKANIDREILLMKRNSFVYMFRTFQLMVVSLIAMTLFFRTKMKRDSVTSGGIYMGALFFGVLMIMFN
GFSELALTVFKLPVFFKQRDLLFYPAWSYTIPSWILKIPITFIEVGGYVFLTYYVIGFDSNVGSFFKQYLLMLAIN
QMAGSLFRFIGGAARNMIVANVFASFMLLIFMVLGGFILAREQVKKWWIWGYWISPMMYAQNAISVNELMG
HSWNKIVNSSASNETLGVQVLKSRGVFPEARWYWIGFGAMIGFTILFNALFTLALTYLRPYGNSRQSVSEEELK
EKRANLNGEIVGDVHLSSGSTRRPMGNGTENDSTIVDDDTEVTQRGMVLPFTPLSLSFDNVRYSVDMPQEMK
AQGVADDRLELLKGVSGSFRPGVLTALMGVSGAGKTTLMDVLAGRKTGGYIEGSINISGYPKKQETFARVSG
YCEQNDIHSPQVTVYESLLFSAWLRLPEDVDSNTRKMFIEEVMELVELKSLRDALVGLPGVNGLSTEQRKRLTI
AVELVANPSIIFMDEPTSGLDARAAAIVMRTVRNTVNTGRTVVCTIHQPSIDIFEAFDELFLMKRGGEEIYAGPL
GHHSSELIKYFESIPGVSKIKDGYNPATWMLEVTTIGQEQALGVDFSDIYKKSELYQRNKALIKDLSQPAPDSSD
LYFPTQYSQSSLTQCMACLWKQNLSYWRNPPYNAVRFFFTTVIALLFGTIFWDLGGKVTKSQDLFNAMGSMY
AAVLFIGVMNCTSVQPVVAVERTVFYRERAAGMYSAFPYAFGQVVIEIPYTLVQATVYGIIVYAMIGFEWTAA
KFFWYLFFMVFTLLYFTFYGMMAVGLTPNYHIASIVSSAFYAIWNLFSGFVIPRPRVPIWWRWYCWACPVAW
TLYGLVVSQFGDIETPMEDGTPVKVFVENYFGFKHSWLGWVATVVAAFAFLFASLFGFAIMKFNFQKR

>XP_015648329.1_ABCG37_X1_Os
MDREVHRMASLRREGSMWRSGGDVFSRSSSRFQDEDDDEEALRWAALERLPTYDRVRRGILAVSSEDGGAG
GEKVEVDVGRLGARESRALIERLVRAADDDHERFLLKLRERMDRVGIDYPTIEVRFENLEVEADVHVGNRGLP
TLLNSVTNTVEAIGNALHILPNKKQPMTVLHDVSGIIKPRRMTLLLGPPGSGKTTLLLALAGKLDKDLKVSGKV
TYNGHGMHEFVPERTAAYISQHDLHIGEMTVRETLAFSARCQGVGTRYEMLTELARREKAANIKPDHDIDIYM
KASAMGGQESSVVTDYILKILGLDICADTVVGNEMLRGISGGQRKRVTTGEMLVGPARALFMDEISTGLDSST
TYQIVNSLRQTIHILGGTAVISLLQPAPETYNLFDDIILLSDGQVVYQGPREHVLEFFEFMGFRCPARKGVADFL
QEVTSRKDQGQYWCRRDRPYRFVPVKQFADAFRSFHVGRSIQNELSEPFDRTRSHPAALATSKYGVSRKELLK
ATIDRELLLMKRNAFMYIFKAVNLTLMALIVMTTFFRTSMRHDRDYGMIYLGALYFALDTVMFNGFAELAMT
VMKLPVFFKQRDLLFFPAWAYTIPSWILQIPITFLEVGVYVFITYYVIGFDPSVSRFFKQYLLLLALNQMSSALFR
FIAGIGRDMVVSHTFGPLSLLAFAALGGFILARPDVKKWWIWGYWISPLSYAQNAISTNEFLGHSWSQILPGEN
VTLGVSVLKSRGIFTEAKWYWIGLGALLGYTLLFNLLYTVALSVLSPFTDSHASMSEDALKEKHANLTGEVVE
GQKDTKSRKQELELSHIADQNSGINSADSSASRKGMVLPFAPLSISFNDVRYSVDMPEAMKAQGITEDRLLLLK
GVSGSFRPGVLTALMGVSGAGKTTLMDVLAGRKTGGYIEGDIRISGYPKKQETFARISGYCEQNDIHSPHVTVY
ESLVFSAWLRLPSEVDSEARKMFIEEVMDLVELTSLRGALVGLPGVSGLSTEQRKRLTIAVELVANPSIIFMDEP
TSGLDARAAAIVMRTVRNTVNTGRTVVCTIHQPSIDIFEAFDELFLMKRGGEEIYVGPVGQNSSKLIEYFEGIDG

VSRIKDGYNPATWMLEVTSSAQEEMLGVDFSEIYRQSELYQRNKELIEELSTPPPGSTDLNFPTQYSRSFITQCL
ACLWKQNWSYWRNPSYTAVRLLFTIVIALMFGTMFWNLGTRTKKQQDLFNAMGSMYAAVLYIGVQNSGSV
QPVVVVERTVFYRERAAGMYSAFPYAFGQVAIELPYIMVQTLIYGVLVYSMIGFEWTVAKFLWYLFFMYFTLL
YFTFYGMMAVGLTPNESIAAIISSAFYNVWNLFSGYLIPRPKIPVWWRWYCWICPVAWTLYGLVASQFGDIQH
VLEGDTRTVAQFVTDYFGFHHNFLWVVAVVHVVFAVTFAFLFSFAIMKFNFQRR

>XP_015650488.1_ABCG44_Os
MDTGEAAFGVASLRLRGSMASASSRRAPSYRDYDVFSIASSSRAEAEDDEEALKWAALEKLPTHARVRKGIVA
AADDGQGSGAAGEVVDVAGLGFQERKHLLERLVRVAEEDHESFLLKLKQRIDRVGLDFPTIEVRYEHLSIDAL
AHVGSRGLPTFLNTTLNSLESLANLLHVVPNKKRPLNILHDVHGVIKPRRMTLLLGPPGSGKTTLLLALAGKLG
SDLKVSGKVTYNGYGMDEFVAQRSAAYISQHDLHIPEMTVRETLAFSARCQGVGTRYDMLTELARREKAANI
KPDPDLDVYMKAISVGGQETNIITDYVLKILGLDICADTIVGNEMLRGISGGQRKRVTTGEMIVGPARAMFMD
EISTGLDSSTTFQIVKSLGQITSILGGTTVISLLQPAPETYNLFDDIILLSDGHIVYQGPREHVLEFFESMGFKCPDR
KGVADFLQEVTSRKDQQQYWARTHQPYRYIPVQEFACAFQSFHVGQTLSDELSHPFDKSTSHPASLTTSTYGA
SKLELLRTCIARELLLMKRNMFVYRFRAFQLLVITIIVMTLFLRTNMHHETRTDGIVYLGALFFAMVAHMFNGF
SELAMATIKLPVFFKQRDYLFFPSWAYTIPTWILKIPISCFEVAITVFLSYYVIGFDPNVGRLFKQYLLLLLVNQM
AAALFRFIAALGRTMVVANTLASFALLVLLVLSGFILSHHDVKKWWIWGYWISPLQYAMNAIAVNEFLGHKW
NRLVQGTNTTLGIEVLKSRGMFTEAKWYWIGVGALFGYVIVFNILFTIALGYLKPSGKAQQILSEEALKEKHAN
ITGETINDPRNSASSGQTTNTRRNAAPGEASENRRGMVLPFAPLAVAFNNIRYSVDMPPEMKAQGVDQDRLLL
LKGVSGSFRPGVLTALMGVSGAGKTTLMDVLAGRKTGGYIEGDISISGYPKKQETFARVSGYCEQNDIHSPNV
TVYESLAYSAWLRLPSDVDSETRKMFIEQVMELVELNPLRDALVGLPGVNGLSTEQRKRLTIAVELVANPSIIF
MDEPTSGLDARAAAIVMRTVRNTVDTGRTVVCTIHQPSIDIFEAFDELFLMKRGGEEIYVGPLGHHSCDLIEYFE
GVEGVSKIKPGYNPATWMLEVTTLAQEDVLGISFTDVYKNSDLYQRNQSLIKGISRPPQGSKDLFFPTQFSQSFS
TQCMACLWKQNLSYWRNPPYTVVRFFFSLIVALMFGTIFWRLGSKRSRQQDLFNAMGSMYAAVLFMGISYSS
SVQPVVAVERTVFYRERAAGMYSALPYAFGQVVVELPYVLVQSAVYGVIVYAMIGFEWEAKKFFWYLYFMY
FTLLYFTFYGMLAVGLTPSYNIASIVSSFFYGIWNLFSGFVIPRPSMPVWWRWYSWACPVSWTLYGLVASQFG
DLKEPLRDTGVPIDVFLREYFGFKHDFLGVVAVAVAGFATLFAVSFSLSIKMLNFQRR

>XP_019069398.1_PDR1_-l_Sl
MMEPANLNNFRGSMRGSLRADSSNSIFSRSARDEDDEEALKWAALEKLPTFDRMRKGLLFGKEGEAAAEVDT
NDIGHQERKNLLDRLVKVADEDNEKFLLKLKNRIETVGIDLPSIEVRYEHVNIDADAYVGSRALPTFINFMTNF
VESFLNSIHILPSRKRQITILKHVSGMIKPSRMTLLLGPPSSGKTTLLLALAGKLDSTLKVTGNVTYNGHELHEFV
PQKTAVYISQYDLHIGEMTVRETLEFSARCQGVGPRYEMLAELSRREKAANIKPDHDVDIYMKASVTKGQEA
NVVTDYVLKILGLDVCADTMVGDEMLRGISGGQKKRVTTGEMLVGPSKALFMDEISTGLDSSTTFSIVNSLRQ
SVQLLNGTAVISLLQPAPETYNLFDDIILLSDGRIVYQGPREAVLDFFESMGFKCPERKGVADFLQEVTSKKDQ
QQYWAKRDEAYRFITSKEFAEAYESFHVGKKLADELATPYDKTKSHPAALSTQKYGLGTKEMLKVCAEREFL
LMKRNSFVYIFKLFQLVVMALIMMTVFFRTEMPRDNMDDGGMYAGALFFVVVVIMFNGMAEINLTILKLPVY
FKQRDLLFYPSWAYALPTWILKIPITFIEVGLWTFLTYYVMGFDPNVSRLFKQFLLLVLVHQMASGLFRFIGAA
GRTMGVATTFGAFALVLQFALSGFVLSRNDVKKWWIWGYWISPLMYSVNSILVNEFDGKKWDHIAPNGAEPL
GHAVVRSRGFFPDAYWYWVGVVALIGFIIIFNLCYSVGLAYLNPFGKQVMISEDDENDRLIEGSETEGEKKKG
MVLPFEPHSITFDNVVYSVDMPQEIKDQGSTEDRLVLLKGVSGAFRPGVLTALMGVSGAGKTTLMDVLAGRK
TGGYIDGDIKISGYPKKQETFARISGYCEQNDIHSPYITVYESLVYSAWLRLPQDVDKNKRKMFVEEVMELVEL
TPLRSALVGLPGVNGLSTEQRKRLTIAVELVANPSIIFMDEPTSGLDARAAAIVMRAVRNTVDTGRTVVCTIHQ
PSIDIFEAFDELFLMKRGGQEIYVGPLGRYSCHLIKYFESLPGVSKIKEAYNPATWMLEVTAASQEMMLGVDFT
DLYKKSDLYKRNKALIAELSTPRPGTKDLHFETQFSQSFWTQCMACLWKQHLSYWRNPSYTAVRFIFTVILAL
VFGTLFWDLGSRLSRSQDLFNAMGSMYAATLFLGVQNSSSAQPVVAVERTVFYRERAAGMYSALPYAFGQVI
VEIPYVFLQAVFYGIIVYAMIGFEWTVAKFFWYLFIMYFTLLYFTFYGMLTVAVSPNQNVASIIAAFFYALWNL
FSGFIVPRPRIPIWWRWYYWLCPVAWTLYGLVASQFGDLQTMLSDDENVEQFLGRYFGFEHDFLGVVAAIIAA
WPVVFAFLFAFAIKAFNFQKR

>XP_020405525.1_X1_Zm
MDAAGDIQKVASMRRGDSGSIWRRGDDVFSRSSREEDDEEALRWAALEKLPTYDRVRRAMVPLGLGADGAE
AAGRKGLVDVDVLSLGPRERRALLERLVRVADEDNERFLLKLKDRVDRVGIDMPTIEVRFQNLEAEAEVRVG
SSGLPTVLNSVVNTIEEAANALHILPSRKRIMPILHDVSGIIKPRRMTLLLGPPGSGKTTLLLALAGRLDKDLKVS

GKVTYNGHEMTEFVPERTAAYISQHDLHIGEMTVRETLAFSARCQGVGSRFDMLTELSRREKAANIKPDADID
AFMKASAMGGQDANVVTDYILKILGLEICADTMVGDEMLRGISGGQRKRVTTGEMLVGPARALFMDEISTGL
DSSTTFQIVNSLRQSIHILGGTAVISLLQPAPETYNLFDDIILLSDGQVVYQGPREEVLEFFESVGFRCPERKGVA
DFLQEVTSKKDQKQYWARPNEPYRFVAVKEFATAFKSSHTGRSITNELAVPFDKSKSHPAALTTTRYGVSGKE
LLKANIDREILLMKRNSFVYMFRTFQLMLMSIIAMTLFFRTKMKHGTVNDGGLYMGALFFGVLMIMFNGFSEL
ALTVFKLPVFFKQRDLLFFPAWSYTIPSWILKVPITFIEVGGYVFLTYYVIGFDPNVGRFFKQYLLLLVVNQMAA
SLFRFIGGVSRNMIVANVFASFMLLVVMVLGGFILVRDKVKKWWIWGYWISPMMYAQNAISVNEMLGHSWD
KILNSTASNETLGVQVLKSRGVFTEAKWYWIGFGAMVGFTILFNALFTVALTYLKPYGNSRPSVSEEELKEKH
ANIKGEVLDGNHLVSASSHRSTGVNPETDSAIMEDDSALTKRGMILPFVPLSLTFDNIKYSVDMPQEMKAQGV
QEDRLELLKGVSGSFRPGVLTALMGVSGAGKTTLMDVLAGRKTGGYIEGDIRISGYPKKQDTFARVSGYCEQN
DIHSPQVTVYESLLFSAWLRLPKDVDSNKRKIFIEEVMELVELKPLRNALVGLPGVNGLSTEQRKRLTIAVELV
ANPSIIFMDEPTSGLDARAAAIVMRTVRNTVDTGRTVVCTIHQPSIDIFEAFDELFLMKRGGEEIYAGPLGHHSS
DLIKYFESLHGVSKIKDGYNPATWMLEVTTTSQEQILGVDFSDIYKKSELYQRNKALIKELSQPAPGSTDLHFPS
KYAQSSITQCVACLWKQNLSYWRNPPYNTVRFFFTTIIALLLGTIFWDLGGKTYTSQDLMNAMGSMYSAVLFI
GVMNCTSVQPVVAVERTVFYRERAAGMYSAFPYAFGQVNGSPGNQFHTRLFFFVLLLFDPTNLSPVSAQVVIE
LPYALAQDILYGVIVYSMIGFEWTAAKFFWYLFFGYFTLLYFTFYGMMAVGLTPNYHIAAIVSSAFYAIWNLFS
GFIIPRPKVPIWWRWYCWICPVAWTLYGLVVSQFGDVMTPMDDGRAVKVFVEDYFDFKHSWLGWVAAVVV
AFAVLFATLFGFAIMKLNFQKR

>XP_021306845.1_ABCG50_Sb
MEFSGRHTSCLSSQHENSSWAANKEGPSDDMELEVVTEECTNGGAAEEARENMLLLDDDNLWFLQRQKDIN
GRVGVKPPAIEVRYEKLCVEAESRYSSGATHLPTLWNSIKAAYSGFIMLFGLKSDDKVKINILEDVSGIIRPCRL
TLLLGPPGCGKSTLLRALAGQLDKSLKATGDISYNGYGLDEFAPEKTAAYISQYDLHIPEMTVRETLDFSARCQ
CVGTRDEILDEVNKREKMAGIIRGHDIHMKATSIGASEKKLHTDNILKIMGLDICADTMVGDAMRRGISGGEK
RRLTTAELIIGPAKAFFMDEISNGLDSSTTFRIIKCFQQLANINECTMLISLLQPTPEIFDLFDDLILMAEGKIIYHG
PRNEAHIFFEESGFRCPERKGMADFLQEVLHKKDQRKYWSGTEESYRYVSPHQLSSMFKKYQKLTKLENPSVA
QKIKMGKESLSFNNYSLSTLELFKACGARETLLIKRSMPFYAFKTVQLSIVAVITMSVFYRTHMTTDLTHANYY
MGALFYSILIIMLNGTPEISMQIARLPSFYKQRGYHFYPSWAYAIPASILKVPFSLSDSLVWICITYYGIGYTCTAS
RLFYQFLILCLLHQSVTSLYRFIASHAQTHILSFLYHFMSVALLQLFGGFILPKSSMPGWLSWGFWVSPLSYAQI
SIAINEFLAPKWQKETMQNKTVGNQILINHSLDYSWEFYWISVGVLLGYTFFFYIAFGLALAYRKPIQFQAYRG
NMPRKCSTNGQEEEINIKKESDDHANNVPQKAKMAMPTMQLALTFCNLNYYVDTPPEMLKQGCTTRRLQLL
NNVNGAFRPGVLSALMGVSGAGKTTLLDVLAGRKTGGYIEGDIRIGGYPKVQETFVRILGYCEQVDIHSPQLT
VEESVTYSAWLRLPSKVNEKTRSEFVDEVLKTVELDEIKYTLVGRPGMDGLSLEQRKRLTVAVELVSNPSVIL
MDEPTTGLDARSAAIVIRAVKNISETGRTVVCTIHQPSTEIFEAFDELILMKNGGNIIYNGPIGEQSCKVIEYFEKI
SGVPKIERNSNPATWMMEVTSTSMEAQSNIDFASTYQESSLHRERQELVKQLSTPLPNSENLCFSNCFRQNGW
GQFKACLWKQNIIYWRSPQYNLNRMVITILIALILGVLYWRYAKMLNNEQDLFNVLGSMYMGVIQLGVYSDM
SIISFSTTERIIMYREKFAGMYSSWSYSFAQAAIEIPYVFIQVLLYTFIIYPTIGYYWTAYKLIWFFYTTFCSLLSYI
YVGLLLVSITPNVQVATILGTFFNTMQALFSGFILPAPQIPKWWKWMYYLVPTSWVLNSMLTSQYGNVDKEIK
AFGETKTVAVFLNDYFRFHQDRMGVVAAVITAVPVVFVTLYSLSVEKLSFQKR

>XP_021312973.1_ABCG36_Sb
MDAAGDIQKVASMRRGGSGSVWRRGDDVFSRSSREEDDEEALRWAALEKLPTYDRVRRAIVPLDGDEAAGG
KGLVDVDVLSLGPRERRALLERLVRVADEDNERFLLKLKDRIDRVGIDMPTIEVRFQNLEAEAEVRVGSSGLPT
VLNSVVNTVEEAANALHILPSRKRIMPILHDVSGIIKPRRLTLLLGPPGSGKTSLLLALAGRLDKDLKFSGKVTY
NGHEMTEFVPERTAAYISQHDLHIGEMTVRETLAFSARCQGVGSGFDMLTELSRREKAANIKPDADIDAFMKA
YAMGGQDANVVTDYILKILGLEICADTMVGDEMLRGISGGQRKRVTTGEMLVGPARALFMDEISTGLDSSTTF
QIVNSLRQSIHILGGTAVISLLQPAPETYNLFDDIILLSDGQVVYQGPREEVPEFFESVGFRCPERKGVADFLQEV
TSKKDQKQYWVRPDEPYRFVSVKEFATAFKSFHTGRAIANELAVPFDKSKSHPAALTTTRYGVSGKELLKANI
DREILLMKRNSFVYTFRTFQLILNSIITMTLFFRTKMKHDTVNDGGLYMGAVFFGVVLIMFNGMSELSLTVFKL
PVFFKQRDLLFFPAWSYTLPSWIVKVPITFIEVGGYVFLTYYVIGFDPNVSRFFKQYLLLLAVNQMAAALFRFIS
GASRNMIVANVSASFMLLVVMVLGGFILQKDKIRKWWIWGYWISPMMYAQNAISVNEMLGHSWDKILNSTA
SNETLGVQSLKSRAVFTEAKWYWIGFGAMVGFTILFNALFTLALTYLKPYGNSRPSVSEEQLQEKHANIKGEV
LDANHLVSAFSHRSTDVNTETDLAIMEDDSASSKKGMILPFDPLSLTFDNIKYSVDMPQEMKAQGVQEDRLEL
LKGVSGSFRPGVLTALMGVSGAGKTTLMDVLAGRKTGGYIEGDIRISGYPKKQETFARVSGYCEQNDIHSPQV

TVYESLLFSAWLRLPKDVDSNKRKIFIEEVMELVELKPLRNALVGLPGVNGLSTEQRKRLTIAVELVANPSIIFM
DEPTSGLDARAAAIVMRTVRNTVDTGRTVVCTIHQPSIDIFEAFDELFLMKRGGEEIYAGPLGHHSSELINYFEA
IQGVSKIKDGYNPATWMLEVTTTSQEQILGLDFSDMYKKSELYQRNKALIKELSQPAPGSSDLHFPSKYAQSSIT
QCVACLWKQNMSYWRNPPYNTVRFFFTTIIALLLGTIFWDLGGKVSTQQDLMNAMGSMYSAVLFIGIMNCTS
VQPVVAVERTVFYRERAAGMYSAFPYAFGQVVIELPYALVQDILYGVIVYSMIGFEWTAAKFFWYLFFGYFTL
LYFTFYGMMTVGLTPNYHIASIVSSAFYALWNLFSGFIIPRPKTPIWWRWYCWICPVAWTLYGLVVSQFGDIM
TPMDDNRPVKVFVEDYFDFKHSWLGWVAAVVVAFTVLFATLFAFAIMKLNFQKR

>XP_021606416.1_PDR1_-l_Me
MEGGDITSRVSSARLSSSNIWRNTTLEVFSKSSSCNEDDEEALKWAALEKLPTYLRVRRGILTEEEGQSREIDIN
NLGFIEKRNLLERLVKIAEQDNEKFLLKLKNRIEKVGLDMPTIEVRFEHLTVETEAYVGSRALPTMFNFSANMF
EGFLNYLHILPSRKKPLSILNDVSGIIKPRRMTLLLGPPSSGKTTLLLALAGKLGKELKFSGKVTYNGHGMDEFV
PQRTSAYISQYDLHIGEMTVRETLAFSARCQGVGTRYEMLAELARREKAANIKPDPDIDIYMKAAALEGQEAN
VVTDYILKILGLEICADILVGDEMVRGISGGQKKRVTTGEMLVGPARALFMDEISTGLDSSTTFQIVNSLRQSIHI
LNGTALVSLLQPAPETYDLFDDIILLSDGQIVYQGPRENVLEFFEYMGFRCPERKGVADFLQEVTSKKDQEQY
WAFKDQPYSFVSVKEFAEAFQSFHVGRKLGDELATPFDKSKSHPASLTTKKYGVSKKELLKACISREYLLMKR
NSFVYIFKMTQLIIMAFVSMTIFLRTEMRRNTVADGGIYMGALFYTIIIIMFNGFSELAMTIMKLPVFYKQRDLLF
YPAWAYALPTWILKVPVTFVEVAVWVVMTYYVIGFDPNIGRFLKQYFILLITNQTSSALFRLTAALGRSVIVAN
TVGSFALLAVLILGGFIISRDSVKKWWIWGYWFSPMMYVQNGMSVNEFLGNSWNHFPPNSTEPLGVTVMKSR
GLFPEAYWYWIAVGALTGYIFLFNFLFTLALKYLDPFGRPQATISEEAYAEKTANETAEFIEQSSKGESSLEKGS
VSQRSASSRTPSTRVGSFSDVNQNRRRGMILPFQPLSITFDEIKYVVDMPQEMKAQGITEDRLELLKGVSGAFRP
GVLTALMGVSGAGKTTLMDVLAGRKTGGYIHGSISISGYPKKQETFARISGYCEQTDIHSPHVTVYESLLYSAW
LRLPPEVDSDTRKMFIEEVMELVELTNLRVALVGLPGVNGLSIEQRKRLTIAVELVANPSIIFMDEPTSGLDARA
AAIVMRTVRNTVDTGRTVVCTIHQPSIDIFDAFDELLLLKRGGEEIYVGPVGRHACHLIKYFEDIEGIPKVKDGY
NPATWMLEVTTAAQEVSLGINFSDIYKNSELYRRNKALIKELSIPPPGSRDLYFPTQYSQSFFTQCMACLWKQH
WSYWRNPPYSAVRLLFTTFIALMFGTIFWNLGSKRSRKQDLFNAMGSMYAAILFLGFQNSTSVQPVVAIERTV
FYRERAAGMYSELPYAFGQVVIELPYILVQTLIYGGIVYAMLGFEWTASKFLWYLFIMYFTLAYFTFYGMMTV
AITPNHNIAAIISSAFYGIWNLFSGFIIPRTRMPVWWRWNYWACPIAWTLYGLIASQYGDIKEELDSGETVEHFL
RSYFGFRHDFVGIVAIVLVGIAVFFGFTFAFSIKAFNFQHR

>XP_021608284.1_PDR1_-l_X1_Me
MEASDTGRVISSRRTHSFNGWTNNNTMEVFSKSSHHLEDDEEALKWAAVERLPTYLRVRRAILDKKEIDVNKI
GFLERRNLLERLVKIAEQDNETFLLKLRDRMERVGLDMPTIEVRFEHLNVEAEAYIGSRSLPTIFNFSINLLEGFL
NCLHIFPSRKKPLPILRDVSGIIKPRRMTLLLGPPSSGKTTLLLALAGKLGKDLKFSGRVTYNGHEMGEFVPQRT
AAYISQYDLHIAEMTVRETLAFSARCQGVGPRYEMLAELSRREKAANIKPDPDIDVYMKAAALEGQEANVVA
DYILKILGLEGCADTMVGDEMIRGISGGEKKRVTTGEMLVGPARALFMDDISTGLDSSTTFQIVNSLRQSIHILS
GTALVSLLQPAPETYDLFDDIILLSDGQIVYQGPRENVLEFFEHMGFKCPERKGVADFLQEVTSRKDQEQYWA
LKDLPYSYVSVKEFAEAFQSFHVGRKLGDELATPFDKSKCHPAALTTKKYGISKKELLKACFSRELLLMKRNSF
IYIFKMTQLVIMALISVTVFLRTEMHRETLTDGGIYLGALFFAIVTLMFNGFTELALTIMKLPVFYKQRDLLFYPS
WAYALPTWILKIPVTFVEVAIWVIITYYVIGFDPNIERFFKQYLILLCTNQMASGLFRLMAALGRNIIVANTVGSF
ALLVVLVLGGFVISRDNINKWWLWGYWISPLTYVQNAISVNEFLGKNWRHVPFLSTEPLGVGLLKSRGIFLEA
HWYWIGVGALIGYILLFNFLYTLALKYLDPFGKPQATLSKEVLAEQNANRTGEFSKSSTSGKSYLERGNESHKS
ISSRTLSARVDSFNDANQNKKRGIVLPFQPLSIAFNEIKYAVDMPKEMQAQGIPEDRLELLKGISGAFRPGVLTA
LIGISGAGKTTLMDVLAGRKTDGYIEGSIFISGYPKKQETFARISGYCEQTDIHSPHVTVYESLLYSAWLRLPPEV
NSHTRKMFIEEIMELVELTSLREALVGLPGVNGLSTEQRKRLTIAIELVANPSIIFMDEPTSGLDARAAAIVMRTV
RNTVDTGRTVVCTIHQPSIDIFDAFDELLLLQRGGEEIYAGPIGRHSCHLIKYFEDIKGVPKIKDGYNPATWMLEI
TSAAQEAALGINFADVYKNSELYRKSKAFIKELSTPQPGSKELYFLSHYSQPFLTQCMACLWKQHWSYWRNPS
YTAVKLLFTTVIALMFGTIFWDLGCKRRRQQDIFNAIGSMYVALIFIGVQNAVSVQPVVAIERTVFYRERAAGM
YSALPYAFGQVMIELPYVFIQTIIYGVIVYAMIGFDWTLSKFFWYIFFMYFTFLYFSFYGMMTTAITPDHNIAAV
VASAFYALWNLFSGFIIPLPRIPVWWKWYYWSCPLAWTLYGLVASQFGDFKDMLETGEPLDLFLRRYFGFRH
DFVRIAAVVTIGISVLFAFIFALAIKSLNFQKR

>XP_021609606.1_PDR1_-l_Me
MDGADLYRASSSIRRSSSIWRNNSADVFSRSSREEDDEEALKWAVLEKLPTYDRLRKGILISGSKGEANEVEIDS

LGFQERKSLLERLVRVTEEDNEKFLLKLKDRIDRVGIEVPTIEVRFEHLNIEAEALVGTSALPTFFNFLINILEGFL
NNLHVFPSRKKPFTILKDISGVIKPSRMTLLLGPPSSGKTTLLLALAGKLDPNLKFSGSVTYNGHGLNEFIAQRT
AAYISQHDLHIGEMTVRETLGFSARCQGVGCLQDMLAELSRREKAANIKPDPDIDVFMKAAATEGQEASLVTD
YILKILGLDVCADTLVGDEMLRGISGGQRKRVTTGEMLVGPSRALFMDEISTGLDSSTTYQIVNSLKQSIHILNG
TAVISLLQPAPETYDLFDDIILLADGQIVYQGPREDVLGFFEYMGFKCPERKGVADFLQEVTSRKDQQQYWAN
RDQPYSFISVQEFSEAFQSYDVGQRLGQELSTPFDKAKSHPAALPTVKYGVGMVELLKACFSREYLLMKRNSF
VYIFKLIQLTTMAIIGMTLFLRTNMHRDNLIDGGIYLGALFFSVVMIMFNGMSELSMTIAKLPVFYKQRDLLFYP
SWAYSLPSWILKIPISFLEVAVWVFITYYVMGFDPNVGRLFKQYILLFLVNQMASALFRFIAAVGRNMIVANTF
GSFALLILFALGGVVLSREEIKKWWIWGYWLSPMMYGQNAIVVNEFLGKSWSHIPPNSTESLGVLLMKSRGFF
PHAYWYWIGVGASAGFVLLFNLCFTLALTFLNPFEKPQAVISDEPETSGRLESRHTTNTENEMSDIDESNHKKK
KGMVLPFEPHSITFDNVIYSVDMPQEMRNQGIAEDKLVLLKGVSGAFRPGVLTALMGVSGAGKTTLMDVLAG
RKTGGYIEGSIKISGYPKKQETFARISGYCEQNDIHSPHVTVYESLIYSAWLRLPPEVDSETRKMFVDEVMELVE
LNPLRQALVGLPGVNGLSTEQRKRLTIAVELVANPSIIFMDEPTSGLDARAAAIVMRTVRNTVDTGRTVVCTIH
QPSIDIFEAFDELFLMKRGGEEIYVGPLGRHSSRLINYFEEINGVSKITDGYNPATWMLEVTSSAQELSLGVDFA
VIYKNSELYRRNKATIEALSTPAPGSKDLYFPTQYSQSFFTQCLACLWKQRLSYWRNPPYTAVRFLFTTFISLMF
GTMFWDLGTKTSKQQDIFNSLGSMYAAVLFIGIQNAASVQPVVAVERTVFYRERAAGMYSAMPYAYAQVLIE
LPYIFIQAAVYGLITYAMIGFEWTAAKFFWYLFFMYFTLLYFTYYGMMTVAVTPNQHIASIISSAFYAIWNLFSG
FIIPRTRMPVWWRWFYWVCPVSWTLYGLIASQFADIKDPIEGGVTVEQFVKAYYGVKHDFLGVVAAMIVGFT
VLFAFIFAVSVRSFNFQKR

>XP_021610343.1_PDR1_-l_Me
MEGDHYRASNSLRRGSSSVWRNNGLDVFSRSSREEDDEEALKWAALEKLPTYDRLRKGILVSVSKGGANEID
VDNLGFQERKTLLERLVKVAEEDNEKFLLKLKNRIDRVGIEVPTIEVRYEHLNVEAEALVGSNALPSFLNFSFSL
VEGLFRYILPNRKRPLTILKDVSGVIKPSRMTLLLGPPSSGKTSLLLALAGKLDPSLKVSGTVTYNGHSLNEFIPQ
RTAAYISQHDLHIGEMTVRETLAFSARCQGVGTQHEMLAELSRREKAANIKPDPDLDVFMKAAATEGQETSV
VTDYVLKILGLDICADTLVGNEMIRGISGGQRKRVTTGEMLVGPAKALFMDEISTGLDSSTTFSIVNSLKQSIHIL
NGTAVISLLQPAPETYNLFDDIILISDGQIVYQGPRENVLEFFEYMGFKCPERKGVADFLQEVTSKKDQQQYWA
RKDQPYRFVTVNEFAEAFQSYEVGRKIAEDLSVPFDRTKNHPAALTTKPYGVGKMELIKANFSREYLLMKRNS
FVYVFKLTQLIVMAIIGMTLFFRTEMKHDSFEDAGVYAGALFFTLITIMFNGMAELSMTIAKLPVFYKQRNLLF
FPAWSYAIPSWILKIPVTFLEVGVWVFITYYVIGFDPNVGRLFKQYMLLLLVNQMASGLFRFIASVGRNMIVAN
TFGSFALLTLFALGGFVLKRSDIKKWWIWGYWVSPLMYGQNAIVANEFLGNSWNHIPANSTSTDSLGVQFLKT
NGFFPHAYWYWLGVGASAGYILVFNLLYTVALTFLDQFEKPQAVISDEPEESNRSGGAIQLSQAESSHRTQTES
GTSGIDESNHNKKKGMVLPFEPHSITFDNVIYSVDMPQEMKSQGVVEDKLVLLKGVSGAFRPGVLTALMGVS
GAGKTTLMDVLAGRKTGGYIEGDIRISGYPKKQETFARISGYCEQNDIHSPYVTVYESLVYSAWLRLPPDVDSE
TRRMFVEEVMELVELNPLRQALVGLPGVNGLSTEQRKRLTIAVELVANPSIIFMDEPTSGLDARAAAIVMRTVR
NTVDTGRTVVCTIHQPSIDIFEAFDELFLMKRGGEEIYVGPLGRHSCHLIKYFEGIGGVSKITDGYNPATWMLEV
SSSAQELTLGVDFANVYRNSDLYRRNKAMIQELSKPAPGTEDLYFPTQYSQPFLTQCMACLWKQSWSYWRNP
PYTGVRFWFTTFIALMFGTIFWDLGSKMEKEGDLTNAMGSMYAAVLFLGVQNSSSVQPVVAVERTVFYREKA
AGMYSAMPYAYAQALIELPYIFAQAGVYSLITYAMIGFEWTAAKFFWYLFFLYFTLLYFTFYGMMTVAVTPN
HHIASIVSSAFYSIWNLFSGFIVPRPKMPVWWRWYYWGCPISWTLYGLFASQFADITKPLGTTGKTVEEYVNET
YGIKHDFLGASAGVIFGIALLFAVIFAVSIKAFNFQRR

>XP_021610345.1_PDR1_-l_Me
MESADLYRASSSLRRGSFSTWRNNPSDVFTRSSREEDDEEALKWAALEKLPTYDRLRKGIFLSASKGAVNEIDV
DNLGFQERKTLLERLVKVAEEDNEKFLLKLKNRIDRVGIEIPTIEVRFEHLNIEADAYVGSSALPSFINFSVHMLE
GFLNYLHVLPSRKRPLTILKDVSGVIKPSRMTLLLGPPSSGKTTLLLALAGKLDPNLKFSGTVTYNGHGMDEFIP
QRTAAYISQHDVHIGEMTVRETLAFSARCQGVGTQHELLAELSRREKDANIKPDPDIDVFMKAAATEGQETSV
VTDYILKILGLEICADTLVGNEMIRGISGGQRKRVTTGEMLVGPAKALFMDEISTGLDSSTTYQIVNSLKQSIHIL
NGTAVISLLQPAPETYDLFDDIILLSDGQIVYQGPREQVLGFFEYMGFKCPERKGVADFLQEVTSRKDQKQYW
ARRDQPYSFVTVQEFAEAFQSYDVGRRIGDELSTPFDKTKSHPAALSTKKFGVGKMELLKACMSREYLLMKR
NSFVYIFKLTQLTFMAIIMMTLFLRTEMHRDNIMDGGVYLGALFFTVIMVMFNGMAELSMTIAKLPVFYKQRE
LLFYPAWAYSIPTWILKIPVTFVEVAVWVFLTYYVVGFDPNVTRFFKHYFVLLLVNQMASALFRCIAATGRNVI
VANTFGSFSLLTLFALGGFVLSRDEIKKWWIWGYWMSPLMYGQNAIVANEFLGKSWSHIPPNSTESLGVQVM
KGRGFFPDAYWYWLGAGASAGFIIVFNICFALALTFLDPFEKPQAVITEDSQSNDPDDEDGGDILLTNNGSSHK

SSTGAGEEIRQVNHNKKKGMVLPFEPHSVAFDNVVYSVDMPQEMKSQGVLEDKLVLLKGVSGAFRPGVLTAL
MGVSGAGKTTLMDVLAGRKTGGYIEGNITISGFPKKQETFARISGYCEQNDIHSPHVTVYESLVYSAWLRLPPE
VDSKTRKMFVDEVMELVELNPLRQALVGLPGVSGLSTEQRKRLTIAVELVANPSIIFMDEPTSGLDARAAAIV
MRTVRNTVDTGRTVVCTIHQPSIDIFDAFDELFLMKRGGEEIYVGPLGRHSCHLIKYFEGIEGVSKITDGYNPAT
WMLDITSYAQELALNVDFAAIYKNSELYRRNKAMIMELSTPAPGSKDLYFPTQYSQSFLTQCIACIWKQRLSY
WRNPPYTAVRFLFTTFIALMFGTMFWKIGSKLKKQQDLFNAAGAMYAAVLFLGVQNASSVQPVVAIERTVFY
RERAAGMYSAMPYAYAQVLVELPYVFAQAIFYGTITYAMMGFEWTIAKFFWYLFFMYFTLLYFTLYGMMTV
AVTPNHHIAAIVSSAFYGMWNIFAGFILPRTRMPVWWRWYFWICPVAWTLYGLIASQFGDVKDVLANGQTVE
DFIREYYGYKHDFVGVTACVIVGIVVLFAFIFGISIRSFNFQRR

>XP_021610859.1_PDR1_-l_Me
MEGPEVYVGGGSFRRGDSSIWRSNAMDTFSKSSREEDDEEALKWAALERLPTYDRLKKGILTTSKGEASEIYV
QDLGFQERRTLVDRLVNVAEEDNERFLLNLKNRIERVGIELPTIEVRFEHLNIETEAHVGNRALPTFINFSIDMVE
GFLNKLHILPSRKKRLSILQDISGIIKPRRMTLLLGPPSSGKTTLLLALAGKLDPKLKFSGRVTFNGHEMNEFVPQ
RTAAYISQYDKHIGEMTVRETLAFSARCQGVGHRYEMLTELLRREKASNIKPDSDLDVFMKAIATEGQETSVIT
DYILKVLGLEVCADIMVGNEMLRGVSGGQRKRVTTGEMLVGPAKALFMDEISTGLDSSTTFQIVNSIKQYIKIL
NGTAVISLLQPAPETYDLFDDIILLSDGWIVYQGPREHVLEFFEFMGFKCPERKGVADFLQEVTSRKDQQQYWT
RKDDPYCFVTVQQFSEAFQSFHVGRNLQAELSTPFDKTKSHPAALTTKKYGVGKMELLKACFSRELLLMKRN
SFVYIFKLSQLTIMAIIAMTLFLRTEMHRESVIDGGIYVGALFYSVVFIMFNGLSEISMTISKLPVFYKQRNLLFYP
AWAFSLPPWIIKIPITLVQVALWVFITYYVIGYDPYVGRLFRQYLLLVLVSQMASALFRFIAAAGRDMIVANTF
GSFALLTLFALGGFILSRDNIKKWWIWGYWISPLMYGQNAIVVNEFLGKSWSRVLPNSSEPLGVEVLKSRGFFT
NAYWYWIGVGALVGFTLLYNLCFTLALTFLGPLQKPQAVISEDSPSDESGSDHQTSAKSGSGSGSSSARAEVRV
NSSHQNKKGMVLPFEPHFITFDEIRYSVDMPQEMKNQGVTEDKLELLRGVSGSFRPGVLTALMGVSGAGKTTL
MDVLAGRKTGGYIEGNITISGYPKKQETFARISGYCEQNDIHSPHVTVYESLLYSAWLRLSPDVSSETRKMFIEQ
VMHLVELEPLRQALVGLPGVSGLSTEQRKRLTIAVELVANPSIIFMDEPTSGLDARAAAIVMRTVRNTVDTGRT
VVCTIHQPSIDIFEAFDELFLMKRGGQEIYVGPLGRHSCHLIKYFEGIEGVPNIKDAYNPATWMLEVTSSAQESV
LGVDFAAVYRNSELYRSNKEMIEKLSTPAPDSKDLYFPSKYSQSFFTQCMACLWKQRWSYWRNPPYTAVRLL
FTTVIALMFGTMFWNLGSKTKKRQDLFNAMGSMYAAIVFLGVQNASSVQPVVAVERSVFYRERAAGMYSPL
PYAFAQVLIELPYIFIQSVVYGLIVYAMIGFEWNAAKFFWYLFFMYFTLLYYTFYGMMSVAATPNQHVGAIVS
SAFYSLWNLFSGFIIPRPRIPVWWRWYAWACPVAYTLYGLISSQFGDLKHTLESGETVEDFVRSYFGFRHELLG
AVAAAVVGFATLFAFIFAICIKFFNYQRR

>XP_021626775.1_PDR1_-l_Me
MESADLYRAGSSFRRGSSLTRRNNGLEIFAQSFREDDDEESLKWAALEKLPTYDRLRKGIITTMTGGASEIDVH
NIGLQERKNLLERLVRVADEDNEKFLLKLKNRIDRVGIDIPTIEVRFEHLTVEAEAYEGSRALPTFFNYFINMLE
GILNYFHILSSRKKHLHILKDVSGIIKPSRMTLLLGPPSSGKTTLLLALAGKLDPALKVSGWVTYNGHHLHEFIP
QRTAAYISQHDLHIGEMTVRETLAFSARCQGVGSRYDLLAELSRREIAANIKPDSDIDVFMKAAATEGQEANVI
TDYILKVLGLEVCADTMVGDEMLRGISGGQRKRVTTGEMLVGPALALFMDEISTGLDSSTTYQIVNSLRQNVQ
ILKGTALISLLQPAPETYDLFDDIVLLSDGLIVYQGPREQVLRFFEFMGFQCPIRKGVADFLQEVTSRKDQMQY
WARKDEPYRFITAKEFSEAYESFQVGRRLVEELATPFEKANSHPAALTTKKYGVNKKELFKACFSRELLLMKR
NSFFYVFKFSQLTILALITMALFFRTEMHRDSVIDGGIYMGALFFIMLMVLFNGMAEIPMTIAKLPVFYKQRDLR
FYPAWAYALPSWILKIPITFIEVGISVFLTYYVIGFDPNVGRLFRQYLLLLLVNQMASGLFRSIAAVARNMIVAN
TFGSFILLLLFVLGGFILSRDHIKKWWLWAYWTSPTMYGQNAIVVNEFLGHSWSHVLPNSTEPLGVQVLKSRG
FFTEAYWYWLGLGALCGFTILFNFLYTIALTYLNEFSKPQAVTSKEPQDNGTARMEDGVHLSYLGSSSNQQTN
TAVIGDEIIRGKSPKSSQNNHRKGMVLPFEPYSITFDEIVYSVDMPREMKNEGVCEDKLVLLNGVSGAFRPGVL
TALMGVSGAGKTTLMDVLAGRKTGGYIEGSIKISGYPKKQETFARISGYCEQNDIHSPHITVYESLVFSAWLRL
SHEVDNETREMFVEEVMELVELNTLRQALVGLPGVNGLSTEQRKRLTIAVELVANPSIIFMDEPTSGLDARAA
AIVMRTVRNTVDTGRTVVCTIHQPSIDIFEAFDELFLLKRGGHEIYVGPLGRYSCHLIKYFEGIEGVNKIKDGYN
PATWMLEVTTAAQEMALGVDFADIYRNSELYGRNKALIKDLSKSAPGSKDLHFPNKYSESFFGQFSACLWKQ
HLSYWRNPPYTAIRFLFTAFIGLVFGTMFWDLGPKLTKQQDLFNAMGSMYAAVQFLGVQNASSVQPVVAVER
TVFYRERAAGMYSALPYAFGQVVIELPYIFMQAAVYGVTVYAMIGFEWNASKFFWYLYFTYFTLLYFTFYGM
MAVGVSPNHHISSIISSAFYGIWNLFSGFIIPRTRMPAWCSWYYWLNPVSWTLYGLVASQFGDIKEKLETGETV
ELFTRDYFGFRHDFLGLVAAVVFGFVILFAFIFAVSIKMFNFQRR

>XP_021626777.1_PDR1_-l_Me
MESVDLYRARSSFRRSSPLTRRNSGLEIFAPSFREEDDEESLKWAALEKLPTYERLRKGILTTMTGGVSEIDVHN
IGSQERKNFLERLVKVADKDNEKFLLKLKNRIDRVGIDIPTIEVRFEHLTVEAEAYEGSRALPTFFNYYINMLEG
LLNNFHILSSRKKRLHILKDVSGIIKPSRMTLLLGPPSSGKTTILLALAGKLDPTLKVSGRVTYNGHHLHEFIPQR
TAAYISQHDLHIGEMTVRETLAFSARCQGVGSRYDLLAELSRREIAENIKPDSDIDVFMKAAATEGQEVNVMT
DYILKVLGLEVCADTMVGDEMLRGISGGQRKRVTTGEMLVGPSLALFMDEISTGLDSSTTYQIVESLRQYVQIL
KGTALISLLQPAPETYDLFDDIILLSDGLIVYQGPREQVLQFFEFMGFQCPERKGVADFLQEVTSRKDQMQYWA
RKDEPYRFITAKEFSEAYKSFHVGRGLREELATPFEKANSHPAALTTKKYGVNKKELFKACCSREFLLMKRNSF
FYVFKLCQLTITTLIAMALFFRTEMHRDSVTDGGIYVGALFFIVLVVLFNGMAEISMTIAKLPVFYKQRDLCFYP
AWAYALPTWILKIPITFIEIGISVFMTYYVIGFDPNVGRLFRHYLVLLLVNQMASGLFRSIAAVGRNMIVANTFG
SFVLLLLFVSGGVVLSRDNIKKWWMWSYWTSPMMYGQNAIVVNEFLGHSWSHVLPKSIEPLGIQVLKSRGFF
TEAYWYWLGAGALCGFTIVFNLLYTVALTFLSEYSKPQAVTSKEPQDNGTGRMEDGVRLGYHGNSSNQQTST
VSRDEIIREKSSRSSQNNRKGMVLPFEPHSITFDEIVYSVDMPQAMKNEGVHEDKLVLLNSVSGSFRPSVLTAL
MGVSGAGKTTLMDVLAGRKTGGYIEGSITISGYPKKQETFARISGYCEQNDIHSPHITVYESLLFSAWLRLPCEV
ETREMFIKEVMELVELNTLGQALVGLPGVNGLSTEQRKRLTIAVELVANPSVIFMDEPTSGLDARAAAIVMRT
VRNTVDTGRTVVCTIHQPSIDIFEAFDELFLMKRGGQEIYVGPLGRHSCHLINYFEGIEGVEKIKDGYNPATWM
LEVTSTTQEMALGVDFADIYRNSELYRRNKALIKDLSKSAPGSKDLYFPNQYSLSFFGQYLACLWKQHLSYWR
NPPYTAIRFLFTAFIGLIFGTMFWDLGSKMKKQQDLFNAVGSMYAAVLFLGFIYASAVQPVVSVERTVFYRER
AAGMYSALPYAFGQIVVELPYVFMQAAVYGVTVYAMIGFEWNASKFFWYLYFTYFSLLYFTFYGMMAVGVS
PNHQISSIISFAFFIIWNLFSGFIIPRTMMPAWCSWYYWLNPVSWTLYGLITSQFGDIKETLETGETVEHFTRHYF
GFRHDFLGLVAAMVFVFVILFALSFAVSLKVFNFQKR

>XP_021627417.1_PDR1_-l_Me
MEGADHYRASNSFKRNSFIWRNNSFQNSFVWRTDTCEAFSKSCREEDDEEALKWAALEKLPTYDRLRKGILVS
VSKGGANEIDVDALGFHDRQRLLERLVKVAEEDNEKFLLKLRRRIDRVGIELPKVEVRFENLNVEAEAFVGNR
ALPTFVNFCVNIIEGCLNSLHILPSRKKPLTILKDVSGVIKPSRMTLLLGPPSSGKTTLLLALAGKLDPDLKLSGS
VTYNGHGINEFIPQKTAAYISQHDLHIGEMTVRETLAFSARCQGVGWQHEMLAELSRREKASNIKPDPDIDVF
MKAAAIEGQESSVVTDYILKILGLEVCADTMVGDDMLRGISGGQRKRVTTGEMLVGPSKALFMDEISTGLDSS
TTFQIVNSLRQTVHILNGTAVISLLQPAPETYDLFDDIILLSDGQIVYQGPREDVLGFFEHMGFKCPERKGVADF
LQEVTSRKDQKQYWAHKDQPYSFVTVQEFVEAFQSYAVGRRIGQELSTPFDKSKSHPAALATGKYGVGKMEL
LKACLSREYLLMKRNSFVYFFKLFQLSLMGIIAMTLFLRTNMHRNDLNGGGIYLGALFFTVIMIMFNGMSELS
MTVAKLPVFYKQRDLLFYPSWAYSLPTWILKIPITFFEVAVWVLLTYYPIGFDPNVVRLLKQYILLLFVNQMAS
ALFRFIAAAGRNMIVANTFGSFALLIIFALGGVILSRDDIKKWWIWGYWISPMMYGQNAIVANEFLGKSWSHIP
PNSTESLGVQVLKSRGFFPQAHWFWLGVGALAGFIIVFNICFTLALTYLNPFDKPQAVISDEPEHSERTNTEDGT
SGINEAKHKKKGMVLPFEPHSITFDNVIYSVDMPQEMKNQGVLDDKLVLLKGVSGAFRPGVLTALMGVSGAG
KTTLMDVLAGRKTGGYIEGDIRISGYPKKQETFARISGYCEQNDIHSPHVTVYESLLYSAWLRLPKEVNSETRK
MFVEEVMELVELNPLRQALVGLPGVNGLSTEQRKRLTIAVELVANPSIIFMDEPTSGLDARAAAIVMRTVRNT
VDTGRTVVCTIHQPSIDIFEAFDELFLMKRGGEEIYVGPLGRQSGHLINYFEEIEGVEKIQDGYNPATWMLEVSS
SAQELSLGVDFAAIYKNSELYRRNKATIEELSRPAPGSKDLYFPTQYSQSFFTQCIACLWKQRLSYWRNPPYTA
VRFLFTTIIAMMFGTMFWGLGSKTSKEQDIFNSAGSMYAAVQFLGVQNAGSVQPVVAVERTVFYRERAAGMY
SPLPYAYAQVLVELPYIFVQAVVYGLMTYAMIGYEWTAVKFLWYIFFMYFTLLYFTYYGMMSVAATPNYHIA
SISSSAFYTIWNLFSGFIIPRTRMPVWWRWYYWLNPVSWTLYGLITSQFGDMKHILEGGQTVGEFVRDYYGIN
HHFIGVVAAVVLGFTLLFAFIFAISIRFFNFQRR

>XP_021627707.1_PDR1_-l_Me
MESTEVFMGGGSFRRGDSSIWRSNAMDSFSRSSREEDDEEALKWAALERLPTYDRLKKGILTTSKGEAREIDV
ENLGFQERRSLVDRLVKVAEEDNEKFLLKLRNRIDRVGIELPTIEVRFEHLKIETEAHVGSRALPTFINFSVDIVE
GFLNNLHILPSRKKRLTILQDISGIVKPRRMTLLLGPPSSGKTTLLLALAGKLDPKLKFSGRVIYNGHEMNEFVP
QRTAAYISQYDTHIGEMTVRETLAFSARCQGVGHRYEMLTELLRREKESKIKPDSDIDVFMKAIATQGQETSVI
TDYVLKILRLEVCADIRVGNEMLRGVSGGQRKRVTTGEMLVGPAKALFMDEISTGLDSSTTFQIVNSIKQYIQIL
NGTAVISLLQPAPETYDLFDDIILLSDGQIVYQGPREHVLEFFEFMGFKCPERKGVADFLQEVTSRKDQQQYWA
RKDEPYNFVRIKEFAEAFQSFHVGRHLQNDLSTPFDKAKSHPAALTTKRYGVGKMELLKACFSREILLMKRNS
FVYTFKLTQLAIMALVAMTLFLRTEMHRDSVTDGGVYVGSLFFSVAFNMFNGLSDISMTIAKLPVFYKQKNLL
FYPAWAYSIPPWITRIPLTLAQVSIWVFLTYYVIGYDPNVGRLYRQYLLLVLVSQMASALFRFIAAAGRTMIVA

NTFGSFALLVLFALGGFILSPDDIKKWWTWGYWISPLMYGQNAIVVNEFTSKSWSQVLPNSTEPLGIQVLKSRG
FFTNAYWYWIGVGALVGFTLLFNLCFTLALTFLGPLRKPQAVISEESQCNEPGIGQIKFIRHSESGRGSSTLWAE
AIDNDKHQNKRGMVLPFEPHSITFDEIRYSVDMPQEMKNQGVTEDKLELLRGVSGAFRPGILTALMGVSGAGK
TTLMDVLAGRKTGGYIKGNITISGYPKKQETFARISGYCEQNDIHSPHVTVYESLLCSAWLRLSHEVSSETRKM
FIEEVMQLMELQPLRQALVGLPGVSGLSTEQRKRLTIAVELVANPSIIFMDEPTSGLDARAAAIVMRTVRNTVD
TGRTVVCTIHQPSIDIFEAFDELFLMKKGGQEIYVGPLGPHSCHLIKYFEGIEGVPKIKDGCNPATWMLEVTSSA
QESVFGVDFTAIYGNSELYRRNKGLIERFSTPSPESTELYFPTQYSQSFFTQCMACLCKQHWSYWRNPPYTAVR
LLFTTVIALMFGTMFWDLGSKKTKRQDLFNAMGSMYAAIVFLGIQNASSVQPVVAVERTVFYRERAAGMYSP
LPYAFAQVLIELPYVFIQSVVYGLIVYAMIGFEWTAAKFFWYLFFMYFTLLYYTFYGMMSVAVTPNQHIASIVS
SAFYSIWNLFSGFIIPRPRIPVWWRWYAWACPVAYTLYGLVSSQFGDIKHTLESGETVEDFVRSYFGFKHELLG
AVAVAVFGFATLFAFIFAISIKFFNYQRR

>XP_021628884.1_PDR1_-l_X1_Me
MEGDLYKASSSLRRGSSSVWRNNGLDVFSRSSREEDDEEALKWAALEKLPTYDRLRKGILVSVSKGGANEIDV
ENLGFQERKALLERLVKVAEEDNEKFLLKLKNRIDRVGIEVPTIEVRYEHLNVEAEALVGSNALPSFLNFSISIV
EGLLNYLHILPNRKRPLTILKDVSGVIKPSRMALLLGPPSSGKTTLLLALAGKLDPNLKVSGNVTYNGHALNEFI
PQRTAAYISQHDLHIGEMTVRETLAFSARCQGVGTQHEMLAELSRREKAANIKPDPDLDVFMKAAATEGQETS
VVTDYVLKILGLDICADTMVGNEMIRGISGGQRKRVTTGEMLVGPAKALFMDEISTGLDSSTTFSIVNSLRQSV
HILNGTAVISLLQPAPETYNLFDDIILLSDGQIVYQGPREHVLEFFENMGFKCPERKGVADFLQEVTSKKDQQQ
YWARKDQPYRFVTVKEFAEAFKSFEVGQTITQVLSTPFDKSKNHPAALTTKPYGVGKMELLKANFSREYLLM
KRNSFVYIFKLSQVIINLIWIILFSVSKFCMWANDFSLFLRYQLIIMAIIGMTLFFRTEMSKDDLEGGGIYIGALFF
TLITIMFNGMSELSMTIAKLPVFYKQRNILFFPPWAYSIPSWILKIPITFLEVGVWVFLSYYVIGFDPNVGRLFKQ
YLLLLLVNQMASALFRFIASVGRNMIVANTFGSFALLTLFALGGVILSRENIKKWWIWGYWVSPLMYGQNSIL
ANEFLGNSWSHVPANSSSTESLGVQVLKNGGYFPHAYWYWIGVGASAGYMFLFNFLYTVALTLLDTFEKPQA
VISDEPEENDKTRGAIQLSQLESSHRTNTESGTSENNESSHNKKKGMVLPFEPHSITFDNVIYSVDMPQEMKHQ
GVVEDKLMLLKGVSGAFRPGVLTALMGVSGAGKTTLMDVLAGRKTGGYIEGDVRISGYPKQQETFARISGYC
EQNDIHSPHVTVYESLVYSAWLRLPSDVDSETRKMFVEEVMELVELNPLRQALVGLPGVNGLSTEQRKRLTIA
VELVANPSIIFMDEPTSGLDARAAAIVMRTVRNTVDTGRTVVCTIHQPSIDIFEAFDELFLMKRGGEEIYVGPLG
RHSCHLIEYFEGIEGVSKITDGYNPATWMLEVSSSAQELTLGVDFANIYRNSDLYRRNKEMIQELSKPAPGTED
LYFPTQYSQPFLTQCIACLWKQSWSYWRNPPYTAVRFWFTTFIALMFGTIFWDLGTKTERQSDLSNALGSMYA
AVLFLGLQNATSVQPVVAVERTVFYREKAAGMYSAMPYAYAQALIEIPYIFVQTVVYSVITYAMIGFEWTAAK
FFWYLFFLLFTLLYFTYYGMMTVAVTPNHHIASIVSSAFYSIWSLFSGFMIPRTKMPVWWRWYYWGCPISWTL
YGLLGSQFGDVKTMLGNTGQTVEEYVNDYYGIKHDFLGVVAGVVVGITVLFAFTFAISIKAFNFQKR

>XP_021628887.1_PDR1_-l_Me
MEGDHYRASTSLRRGSSSAWRNNVLDVFSASSRDEDDEEALKWAALEKLPTYDRLRKGILVSVSKGGANELD
VDNLGFNERKTLLERLVKVAEEDNEKFLLKLKNRLDRVGIEVPTIEVRYEHLNIEAEALVGSNALPSFLNFTISI
AEGLLNYLHIFPSRKRPLTILNDVSGVIKPSRMTLLLGPPSSGKTTLLLALAGKLDPNLKVSGNVTYNGHTLNEF
IPQRTAAYISQHDLHIGEMTVRETLAFSARCQGVGTQHEMLAELSRREKAANIKPDPDLDFFMKAAATEGQET
SVVTDYILKILGLDICADTLVGNEMIRGISGGQRKRVTTGEMLVGPAKALFMDEISTGLDSSTTFSIVNSLRQSIH
ILNGTAVISLLQPAPETYNLFDDIILLSDGQIVYQGPREHVLEFFEYMGFKCPERKGVADFLQEVTSKKDQQQY
WARKDRLYRFITVREFAEAFQSYEVGRKIAGDLKTPFDRRKNHPAALATKHYGVGKMELLKANFSREYLLMK
RNSFVYIFKLSQLIMMATIGMTLFFRTEMKRDDLEDAGVYLGALFFTLITIMFNGMAELSMTIAKLPVFYKQRN
LLFFPAWSYSIPSWILKIPVTFLEVGVWVFLTYYVIGFDPNVGRLFKQYMLLLLVNQMASALFRFIASVGRNMI
VANTFGSFALLTLFALGGFVLSREDIKKWWIWGYWVSPLMYGQNAIVANEYLGHSWSHIPANSNSTDSLGVQ
FMKSRGFFPNAYWYWLGVAASAGYILLFNLAYTVALTFLDSFEKPQAVISDEPEESKSSERAIQLSKLESSHRT
NTESRTSGIDESNHNRKKGMVLPFEPRSITFDNVMYSVDMPQEMKNQGVLDDKLVLLKGVSGAFRPGVLTAL
MGVSGAGKTTLMDVLAGRKTGGYIEGDIRISGYPKKQETFARISGYCEQNDIHSPHVTVYESLVYSAWLRLPQ
EVDSETRRMFVEEVMELVELNPLRQALVGLPGVNGLSTEQRKRLTIAVELVANPSIIFMDEPTSGLDARAAAIV
MRTVRNTVDTGRTVVCTIHQPSIDIFEAFDELFLMKRGGEEIYVGPLGRHSCHLIKYFEGMEGVSKITDGYNPA
TWMLEVTSAAQELALGVDFAEIYRNSDLYRRNKTMIQELSKPAPGTQDLYFPTKYSQPFLTQCLACLWKQSW
SYWRNPPYTGVRFWFTTFIALMFGTIFWDLGSKKGKEGDLSNAMGSMYAAVLFLGVQNASSVQPVVAVERT
VFYREKAAGLYSAMPYAYAQALIELPYIFAQAGVYSVITYAMIGFEWTAAKFFWYLFFLYFTLLYFTFYGMMT
VAVTPNHHIASIVSSAFYAIWNLFSGFIIPRTKMPVWWRWYYWGCPISWTLYGLIASQFADIKTMLGDSGKTVE

EYVNDFYGIKHDFVGVAAGVIVGITVLFAFIFGISIKAFNFQRR

>XP_021628888.1_PDR1_-l_Me
MESGDIYKASSSLRRGSFSTWKHHPSGIFSTSSREEDDEEALKWAALEKLPTYDRLRKGILLSASKGAVNEIDV
DNLGFQERKTLLERLVKVAEEDNEKFLFKLKNRIDRVGIEIPTIEVRFEHLNVEADAFVGSRALPSFINFSVNML
EGFLNYIPIFPSRKRPLTILKDVSGVIKPSRMTLLLGPPSSGKTTLLLALAGKLDPNLKFSGTVTYNGYKMNEFIP
QRTAAYISQHDVHLGEMTVRETLAFSARCQGVGTQHELLAELSRREKAANIKPDPDIDVFMKAAATEGQETSV
VTDYILKILGLEICADILVGDEMVRGISGGQRKRVTTGEMLVGPAKALFMDEISTGLDSSTTYQIVNSLKQSIHIL
NGTAVISLLQPAPETYDLFDDIILLSDGQIVYQGPREQVLGFFEHMGFKCPERKGVADFLQEVTSRKDQKQYW
ARRGQPHRFITVQEFSEAFQSYELGRRIADELSTPFDKTKSHPAALSTKKYGVGKMELLKACISREFLLMKRNS
FVYIFKLTQLTFMASVSMTLFLRTEMHRNNLTDGGIYLGALFFSVIMVMFNGMAELSLTIAKLPVFYKQRDFLF
YPAWAYSLPTWILKTPVTFVEAAVWTVLTYYEIGFDPNVVRFFKHYLVLLLVNQMASSLFRFIAATGRNVIVA
NTFGSFSLLTLFALGGFILSREEIKKWWIWGYWISPLMYGQHAVVVNEFLAKSWRHIPPNSTEPLGVQLLKTRG
FFPHAYWYWLGVGALGGFVLLFNFCFTLALTFLDPFEKPQAVIVEDSQSNEPGDENGKGAGEEISEANHNKKK
GMVLPFEPRSVAFDNVMYSVDMPQEMKSQGVLEDKLVLLKNVSGAFRPGVLTALMGVSGAGKTTLMDVLA
GRKTGGYIEGDIRISGYPKKQETFARVSGYCEQNDIHSPFVTVYESLVYSAWLRLPPEVDSDTRKMFVDEIMEL
VELDPLKQALVGLPGVSGLSTEQRKRLTIAVELVANPSIIFMDEPTSGLDARAAAIVMRTVRNTVDTGRTVVCT
IHQPSIDIFDAFDELFLMKRGGEEIYVGPLGRLSCHLIKYFEGIEGVSKITDGYNPATWMLEVTSSAQELALNVD
FAAIYKNSELYRRNKEMIKELSTPAPGSTDLYFPTKYSQSFFTQCIACLWKQRLSYWRNPPYTAVRFLFTTFIAL
MFGTMFWDLGSKKTKQQDIFNAAGSMYAAVFFLGVQNASSVQPVVAIERTVFYRERAAGMYSAMPYAYAQ
VLVELPYIFAQAIVYAVITYAMMGFEWTGIKFFWYIFFMYFTLLYFTFYGMMTVAVTPNHHIAAIVSSAFYGL
WNLFAGFIVPRTKMPVWWRWYYWGCPVSWTLYGLVASQYGDVKDVLDTNQTVEDFVREYYGFKHDFVGVI
AGGIVGISVLFAFIFGFSIRFFNFQRR

>XP_024445693.1_PDR1_Pt
MDGGGDIYRVSSARLSSSSNIWRNSTLDVFSRSSRDEDDEEALKWAAIEKLPTCLRMRRGILTEEEGQAREIDIA
SLGLIEKRNLVERLVKIAEEDNERFLLKLKERIHRVGLDIPTIEVRFEHLSIEAEAYVGGRALPTIFNFSANMLEG
FLSFLHILPSRKQPFPILHDLSGIIKPRRMTLLLGPPSSGKTTLLLALAGKLGKDLKSSGSVTYNGHGMAEFVPQR
TSAYISQYDLHIGEMTVRETLSFSARCQGVGPRYEMLTELSRREREANIKPDPDIDIFMKAAALEGQETTVTTD
YILKILGLDICADTMVGDEMIRGISGGQKKRLTTGEMLVGPARALFMDEISTGLDSSTTFQIANSLRQTTHILNG
TTFISLLQPAPETYDLFDDIILLSEGLIIYQGPRENVLEFFESLGFKCPERKGVADFLQEVTSRKDQEQYWACRDQ
PYSFVSAKEFSEAFQSFHIGRKLGDELATPFDKSKSHPAALTTEKYGVSKKELLKACISREFLLMKRNSFVYIFK
FTQLIILASITMTIFLRTEMHRNTIVDGGIYLGALFFAIIVIMFNGFSELAMTIMKLPIFYKQRDLLFYPPWAYAIPT
WILKIPITFVEVAIWTIMTYYVIGFDPNIGRFFKQYLIFVLANQMSSGLFRMTGALGRNIIVANTFGSFAFLAVLV
LGGFILSRDNVKPWWIWGYWVSPLMYVQNAASVNEFLGHSWRHIPPNSTESLGVVVLKSRGIFPEAHWYWIG
IGALIGYTLLFNFLFTLALKYLNPFGKPQAMLSKEALAERNANRTGELIELSTRGKSSSVRGIDSRRSSSARPPSL
RMHSFGDASQNKRGMVLPFQPLSITFDEIRYSVDMPQEMKAQGILEDRLELLKGVSGAFRPGVLTALMGVSGA
GKTTLMDVLSGRKTGGYIEGRISISGYPKNQQTFARISGYCEQMDIHSPHVTVYESLVYSAWLRLSPDVDSETR
KMFIEEVVELVELNPLREALVGLPGVNGLSTEQRKRLTIAVELVANPSIIFMDEPTSGLDARAAAIVMRTVRNT
VDTGRTVVCTIHQPSIDIFDAFDELFLLKRGGEEIYVGPVGRHACHLIKYLEEIEGVPKIKDGHNPATWMLEVTS
AAQEALLGVDFTDIYKNSELFRRNKALIKELSSPPPGSNDLYFPTQYSHSFFTQCMACLWKQHWSYWRNPPYT
AVRLLFTTFIALMFGTIFWDMGSKRRNRQDIFNSMGSMYAAVLFIGVQNATSVQPVVAIERTVFYRERAAGMY
SALPYAFAQVMIEIPYVLVQTLIYGVIVYTMIGFDWTVSKFFWYIFFMYFTLLYMTFYGMMTVAVTPNHNVAA
IVSSAFYAIWNLFSGFIVPRTRIPIWWRWYFWACPISWTLYGLIASQYGDIKDKLEGDETVEDFVRNYFGFRHDF
VGTCAIVIVGICVLFAFTFAFSIRAFNFQRR

>XP_024453096.1_PDR1_X1_Pt
MESAVISRGSDSFRGSSRGVSSVWRNSTVEVFSRSSREEDDEEALKWAALEKLPTYDRLRKGILTSASRGIISEV
DIENLGVQERKQLLERLVKVADEDNEKFLWKLKNRVERVGIEFPTIEVRYENLNIEAEAYVGSSALPSFAKFIFH
IIEGFFIALHVLPSRKKPLTILKDVSGIIKPSRLTLLLGPPNSGKTTLLLAMAGKLDPSLKFSGHVTYNGHEMNEF
VPQRTAAYVSQHDLHIGEMTVRETLEFSARCQGVGHLHEMLAELSRREKEANIKPDQDVDVFMKAVATQGQ
EASVITDYVLKILGLEVCADTLVGDEMIRGISGGQRKRVTTGEMLVGPSRALLMDEISTGLDSSTTYQIVNSLK
QTIHVLNCTAVISLLQPAPETYDLFDDIILLSDGQIVYQGPRENVLGFFEHMGFKCPDRKGVADFLQEVTSKKD
QEQYWAIKDQPYRFVRVNEFSEAFQSFNVGRKIADELSIPFDKTKNHPAALVNKKYGAGKMDLLKANFSREY

LLMKRNSFVYIFKICQLTVVALISMSLFFRTKMHHDTVADGGIYTGALFFTVIIIMFNGMSELSMTIVKLPVFYK
QRELLFFPPWAYSIPPWILKIPVTFVEVAAWVLLTYYVIGFDPNVERLLRQYFLLLLINQMASALFRFIAAAGRN
MIVANTFGSFALLTLFALGGFILSREQIKKWWIWGYWLSPLMYGQNAIVVNEFLGHSWSHIPGTSTEPLGIQVL
KSREFFTEANWYWIGVGATVGFMLLFNICFALALTFLNAFEKPQAFIFEESEREGSVGKTGGAVQLSNHGSSHK
NKTENGDEINRNGFASIGEASDNRKRGMVLPFEPHSITFDDVIYSVDMPQEMKIQGVVEDRLVLLKGVNGAFR
PGVLTTLMGVSGAGKTTLMDVLAGRKTGGYIEGDIKISGYPKKQETFARIAGYCEQNDIHSPHVTVYESLLYSA
WLRLPPEVDSETRKMFIDEVMELVELDSLRNALVGLPGVNGLSTEQRKRLTIAVELVANPSIIFMDEPTSGLDA
RAAAIVMRTVRNTVDTGRTVVCTIHQPSIDIFDAFDELFLMKRGGEEIYVGPLGHHSTHLIKYFEAIEGVSKIKD
GYNPATWMLEVTASSQEMALEVDFANIYKNSDLFRRNKALIAELSTPAPGSKDVHFPTRYSTSFFTQCMACLW
KQHWSYWRNPPYTAVRFLFTTFIALMFGTMFWDLGSKVKTTQDLSNAMGSMYAAVLFLGFQNGTAVQPVV
AVERTVFYRERAAGMYSALPYAFAQALIELPYVFVQAAVYGVIVYAMIGFEWTAAKFFWYLFFMYFTLLYFT
FYGMMAVAVTPNHHIAGIVSTAFYAIWNLFSGFIIPRTRIPIWWRWYYWGCPVSWSLYGLVVSQYGDIQEPITA
TQTVEGYVKDYFGFDHDFLGVVAAVVLGWTVLFAFIFAFSIKAFNFQRR

>XP_024460182.1_PDR1_Pt
MDGAGDIYRVSSARLSTSSNKWRNSIPEVFSRSSRDEDDEEALKWAALEKLPTYLRLTRGILTEEEGKAREIDI
MNLGLVEKRDLLERLVKIAEEDNERFLLKLKERIDRVELEIPTIEVRFEHLNVEAEAYVGGRALPTILNFSANML
EGFLSFLHLLPSRKQPFPILRDVSGIIKPRRMTLLLGPPSSGKTTLLMALAGKLGKDLQCSGSVTYNGHGMEEFV
PQRTSAYISQFDLHIGEMTVRETLSFSARCQGVGPRYEMLTELSRREKEANIKPDPDLDIYMKAAALEGQETSV
TTYYILKITGLDICADTMVGDEMIRGISGGQKKRLTTGEMLVGPARALFMDEISTGLDSSTTFQIVNSLRQTTHI
LNGTTLISLLQPAPETYDLFDDVILLSDGLIVYQGPRENVLEFFESLGFKCPERKGVADFLQEVTSRKDQEQYW
ASRDQPYSFVSAKEFSEAFQSFHIGRKLGDELAIPFDKSKSHPSALSTEKYGVSKKELLKACISREFLLMKRNSF
VYIFKFTQLILLASIAMTVFLRTEMHRNTITDGGIYIGALFFAIIVIMFNGFSELVMTIMKLPVFYKQRDLLFYPP
WAYAIPTWILKIPITFVEVAIWTTMTYYAVGFDPNIGRFFKQYLIFVLANQMSSGLFRMMGALGRNVIVANNV
GSFALLAVLVMGGFILSRDNVKSWWIWGYWVSPLMYVQNAVSVNEFLGNSWRHIPPSSTESLGVTLLKSRGV
FPEARWYWIGVGALIGYTLLFNFLFTLALKYLNPFGKPQAILSKEALAERDANRTGNFIELSTRGKSSSERGKDS
KTNSSARAPSLRMPSLGDANQNKRGMVLPFQPLSITFEEIRYSVDMPQEMKAQGIPEDRLELLKGVSGAFRSGV
LTALMGVSGAGKTTLMDVLSGRKTGGYIDGRISISGYAKNQQTFARISGYCEQTDIHSPHVTVYESLVYSAWL
RLSPDVDSETRKMFIEEVMELVELNPLREALVGLPGVDGLSTEQRKRLTIAVELVANPSIIFMDEPTSGLDARAA
AIVMRAVRNTVDTGRTVVCTIHQPSIDIFDAFDELFLLKRGGEEIYVGPVGRHACHLIKYFEEIEGVPKIKDGYN
PATWMLEVTSAAQEAVLNDNFTDIFKNSELYRRNKALIKELSAPPPGSKDLYFPTRYSQSFFTQCMACLWKQH
WSYWRNPPYNAVRLLSTTVIALMFGTIFWNLGSKRNRKQDIFNSMGSMYAAVLFIGVQNATSVQPVVAIERTV
FYRERVAGMYSALPYAFAQVMIEIPYTLVQALIYGVIVYSMIGFEWTAIKFFWYIFFMYFTLLYMTFYGMMNV
AITPNHSIASLVSSAFYAIWNLFSGFIIPRTRVPIWWRWYCWACPFSWTLYGLIASQYGDLEDKLESDETVKDFL
RNYFGFRHDFVGICAIVVVGMSVLFAFTFAFSIRTFNFQRR

>XP_024460925.1_PDR1_Pt
MESADIYRASSSLRDSFRAGSSAWRNTTVEAFSRSSREEDDEEALKWAAIEKLPTYDRLRKGILTSASKGVANE
VDIEKLGLQERKQLLERLVKVAEEDNEKFLWKLKDRVERVGIDVPTIEVRYDNLNIEAEAYVGSSALPSFAKFT
FNIIEGLLISLNILRNRKKPLTILKDVSGIVKPSRLTLLLGPPSSGKTTLLLALAGKLDPNLKFSGRVTYNGHEMN
EFVPQRTAAYISQHDVHIGEMTVRETLAFSARCQGAGYLHDMLAELSRREKEANIKPDPDVDVFMKAVASQG
DEANVITDYVLKILGLEICADTMVGDEMIRGISGGQRKRVTTGEMLVGPSRALFMDEISTGLDSSTTYQIVNSL
RHTVHILNCTAVISLLQPAPETYDLFDDIILLSDGQIVYQGPRERVLEFFEHMGFKCPERKGVADFLQEVTSRKD
QEQYWARKDQPYRFITANEFAEAFQSFTVGRRTAEELSIPFDKSKNHPAALVTKTHGAGKKDLLKANFSREYL
LMKRNSFVYIFKICQLTIMALISMTLFFRTKMHRDTVRHGGIYTGALFFTAIMIMFNGMSELSMTIAKLPVFYKQ
RDLRFFPSWAYAFPQWILKIPVSFVEVAAWVFLTYYVIGFDPNVERLFKQYLVLLLINQTASALFRFIAAAGRN
MIIANTFGSFALLTLFTLGGFILSREKIKKWWIWGYWSSPLMYGQTAILVNEFLGNSWSHVPENSTEPLGIQVLK
SRGFFTEAYWYWIGAGATIGFILLLNLFFVLALTFLNAFDKPQAFISEEPESDESGRKTERAIQLSNHGSSHGTNT
EGGVGISRASSEVIGGVSNNRKKGMVLPFEPHSITFDDIIYSVDMPQEMKVQGVVQDRLVLLNGVNGAFRPGV
LTALMGVSGAGKTTLMDVLAGRKTGGYIDGEIKISGYPKKQETFARVSGYCEQNDIHSPQVTVYESLLYSAWL
RLPPEVDSETRRMFIEEVMDLVELNPLRHALIGLPGVNGLSTEQRKRLTIAVELVANPSIIFMDEPTSGLDARAA
AIVMRTVRNTVDTGRTVVCTIHQPSIDIFEAFDELFLMKRGGQEIYVGPLGRHSTHLIKYFEAIEGVSKIKDGYN
PATWMLEISSSAQEMALEVDFSNIYKNSDLFRRNKALIVELSTPAPGSTDLYFPTKYSTSFLTQCMACLWKQH
WSYWRNPPYTAVRFIFTTFIALMFGTMFWDLGSKVSSTQDLSNAMGSMYAAVLFLGVQNASSVQPVVAVERT

VFYRERAAGMYSALPYAFAQVLIELPYIFAQAAVYGIIVYAMIGFDWTVAKFFWYLFFMYFTLLYFTYYGMM
AVAVTPNHHIASIVSSAFYGIWNLFSGFIVPRPSIPIWWRWYSWACPVAWTLYGLVVSQFGDIQKKLTETQTVK
EYVKDYFGFHHDFLGVVAAAIVGWTVLFAFIFAFAIKAFNFQRR

>XP_024460929.1_PDR1_X1_Pt
MESADIYRASSSLRDSLRAGSFVWRNSTVEAFSRSSREEDDEEALKWAALEKLPTYDRLRKGILMSASRGVSSE
VDIEKLGVQERKQLLERLVKAADEDNEKFLWKLKNRIERVGIEFPTIEVRYEHLNIGAEAYVGSGALPSFAKFT
FSIIEDLLIALRIIPSRKKPLTILKDVSGIIKPSRLTLLLGPPSSGKTTLLLALAGKLDPSLKYSGRVTYNGHGMNEF
VPQRTASYTSQQDLHIGEMTVRETLAFSARCQGVGNLHDMLAELSRREKEANIKPDPDIDVFLKAVATQGQEA
NVITDYVLKILGLEVCADTLVGDEMIRGISGGQRKRVTTGEMLVGPSRALFMDEISTGLDSSTTHQIVNSLKQTI
HILNYTAVISLLQPAPETYDLFDDIILLSDGQIVYQGPRENVLGFFEHLGFQCPERKGVADFLQEVTSRKDQEKY
WARKDQPYRFVTVNEFAEAFQSLSVGRRVIEELSIPFDKTKNHPAALVNKKYGAGKMDLLKANFSREFLLMK
RNSFVYIFKIFQLTMMAIISMTLFFRTKMPRDTVEDGGIYAGALFFTAIMIMFNGMAELSMTIAKLPVFYKQREL
LFFPPWTYSIPPWILKIPITFVEVAAWVFLTYYVIGFDPNIGRFFKLYAVLVLINQMASALFRFIAAAGRNMIVAN
TFGSFVLLAVFALGGVILSREQIKKWWIWGYWASPLMYGQNAIVVNEFLGNSWSHIPAGSTESLGIQVLKSREF
FTEAYWYWIGIGATAGFILLFNVCFVVALTVLDAYEKPQAVISEEPESGDSEGAVQLSNRGISHQTNTDSIGEAS
NNRKKGMVLPFEPHSITFDDVIYSVDMPQEMKVQGVAEDRLALLKGVSGAFRPGILTALMGVSGAGKTTLMD
VLAGRKTGGYIEGDIKISGYPKKQETFARISGYCEQNDIHSPQVTVYESLLYSAWLRLPSEVDSETRKMFIEEVM
DLVELNPLRSALVGLPGVNGLSTEQRKRLTIAVELVANPSIIFMDEPTSGLDARAAAIVMRTVRNTVDTGRTVV
CTIHQPSIDIFDAFDELFLMKRGGEEIYVGPVGRHSTHLIKYFEEIEGVSKIKDGYNPATWMLEVTSSSQEMALG
VDFANIYKNSNLLRRNKALIAELSTPAPGSKDIYFSTQYSTSFFTQCMACLWKQHWSYWRNPPYTAVRFLFTTF
IGLMFGTMFWDLGSKVGTAQDLSNAMGSMYAAVLFLGFQNGSAVQPVVAVERTVFYRERAAGMYSALPYA
FAQVLIEIPYVFVQSAVYGVIVYAMIGFEWTAAKFFWYLFFMYFTLLYFTFYGMMSVAVTPNHHIAAIVSTAF
YLIWNLFSGYIVPRPRIPIWWRWYYWACPVSWSLYGLVVSQYGDIQKNLTETETVKQYVKNYFGFDHDFVGV
VAAAVLGWTVLFAFIFAFSIRAFNFQRR

>XP_024625156.1_PDR1_Mt
MMEGEASFRISSSSIWRNSDAAEIFSNSFRQEDDEEALKWAAIQKLPTFARLRKGLLSLLQGEATEIDVEKLGLQ
ERKDLLERLVRLAEEDNEKFLLKLKDRIDRVGIDLPTIEVRFEHLNIEAEANVGSRSLPTFTNFMVNIVLGLLNS
LHVLPSRKQHLNILREVSGIIKPSRITLLLGPPSSGKTTILLALAGKLDPKLKVSGKVTYNGHEMGEFVPQRTAA
YVDQNDLHIGEMTVRETLAFSARVQGVGPRYDLLAELSRREKHANIMPDPDIDVYMKAIATEGQKANLITDY
VLRILGLEICADTVVGNAMLRGISGGQKKRVTTGEMLVGPTKALFMDEISTGLDSSTTFQIVNSIKQYVHILKGT
AVISLLQPPPETFNLFDEIILLSDSHIIYQGPREHVLEFFESIGFKCPDRKGVADFLQEVTSRKDQEQYWEHKDQP
YRFITAEEFSEAFQSFHVGRRLGDELGTEFDKSKSHPAALTTKKYGVGKWELFKACLSREYLLMKRNSFVYIFK
IFQLCVMAMIAMTIFFRTEMHRDSLTHGGIYVGAIFYGVVTIMFNGMAEISMVVSRLPVFYKQRGYLFFPPWA
YALPEWILKIPLSFVEVAVWVFLTYYVIGFDPYIGRFFRQYLILVLVHQMASALFRFIAAVGRDMTVALTFGSF
ALAILFAMSGFVLSKDSIKKWWIWAFWISPMMYAQNAMVNNEFLGNKWKRVLPNSTEPIGVEVLKSHGFFSE
PYWYWIGVGALIGYTLIFNFGYILALTFLNPLGKHQTVIPEESQIRKRADVLKFIKDSFSQHSNRLRNGKSRSGSI
SPKTNHRRKRGMVLPFEPHSITFDEVSYSVDMPQEMRTRGVVENMLVLLKGLSGAFRPGVLTALMGVTGAGK
TTLMDVLSGRKTGGYIGGNITISGYPKKQDTFARISGYCEQTDIHSPYVTVYESLLYSAWLRLSPDINAETRKMF
IEEVMELVELKPLRNALVGLPGVSSLSTEQRKRLTIAVELVANPSIIFMDEPTSGLDARAAAIVMRTVRNTVDTG
RTVVCTIHQPSIDIFESFDELFLLKQGGQEIYVGPLGHNSSNLISYFEGIKGVSKIKYGYNPATWMLEVTTSSKER
ELGIDFAEVYKNSELYRRNKALIKELSTPAPCSKDLYFTSQYSRSFWTQCMACLWKQHWSYWRNPVYTAIRF
MYSTAVAVMLGTMFWNLGSKIEKVQDLFNAMGSMYSAVLLIGIKNGNAVQPVVSVERTVFYRERAAGMYSA
LPYAFAQVVIELPHVFVQSVVYGFIVYAMIGFEWTLVKFLWCLFFMYFTFLYFTFYGMMSVAMTPNNHISIIVS
SAFYSIWNLFSGFIVPRPRIPVWWRWYSWANPVAWSLYGLVTSQYGDVKQNIETSDGRQTVEDFLRNYFGFK
HDFLGVVALVNVAFPIVFALVFALSIKMFNFQRR

>XP_024625540.1_PDR1_X1_Mt
MEVGGSFRIGSSSIWRNSDAAEIFSNSFHQGDDEEALKWAAIQILPTFERLRKGLLTSLQGGTIEIDVENLGMQE
KKDLLERLVRLAEEDNEKFLLKLKDRIDRVGIDLPTIEVRFEHLNIEAEARVGSRSLPTFTNFMVNIVERIFNSLL
VLPSRKQHLNILKDVSGIIKPSRMTLLLGPPSSGKTTLLLALAGKLDQKLKFSGRVTYNGHEMSEFVPQRTAAY
VDQNDLHIGELTVRETLAFSARVQGVGPQYDLLAELSRREKDANIKPDPDIDVYMKAVATEGQKANLITDYVL
RVLGLEICADTVVGNAMIRGISGGQKKRLTTGEMLVGPTKALFMDEISTGLDSSTTFQIVNSMKQYVHILKGTA

VISLLQPPPETYNLFDSIILLSDSHIIYQGPREHVLEFFESIGFKCPNRKGVADFLQEVTSVKDQEQFWEHKDQPY
KFVTAEEFSEAFQTFHVGRRLGDELGTEFDKSKSHPAALTTKKYGVGKIELLKACSSREYLLMKRNSFVYIFKL
CQLAVMAMITMTVFLRTEMRKDSVVHGGIYVGALFFGVTVIMFIGMAELSMVVSRLPVFYKQRGCLFFPPWA
YSLPSWILKIPLTCVEVAVWVFLTYYVIGFDPYIGRFFRQYLILVLVHQMAAALFRFIAAVGRDMTVALTFGSF
AIAILFSMSGFVLSKDGIKKWWIWAFWISPLMYGQNAMVNNEFLGNKWKHVLPNSTESLGVEVLKSRSFFTET
YWYWICVGALIGYTLLFNFGYILALTFLNPLGKHQAVIPDESQSNEQIGGSQKRTNALKFIKDGFSKLSNKVKK
GESRRGSISPSRQEIVAAATNHSRKKGMVLPFEPHSITFDEVTYSVDMPQEMRNRGVLEDKLVLLKGVSGAFRP
GVLTALMGITGAGKTTLMDVLSGRKTGGYIGGNIKISGFPKKQETFARISGYCEQTDIHSPHVTVYESLLYSAW
LRLSPDINAETRKMFIEEVMELVELKPLQNAIVGLPGVSGLSTEQRKRLTVAVELVANPSIIFMDEPTSGLDARA
AAIVMRTVRNTVDTGRTVVCTIHQPSIDIFESFDELLLLKQGGQEIYVGPLGHNSSNLINHFEGIQGVSKIKDGY
NPATWMLEVTNSSKEVELGIDFVELYKNSELYRINKALIKELGSPAPCSKDLYFPTQYSRSFFTQCMACLWKQH
WSYWRNPEYNAIRFLYSTAVAVLLGSMFWDLSSKIEKEQDLFNAMGSMYAAVILIGVMNGNSVQPVVAVERT
VFYRERAAGMYSAFPYAFGQVVIELPYVFVQAVVYGIVVYVMIGFEWTLVKVVWCLFFMYFTFLYFTFYGM
MSVAMTPNNHISIIVSSAFYSVWNLFSGFVVPRPSIPVWWRWYSWANPVAWSLYGLVTSQYGDVKQNIETSD
GRQTVEDFLRNYFGFKHDFLGVVALVNIAFPIVFALVFAIAIKMFNFQRR

>XP_024633520.1_PDR1_Mt
MEGGELRVASGRVGSSSIWRSGAVDVFSGSSRRDDDEQELQWAAIEKLPTYLRMTRGILNESQSEQPIEIDINKL
GPLQRKNLVERLVKIAEEDNEKFLLKLRQRIDRVGLDFPTIEVRFEHLNVEAEAHVGSRALPTILNFSINLLEGFL
NNLHLIPSRKKPLTVLHDVSGIIKPKRMTLLLGPPSSGKTTLLLALAGRLSRDLKFSGRVAYNDHGMEEFVPQR
TSAYISQTDLHIGELTVRETLAFSARCQGIGTRYDMLAELSRREKAENIKPDPDLDIYMKAEALEGQETNIVTDY
IIKILGLDVCADTMVGDDMIRGISGGQKKRVTTGEMLVGPARALFMDEISTGLDSSTTFQMINSLRQSIHILNGT
ALISLLQPTPETYDLFDDIILLSDGQIVYQGPRENVLEFFEHVGFKCPERKGVADFLQEVTSRKDQEQYWSNKD
KPYTFITVREFAEEFQLFHVGQKLGDELGTPFDASKGHPAVLTKNKYGVSRKELLKACVSRELLLMKRNSFVYI
FKMWQLIFTGIVTMTMFLRTEMHRNTETDGGIYMGALFFILIVIMFNGYSELSMFIMKLPVFYKQRDLLLFPAW
AYSLPTWILKIPITFVEVGIWVVLTYYVIGFDPCFERFIKQYFLLVCINQMASALFRFIGAVGRNVIVANTVGSFA
LLAVLVMGGFILSRVDVKKWWLWGYWVSPMMYGQNAIAVNEFLGKSWSHIPPDSTEPLGVQILKSRGIFPEA
YWYWIGVGASIGYMLLFNFLFPLALHYLDAFGKPQALISEEALAERNAATAGSKQIIELSPKLECSSGNASRRSF
SSTTLSTKVGSINAADHTRKRGMVLPFTPLSITFDEIGYAVDMPQEMKAKGIPEDRLELLTGVNGAFRPGVLTA
LMGISGAGKTTLMDVLSGRKTTGYVQGQITISGYPKKQETFSRISGYCEQTDIHSPHVTVYESLVYSAWLRLPP
EVDTSTRKMFIEEVMELIELTSIREALVGLPGVNGLSTEQRKRLTIAVELVANPSIIFMDEPTSGLDARAAAIVMR
TVRNTVDTGRTVVCTIHQPSIDIFDAFDELLLLKRGGEEIYVGPLGRHCSHLINYFEGINGVPKIKNGYNPATWM
LEVTSEAQEEALGINFAELYKNSDLYRTNKALIRELSTPPEGSKDLYFTTQHSQSFLTQCMACLWKQNLSYWR
NPPYSAVRLLFTTVIAFLFGTIFWNIGSKRERRQDLFNAMGSMYAAVLFIGVQNATSVQPVVAIERTVFYREKA
AGMYSALPYAFGQVAVEIPYILIQSLVYGVIVYTMVGFERTPTKFFWYLFFMFFTFLYFTFFGMMLVGATPDH
NVAAIVSFGFYLLWNLFSGFVIPRTRMPVWWRWFFWICPISWTLYGLITTQFGDVNERMDTGETVEEFVRSYF
GYRDDFKDVAAAVVVSFSLIFGSAFAFSIKAFNFQKR

>XP_025887045.1_PDR1_-l_Sl
MEPANLNNFRGSLRASMRADSSRSVFSRSARDEDDEEALKWAALEKLPTFDRMRKGLLFGKEGESATEVDTN
DIGHQERKNLLDRLVKVADEDNEKFLLKLKDRIQTVGIDLPSIEVRYEHLNIVADAYVGSRALPTFINFMTNFV
ETFLNTIHILPSRKRQITILKDVSGMIKPSRMTLLLGPPSSGKTTLLLALAGKLDPTLKVTGKVTYNGHELHEFVP
QKTAVYISQYDLHIGEMTVRETLEFSARCQGVGPRYEMLAELSRREKAANIKPDHDIDIYMKASVTKGQEANI
VTDYVLKILGLDVCADTMVGDEMLRGISGGQKKRVTTGEMLVGPSKALFMDEISTGLDSSTTFSIVNSLRQLV
QLLKGTAVISLLQPAPETYNLFDDIILLSDACIVYQGPREDVLDFFESMGFKCPERKGVADFLQEVTSKKDQQQ
YWAKKDKPYRFITSKEFAEAYQSFHVGKELADELTTPYDKTKSHPAALSTQKYGIGTKELLNVCAEREFLLMK
RNSFVYIFKLFQLMVMAFIMMTVFFRTEMPRDDMDDGGMYAGALFFVVVVIMFNGMAEINLTILKLPVYFKQ
RDLLFYPSWAYALPTWILKIPITFIEVGLWTFLTYYVMGFDPNVSRLFKQFLLLVLVHQMASGLFRFIGAAGRT
MGVATTFGAFALVLQFALSGFVLSRNDVKKWWIWGYWISPLMYSVNSILVNEFDGKKWDHIVPNGAEPLGH
AVVRSRGFFPDAYWYWIGVGALIGYIIIFNLCYSIGLAYLNPFGKPQAIISEDSENVRLIEESETDSQDKKRGMVL
PFEPHSITFDNVVYSVDMPQEIKDQGSTEDRLVLLKGVSGAFRPGVLTALMGVSGAGKTTLMDVLAGRKTGG
YIDGDIKISGYPKKQETFARISGYCEQNDIHSPYITVYESLVYSAWLRLPQDVDKNKRKMFVEEVMELVELTPL
RSALVGLPGVNGLSTEQRKRLTIAVELVANPSIIFMDEPTSGLDARAAAIVMRAVRNTVDTGRTVVCTIHQPSID
IFEAFDELFLMKRGGQEIYVGPLGRHSCHLIKYFESLPGVSKIKEAYNPATWMLEVTAASQEMMLGVDFTDLY

KKSDLYKRNKALISELSMPRPGTKDLHFETQFSQPFWTQCMACLWKQHLSYWRNPSYTAVRFIFTVILALVFG
TLFWDLGSRVSQSQDLFNAMGSMYAATLFLGVQNSSSVQPVVAVERTVFYRERAAGMYSALPYAFGQVIVEI
PYVFVQAAFYGIIVYAMIGFEWTVAKFFWYLFIMYFTLLYFTFYGMMTVAISPNQNVASIVAAFFYAVWNLFS
GFIVPRPRIPIWWRWYYWLCPVAWTLYGLVASQFGDLQTMISNDENVEQFLGRYFGFEHDFLGVVAAVIVVW
PAVFAFLFAYAIKAFNFQKR

>XP_025982765.1_PDR1_X1_Gm
MESGGSFRIGSSSIWRDSDAKIFSNSYHRENDEEALKWATIQKLPTVVRLRKGLLTSPEGEVNEIDVQKLGFQER
RTLLDRLVRTVEDDNEKFLLKLKERVDRVGIDLPTIEVRFENLNIAAEACVGTRPLPTFTNFTVNIVQGLLNSLL
TLPSRRQQINILQDVSGIIKPGRMALLLGPPSSGKTTLLLALAAKLDPKLKFSGKVTYNGHGMNEFVPQRTAAY
VNQNDLHIAELTARETLAFSARVQGVGTRYDLLAELSRREKEANIKPDPDIDIYMKAVTTGVQKANLITDYVL
RILGLEVCADTIVGNAMLRGISGGQKKRLTTGEMLVGPVKALFMDEISTGLDSSTTFQIVNSLKQYVHILKGTA
VISLLQPAPETYNLFDDIIVLSDSHIGYQGPREYVLEFFESMGFKCPERKGVADFLQEVTSWKDQEQYWADKD
QPYRFVTSKEFSEAHRSFHVGRSLGEELATEFDKSKSHPAALTTKRYGVGKWELLKACLSREYLLMKRNSFYY
TFKLSKLAVMAFITMTIFLRTEMHRDSVTDGGIYVGAMFYGIVTVMFNGLAEISVIVSRLPVFYKQRDNIFFPS
WAYALPEWILKIPMSFAEVGVWVFLTYYVIGFDPYIERFFRQYLVLVLLNQMTSALFRFIAALGREPTVATTLA
WLTLAILYSISGFVLSKGIENTNNPSPSPIMFWTNNAPSFIPLMPTDKIKKWWLWGFWISPMMYGQNAMVNNE
FLGKRWRHILPDSTEPLGVEVLKSWGFFTQSHWYWIGVGALIGYTLLFNFGYILALMYLSPPGKHQAVISEEAQ
SNDQNGVRKFGSASGSTSSHTLPARGIVLPFQPHSITFDEVTYDVDMPQEMRKRGVVEDKLVILKGVSGAFRPG
VLTALMGITGAGKTTLLDVLAGRKTGGYVGGNITISGYQKKQETFPRISGYCEQNDIHSPHVTVYESLLYSAWL
RLSPDINTETKRLQMFIEEVMELVELKPLRHALVGLPGVNGLSTEQRKRLTIAVELVANPSIIFMDEPTSGLDAR
AAAIVMRTVRNTVDTGRTVVCTIHQPSIDIFESFDELLLMKQGGQQIYVGPLGQYSSNLISYFEGIQGVNKIKDG
YNPATWMLEVTTSAKEIELGIDFADVYKNSEHYRRNKALVKELSSPAPGSVDLYFPSQYSTSFITQCIACLWKQ
HWSYWHNSQYTTVSFLYSTTVAILFGSMFWNLGSKIEKQKDLFNAMGSMYASVLLIGIQNAYAVQPSISVERI
VFYRERAAGMYSALPYALAQVLIELPYVLVKAVVCSIISYAMIGFEWTVTKFFWYLFFLYFTFLYFTYYGMISV
AVTPNLHISSMVSSGFNSLWNIFSGFIVPRPRIPVWWRWYSWANPISWSLYGLVASQYGDIKQSIESTDGSSTTV
EDFVRSYFGFRHDFLWVVAAVIVAFPVVFALMFAISVKMLNFQRR

>XP_025982896.1_PDR1_X1_Gm
MEDGGSVKAGSTTNTMSSFRIGSRSVWSNSGVEIFANSFHQEDDEEALKWAAIQKLPTFARLRTGLMTSPEGV
ANEVNVHQLGLQERRGLLERLVRVAEEDNEKFMLKLRDRIDRVGITIPTIEVRFENMNIGAEVHVGSRALPTFT
NYMVNKVEGLLNFLHVLPSRKQRINILQNVSGIIRPARMTLLLGPPSSGKTTLLLALAGRLDSKLKFTGKVTYN
GHGMNEFVPQRTAAYVSQNDLHIGEMTVRETLAFSARVQGVGARYDLLAEVSRREKEANIKPDPDIDVYMKA
VATEGQKANFITDYILRILGLEVCADTIVGNAMLRGISGGQRKRVTTGEMLVGPAKAVFMDEISTGLDSSTTFQ
VVNSLKHFIHSLKGTAVVSLLQPAPETYNLFDDIILLSDGQIVYQGPREHVLEFFASVGFKCPERKGVADFLQEV
TSRKDQEQYWVHRDQPYRFVTTEEFVEAFQSFHVGRSLADELATQFDKSKSHPAALATKMYGLGKWELLKA
CLSREYLLMKRNSFVHIFQLCQLAIVAFIAMTVFFRTEMHPDSVTSGGIYAGALFYGLLVILLDGFADLTMTVS
KLPVFYKQRDFLFFPSWVYALPAWILKIPMTFAQVGIWVFLTYYVIGFDPYVGRFFRQFLLLLFVNQMASALFR
FIGALGRELTVAFTIGSFVLAILIAMSGFILSKGNMKKWWLWGFWSSPMMYGLNAMINNEFQGKRWRHVLPN
STTPLGVQVLKSRGFFTQSKWYWIGVGALIGYTIVFNIAYILALTYLNREFLHLKKKMFCNQMREANMCTNIIT
NRLAAIVQHQAVKSEKSQSNEQDGGSTSARSSSRRKEADRRRGMALPFEPHSITFDDVTYSVDMPQEMKNQG
VLEDRLNLLKGVSGTFRPGVLTALMGSTGAGKTTLMDVLAGRKTGGYIGGNITISGYPKKQETFARISGYCEQ
NDIHSPYVTVYESLLYSAWLRLSAEINSETRKMFIEEVIELVELNPLKHTIVGLPGVNGLSTEQRKRLTISVELVA
NPSIIFMDEPTSGLDARAAAVVMRAIRKIVDTGRTVVCTIHQPSIDIFESFDELFLMKRGGQEIYVGPLGHHSYH
LISYFEGIKGVRTIEDGYNPATWMLEVTTSAKEMELGIDFAELYKNSDLYRRNKELIEELSTPAPGSKDLYFSSK
YSRSFITQCMACLWKQHWSYWRNNEYTALRFLFTIAVALLFGSIYWNLGSKIKKQQDLFNAMGSMYAAVLLL
GIKNSNSAQPLVAVERTVFYREKAAGMYSALAYAFAQVVVELPHVLLQTVVYSAIVYAMIGFEWSVTKFFWY
LFFMYFTFLYFTYYGMMSAAMTPNPSLAVIISSGFYEVWNLFSGFIIPRPRMPVWWRWYYWANPVAWTLYGL
VTSQFGDIQDHIEFNGRSTTVEDFLRNYFGFKHDFLGVVAAVLIGFAVTFALIFAIAIKMLNFQRR

>XP_015649181.1_ABCD1_Os
MPSLQLLQLTEHGRNLLSSRRRTLAVVSGALLAGGTLAYAHSARRQKRQEEYSHSDASTQTTGNQSICQNGVD
GKLVKTRKKKNGLKSLQFLAAILLKKIGPNGINHLLGLMITAVLRTAVGHRLAKVQGYLFRVAFLRRVPTFTR
LIIENLLLCFLQSTIYQTSKYLTGSLGLHFKKILTDLVHADYFENMVYYKLSHVDHRISNPEQRIASDIPKFCSELS
GLVQDDLTAVADGLIYIWRLCSYASPKYVLWILAYVLGAGGAIRKFSPAFGKLKSMEQQLEGEYRQVHSRLRT
HAESVAFYGGENREASHIMQRFQALVKHLNVVLHENWWFGMIQDFLLKYLGATVGVILIVEPFFAGNLKPESS
TLGRAEMLSNLRYHTSVIISLFQSLGTLSISSRRLNLLSGYADRIRELLDVSRELSGVRDLSMNKKSSVDNYISEA
NYIEFSGVKVVTPSGNVLVDDLTLRVESGSNLLITGPNGSGKSSLFRVLGGLWPLMSGHIVKPGVGSNLNKEIF
YVPQRPYTAVGTLRDQLIYPLTADQETEPLSYGGMVDLLKNVDLEYLLERYPLDKEVNWGDELSLGEQQRLG
MARLFYHRPKFAILDECTSAVTTDMEERFCKRVQAMGTSCITISHRPALVAFHDIVLSLDGEGGWTVQENRNG
SFISAEPEFDALNSSETDRKSDALAVQRAFIANTKGNALMGPKDHSYSTQLIATSPNMEIEHTERSNLVPQLQCS
PRPLPLRAAAMSKILVPKLFDKQGGQLLAVALLVFSRTWISDRIASLNGTSVKYVLEQDKAAFLRLIGISVLQSA
ANSIVSPSLRNLTSKIALGWRIRMTNHLLQYYLKRNAFYKVFNMSGIDIDADQRITHDVEKLTNDLAGLVTGM
VKPLVDILWFTWRMKILSGRRGVAILYAYMLLGLGFLRAVSPDFGDLANQEQELEGTFRFMHSRLRTHAESIA
FFGGGSREKAMVEAKFTTMLNHSRTLLRKRWLYGIFDDFVTKQLPHNVTWGLSLLYALEHKGDRALTSTQGE
LAHALRFLASVVSQSFIAFGDILELHKKFLELSGGINRVFELEELLQTSQSNAAMPSNPIIAASEEIISFHDVDIVTP
SQKLLATQLSCDVSQGKSLLVTGPNGSGKSSIFRVLRGLWPIASGRLTMPSDGIFHVPQRPYTCLGTLRDQIIYP
LSHEEAELKVLSLYKSGDKAITSGSLDDHLKTILENVRLVYLLEREGWDATPNWEDILSLGEQQRLGMARLFF
HCPKFGILDECTNATSVDVEEHLYKIATSMGITVITSSQRPALIPFHSLELKLIDGEGKWELCTINQ

>NP_001335399.1_P_Zm
MSSLQLLQLTERGRNLLSSRRRTLAIVSGAVLAGGALAYAQSGRWKKHQEANSCSDANSHSSNNGRTSQNGI
DGKLVKTRKKKSGLKSLHFLAAILLKKIGPNGTNYLIGLILTAVLRTAVGHRLAKVQGYLFRSAFLRRIPTFTRL
IIENLLLCFLQSTLYQTSKYLTGSLGLRFKKILTDLVHADYFENMVYYKISHVDHRITNPEQRIASDIPKFCSELS
DLVQDDLAAIVEGLIYIWRLCSYASPKYVLWILAYVIGAGGTIRKFSPAFGKLKSTEQQLEGEYRQLHSRLRTH
AESVAFYGGENREASHIKQRFRALVKHLNVVLHENWWFGMIQDFLLKYLGATVGVILIIEPFFAGNLKPESSTL
GRAEMLSNLRYHTSVIISLFQSLGTISISSRRLNILSGYANRIHELLDVSRELSGGRDRLITQNSSDGNYISEANYIE
FSDVKVVTPSGNVLVDNLNLHLESGSNLLITGPNGSGKSSLFRVLGGLWPMVSGHIVKPGVGSNLNKEIFYVPQ
RPYTAVGTLRDQLIYPLTADQETEPLSYGGMVDLLKNVDLEYLLERYPLDKEVNWGDELSLGEQQRLGMARL
FYHKPKFAILDECTSAVTTDMEERFCKRVRAMGTSCITISHRPALVAFHEIVLSLDGEGGWDIQDNRNGSFSPE
VEFDVLKASESDRKSDALTVQRAFITSTKGNASMKPKKHSYSTEVIASSPSMEIEHTVQSSIVTQLQCSPRPLPV
RVAAMSQILVPKLFDKQGGQLLAVVLLVFSRTWISDRIASLNGTSVKYVLEQDKAAFIRLAGTSVLQSAANSIV
SPSLRNLTSRIALGWRIRMTNHLLQYYLKRNAFYKVFNISGMSMDADQRMTHDVEKLTNDLAGLLTGMVKPL
VDIIWFTWRMKLLSGRRGVAILYAYMFLGLGFLRAVSPDFGDLANQEQELEGTFRFMHSRLRTHAESIAFFGG
GSREKAMIDAKFMTLLNHSKVLLRKKWLYGIFDDFVTKQLPHNVTWGLSMLYALEHKGDRALTSTQGELAH
ALRFLASVVSQSFIAFGDILELHKKFLELSGGINRIFELEELLQASQSNPVMPSDATNAGSEEIISFRGVNIVTPSQ
KLLASQLSCDVSQGKSLLVTGPNGSGKSSIFRVLRGLWPIASGSLSKPSEGIFNVPQRPYTCLGTLRDQIIYPLSH
EEAKLKMLSGETSDKSTASELLDDHLRTILENVRLLYLLEREGWDATTNWEDTLSLGEQQRLGMARLFFHCPK
YGILDECTNATSVDVEEHLYRLATNMGITVITSSQRPALIPFHSLELKLIDGEGKWELCSIHQ

>XP_002457002.2_ABCD1_Sb
MSSLQLLQLTERGRNLLSSRRRTIAIVSGAVLAGGTLAYAQSGRWKKHQEVNSCSDANSHSSNNGRTSQNGID
GKLVKTRKKKSGLKSLHFLAAILLKKIGPNGTNYLIGLILTAVLRTAVGHRLAKVQGYLFRSAFLRRIPTFTRLII
ENLFLCFLQSTLYQTSKYLTGSLGLHFKKILTDLVHADYFENMVYYKISHVDHRISNPEQRIASDIPKFCSELSDL
VQDDLAAIIEGLIYIWRLCSYASPKYVLWILAYVLGAGGTIRKFSPAFGKLKSMEQQLEGEYRQLHSRLRTHAE
SVAFYGGENREASHIKQRFRALVKHLNVVLHENWWFGMIQDFLLKYLGATVGVILIIEPFFAGNLKPESSTLGR
AEMLSNLRYHTSVIISLFQSLGTLSIGSRRLNILSGYANRIHELLDVSRELSGGRDRLITQNSSDGNYISEANYIEF
SGVKVVTPSGNVLVDNLNLHLESGSNLLITGPNGSGKSSLFRVLGGLWPMVSGHIVKPGVGSNLNKEIFYVPQ
RPYTAVGTLRDQLIYPLTADQETEPLSYGGMVDLLKNVDLEYLLERYPLDKEVNWGDELSLGEQQRLGMARL
FYHKPKFAILDECTSAVTTDMEERFCKRVRAMGTSCITISHRPALVAFHEIVLSLDGEGGWNIQDNRNGSSFSPE
LEFDVLESSESDRKSDALTVQRAFITSTKGNSSMKPKKLSYSTEVIASSPIVEIEHTVQSSIVTQLQCSPRPLPVRV
AAMSQILVPKLFDKQGGQLLAVALLVFSRTWISDRIASLNGTSVKYVLEQDKAAFIRLAGISVLQSAANSIVSPS
LRNLTSRIALGWRIRMTNHLLQYYLKRNAFYKVFNISGMSMDADQRMTHDVEKLTNDLAGLLTGMVKPLVDI

IWFTWRMKLLSGRRGVAILYAYMFLGLGFLRAVSPDFGDLANKEQELEGTFRFMHSRLRTHAESIAFFGGGSR
EKAMIDAKFTTLLNHSKVLLRKKWLYGIFDDFVTKQLPHNVTWGLSMLYALEHKGDRALTSTQGELAHALRF
LASVVSQSFIAFGDILELHKKFLELSGGINRIFELEELLQASQSNPVVPSDAINATSEEIISFRGVDIVTPSQKLLAS
QLSCDVSQGKSLLVTGPNGSGKSSIFRVLRGLWPIASGRLTKPSEGIFNVPQRPYTCLGTLRDQVIYPLSHEEAK
LKMLSYETSDKSTASKMLDDHLKTILENVRLVYLLEREGWDATPNWEDILSLGEQQRLGMARLFFHCPKYGIL
DECTNATSVDVEEHLYRLATNMGITVITSSQRPALIPFHSLELKLIDGEGKWELCLALLREQDEQALWADEDLA
EEADESKPDDGRDVDAAERRDEPAGDGEERLRGMKRKPNADSTGPATPATAFAVAGSRSPSTATARYRVSTS
DGPDSTTPAAESIREDGRGHGSKEASDLGGGRDGEAPPSPSGSSGGEDGRDGPGHGRLGSYEQGRLHLHERQW
WKQQLQLLPLPLLGLLVFEVKPKANPTAQQSKHQTPSPSRPFPRVATGLTPPSPPMLSRMLPRLRSSARVCGQE
PAAAARRLSAAEAFAECEGSLLRPLPGLDLPPCLPDNLSRSPTRVTTLPNGLRIASEDIPGPSACIGFFVNSGSVY
ESGETTGVSHMLERMAFKDTKHRSHLNIVHELELAGGNVGASASREQMVYSYDTLKGYMPEALEILIDCMRN
PLFLQEEVERQLVLAREEVQELQKNPERFLHEQLNLVGFSGALANPLIAPEDALARINDKIIQKFYSENFTADRV
VLAASGVDHEHLLGYADLLLKDWHKGTPIEKPKSTYVGGDSRHKADSDMTHVALAFEVPGGWLQERDATIM
TVIQTLMGGGGSFSSGGPGKGMHSRLYLRVLNKYHSVESFSAFSNVYDSSGLFGIYLTTPSDFVAKAVDIAISEL
VAVATPGEVTEVELQRAKNSTISSVLMNLESRVVVAEDIGRQMLSYGCRKPIDYFLQCMEEITLDDVATFARK
MLASQPTMVSWGNVDKVPPYEFICKRLQ

>XP_015640039.1_ABCD1_Os
MPSLQLLQLTERGRGLLASRRKTLAVVSGALLAGGALAYARSSQGQRRRRSEGDDATTALARNGDRMGQNG
VDGRLAGTKRRKGGLRSLHFLAAILLKKIGPNGTRYLLGLTLTAVLRTAVGHRLARVQGYLFRAAFLRRVPTF
TRLIIENLILCFLQSAVYQTSKYLTGSLSLRFKKILTDLVHADYFQNMVYYKISHVDHRISNPEQRIASDIPKFCSE
LSELVQDDLAAVAEGLIYTWRLCSYASPKYMLWIVGYILVAGGAIRNFSPAFGKLKSTEQQLEGDYRQLHSRL
RTHAESVAFYGGENREAYYIMQRFQALIGHLNCVLHENWWFGMIQDFFLKYFGATVAVVLIIEPFFSGHLRPD
SSTLGRAEMLSNLRYHTSVIISLFQSLGILSISTRRLNILSGYADRIRELLDVSRELSGVRDKSLNHNSSAGNYISE
ANHIEFSGVKVVTPASNVLVDDLTLRVERGSNLLITGPNGSGKSSLFRVLGGLWPLVSGHIVKPGVGSDLNKEI
FYVPQRPYTAVGTLREQLIYPLTADQEIEPLSYDGMVDLLKNVDLEYLLERYPLDKEVNWGDELSLGEQQRLG
MARLFYHKPKFAILDECTSAVTIDMEERFCKKVRAMGTSCITISHRPALVAFHDIVLSLDGEGGWDVQHRRDD
SSFSTEESDYTLLETDRKSDALTVQRAFMGRAKSNASSRSKEHCYTTKVIATSPKLEIEQTIQTHRVPHLRCFPR
PLPARVAAMVKILVPKLLDKQGGQLLAVALLVFSRTWISDRIASLNGTTVKFVLEQDKAAFIRLIGISVLQSSAN
SFVAPSLRTLTGRLALGWRIRLTNHLLQYYLKRNAFYKVFNMSGKSIDADQRLTLDVDKLTTDLAGLVTGMV
KPLVDILWFTWRMKLLSGRRGVAILYAYMLLGLGFLRAVSPDFGHLAGQEQELEGTFRFMHSRLRTHAESIAF
FGGGSREKAIVEAKFMKLLDHSKILLRKQWLYGIVDDFVTKQLPHNVTWGLSLLYALEHKGDRALTSTQGEL
AHALRFLASVVSQSFIAFGDILDLHKKFLELSGGINRIFELEELLRVSQRDTFVPSDATSAEETISFHEVDIVTPSQ
KLLASKLSCNVVQGKSLLLTGPNGSGKSSIFRVLRDLWPVCSGRVTKPSDGMFHVPQRPYTSLGTLRDQIIYPL
SREEAEMKICSLYNDGNGSSASNLLDDHLKTILVNVRLVYLLEREGWDSTSNWEDVLSLGEQQRLGMARLFF
HHPKFGILDECTNATSVDVEEHLYKLATSMGITVITSSQRPALIPFHSLELKLIDGEGNWELCEIHQ

>XP_021302707.1_ABCD1_Sb
MPSLQLLQLTDRGRGLLASRRRTLAVVSGAVLAGGALAYARSSQSRRRRRSEANHGGETSALATNGDGAGGG
LAAAKQKRSGLKSLHFLAAILLKKIGPSGTRFLLGLVLTAVLRTAVGHRLARVQGFLFKAAFLRRVPTFTRLIIE
NLMLCFLQSTLYQTSKYLTGSLSLRFKKILTDIAHTDYFENMVYYKISHVDHRISNPEQRIASDIPKFSSELSELV
QDDLAAVAEGLIYTWRLCSYASPKYVFWIMAYVLVAGGVIRNFSPAFGKLKSTEQQLEGDYRQLHSRLRTHA
ESVAFYGGENREASHIMQRFEALVEHLNLVRHENWWFGMIQDFFLKYFGATVAVVLIIEPFFSGNLRPDSSTLG
RAEMLSNLRYHTSVIISLFQSLGILSISTRRLNILSGYADRIRELLDVSRELSGVCDRSLNHNSPGNYVSEANHIEF
SDVKVVTPAGNVLVDDLTLRVETGSNLLITGPNGSGKSSLFRVLGGLWPLVSGYIVKPGVGSDLNKEIFYVPQR
PYTAVGTLREQLIYPLTADQEIEPLTYDGMVDLLKNVDLEYLLERYPLDKEVNWGDELSLGEQQRLGMARLF
YHKPKFAILDECTSAVTTDMEERFCKKVRAMGTSCITISHRPALVAFHDIVLSLDGEGGWNVQHRRDDSSFSTE
ESDFSSSETDRKSDALTVQRAFMSRAKSNASLGSKDHSYCTEVIATSAKVEIEHAARRIPHLRCHPRPLPLRVAA
MLKILVPRLFDKQGGQLLAVAVLVFSRTWISDRIASLNGTTVKFVLEQDKAAFIRLIGVSILQSGANSFVAPSLR
TLTAKLALGWRIRMTNHLLRYYLKRNAFYKVFNMSGKSIDADQRLTLDVDKLTTDLAGLVTGMVKPLVDIL
WFTWRMKLLSGRRGVAILYAYMLLGLGFLRAISPDFGHLSGQEQELEGTFRFMHSRLRTHAESIAFFGGGSRE
KAMVEAKFVKLINHSKILLRKQWLYGIVDDFVTKQLPHNVTWGLSLLYALEHKGDRALTSTQGELAHALRFL
ASVVSQSFIAFGDILELHKKFLELSGGINRIFELEEFTRFAQRNTAVSPNAISAASEEIISFHEVDIVTPSQKLLARK
LSCNVVQGKSLLLTGPNGSGKSSIFRVLRDLWPTFSGRVTKPSEGMFHVPQRPYTSLGTLRDQIIYPLSREEAKI

KVLSLHRSGNNSSASVLLDDHLKTILENVRLLYLLEREGWDSTPNWEDVLSLGEQQRLGMARLFFHHPKFGIL
DECTNATSVDVEEHLYRLATSMGITVITSSQRPALIPFHALELKLIDGEGNWELCAIQQ

>XP_006591509.1_ABCD1_Gm
MSSLQLLQLTRRGQSFLASRRRTLLLASGILIAGGTAAYVQSRFRVNKHDLFGHCNGHNNDKEVTEEEVVKGV
SAPKKKQKKGLKSLQVLAAILLSGMGKFGARDLLGLVVIAVLRTALSNRLAKVQGFLFRAAFLRRVPLFLRLIS
ENILLCFLLSTIHSTSKYITGTLSLHFRKILTKLIHSHYFENMVYYKISHVDGRITNPEQRIASDVPRFCSELSEIVQ
DDLTAVTDGLLYTWRLCSYASPKYVVWILVYVLGAGAAIRNFSPSFGKLMSKEQQLEGEYRQLHARLRTHSE
SIAFYGGERKEETHIQQKFKTLVRHMYSVLHDHWWFGMIQDLLLKYLGATVAVILIIEPFFSGHLRPDSSTLGR
ADMLSNLRYHTSVIISLFQSLGTLSISARRLNRLSGYADRIYELMAVSRELSLVNEKSSLQRNASRNCIREANYIE
FDGVKVVTPTGNVLVDDLTLRVESGSNLLITGPNGSGKSSLFRVLGGLWPLISGHIVKPGIGSDLNNEIFYVPQR
PYTAVGTLRDQLIYPLTEDQEIEPLTDRGMVELLKNVDLEYLLDRYPPEREVNWGDELSLGEQQRLGMARLFY
HKPKFAILDECTSAVTTDMEERFCAKVRAMGTSCITISHRPALVAFHDVVLSLDGEGGWSVHYKREGSSTEVGI
DTMKASETKRQSDAKAVQRAFSMSKKDSAFSNPKAQSYFAEVISSSPSMNHTIPPSVVPQLHCNTRVLPLRVA
AMCKVLVPTVLDKQGAQLLAVAFLVVSRTWVSDRIASLNGTTVKFVLEQDKASFIRLIGLSVLQSVASSFIAPSI
RHLTARLALGWRVRLTQHLLKNYLRNNAFYKVFHMANKNIDADQRITHDLEKLTADLSGLVTGMVKPSVDIL
WFTWRMKLLTGQRGVAILYAYMLLGLGFLRTVTPDFGNLISQEQQLEGTFRFMHERLCTHAESVAFFGGGAR
EKAMVESRFRELLSHSKYLLKKKWLFGILDDFITKQLPHNVTWLLSLLYAMEHKGDRASISTQGELAHALRFL
ASVVSQSFLAFGDILELHRKFVELSGGINRIFELEELLDASQSGDSINSSITSPIWDYHGKDAISFCMVDIVTPTQK
MLARELTCDIEFGKSLLVTGPNGSGKSSIFRVLRGLWPIASGRLSRPSEDVDLEAGSGCGIFYVPQRPYTCLGTL
RDQIIYPLSREEAQFQALKMHGKGEKHPDLRIMLDTHLQVILENVRLNYLLERDNNGWDANLNWEDILSLGEQ
QRLGMARLFFHKPKFGILDECTNATSVDVEEHLYGLANKMGITVVTSSQRPALIPFHSMELHLIDGEGNWELRS
IKQ

>XP_006355351.1_PREDICTED:_ABCD1_St
MPSLQLLQLTEHGRGLLASKRKALLLTTGIIVAGGTAAAYMQSRRTYKGHDSTQCDGVNDGIIEPNNQTGKGN
NVKKSRQKKGGLKSVKVLAAILLSRMGRMGTRDLLALVATVVLRTAVSNRLAKVQGFLFRAAFLRRVPMFF
RLILENILLCFLQSALHSTSKYITGTLSLRFRSILTRLIHAQYFQDMVYYKLSHVDGRITNPEQRIASDVPKFSREL
SDLVQEDLIAVTDGLLYTWRLCSYASPKYLFWILAYVLGAGLTIRNFSPPFGKLISKEQQLEGEYRQLHSRLRT
HAESIAFYGGETREDFHIQQKFKTLVRHMKAVLHEHWWFGMIQDFLHKYLGATVAVVLIIEPFFSGNLRPDAS
TLGRAEMLSNLRYHTSVIISLFQALGTLAISSRRLNRLSGYADRIHELMIISRDLGGRNASSIQSNGSSNYVTEAN
YIEFDGVKVVTPTGNVLVEDLSLRVESGSNLLITGPNGSGKSSLFRVLGGLWPLVSGHIVKPGIGSDLNKEIFYV
PQRPYTAIGTLRDQIIYPLTADQEVEPLTRSGMVELLKNVDLEYLLDRYPPEKEVNWGEELSLGEQQRLGMAR
LFYHKPKFAILDECTSAVTTDMEERFCSKVRAMGTSCITISHRPALVAFHDVVLSLDGEGGWRVHYKRAEAPS
LTDSEFNKNQHNETDRQSDAMTVQRAFATAKKGTKFSKSEAELYFSELISASPSEADEPPLHVFPHLKSVPRKL
PLRIAAMSKVLVPRLLDKQGAQFLAVALLVVSRTWVSDRIASLNGTTVKFVLEQDKAAFLRLIFVSVLQSAASS
FIAPSLRHLTQTLALGWRIRLTKHLLKNYLRNNAYYKVFNMSGVNLDADQRLTQDLEKLTADLSSLVTGMVK
PTVDILWFTWRMKLLTGQRGVAILYAYMLLGLGFLRCVTPDFGDLASREQQLEGTFRFMHERLRTHAESVAFF
GGGAREKEMVEARFKELLHHSSLLLKKKWLFGIIDEFITKQLPHNVTWGLSLLYAMEHKGDRALTSTQGELAH
ALRFLASVVSQSFLAFGDILELHKKFVELSGGINRIFELEEFLDAAQYDLPEGVSSSPSSEDVISFSEVDIITPGQKI
LARKLTCDIVKGKSLLVTGPNGSGKSSIFRVLRGLWPVVSGKLVKPCQPLNTELGSGIFYVPQRPYTCLGTLRD
QIIYPLSHEVAEKRVQAMREGLRHLGSSNILDSHLQSILEDVKLVYLLEREGGWDANQNWEDILSLGEQQRLG
MARLFFHKPRFGILDECTNATSVDVEEHLYRLAKDAGITVVTSSQRPALIPFHSAELRLIDGEGKWQLRSIKMD
EEGEGEPNEYTQQL

>XP_004237396.1_ABCD1_Sl
MPSLQLLQLTEHGRGLLASKRKALLLTTGIIVAGGTAAAYMQSRKTYKGHDSLQCDGVNDGIIEPNKQTRKGN
NVKKSRQKKGGLKSVKVLAAILLSRMGRMGTRDLLALVATVVLRTAVSNRLAKVQGFLFRSAFLRRVPMFFR
LILENILLCFLQSALHSTSKYITGTLSLRFRSILTRLIHAQYFQDMVYYKLSHVDGRIANPEQRIASDVPRFSRELS
DLVQEDLIAVTDGLLYTWRLCSYASPKYLFWILAYVLGAGLTIRNFSPPFGKLMSKEQQLEGEYRQLHSRLRT
HAESIAFYGGETREDFHIQQKFKTLVRHMKAVLHEHWWFGMIQDFLHKYLGATVAVVLIIEPFFSGNLRPDAS
TLGRAEMLSNLRYHTSVIISLFQALGTLAISSRRLNRLSGYADRIHELMIISRDLGGRNASSIQSNGSGNYVTEAN
YIEFDGVKVVTPTGNVLVEDLSLRVESGSNLLITGPNGSGKSSLFRVLGGLWPLVSGHIVKPGIGSDLNKEIFYV
PQRPYTAIGTLRDQIIYPLTADQEVEPLTRIGMVELLKNVDLEYLLDRYPPEKEVNWGEELSLGEQQRLGMARL

FYHKPKFAILDECTSAVTTDMEERFCSKVRAMGTSCITISHRPALVAFHDVVLSLDGEGGWRVHYKRAEAPSL
TDSEFNKNQCNETDRQSDAMTVQRAFATAKKSTKFSKSEAELYFSELISASPSEADESPLHVFPHLKSVPRKLP
QRIAAMSKVLVPRLLDKQGAQFLAVALLVVSRTWVSDRIASLNGTTVKFVLEQDKAAFLRLIFISVLQSAASSF
IAPSLRHLTQTLALGWRIRLTKHLLKNYLRNNAYYKVFNMSGVNLDADQRLTQDLEKLTADLSSLVTGMVKP
TVDILWFTWRMKMLTGQRGVAILYAYMLLGLGFLRCVTPDFGELASREQQLEGTFRFMHERLRTHAESVAFF
GGGAREKEMVEARFKELLHHSSLLLKKKWLFGIIDEFITKQLPHNVTWGLSLLYAMEHKGDRALTSTQGELAH
ALRFLASVVSQSFLAFGDILELHKKFVELSGGINRIFELEEFLDAAQYDVPEGVSSSPSSEDVISFSEVDIITPGQK
VLARKLTCDIVKGKSLLVTGPNGSGKSSIFRVLRGLWPVVSGNLVKPGQPLNSELGSGIFYVPQRPYTCLGTLR
DQITYPLSHEVAEKRVQAMREGLRHLGSSNILDSHLQSILEDVKLVYLLEREGGWDANQNWEDILSLGEQQRL
GMARLFFHKPRFGILDECTNATSVDVEEHLYRLAKDAGITVVTSSQRPALIPFHSVELRLIDGEGKWQLRSIKM
DEEGEDEPNEYTQQL

>XP_025984277.1_ABCD1-l_X1_Gm
MSSLQLFQLTHHGRSFLASRRKTLLLATGILVAGGTAAYVQSRFRGNRDDLLGDSYERNNDKELTKEEVMKG
TSAPKNKQKKGGLKSLQVLAAILLSEMGQLGAKNLLSLVSIVVLRTTLSNRLAKVQGFLFRAAFLRRVPLFLRL
ISENILLCFLLSTMQSTSKYITGTLSLHFRKILTKLIHSRYFENMVYYKISHVDGRITNPEQRIASDVPRFCSELSEI
VQDDLTAVTDGLLYTWRLCSYASPKYIFWILAYVLGAGATIRNFSPAFGKLMSREQELEGEYRQLHSRLRTHS
ESIAFYGGERREEAHIQQKFRTLVRHINRVLHDHWWFGMIQDFLLKYLGATVAVILIIEPFFSGHLRPDSSTLGR
AEMLSNLRYHTSVIISLFQSLGTLSISARRLNRLSGYADRIHELMAISRELSLDNGKSSLQRQGSRNYISEANYVG
FYGVKVVTPTGNVLVDDLTLKVQSGSNLLITGPNGSGKSSLFRVLGGLWPLVSGHIVKPGVGSDLNKEIFYVPQ
RPYTAVGTLRDQLIYPLTADQEVEPLTDSRMVELLKNVDLEYLLDRYPSETEVNWGDELSLGEQQRLGMARL
FYHKPKFAILDECTSAVTTDMEERFCANVLAMGTSCITISHRPALVAFHDVVLSLDGEGGWSVHHRREDSSTEL
GNDTVKALETKRQSDAKAVQRAFAMNKKGSAFSNSKAQSDISEVIIASSPSMKRNISPSAVPQLHGNTRALPM
RVAAMCKVLVPTIFDKQGARLLAVAFLVVSRTWVSDRIASLNGTTVKLVLEQDKASFIRLIGISVIQSAASSFIA
PSIRHLTARLALGGRIRLTQHLLKNYLRNNAFYKVFHMASKNVDADQRITHDLEKLTTDLSGLVTGMVKPSVD
ILWFTWRMKLLTGRRGVAILYAYMLLGLGFLRTVTPDFGDLISQEQQLEGTFRFMHERLCTHAESVAFFGGGA
REKAMVESRFRELLLHSKYLLKKKWLFGILDDFITKQLPHNVTWGLSLIYAMEHKGDRASVTTQGKIFALNHL
LSIFLLLLSSLTDNTILIGELAHALRFLASVVSQSFLAFGDILELHRKFVELSGGINRIFELEELLDAAQSENFTSVS
AIPPVRDVHSSDVISFSKVDIVTPSQKMLARELIFDIKHGGSLLVTGPNGSGKSSIFRVLRGLWPIASGRLSRPSEV
VDEEDGSGCGIFYVPQRPYTCLGTLRDQIIYPLSCEEAEVKVLKMYGKDEKHADTRNLLDTRLKAILESVRLNY
LLEREGSNWDANLKWEDILSLGEQQRLGMARLFFHKPKFGILDECTNATSVDVEEHLYGLAKDMGITVVTSSQ
RPALIPFHSMELRLIDGEGNWKLRLIKQ

>XP_014631244.1_ABCD1-l_X2_Gm
MSSLQLFQLTHHGRSFLASRRKTLLLATGILVAGGTAAYVQSRFRGNRDDLLGDSYERNNDKELTKEEVMKG
TSAPKNKQKKGGLKSLQVLAAILLSEMGQLGAKNLLSLVSIVVLRTTLSNRLAKVQGFLFRAAFLRRVPLFLRL
ISENILLCFLLSTMQSTSKYITGTLSLHFRKILTKLIHSRYFENMVYYKISHVDGRITNPEQRIASDVPRFCSELSEI
VQDDLTAVTDGLLYTWRLCSYASPKYIFWILAYVLGAGATIRNFSPAFGKLMSREQELEGEYRQLHSRLRTHS
ESIAFYGGERREEAHIQQKFRTLVRHINRVLHDHWWFGMIQDFLLKYLGATVAVILIIEPFFSGHLRPDSSTLGR
AEMLSNLRYHTSVIISLFQSLGTLSISARRLNRLSGYADRIHELMAISRELSLDNGKSSLQRQGSRNYISEANYVG
FYGVKVVTPTGNVLVDDLTLKVQSGSNLLITGPNGSGKSSLFRVLGGLWPLVSGHIVKPGVGSDLNKEIFYVPQ
RPYTAVGTLRDQLIYPLTADQEVEPLTDSRMVELLKNVDLEYLLDRYPSETEVNWGDELSLGEQQRLGMARL
FYHKPKFAILDECTSAVTTDMEERFCANVLAMGTSCITISHRPALVAFHDVVLSLDGEGGWSVHHRREDSSTEL
GNDTVKALETKRQSDAKAVQRAFAMNKKGSAFSNSKAQSDISEVIIASSPSMKRNISPSAVPQLHGNTRALPM
RVAAMCKVLVPTIFDKQGARLLAVAFLVVSRTWVSDRIASLNGTTVKLVLEQDKASFIRLIGISVIQSAASSFIA
PSIRHLTARLALGGRIRLTQHLLKNYLRNNAFYKVFHMASKNVDADQRITHDLEKLTTDLSGLVTGMVKPSVD
ILWFTWRMKLLTGRRGVAILYAYMLLGLGFLRTVTPDFGDLISQEQQLEGTFRFMHERLCTHAESVAFFGGGA
REKAMVESRFRELLLHSKYLLKKKWLFGILDDFITKQLPHNVTWGLSLIYAMEHKGDRASVTTQGELAHALRF
LASVVSQSFLAFGDILELHRKFVELSGGINRIFELEELLDAAQSENFTSVSAIPPVRDVHSSDVISFSKVDIVTPSQ
KMLARELIFDIKHGGSLLVTGPNGSGKSSIFRVLRGLWPIASGRLSRPSEVVDEEDGSGCGIFYVPQRPYTCLGT
LRDQIIYPLSCEEAEVKVLKMYGKDEKHADTRNLLDTRLKAILESVRLNYLLEREGSNWDANLKWEDILSLGE
QQRLGMARLFFHKPKFGILDECTNATSVDVEEHLYGLAKDMGITVVTSSQRPALIPFHSMELRLIDGEGNWKL
RLIKQ

>XP_021616683.1_ABCD1-l_Me
MPSLQLLQLTEHGRSLLASRRKSLLLAAGILVVGGTTAYVKSRNSSKKFDSFGHYNGLREDNDDSDERVTKEV
KKTIKKKGSLKSLHVLASVLLSEMGKRGTSDLFAMIAIAVMRTALSNRLAKVQGFLFRAAFLRRVPLFFRLISE
NILLCFLLSTIHSTSKYVTGTLSLCFRRILTKRIHSHYFENMAYYKISHVDGRITNPEQRIASDVPRFCSELSELVQ
EDLTAVTDGLLYTWRLCSYASPKYIFWILAYVLGAGTMIRNFSPAFGKLMSKEQQLEGEYRQLHSRLRTHAESI
AFYGGERREESHIQQKFKDLVRHMRIVLHDHWWFGMIQDFLVKYLGATVAVVLIIEPFFAGNLRPDASTLGRA
TMLSNLRYHTSVIISLFQSLGTLSISSRRLNRLSGYADRIHELIVISRELNCEDKTSLQRSGSRNYFSEADYVEFSK
VKVVTPTRNVLVEDLTLKVESGSNLLITGPNGSGKSSLFRVLGGLWPLVSGHIVKPGVGSDLNKEIFYVPQRPY
TAVGTLRDQLIYPLTVDQEVEPLTQSGMLELLKNVDLEYLLDRYPPEQEVNWGEELSLGEQQRLGMARLFYH
KPKFAILDECTSAVTTDMEERFCAKVRAMGTSCITISHRPALVAFHDVVLSLDGEGGWQVSYKRRDSAALTEP
GTNDTSACETERKSDAMLVQRAFSTSNKDSAFSNSKAQSYISEVIVAFHGADPGLQLPIVPQLQRAPRALALRV
AAMSKILVPTVLDKQGAHLLAVAFLVVSRTWVSDRIASLNGTTVKFVLEQDKTSFIRLIGVSILQSAASSIIAPSL
RHLTARLALGWRIRLTQHLLRSYLRNNAFYKVFHLSSKNIDADQRITDDLEKLTRDLSGLVTGMVKPSVDILW
FTWRMKLLTGRRGVAILYAYMLLGLGFLRTVTPDFGDLASREQQLEGTFRFMHERLRTHAESVAFFGGGARE
KAMIESRFRELLDHSLLLLKKKWLYGILDDFVTKQLPHNVTWGLSLLYAMEHKGDRAQISTQGELAHALRFL
ASVVSQSFLAFGDILELHKKFLELSGSINRIFELEELLDAAQSGDWLTEKLSPFKESDSDGQDAISFVDVDIITPA
QKLLARQLRCDVVQGKSLLVTGPNGSGKSSVFRVLRGLWPIVSGRLSKPAHSNEDSEYGCGIFYVPQRPYTCL
GTLRDQIIYPLSRDEAVRMTLKLHGRGKISGDTTKLLDTRLKAILENVRLNYLLEREEGGWDANLNWEDILSLG
EQQRLGMARLFFHKPKFGILDECTNATSVDVEEQLYRLATDMNITFVTSSQRPALIPFHSVELRLIDGEGNWEL
RTIKQSLDSDHLLS

>XP_003601966.2_ABCD1_Mt
MPSLQLLKFTRHGQNFLASRRKAILLASGILIAGGTAAYMQSRFRVNKHDLFGHCNEQNNDKEVKKEEVINDS
TKPKNKQKKGGMKSLQVLTAILLSDMGQLGVKNLLALVVTVVLRTALSNRLAKVQGFLFRAAFLRRAPLFFR
LISENIILCFLLSTIHSTSKYITGTLSLHFRKVLTKLIHSHYFENMVYYKISHVDGRITNPEQRIASDVPKFCSELSEI
VQDDLAAVTDGLLYTWRLCSYASPKYVFWILAYVLGAGAAIRNFSPPFGKLMSTEQQLEGDYRQLHSRLRTH
SESIAFYGGERREEAHIQHKFKTLVRHMRRVLHDHWWFGMIQDFLLKYLGATVAVILIIEPFFSGNLRPDSSTL
GRAEMLSNLRYHTSVIISLFQSLGTLSIGARRLNRLSGYADRIYELMAVSRELSLVDEKSSLQRQGSRNCISEAN
YIEFSNVKVVTPTGNVLVDDLSLRVEQGSNLLITGPNGSGKSSLFRVLGGLWPLISGHIVKPGIGSDLNKEIFYVP
QRPYTAVGTLRDQLIYPLTSNQEVEPLTDHGMVELLKNVDLEYLLDRYLPEKEVNWGDELSLGEQQRLGMAR
LFYHKPKFAILDECTSAVTTDMEERFCAKVRAMGTSCITISHRPALVAFHDVVLSLDGEGGWSVHYRREDSST
EMGIDTMKASETKRQTDAKAVQRAFAMSKKDSAFSSSKAESYIADVIYSSPSTNHTNLPSTVPQLHGNTRILPL
RVAAMFKVLVPTVFDKQGAQLLAVALLVVSRTWVSDRIASLNGTTVKFVLEQDKAAFIRLIGISVLQSAASSFI
APSIRHLTARLALGWRIRLTQHLLKNYLRSNVFYKVFHMASKSVDADQRITQDLEKLTTDLSGLVTGLVKPSV
DILWFTWRMKLLTGQRGVAILYAYMLLGLGFLRTVTPDFGDLISQEQQLEGIFRFMHERLCTHAESVAFFGGG
AREKAMVESRFSDLLIHSQYLLKKKCLFGILDDFITKQLPHNVTWLLSLLYAMEHKGDRAVISTLGELAHALRF
LASVVSQSFLAFGDILELNRKLVELSGGVNRIFELEELLDAAHSGEFINGGPISSATDYHSKDVISFSKVNIVTPSQ
KMLARELTCDVELGRSLLVTGPNGSGKSSIFRVLRGLWPIASGRFSRPSEDLDQDVGSGCSIFYVPQRPYTCLGT
LRDQIIYPLSREEAELRALKMYGKGEKHPDTVKLLDKHLEVILENVRLNYLLERDTSGWDANLNWEDTLSLGE
QQRLGMARLFFHKPKFGILDECTNATSVDVEEHLYGLAKKMEITFITSSQRPALIPYHSMELRLIDGEGNWQLR
SIKQ

>XP_024456070.1_ABCD1_Pt
MPSLQLLQLTEHGRGILASRRKSLLFAAGILAAGGTAVYVQSRIRSKKSDSFLYYNGIKDDKKISDKLVTNGKK
TVQKKGGLKALQILASVLLSHMGKTGAKDLLAMIAIAVLKTTLSNRLAKVQGFLFRAAFLKRVPLFFRLISENI
LLCFLLSTINSTSKYVTGTLSLCFRKILTKVIHAHYFENMAYYKISHVDGRITNPEQRIASDVPRFCSELSELVLD
DLTAVTDGLLYTWRLCSYASPKYLFWMVAYVLGAGTLIRNFSPAFGKLMSKEQQLEGEYRQLHSRLRTHAES
IAFYGGENREEFHIQQKFKTLIGHMRTVLHDHWWFGMIQDFLLKYFGATVAVILIIEPFFAGQLRPDASTLGRA
EMLSNLRYHTSVIISLFQSLGTLSISSRRLNRLSGYADRIHELIAVSRELSNGDKSSLQRSGSRNYFSEANYVEFF
GVKVVTPSGNVLVQDLTLKVDSGSNLLITGPNGSGKSSLFRVLGGLWPLVSGHIVKPGVGSDLNKEIFYVPQRP
YTAVGTLRDQLIYPLTADQEIEPLTHSGMVELLKNVDLEYLLDRYPPEKEVNWGEELSLGEQQRLGMARLFYH
KPKFAILDECTSAVTTDMEERFCAQVQAMGTSCITISHRPALVAFHDVVLSLDGEGGWLVNYKGKDSPALTEA
GGDLTGDFETERKNDAMIVQKAFSTSDKATHSYISEVIAASPNIDHNVLLPIVPPLQRAPRALPLRVAAMFKILV
PTILDKQGAHLLAVAFLVISRTFVSDRIASLNGTTVKFVLEQDKASFVRLIGVSVLQSAASSFIAPSLRHLTTRLA

LGWRIRLTQHLLKNYLRNNTFYKVFHMSSKNIDADQRITHDLEKLTTDLSGLVTGMVKPLVDILWFTWRMKL
LTGQRGVAILYTYMLLGLGFLRAVTPDFGDLASEEQQLEGTFRFMHERLRTHAESVAFFGGGKREKAMIESRF
RELLDHSMLLLKKKWSYGILDDFVTKQLPHNVTWGLSLLYAMEHKGDRAMTSTQGELAHALRFLASVVSQS
FLAFGDILELHKKFAELSGSINRIFELEELLDAAQSGDSLNSKLSQSKNIELYSKDAISFMEVDIITPAQKLLARQL
TFDIEQRKSLLLTGPNGSGKSSVFRVLRGLWPIASGRIAKPSQHISKETGSGCAVFYVPQRPYTCLGTLRDQIIYP
LSRDEAEVMTLELYEKGKLSTEITNMLDSCLKNILENVRLNYLLEREGGWDANMNWEDTLSLGEQQRLGMA
RLFFHKPKFAILDECTNATSVDVEEQLYRLASDMGITFITSSQRPALIPFHSLELRLIDGEGHWELRAIKQ

>XP_006585277.1_ABCD1_X2_Gm
MSSLQLFQLTQHGRSFLASRRKTLLLATGILVAGGTTAYVQSRFRVNRDDLLGDSYECNNDKELTKEEVMKG
TSAPKNKQKKGGLKSLQVLAAILLSEMGQLGAKNLLALVSIVVLRTTLSNRLAKVQGFLFRAAFLRRVPLFLR
LISENILLCFLLSTMQSTSKYITGTLSLHFRKILTKLIHSRYFENMVYYKISHVDGRITNPEQRIASDVPRFCSELSE
IVQDDLTAVTDGLLYTWRLCSYASPKYIFWILAYVLGAGAAIRNFSPAFGKLMSREQELEGGYRQLHSRLRTH
SESIAFYGGEKREEAHIQQKFRTLVRHINRVLHDHWWFGMIQDFLLKYLGATVAVILIIEPFFSGHLRPDSSTLG
RAEMLSNLRYHTSVIISLFQSLGTLSISARRLNRLSGYADRIHELMAISRELSLENGKSSLQRQGSRNCISEANYV
GFYGVKVVTPTGNVLVNDLTLKVESGSNLLITGPNGSGKSSLFRVLGGLWPLVSGHIVKPGVGSDLNKEIFYVP
QRPYTAVGTLRDQLIYPLTVDQEVEPLTDSRMVELLKNVDLEYLLDRYPPEKEVNWGDELSLGEQQRLGMAR
LFYHKPKFAILDECTSAVTTDMEERFCANVLAMGTSCITISHRPALVAFHDVVLSLDGEGGWSVHHRREDSSTE
LGNDMMKASETKRQSDAKAVQRAFAMNKKDSAFLNSKAQSDISEVIIASSPSMKRNISPSAVPQLHGNTRALP
MRVAAMCKVLVPTIFDKQGARLLAVVFLVVSRTWVSDRIASLNGTTVKLVLEQDKASFIRLIGLSVLQSAASSF
IAPSIRHLTARLALGGRTHLTQHLLKNYLRNNAFYKVFHMASKNIDADQRITHDLEKLTTDLSGLVTGMVKPS
VDILWFTWRMKMLTGRRGVAILYAYMLLGLGFLRTVTPDFGNLISQEQQLEGTFRFMHERLCTHAESVAFFG
GGAREKAMVESRFRELLTHSEYLLKKKWLFGILDDFITKQLPHNVTWGLSLIYAMEHKGDRASVTTQGELAH
ALRFLASVVSQSFLAFGDILELNKKFVELSGGINRIFELEELLDAAQSENFTSVSAIPPMRDVHSSDVISFSKVDII
TPAQKMLVRELICDIKRGGSLLVTGPNGSGKSSIFRVLRGLWPIASGRLSRPSEVVDEEDGSGCGIFYVPQRPYT
CLGTLRDQIIYPLSREEAEVKALKMYGKGEKHADTRNLLDTRLKVILESVRLNYLLEREGSNWDANLKWEDIL
SLGEQQRLGMARLFFHEPKFGILDECTNATSVDVEEHLYGLAKDMGITVVTSSQRPALIPFHSMELRLIDGEGN
WKLRLIEQ

>XP_025985394.1_ABCD1_X1_Gm
MSSLQLFQLTQHGRSFLASRRKTLLLATGILVAGGTTAYVQSRFRVNRDDLLGDSYECNNDKELTKEEVMKG
TSAPKNKQKKGGLKSLQVLAAILLSEMGQLGAKNLLALVSIVVLRTTLSNRLAKVQGFLFRAAFLRRVPLFLR
LISENILLCFLLSTMQSTSKYITGTLSLHFRKILTKLIHSRYFENMVYYKISHVDGRITNPEQRIASDVPRFCSELSE
IVQDDLTAVTDGLLYTWRLCSYASPKYIFWILAYVLGAGAAIRNFSPAFGKLMSREQELEGGYRQLHSRLRTH
SESIAFYGGEKREEAHIQQKFRTLVRHINRVLHDHWWFGMIQDFLLKYLGATVAVILIIEPFFSGHLRPDSSTLG
RAEMLSNLRYHTSVIISLFQSLGTLSISARRLNRLSGYADRIHELMAISRELSLENGKSSLQRQGSRNCISEANYV
GFYGVKVVTPTGNVLVNDLTLKVESGSNLLITGPNGSGKSSLFRVLGGLWPLVSGHIVKPGVGSDLNKEIFYVP
QRPYTAVGTLRDQLIYPLTVDQEVEPLTDSRMVELLKNVDLEYLLDRYPPEKEVNWGDELSLGEQQRLGMAR
LFYHKPKFAILDECTSAVTTDMEERFCANVLAMGTSCITISHRPALVAFHDVVLSLDGEGGWSVHHRREDSSTE
LGNDMMKASETKRQSDAKAVQRAFAMNKKDSAFLNSKAQSDISEVIIASSPSMKRNISPSAVPQLHGNTRALP
MRVAAMCKVLVPTIFDKQGARLLAVVFLVVSRTWVSDRIASLNGTTVKLVLEQDKASFIRLIGLSVLQSAASSF
IAPSIRHLTARLALGGRTHLTQHLLKNYLRNNAFYKVFHMASKNIDADQRITHDLEKLTTDLSGLVTGMVKPS
VDILWFTWRMKMLTGRRGVAILYAYMLLGLGFLRTVTPDFGNLISQEQQLEGTFRFMHERLCTHAESVAFFG
GGAREKAMVESRFRELLTHSEYLLKKKWLFGILDDFITKQLPHNVTWGLSLIYAMEHKGDRASVTTQGKIFPL
NHLLSLYFSCCSPLSDSTILIGELAHALRFLASVVSQSFLAFGDILELNKKFVELSGGINRIFELEELLDAAQSENF
TSVSAIPPMRDVHSSDVISFSKVDIITPAQKMLVRELICDIKRGGSLLVTGPNGSGKSSIFRVLRGLWPIASGRLSR
PSEVVDEEDGSGCGIFYVPQRPYTCLGTLRDQIIYPLSREEAEVKALKMYGKGEKHADTRNLLDTRLKVILESV
RLNYLLEREGSNWDANLKWEDILSLGEQQRLGMARLFFHEPKFGILDECTNATSVDVEEHLYGLAKDMGITV
VTSSQRPALIPFHSMELRLIDGEGNWKLRLIEQ

>XP_024461106.1_ABCD1_X2_Pt
MTSLQLLQLTERGRGLLANRRKSLLLAAGVLAAGGTAAYVQSRISSKKSDSFLHSNGPKDDKKISNKLVTNDK
KNTQKKRGLKSLQVLAAVLLSRMGKMGAKDLLAMIAIAVLKTTLSNRLAKVQGFLFRAAFLRRVPLFFRLISE
NILLCFLLSTMHSTSKYVTGTLSLCFRKILTKLIHAHYFENMAYYKISHVDGRITNPEQRIASDVPRFCSELSELV

LDDLTAVADGVLYTWRLCSYTSPKYLFWMVAYILGAGTLIKNFTPAFGKLMSKEQQLEGEYRQLHSRLRTHA
ESIAFYGGERREEFHIQQKFETLIRHMSTVLHDQWWFGMIQDFLFKYLGATVAVILIIEPFFSGQLRPDSSTLGRA
EMLSNLRYHTSVIISLFQSLGTLSTSSRRLNRLSGYADRIHELISISRELSNDDKSSLQRSGSKNYFSEADYVEFSG
VKVVTPSGNVLVQDLTLKVESGSNLLITGPNGSGKSSLFRVLGGLWPLVSGHIVKPGVGSDLNKEIFYVPQRPY
TAVGTLRDQLIYPLTADQEIEPLTHSGMVELLKNVDLEYLLDRYPPEKEVNWGEELSLGEQQRLGMARLFYHK
PKFAILDECTSAVTTDMEERFCAKVQATGTSCITISHRPALVAFHDVVLSLDGEGGWHVNYKGKDSPALTEAR
NDITGACETDRKNDAMVVQKAFSTSDKVAHSYISEVIAASPSVDHNLPLPIVPPLQRAPRVLPLRVAAMFKILV
PSILDKQGAHLLAVAFLVVSRTFVSDRIASLNGTTVKLVLEQDRASFVRLIGVSVLQSAASSFIAPSLRHLKTLL
ALGWRIRLTQHLLKNYLRNNTFYKVFNMSRKNIDADQRITHDLEKLTTDLSGLVTGMVKPSVDILWFTWRMK
LLTGQRGVAILYTYMLLGLGFLRAVTPDFGDLASQEQQFEGTFRFMHERLRTHAESVAFFGGGKREKDMIESR
FRELLDHSMLLLKKKWSYGILDDFVTKQLPNNVTWGLSLLYAVENNGDRAMSSTQGDLAHALRYLASVVSQ
SFLAFGDILELHKKFAELSGSINRIFELEELLDSAQSGDSLNGKLSPSKNSELYSKDAISFMEVDIITPAQKLLARQ
LTFDIGQGKSLLLTVLHLIFMSNY

>XP_006380421.2_ABCD1_X1_Pt
MTSLQLLQLTERGRGLLANRRKSLLLAAGVLAAGGTAAYVQSRISSKKSDSFLHSNGPKDDKKISNKLVTNDK
KNTQKKRGLKSLQVLAAVLLSRMGKMGAKDLLAMIAIAVLKTTLSNRLAKVQGFLFRAAFLRRVPLFFRLISE
NILLCFLLSTMHSTSKYVTGTLSLCFRKILTKLIHAHYFENMAYYKISHVDGRITNPEQRIASDVPRFCSELSELV
LDDLTAVADGVLYTWRLCSYTSPKYLFWMVAYILGAGTLIKNFTPAFGKLMSKEQQLEGEYRQLHSRLRTHA
ESIAFYGGERREEFHIQQKFETLIRHMSTVLHDQWWFGMIQDFLFKYLGATVAVILIIEPFFSGQLRPDSSTLGRA
EMLSNLRYHTSVIISLFQSLGTLSTSSRRLNRLSGYADRIHELISISRELSNDDKSSLQRSGSKNYFSEADYVEFSG
VKVVTPSGNVLVQDLTLKVESGSNLLITGPNGSGKSSLFRVLGGLWPLVSGHIVKPGVGSDLNKEIFYVPQRPY
TAVGTLRDQLIYPLTADQEIEPLTHSGMVELLKNVDLEYLLDRYPPEKEVNWGEELSLGEQQRLGMARLFYHK
PKFAILDECTSAVTTDMEERFCAKVQATGTSCITISHRPALVAFHDVVLSLDGEGGWHVNYKGKDSPALTEAR
NDITGACETDRKNDAMVVQKAFSTSDKVAHSYISEVIAASPSVDHNLPLPIVPPLQRAPRVLPLRVAAMFKILV
PSILDKQGAHLLAVAFLVVSRTFVSDRIASLNGTTVKLVLEQDRASFVRLIGVSVLQSAASSFIAPSLRHLKTLL
ALGWRIRLTQHLLKNYLRNNTFYKVFNMSRKNIDADQRITHDLEKLTTDLSGLVTGMVKPSVDILWFTWRMK
LLTGQRGVAILYTYMLLGLGFLRAVTPDFGDLASQEQQFEGTFRFMHERLRTHAESVAFFGGGKREKDMIESR
FRELLDHSMLLLKKKWSYGILDDFVTKQLPNNVTWGLSLLYAVENNGDRAMSSTQGDLAHALRYLASVVSQ
SFLAFGDILELHKKFAELSGSINRIFELEELLDSAQSGDSLNGKLSPSKNSELYSKDAISFMEVDIITPAQKLLARQ
LTFDIGQGKSLLLTGPNGSGKSSVFRVLRGLWPIVSGRLAKPSQHISKETGSGCGIFYVPQRPYTCLGTLRDQIIY
PLSHDEAEVMTLKLSEKGMQSTEMTNILDTRLKNILENVRLNYLLEREGGWDANMNWEDTLSLGEQQRLGM
ARLFFHKPKFAILDECTNATSVDVEEQLYRLAKDMGITFVTSSQRPALIPFHSLELRLIDGEGQWELRAIKQ

>XP_025984282.1_ABCD1-l_X3_Gm
MSREQELEGEYRQLHSRLRTHSESIAFYGGERREEAHIQQKFRTLVRHINRVLHDHWWFGMIQDFLLKYLGAT
VAVILIIEPFFSGHLRPDSSTLGRAEMLSNLRYHTSVIISLFQSLGTLSISARRLNRLSGYADRIHELMAISRELSLD
NGKSSLQRQGSRNYISEANYVGFYGVKVVTPTGNVLVDDLTLKVQSGSNLLITGPNGSGKSSLFRVLGGLWPL
VSGHIVKPGVGSDLNKEIFYVPQRPYTAVGTLRDQLIYPLTADQEVEPLTDSRMVELLKNVDLEYLLDRYPSET
EVNWGDELSLGEQQRLGMARLFYHKPKFAILDECTSAVTTDMEERFCANVLAMGTSCITISHRPALVAFHDVV
LSLDGEGGWSVHHRREDSSTELGNDTVKALETKRQSDAKAVQRAFAMNKKGSAFSNSKAQSDISEVIIASSPS
MKRNISPSAVPQLHGNTRALPMRVAAMCKVLVPTIFDKQGARLLAVAFLVVSRTWVSDRIASLNGTTVKLVL
EQDKASFIRLIGISVIQSAASSFIAPSIRHLTARLALGGRIRLTQHLLKNYLRNNAFYKVFHMASKNVDADQRITH
DLEKLTTDLSGLVTGMVKPSVDILWFTWRMKLLTGRRGVAILYAYMLLGLGFLRTVTPDFGDLISQEQQLEGT
FRFMHERLCTHAESVAFFGGGAREKAMVESRFRELLLHSKYLLKKKWLFGILDDFITKQLPHNVTWGLSLIYA
MEHKGDRASVTTQGKIFALNHLLSIFLLLLSSLTDNTILIGELAHALRFLASVVSQSFLAFGDILELHRKFVELSG
GINRIFELEELLDAAQSENFTSVSAIPPVRDVHSSDVISFSKVDIVTPSQKMLARELIFDIKHGGSLLVTGPNGSGK
SSIFRVLRGLWPIASGRLSRPSEVVDEEDGSGCGIFYVPQRPYTCLGTLRDQIIYPLSCEEAEVKVLKMYGKDEK
HADTRNLLDTRLKAILESVRLNYLLEREGSNWDANLKWEDILSLGEQQRLGMARLFFHKPKFGILDECTNATS
VDVEEHLYGLAKDMGITVVTSSQRPALIPFHSMELRLIDGEGNWKLRLIKQ

>XP_021629213.1_ABCD1_X3_Me
MIRNFSPAFGKLMSKEQQLEGEYRQLHSRLRAHAESIAFYGGECREESHIQQKFKDLVKHMRVALHDHWWFG
MIQDFLLKYLGATVAVILTMEPFFAGPLRPDTSTLGRATMLSNLRYHTSVITSLFQSLGTLSRSARQLNRLSGYA

GRIYELIVVSRELNSDDRAYLQKIGSGNYFSEADHVEFSGVKVVTPTGNVLVEDLTLKVESGSNLLITGPNGSG
KSSLFRVLGGLWPLLSGHIVKPGVGSDLNKEIFYVPQRPYTVIGTLRDQLIYPLTVDQEVEPLTPTGMMELLKN
VDLEYLLDRYPAEQEVNWGEELSLGEQQRLGMARLFYHRPKFAILDECTSAVTTDMEERFCTKVRAMGTSCIT
ISHRPALVAFHDVVLSLDGEGGWQVSYKRKDSSSLMTNVKKTTATERRSDAVVVQRAFATSDKDSAFSKSKA
QSYISEVIVASPRADAGLPLPIFPQLQRAPRVLALRVAAMFRILASTVLDKQGAHLLALAFLVLSRTWVTDRIAS
LNGTTLKYVLEQDKTSFIQLIGVSILQSAASSFIAPSLRHLKARLALGWRICLTQHLLKKYLRNNAFYKVFHML
GKNIDADQRITHDLEKLSRDFSGLLTGMIKPSVDILWFTWRMKLLMGQKGVAILYAYMLLGLGFLRTTTPDFG
DLTSQAQQLEGTFRFIHERLRMHAESIAFFGGGTREKAMIDSRFRELLDHSLLLLKKKWLYGILDDFVTKQLPH
NVTWGLSLLYAMEHKGDRALTSNQAELAHALRFLASVVSQSFLAFGDILELHKKFLELSGGVNRLFELEEILD
AAQFGDWPTDKLSPSKENDVDAKDFISFTEVDIISPAQKLLAKQLTFDIVQGKSLLVTGPNGSGKSSVFRVLRG
LWPIVSGRLSKPSRHINGDSESGCGIFYVPQQPYTCFGTLRDQIIYPLSHDEAVQMTLKLHREDKISDDTTEILDA
RLKAILENVRLNYLLERDEGGWDANLNWEDILSLGEQQRLGMARLFFHKPKFGILDECTNATSVDVEEQLYEL
AHDMNITVVTSSQRPALIPFHSLELRLTDGEGNWELRTIKQ

>XP_021629209.1_ABCD1_X1_Me
MPSLQLLQLTKQGQSLLASRRKSLLLATGILVVGGTAAYVQSRHSSKKPDSFGYSNGLKDNNDKLDKQVTDE
YYLKKTIQNKGSLKPLHVLAAVLLSDMGKMGARDLFTMVAIAVLRTALSNRLAKVQGFLFRAAFLRRVPLFF
RLISENILLCFLLSVIHSTSKYVTGTLSLCFRKILTKHIHANYFENMAYYRMSHVDCRITNPEQRIASDVPRFCSE
LSELVQDDLTAVTDSLLYTWRLCSYAGPKYFFWILAYVVGAGTMIRNFSPAFGKLMSKEQQLEGEYRQLHSR
LRAHAESIAFYGGECREESHIQQKFKDLVKHMRVALHDHWWFGMIQDFLLKYLGATVAVILTMEPFFAGPLR
PDTSTLGRATMLSNLRYHTSVITSLFQSLGTLSRSARQLNRLSGYAGRIYELIVVSRELNSDDRAYLQKIGSGNY
FSEADHVEFSGVKVVTPTGNVLVEDLTLKVESGSNLLITGPNGSGKSSLFRVLGGLWPLLSGHIVKPGVGSDLN
KEIFYVPQRPYTVIGTLRDQLIYPLTVDQEVEPLTPTGMMELLKNVDLEYLLDRYPAEQEVNWGEELSLGEQQ
RLGMARLFYHRPKFAILDECTSAVTTDMEERFCTKVRAMGTSCITISHRPALVAFHDVVLSLDGEGGWQVSYK
RKDSSSLMTNVKKTTATERRSDAVVVQRAFATSDKDSAFSKSKAQSYISEVIVASPRADAGLPLPIFPQLQRAP
RVLALRVAAMFRILASTVLDKQGAHLLALAFLVLSRTWVTDRIASLNGTTLKYVLEQDKTSFIQLIGVSILQSA
ASSFIAPSLRHLKARLALGWRICLTQHLLKKYLRNNAFYKVFHMLGKNIDADQRITHDLEKLSRDFSGLLTGMI
KPSVDILWFTWRMKLLMGQKGVAILYAYMLLGLGFLRTTTPDFGDLTSQAQQLEGTFRFIHERLRMHAESIAF
FGGGTREKAMIDSRFRELLDHSLLLLKKKWLYGILDDFVTKQLPHNVTWGLSLLYAMEHKGDRALTSNQAEL
AHALRFLASVVSQSFLAFGDILELHKKFLELSGGVNRLFELEEILDAAQFGDWPTDKLSPSKENDVDAKDFISFT
EVDIISPAQKLLAKQLTFDIVQGKSLLVTGPNGSGKSSVFRVLRGLWPIVSGRLSKPSRHINGDSESGCGIFYVPQ
QPYTCFGTLRDQIIYPLSHDEAVQMTLKLHREDKISDDTTEILDARLKAILENVRLNYLLERDEGGWDANLNW
EDILSLGEQQRLGMARLFFHKPKFGILDECTNATSVDVEEQLYELAHDMNITVVTSSQRPALIPFHSLELRLTDG
EGNWELRTIKQ

>XP_006603092.1_ABCD1_Gm
MSSLQLLQLTRHGQSFLASRRKTLLLASGILIAGGTAAYVQSRFRVNNHDLFGHCNGHSNDKEVAEEVVKDVS
TPKNKQKKGLKSLQVLAAILLSGMGKFGASDLLGLVAIAVLRTALSNRLAKVQGFLFRAAFLRRVPLFLRLISE
NILLCFLLSTIHSTSKYITGTLSLHFRKILTKLIHSHYFENMVYYKISHVDGRITNPEQRIASDVPRFCSELSEIVQD
DLTAVTDGLLYTWRLCSYASPKYVVWILAYVLGAGAAIRNFSPSFGKLMSKEQQLEGEYRQLHARLRTHSESI
AFYGGERKEEAHIQQKFKTLVRHMYNVLHDHWWFGMIQDLLLKYLGATVAVILIIEPFFSGHLRPDSSTLGRA
EMLSNLRYHTSVIISLFQSLGTLSISARRLNRLSGYADRIYELMAVSRELSLVNEKSSLQRNASRNCIREANYIEF
DGVKVVTPTGNVLVDDLTLRVESGSNLLITGPNGSGKSSLFRVLGGLWPLISGHIVKPGIGSDLNKEIFYVPQRP
YTAVGTLRDQLIYPLTEDQEIELLTDRGMVELLKNVDLEYLLDRYPPEKEVNWGDELSLGEQQRLGMARLFY
HKPKFAILDECTSAVTTDMEERFCAKVRAMGTSCITISHRPALVAFHDVVLSLDGEGGWSVHYKREGSSTEMG
IDTMKASEKKRQSDAKAVQRAFSMSKKDSAFSSPKAQSYFAEVISSSPSINHTISPSAVPQLHGNTRVLPLRAAA
MCKVLVPTVLDKQGAQLLAVAFLVVSRTWVSDRIASLNGTTVKFVLESHTNNKNEKYFKWSEVFHMANKNI
DADQRITHDLEKLTADLSGLVTGMVKPSVDILWFTWRMKLLTGQRGVAILYAYMLLGLGFLRTVTPDFGDLIS
QEQQLEGTFRFMHERLCTHAESVAFFGGGAREKAMVESRFRELLSHSKYLLKKKWLFGILDDFITKQLPHNVT
WLLSLLYAMEHKGDRASISTQGELAHALRFLASVVSQSFLAFGDILELHRKFVELSGGINRIFELEELLDAAQSG
DSINSSITSPIWGYHGKDAISFSKVDIVTPTQKMLARELTCDIELGKSLLVTGPNGSGKSSIFRVLRGLWPIASGR
LSRPSEDVDQEAGSGCGIFYVPQRPYTCLGTLRDQIIYPLSREEAQFRALKMHGKGEKHPDPRKMLDTHLQVIL
ENVRLNYLLERDNNGWDANLNWEDILSLGEQQRLGMARLFFHKPKFGILDECTNATSVDVEEHLYGLANKM
GITVVTSSQRPALIPFHSMELRLIDGEGNWELRSIKQ

>AtABCD1
MPSLQLLQLTERGRGLVASRRKSILLAAGIVAAGGTAVYLKSRVASRRPDSSRLCNGQSDDDETLEKLTATDQ
NAKITTKKKKGGGLKSLQVLTAILLSQMGKMGARDLLALVATVVFRTALSNRLAKVQGFLFRAAFLRRAPLF
LRLISENIMLCFMLSTLHSTSKYITGALSLRFRKILTKIIHSHYFENMVYYKISHVDGRITHPEQRIASDVPRFSSEL
SDLILDDLTAVTDGILYAWRLCSYASPKYIFWILAYVLGAGTAIRNFSPSFGKLMSKEQQLEGEYRQLHSRLRT
HSESIAFYGGETREESHIQQKFKNLVSHMSHVLHDHWWFGMIQDFLLKYLGATVAVILIIEPFFSGHLRPDDSTL
GRAEMLSNIRYHTSVIISLFQALGTLSISSRRLNRLSGYADRIHELMAVSRELSGDDKSSFQRNRSRNYLSEANY
VEFSDVKVVTPTGNVLVEDLTLRVEQGSNLLITGPNGSGKSSLFRVLGGLWPLVSGHIVKPGVGSDLNKEIFYV
PQRPYMAVGTLRDQLIYPLTSGQESELLTEIGMVELLKNVDLEYLLDRYQPEKEVNWGDELSLGEQQRLGMA
RLFYHKPKFAILDECTSAVTTDMEERFAAKVRAMGTSCITISHRPALVAFHDVVLSLDGEGGWSVHYKRDDSA
LLTDAEIDSVKSSDTDRQNDAMVVQRAFAAARKESATNSKAQSYQTQLIARSPVVDKSVVLPRFPQPQTSQRA
LPSRVAAMLNVLIPTIFDKQGAQLLAVACLVVSRTLISDRIASLNGTTVKYVLEQDKAAFVRLIGLSVLQSGASS
IIAPSLRHLTQRLALGWRIRLTQHLLRNYLRNNAFYKVFHMSGNSIDADQRLTRDLEKLTADLSGLLTGMVKPS
VDILWFTWRMKLLTGQRGVAILYTYMLLGLGFLRRVAPDFGDLAGEEQQLEGKFRFMHERLNTHAESIAFFG
GGAREKAMVDKKFRALLDHSLMLLRKKWLYGILDDFVTKQLPNNVTWGLSLLYALEHKGDRALVSTQGELA
HALRYLASVVSQSFMAFGDILELHKKFLELSGGINRIFELDEFLDASQSGVTSENQTSRLDSQDLLSFSEVDIITP
AQKLMASKLSCEIVSGKSLLVTGPNGSGKTSVFRVLRDIWPTVCGRLTKPSLDIKELGSGNGMFFVPQRPYTCL
GTLRDQIIYPLSKEEAEKRAAKLYTSGESSTEAGSILDSHLKTILENVRLVYLLERDVGGWDATTNWEDILSLGE
QQRLGMARLFFHRPKFGVLDECTNATSVDVEEQLYRVARDMGVTFITSSQRPALIPFHSLELRLIDGEGNWELR
SIEQTTE

>HvABCD2
MPSLQLLQLTERGRGLLASRRCALAVVSGALVAGGALAYARSGRGQRRRGRPEAAAANDGGDALGRNGERL
GHSGTDGRLAGTTKRRKSALKSLHFLAAILLKKIGPSGTRYLLGLMLTAVLRTAVGHRLAKVQGFLFKAAFLR
RVPTFTRLIIENLILCFLQSAVYQTSKYLTGSLNLRFKKILTDLVHADYFQNMVYYKISHVDHRISNPEQRIASDI
PKFSSELSELVQDDLAAVAEGLIYTWRLCSYASPKYMLWILAYILVAGGAIRNFSPAFGKMKSTEQHLEGEYR
QLHSRLRTHAESVAFYGGEKREEYHIMQRFRALVGHLKHVLHENWWFGMIQDFFLKYFGATVAVVLIIEPFFS
GDLRPDSSTIGRADMLSNLRYHTSVIIALFQSLGTLSISSRRLNILSGYADRIRELLDVSRELSGVRDRSLNHSSSV
GNYISEANHIEFSGVKVVTPAGNVLVDDLTLRVETGSNLLITGPNGSGKSSLFRVLGGLWPLVSGHIVKPGVGS
DLNKEIFYVPQRPYTAVGTLREQLIYPLTADQETEPLTYDGMVDLLKNVDLEYLLERYPLDKEVNWGDELSLG
EQQRLGMARLFYHKPKFAILDECTSAVTIDMEERFCKKVRAMGTSCITISHRPALVAFHNIVLSLDGEGGWDV
QHRRDDSSFYTEDSELTSLETERKSDALIVQRAFMNRAKSNASLRSKDSYSTKVIANSTKLETGQTVRTPVIPHL
QCSPRPLPLRATAMLKILIPKLLDKQGGQLLAVALLIFSRTVISDRIASLNGTTVKFVLEQDKAAFVRLVGVSVL
QSAANSFVAPSLRTLTSRLALGWRIRLTNHLLQYYLRRNAFYKVFNMSGKSIDADQRLTLDVDKLTTDLAGLV
TGMVKPLVDILWFTWRMKLLSGRRGVGILYAYMLLGLGFLRAISPDFGHLASEEQELEGTFRFMHSRLRTHAE
SIAFFGGGSREKAMVEAKFMKLLNHSKVLLRKQWLYGVVDDFVTKQLPHNVTWGLSLLYALEHKGDRALTS
TQGELAHALRFLASVVSQSFIAFGDILELHKKFLELSGGINRIFELEEILRVAQKDTPVPSSAISAASDEIIEFHEVD
IVTPSQKLLARKLSCSVVQGKSLLLTGPNGTGKSSVFRVLRDLWPAFSGRVTKPSEGMFHVPQSPYTSLGTLRD
QIIYPLSREEAEMKILSLYKSSNRSSAPELLDDHLKTVLVNVRLVYLLEREGWDSTPNWEDVLSLGEQQRLGM
VSVNSTASCANCRNHCLSP

>HvABCD1
MSSLQLLQLTEHGRNLFSSRRRTLAVVSGALLAGGTLAYAQTSRRRKHREENPCNDANVHSRSEENISQNGVD
GKVVKTRKKKNLLKSLHFLAAILLKKIGPSGTNYLLGLMLTAVLRTAIGHRLAKVQGYLFKSAFLRRVPTFTRL
IIENLLLCFLQSTVYQTSKYLTGSLSLRFKKILTDLIHADYFENMVYYKISHVDHRISNPEQRIASDIPKFCAGLSD
LVQDDLIAVADGLIYIWRICSYASPKYVLWILAYVLGAGGTIRKFSPSFGKLKAMEQQLEGEYRQVHSRLRTH
AESVAFYGGENREESHIMQRFQALVRHLNVVLHENWWFGMIQDFLLKYLGATVGVILIVEPFFAGDLKPDTST
LGRAEMLSNLRYHTSVIISLFQSLGTLSTSSRRLNLLSGYADRIHELLDVSRELSGVRDRSMSRNSSAKNYISEA
NYIEFSGVKVVTPSGNVLVDDLTLRVESGSNLLITGPNGSGKSSLFRVLGGLWPLVSGHIVKPGVGSNLNKEIFY
VPQRPYTAVGTLRDQLIYPLTADQETEPLSYGGMVDLLKNVDLEYLLERYPVDKEVNWGDELSLGEQQRLGM
ARLFYHKPKFAILDECTSAVTTDMEERFCNRVRAMGTSCITISHRPALVAFHDVVLSLDGEGGWKVQDNRNGS
FLPTESEFDALKSSETDRKSDALAVQRAFSANTKENTLSGPKDHSYSTQVIATSPNMEIESTEQPHLIPQLQCSPR
PLPVRVAAMSKILVPKVIDKQGAQLLAVALLVLSRTWISDRIASLNGTSVKYVLEQDKAAFIRLIGTSVMQSAA

NSIVAPSLRHLTSKIALGWRIRMTNHLLAYYLKRNAFYKVFNMTGTDIDADQRITRDVEKLTNDLAGLVTGMV
KPSVDILWFTWRMKLLSGRRGVAILYAYMLLGLGFLRAVSPDFGDLSNQEQELESSFRLVI
